# Comparative Analysis of miRNAs and Their Target Transcripts between a Spontaneous Late-Ripening Sweet Orange Mutant and Its Wild-Type Using Small RNA and Degradome Sequencing

**DOI:** 10.3389/fpls.2016.01416

**Published:** 2016-09-21

**Authors:** Juxun Wu, Saisai Zheng, Guizhi Feng, Hualin Yi

**Affiliations:** Key Laboratory of Horticultural Plant Biology (Ministry of Education), College of Horticulture and Forestry Science, Huazhong Agricultural UniversityWuhan, China

**Keywords:** citrus, fruit ripening, miRNA, small RNA sequencing, degradome sequencing

## Abstract

Fruit ripening in citrus is not well-understood at the molecular level. Knowledge of the regulatory mechanism of citrus fruit ripening at the post-transcriptional level in particular is lacking. Here, we comparatively analyzed the miRNAs and their target genes in a spontaneous late-ripening mutant, “Fengwan” sweet orange (MT) (*Citrus sinensis* L. Osbeck), and its wild-type counterpart (“Fengjie 72-1,” WT). Using high-throughput sequencing of small RNAs and RNA degradome tags, we identified 107 known and 21 novel miRNAs, as well as 225 target genes. A total of 24 miRNAs (16 known miRNAs and 8 novel miRNAs) were shown to be differentially expressed between MT and WT. The expression pattern of several key miRNAs and their target genes during citrus fruit development and ripening stages was examined. Csi-miR156k, csi-miR159, and csi-miR166d suppressed specific transcription factors (*GAMYBs, SPLs*, and *ATHBs*) that are supposed to be important regulators involved in citrus fruit development and ripening. In the present study, miRNA-mediated silencing of target genes was found under complicated and sensitive regulation in citrus fruit. The identification of miRNAs and their target genes provide new clues for future investigation of mechanisms that regulate citrus fruit ripening.

## Introduction

Citrus is one of the most widely grown fruit tree crops in the world, with a high economic value. The maturity time of citrus fruits varies among different varieties. The ripening stage is accompanied by the synthesis of numerous proteins and the transcription of many genes. Carotenoids, sugars, and other soluble compounds accumulate; organic acid contents and chlorophyll are reduced; the cell wall is extensively modified; and the concentration of a number of volatiles increases (Katz et al., 2011; Yu et al., 2012). The elucidation of citrus fruit ripening regulatory pathways and networks is important for the improvement of citrus varieties.

MicroRNAs (miRNAs), which are typically 20–24 nucleotides (nt) in length, represent an important class of regulatory molecules found in plants and animals. Derived from stem loop hairpin primary miRNA transcripts (pri-miRNAs), miRNAs negatively regulate target genes through homology-directed cleavage or translation inhibition of mRNAs (Bartel, 2004; Mallory and Vaucheret, 2006). The cleavage of target genes by some 22 nt miRNAs, which are generated from perfect duplexes comprising a 22 nt miRNA and a 22 nt miRNA^*^ by DCL2, can trigger the biogenesis of another class of sRNAs (Chen et al., 2010); an RNA-dependent RNA polymerase (RDR6) is recruited to convert an upstream or downstream cleaved fragment into double-stranded RNA that is subsequently cleaved by DICER-LIKE 4 into 21 nt sRNAs (Axtell et al., 2006; Allen and Howell, 2010). These sRNAs are named phased secondary small interfering RNAs (phasiRNA), some of which function in *trans* (*trans*-acting siRNA, tasiRNA) or *cis* (Fei et al., 2013). In dicots, phasiRNAs have been found to be generated from large and conserved gene families and presumably to regulate large and conserved gene families, including those encoding nucleotide binding leucine-rich repeat proteins (NB-LRR genes), MYB transcription factors and pentatricopeptide repeat proteins (PPR genes; Fei et al., 2013; Xia et al., 2015a,b).

miRNAs are important regulators in transcriptional and post-transcriptional silencing of genes in plant development (Debat and Ducasse, 2014). During the past decade, many miRNAs have been shown to play an important role in regulating development and ripening of fruit (Moxon et al., 2008; Zuo et al., 2012, 2013; Liu Y. et al., 2014; Bi et al., 2015; Chen et al., 2015). For example, over-expression of an *AtMIR156b* precursor generated abnormal flower and fruit morphologies in tomato (Silva et al., 2014). miR156 and miR172 coordinately regulate the transition from the juvenile to the adult phase of shoot development in plants, and miR156/157 and miR172 affect the ripening process of tomatoes by regulating the known ripening regulators *CNR* and *SlAP2a* (Chen et al., 2015). miR159 was shown to be involved in strawberry fruit ripening by regulating *FaGAMYB* which plays a central role in the transition of the strawberry receptacle from development to ripening (Csukasi et al., 2012; Vallarino et al., 2015).

In citrus, many miRNAs have been identified in different tissues, such as the leaf, flower, fruit, and callus (Xu et al., 2010; Zhang et al., 2012; Liu Y. et al., 2014; Wu et al., 2015). However, the miRNAs involved in the citrus fruit ripening process remain largely unknown. To gain a better understanding the role of miRNAs in citrus fruit ripening, small RNA and degradome sequencing were combined to identify miRNAs and their target genes in “Fengjie 72-1” navel orange and its spontaneous late-ripening mutant “Fengwan.” In our previous study (Wu et al., 2014b), the physiological changes (including sucrose, fructose, glucose, citric acid, quinic acid, malic acid, and abscisic acid) of fruits were different between “Fengjie 72-1” and “Fengwan” during fruit ripening. And the 170 DAF (days after flowering) stage was found to be the turning point at which the fruit of “Fengwan” diverged in its development from that of the wild type. In this study, the differentially expressed miRNAs between “Fengjie 72-1” and “Fengwan” were comparatively analyzed, and the role of miRNAs in the regulation of fruit ripening was also explored, contributing to the regulatory network of citrus fruit ripening.

## Materials and methods

### Plant materials and illumina sequencing

The “Fengjie 72-1” navel orange (*Citrus sinensis* L. Osbeck) (WT) and its spontaneous late-ripening mutant “Fengwan” (MT) were cultivated in the same orchard located in Fengjie, Chongqing City, China (N31°03′35′, E109°35′25′). Fruit samples of WT and MT used in sRNAome and degradome sequencing were collected at 170 days after flowering (DAF) in 2013. The pulps of fruit samples (from six trees, three trees represented one biological replicate) of WT and MT were used for sRNAome sequencing, respectively. And the pulps of fruit samples from WT and MT were mixed as a pool for degradome sequencing. To detect the expression pattern of key miRNAs and target genes in fruit development, the fruit samples (from nine trees, three trees represented one biological replicate) were collected in 2015 at different developmental stages, including 50 DAF, 80 DAF, 120 DAF, 155 DAF, 180 DAF, and 220 DAF. Fruit samples were separated into peel and pulp after collection. Pulp was used in all analyses in this study. All samples were frozen in liquid nitrogen immediately after collection and kept at −80°C until use. Total RNA was extracted according to Xu et al. (2010). Four small RNA libraries (MT_bio1, MT_bio2, WT_bio1, and WT_bio2) and one degradome library (uniform mixture of total RNA extracted from WT and MT) were constructed (Addo-Quaye et al., 2008; Hafner et al., 2008) and sequenced using an Illumina HiSeq™2000 at Beijing Genomics Institute (BGI; Shenzhen, China). The sequencing data were deposited at NCBI Gene Expression Omnibus (GEO) under the accession number GSE84191.

### Deep sequencing data analysis

The raw reads of small RNA libraries were pre-processed to remove low-quality reads, adaptors and contaminants to obtain clean reads. The clean reads were used to search GenBank and the Rfam 11.0 database (http://rfam.sanger.ac.uk/) to annotate rRNAs, tRNAs, snRNAs, and snoRNAs. Reads matching repeat sequences, reads matching the exon and intron of genome sequence of *C. sinensis* (http://citrus.hzau.edu.cn/; Xu et al., 2013) and reads matching the known miRNAs of all plants in the miRBase 20.0 database were also annotated. Based on the following priority rule: rRNAetc (in which Genbank > Rfam) > known miRNA > repeat > exon > intron (Calabrese et al., 2007), each small RNA was reannotated to obtain a unique category that is summarized in Table 1. The reads in the miRNA category were used to identify the known/conserved miRNAs based on the criteria of a previous publication (Meyers et al., 2008). The unannotated reads (unann category) were used to predict novel/unconserved miRNAs.

**Table 1:** **Data set summary of the sequencing of four (MT_bio1, MT_bio2, WT_bio1, and WT_bio2) small RNA and one degradome libraries**.

		**Unique reads**				**Total reads**			
	**Category**	**MT**		**WT**		**MT**		**WT**	
		**bio1**	**bio2**	**bio1**	**bio2**	**bio1**	**bio2**	**bio1**	**bio2**
Small RNA data	Clean reads	9,503,927	8,755,251	8,247,653	7,105,581	34,248,996	31,898,997	30,815,849	23,377,054
	Match genome	5,220,473 (54.93%)	4,813,413 (54.98%)	4,432,917 (53.75%)	3,640,580 (51.24%)	20,653,753 (60.3%)	18,950,663 (59.41%)	16,724,660 (54.27%)	11,820,593 (50.56%)
	miRNA	39,836 (0.42%)	38,104 (0.44%)	37,762 (0.46%)	34,132 (0.48%)	967,988 (2.83%)	907,006 (2.84%)	730,625 (2.37%)	615,550 (2.63%)
	Match GenBank/Rfam	83,655 (0.88%)	85,614 (0.98%)	99,252 (1.2%)	67,674 (0.96%)	2,526,031 (7.37%)	2,650,732 (8.31%)	3,446,923 (11.18%)	1,438,855 (6.15%)
	unann	8,516,707 (89.61%)	7,823,090 (89.35%)	7,359,189 (89.23%)	6,386,719 (89.88%)	25,532,148 (74.55%)	23,631,892 (74.08%)	22,816,778 (74.04%)	18,379,048 (78.62%)
Degradome data	Clean reads	4,443,212				33,690,279			
	Match genome	3,210,105(72.25%)				232,62,347 (69.05%)			
	Match cDNA_sense	3,045,152 (68.53%)				20,441,974 (60.68%)			
	Match cDNA_antisense	34,079 (0.77%)				230,844 (0.69%)			
	Match GenBank/Rfam	13,558 (0.3%)				199,304 (0.59%)			
	polyN	10,019 (0.23%)				41,269 (0.12%)			
	unann	1,340,404 (30.17%)				12,776,888 (37.92%)			

The raw data of the degradome was trimmed by pre-analysis similar to the sRNAome raw data to obtain clean reads. The GenBank and Rfam 11.0 databases were used to annotate the clean reads to remove rRNAs, snoRNAs, snRNAs, and tRNAs. The reads with a single base over 70% in the sequences were identified as poly N. Then, the reads were reannotated according to the Rfam > GeneBank > poly N rule. The remaining unannotated sequences were mapped to the reference genes (cDNA) of the *C. sinensis* genome (Xu et al., 2013) by SOAP2 (Li et al., 2009). The reads mapped to cDNA_sense were used to predict cleavage sites by PAREsnip (version 2.3; Folkes et al., 2012). The cleaved target transcripts were categorized into five classes based on the abundance of degradome reads indicative of miRNA-mediated cleavage. Category 0 comprised the sequences whose abundance at the cleavage site was the only maximum on the transcript; in category 1, the reads abundance at the cleavage site was the maximum but not unique; category 2 consisted of sequences whose abundance at the cleavage site was higher than the median but not the maximum; category 3 included sequences whose abundance at the cleavage site was equal to or below the median; the remaining sequences, which were the only raw reads at the cleavage site, were classified as category 4. When the alignment score was no more than 4.5, the transcripts were considered as miRNA targets. The alignment score was calculated by the Rule-Based Complementarity Search algorithm (Folkes et al., 2012). The T-Plot figures were generated by VisSR (version 1.0).

### Identification of known miRNAs and prediction of novel miRNAs

The software tag2miRNA (developed by BGI) was used to identify known miRNAs. We take full account of miRNA conservation. The steps are as following: first, considering the difference among species, align clean reads to the miRNA precursor/mature miRNA of all plants in the miRBase allowing two mismatches and three gaps; second, choose the highest expression miRNA for each mature miRNA family which is regarded as a temporary miRNA database; third, align clean reads to the above temporary miRNA database and the expression of miRNA is generated by summing the count of tags which can align to the temporary miRNA database within two mismatches; fourth, predict the precursor of the identified miRNA, if the precursor of the identified miRNA can't fold into hairpin structure, it will be regarded as pseudo-miRNA. The feasibility of our result can be greatly improved by this verification. The MIREAP pipeline (http://sourceforge.net/projects/mireap/) was used to identify novel miRNAs with default parameters for plant. Some key conditions are as following: (a) the reads which be used to predict novel miRNA are from the unannotated reads which can match to reference genome, the reads which can align to intron region and the reads which can align to antisense exon region; (b) those genes whose sequences and structures meet these two criteria (i.e., the sequences can fold into hairpin secondary structures and mature miRNAs are present in one arm of the hairpin structures) will be considered as candidate miRNA genes; (c) the mature miRNA strand and its complementary strand (miRNA^*^) present 2-nucleotide 3′ overhangs; (d) hairpin precursors lack large internal loops or bulges; (e) the secondary structures of the hairpins are steady, with the free energy of hybridization lower than or equal to −18 kcal/mol; (f) the number of mature miRNA with predicted hairpin must be no fewer than 5 in the alignment result. The hairpin structures of pre-miRNA sequences were visualized by VisSR (version 1.0) and selected manually. In addition, considering many of the known miRNAs in model plant species were not found to have miRNAs^*^ in small RNA sequencing, only novel miRNAs were required to have miRNAs^*^ in this study (Xie et al., 2012).

### miRNA expression profiles and comparison between MT and WT

Raw miRNA counts were normalized to RPM (reads per million) using the equation RPM = (raw count/total raw count) × 1,000,000. According to previous reports (Murakami et al., 2006; Xie et al., 2015), original miRNA expression equal 0 in a library was normalized expression to 0.01. The miRNA expression fold change between MT and WT was computed with the formula “Fold change = log_2_(MT/WT).” The differential expression analysis was performed using the R-package NOISeq (Tarazona et al., 2012, 2015). NOISeq method can screen differentially expressed genes between two groups, showing a good performance when comparing it to other differential expression methods, like Fisher's Exact, Test (FET), edgeR, DESeq, and baySeq. NOISeq maintains good True Positive and False Positive rates when increasing sequencing depth, while most other methods show poor performance. What's more, NOISeq models the noise distribution from the actual data, so it can better adapt to the size of the data set, and is more effective in controlling the rate of false discoveries. First, NOISeq uses sample's gene expression in each group to calculate log_2_(fold change) M and absolute different value D of all pair conditions to build noise distribution model. Second, for gene A, NOISeq computes its avearge expression “Control_avg” in control group and average expression “Treat_avg” in treatment group. Then the foldchange [M_A_ = log_2_((Treat_avg)/(Control_avg))] and absolute different value D (D_A_ = |Congrol_avg-Treat_avg|) will be got. If M_A_ and D_A_ diverge from noise distribution model markedly, gene A will be defined as a DEG. There is a probability (Prob.) value to assess how M_A_ and D_A_ both diverge from noise distribution model. Fold change and probability (Prob.) were combined to determine the final miRNA expression significance. The value of |log_2_(fold change)| ≥ 1.0 with a Prob. > 0.8 was defined as a significant difference.

### Identification of *PHAS* genes and phasiRNAs

Ta-si prediction tool (version 2.0), one tool of the UEA sRNA workbench (Stocks et al., 2012), was used to identify phasiRNAs and *PHAS* genes. The clean reads of MT and WT were supplied as sRNA dataset and the *C. sinensis* genome (Xu et al., 2013) was supplied as reference genome. The cutoff *P*-value calculated by the algorithm described by Chen et al. (2007) was set as 1.0E−4. The sRNAs that did not match the genome were discarded, and only 21 nt sRNAs were used in the phasing analysis.

### Target gene functional annotation

The protein sequences of the identified miRNA target genes were aligned against GO database and the KEGG pathway database using KOBAS 2.0 (http://kobas.cbi.pku.edu.cn/; Xie et al., 2011) to perform enrichment analysis. The corrected *P* < 0.05 was set as cutoff for enrichment. REVIGO (Supek et al., 2011) was used to visualize and summarize the biological process and molecular function terms that were identified by KOBAS 2.0. In addition, the target genes were also annotated by a BLASTP search against the NR database of NCBI with cutoff *E* < 1e−8, and BlastKOALA was used to annotate the KOs of the KEGG ORTHOLOGY database.

### Validation of miRNA and target gene expression in fruit development and ripening by qRT-PCR

Stem-loop qRT-PCR was performed to validate the expressions of miRNAs with three biological replicates based on a previous method (Chen et al., 2005; Liu Y. et al., 2014). The stem-loop reverse transcriptase reaction used 20 μl PCR system: firstly, added 10 mM dNTPs (Promega) and 500 ng purified total RNA into a 200 μl centrifuge tube, slightly centrifuged, then incubated 5 min at 65°C, 2 min on ice; secondly, added 4 μl 5x RT buffer (Invitrogen), 2 μl DTT (Invitrogen, 0.1 M), 0.1 μl RNaseOUT (Invitrogen, 40 U/μl), 0.25 μl SuperScript III (Invitrogen, 200 U/μl), and 1 μl stem-loop RT primer. The thermal cycling program of stem-loop RT PCR was set at 16°C for 30 min, 60 cycles of 30°C for 30 s, 42°C for 30 s, and 50°C for 1 s, then, 85°C for 5 min and 4°C for 30 min. U4 was used as the endogenous reference gene (Kou et al., 2012). The primers are listed in Table S8. Next, the qRT-PCR was performed according to our previous study (Wu et al., 2014b), which was briefly described as following.

qRT-PCR was performed to confirm the expression of target genes with three biological replicates according to our previous study (Wu et al., 2014b). qRT-PCR was performed in ABI 7900HT Fast Real-time system (PE Applied Biosystems, Foster City, CA, USA) with optical 384-well plates. The SYBR Green PCR Master Mix (PE Applied Biosystems) was used in reactions. *CsActin* was used as the endogenous reference gene (Wu et al., 2014a). The primers are listed in Table S8. Ten microlitres of the reaction mixture was added to each well. The thermal cycling program was set at 50°C for 2 min, 95°C for 1 min, and 40 cycles of 95°C for 15 s, and of 60°C for 1 min.

## Results

### Deep sequencing of small RNA libraries and the degradome library

**Figure 1:**
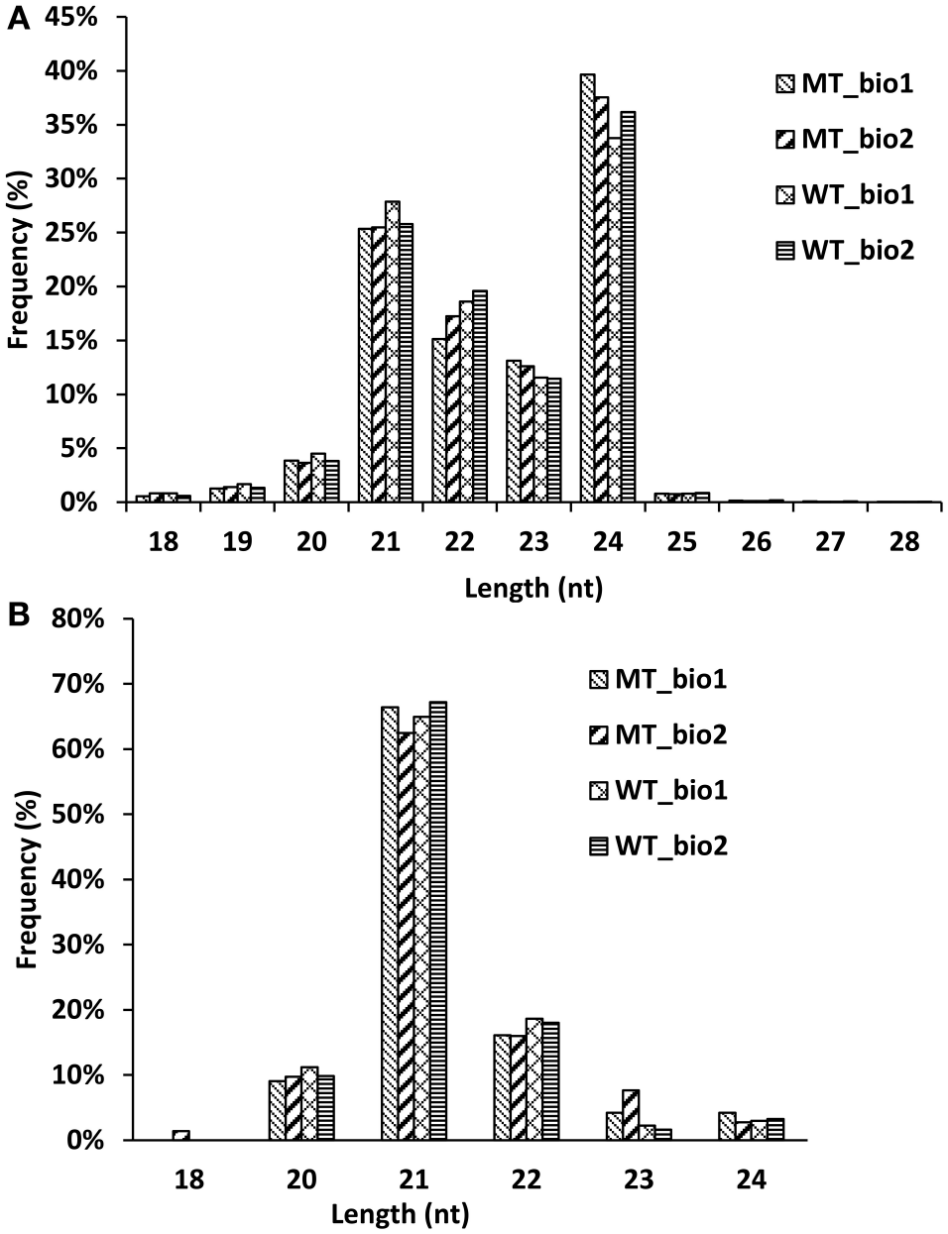
**Length distribution of the small RNA reads (A) and known miRNAs (B) in the sequencing samples**.

In our previous study (Wu et al., 2014b), we comparatively analyzed the transcriptomes and proteomes of MT and WT during citrus fruit ripening, and the 170 DAF stage was identified as the stage at which the most number of differentially expressed genes and proteins between MT and WT were found. To identify the miRNAs involved in the citrus fruit ripening process, the pulps of MT and WT harvested at 170 DAF were used to construct four sRNA libraries with two biological replicates for each sample, which were then sequenced using Illumina HiSeq™2000. After removing the low-quality reads, clean reads were produced. A total of 34,248,996; 31,898,997; 30,815,849; and 23,377,054 reads were generated from two MT samples and two WT samples, respectively, including 9,503,927; 8,755,251; 8,247,653; and 7,105,581 unique reads, respectively (Table 1). Less than 20.65 million (60.3%) redundant reads matched the *C. sinensis* genome (Xu et al., 2013) in each library (Table 1). A search of the miRBase database (v20) identified 39,836 (0.42%), 38,104 (0.44%), 37,762 (0.46%), and 34,132 (0.48%) unique reads for the sRNAs of the two MT and the two WT samples, respectively (Table 1). For annotation, the small RNAs were grouped into several categories, including GenBank/Rfam (the sum of rRNAs, snRNAs, snoRNAs, and tRNAs) and miRNAs (Table 1). The unannotated sRNAs were mapped to the genome and were used to predict novel miRNAs based on the structure and expression criteria (Meyers et al., 2008). Sequences with lengths between 18 and 28 nt accounted for ~99.9% of all the clean reads and sequences matching the known miRNAs were counted to determine the size distribution (Figure 1). The length distribution patterns of all clean reads (Figure 1A) and the reads matching known miRNAs (Figure 1B) in the four sRNA libraries were similar and did not show significant differences between MT and WT.

To identify the target genes of miRNAs, a high-throughput approach, called degradome sequencing, or parallel analysis of RNA ends (PARE; Addo-Quaye et al., 2008; German et al., 2008) was performed to experimentally validate target genes of miRNAs by capturing mRNA cleavage segments. This approach can concurrently identify all cleavage products in the genome with a high sensitivity detection, compared with older techniques, such as northern blotting or 5′ RACE (Xia et al., 2015b). To maximize the identification of miRNA targets, we pooled RNA from the pulps of MT and WT into one library. In total, 33.69 million total reads with 4.44 million unique reads were obtained (Table 1). A search of the Rfam and GenBank database was used to remove the structural RNAs (rRNAs, tRNAs, snRNAs, and snoRNAs), which represented 0.3% of the unique reads. Additionally, the polyN reads, representing 0.23% of the unique reads, were also removed. The remaining sequences were mapped to the *C. sinensis* genome (Xu et al., 2013). Finally, 3.21 million (72.25%) unique reads were mapped to the reference genome and 3.05 million (68.53%) unique reads were mapped to the cDNA sequences of the reference genome. All sequences that mapped to the cDNA library (the sequences in the cDNA_sense category in Table 1) were analyzed to detect candidate targets of miRNAs.

### Identification and expression analysis of known and novel miRNAs in the fruits of MT and WT

A total of 107 known miRNAs, belonging to 53 families, and 21 novel miRNAs were identified from the four libraries of MT and WT (Tables S1, S3). Only the miRNAs identified in both biological replicates were retained. As shown in Figure S1, 99 known miRNAs and 17 novel miRNAs were identified in MT, 92 known miRNAs and 16 novel miRNAs were identified in WT. And among these miRNAs 84 known miRNAs and 12 novel miRNAs were identified in both MT and WT. All of the precursors of known and novel miRNAs had regular stem-loop secondary structures, and the sequences of the miRNAs are shown in blue and the sequences of the miRNAs^*^ are in red (if the miRNA^*^ was identified; Figures S3, S4). Pre-miRNAs (the precursors) of known and novel miRNAs were not evenly distributed in the citrus genome; there were more pre-miRNAs located in chromosome 2 (chr2) and chromosome 7 (chr7; Figure S2).

The normalized expressions (RPM) of known miRNAs in MT and WT ranged from 0.78 to 2151.12 and 0.34 to 1780.005, respectively (excluding the miRNAs that were not identified; Table S1). The results indicated that the known miRNAs exhibited extensive variation in their abundances. The RPM of most novel miRNAs was low; however, there were two novel miRNAs, csi-miRN03, and csi-miRN11, that were highly expressed in both MT and WT (Table S2). The RPMs of csi-miRN11 and csi-miRN03 were much higher than those of known miRNAs.

**Figure 2:**
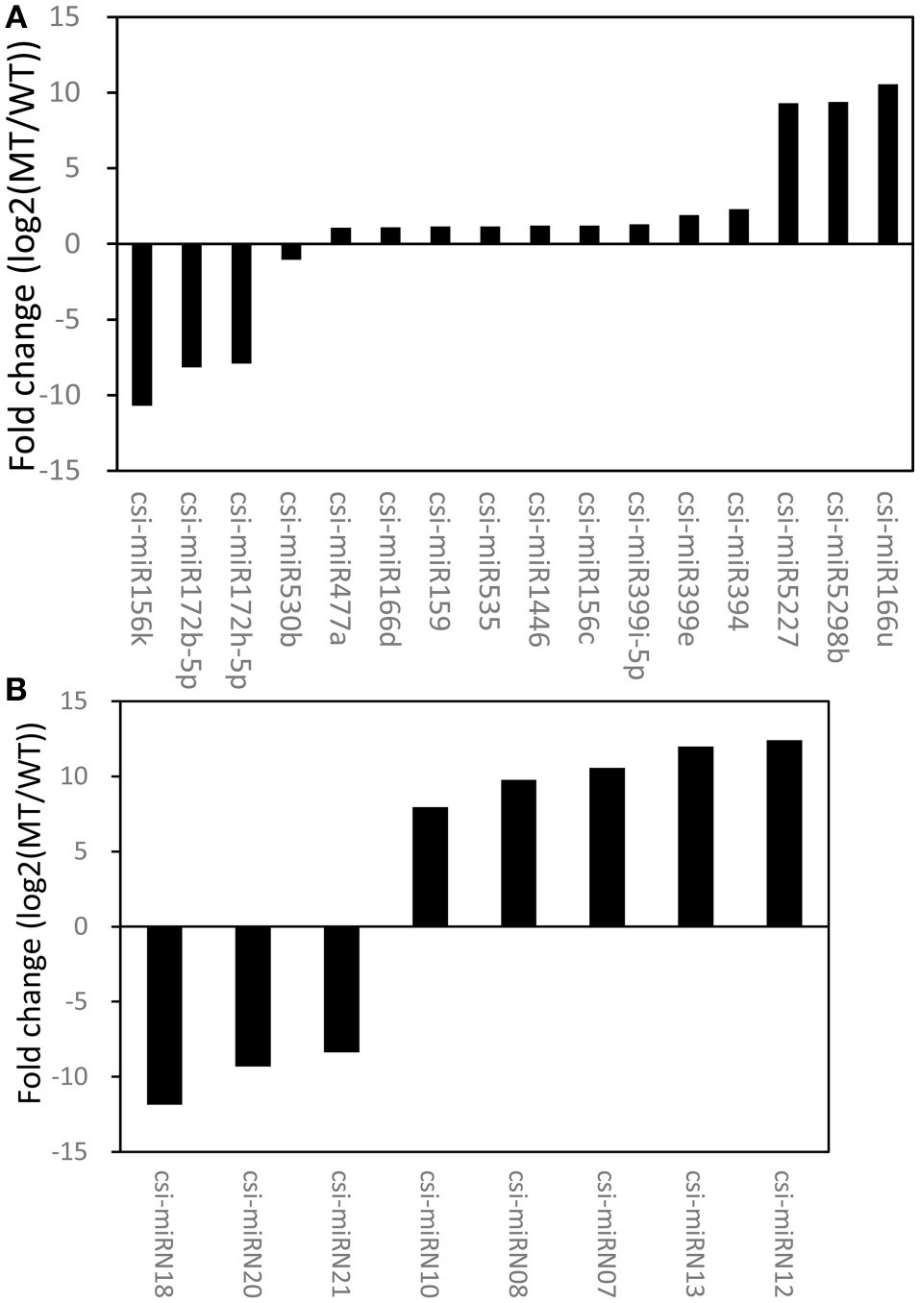
**The differentially expressed known miRNAs (A) and novel miRNAs (B) between MT and WT**.

An R package, NOISeq, was used to perform differential expression analysis of miRNAs between MT and WT. Based on the criteria of significant difference, |log_2_(MT/WT)| ≥ 1 and prob. > 0.8, 16 known miRNAs and 8 novel miRNAs were identified as differentially expressed between MT and WT (Figure 2, Tables S1, S2). The results showed that 14.95% (16/107) of the known miRNAs and 38.10% (8/21) of the novel miRNAs were differentially expressed between MT and WT. As shown in Figure 2, there were more up-regulated miRNAs than down-regulated miRNAs. For the known miRNAs, 12 miRNAs were up-regulated and 4 miRNAs were down-regulated. For the novel miRNAs, 5 miRNAs were up-regulated and 3 miRNA were down-regulated. These results were consistent with our previous study (Wu et al., 2014b), in which there were more down-regulated transcripts than up-regulated transcripts at 170 DAF comparing MT with WT.

### Identification and annotation of the targets of miRNAs

From the degradome data, a total of 401 transcripts from 225 genes were predicted to be targeted by 57 miRNAs (47 known miRNAs and 10 novel miRNAs), with 692 miRNA-target pairs (Table S3). These 401 target transcripts were classified into five categories (category 0, 1, 2, 3, and 4) based on the confidence evaluation of the degradome data. Category 0 is the most reliable for the detection of miRNA target genes, in which the miRNA-guided cleavage fragment was the most abundant tag matching the transcript. In category 1 and 2, the miRNA-guided cleavage fragment was not the most abundant tag, but it still formed a clear peak in the T-plot. In category 3 and 4, the miRNA-target pairs are not reliable. There were 188, 15, 133, 59, and 6 target transcripts in category 0, 1, 2, 3, and 4, respectively (Table S3 and Figure S5). The miRNA-target pairs were visualized on the citrus genome by the Circos software package (http://circos.ca/; Figure S6). We found that the target genes of miRNAs, represented by arrows, were evenly distributed in the citrus genome (Figure S6). The detailed information on these target genes is listed in Table S4.

**Figure 3:**
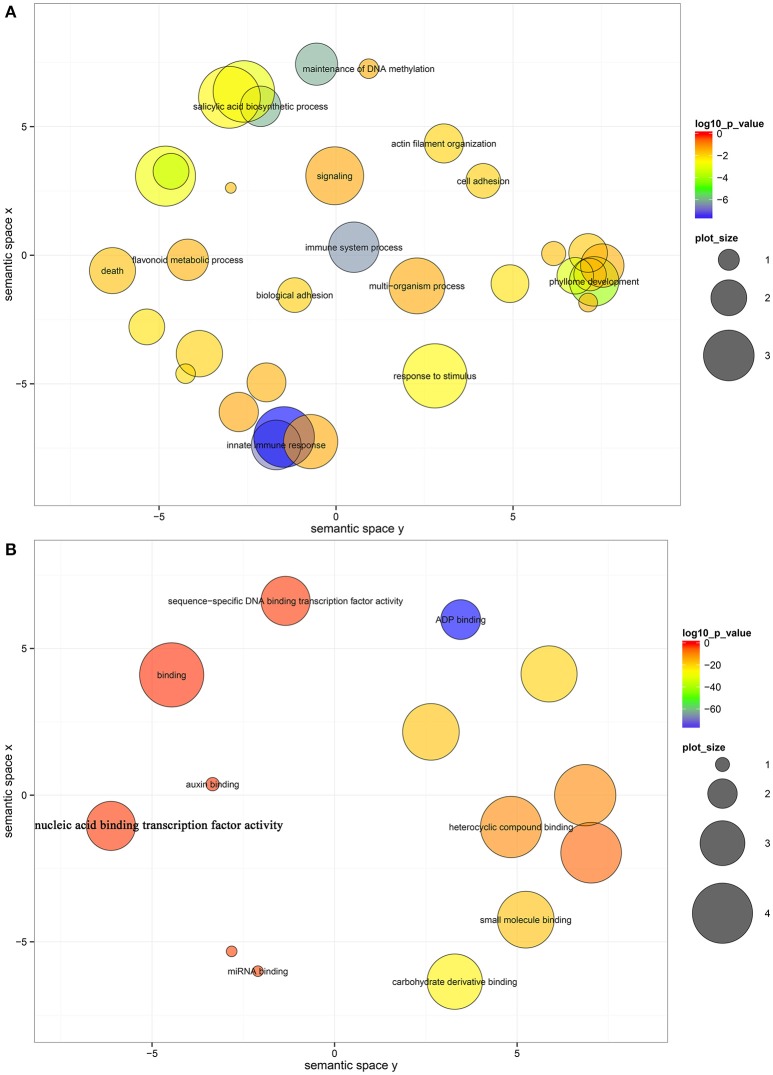
**Biological process (A) and molecular function (B) enrichment analysis of the target genes of miRNAs**. The bubble color indicates the *P*-value; the plot size indicates the frequency of the GO term in the underlying GOA database (bubbles of more general terms are larger).

To further elucidate the roles of miRNAs in fruit ripening, GO-based term classification and KEGG-based pathway enrichment analyses were performed. Under a cutoff of corrected *P* < 0.05, the targets of miRNAs were shown to be enriched in 33 biological processes, 14 molecular functions, and 2 cellular components after summarizing the GO terms by removing redundant GO terms by REViGO (Supek et al., 2011; Table S5). In biological processes, several hubs were significantly enriched, including innate immune response, salicylic acid biosynthetic process, phyllome development, response to stimulus, flavonoid metabolic process, and signaling (Figure 3A). ADP binding, carbohydrate derivative binding, small molecule binding, heterocyclic compound binding, and sequence-specific DNA binding transcription factor activity were significantly enriched in molecular function (Figure 3B). The RNAi effector complex and RISC complex were the enriched cell components (Table S5). However, no enrichment in KEGG pathway was identified, and only 58 genes were annotated to KOs (Table S4).

### Functional analysis of genes targeted by differentially expressed miRNAs between MT and WT

In the present study, 27 target genes of 14 differentially expressed miRNAs were identified (Tables 2, 3). After removing redundant GO terms, the targets of differentially expressed miRNAs were enriched in 13 biological processes and 5 molecular functions (corrected *P* < 0.05; Table 4). In molecular functions, 11 genes were enriched in sequence-specific DNA binding transcription factor activity (GO:0003700), indicating that transcription factors were the major class of genes targeted by differentially expressed miRNAs; in biological processes, several enriched GO terms were related to fruit development, such as xylem and phloem pattern formation (GO:0010051), developmental maturation (GO:0021700), regulation of growth (GO:0040008), and phyllome development (GO:0048827; Table 4).

**Table 2:** **Targets of the known miRNAs identified in MT and WT**.

**miRNA/Target gene**	**Category**	**Cleavage position**	***P*-value**	**Fragment abundance**	**Alignment score**	**RPM**		**Prob**.	**log2(MT/WT)**	**Target annotation**
						**WT**	**MT**			
**csi-miR156k**						16.63	0.01	1.00	−10.70	
Cs2g23550	0	934	0.01	27	3					SPL4
Cs7g10830	0	1183	0	15	1					SPL2
Cs7g10990	0	1547	0	15	2					SPL12
Cs7g11770	0	1488	0	12	1.5					SPL6
**csi-miR159**						25.11	55.64	0.99	1.15	
Cs1g03470	0	108	0	22	2					GAMYB
Cs1g06080	2	589	0.03	3	2.5					NOZZLE
Cs3g06390	0	773	0	58	2.5					GAMYB
Cs8g05120	0	205	0	32	3					Polygalacturonase inhibitor 1
**csi-miR166d**						1.45	3.12	0.87	1.10	
Cs1g15640	0	1781	0	94	2.5					ATHB15
Cs2g09770	1	1069	0	10	2.5					ATHB14
Cs4g19310	0	677	0	189	2.5					ATHB8
Cs6g14050	2	454	0.03	7	3					IAA-amino acid hydrolase ILR1-like 1
Cs8g16510	0	1434	0	211	2.5					REVOLUTA
**csi-miR172h-5p**						2.43	0.01	1.00	−7.92	
Cs6g14320	0	902	0.04	32	4					Abhydrolase domain-containing protein 11
**csi-miR394**						0.35	1.72	0.98	2.31	
Cs7g10850	0	1168	0	85	1					*F*-box family protein
csi-miR399e						0.72	2.72	0.99	1.91	
Cs2g06030	2	1077	0.03	13	3					UBC24
**csi-miR5227**						0.01	6.27	1.00	9.29	
Cs3g22200	0	55	0.02	8	3					50S ribosomal protein L18
**csi-miR530b**						1.63	0.79	0.86	−1.05	
Cs9g08500	0	1784	0	20	1.5					Hypothetical protein VITISV_041073
orange1.1t02948	2	2768	0.04	5	2.5					PWD
**csi-miR535**						8.01	17.85	0.96	1.16	
Cs6g07930	0	1353	0	17	2.5					Serine/Threonine-protein kinase HT1

**Table 3:** **Targets of the novel miRNAs identified in MT and WT**.

**miRNA/Target**	**Category**	**Cleavage**	**Abundance**	**Alignment**	**RPM**		**Prob**.	**log_2_ (MT/WT)**	**Target**
**gene**		**position**		**score**					**annotation**
					**WT**	**MT**			
**csi-miRN02**					31.04	26.06	0.41	−0.25	
Cs9g17500	0	2474	15	4.5					Cleavage and polyadenylation specificity factor subunit 2
**csi-miRN03**					1533	2136	1.00	0.48	
Cs8g13560	0	302	434	2					Unknown protein
**csi-miRN04**					2.89	3.56	0.33	0.30	
Cs5g05430	2	549	22	3					Peroxisomal membrane protein PEX14
**csi-miRN06**					71.74	46.56	0.86	−0.62	
Cs4g20380	0	2650	71	4.5					Transcriptional corepressor LEUNIG
**csi-miRN12**					0.01	54.57	1.00	12.41	
Cs3g22150	0	1030	15	4.5					Oxidoreductase, putative
Cs1g11750	1	1782	8	4.5					30S ribosomal protein S15
**csi-miRN13**					0.01	40.54	1.00	11.98	
orange1.1t03349	0	1447	25	4.5					ABC transporter-like [*Arabidopsis thaliana*]
**csi-miRN14**					20.01	14.91	0.55	−0.42	
Cs7g10090	0	31	3	3.5					Unknown protein
**csi-miRN18**					37.36	0.01	1.00	−11.87	
Cs4g07790	3	773	5	4.5					Probable amino acid permease 7 [Vitis vinifera]
**csi-miRN19**					0.99	0.01	NA	−6.63	
orange1.1t00200	2	1514	174	0.5					SCL6 [Citrus trifoliata]
orange1.1t00199	2	1615	174	0.5					SCL6 [Citrus trifoliata]
Cs5g08980	0	1700	110	0.5					SCL15
**csi-miRN21**					3.29	0.01	1.00	−8.36	
Cs6g02130	2	1054	6	2					cc-nbs-lrr resistance protein [*Populus trichocarpa*]
Cs6g02120	2	913	6	2					Disease resistance protein RFL1, putative
Cs6g02100	2	1054	6	2					PREDICTED: disease resistance protein At4g27190-like [Vitis vinifera]

**Table 4:** **Gene Ontology enrichment analysis of the target genes of miRNAs differentially expressed between MT and WT**.

**Gen ontology (GO) terms**	**Term ID**	**Corrected *P*-value**	**Gene number**	**Gene list**
**BIOLOGICAL PROCESS**				
Xylem and phloem pattern formation	GO:0010051	0.0019	4	Cs2g09770 |Cs7g10850 |Cs8g16510 |Cs1g15640
Regulation of actin filament-based process	GO:0032970	0.0019	4	Cs2g09770 |Cs4g19310 |Cs8g16510 |Cs1g15640
Positive regulation of cellular component biogenesis	GO:0044089	0.0019	4	Cs2g09770 |Cs4g19310 |Cs8g16510 |Cs1g15640
Regulation of cytoskeleton organization	GO:0051493	0.0020	4	Cs2g09770 |Cs4g19310 |Cs8g16510 |Cs1g15640
Integument development	GO:0080060	0.0023	2	Cs2g09770 |Cs1g15640
Actin filament-based process	GO:0030029	0.0023	5	Cs6g07930 |Cs2g09770 |Cs4g19310 |Cs8g16510 |Cs1g15640
Embryonic meristem development	GO:0048508	0.0034	3	Cs2g09770 |Cs4g19310 |Cs8g16510
Regulation of cellular component biogenesis	GO:0044087	0.0035	4	Cs2g09770 |Cs4g19310 |Cs8g16510 |Cs1g15640
Trichome morphogenesis	GO:0010090	0.0035	4	Cs2g09770 |Cs4g19310 |Cs8g16510 |Cs1g15640
Regulation of anatomical structure size	GO:0090066	0.0050	4	Cs2g09770 |Cs4g19310 |Cs8g16510 |Cs1g15640
Phyllome development	GO:0048827	0.0050	8	Cs7g10990 |Cs1g15640 |Cs7g10830 |Cs1g11750 |Cs2g09770 |Cs1g06080 |Cs7g10850 |Cs8g16510
Developmental maturation	GO:0021700	0.0416	4	Cs2g09770 |Cs4g19310 |Cs8g16510 |Cs1g15640
Regulation of growth	GO:0040008	0.0448	4	Cs2g09770 |Cs3g06390 |Cs8g16510 |Cs1g15640
**MOLECULAR FUNCTION**				
Nucleic acid binding transcription factor activity	GO:0001071	0.0061	11	Cs7g11770 |Cs7g10990 |Cs1g03470 |Cs1g15640 |Cs7g10830 |Cs2g09770 |Cs1g06080 |Cs4g19310 |Cs3g06390 |Cs2g23550 |Cs8g16510
Sequence-specific DNA binding transcription factor activity	GO:0003700	0.0061	11	Cs7g11770 |Cs7g10990 |Cs1g03470 |Cs1g15640 |Cs7g10830 |Cs2g09770 |Cs1g06080 |Cs4g19310 |Cs3g06390 |Cs2g23550 |Cs8g16510
ADP binding	GO:0043531	0.0355	3	Cs6g02120 |Cs6g02100 |Cs6g02130
Lipid binding	GO:0008289	0.0368	4	Cs2g09770 |Cs4g19310 |Cs8g16510 |Cs1g15640
DNA binding	GO:0003677	0.0422	11	Cs7g11770 |Cs7g10990 |Cs1g03470 |Cs1g15640 |Cs7g10830 |Cs2g09770 |Cs4g19310 |Cs3g06390 |Cs2g23550 |Cs8g16510 |Cs9g08500

As shown in Table 2, csi-miR156k was strongly down-regulated in MT. The targets of miR156k are four *SPL* genes, including *SPL4* (Cs2g23550), *SPL2* (Cs7g10830), *SPL6* (Cs7g11770), and *SPL12* (Cs7g10990), which are important transcription factors during plant development (Manning et al., 2006; Wang et al., 2009; Miura et al., 2010; Gou et al., 2011). Csi-miR159 also targeted four genes, including two *GAMYBs* (Cs1g03470 and Cs3g06390) which play a central role during fruit ripening of strawberry (Vallarino et al., 2015). *ATHB8/14/15* (Cs4g19310, Cs2g09770, and Cs1g15640) were the targets of csi-miR166d and are involved in vascular development and auxin signaling (Ohashi-Ito and Fukuda, 2003; Baima et al., 2014). Csi-miRN21 targeted three disease resistance proteins. These results indicated that the targets of one miRNA had similar function (Table 3).

### Identification of phasiRNAs and *PHAS* genes

From the four deep sequencing sRNA libraries of MT and WT, we were able to identify 205 *PHAS* genes (176 *PHAS* genes from MT and 154 *PHAS* genes from WT), which were capable of secondary siRNA production with a *P* ≤ 0.0001 (Table S6). Most of these 205 *PHAS* genes encoded resistance proteins, and the number of phasiRNAs produced from each *PHAS* gene ranged from 3 to 22 (Table S6). From the degradome data, 66 *PHAS* genes targeted by 9 miRNAs were identified (Table S7). As shown in Table S7, the miR482 family (csi-miR472, csi-miR482a-3p, csi-miR482b, and csi-miR482c) targeted 53 *PHAS* genes to generate phasiRNAs, and most of these target *PHAS* genes were *NB-LRR* (nucleotide binding leucine-rich repeat) genes that encode disease resistance proteins, which was consistent with previous studies (Wu et al., 2015; Xia et al., 2015a). In addition, csi-miR3950 was predicted to trigger NAC domain-containing proteins, csi-miR393h triggered two TIR/AFB auxin receptor proteins, csi-miR167a triggered *ARF8* and csi-miR1515 triggered two dicer-like proteins.

### Comparison of distinct expression patterns of miRNAs and their targets during fruit development of MT and WT

**Figure 4:**
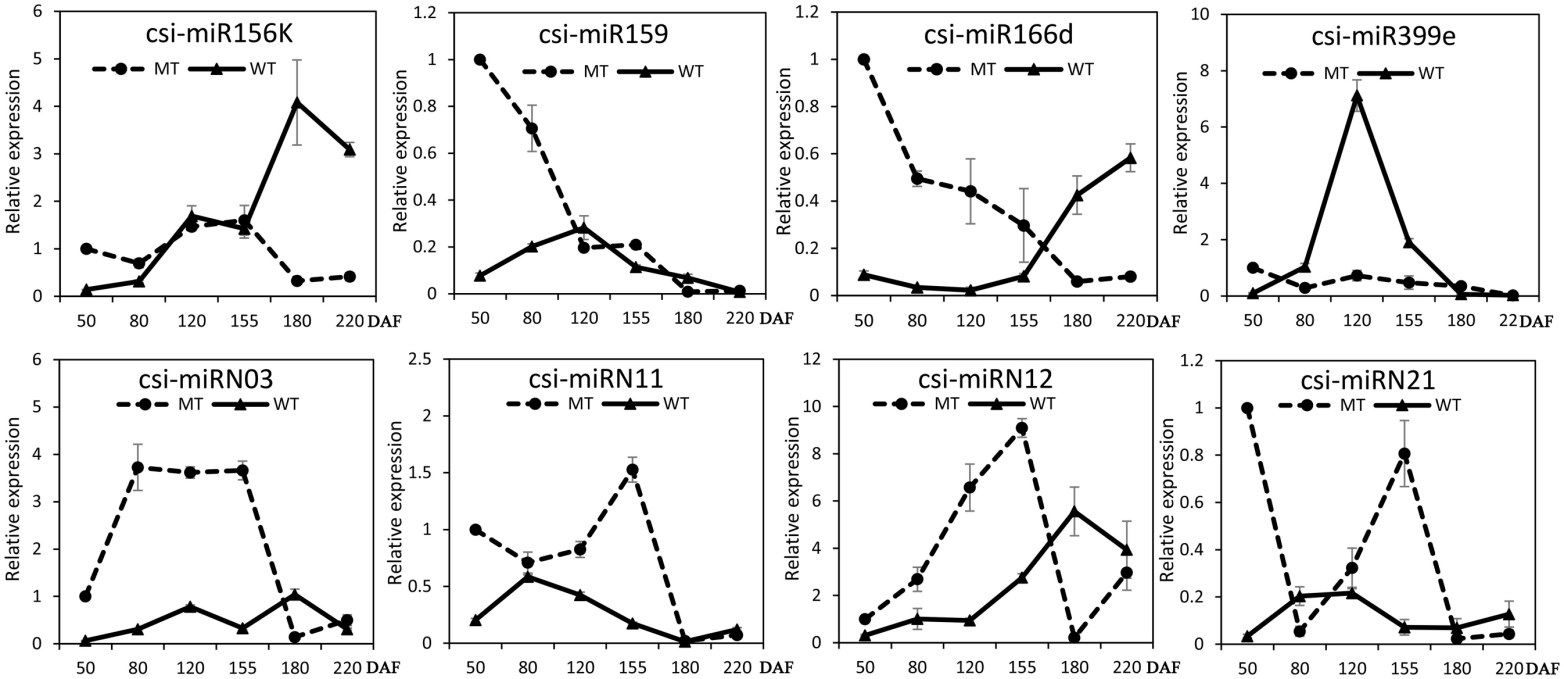
**Expression patterns of several candidate miRNAs in MT and WT during fruit development and ripening**. The relative expression levels were obtained from stem-loop qRT-PCR.

The expression patterns of the miRNAs (including four known miRNAs and four novel miRNAs) that showed differential expression between MT and WT or high expression levels during fruit ripening were validated and compared by stem-loop qRT-PCR in the fruit pulp of MT and WT during six developmental stages (50, 80, 120, 155, 180, and 220 DAF). As shown in Figure 4, these eight miRNAs all showed differential expression patterns between MT and WT. Csi-miR156k was remarkably up-regulated after the 155 DAF stage in WT; however, it was dramatically down-regulated in MT after 155 DAF. The expression patterns of csi-miR159 differed in MT and WT before the 120 DAF stage. Notably, csi-miR166d displayed opposing expression in MT and WT. Csi-miRN03 and csi-miRN11, two novel miRNAs, had low CT values (data not shown) and were highly expressed in the fruits of WT and MT (Table S2). They also showed distinct expression patterns between MT and WT.

**Figure 5:**
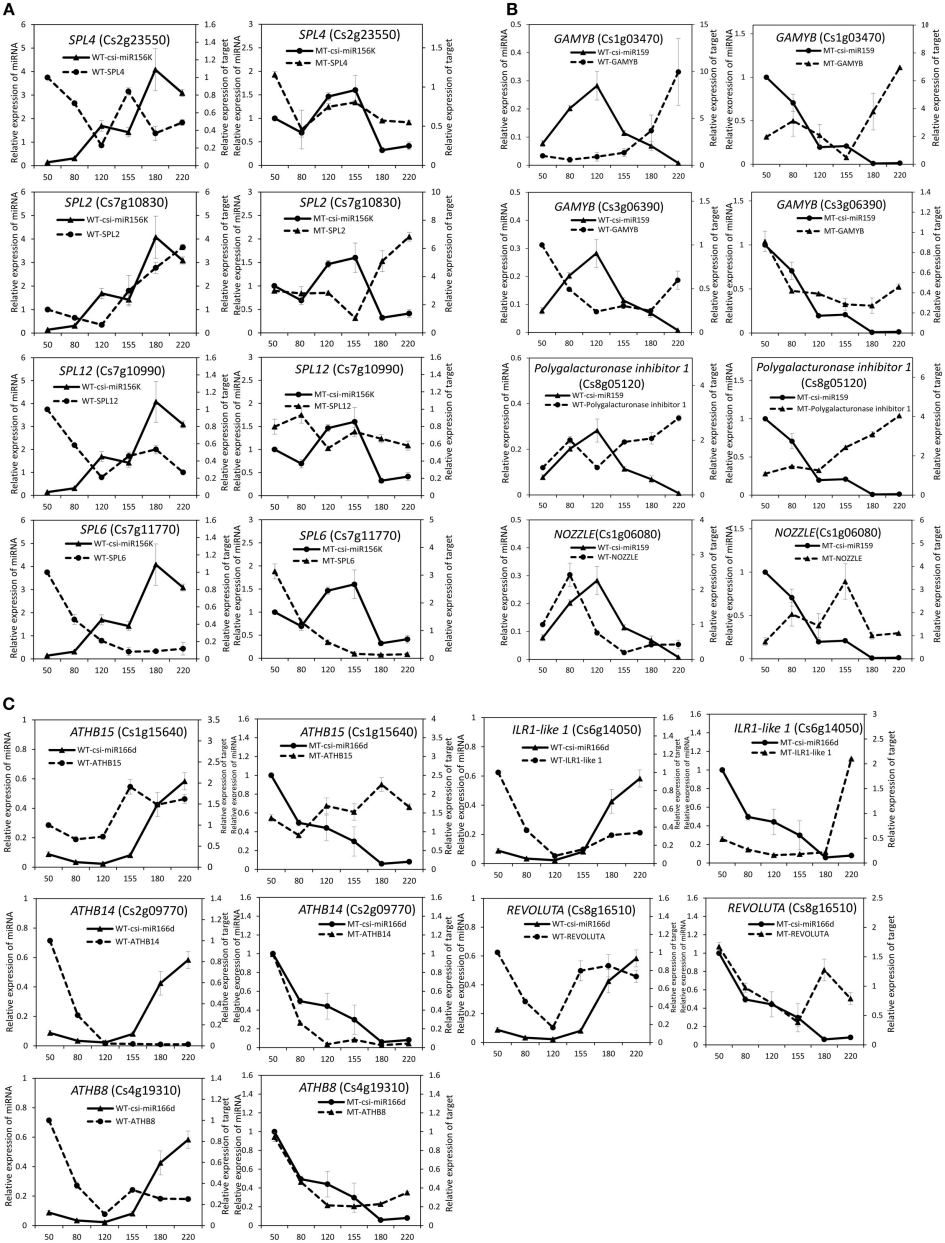
**Expression patterns of known miRNAs and their target genes in MT and WT during fruit development and ripening**. **(A)**, csi-miR156k; **(B)**, csi-miR159; **(C)**, csi-166d.

**Figure 6:**
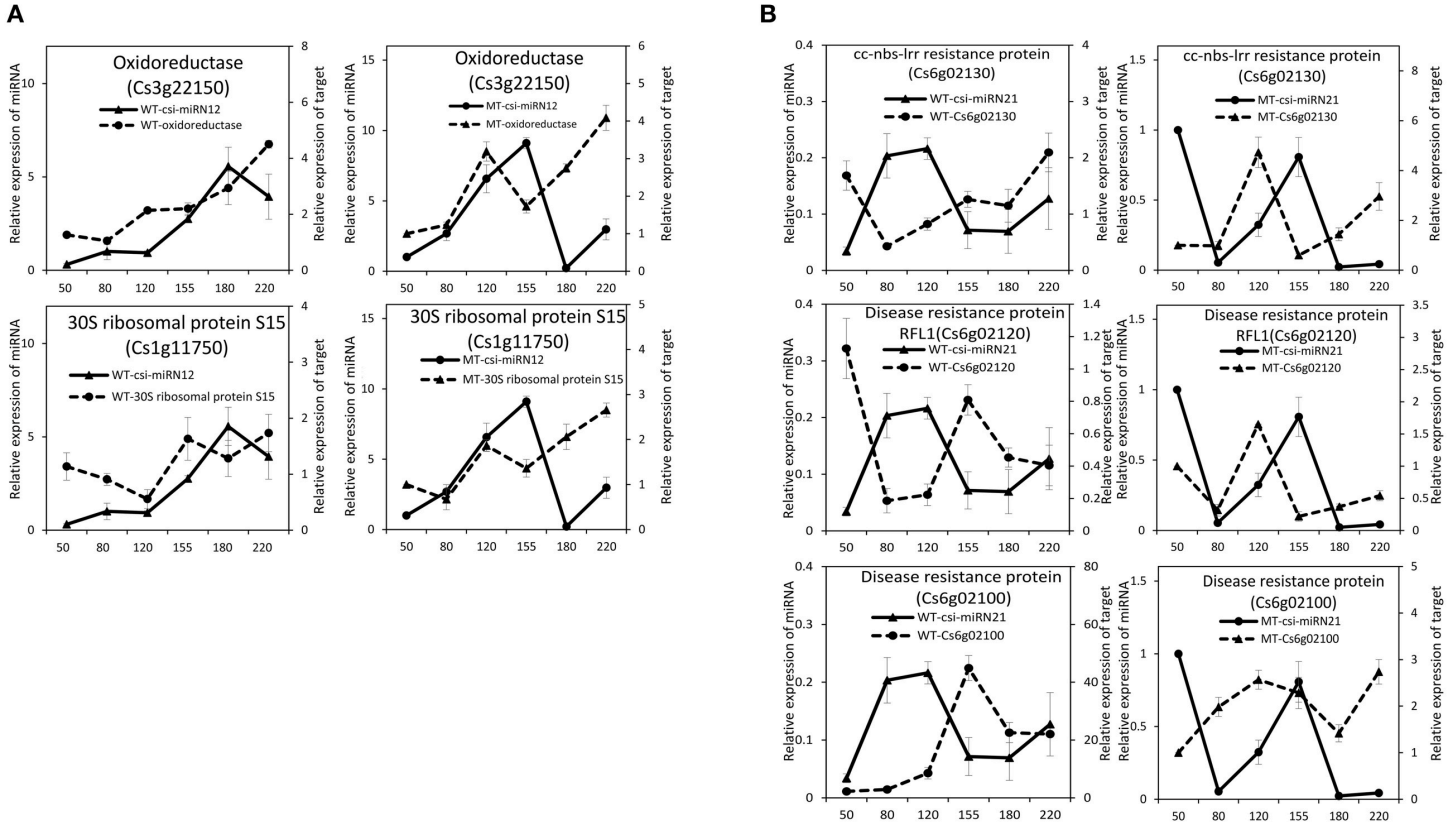
**Expression patterns of novel miRNAs and their target genes in MT and WT during fruit development and ripening**. **(A)**, csi-miRN12; **(B)**, csi-miRN21.

To validate the regulatory function of the miRNAs, the expression patterns of 18 target genes of several candidate miRNAs were also analyzed by qRT-PCR in the fruit pulp of MT and WT during six developmental stages (Figures 5, 6). In our results, 14 of the18 target genes displayed different expression patterns between MT and WT; *SPL6, GAMYB* (Cs3g06390), *ATHB14*, and *ATHB8* showed similar expression patterns between MT and WT; and the expressions of most target genes were negatively correlated with their miRNAs. These results indicated that the miRNAs had significant effects on the expression of their target genes, which were also regulated by other factors, such as transcription factor. As shown in Figure 5A, the four *SPL* genes targeted by csi-miR156k showed different expression patterns during citrus fruit development; in WT, the expressions of *SPL4* and *SPL6* were negatively correlated with that of csi-miR156k. However, in MT, the expression of csi-miR156k was negatively correlated with those of *SPL2* and *SPL12*, indicating csi-miR156k regulated the expression of different genes in MT and WT. This phenomenon was also observed in other miRNA-targets groups (Figures 5, 6). These results indicated that miRNA-mediated silencing of target genes was under sophisticated regulation and within limits in citrus fruit.

## Discussion

### Identification and characterization of miRNAs and their targets in citrus fruits

In recent years, more and more miRNAs have been excavated in citrus (Xu et al., 2010; Zhang et al., 2012; Liu Y. et al., 2014; Wu et al., 2015). To date, 60 miRNAs from *C. sinensis* have been annotated in the miRBase database (release 21.0). However, there are 325 and 592 miRNAs uploaded to the miRBase database (release 21.0) from *Arabidopsis* and rice, respectively. Therefore, there are still a large number of novel miRNAs that needed to be identified in citrus. In this study, we identified 107 known miRNAs belonging to 53 families and 21 novel miRNAs from the fruit of navel orange (Tables S1, S2). Our study predominantly focused on the annotation of miRNAs in citrus fruit based on a later-ripening mutant (“Fengwan”) and its wild-type (“Fengjie 72-1”), with the aim of broadening our understanding of the regulation networks of miRNAs in citrus fruit ripening. To obtain high confidence miRNAs, two biological replicates of each sample were submitted for deep sequencing, and only the miRNAs detected in both two biological replicates were used. According to the results of distribution of different size smallRNAs (Figure 1) and amount of known and novel miRNAs identified in MT and WT (Figure S1), we found that the kinds of miRNAs did not show significant difference between MT and WT. Seventy-five percent (96/128) miRNAs were the same in MT and WT. Interestingly, two novel miRNAs (csi-miRN03 and csi-miRN11) were expressed at much higher levels than the known miRNAs in both MT and WT, indicating their importance in fruit ripening (Table S2).

Among the identified miRNAs, we found several differentially expressed miRNAs between MT and WT under a criterion (|log_2_(MT/WT)| ≥ 1 and prob. > 0.8). Only 14.95% (16/107) of the known miRNAs were differentially expressed between MT and WT; however, the ratio of differentially expressed novel miRNAs (38.10%) was much higher than that of known miRNAs. These results suggested that the known/conserved miRNAs were probably responsible for controlling the basic cellular and developmental processes, while the novel/non-conserved miRNAs were involved in the regulation of the specific or species-specific regulatory pathways and functions (Glazov et al., 2008).

Degradome sequencing or PARE has been applied to identify miRNA targets in many plants (Addo-Quaye et al., 2008; German et al., 2008; Liu, N. A. et al., 2014; Wu et al., 2015; Xia et al., 2015b). This method identifies authentic miRNA targets in a high-throughput manner. The RLM-RACE was generally used to evaluate the degradome results in many studies (Yang, X. et al., 2013; Liu, N. A. et al., 2014; Liu Y. et al., 2014) and showed that the miRNA targets identified by degradome sequencing were reliable. In addition, this approach can concurrently identify all cleavage products in the genome with a high sensitivity, compared with older techniques such as northern blotting or 5′ RACE (Xia et al., 2015b). In this study, we performed degradome sequencing and identified 225 target genes of 57 miRNAs (47 known miRNAs and 10 novel miRNAs; Table S3). However, there are still 60 known miRNAs and 11 novel miRNAs without identified targets in our results. There are two possible reasons for this result. For the miRNAs with low expression, the abundance of their cleaved targets might be too low to detect; for those miRNAs with high expression, they may primarily function by repressing translation of their targets.

**Figure 7:**
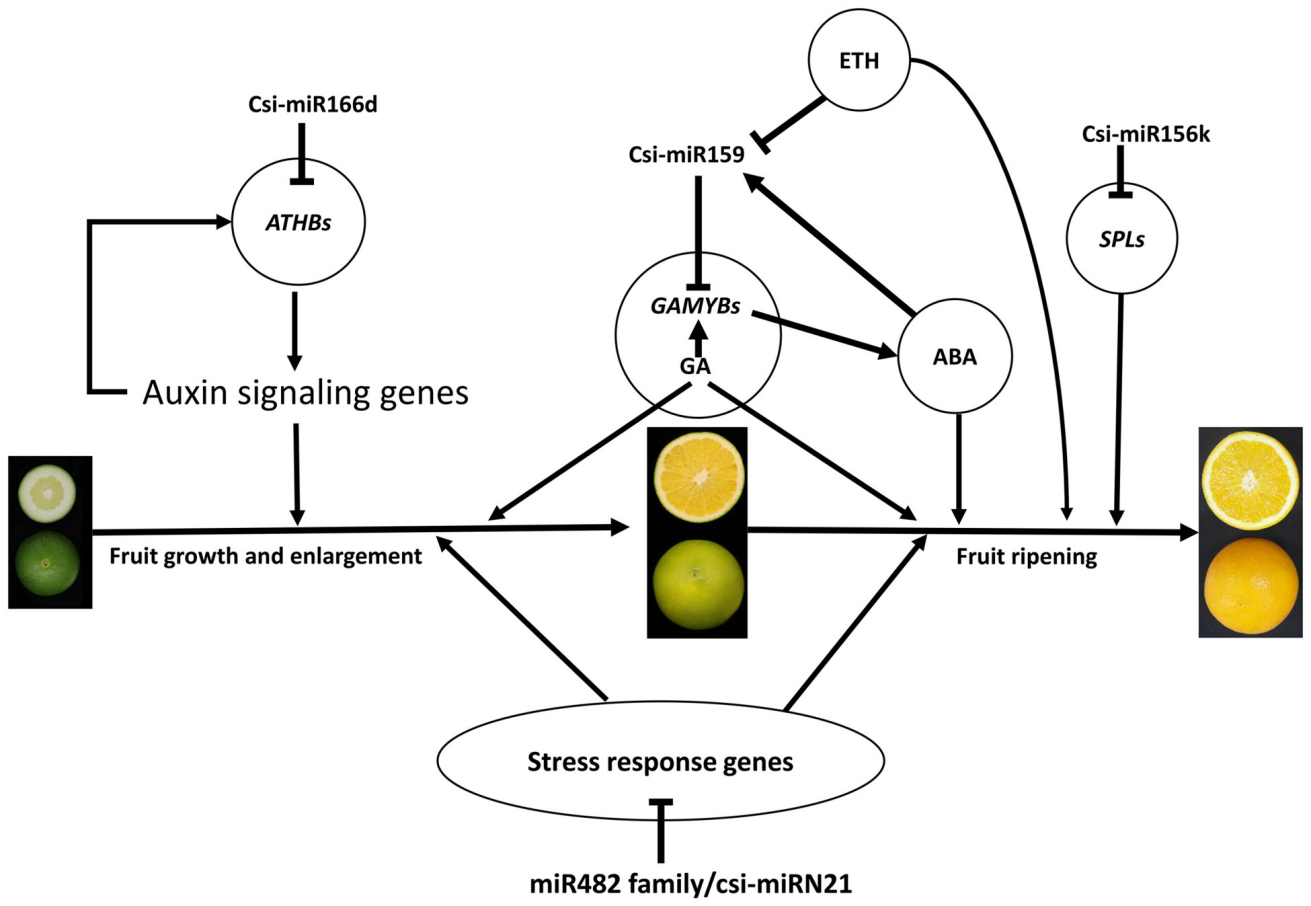
**A schematic model with the proposed roles of miRNAs involved in citrus fruit development and ripening**. ABA, abscisic acid; GA, gibberellic acid; ETH, ethylene.

Based on the results of this study, we generated an interaction diagram (Figure 7), which showed that the miRNAs may play important roles during citrus fruit development and ripening. Among these, csi-miR159-*GAMYBs* module may play a central role in citrus fruit ripening, and this regulation may function in combination with plant hormones, including ABA, gibberellin (GA), and ethylene (ETH). These data depict a picture of the regulation network involved in citrus fruit ripening at post-transcription level.

### The stress response may play a significant role during citrus fruit ripening

According to the GO enrichment analysis of the targets of miRNAs, the biological processes related to the stress response were significant enriched, such as the innate immune response, salicylic acid biosynthetic process, response to stimulus, and response to stress (Figure 3 and Table S5). In addition, most of identified *PHAS* genes encoded resistance proteins (Table S6). The abundantly expressed miR482 family triggered a massive release of phasiRNAs from transcripts of disease resistance proteins which included many *NB-LRR* genes (Table S7). *NB-LRR* genes are one of the first lines of defense against pathogen infection (Dangl and Jones, 2001; Meyers et al., 2005; Yang,S. H. et al., 2013). The miR482/miR2118 superfamily, a significant trigger of the *NB-LRR* phasiRNAs, serves as the master regulator of *NB-LRR* genes in many eudicots by targeting the region coding for the critical P-loop motif in the highly conserved NBS (nucleotide-binding site) domain (Zhai et al., 2011; Li et al., 2012). This miRNA mediated regulation spawns secondary phasiRNAs, a layer of control with potential roles in defense or symbiosis (Zhai et al., 2011). In our previous studies (Wu et al., 2014b; Zhang et al., 2014), several differentially expressed genes and proteins related to the stress response were also identified during citrus fruit ripening. For instance, seven differential abundance heat shock proteins and 11 differential expression heat shock protein transcripts were identified between “Fengjie 72-1” and “Fengwan” during fruit ripening in proteomic data and transcriptomic data, respectively (Wu et al., 2014b). In addition, several genes related to ascorbate and aldarate metabolism and jasmonic acid metabolism, which are involved in the stress response, were also identified to be differential expressed between citrus wild type and late-ripening mutant and/or among different development stages of citrus fruit (Wu et al., 2014b; Zhang et al., 2014). ABA was shown to be a significant regulator during fruit ripening in both climacteric and non-climacteric fruits and is also a significant regulator of the stress response (Zhang et al., 2009; Jia et al., 2011, 2013; Leng et al., 2014; Luo et al., 2014). Therefore, we proposed that the stress response process may play an important role during citrus fruit ripening, in which ABA may act as a junction between the stress response and the ripening process (Figure 7).

### Regulation networks of miRNAs in the ripening of citrus fruit

miR156 is a highly conserved and expressed miRNA family in the plant kingdom and has been shown to take part in the regulation of flower and fruit development by targeting the SQUAMOSA-promoter binding-like (*SPL*) family (Xing et al., 2013; Silva et al., 2014; Wang, 2014). In the present study, the miR156 family was highly expressed in citrus fruit, and csi-miR156k was significantly different between MT and WT (Figure 4 and Table S1). In *Arabidopsis*, miR156-targeted *SPL3* positively and directly regulates the MADS box genes *AP1, FUL*, and the central regulator of flowering *LEAFY* (Yamaguchi et al., 2009). Interestingly, *FUL* is a well-characterized regulator of cell differentiation during the early stages of *Arabidopsis* fruit development and *FUL-like* genes appear to play a role in fruit development in two basal eudicot *Papaveraceae* species by promoting normal development of the fruit wall during fruit maturation (Gu et al., 1998; Pabón-Mora et al., 2012). In tomato, the miR156-targeted *SlySBP* gene *CNR* (a *SPL* family member) acts as a crucial factor controlling fruit ripening (Manning et al., 2006). Over-expression of the *AtMIR156b* precursor in tomato cv. Micro-Tom led to the down-regulation of most *SlySBP* genes in the developing ovaries and an alteration of morphology, with fruits characterized extra carpels and ectopic structures (Silva et al., 2014). In the present study, four csi-miR156k-targeted *SPL* genes were identified, and the expression patterns of these genes were different between MT and WT during fruit development and ripening (Figure 5A). These results indicated that miR156 might play an important role in citrus fruit ripening (Figure 7).

In previous studies, miR159 was characterized to regulate the expressions of *GAMYB-like* genes at the post-transcriptional level and play significant roles in leaf, flower and seed maturation (Millar and Gubler, 2005; Tsuji et al., 2006; Reyes and Chua, 2007). In strawberry (*Fragaria* × *ananassa*), transient silencing of *FaGAMYB* using RNAi caused an arrest in the ripening of the receptacle and inhibited color formation; in addition, a reduction of ABA biosynthesis and sucrose content was also caused by silencing *FaGAMYB* which act upstream of the known regulator ABA (Vallarino et al., 2015). During *Arabidopsis* seed germination, ABA induces the accumulation of miR159, and over-expression of miR159 renders plants hyposensitive to ABA (Reyes and Chua, 2007). The *GAMYB* gene in barley is upregulated by the GA transduction pathway in both anthers and seeds (Murray et al., 2003) and over-expression of miR159 or disruption of the GA biosynthesis pathway delays flowering and reduces fertility (Achard et al., 2004; Cheng et al., 2004). In tomato fruit, Zuo et al. (2012) reported that the expression of miR159 is efficiently repressed by ethylene (ETH) treatment. Therefore, miR159, which regulates the spatiotemporal expression pattern of *GAMYB* genes, constitutes a major connection among at least three hormones—namely GA, ABA, and ETH (Curaba et al., 2014). In the present study, csi-miR159 was differentially expressed between MT and WT, and the targets of csi-miR159, including two *GAMYBs*, Polygalacturonase inhibitor 1 and *NOZZLE*, were also differentially expressed between MT and WT during fruit development and ripening (Table 2, Figures 4, 5). These results indicated that csi-miR159 may be an important regulator of citrus fruit development and ripening and may play a significant role in the formation of later-ripening trait in MT (Figure 7).

Moreover, in the present study, csi-miR166d and its targets were all differentially expressed between MT and WT (Figures 4, 5). The targets of csi-miR166d included three *ATHBs* genes and one HD-ZIP III protein REVOLUTA which are involved in regulation of auxin signaling (Baima et al., 2014). In previous studies, miR166 was reported to be a critical factor for vascular development (Kim et al., 2005) and played a role in regulating SAM formation and floral development (Jung and Park, 2007). Here, csi-miR166d might be involved in the regulation of citrus fruit development through interacting with auxin signaling pathway (Figure 7).

In conclusion, numerous miRNAs are expressed during citrus fruit ripening and are differentially regulated between MT and WT. Several significant miRNAs and targets were identified in this study, such as csi-miR156k, csi-miR159, csi-miR166d, csi-miRN21, *GAMYBs, SPLs*, and *ATHBs*. The identification of miRNAs and their target genes provide new clues for future investigation of the mechanisms that regulate citrus fruit ripening.

## Author contributions

Conceived and designed the experiments: HY, JW. Performed the experiments: JW, SZ, and GF. Analyzed the data: JW. Contributed reagents/materials/analysis tools: HY. Wrote the paper: JW, HY.

### Conflict of interest statement

The authors declare that the research was conducted in the absence of any commercial or financial relationships that could be construed as a potential conflict of interest.

## Supplementary material

Figure S1 **The venn diagram of the number of known miRNAs (A) and novel miRNAs (B) identified in MT and WT**.

Figure S2 **The distribution of pre-miRNAs in the ***Citrus sinensis*** genome. Precursors of novel miRNAs are shown in pink**. The chromosome/scaffold scale is shown at the left of the figure. The chromosomal locations of the pre-miRNAs are indicated.

Figure S3 **Predicted secondary structures of the known miRNAs**. The mature miRNA sequences are highlighted in blue and the miRNA^*^ sequences are highlighted in red.

Figure S4 **Predicted secondary structures of the novel miRNAs**. The mature miRNA sequences are highlighted in blue and the miRNA^*^ sequences are highlighted in red.

Figure S5 **T-plots of the miRNA targets**.

Figure S6 **Distribution of the identified miRNAs and their targets in the ***Citrus sinensis*** genome**. The arrows indicate target genes.

Table S1 **Summary of the known miRNAs in WT and MT**.

Table S2 **Summary of the novel miRNAs in WT and MT**.

Table S3 **Targets of the miRNAs identified using degradome sequencing**.

Table S4 **Annotations of all target genes**.

Table S5 **Gene ontology classification of miRNA targets**.

Table S6 *****PHAS*** genes and phasiRNAs identified in MT and WT**.

Table S7 **Identified ***PHAS*** genes in citrus fruit**.

Table S8 **Primers used in miRNA stem-loop RT-PCR and mRNA qRT-PCR**.

## Abbreviations

DAF: Days after flowering
MT_bio1/MT_bio2: Biological replicate 1/2 of MT
WT_bio1/WT_bio2: Biological replicate 1/2 of WT
phasiRNA: Phased secondary small interfering RNA
*PHAS* gene: The gene targeted by miRNAs to generate phasiRNAs
pre-miRNA: Precursor of miRNA
tasiRNA: *Trans*-acting siRNA
qRT-PCR: Quantitative real time transcription-polymerase chain reaction.
