# Supplementary figures and images for: Comparative Analysis of miRNAs and Their Target Transcripts between a Spontaneous Late-Ripening Sweet Orange Mutant and Its Wild-Type Using Small RNA and Degradome Sequencing
Source: Front Plant Sci. 2016 Sep 21;7:1416. doi: 10.3389/fpls.2016.01416 (PMC5030777; doi:10.3389/fpls.2016.01416)

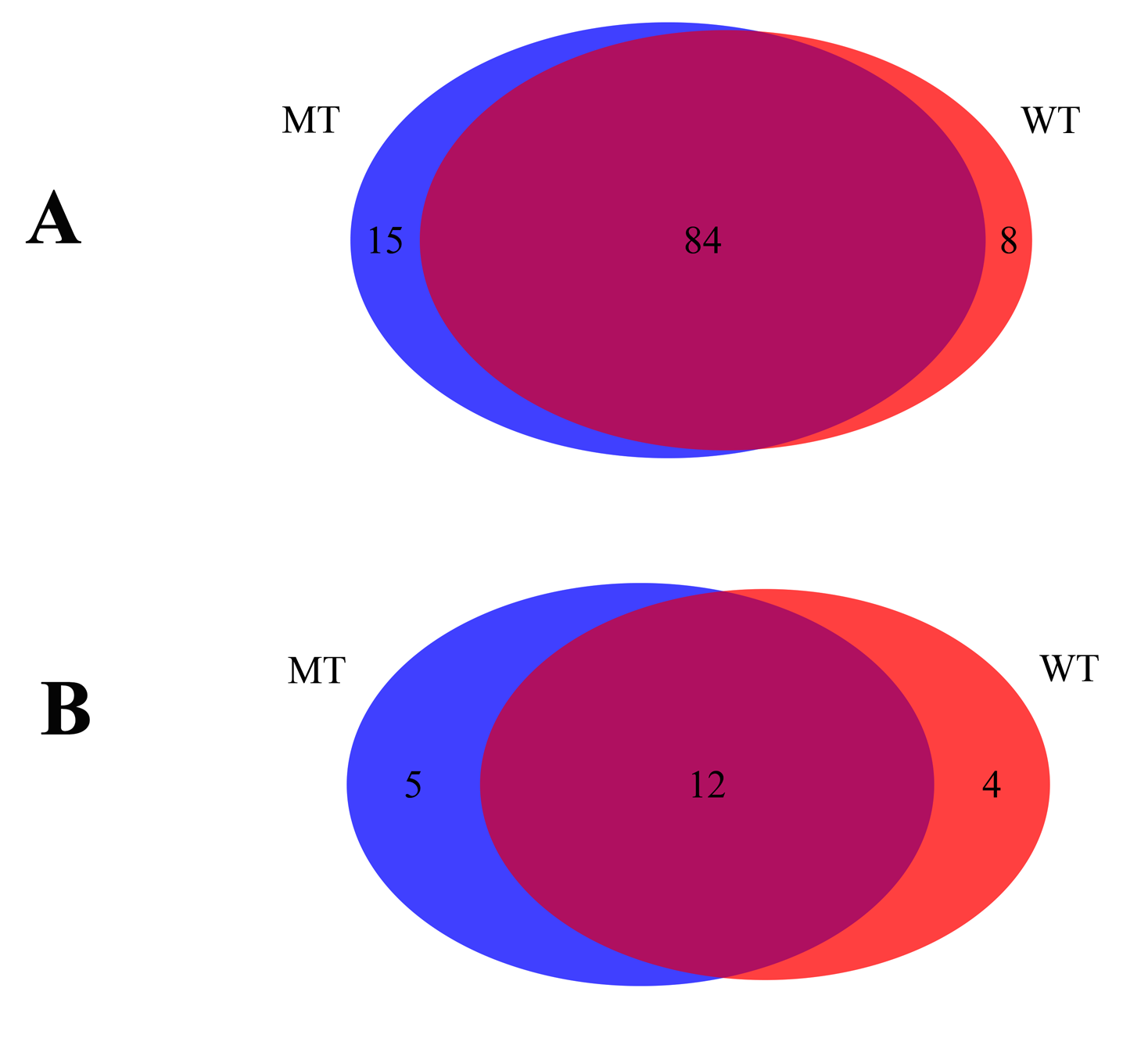

Supplement: Figure S1 — The venn diagram of the number of known miRNAs (A) and novel miRNAs (B) identified in MT and WT. [file Image1.TIF]

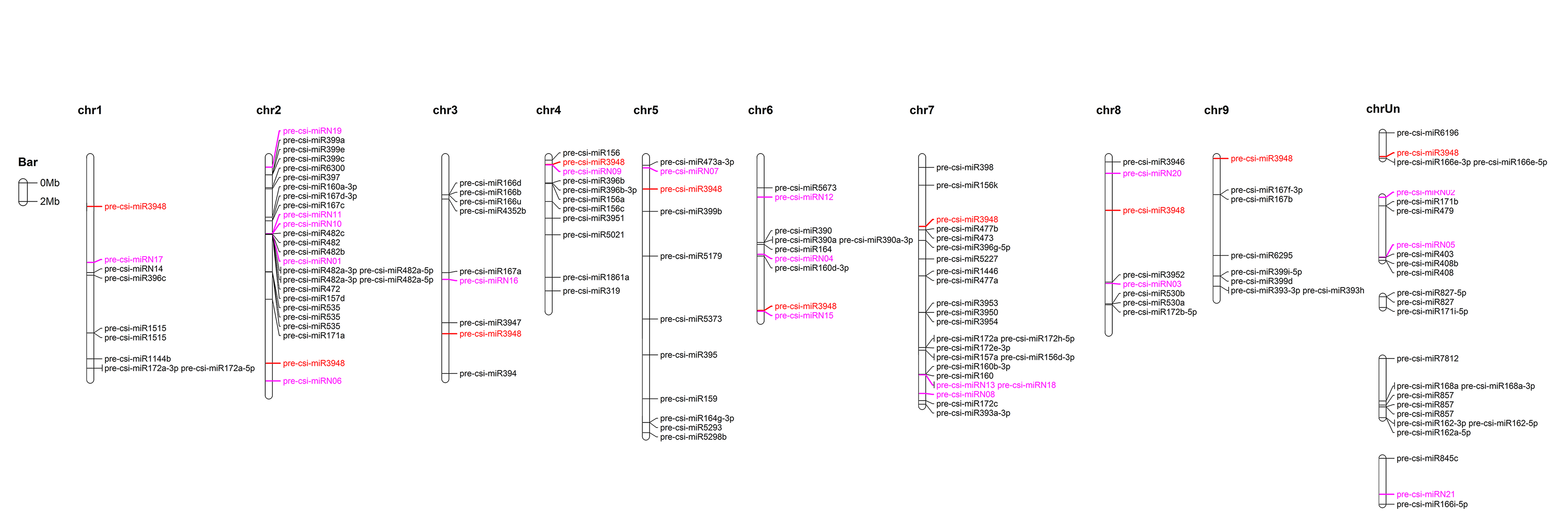

Supplement: Figure S2 — The distribution of pre-miRNAs in the Citrus sinensis genome. Precursors of novel miRNAs are shown in pink. The chromosome/scaffold scale is shown at the left of the figure. The chromosomal locations of the pre-miRNAs are indicated. [file Image2.TIF]

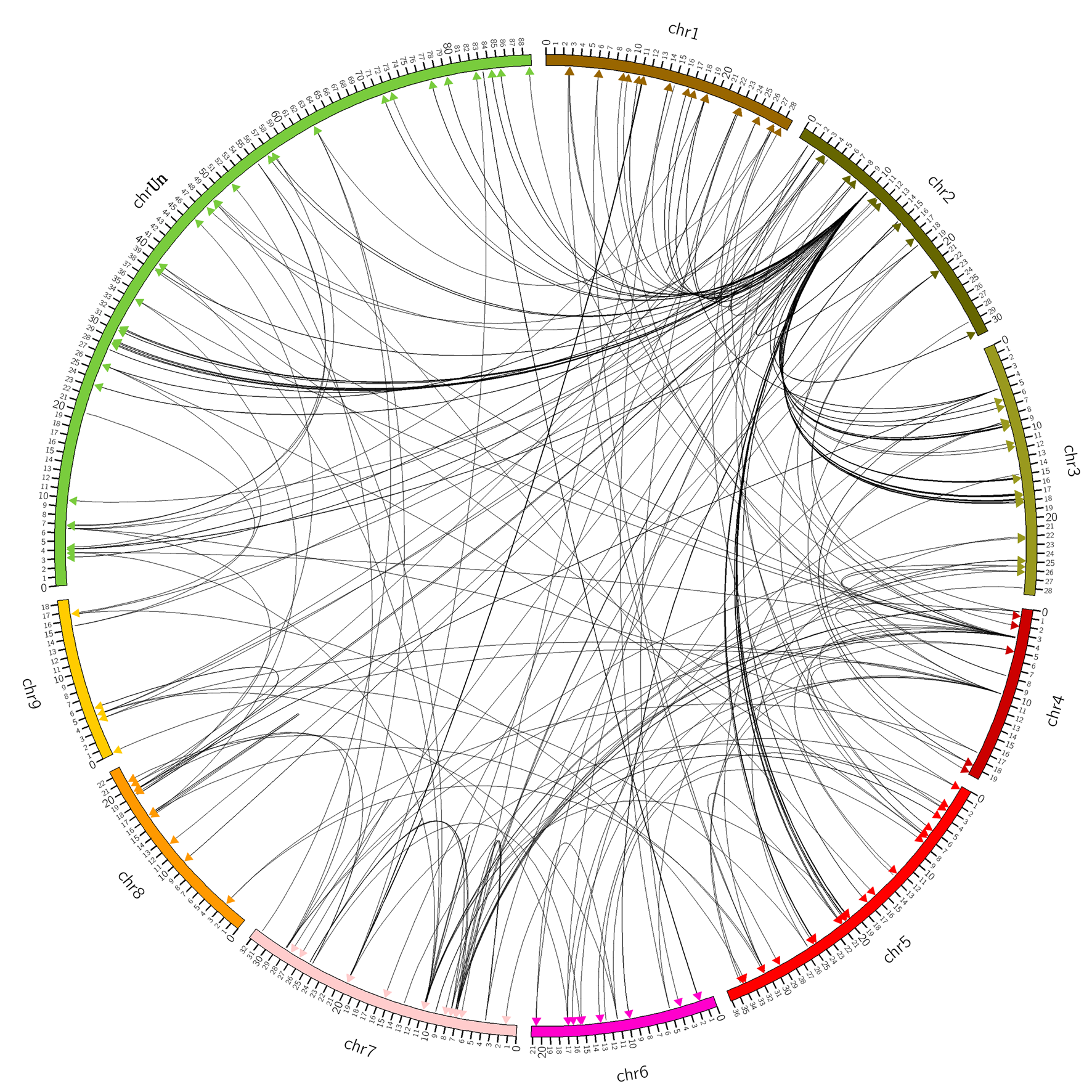

Supplement: Figure S6 — Distribution of the identified miRNAs and their targets in the Citrus sinensis genome. The arrows indicate target genes. [file Image6.TIF]
