# Supplementary material for: Comparative Analysis of miRNAs and Their Target Transcripts between a Spontaneous Late-Ripening Sweet Orange Mutant and Its Wild-Type Using Small RNA and Degradome Sequencing
Source: Front Plant Sci. 2016 Sep 21;7:1416. doi: 10.3389/fpls.2016.01416 (PMC5030777; doi:10.3389/fpls.2016.01416)

Secondary structure for csi-miR144b

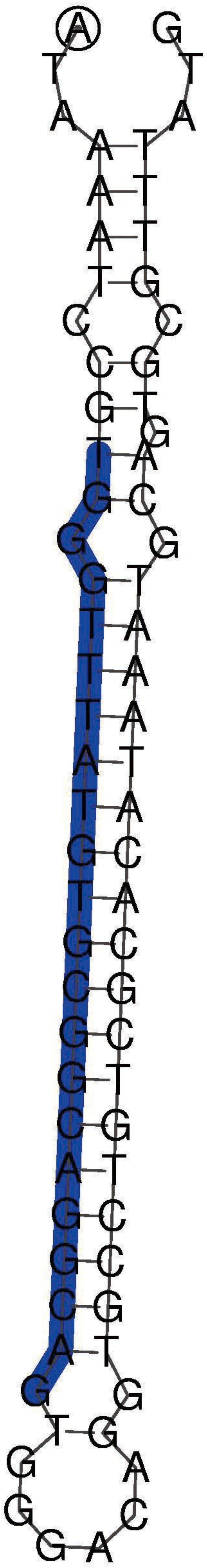

Secondary structure for csi-miR1446

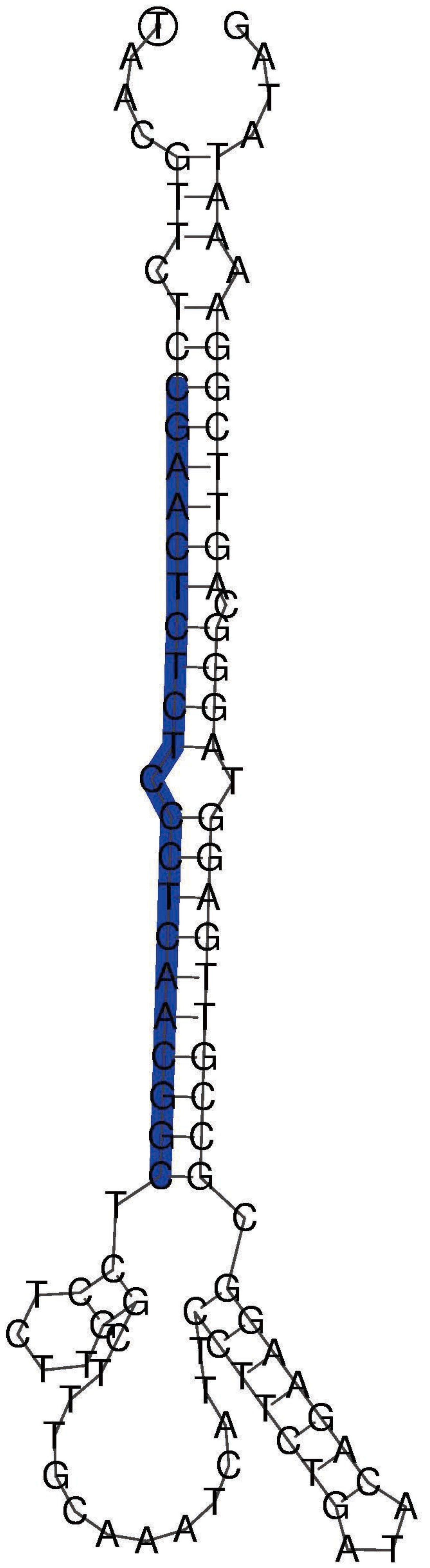

Secondary structure for csi-miR1515

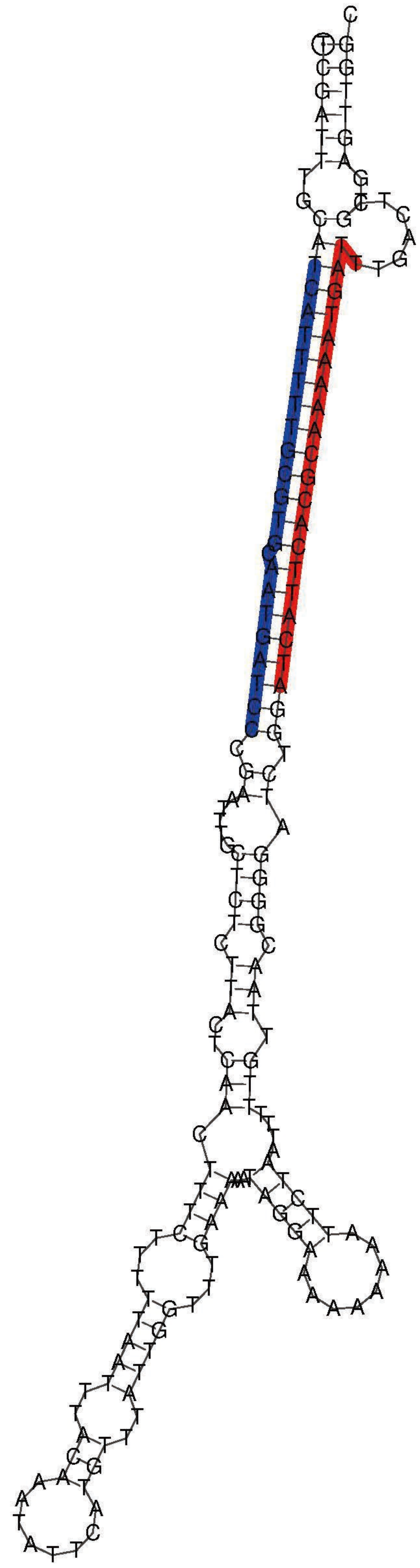

Secondary structure for csi-miR156

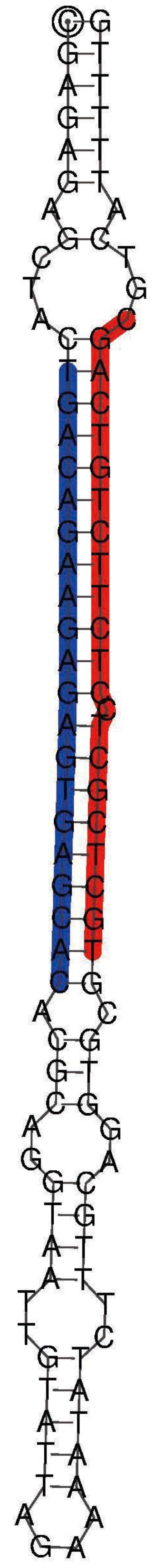

Secondary structure for csi-miR156a

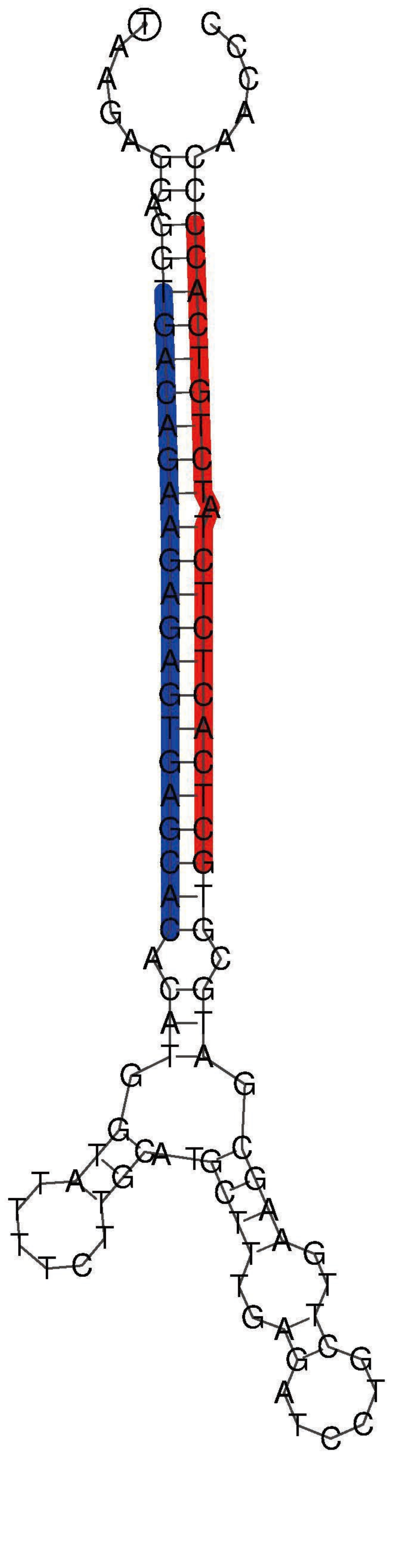

Secondary structure for csi-miR156c

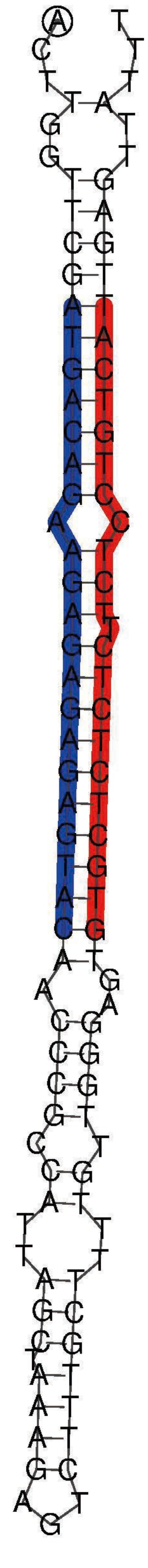

Secondary structure for csi-miR156d-3p

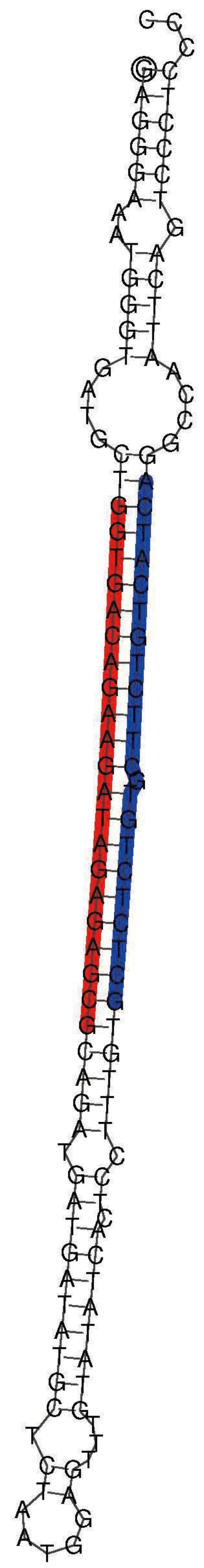

Secondary structure for csi-miR156k

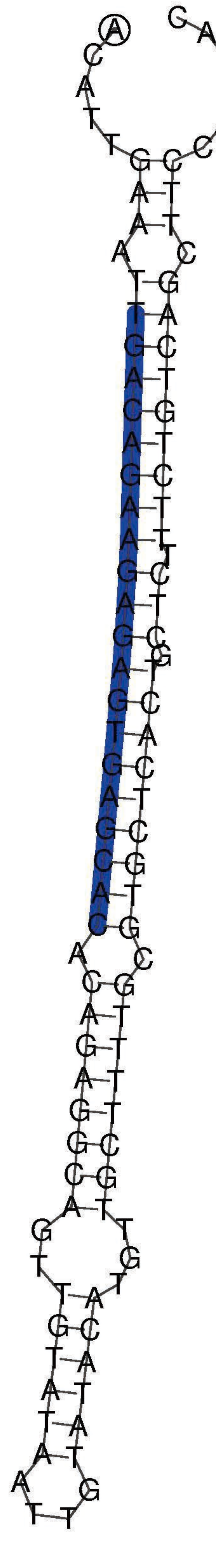

Secondary structure for csi-miR157a

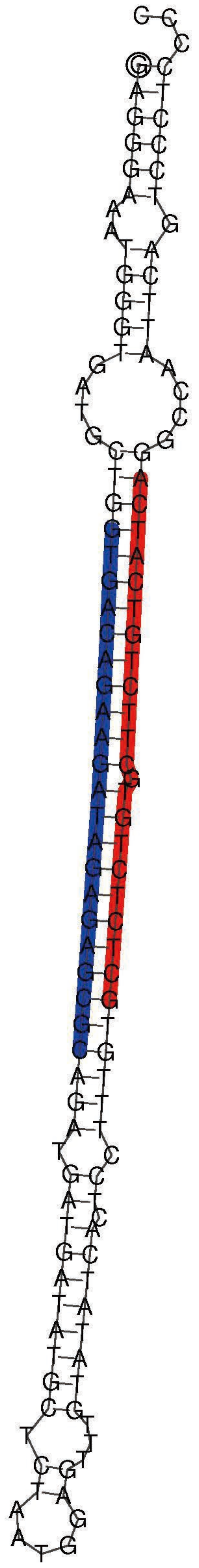

Secondary structure for csi-miR157d

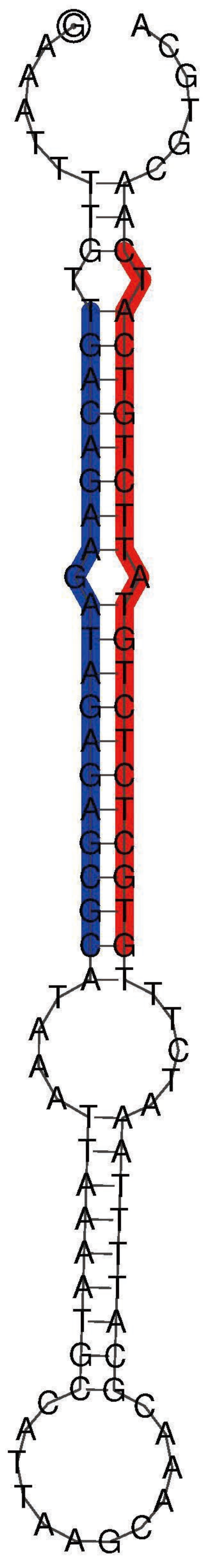

Secondary structure for csi-miR159

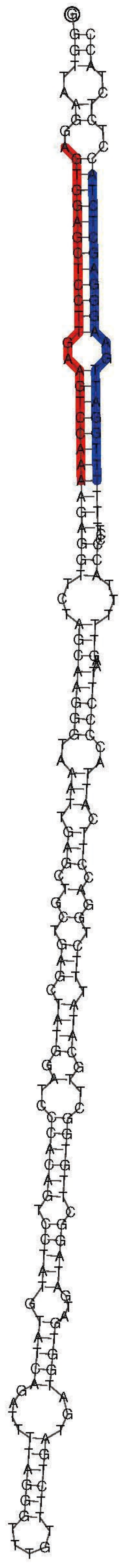

csi-miR159

Secondary structure for csi-miR160

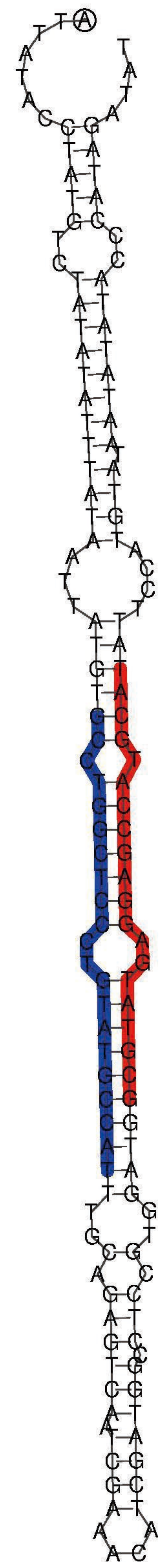

Secondary structure for csi-miR160a-3p

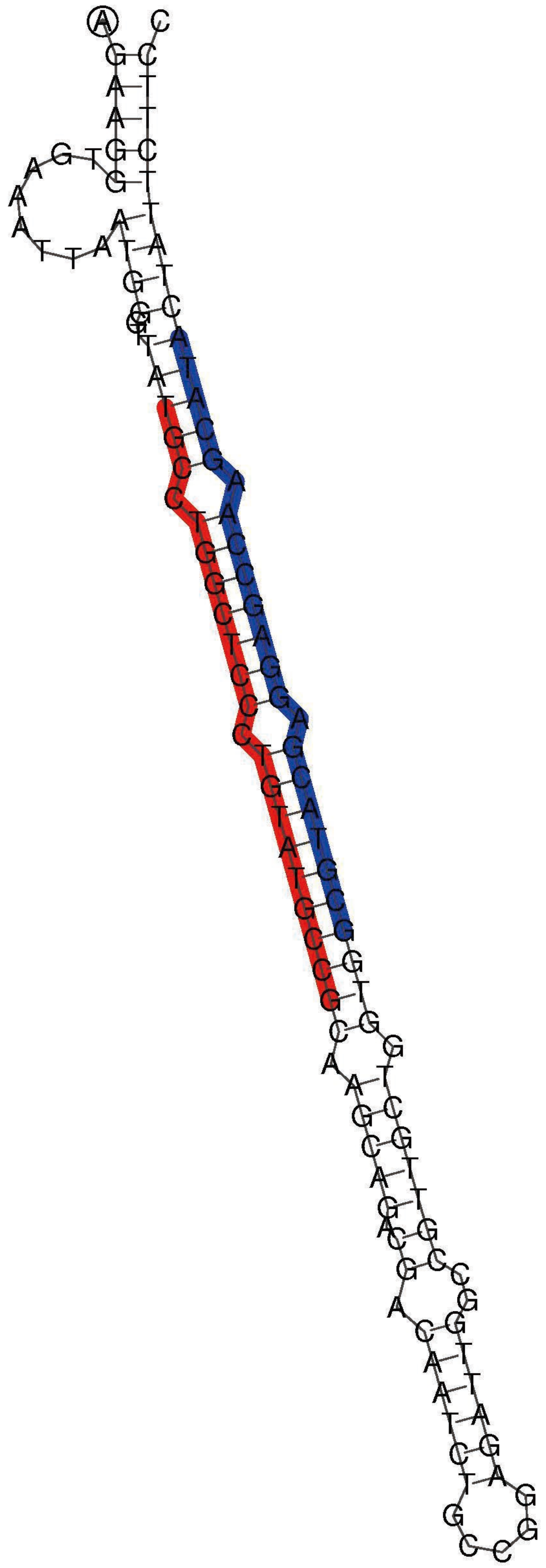

Secondary structure for csi-miR160b-3p

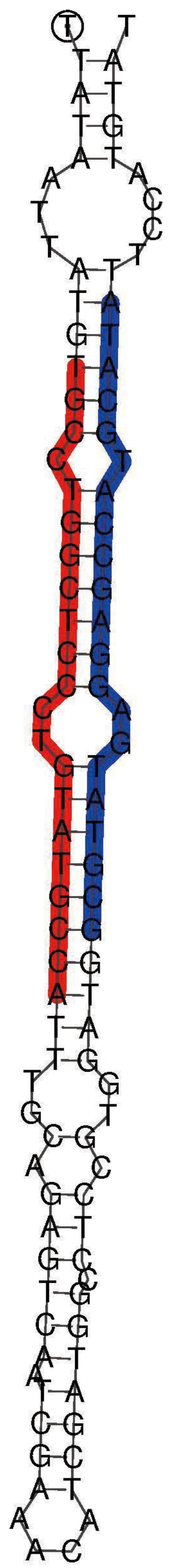

Secondary structure for csi-miR160d-3p

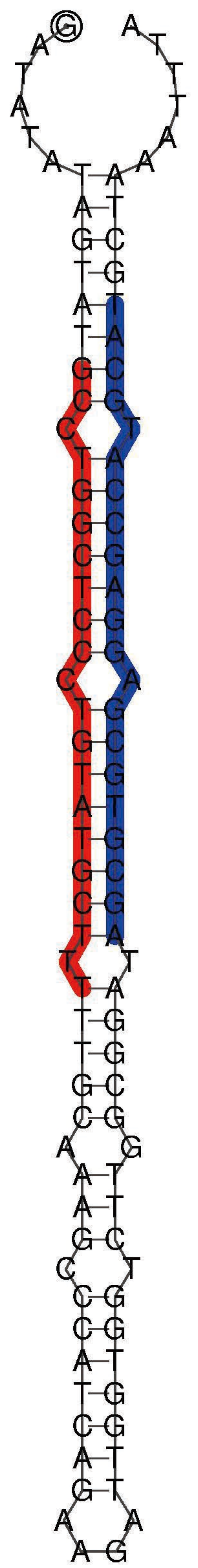

Secondary structure for csi-miR162-3p

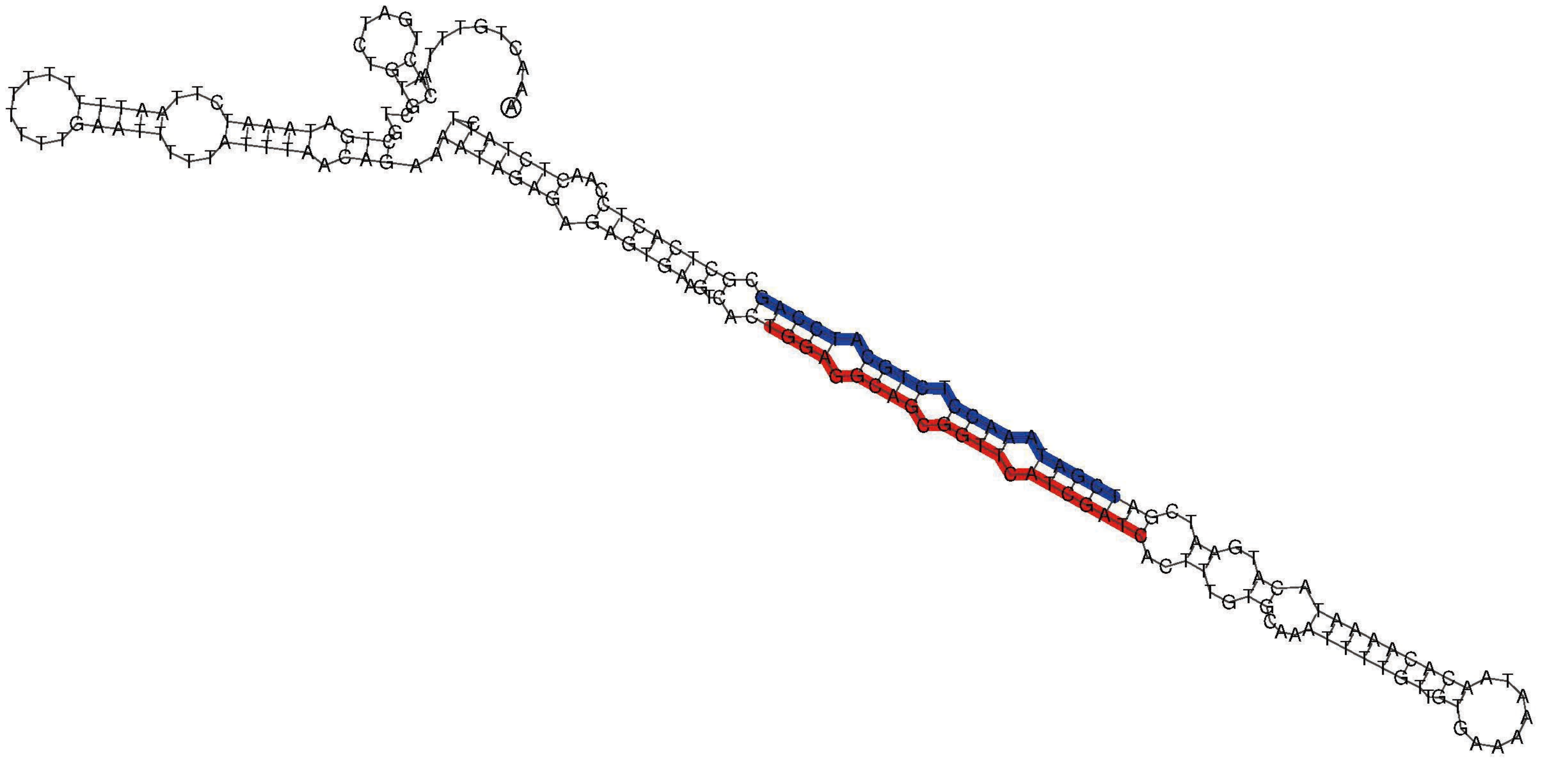

Secondary structure for csi-miR162-5p

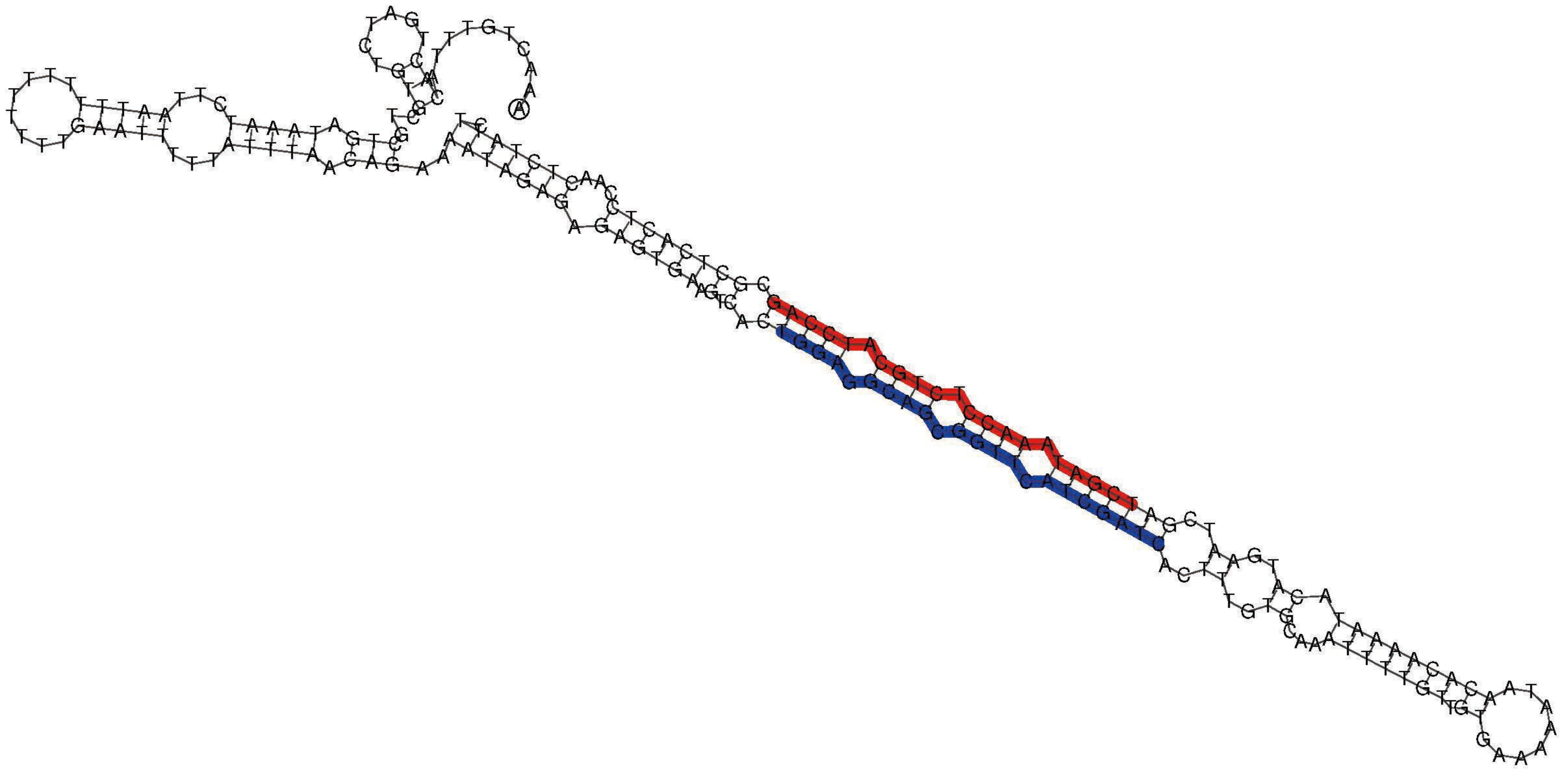

Secondary structure for csi-miR162a-5p

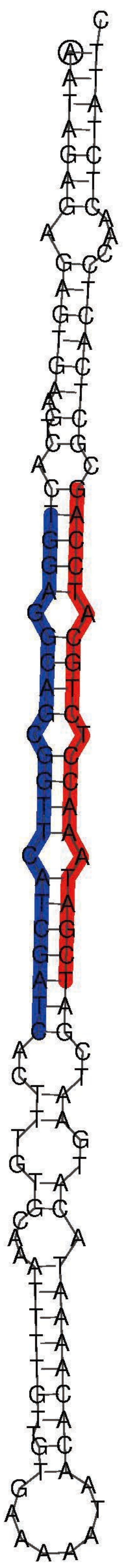

Secondary structure for csi-miR164

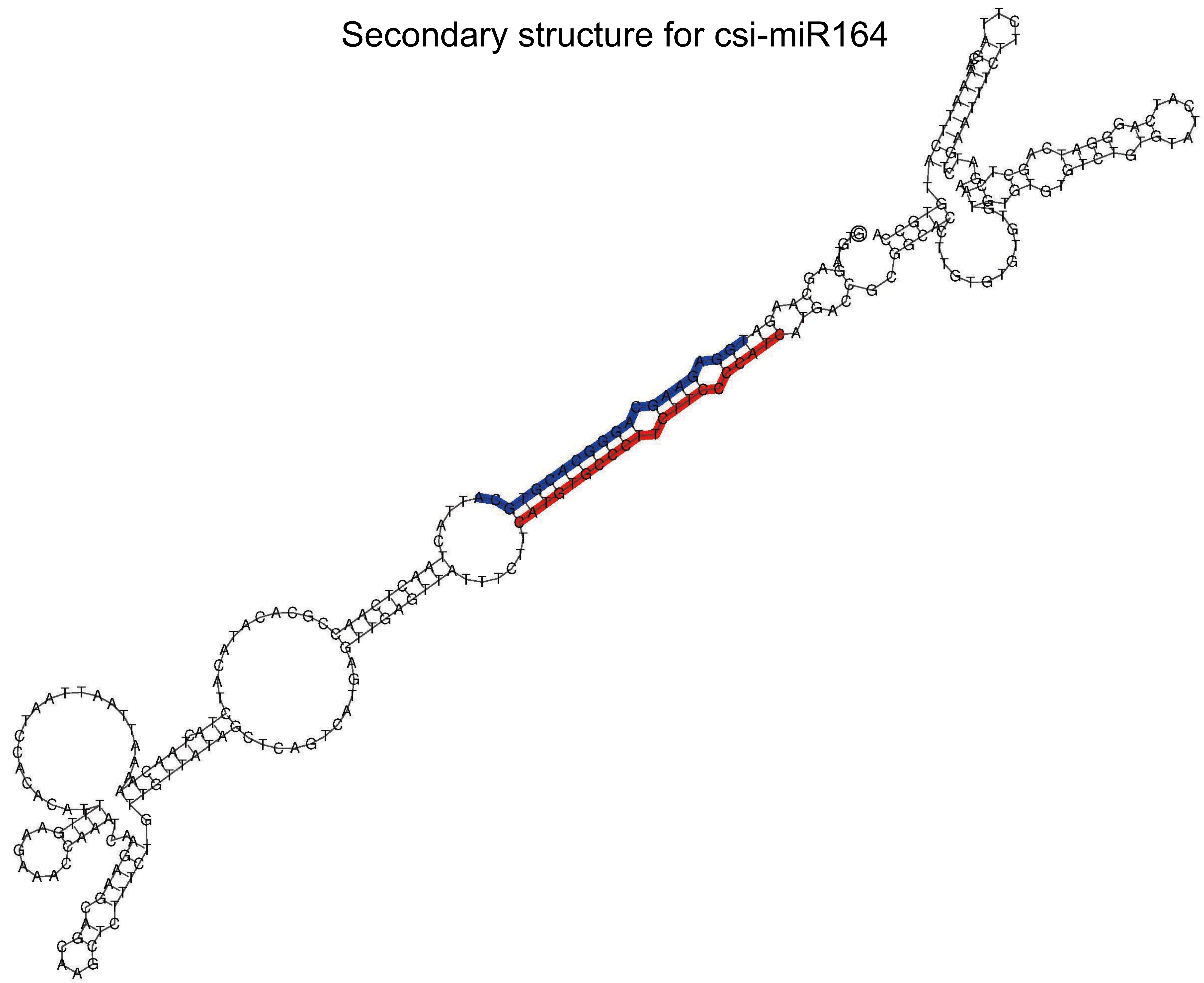

Secondary structure for csi-miR164g-3p

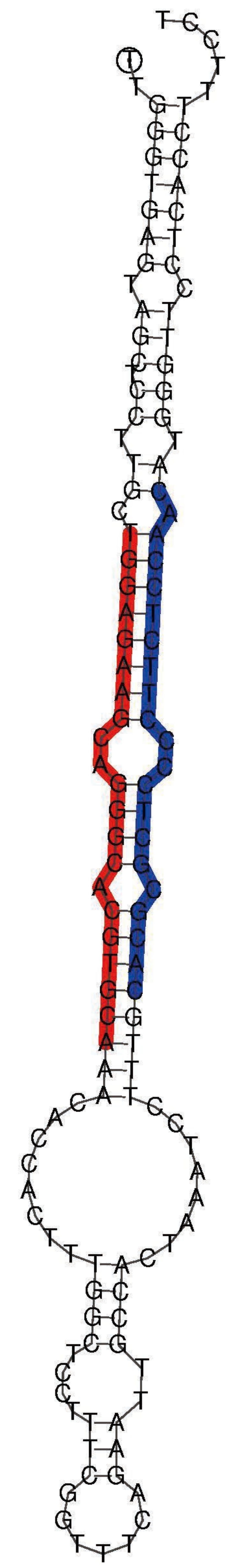

Secondary structure for csi-miR166b

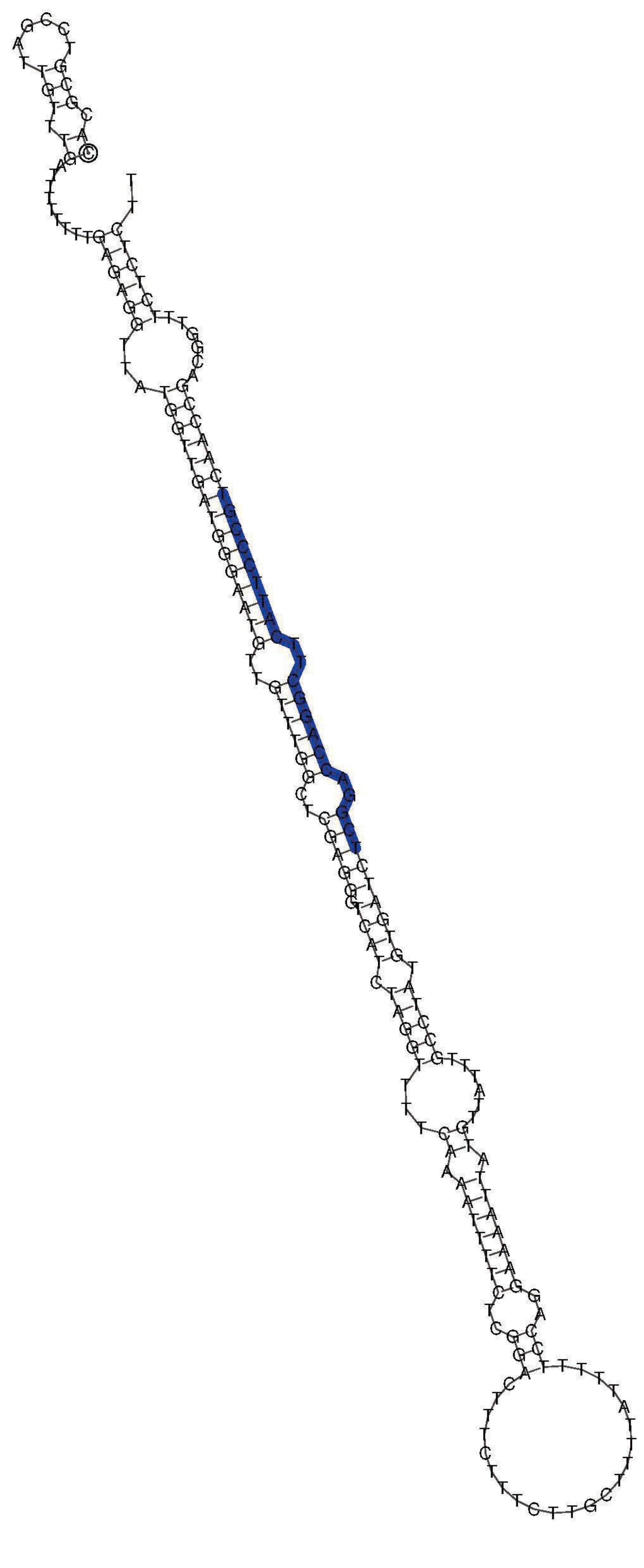

Secondary structure for csi-miR166d

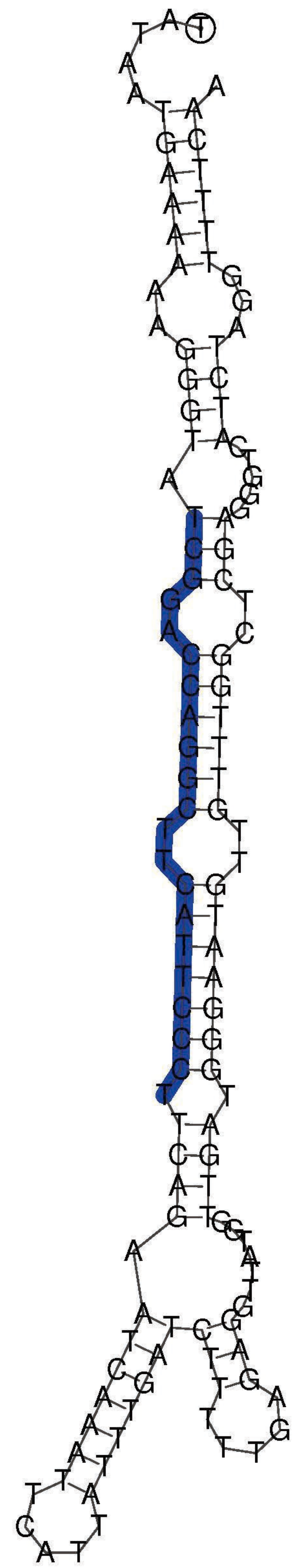

Secondary structure for csi-miR166e-3p

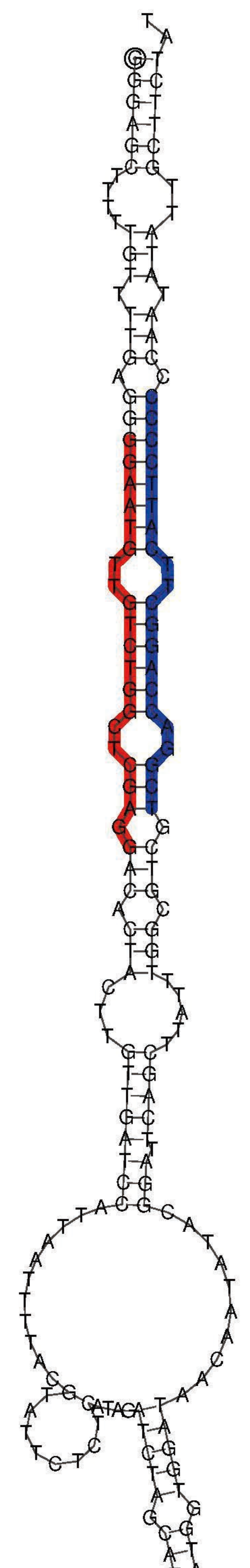

Secondary structure for csi-miR166e-5p

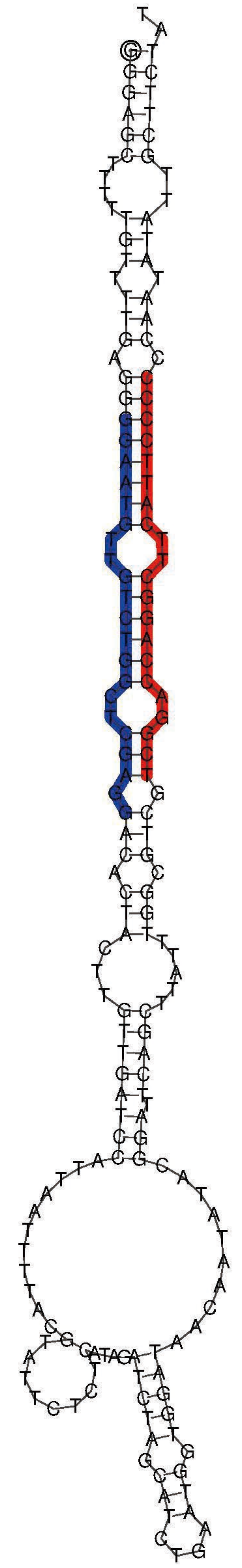

Secondary structure for csi-miR166i-5p

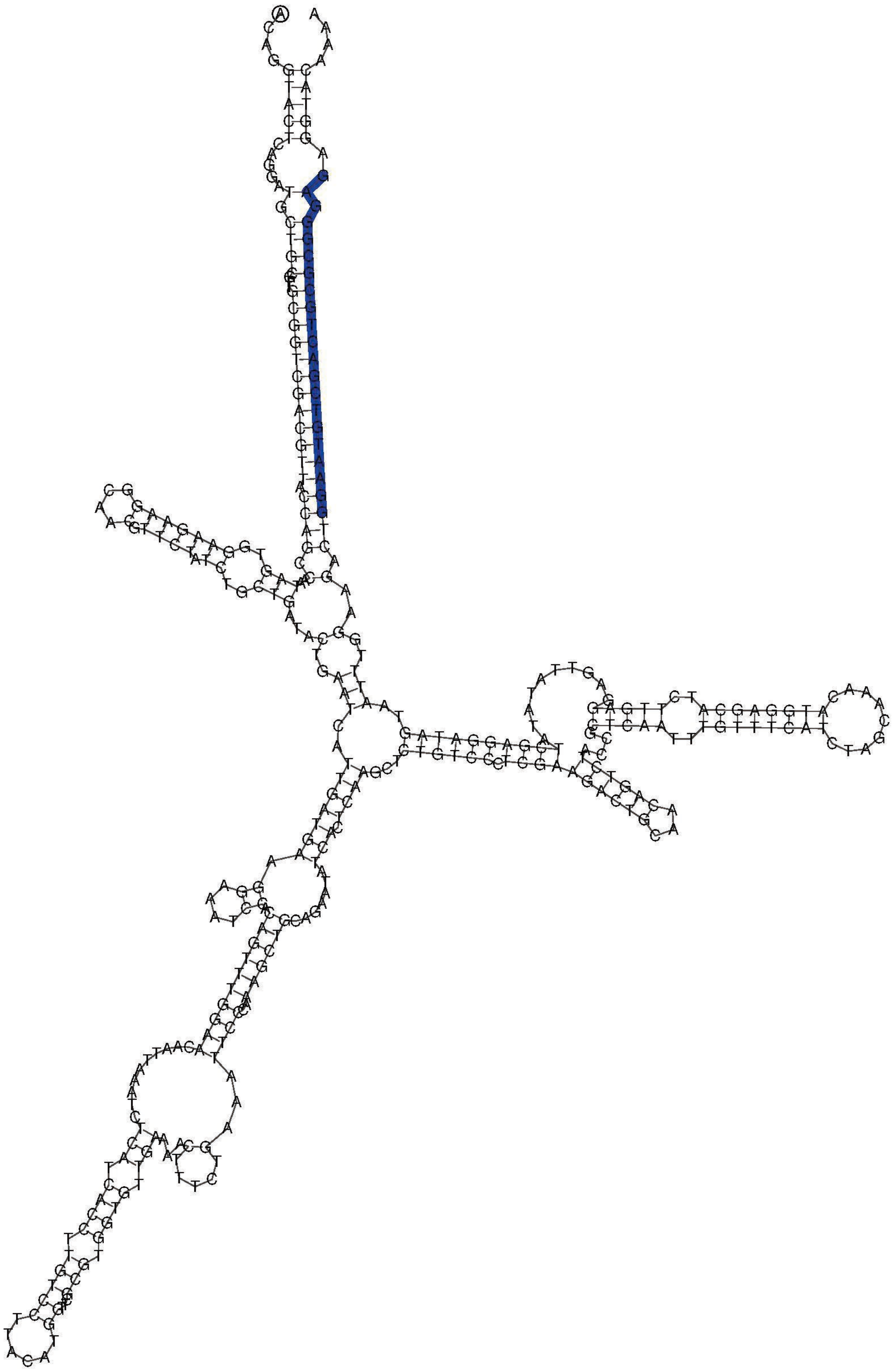

Secondary structure for csi-miR166u

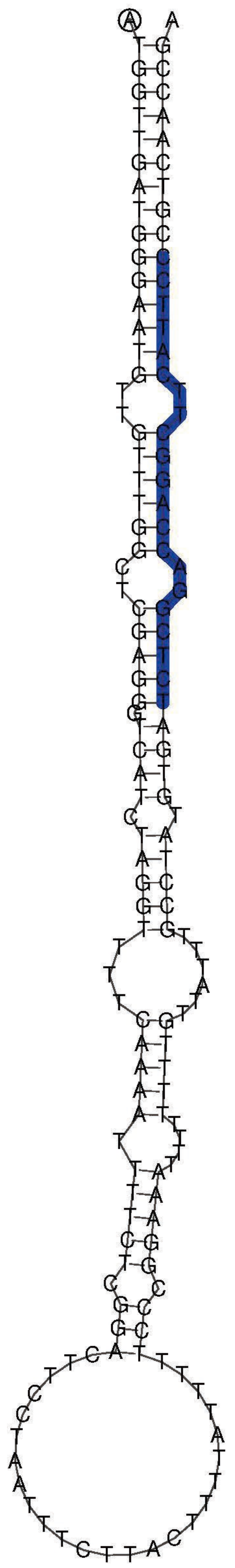

Secondary structure for csi-miR167a

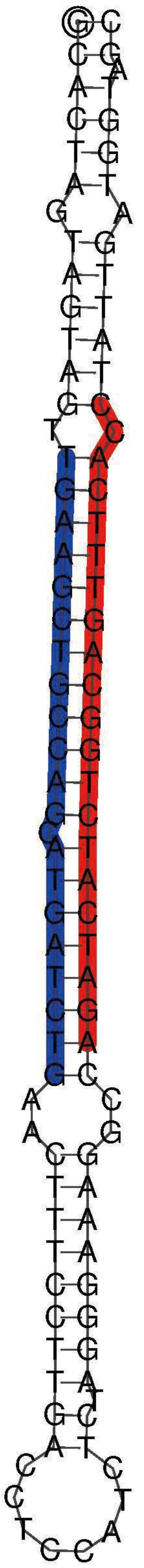

Secondary structure for csi-miR167b

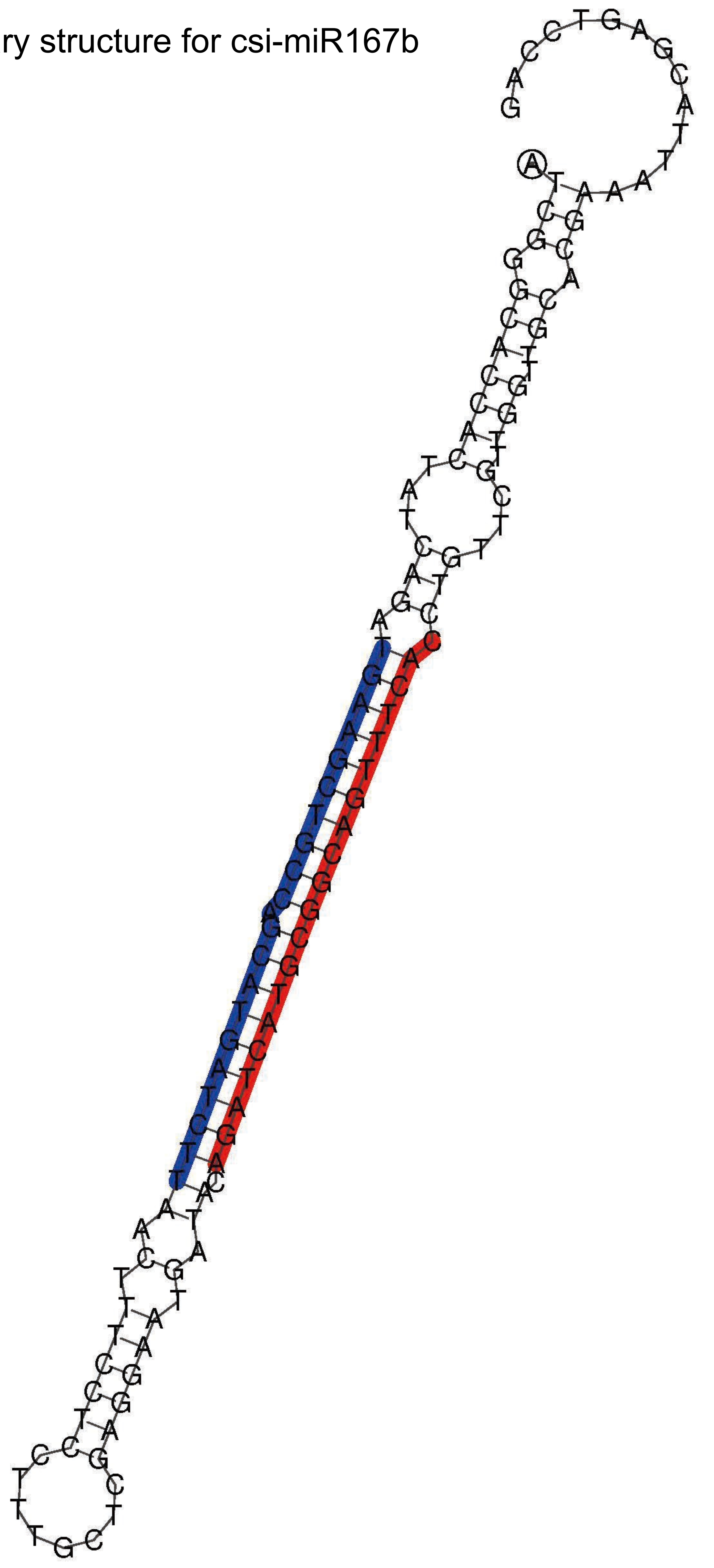

Secondary structure for csi-miR167c

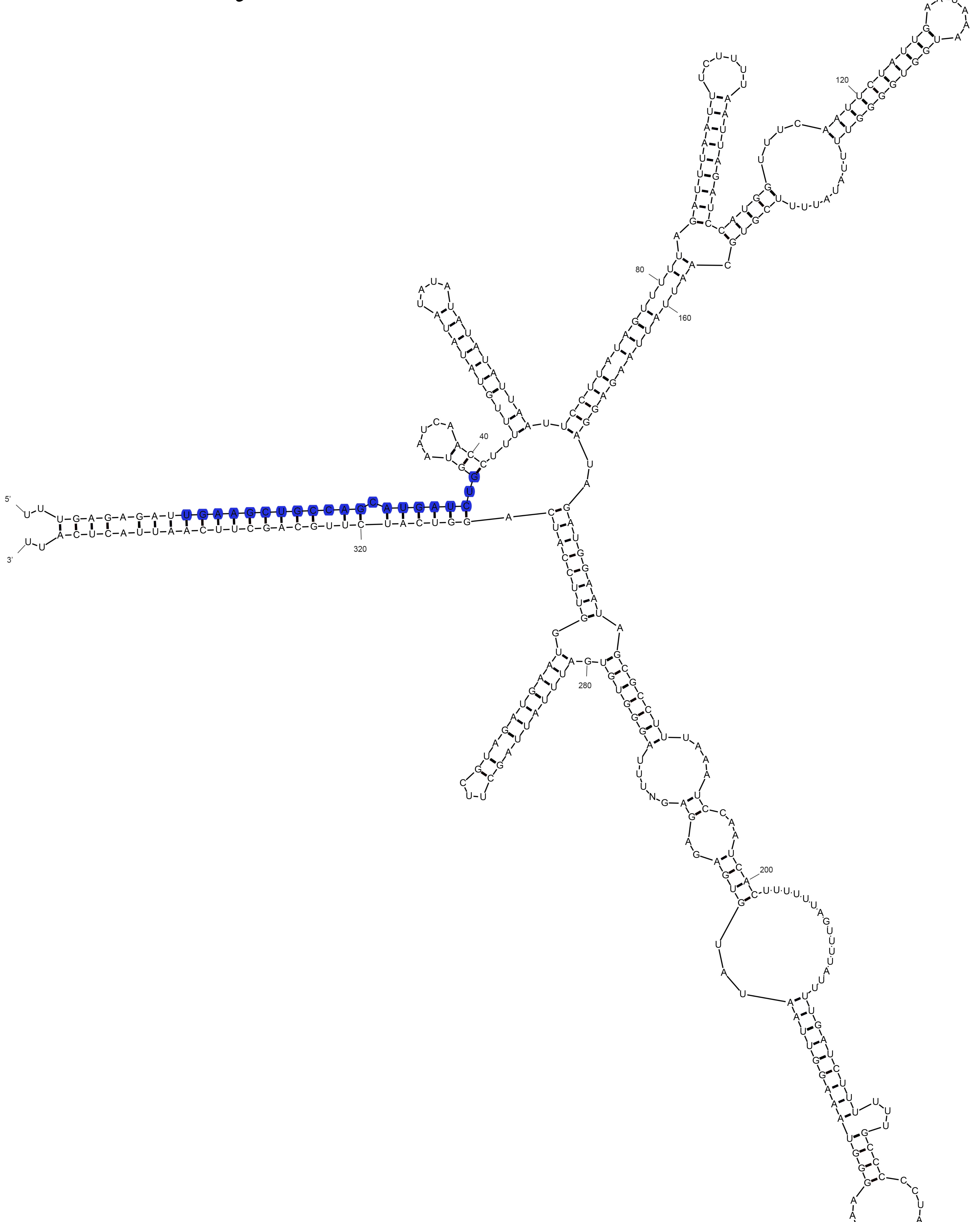

csi-miR167c

Secondary structure for csi-miR167d-3p

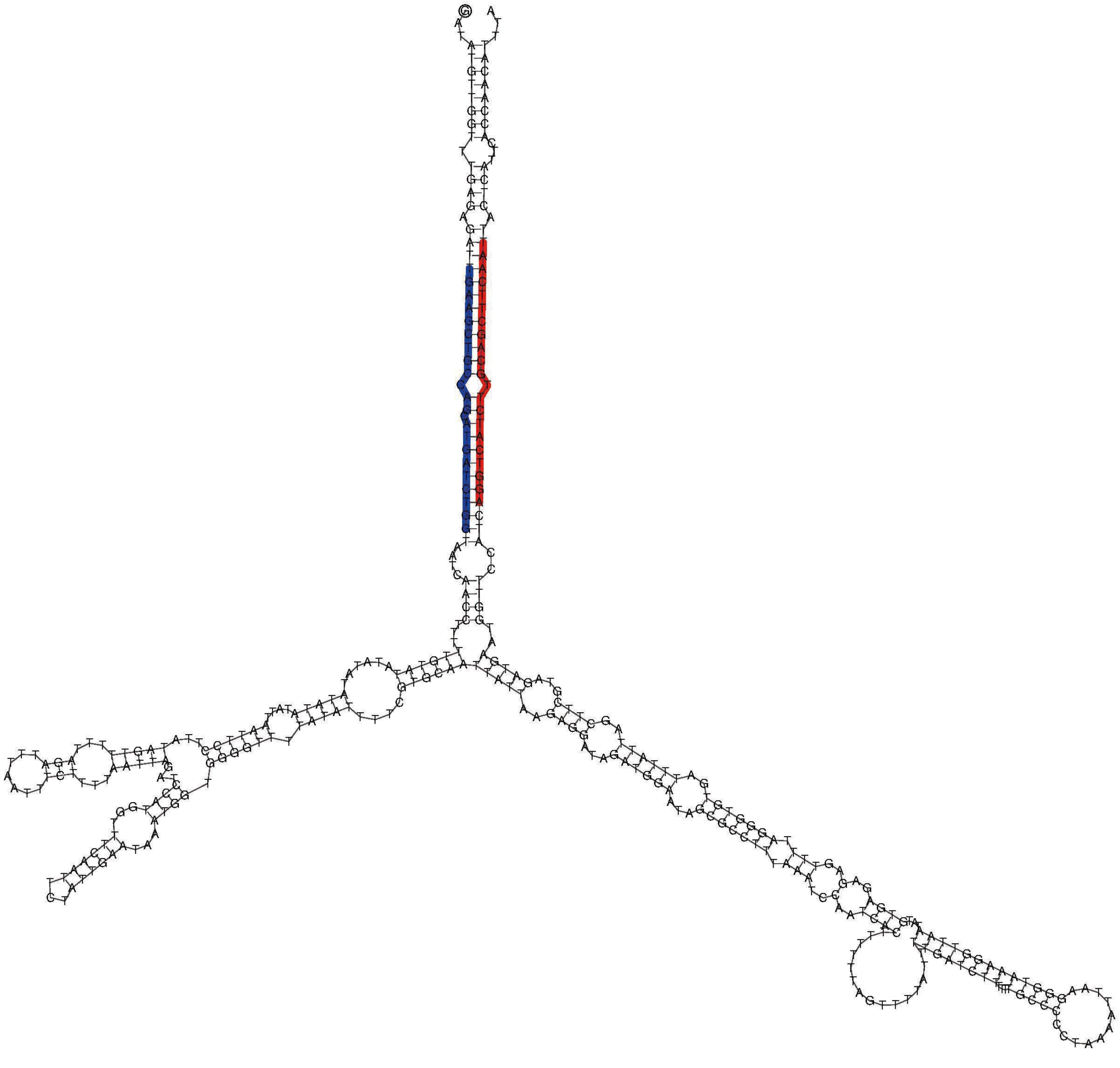

csi-miR167d-3p

Secondary structure for csi-miR167f-3p

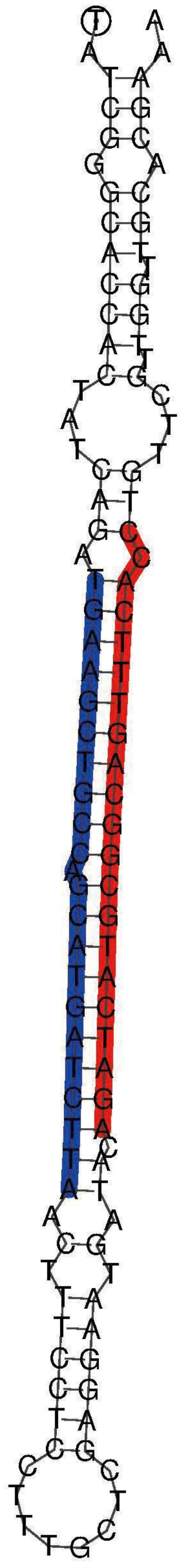

Secondary structure for csi-miR168a

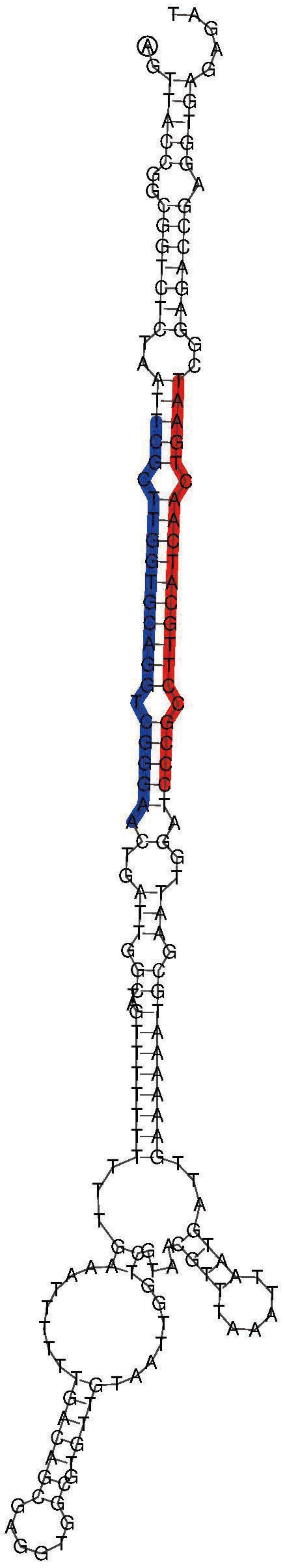

Secondary structure for csi-miR168a-3p

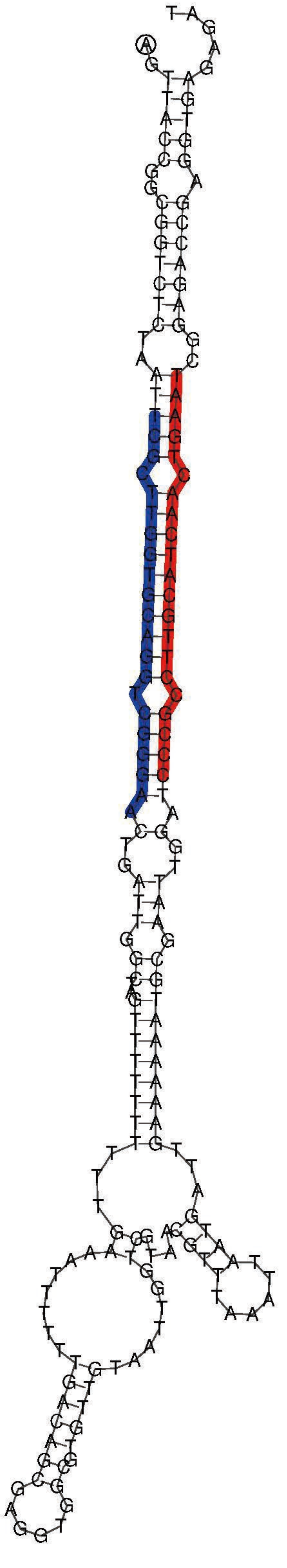

Secondary structure for csi-miR171a

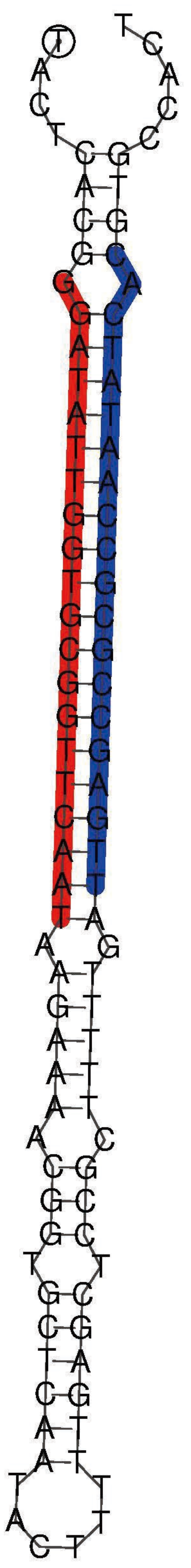

Secondary structure for csi-miR171b

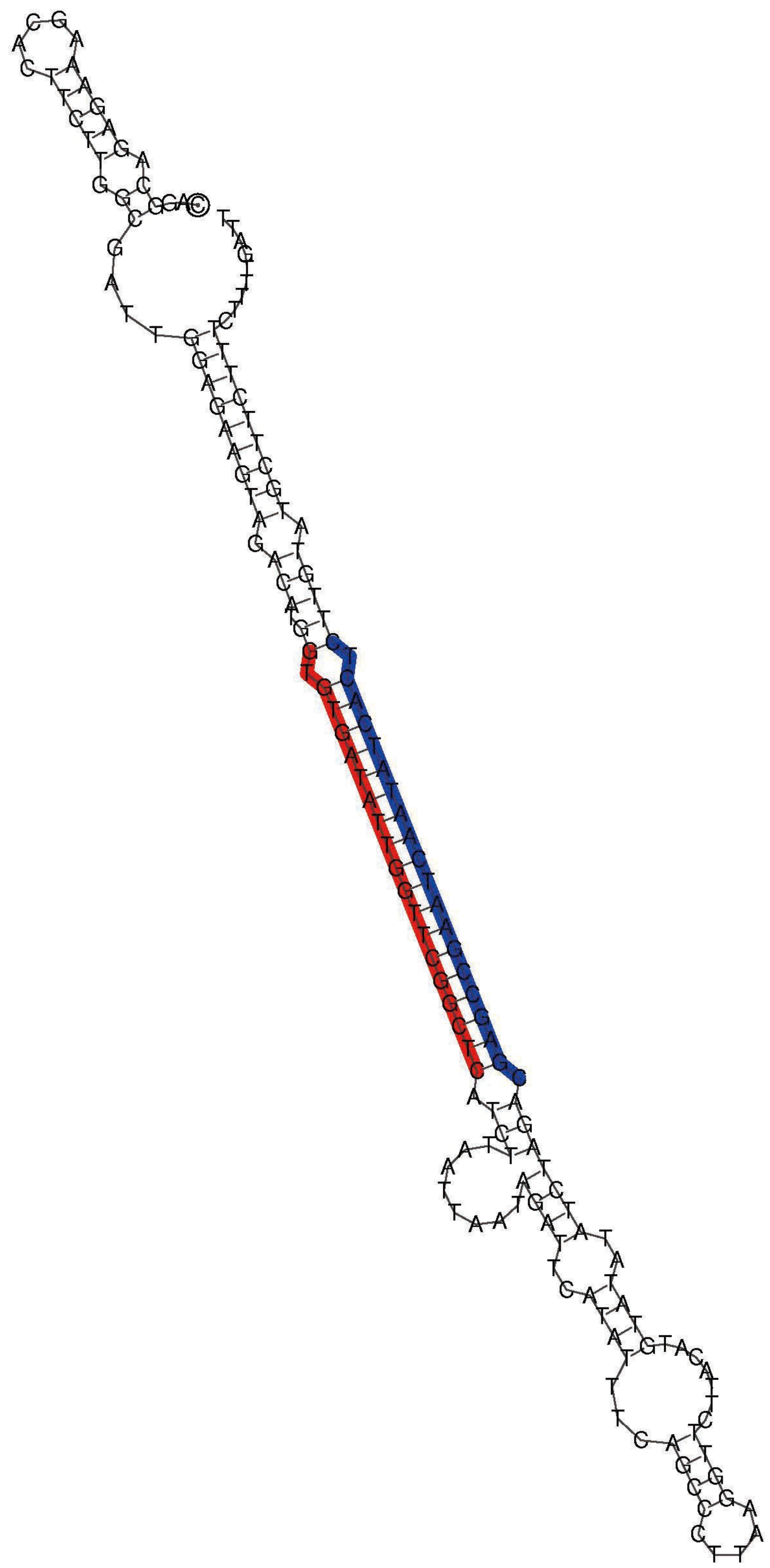

Secondary structure for csi-miR171i-5p

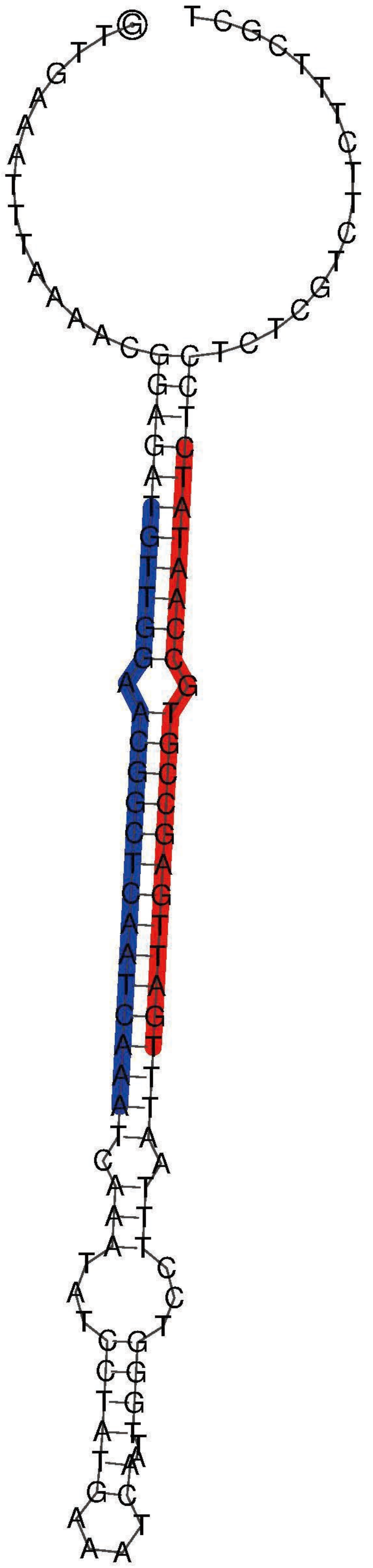

Secondary structure for csi-miR172a

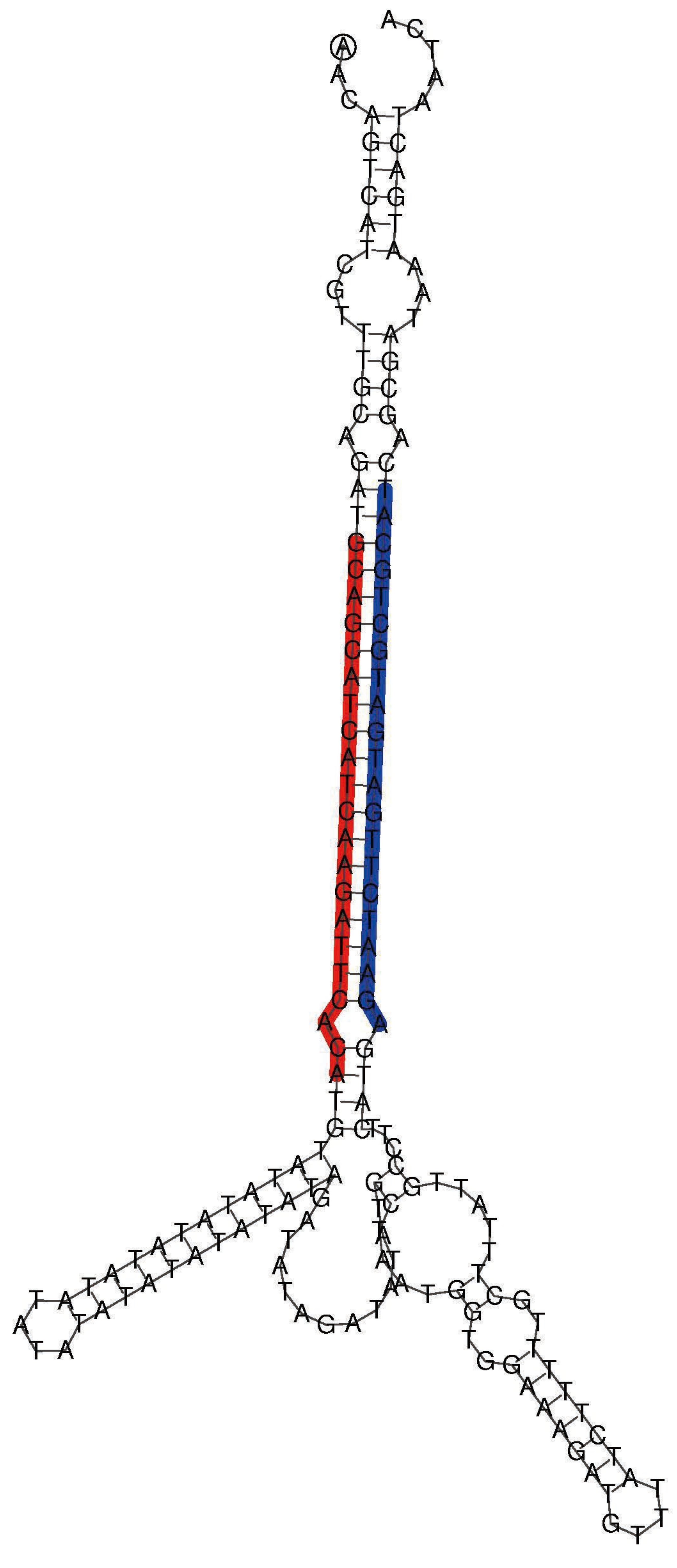

Secondary structure for csi-miR172a-3p

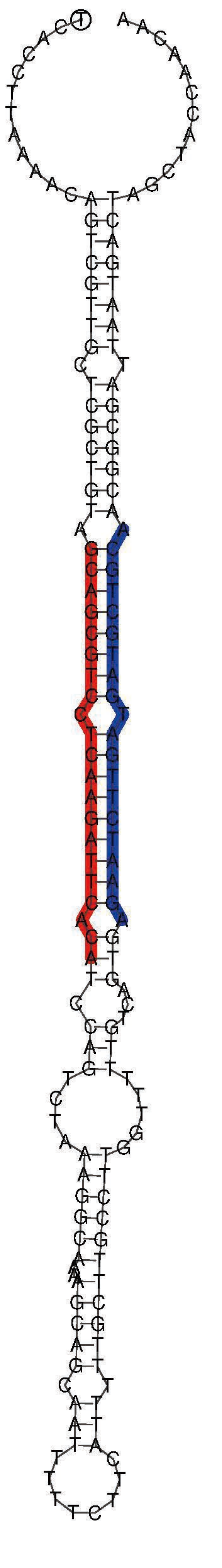

Secondary structure for csi-miR172a-5p

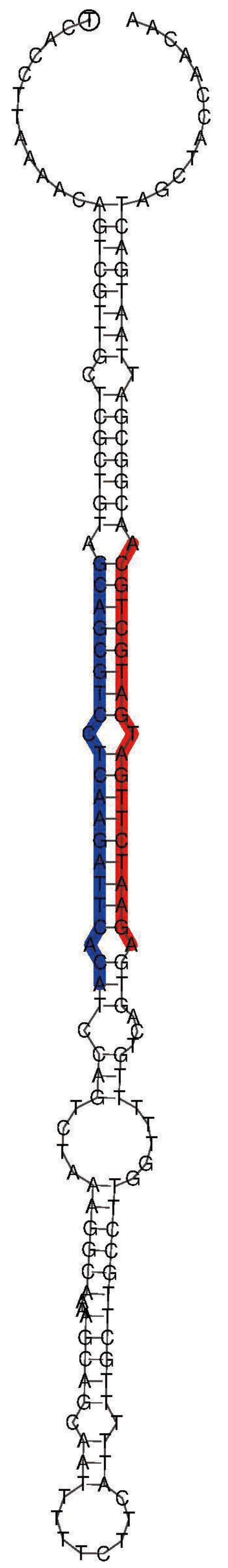

Secondary structure for csi-miR172b-5p

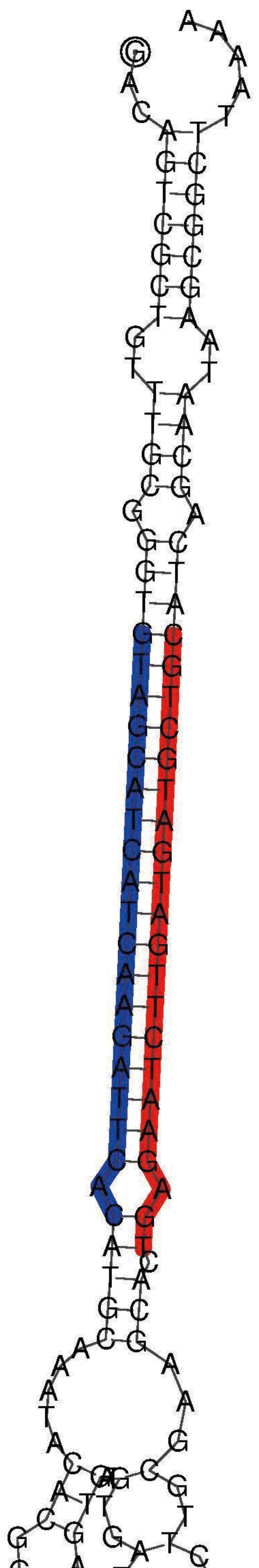

Secondary structure for csi-miR172c

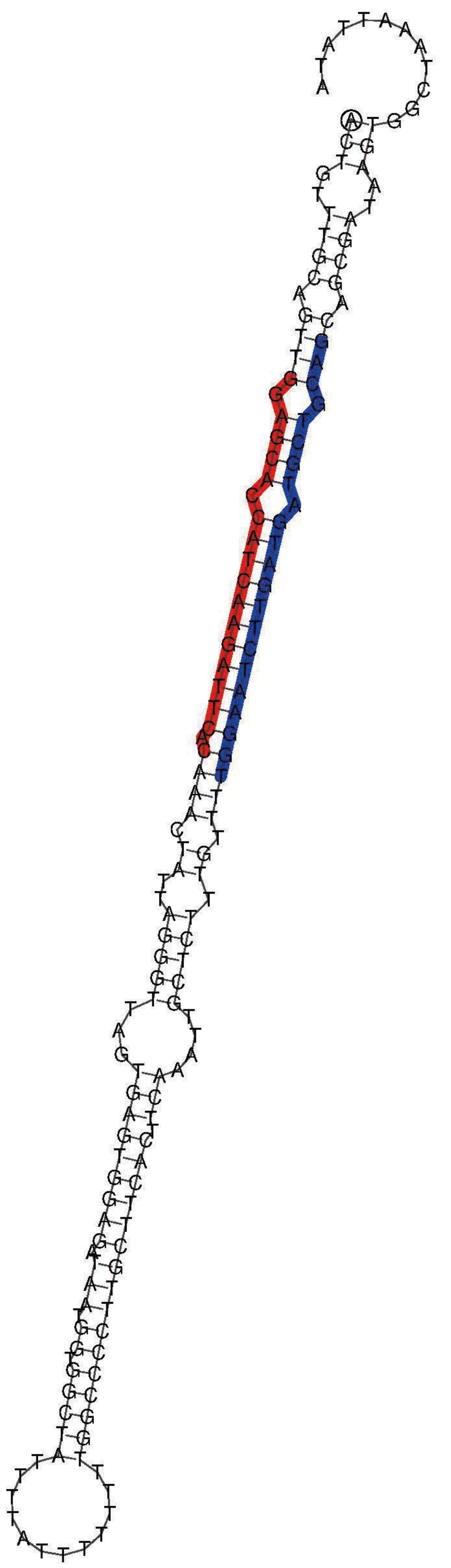

Secondary structure for csi-miR172e-3p

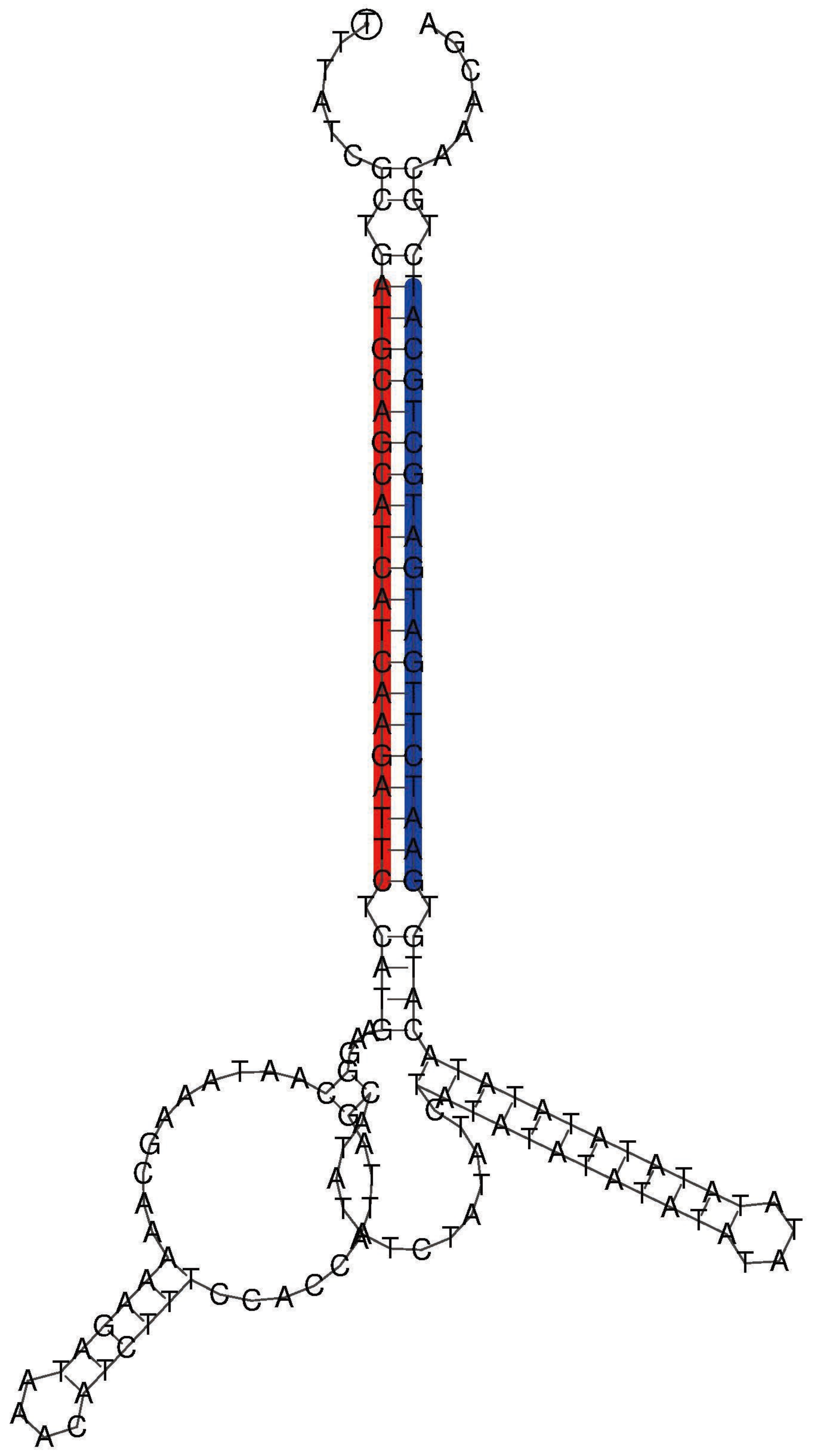

Secondary structure for csi-miR172h-5p

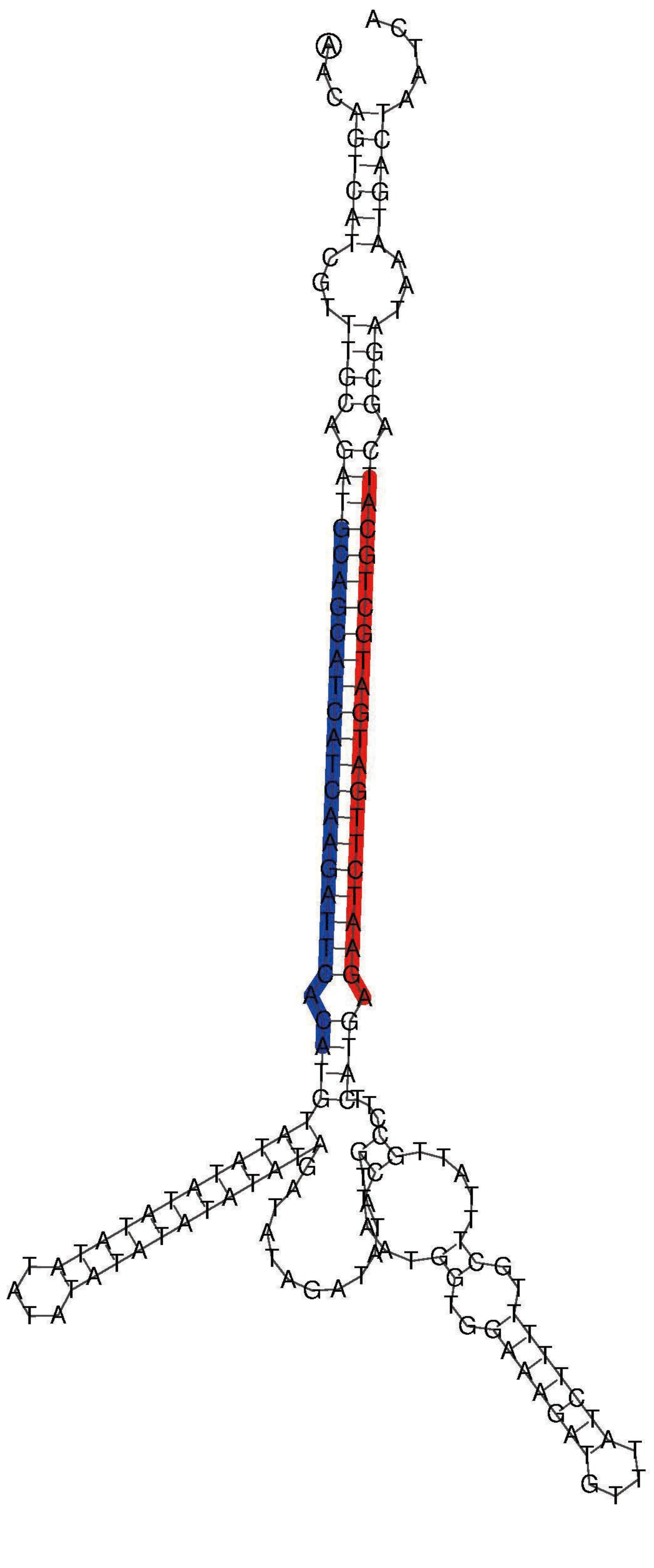

Secondary structure for csi-miR1861a

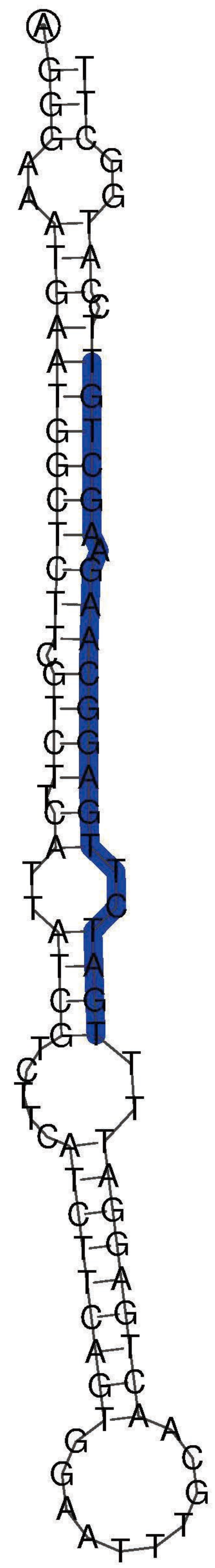

Secondary structure for csi-miR319

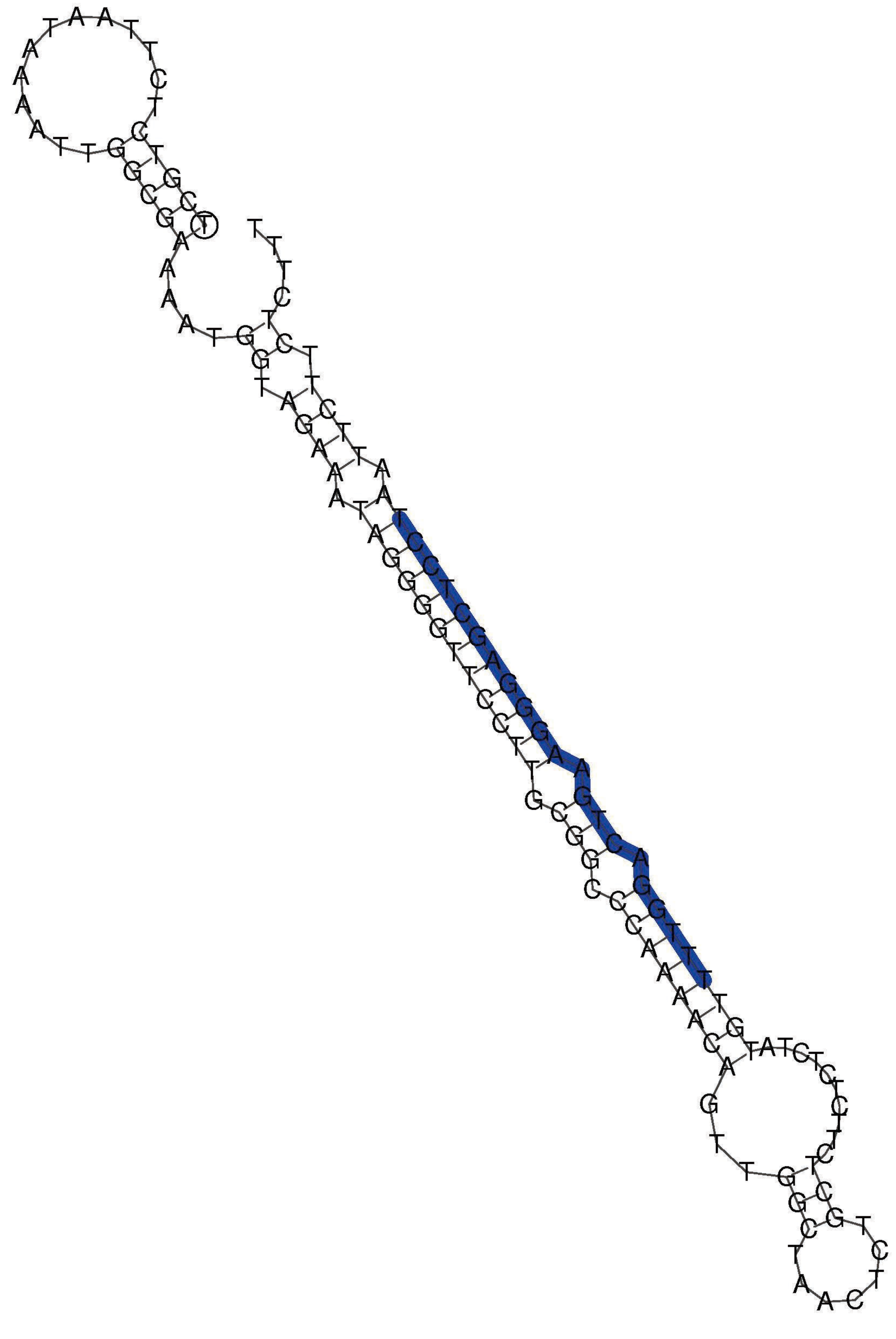

Secondary structure for csi-miR390

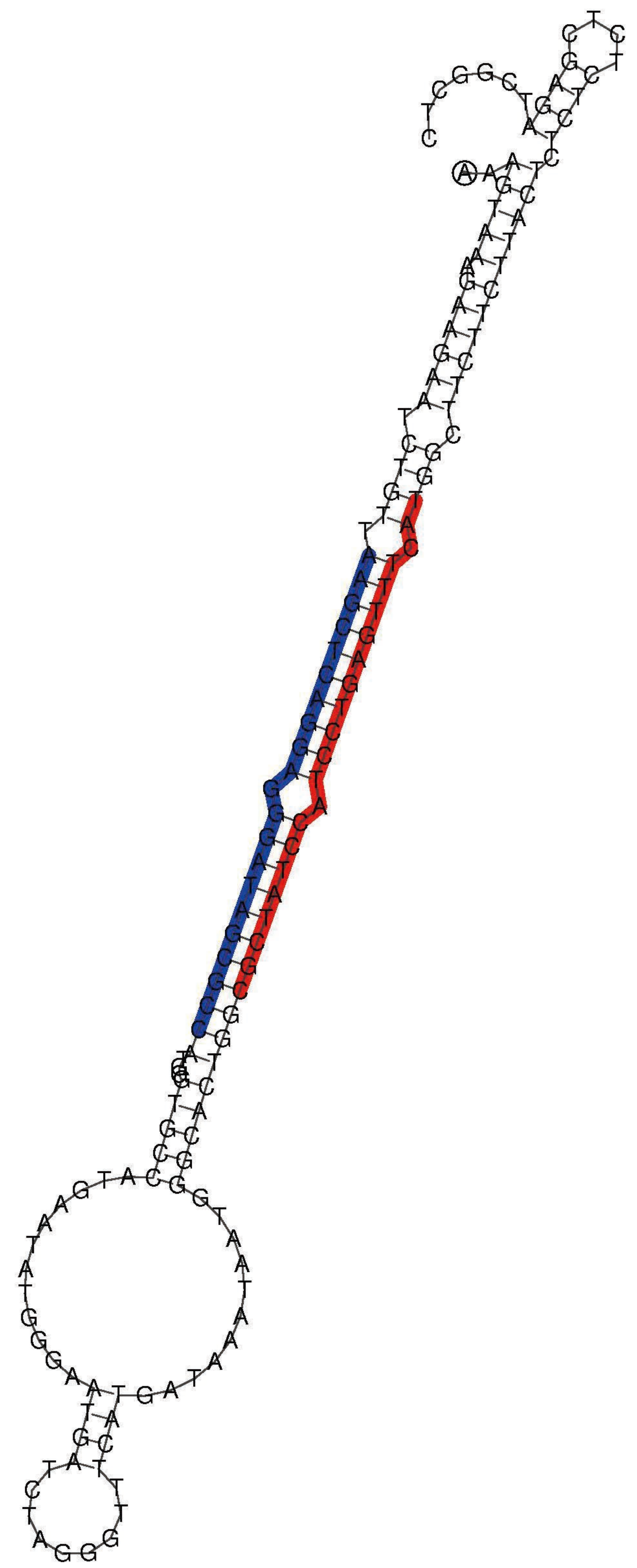

Secondary structure for csi-miR390a

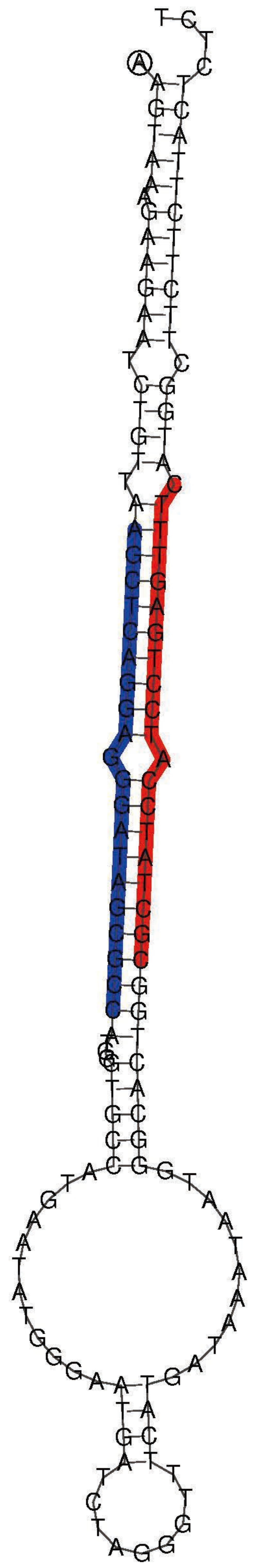

Secondary structure for csi-miR390a-3p

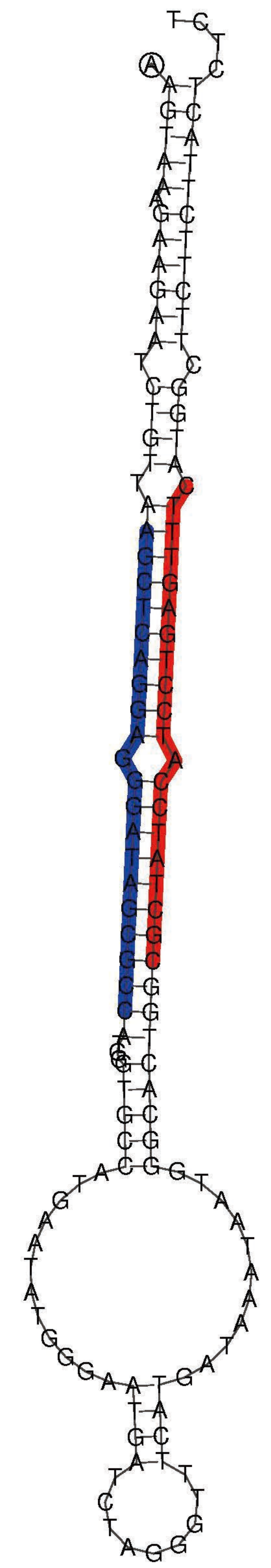

Secondary structure for csi-miR393-3p

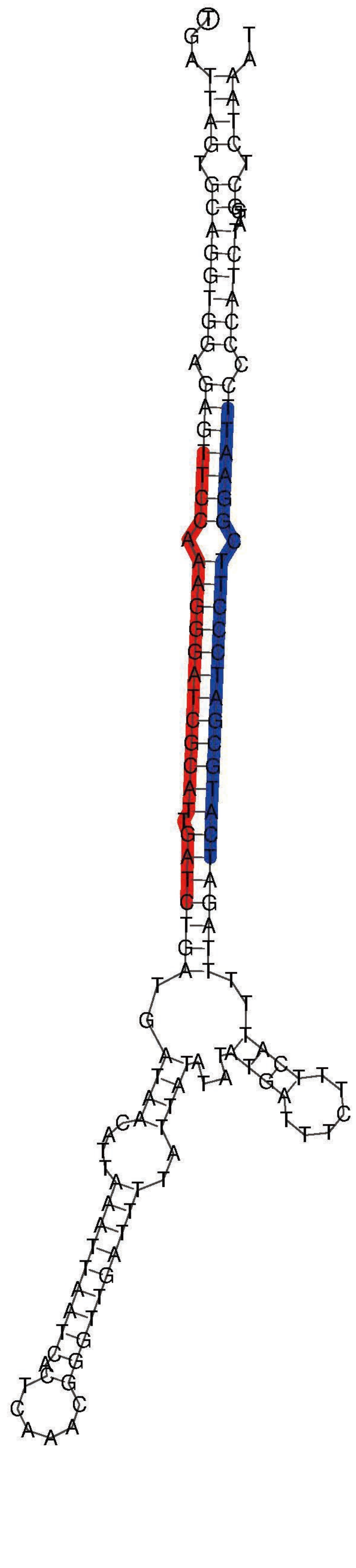

Secondary structure for csi-miR393a-3p

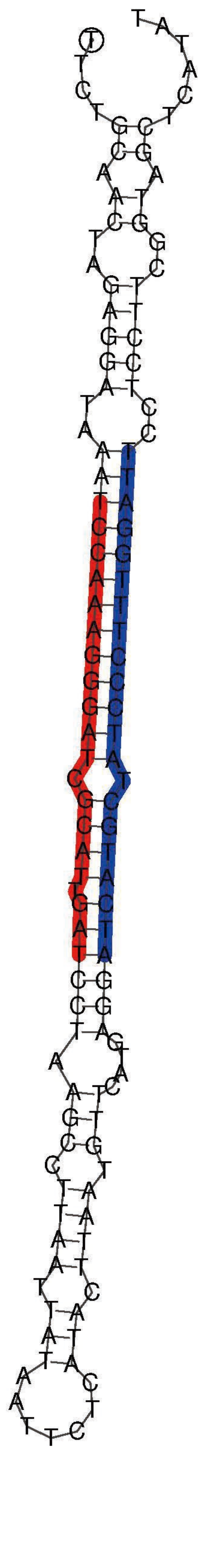

Secondary structure for csi-miR393h

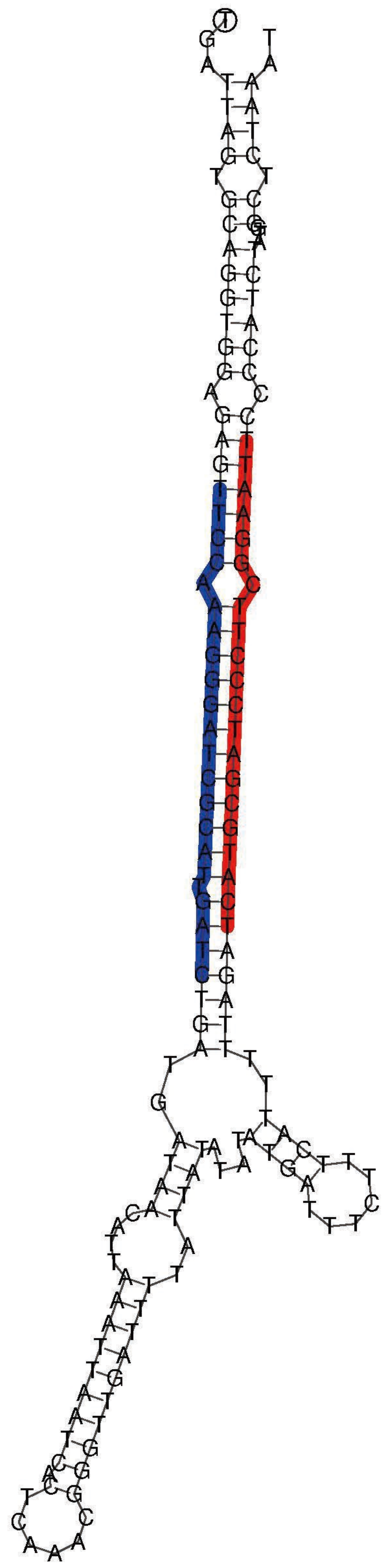

Secondary structure for csi-miR394

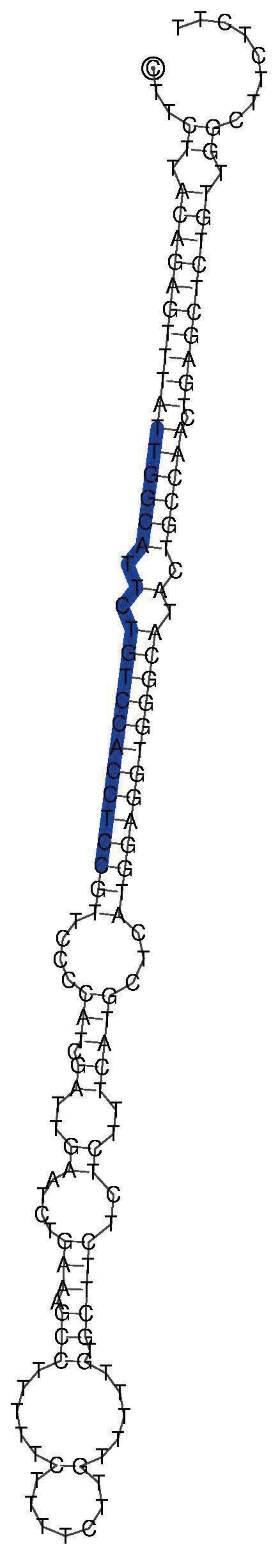

Secondary structure for csi-miR3946

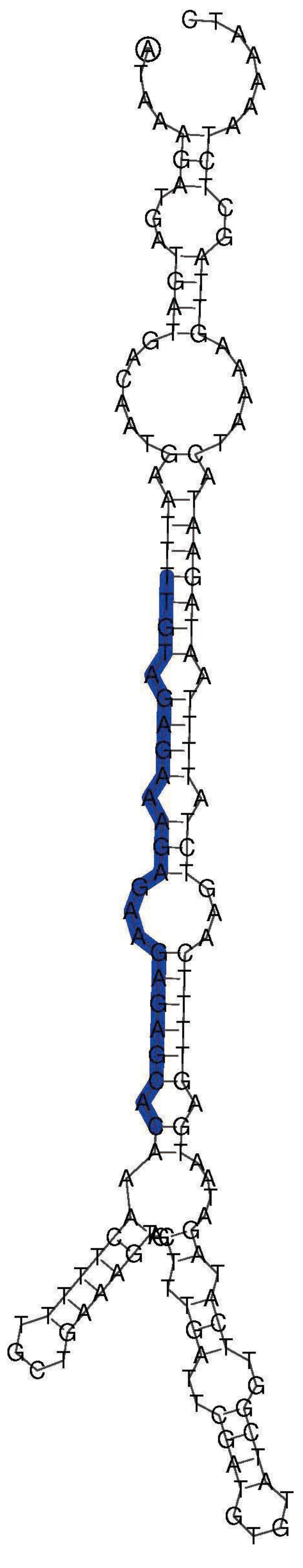

Secondary structure for csi-miR3947

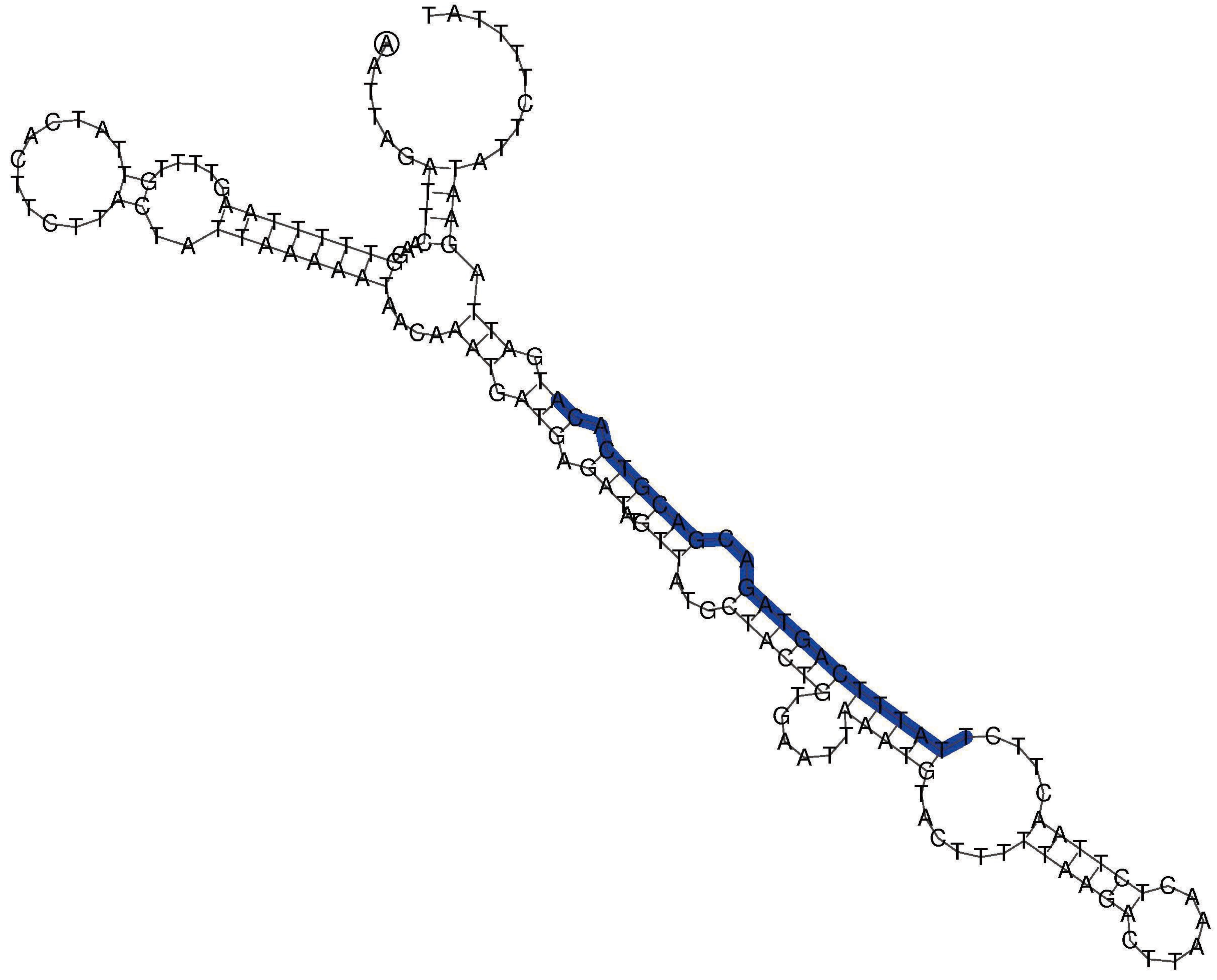

Secondary structure for csi-miR3948

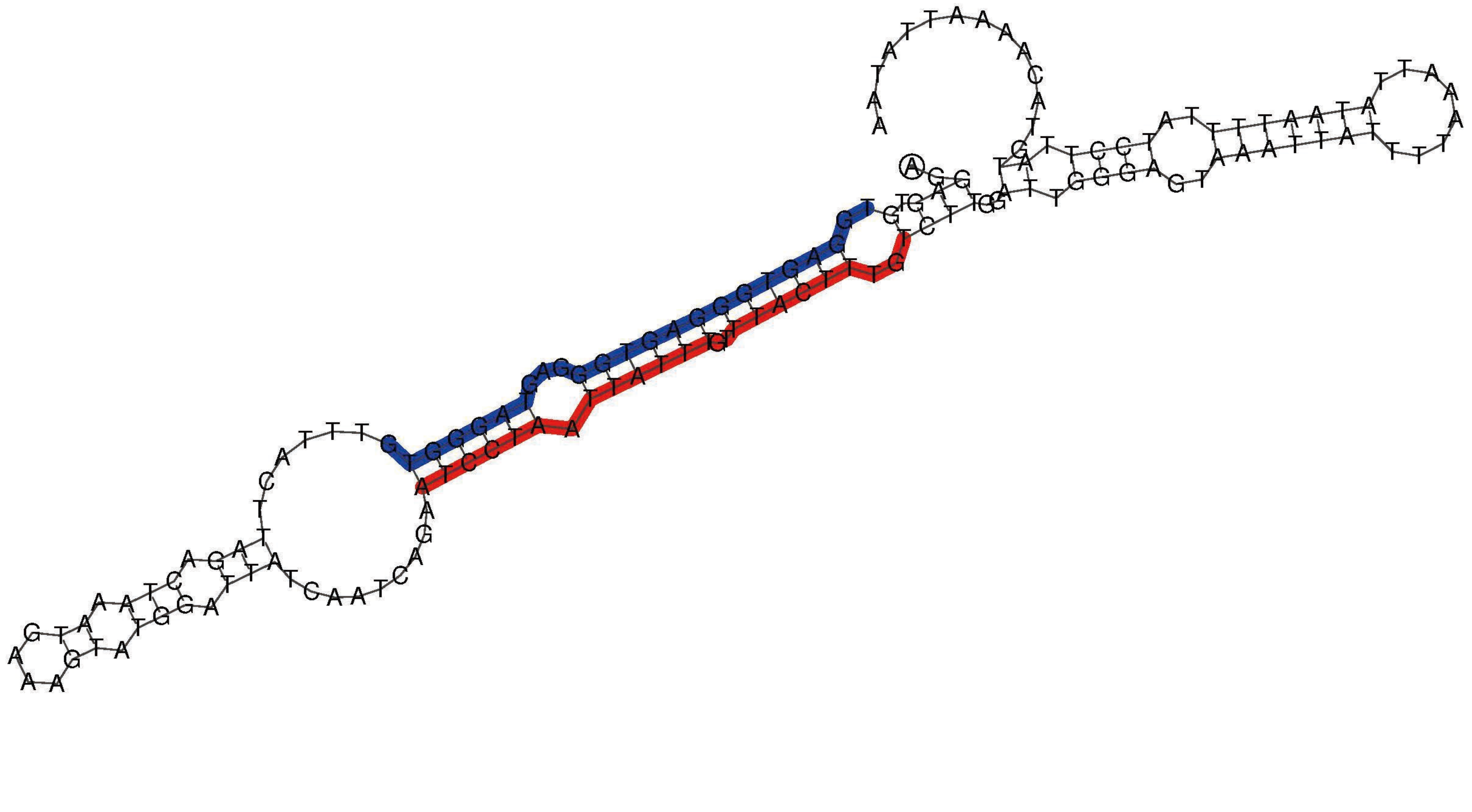

Secondary structure for csi-miR395

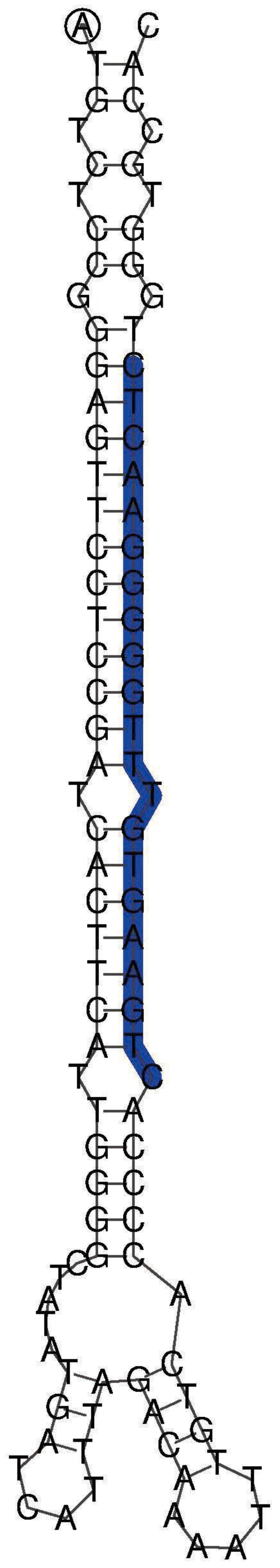

Secondary structure for csi-miR3950

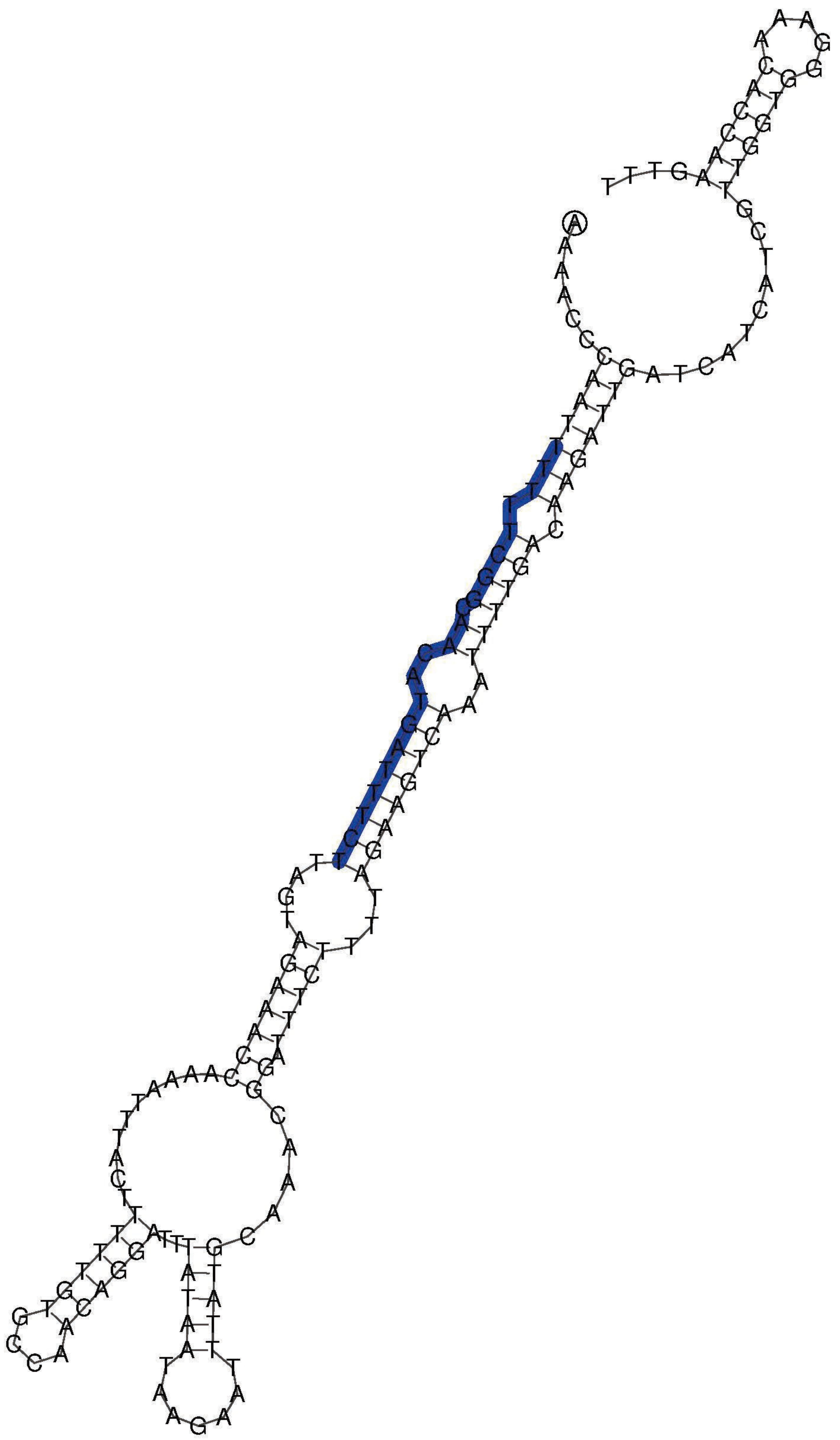

Secondary structure for csi-miR3951

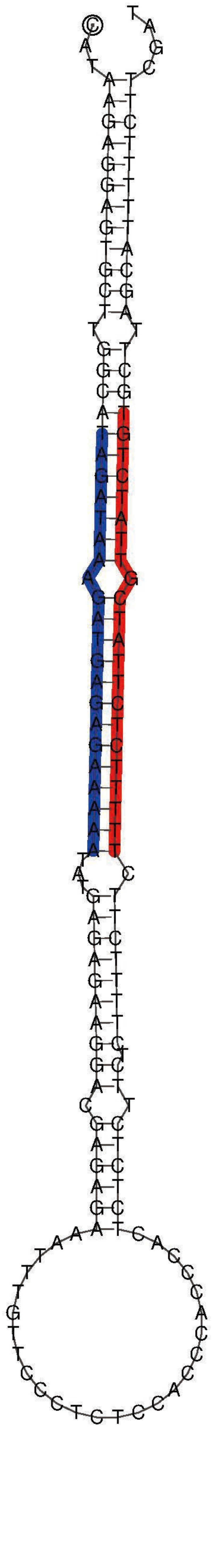

Secondary structure for csi-miR3952

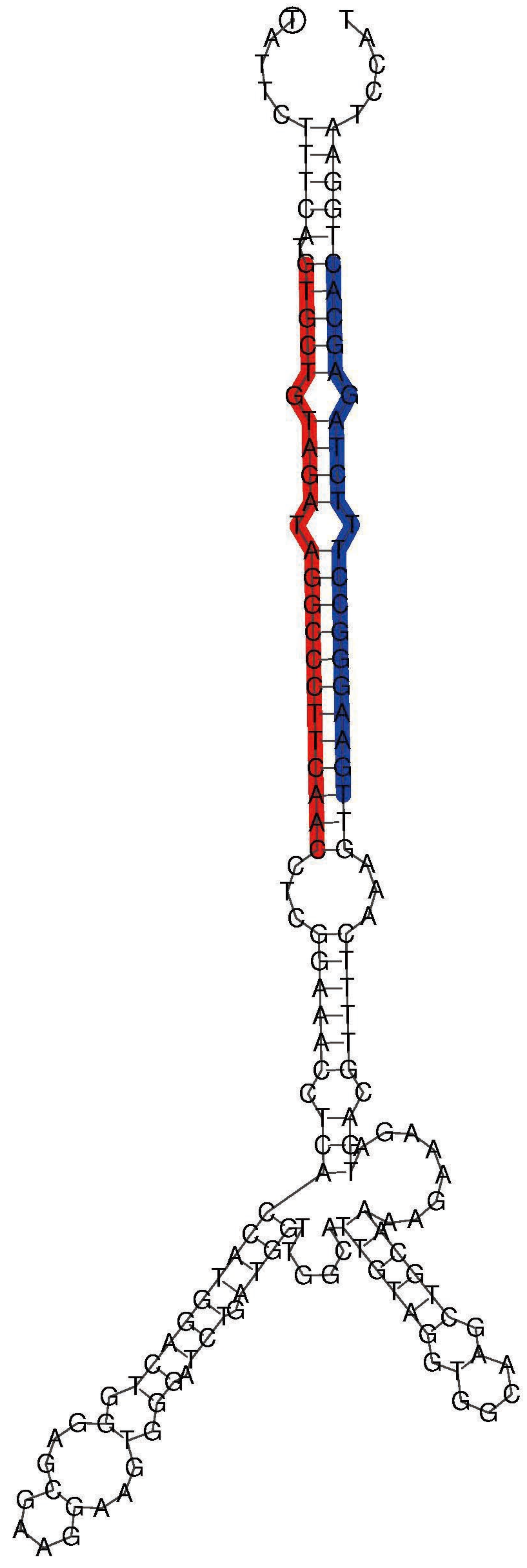

Secondary structure for csi-miR3953

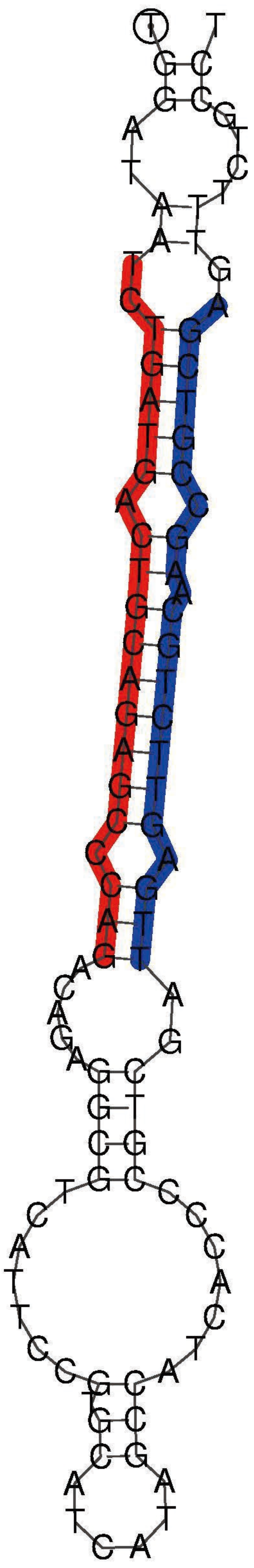

Secondary structure for csi-miR3954

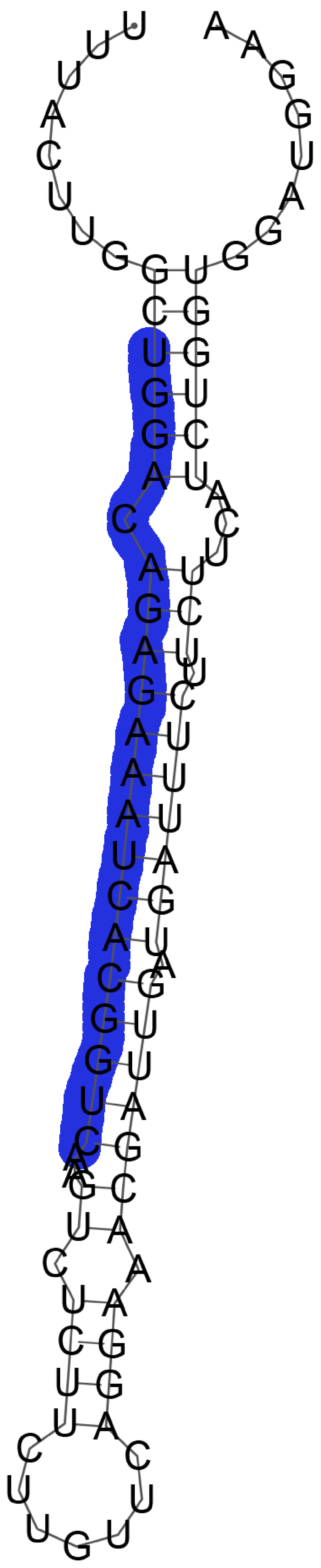

Secondary structure for csi-miR396b

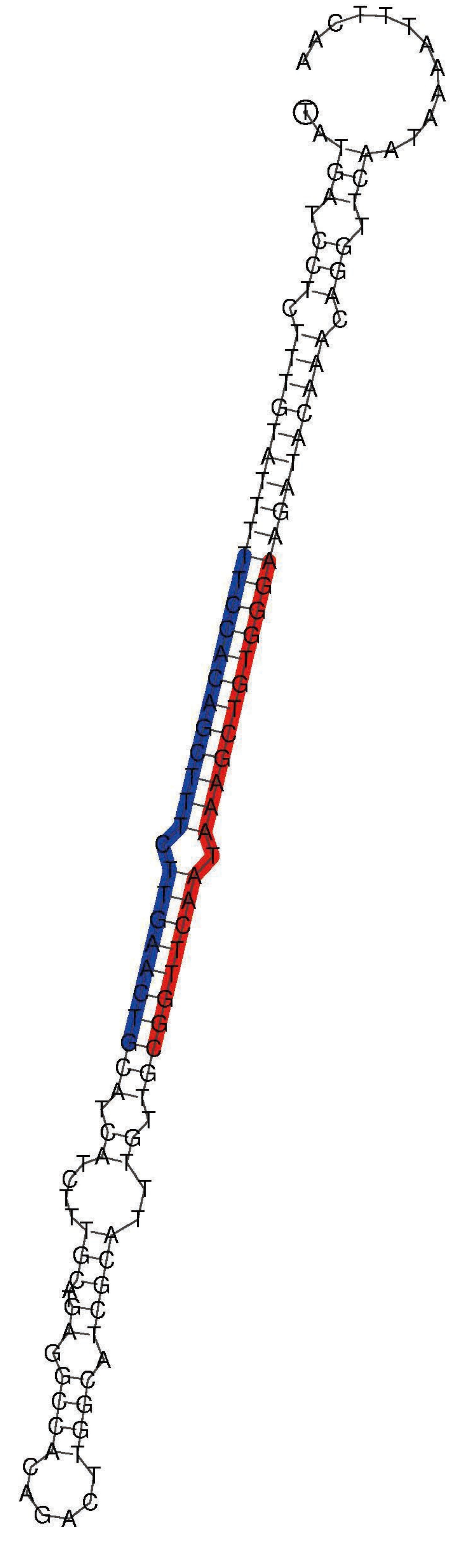

Secondary structure for csi-miR396b-3p

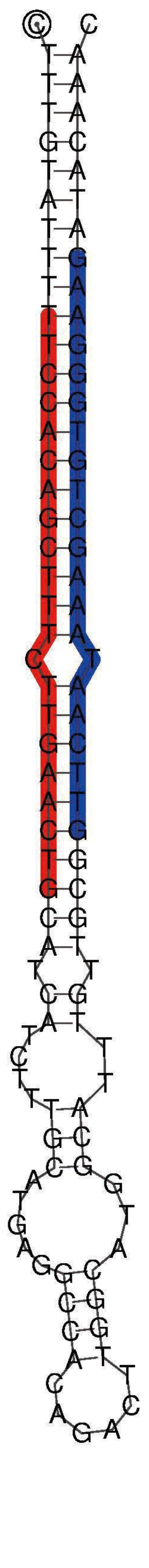

Secondary structure for csi-miR396c

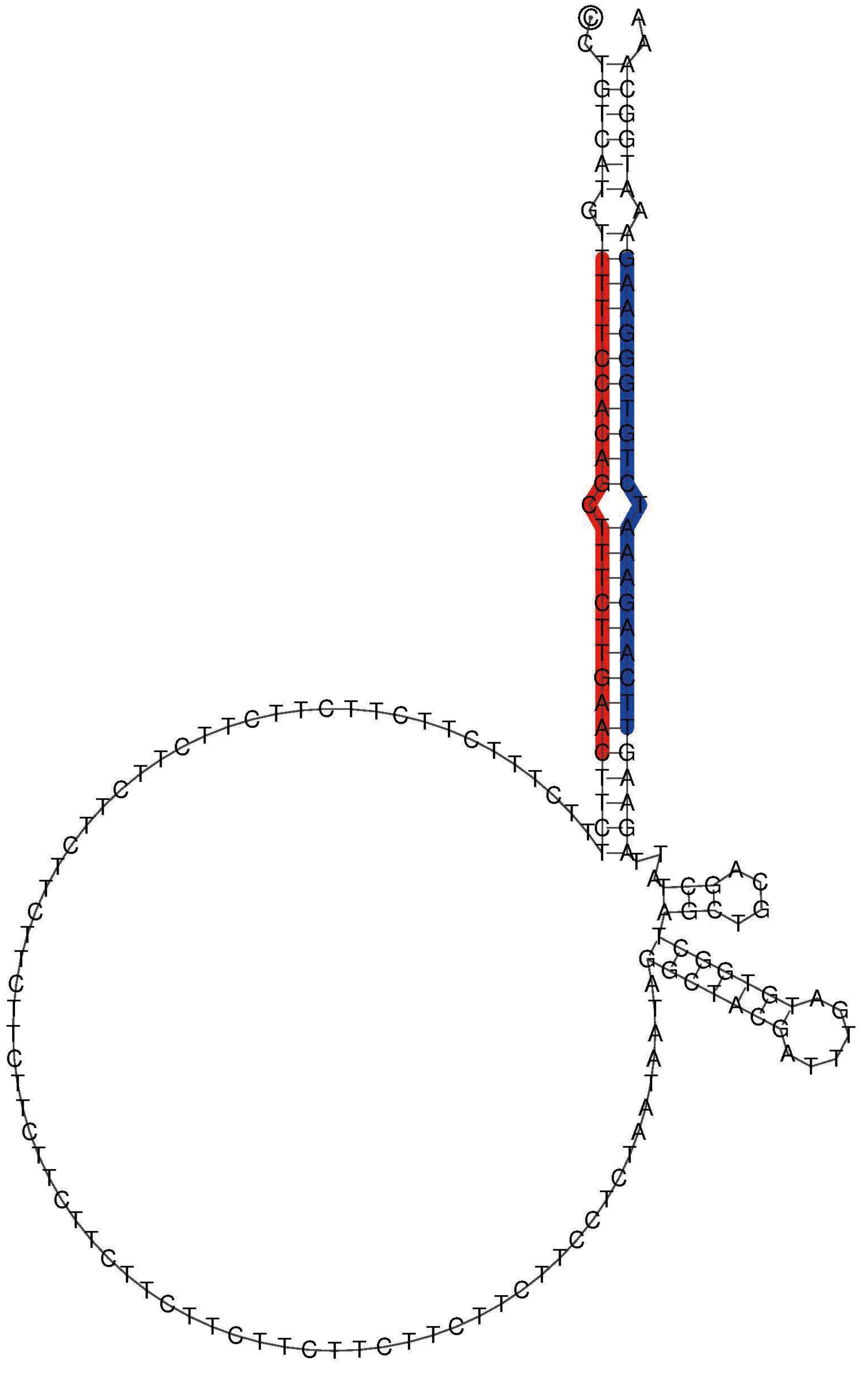

Secondary structure for csi-miR396g-5p

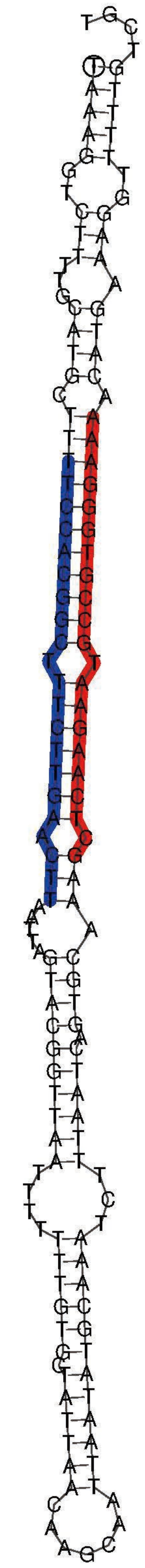

Secondary structure for csi-miR397

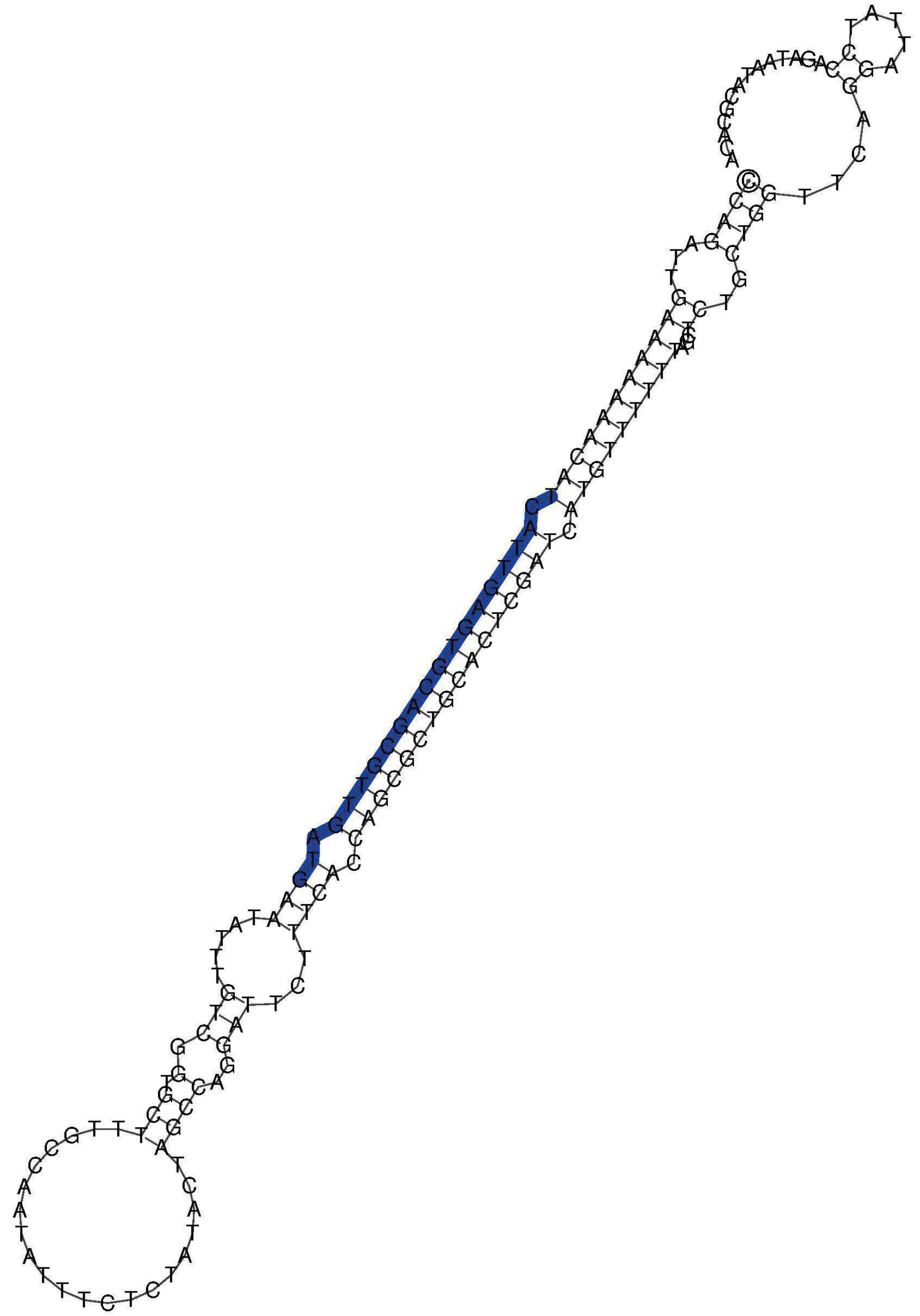

Secondary structure for csi-miR398

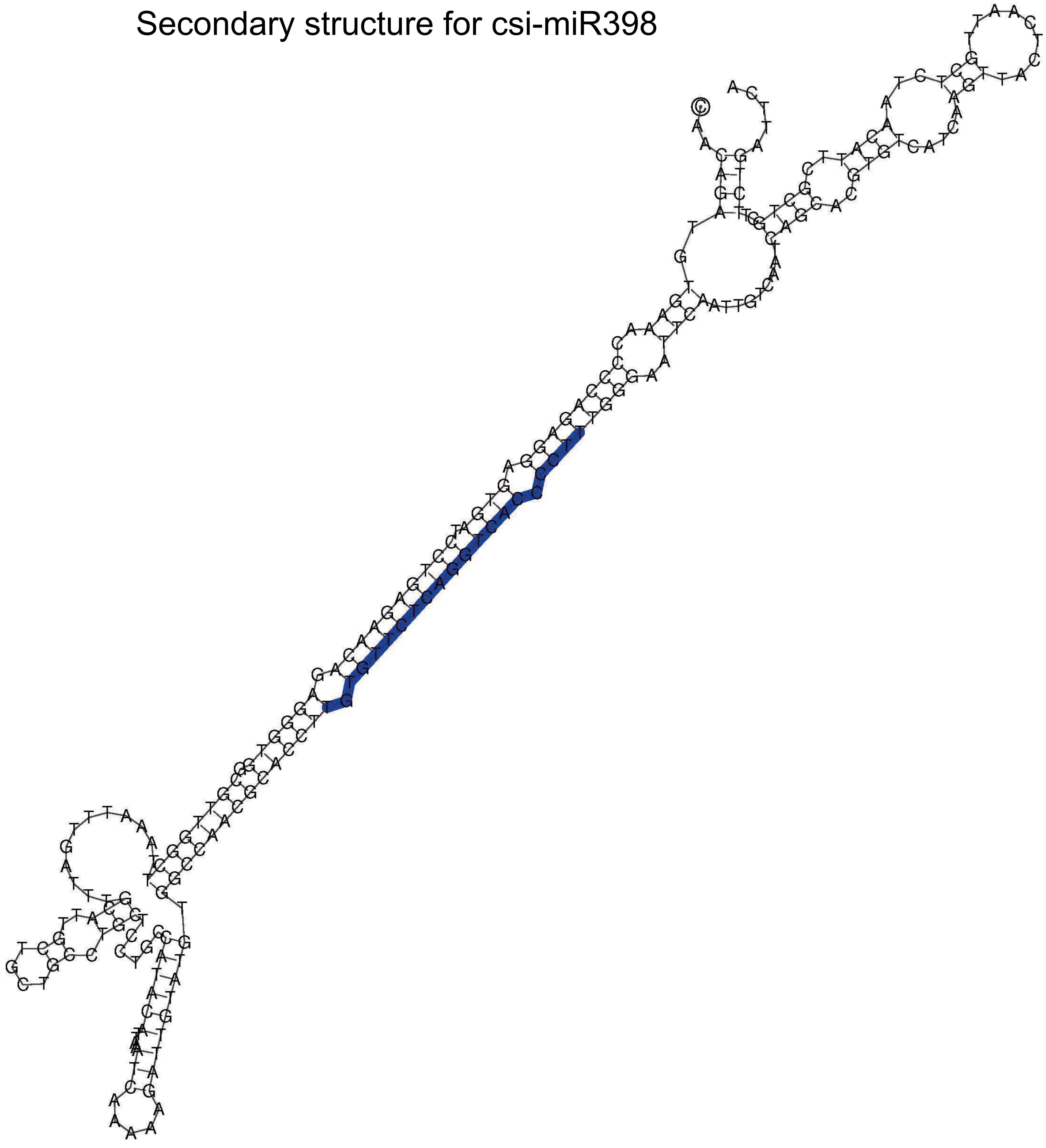

Secondary structure for csi-miR399a

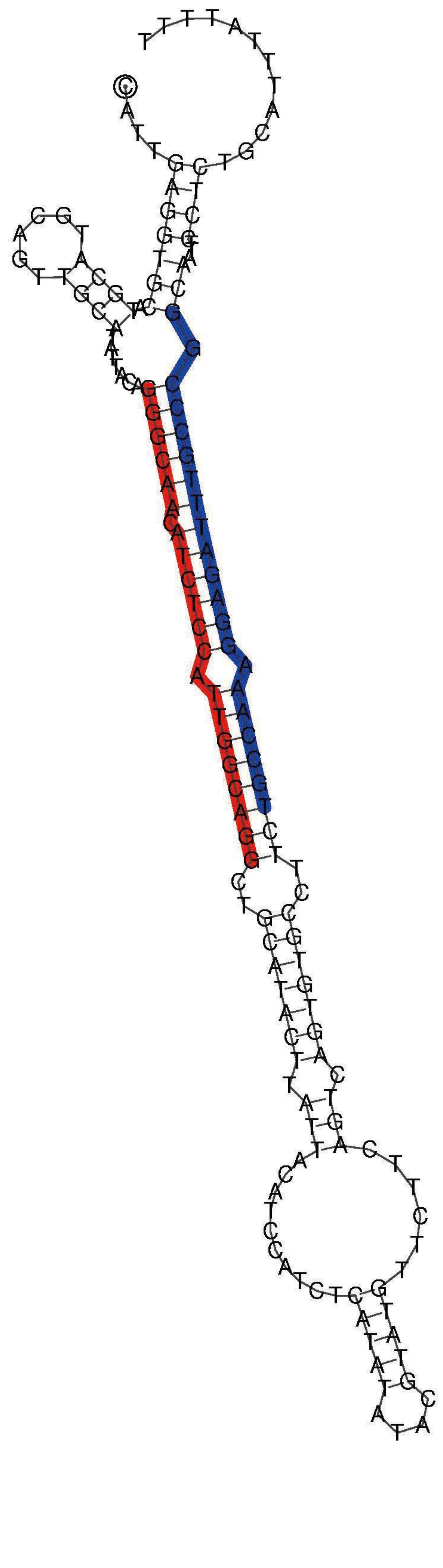

Secondary structure for csi-miR399b

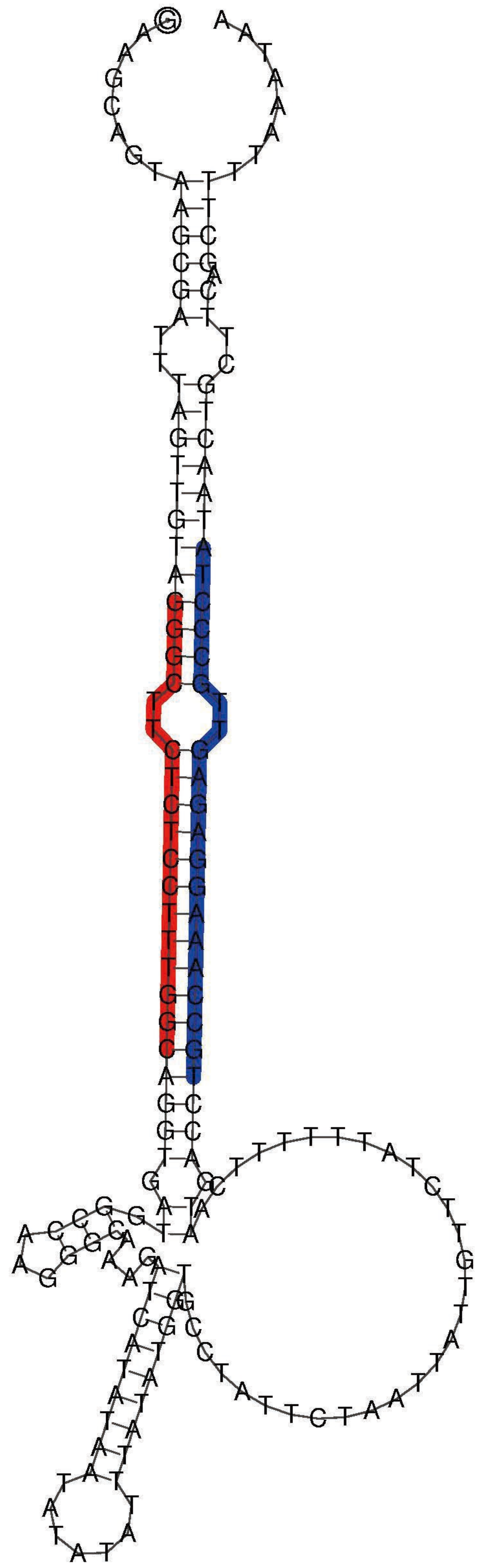

Secondary structure for csi-miR399c

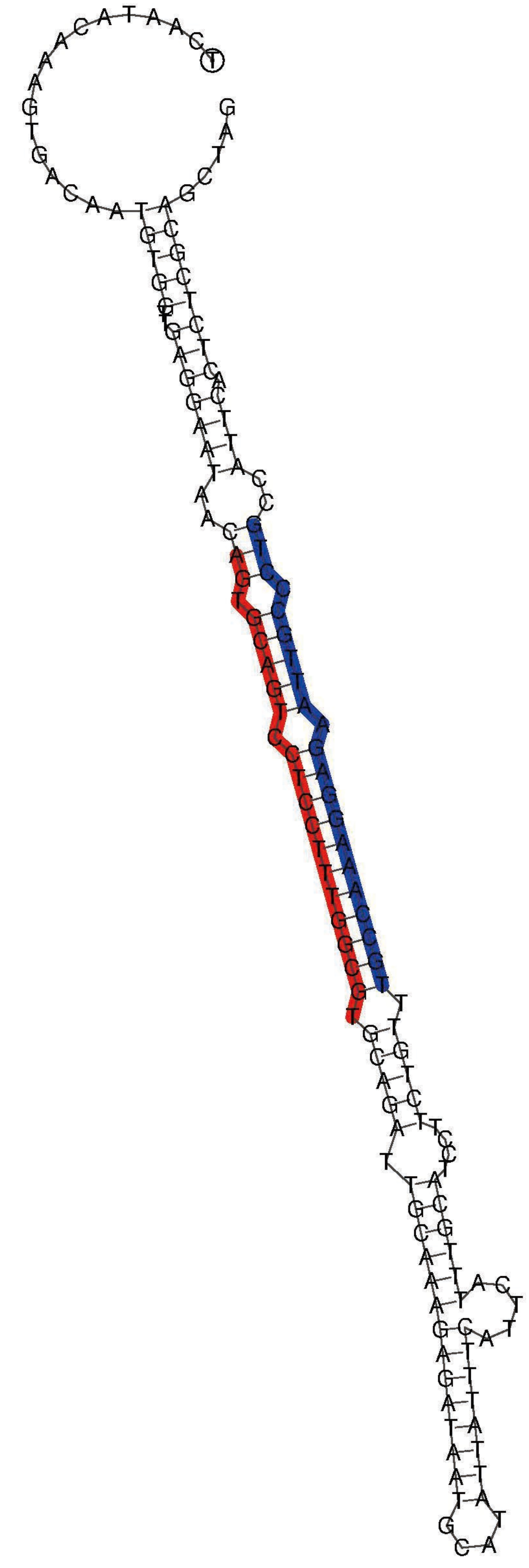

Secondary structure for csi-miR399d

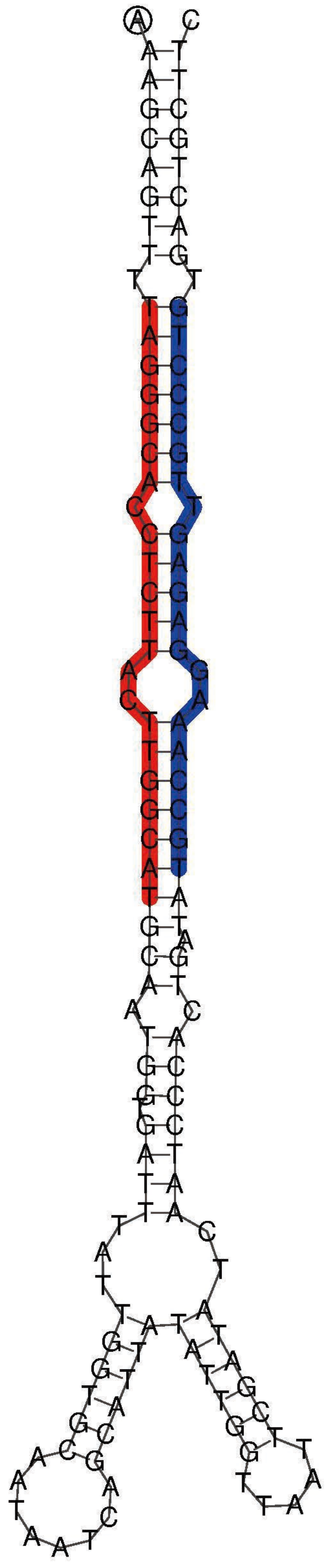

Secondary structure for csi-miR399e

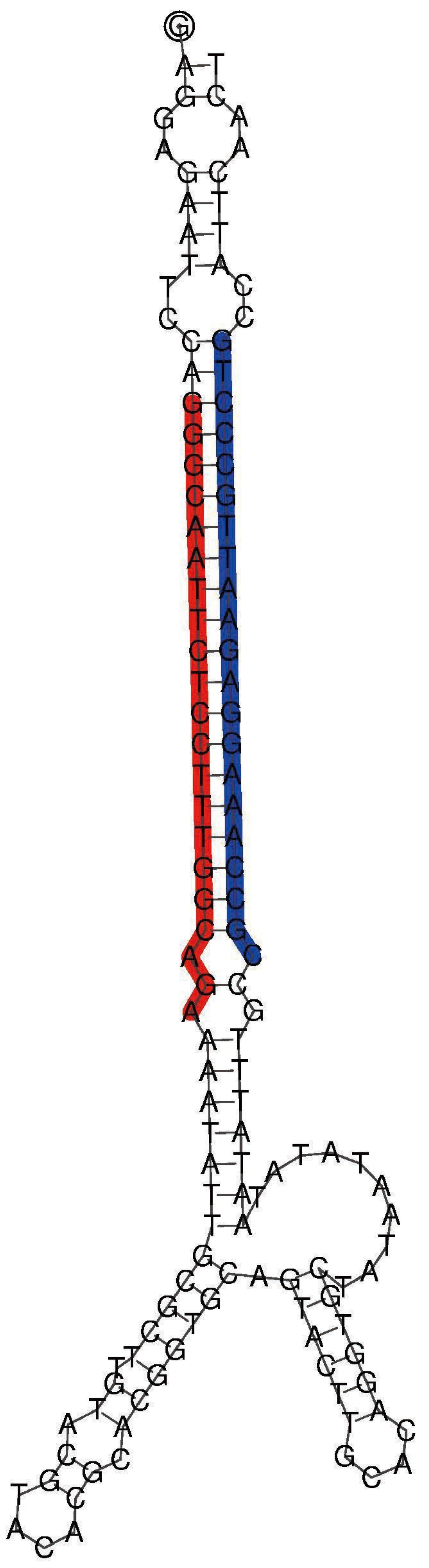

Secondary structure for csi-miR399i-5p

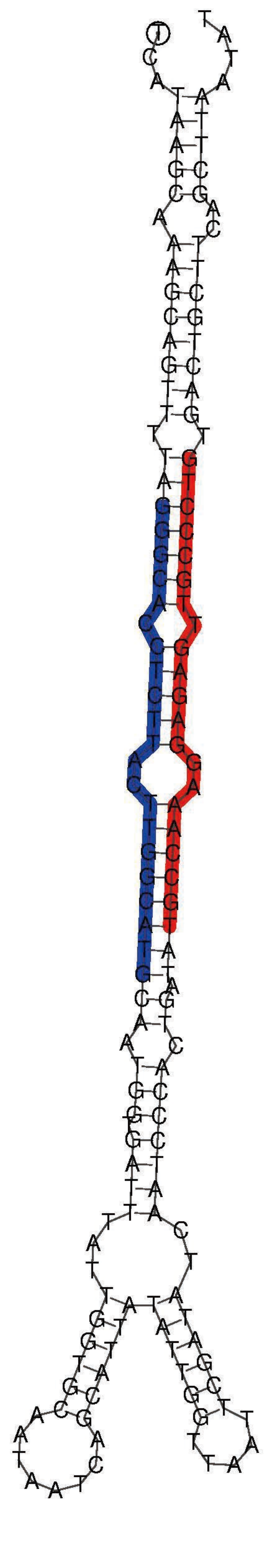

Secondary structure for csi-miR403

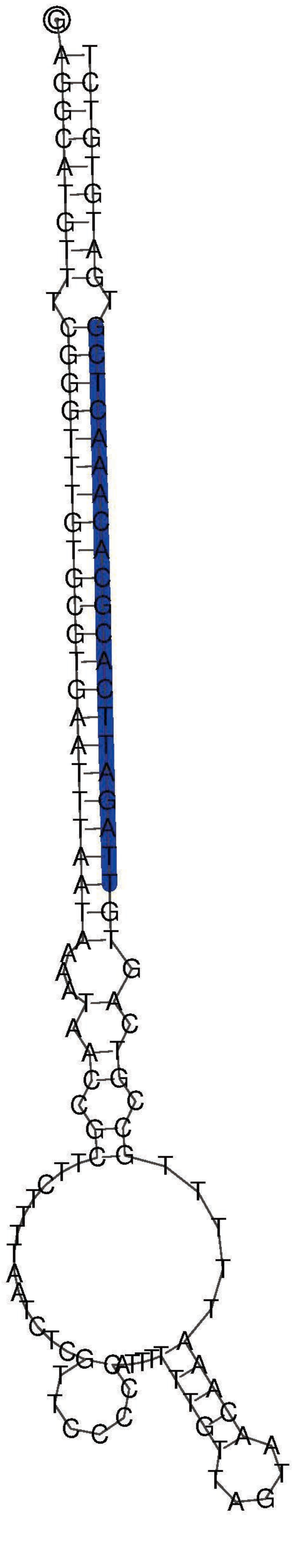

Secondary structure for csi-miR408

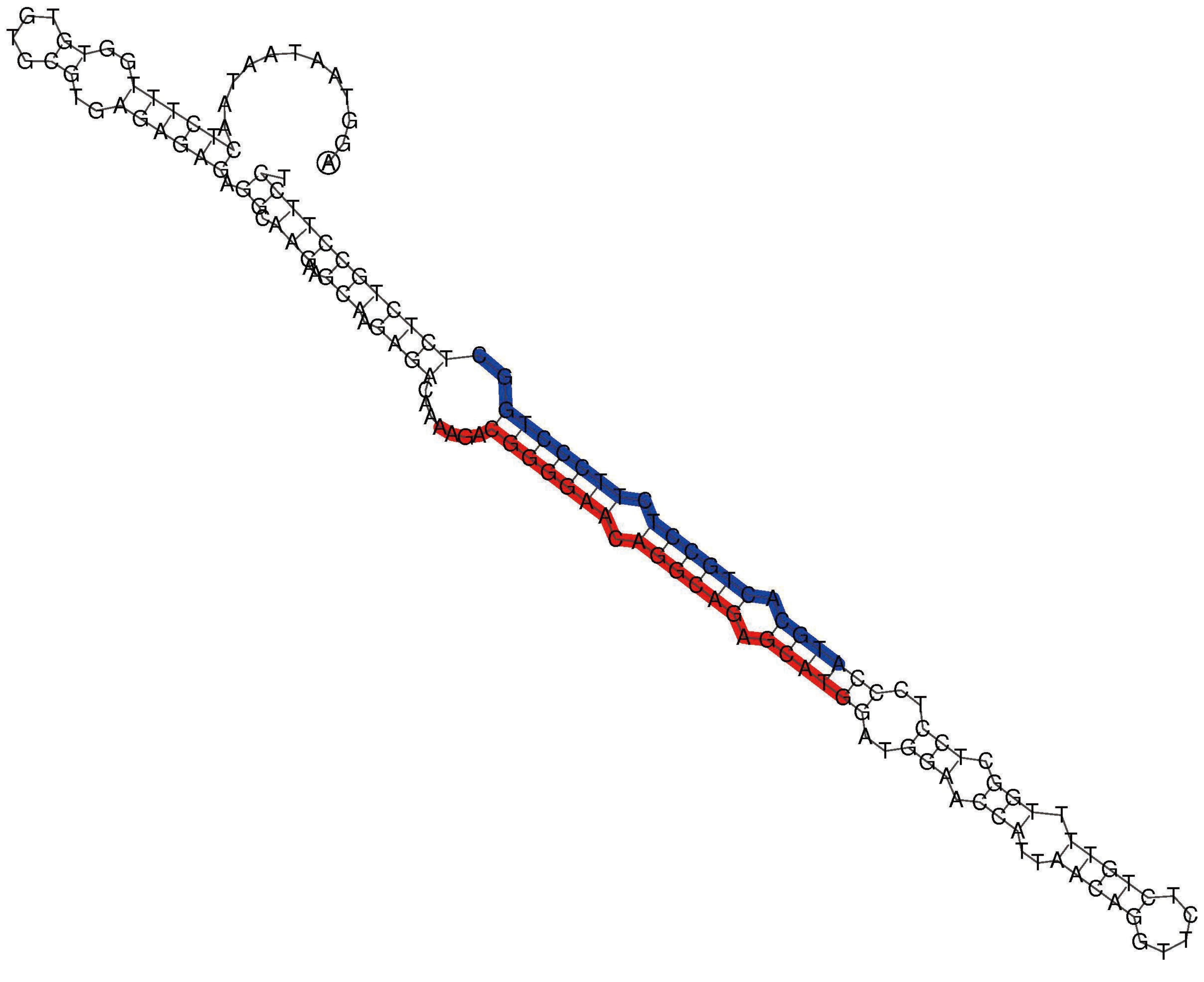

Secondary structure for csi-miR408b

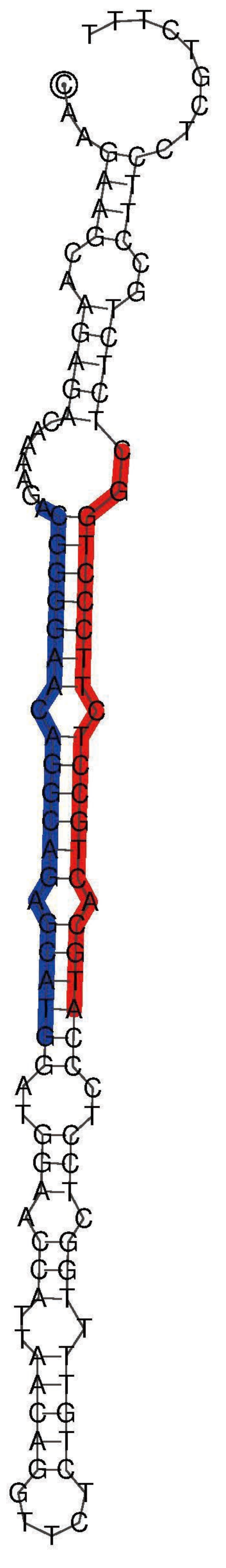

Secondary structure for csi-miR4352b

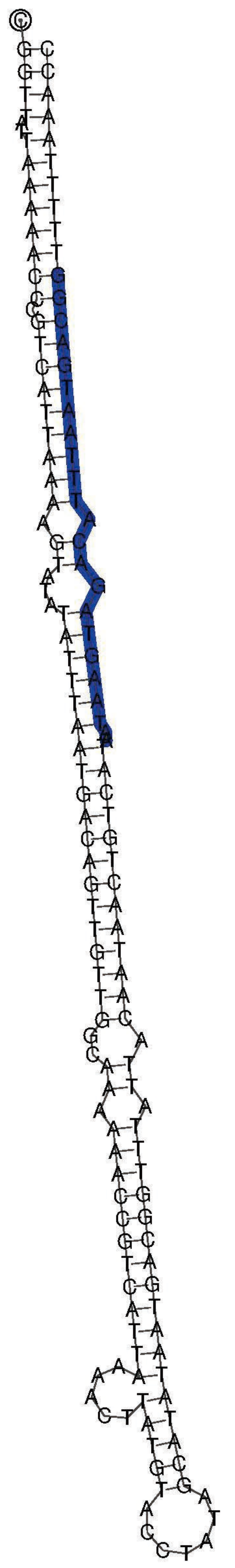

Secondary structure for csi-miR472

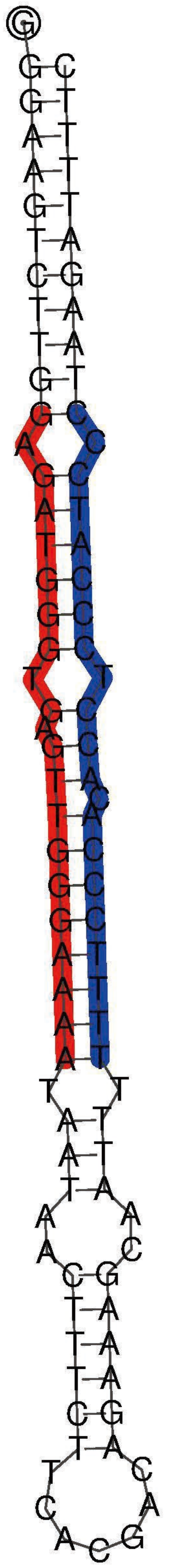

Secondary structure for csi-miR473

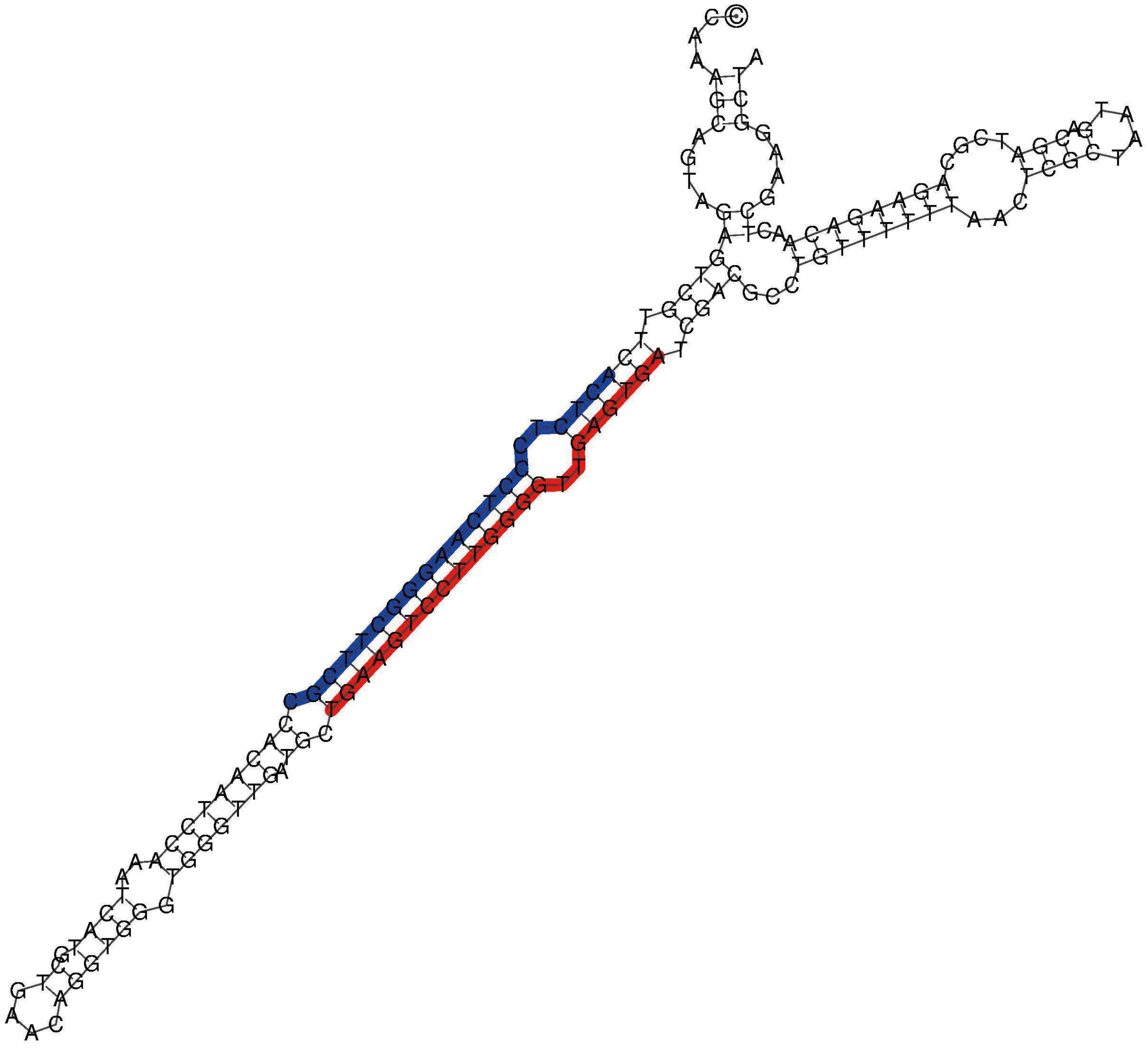

Secondary structure for csi-miR473a-3p

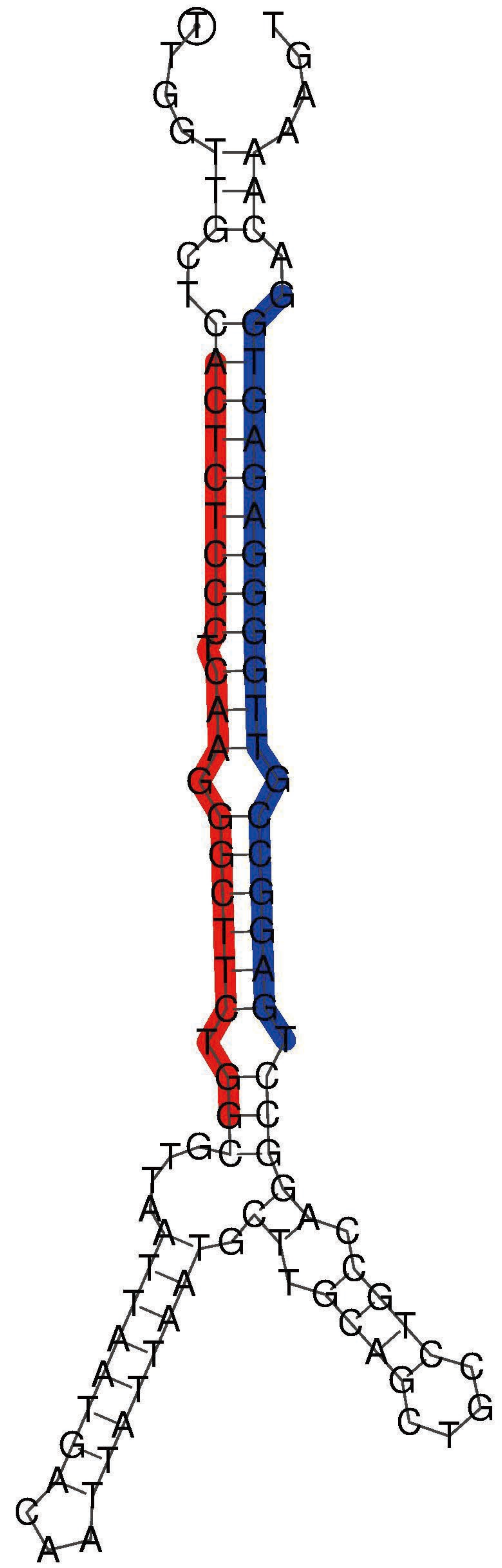

Secondary structure for csi-miR477a

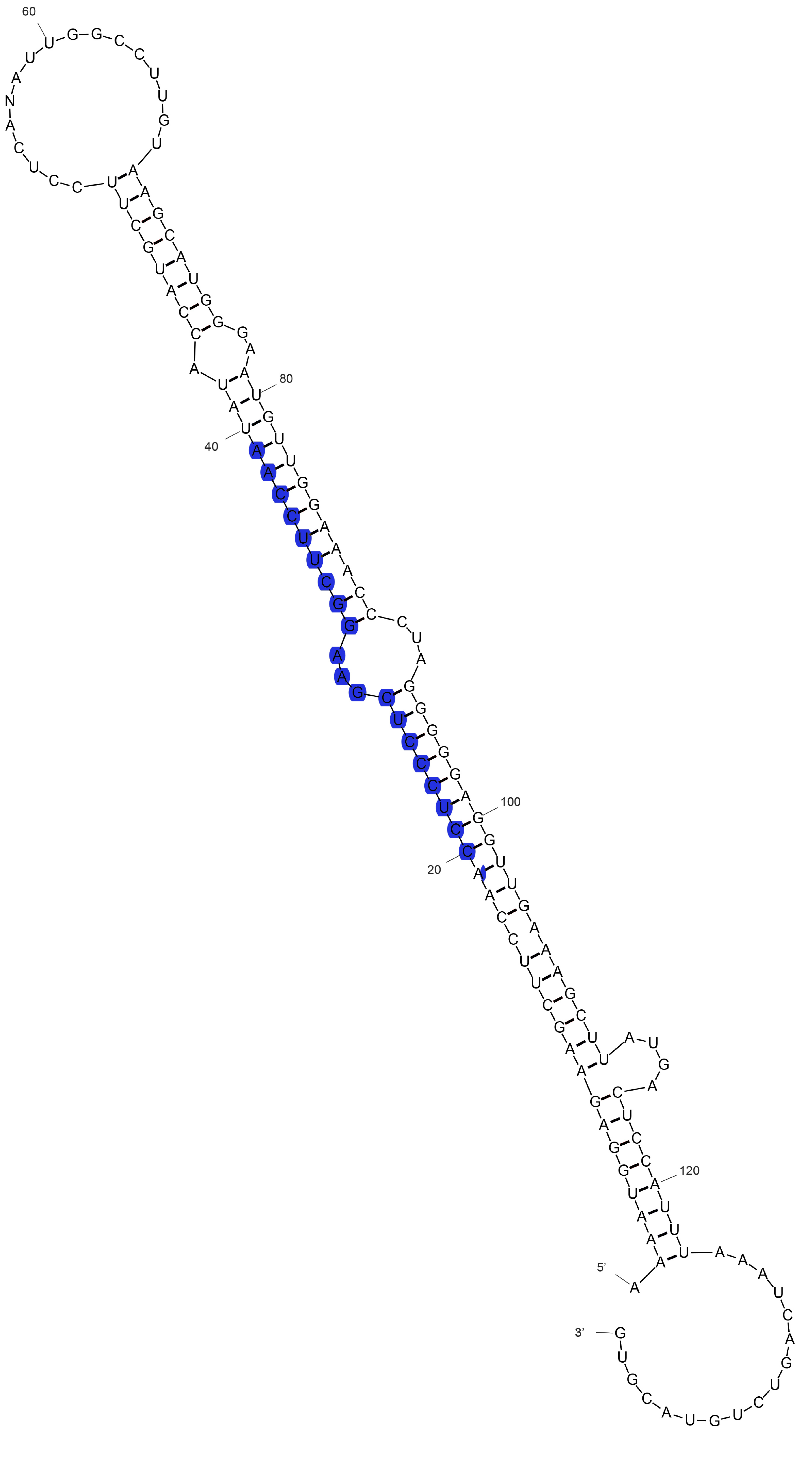

csi-miR477a

Secondary structure for csi-miR477b

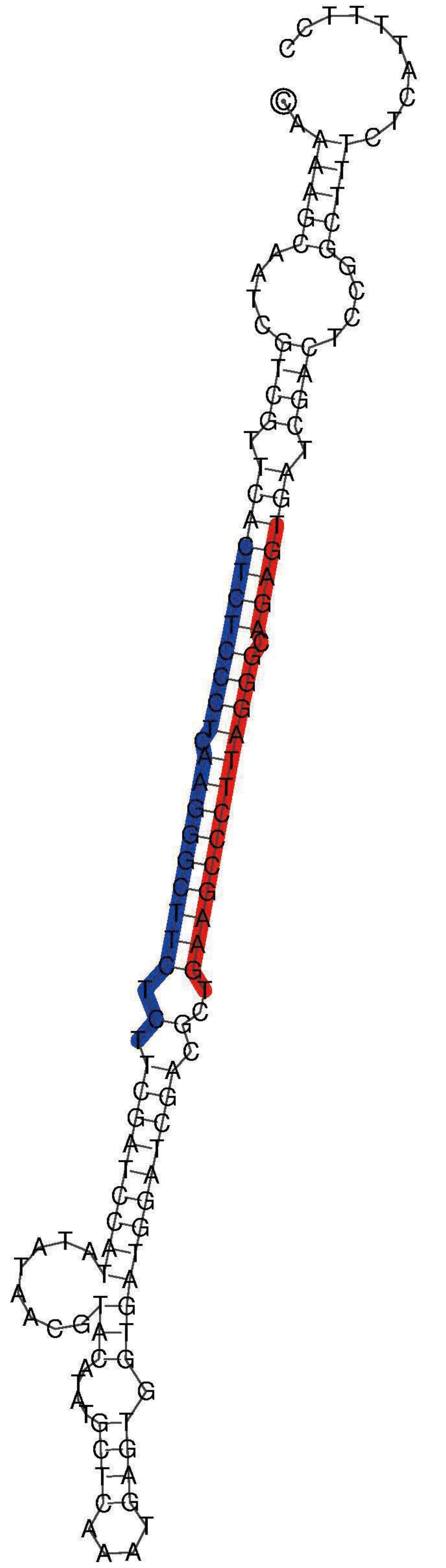

Secondary structure for csi-miR479

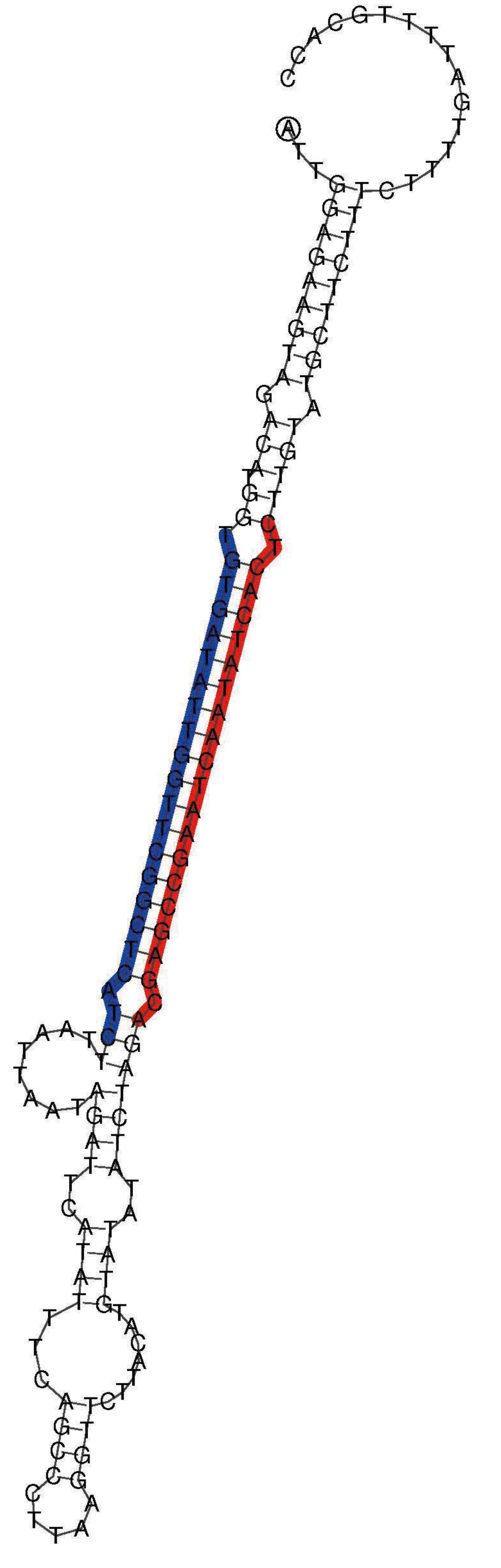

Secondary structure for csi-miR482

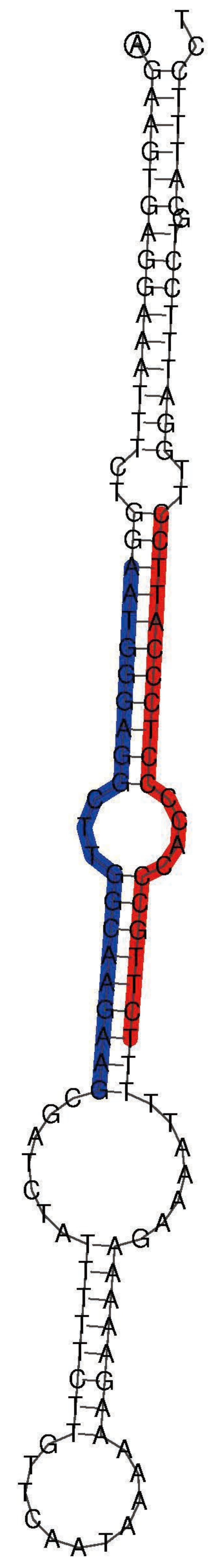

Secondary structure for csi-miR482a-3p

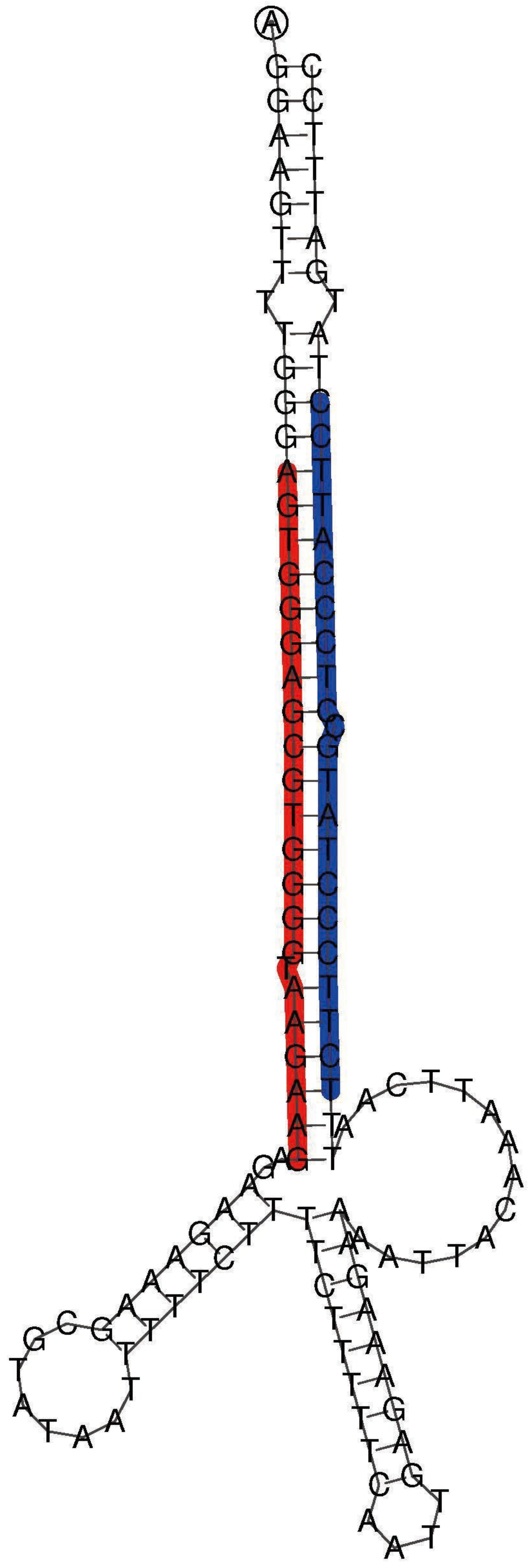

Secondary structure for csi-miR482a-5p

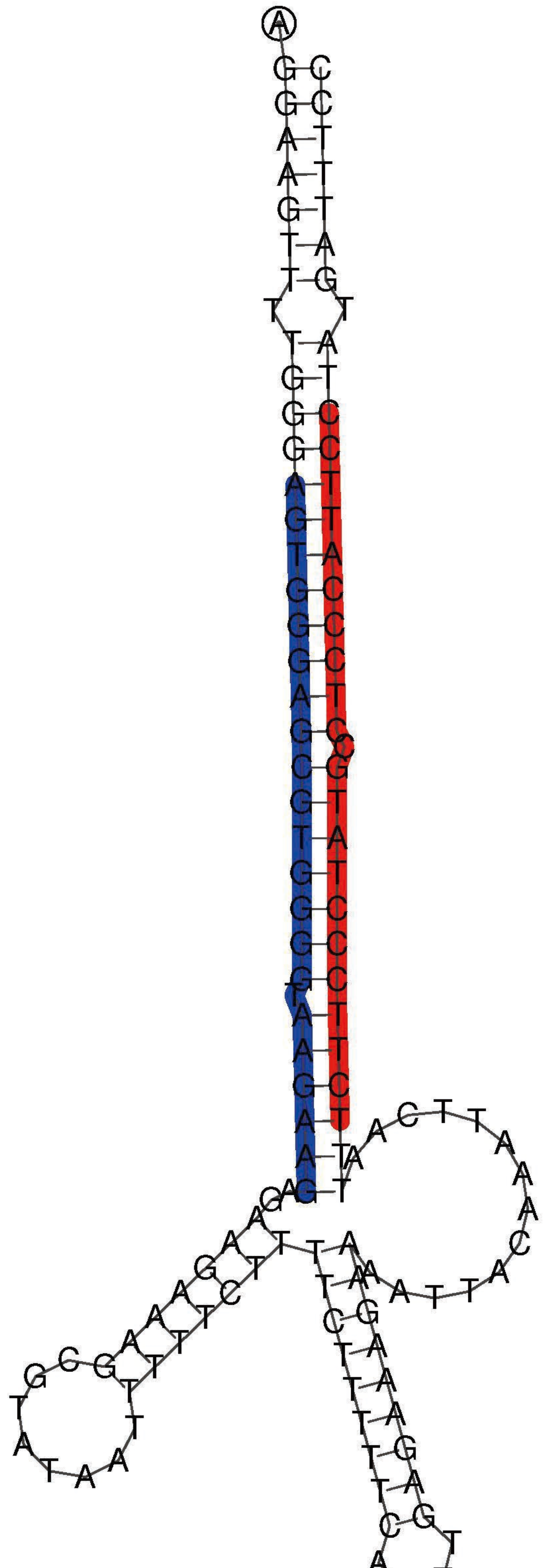

Secondary structure for csi-miR482b

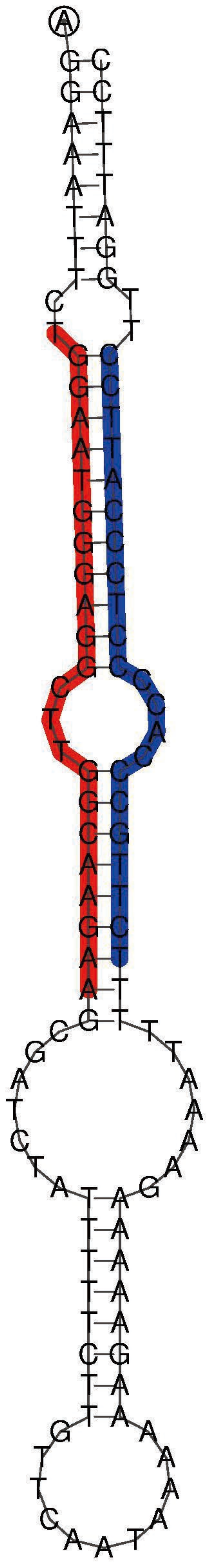

Secondary structure for csi-miR482b-5p

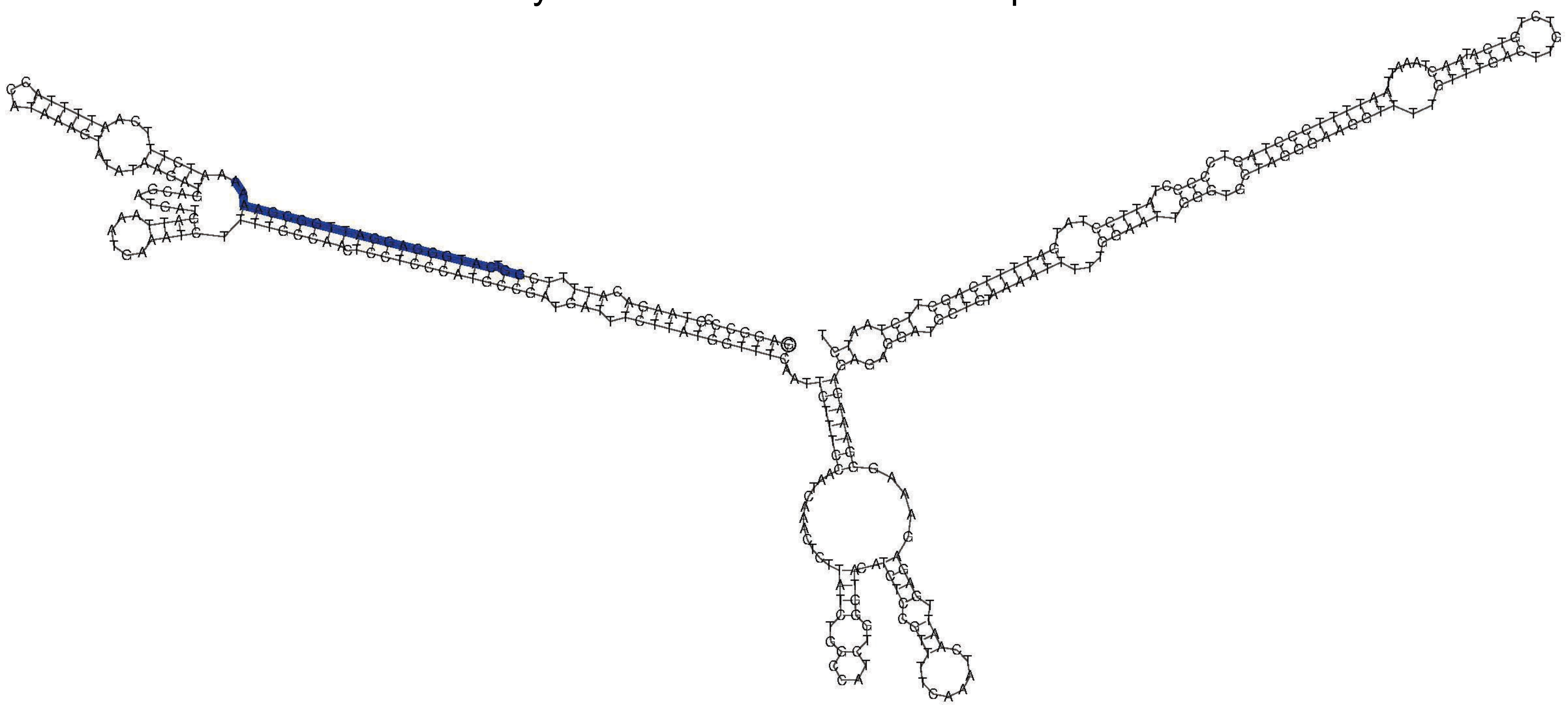

Secondary structure for csi-miR482c

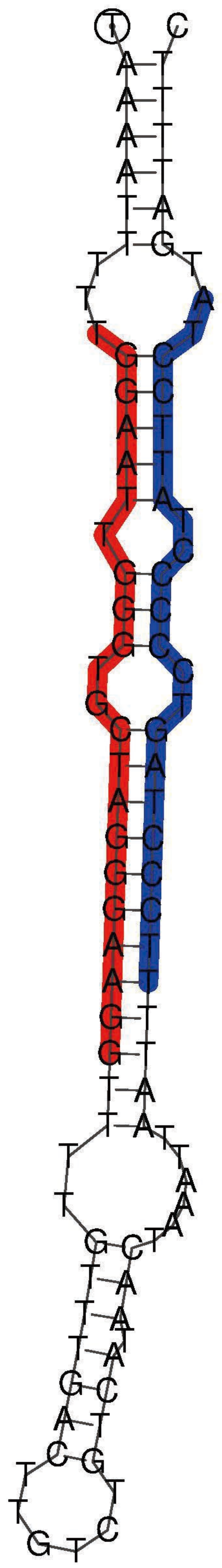

Secondary structure for csi-miR5021

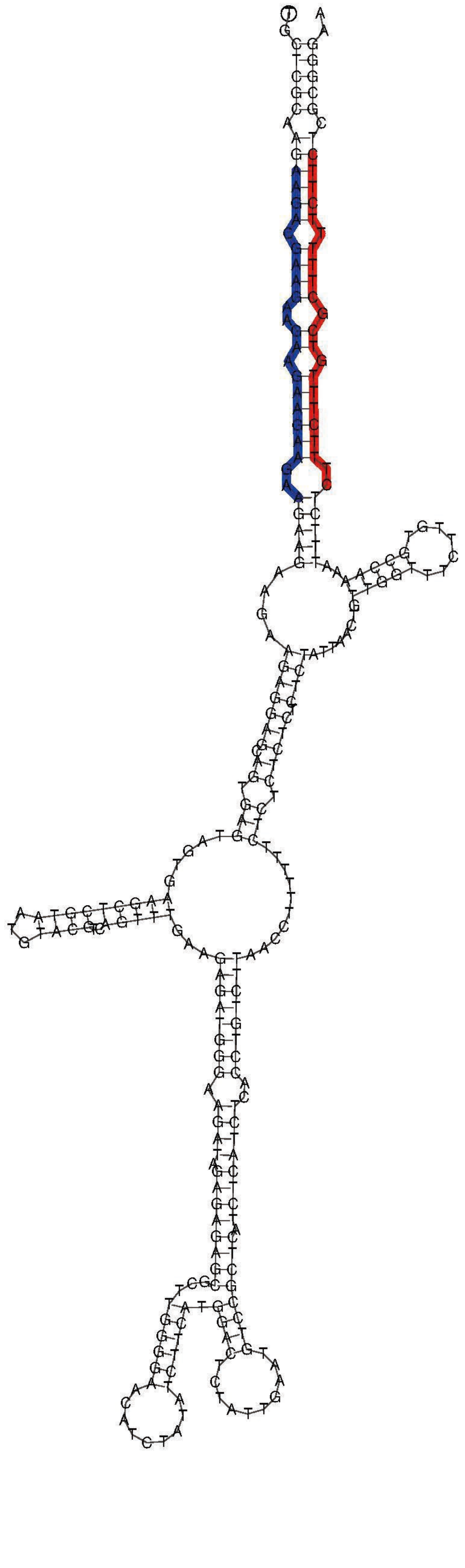

Secondary structure for csi-miR5179

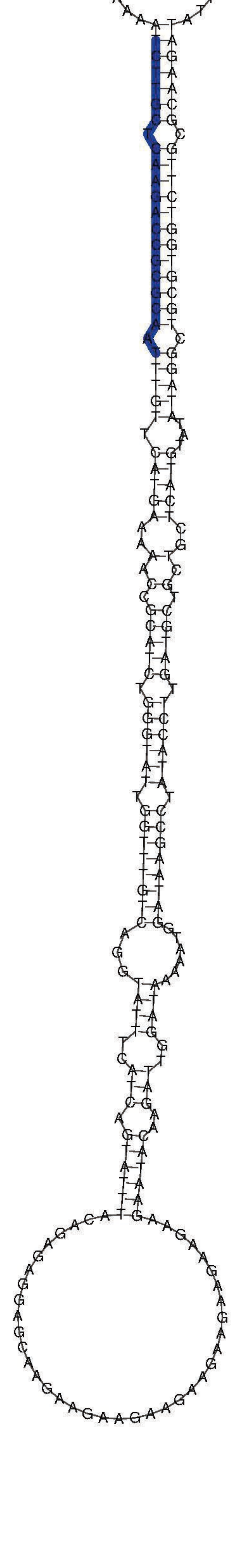

csi-miR5179

Secondary structure for csi-miR5227

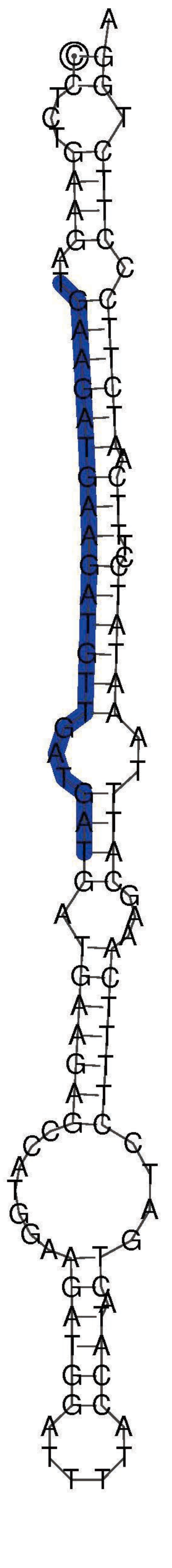

Secondary structure for csi-miR5293

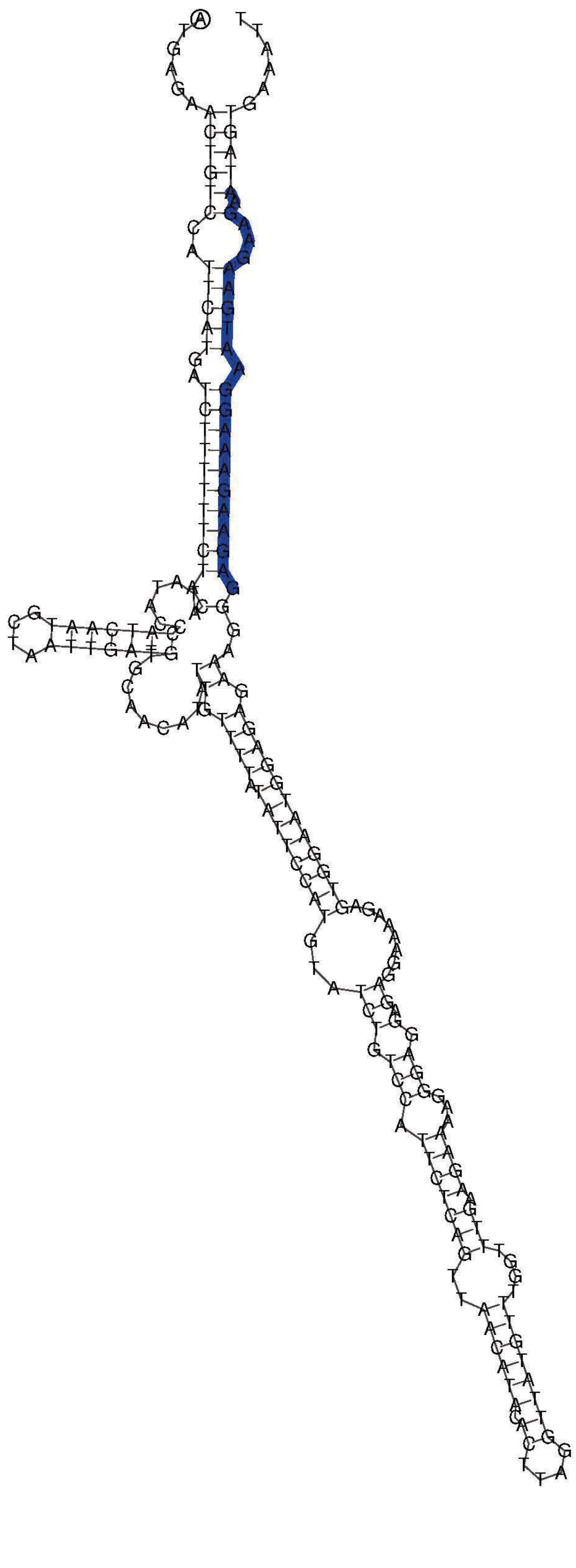

Secondary structure for csi-miR5298b

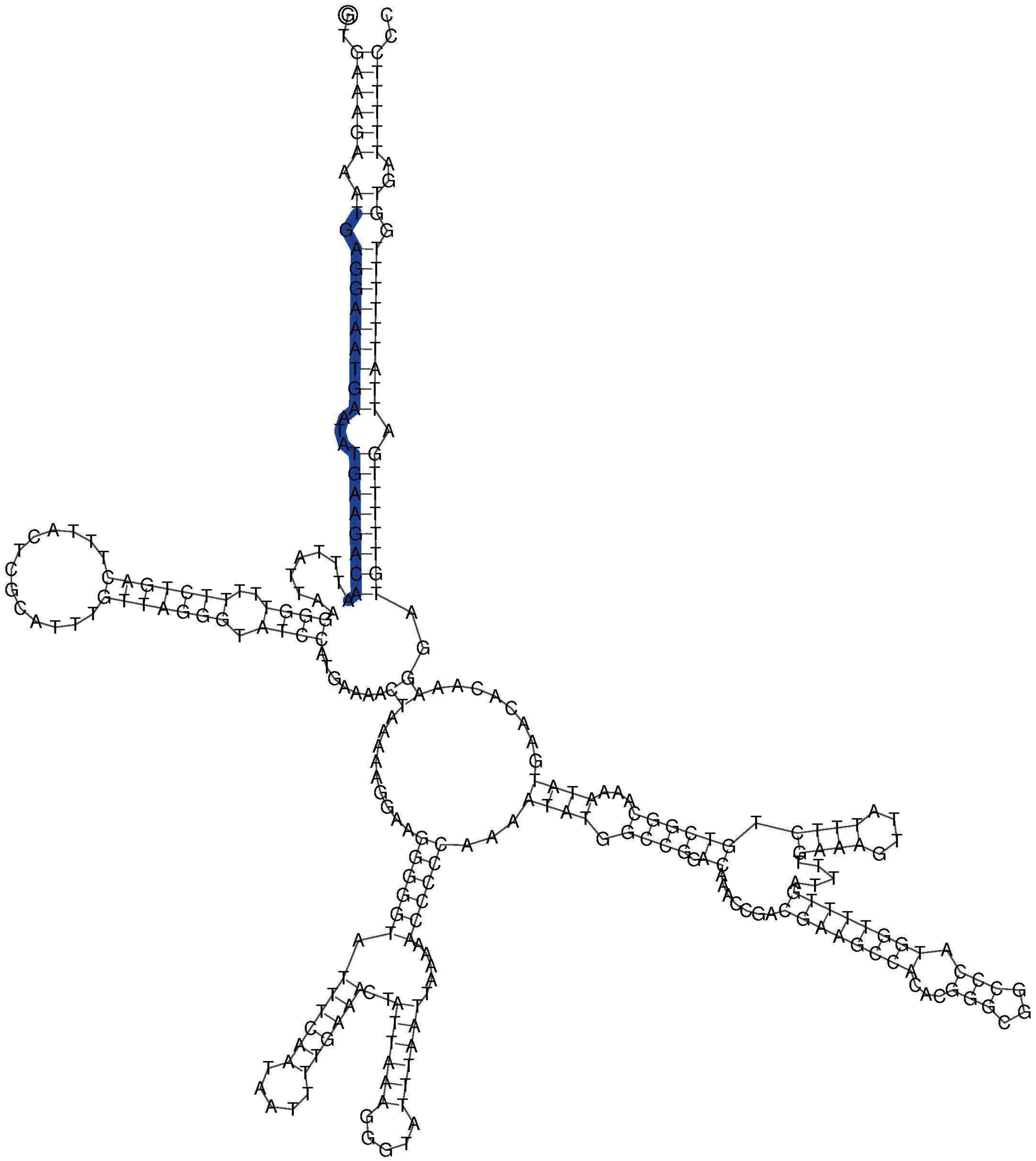

Secondary structure for csi-miR530a

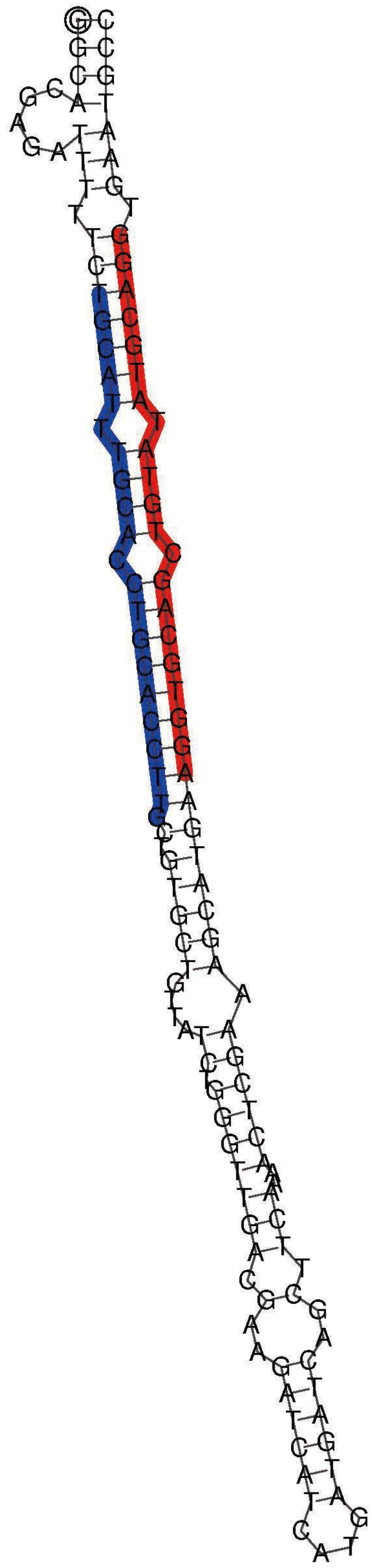

Secondary structure for csi-miR530b

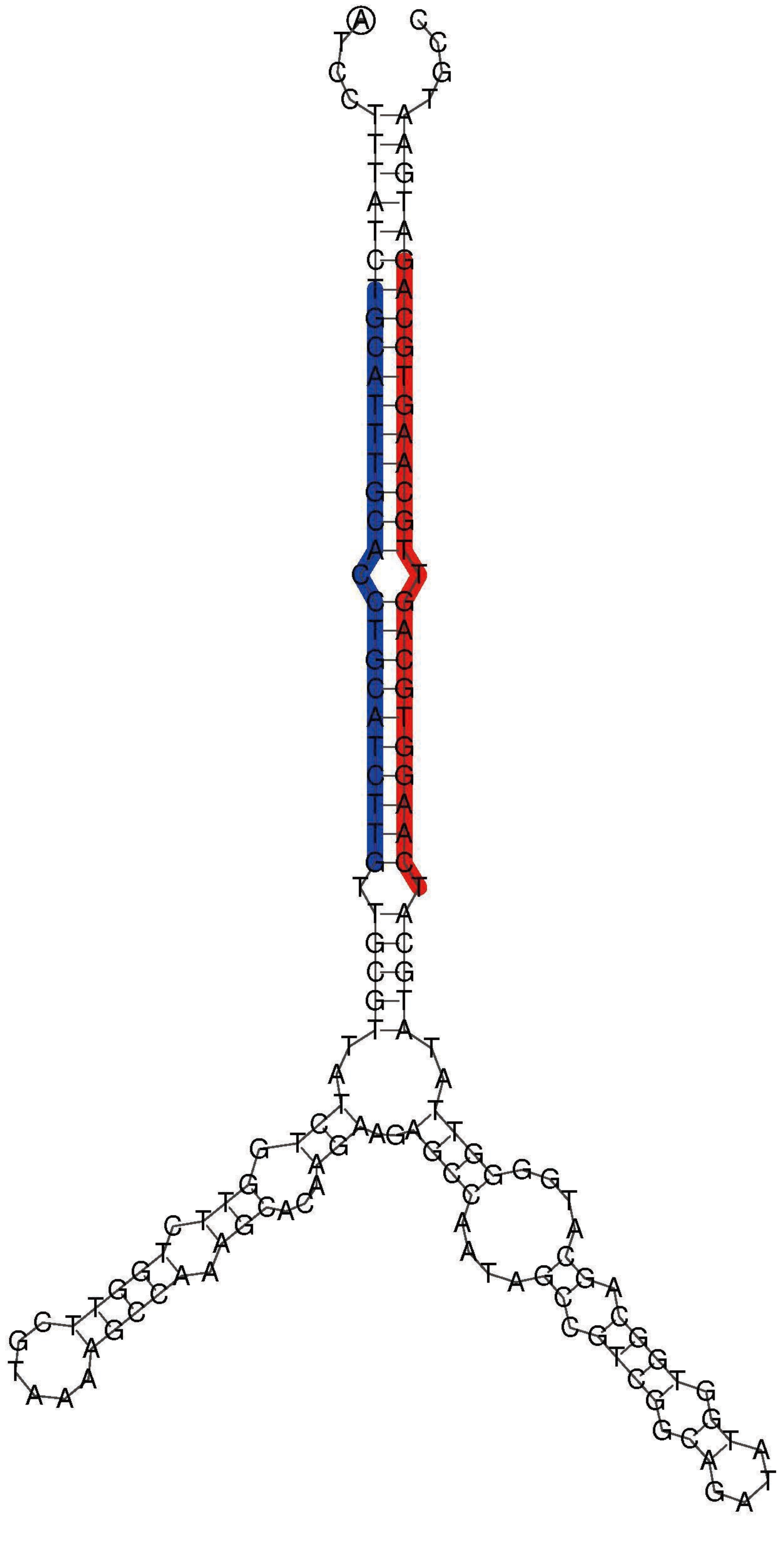

Secondary structure for csi-miR535

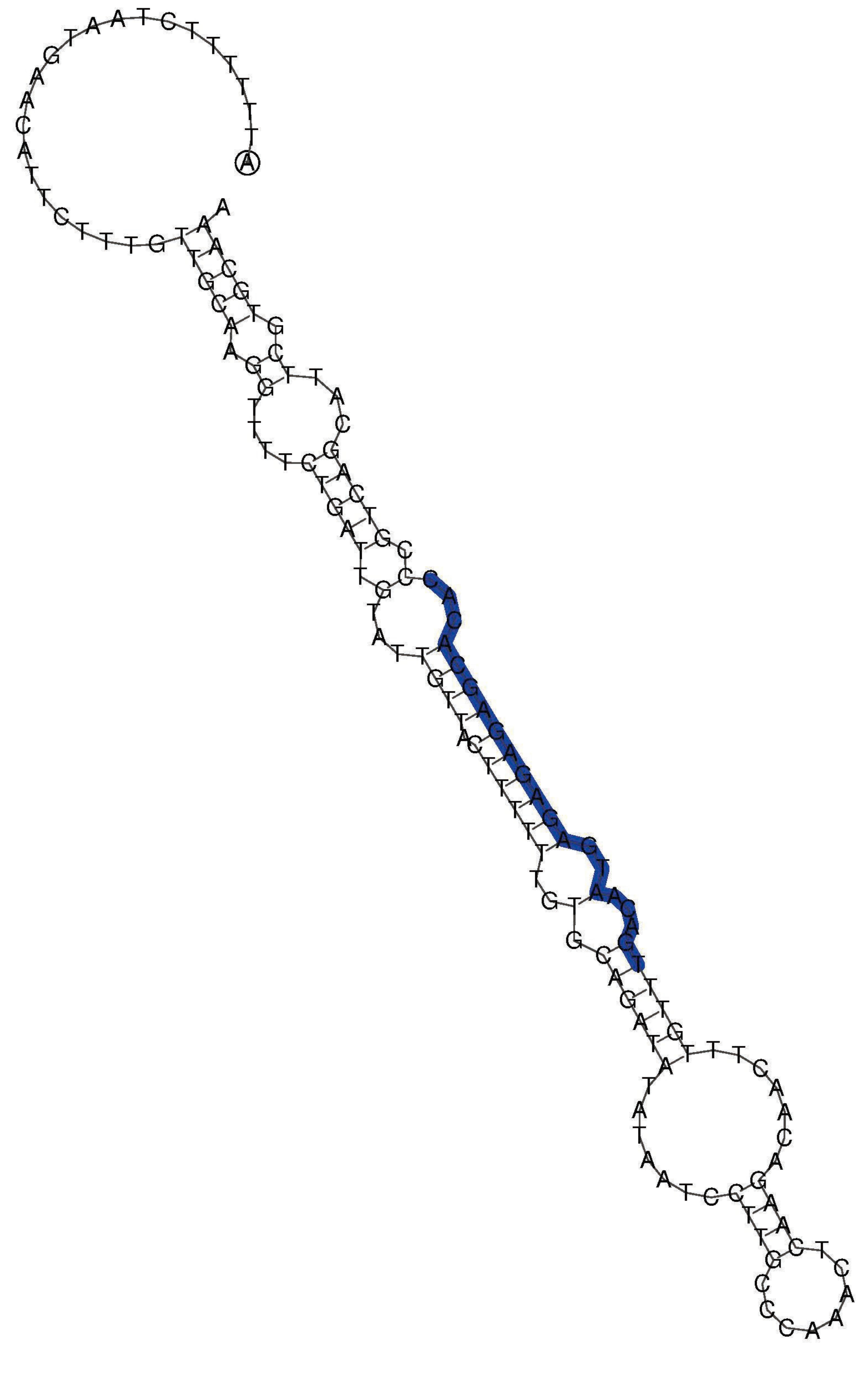

Secondary structure for csi-miR5373

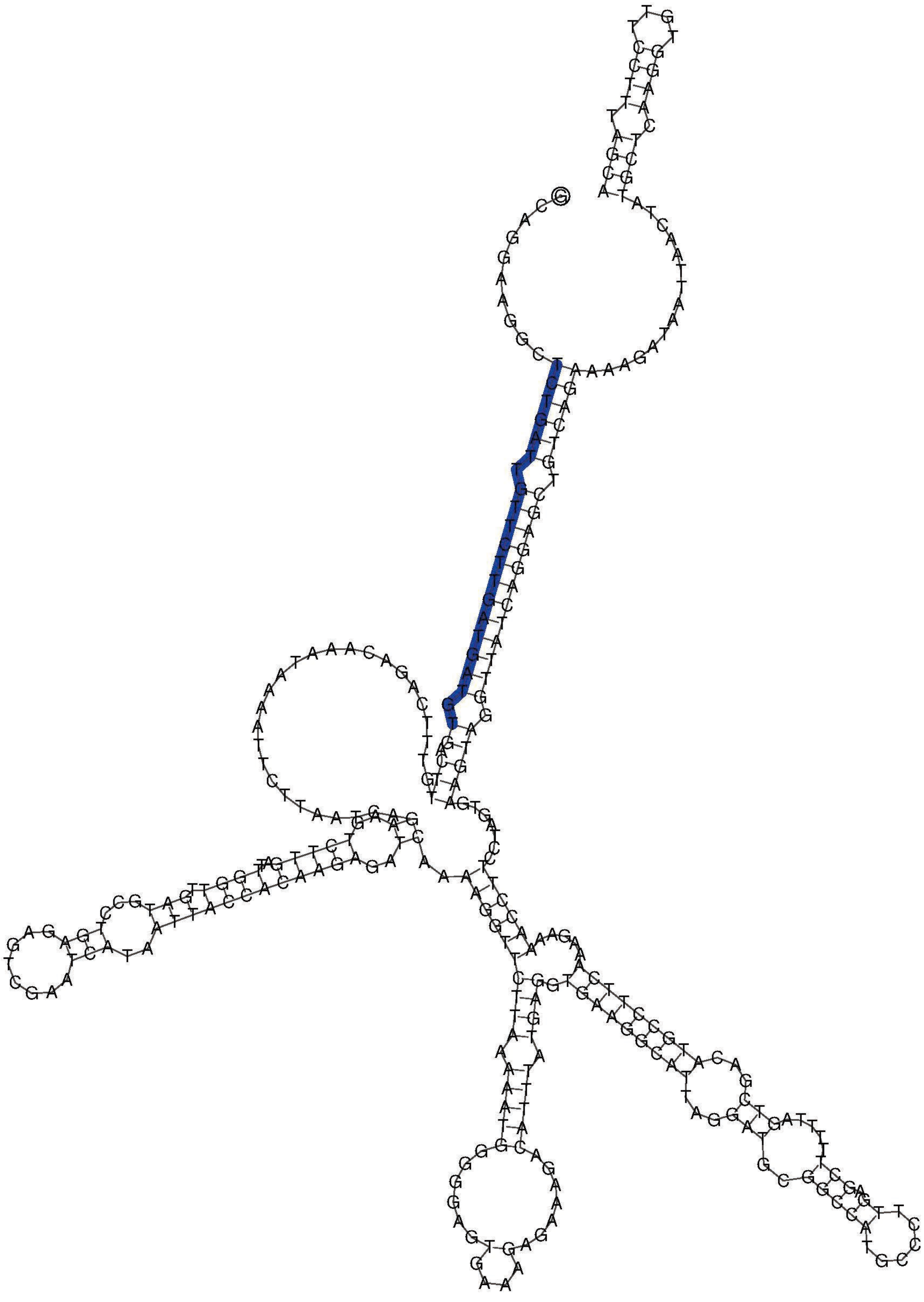

Secondary structure for csi-miR5673

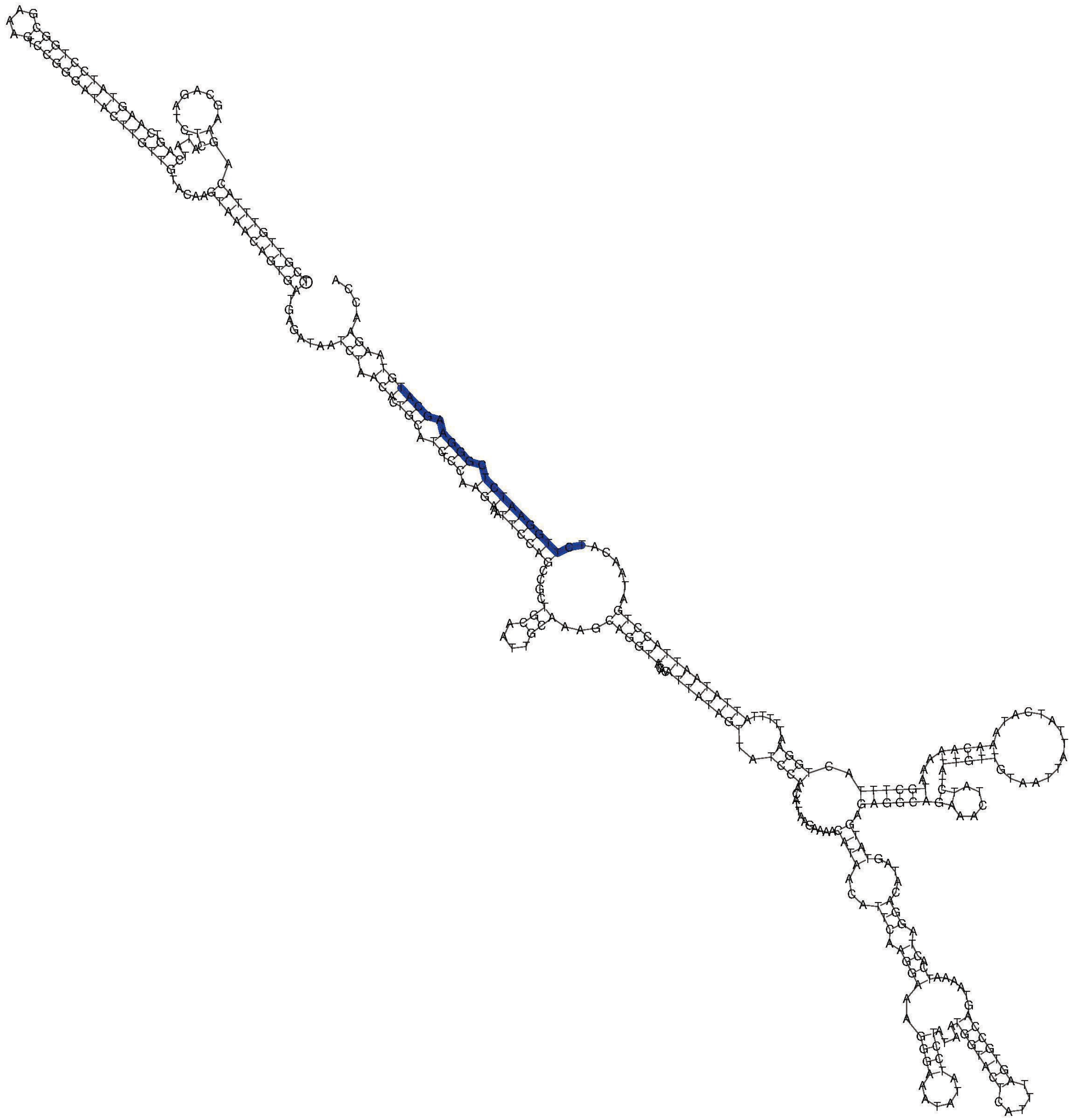

Secondary structure for csi-miR6196

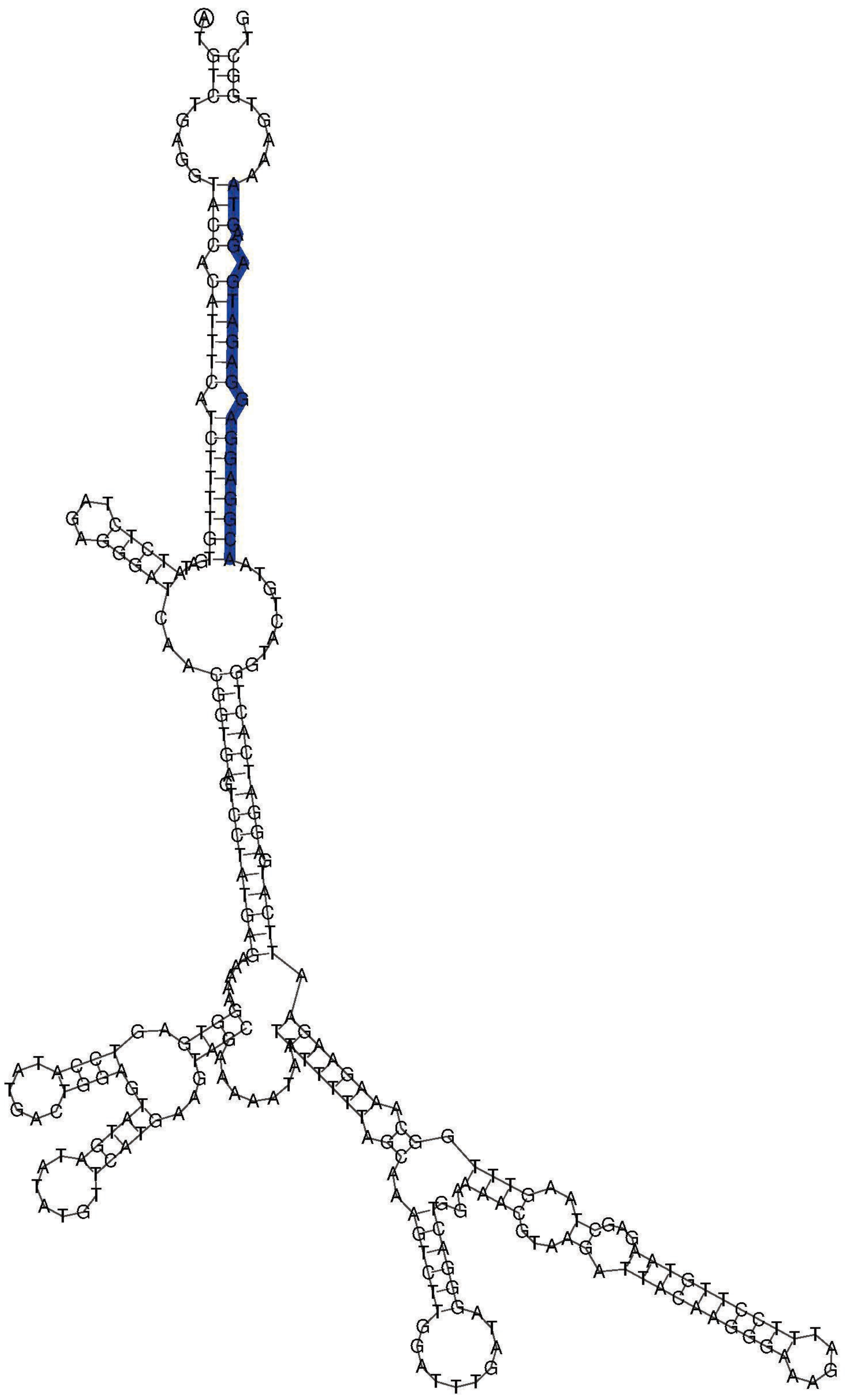

Secondary structure for csi-miR6295

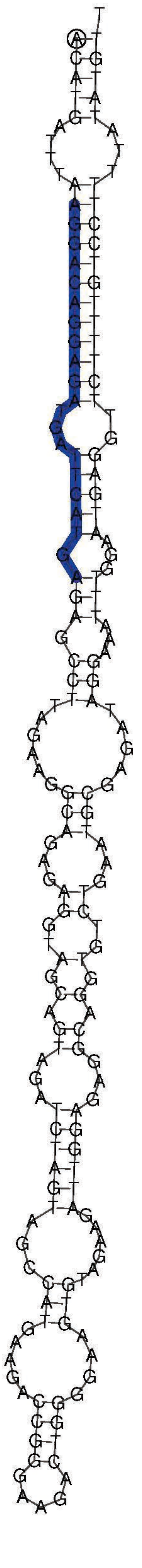

Secondary structure for csi-miR6300

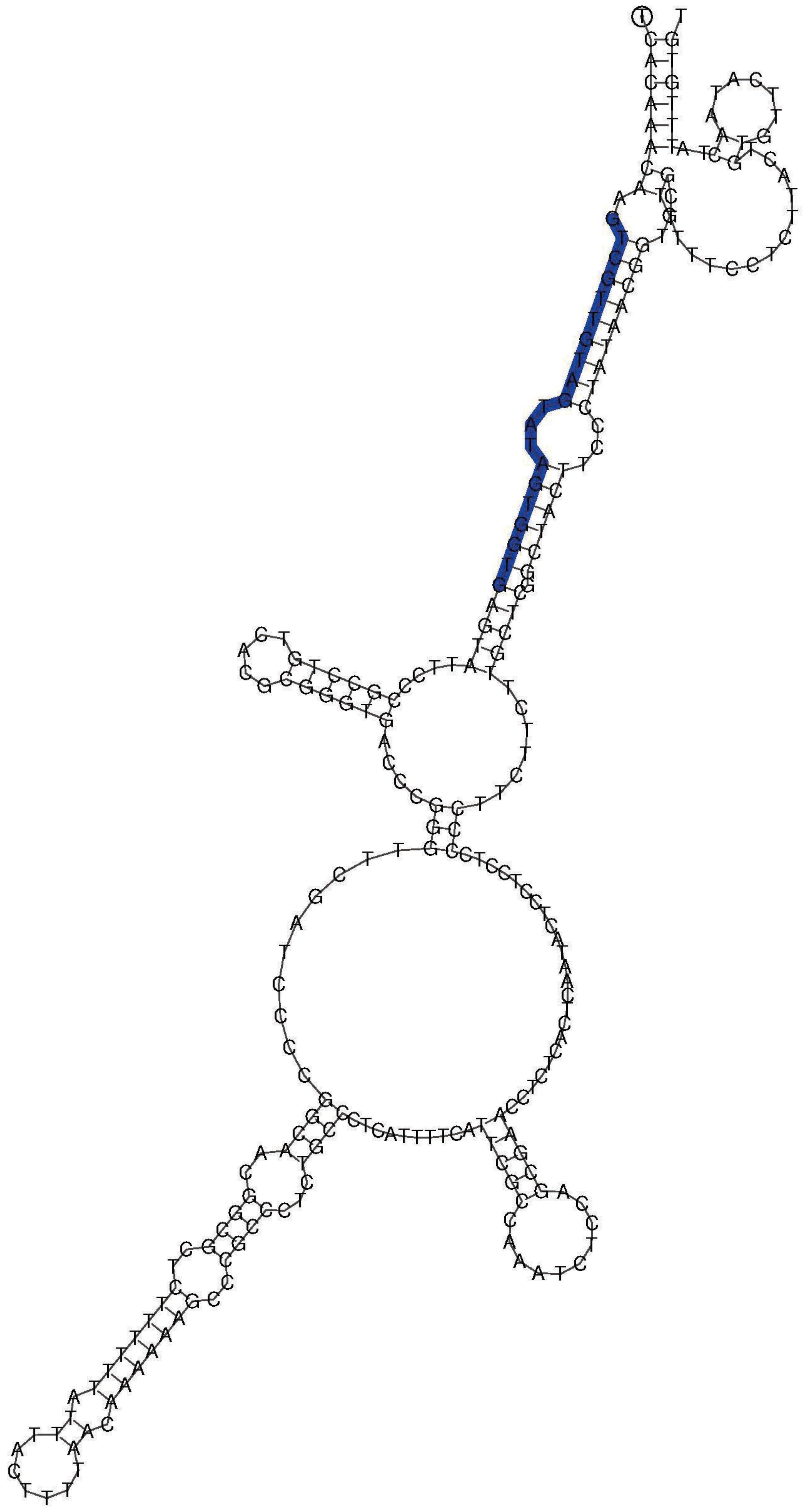

Secondary structure for csi-miR7812

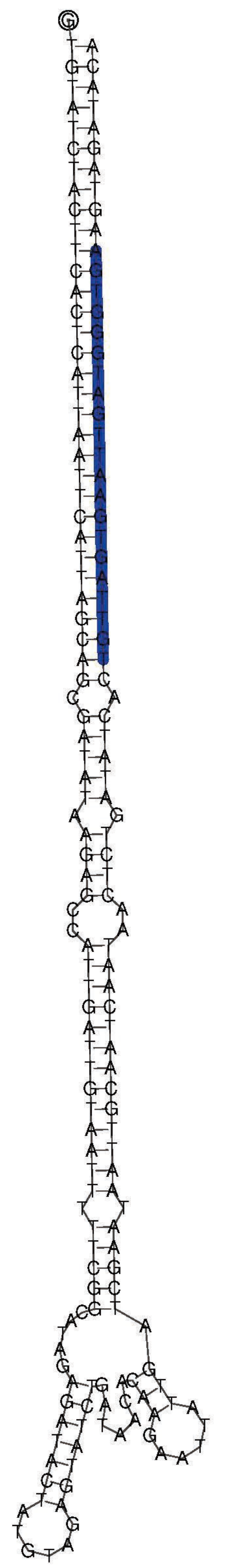

Secondary structure for csi-miR827

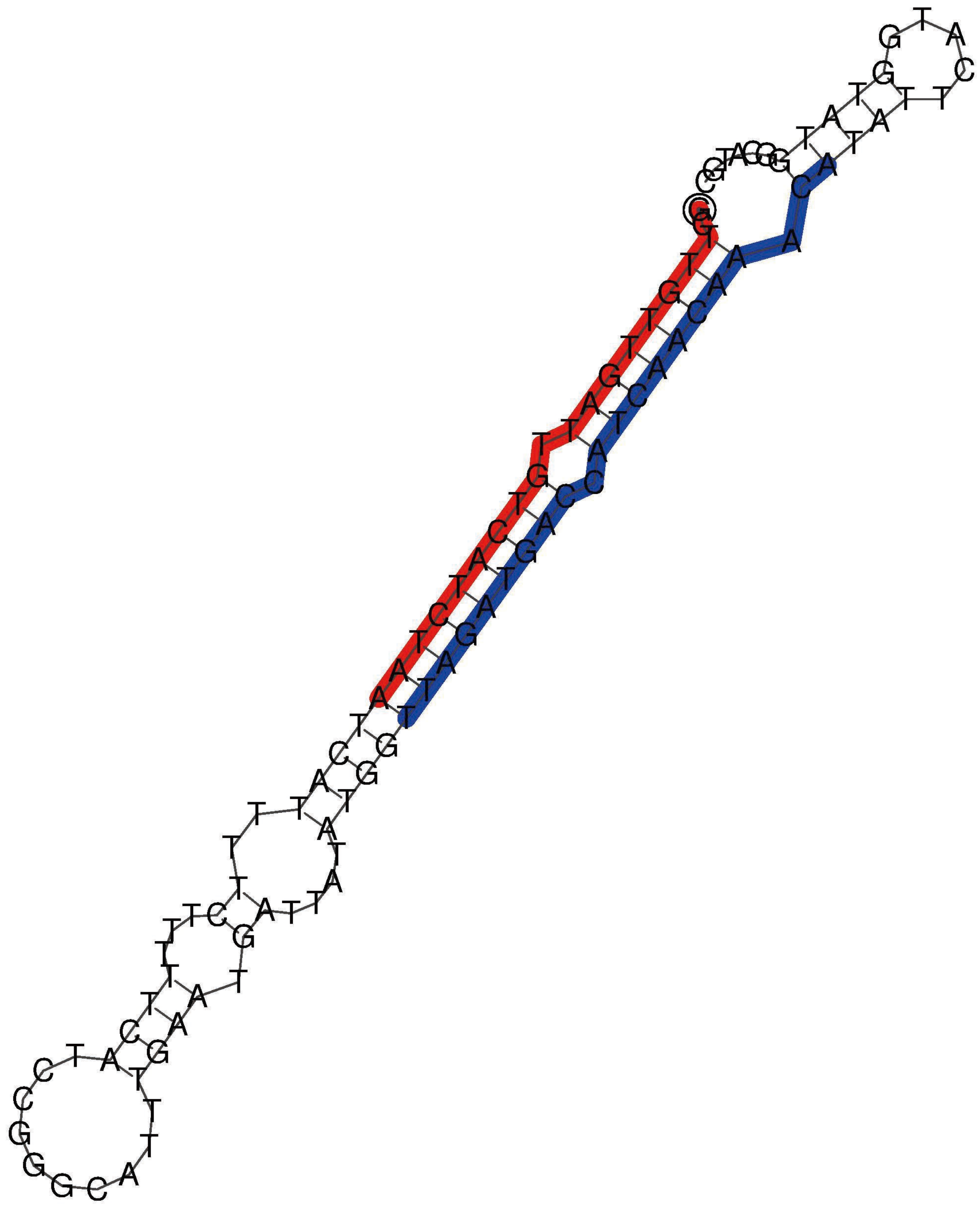

Secondary structure for csi-miR827-5p

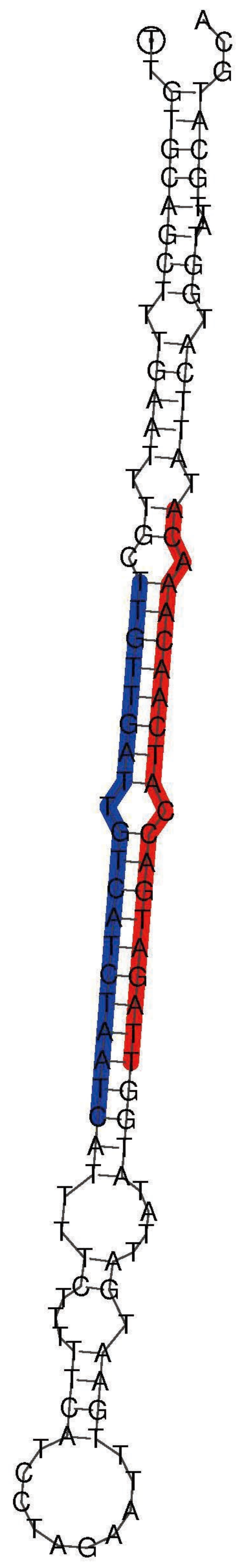

Secondary structure for csi-miR845c

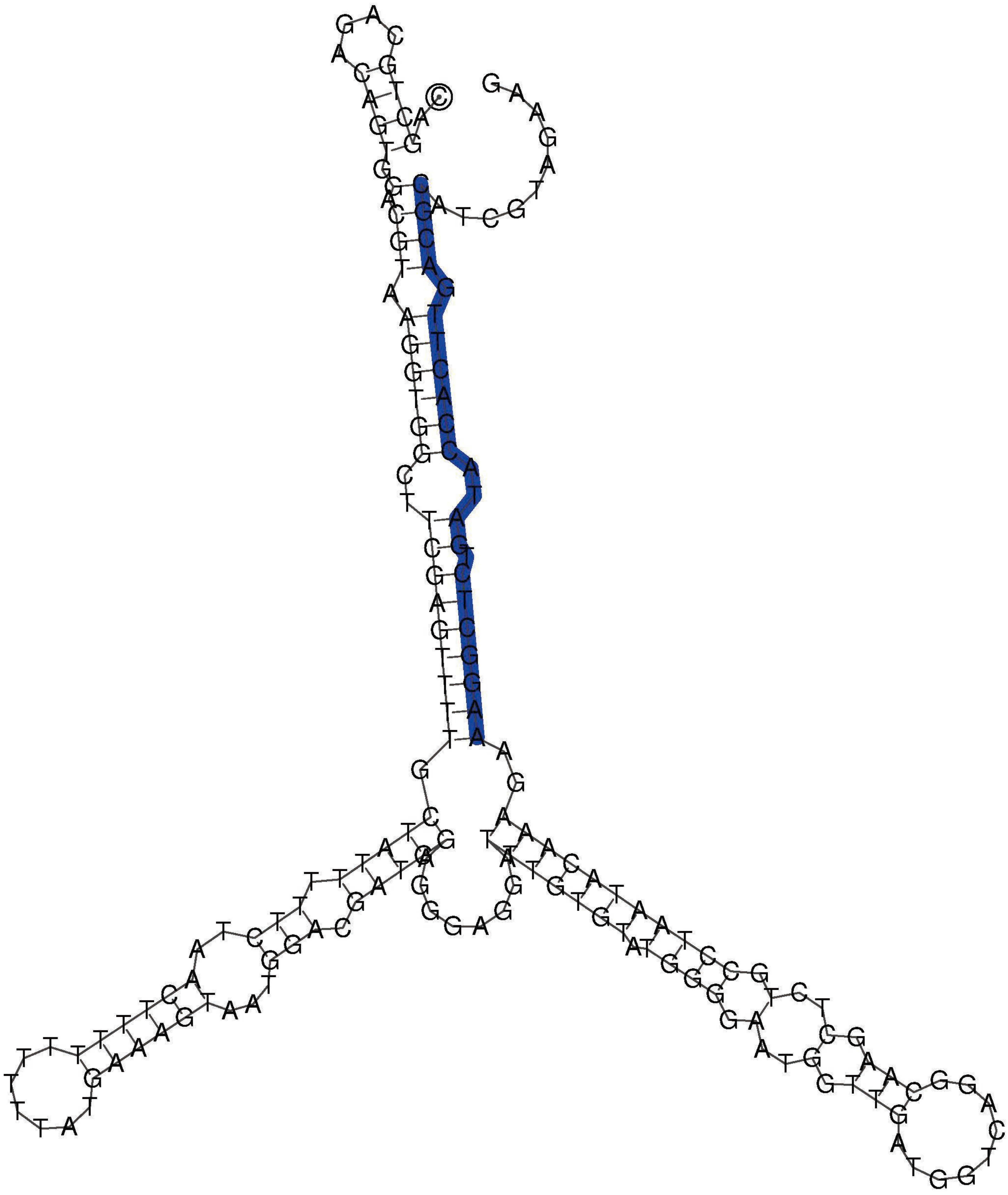

Secondary structure for csi-miR857

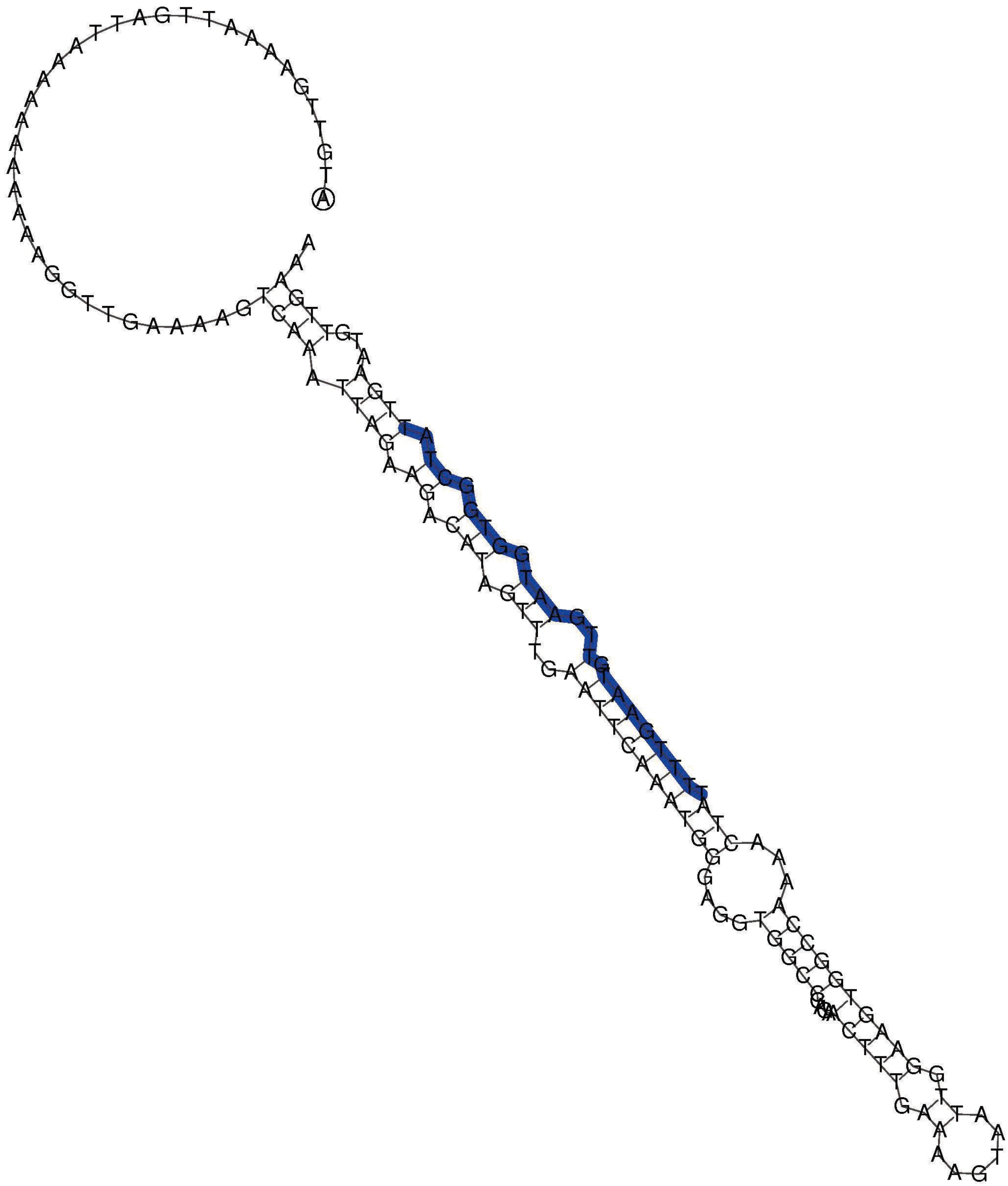

Supplement: Figure S3 — Predicted secondary structures of the known miRNAs. The mature miRNA sequences are highlighted in blue and the miRNA* sequences are highlighted in red. [file Image3.PDF]
