# Supplementary material for: Comparative Analysis of miRNAs and Their Target Transcripts between a Spontaneous Late-Ripening Sweet Orange Mutant and Its Wild-Type Using Small RNA and Degradome Sequencing
Source: Front Plant Sci. 2016 Sep 21;7:1416. doi: 10.3389/fpls.2016.01416 (PMC5030777; doi:10.3389/fpls.2016.01416)

Secondary structure for csi-miRN01

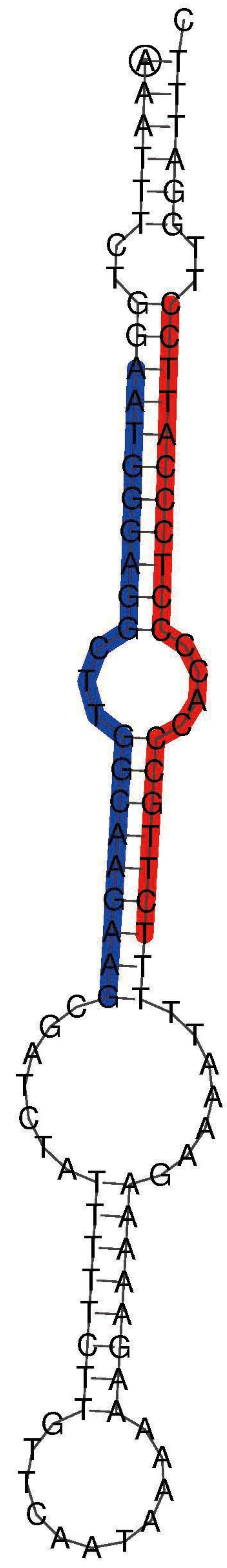

Secondary structure for csi-miRN02

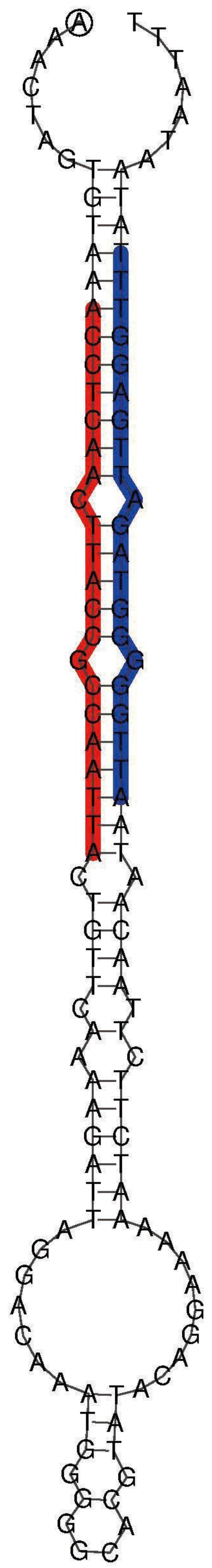

Secondary structure for csi-miRN03

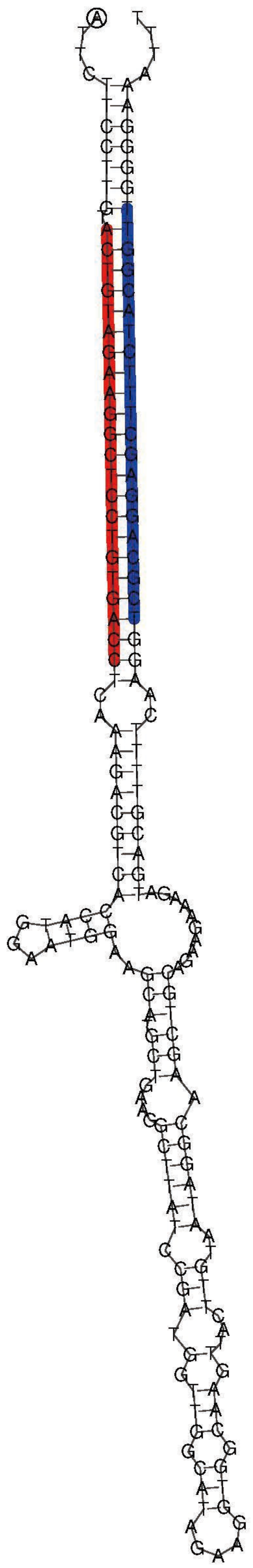

Secondary structure for csi-miRN04

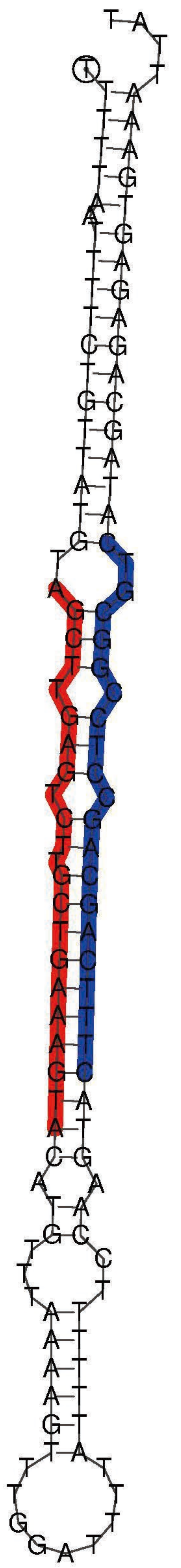

Secondary structure for csi-miRN05

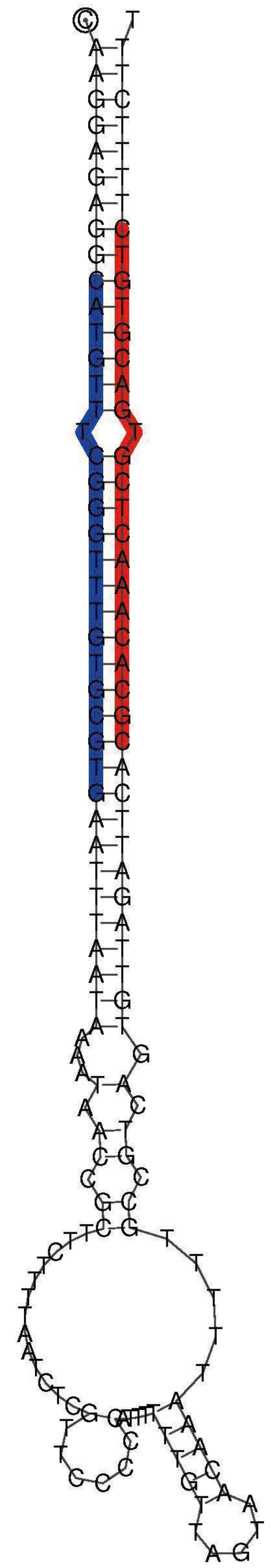

Secondary structure for csi-miRN06

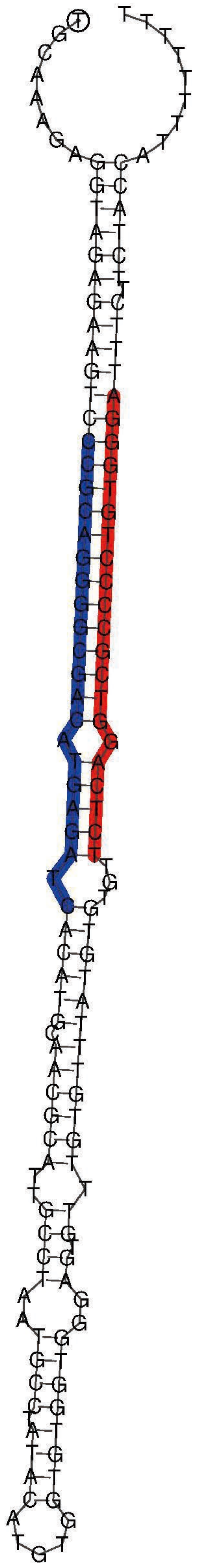

Secondary structure for csi-miRN07

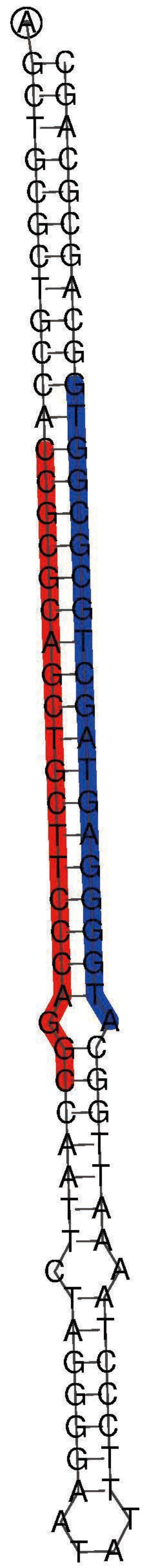

Secondary structure for csi-miRN08

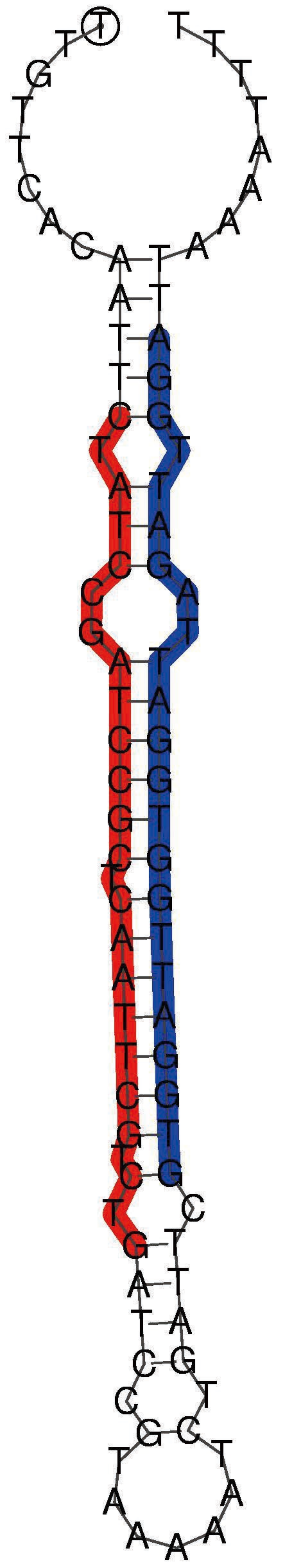

Secondary structure for csi-miRN09

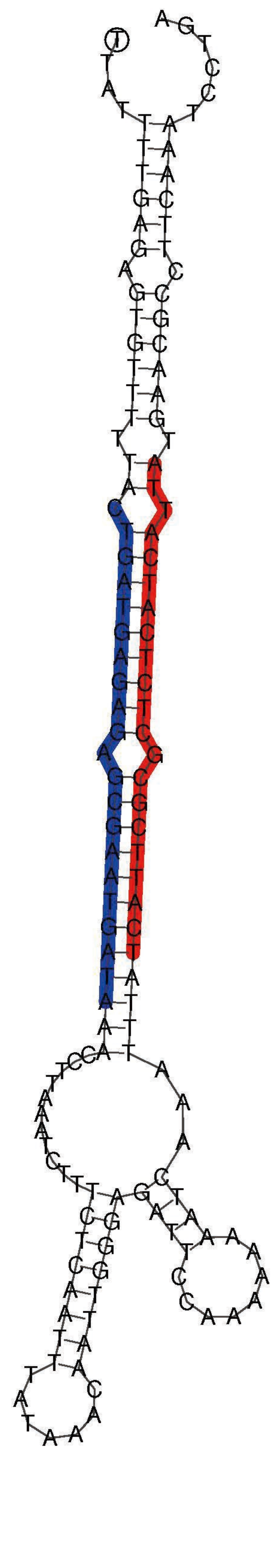

Secondary structure for csi-miRN10

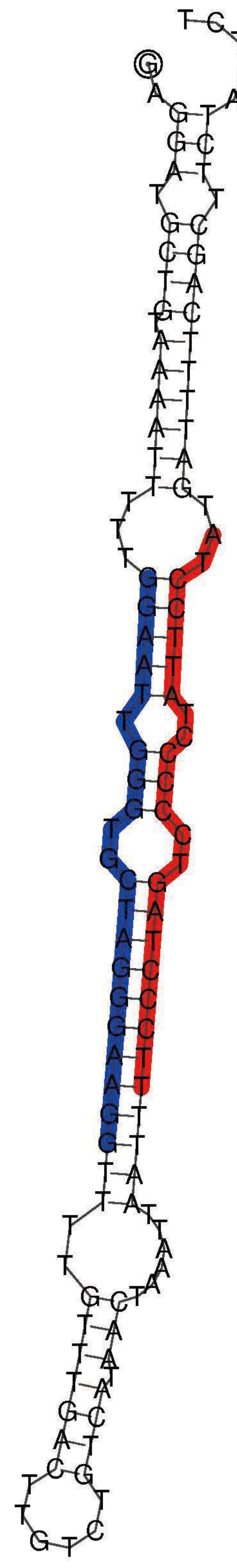

Secondary structure for csi-miRN11

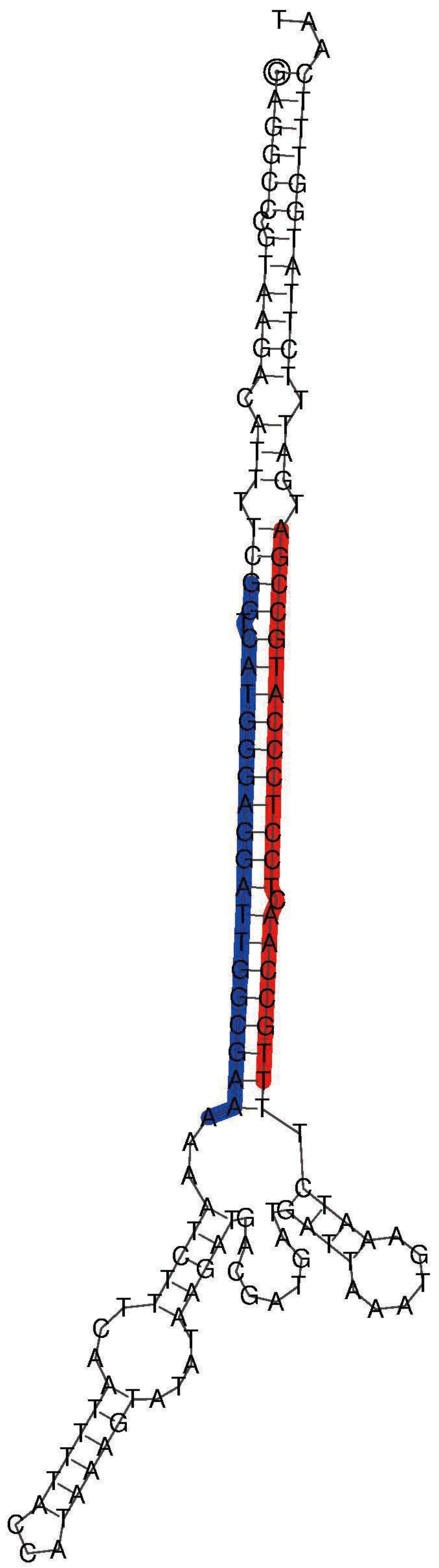

Secondary structure for csi-miRN12

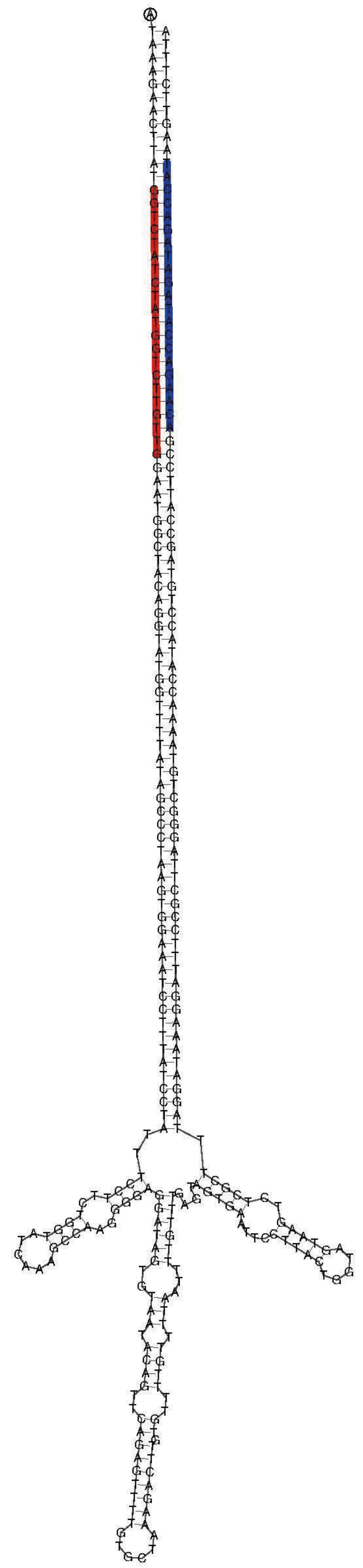

Secondary structure for csi-miRN13

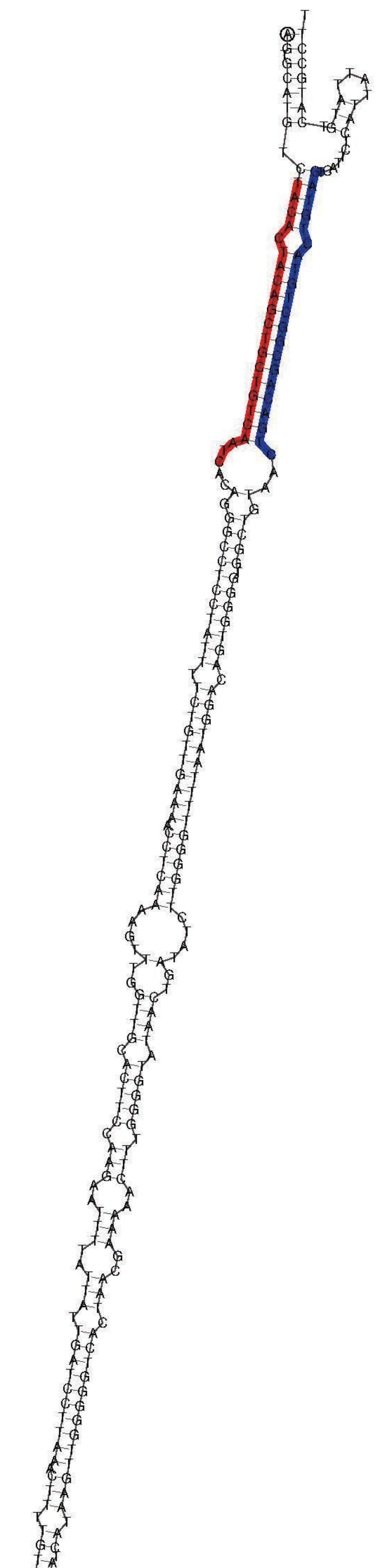

Secondary structure for csi-miRN14

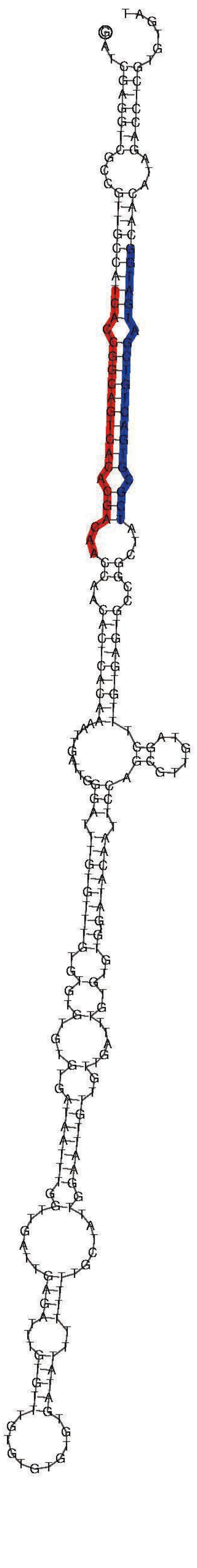

Secondary structure for csi-miRN15

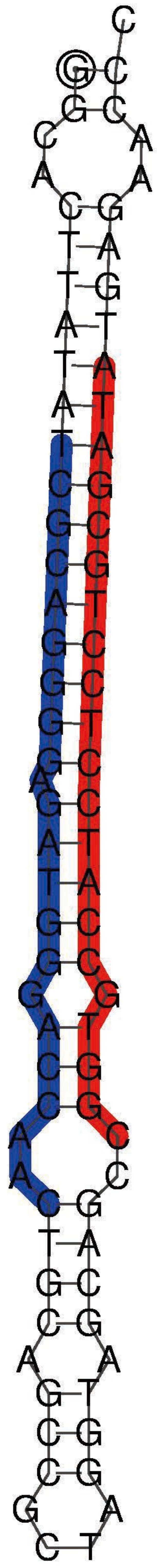

Secondary structure for csi-miRN16

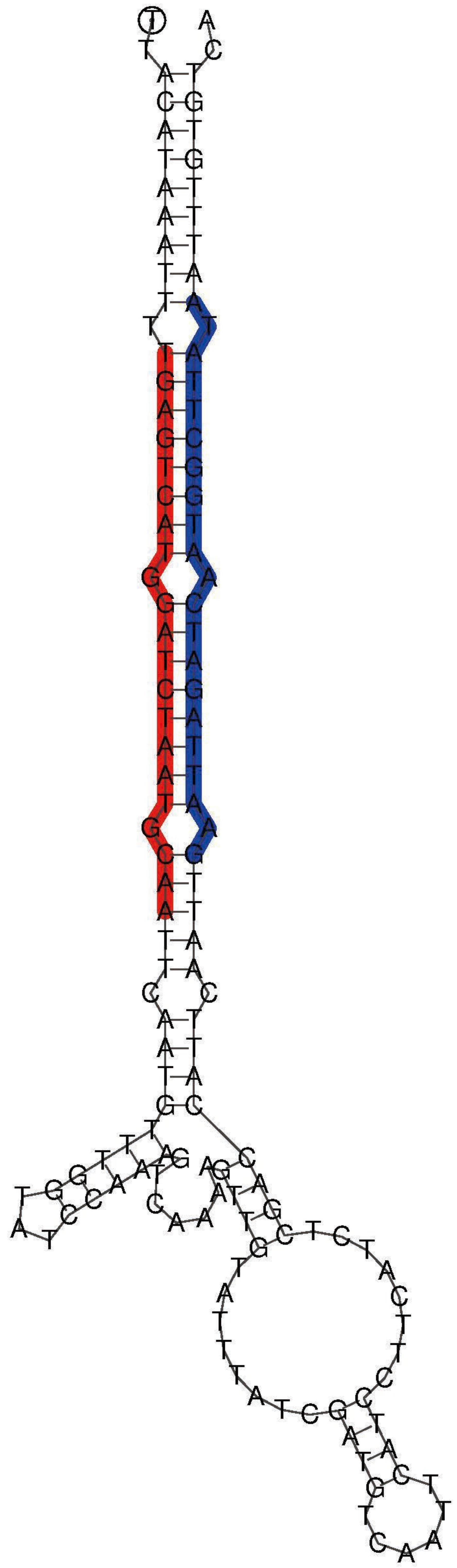

Secondary structure for csi-miRN17

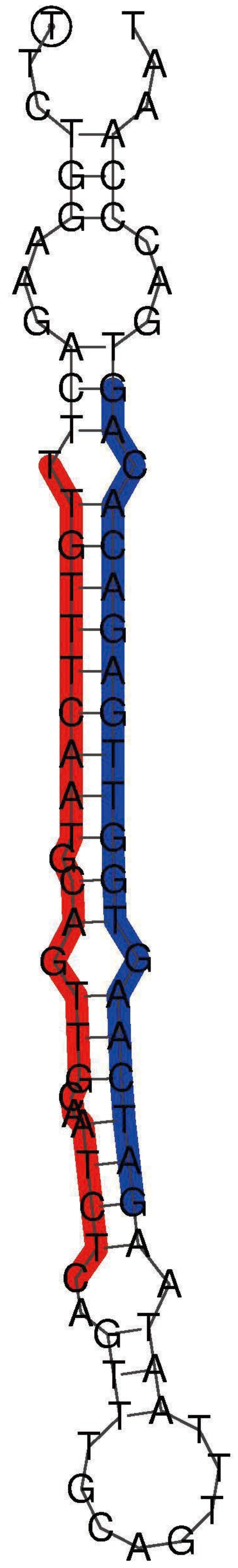

Secondary structure for csi-miRN18

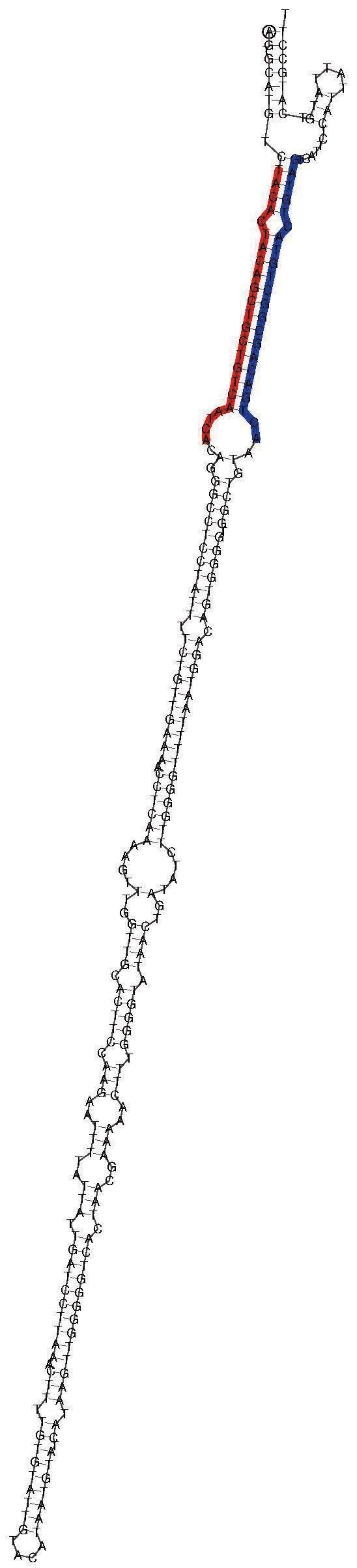

Secondary structure for csi-miRN19

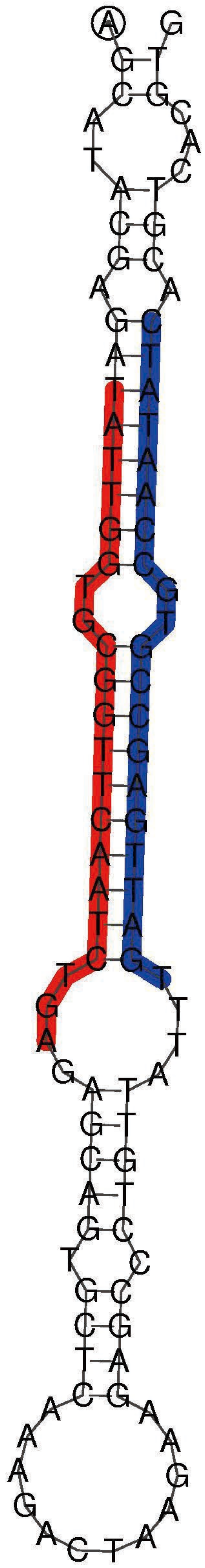

Secondary structure for csi-miRN20

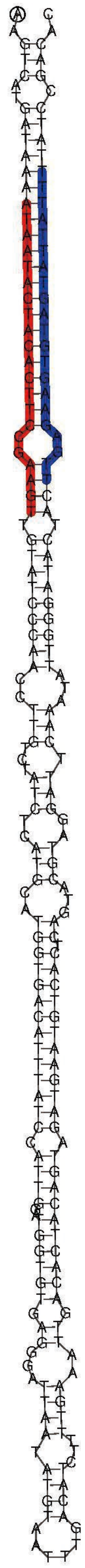

Secondary structure for csi-miRN21

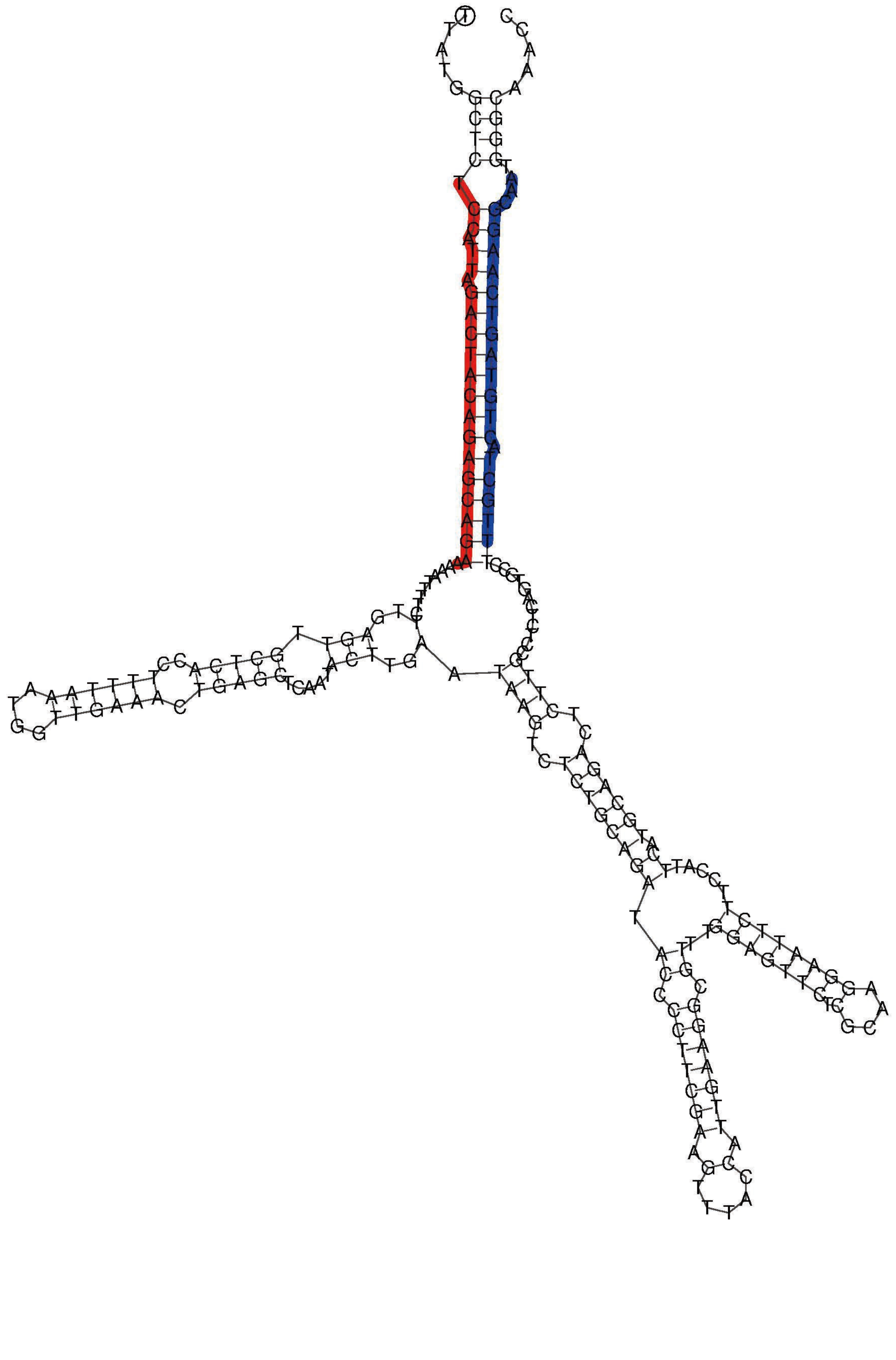

Supplement: Figure S4 — Predicted secondary structures of the novel miRNAs. The mature miRNA sequences are highlighted in blue and the miRNA* sequences are highlighted in red. [file Image4.PDF]
