# Supplementary material for: Comparative Analysis of miRNAs and Their Target Transcripts between a Spontaneous Late-Ripening Sweet Orange Mutant and Its Wild-Type Using Small RNA and Degradome Sequencing
Source: Front Plant Sci. 2016 Sep 21;7:1416. doi: 10.3389/fpls.2016.01416 (PMC5030777; doi:10.3389/fpls.2016.01416)
Supplement: Figure S5 — T-plots of the miRNA targets. [file Image5.PDF]

Cs3g23180.1 gene=Cs3g23180 CDS=151-1557

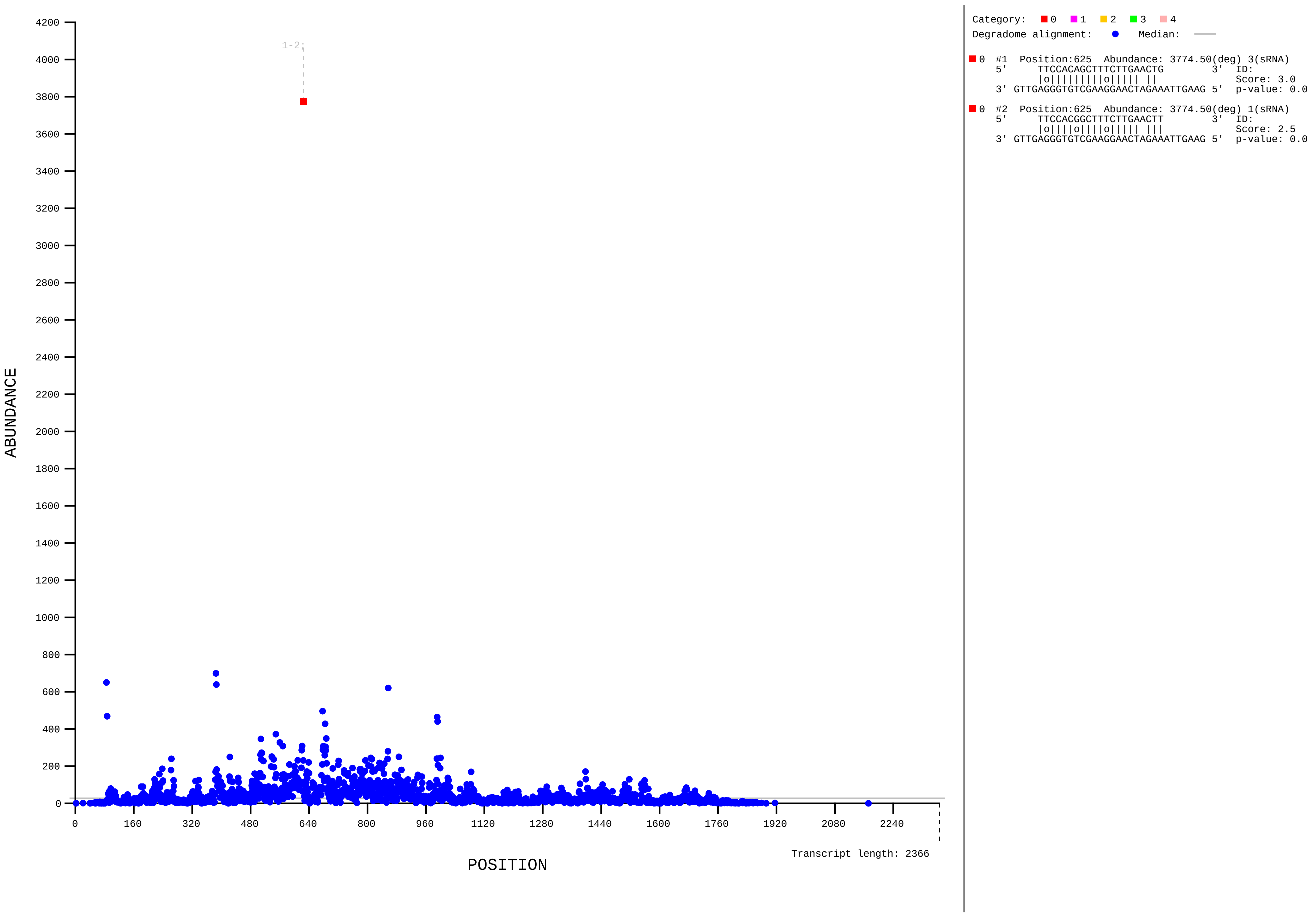

Cs3g23180.2 gene=Cs3g23180 CDS=151-1503

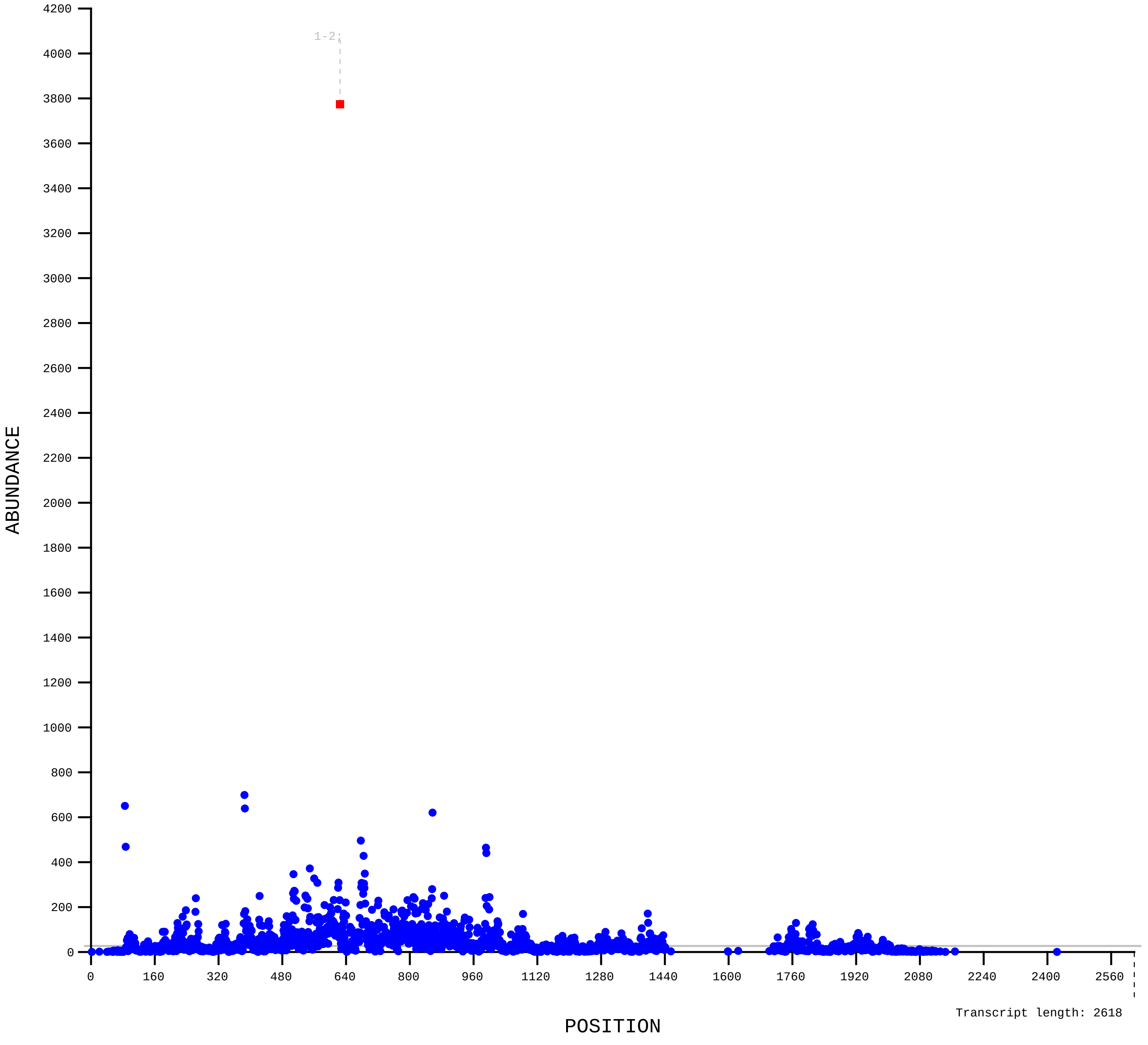


```
Category:  ■0  ■1  ■2  ■3  ■4
Degradome alignment:  ●   Median: ——

■0  #1  Position:625  Abundance: 3774.50(deg) 3(sRNA)
    5'     TTCCACAGCTTTCTTGAACTG        3'  ID:
           |o|||||||||o||||| ||             Score: 3.0
    3' GTTGAGGGTGTCGAAGGAACTAGAAATTGAAG 5'  p-value: 0.0

■0  #2  Position:625  Abundance: 3774.50(deg) 1(sRNA)
    5'     TTCCACGGCTTTCTTGAACTT        3'  ID:
           |o||||o||||o||||| |||            Score: 2.5
    3' GTTGAGGGTGTCGAAGGAACTAGAAATTGAAG 5'  p-value: 0.0
```

orange1.1t05622.1 gene=orange1.1t05622 CDS=3-347

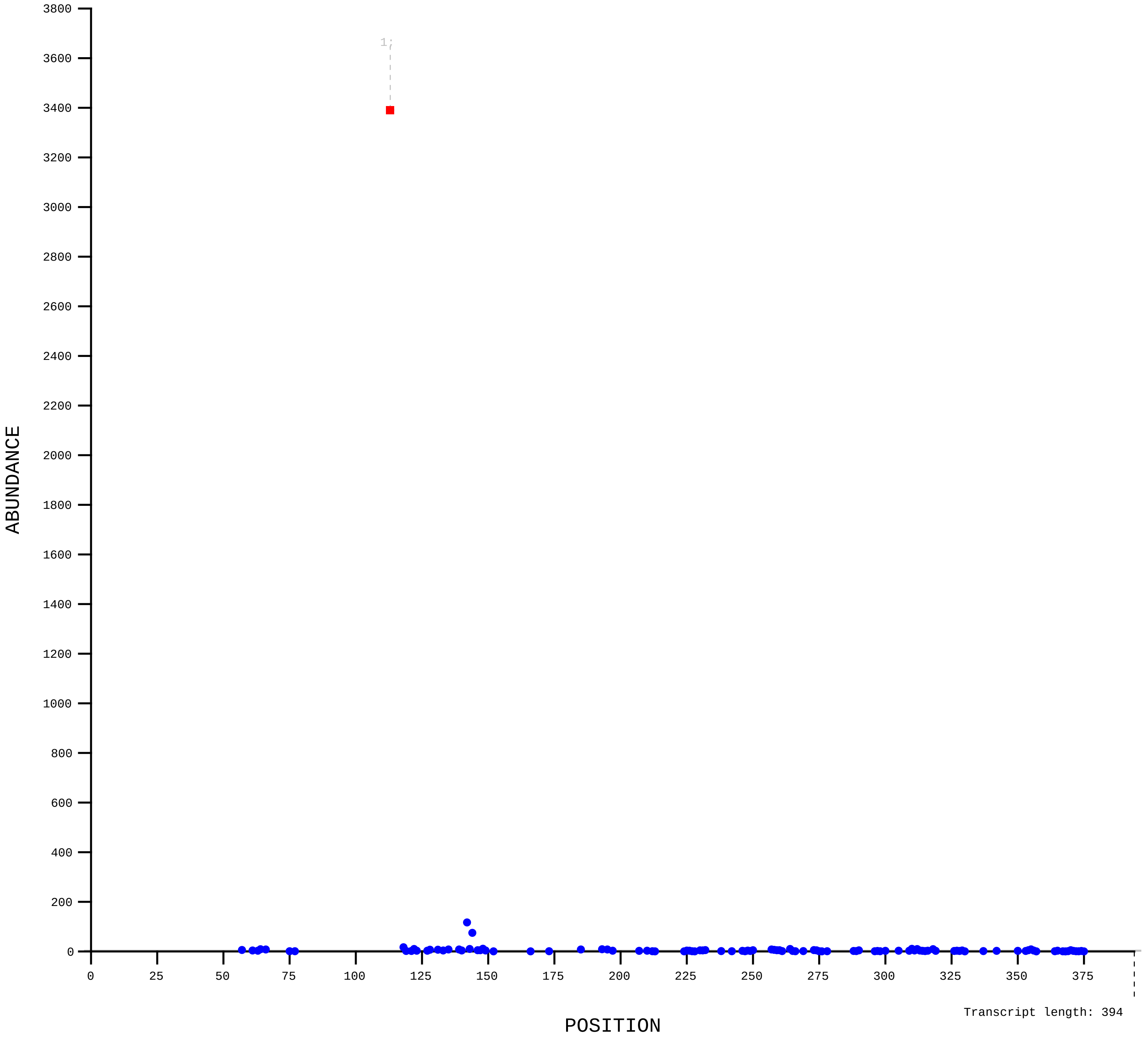


```
Category:  0  1  2  3  4
Degradome alignment:      Median:

0  #1  Position:113  Abundance: 3391.00(deg) 1(sRNA)
   5'     TAGATAAAGATGAGAGAAAAA        3'  ID:
          o|||||||||| ||||||||||           Score: 1.5
   3' GGGTGTCTATTTCT-CTCTCTTTTTTTTTCTC 5'  p-value: 0.0
```

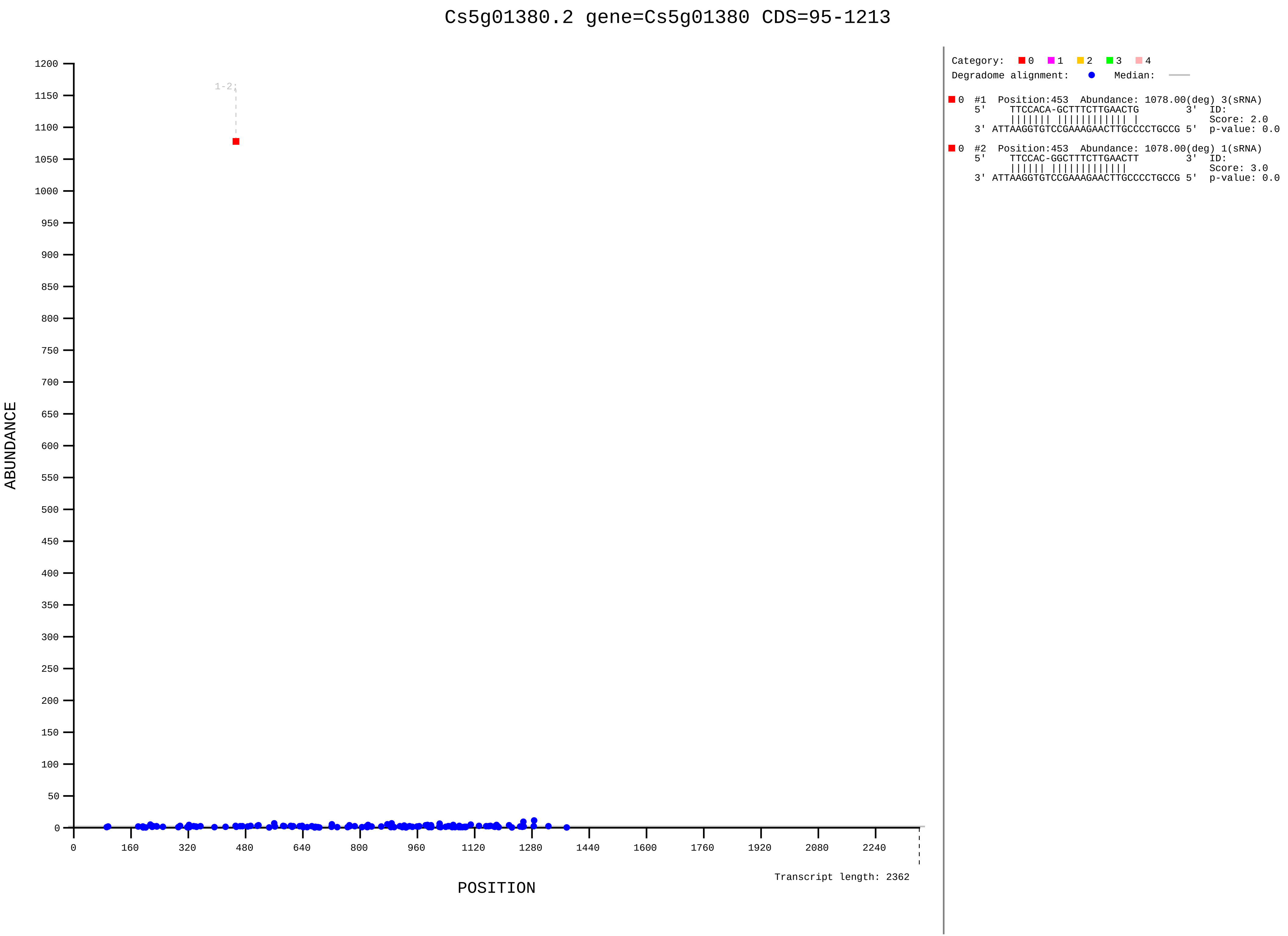
Cs5g01380.2 gene=Cs5g01380 CDS=95-1213
ABUNDANCE
POSITION
Transcript length: 2362
1-2
Category: 0 1 2 3 4
Degradome alignment:
Median:
0 #1 Position:453 Abundance: 1078.00(deg) 3(sRNA)
5' TTCCACA-GCTTTCTTGAACTG 3' ID:
Score: 2.0
3' ATTAAGGTGTCCGAAAGAACTTGCCCCTGCCG 5' p-value: 0.0
0 #2 Position:453 Abundance: 1078.00(deg) 1(sRNA)
5' TTCCAC-GGCTTTCTTGAACTT 3' ID:
Score: 3.0
3' ATTAAGGTGTCCGAAAGAACTTGCCCCTGCCG 5' p-value: 0.0

Cs5g01380.1 gene=Cs5g01380 CDS=451-1941

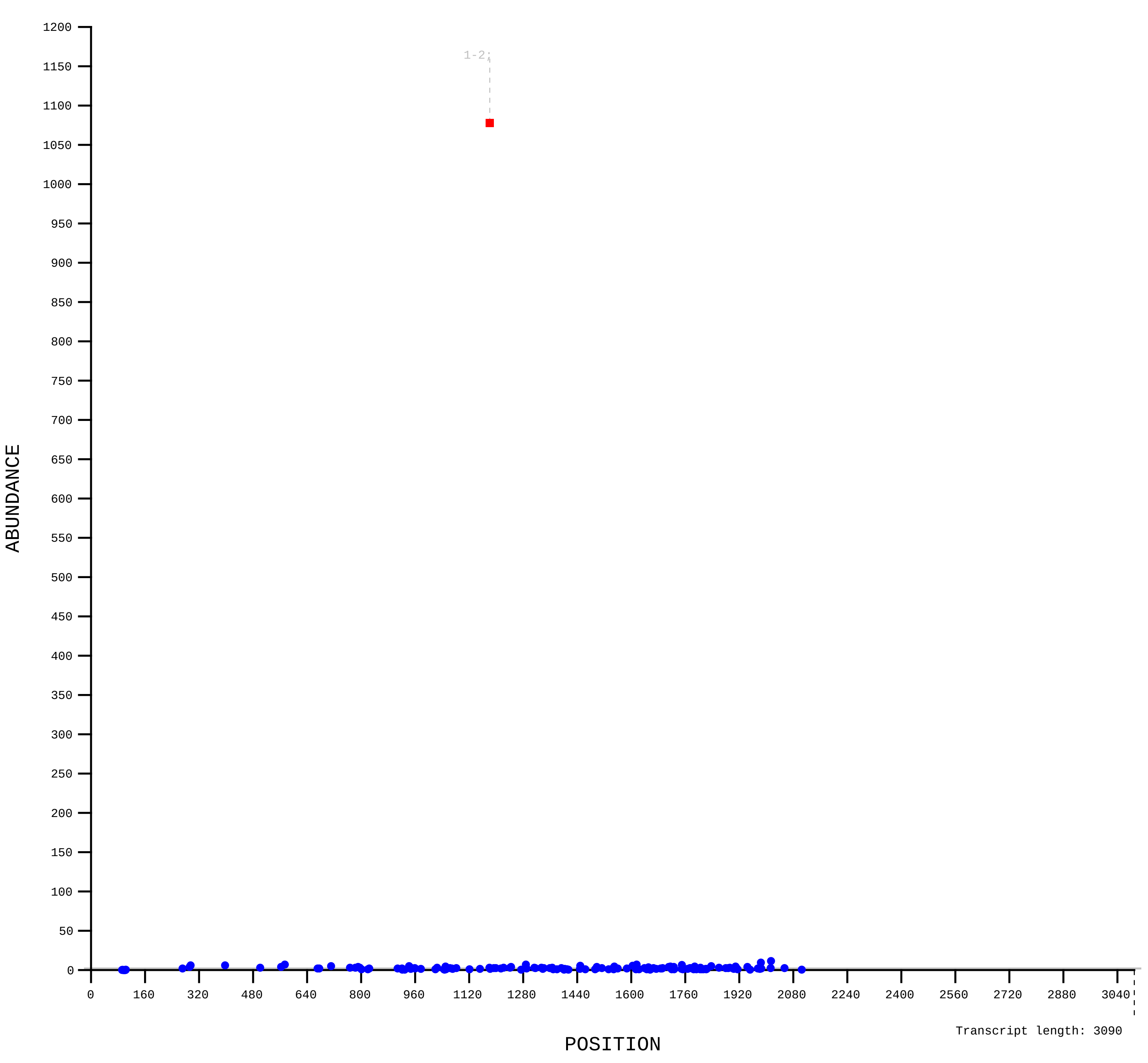


```
Category: 0 1 2 3 4
Degradome alignment:     Median:

0  #1  Position:1181  Abundance: 1078.00(deg) 3(sRNA)
   5'    TTCCACA-GCTTTCTTGAACTG        3'  ID:
         ||||||| |||||||||||| |            Score: 2.0
   3' ATTAAGGTGTCCGAAAGAACTTGCCCCTGCCG 5'  p-value: 0.0

0  #2  Position:1181  Abundance: 1078.00(deg) 1(sRNA)
   5'    TTCCAC-GGCTTTCTTGAACTT        3'  ID:
         |||||| |||||||||||||              Score: 3.0
   3' ATTAAGGTGTCCGAAAGAACTTGCCCCTGCCG 5'  p-value: 0.0
```

Cs7g26730.1 gene=Cs7g26730 CDS=194-3598

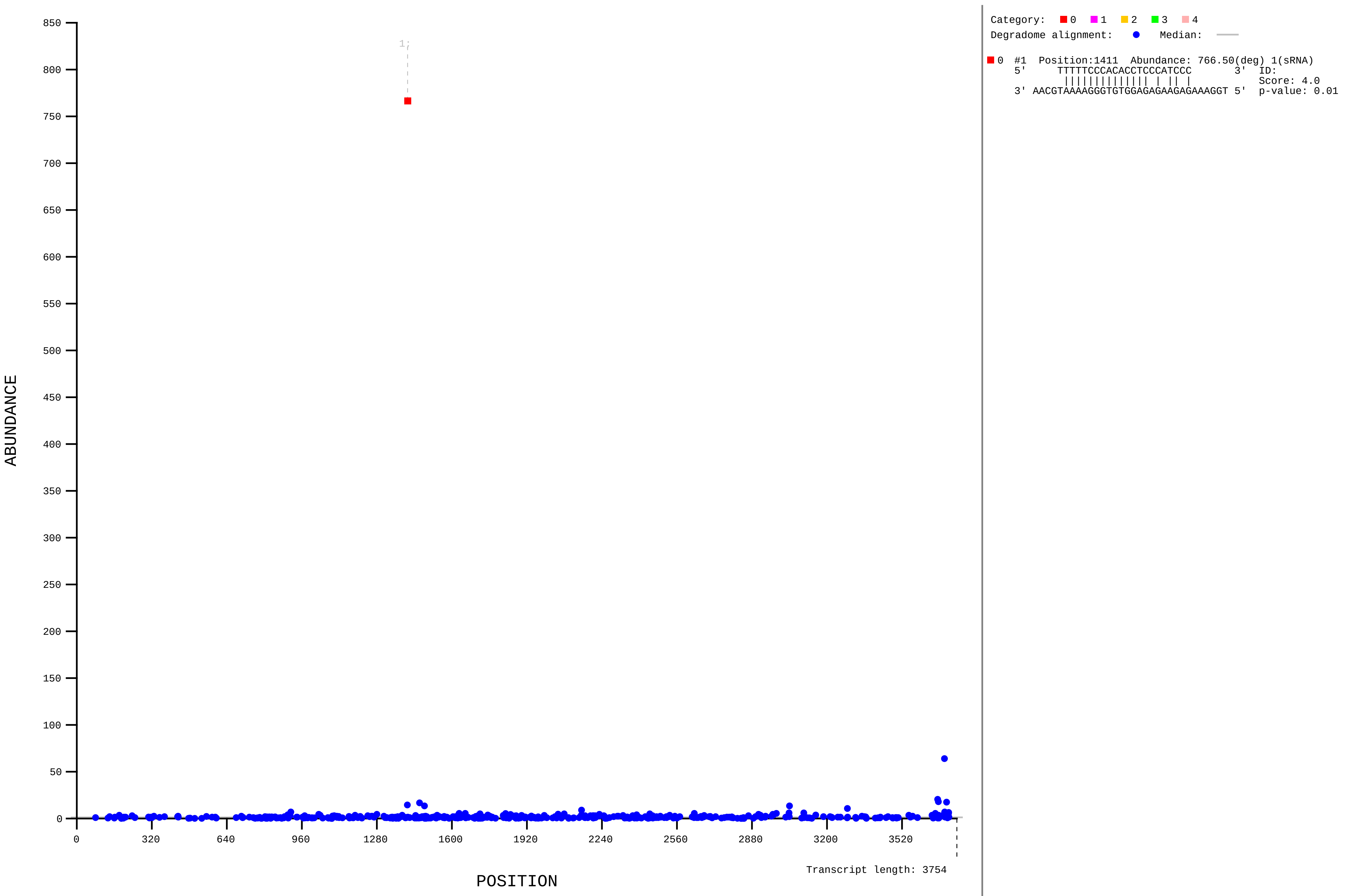

Cs7g26730.2 gene=Cs7g26730 CDS=1038-3842

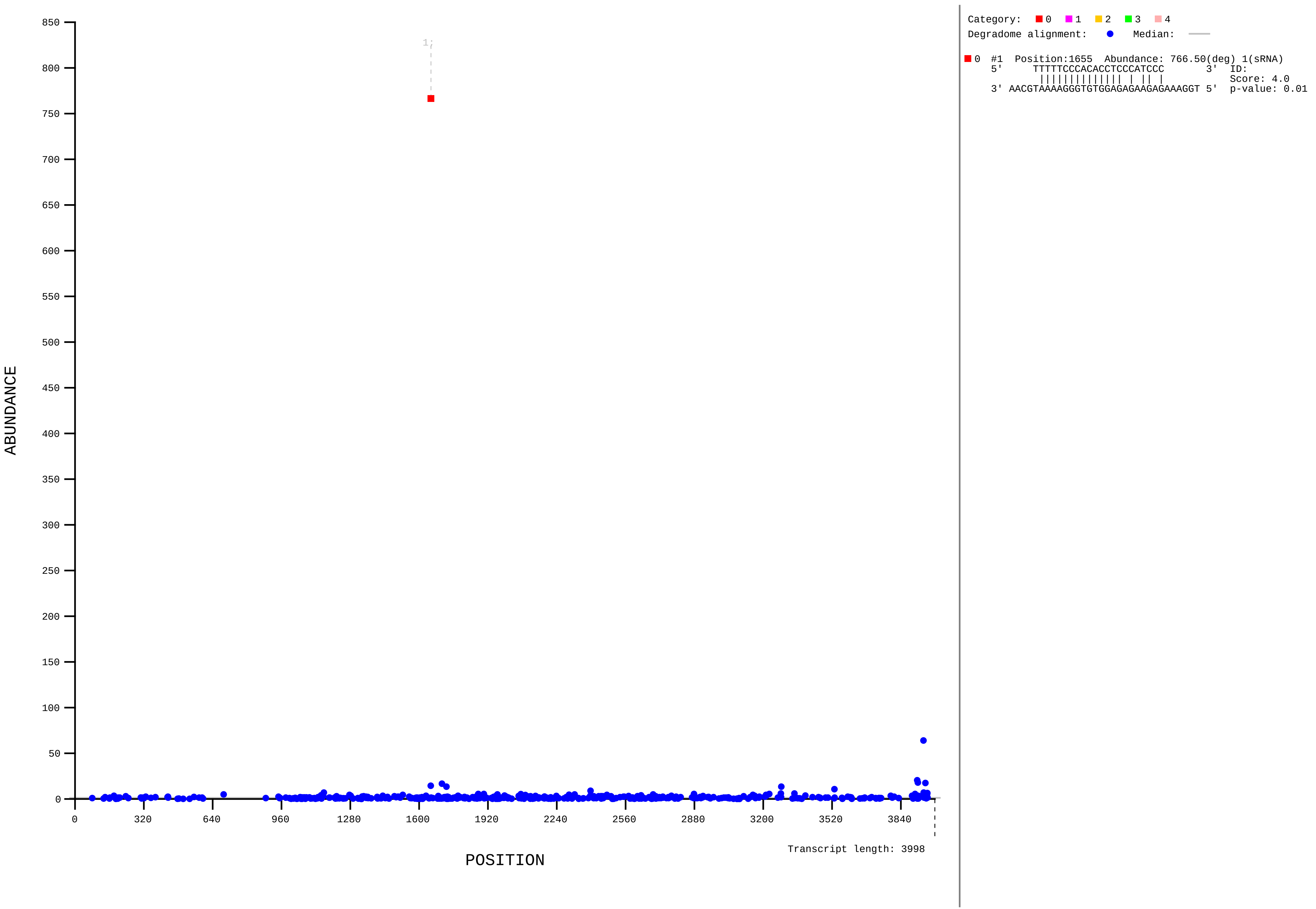

Cs8g13560.1 gene=Cs8g13560 CDS=28-702

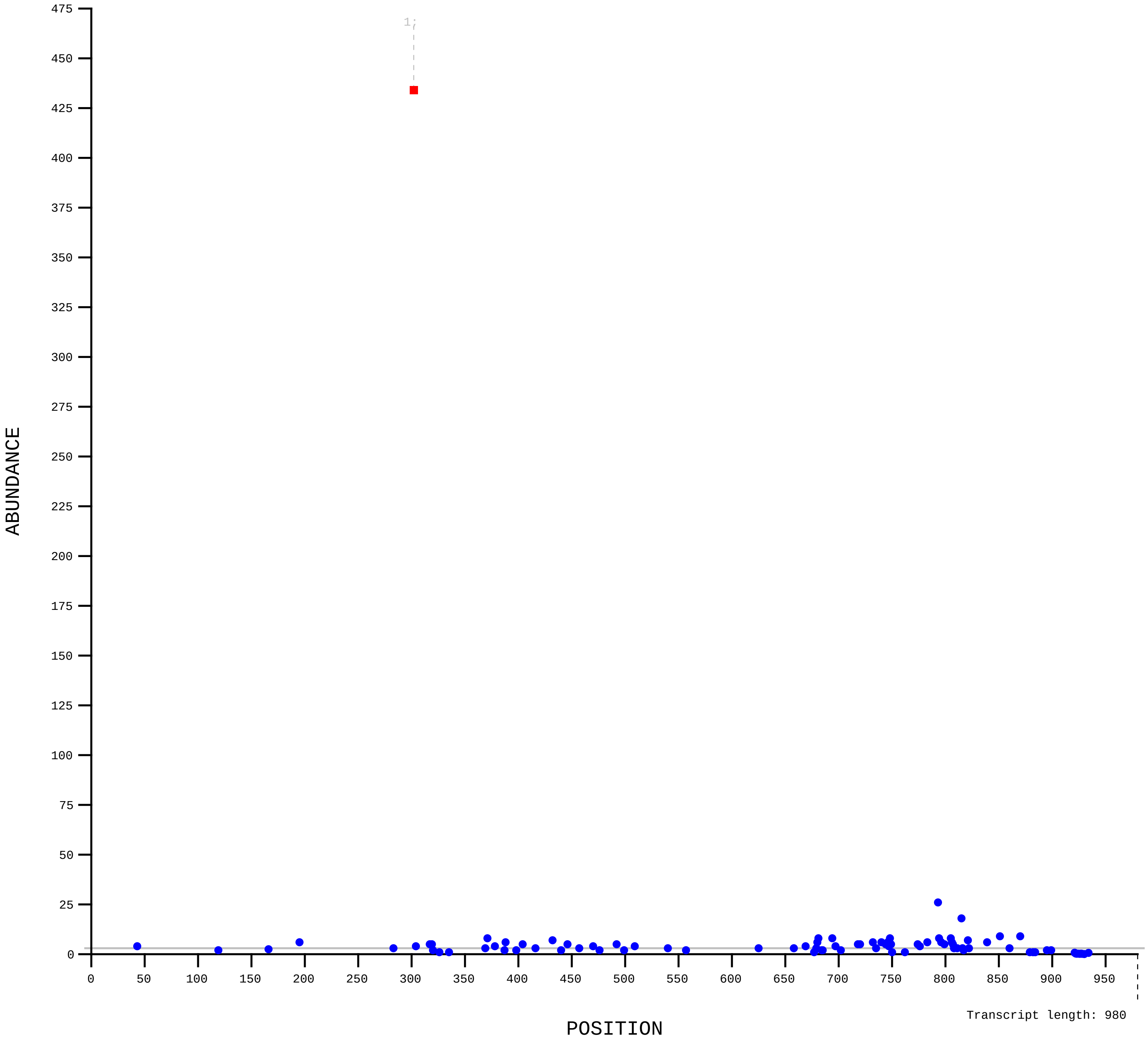

Category: 0 1 2 3 4
Degradome alignment: Median:

```
0  #1  Position:302  Abundance: 434.00(deg) 1(sRNA)
   5'     TCGCAGGAGCTTTCTACGGTT        3'  ID:
          ||||||||||||| ||||||             Score: 2.0
   3' GTACAGCGTCCTCGAAAAATGCCATGATGTAC 5'  p-value: 0.0
```

Cs2g10760.1 gene=Cs2g10760 CDS=227-3172

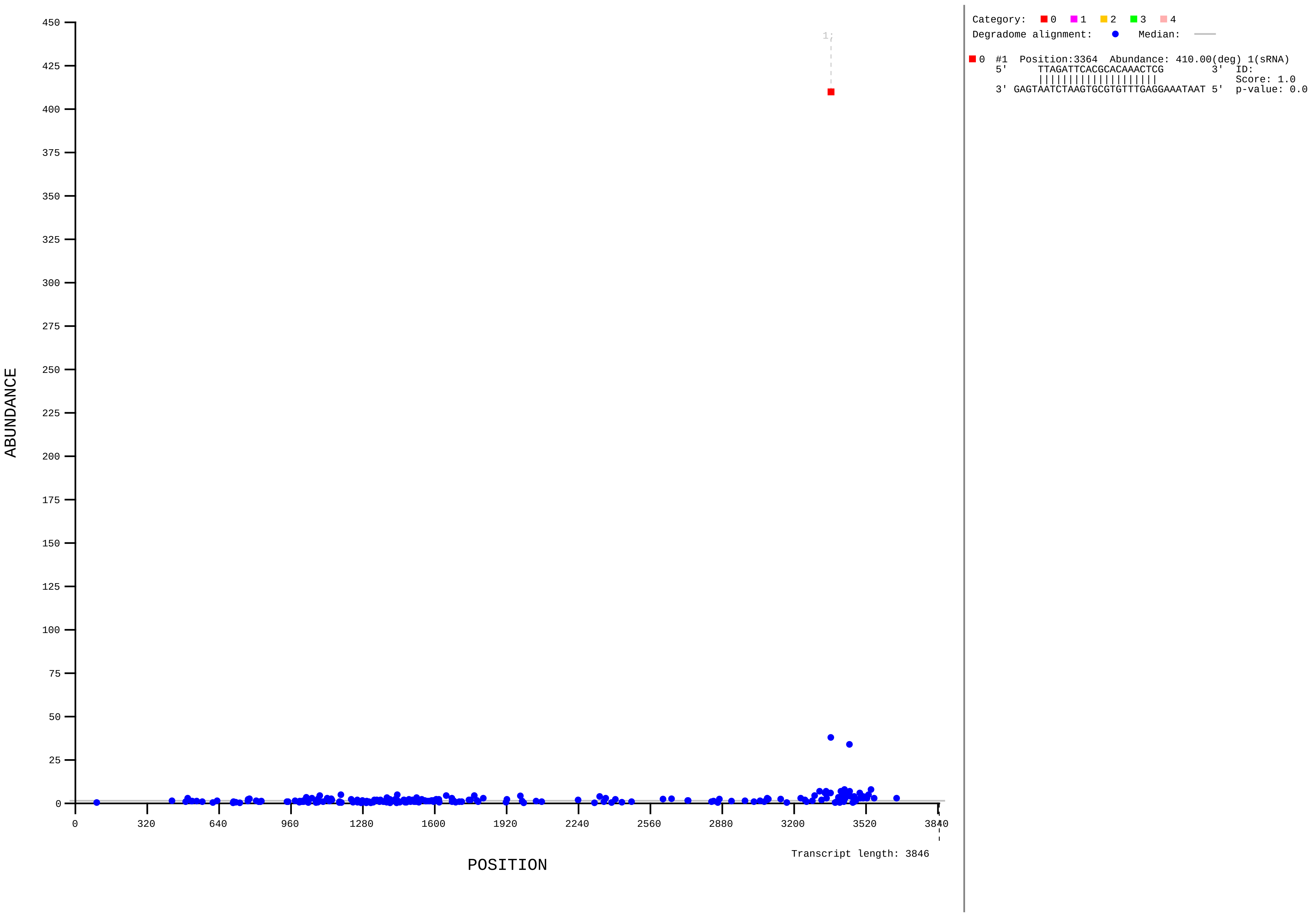

orange1.1t04055.1 gene=orange1.1t04055 CDS=576-2135

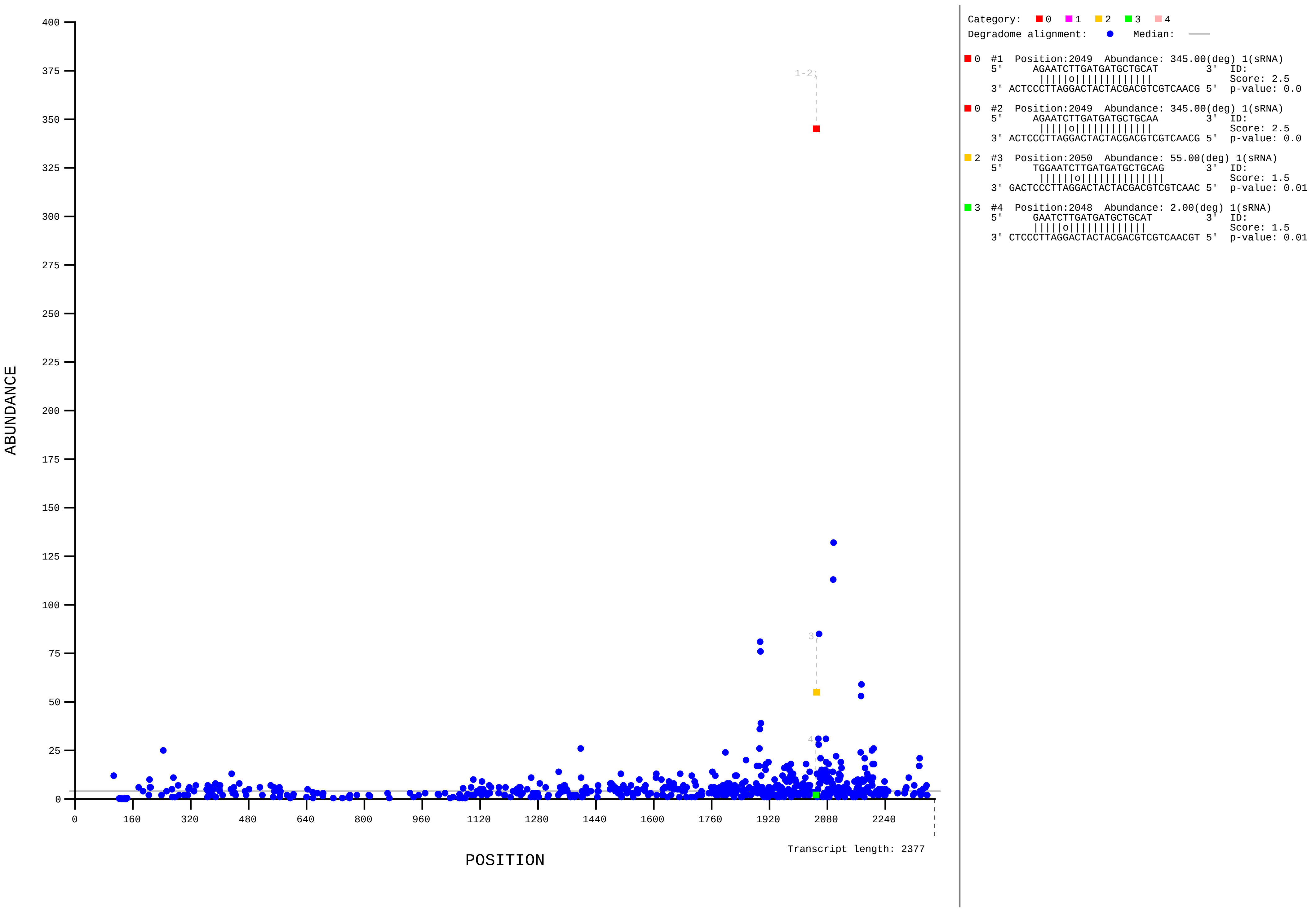

Cs1g06060.1 gene=Cs1g06060 CDS=1-387

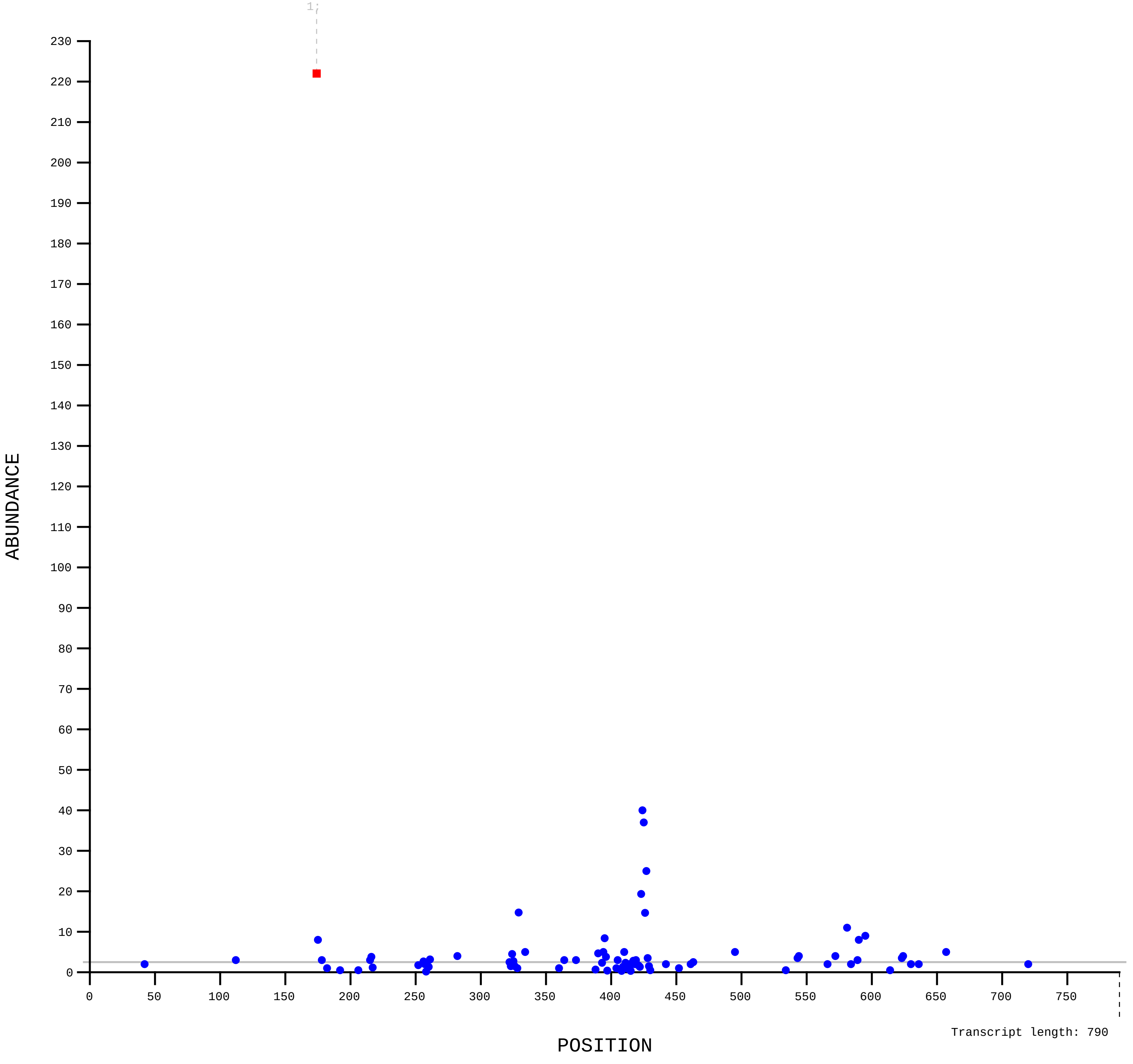


```
Category: 0 1 2 3 4
Degradome alignment:     Median:

0  #1  Position:174  Abundance: 222.00(deg) 1(sRNA)
   5'     TAGATAAAGATGAGAGAAAAA        3'  ID:
          o||||||||| ||||||||||            Score: 1.5
   3' GGGTGTCTATTTCT-CTCTCTTTTTTTTTTCCC 5'  p-value: 0.0
```

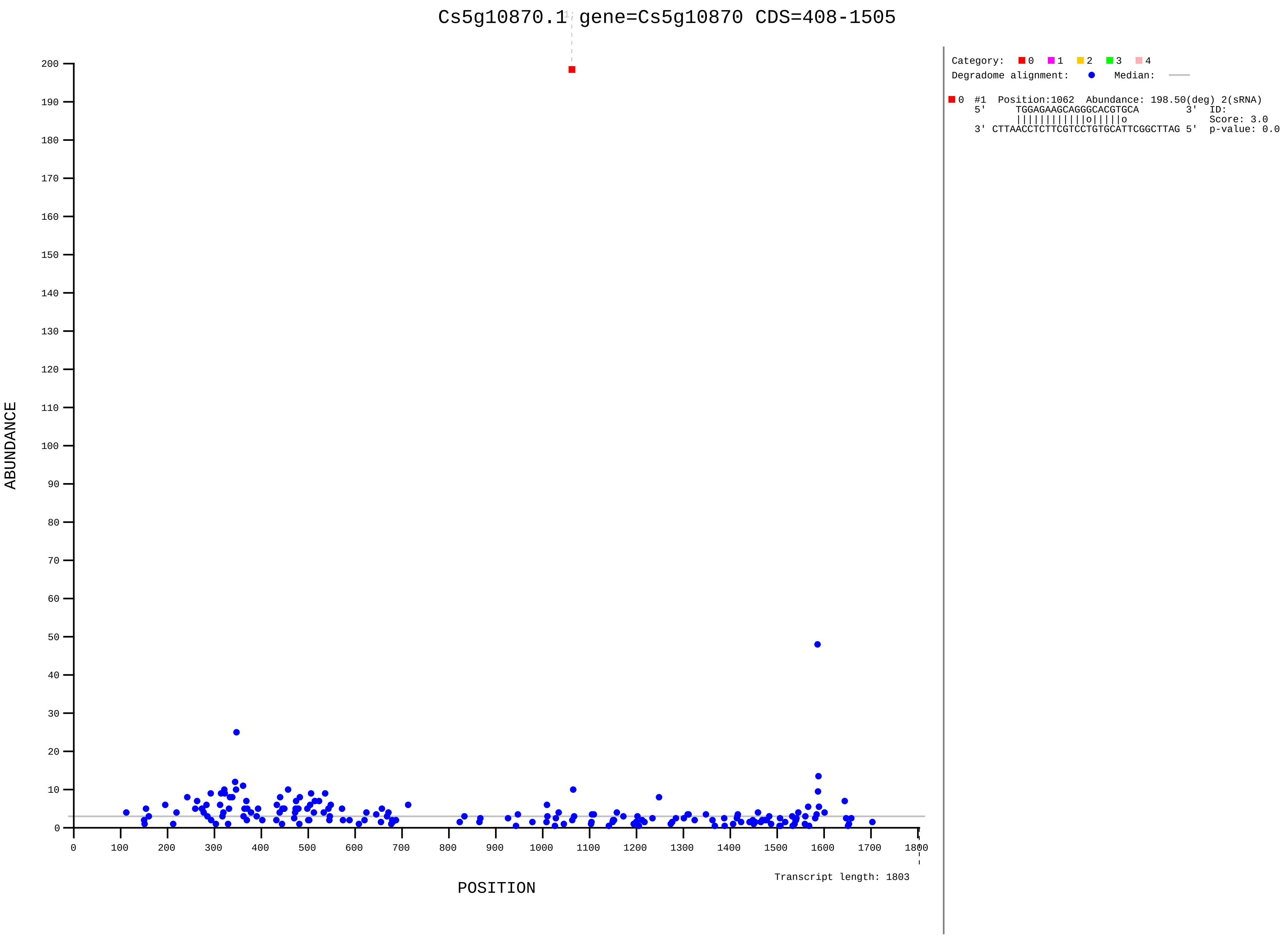

Cs5g10870.1 gene=Cs5g10870 CDS=408-1505
ABUNDANCE
POSITION
0
10
20
30
40
50
60
70
80
90
100
110
120
130
140
150
160
170
180
190
200
0
100
200
300
400
500
600
700
800
900
1000
1100
1200
1300
1400
1500
1600
1700
1800
Transcript length: 1803
Category: 0 1 2 3 4
Degradome alignment: Median:
0 #1 Position:1062 Abundance: 198.50(deg) 2(sRNA)
5' TGGAGAAGCAGGGCACGTGCA 3' ID:
||||||||||||o|||||o Score: 3.0
3' CTTAACCTCTTCGTCCTGTGCATTCGGCTTAG 5' p-value: 0.0

Cs5g10870.2 gene=Cs5g10870 CDS=88-987

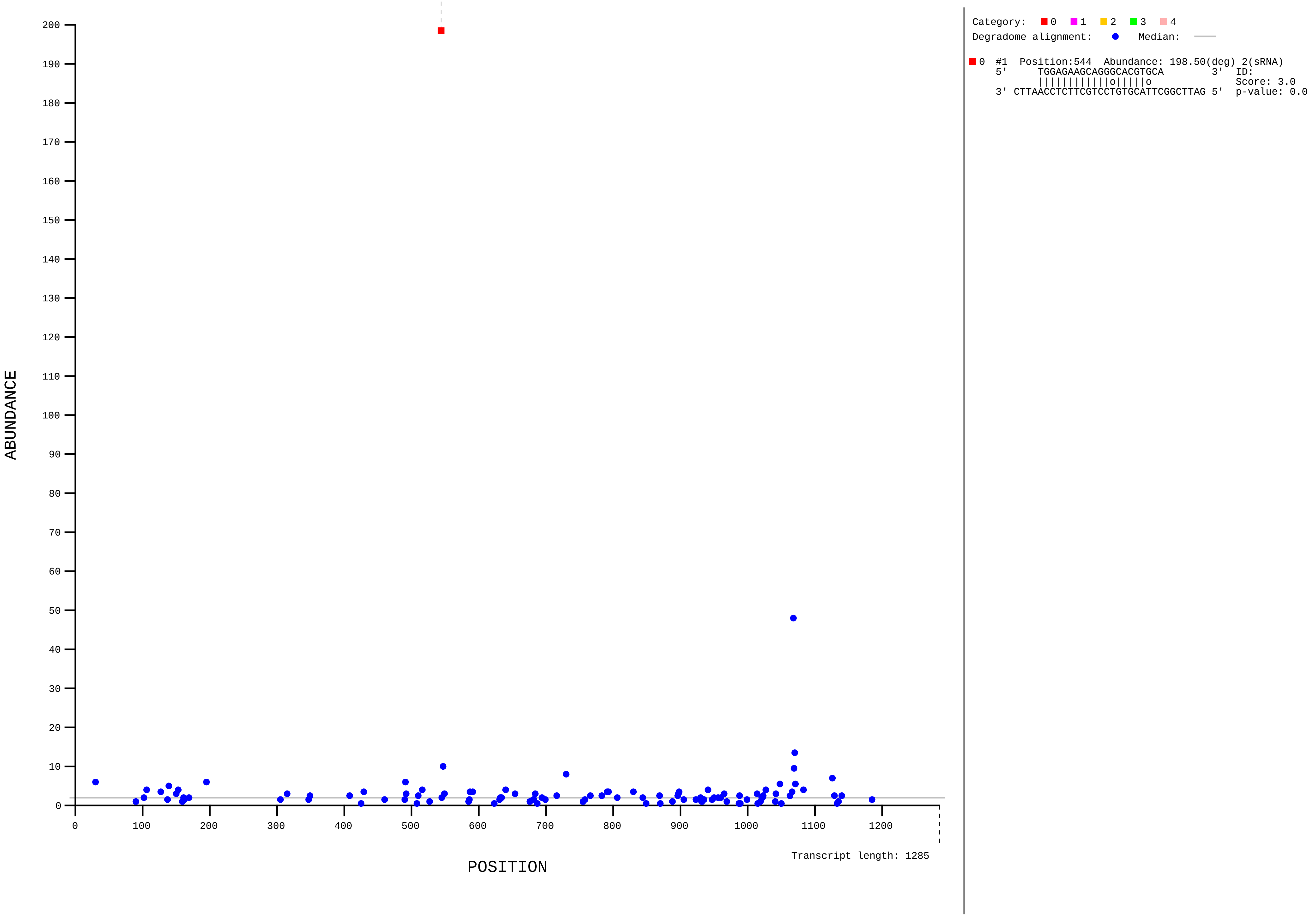

Cs5g19570.1 gene=Cs5g19570 CDS=1483-3201

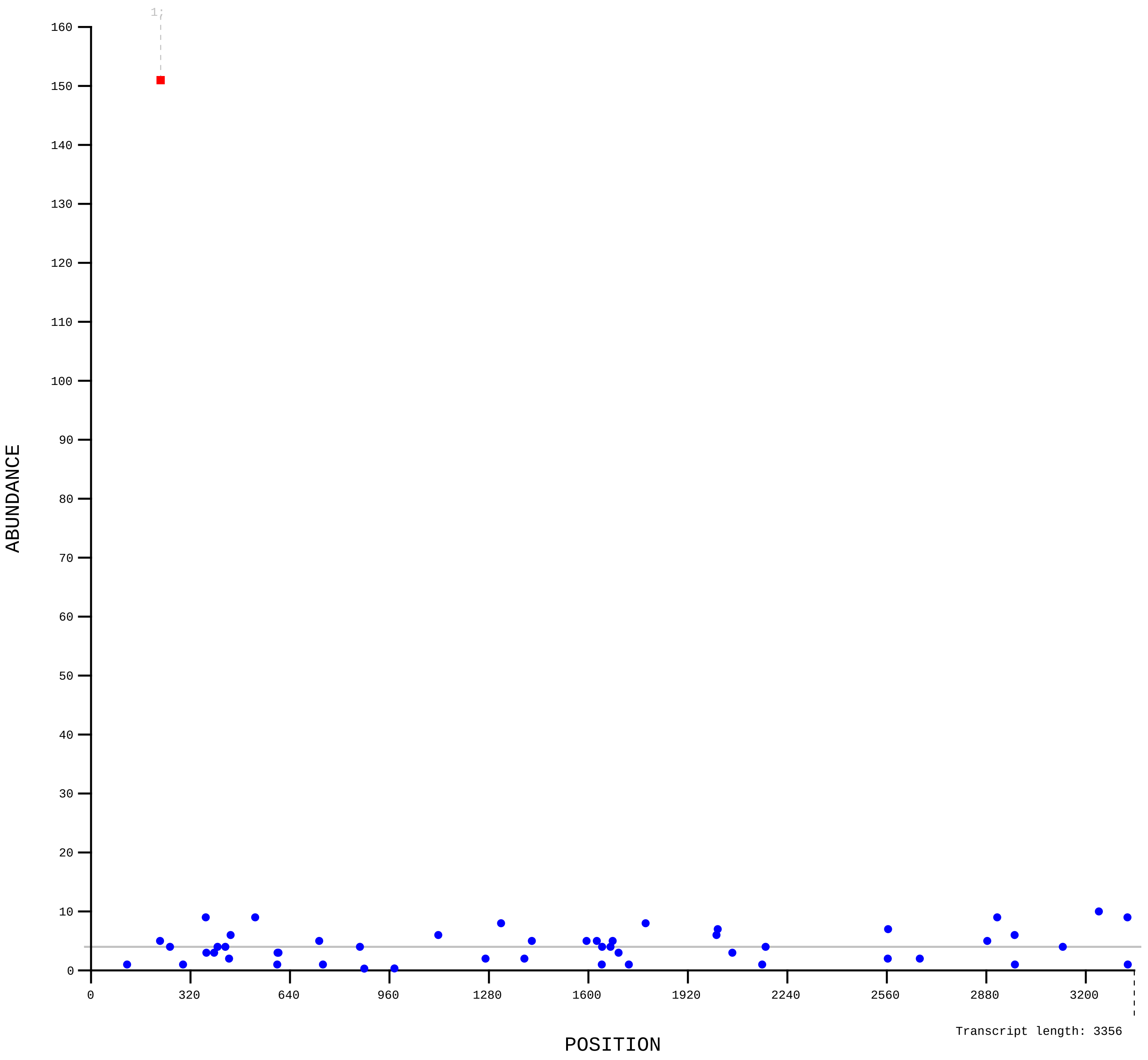


```
Category: ■0 ■1 ■2 ■3 ■4
Degradome alignment: ●   Median: ——

■0  #1  Position:224  Abundance: 151.00(deg) 1(sRNA)
    5'     TCTTCCCTATGCCTCCCATTCC       3'  ID:
           |||| |||||o|| || |||||           Score: 3.5
    3' CAACAGAAAGGATATGGTGGCTAAGGGGTTTA 5'  p-value: 0.0
```

Cs5g21975.1 gene=Cs5g21975 CDS=82-3237

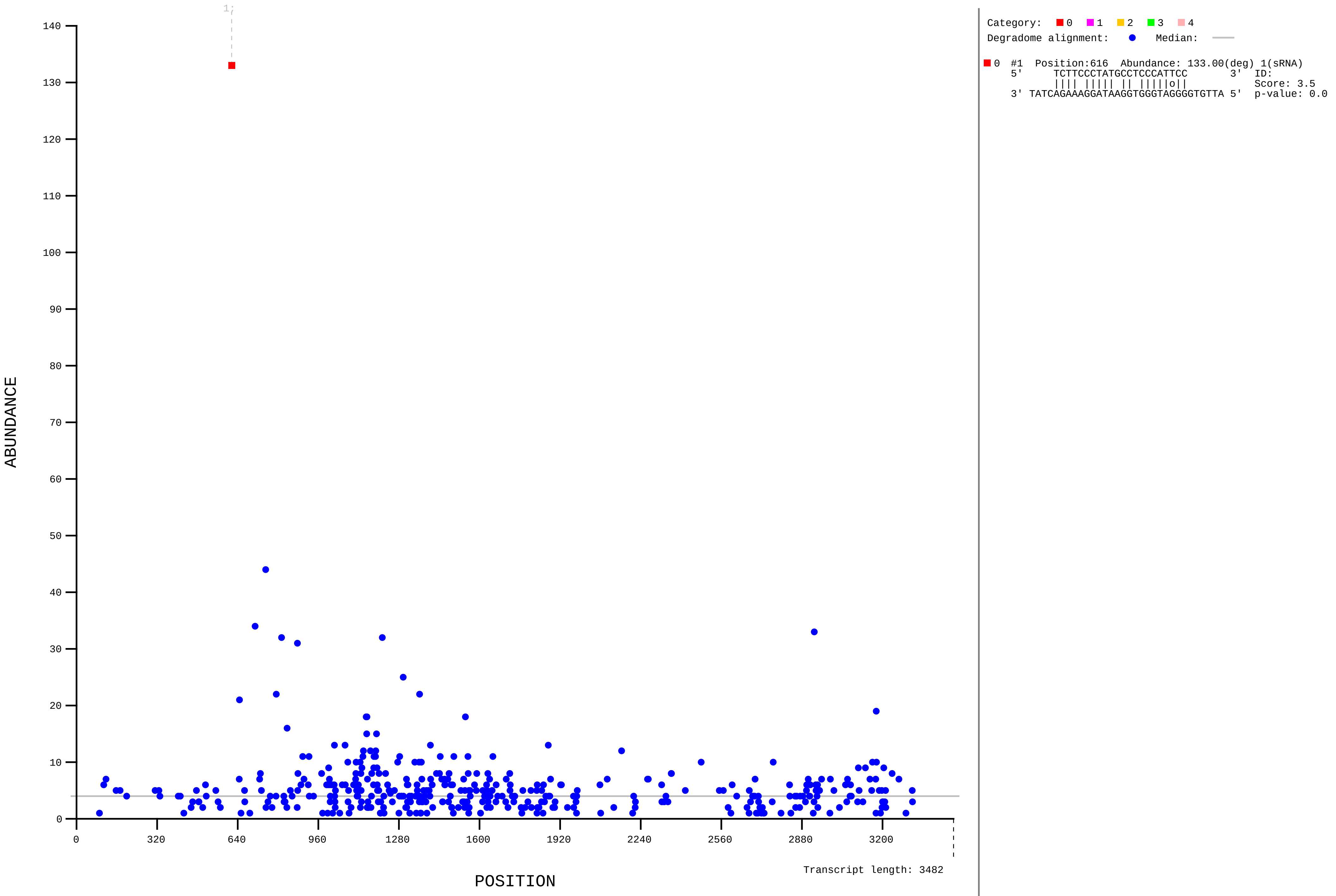

Cs2g10770.1 gene=Cs2g10770 CDS=192-3137

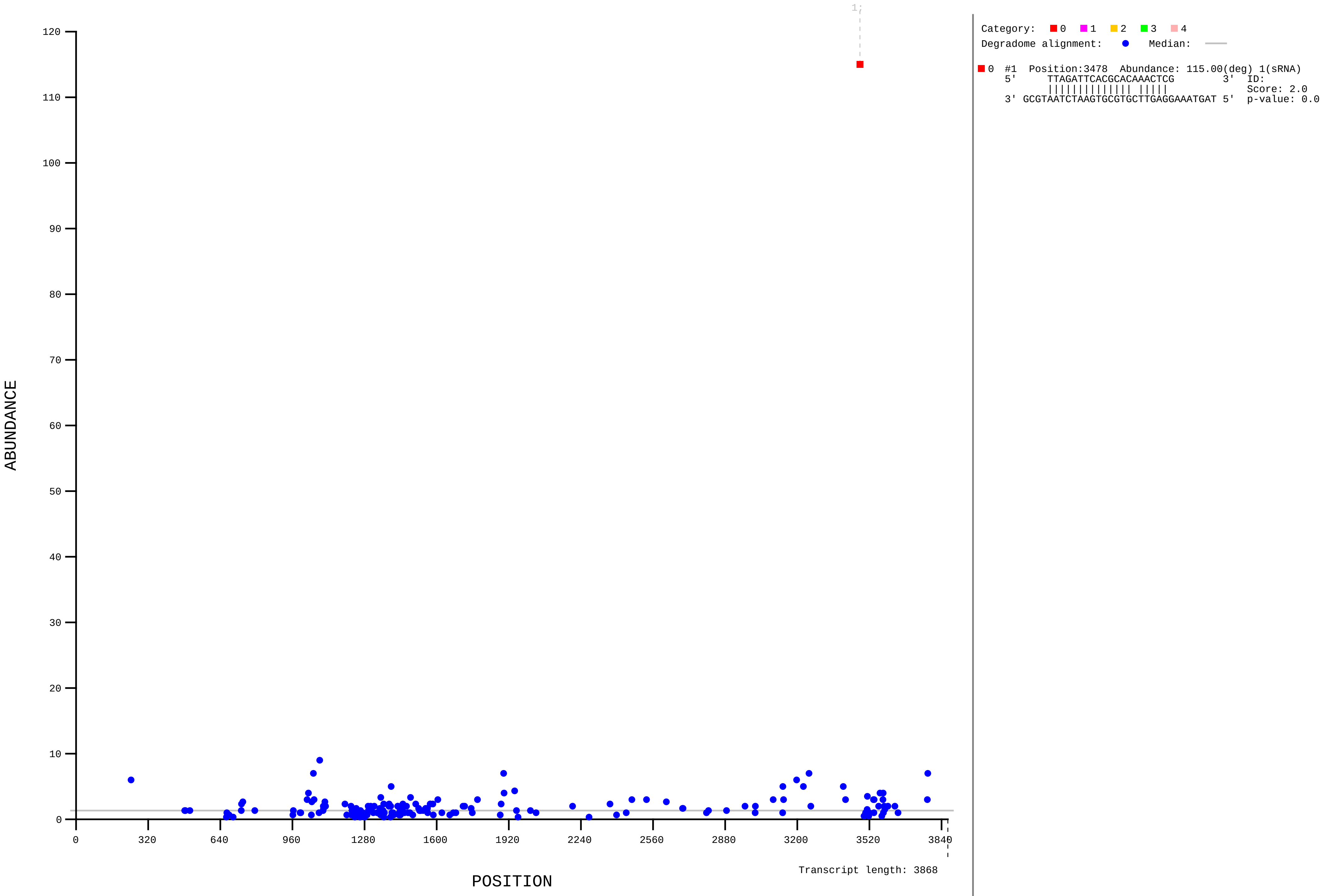

Cs5g04670.2 gene=Cs5g04670 CDS=439-753

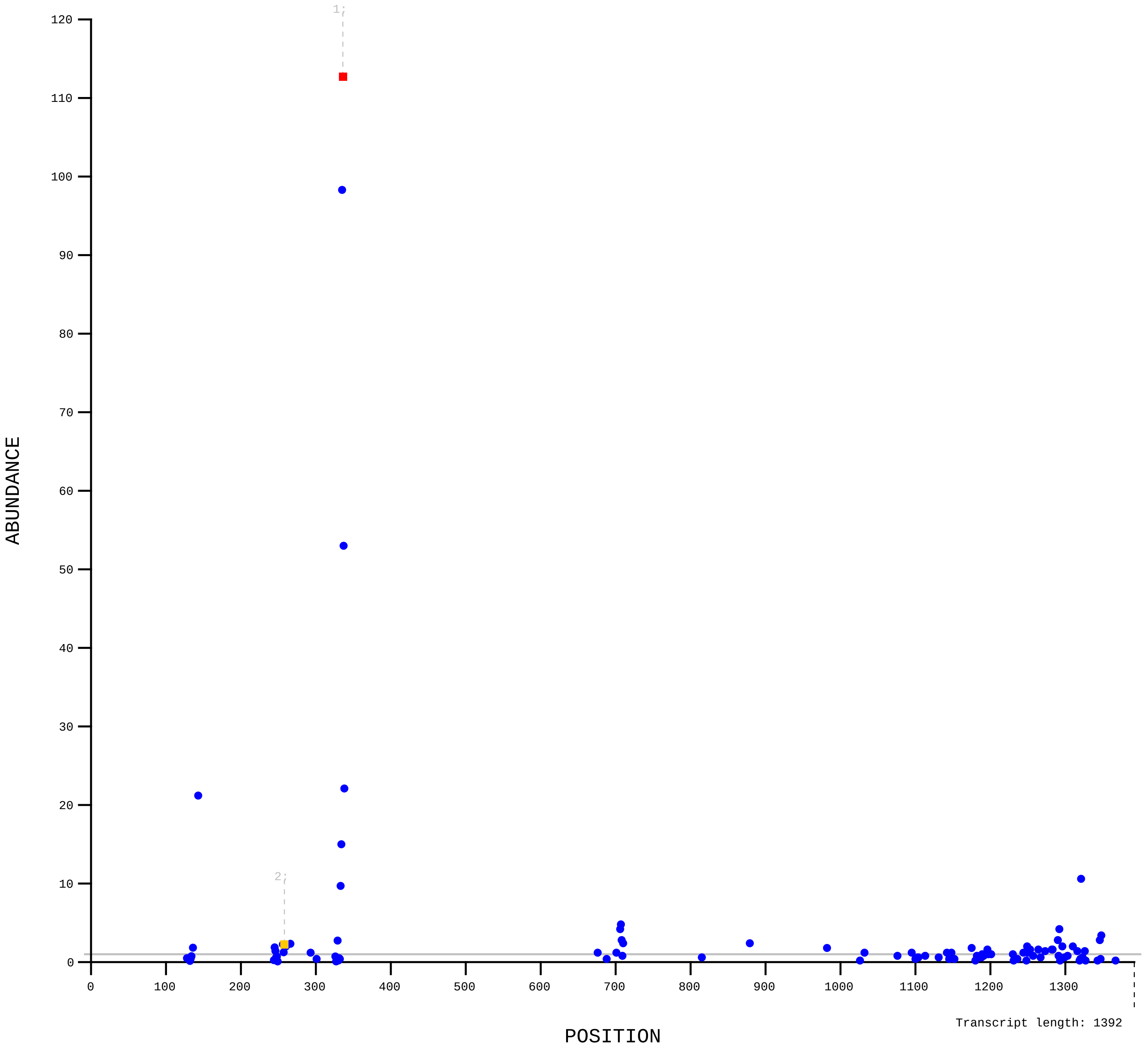

Category: 0 1 2 3 4
Degradome alignment: Median:

```
0  #1  Position:336  Abundance: 112.70(deg) 1(sRNA)
   5'     TTTTTCGGCAACATGATTTCT        3'  ID:
           ||||| |||||||||||||o            Score: 2.5
   3' GTCATAAAAGACGTTGTACTAAAGGTGTTAGT 5'  p-value: 0.01

2  #2  Position:258  Abundance: 2.25(deg) 1(sRNA)
   5'     TTTTTCGGCAACATGATTTCT        3'  ID:
           ||||| ||||||||||||||            Score: 2.0
   3' TCGATAAAAGACGTTGTACTAAAGACTTCAAT 5'  p-value: 0.02
```

Cs5g04670.5 gene=Cs5g04670 CDS=1020-1334

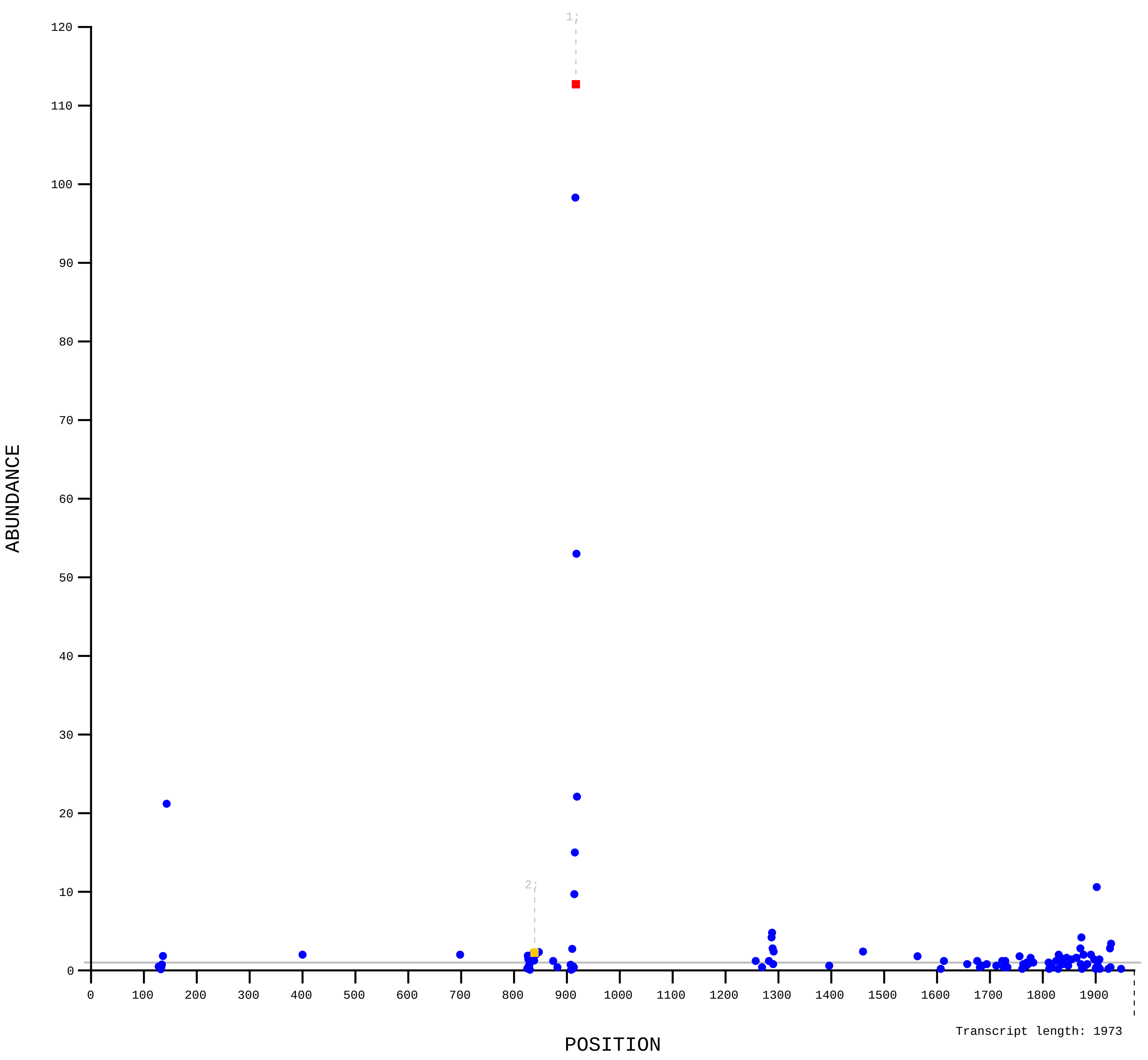


```
0  #1  Position:917  Abundance: 112.70(deg) 1(sRNA)
   5'     TTTTTCGGCAACATGATTTCT        3'  ID:
            ||||| |||||||||||||o           Score: 2.5
   3' GTCATAAAAGACGTTGTACTAAAGGTGTTAGT 5'  p-value: 0.01

2  #2  Position:839  Abundance: 2.25(deg) 1(sRNA)
   5'     TTTTTCGGCAACATGATTTCT        3'  ID:
            ||||| ||||||||||||||           Score: 2.0
   3' TCGATAAAAGACGTTGTACTAAAGACTTCAAT 5'  p-value: 0.01
```

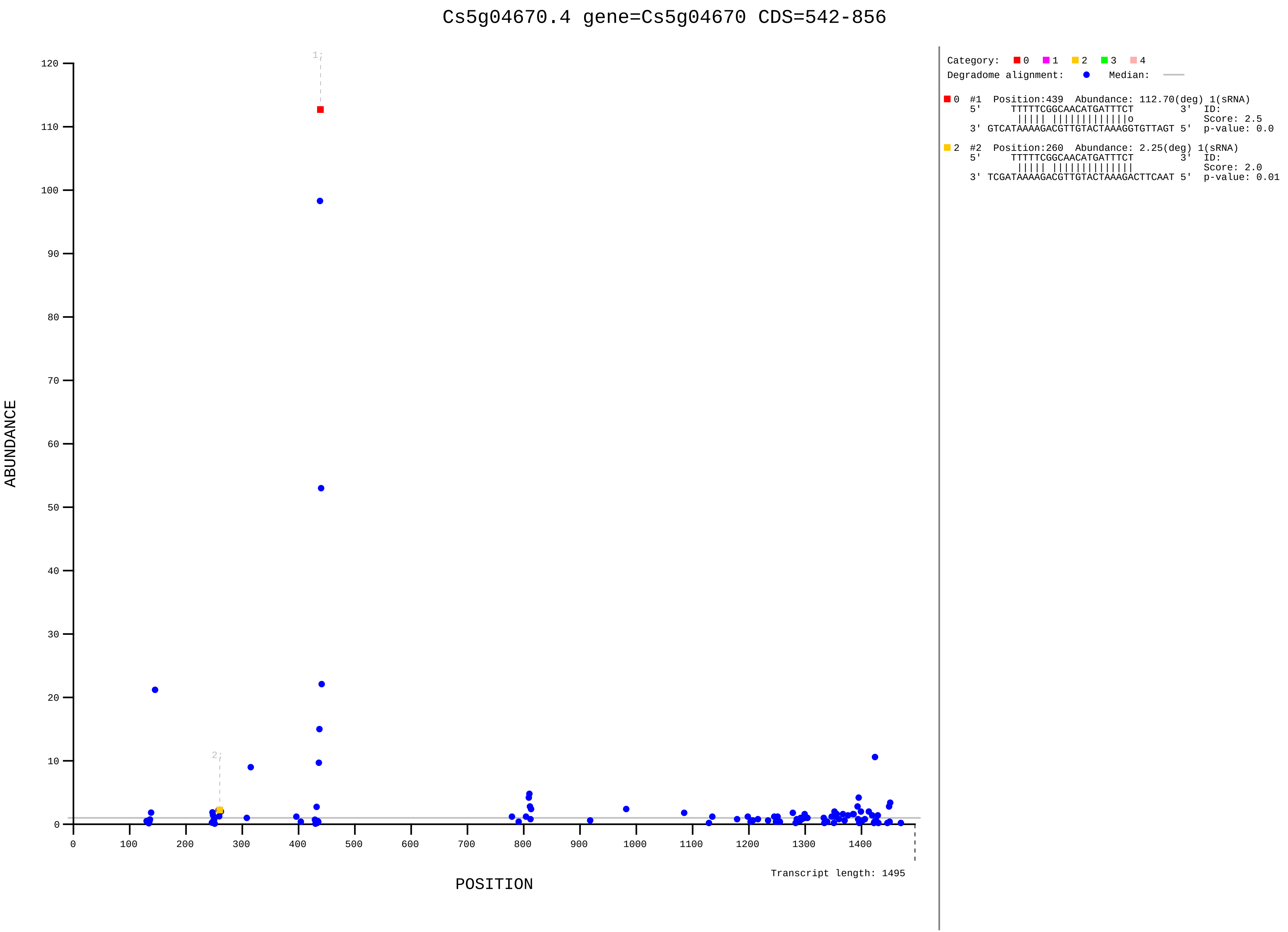

Cs5g04670.4 gene=Cs5g04670 CDS=542-856
ABUNDANCE
POSITION
Transcript length: 1495
Category: 0 1 2 3 4
Degradome alignment: Median:
0 #1 Position:439 Abundance: 112.70(deg) 1(sRNA)
5' TTTTTCGGCAACATGATTTCT 3' ID:
||||| |||||||||||||o Score: 2.5
3' GTCATAAAAGACGTTGTACTAAAGGTGTTAGT 5' p-value: 0.0
2 #2 Position:260 Abundance: 2.25(deg) 1(sRNA)
5' TTTTTCGGCAACATGATTTCT 3' ID:
||||| |||||||||||||| Score: 2.0
3' TCGATAAAAGACGTTGTACTAAAGACTTCAAT 5' p-value: 0.01

Cs5g04670.3 gene=Cs5g04670 CDS=360-674

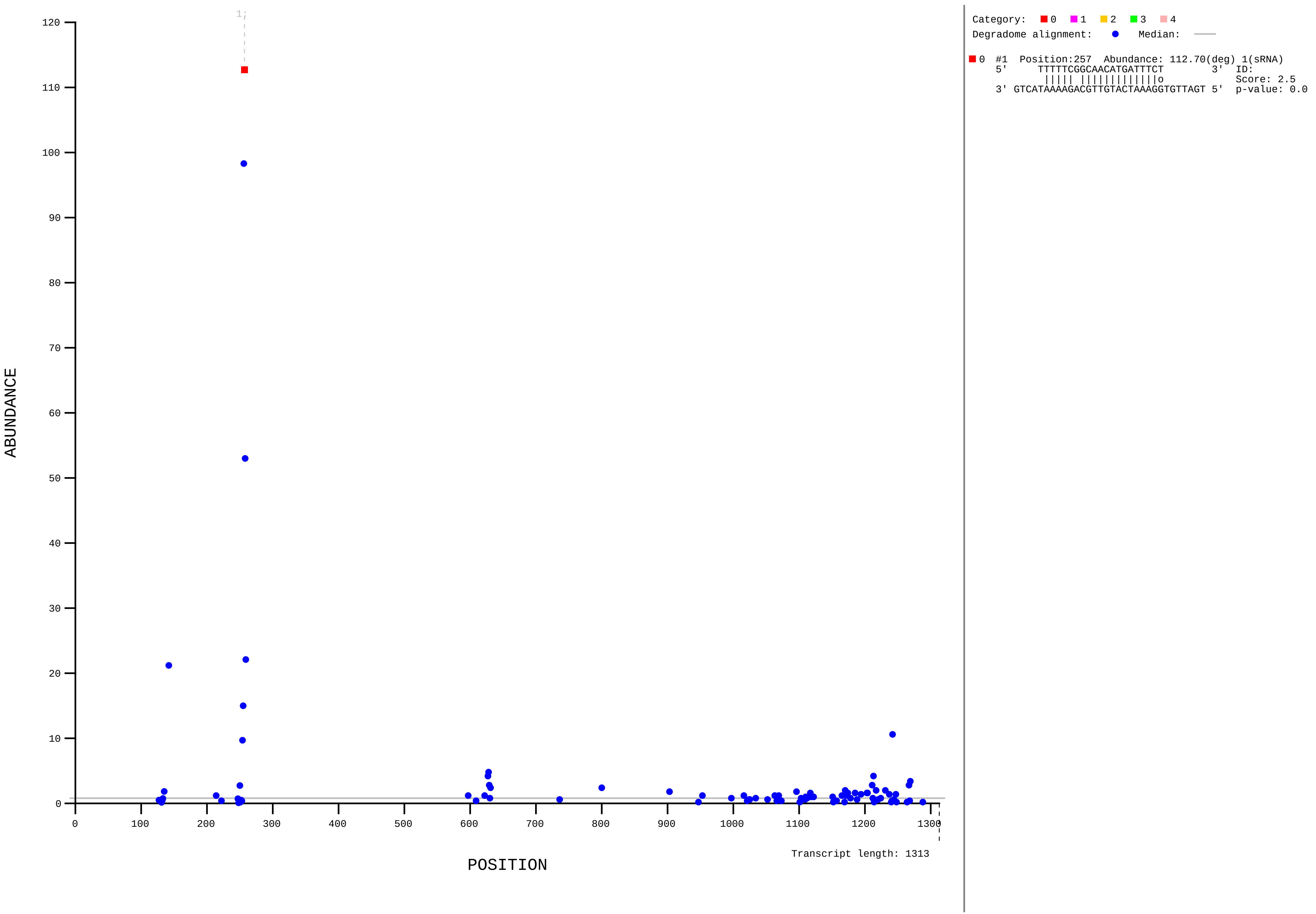

Cs5g04670.1 gene=Cs5g04670 CDS=231-641

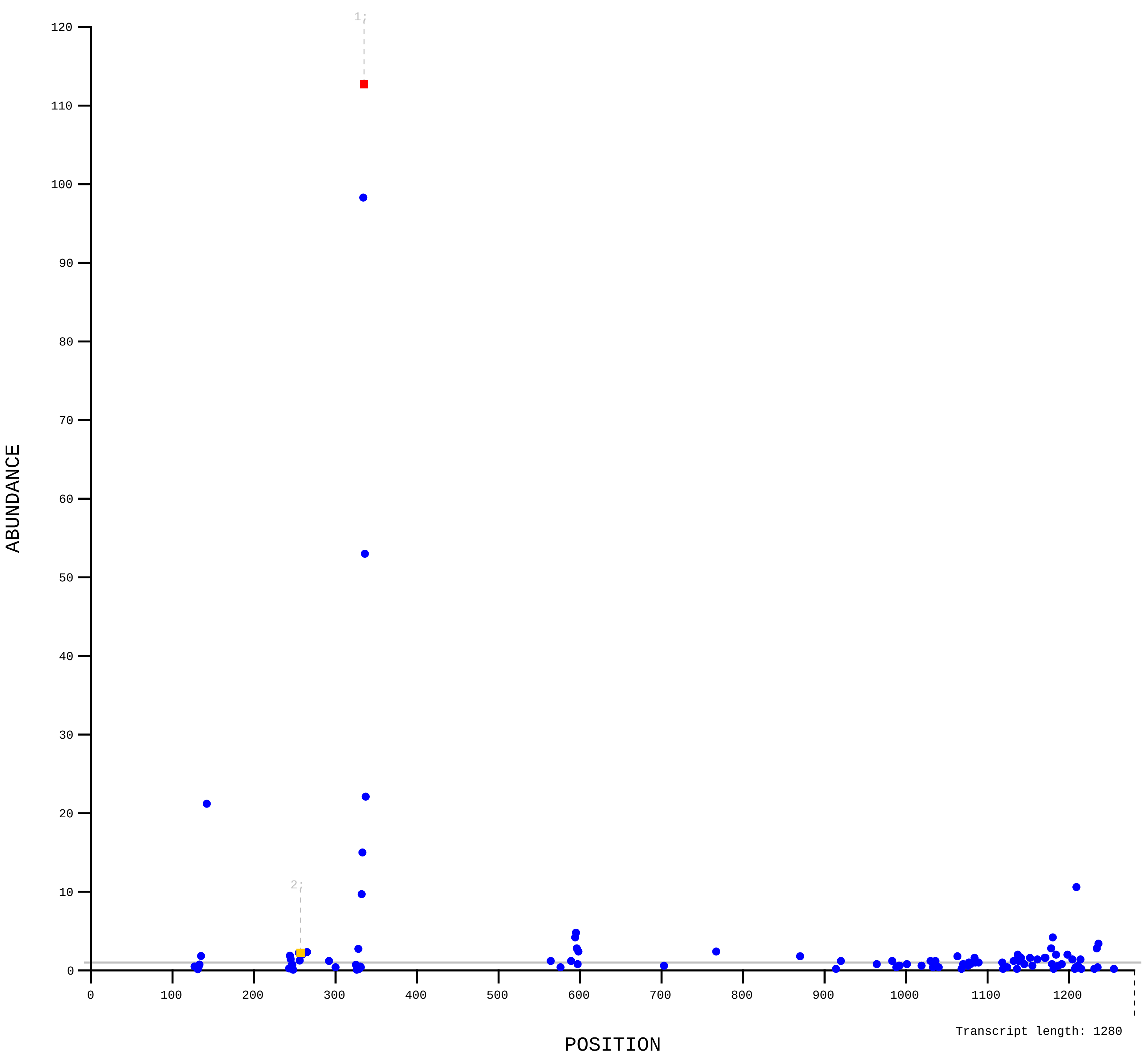

Category: 0 1 2 3 4
Degradome alignment: Median:

0 #1 Position:335 Abundance: 112.70(deg) 1(sRNA)
```
5'     TTTTTCGGCAACATGATTTCT        3'  ID:
         ||||| |||||||||||||o           Score: 2.5
3' GTCATAAAAGACGTTGTACTAAAGGTGTTAGT 5'  p-value: 0.0
```

2 #2 Position:257 Abundance: 2.25(deg) 1(sRNA)
```
5'     TTTTTCGGCAACATGATTTCT        3'  ID:
         ||||| ||||||||||||||           Score: 2.0
3' TCGATAAAAGACGTTGTACTAAAGACTTCAAT 5'  p-value: 0.02
```

Cs1g09665.1 gene=Cs1g09665 CDS=467-473

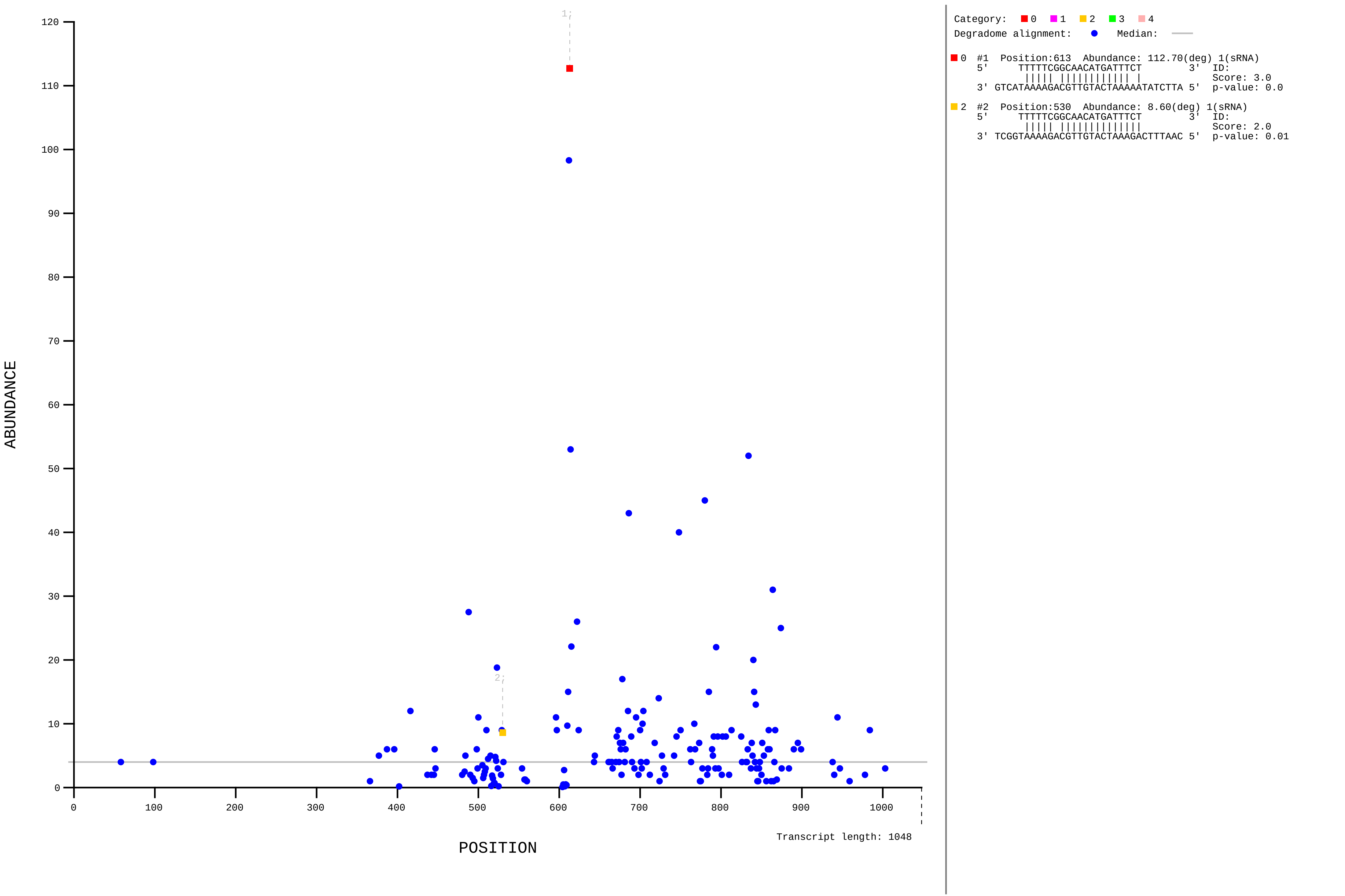

Cs1g19425.1 gene=Cs1g19425 CDS=329-340

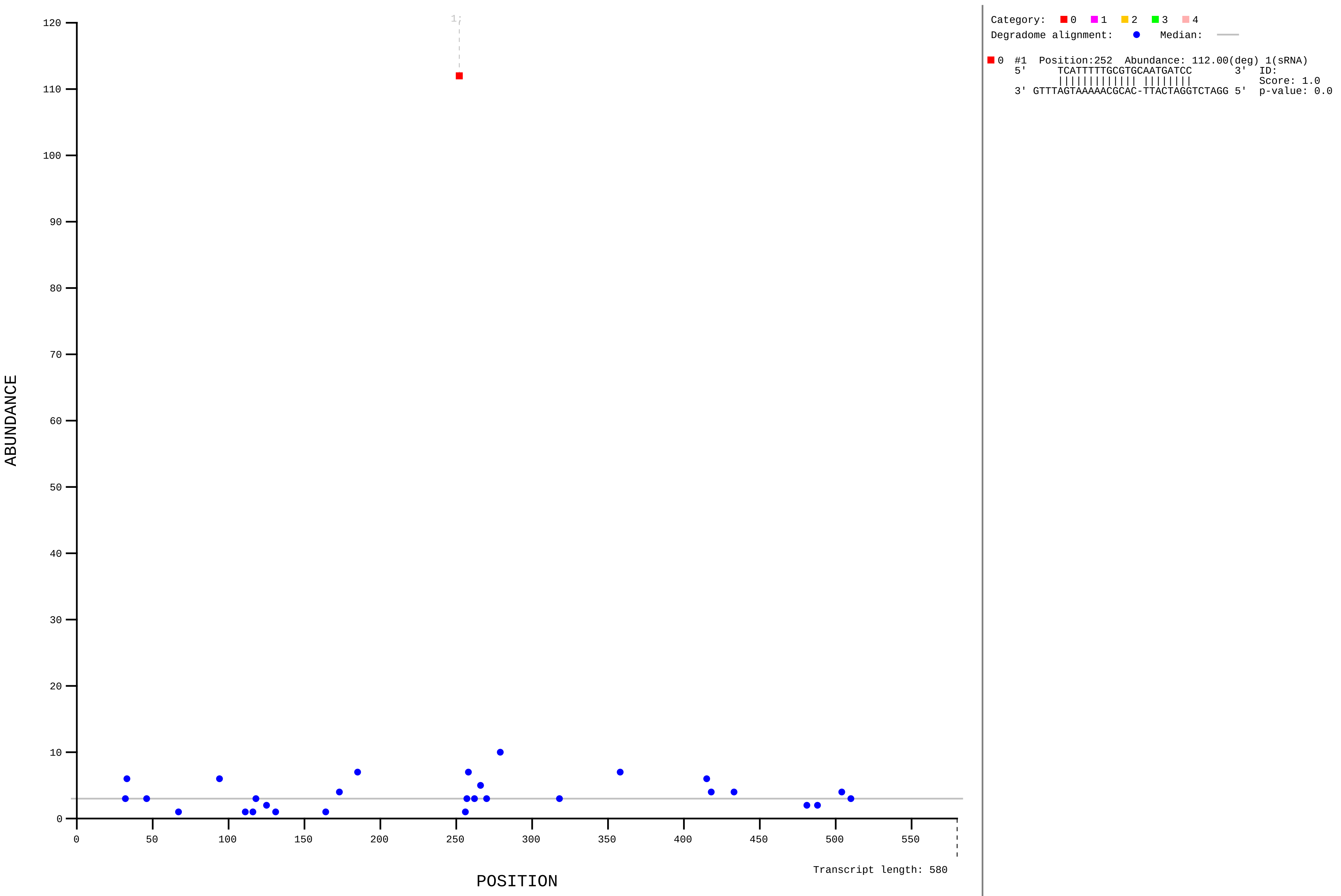

Cs7g15220.1 gene=Cs7g15220 CDS=1-999

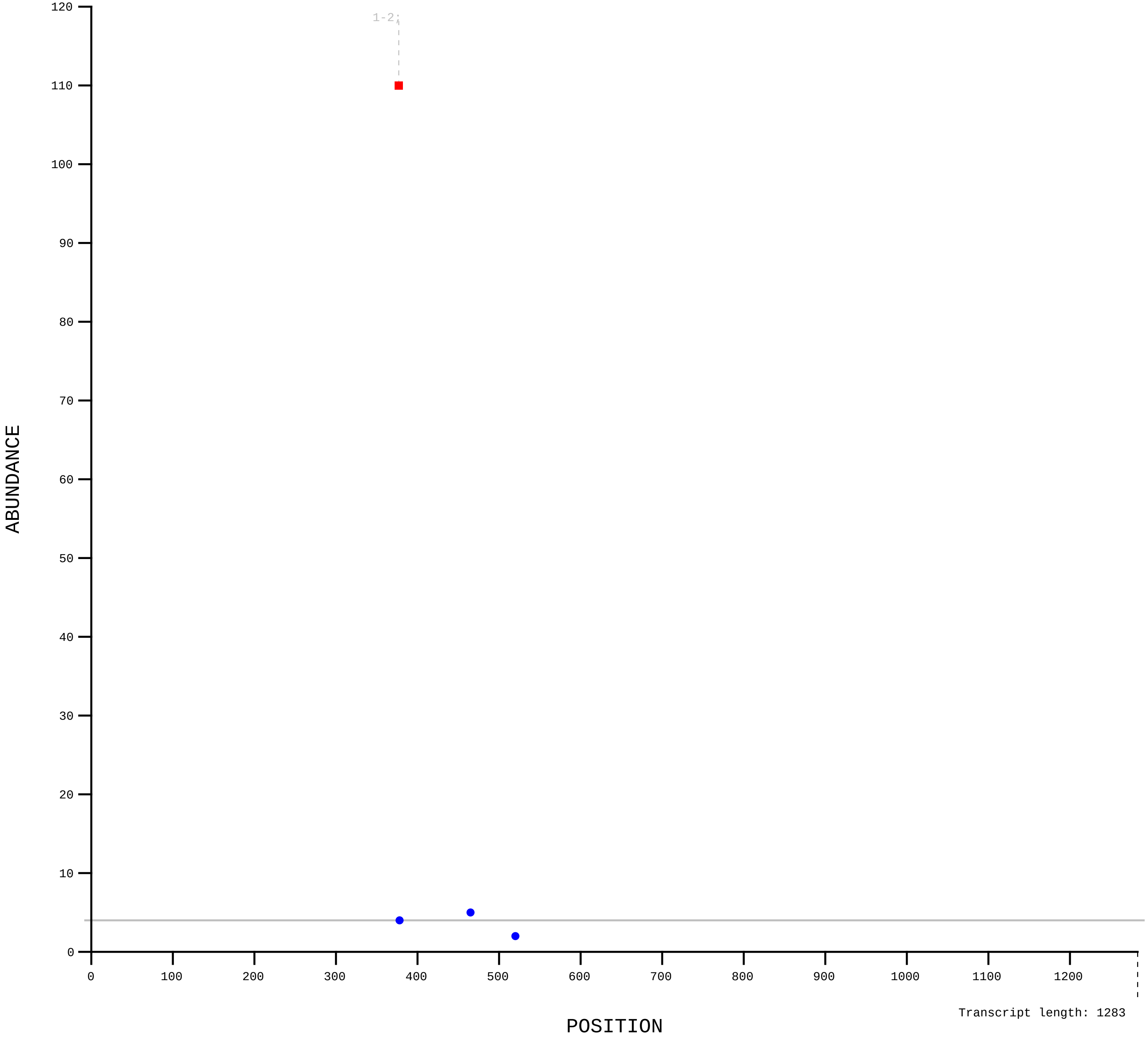


```
Category:  0  1  2  3  4
Degradome alignment:      Median:

0  #1  Position:377  Abundance: 110.00(deg) 3(sRNA)
   5'    TTCCACA-GCTTTCTTGAACTG        3'  ID:
         ||||||| |||||||||||| |            Score: 2.0
   3' ACTAAGGTGTCCGAAAGAACTTGCCAACGCCG 5'  p-value: 0.0

0  #2  Position:377  Abundance: 110.00(deg) 1(sRNA)
   5'    TTCCAC-GGCTTTCTTGAACTT        3'  ID:
         |||||| |||||||||||||              Score: 3.0
   3' ACTAAGGTGTCCGAAAGAACTTGCCAACGCCG 5'  p-value: 0.0
```

Cs1g22520.1 gene=Cs1g22520 CDS=1207-1614

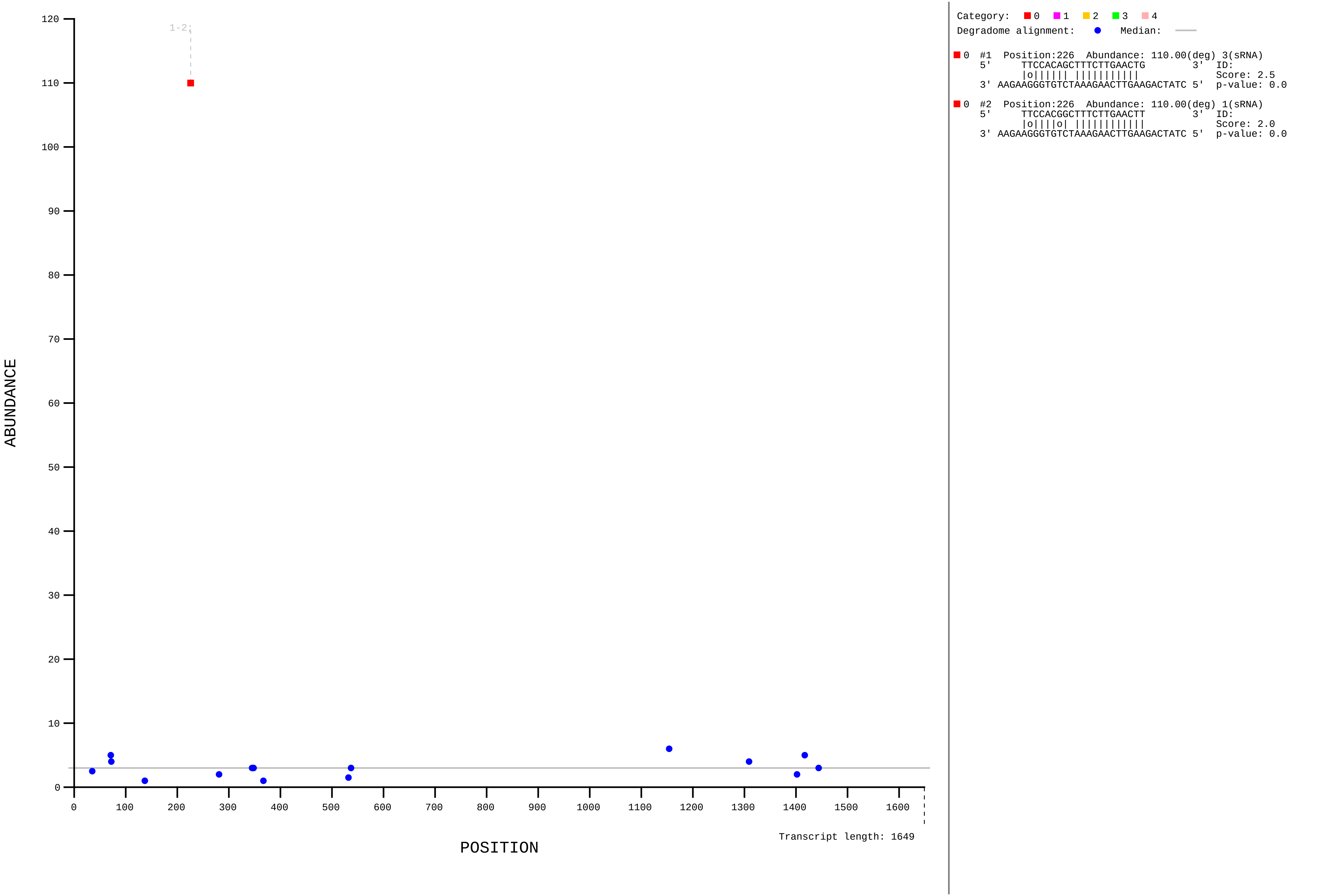

Cs5g08980.1 gene=Cs5g08980 CDS=340-2721

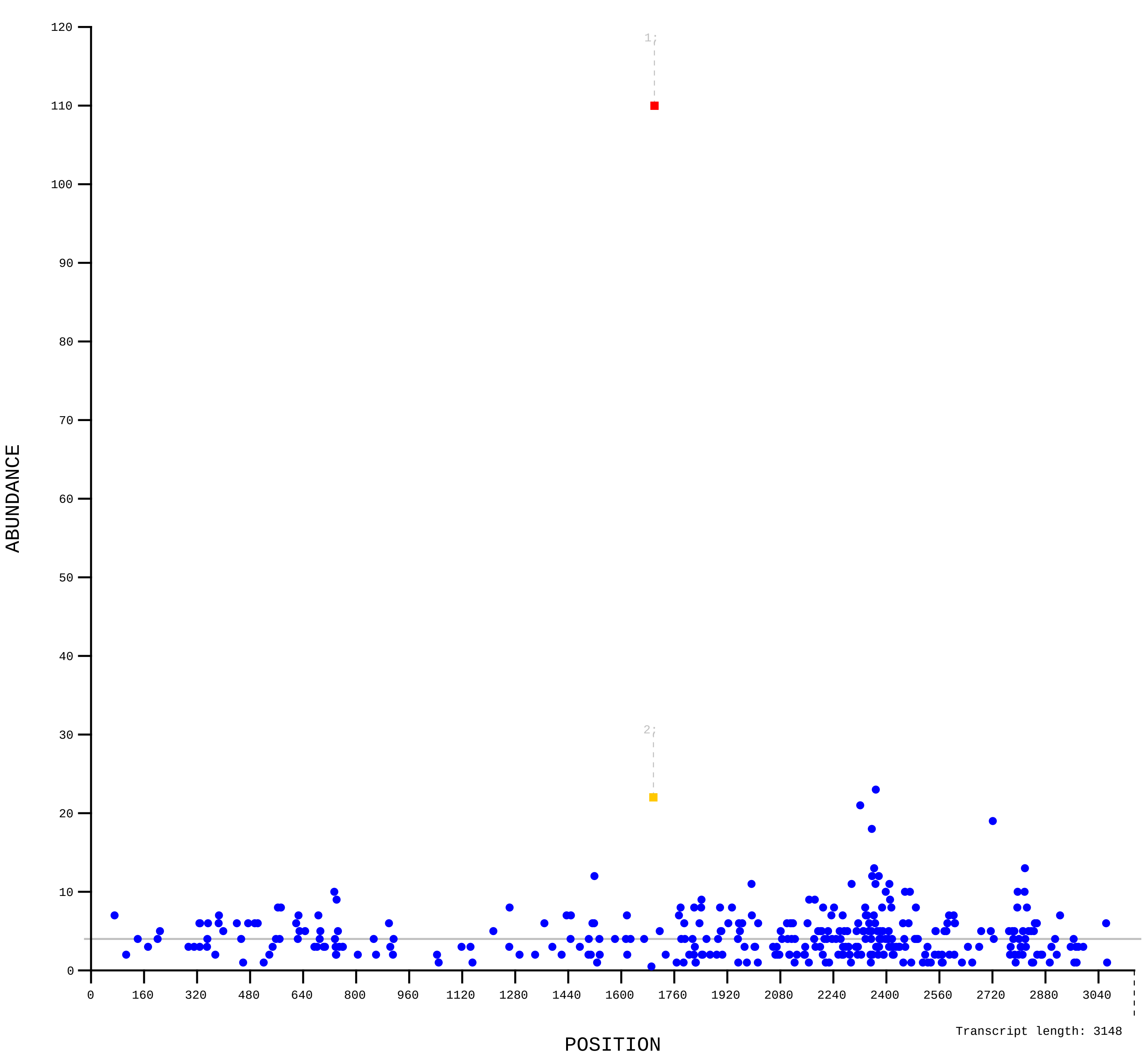

Category: 0 1 2 3 4
Degradome alignment: Median:

0 #1 Position:1700 Abundance: 110.00(deg) 1(sRNA)
5' TGATTGAGCCGTGCCAATATC 3' ID:
||||||||||o|||||||||| Score: 0.5
3' GACCACTAACTCGGCGCGGTTATAGGGAACGC 5' p-value: 0.0

2 #2 Position:1697 Abundance: 22.00(deg) 1(sRNA)
5' TTGAGCCGCGCCAATATCACG 3' ID:
||||||||||||||||||| | Score: 2.0
3' CACTAACTCGGCGCGGTTATAGGGAACGCGCA 5' p-value: 0.0

orange1.1t04537.1 gene=orange1.1t04537 CDS=344-3817

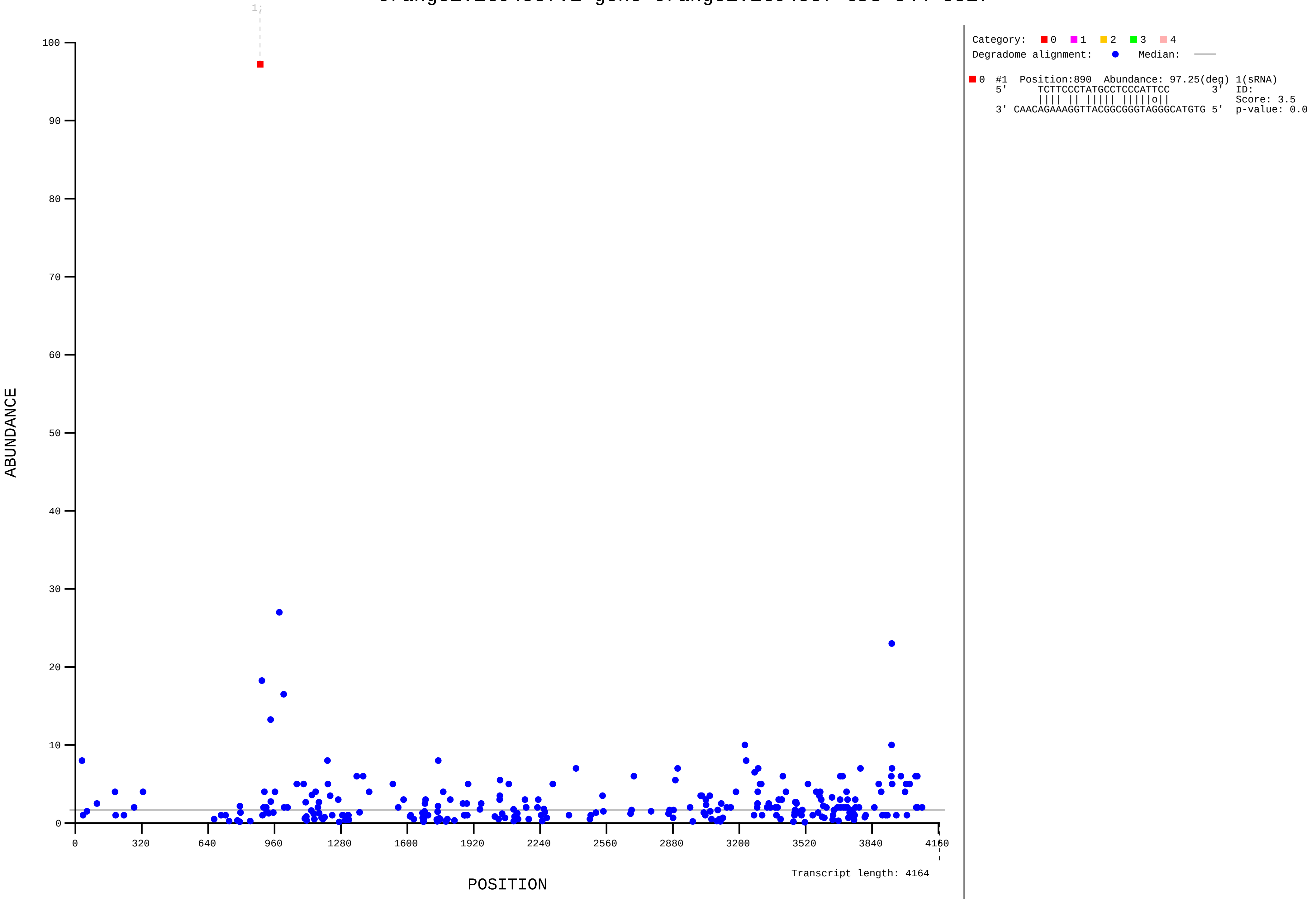

Cs3g12720.1 gene=Cs3g12720 CDS=1-1950

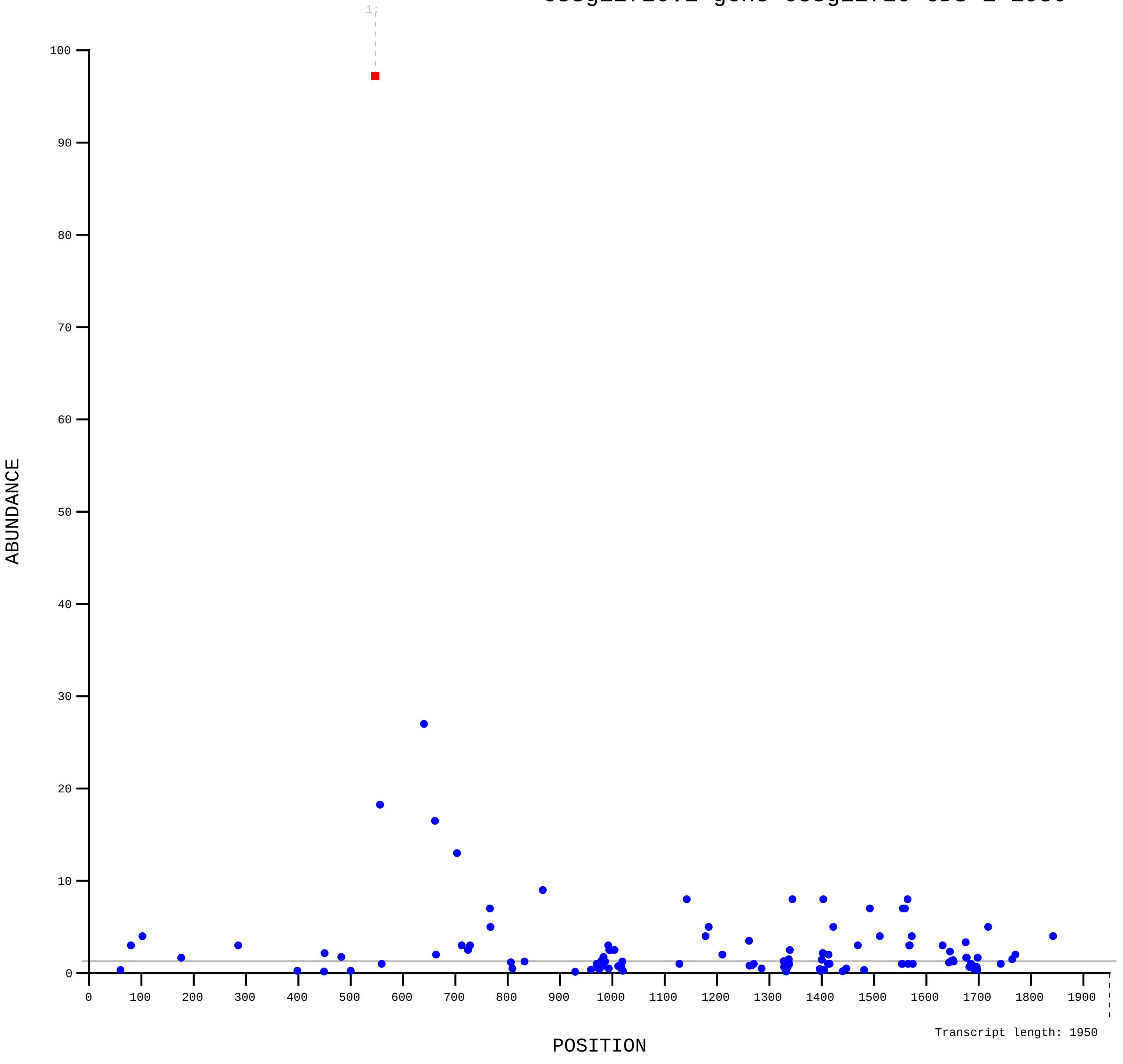

Category: 0 1 2 3 4
Degradome alignment: Median:

```
0  #1  Position:547  Abundance: 97.25(deg) 1(sRNA)
   5'     TCTTCCCTATGCCTCCCATTCC       3'  ID:
          |||| || ||||| ||||| ||           Score: 4.0
   3' CAACAGAAAGGTTACGGTGGGTACGGCATGTG 5'  p-value: 0.0
```

Cs3g12760.1 gene=Cs3g12760 CDS=491-2215

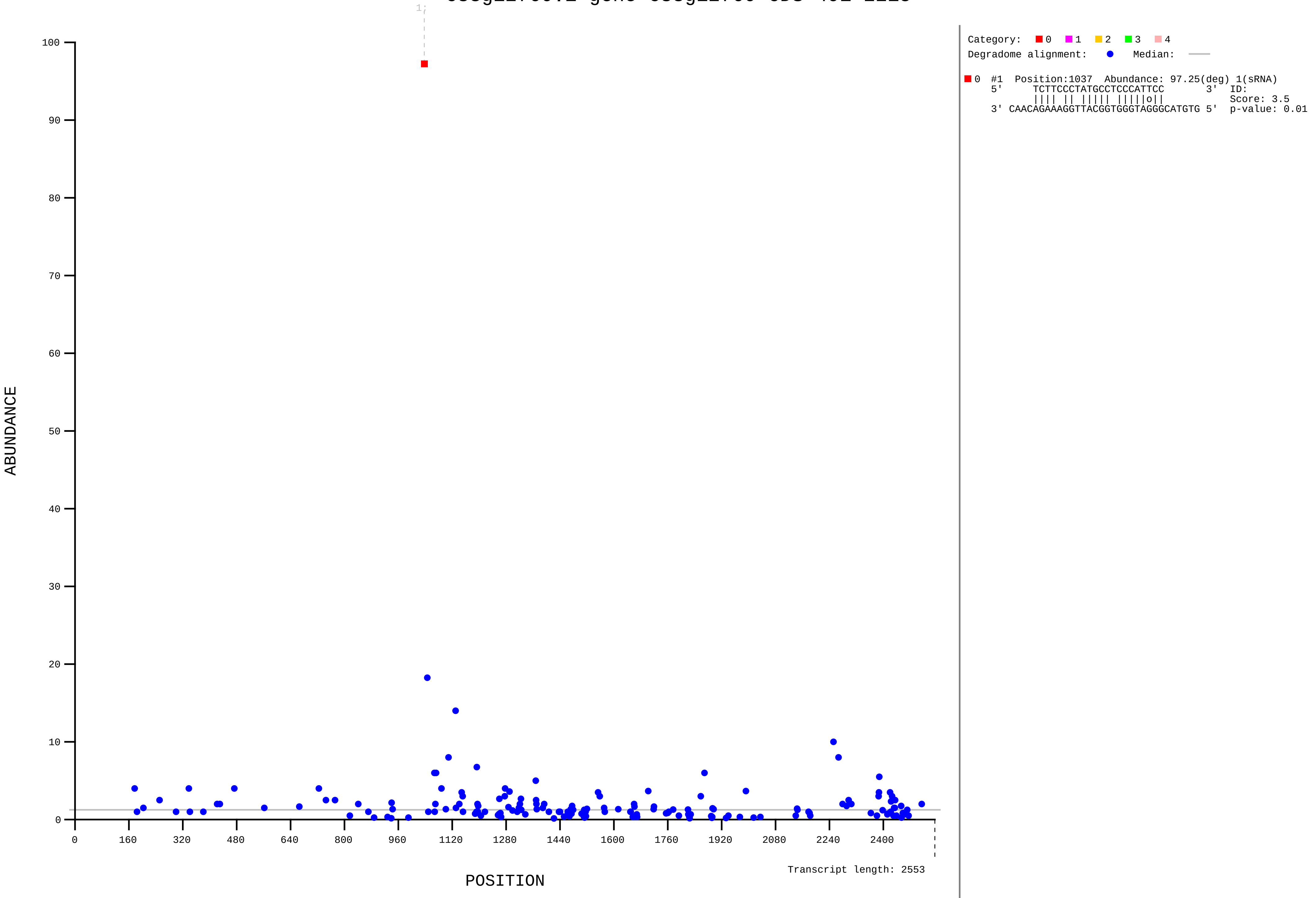

Cs3g12850.1 gene=Cs3g12850 CDS=1-3774

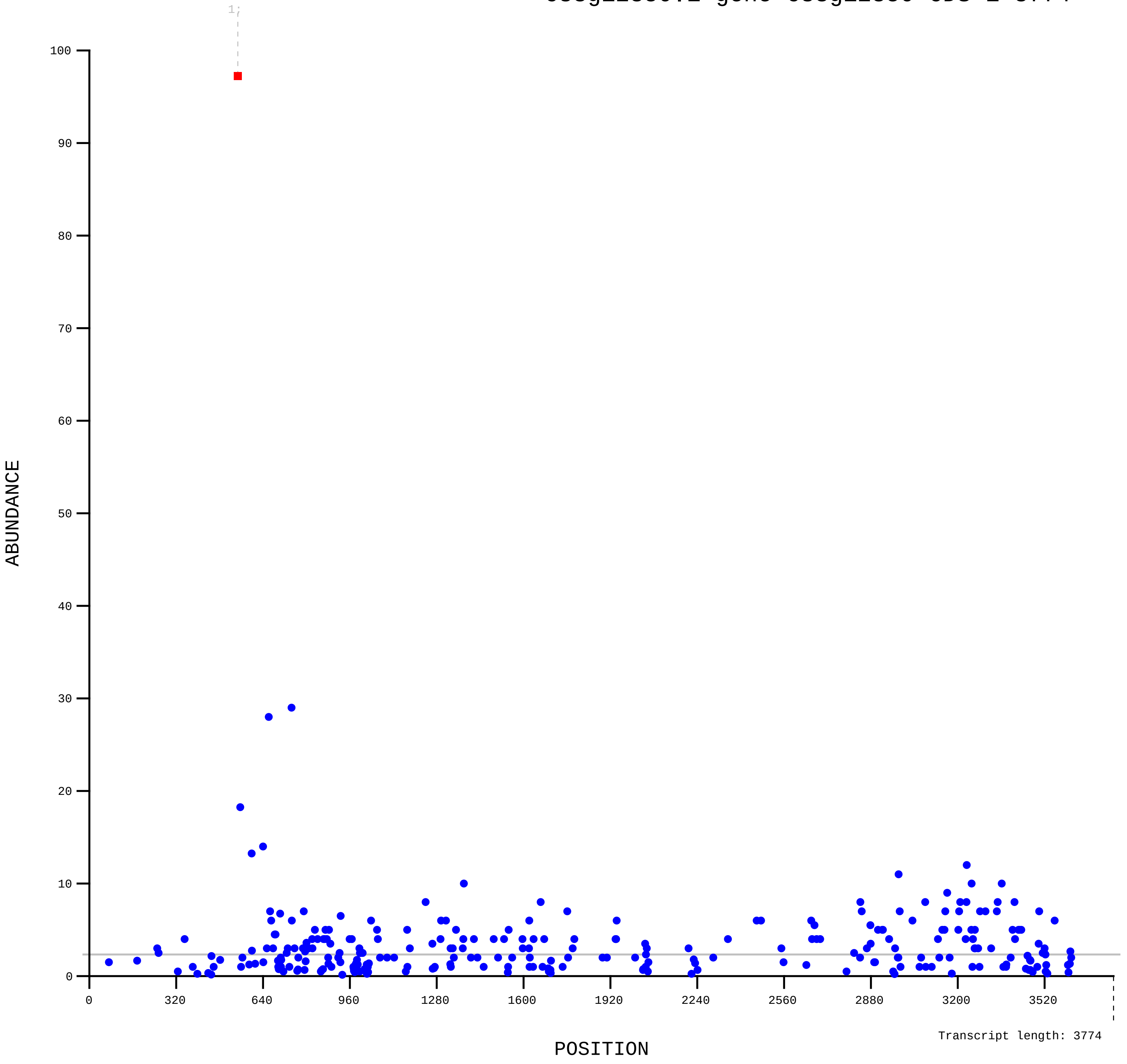

Category: 0 1 2 3 4
Degradome alignment: Median:

0 #1 Position:547 Abundance: 97.25(deg) 1(sRNA)
5' TCTTCCCTATGCCTCCCATTCC 3' ID:
|||| || ||||| |||||o|| Score: 3.5
3' CAACAGAAAGGTTACGGTGGGTAGGGCATGTG 5' p-value: 0.0

Cs7g10850.1 gene=Cs7g10850 CDS=1-1407

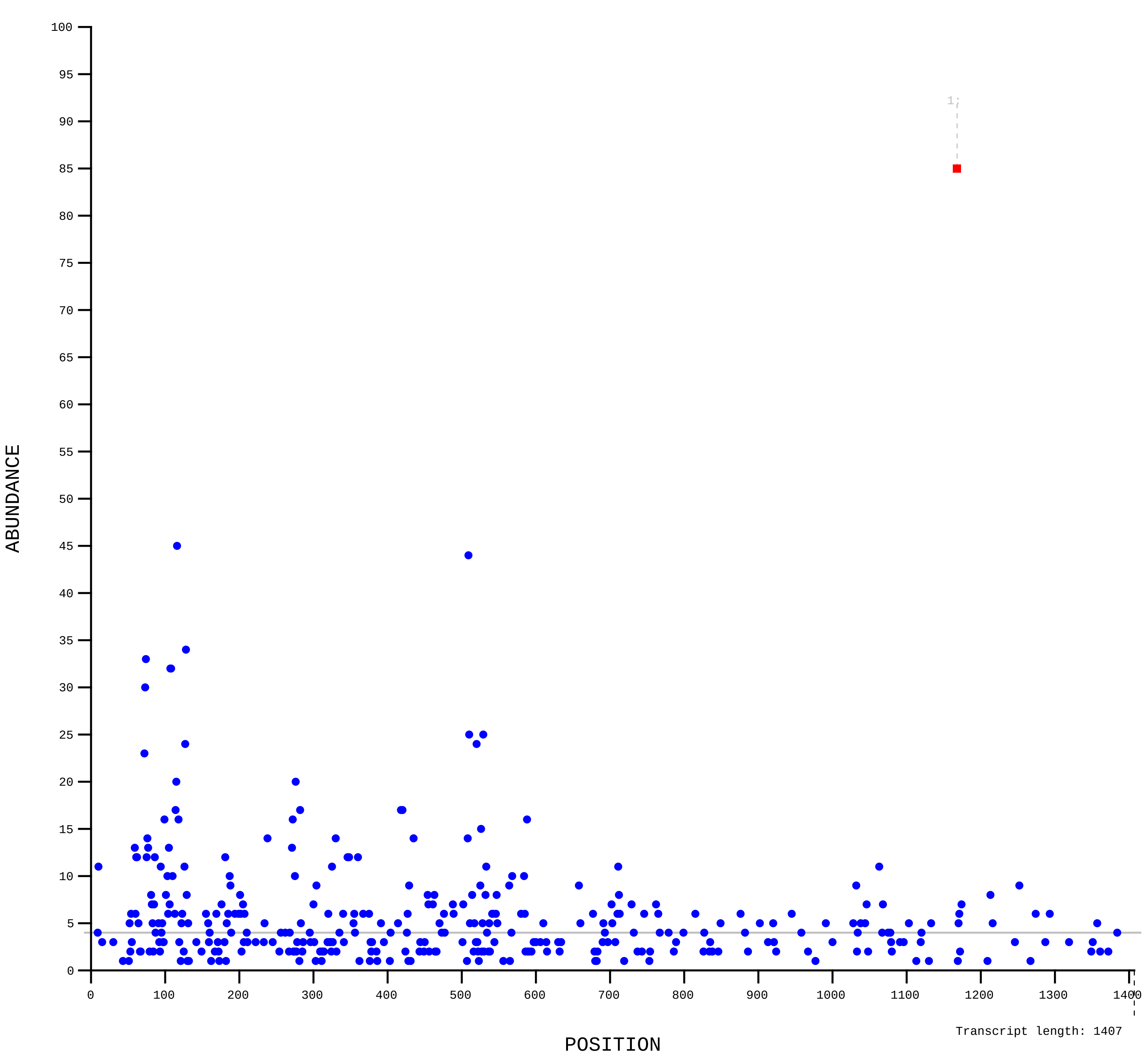

Category: 0 1 2 3 4
Degradome alignment: Median:

0 #1 Position:1168 Abundance: 85.00(deg) 2(sRNA)

```
5'     TTGGCATTCTGTCCACCTCC         3'  ID:
       ||||||||||||| ||||||             Score: 1.0
3' TATAAACCGTAAGACAGTTGGAGGAAGGTGTT 5'  p-value: 0.0
```

orange1.1t00200.2 gene=orange1.1t00200 CDS=412-2520

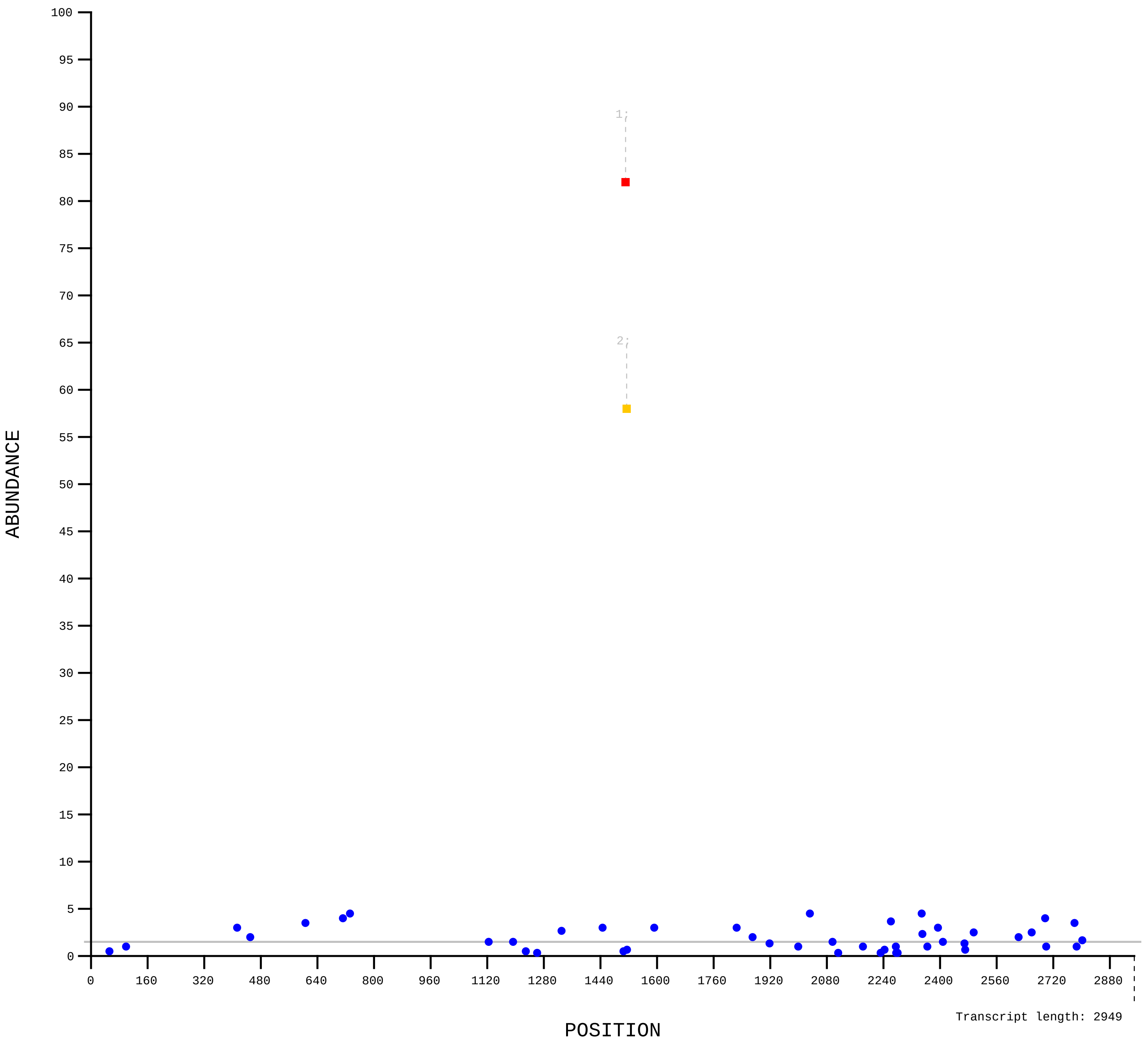

Category: 0 1 2 3 4
Degradome alignment: Median:

```
0  #1  Position:1511  Abundance: 82.00(deg) 1(sRNA)
   5'     TTGAGCCGCGCCAATATCACG        3'  ID:
          |||||||||||||||||| |             Score: 2.0
   3' CACTAACTCGGCGCGGTTATAGGGAACGCGCA 5'  p-value: 0.0

2  #2  Position:1514  Abundance: 58.00(deg) 1(sRNA)
   5'     TGATTGAGCCGTGCCAATATC        3'  ID:
          |||||||||||o|||||||||            Score: 0.5
   3' GACCACTAACTCGGCGCGGTTATAGGGAACGC 5'  p-value: 0.0
```

orange1.1t00200.1 gene=orange1.1t00200 CDS=412-2337

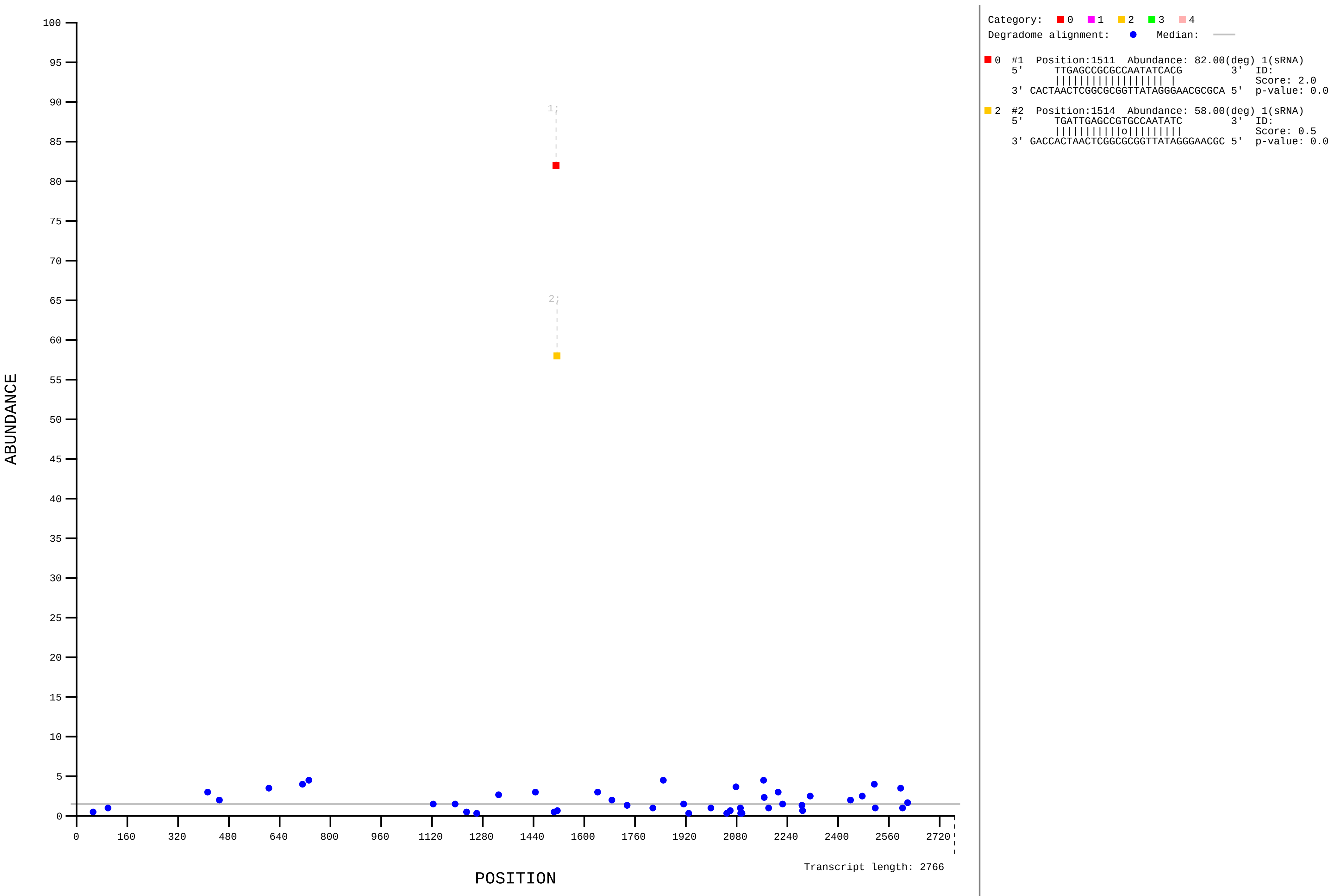

orange1.1t00199.1 gene=orange1.1t00199 CDS=501-2621

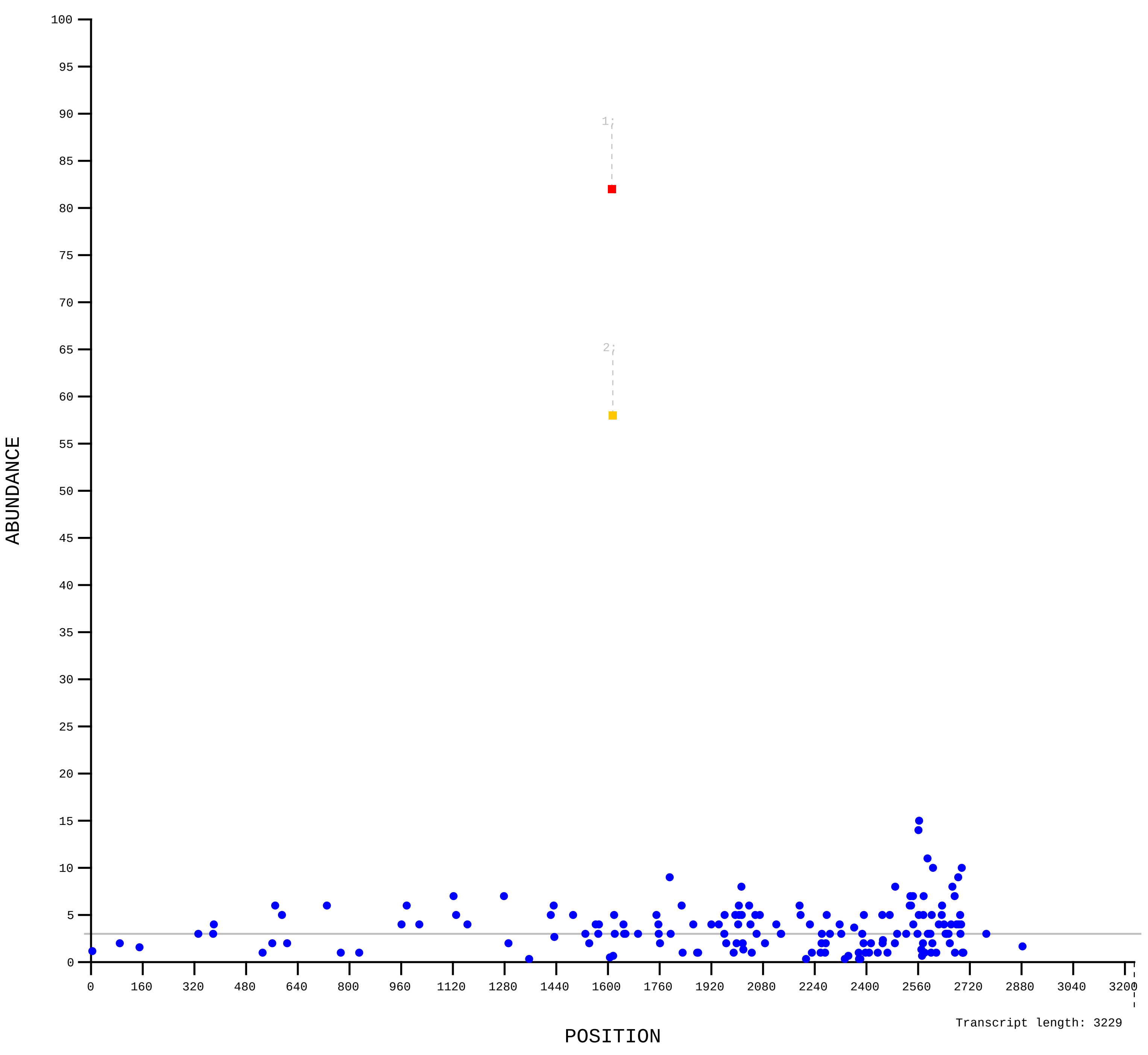


```
Category:  ■0  ■1  ■2  ■3  ■4
Degradome alignment:  ●   Median: ——

■0  #1  Position:1612  Abundance: 82.00(deg) 1(sRNA)
    5'     TTGAGCCGCGCCAATATCACG        3'  ID:
           |||||||||||||||||| |             Score: 2.0
    3' CACTAACTCGGCGCGGTTATAGGGAACGCGCT 5'  p-value: 0.0

■2  #2  Position:1615  Abundance: 58.00(deg) 1(sRNA)
    5'     TGATTGAGCCGTGCCAATATC        3'  ID:
           |||||||||||o|||||||||            Score: 0.5
    3' GACCACTAACTCGGCGCGGTTATAGGGAACGC 5'  p-value: 0.0
```

Cs8g16120.1 gene=Cs8g16120 CDS=132-1373

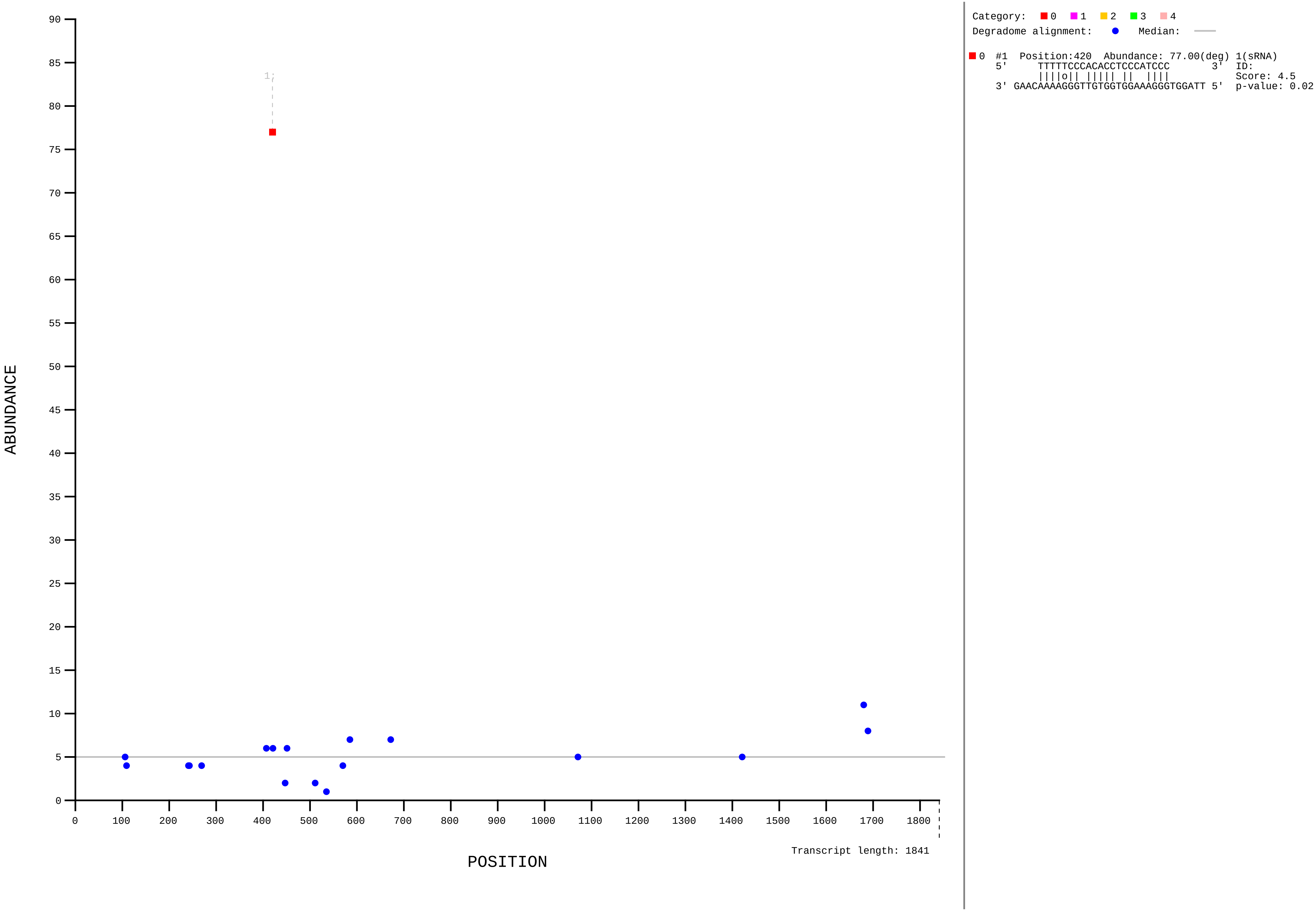

Cs5g16710.2 gene=Cs5g16710 CDS=523-3744

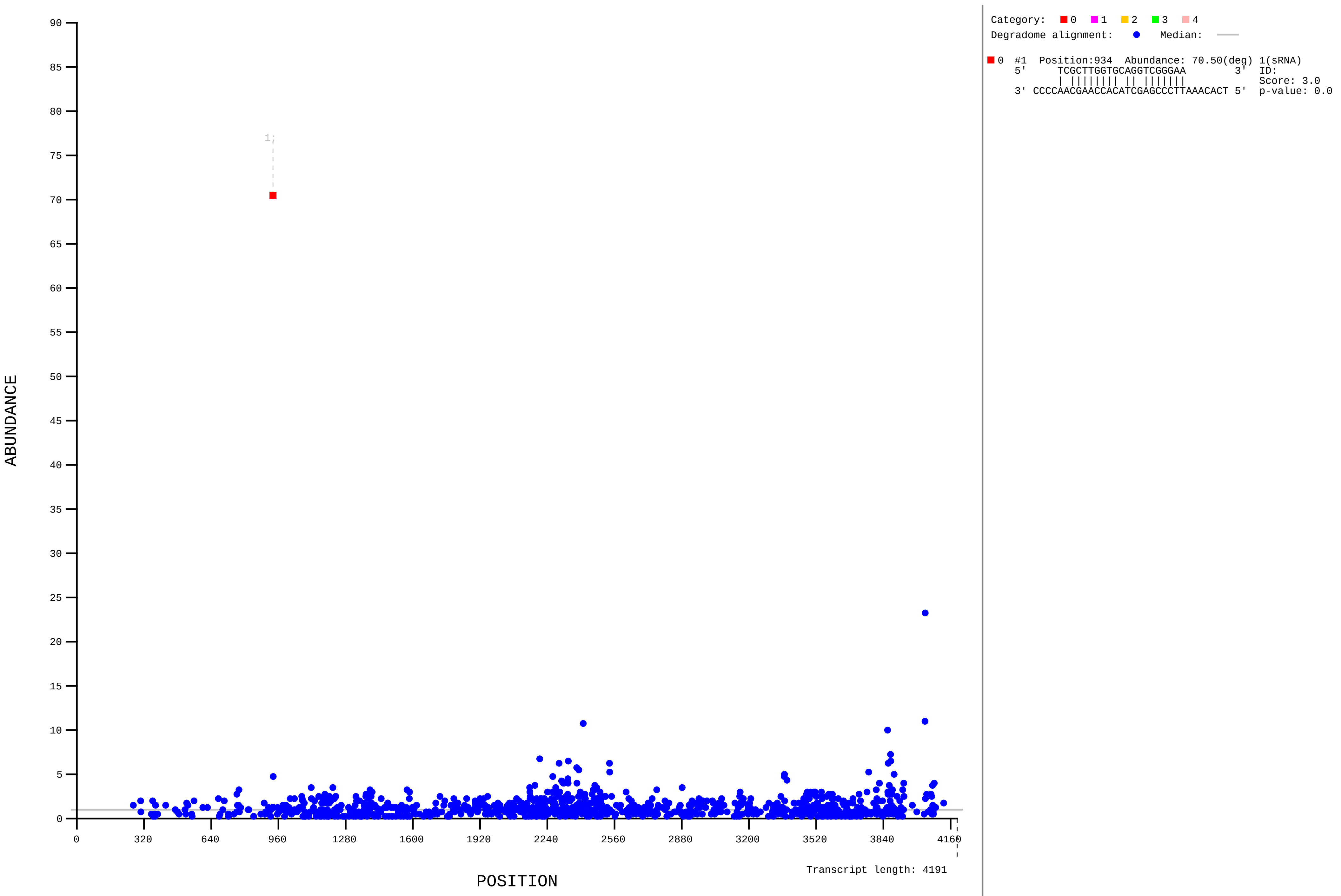

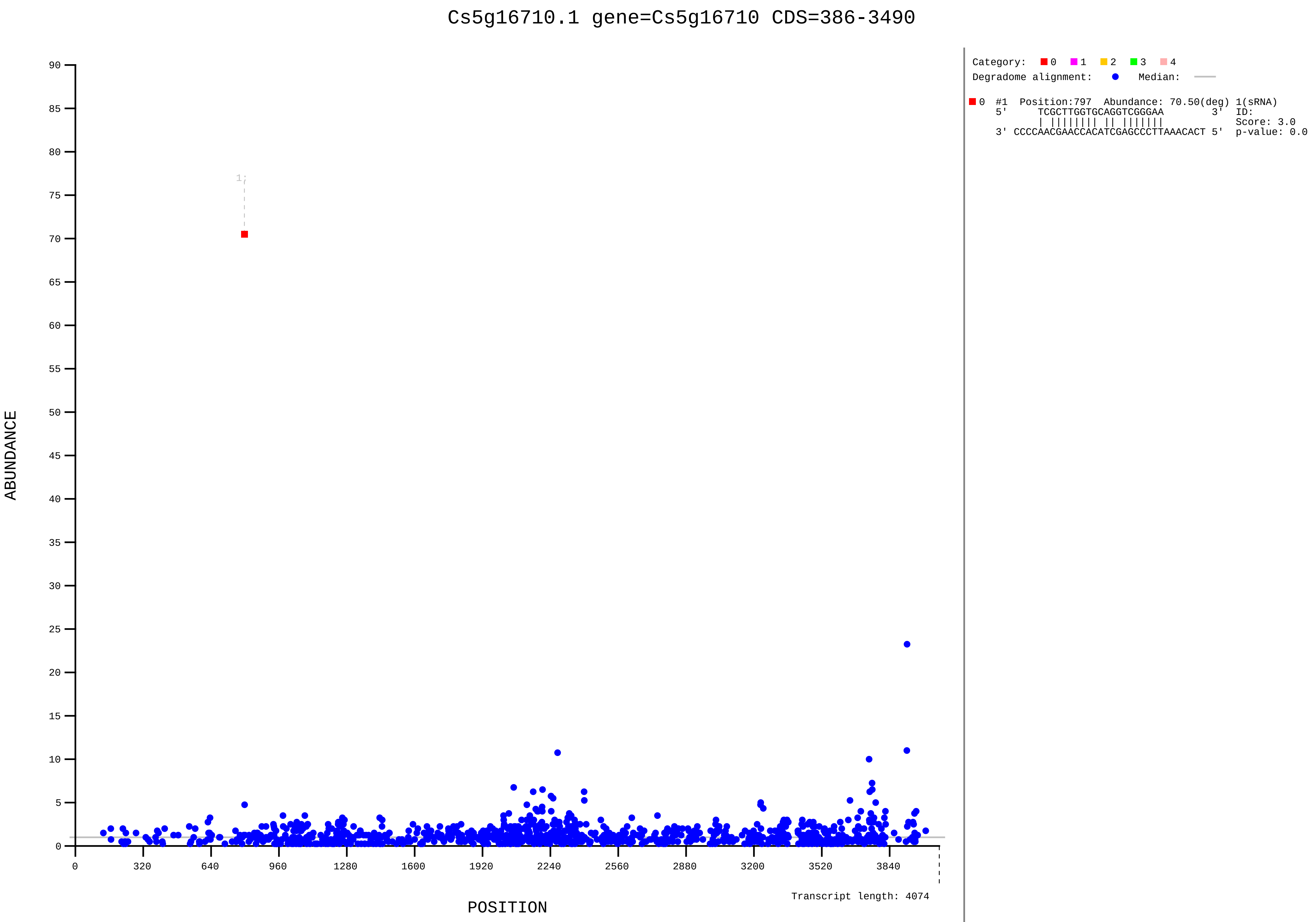

Cs5g16710.1 gene=Cs5g16710 CDS=386-3490
ABUNDANCE
POSITION
Transcript length: 4074
Category: 0 1 2 3 4
Degradome alignment:
Median:
0 #1 Position:797 Abundance: 70.50(deg) 1(sRNA)
5' TCGCTTGGTGCAGGTCGGGAA 3' ID:
| |||||||| || ||||||| Score: 3.0
3' CCCCAACGAACCACATCGAGCCCTTAAACACT 5' p-value: 0.0

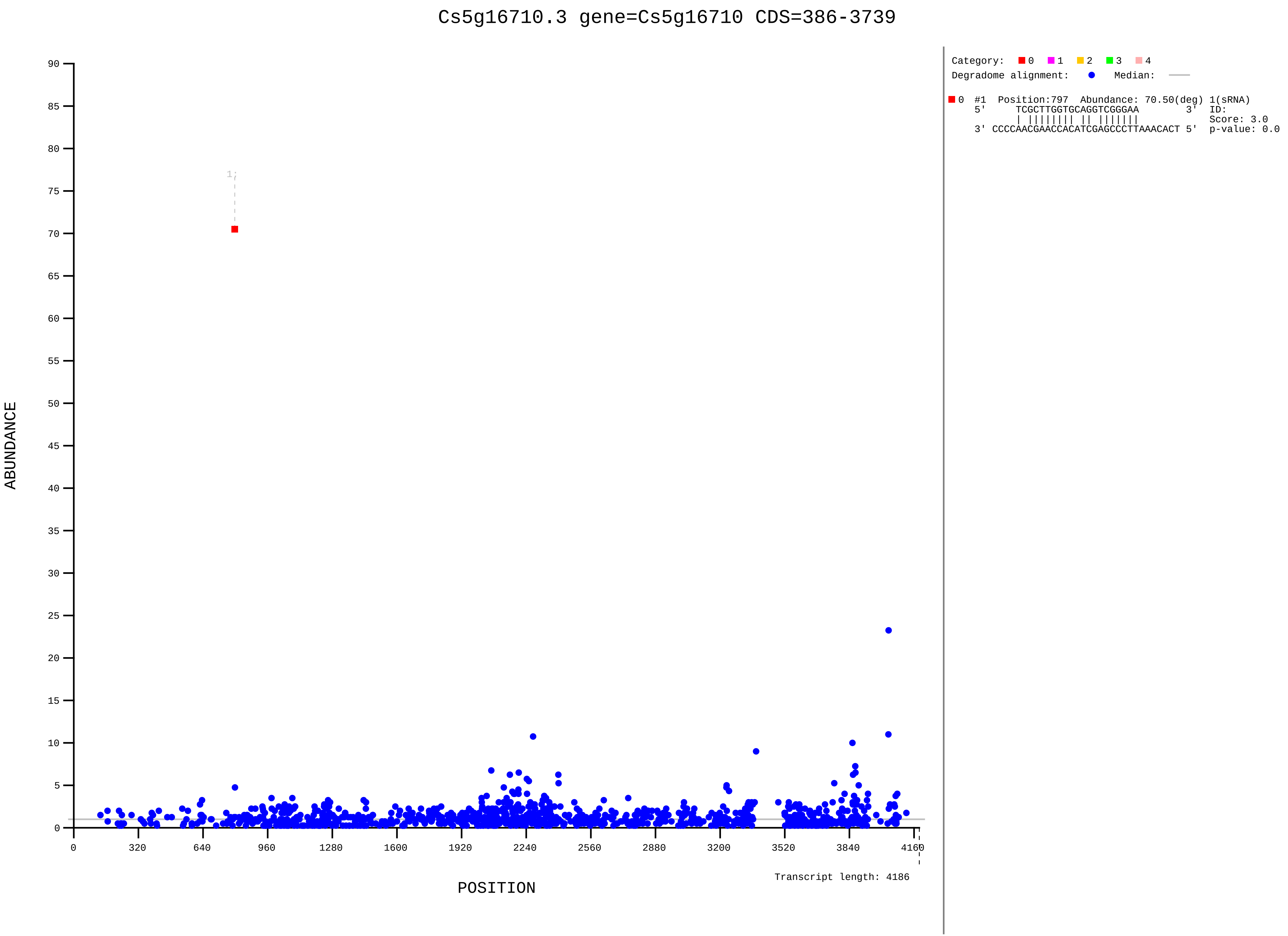
Cs5g16710.3 gene=Cs5g16710 CDS=386-3739
ABUNDANCE
POSITION
Transcript length: 4186
Category: 0 1 2 3 4
Degradome alignment:
Median:
0 #1 Position:797 Abundance: 70.50(deg) 1(sRNA)
5' TCGCTTGGTGCAGGTCGGGAA 3' ID:
| |||||||| || ||||||| Score: 3.0
3' CCCCAACGAACCACATCGAGCCCTTAAACACT 5' p-value: 0.0

Cs5g16710.4 gene=Cs5g16710 CDS=386-3289

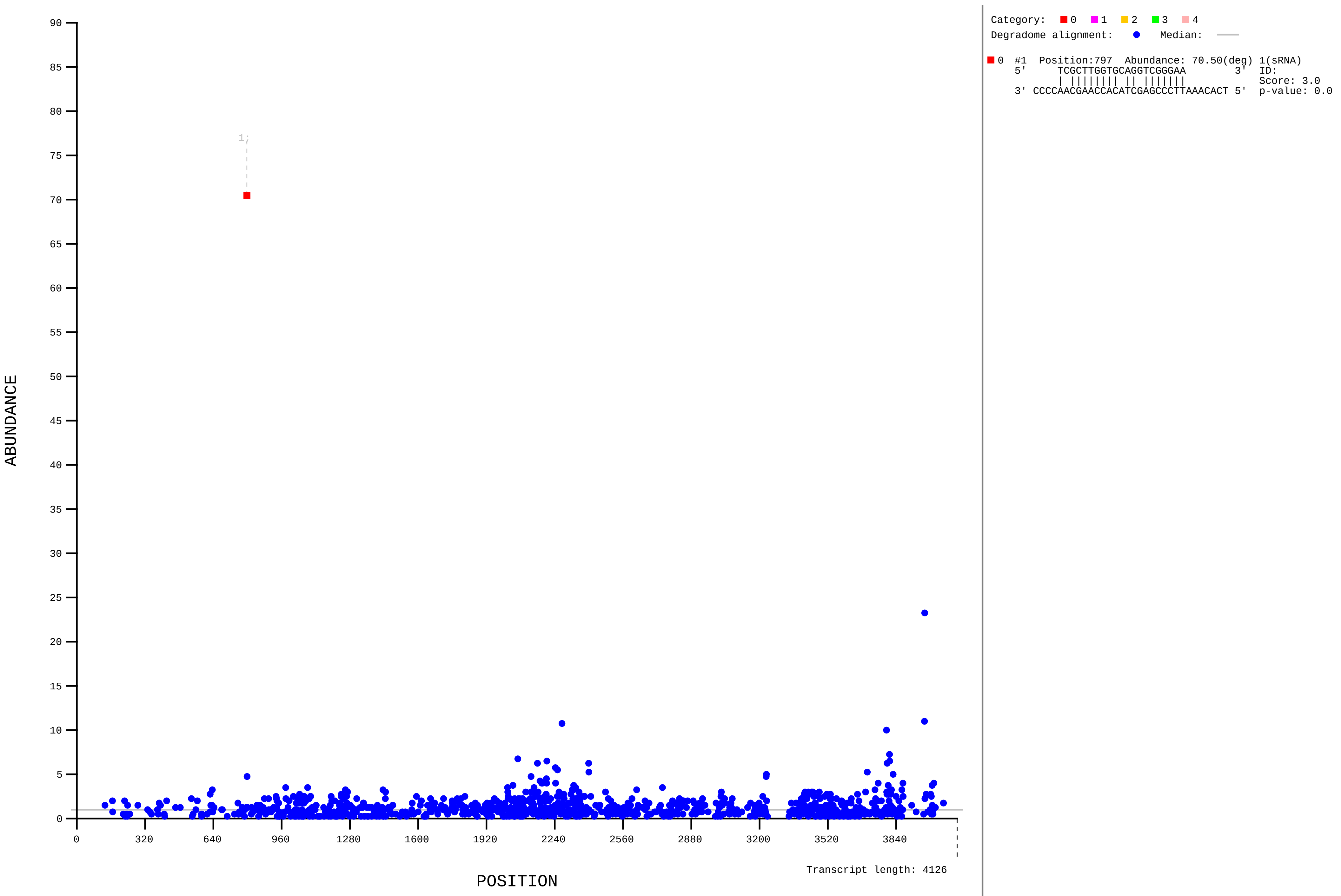

Cs8g16510.2 gene=Cs8g16510 CDS=184-2415

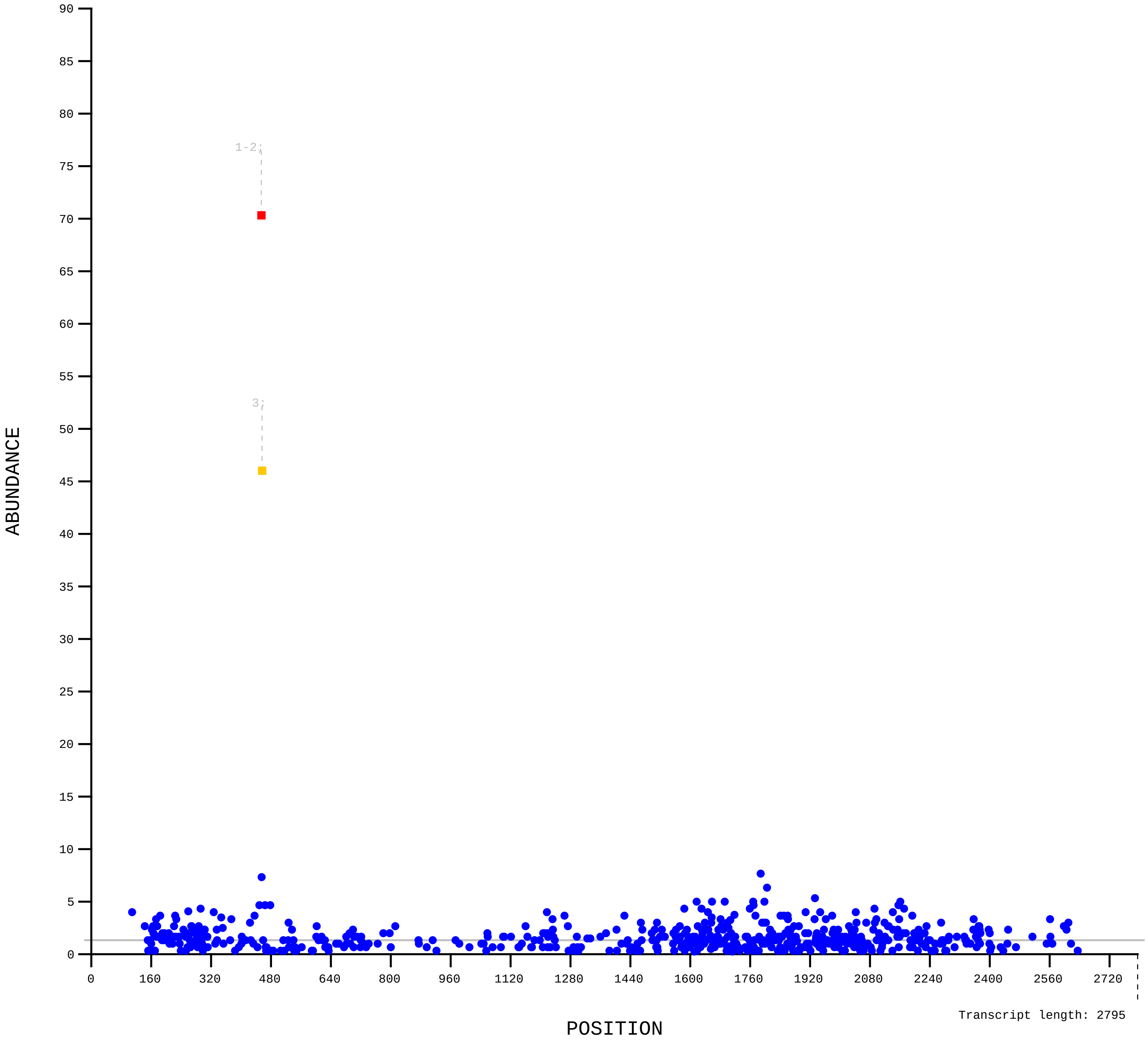

Category: 0 1 2 3 4
Degradome alignment: Median:

0 #1 Position:454 Abundance: 70.33(deg) 2(sRNA)
```
5'     TCGGACCAGGCTTCATTCCCC        3'  ID:
       o|||||||||||||||| |||            Score: 2.5
3' CTTAGGCCTGGTCCGAAGTA-GGGTCCGTAGA 5'  p-value: 0.0
```

0 #2 Position:454 Abundance: 70.33(deg) 1(sRNA)
```
5'     TCGGACCAGGCTTCATTCCCT        3'  ID:
       o|||||||||||||||| |||            Score: 2.5
3' CTTAGGCCTGGTCCGAAGTA-GGGTCCGTAGA 5'  p-value: 0.0
```

2 #3 Position:456 Abundance: 46.00(deg) 2(sRNA)
```
5'     TCTCGGACCAGGCTTCATTCC        3'  ID:
       || ||||||||||||||||o||           Score: 1.5
3' GCTTAG-GCCTGGTCCGAAGTAGGGTCCGTAG 5'  p-value: 0.0
```

Cs8g16510.1 gene=Cs8g16510 CDS=855-3395

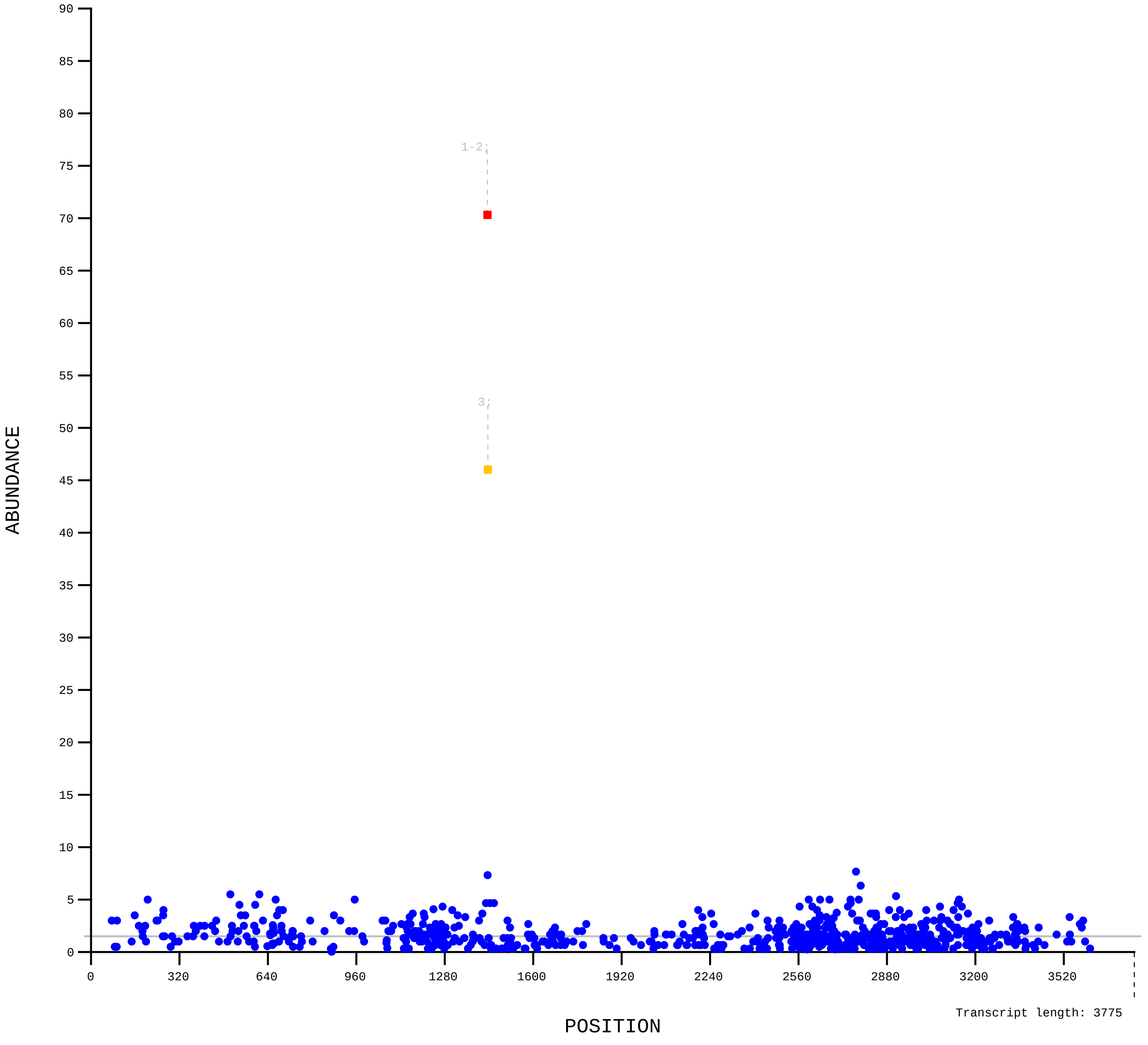


```
Category:  0  1  2  3  4
Degradome alignment:     Median:

0  #1  Position:1434  Abundance: 70.33(deg) 2(sRNA)
   5'     TCGGACCAGGCTTCATTCCCC        3'  ID:
          o|||||||||||||||| |||            Score: 2.5
   3' CTTAGGCCTGGTCCGAAGTA-GGGTCCGTAGA 5'  p-value: 0.0

0  #2  Position:1434  Abundance: 70.33(deg) 1(sRNA)
   5'     TCGGACCAGGCTTCATTCCCT        3'  ID:
          o|||||||||||||||| |||            Score: 2.5
   3' CTTAGGCCTGGTCCGAAGTA-GGGTCCGTAGA 5'  p-value: 0.0

2  #3  Position:1436  Abundance: 46.00(deg) 2(sRNA)
   5'     TCTCGGACCAGGCTTCATTCC        3'  ID:
          || ||||||||||||||||o||           Score: 1.5
   3' GCTTAG-GCCTGGTCCGAAGTAGGGTCCGTAG 5'  p-value: 0.0
```

Cs8g16510.3 gene=Cs8g16510 CDS=1500-3476

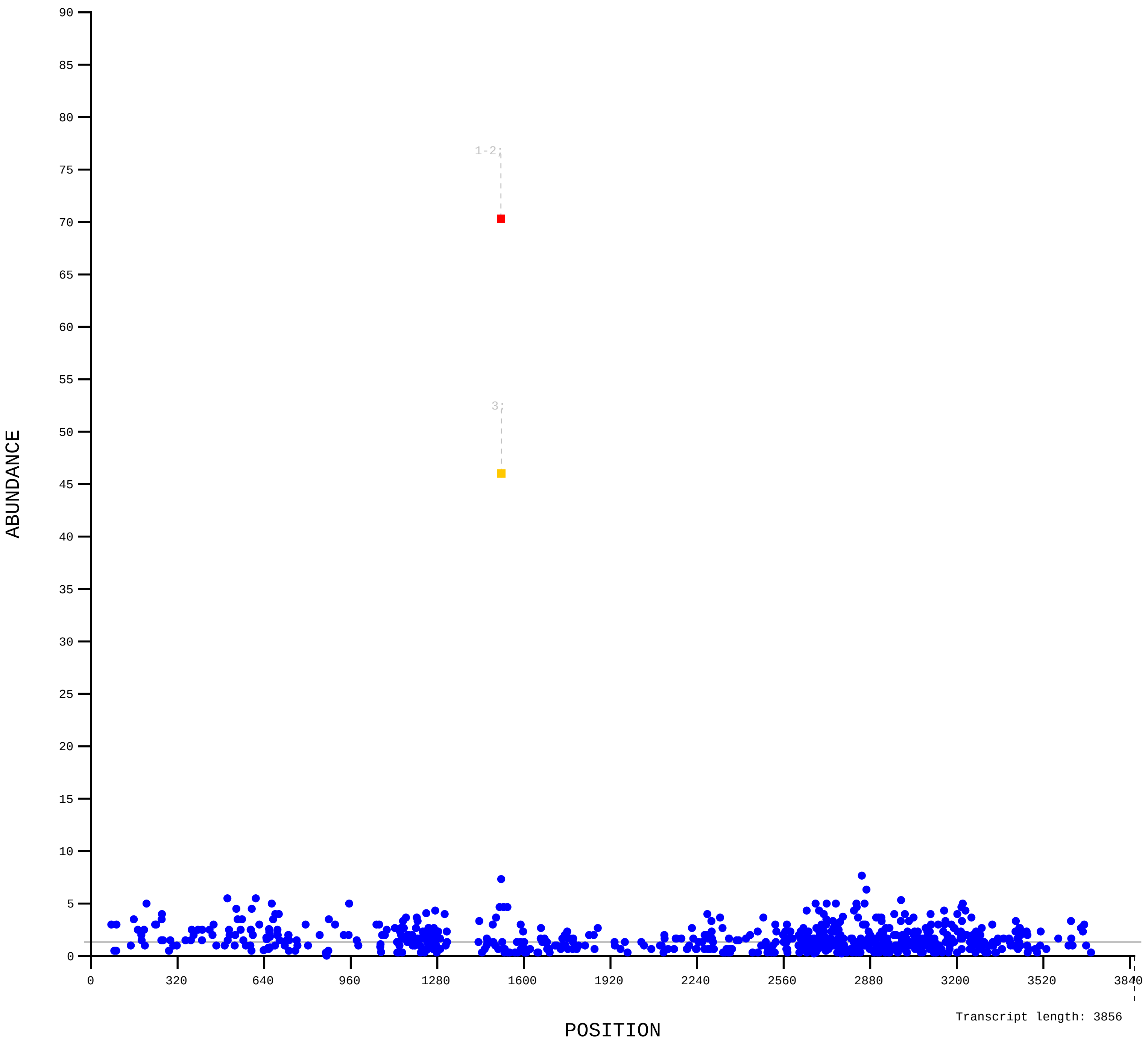


```
Category:  ■0  ■1  ■2  ■3  ■4
Degradome alignment:  ●   Median: ——

■0  #1  Position:1515  Abundance: 70.33(deg) 2(sRNA)
    5'     TCGGACCAGGCTTCATTCCCC        3'  ID:
           o|||||||||||||||| |||            Score: 2.5
    3' CTTAGGCCTGGTCCGAAGTA-GGGTCCGTAGA 5'  p-value: 0.0

■0  #2  Position:1515  Abundance: 70.33(deg) 1(sRNA)
    5'     TCGGACCAGGCTTCATTCCCT        3'  ID:
           o|||||||||||||||| |||            Score: 2.5
    3' CTTAGGCCTGGTCCGAAGTA-GGGTCCGTAGA 5'  p-value: 0.0

■2  #3  Position:1517  Abundance: 46.00(deg) 2(sRNA)
    5'     TCTCGGACCAGGCTTCATTCC        3'  ID:
           || |||||||||||||||o||            Score: 1.5
    3' GCTTAG-GCCTGGTCCGAAGTAGGGTCCGTAG 5'  p-value: 0.0
```

Cs5g10180.2 gene=Cs5g10180 CDS=1719-3230

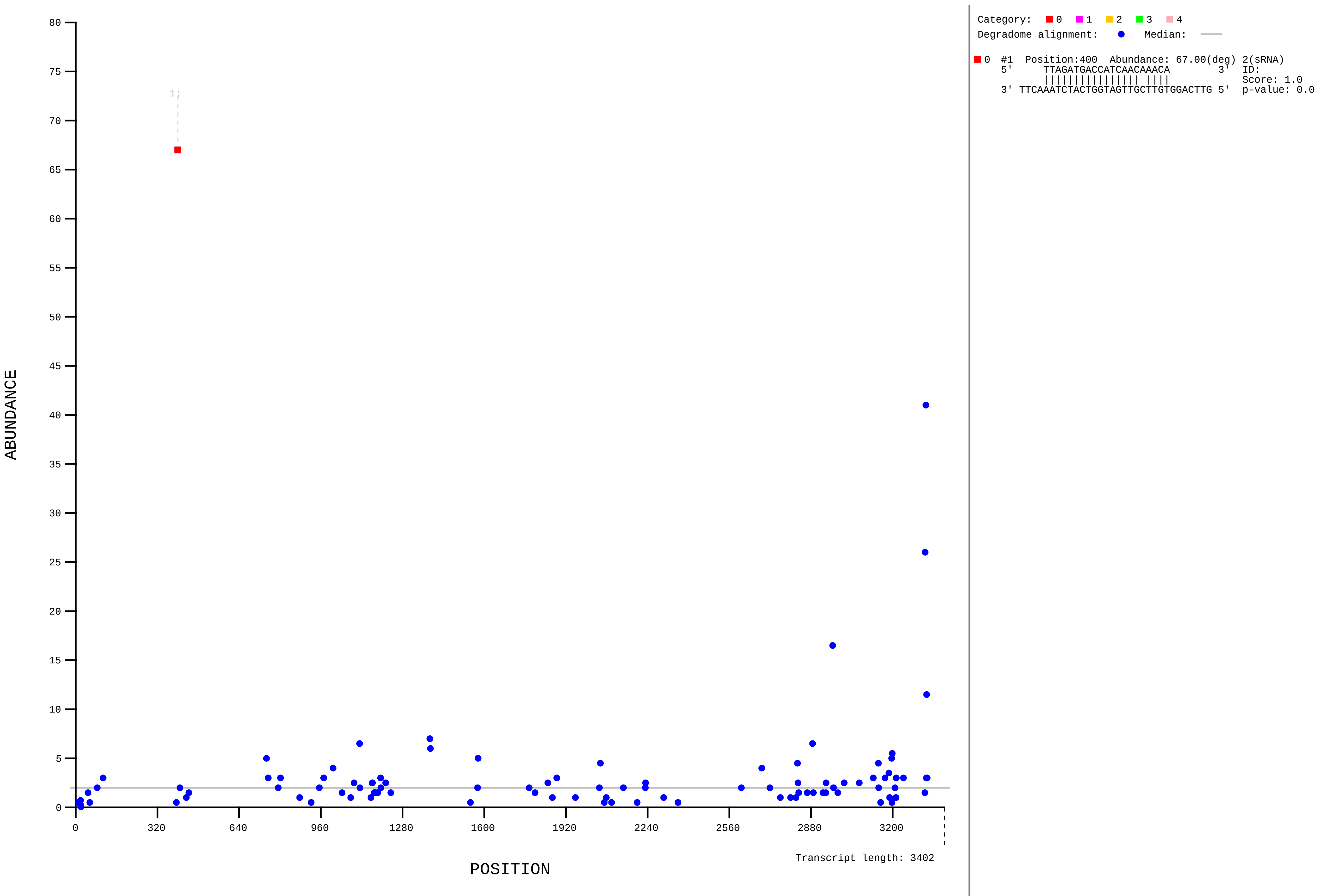

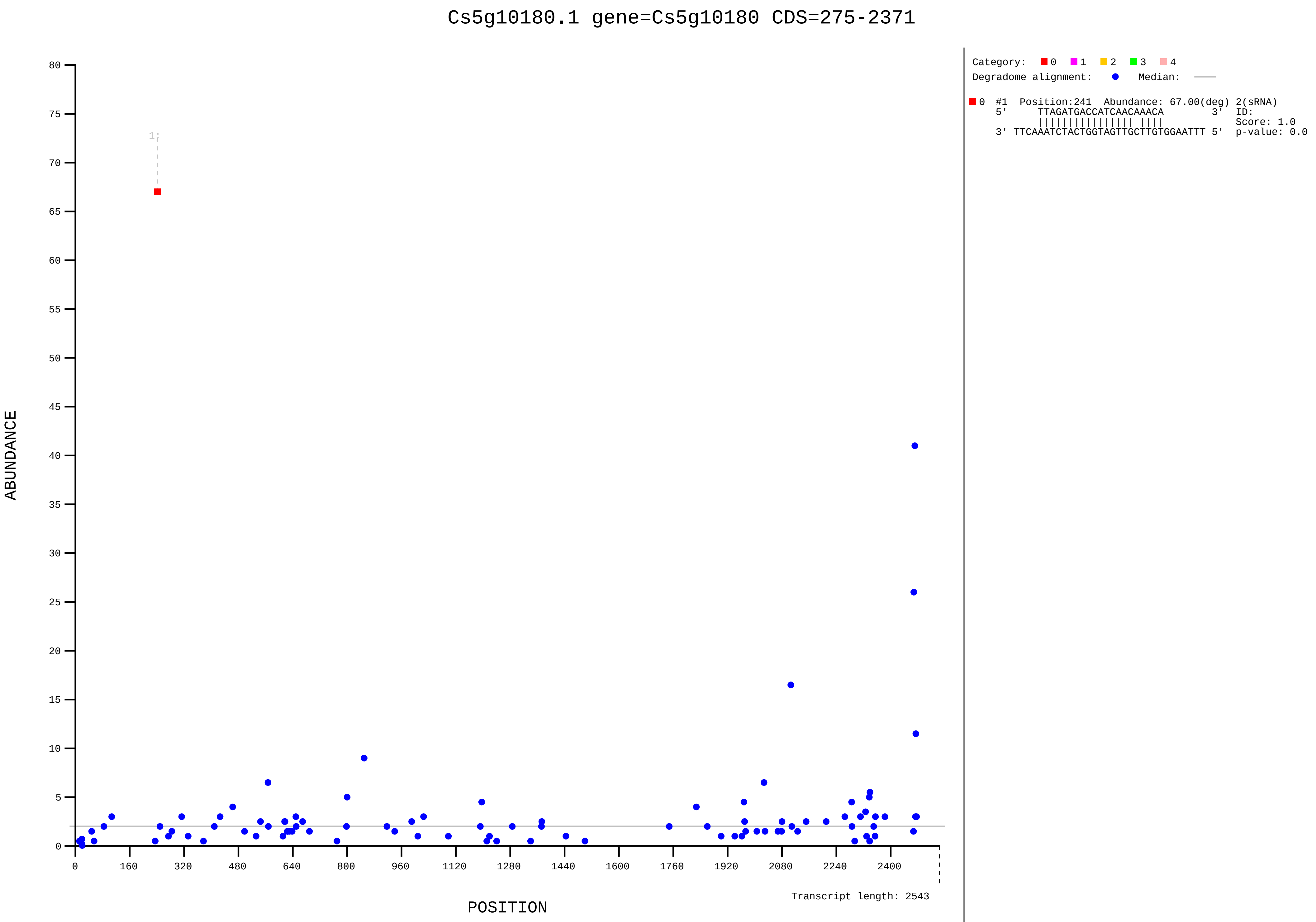
Cs5g10180.1 gene=Cs5g10180 CDS=275-2371
Category: 0 1 2 3 4
Degradome alignment:
Median:
0 #1 Position:241 Abundance: 67.00(deg) 2(sRNA)
5' TTAGATGACCATCAACAAACA 3' ID:
|||||||||||||||| ||||
3' TTCAAATCTACTGGTAGTTGCTTGTGGAATTT 5'
Score: 1.0
p-value: 0.0
ABUNDANCE
POSITION
Transcript length: 2543
0
5
10
15
20
25
30
35
40
45
50
55
60
65
70
75
80
0
160
320
480
640
800
960
1120
1280
1440
1600
1760
1920
2080
2240
2400

Cs1g15550.1 gene=Cs1g15550 CDS=101-2779

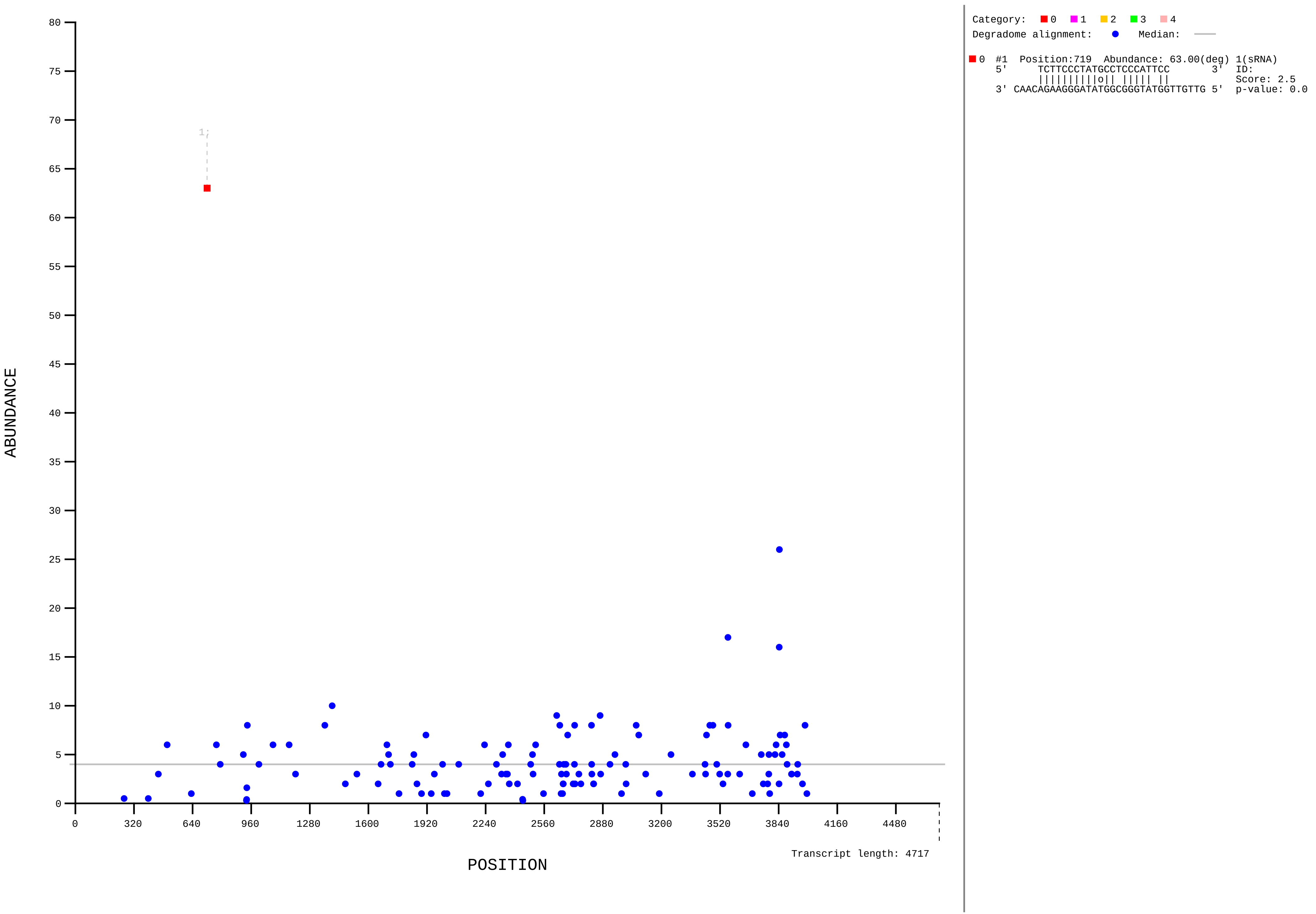

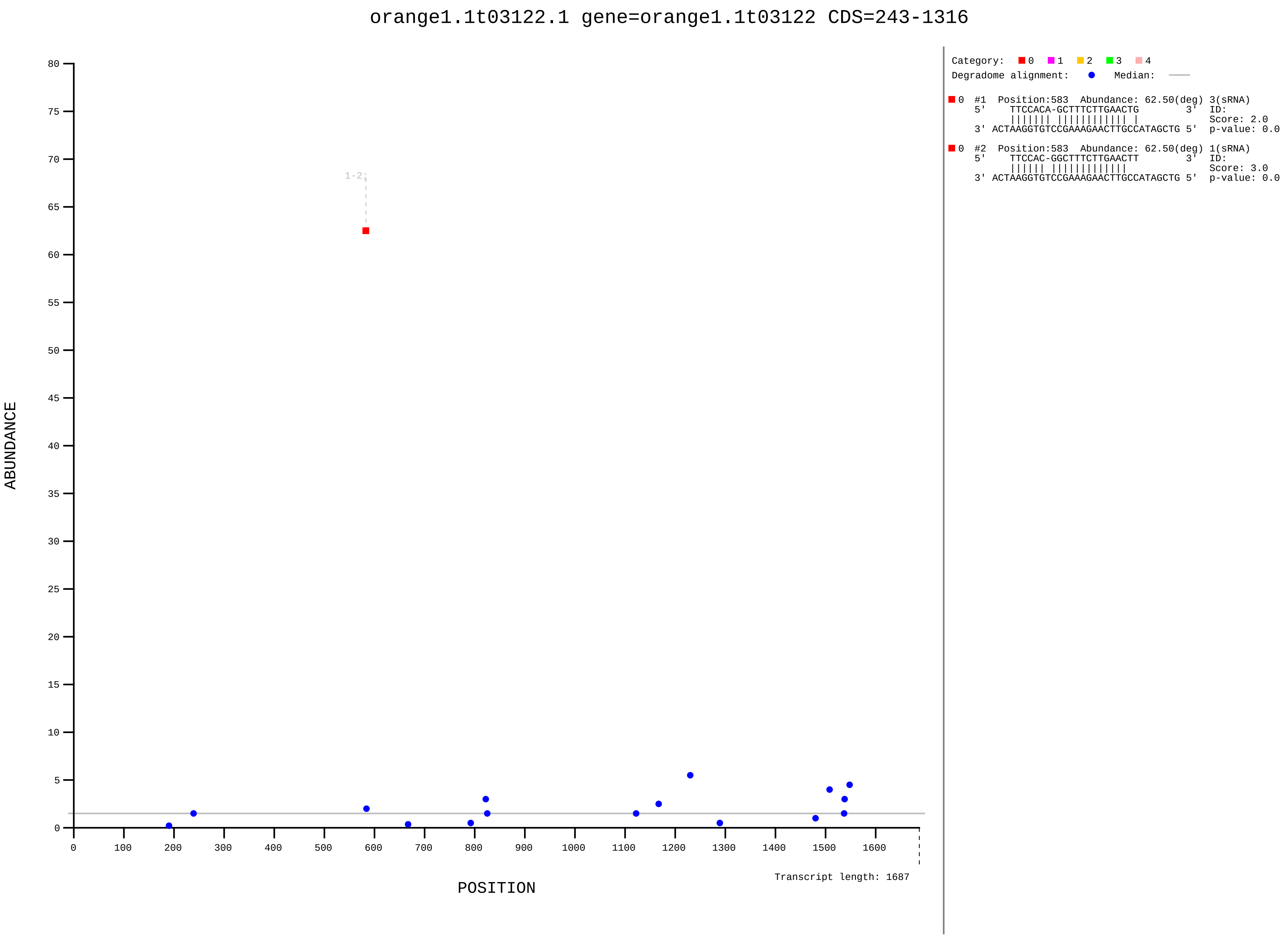
orange1.1t03122.1 gene=orange1.1t03122 CDS=243-1316
ABUNDANCE
POSITION
Transcript length: 1687
1-2:
Category: 0 1 2 3 4
Degradome alignment: Median:
0 #1 Position:583 Abundance: 62.50(deg) 3(sRNA)
5' TTCCACA-GCTTTCTTGAACTG 3' ID:
||||||| |||||||||||| | Score: 2.0
3' ACTAAGGTGTCCGAAAGAACTTGCCATAGCTG 5' p-value: 0.0
0 #2 Position:583 Abundance: 62.50(deg) 1(sRNA)
5' TTCCAC-GGCTTTCTTGAACTT 3' ID:
|||||| ||||||||||||| Score: 3.0
3' ACTAAGGTGTCCGAAAGAACTTGCCATAGCTG 5' p-value: 0.0

orange1.1t03122.2 gene=orange1.1t03122 CDS=243-1328

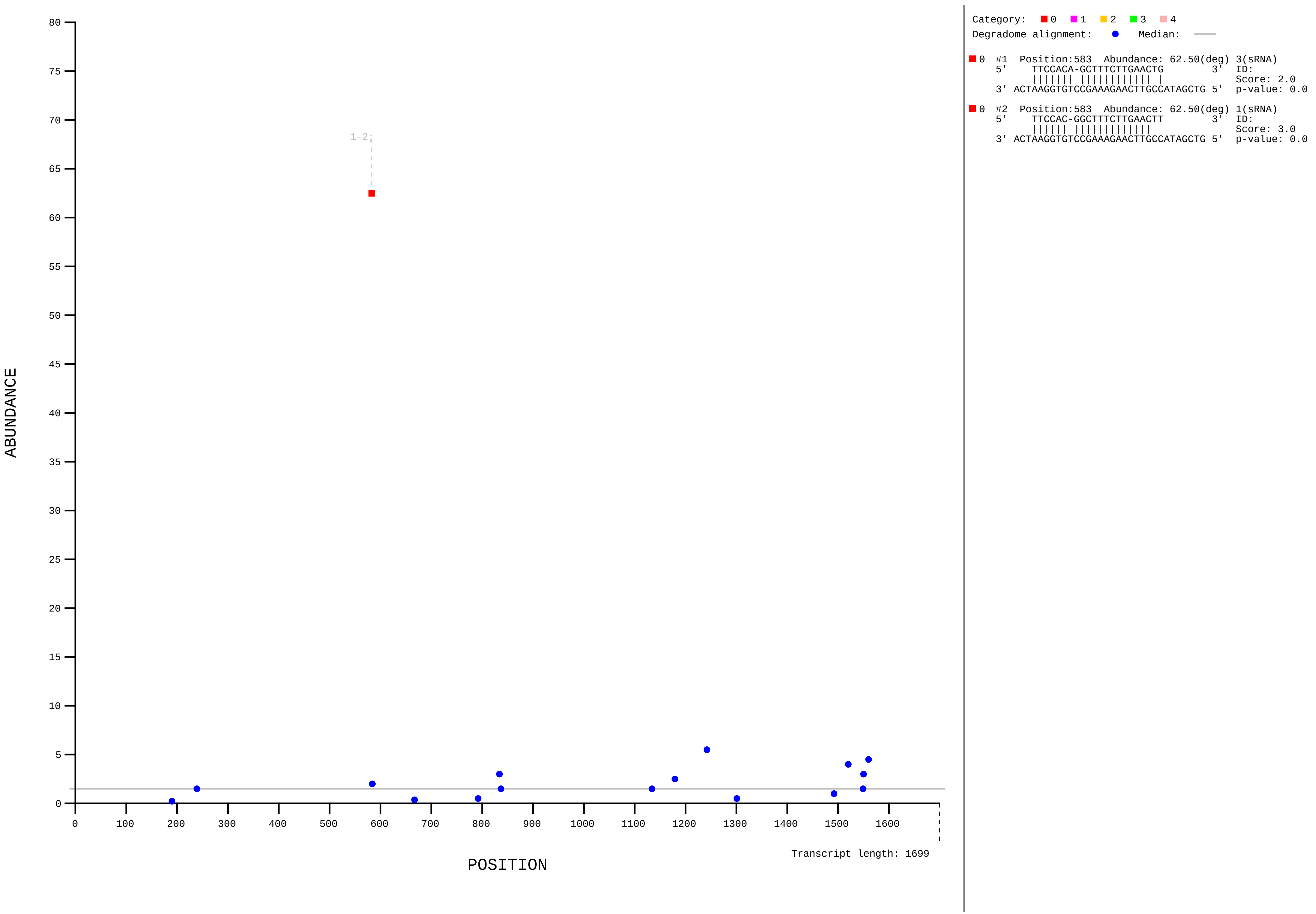

Cs1g13430.1 gene=Cs1g13430 CDS=175-2796

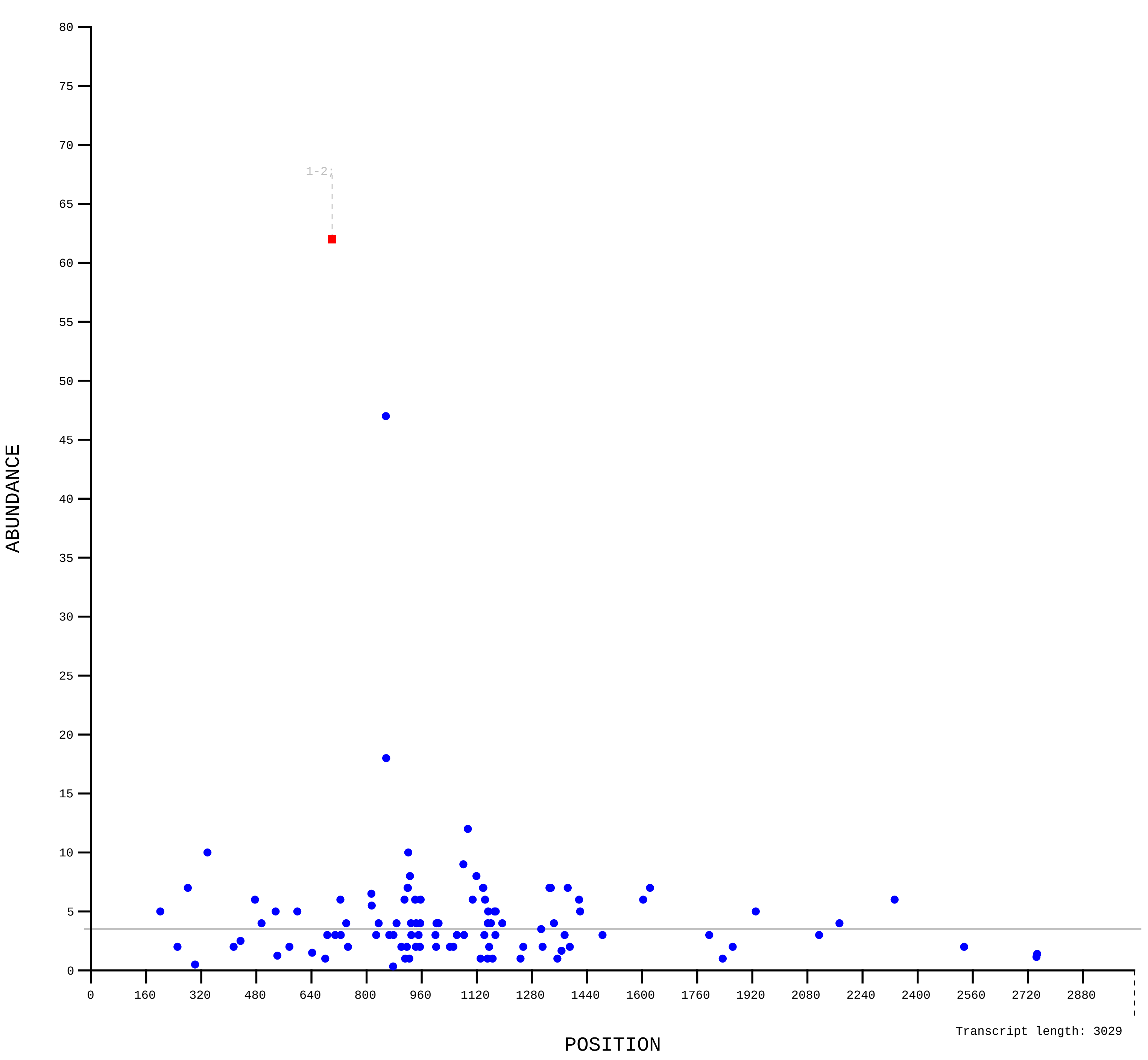

0 #1 Position:700 Abundance: 62.00(deg) 1(sRNA)

```
5'     TCTTGCCCACCCCTCCCATTCC       3'  ID:
       | ||o|||||||||||||| ||           Score: 2.5
3' CATCAAAATGGGTGGGGAGGGTACGGCATGTT 5'  p-value: 0.0
```

0 #2 Position:700 Abundance: 62.00(deg) 1(sRNA)

```
5'     TTTTTCCCACACCTCCCATCCC       3'  ID:
       |||| ||||| |||||||| ||           Score: 3.0
3' CATCAAAATGGGTGGGGAGGGTACGGCATGTT 5'  p-value: 0.0
```

Cs3g06390.1 gene=Cs3g06390 CDS=101-1471

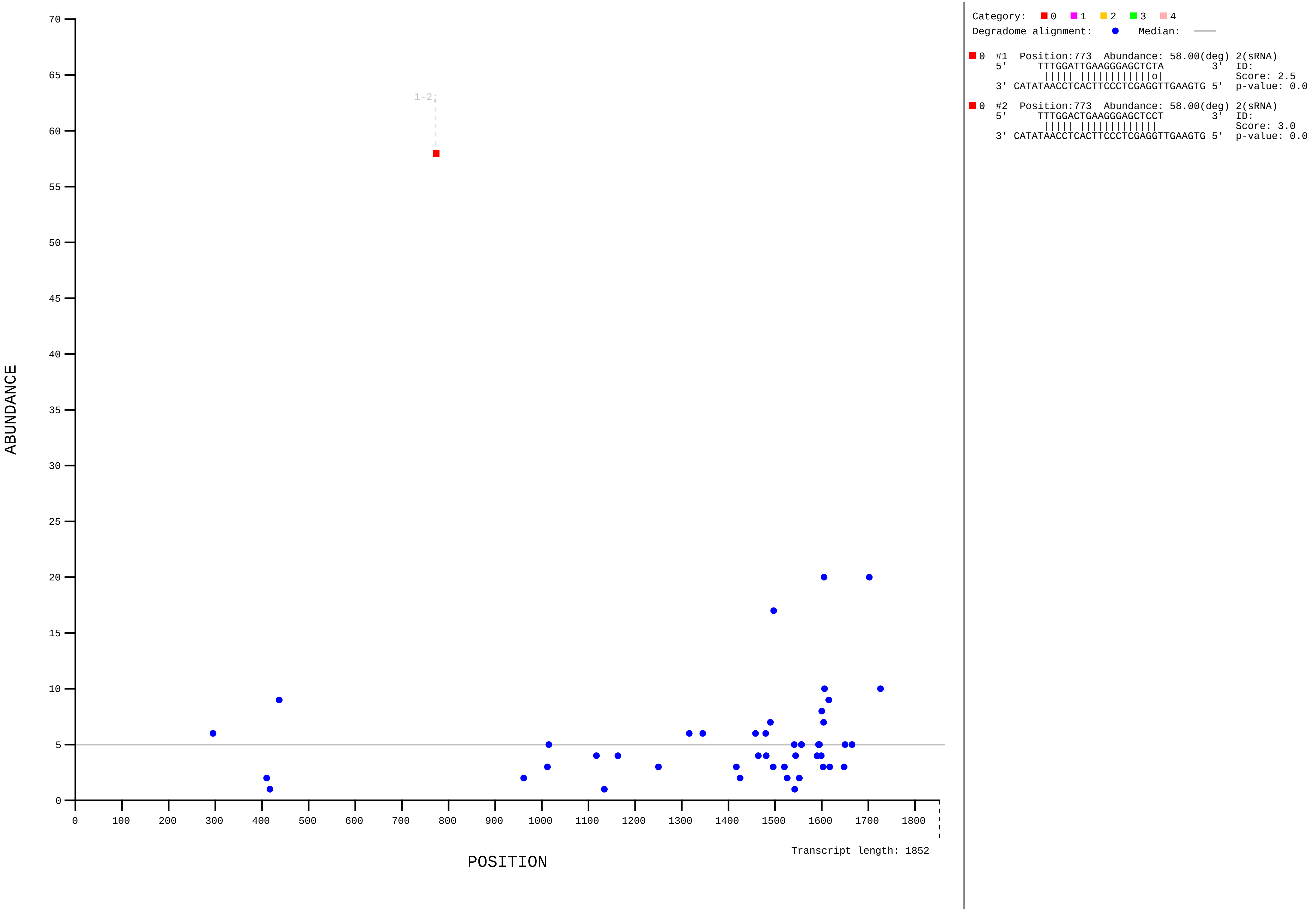

orange1.1t00149.4 gene=orange1.1t00149 CDS=30-980

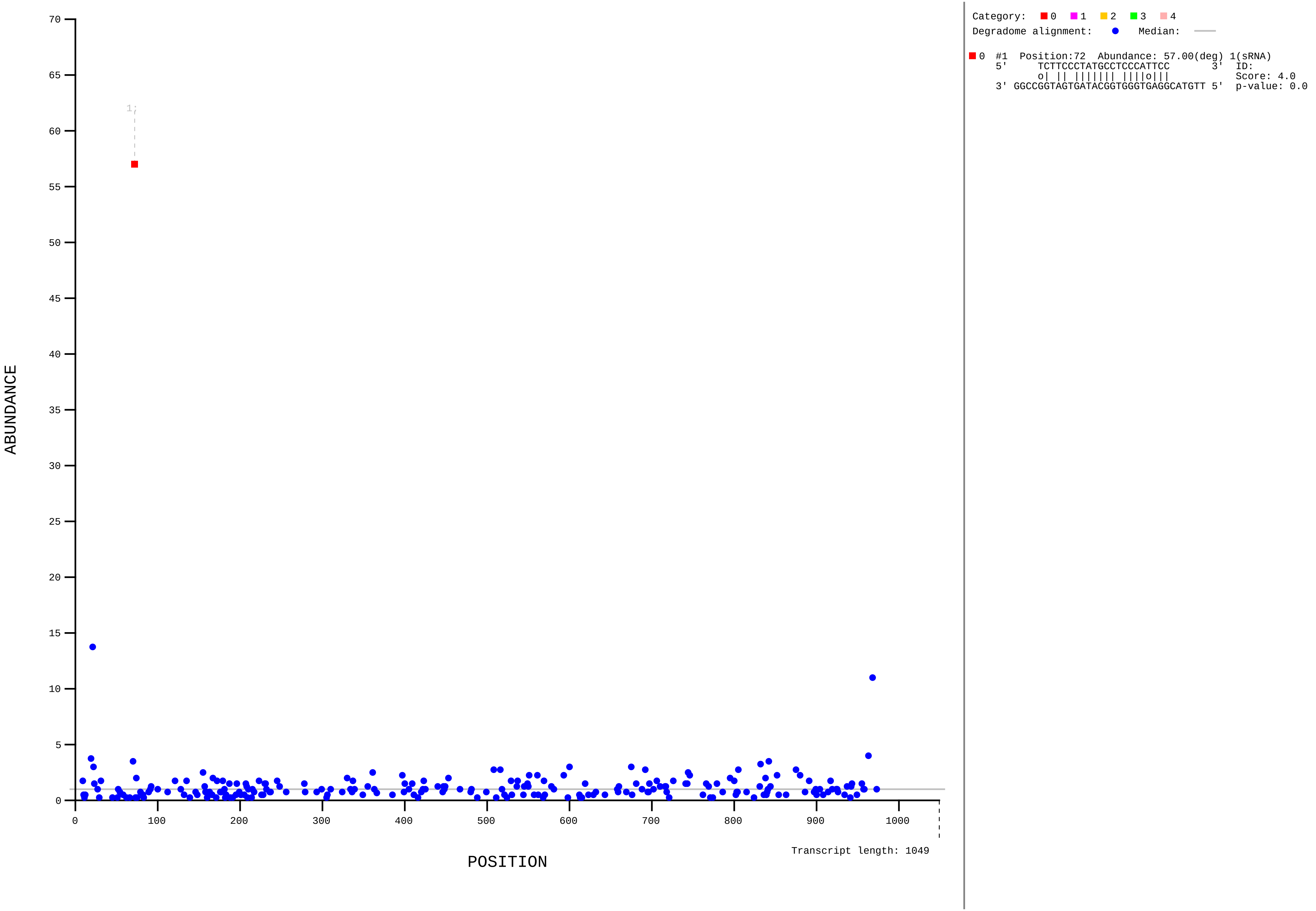

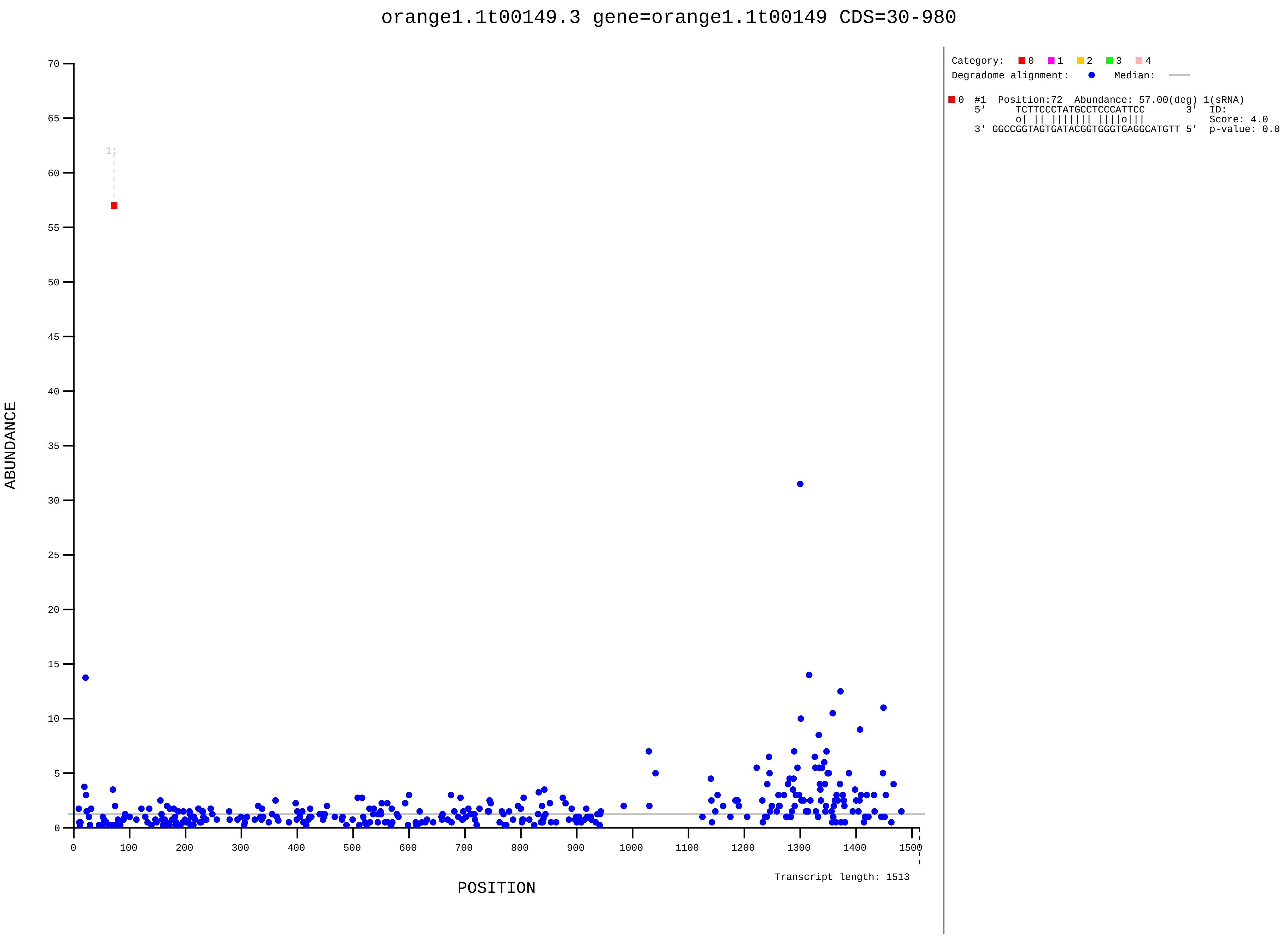

orange1.1t00149.3 gene=orange1.1t00149 CDS=30-980
ABUNDANCE
POSITION
0
5
10
15
20
25
30
35
40
45
50
55
60
65
70
0
100
200
300
400
500
600
700
800
900
1000
1100
1200
1300
1400
1500
1
Transcript length: 1513
Category: 0 1 2 3 4
Degradome alignment:
Median:
0 #1 Position:72 Abundance: 57.00(deg) 1(sRNA)
5' TCTTCCCTATGCCTCCCATTCC 3' ID:
o| || ||||||| ||||o||| Score: 4.0
3' GGCCGGTAGTGATACGGTGGGTGAGGCATGTT 5' p-value: 0.0

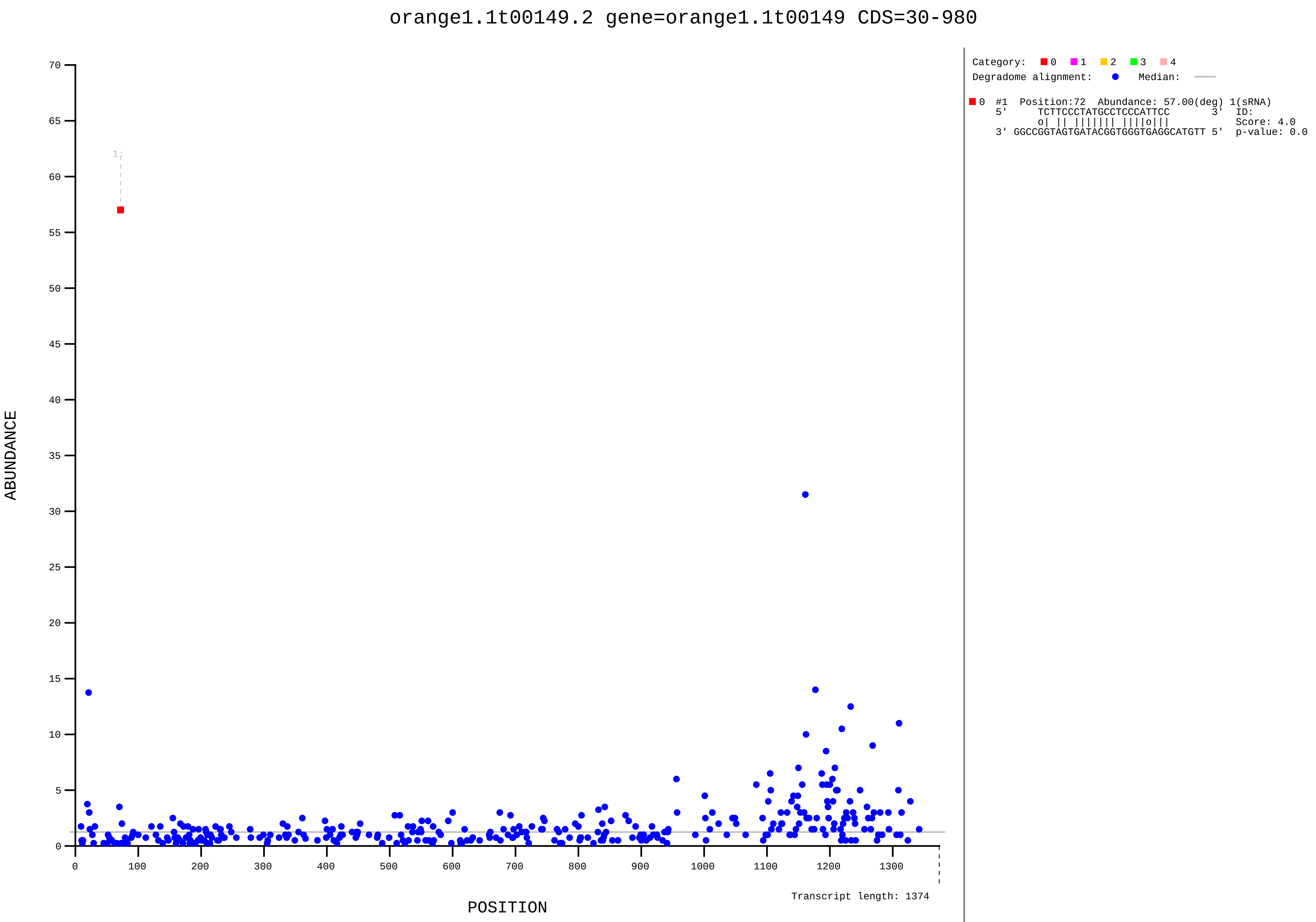

orange1.1t00149.2 gene=orange1.1t00149 CDS=30-980
Category: 0 1 2 3 4
Degradome alignment:
Median:
0 #1 Position:72 Abundance: 57.00(deg) 1(sRNA)
5' TCTTCCCTATGCCTCCCATTCC 3' ID:
o| || ||||||| ||||o|||
Score: 4.0
3' GGCCGGTAGTGATACGGTGGGTGAGGCATGTT 5' p-value: 0.0
ABUNDANCE
POSITION
Transcript length: 1374

orange1.1t00149.1 gene=orange1.1t00149 CDS=58-1011

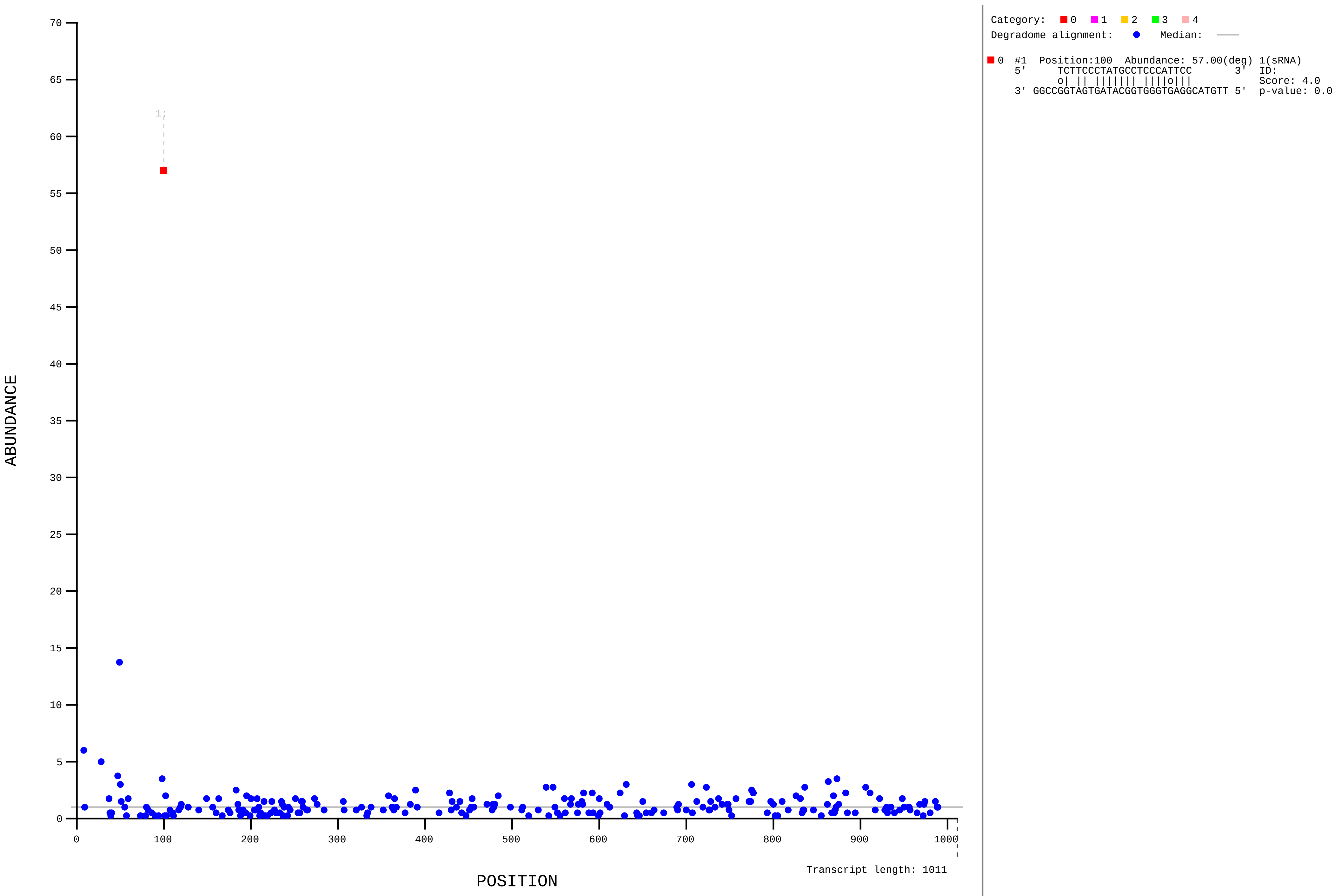

Cs7g25670.2 gene=Cs7g25670 CDS=375-2459

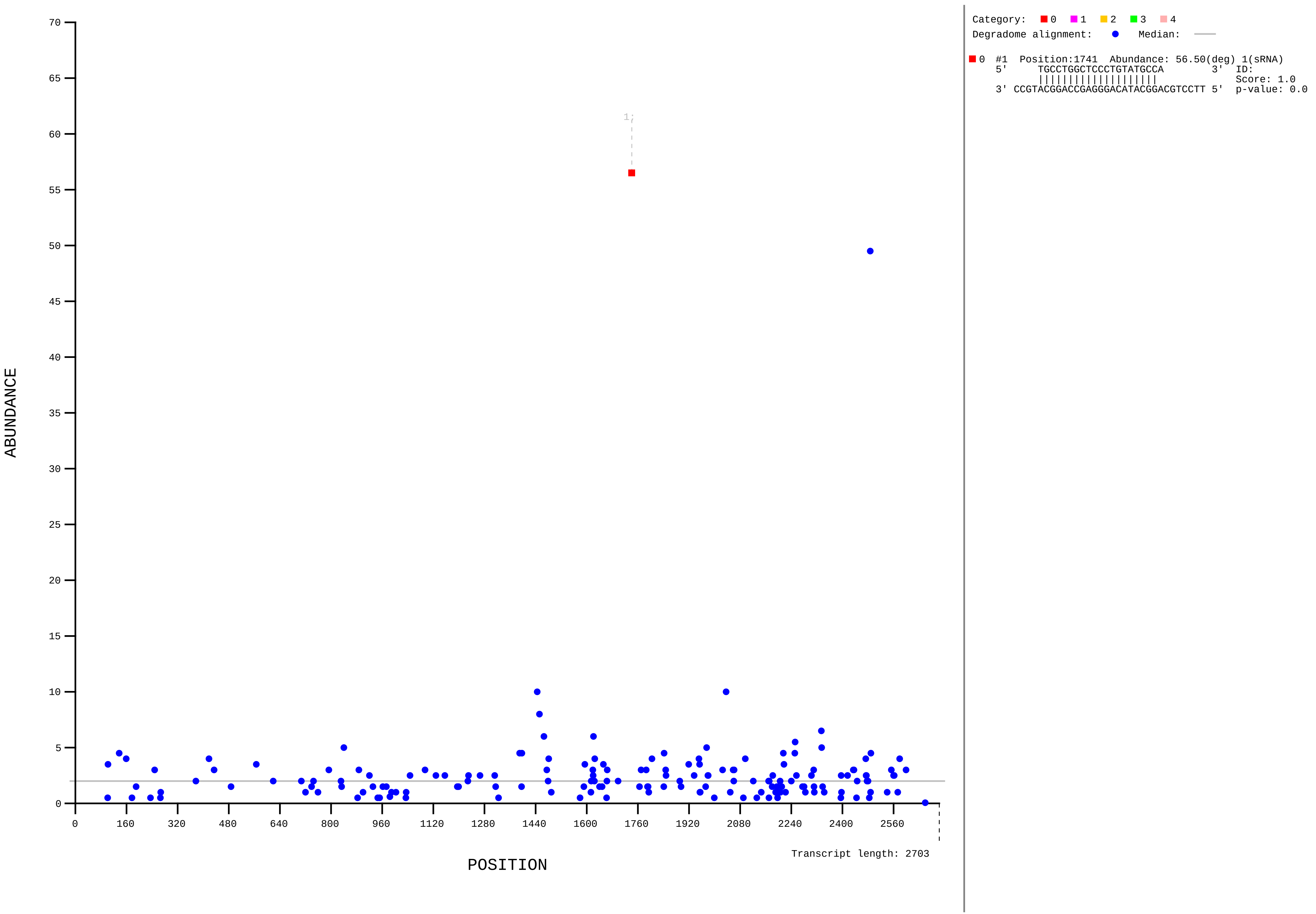

Cs7g25670.1 gene=Cs7g25670 CDS=375-2411

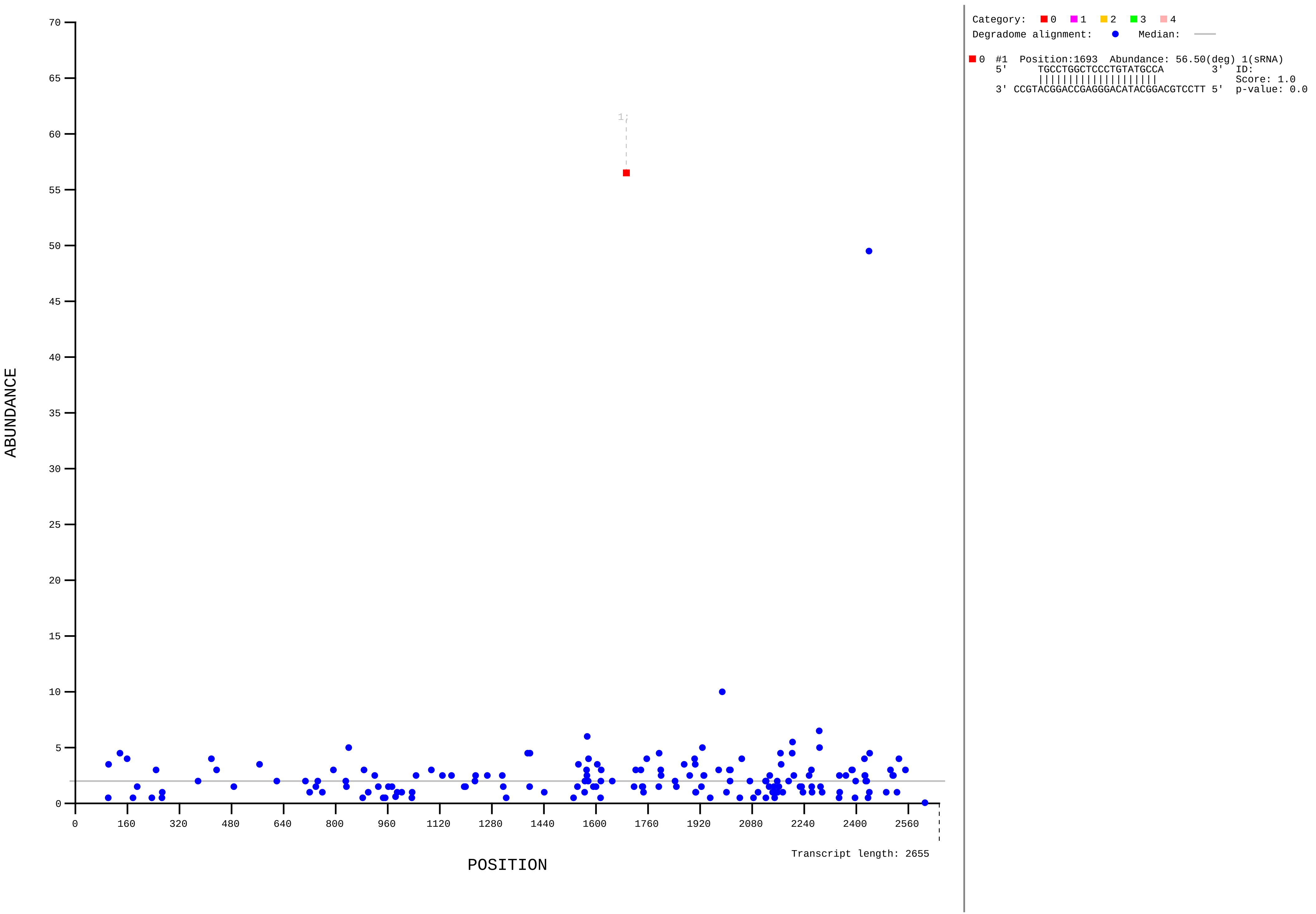

Cs2g15130.2 gene=Cs2g15130 CDS=135-2582

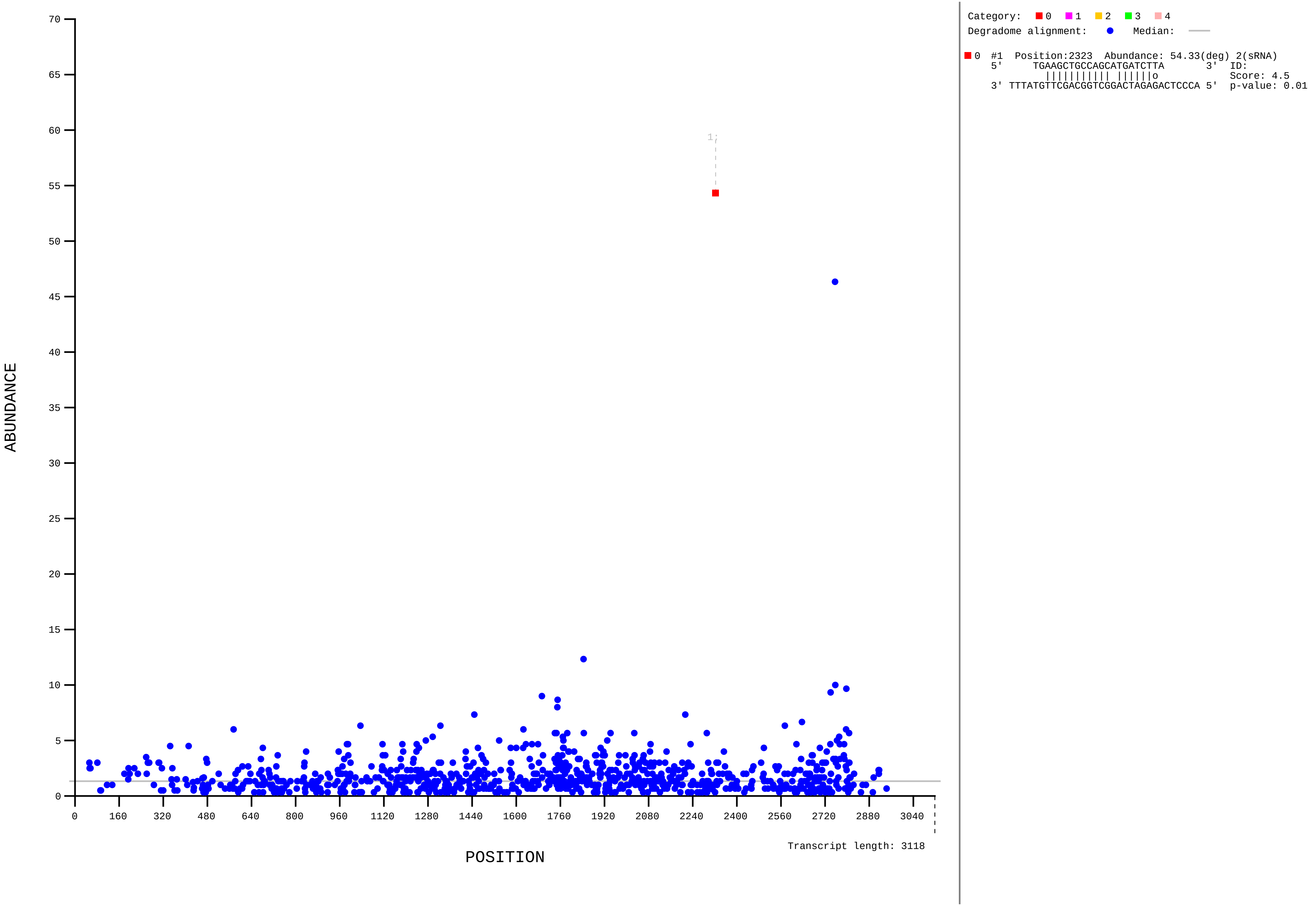

Cs2g15130.1 gene=Cs2g15130 CDS=1007-3703

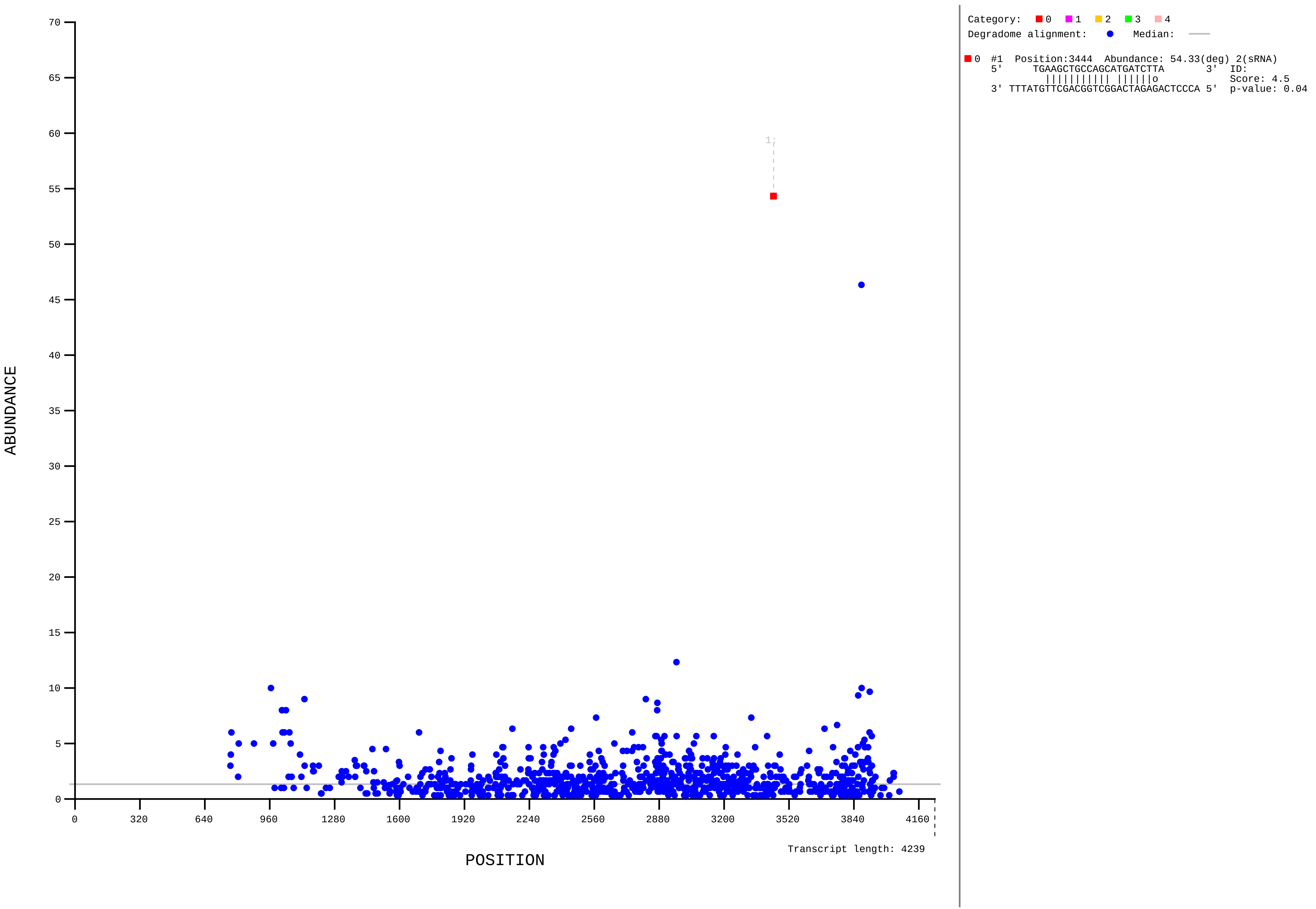

Cs2g15130.3 gene=Cs2g15130 CDS=243-2234

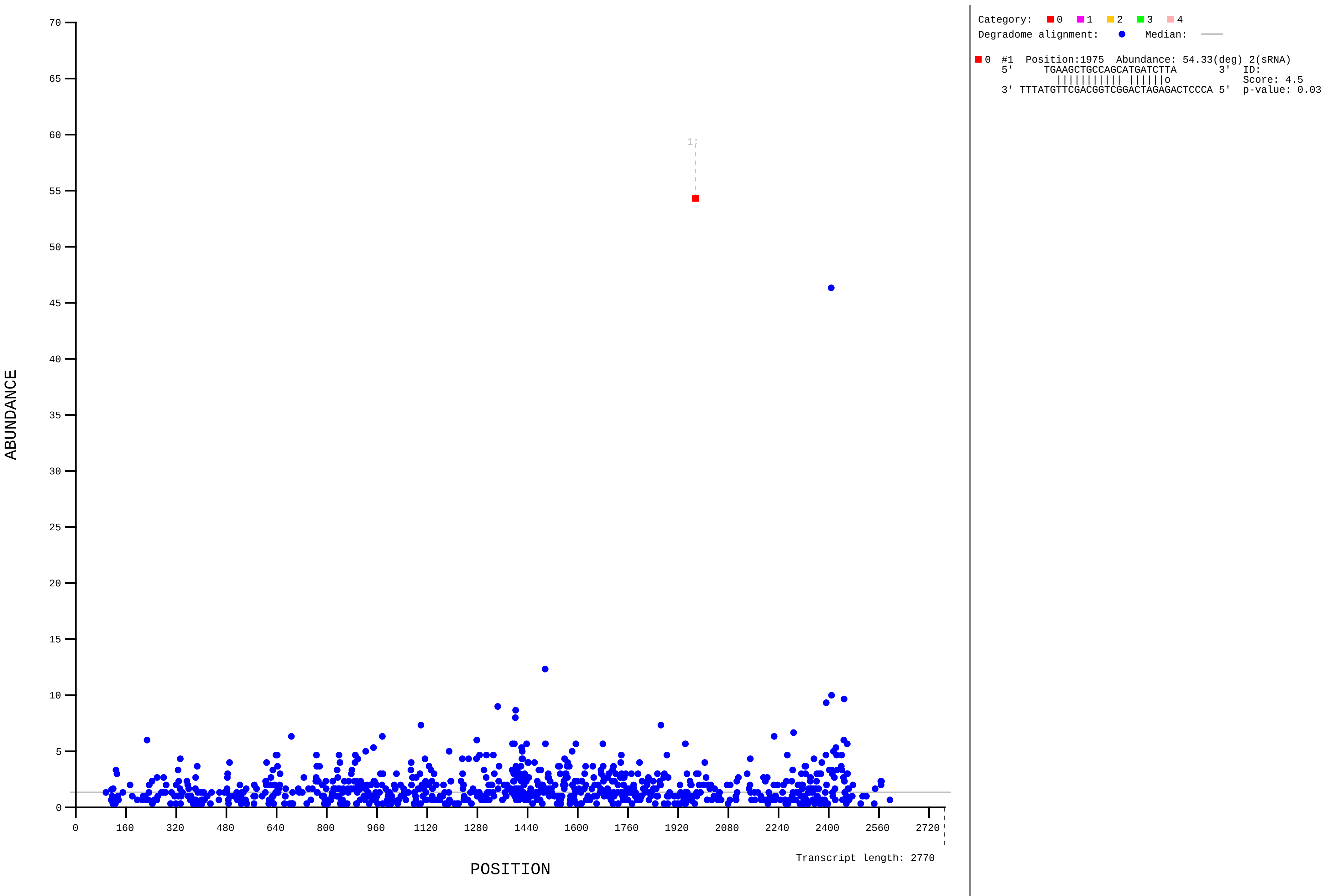

Cs7g22460.9 gene=Cs7g22460 CDS=116-628

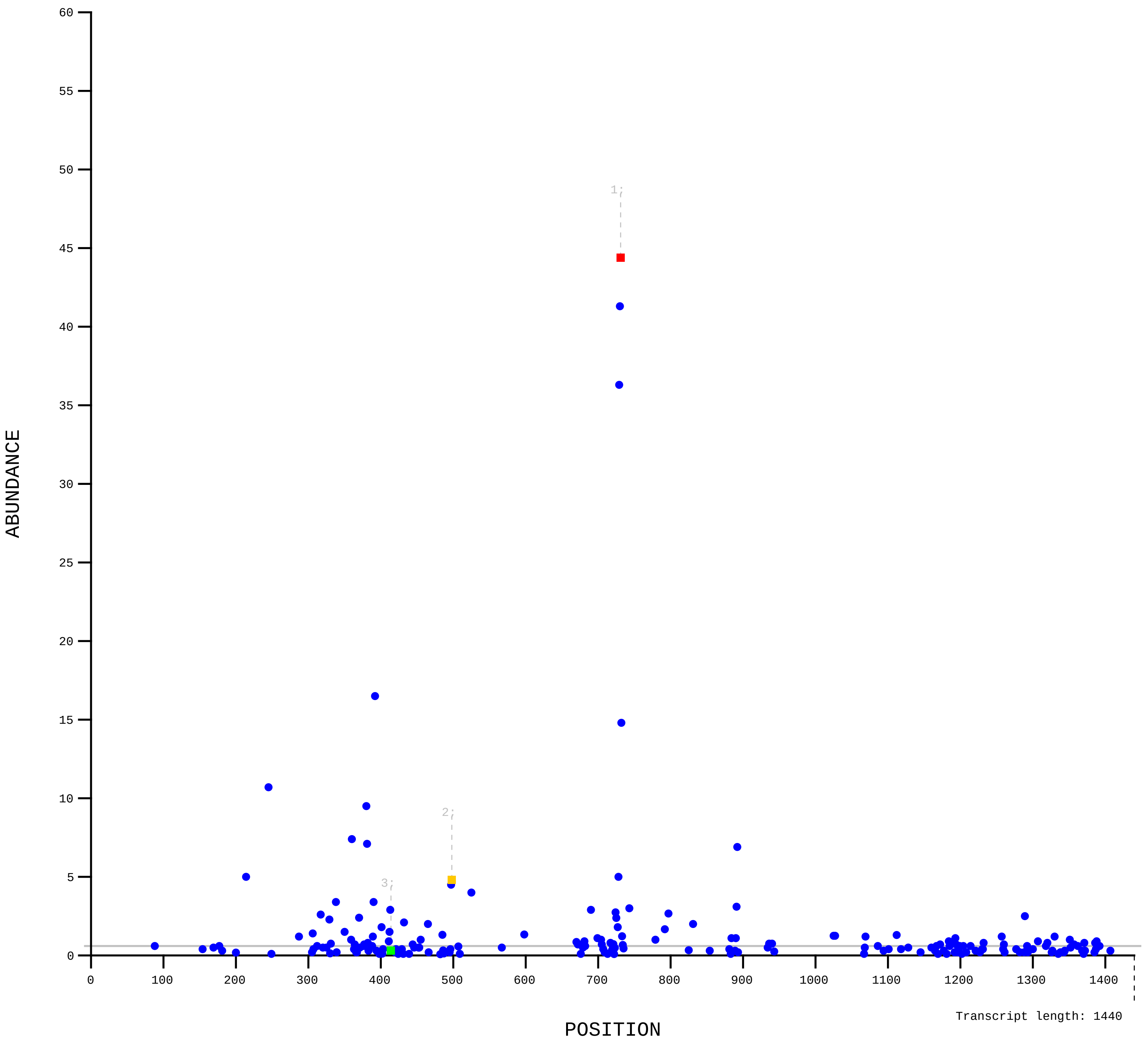

Category: 0 1 2 3 4
Degradome alignment: Median:

0 #1 Position:731 Abundance: 44.40(deg) 1(sRNA)
```
5'     TTTTTCGGCAACATGATTTCT        3'  ID:
        ||||| ||||||||||||||            Score: 2.0
3' TTCATAAAAGACGTTGTACTAAAGATATCACG 5'  p-value: 0.0
```

2 #2 Position:498 Abundance: 4.80(deg) 1(sRNA)
```
5'     TTTTTCGGCAACATGATTTCT        3'  ID:
       |||||| ||||||||||||||            Score: 1.0
3' GTTAAAAAAGACGTTGTACTAAAGATCTTGGT 5'  p-value: 0.0
```

3 #3 Position:414 Abundance: 0.30(deg) 1(sRNA)
```
5'     TTTTTCGGCAACATGATTTCT        3'  ID:
        o|||| ||||||||||||||            Score: 2.5
3' TATGCGAAAGACGTTGTACTAAAGATGCAATA 5'  p-value: 0.04
```

Cs7g22460.1 gene=Cs7g22460 CDS=116-601

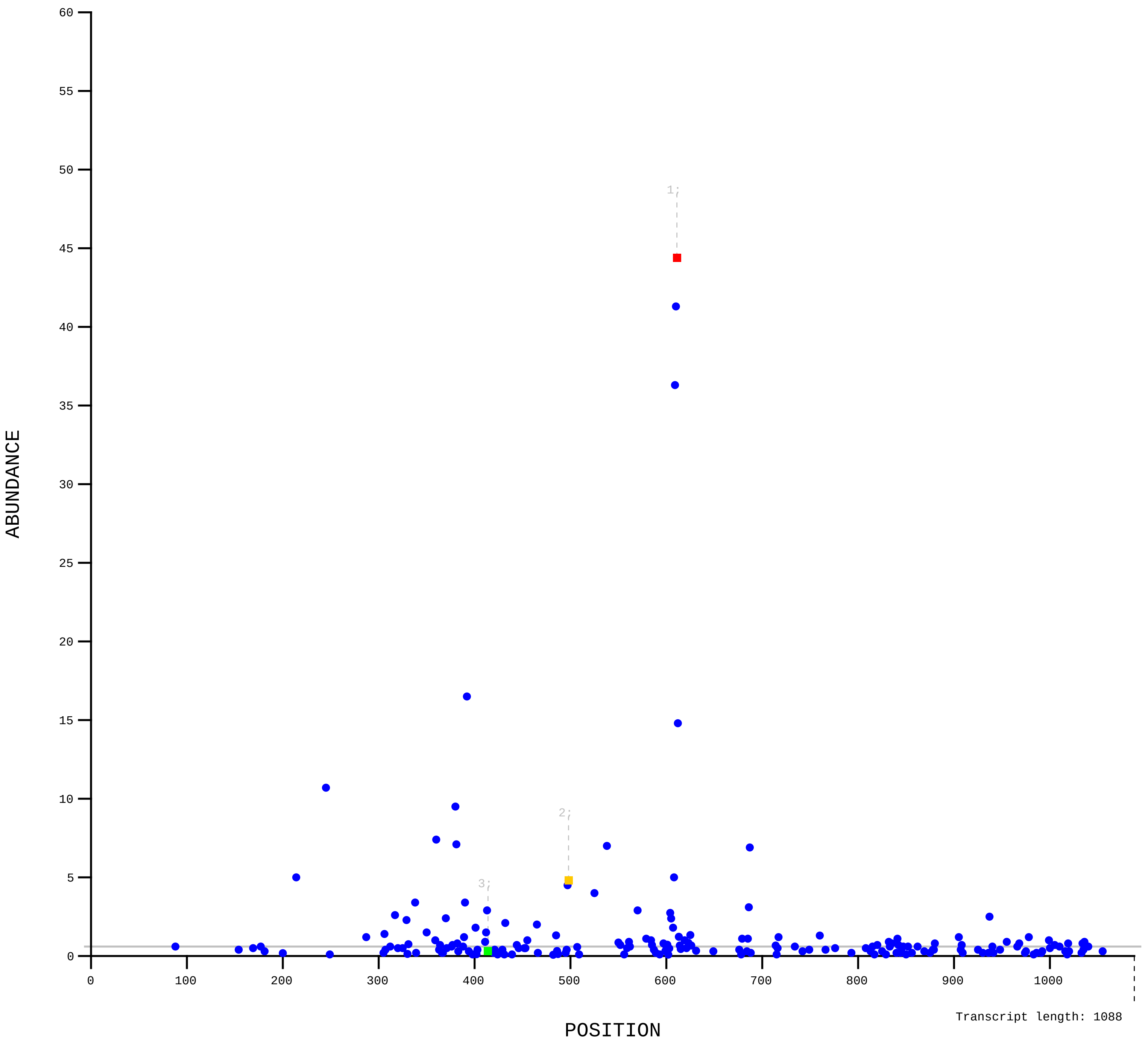


```
Category:  0  1  2  3  4
Degradome alignment:     Median:

0  #1  Position:611  Abundance: 44.40(deg) 1(sRNA)
   5'     TTTTTCGGCAACATGATTTCT        3'  ID:
           ||||| ||||||||||||||            Score: 2.0
   3' TTCATAAAAGACGTTGTACTAAAGATATCACG 5'  p-value: 0.0

2  #2  Position:498  Abundance: 4.80(deg) 1(sRNA)
   5'     TTTTTCGGCAACATGATTTCT        3'  ID:
          |||||| ||||||||||||||            Score: 1.0
   3' GTTAAAAAAGACGTTGTACTAAAGATCTTGGT 5'  p-value: 0.0

3  #3  Position:414  Abundance: 0.30(deg) 1(sRNA)
   5'     TTTTTCGGCAACATGATTTCT        3'  ID:
           o|||| ||||||||||||||            Score: 2.5
   3' TATGCGAAAGACGTTGTACTAAAGATGCAATA 5'  p-value: 0.04
```

Cs7g22460.5 gene=Cs7g22460 CDS=116-580

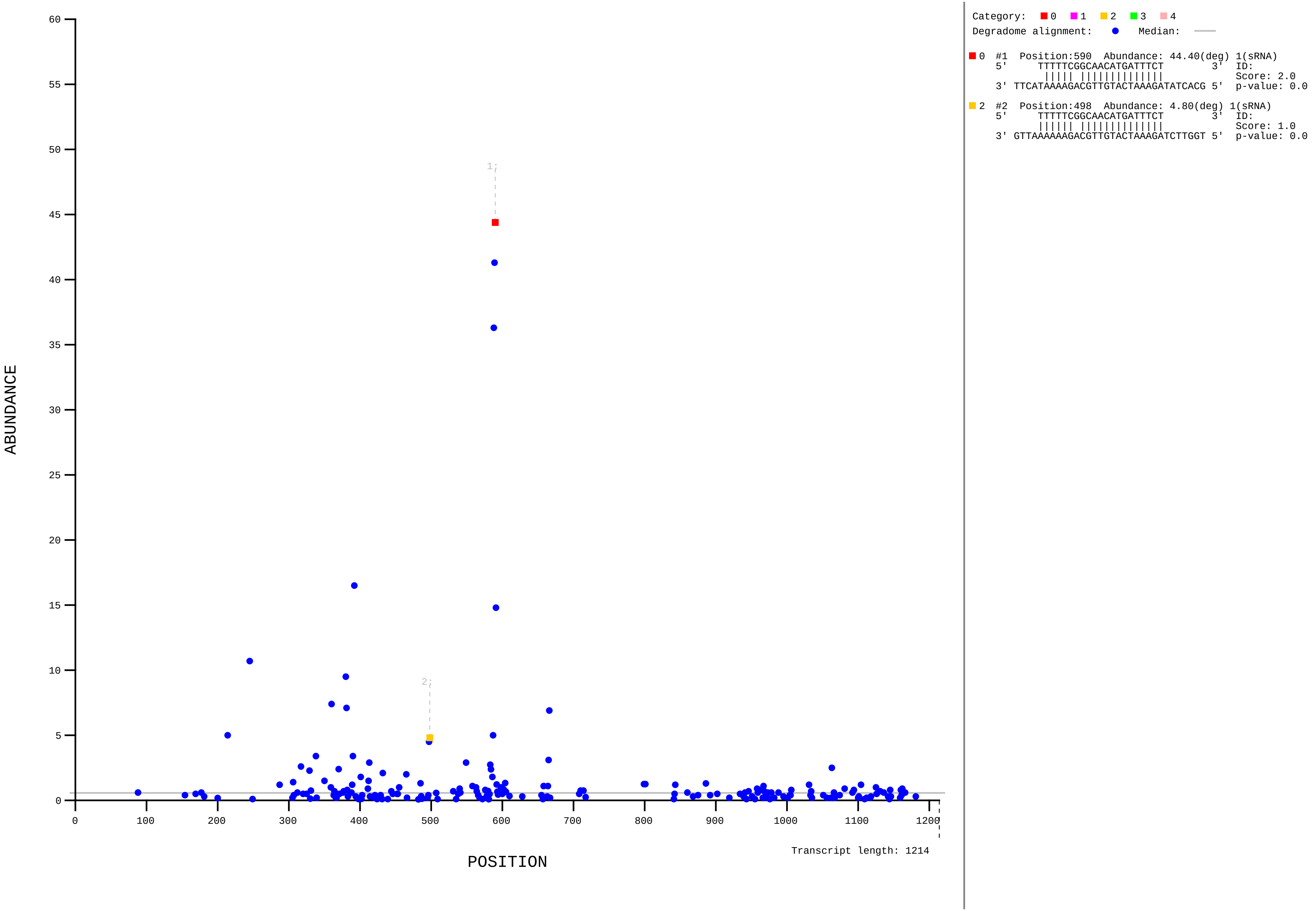

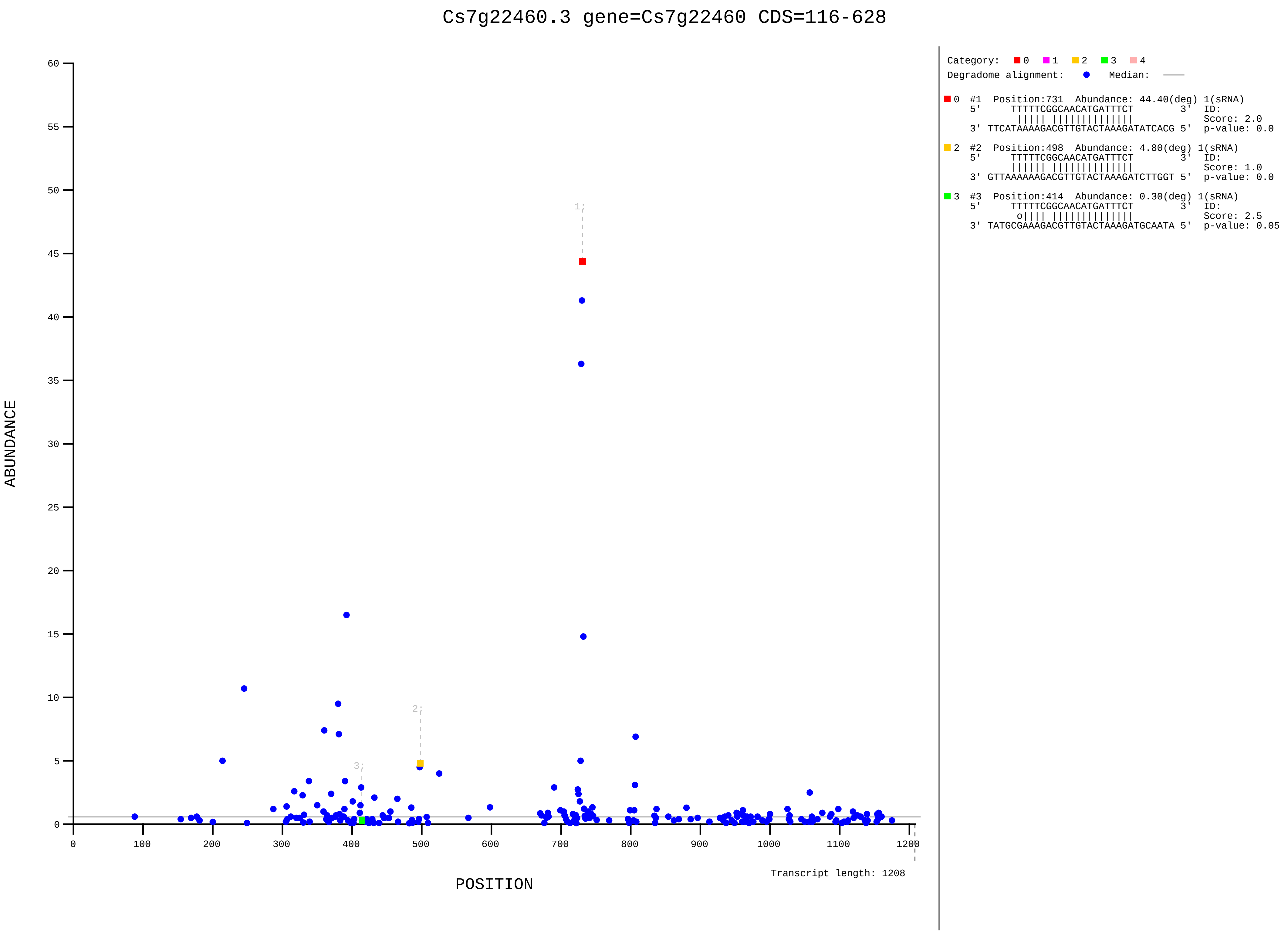

Cs7g22460.3 gene=Cs7g22460 CDS=116-628
ABUNDANCE
POSITION
Transcript length: 1208
Category: 0 1 2 3 4
Degradome alignment:
Median:
0 #1 Position:731 Abundance: 44.40(deg) 1(sRNA)
5' TTTTTCGGCAACATGATTTCT 3' ID:
||||| ||||||||||||||| Score: 2.0
3' TTCATAAAAGACGTTGTACTAAAGATATCACG 5' p-value: 0.0
2 #2 Position:498 Abundance: 4.80(deg) 1(sRNA)
5' TTTTTCGGCAACATGATTTCT 3' ID:
|||||| ||||||||||||||| Score: 1.0
3' GTTAAAAAAGACGTTGTACTAAAGATCTTGGT 5' p-value: 0.0
3 #3 Position:414 Abundance: 0.30(deg) 1(sRNA)
5' TTTTTCGGCAACATGATTTCT 3' ID:
o|||| ||||||||||||||| Score: 2.5
3' TATGCGAAAGACGTTGTACTAAAGATGCAATA 5' p-value: 0.05

Cs7g22460.10 gene=Cs7g22460 CDS=116-610

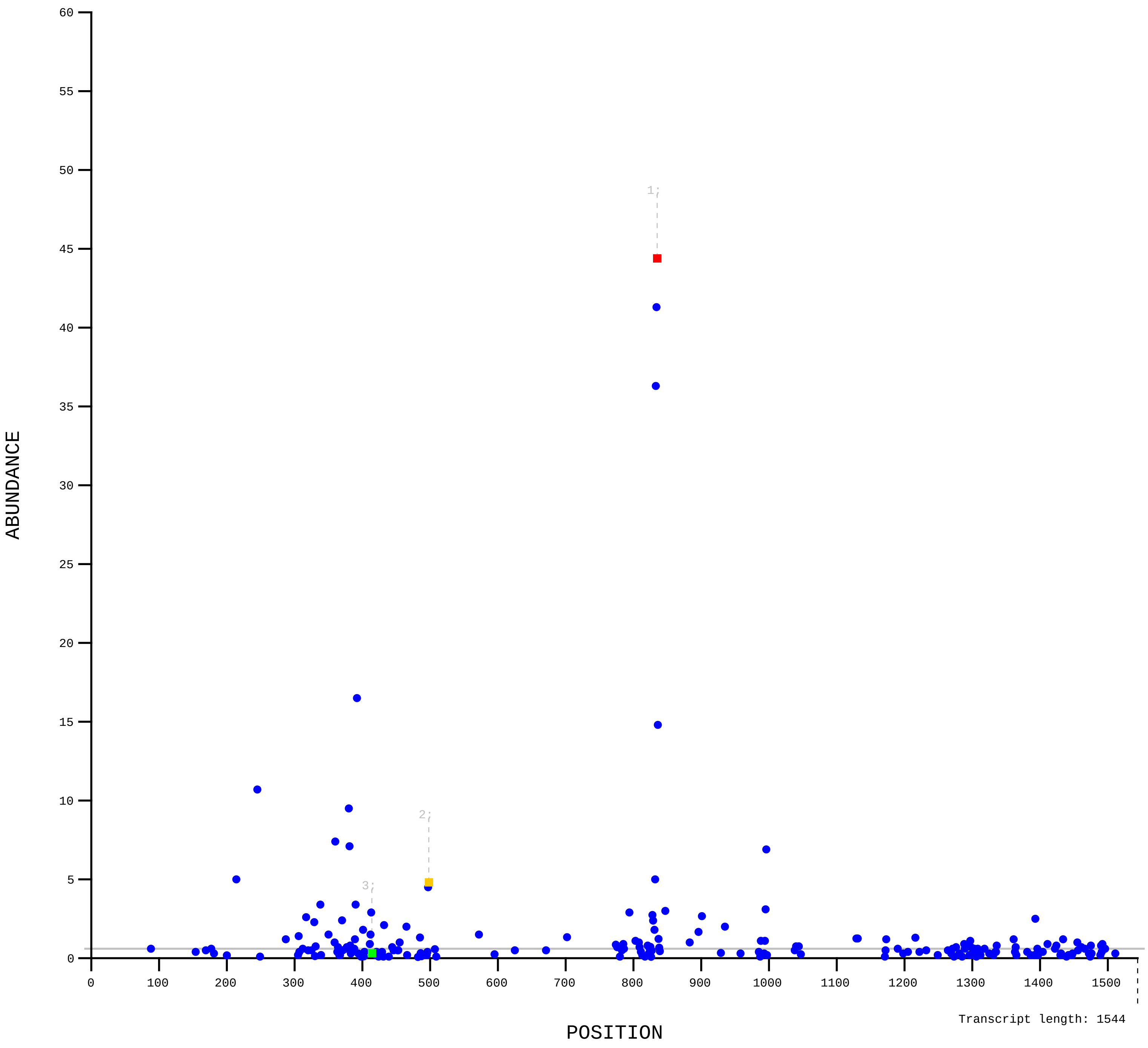


```
0  #1  Position:835  Abundance: 44.40(deg) 1(sRNA)
   5'     TTTTTCGGCAACATGATTTCT        3'  ID:
            ||||| ||||||||||||||           Score: 2.0
   3' TTCATAAAAGACGTTGTACTAAAGATATCACG 5'  p-value: 0.0

2  #2  Position:498  Abundance: 4.80(deg) 1(sRNA)
   5'     TTTTTCGGCAACATGATTTCT        3'  ID:
          |||||| ||||||||||||||            Score: 1.0
   3' GTTAAAAAAGACGTTGTACTAAAGATCTTGGT 5'  p-value: 0.0

3  #3  Position:414  Abundance: 0.30(deg) 1(sRNA)
   5'     TTTTTCGGCAACATGATTTCT        3'  ID:
            o|||| ||||||||||||||           Score: 2.5
   3' TATGCGAAAGACGTTGTACTAAAGATGCAATA 5'  p-value: 0.03
```

Cs7g22460.2 gene=Cs7g22460 CDS=118-582

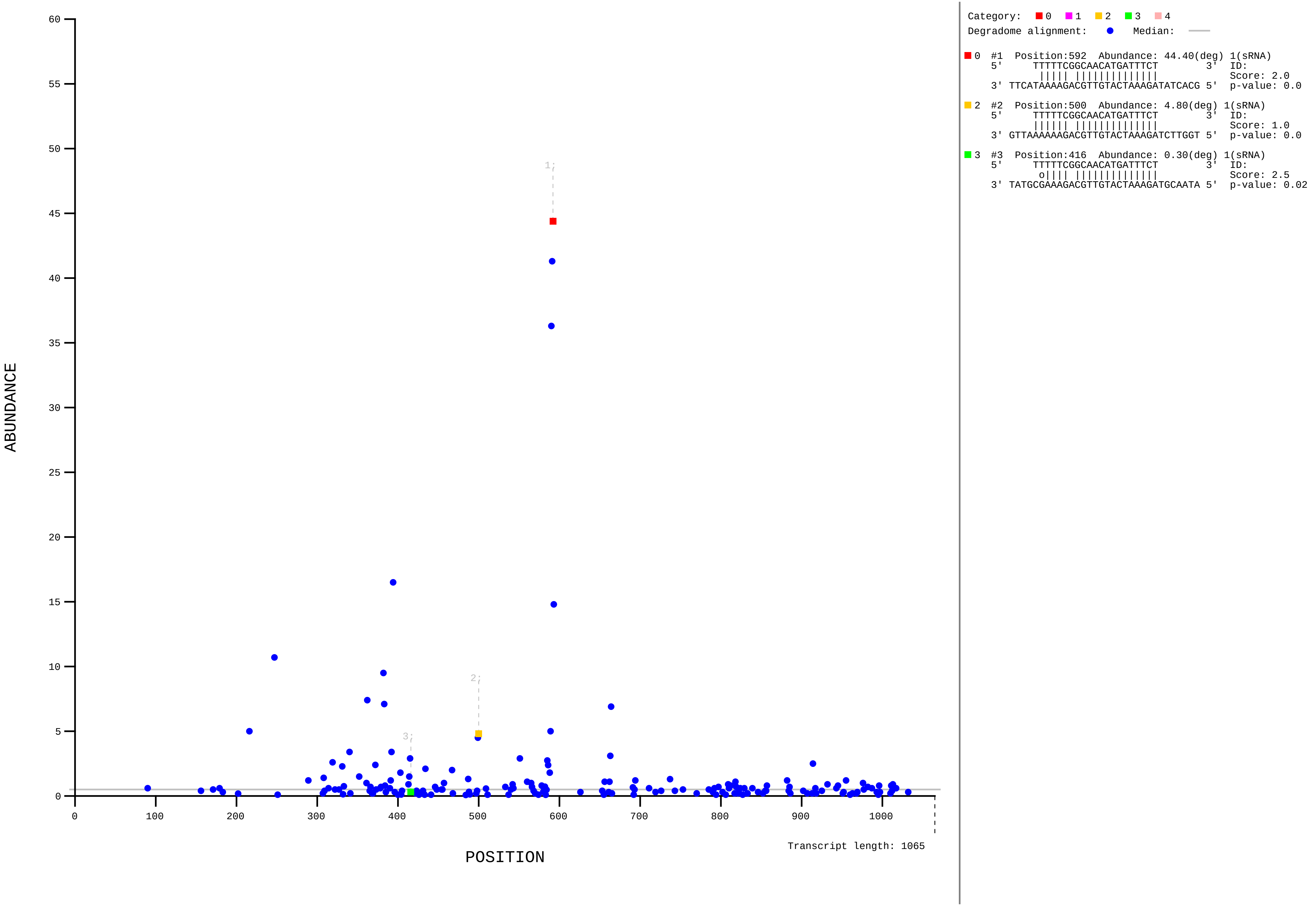

Cs7g22460.7 gene=Cs7g22460 CDS=116-610

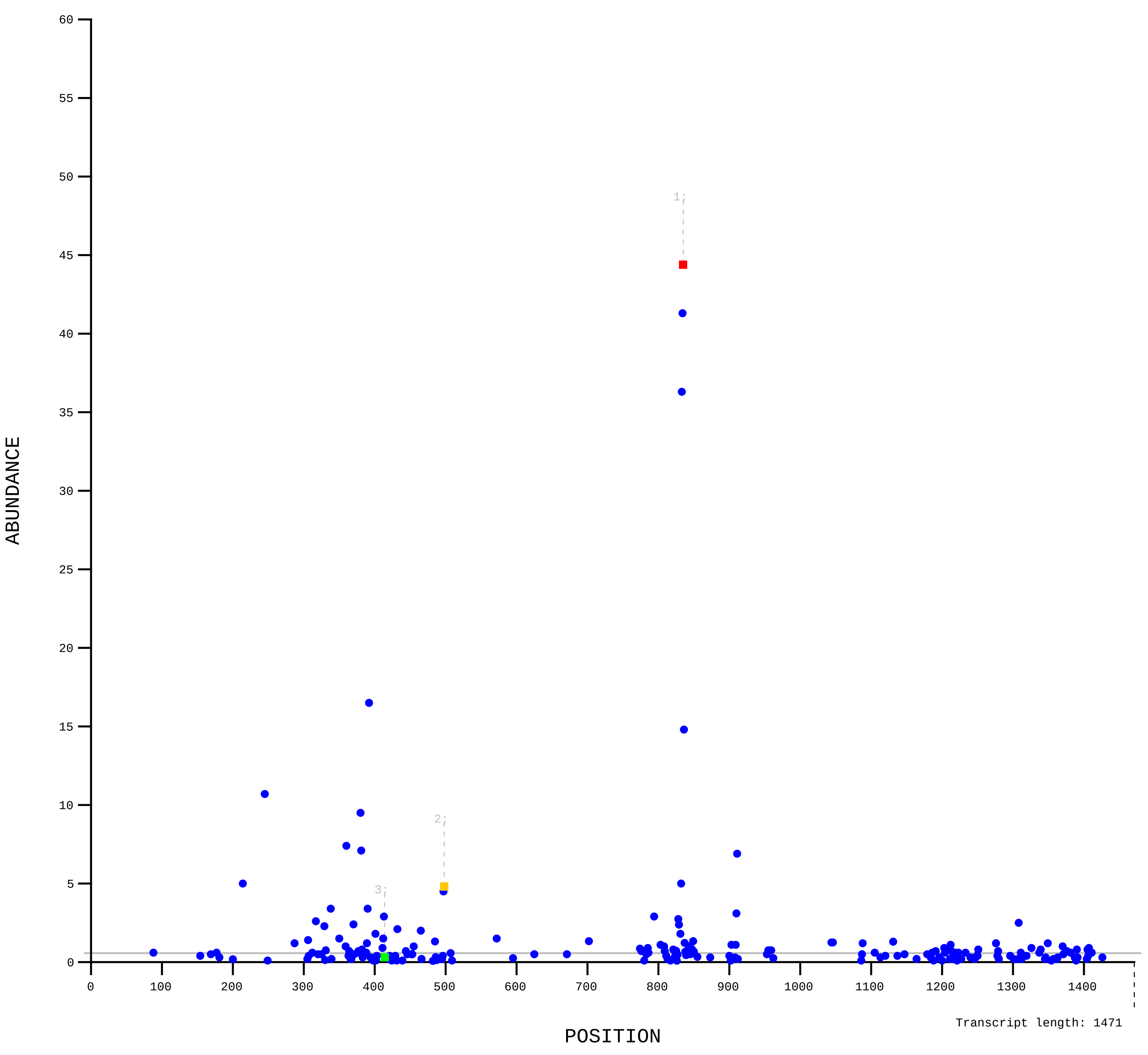


```
Category:  ■0  ■1  ■2  ■3  ■4
Degradome alignment:  ●   Median: ——

■0  #1  Position:835  Abundance: 44.40(deg) 1(sRNA)
    5'      TTTTTCGGCAACATGATTTCT        3'  ID:
             ||||| ||||||||||||||            Score: 2.0
    3' TTCATAAAAGACGTTGTACTAAAGATATCACG 5'  p-value: 0.0

■2  #2  Position:498  Abundance: 4.80(deg) 1(sRNA)
    5'      TTTTTCGGCAACATGATTTCT        3'  ID:
            |||||| ||||||||||||||            Score: 1.0
    3' GTTAAAAAAGACGTTGTACTAAAGATCTTGGT 5'  p-value: 0.0

■3  #3  Position:414  Abundance: 0.30(deg) 1(sRNA)
    5'      TTTTTCGGCAACATGATTTCT        3'  ID:
             o|||| ||||||||||||||            Score: 2.5
    3' TATGCGAAAGACGTTGTACTAAAGATGCAATA 5'  p-value: 0.04
```

Cs7g22460.6 gene=Cs7g22460 CDS=134-628

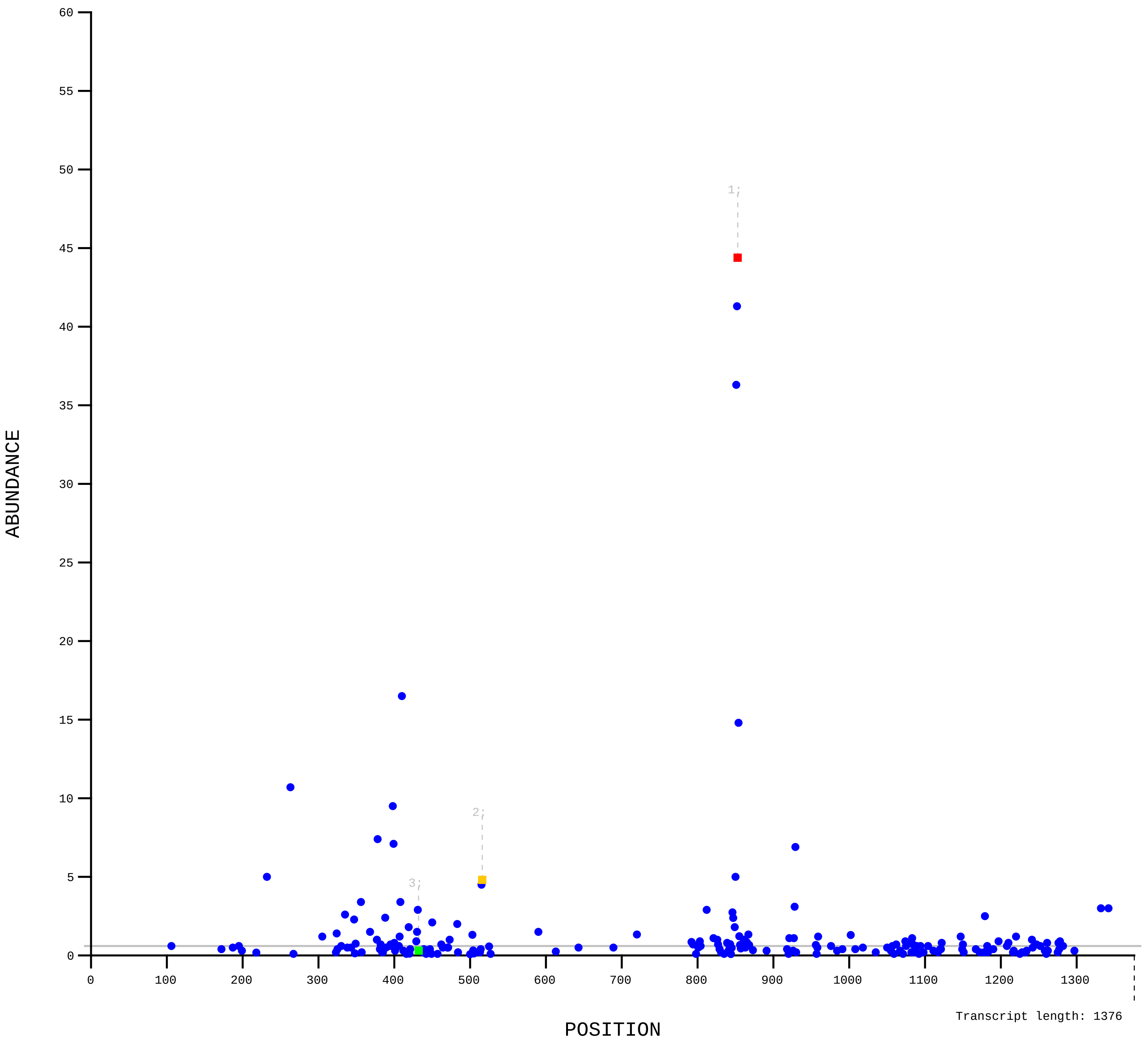

Category: 0 1 2 3 4
Degradome alignment: ● Median: —

```
0  #1  Position:853  Abundance: 44.40(deg) 1(sRNA)
   5'      TTTTTCGGCAACATGATTTCT        3'  ID:
             ||||| ||||||||||||||           Score: 2.0
   3' TTCATAAAAGACGTTGTACTAAAGATATCACG 5'  p-value: 0.0

2  #2  Position:516  Abundance: 4.80(deg) 1(sRNA)
   5'      TTTTTCGGCAACATGATTTCT        3'  ID:
           |||||| ||||||||||||||            Score: 1.0
   3' GTTAAAAAAGACGTTGTACTAAAGATCTTGGT 5'  p-value: 0.0

3  #3  Position:432  Abundance: 0.30(deg) 1(sRNA)
   5'      TTTTTCGGCAACATGATTTCT        3'  ID:
             o|||| ||||||||||||||           Score: 2.5
   3' TATGCGAAAGACGTTGTACTAAAGATGCAATA 5'  p-value: 0.03
```

Cs7g22460.4 gene=Cs7g22460 CDS=116-580

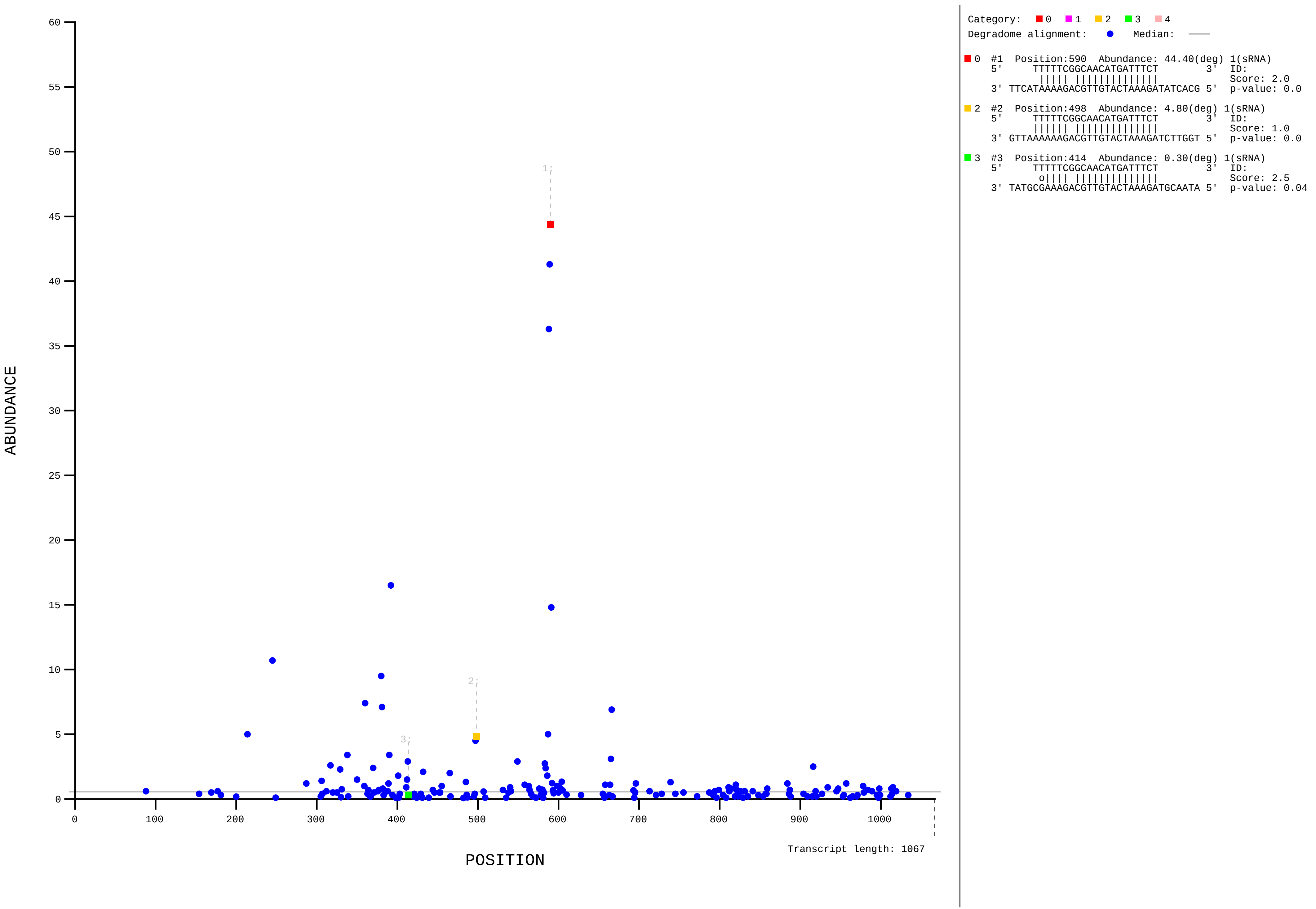

Cs7g22460.8 gene=Cs7g22460 CDS=116-610

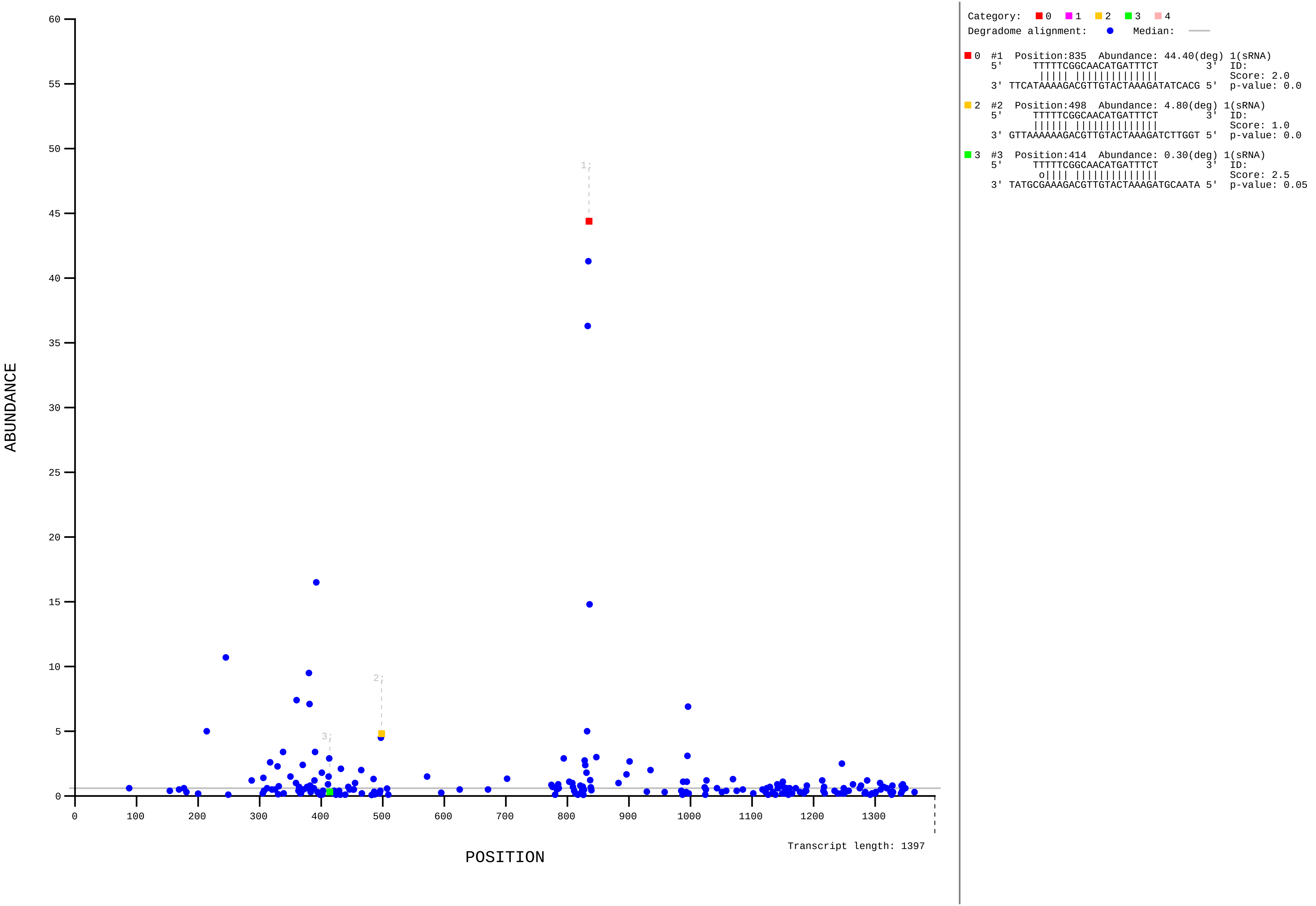

orange1.1t00584.1 gene=orange1.1t00584 CDS=1-5892

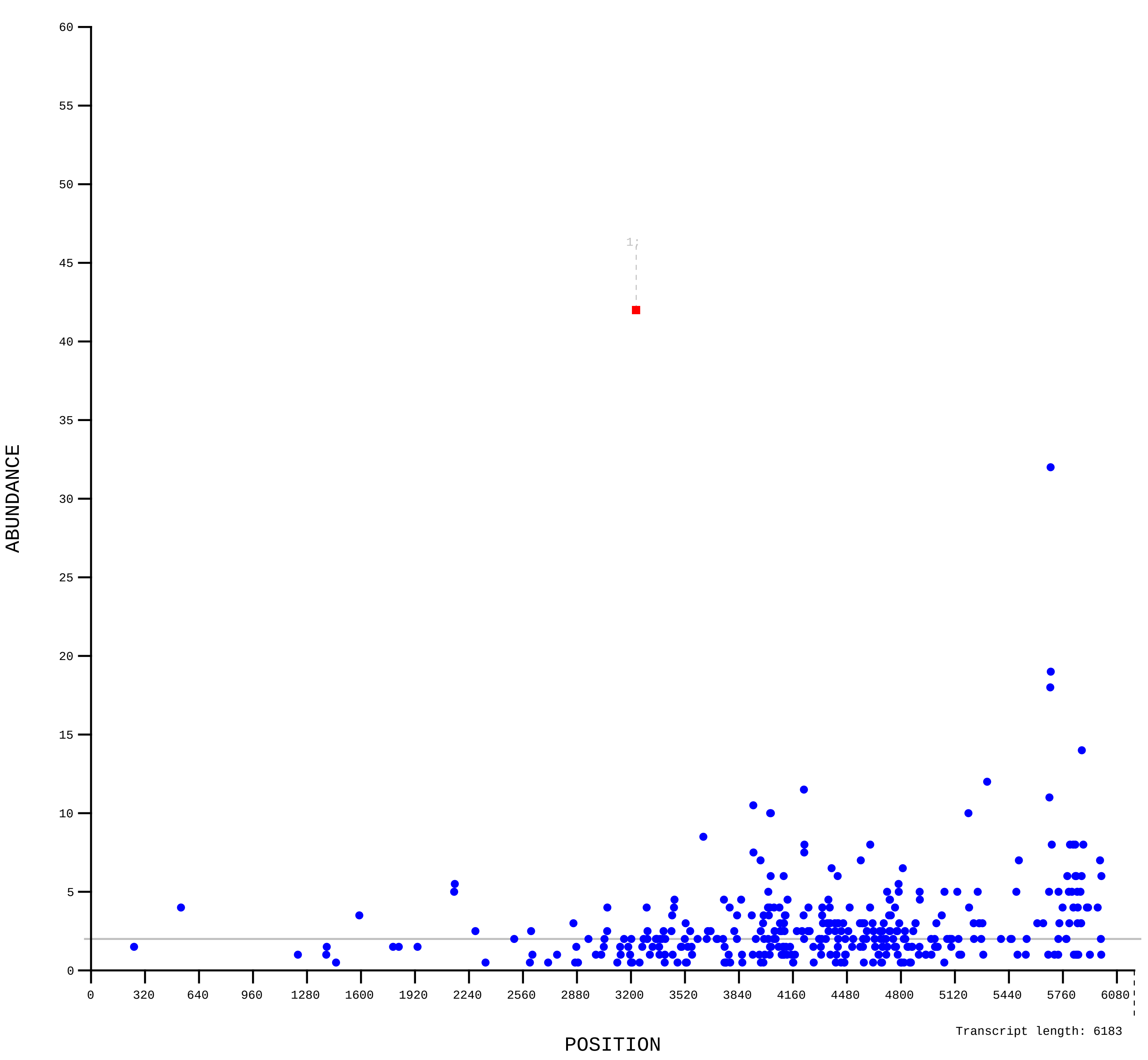

Category: 0 1 2 3 4
Degradome alignment: Median:

```
0  #1  Position:3231  Abundance: 42.00(deg) 2(sRNA)
   5'    TCGATAA-ACCTCTGCATCCAG        3'  ID:
         ||||||| ||||||||||||||            Score: 1.0
   3' TGTAGCTATTGTGGAGACGTAGGTCGAGTGAC 5'  p-value: 0.0
```

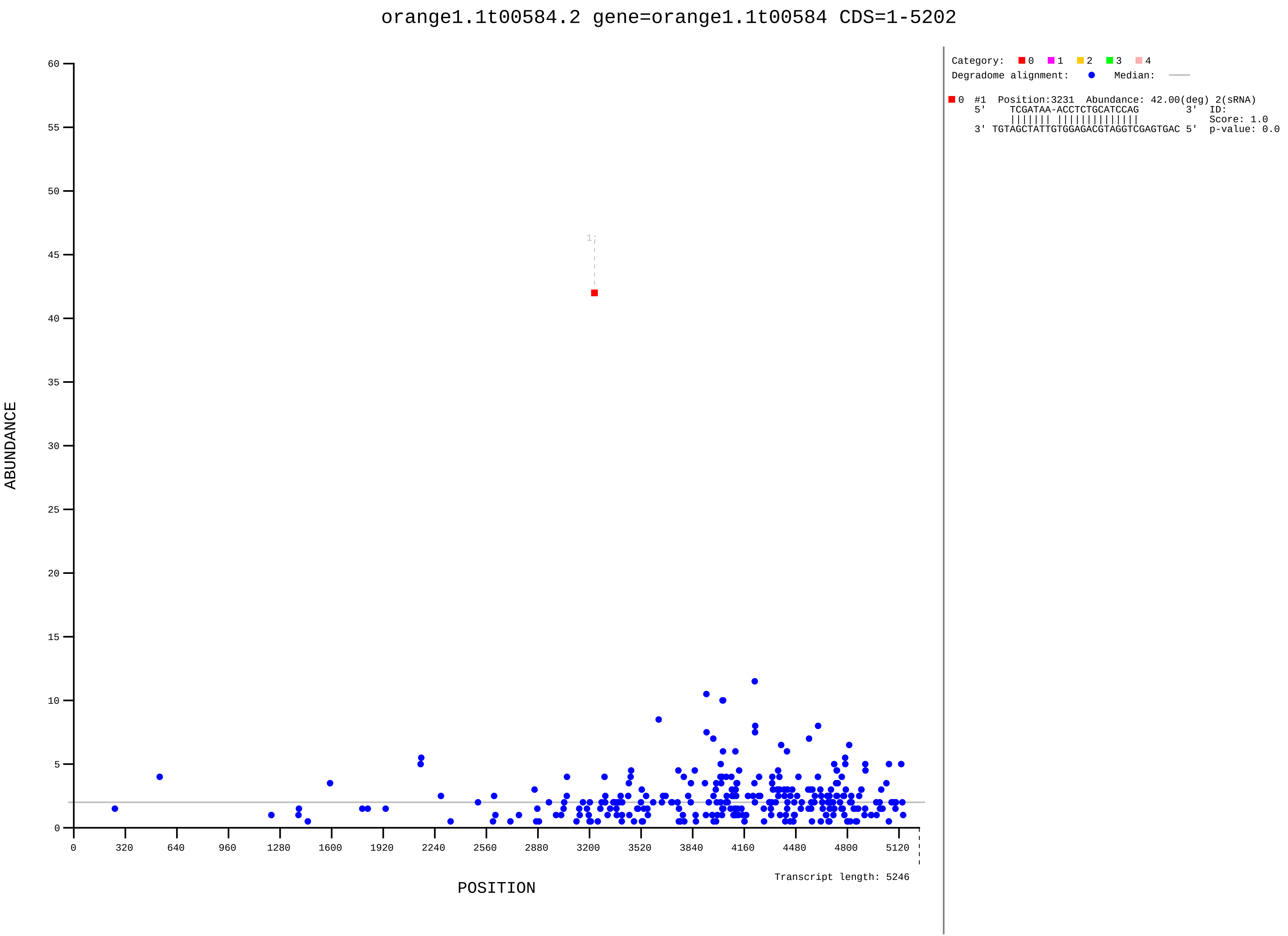
orange1.1t00584.2 gene=orange1.1t00584 CDS=1-5202
ABUNDANCE
POSITION
Transcript length: 5246
Category: 0 1 2 3 4
Degradome alignment:
Median:
0 #1 Position:3231 Abundance: 42.00(deg) 2(sRNA)
5' TCGATAA-ACCTCTGCATCCAG 3' ID:
Score: 1.0
3' TGTAGCTATTGTGGAGACGTAGGTCGAGTGAC 5' p-value: 0.0

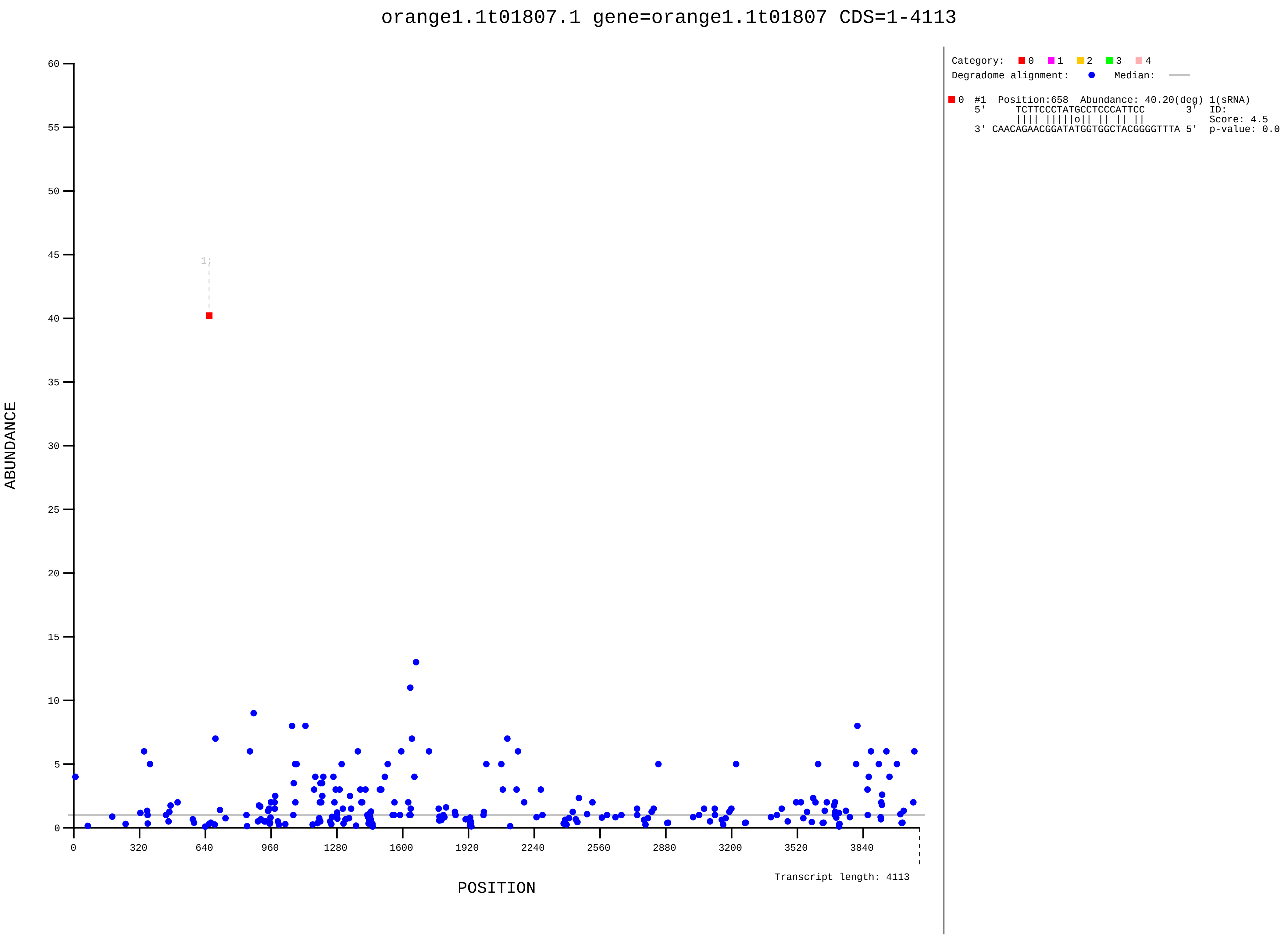
orange1.1t01807.1 gene=orange1.1t01807 CDS=1-4113
Category: 0 1 2 3 4
Degradome alignment:
Median:
0 #1 Position:658 Abundance: 40.20(deg) 1(sRNA)
5' TCTTCCCTATGCCTCCCATTCC 3' ID:
|||| |||||o|| || || ||
Score: 4.5
3' CAACAGAACGGATATGGTGGCTACGGGGTTTA 5' p-value: 0.0
ABUNDANCE
POSITION
Transcript length: 4113

orange1.1t01815.2 gene=orange1.1t01815 CDS=633-3674

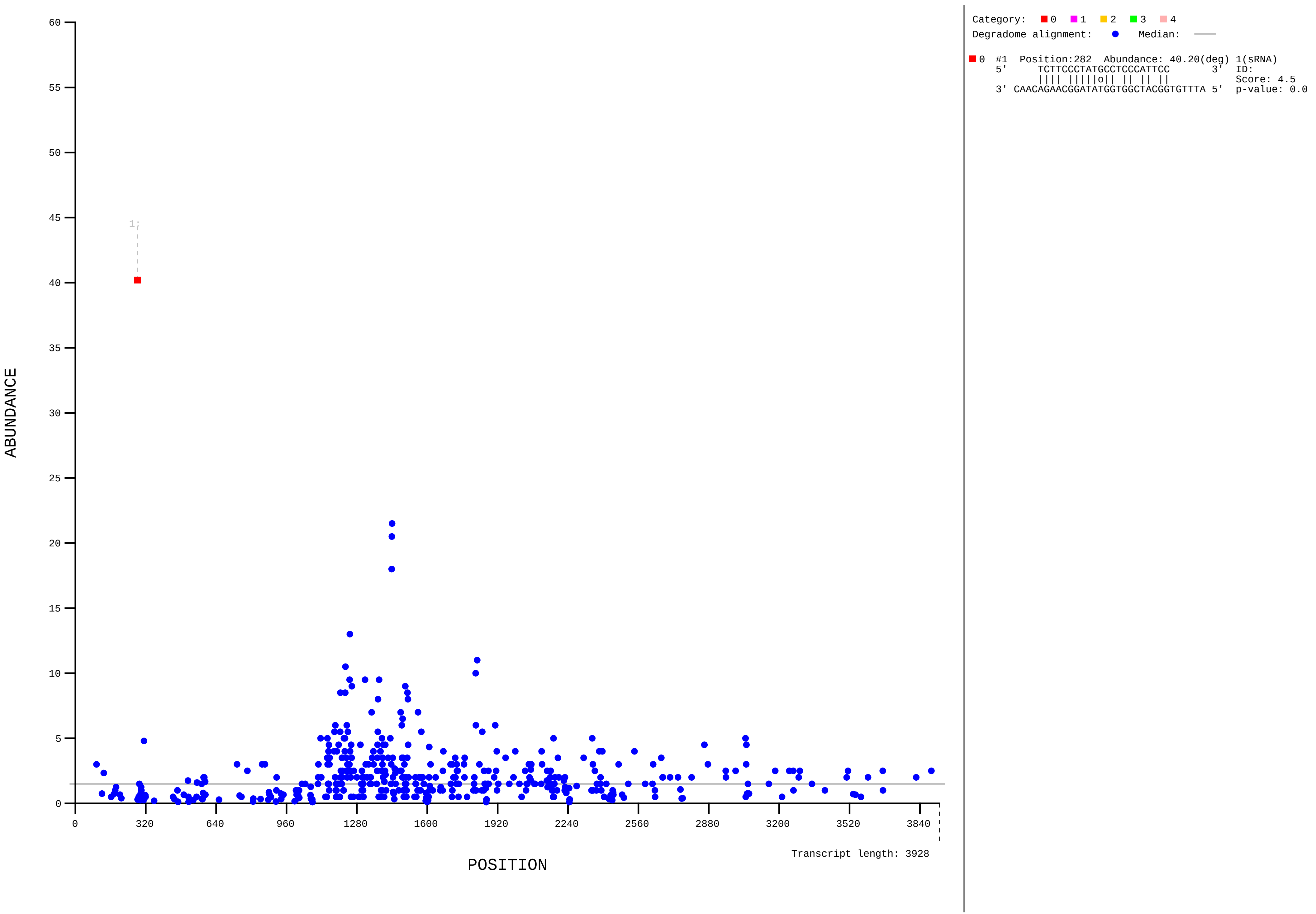

Cs5g19220.1 gene=Cs5g19220 CDS=1-2763

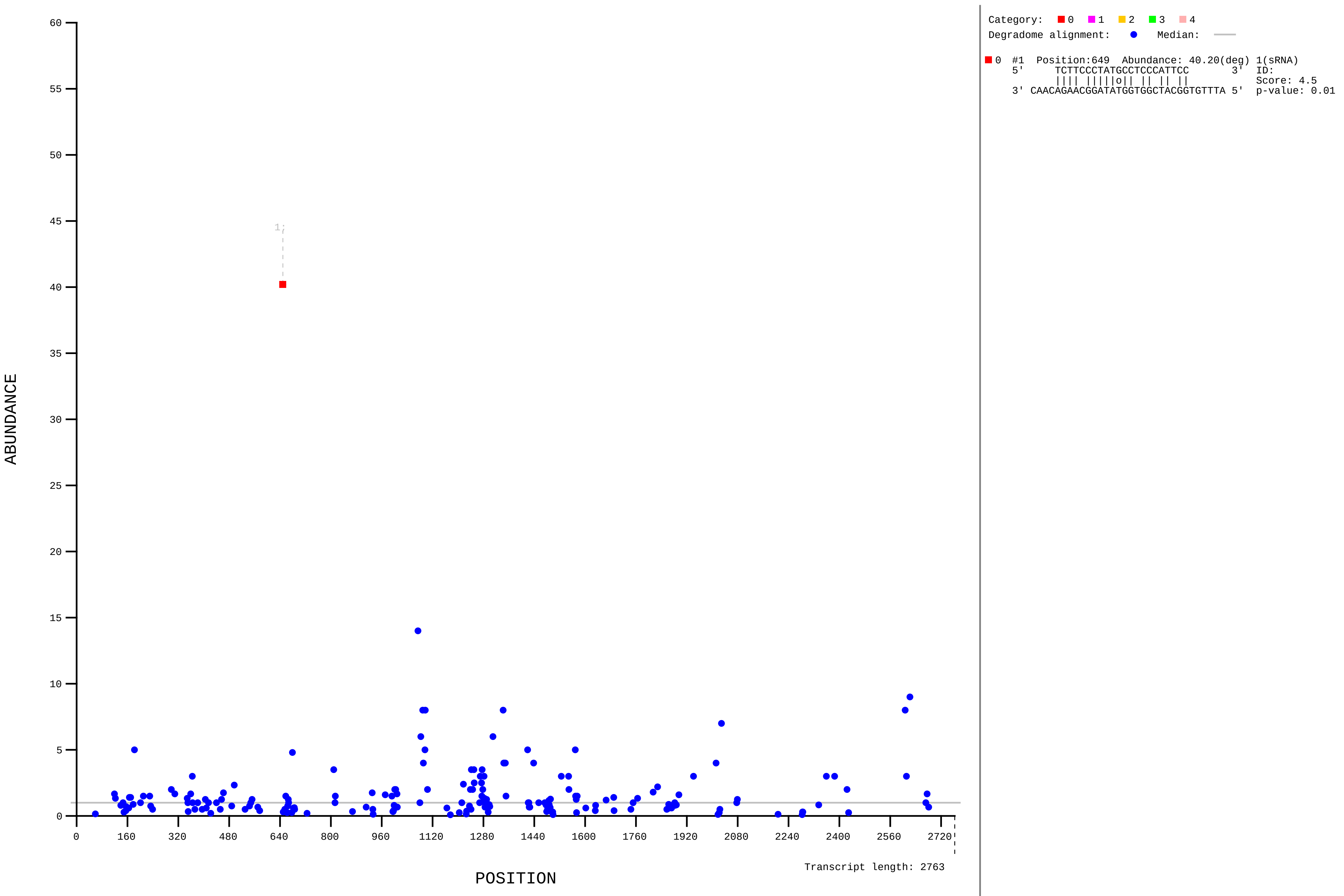

Cs5g19260.1 gene=Cs5g19260 CDS=1-2457

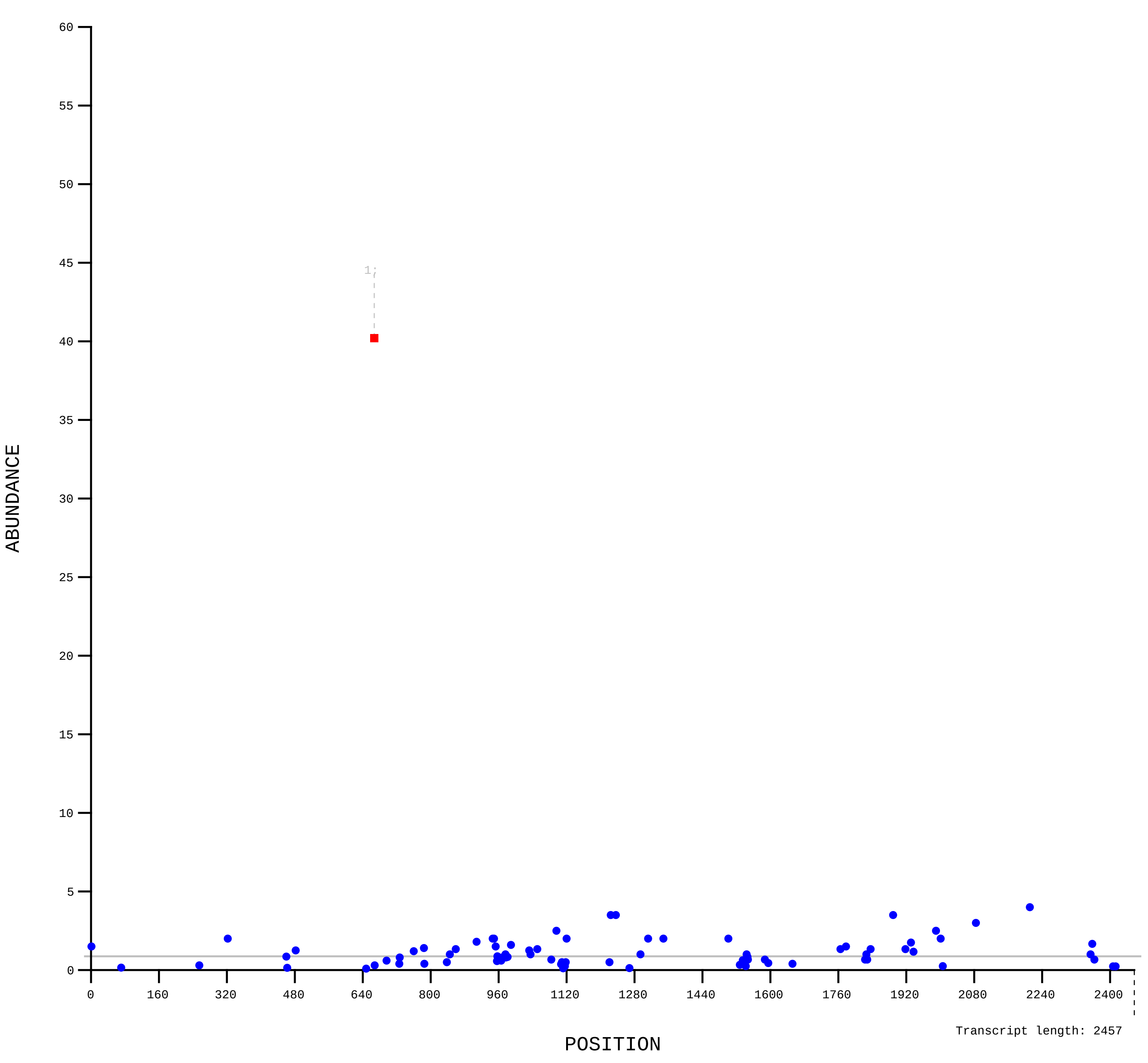

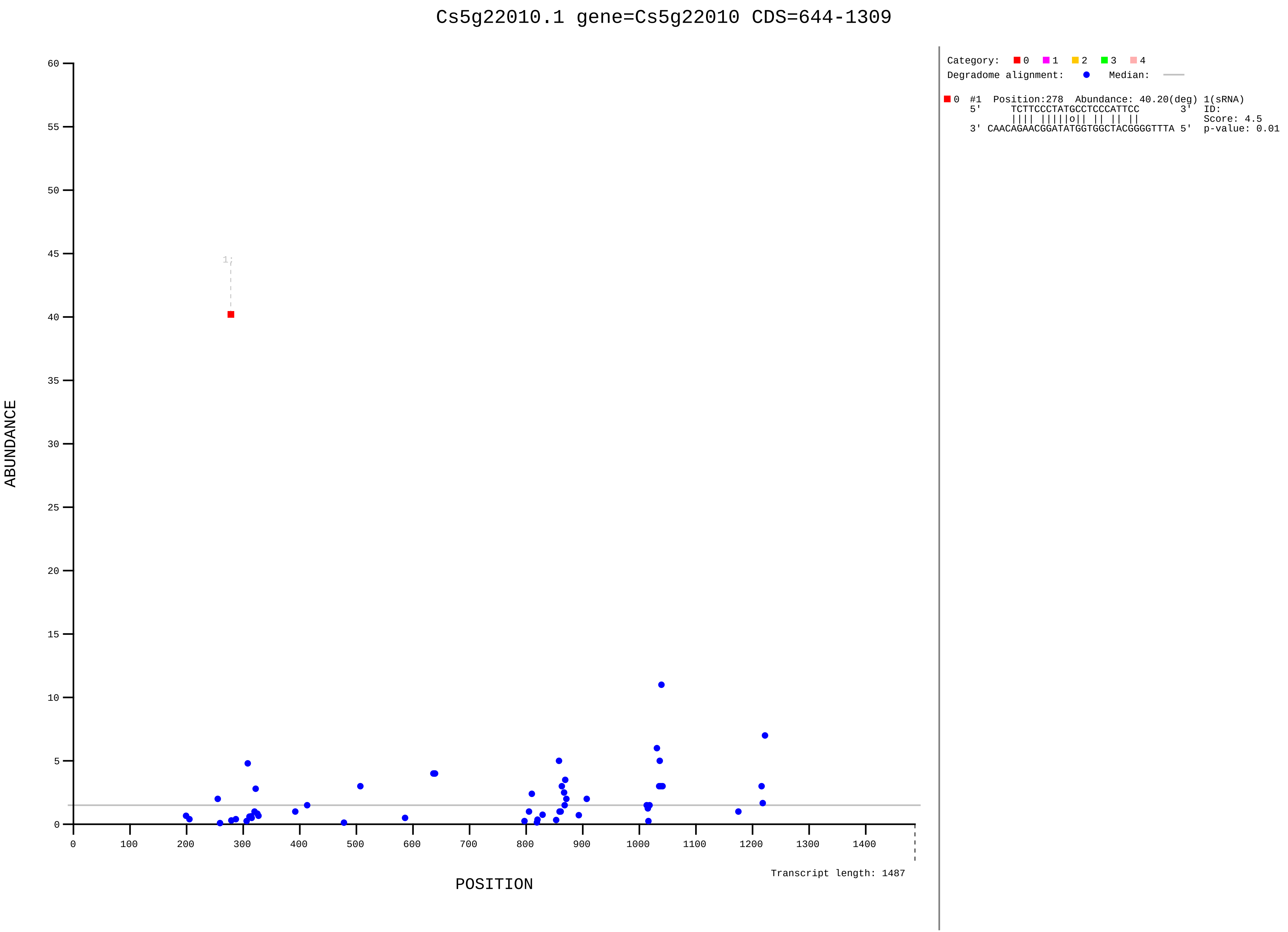

Cs5g22010.1 gene=Cs5g22010 CDS=644-1309
ABUNDANCE
POSITION
Transcript length: 1487
Category: 0 1 2 3 4
Degradome alignment:
Median:
0 #1 Position:278 Abundance: 40.20(deg) 1(sRNA)
5' TCTTCCCTATGCCTCCCATTCC 3' ID:
|||| |||||o|| || || ||
Score: 4.5
3' CAACAGAACGGATATGGTGGCTACGGGGTTTA 5' p-value: 0.01

orange1.1t01823.1 gene=orange1.1t01823 CDS=1-3678

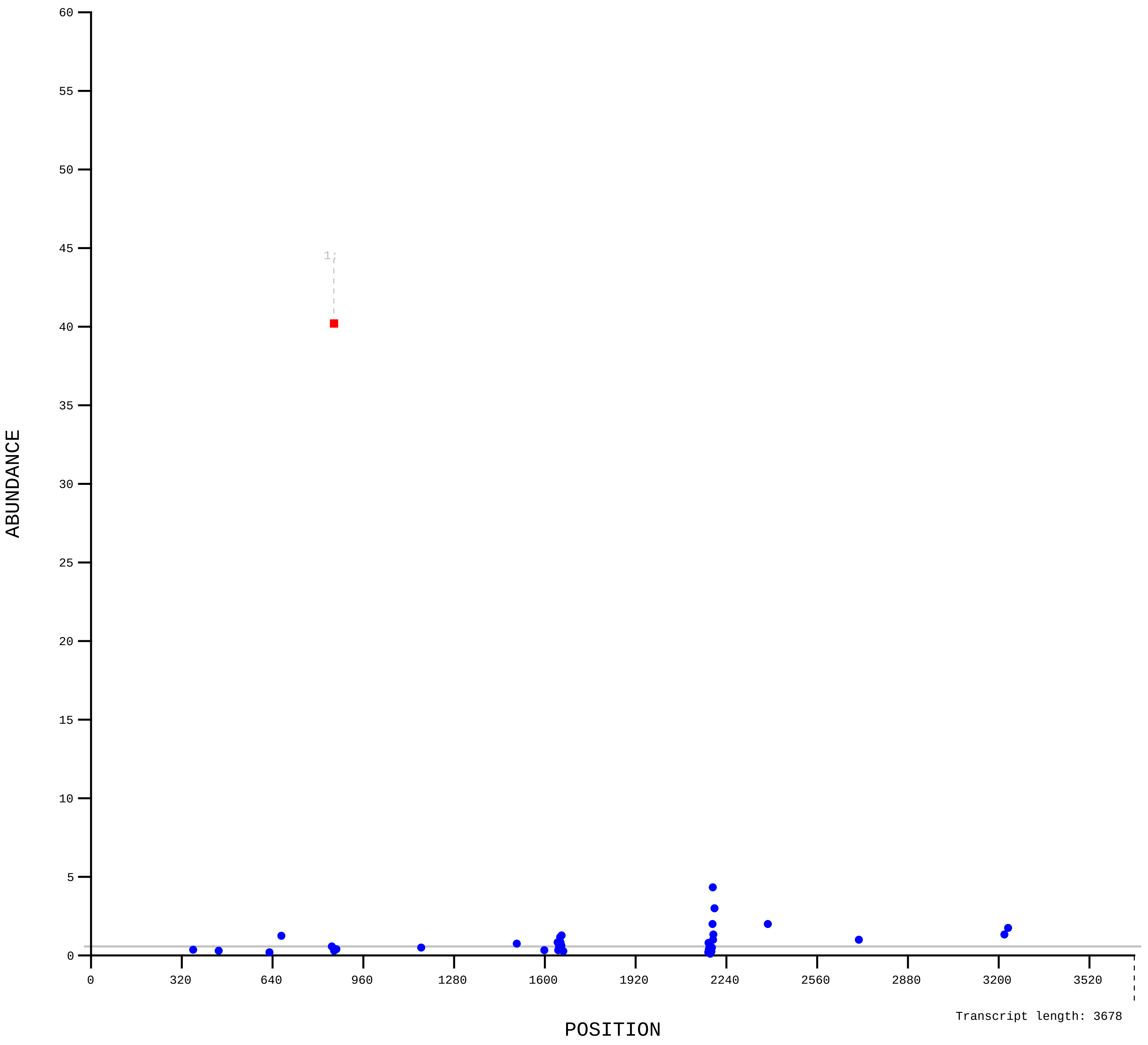

Category: 0 1 2 3 4
Degradome alignment: Median:

0 #1 Position:856 Abundance: 40.20(deg) 1(sRNA)

```
5'      TCTTCCCTATGCCTCCCATTCC       3'  ID:
        |||| |||||o|| || || ||           Score: 4.5
3' CAACAGAACGGATATGGTGGTTACGGGGTTTA 5'  p-value: 0.02
```

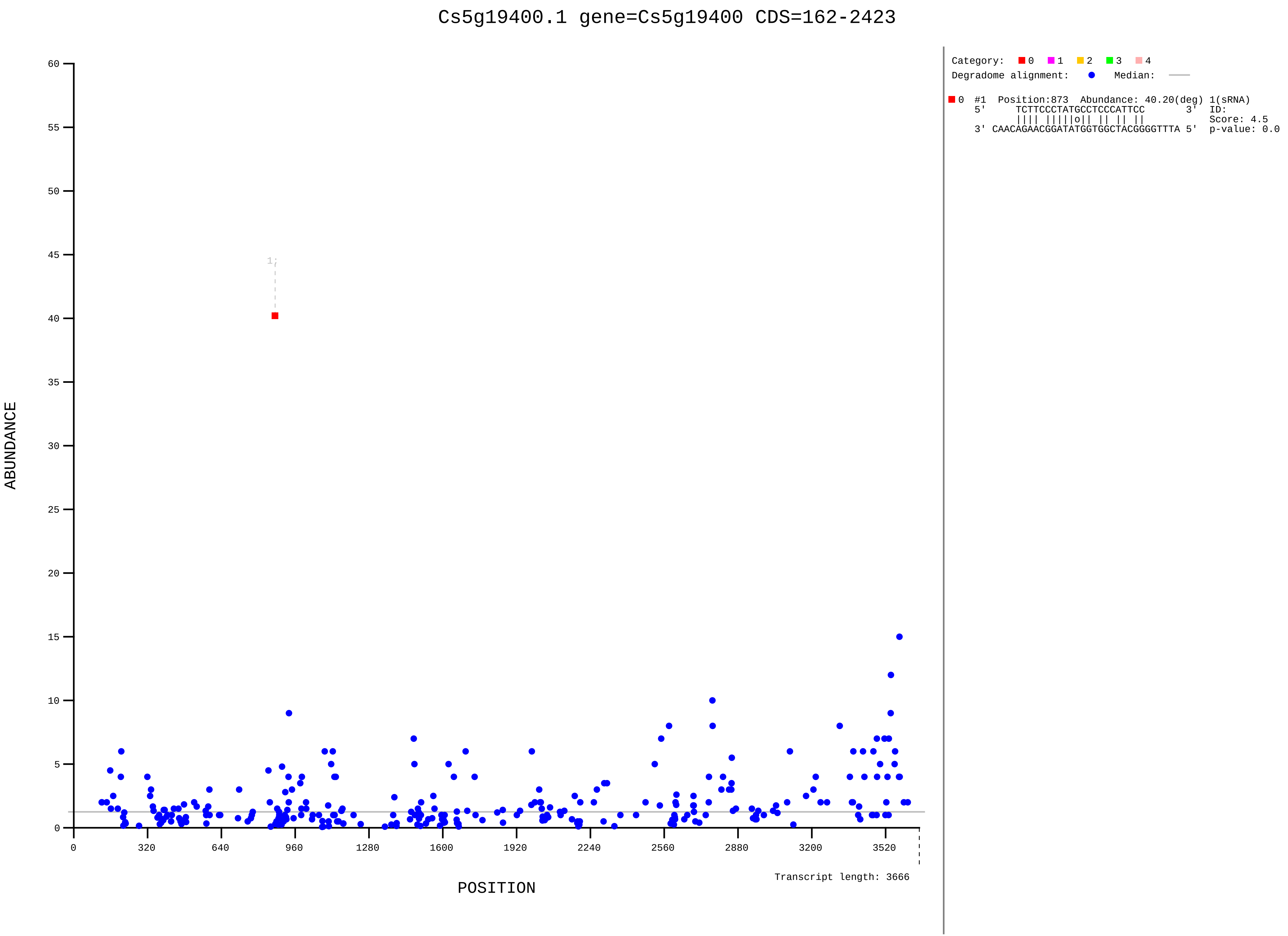
Cs5g19400.1 gene=Cs5g19400 CDS=162-2423
ABUNDANCE
POSITION
Transcript length: 3666
Category: 0 1 2 3 4
Degradome alignment: Median:
0 #1 Position:873 Abundance: 40.20(deg) 1(sRNA)
5' TCTTCCCTATGCCTCCCATTCC 3' ID:
|||| |||||o|| || || ||
Score: 4.5
3' CAACAGAACGGATATGGTGGCTACGGGGTTTA 5' p-value: 0.0

orange1.1t01815.1 gene=orange1.1t01815 CDS=693-4793

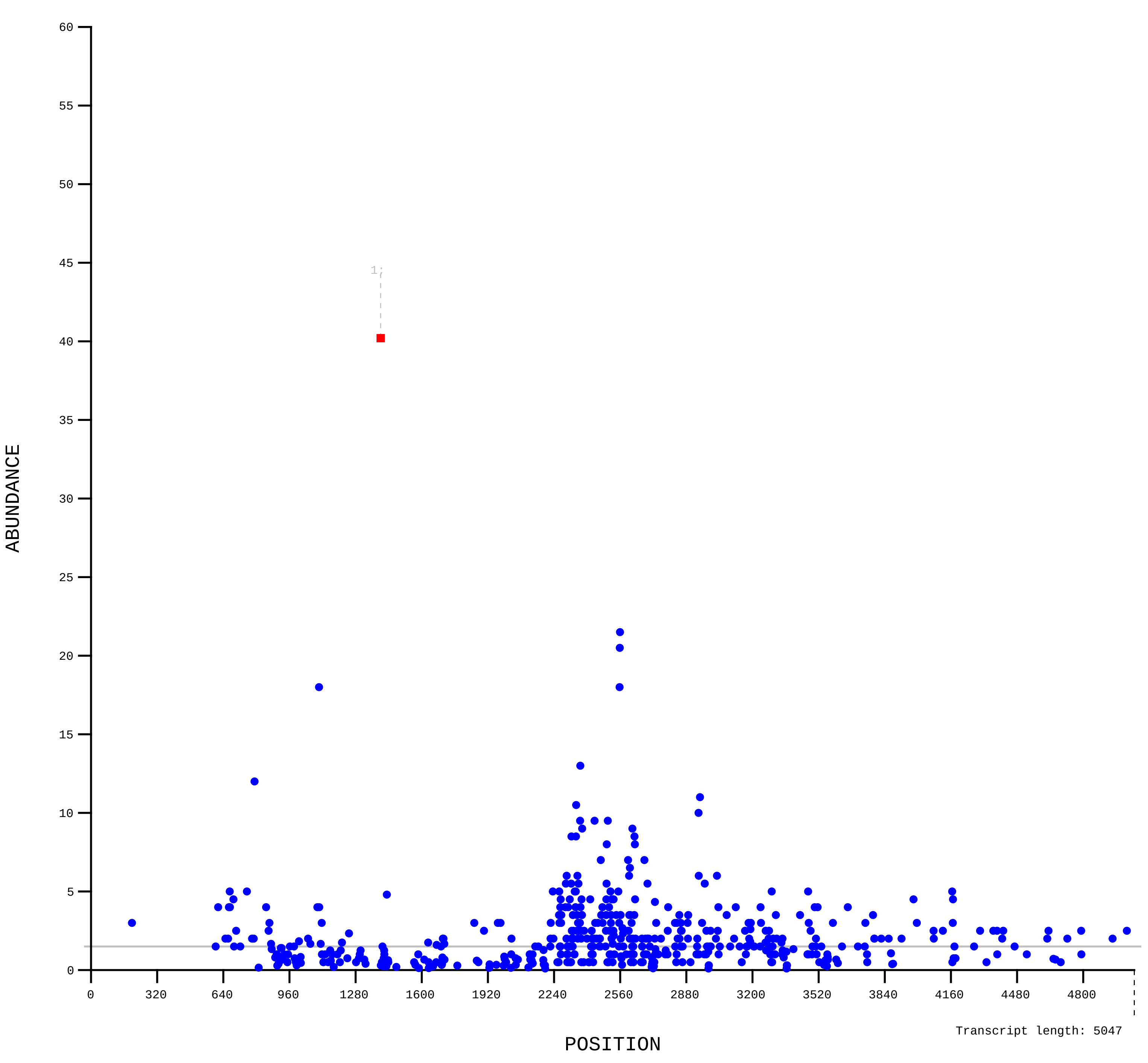


```
Category:  ■0  ■1  ■2  ■3  ■4
Degradome alignment: ●   Median: ——

■0  #1  Position:1401  Abundance: 40.20(deg) 1(sRNA)
    5'     TCTTCCCTATGCCTCCCATTCC       3'  ID:
           |||| |||||o|| || || ||           Score: 4.5
    3' CAACAGAACGGATATGGTGGCTACGGTGTTTA 5'  p-value: 0.0
```

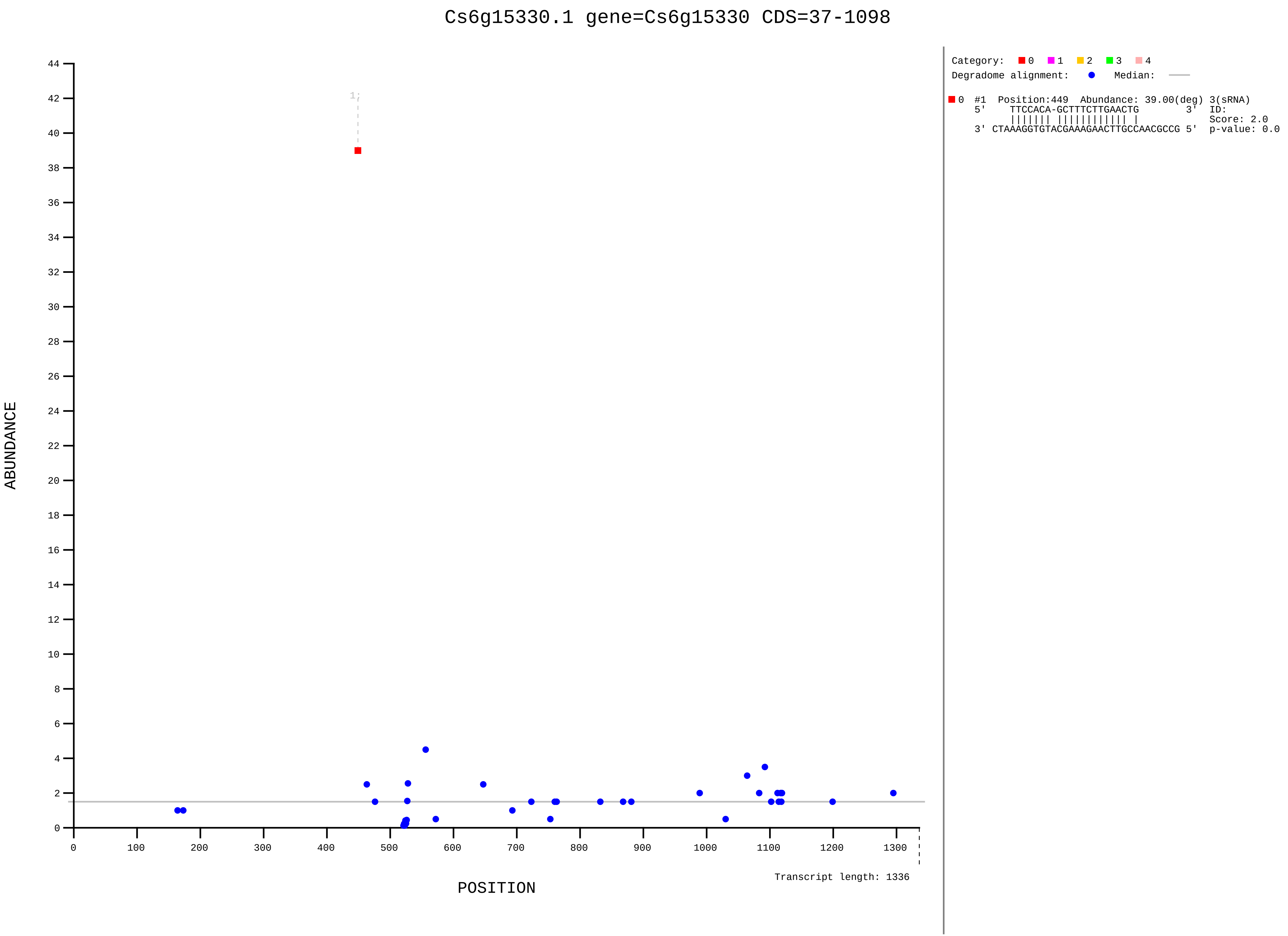

Cs6g15330.1 gene=Cs6g15330 CDS=37-1098
ABUNDANCE
POSITION
Transcript length: 1336
Category: 0 1 2 3 4
Degradome alignment:
Median:
0 #1 Position:449 Abundance: 39.00(deg) 3(sRNA)
5' TTCCACA-GCTTTCTTGAACTG 3' ID:
||||||| |||||||||||| |
Score: 2.0
3' CTAAAGGTGTACGAAAGAACTTGCCAACGCCG 5' p-value: 0.0

Cs6g15330.2 gene=Cs6g15330 CDS=203-1426

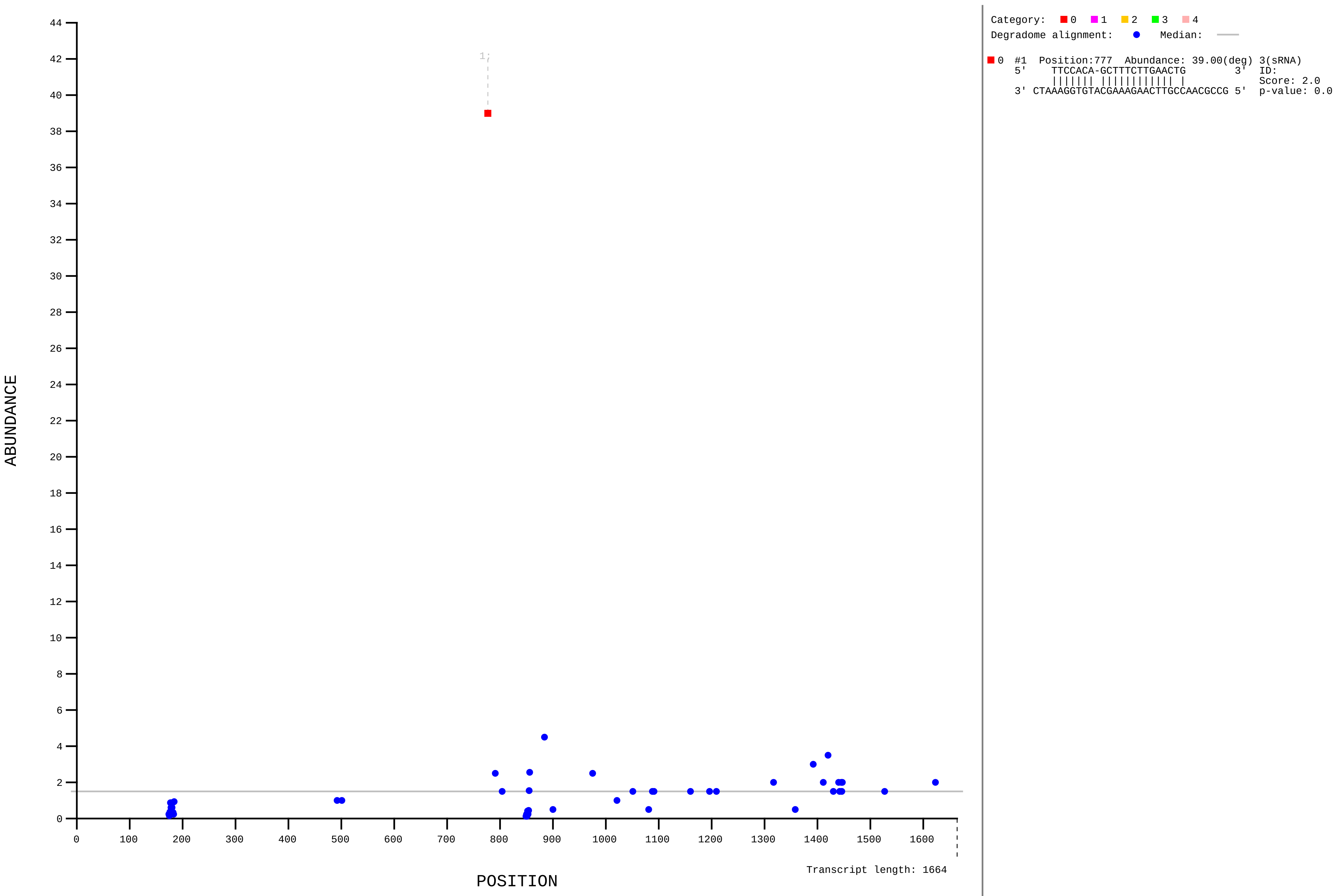

Cs5g09850.1 gene=Cs5g09850 CDS=271-969

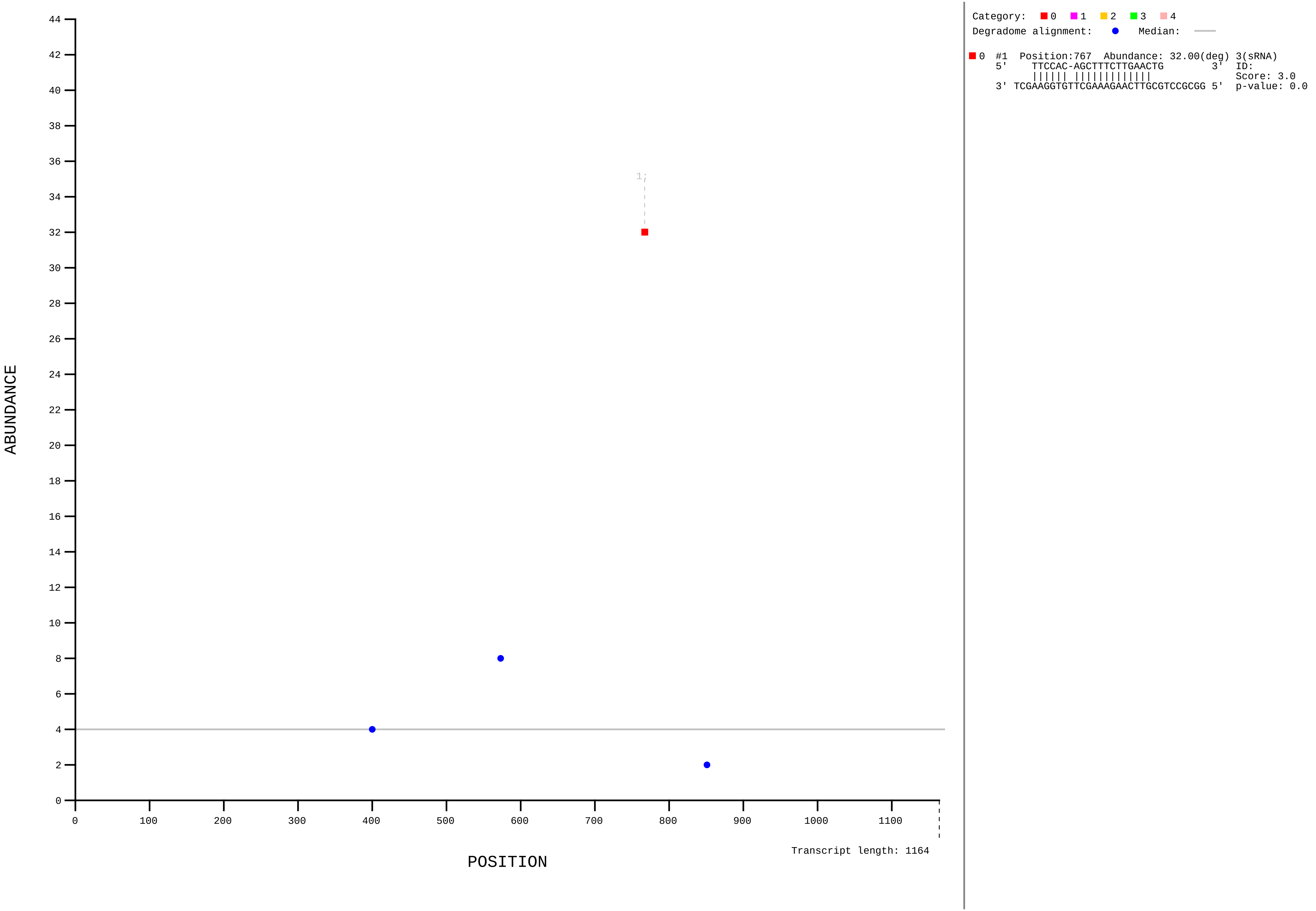

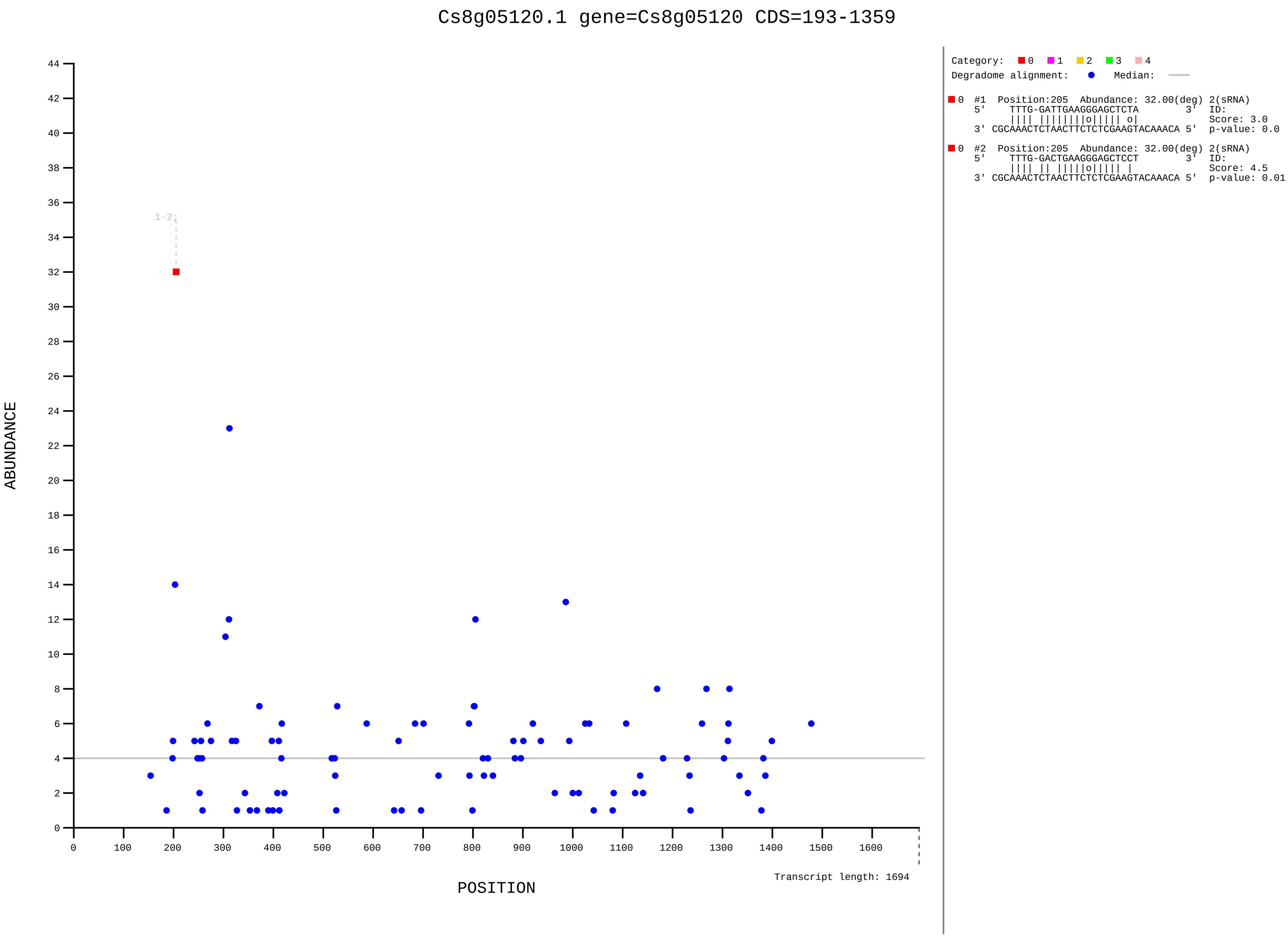

Cs8g05120.1 gene=Cs8g05120 CDS=193-1359
ABUNDANCE
POSITION
1-2
Transcript length: 1694
Category: 0 1 2 3 4
Degradome alignment: Median:
0 #1 Position:205 Abundance: 32.00(deg) 2(sRNA)
5' TTTG-GATTGAAGGGAGCTCTA 3' ID:
|||| ||||||||o||||| o| Score: 3.0
3' CGCAAACTCTAACTTCTCTCGAAGTACAAACA 5' p-value: 0.0
0 #2 Position:205 Abundance: 32.00(deg) 2(sRNA)
5' TTTG-GACTGAAGGGAGCTCCT 3' ID:
|||| || |||||o||||| | Score: 4.5
3' CGCAAACTCTAACTTCTCTCGAAGTACAAACA 5' p-value: 0.01

Cs4g19310.1 gene=Cs4g19310 CDS=74-2626

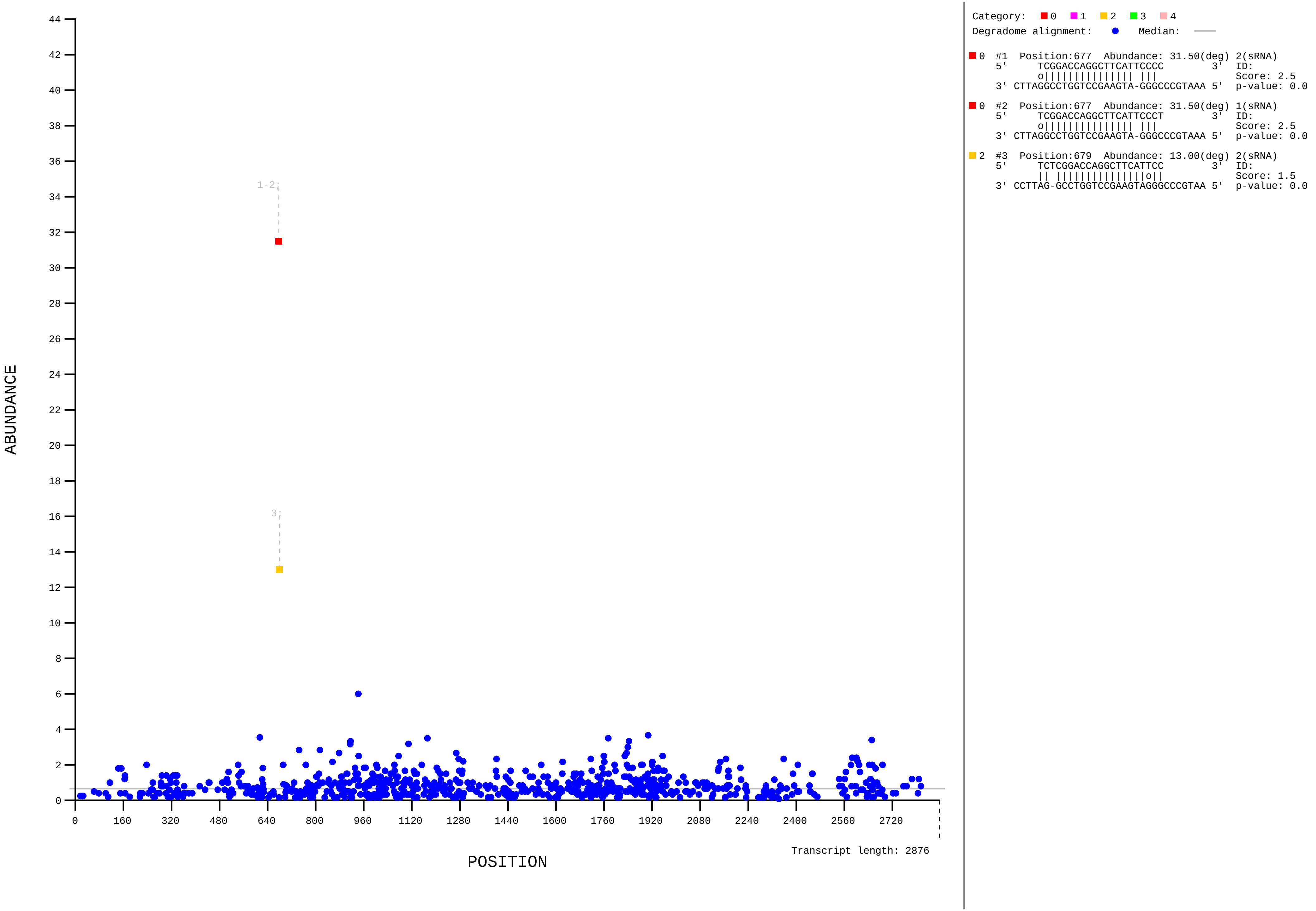

Cs4g19310.2 gene=Cs4g19310 CDS=78-2630

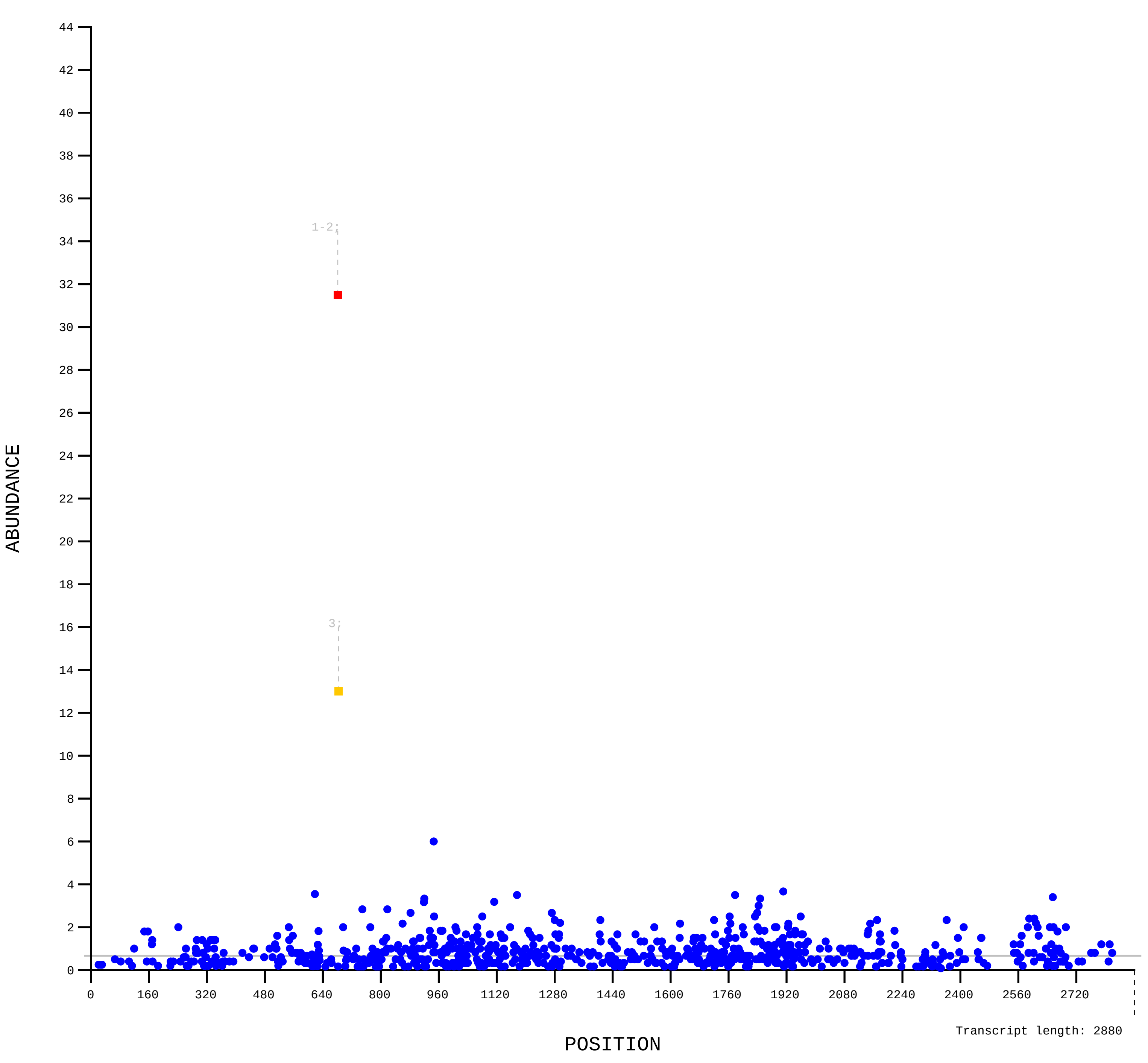


```
Category:  ■0  ■1  ■2  ■3  ■4
Degradome alignment:  ●    Median: ——

■0  #1  Position:681  Abundance: 31.50(deg) 2(sRNA)
    5'     TCGGACCAGGCTTCATTCCCC        3'  ID:
           o||||||||||||||| |||             Score: 2.5
    3' CTTAGGCCTGGTCCGAAGTA-GGGCCCGTAAA 5'  p-value: 0.0

■0  #2  Position:681  Abundance: 31.50(deg) 1(sRNA)
    5'     TCGGACCAGGCTTCATTCCCT        3'  ID:
           o||||||||||||||| |||             Score: 2.5
    3' CTTAGGCCTGGTCCGAAGTA-GGGCCCGTAAA 5'  p-value: 0.0

■2  #3  Position:683  Abundance: 13.00(deg) 2(sRNA)
    5'     TCTCGGACCAGGCTTCATTCC        3'  ID:
           || ||||||||||||||||o||           Score: 1.5
    3' CCTTAG-GCCTGGTCCGAAGTAGGGCCCGTAAA 5'  p-value: 0.0
```

Cs4g19310.6 gene=Cs4g19310 CDS=142-2106

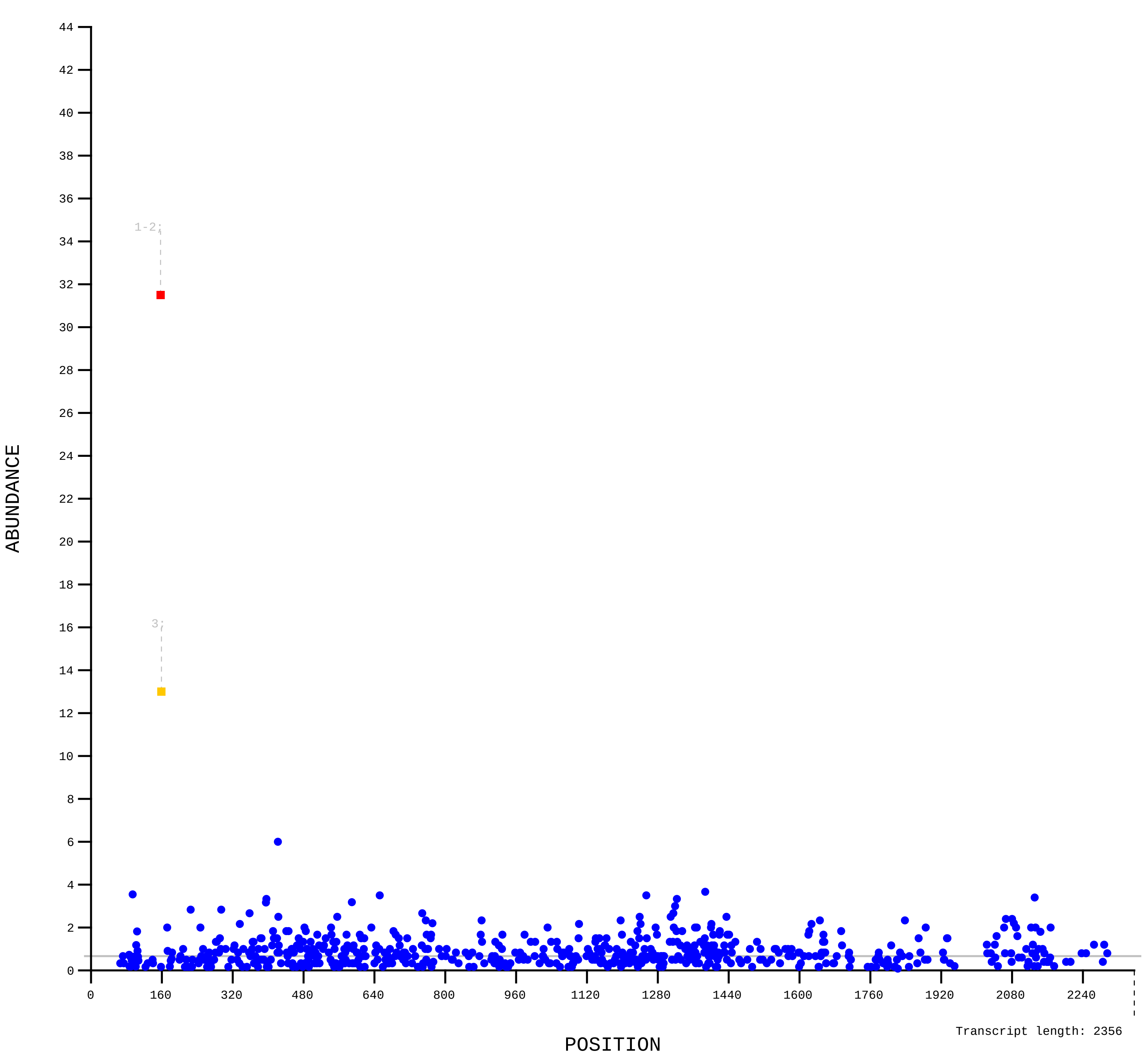

Category: 0 1 2 3 4
Degradome alignment: Median:

```
0  #1  Position:157  Abundance: 31.50(deg) 2(sRNA)
   5'      TCGGACCAGGCTTCATTCCCC         3'  ID:
          o||||||||||||||| |||               Score: 2.5
   3' CTTAGGCCTGGTCCGAAGTA-GGGCCCGTAAA 5'  p-value: 0.0

0  #2  Position:157  Abundance: 31.50(deg) 1(sRNA)
   5'      TCGGACCAGGCTTCATTCCCT         3'  ID:
          o||||||||||||||| |||               Score: 2.5
   3' CTTAGGCCTGGTCCGAAGTA-GGGCCCGTAAA 5'  p-value: 0.0

2  #3  Position:159  Abundance: 13.00(deg) 2(sRNA)
   5'      TCTCGGACCAGGCTTCATTCC         3'  ID:
          || ||||||||||||||||o||             Score: 1.5
   3' CCTTAG-GCCTGGTCCGAAGTAGGGCCCGTAA 5'  p-value: 0.0
```

Cs4g19310.5 gene=Cs4g19310 CDS=275-2770

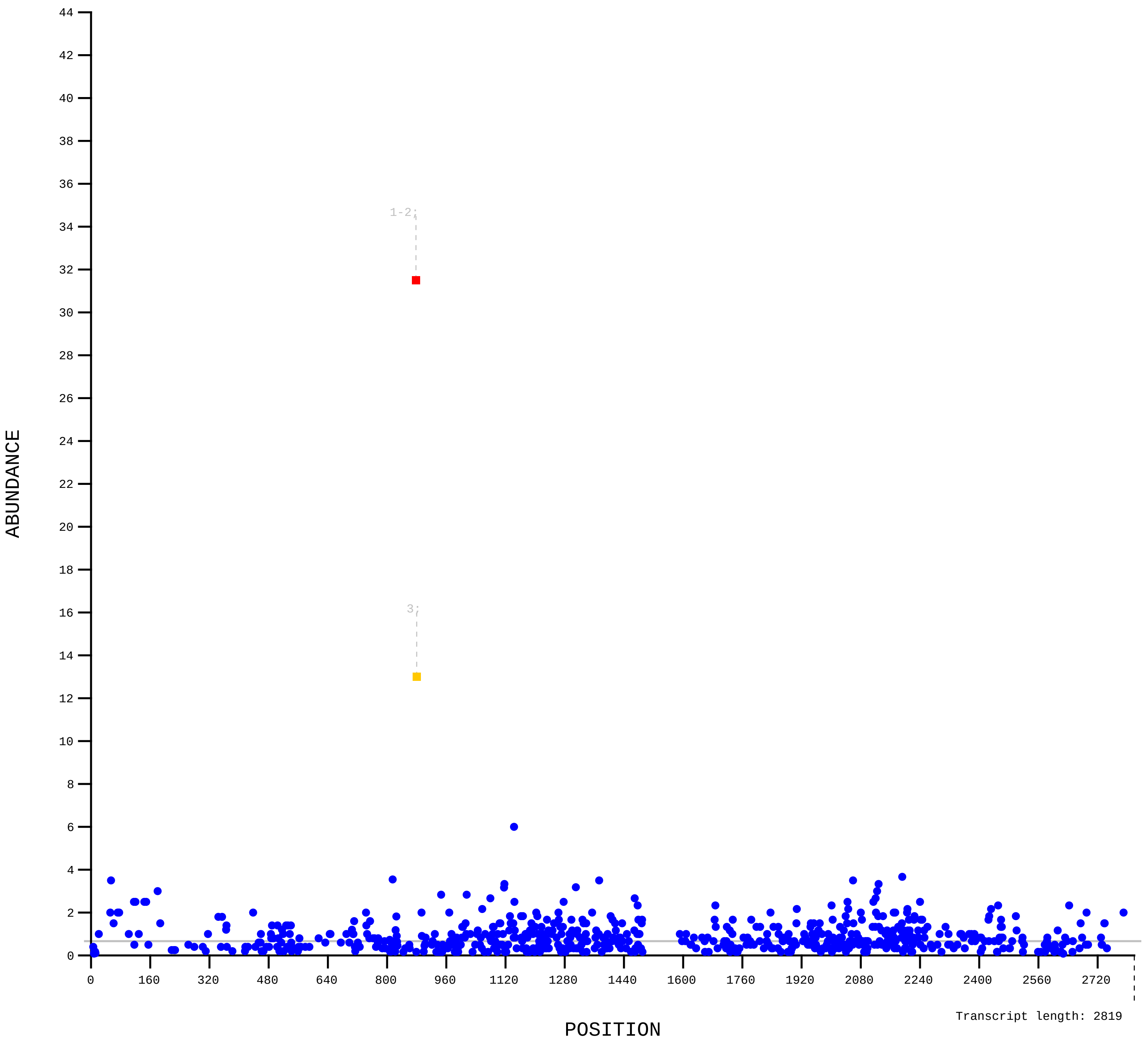

Category: 0 1 2 3 4

Degradome alignment: Median:

```
0  #1  Position:878  Abundance: 31.50(deg) 2(sRNA)
   5'     TCGGACCAGGCTTCATTCCCC        3'  ID:
          o|||||||||||||||| |||            Score: 2.5
   3' CTTAGGCCTGGTCCGAAGTA-GGGCCCGTAAA 5'  p-value: 0.0

0  #2  Position:878  Abundance: 31.50(deg) 1(sRNA)
   5'     TCGGACCAGGCTTCATTCCCT        3'  ID:
          o|||||||||||||||| |||            Score: 2.5
   3' CTTAGGCCTGGTCCGAAGTA-GGGCCCGTAAA 5'  p-value: 0.0

2  #3  Position:880  Abundance: 13.00(deg) 2(sRNA)
   5'     TCTCGGACCAGGCTTCATTCC        3'  ID:
          || ||||||||||||||||o||           Score: 1.5
   3' CCTTAG-GCCTGGTCCGAAGTAGGGCCCGTAA 5'  p-value: 0.0
```

Cs4g19310.4 gene=Cs4g19310 CDS=39-2591

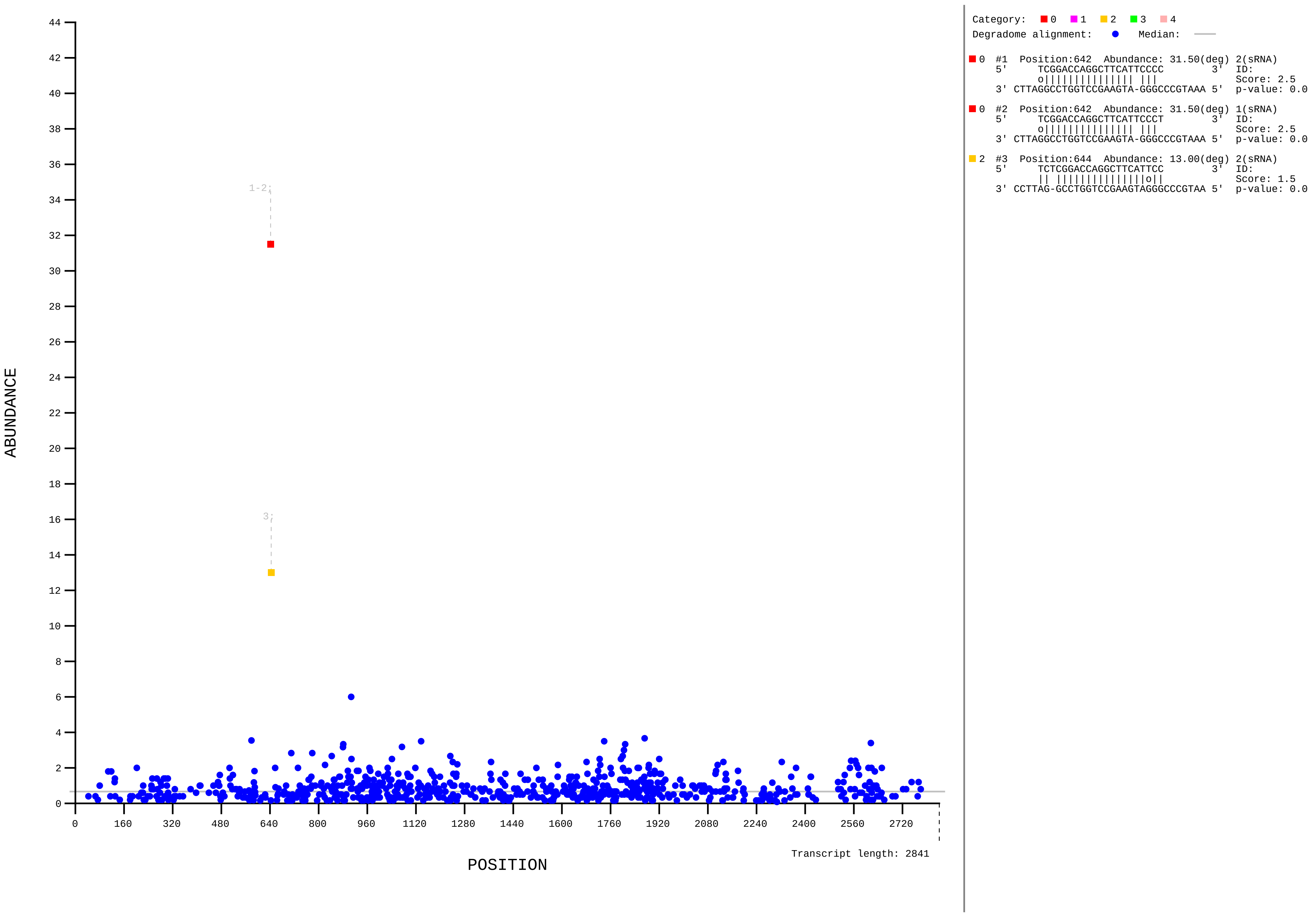

Cs4g19310.3 gene=Cs4g19310 CDS=275-2827

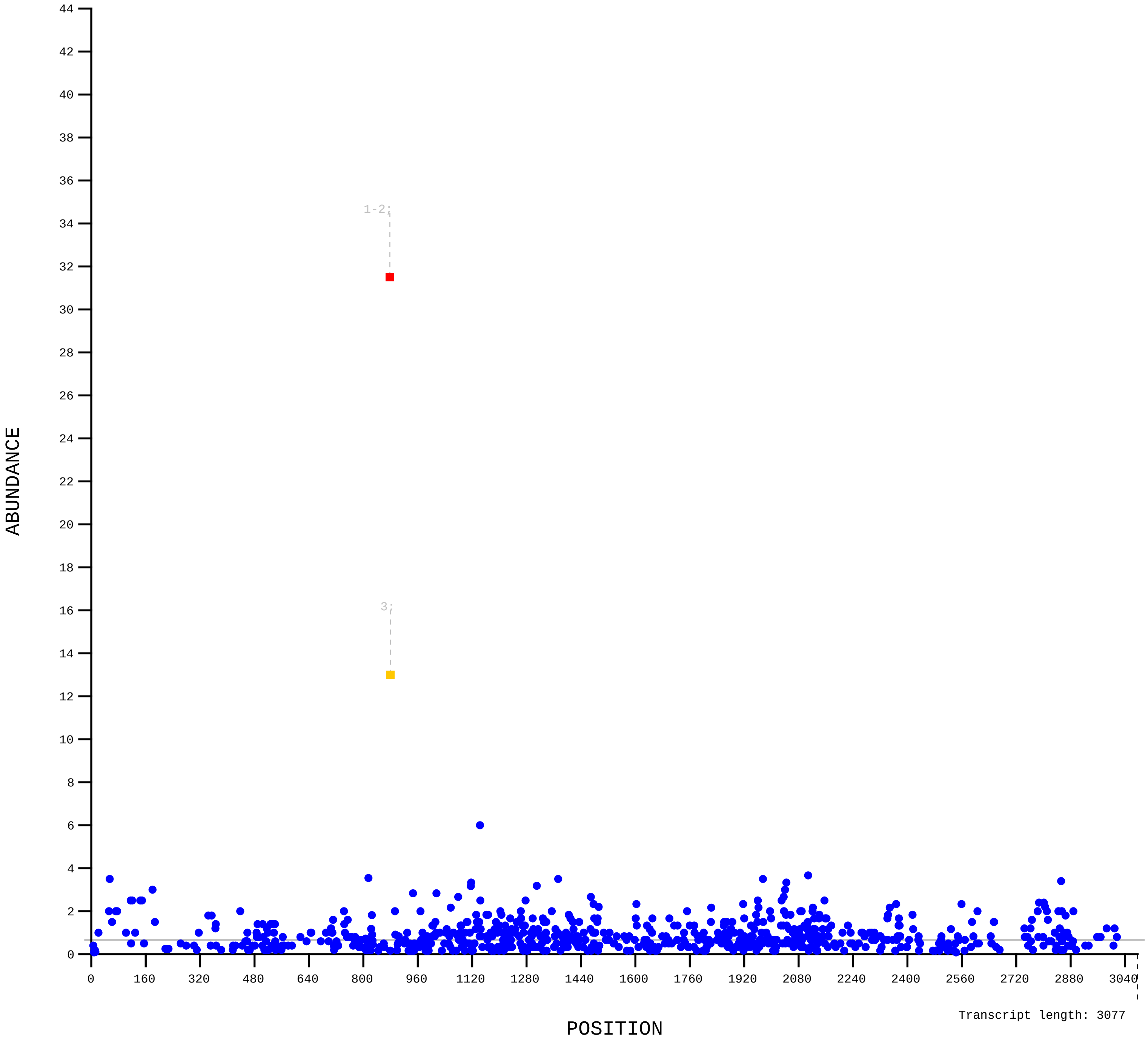

Category: 0 1 2 3 4
Degradome alignment: Median:

0 #1 Position:878 Abundance: 31.50(deg) 2(sRNA)
5' TCGGACCAGGCTTCATTCCCC 3' ID:
o||||||||||||||| ||| Score: 2.5
3' CTTAGGCCTGGTCCGAAGTA-GGGCCCGTAAA 5' p-value: 0.0

0 #2 Position:878 Abundance: 31.50(deg) 1(sRNA)
5' TCGGACCAGGCTTCATTCCCT 3' ID:
o||||||||||||||| ||| Score: 2.5
3' CTTAGGCCTGGTCCGAAGTA-GGGCCCGTAAA 5' p-value: 0.0

2 #3 Position:880 Abundance: 13.00(deg) 2(sRNA)
5' TCTCGGACCAGGCTTCATTCC 3' ID:
|| ||||||||||||||||o|| Score: 1.5
3' CCTTAG-GCCTGGTCCGAAGTAGGGCCCGTAA 5' p-value: 0.0

Cs1g25780.3 gene=Cs1g25780 CDS=335-928

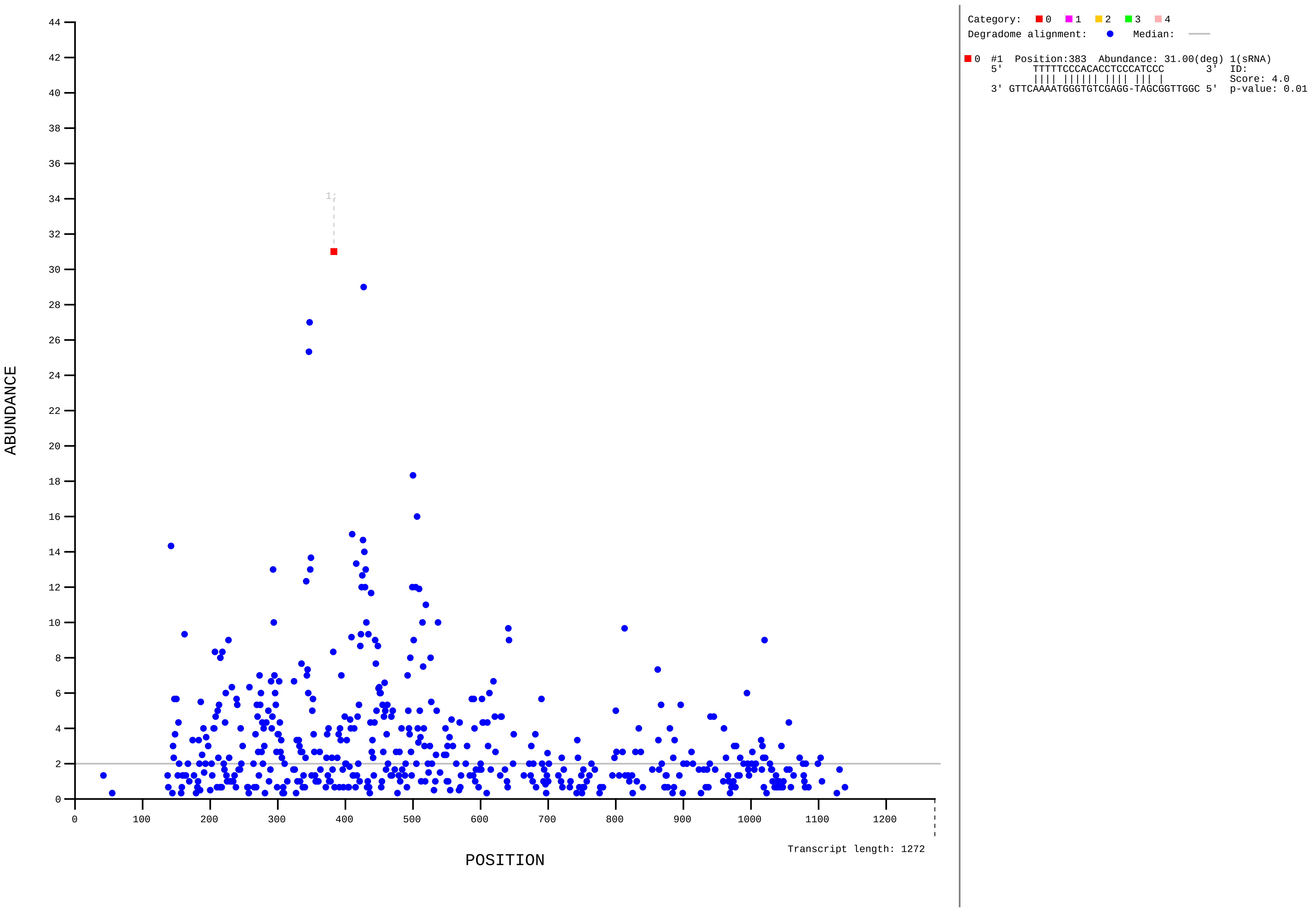

Cs1g25780.2 gene=Cs1g25780 CDS=338-931

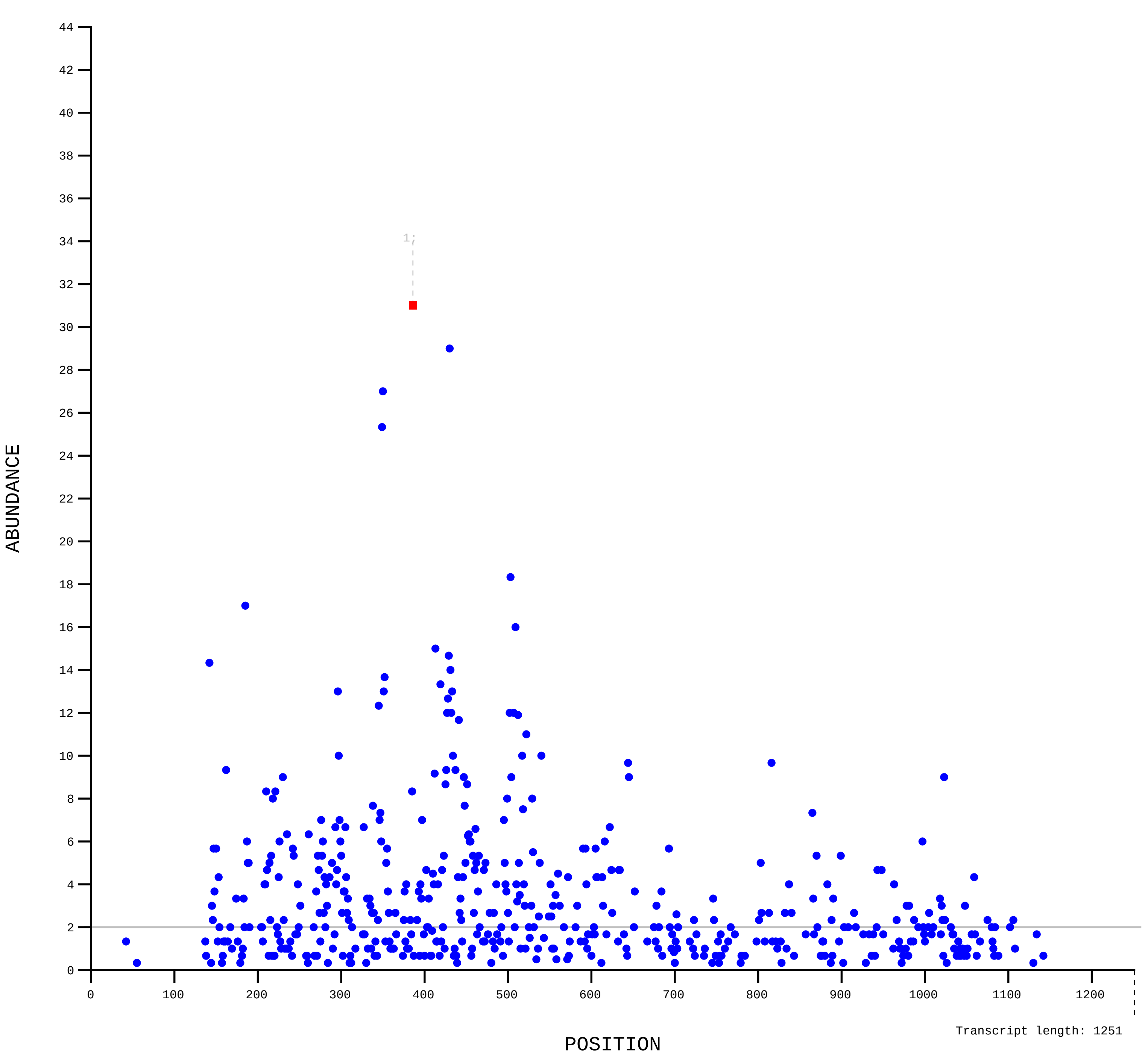

Category: 0 1 2 3 4
Degradome alignment: Median:

0 #1 Position:386 Abundance: 31.00(deg) 1(sRNA)

```
5'     TTTTTCCCACACCTCCCATCCC       3'  ID:
       |||| |||||| |||| ||| |           Score: 4.0
3' GTTCAAAATGGGTGTCGAGG-TAGCGGTTGGC 5'  p-value: 0.01
```

Cs1g25780.1 gene=Cs1g25780 CDS=335-880

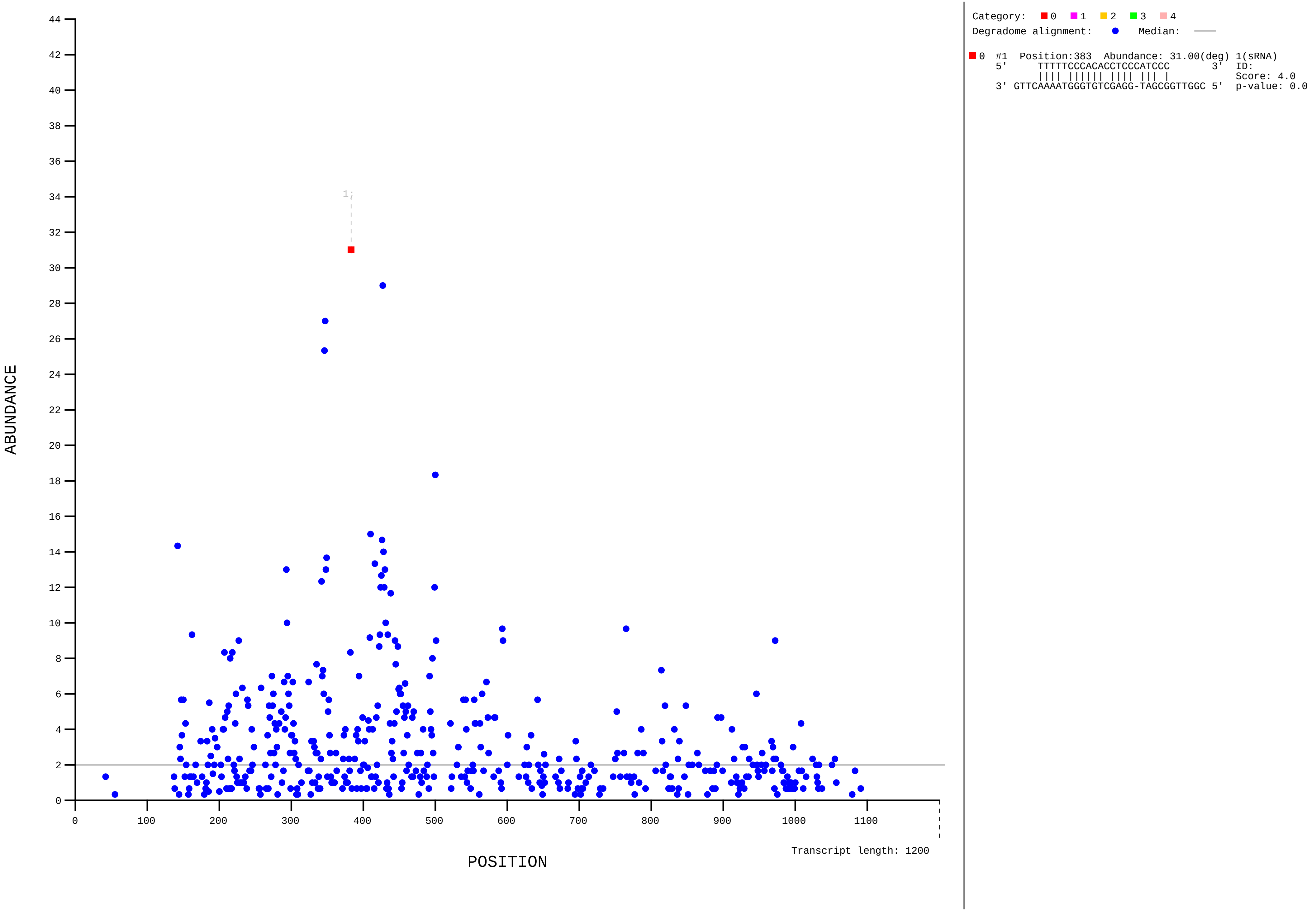

orange1.1t02252.1 gene=orange1.1t02252 CDS=1-4947

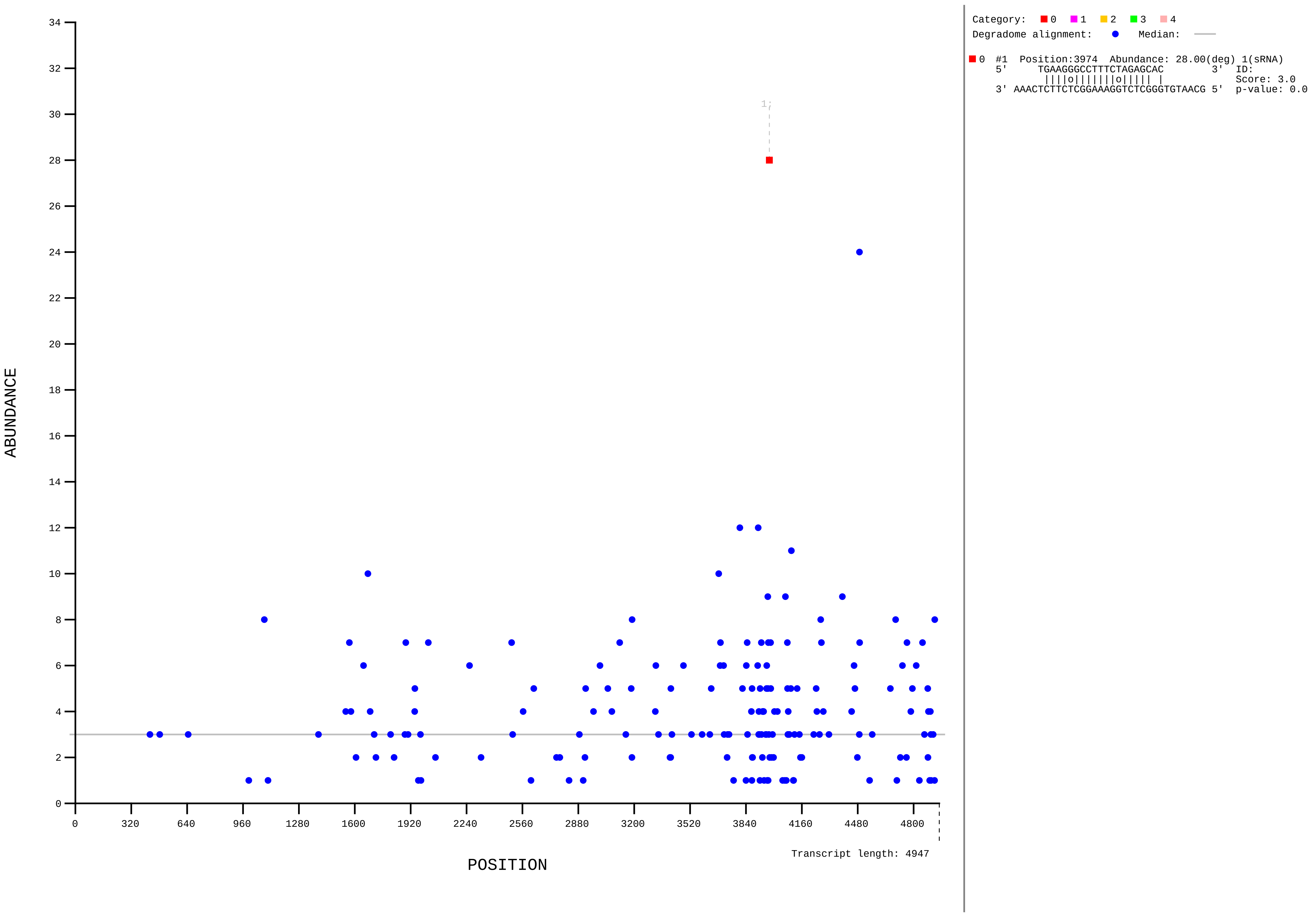

Cs3g12810.1 gene=Cs3g12810 CDS=1-3225

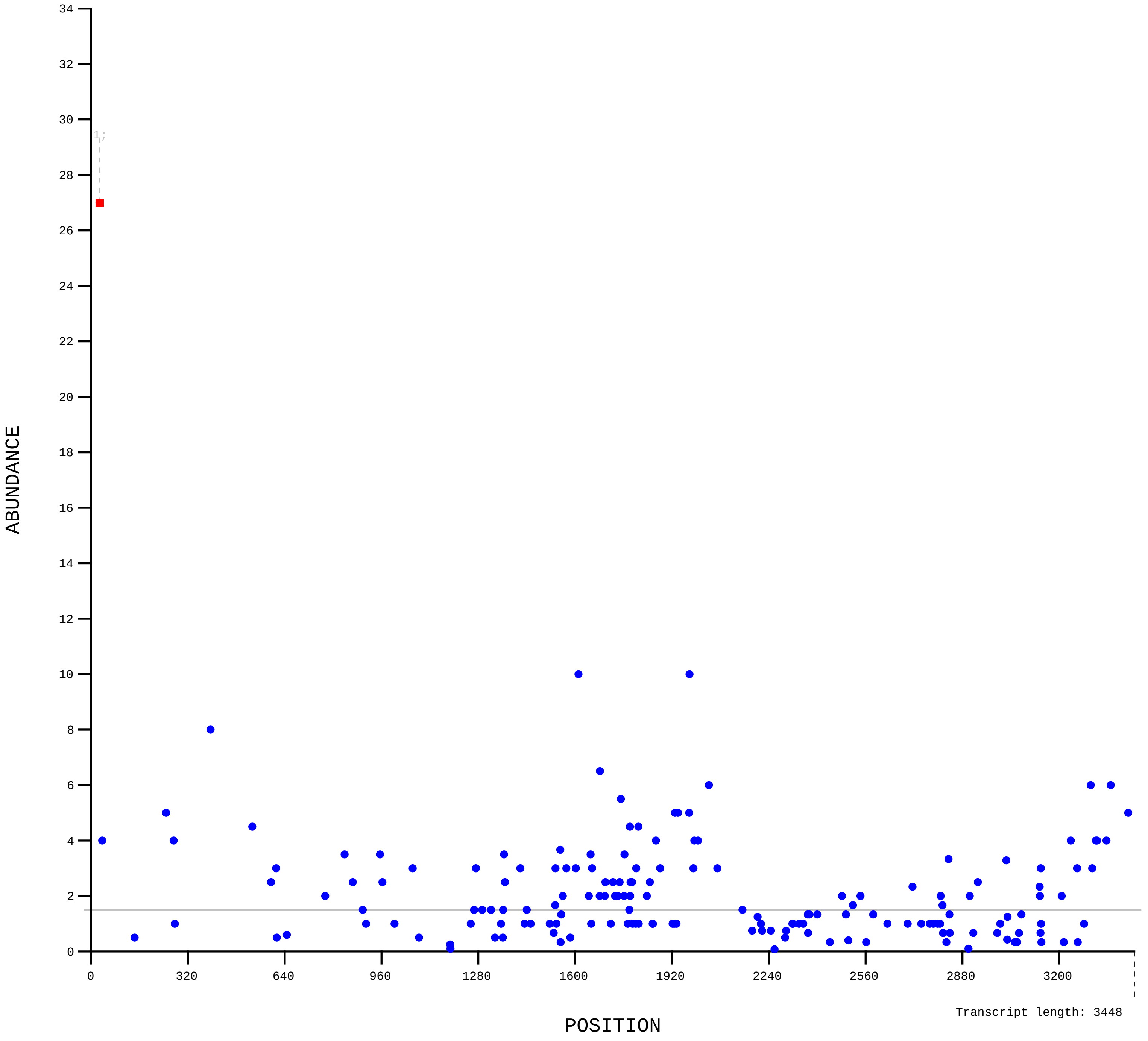

Category: 0 1 2 3 4
Degradome alignment: Median:

0 #1 Position:28 Abundance: 27.00(deg) 1(sRNA)

```
5'     TCTTCCCTATGCCTCCCATTCC       3'  ID:
       |||| || ||||| ||||||||           Score: 3.0
3' CAGCAGAAAGGCTACGGTGGGTAAGGTATTTA 5'  p-value: 0.0
```

Cs8g17390.1 gene=Cs8g17390 CDS=494-2026

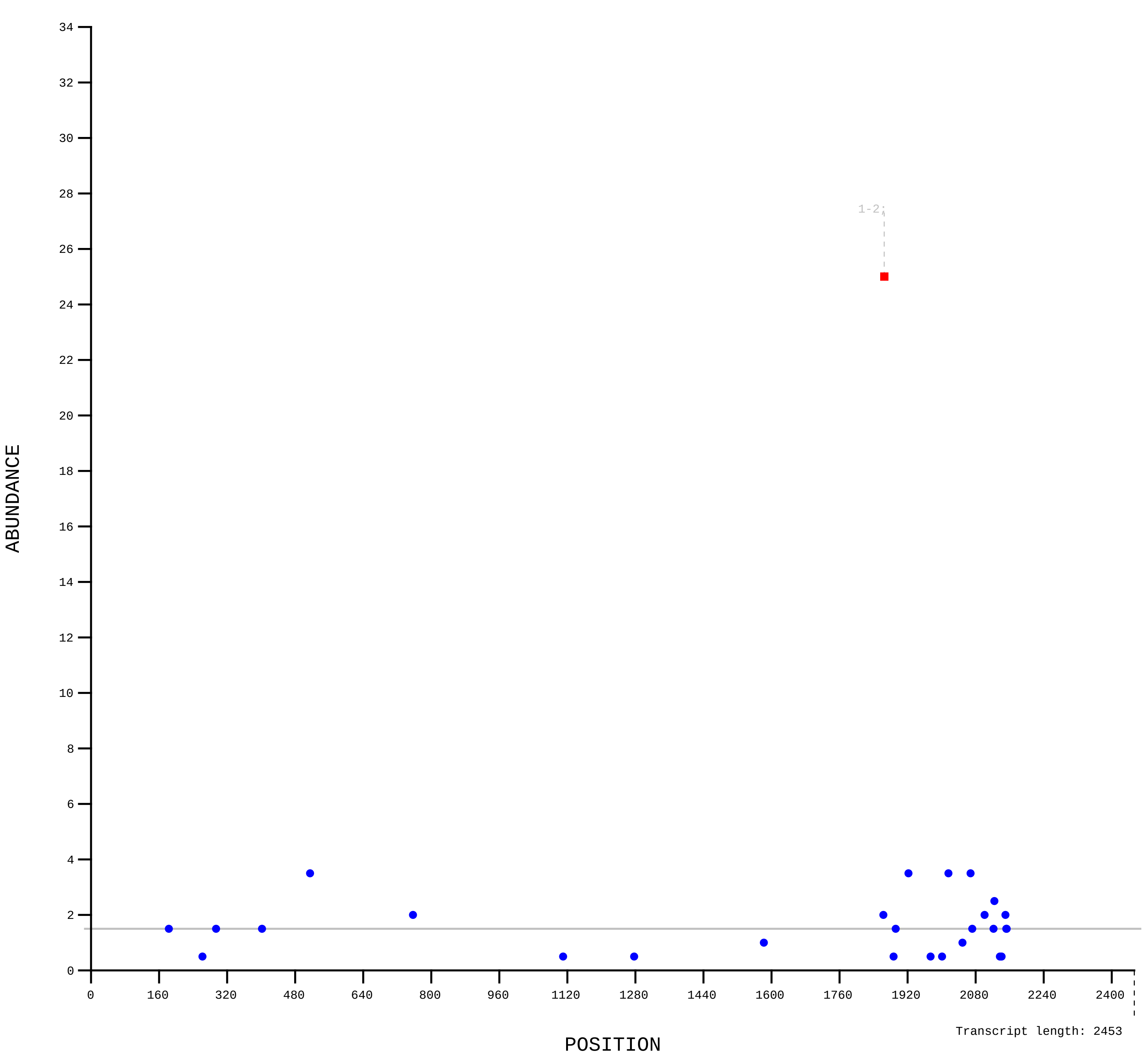

Cs8g17390.2 gene=Cs8g17390 CDS=494-1981

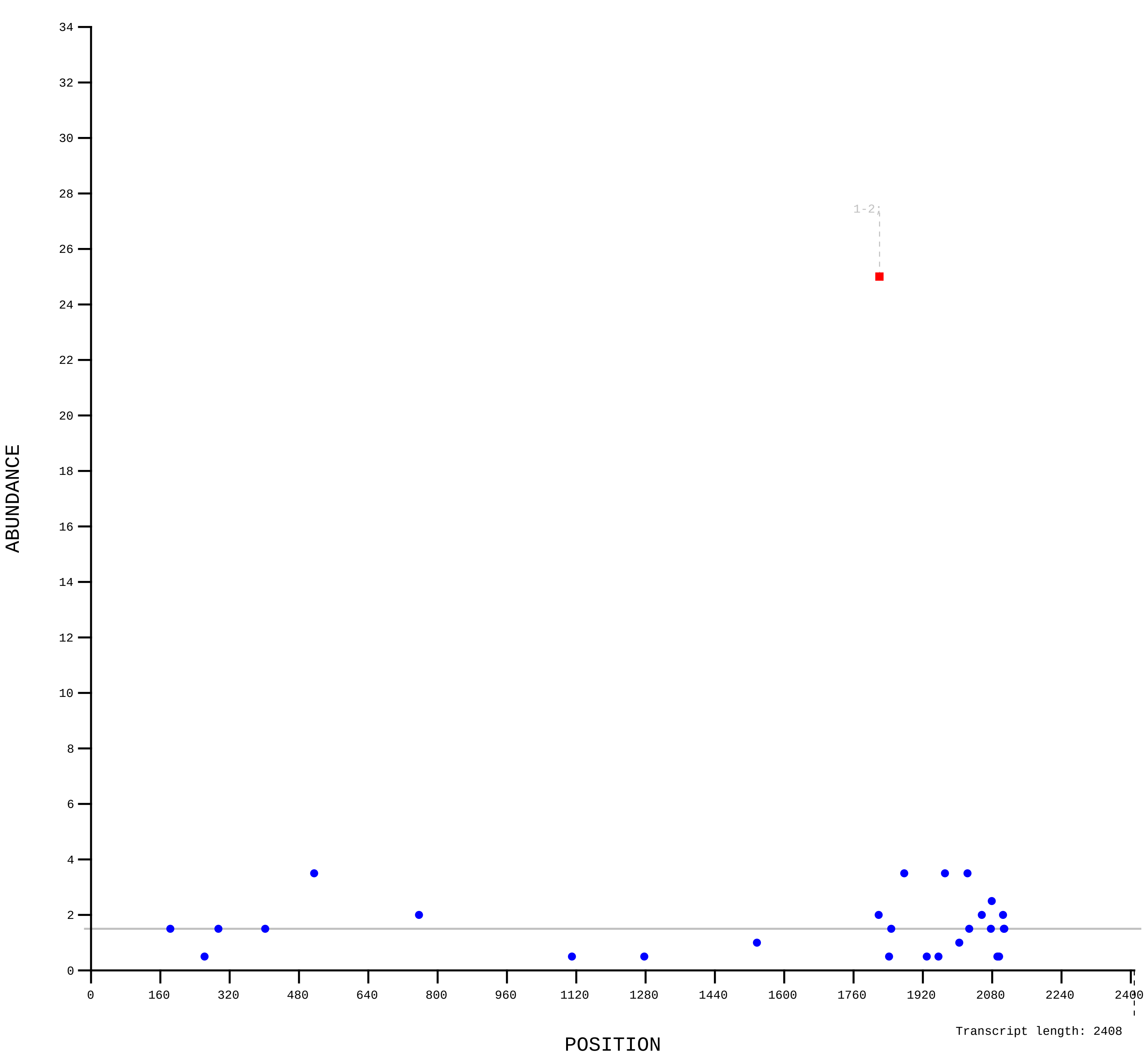

Category: 0 1 2 3 4
Degradome alignment: Median:

```
0  #1  Position:1820  Abundance: 25.00(deg) 1(sRNA)
   5'     AGAATCTTGATGATGCTGCAT        3'  ID:
            |||||o|||||||||||||o           Score: 2.0
   3' TTACCCTTAGGACTACTACGACGTGATCTATT 5'  p-value: 0.0

0  #2  Position:1820  Abundance: 25.00(deg) 1(sRNA)
   5'     AGAATCTTGATGATGCTGCAA        3'  ID:
            |||||o|||||||||||||            Score: 2.5
   3' TTACCCTTAGGACTACTACGACGTGATCTATT 5'  p-value: 0.0
```

Cs8g16440.1 gene=Cs8g16440 CDS=606-2780

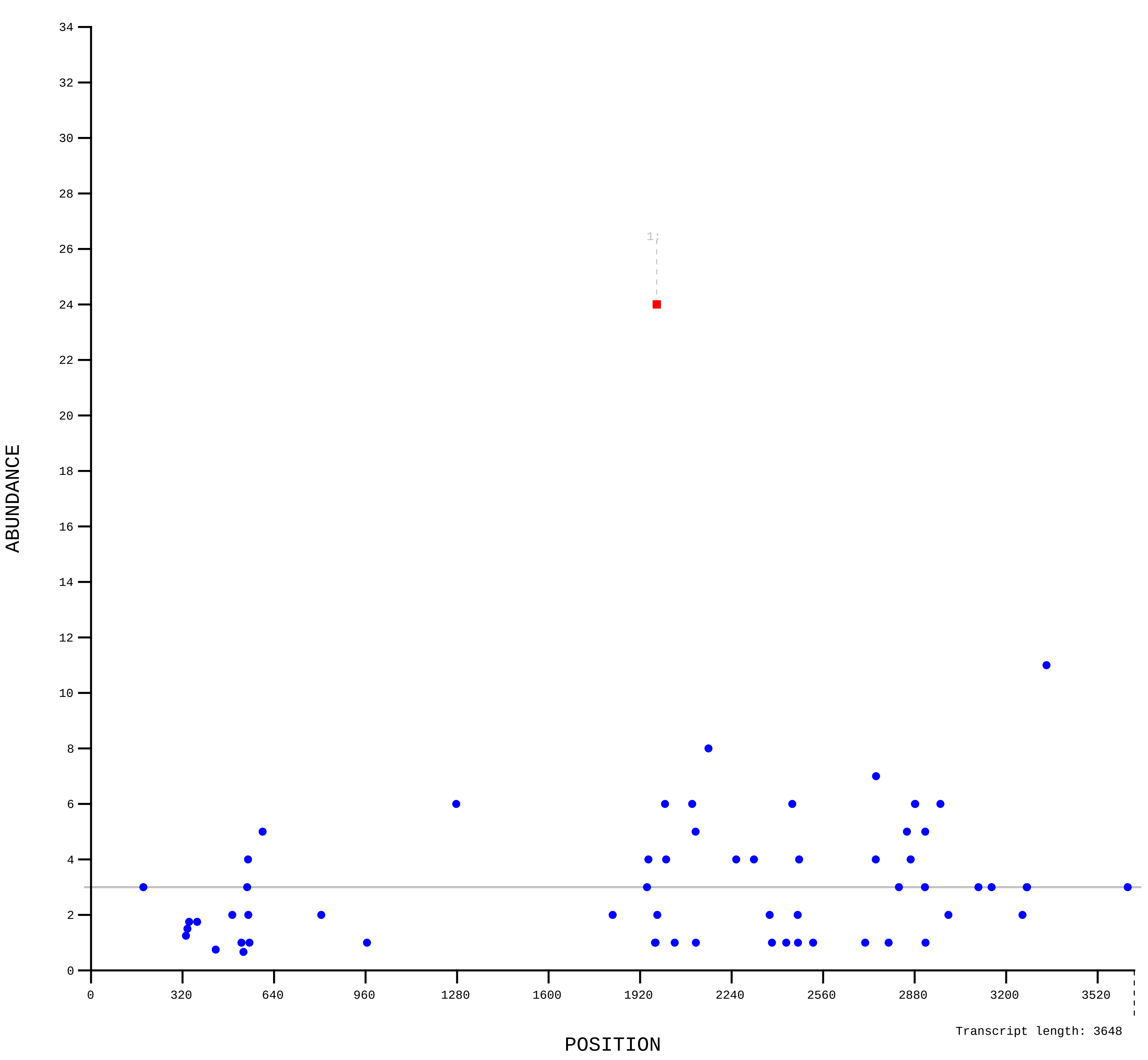

Category: 0 1 2 3 4
Degradome alignment: Median:

0 #1 Position:1978 Abundance: 24.00(deg) 1(sRNA)
5' TGCCTGGCTCCCTGTATGCCA 3' ID:
||||||||||||||||||||| Score: 1.0
3' TCGTACGGACCGAGGGACATACGGACGTCTTC 5' p-value: 0.0

Cs7g19540.1 gene=Cs7g19540 CDS=256-1848

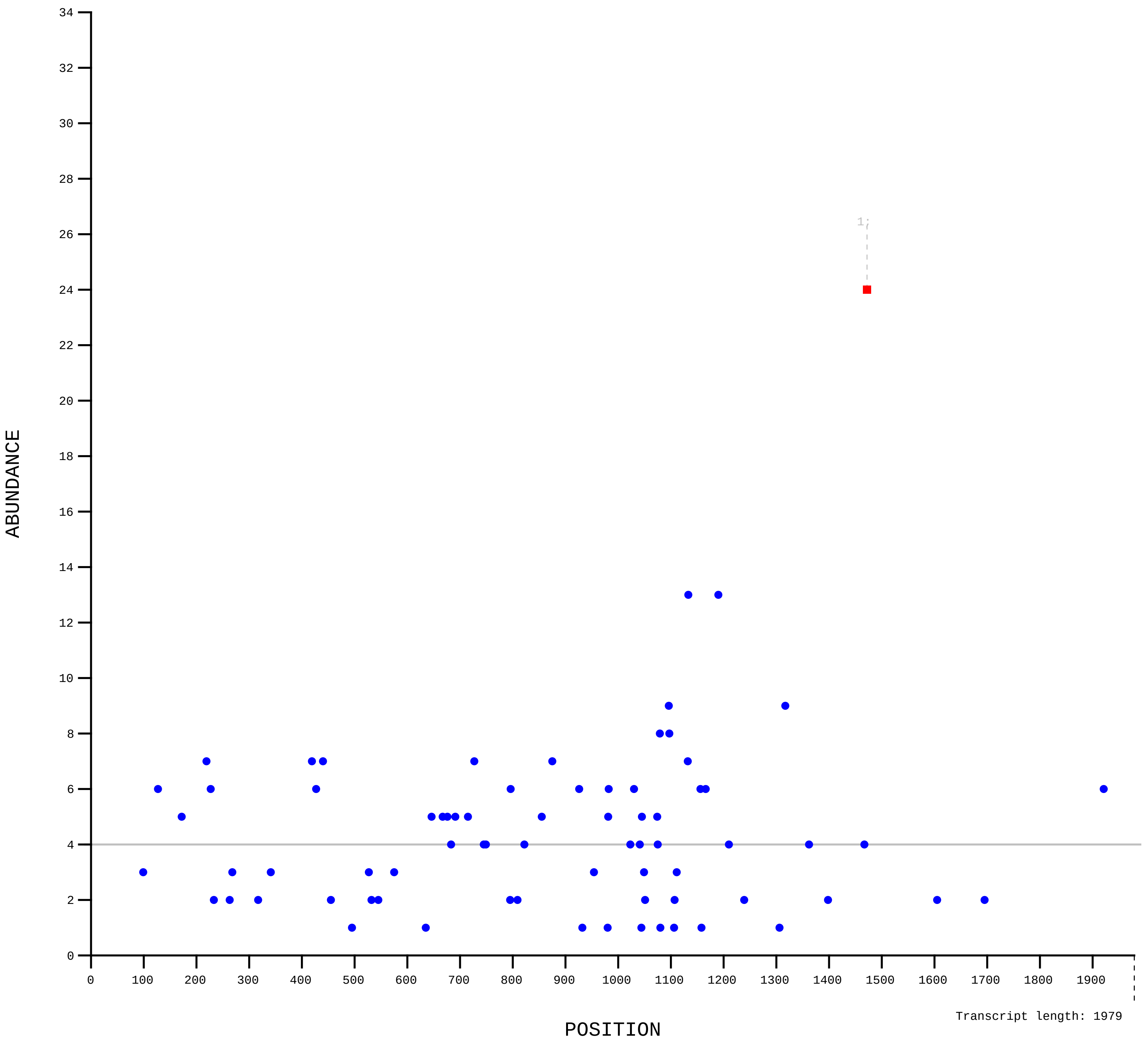

Category: 0 1 2 3 4
Degradome alignment: Median:

```
0  #1  Position:1472  Abundance: 24.00(deg) 1(sRNA)
   5'     TTTTTCCCACACCTCCCATCCC       3'  ID:
           |||| ||||||| || |||||           Score: 4.0
   3' AGACTAAAA-GGTGTGGTGGTTAGGGATATAC 5'  p-value: 0.0
```

Cs4g20380.2 gene=Cs4g20380 CDS=432-2516

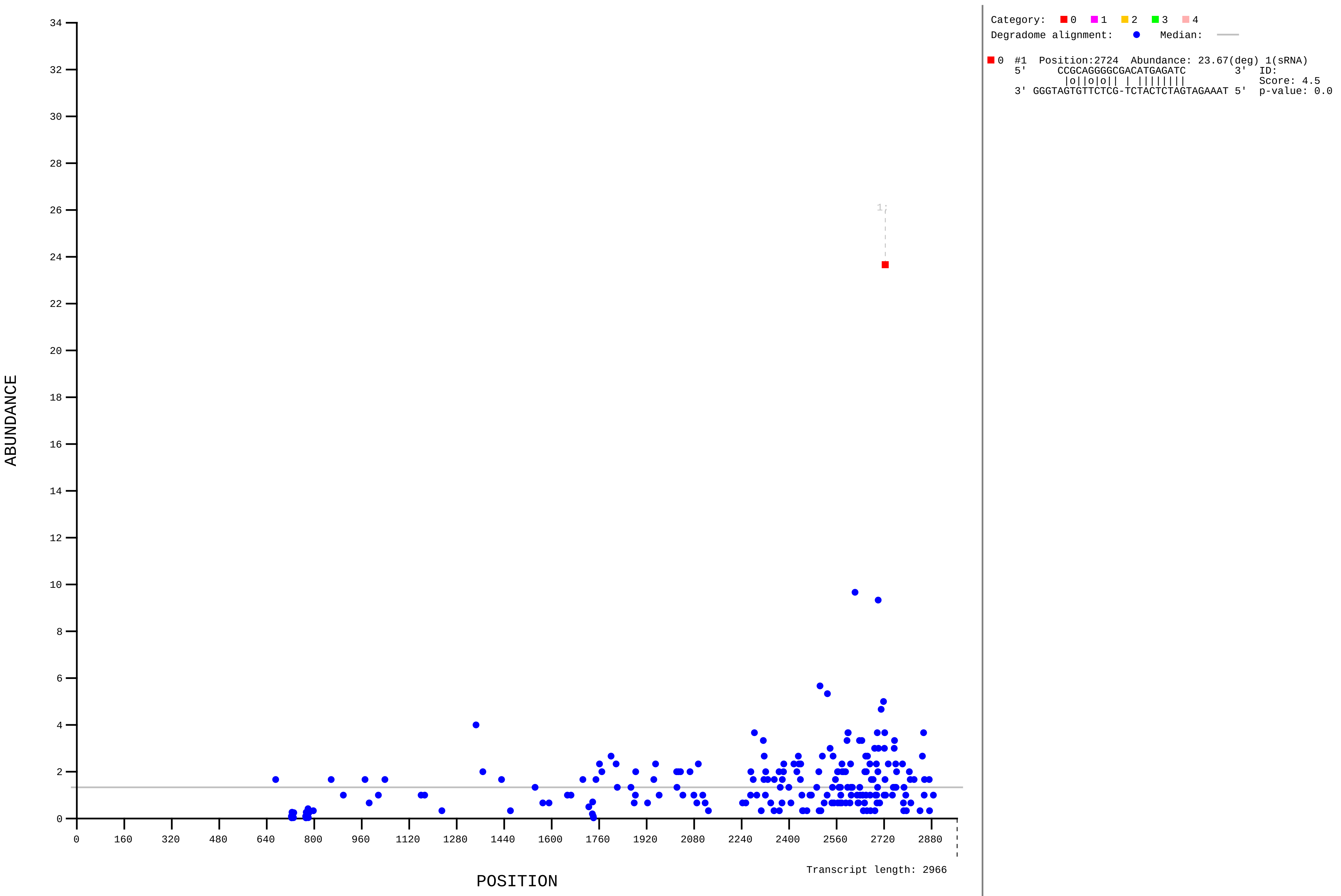

Cs4g20380.3 gene=Cs4g20380 CDS=432-3086

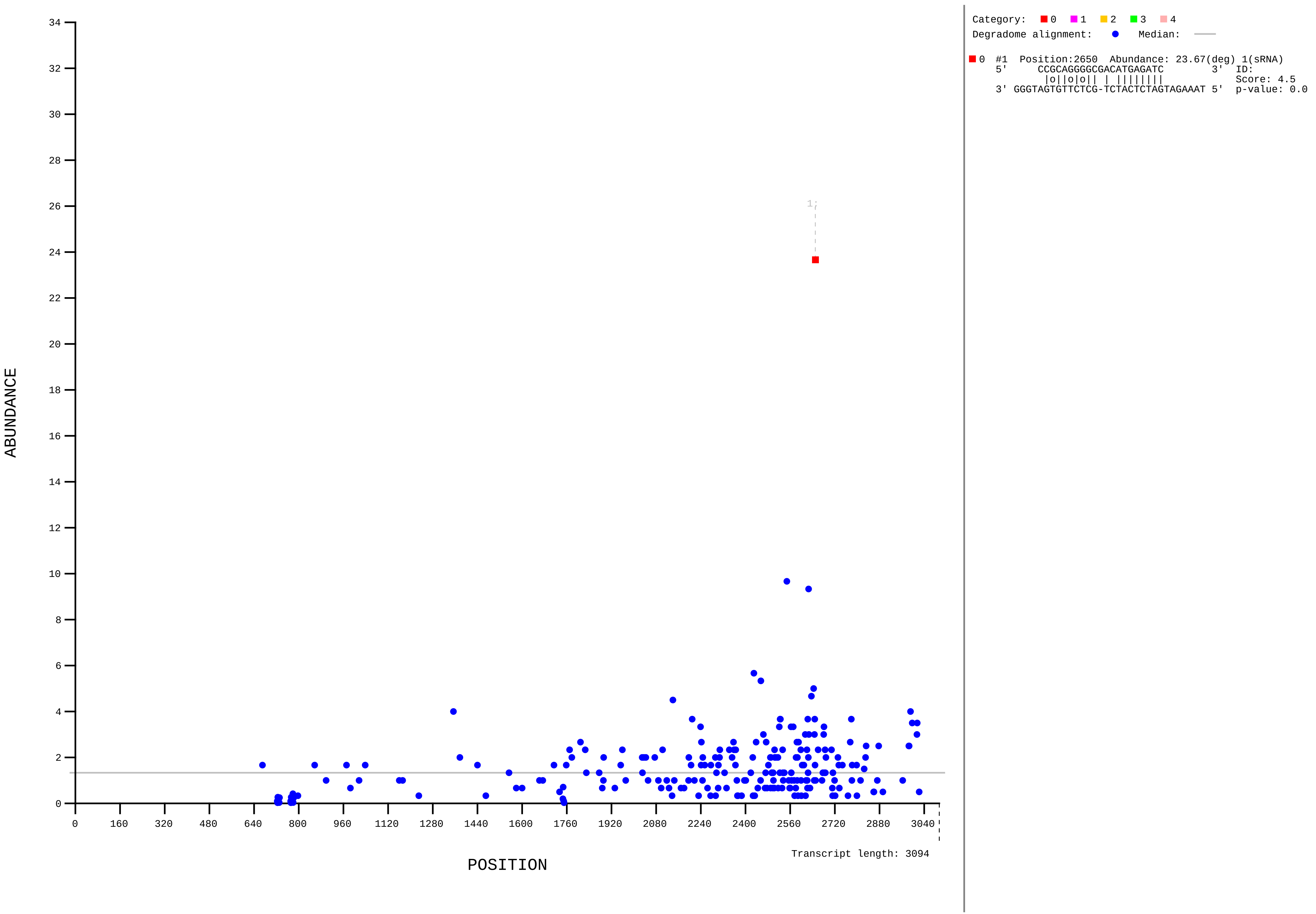

Cs1g03470.1 gene=Cs1g03470 CDS=1-909

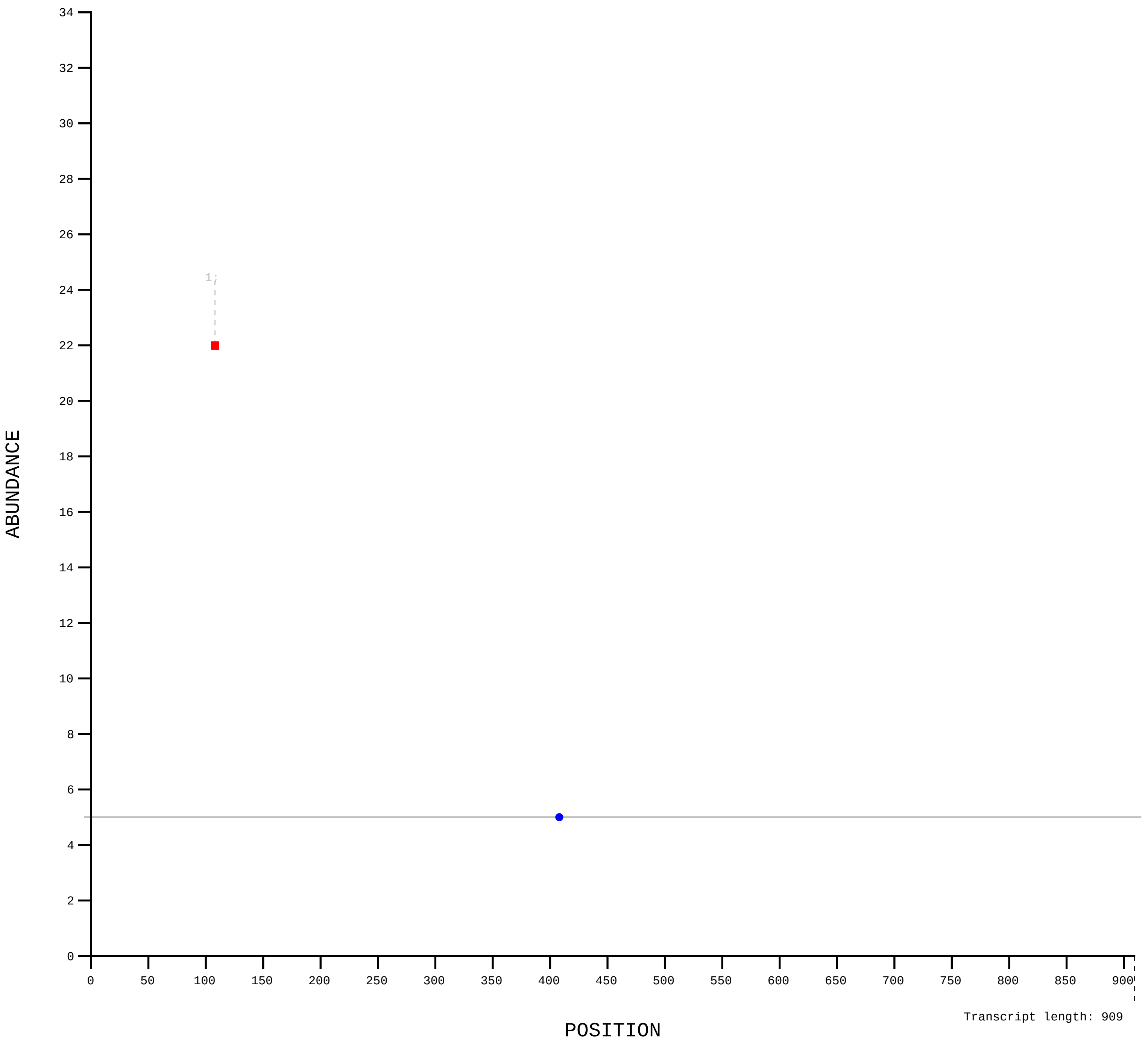


```
Category:  0  1  2  3  4
Degradome alignment:      Median:

0  #1  Position:108  Abundance: 22.00(deg) 2(sRNA)
   5'      TTTGGATTGAAGGGAGCTCTA        3'  ID:
           |||||||o||| |||||||o|            Score: 2.0
   3' CAGGAAACCTAGCTTACCTCGAGGTCAGGGAC 5'  p-value: 0.0
```

orange1.1t02254.1 gene=orange1.1t02254 CDS=544-1587

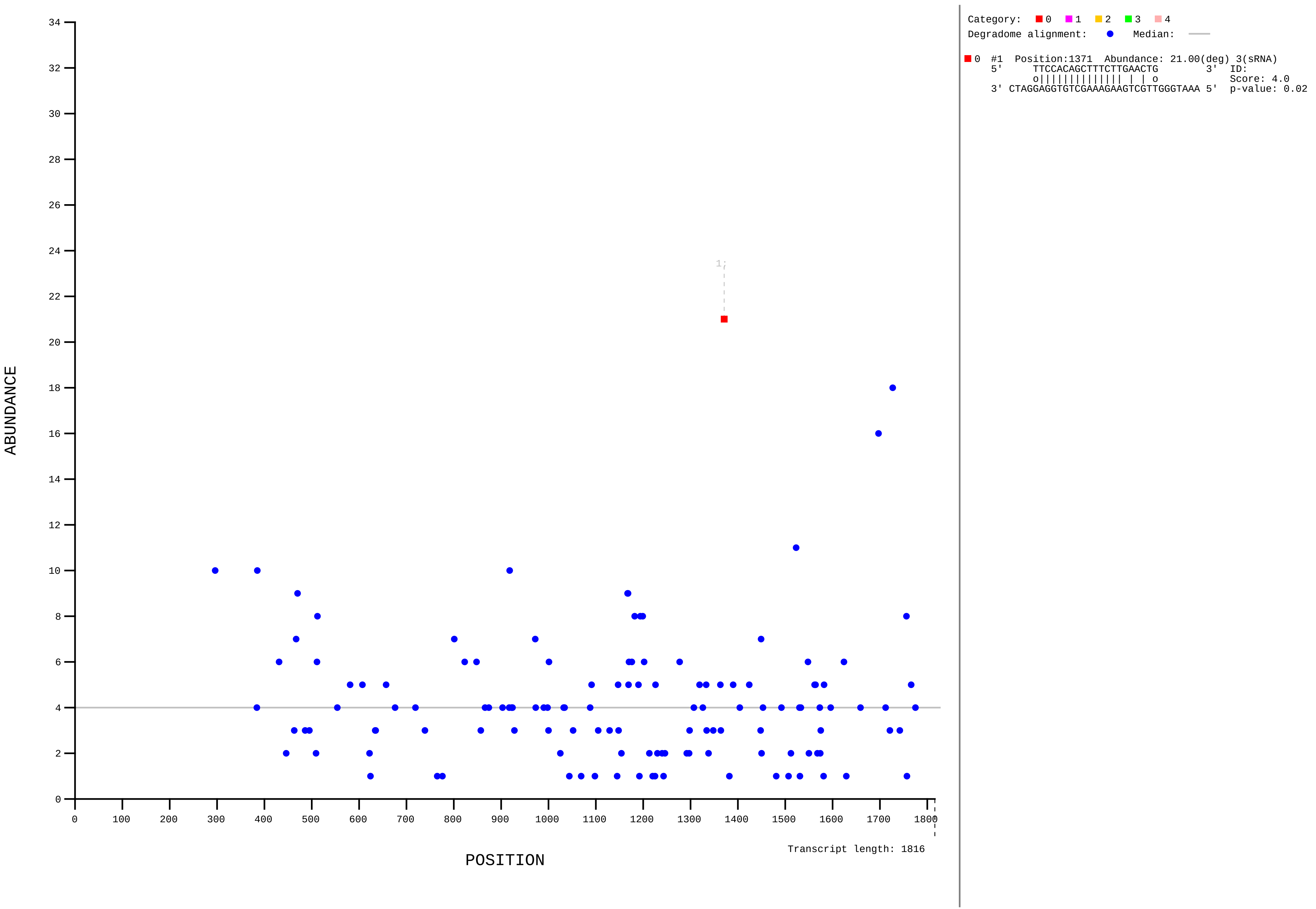

Cs5g19430.1 gene=Cs5g19430 CDS=1-3363

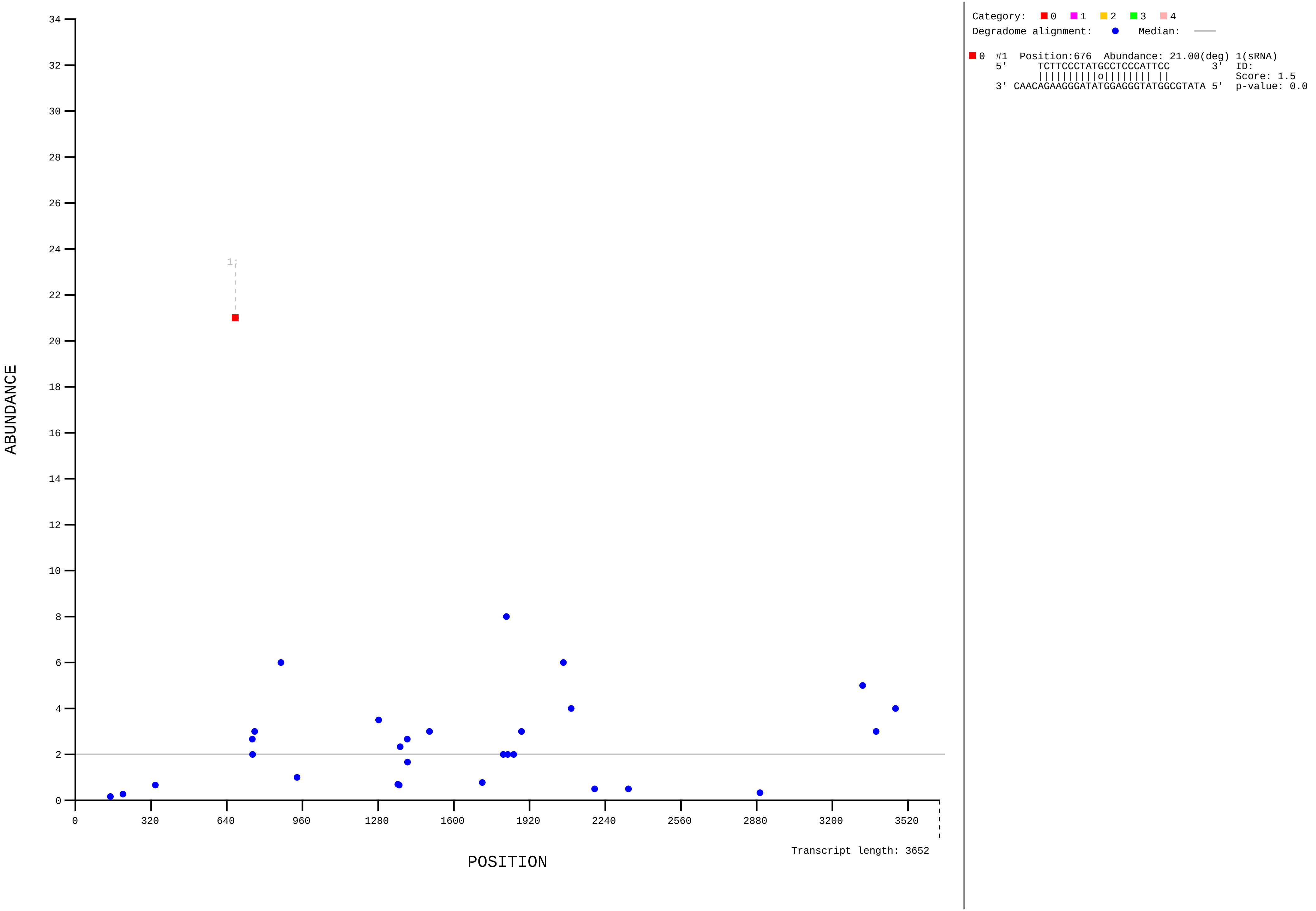

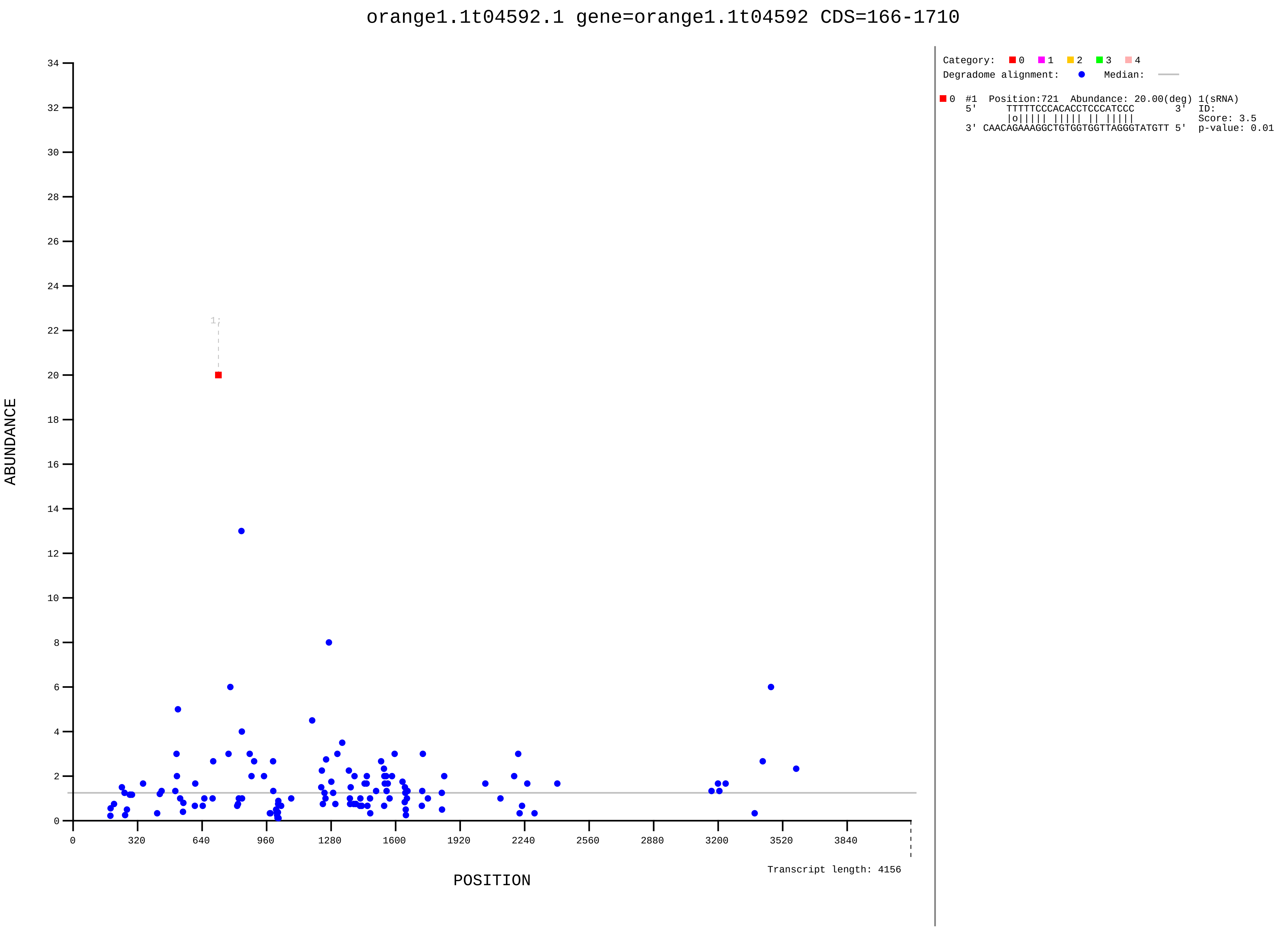
orange1.1t04592.1 gene=orange1.1t04592 CDS=166-1710
ABUNDANCE
POSITION
Transcript length: 4156
Category: 0 1 2 3 4
Degradome alignment:
Median:
0 #1 Position:721 Abundance: 20.00(deg) 1(sRNA)
5' TTTTTCCCACACCTCCCATCCC 3' ID:
|o||||| ||||| || |||||
Score: 3.5
3' CAACAGAAAGGCTGTGGTGGTTAGGGTATGTT 5' p-value: 0.01

Cs3g13690.1 gene=Cs3g13690 CDS=206-3688

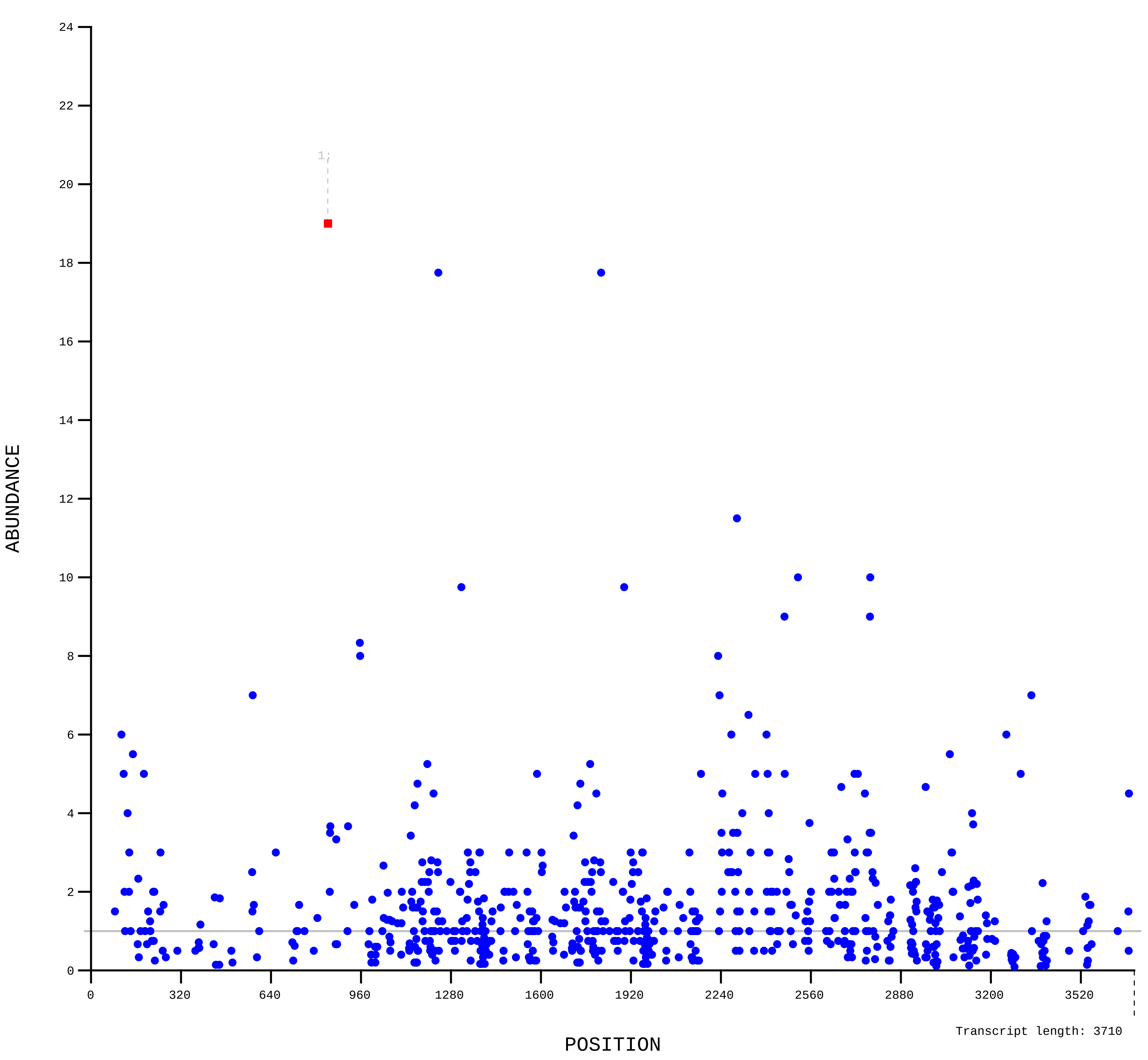

Category: 0 1 2 3 4
Degradome alignment: Median:

0 #1 Position:842 Abundance: 19.00(deg) 1(sRNA)

```
5'     TCTTCCCTATGCCTCCCATTCC       3'  ID:
       | ||  ||||||||o|||||o||          Score: 3.0
3' CAACAAAACGGATACGGGGGGTAGGGTTGTTC 5'  p-value: 0.0
```

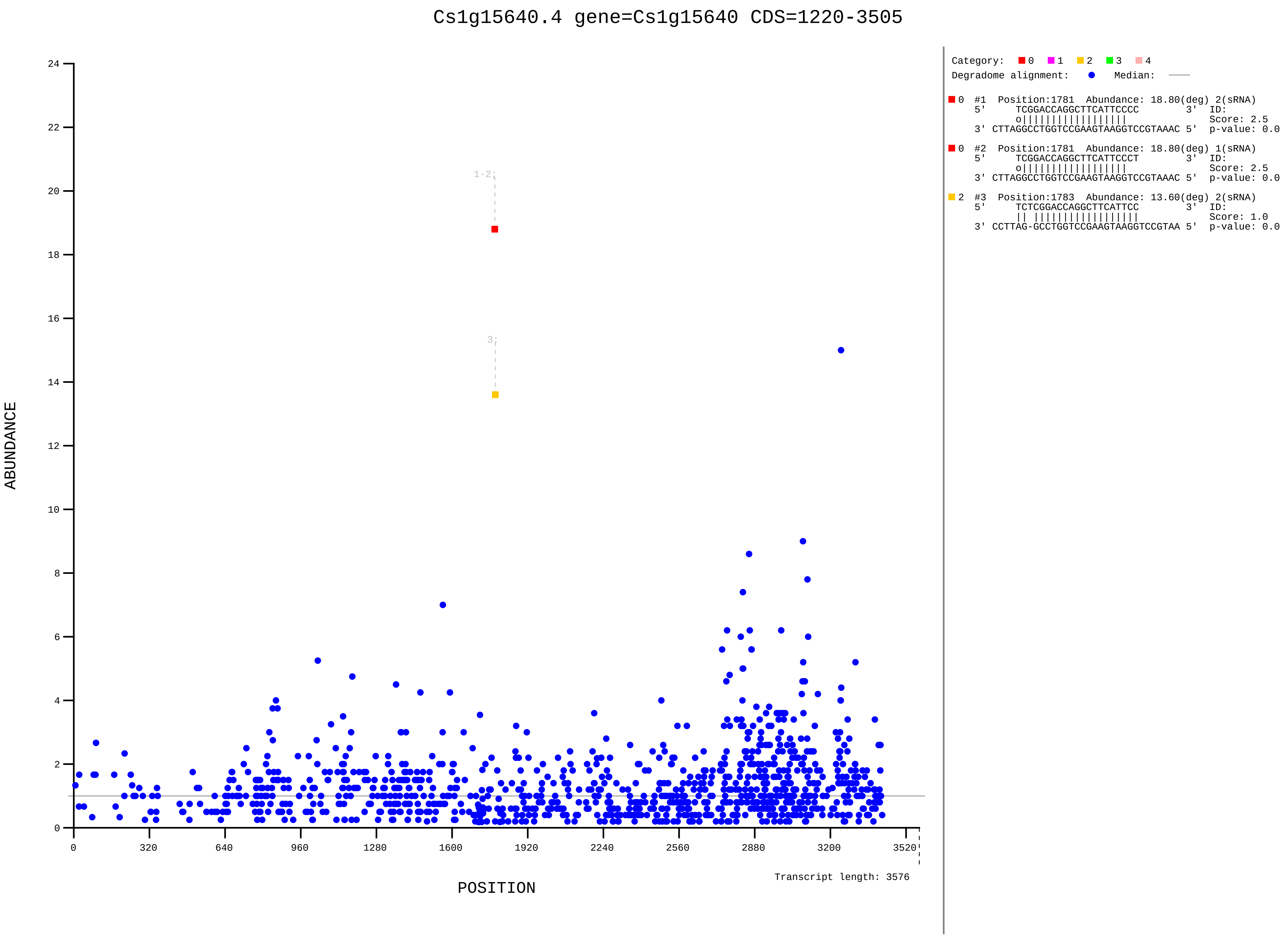

Cs1g15640.4 gene=Cs1g15640 CDS=1220-3505
ABUNDANCE
POSITION
0
2
4
6
8
10
12
14
16
18
20
22
24
0
320
640
960
1280
1600
1920
2240
2560
2880
3200
3520
1-2
3
Transcript length: 3576
Category: 0 1 2 3 4
Degradome alignment: Median:
0 #1 Position:1781 Abundance: 18.80(deg) 2(sRNA)
5' TCGGACCAGGCTTCATTCCCC 3' ID:
o||||||||||||||||||| Score: 2.5
3' CTTAGGCCTGGTCCGAAGTAAGGTCCGTAAAC 5' p-value: 0.0
0 #2 Position:1781 Abundance: 18.80(deg) 1(sRNA)
5' TCGGACCAGGCTTCATTCCCT 3' ID:
o||||||||||||||||||| Score: 2.5
3' CTTAGGCCTGGTCCGAAGTAAGGTCCGTAAAC 5' p-value: 0.0
2 #3 Position:1783 Abundance: 13.60(deg) 2(sRNA)
5' TCTCGGACCAGGCTTCATTCC 3' ID:
|| |||||||||||||||||| Score: 1.0
3' CCTTAG-GCCTGGTCCGAAGTAAGGTCCGTAA 5' p-value: 0.0

Cs1g15640.2 gene=Cs1g15640 CDS=1220-3649

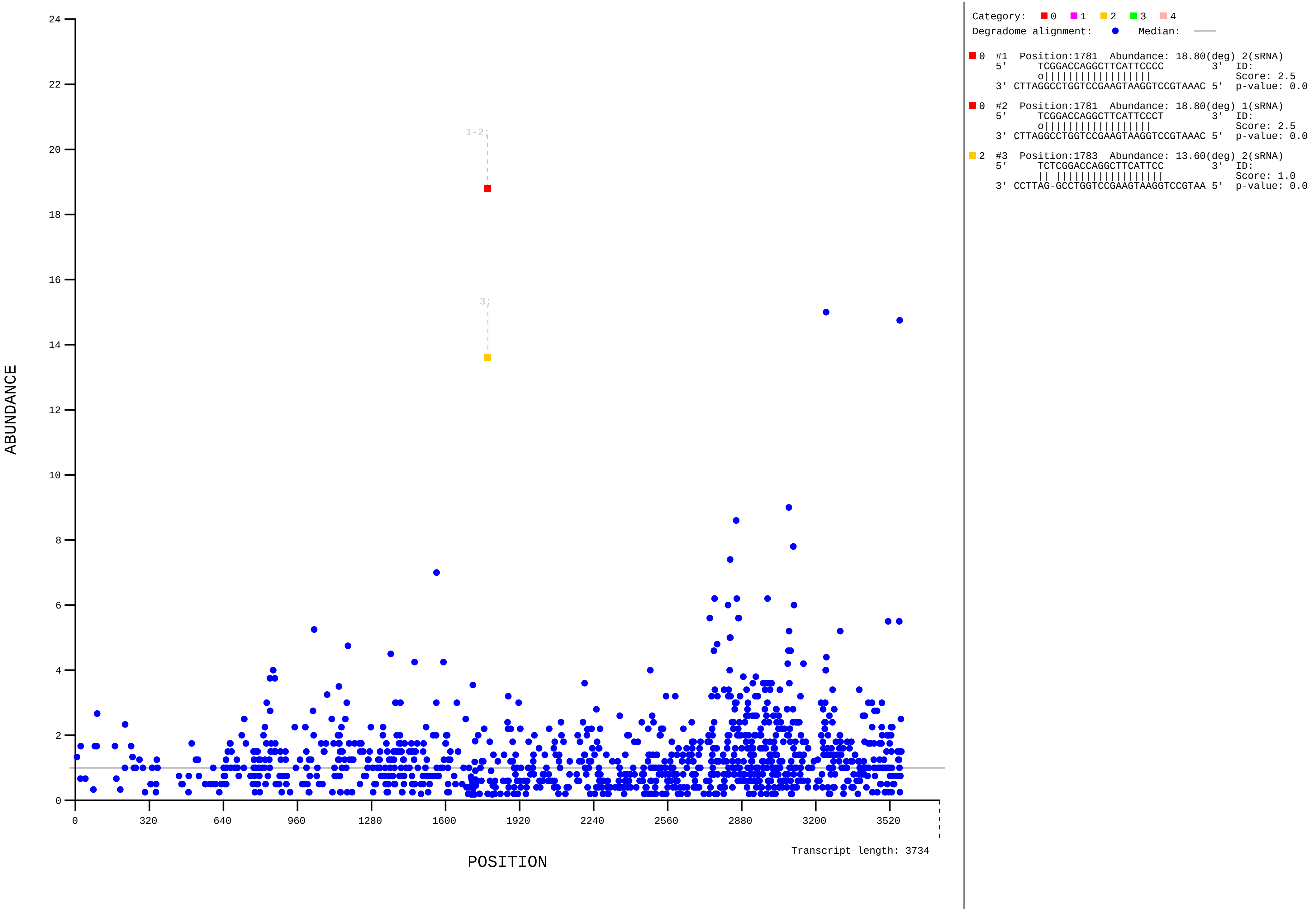

orange1.1t01918.1 gene=orange1.1t01918 CDS=1-2835

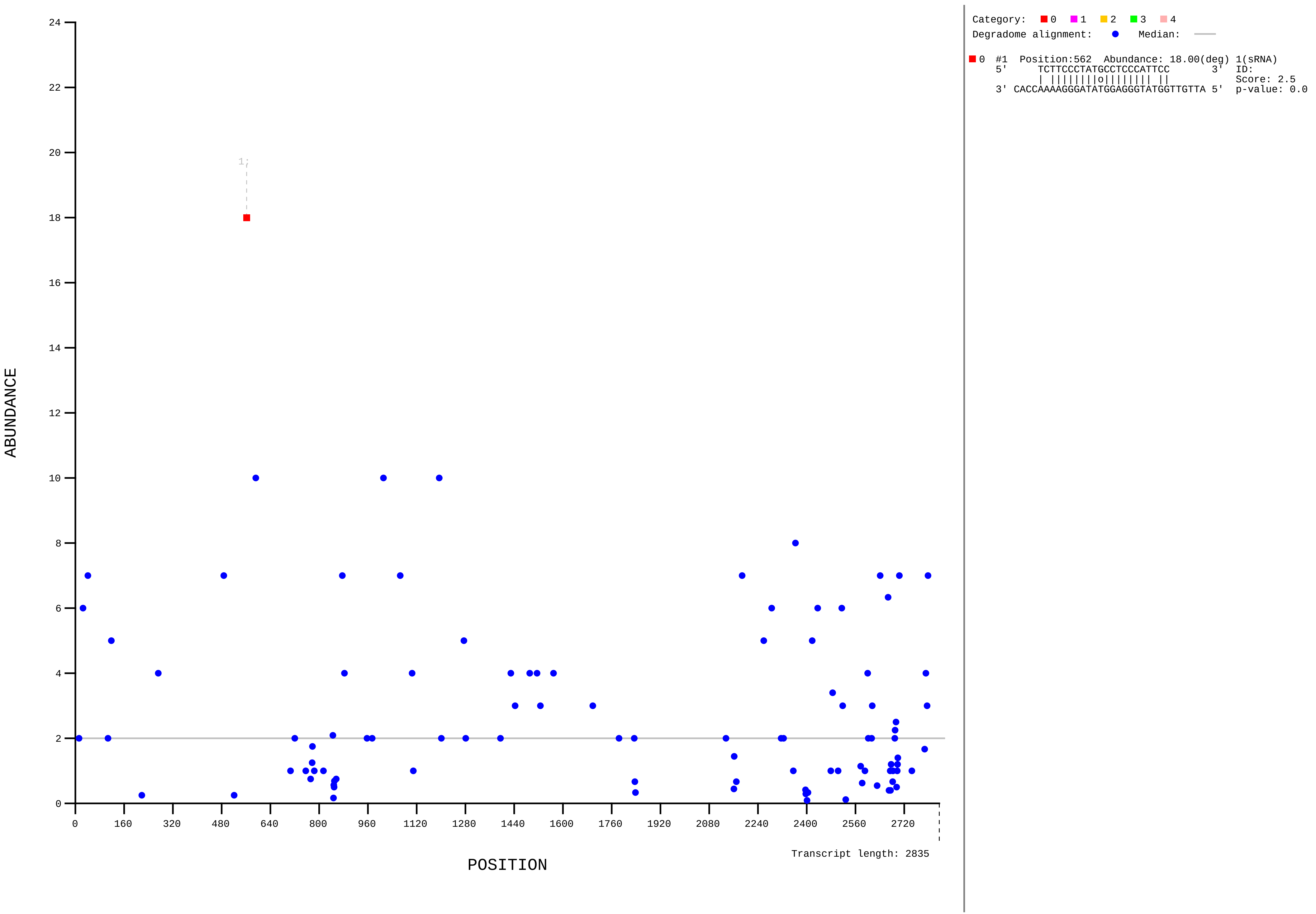

Cs3g11110.1 gene=Cs3g11110 CDS=265-1095

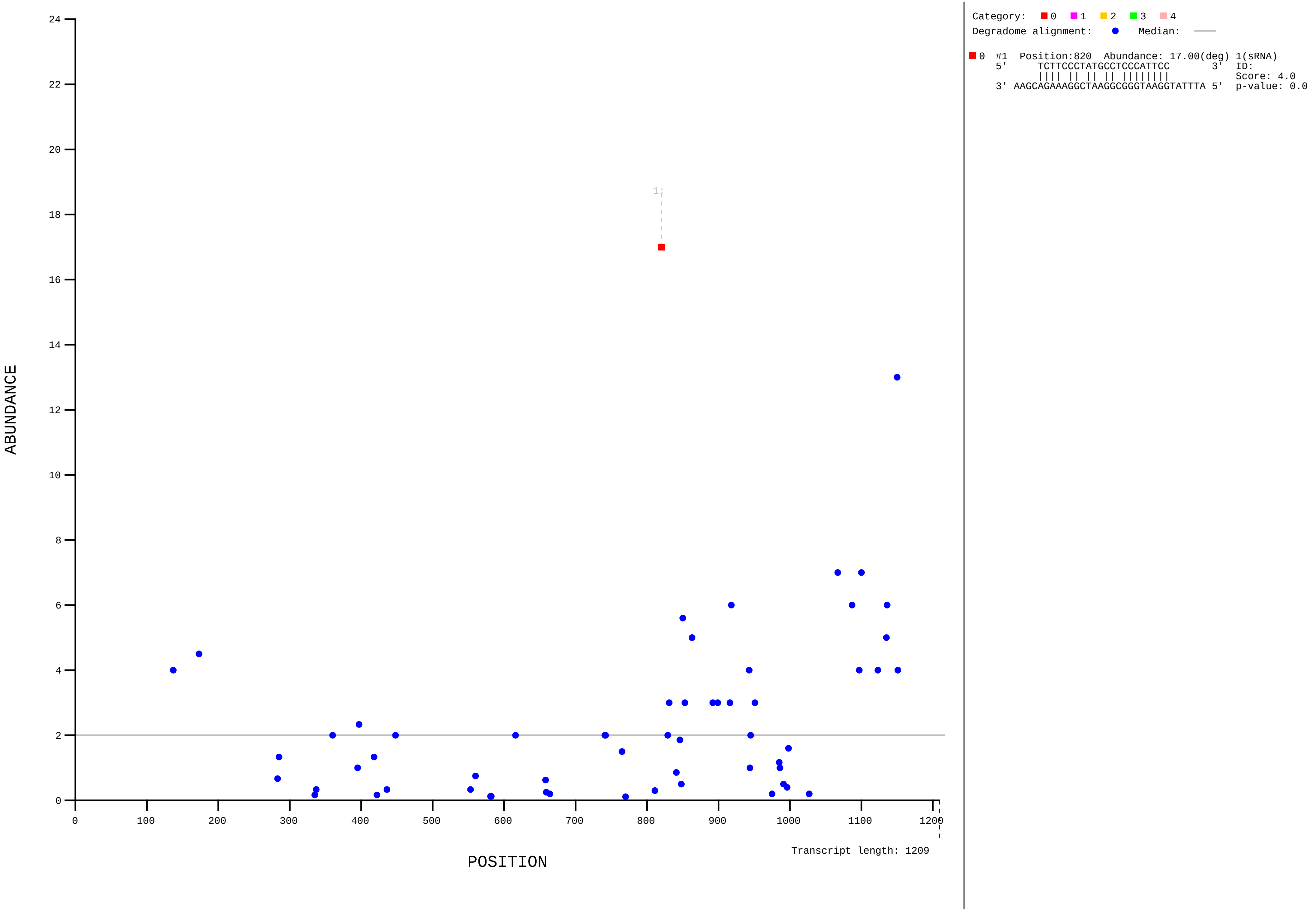

Cs1g08330.1 gene=Cs1g08330 CDS=1-3342

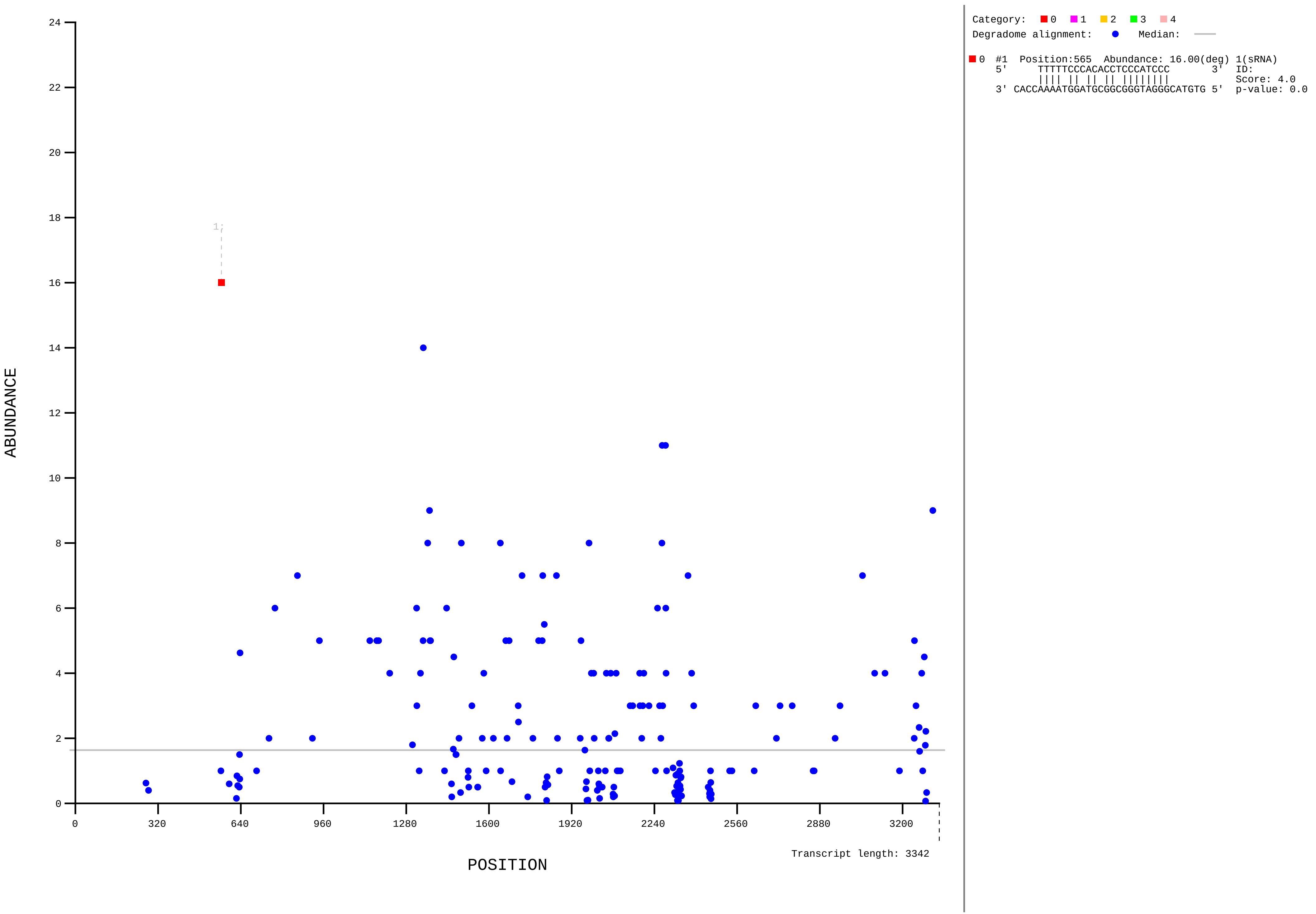

Cs3g22150.1 gene=Cs3g22150 CDS=171-1259

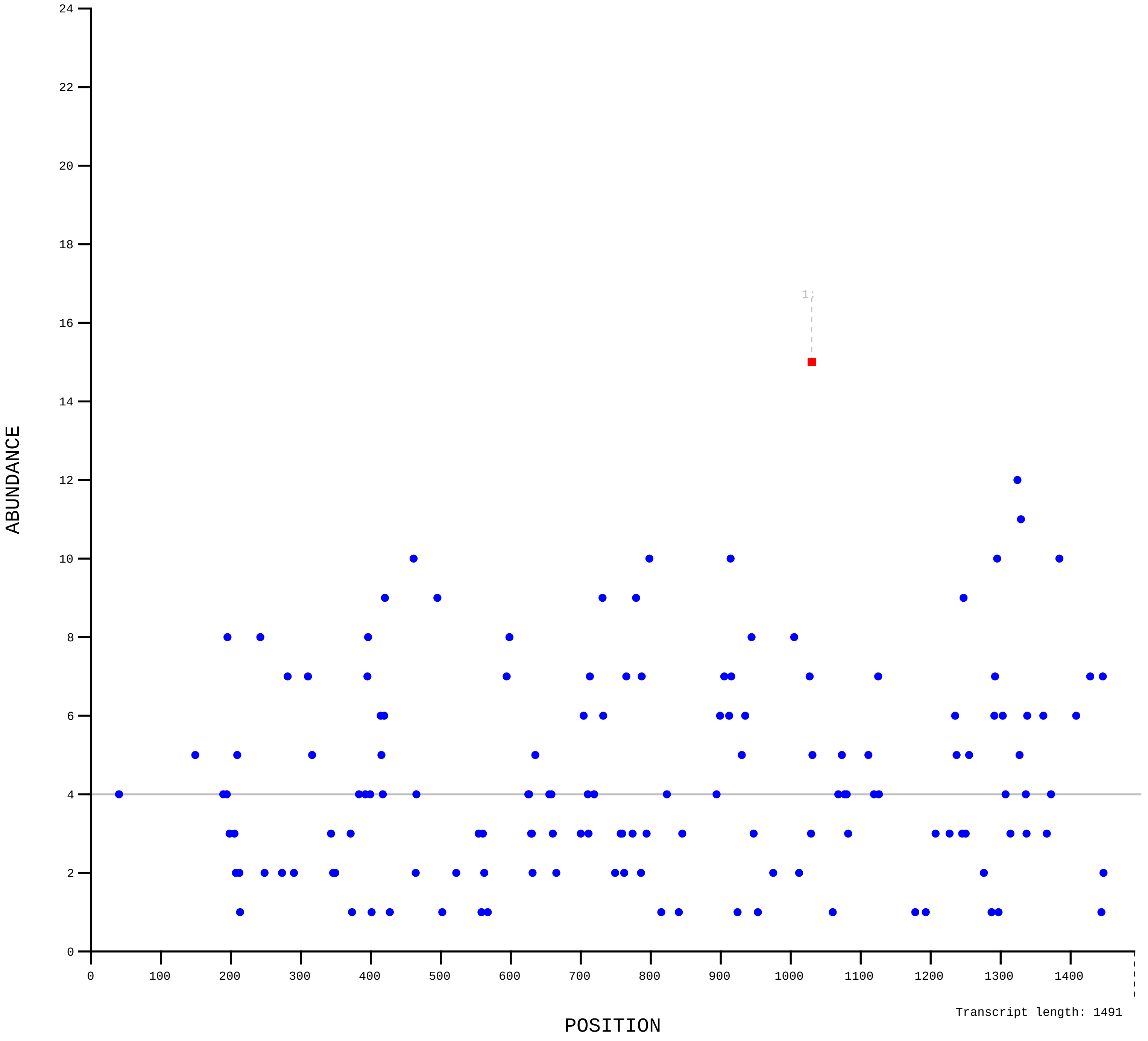

Category: ■0 ■1 ■2 ■3 ■4
Degradome alignment: ● Median: —

■0 #1 Position:1030 Abundance: 15.00(deg) 1(sRNA)

```
5'     ACAAGACCATAGATAGACCAT        3'  ID:
       |||||| ||| || |o| |||            Score: 4.5
3' ACAGTGTTCTAGTAACTTTTTCGTAGATCATA 5'  p-value: 0.0
```

orange1.1t03734.1 gene=orange1.1t03734 CDS=197-2974

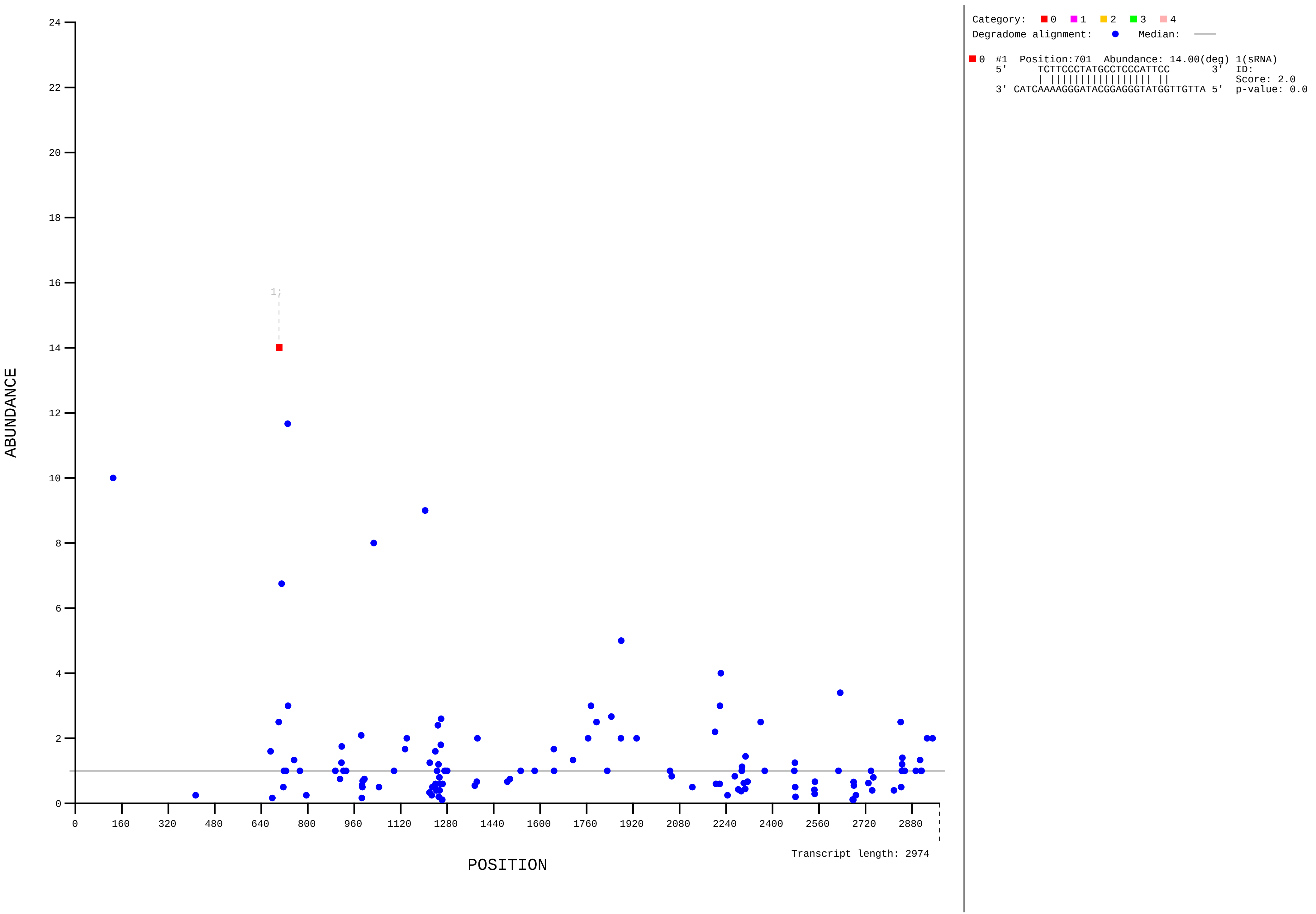

orange1.1t01898.1 gene=orange1.1t01898 CDS=1-2658

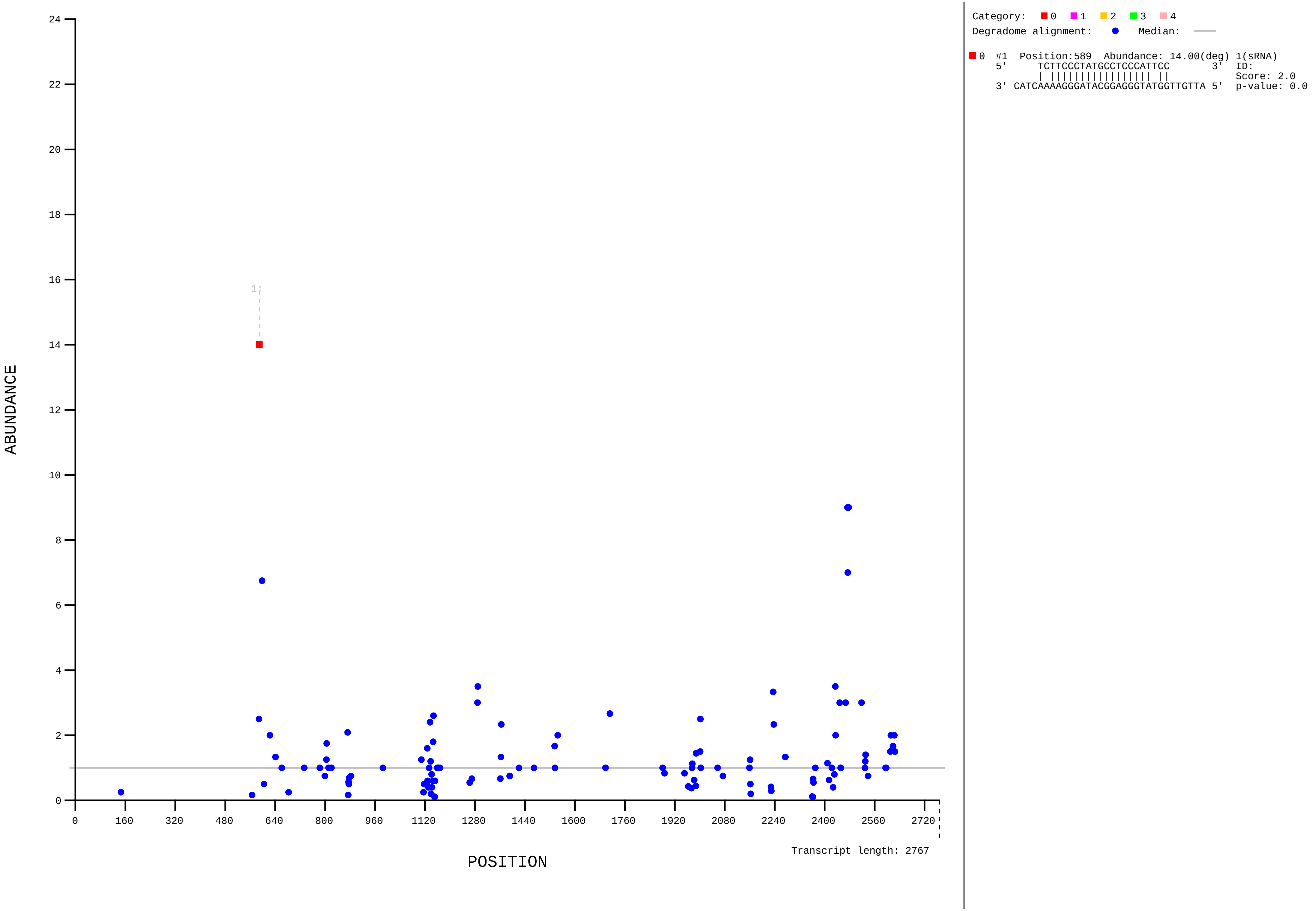

orange1.1t01362.1 gene=orange1.1t01362 CDS=205-2940

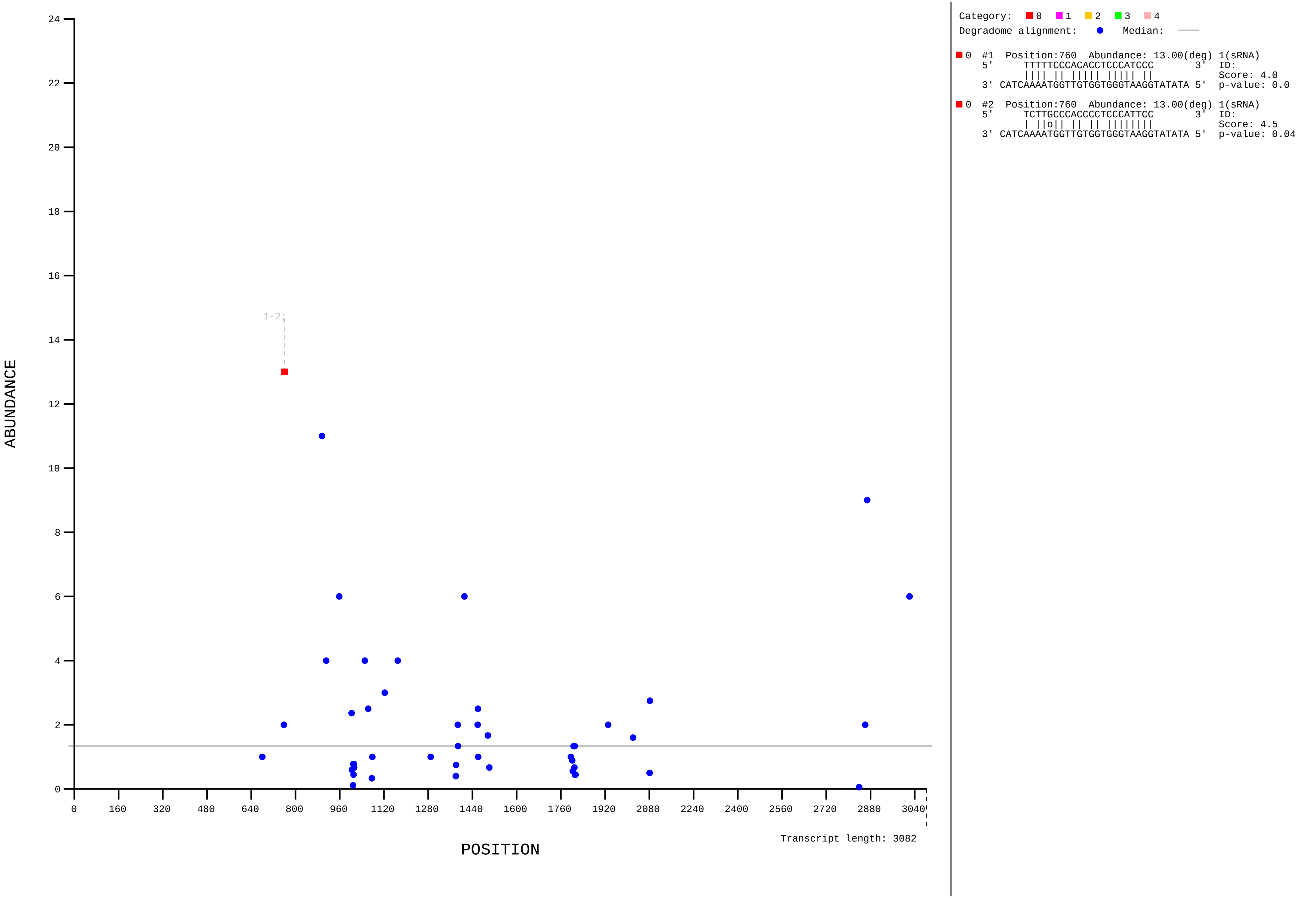

orange1.1t01829.2 gene=orange1.1t01829 CDS=135-3863

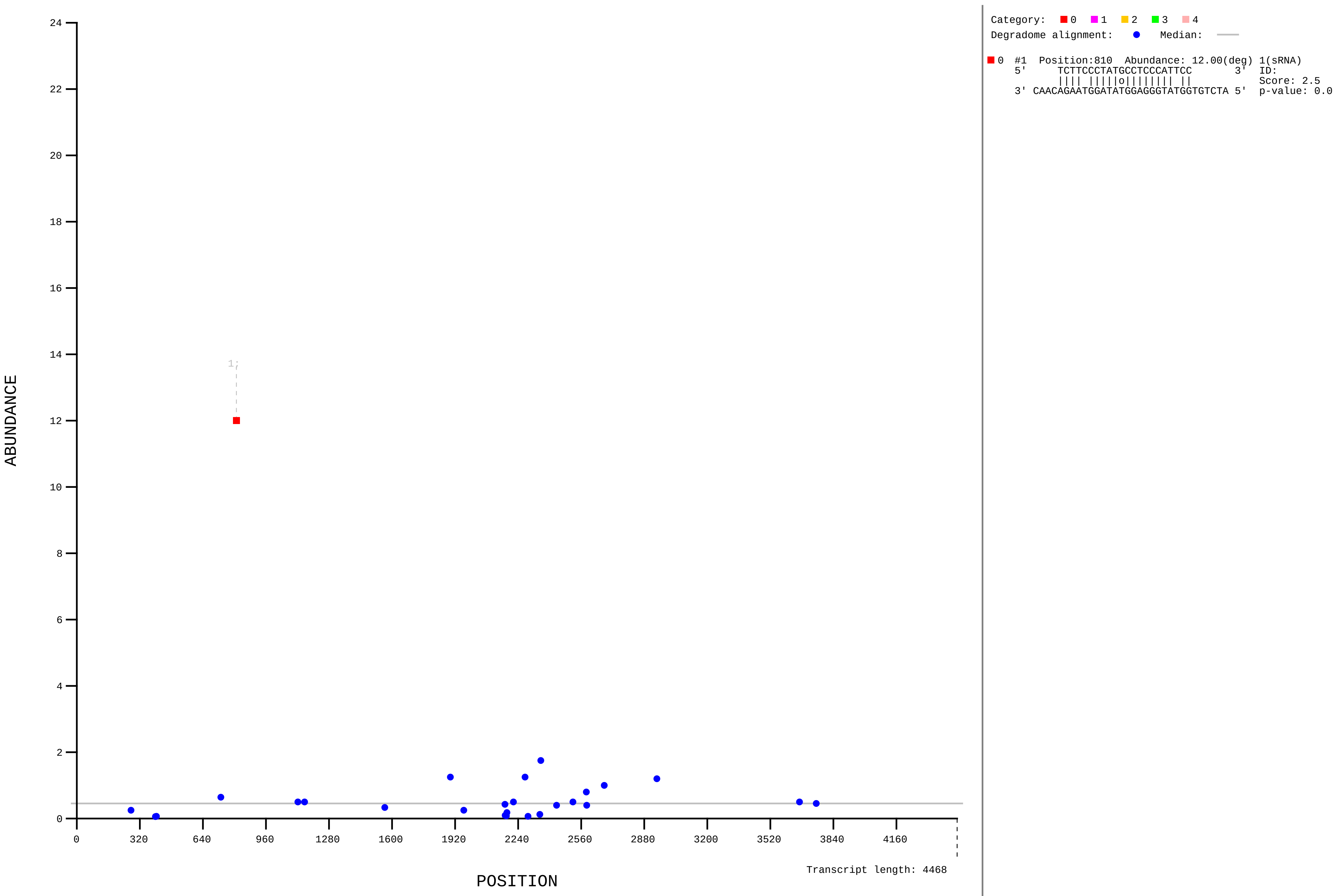

orange1.1t01829.1 gene=orange1.1t01829 CDS=135-3863

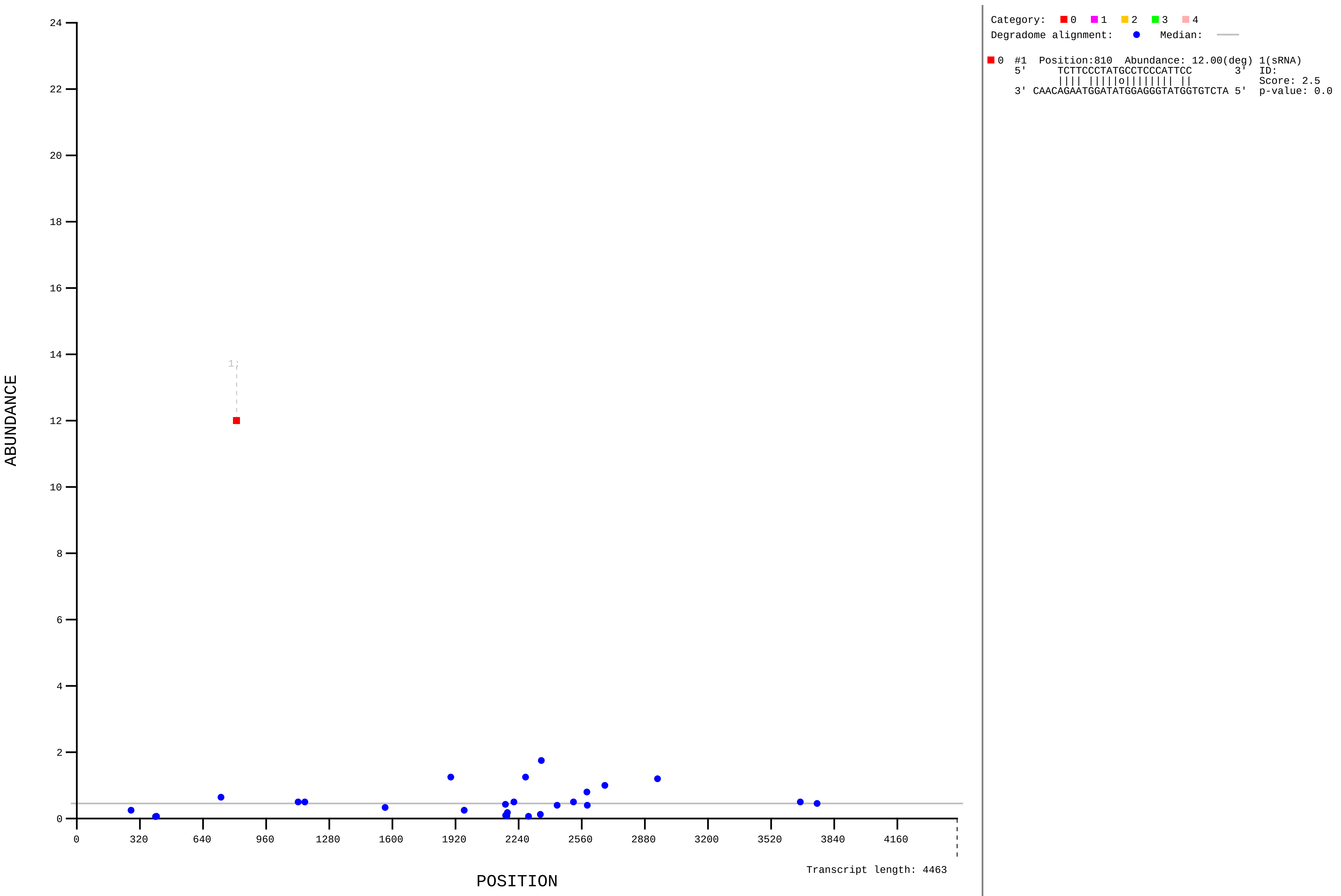

orange1.1t01829.3 gene=orange1.1t01829 CDS=135-3863

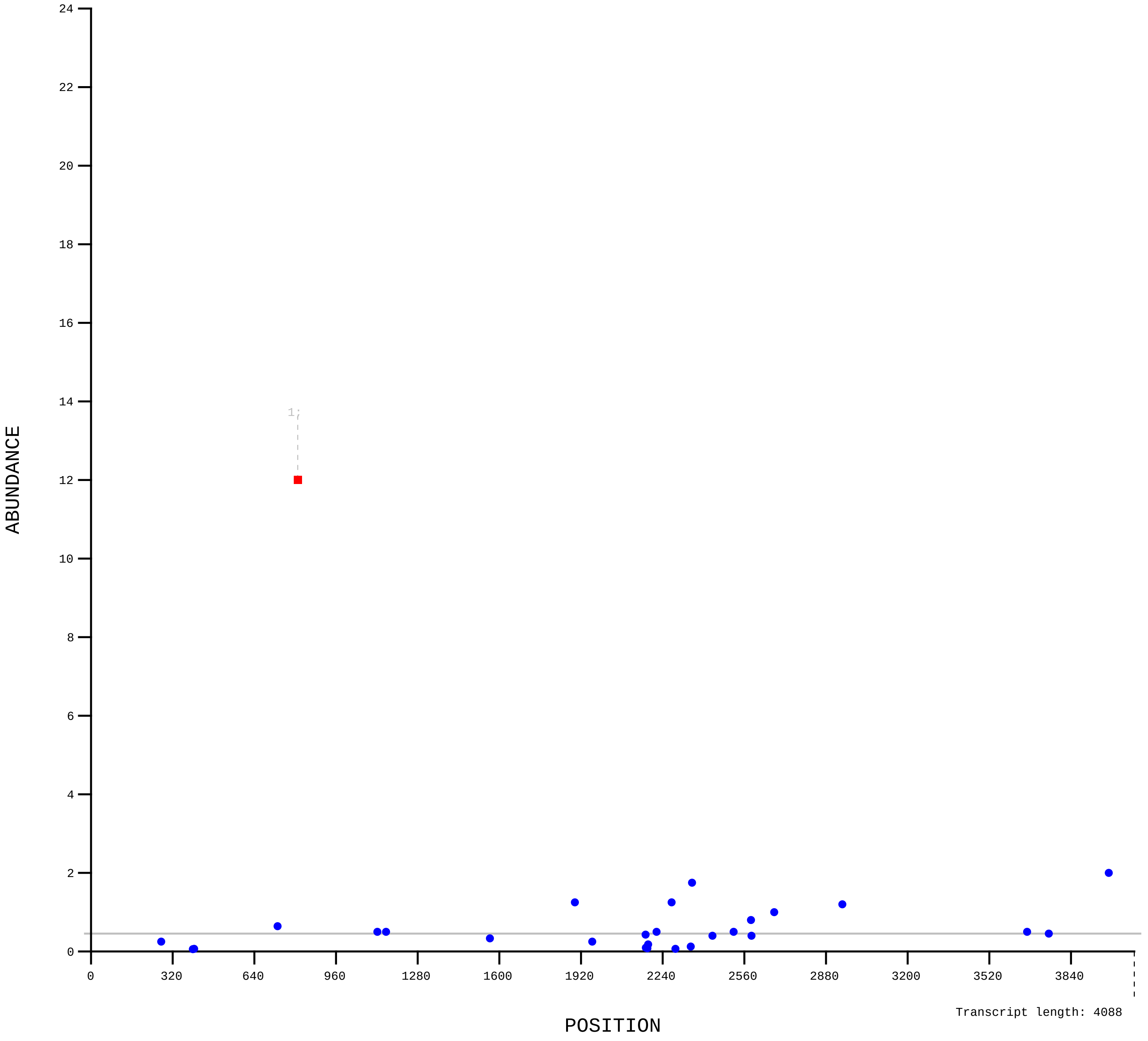

Category: 0 1 2 3 4
Degradome alignment: Median:

```
0  #1  Position:810  Abundance: 12.00(deg) 1(sRNA)
   5'     TCTTCCCTATGCCTCCCATTCC       3'  ID:
          |||| |||||o|||||||| ||           Score: 2.5
   3' CAACAGAATGGATATGGAGGGTATGGTGTCTA 5'  p-value: 0.0
```

orange1.1t01829.4 gene=orange1.1t01829 CDS=135-3905

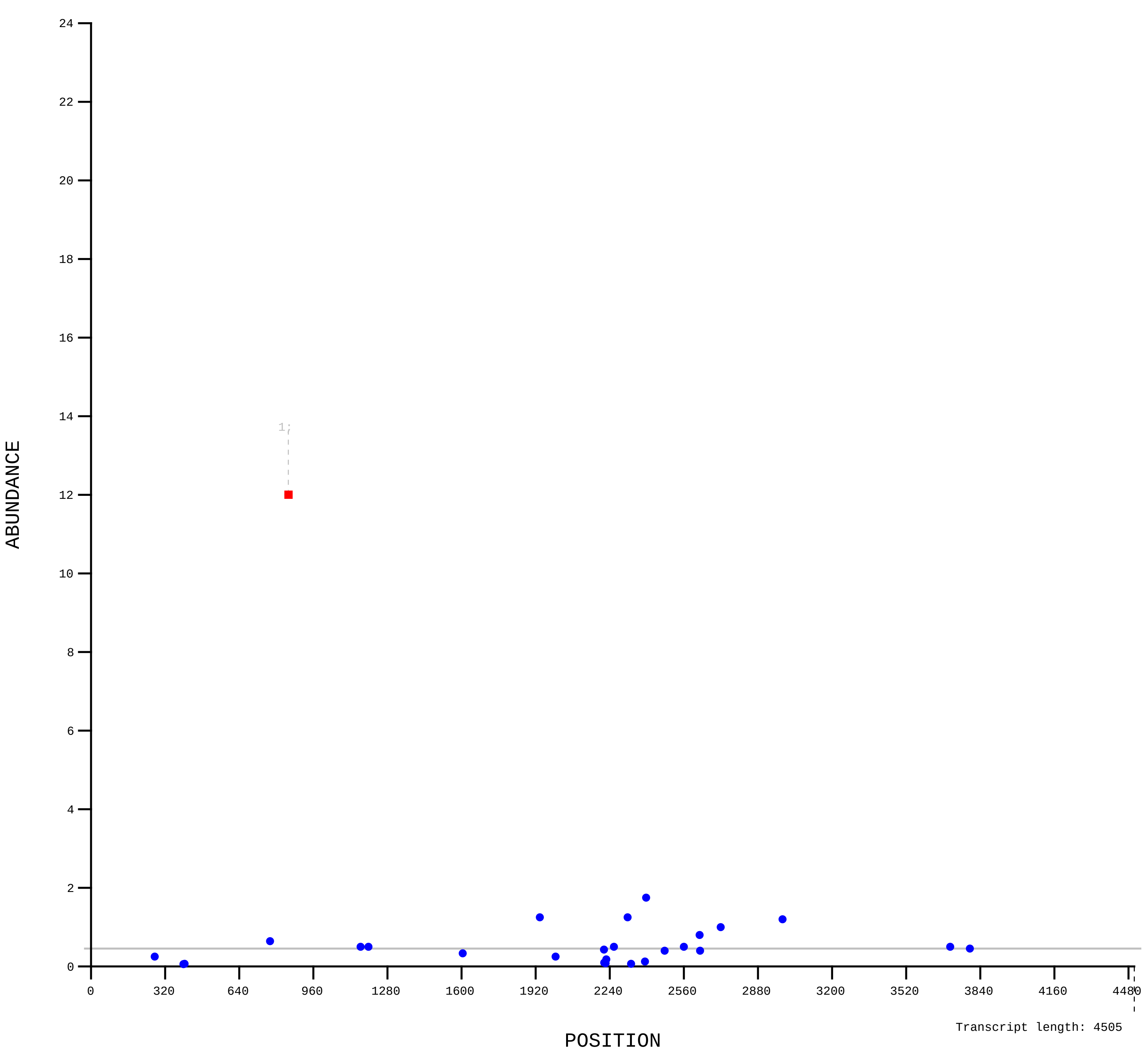

Category: 0 1 2 3 4
Degradome alignment: Median:

0 #1 Position:852 Abundance: 12.00(deg) 1(sRNA)

```
5'     TCTTCCCTATGCCTCCCATTCC       3'  ID:
       |||| |||||o|||||||| ||           Score: 2.5
3' CAACAGAATGGATATGGAGGGTATGGTGTCTA 5'  p-value: 0.0
```

orange1.1t02518.1 gene=orange1.1t02518 CDS=454-3636

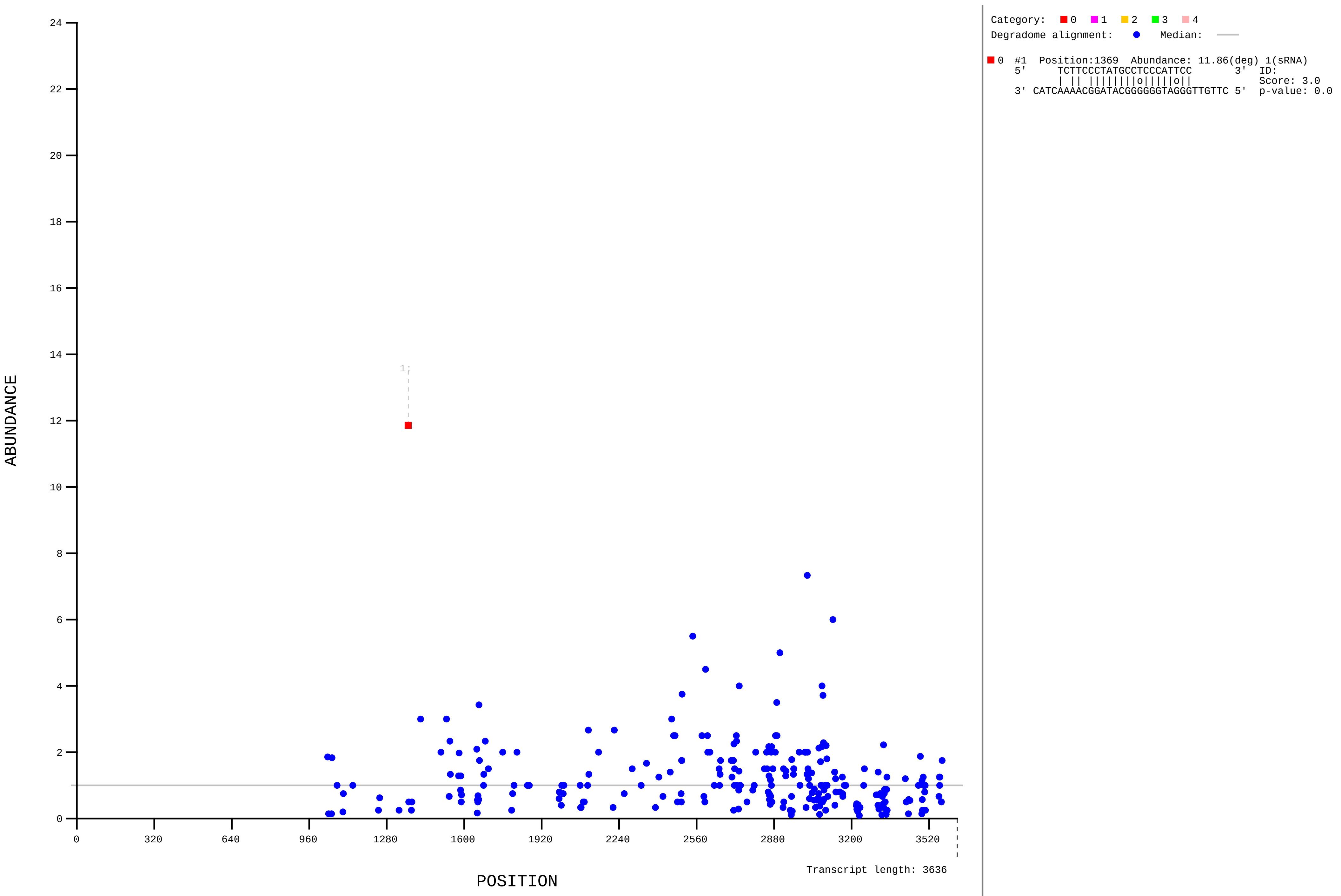

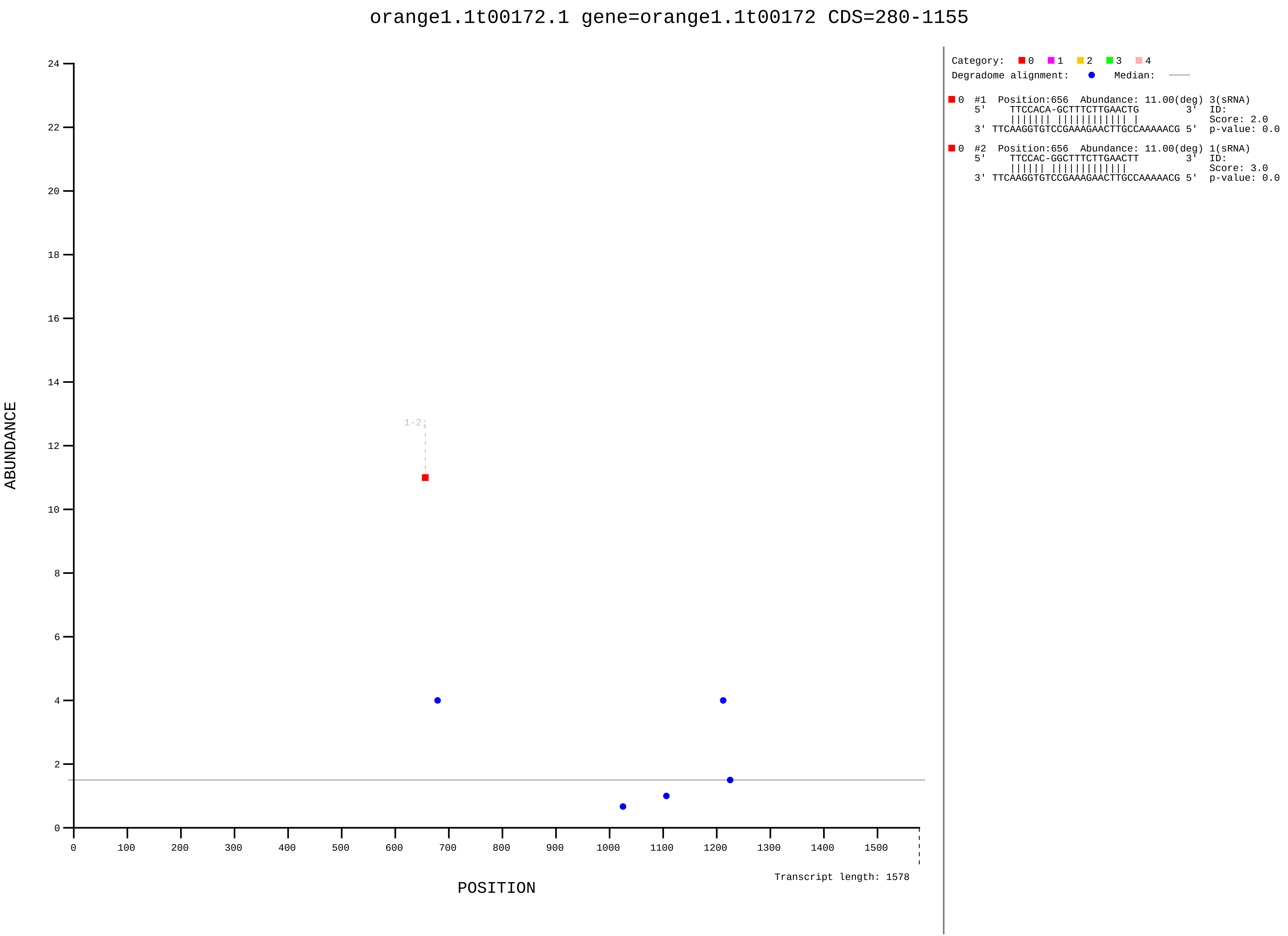
orange1.1t00172.1 gene=orange1.1t00172 CDS=280-1155
ABUNDANCE
POSITION
Transcript length: 1578
1-2
Category: 0 1 2 3 4
Degradome alignment:
Median:
0 #1 Position:656 Abundance: 11.00(deg) 3(sRNA)
5' TTCCACA-GCTTTCTTGAACTG 3' ID:
Score: 2.0
3' TTCAAGGTGTCCGAAAGAACTTGCCAAAAACG 5' p-value: 0.0
0 #2 Position:656 Abundance: 11.00(deg) 1(sRNA)
5' TTCCAC-GGCTTTCTTGAACTT 3' ID:
Score: 3.0
3' TTCAAGGTGTCCGAAAGAACTTGCCAAAAACG 5' p-value: 0.0

orange1.1t00172.3 gene=orange1.1t00172 CDS=280-1248

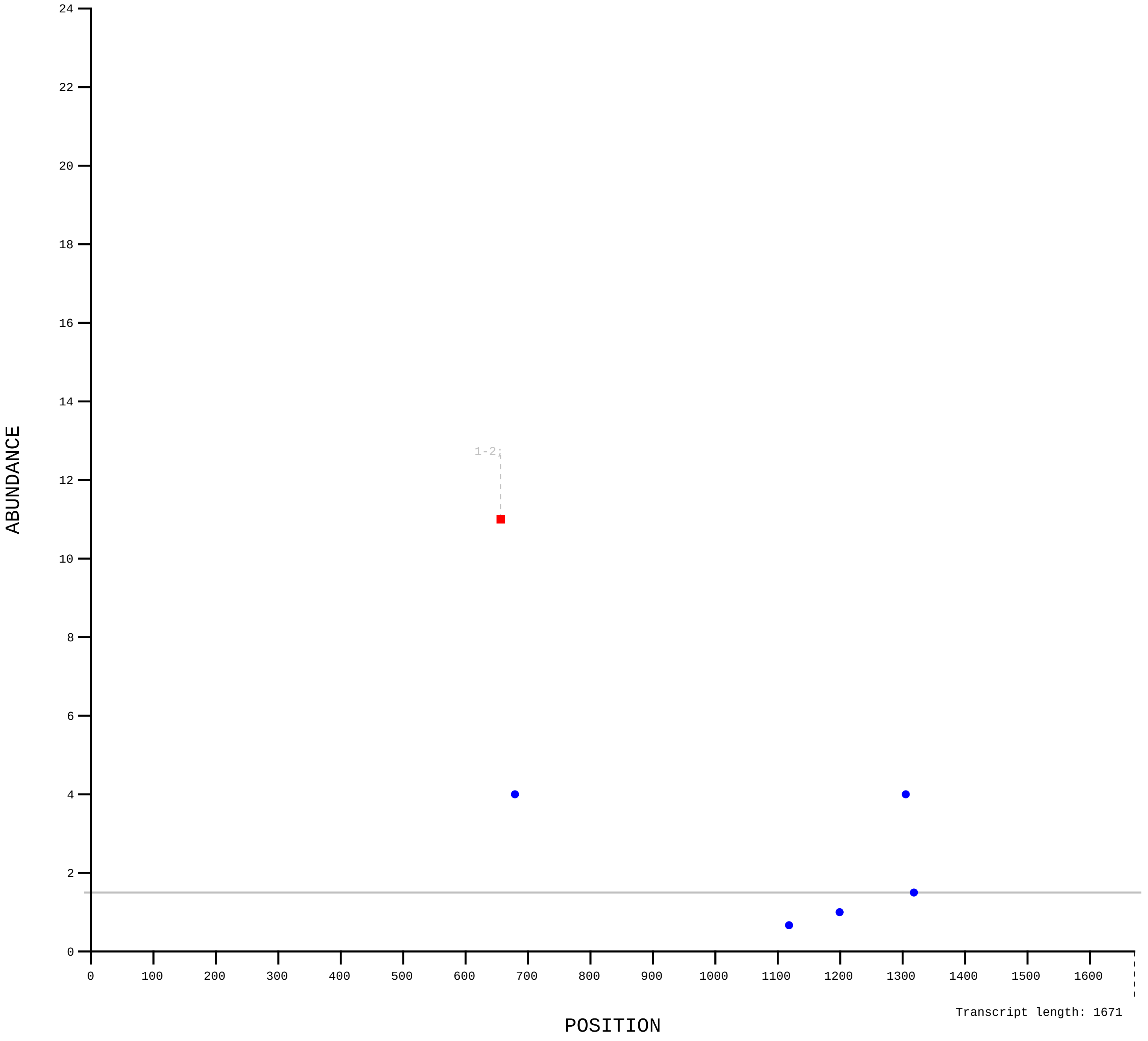

Category: 0 1 2 3 4
Degradome alignment: Median:

```
0  #1  Position:656  Abundance: 11.00(deg) 3(sRNA)
   5'    TTCCACA-GCTTTCTTGAACTG        3'  ID:
         ||||||| |||||||||||| |            Score: 2.0
   3' TTCAAGGTGTCCGAAAGAACTTGCCAAAAACG 5'  p-value: 0.0

0  #2  Position:656  Abundance: 11.00(deg) 1(sRNA)
   5'    TTCCAC-GGCTTTCTTGAACTT        3'  ID:
         |||||| |||||||||||||              Score: 3.0
   3' TTCAAGGTGTCCGAAAGAACTTGCCAAAAACG 5'  p-value: 0.0
```

orange1.1t00172.2 gene=orange1.1t00172 CDS=280-1155

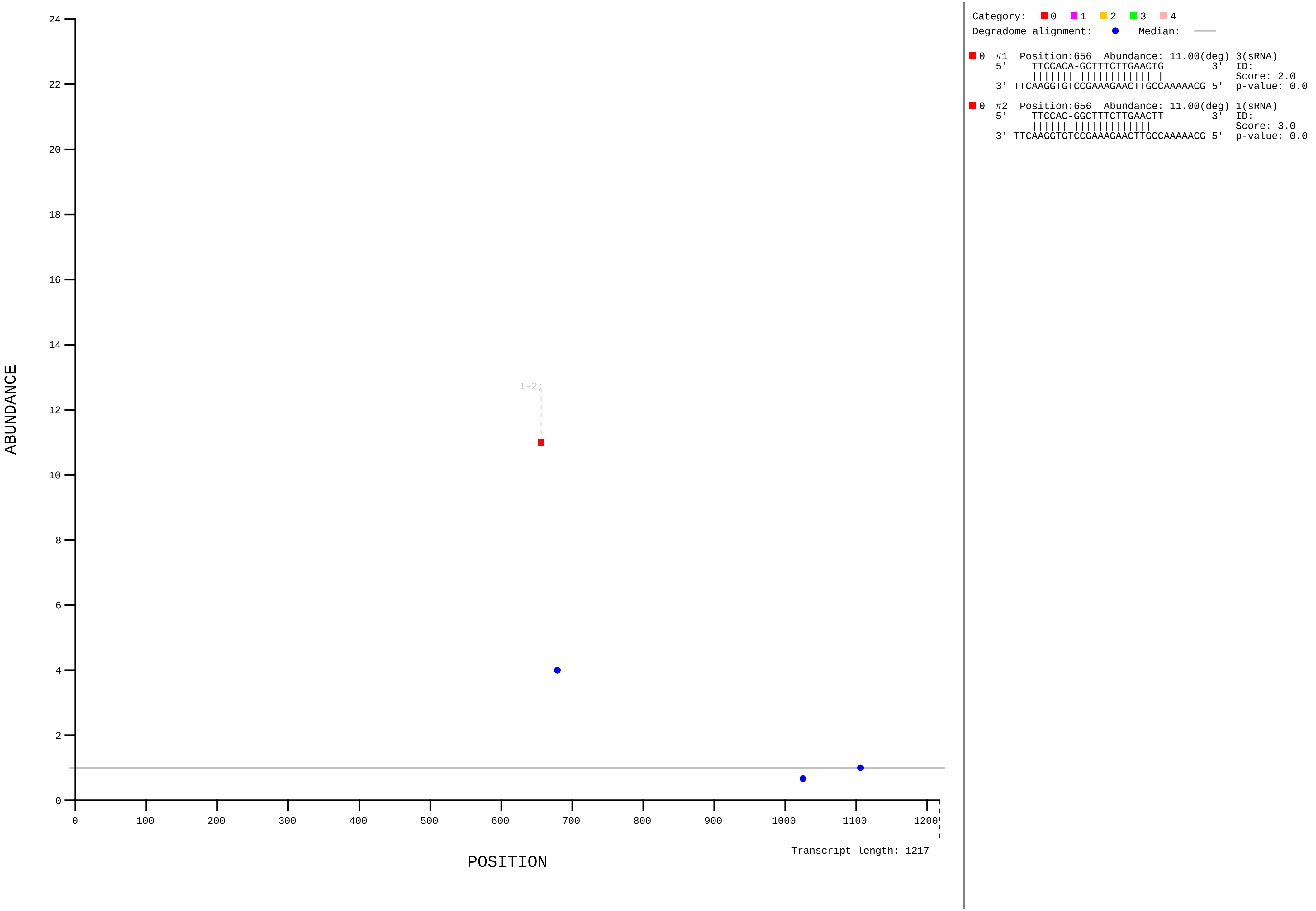

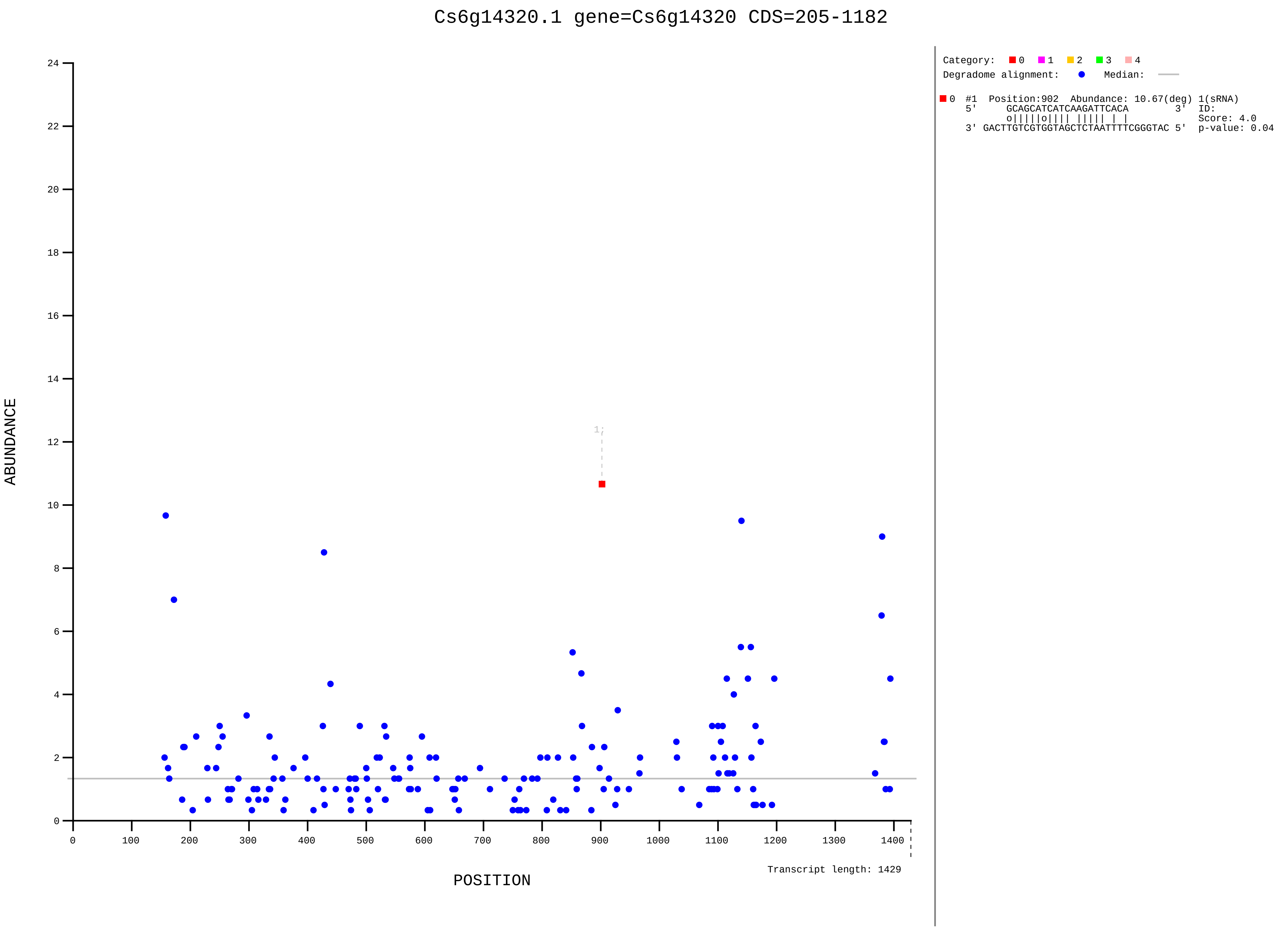
Cs6g14320.1 gene=Cs6g14320 CDS=205-1182
ABUNDANCE
POSITION
Transcript length: 1429
Category: 0 1 2 3 4
Degradome alignment:
Median:
0 #1 Position:902 Abundance: 10.67(deg) 1(sRNA)
5' GCAGCATCATCAAGATTCACA 3' ID:
o|||||o|||| ||||| | | Score: 4.0
3' GACTTGTCGTGGTAGCTCTAATTTTCGGGTAC 5' p-value: 0.04

Cs6g14320.3 gene=Cs6g14320 CDS=205-1278

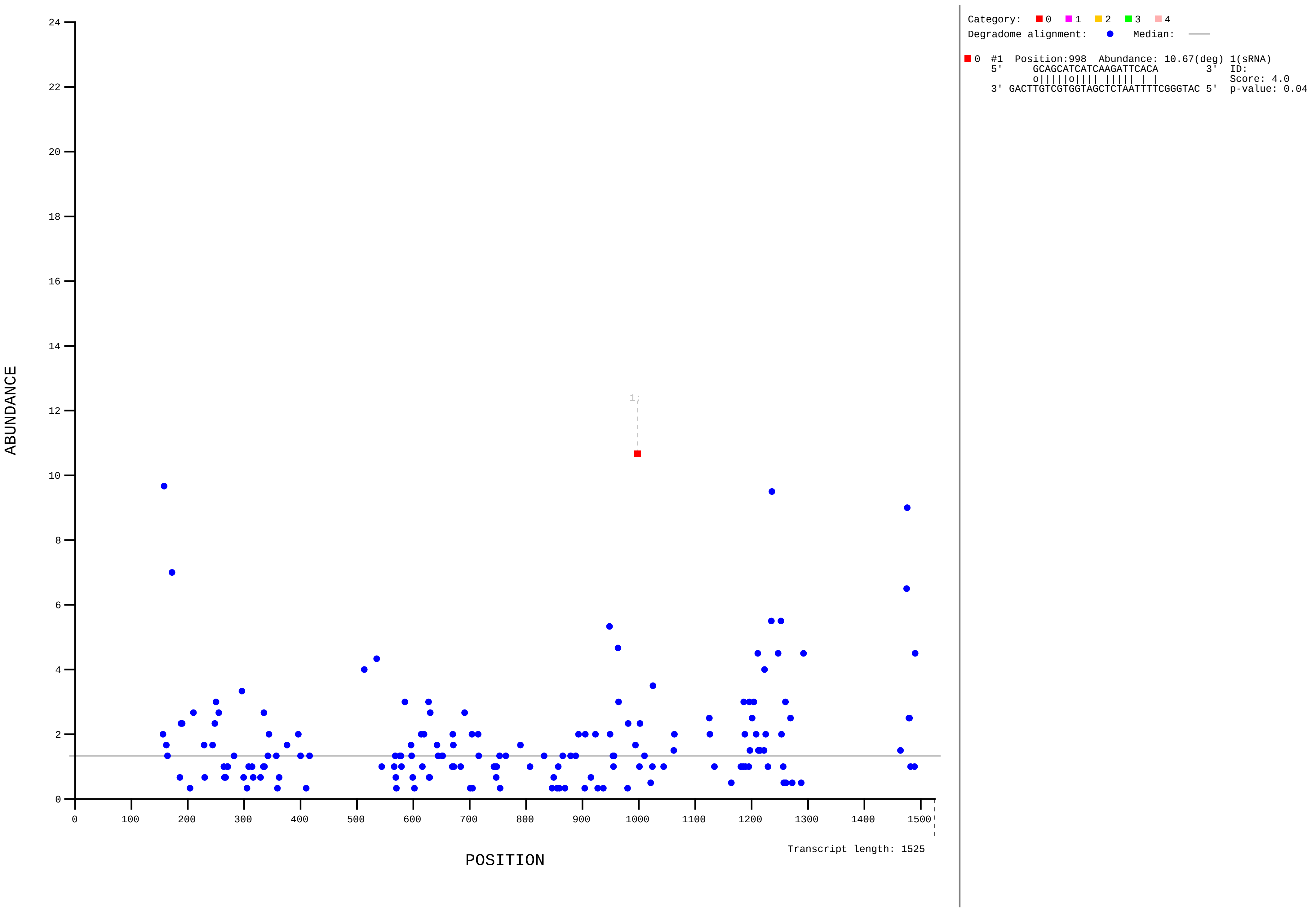

Cs6g14320.2 gene=Cs6g14320 CDS=205-936

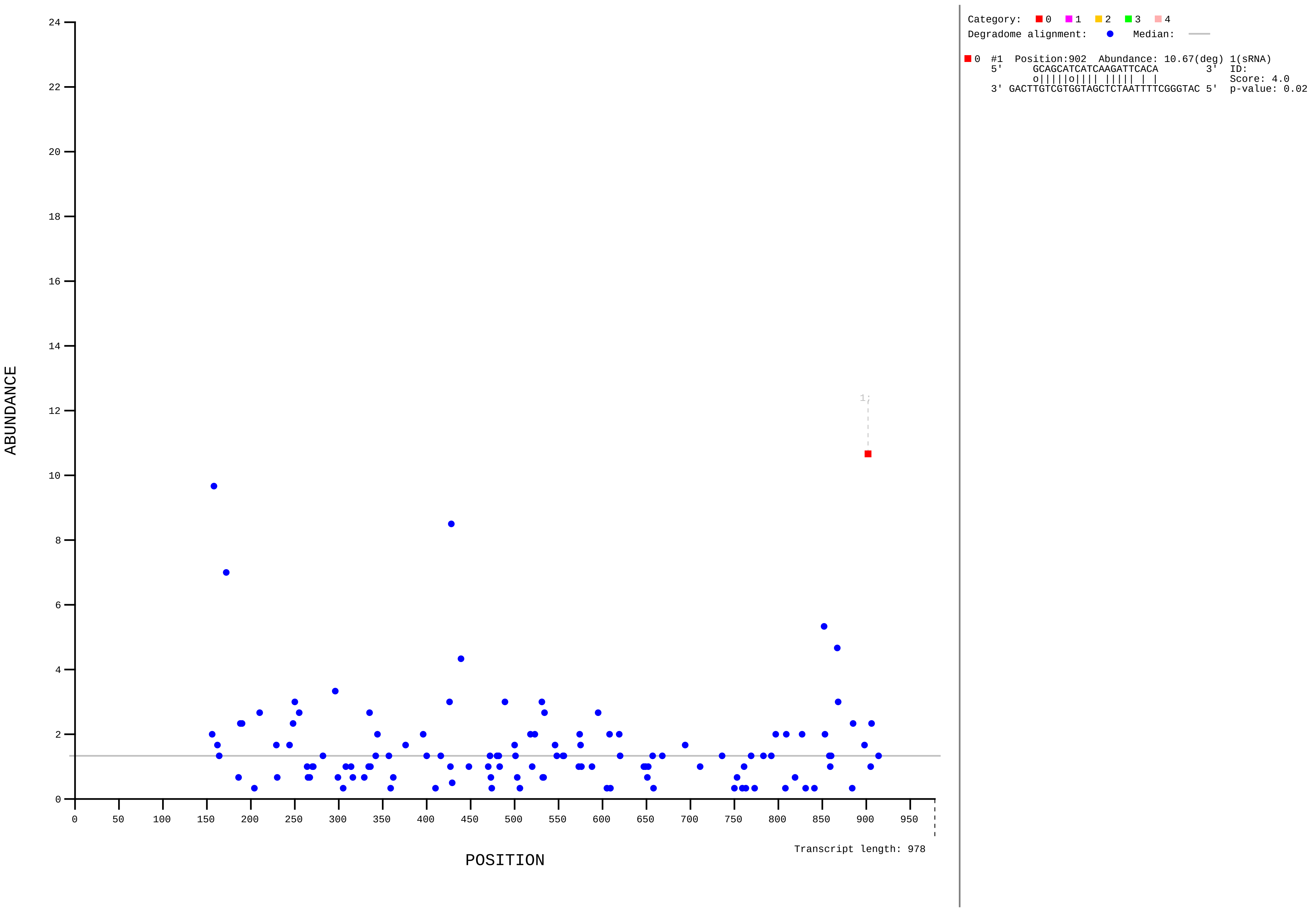

Cs5g19920.1 gene=Cs5g19920 CDS=1-3840

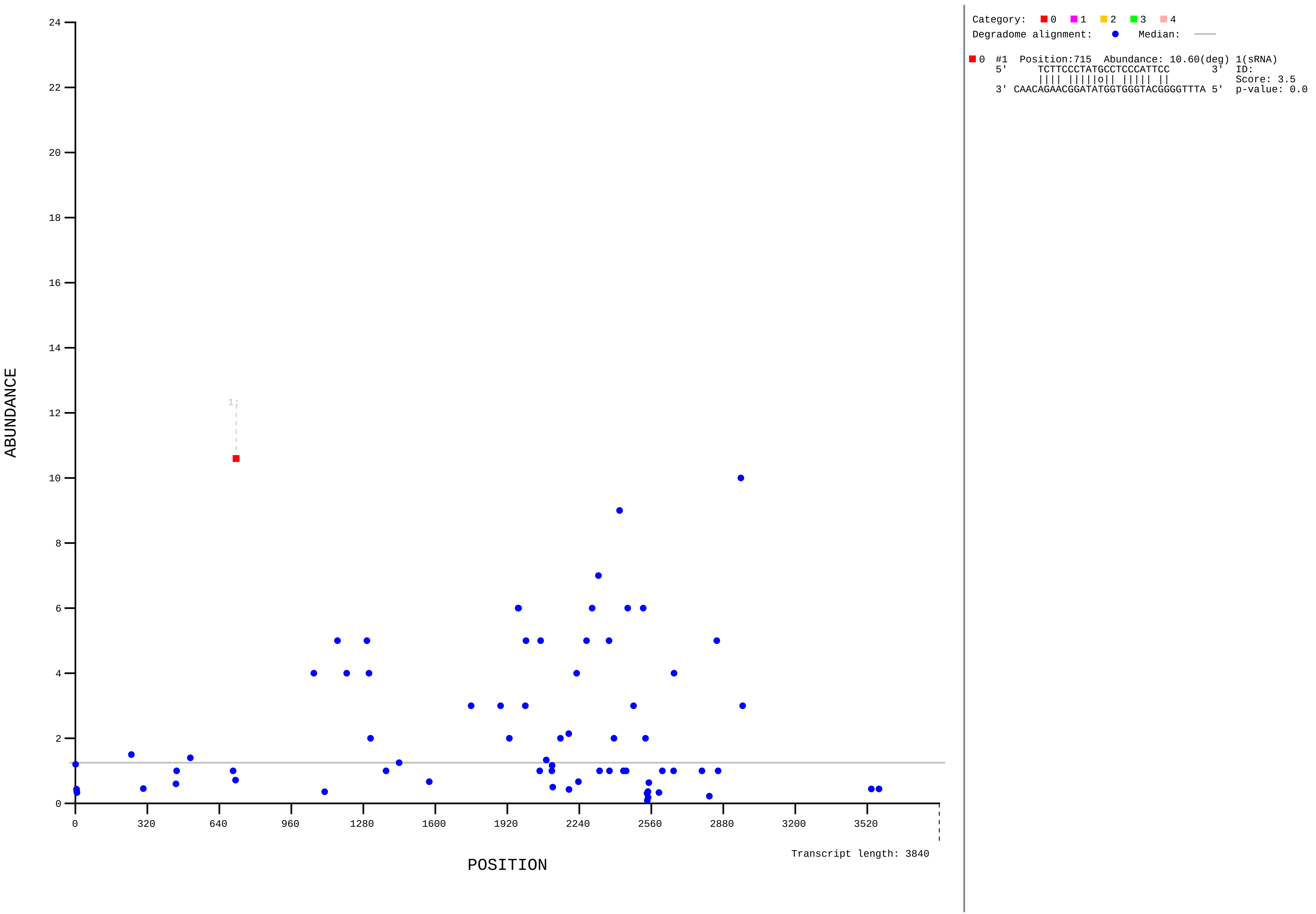

Cs2g30960.1 gene=Cs2g30960 CDS=1-3465

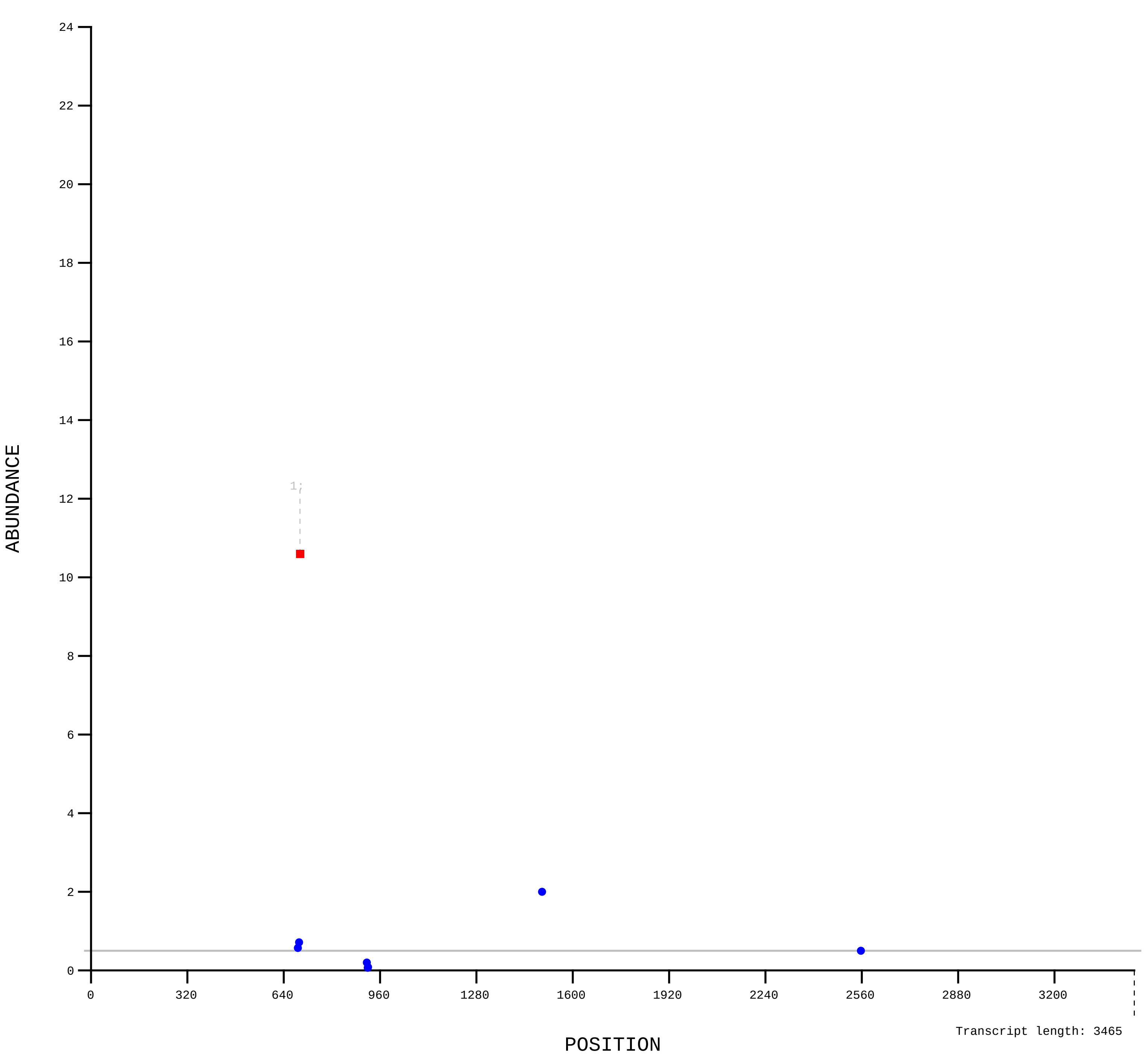

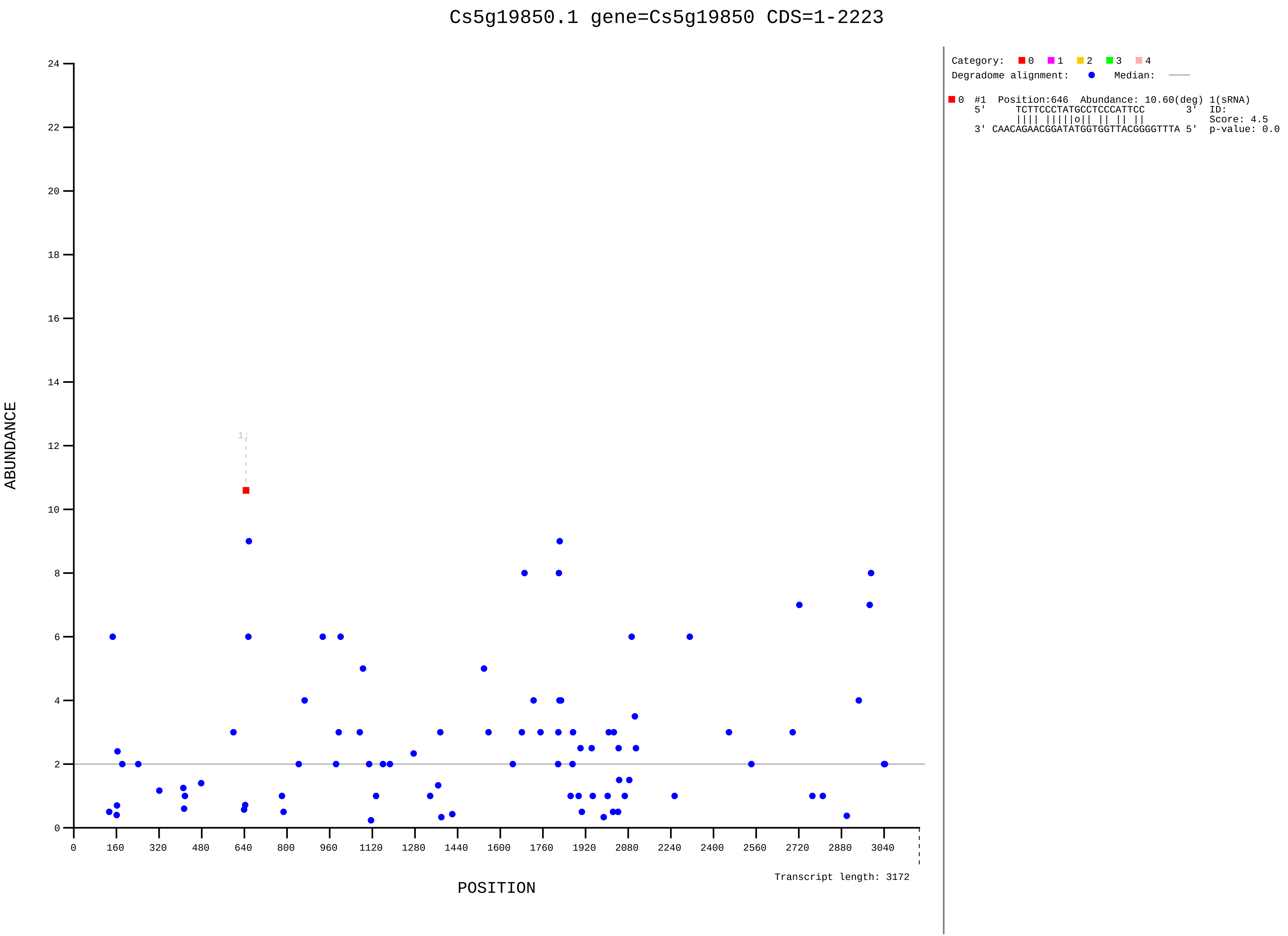

Cs5g19850.1 gene=Cs5g19850 CDS=1-2223
ABUNDANCE
POSITION
Transcript length: 3172
Category: 0 1 2 3 4
Degradome alignment:
Median:
0 #1 Position:646 Abundance: 10.60(deg) 1(sRNA)
5' TCTTCCCTATGCCTCCCATTCC 3' ID:
|||| |||||o|| || || ||
Score: 4.5
3' CAACAGAACGGATATGGTGGTTACGGGGTTTA 5' p-value: 0.0

Cs2g30970.1 gene=Cs2g30970 CDS=1-3558

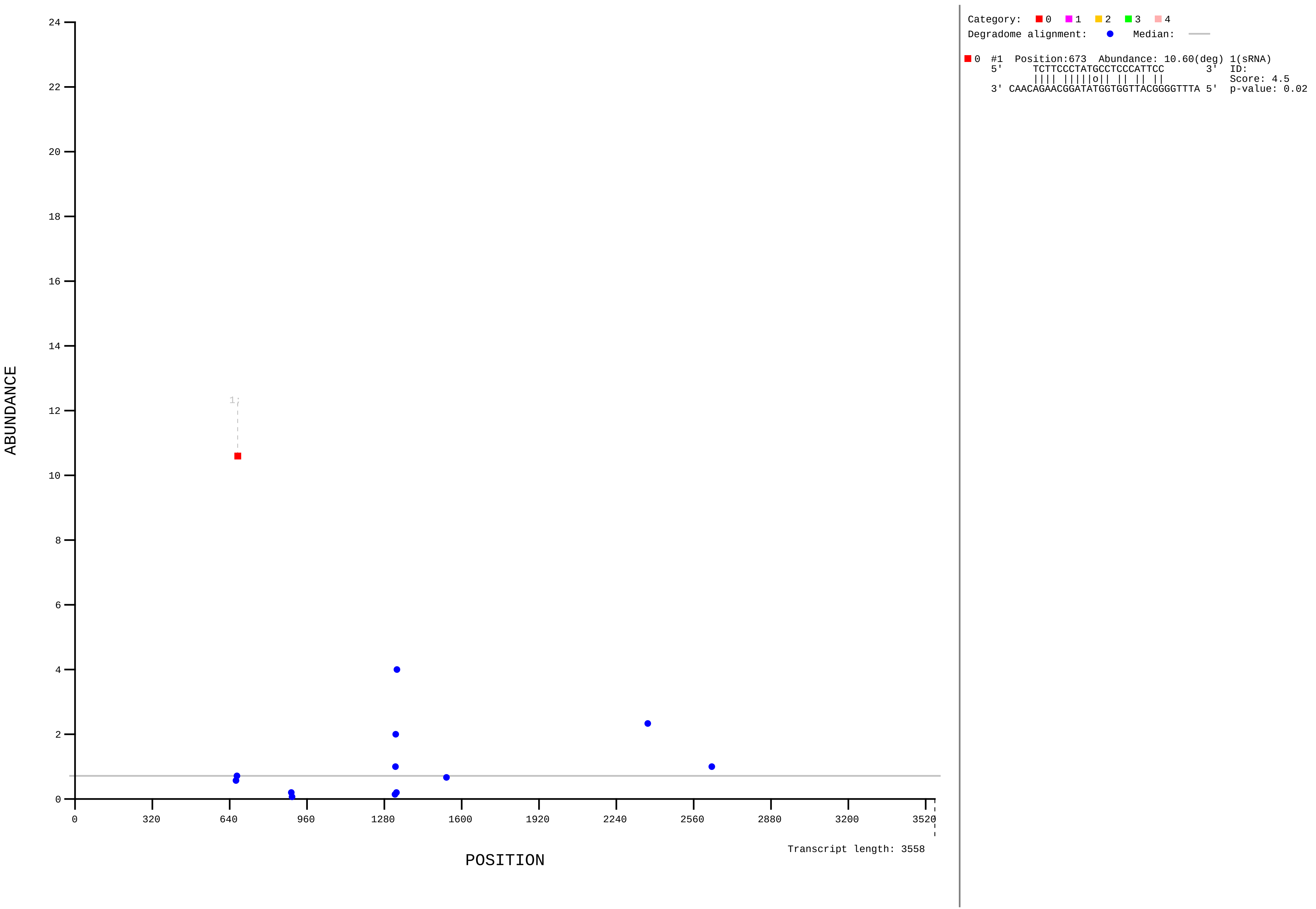

Cs8g10560.1 gene=Cs8g10560 CDS=1-1557

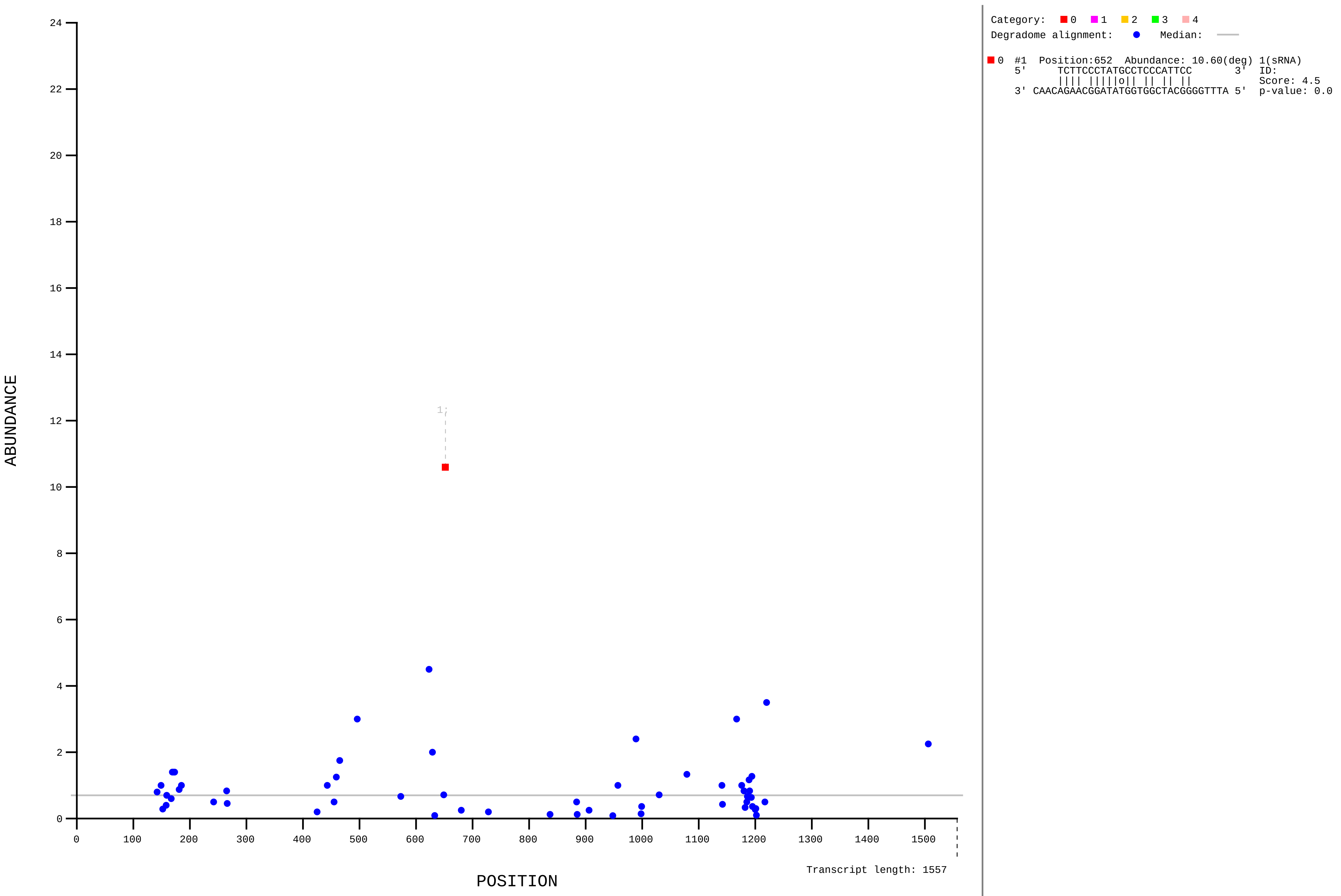

Cs5g29290.4 gene=Cs5g29290 CDS=200-1444

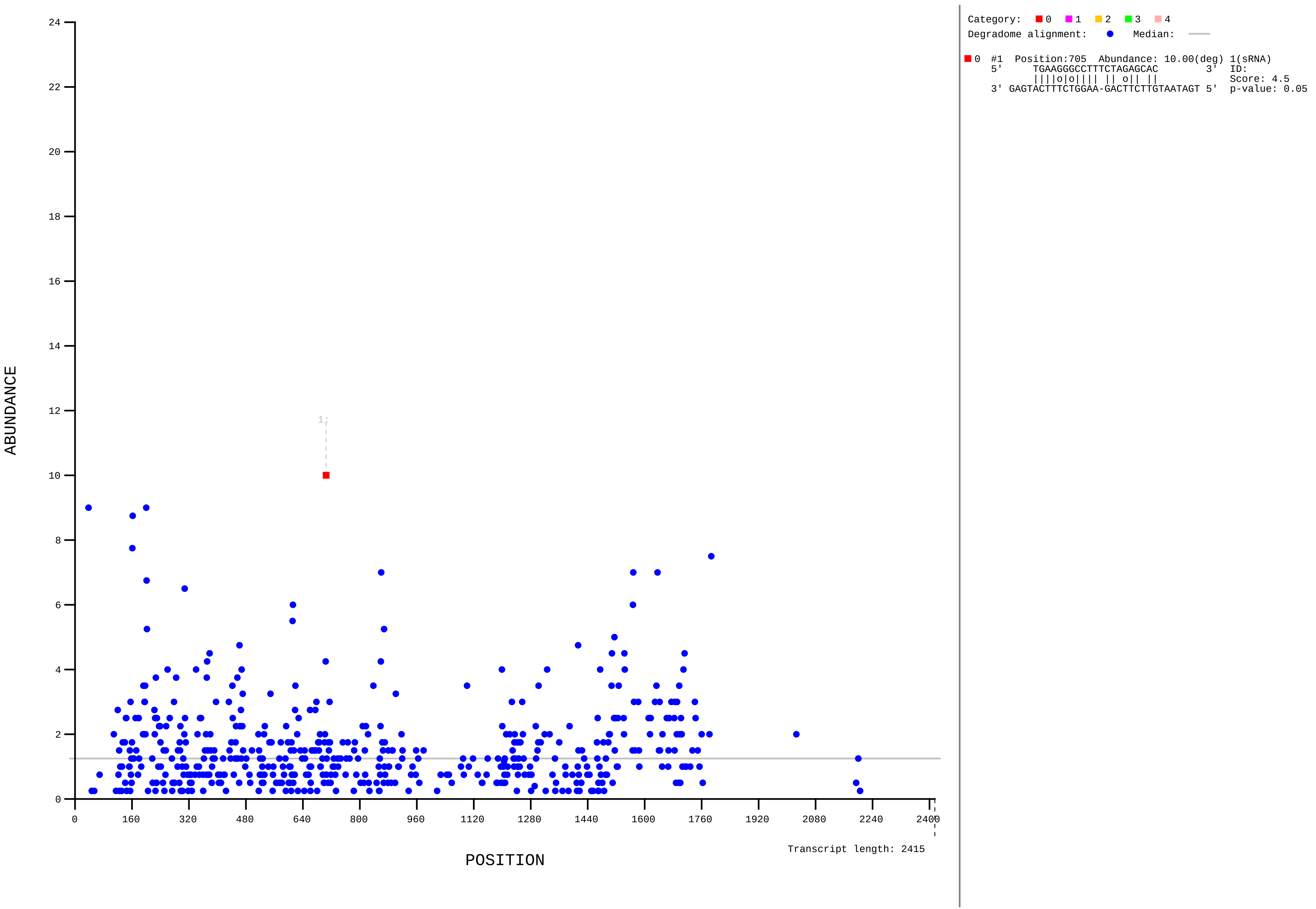

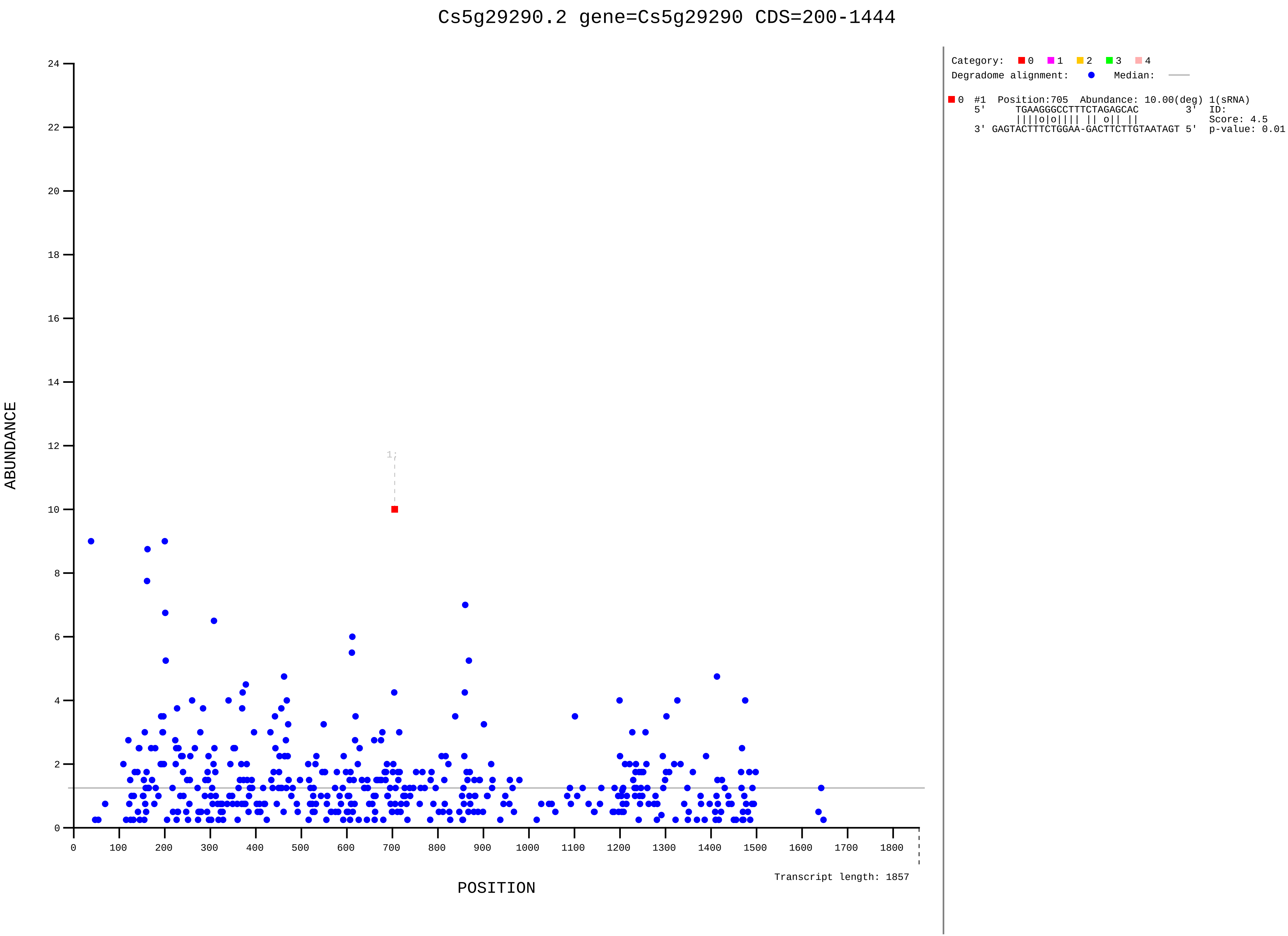

Cs5g29290.2 gene=Cs5g29290 CDS=200-1444
ABUNDANCE
POSITION
Transcript length: 1857
Category: 0 1 2 3 4
Degradome alignment: Median:
0 #1 Position:705 Abundance: 10.00(deg) 1(sRNA)
5' TGAAGGGCCTTTCTAGAGCAC 3' ID:
||||o|o|||| || o|| ||
Score: 4.5
3' GAGTACTTTCTGGAA-GACTTCTTGTAATAGT 5' p-value: 0.01

Cs3g05280.1 gene=Cs3g05280 CDS=121-3564

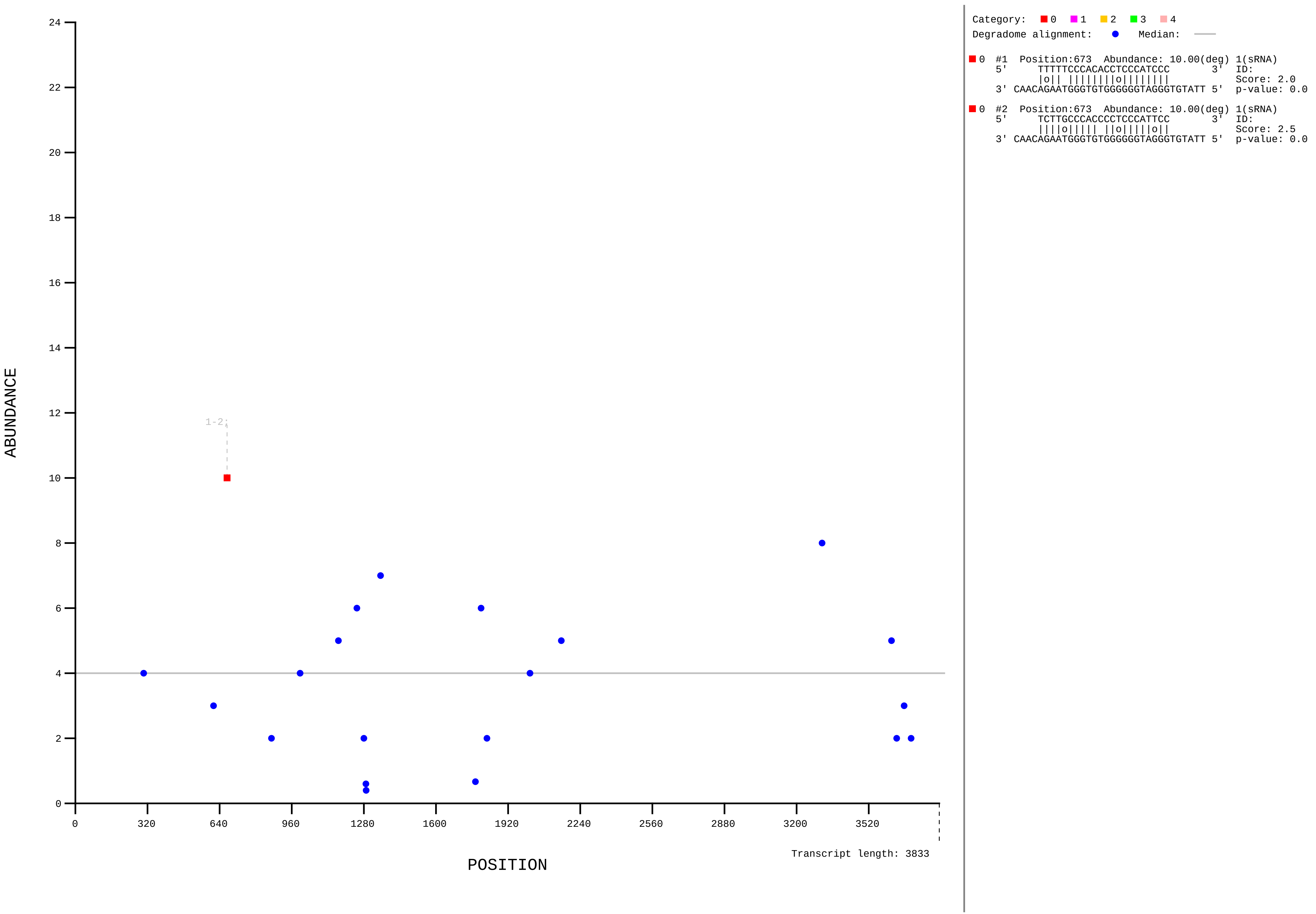

Cs5g29290.1 gene=Cs5g29290 CDS=200-1444

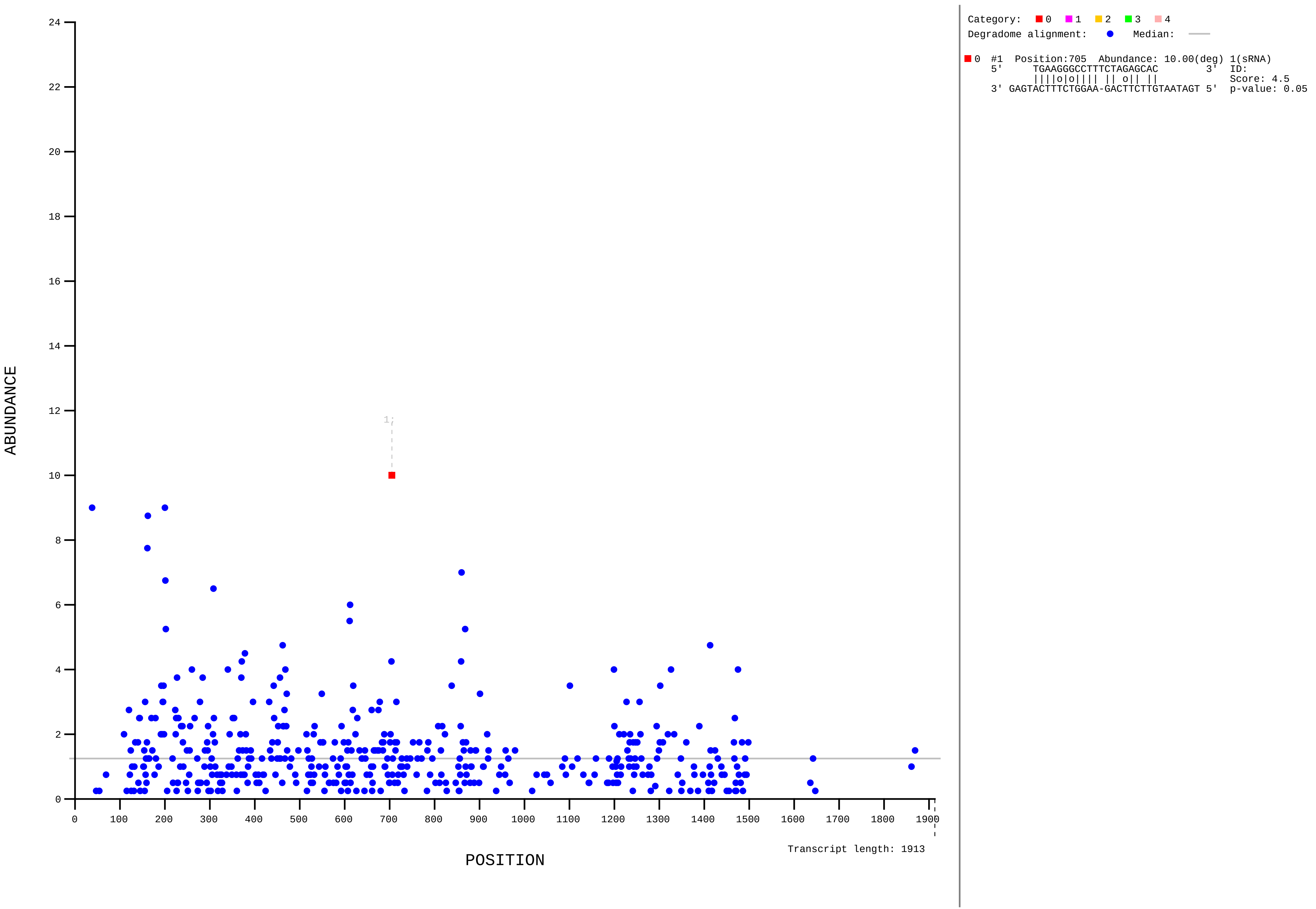

Cs5g14570.1 gene=Cs5g14570 CDS=309-554

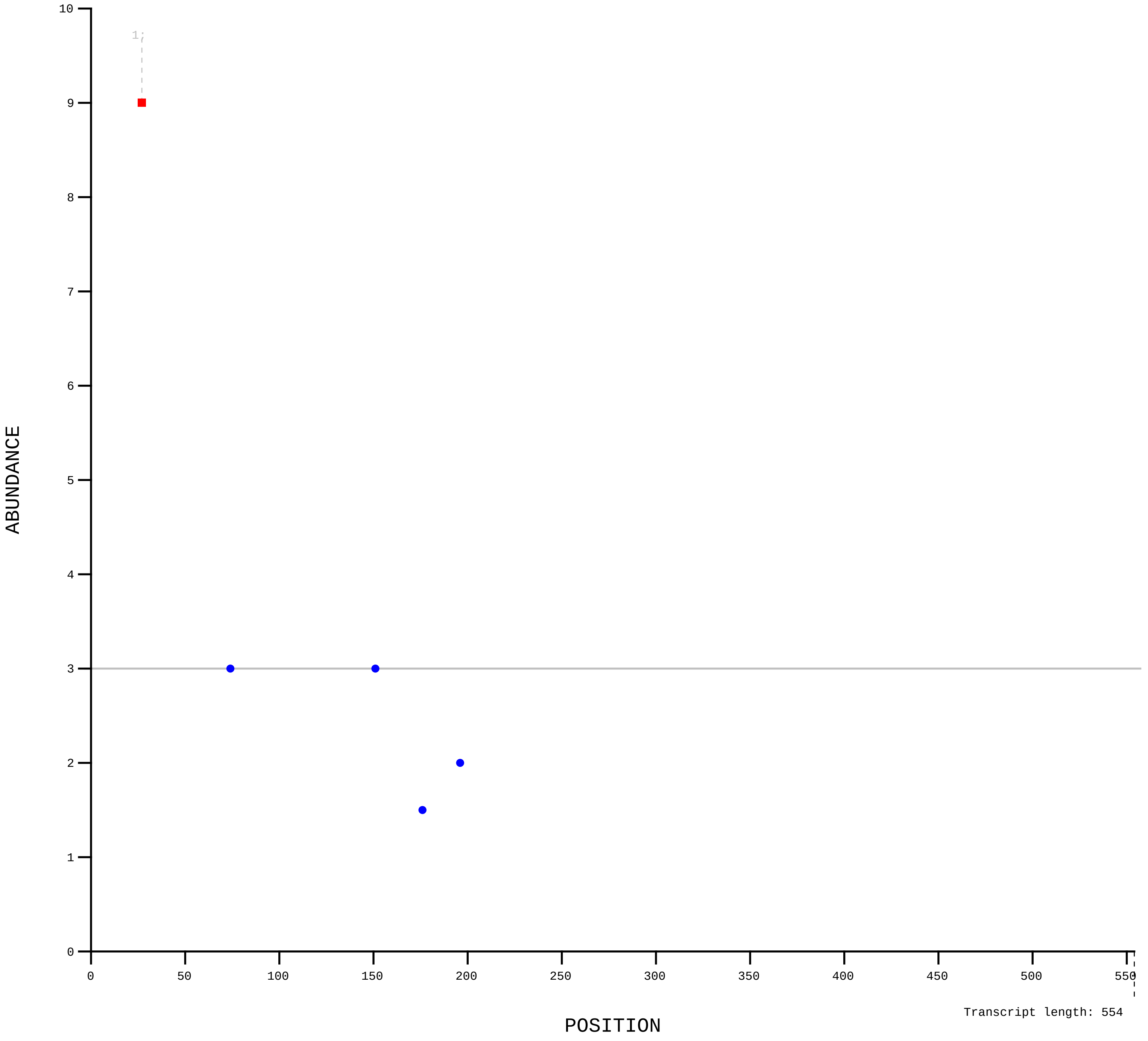

Category: 0 1 2 3 4
Degradome alignment: Median:

```
0  #1  Position:27  Abundance: 9.00(deg) 3(sRNA)
   5'     TTCCACAGCTTTCTTGAACTG        3'  ID:
          | |||||| ||o|||o||| |            Score: 4.0
   3' GAGTACGGTGTCTAAGGAATTTGTCAATTGAA 5'  p-value: 0.03
```

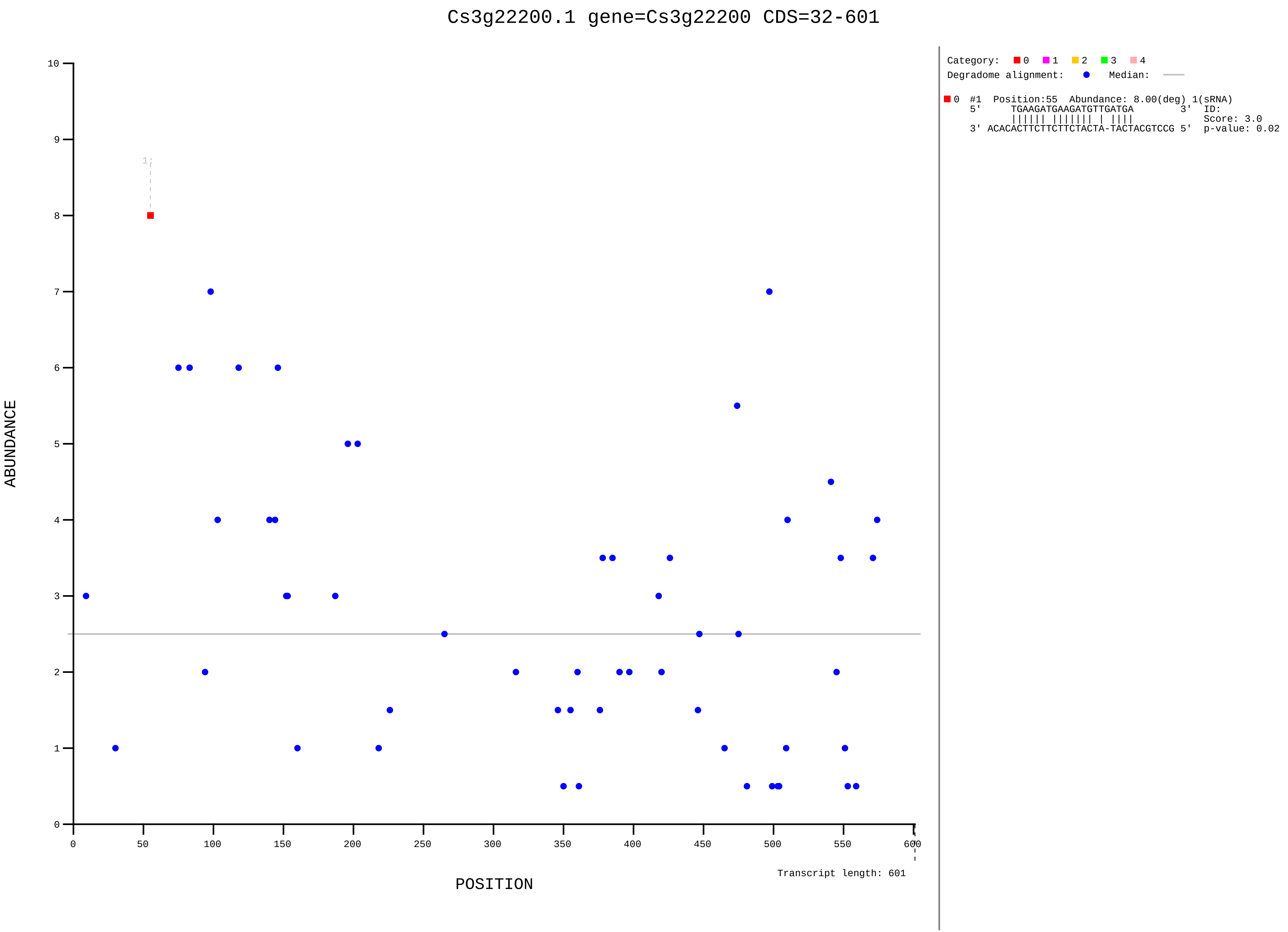

Cs3g22200.1 gene=Cs3g22200 CDS=32-601
ABUNDANCE
POSITION
0
1
2
3
4
5
6
7
8
9
10
0
50
100
150
200
250
300
350
400
450
500
550
600
1
Transcript length: 601
Category: 0 1 2 3 4
Degradome alignment:
Median:
0 #1 Position:55 Abundance: 8.00(deg) 1(sRNA)
5' TGAAGATGAAGATGTTGATGA 3' ID:
|||||| ||||||| | ||||
Score: 3.0
3' ACACACTTCTTCTTCTACTA-TACTACGTCCG 5' p-value: 0.02

orange1.1t00591.1 gene=orange1.1t00591 CDS=1-531

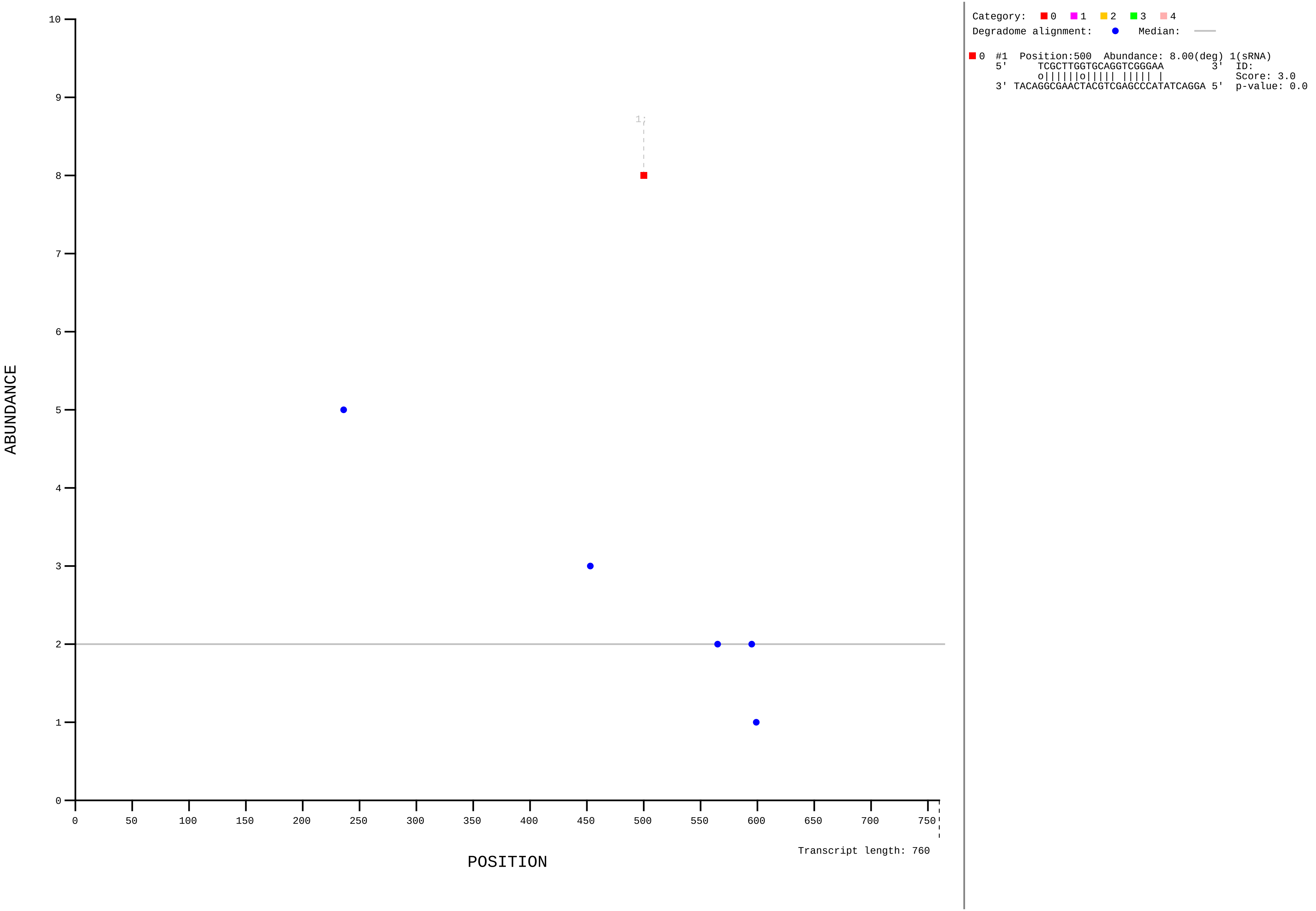

Cs2g06290.2 gene=Cs2g06290 CDS=347-943

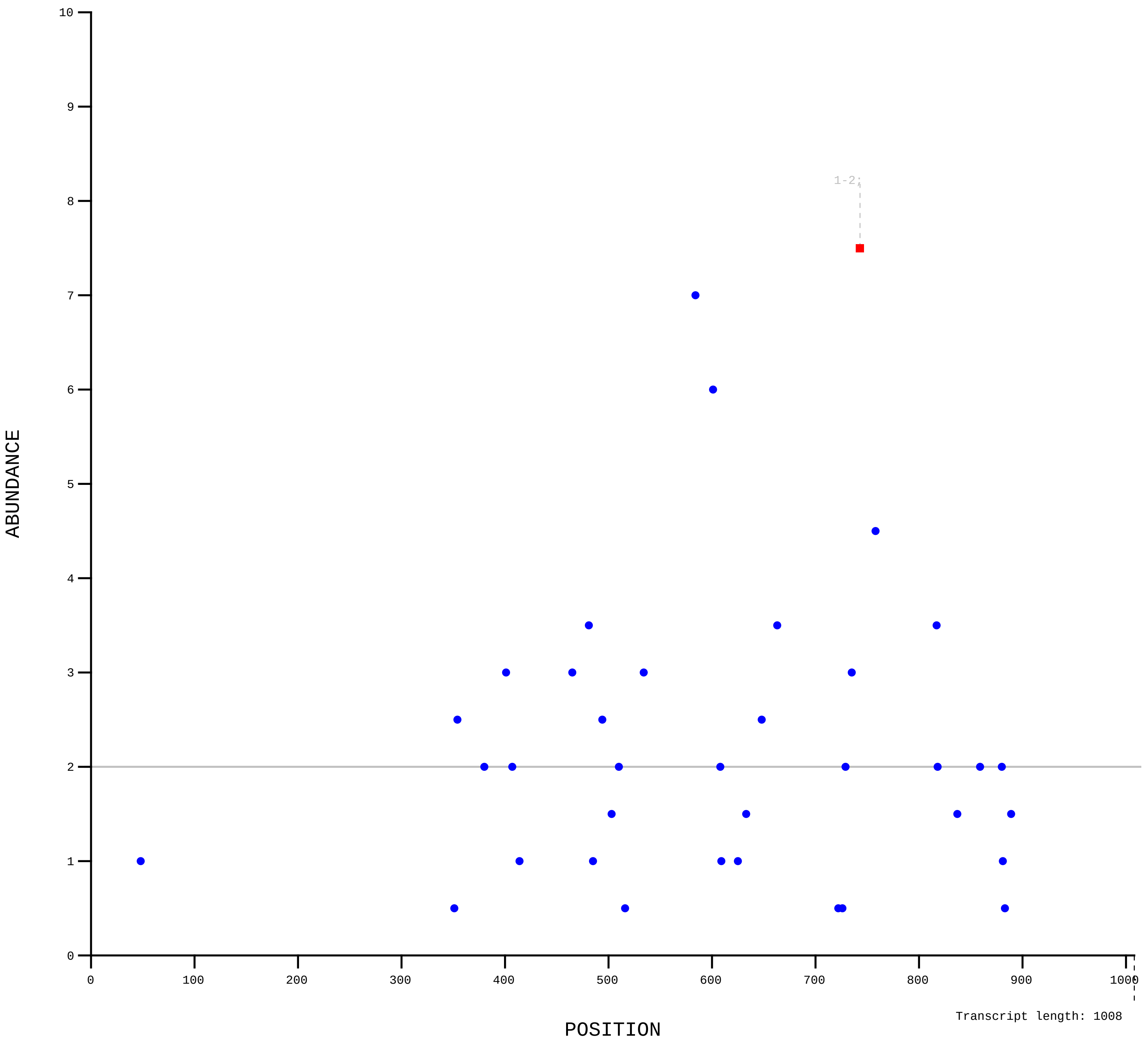


```
0  #1  Position:743  Abundance: 7.50(deg) 2(sRNA)
   5'      TGAAGCTGCCAGCATGATCTGG       3'  ID:
           oo|||||||||| || ||||| |          Score: 4.0
   3' TTCGGTTTCGACGGTAGTTCTAGAGCGTCCTG 5'  p-value: 0.0

0  #2  Position:743  Abundance: 7.50(deg) 2(sRNA)
   5'      TGAAGCTGCCAGCATGATCTTA       3'  ID:
           oo|||||||||| || |||||o           Score: 4.5
   3' TTCGGTTTCGACGGTAGTTCTAGAGCGTCCTG 5'  p-value: 0.01
```

Cs2g06290.1 gene=Cs2g06290 CDS=347-1120

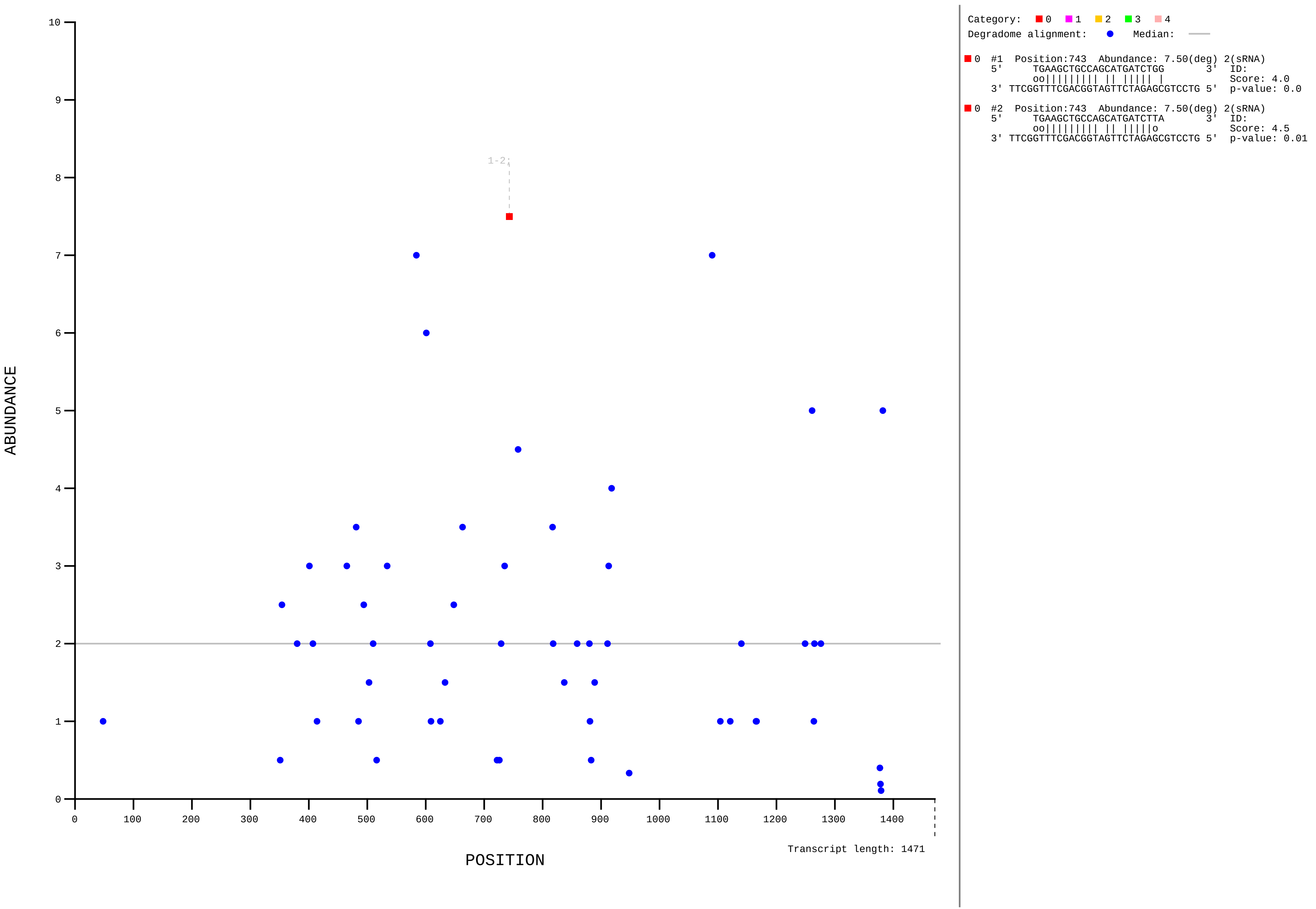

Cs7g10990.2 gene=Cs7g10990 CDS=1040-1978

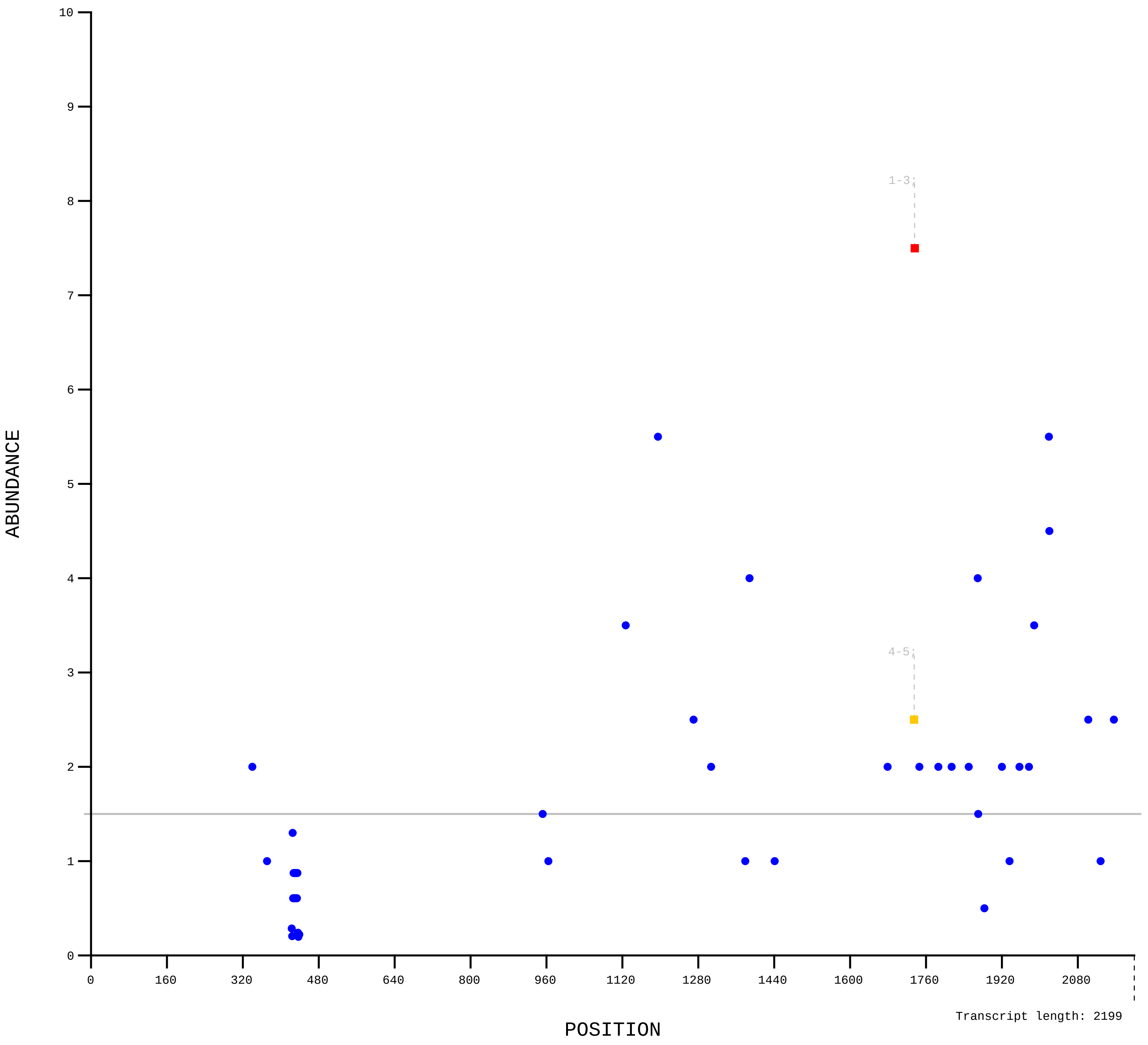

Category: 0 1 2 3 4
Degradome alignment: ● Median: —

```
0  #1  Position:1736  Abundance: 7.50(deg) 1(sRNA)
   5'     TTGACAGAAGAGAGTGAGCAC        3'  ID:
            ||||||||||||| ||||||           Score: 2.0
   3' CCACTACTGTCTTCTCTCTCTCGTGCTGAACT 5'  p-value: 0.0

0  #2  Position:1736  Abundance: 7.50(deg) 1(sRNA)
   5'     GTGACAGAAGATAGAGAGCGC        3'  ID:
          o|||||||||| |||||||o|            Score: 2.0
   3' CCACTACTGTCTTCTCTCTCTCGTGCTGAACT 5'  p-value: 0.0

0  #3  Position:1736  Abundance: 7.50(deg) 1(sRNA)
   5'     CTGACAGAAGAGAGTGAGCAC        3'  ID:
            ||||||||||||| ||||||           Score: 2.0
   3' CCACTACTGTCTTCTCTCTCTCGTGCTGAACT 5'  p-value: 0.0

2  #4  Position:1735  Abundance: 2.50(deg) 1(sRNA)
   5'     TGACAGAAGAGAGTGAGCAC         3'  ID:
          ||||||||||||| ||||||             Score: 1.0
   3' CACTACTGTCTTCTCTCTCTCGTGCTGAACTT 5'  p-value: 0.0

2  #5  Position:1735  Abundance: 2.50(deg) 1(sRNA)
   5'     TGACAGAAGATAGAGAGCGC         3'  ID:
          |||||||||| |||||||o|             Score: 1.5
   3' CACTACTGTCTTCTCTCTCTCGTGCTGAACTT 5'  p-value: 0.02
```

Cs9g17500.1 gene=Cs9g17500 CDS=149-2368

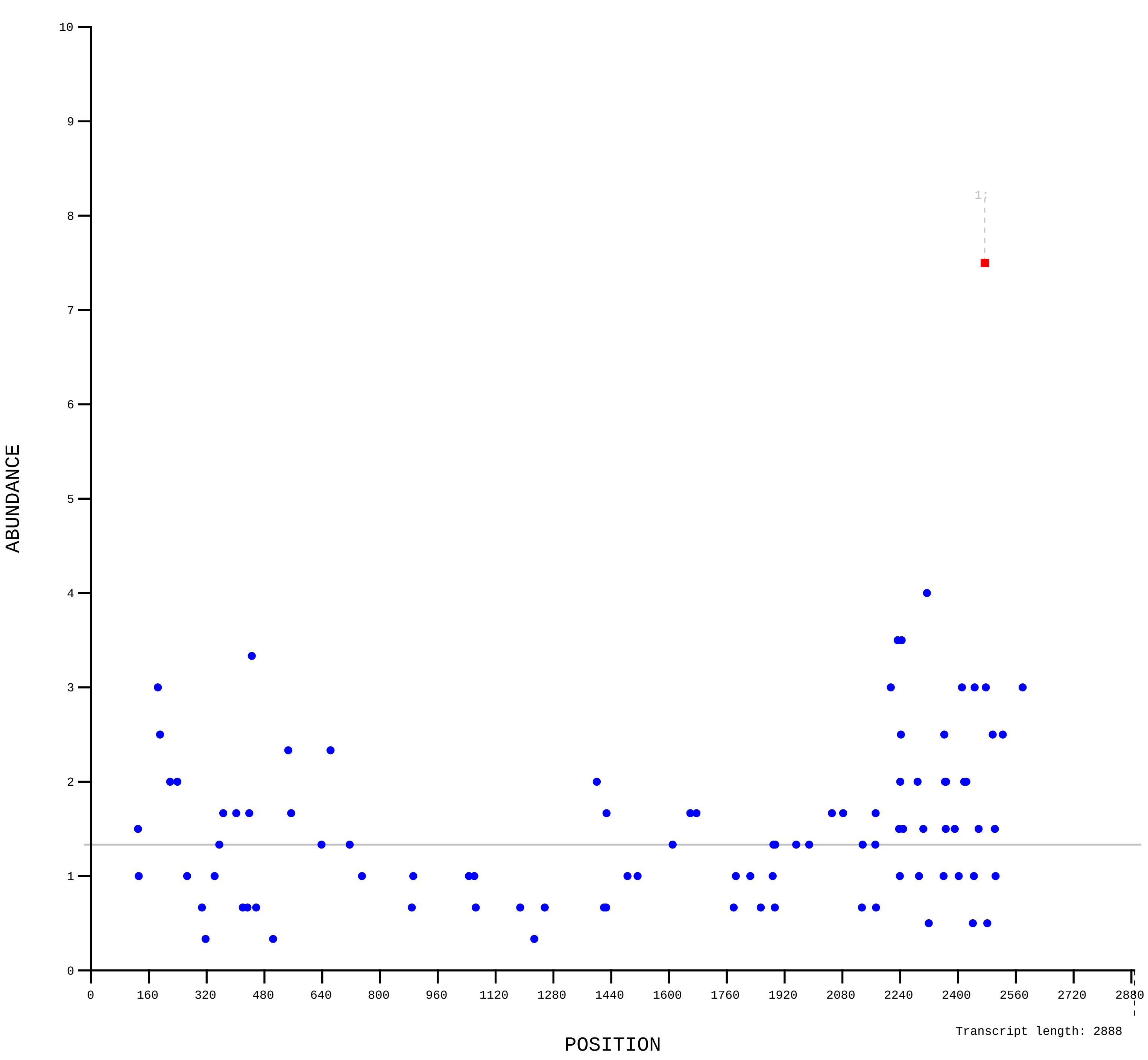

Category: 0 1 2 3 4
Degradome alignment: Median:

0 #1 Position:2474 Abundance: 7.50(deg) 1(sRNA)
5' ATTGGGGGTAGATTGAGGTTT 3' ID:
|||||||||||| o o ||o|| Score: 4.5
3' TGGTTAACCCCCATCGGTTACCGAAACGGTTA 5' p-value: 0.04

Cs7g10990.1 gene=Cs7g10990 CDS=851-1789

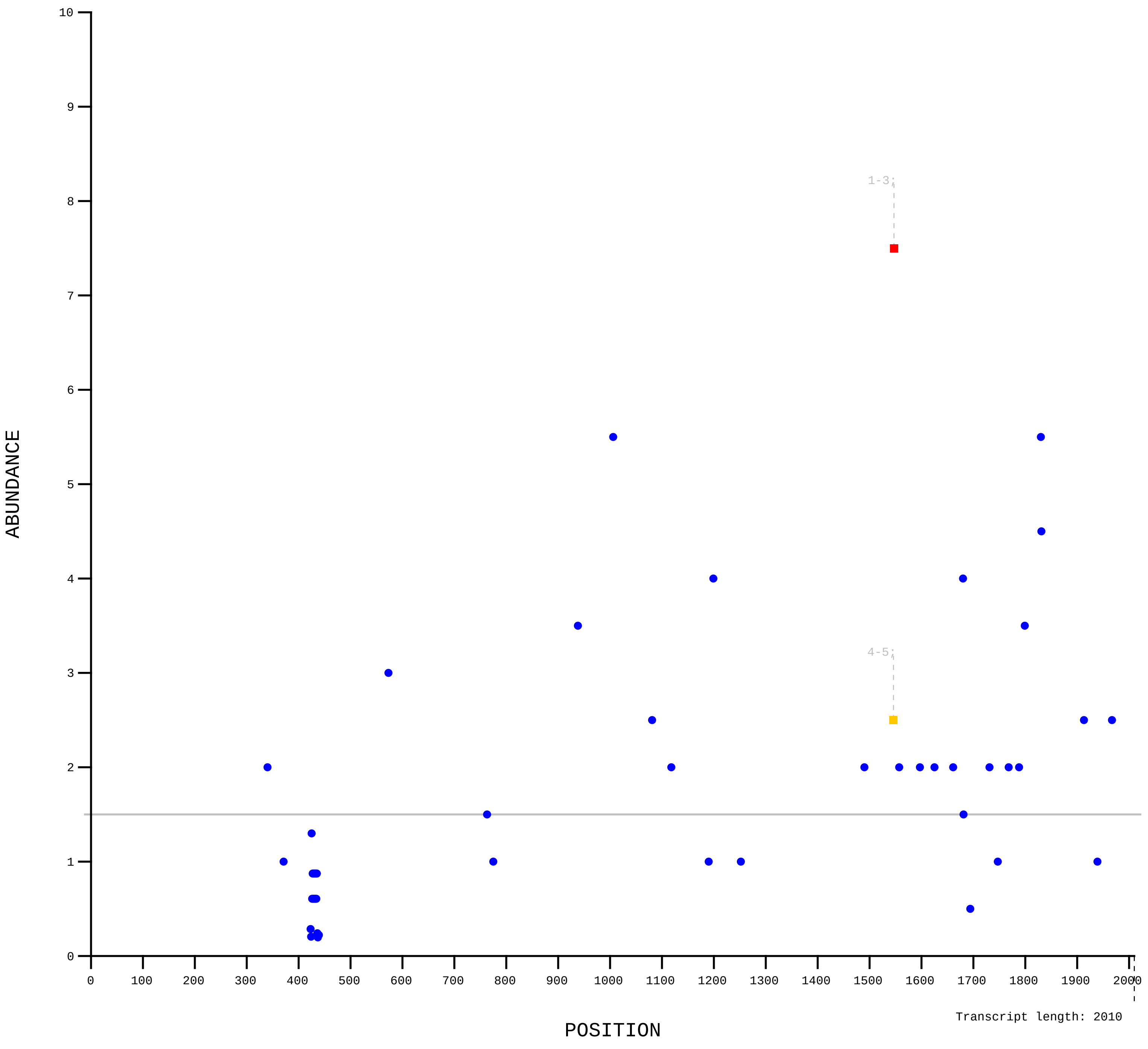


```
Category: ■0 ■1 ■2 ■3 ■4
Degradome alignment: ●   Median: ——

■0  #1  Position:1547  Abundance: 7.50(deg) 1(sRNA)
    5'     TTGACAGAAGAGAGTGAGCAC        3'  ID:
            ||||||||||||| ||||||            Score: 2.0
    3' CCACTACTGTCTTCTCTCTCTCGTGCTGAACT 5'  p-value: 0.0

■0  #2  Position:1547  Abundance: 7.50(deg) 1(sRNA)
    5'     GTGACAGAAGATAGAGAGCGC        3'  ID:
           o|||||||||| |||||||o|            Score: 2.0
    3' CCACTACTGTCTTCTCTCTCTCGTGCTGAACT 5'  p-value: 0.0

■0  #3  Position:1547  Abundance: 7.50(deg) 1(sRNA)
    5'     CTGACAGAAGAGAGTGAGCAC        3'  ID:
            ||||||||||||| ||||||            Score: 2.0
    3' CCACTACTGTCTTCTCTCTCTCGTGCTGAACT 5'  p-value: 0.0

■2  #4  Position:1546  Abundance: 2.50(deg) 1(sRNA)
    5'     TGACAGAAGAGAGTGAGCAC         3'  ID:
           ||||||||||||| ||||||             Score: 1.0
    3' CACTACTGTCTTCTCTCTCTCGTGCTGAACTT 5'  p-value: 0.0

■2  #5  Position:1546  Abundance: 2.50(deg) 1(sRNA)
    5'     TGACAGAAGATAGAGAGCGC         3'  ID:
           |||||||||| |||||||o|             Score: 1.5
    3' CACTACTGTCTTCTCTCTCTCGTGCTGAACTT 5'  p-value: 0.01
```

orange1.1t01536.3 gene=orange1.1t01536 CDS=377-2329

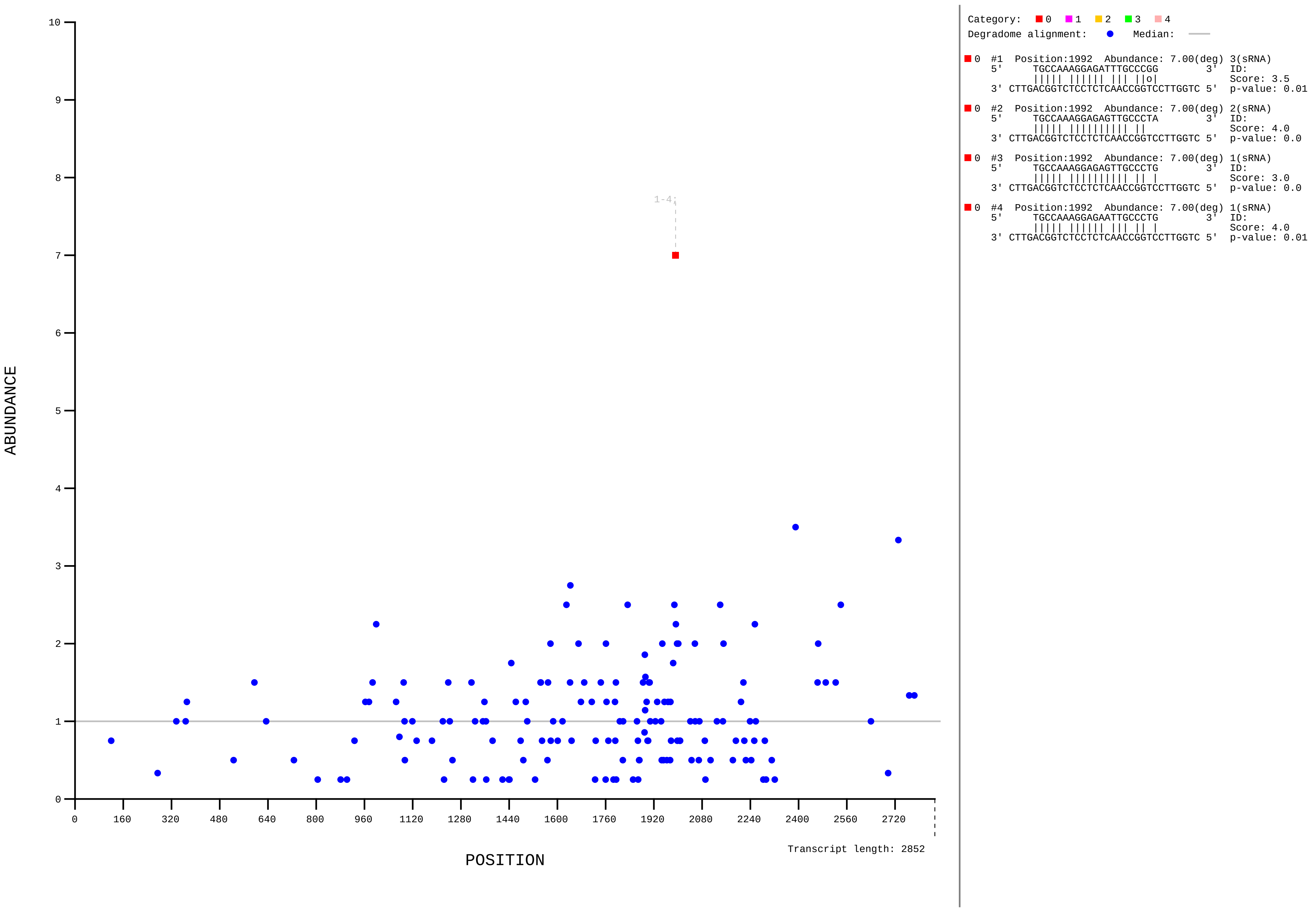

orange1.1t01536.2 gene=orange1.1t01536 CDS=299-2251

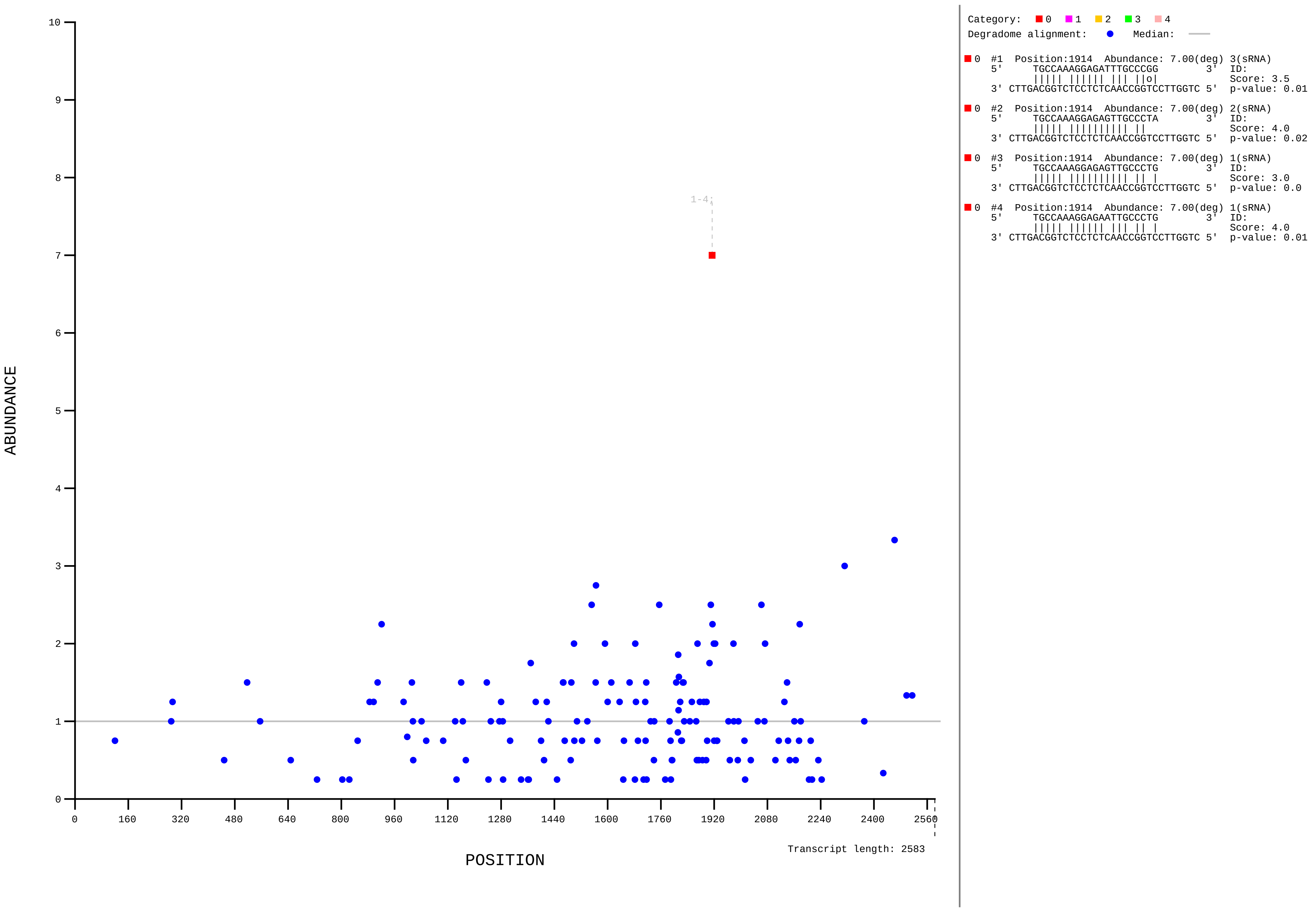

orange1.1t01536.4 gene=orange1.1t01536 CDS=309-2261

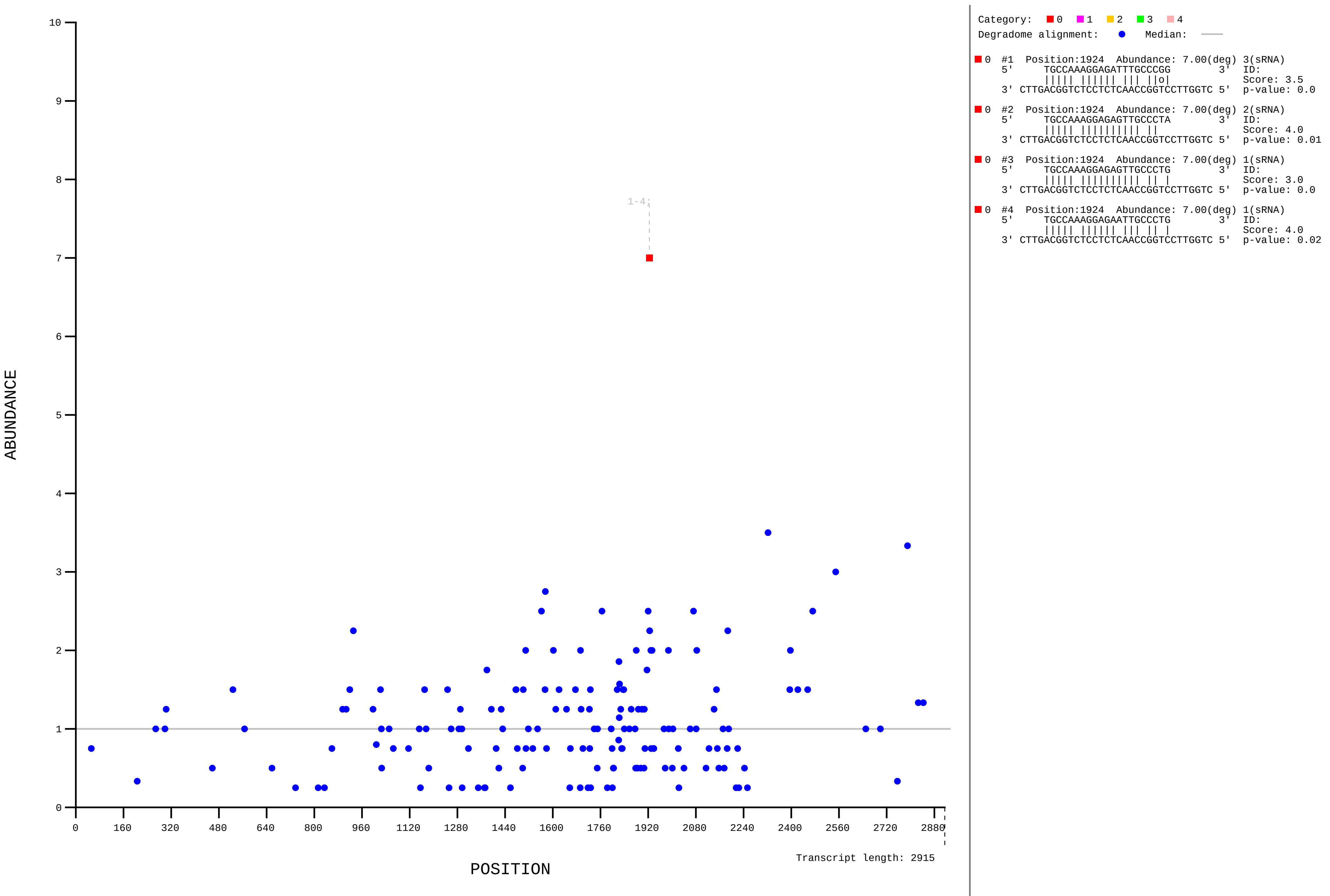

orange1.1t01536.1 gene=orange1.1t01536 CDS=307-2259

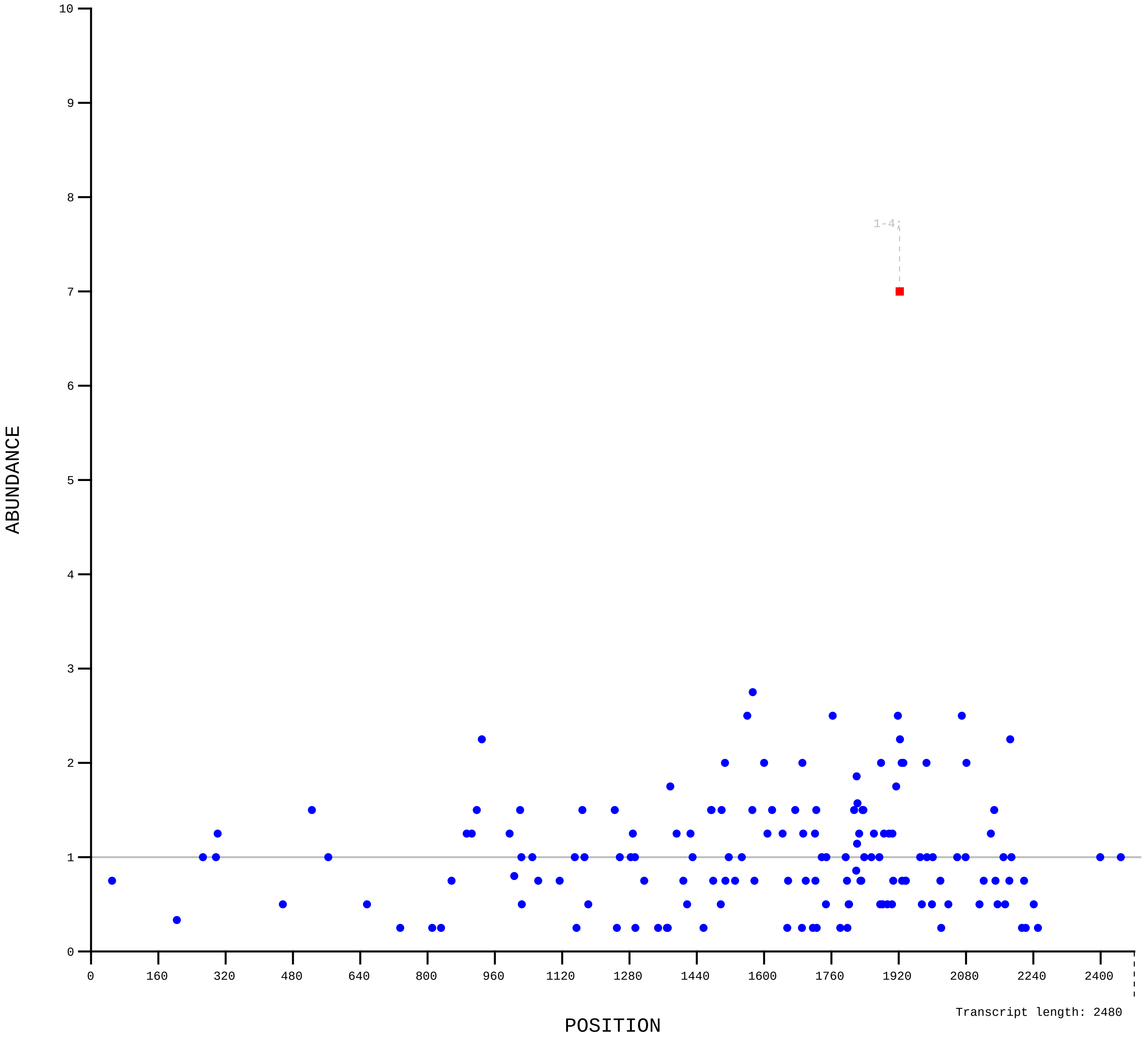

Category: 0 1 2 3 4
Degradome alignment: Median:

0 #1 Position:1922 Abundance: 7.00(deg) 3(sRNA)
```
5'     TGCCAAAGGAGATTTGCCCGG        3'  ID:
       ||||| |||||| ||| ||o|            Score: 3.5
3' CTTGACGGTCTCCTCTCAACCGGTCCTTGGTC 5'  p-value: 0.01
```

0 #2 Position:1922 Abundance: 7.00(deg) 2(sRNA)
```
5'     TGCCAAAGGAGAGTTGCCCTA        3'  ID:
       ||||| |||||||||| ||              Score: 4.0
3' CTTGACGGTCTCCTCTCAACCGGTCCTTGGTC 5'  p-value: 0.02
```

0 #3 Position:1922 Abundance: 7.00(deg) 1(sRNA)
```
5'     TGCCAAAGGAGAGTTGCCCTG        3'  ID:
       ||||| |||||||||| || |            Score: 3.0
3' CTTGACGGTCTCCTCTCAACCGGTCCTTGGTC 5'  p-value: 0.0
```

0 #4 Position:1922 Abundance: 7.00(deg) 1(sRNA)
```
5'     TGCCAAAGGAGAATTGCCCTG        3'  ID:
       ||||| |||||| ||| || |            Score: 4.0
3' CTTGACGGTCTCCTCTCAACCGGTCCTTGGTC 5'  p-value: 0.0
```

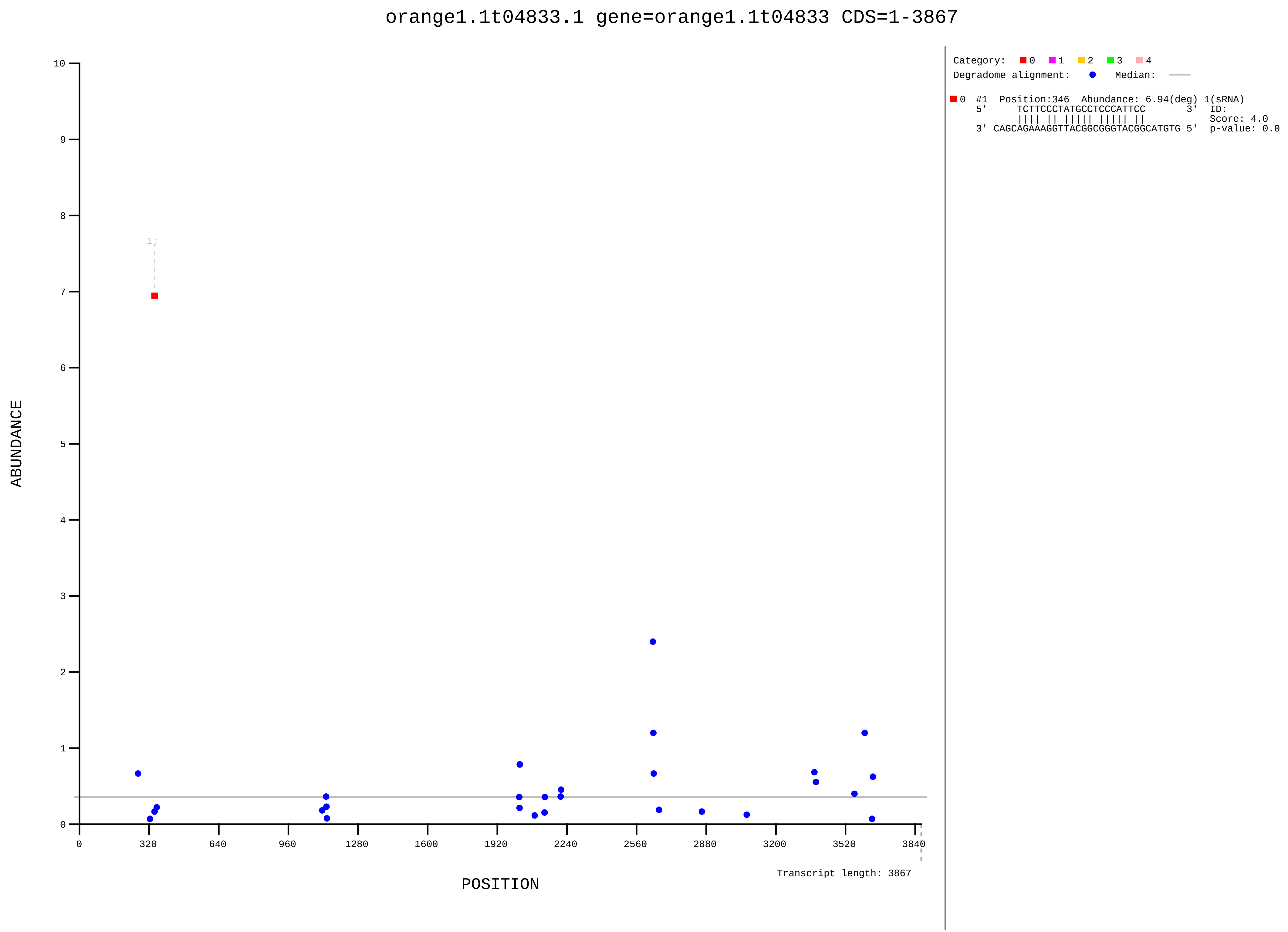
orange1.1t04833.1 gene=orange1.1t04833 CDS=1-3867
ABUNDANCE
POSITION
Transcript length: 3867
Category: 0 1 2 3 4
Degradome alignment:
Median:
0 #1 Position:346 Abundance: 6.94(deg) 1(sRNA)
5' TCTTCCCTATGCCTCCCATTCC 3' ID:
|||| || ||||| ||||| ||
3' CAGCAGAAAGGTTACGGCGGGTACGGCATGTG 5'
Score: 4.0
p-value: 0.0

Cs3g06640.1 gene=Cs3g06640 CDS=1-4224

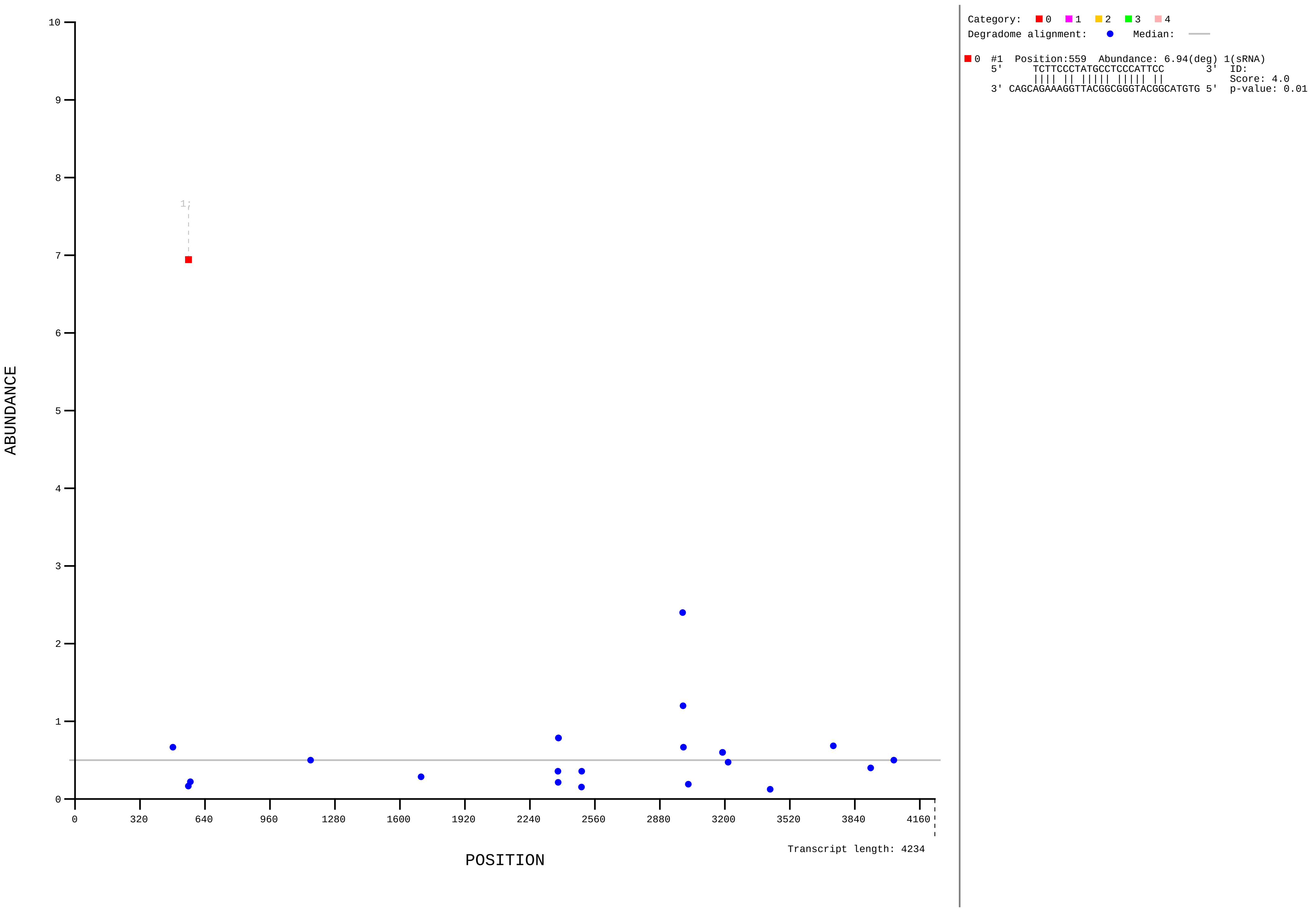

Cs3g06490.1 gene=Cs3g06490 CDS=1-4221

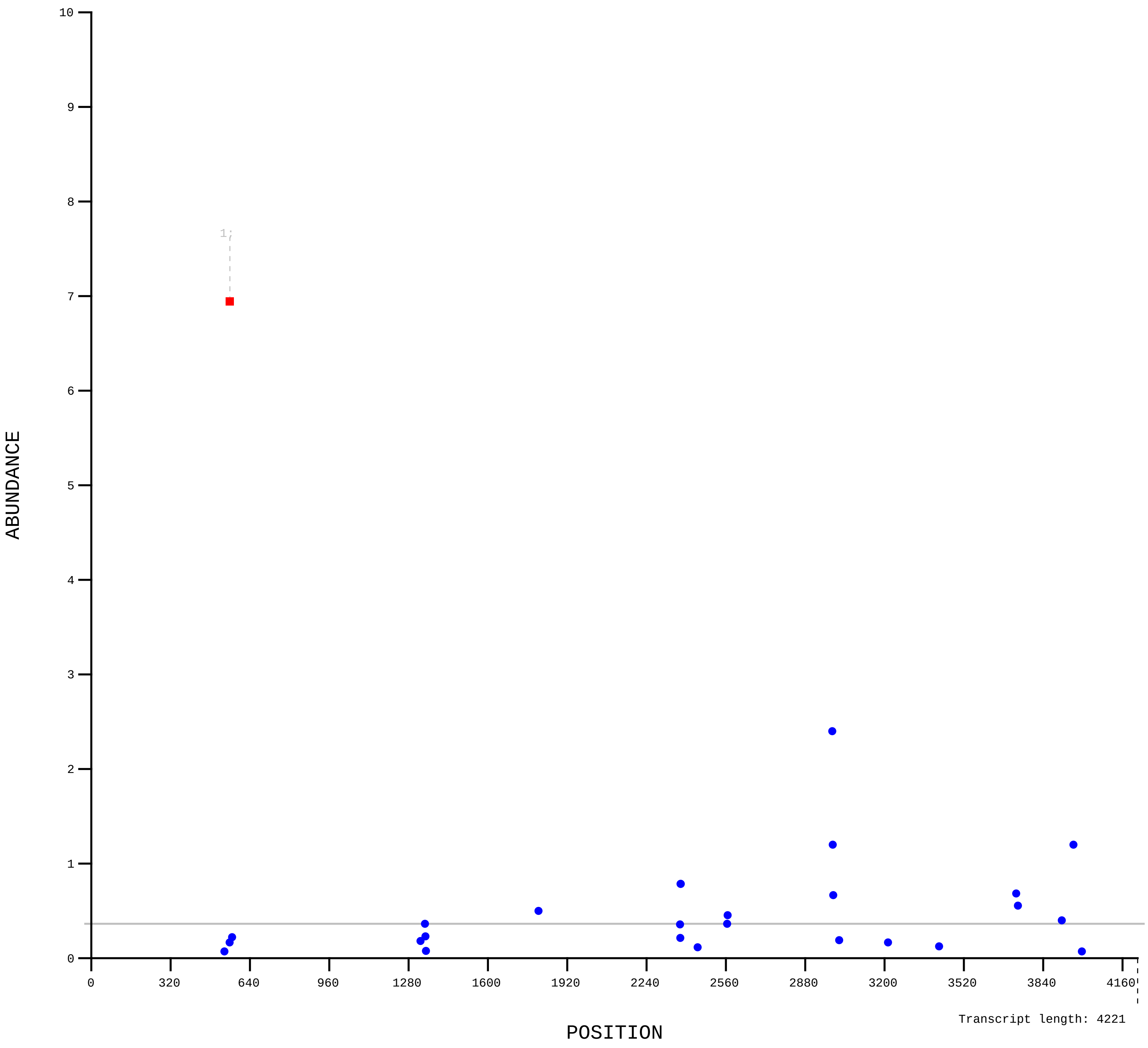

Category: 0 1 2 3 4
Degradome alignment: Median:

0 #1 Position:559 Abundance: 6.94(deg) 1(sRNA)

```
5'     TCTTCCCTATGCCTCCCATTCC       3'  ID:
       |||| || ||||| ||||| ||           Score: 4.0
3' CAGCAGAAAGGTTACGGCGGGTACGGCATGTG 5'  p-value: 0.0
```

Cs3g06600.1 gene=Cs3g06600 CDS=1-4824

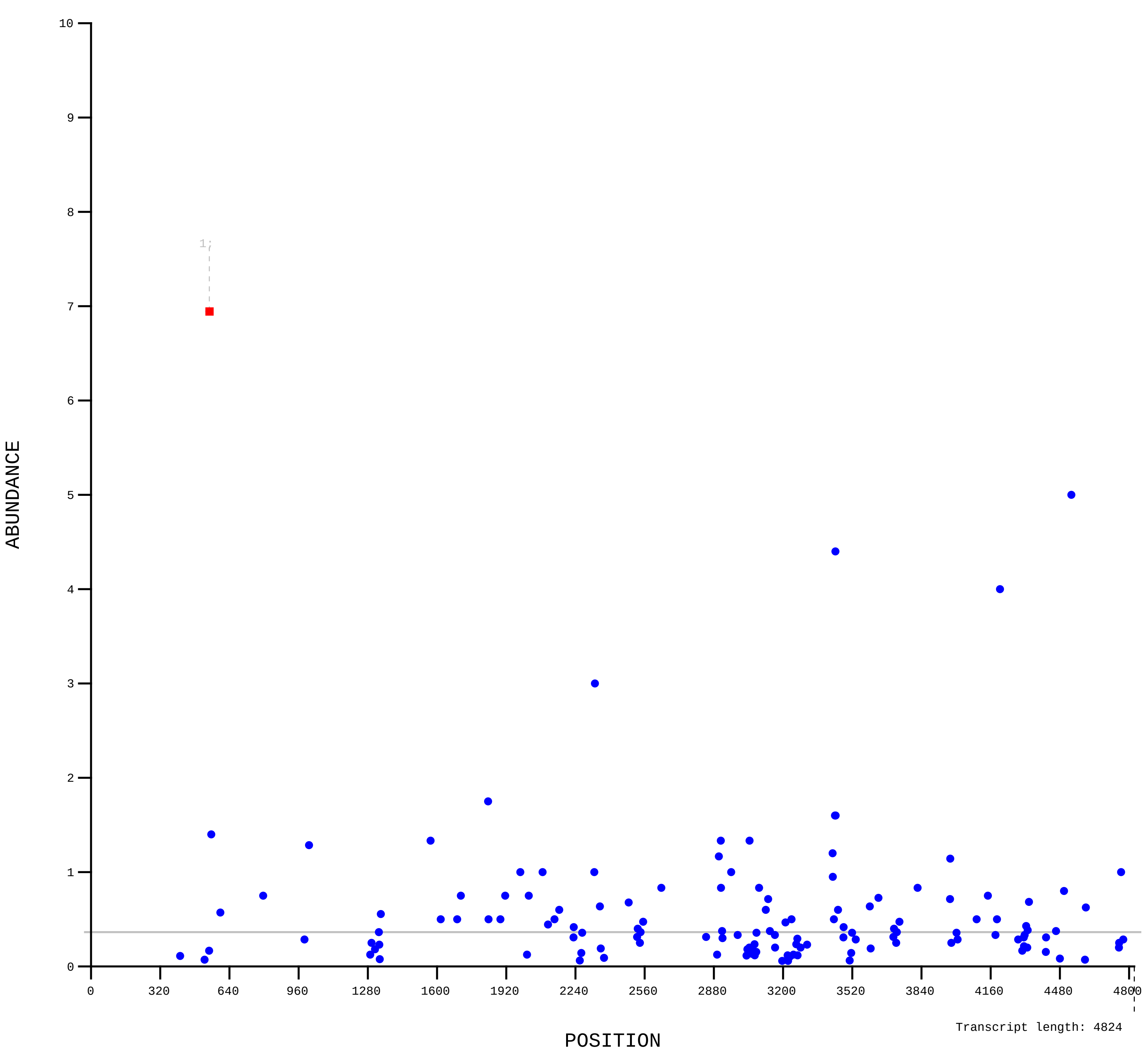

Category: 0 1 2 3 4
Degradome alignment: Median:

0 #1 Position:547 Abundance: 6.94(deg) 1(sRNA)

```
5'     TCTTCCCTATGCCTCCCATTCC       3'  ID:
       |||| || ||||| ||||| ||           Score: 4.0
3' CAGCAGAAAGGTTACGGCGGGTACGGCATGTG 5'  p-value: 0.01
```

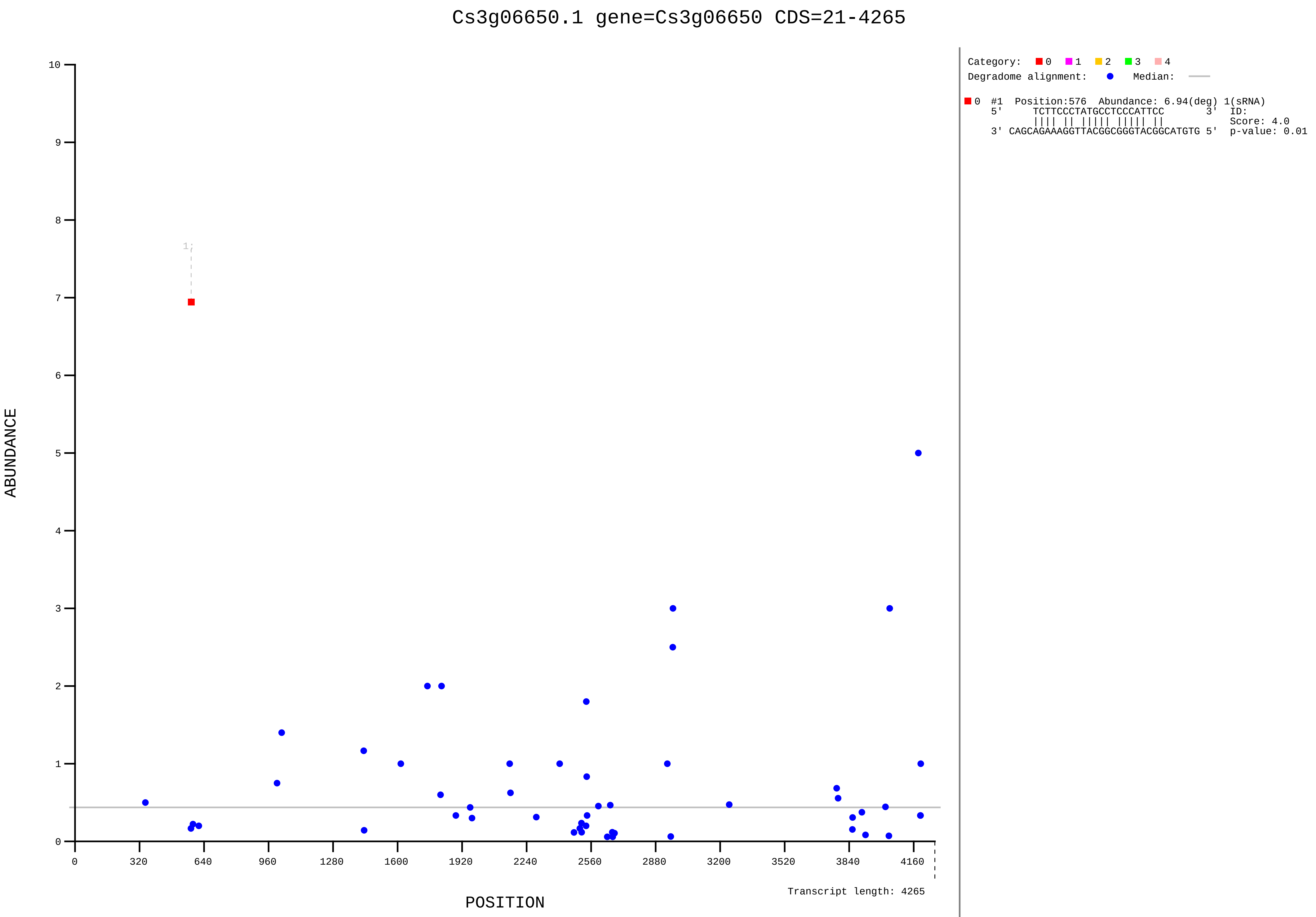

Cs3g06650.1 gene=Cs3g06650 CDS=21-4265
ABUNDANCE
POSITION
Transcript length: 4265
Category: 0 1 2 3 4
Degradome alignment:
Median:
0 #1 Position:576 Abundance: 6.94(deg) 1(sRNA)
5' TCTTCCCTATGCCTCCCATTCC 3' ID:
|||| || ||||| ||||| ||
Score: 4.0
3' CAGCAGAAAGGTTACGGCGGGTACGGCATGTG 5' p-value: 0.01

orange1.1t05193.1 gene=orange1.1t05193 CDS=1-1208

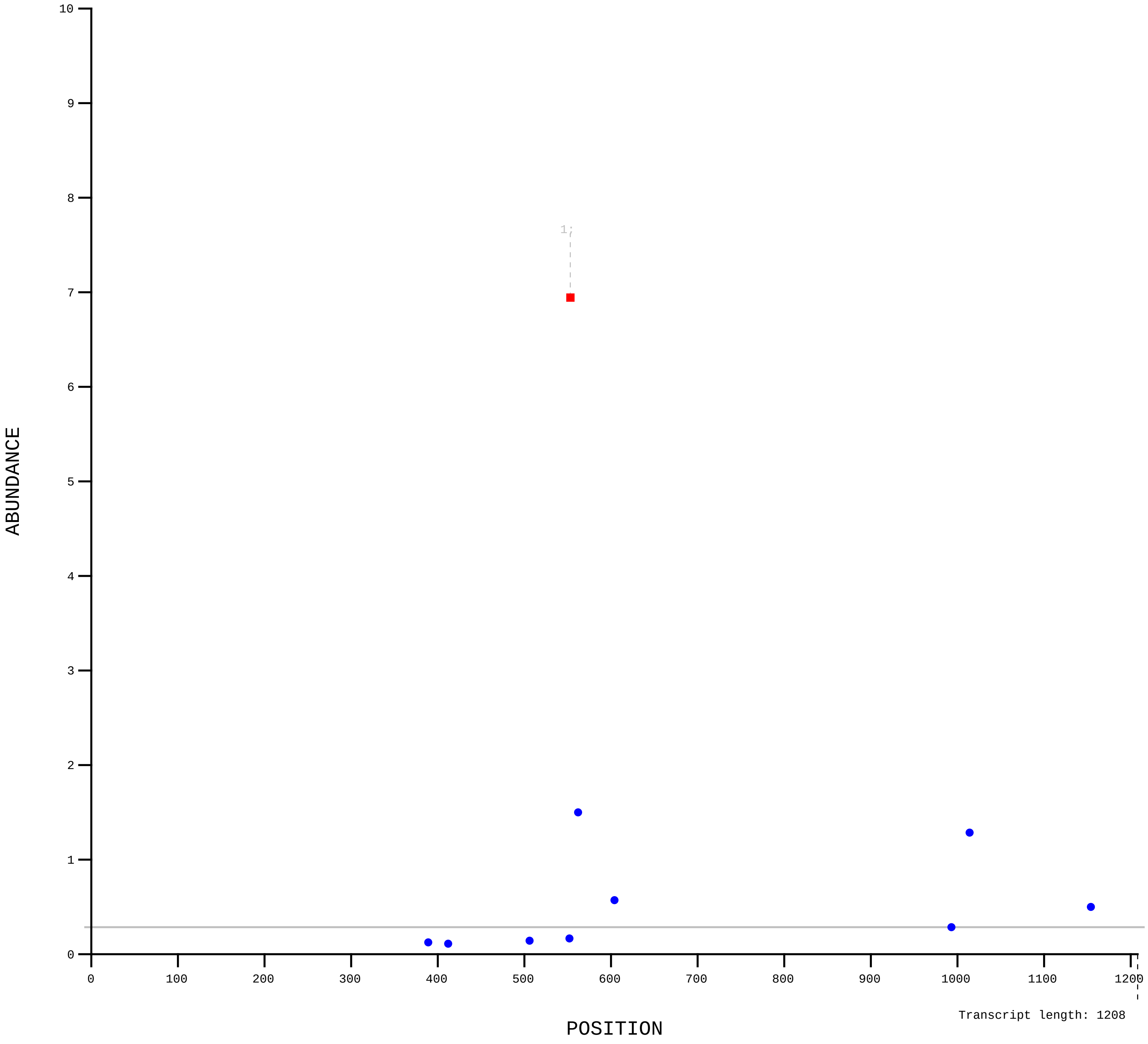

Category: 0 1 2 3 4
Degradome alignment: Median:

```
0  #1  Position:553  Abundance: 6.94(deg) 1(sRNA)
   5'      TCTTCCCTATGCCTCCCATTCC       3'  ID:
           |||| || ||||| ||||| ||           Score: 4.0
   3' CAGCAGAAAGGTTACGGCGGGTACGGCATGTG 5'  p-value: 0.0
```

Cs3g06590.1 gene=Cs3g06590 CDS=1-2781

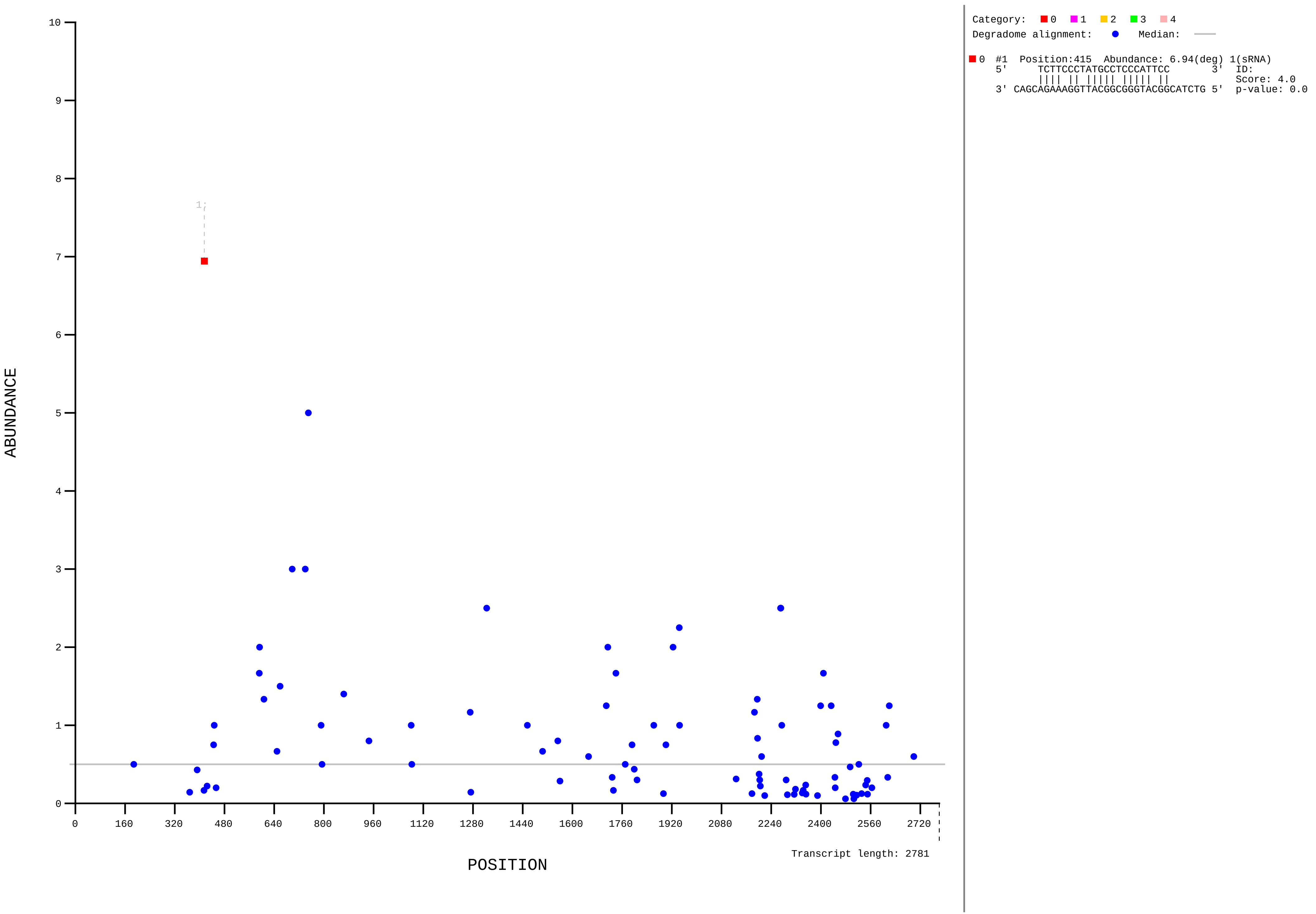

orange1.1t04933.1 gene=orange1.1t04933 CDS=1-1356

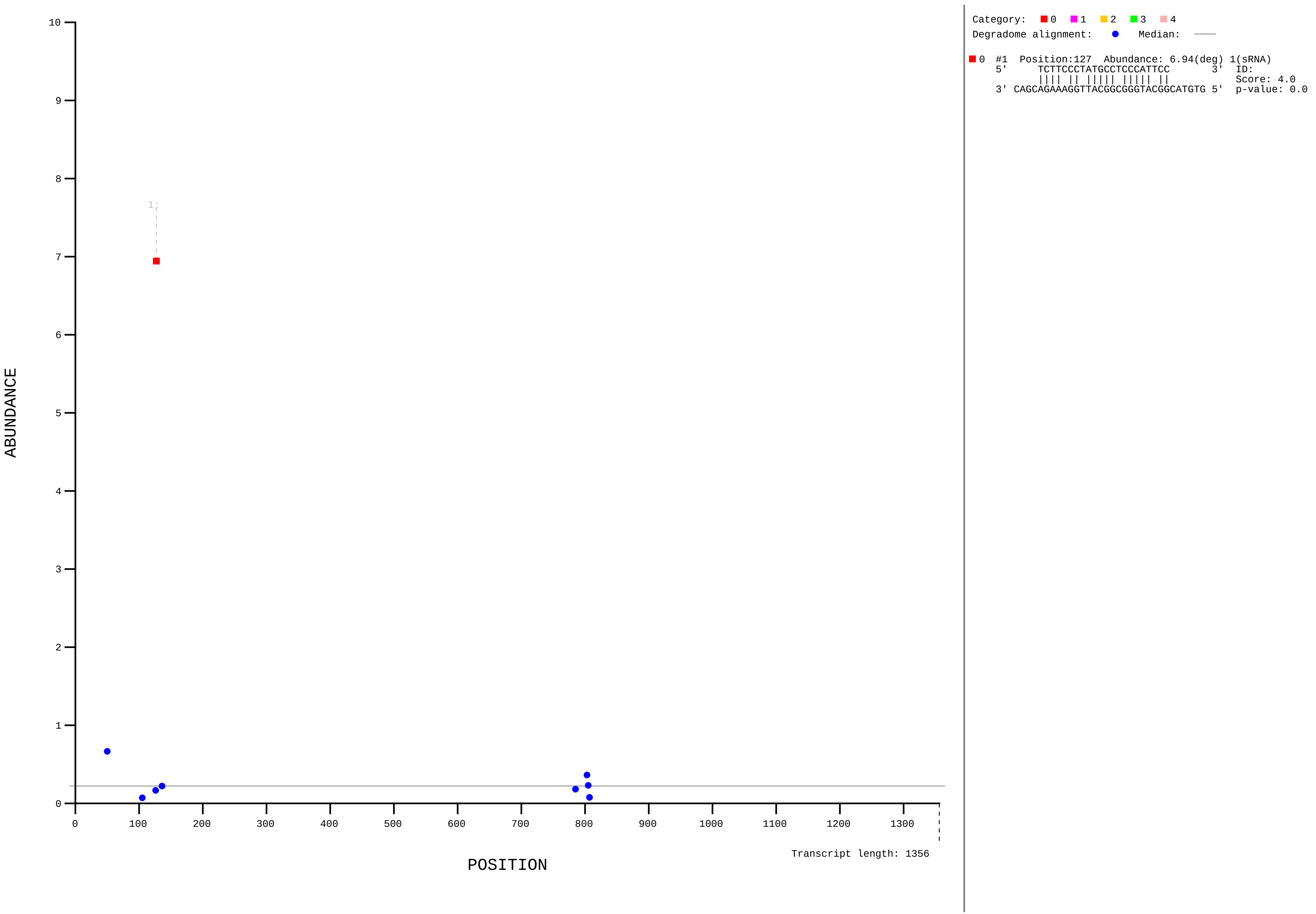

orange1.1t05295.1 gene=orange1.1t05295 CDS=17-980

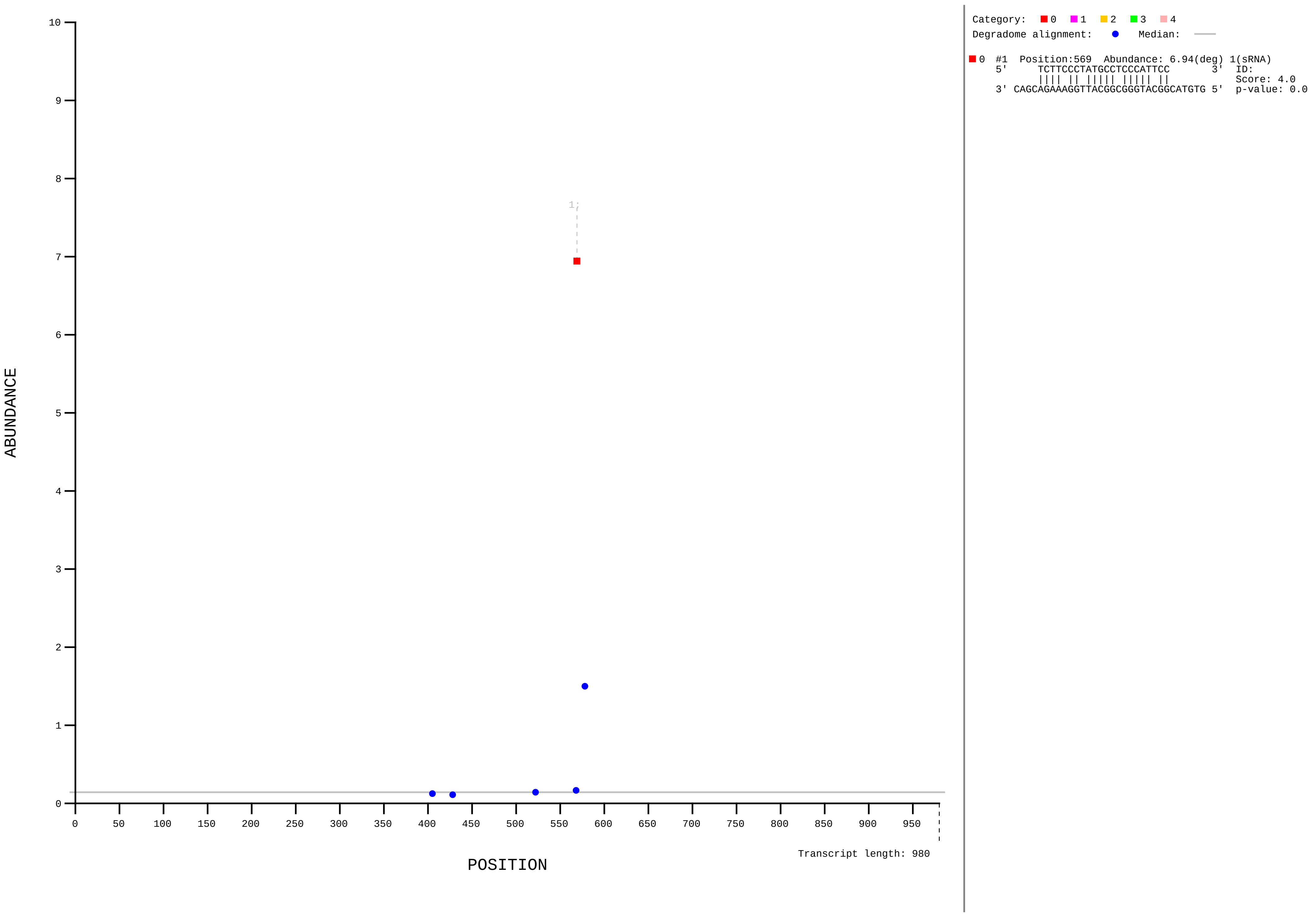

Cs3g06500.1 gene=Cs3g06500 CDS=1-2934

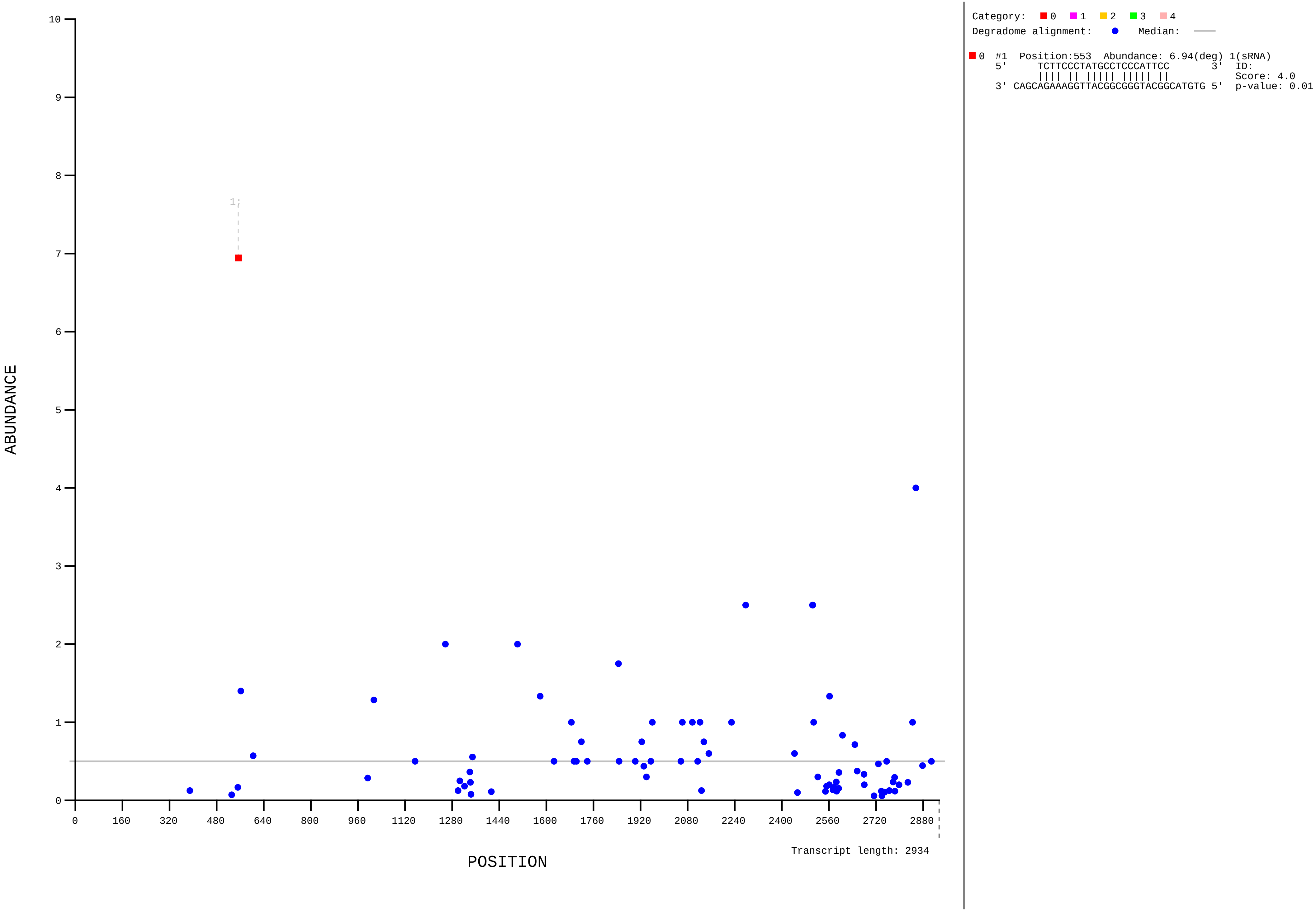

Cs3g04930.3 gene=Cs3g04930 CDS=150-3509

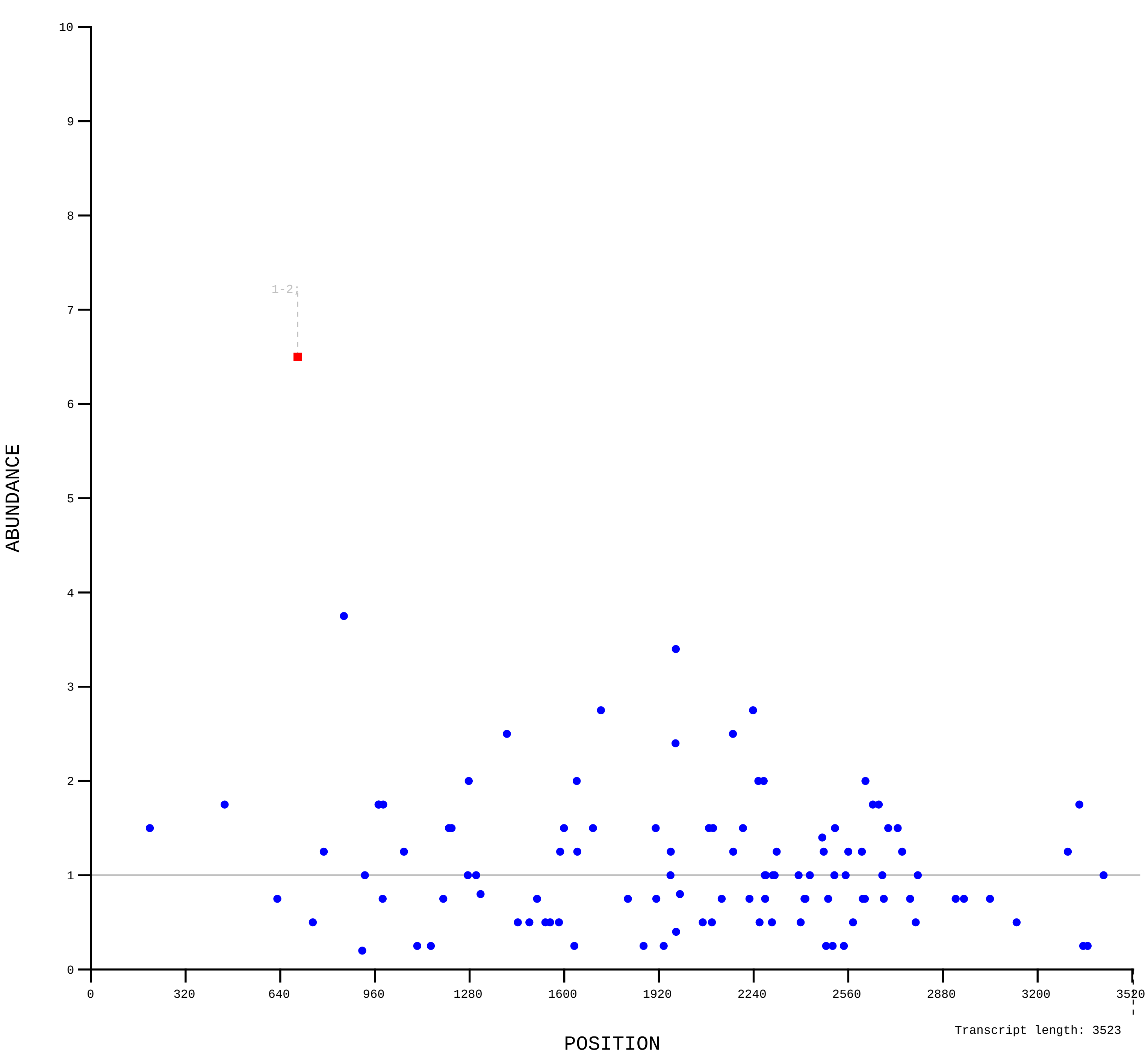

Category: 0 1 2 3 4
Degradome alignment: Median:

0 #1 Position:699 Abundance: 6.50(deg) 1(sRNA)
5' TTTTTCCCACACCTCCCATCCC 3' ID:
|||| || ||||| |||||||| Score: 3.0
3' CACCAAAACGGTTGTGGTGGGTAGGGTATGTT 5' p-value: 0.0

0 #2 Position:699 Abundance: 6.50(deg) 1(sRNA)
5' TCTTGCCCACCCCTCCCATTCC 3' ID:
| ||||| || || |||||o|| Score: 4.5
3' CACCAAAACGGTTGTGGTGGGTAGGGTATGTT 5' p-value: 0.02

Cs3g04930.1 gene=Cs3g04930 CDS=150-3866

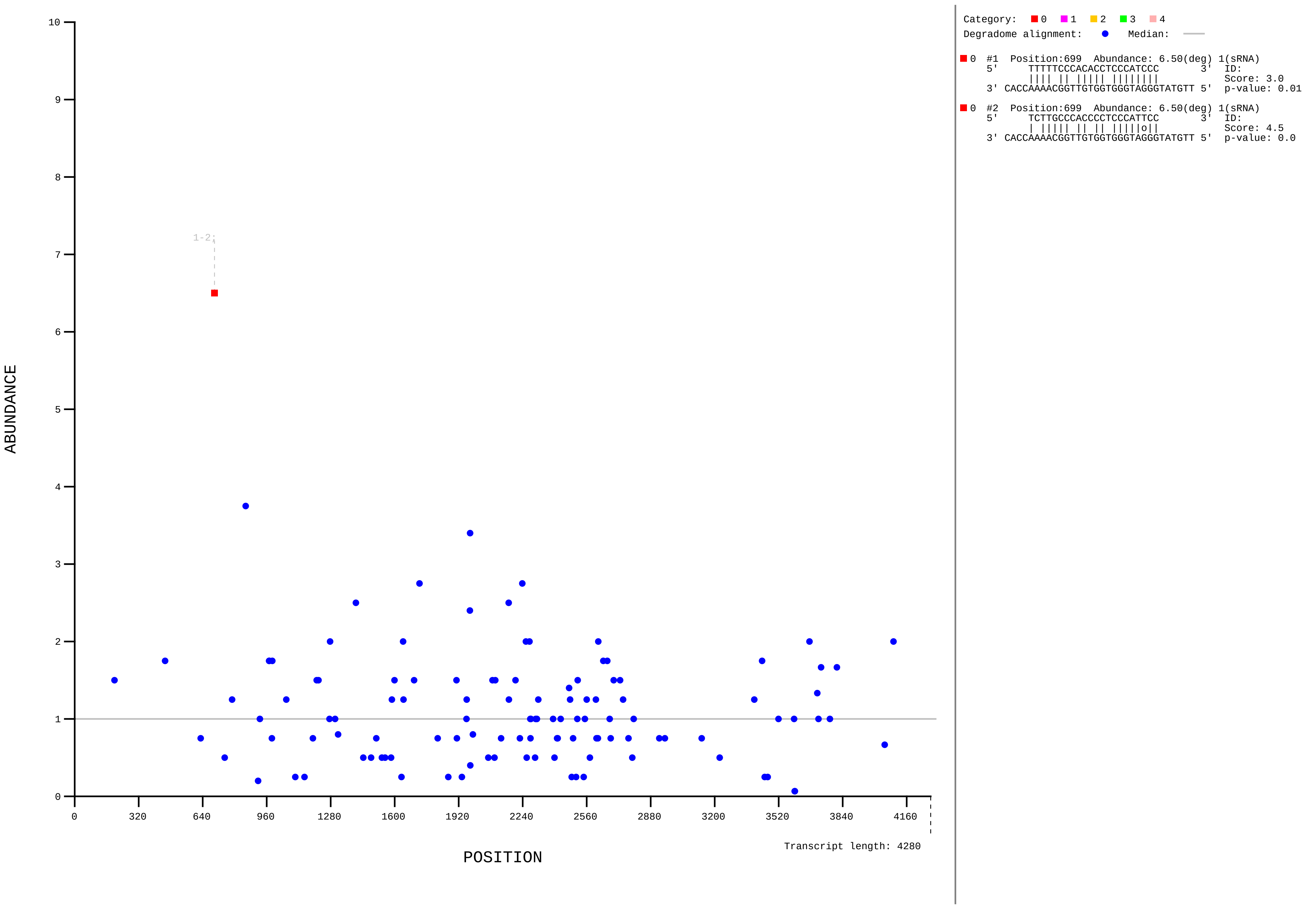

Cs3g04930.2 gene=Cs3g04930 CDS=150-3770

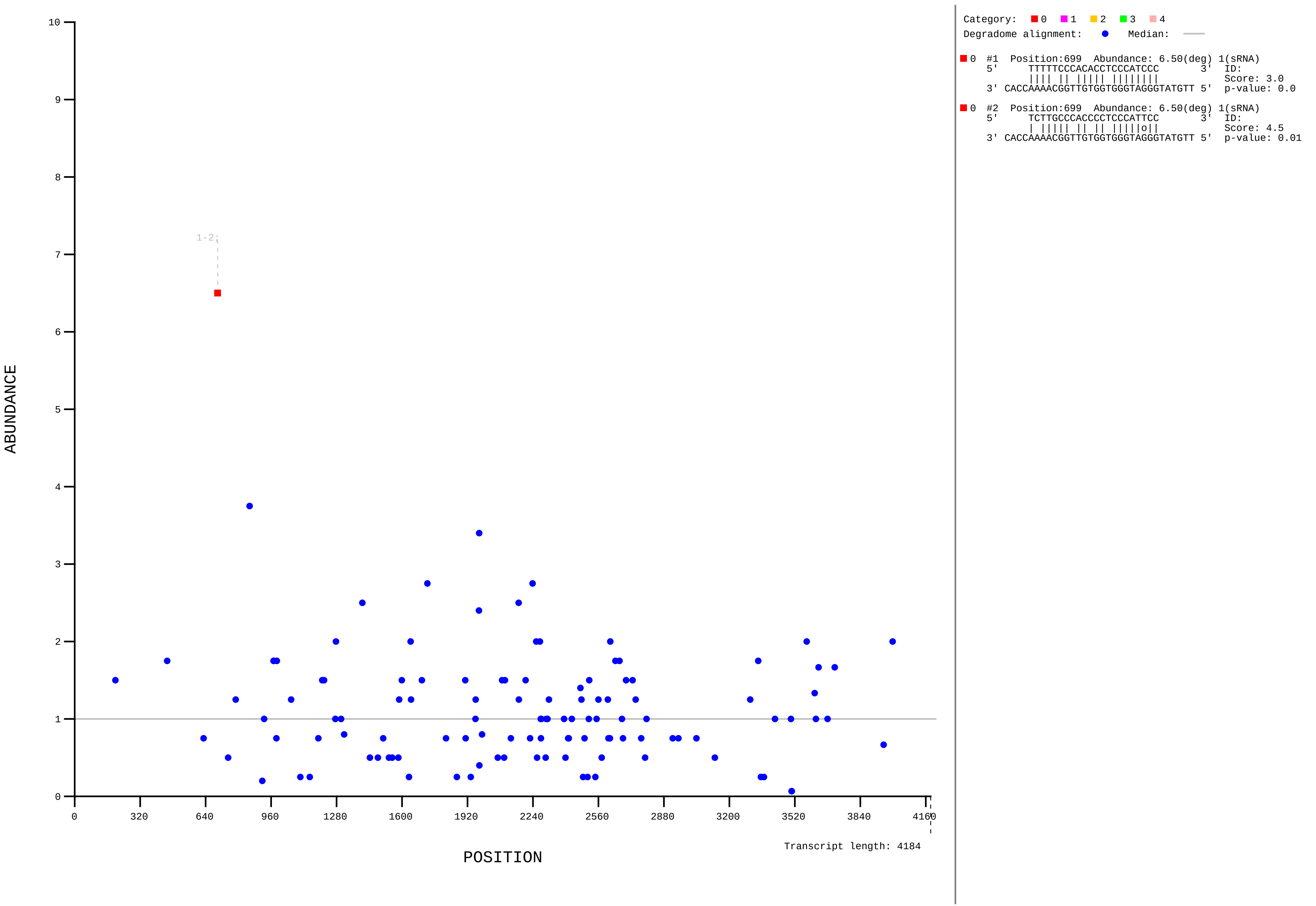

Cs3g04930.4 gene=Cs3g04930 CDS=456-4172

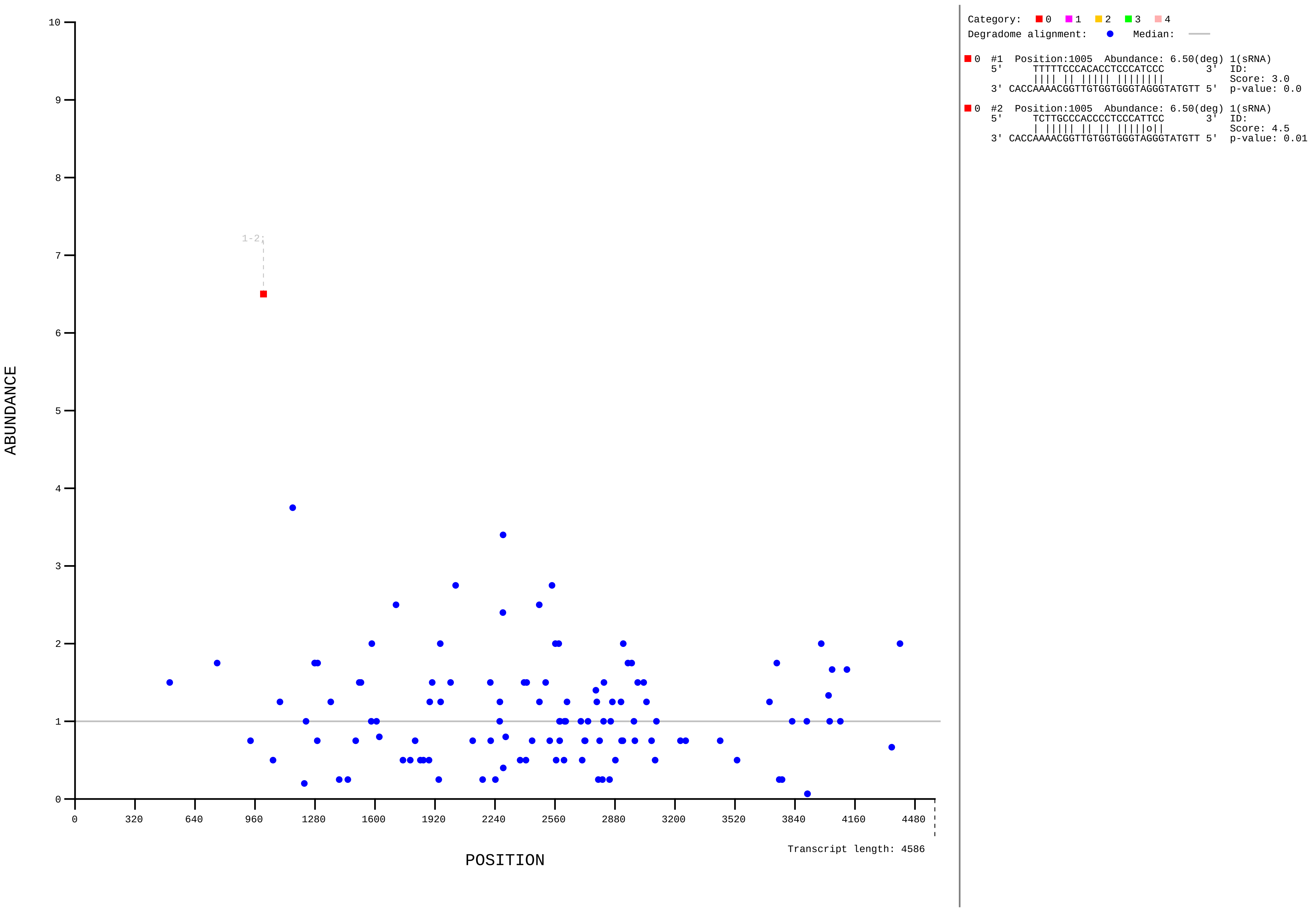

orange1.1t03349.3 gene=orange1.1t03349 CDS=165-2171

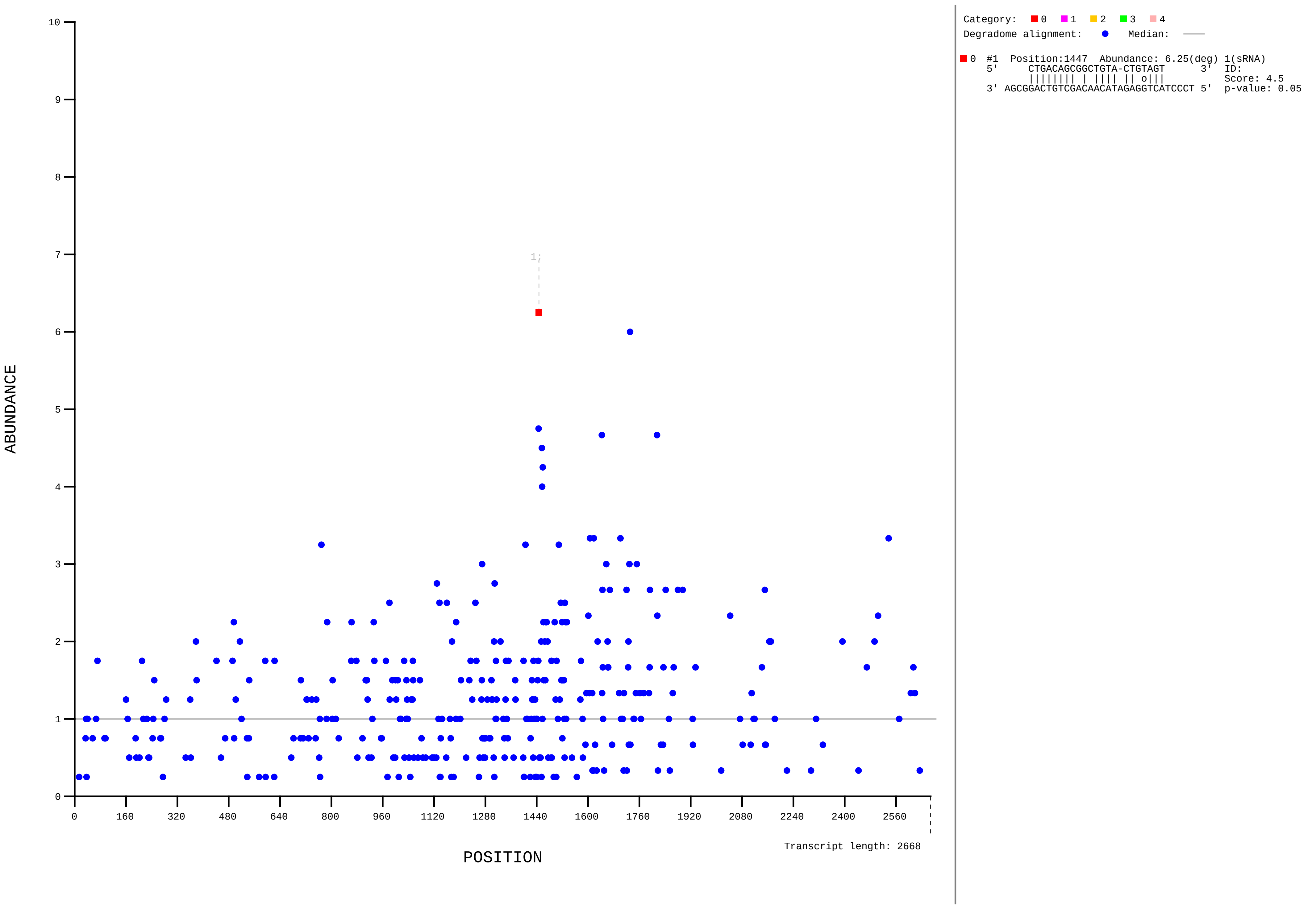

Cs1g24860.1 gene=Cs1g24860 CDS=450-1121

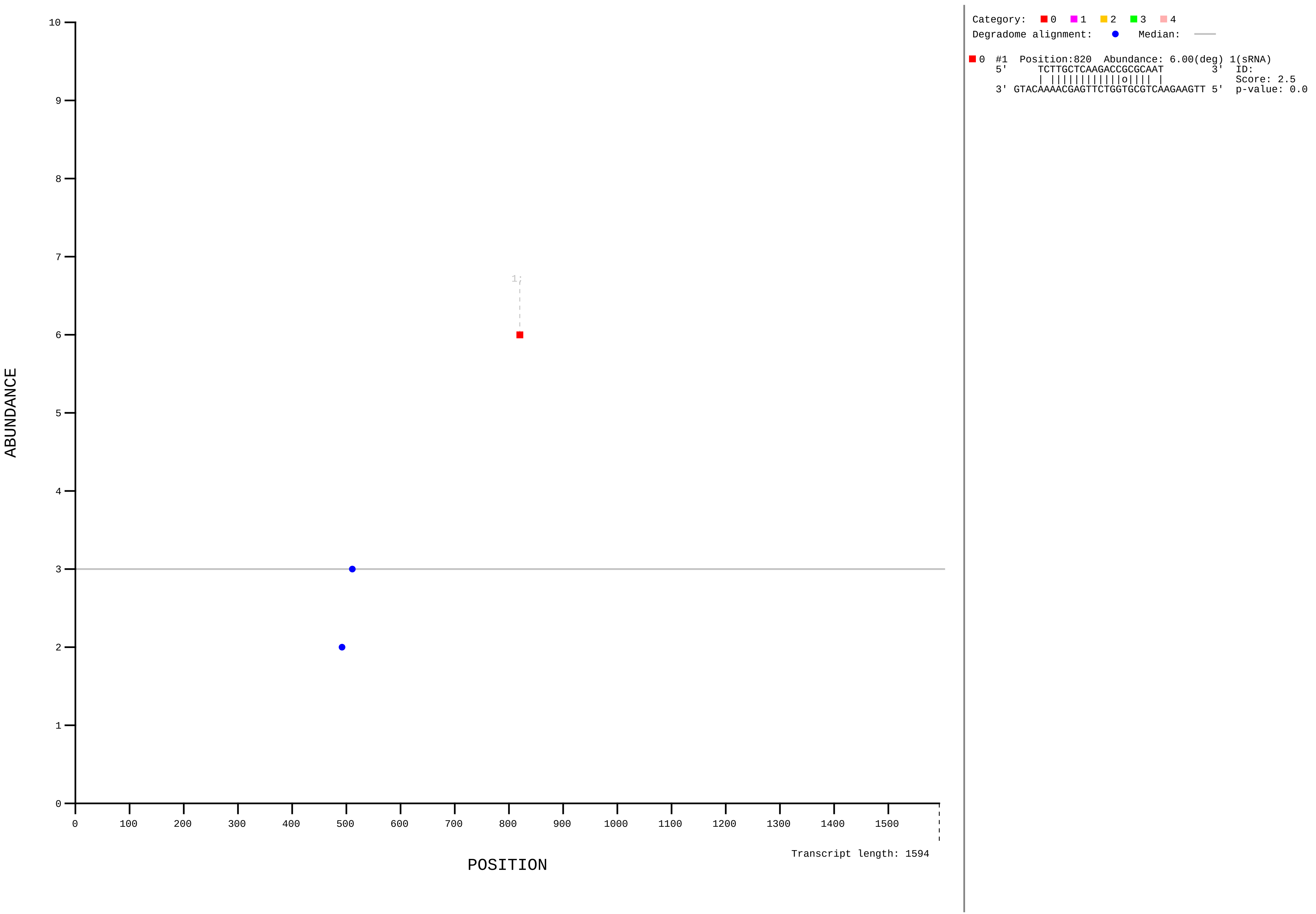

Cs6g07930.2 gene=Cs6g07930 CDS=84-1022

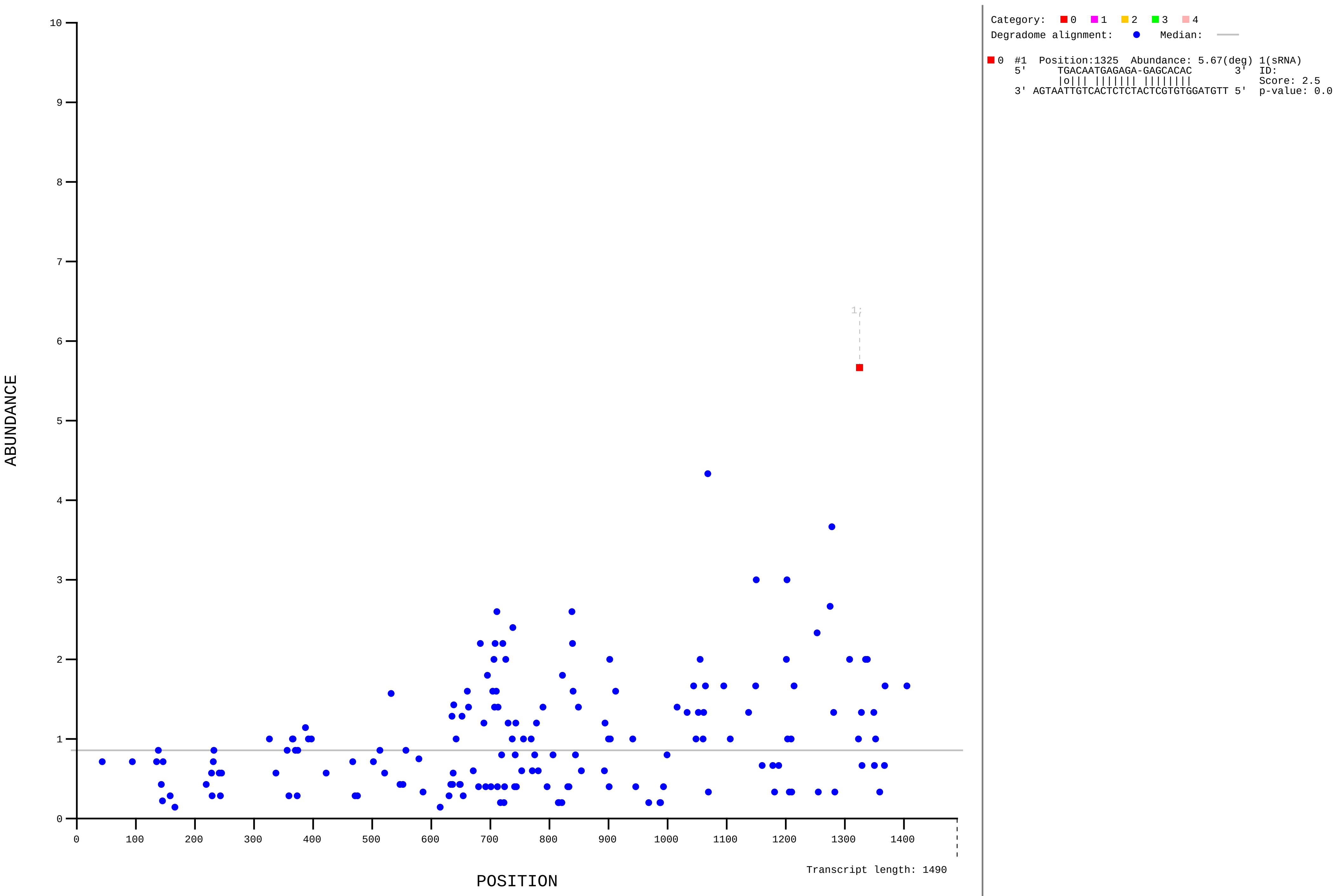

Cs6g07930.1 gene=Cs6g07930 CDS=84-947

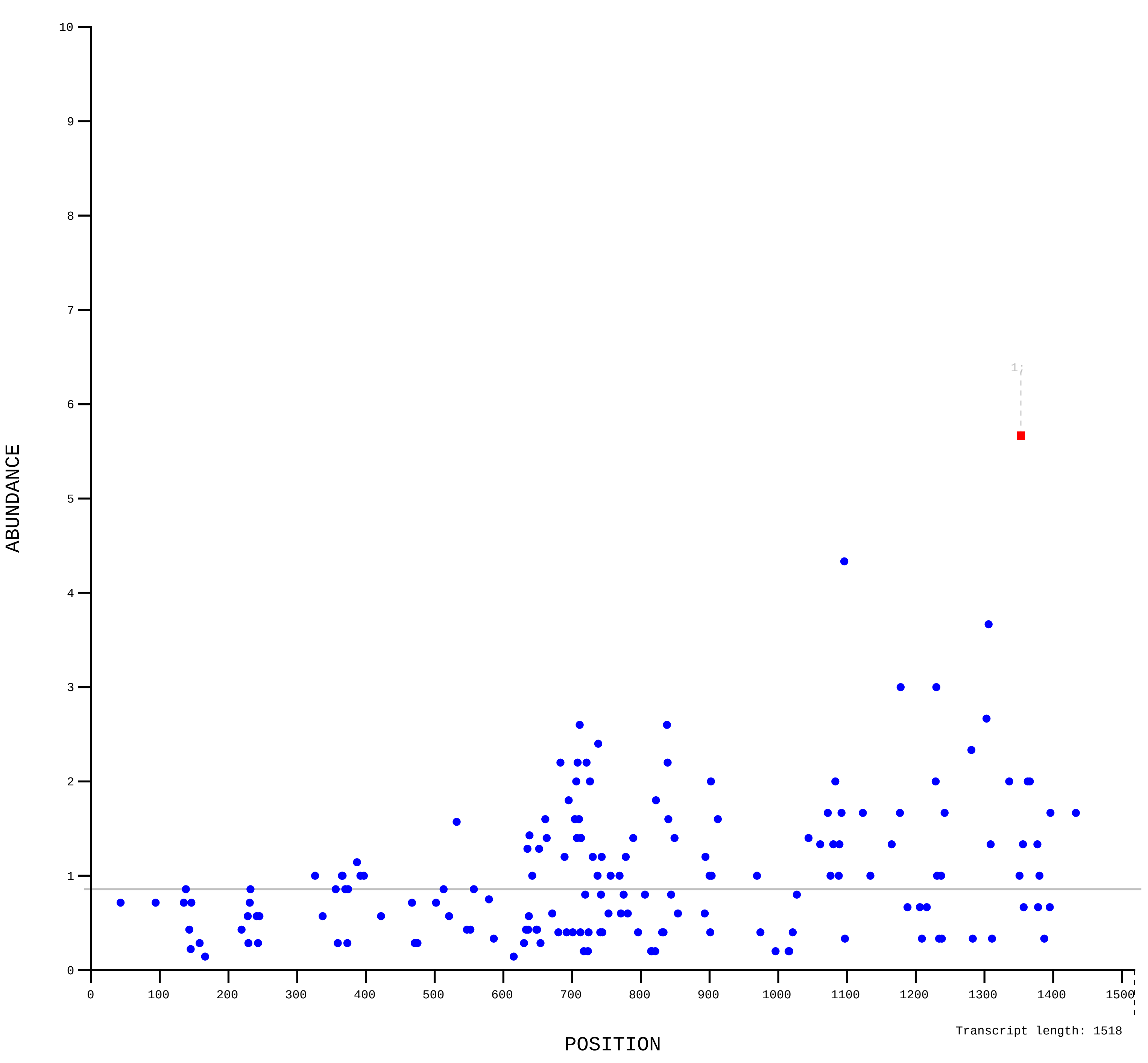

Category: 0 1 2 3 4
Degradome alignment: Median:

0 #1 Position:1353 Abundance: 5.67(deg) 1(sRNA)

```
5'     TGACAATGAGAGA-GAGCACAC       3'  ID:
       |o||| ||||||| ||||||||           Score: 2.5
3' AGTAATTGTCACTCTCTACTCGTGTGGATGTT 5'  p-value: 0.0
```

Cs6g07930.3 gene=Cs6g07930 CDS=220-1206

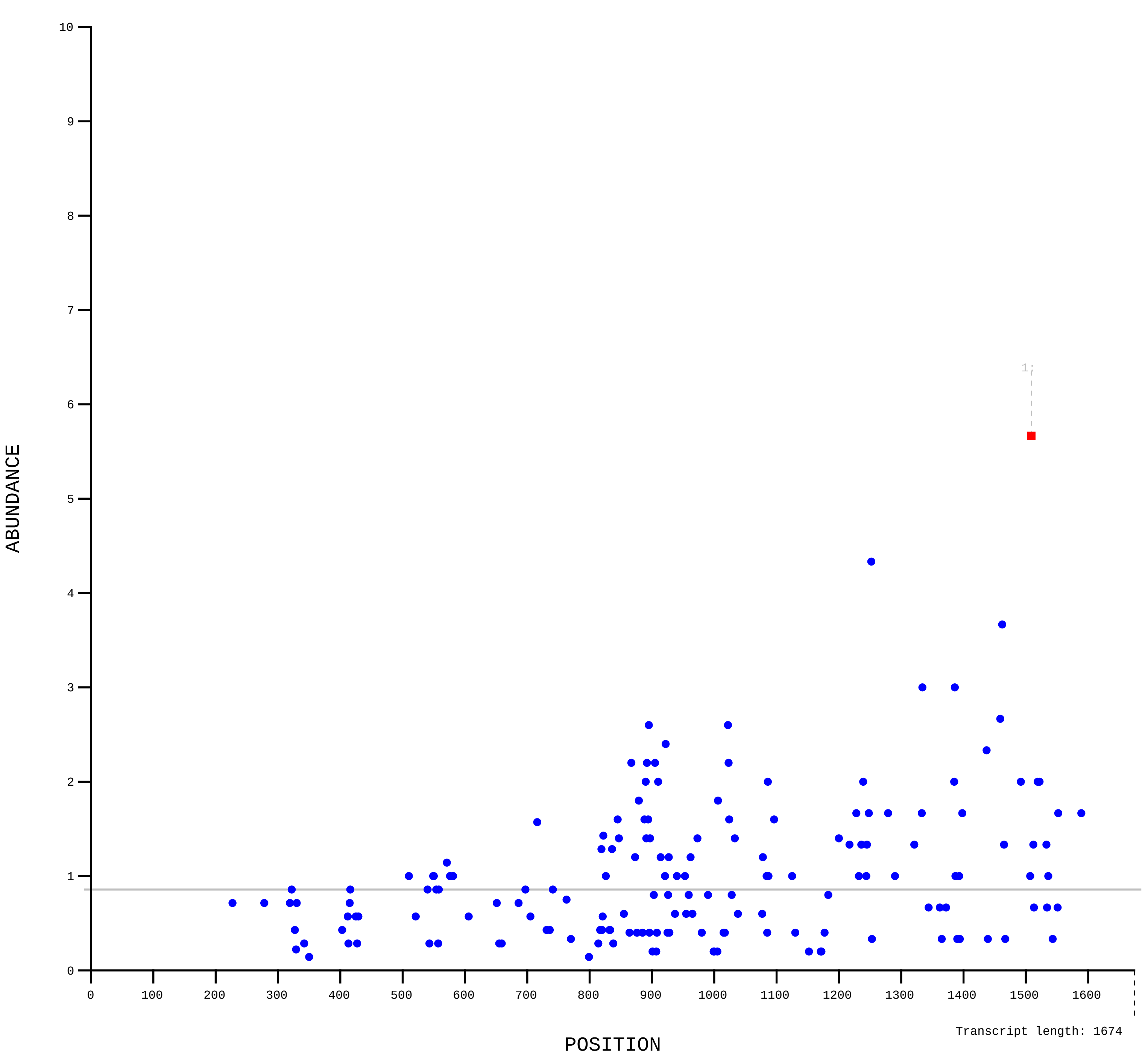

Category: 0 1 2 3 4
Degradome alignment: Median:

0 #1 Position:1509 Abundance: 5.67(deg) 1(sRNA)

```
5'      TGACAATGAGAGA-GAGCACAC       3'  ID:
        |o||| ||||||| ||||||||           Score: 2.5
3' AGTAATTGTCACTCTCTACTCGTGTGGATGTT 5'  p-value: 0.0
```

Cs2g23550.4 gene=Cs2g23550 CDS=389-958

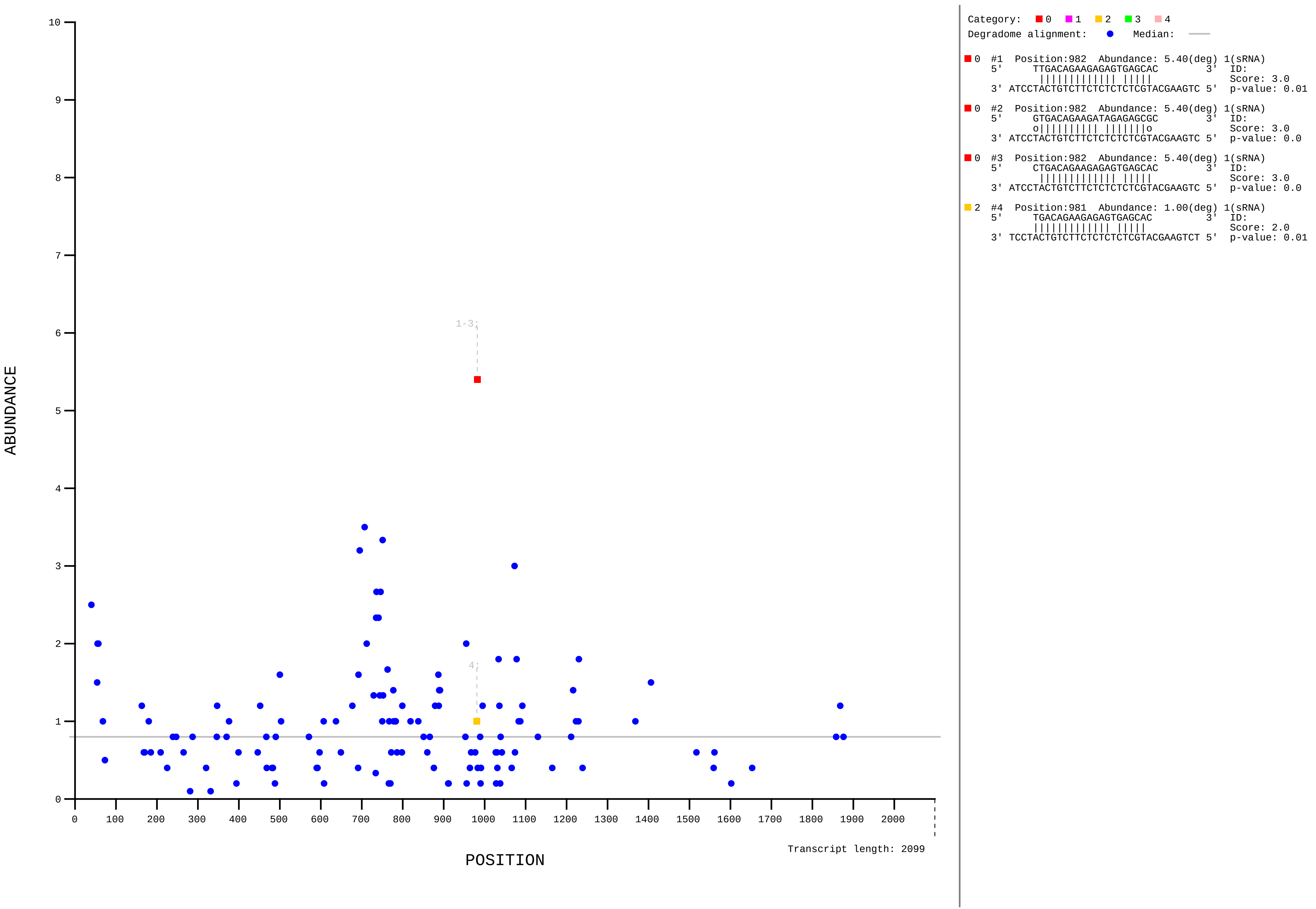

Cs2g23550.5 gene=Cs2g23550 CDS=310-774

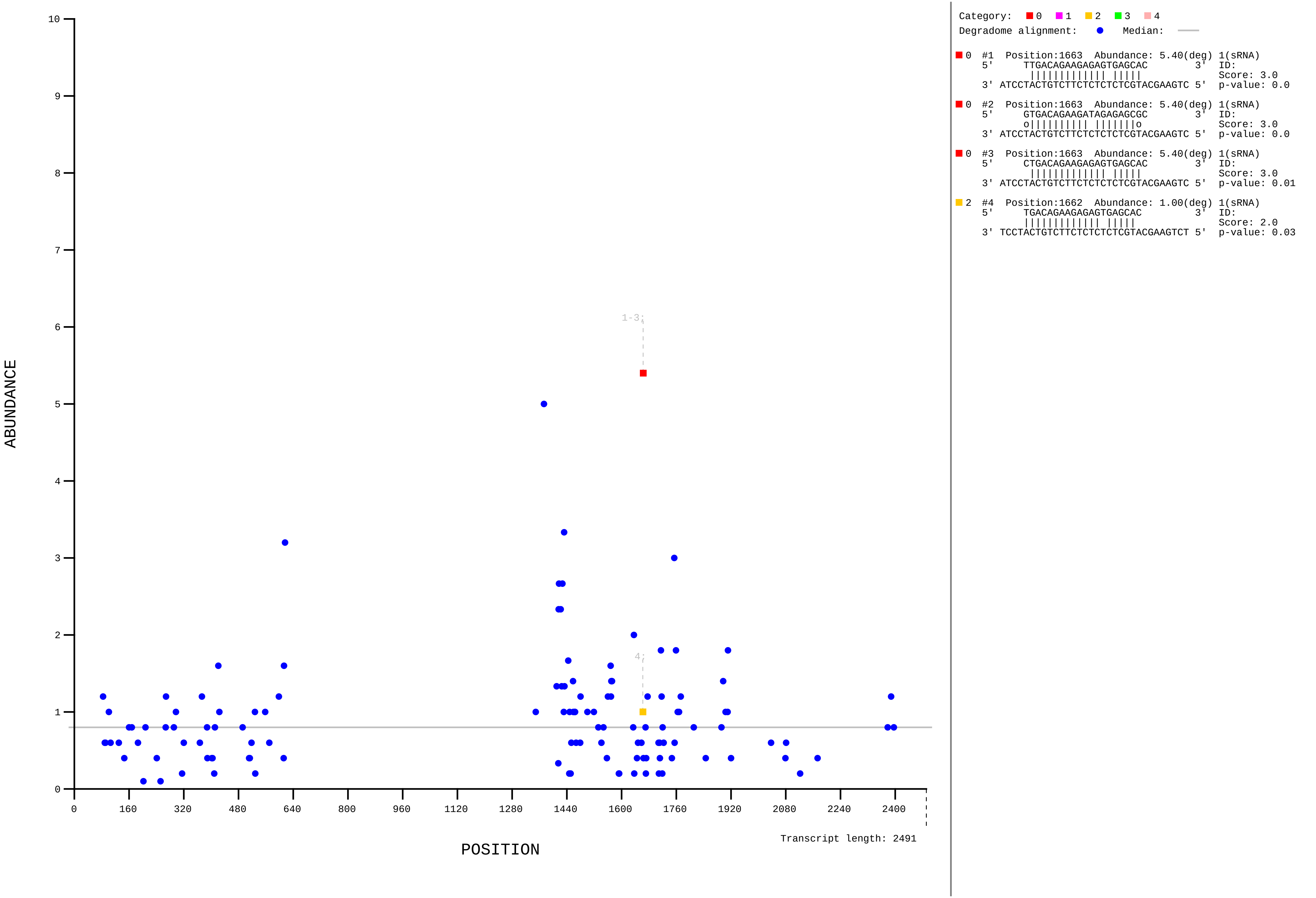

Cs2g23550.3 gene=Cs2g23550 CDS=310-831

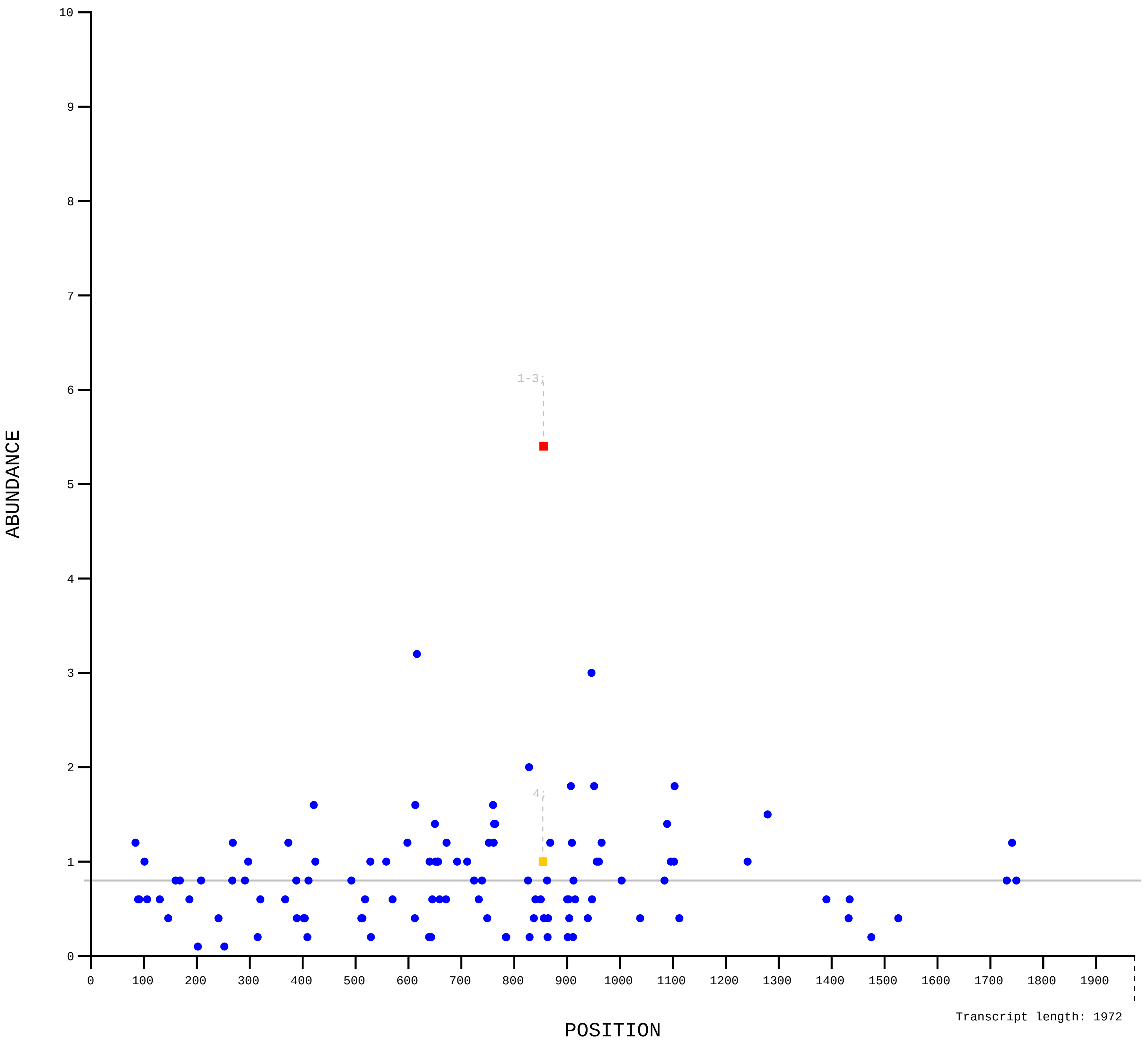


```
Category:  0  1  2  3  4
Degradome alignment:     Median:

0  #1  Position:855  Abundance: 5.40(deg) 1(sRNA)
   5'     TTGACAGAAGAGAGTGAGCAC        3'  ID:
           |||||||||||||| |||||             Score: 3.0
   3' ATCCTACTGTCTTCTCTCTCTCGTACGAAGTC 5'  p-value: 0.0

0  #2  Position:855  Abundance: 5.40(deg) 1(sRNA)
   5'     GTGACAGAAGATAGAGAGCGC        3'  ID:
          o|||||||||| |||||||o              Score: 3.0
   3' ATCCTACTGTCTTCTCTCTCTCGTACGAAGTC 5'  p-value: 0.0

0  #3  Position:855  Abundance: 5.40(deg) 1(sRNA)
   5'     CTGACAGAAGAGAGTGAGCAC        3'  ID:
           |||||||||||||| |||||             Score: 3.0
   3' ATCCTACTGTCTTCTCTCTCTCGTACGAAGTC 5'  p-value: 0.0

2  #4  Position:854  Abundance: 1.00(deg) 1(sRNA)
   5'     TGACAGAAGAGAGTGAGCAC         3'  ID:
          |||||||||||||| |||||              Score: 2.0
   3' TCCTACTGTCTTCTCTCTCTCGTACGAAGTCT 5'  p-value: 0.02
```

Cs2g23550.2 gene=Cs2g23550 CDS=310-879

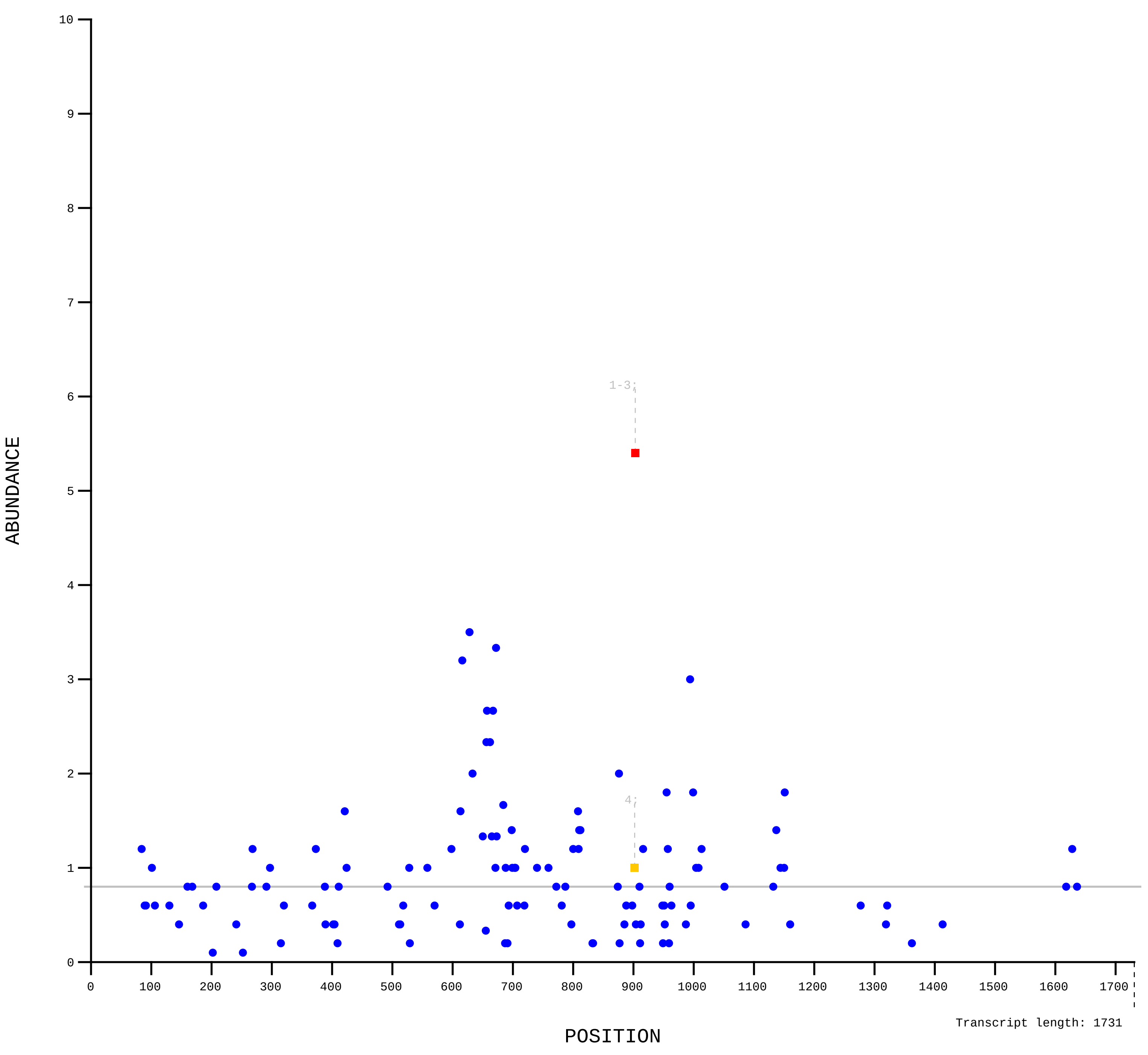


```
Category:  0  1  2  3  4
Degradome alignment:    Median:

0  #1  Position:903  Abundance: 5.40(deg) 1(sRNA)
   5'      TTGACAGAAGAGAGTGAGCAC        3'  ID:
            ||||||||||||| |||||             Score: 3.0
   3' ATCCTACTGTCTTCTCTCTCTCGTACGAAGTC 5'  p-value: 0.01

0  #2  Position:903  Abundance: 5.40(deg) 1(sRNA)
   5'      GTGACAGAAGATAGAGAGCGC        3'  ID:
           o|||||||||| |||||||o             Score: 3.0
   3' ATCCTACTGTCTTCTCTCTCTCGTACGAAGTC 5'  p-value: 0.01

0  #3  Position:903  Abundance: 5.40(deg) 1(sRNA)
   5'      CTGACAGAAGAGAGTGAGCAC        3'  ID:
            ||||||||||||| |||||             Score: 3.0
   3' ATCCTACTGTCTTCTCTCTCTCGTACGAAGTC 5'  p-value: 0.01

2  #4  Position:902  Abundance: 1.00(deg) 1(sRNA)
   5'      TGACAGAAGAGAGTGAGCAC         3'  ID:
           ||||||||||||| |||||              Score: 2.0
   3' TCCTACTGTCTTCTCTCTCTCGTACGAAGTCT 5'  p-value: 0.03
```

Cs2g23550.1 gene=Cs2g23550 CDS=389-910

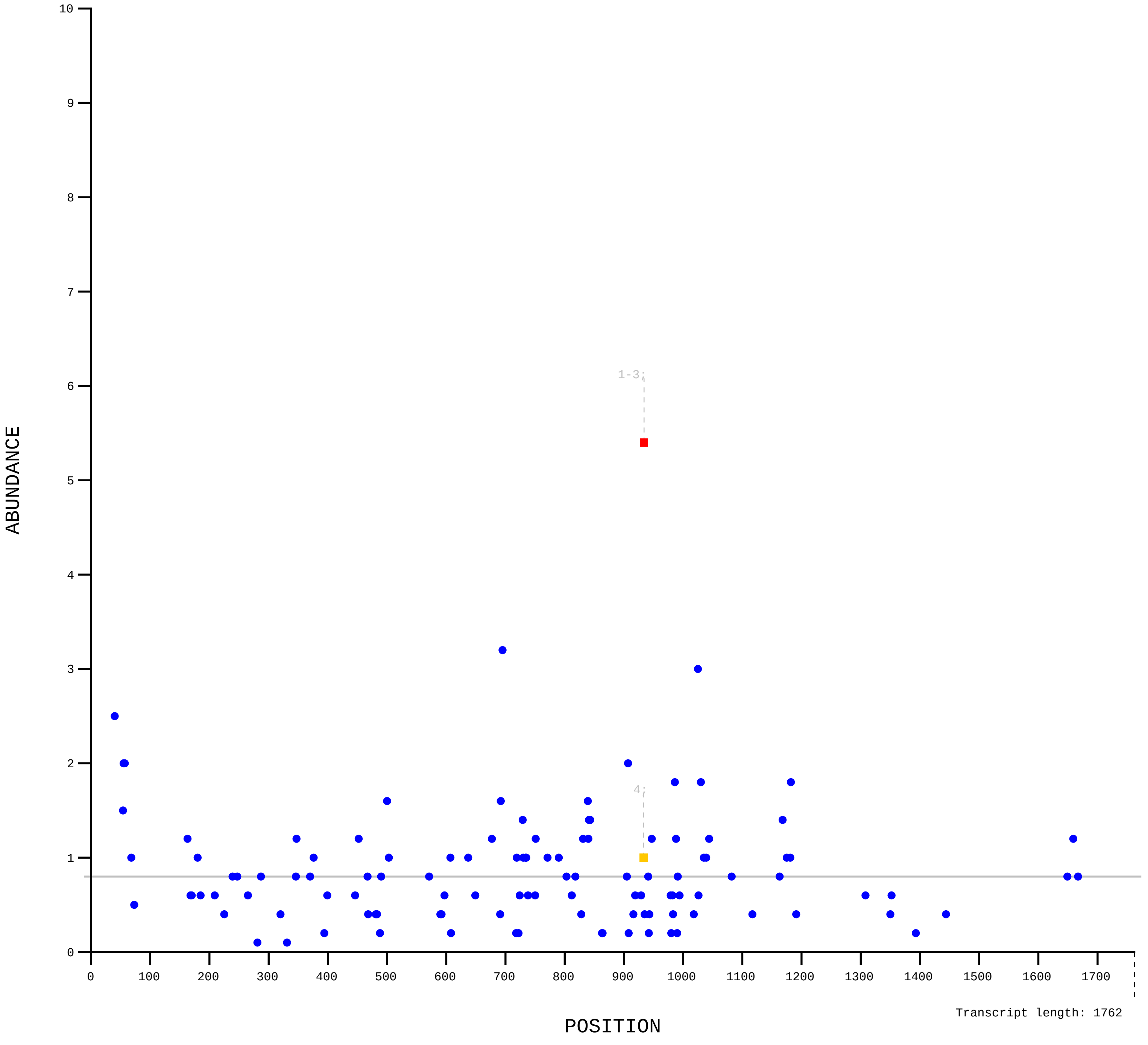


```
Category: 0 1 2 3 4
Degradome alignment:        Median:

0  #1  Position:934  Abundance: 5.40(deg) 1(sRNA)
   5'     TTGACAGAAGAGAGTGAGCAC        3'  ID:
           ||||||||||||| |||||             Score: 3.0
   3' ATCCTACTGTCTTCTCTCTCTCGTACGAAGTC 5'  p-value: 0.01

0  #2  Position:934  Abundance: 5.40(deg) 1(sRNA)
   5'     GTGACAGAAGATAGAGAGCGC        3'  ID:
          o|||||||||| |||||||o             Score: 3.0
   3' ATCCTACTGTCTTCTCTCTCTCGTACGAAGTC 5'  p-value: 0.0

0  #3  Position:934  Abundance: 5.40(deg) 1(sRNA)
   5'     CTGACAGAAGAGAGTGAGCAC        3'  ID:
           ||||||||||||| |||||             Score: 3.0
   3' ATCCTACTGTCTTCTCTCTCTCGTACGAAGTC 5'  p-value: 0.02

2  #4  Position:933  Abundance: 1.00(deg) 1(sRNA)
   5'     TGACAGAAGAGAGTGAGCAC         3'  ID:
          ||||||||||||| |||||              Score: 2.0
   3' TCCTACTGTCTTCTCTCTCTCGTACGAAGTCT 5'  p-value: 0.03
```

Cs6g16030.7 gene=Cs6g16030 CDS=495-2534

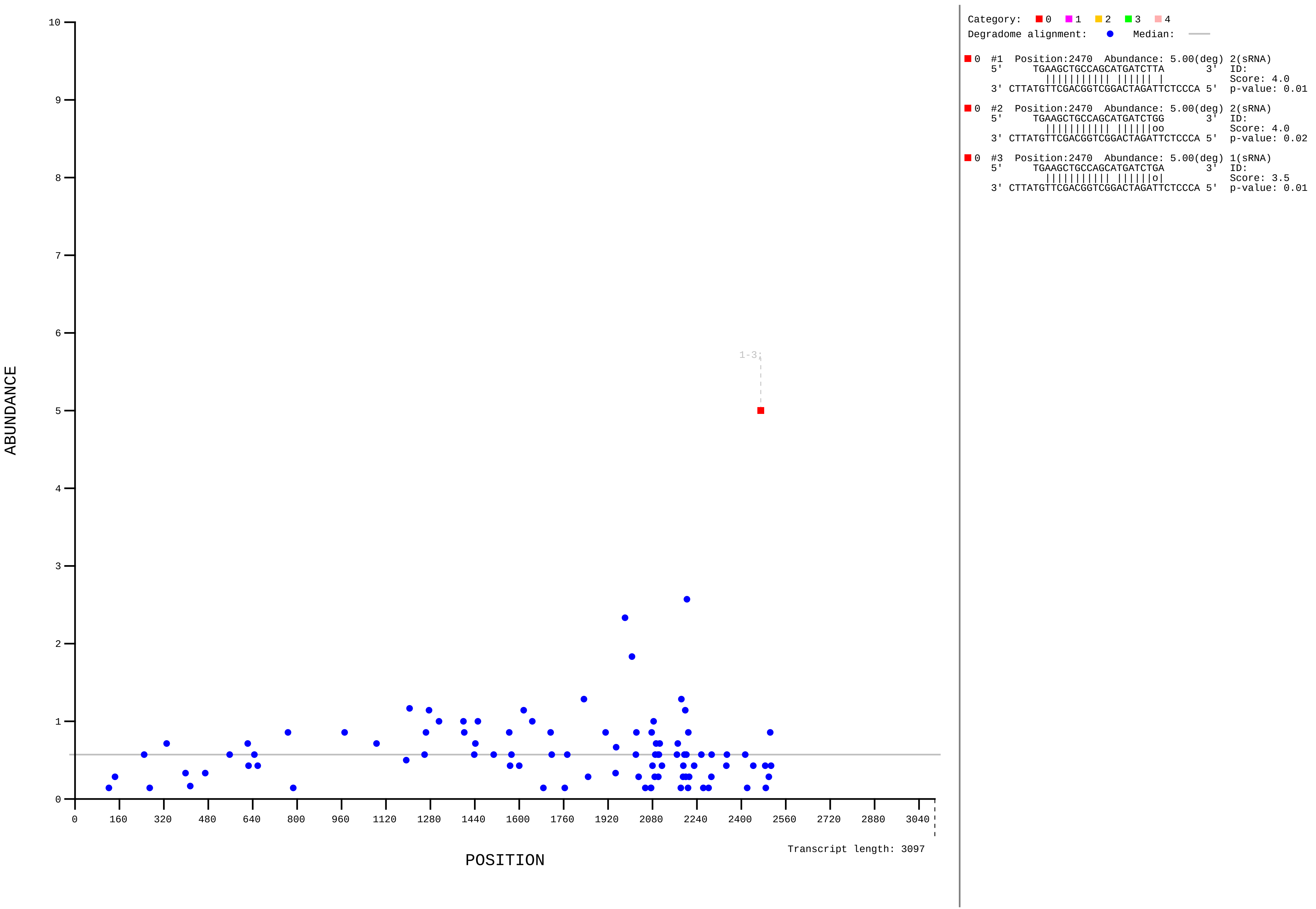

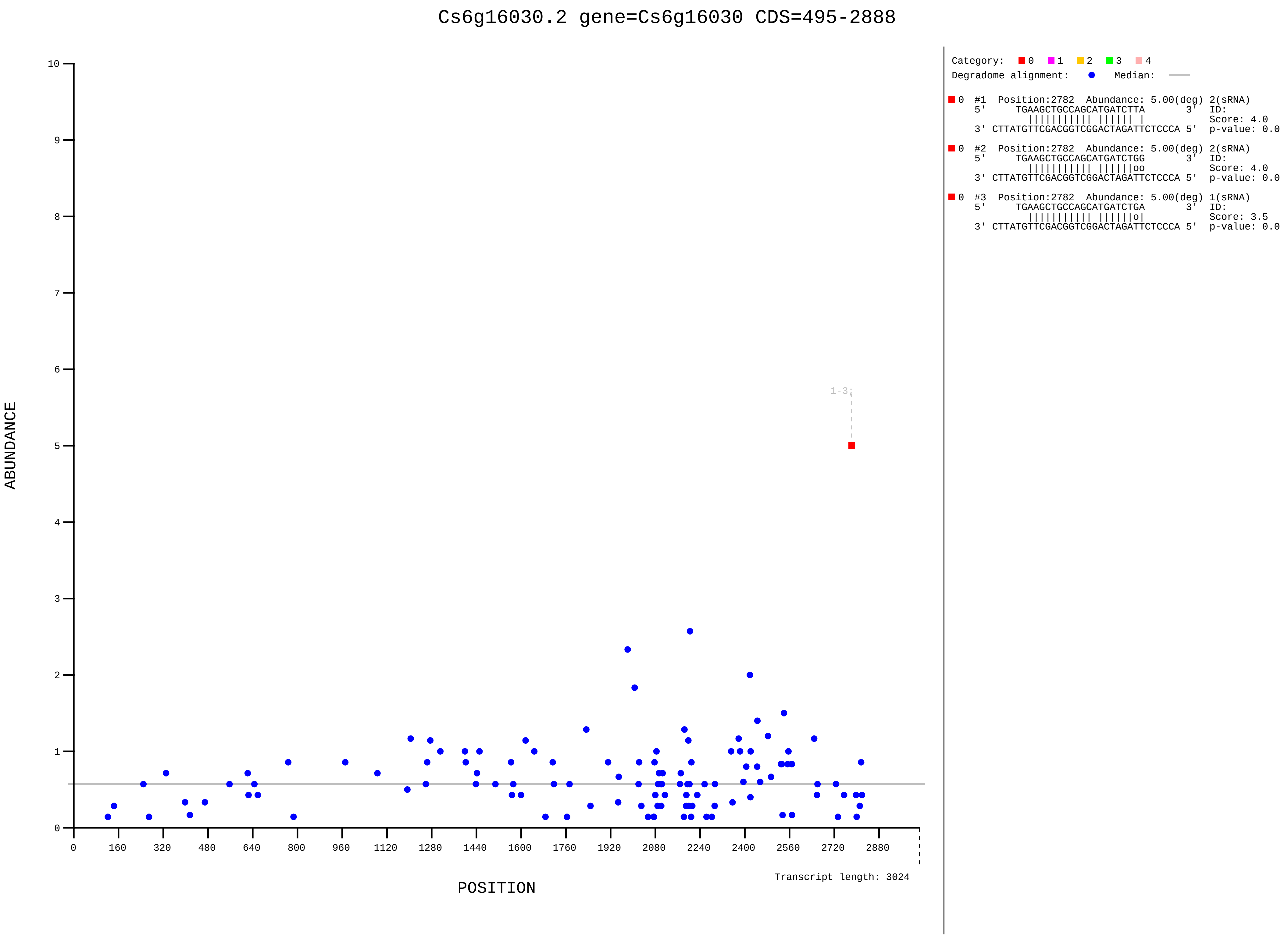

Cs6g16030.2 gene=Cs6g16030 CDS=495-2888
ABUNDANCE
POSITION
Transcript length: 3024
1-3
Category: 0 1 2 3 4
Degradome alignment: Median:
0 #1 Position:2782 Abundance: 5.00(deg) 2(sRNA)
5' TGAAGCTGCCAGCATGATCTTA 3' ID:
||||||||||| |||||| | Score: 4.0
3' CTTATGTTCGACGGTCGGACTAGATTCTCCCA 5' p-value: 0.0
0 #2 Position:2782 Abundance: 5.00(deg) 2(sRNA)
5' TGAAGCTGCCAGCATGATCTGG 3' ID:
||||||||||| ||||||oo Score: 4.0
3' CTTATGTTCGACGGTCGGACTAGATTCTCCCA 5' p-value: 0.0
0 #3 Position:2782 Abundance: 5.00(deg) 1(sRNA)
5' TGAAGCTGCCAGCATGATCTGA 3' ID:
||||||||||| ||||||o| Score: 3.5
3' CTTATGTTCGACGGTCGGACTAGATTCTCCCA 5' p-value: 0.0

Cs9g08500.2 gene=Cs9g08500 CDS=1-3252

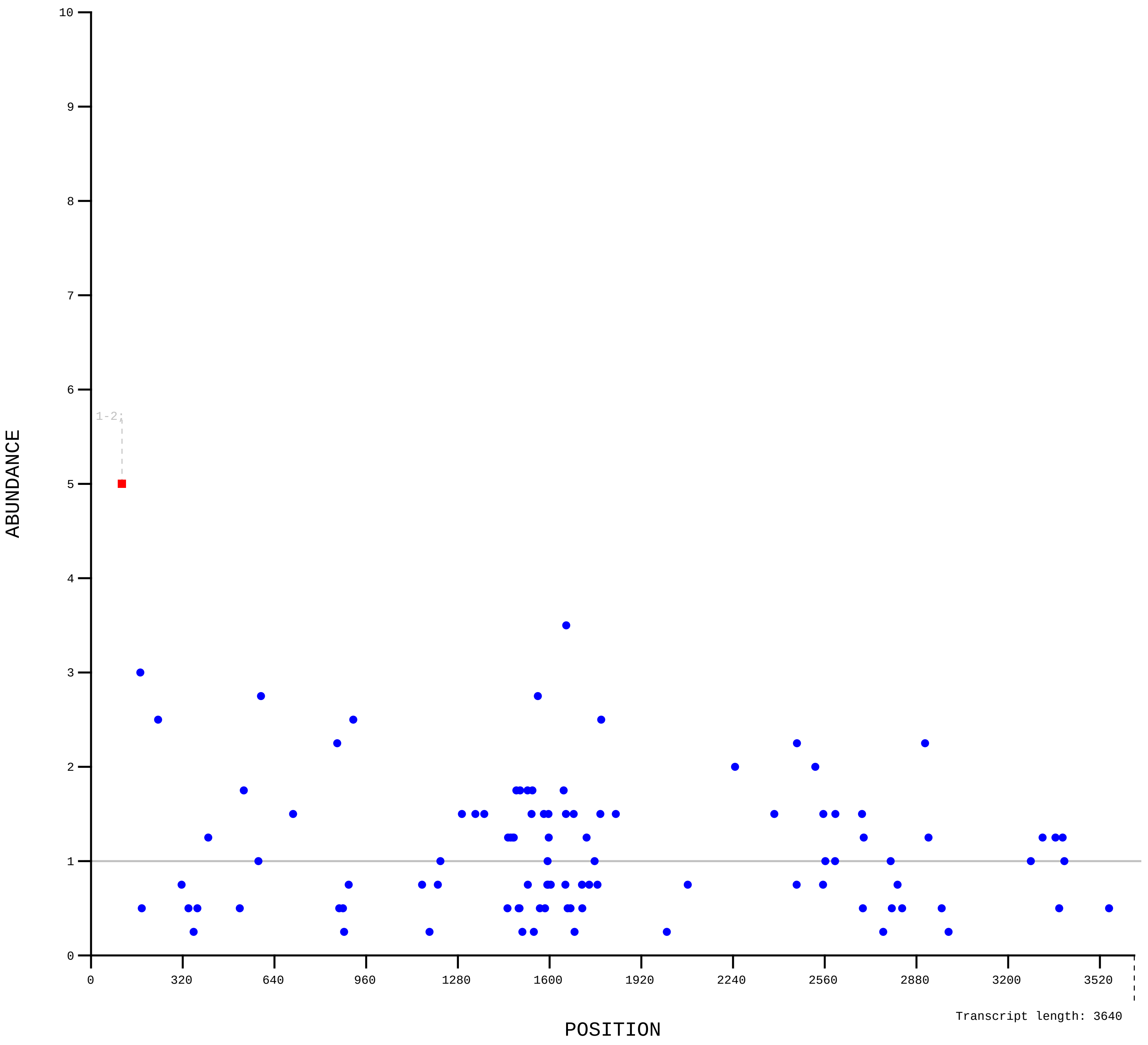

Category: ■0 ■1 ■2 ■3 ■4
Degradome alignment: ● Median: —

```
■0  #1  Position:108  Abundance: 5.00(deg) 1(sRNA)
    5'     TGCATTTGCACCTGCACCTTG        3'  ID:
           |||||||||||||||||| |             Score: 1.0
    3' TCGGACGTAAACGTGGACGTGGA-CTTAGTGA 5'  p-value: 0.0

■0  #2  Position:108  Abundance: 5.00(deg) 1(sRNA)
    5'     TGCATTTGCACCTGCATCTTG        3'  ID:
           ||||||||||||||||o|| |            Score: 1.5
    3' TCGGACGTAAACGTGGACGTGGA-CTTAGTGA 5'  p-value: 0.0
```

Cs9g08500.1 gene=Cs9g08500 CDS=1677-4937

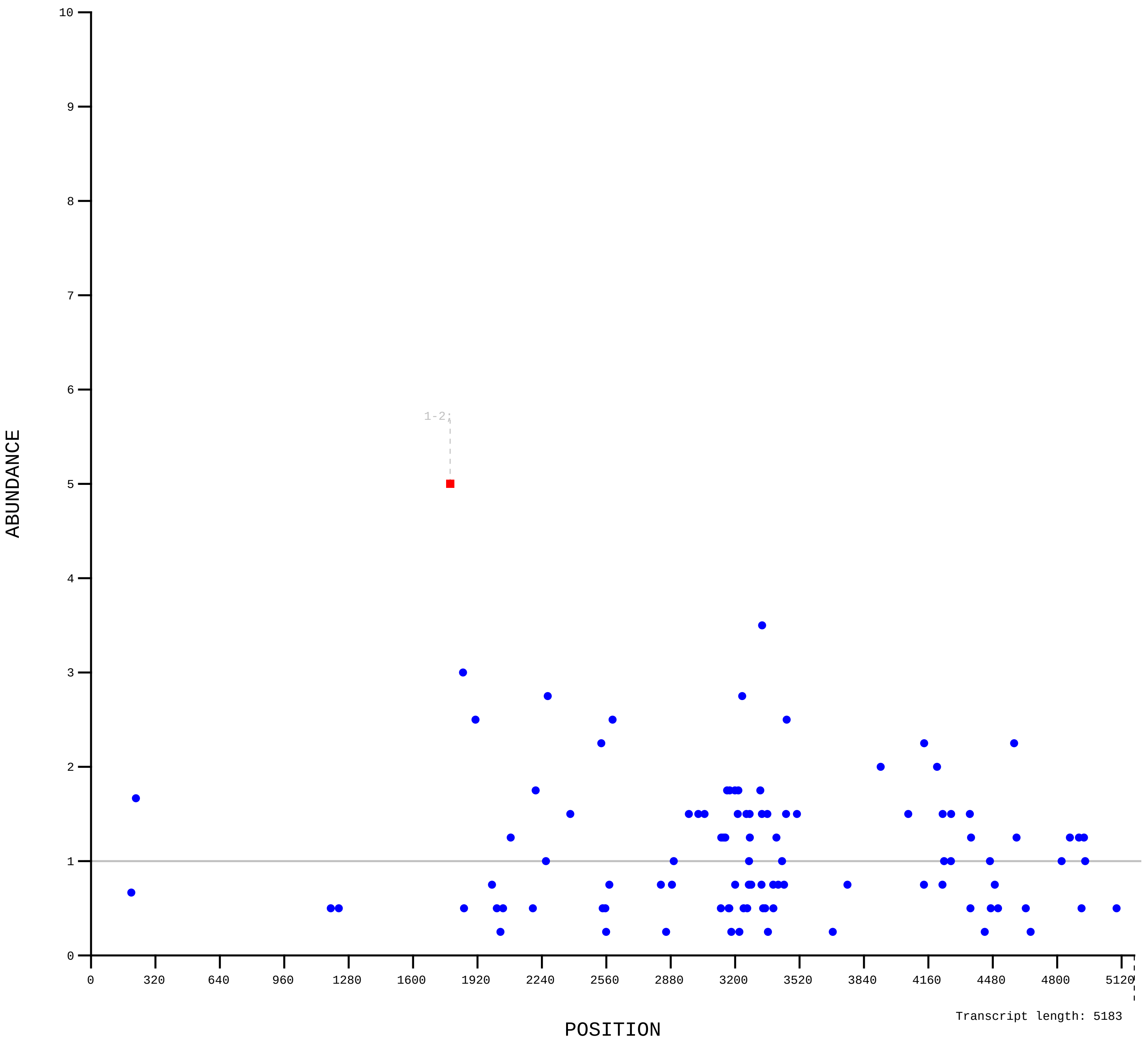


```
Category:  ■0  ■1  ■2  ■3  ■4
Degradome alignment:  ●   Median: ——

■0  #1  Position:1784  Abundance: 5.00(deg) 1(sRNA)
    5'     TGCATTTGCACCTGCACCTTG        3'  ID:
           |||||||||||||||||| |             Score: 1.0
    3' TCGGACGTAAACGTGGACGTGGA-CTTAGTGA 5'  p-value: 0.0

■0  #2  Position:1784  Abundance: 5.00(deg) 1(sRNA)
    5'     TGCATTTGCACCTGCATCTTG        3'  ID:
           |||||||||||||||o|| |             Score: 1.5
    3' TCGGACGTAAACGTGGACGTGGA-CTTAGTGA 5'  p-value: 0.0
```

Cs7g11770.2 gene=Cs7g11770 CDS=381-1778

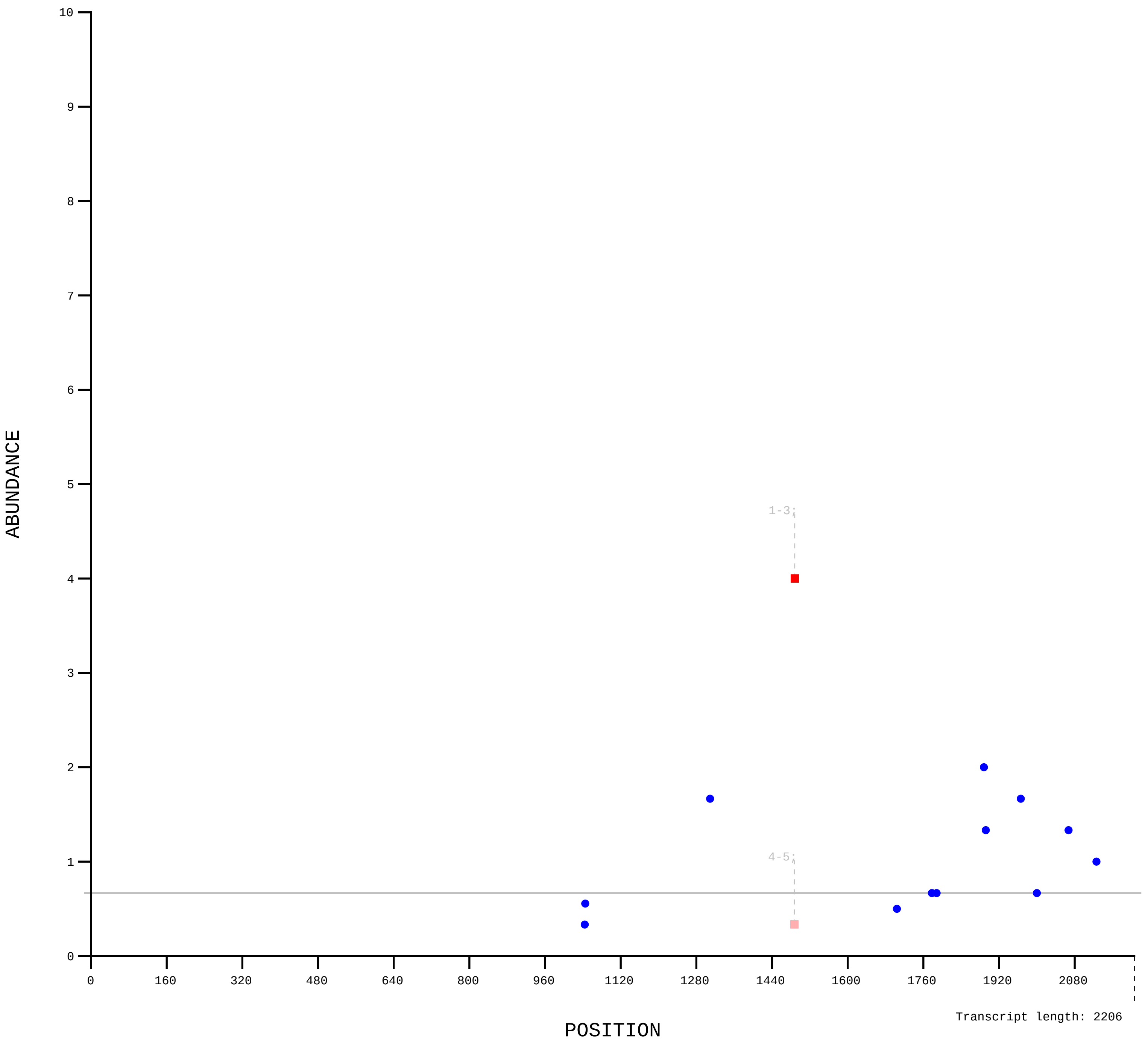


```
Category: 0 1 2 3 4
Degradome alignment:      Median:

0  #1  Position:1488  Abundance: 4.00(deg) 1(sRNA)
   5'     CTGACAGAAGAGAGTGAGCAC        3'  ID:
          |||||||||||||| ||||||            Score: 1.0
   3' ACTCGACTGTCTTCTCTCTCTCGTGGTGACCT 5'  p-value: 0.0

0  #2  Position:1488  Abundance: 4.00(deg) 1(sRNA)
   5'     TTGACAGAAGAGAGTGAGCAC        3'  ID:
          o||||||||||||| ||||||            Score: 1.5
   3' ACTCGACTGTCTTCTCTCTCTCGTGGTGACCT 5'  p-value: 0.0

0  #3  Position:1488  Abundance: 4.00(deg) 1(sRNA)
   5'     GTGACAGAAGATAGAGAGCGC        3'  ID:
           |||||||||| |||||||o|            Score: 2.5
   3' ACTCGACTGTCTTCTCTCTCTCGTGGTGACCT 5'  p-value: 0.0

4  #4  Position:1487  Abundance: 0.33(deg) 1(sRNA)
   5'     TGACAGAAGAGAGTGAGCAC         3'  ID:
          ||||||||||||| ||||||             Score: 1.0
   3' CTCGACTGTCTTCTCTCTCTCGTGGTGACCTC 5'  p-value: 0.0

4  #5  Position:1487  Abundance: 0.33(deg) 1(sRNA)
   5'     TGACAGAAGATAGAGAGCGC         3'  ID:
          |||||||||| |||||||o|             Score: 1.5
   3' CTCGACTGTCTTCTCTCTCTCGTGGTGACCTC 5'  p-value: 0.0
```

Cs7g11770.1 gene=Cs7g11770 CDS=381-1607

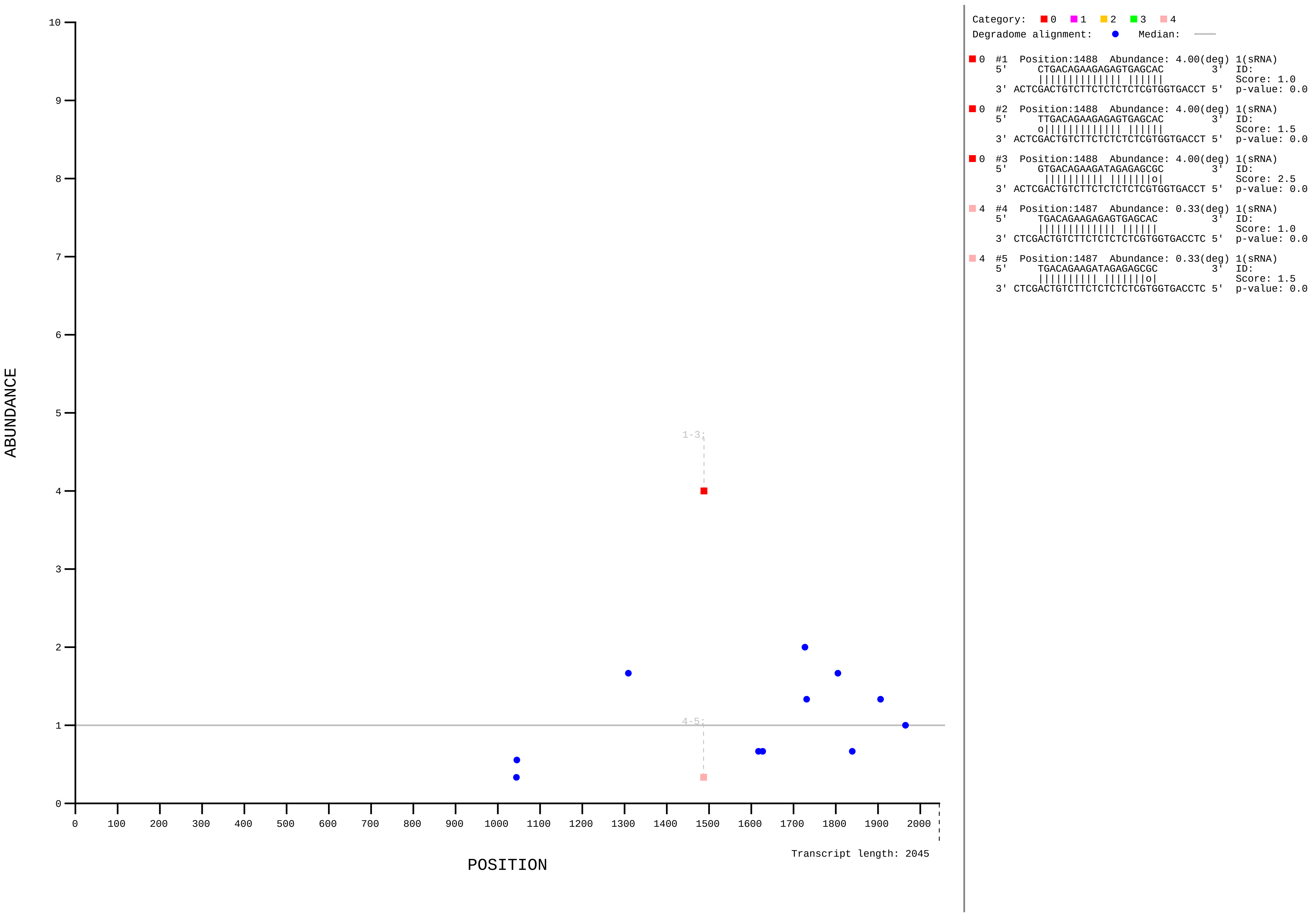

Cs5g17280.1 gene=Cs5g17280 CDS=156-2693

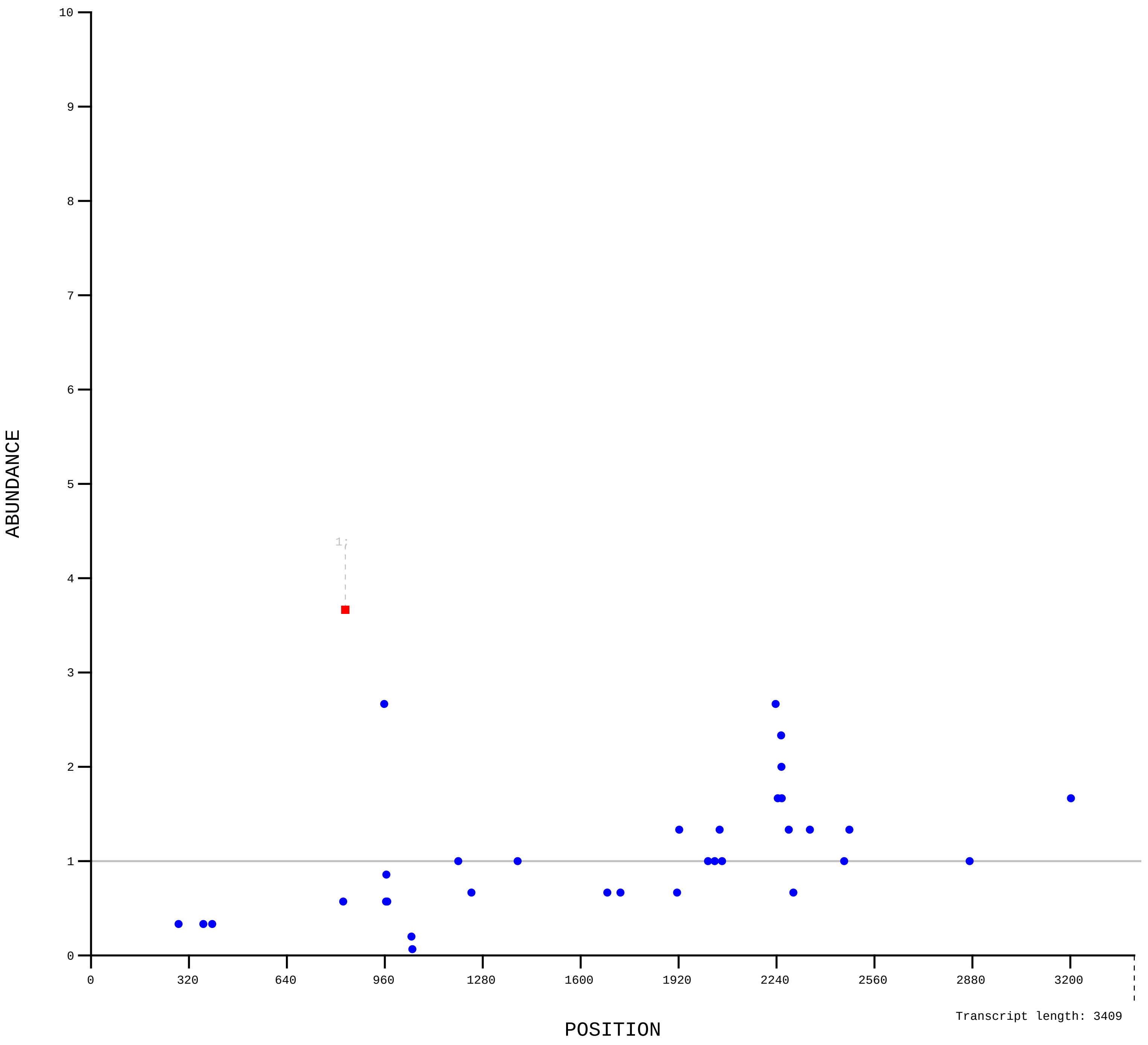

Category: 0 1 2 3 4
Degradome alignment: Median:

0 #1 Position:831 Abundance: 3.67(deg) 1(sRNA)
```
5'     TCTTCCCTATGCCTCCCATTCC       3'  ID:
       |||| |||||o|| || || ||           Score: 4.5
3' CAACAGAACGGATATGGTGGTTACGGGGTTTA 5'  p-value: 0.01
```

Cs5g17280.3 gene=Cs5g17280 CDS=156-2138

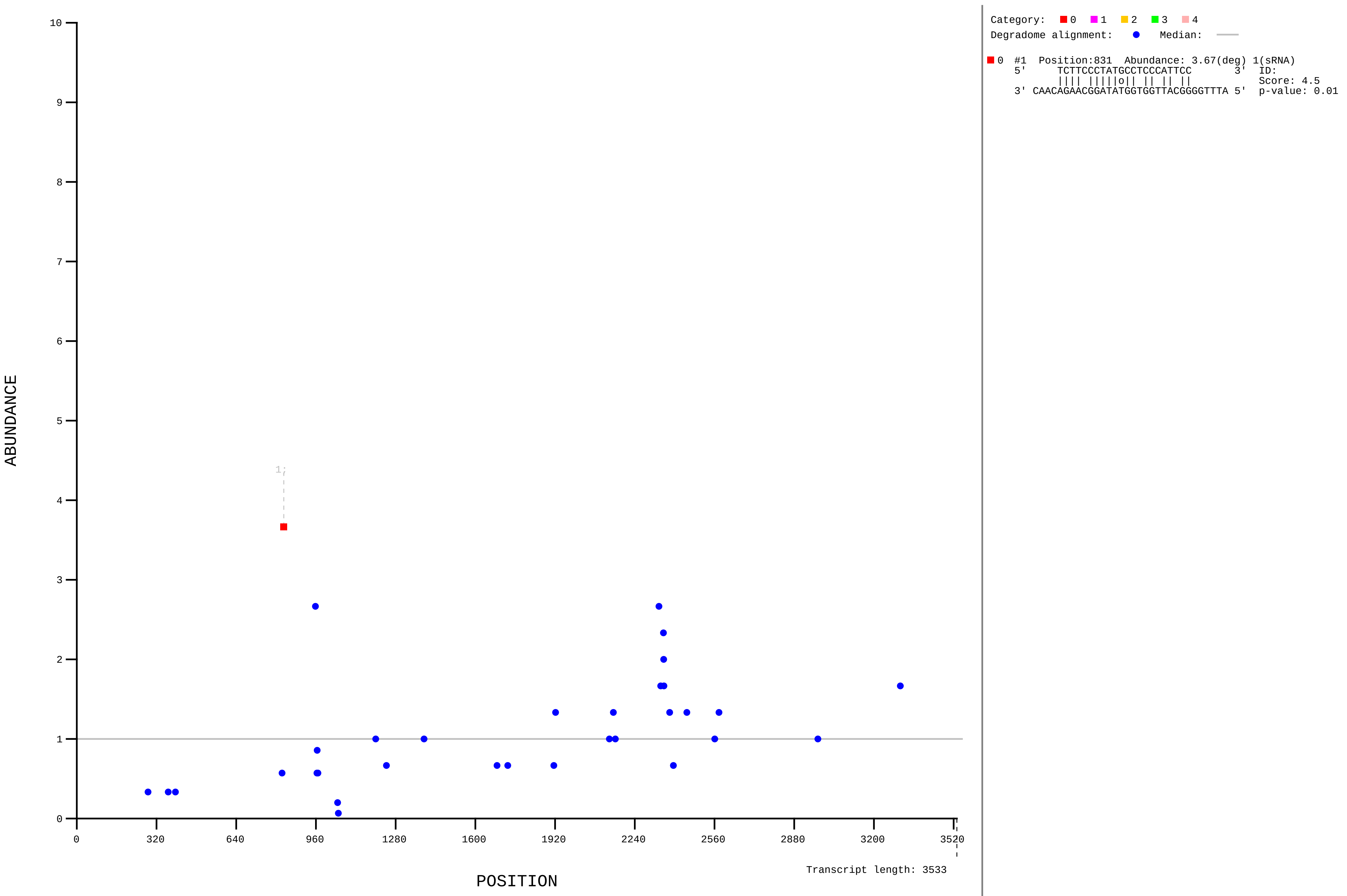

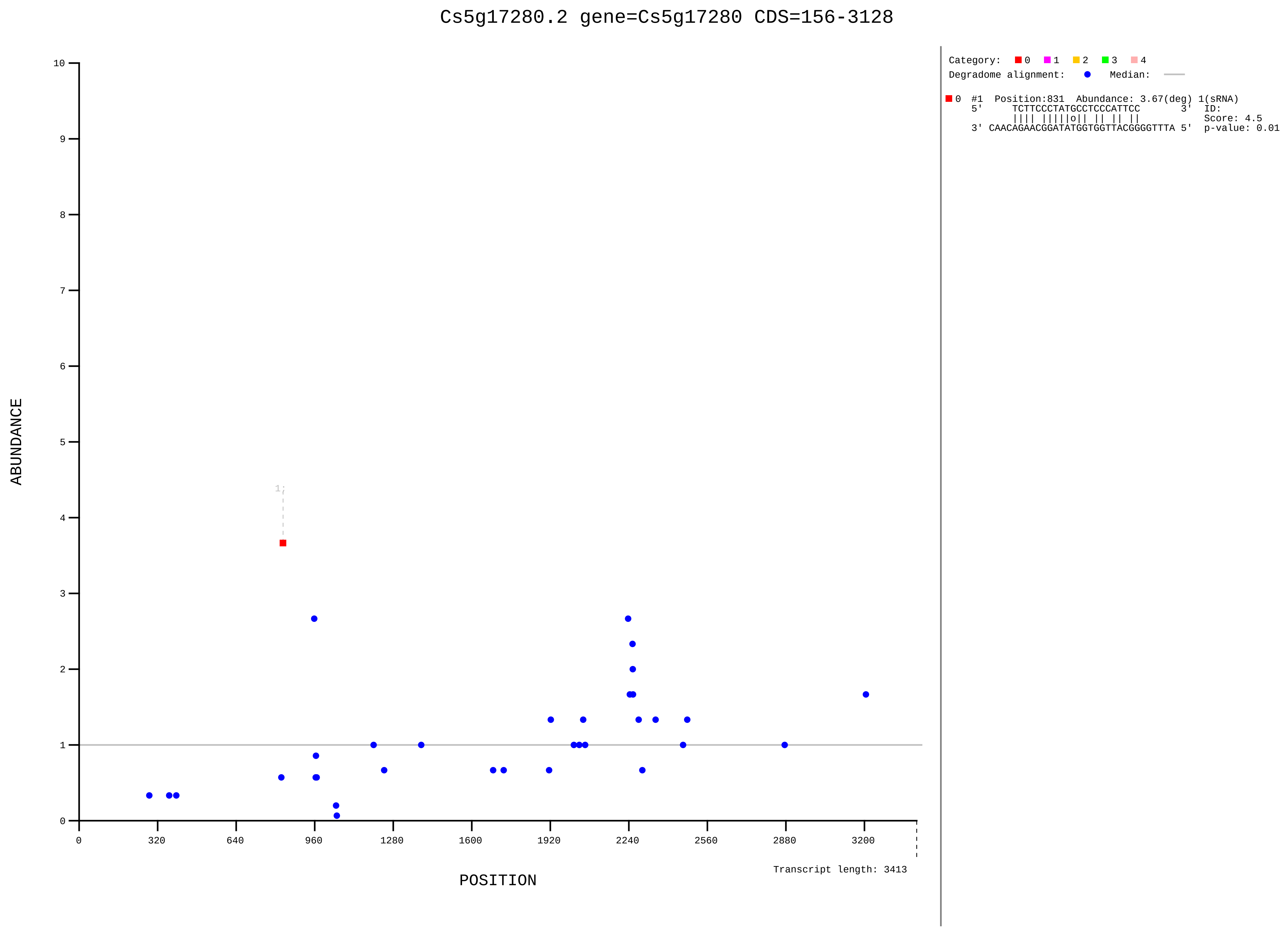
Cs5g17280.2 gene=Cs5g17280 CDS=156-3128
Category: 0 1 2 3 4
Degradome alignment: Median:
0 #1 Position:831 Abundance: 3.67(deg) 1(sRNA)
5' TCTTCCCTATGCCTCCCATTCC 3' ID:
|||| |||||o|| || || ||
3' CAACAGAACGGATATGGTGGTTACGGGGTTTA 5'
Score: 4.5
p-value: 0.01
ABUNDANCE
POSITION
Transcript length: 3413

Cs8g18140.2 gene=Cs8g18140 CDS=186-1217

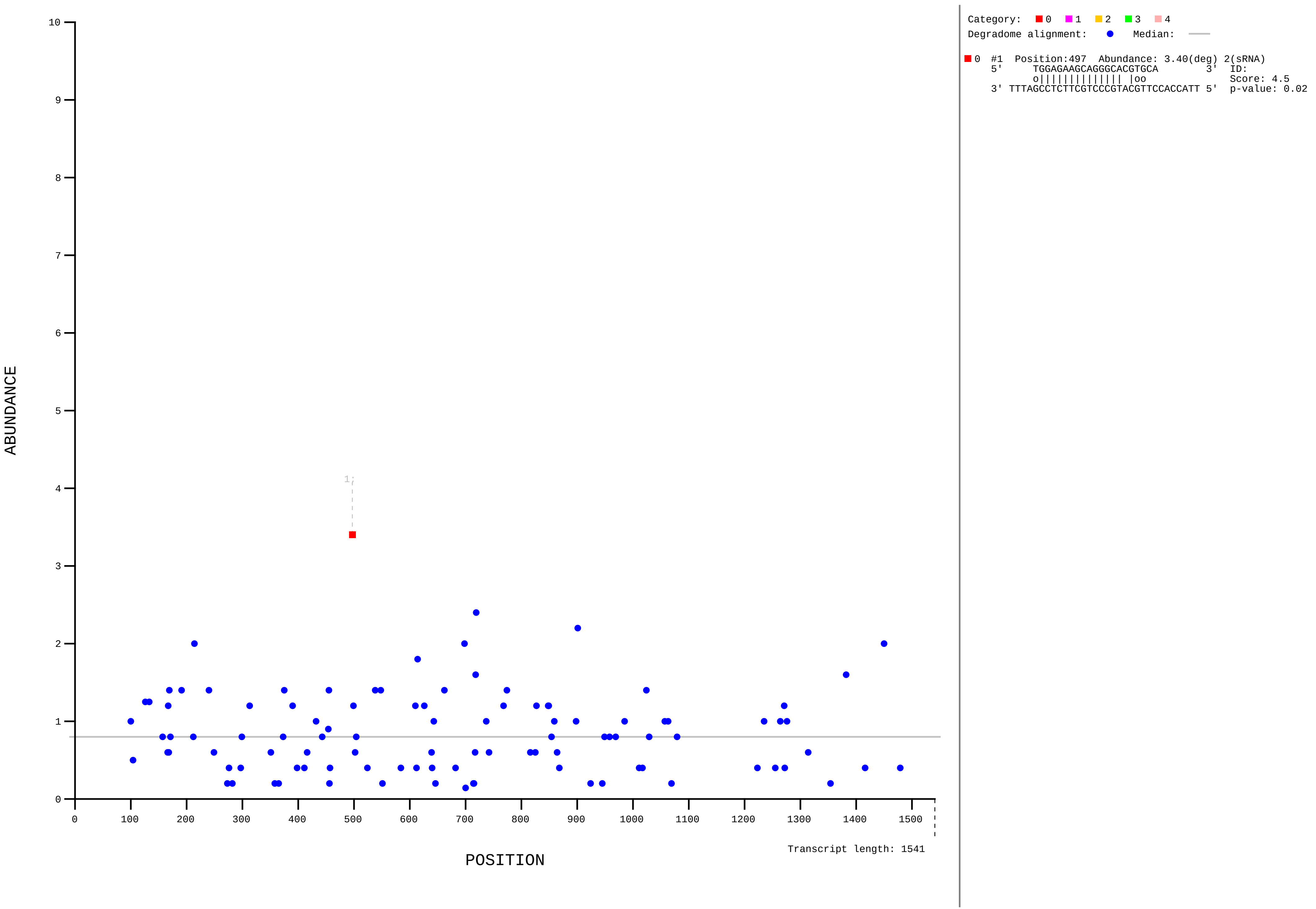

Cs8g18140.3 gene=Cs8g18140 CDS=197-1228

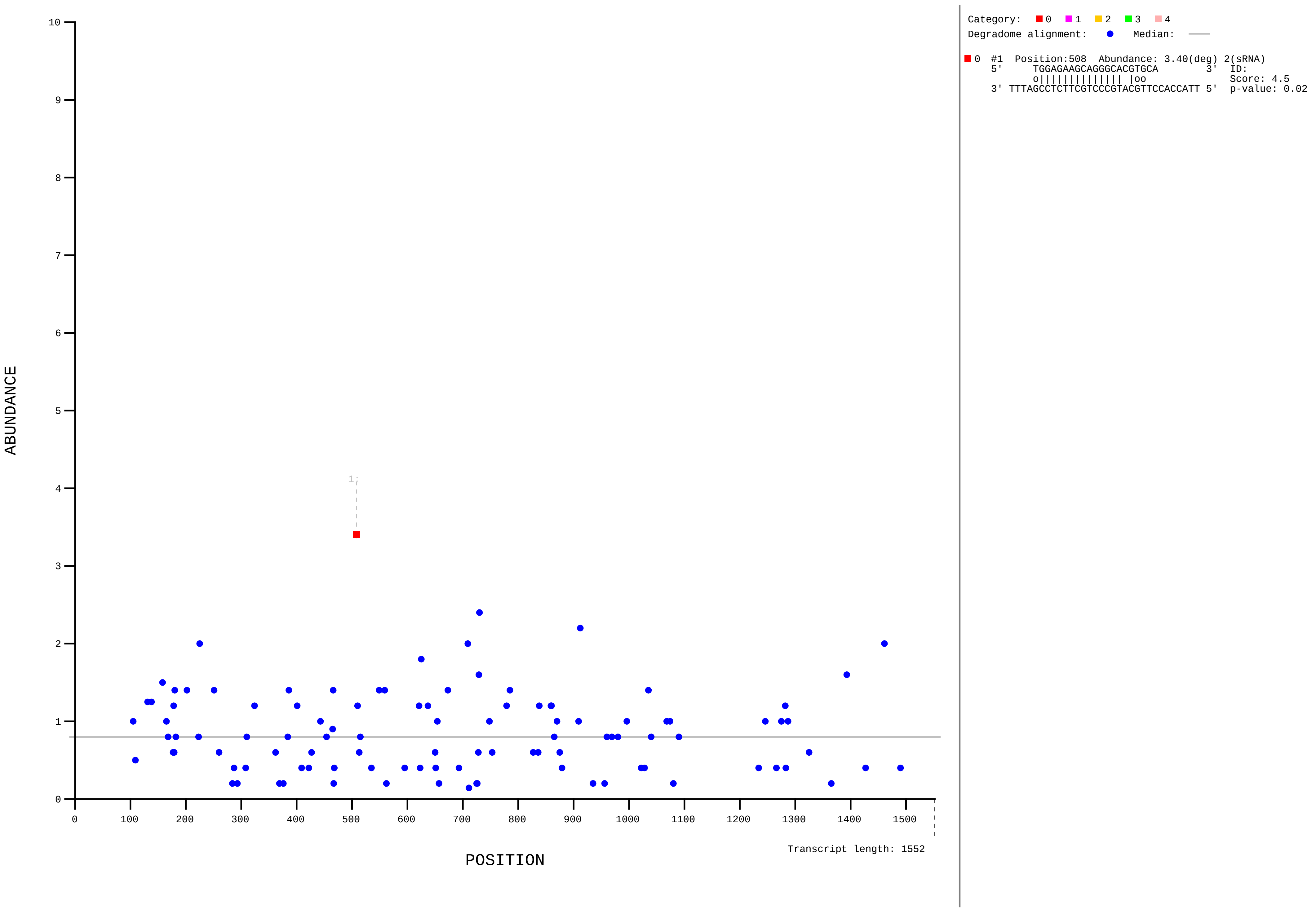

Cs8g18140.5 gene=Cs8g18140 CDS=384-1415

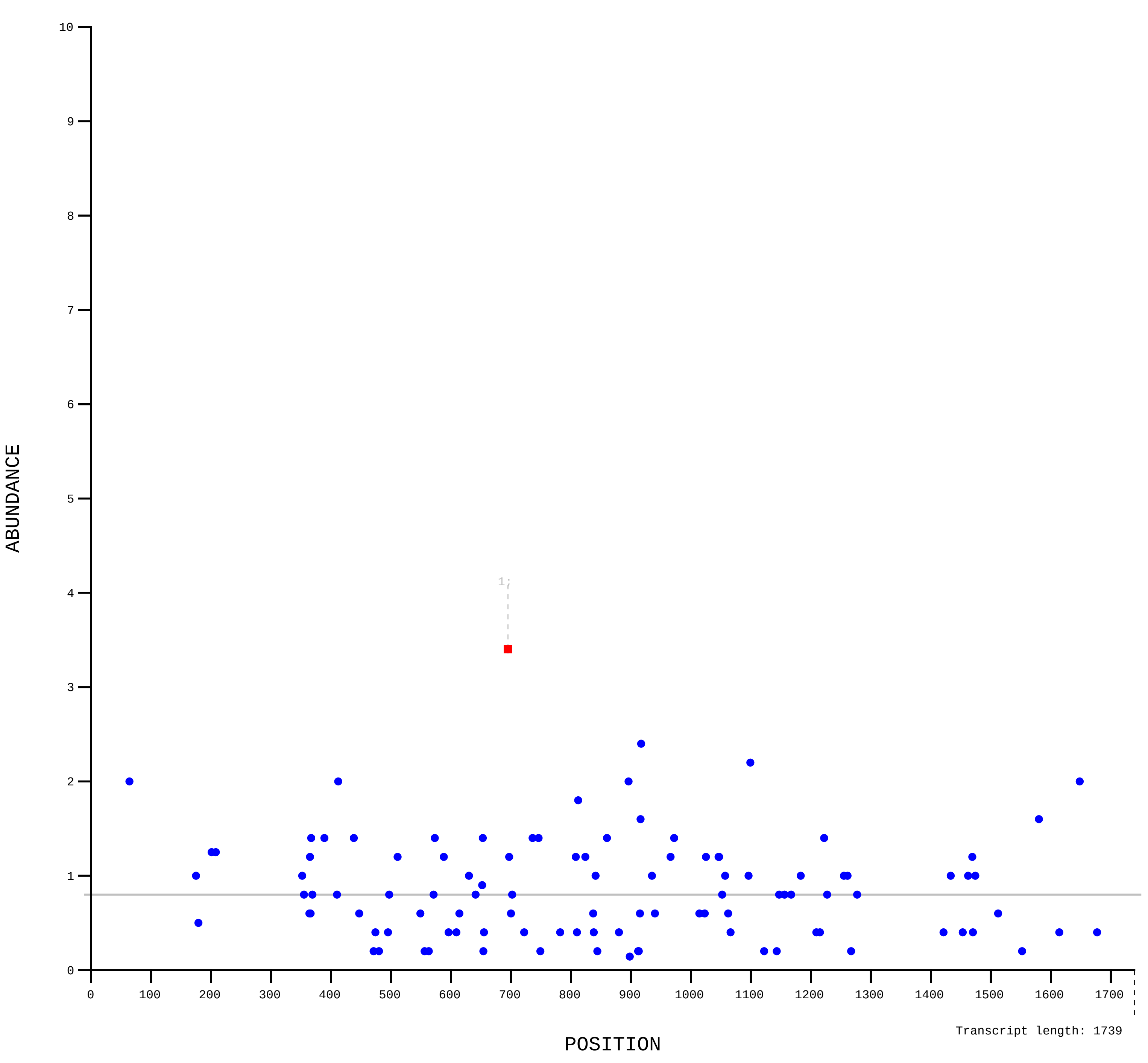

Category: 0 1 2 3 4
Degradome alignment: Median:

```
0  #1  Position:695  Abundance: 3.40(deg) 2(sRNA)
   5'     TGGAGAAGCAGGGCACGTGCA        3'  ID:
          o|||||||||||||| |oo              Score: 4.5
   3' TTTAGCCTCTTCGTCCCGTACGTTCCACCATT 5'  p-value: 0.01
```

Cs3g24010.3 gene=Cs3g24010 CDS=152-964

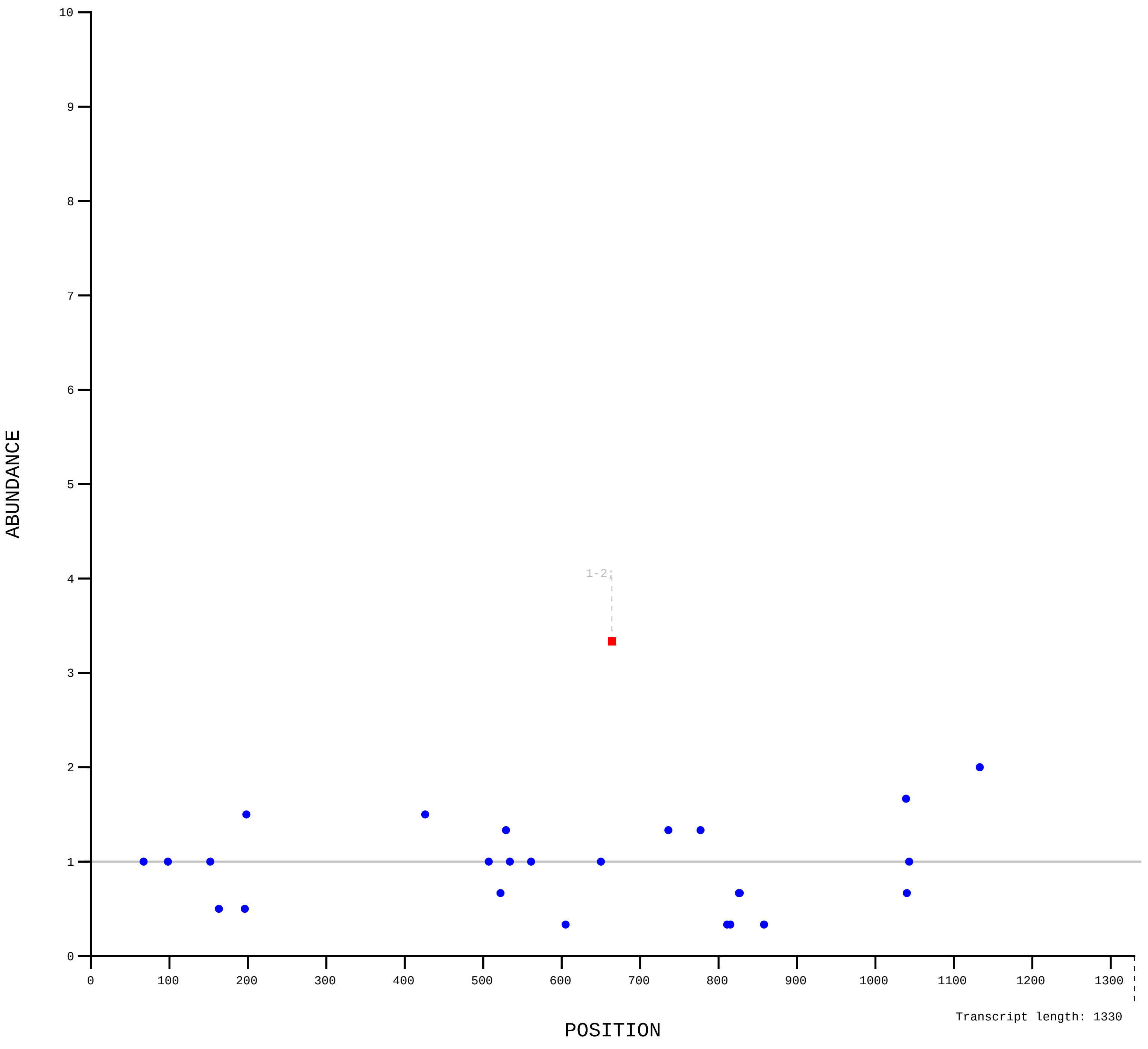


```
Category:  0  1  2  3  4
Degradome alignment:      Median:

0  #1  Position:664  Abundance: 3.33(deg) 3(sRNA)
   5'     TTCCACAGCTTTCTTGAACTG        3'  ID:
          |||||||| |||||||||               Score: 4.0
   3' AACTAAGGTGTCTAAAGAACTTCCGCTCAGGA 5'  p-value: 0.01

0  #2  Position:664  Abundance: 3.33(deg) 1(sRNA)
   5'     TTCCACGGCTTTCTTGAACTT        3'  ID:
          ||||||o| |||||||||  o            Score: 4.0
   3' AACTAAGGTGTCTAAAGAACTTCCGCTCAGGA 5'  p-value: 0.02
```

Cs7g10090.1 gene=Cs7g10090 CDS=1-189

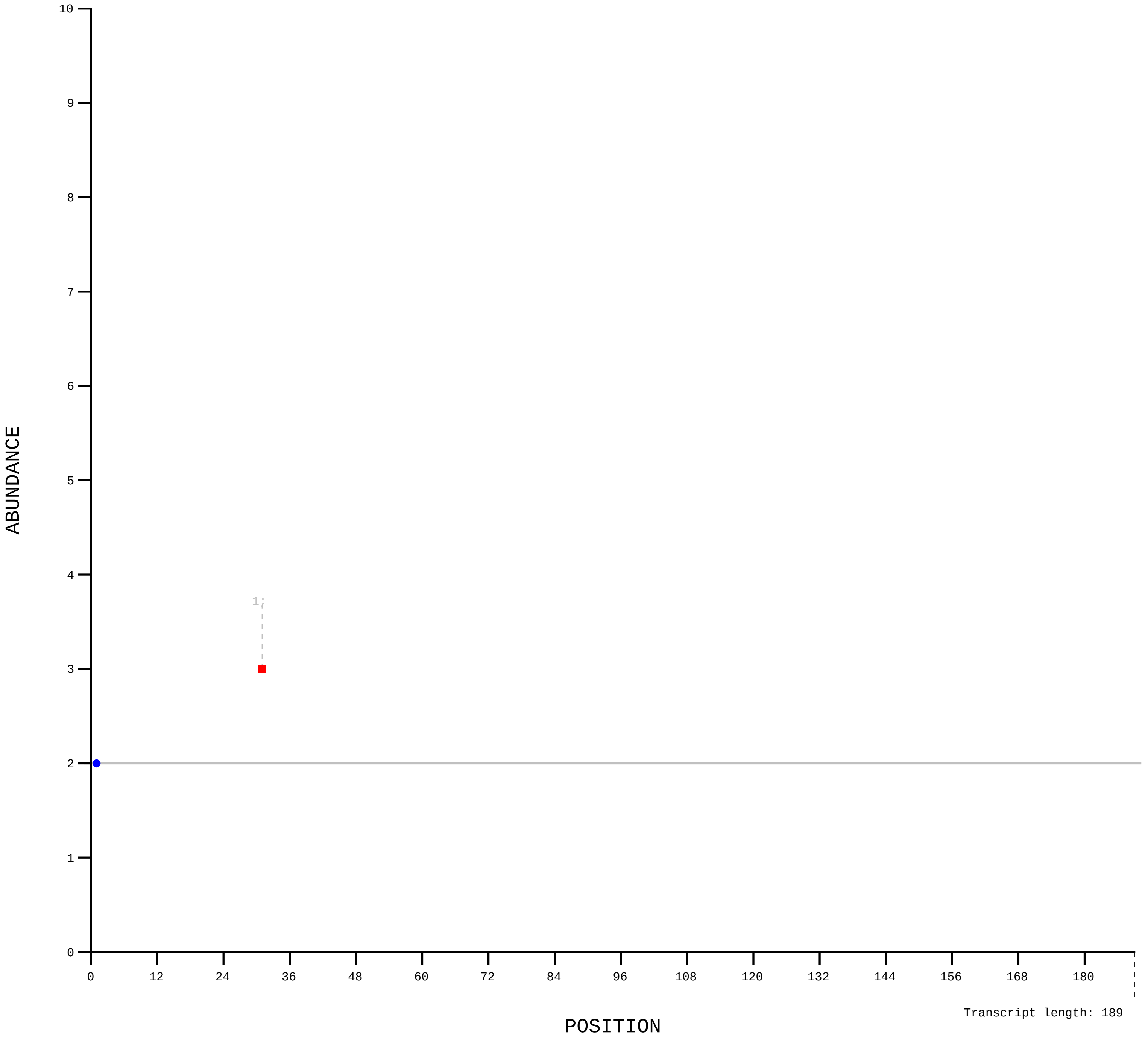

Category: 0 1 2 3 4
Degradome alignment: Median:

```
0  #1  Position:31  Abundance: 3.00(deg) 1(sRNA)
   5'      TCGCGTGACTGTCGATGATGG         3'  ID:
           |||||||| ||| | |||o||              Score: 3.5
   3' ACGAAGCGCACTCACAACCACTGCCTCGTTTC 5'  p-value: 0.0
```

Cs5g22030.6 gene=Cs5g22030 CDS=36-4064

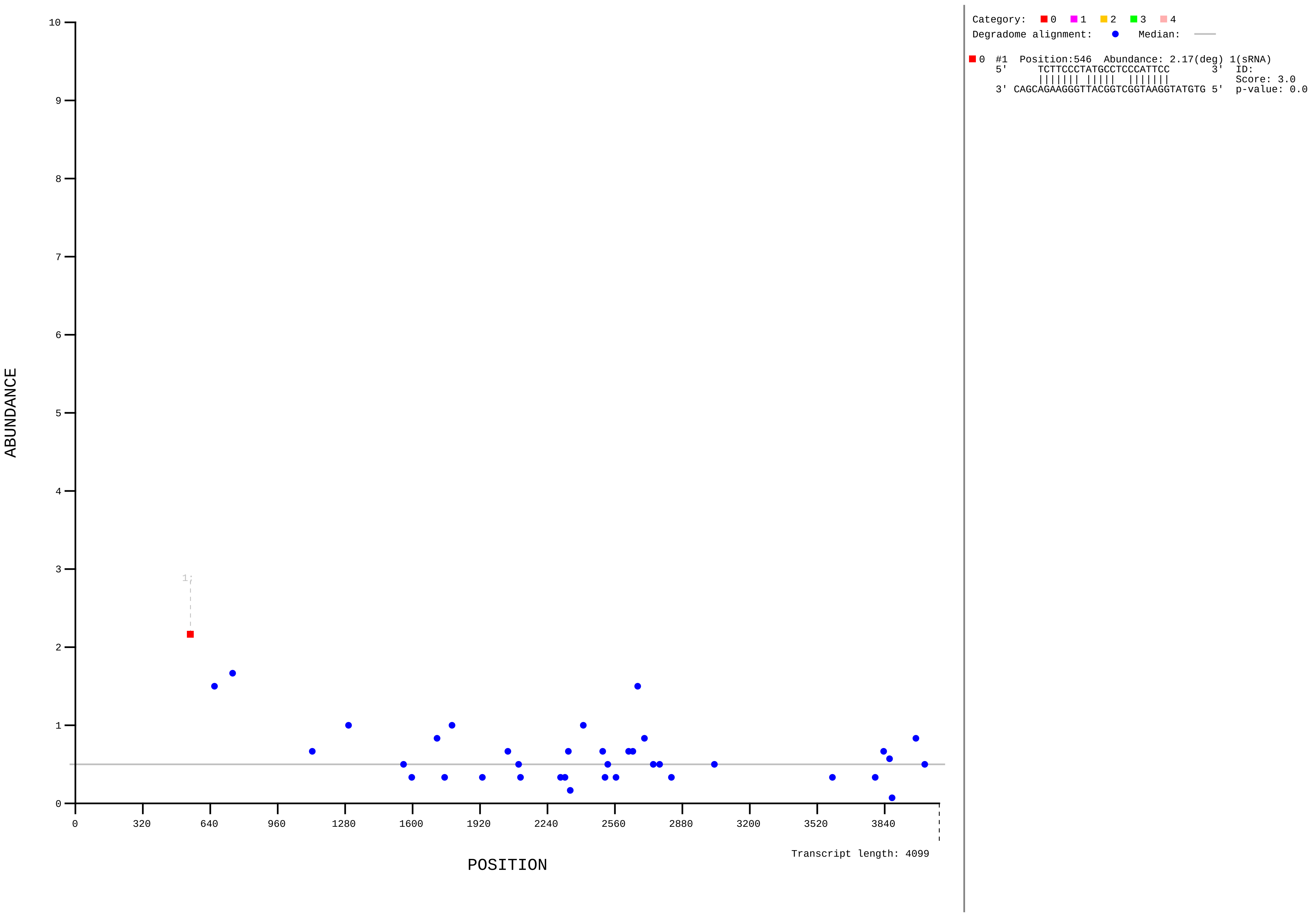

Cs7g10830.4 gene=Cs7g10830 CDS=187-1629

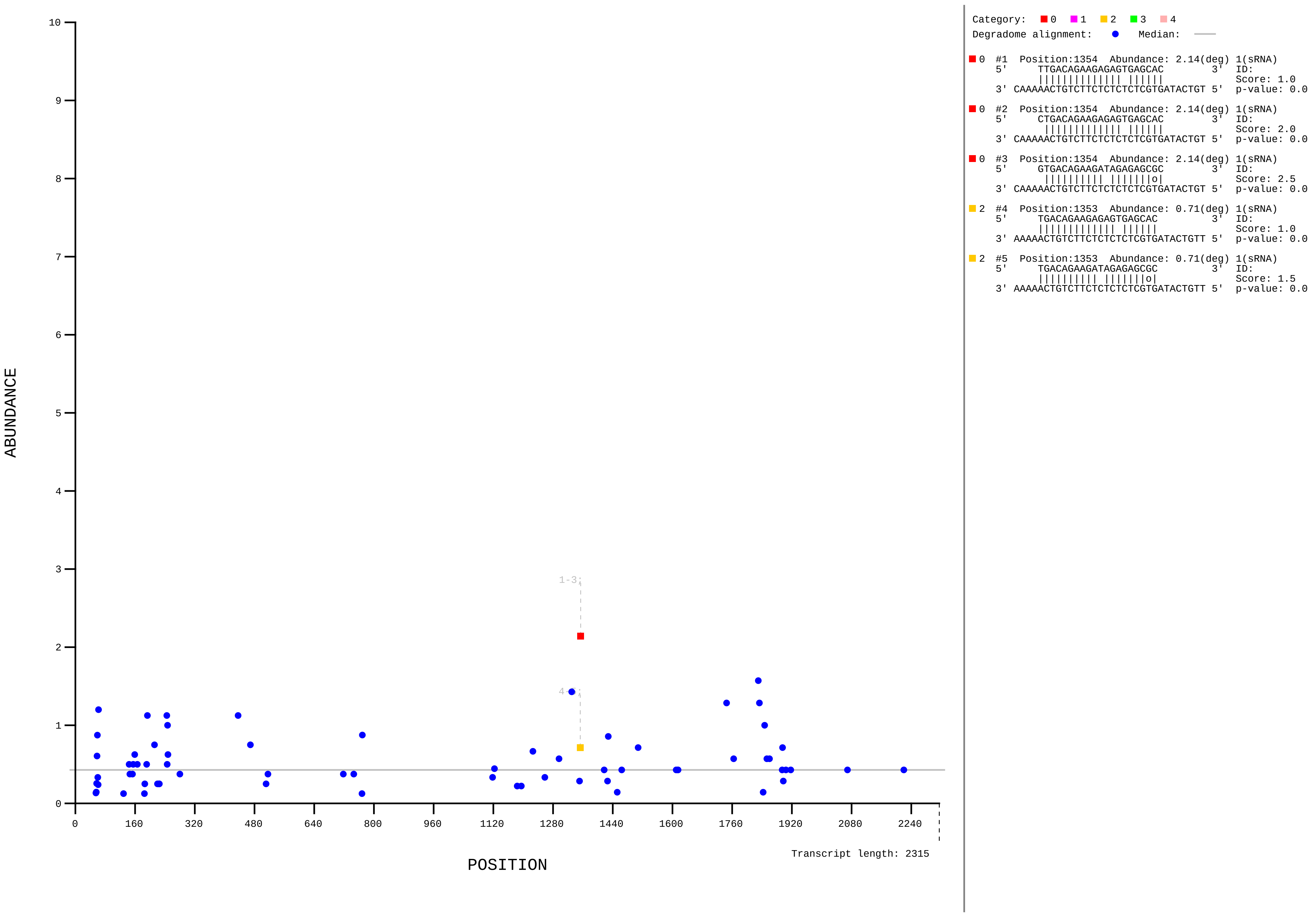

Cs7g10830.1 gene=Cs7g10830 CDS=109-1458

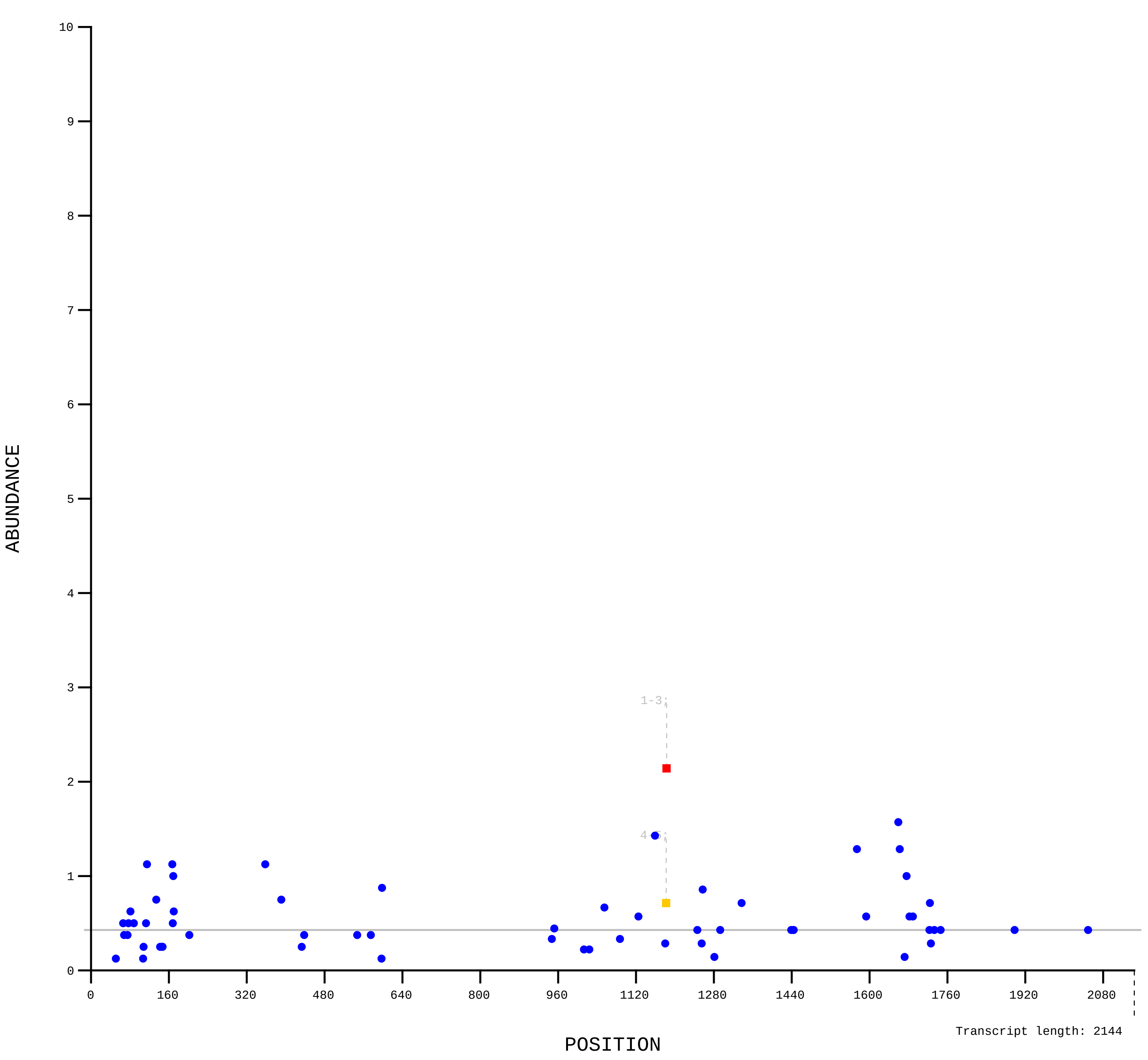

Category: 0 1 2 3 4
Degradome alignment: Median:

```
0  #1  Position:1183  Abundance: 2.14(deg) 1(sRNA)
   5'     TTGACAGAAGAGAGTGAGCAC        3'  ID:
          |||||||||||||| ||||||            Score: 1.0
   3' CAAAAACTGTCTTCTCTCTCTCGTGATACTGT 5'  p-value: 0.0

0  #2  Position:1183  Abundance: 2.14(deg) 1(sRNA)
   5'     CTGACAGAAGAGAGTGAGCAC        3'  ID:
           ||||||||||||| ||||||            Score: 2.0
   3' CAAAAACTGTCTTCTCTCTCTCGTGATACTGT 5'  p-value: 0.0

0  #3  Position:1183  Abundance: 2.14(deg) 1(sRNA)
   5'     GTGACAGAAGATAGAGAGCGC        3'  ID:
           |||||||||| |||||||o|            Score: 2.5
   3' CAAAAACTGTCTTCTCTCTCTCGTGATACTGT 5'  p-value: 0.02

2  #4  Position:1182  Abundance: 0.71(deg) 1(sRNA)
   5'     TGACAGAAGAGAGTGAGCAC        3'  ID:
          ||||||||||||| ||||||            Score: 1.0
   3' AAAAACTGTCTTCTCTCTCTCGTGATACTGTT 5'  p-value: 0.0

2  #5  Position:1182  Abundance: 0.71(deg) 1(sRNA)
   5'     TGACAGAAGATAGAGAGCGC        3'  ID:
          |||||||||| |||||||o|            Score: 1.5
   3' AAAAACTGTCTTCTCTCTCTCGTGATACTGTT 5'  p-value: 0.0
```

Cs7g10830.3 gene=Cs7g10830 CDS=100-1542

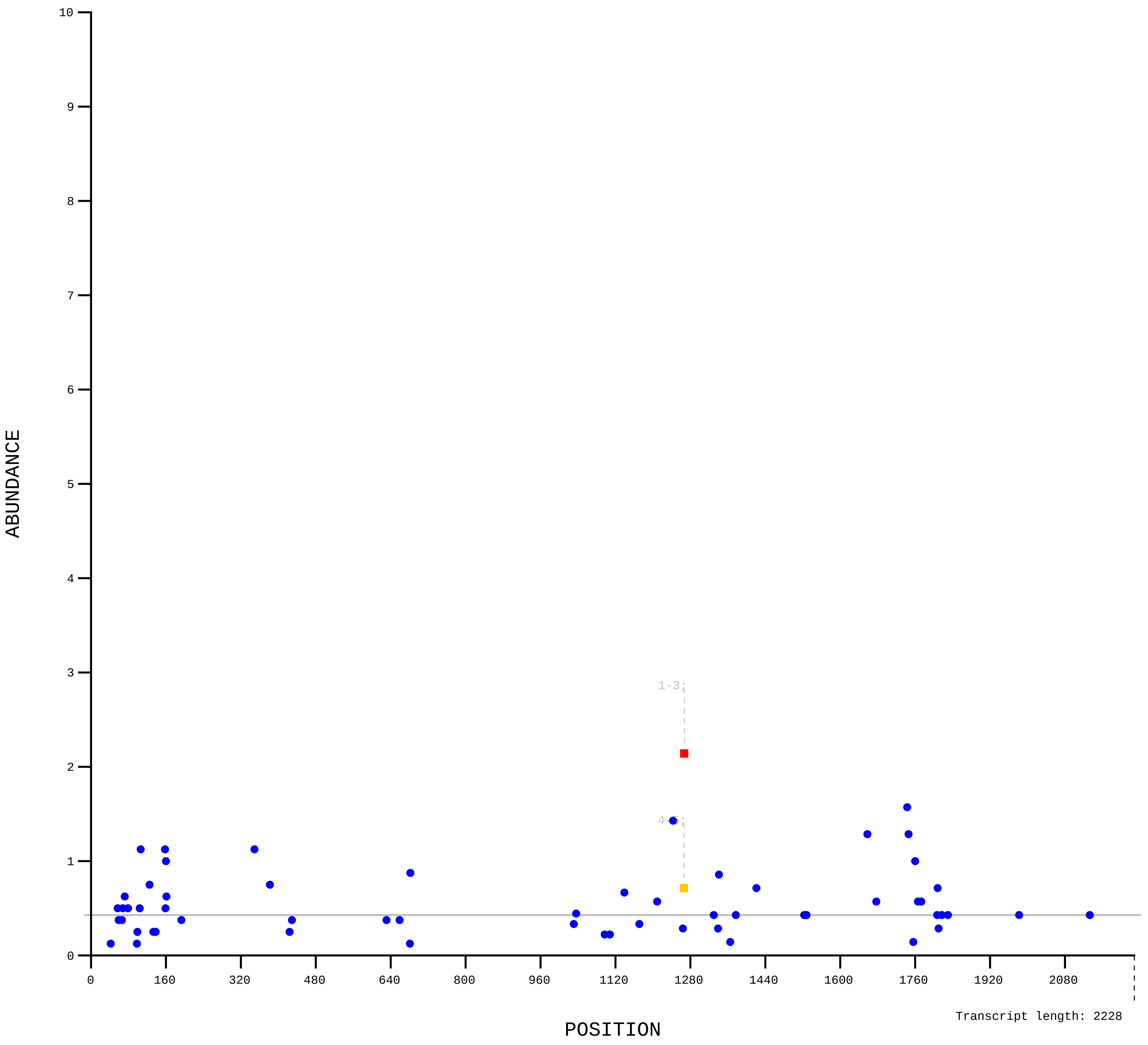


```
Category: ■0 ■1 ■2 ■3 ■4
Degradome alignment: ●   Median: ——

■0  #1  Position:1267  Abundance: 2.14(deg) 1(sRNA)
    5'     TTGACAGAAGAGAGTGAGCAC        3'  ID:
           |||||||||||||| ||||||            Score: 1.0
    3' CAAAAACTGTCTTCTCTCTCTCGTGATACTGT 5'  p-value: 0.0

■0  #2  Position:1267  Abundance: 2.14(deg) 1(sRNA)
    5'     CTGACAGAAGAGAGTGAGCAC        3'  ID:
            ||||||||||||| ||||||            Score: 2.0
    3' CAAAAACTGTCTTCTCTCTCTCGTGATACTGT 5'  p-value: 0.0

■0  #3  Position:1267  Abundance: 2.14(deg) 1(sRNA)
    5'     GTGACAGAAGATAGAGAGCGC        3'  ID:
            |||||||||| |||||||o|            Score: 2.5
    3' CAAAAACTGTCTTCTCTCTCTCGTGATACTGT 5'  p-value: 0.0

■2  #4  Position:1266  Abundance: 0.71(deg) 1(sRNA)
    5'     TGACAGAAGAGAGTGAGCAC         3'  ID:
           ||||||||||||| ||||||             Score: 1.0
    3' AAAAACTGTCTTCTCTCTCTCGTGATACTGTT 5'  p-value: 0.0

■2  #5  Position:1266  Abundance: 0.71(deg) 1(sRNA)
    5'     TGACAGAAGATAGAGAGCGC         3'  ID:
           |||||||||| |||||||o|             Score: 1.5
    3' AAAAACTGTCTTCTCTCTCTCGTGATACTGTT 5'  p-value: 0.0
```

Cs5g32890.2 gene=Cs5g32890 CDS=7-954

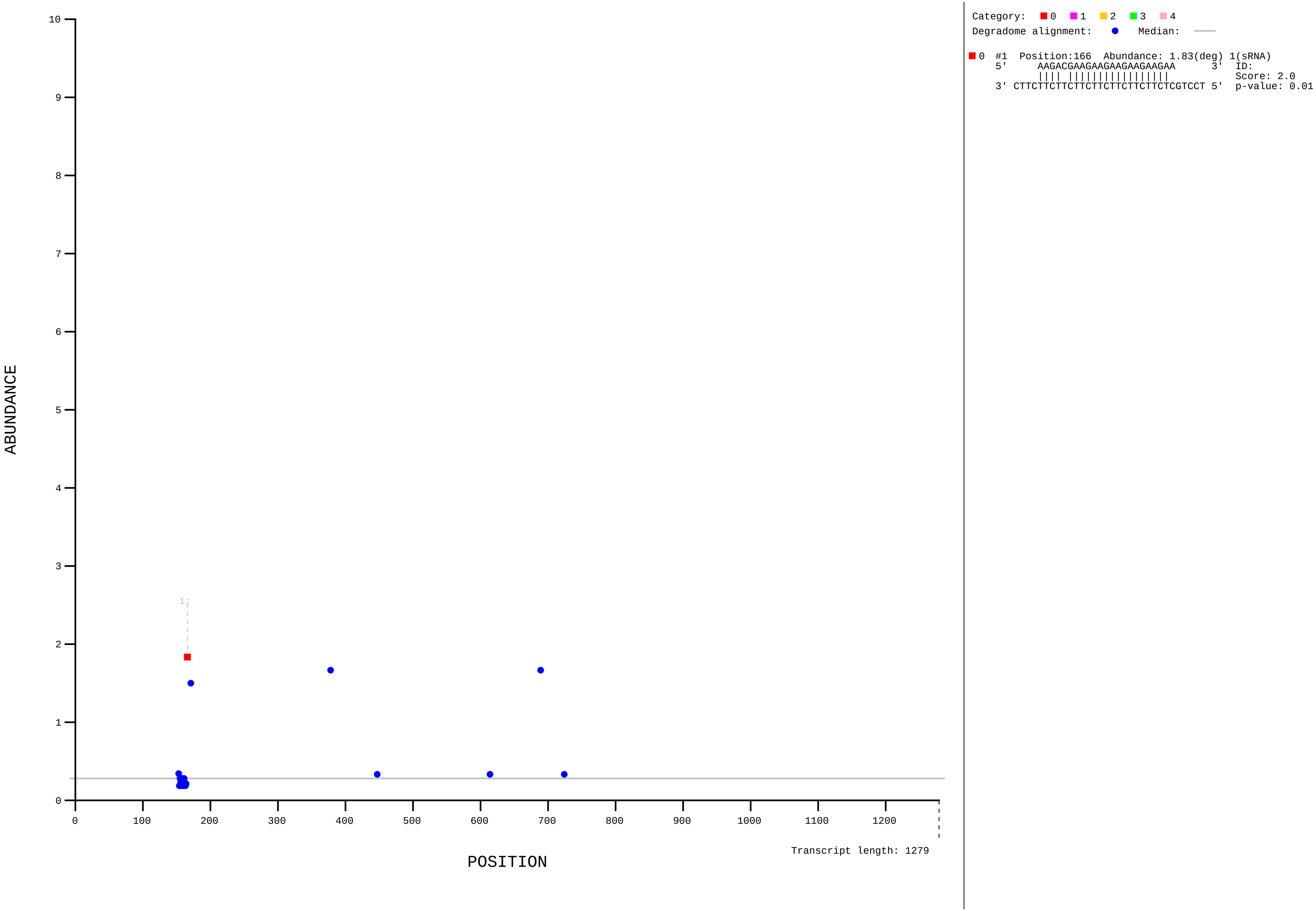

Cs5g19230.1 gene=Cs5g19230 CDS=49-4191

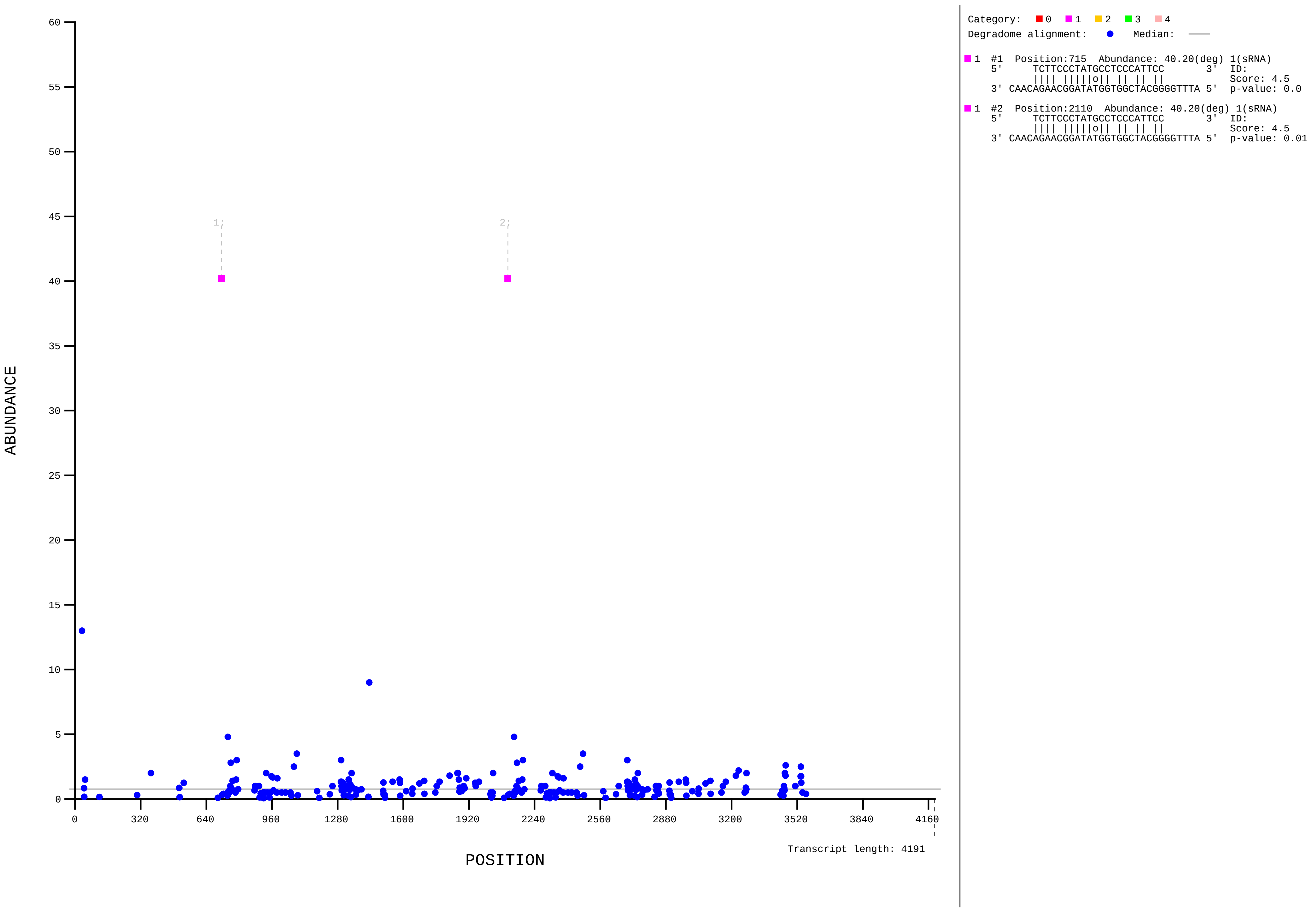

orange1.1t03701.1 gene=orange1.1t03701 CDS=307-1428

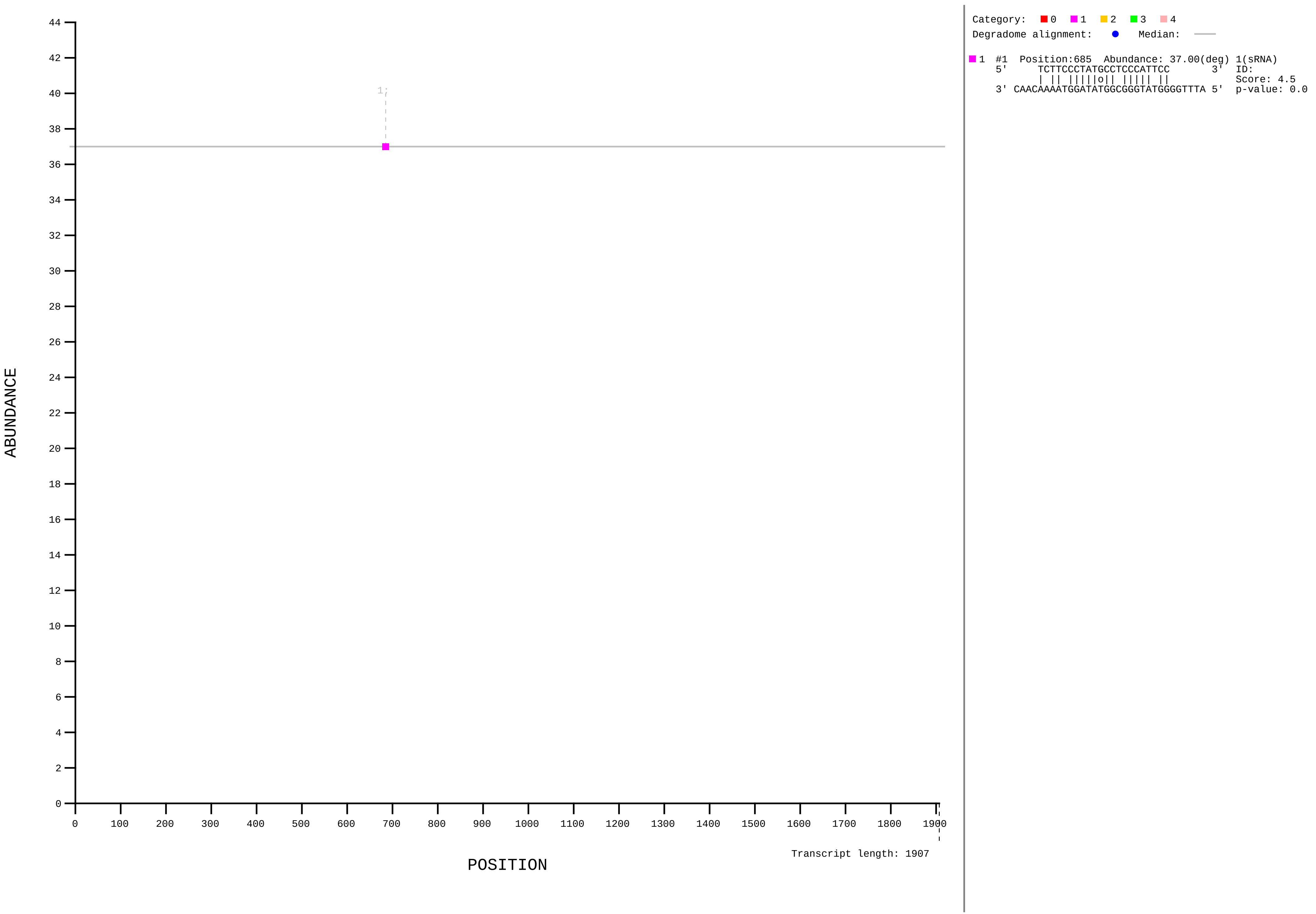

orange1.1t00227.1 gene=orange1.1t00227 CDS=546-2105

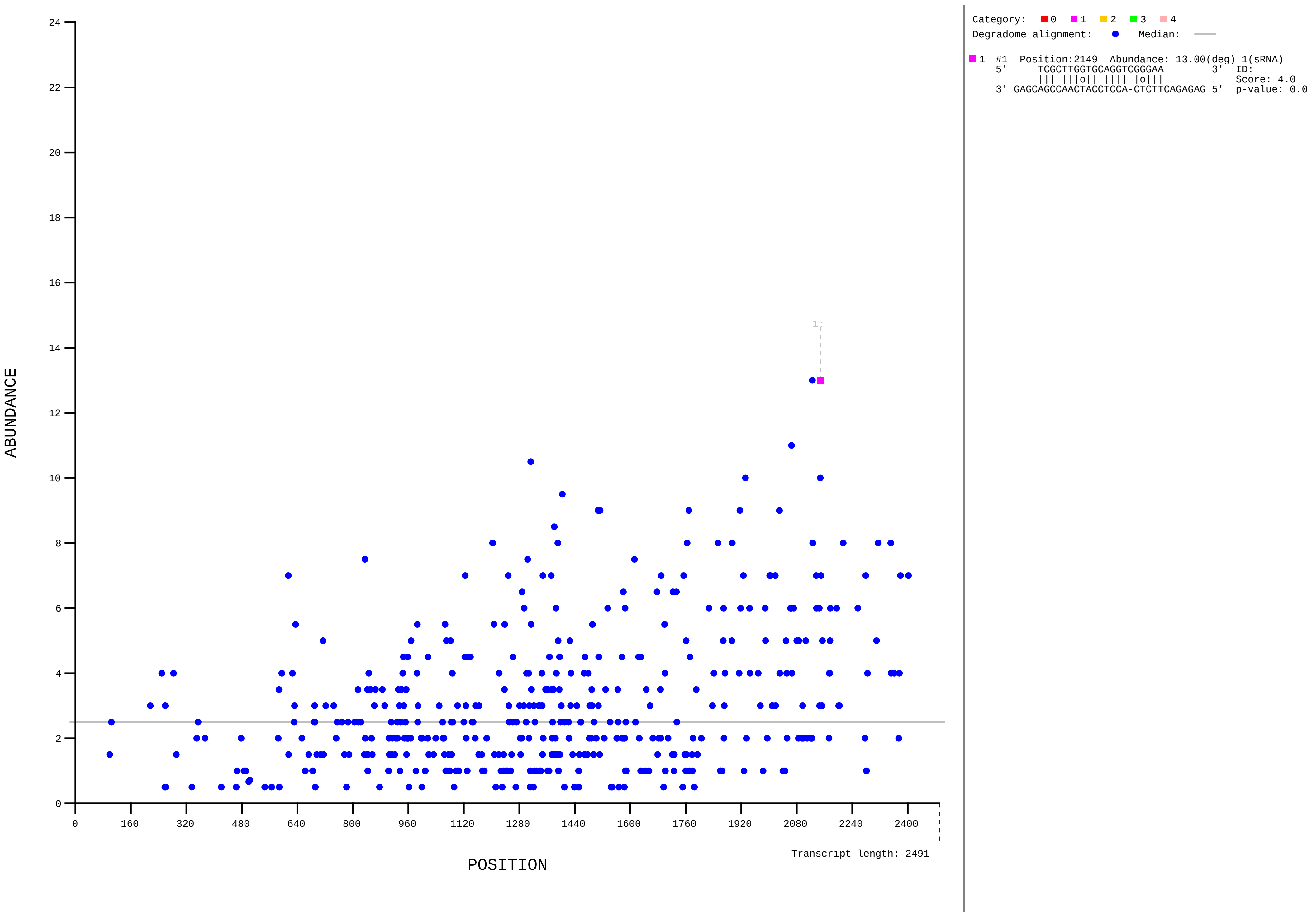

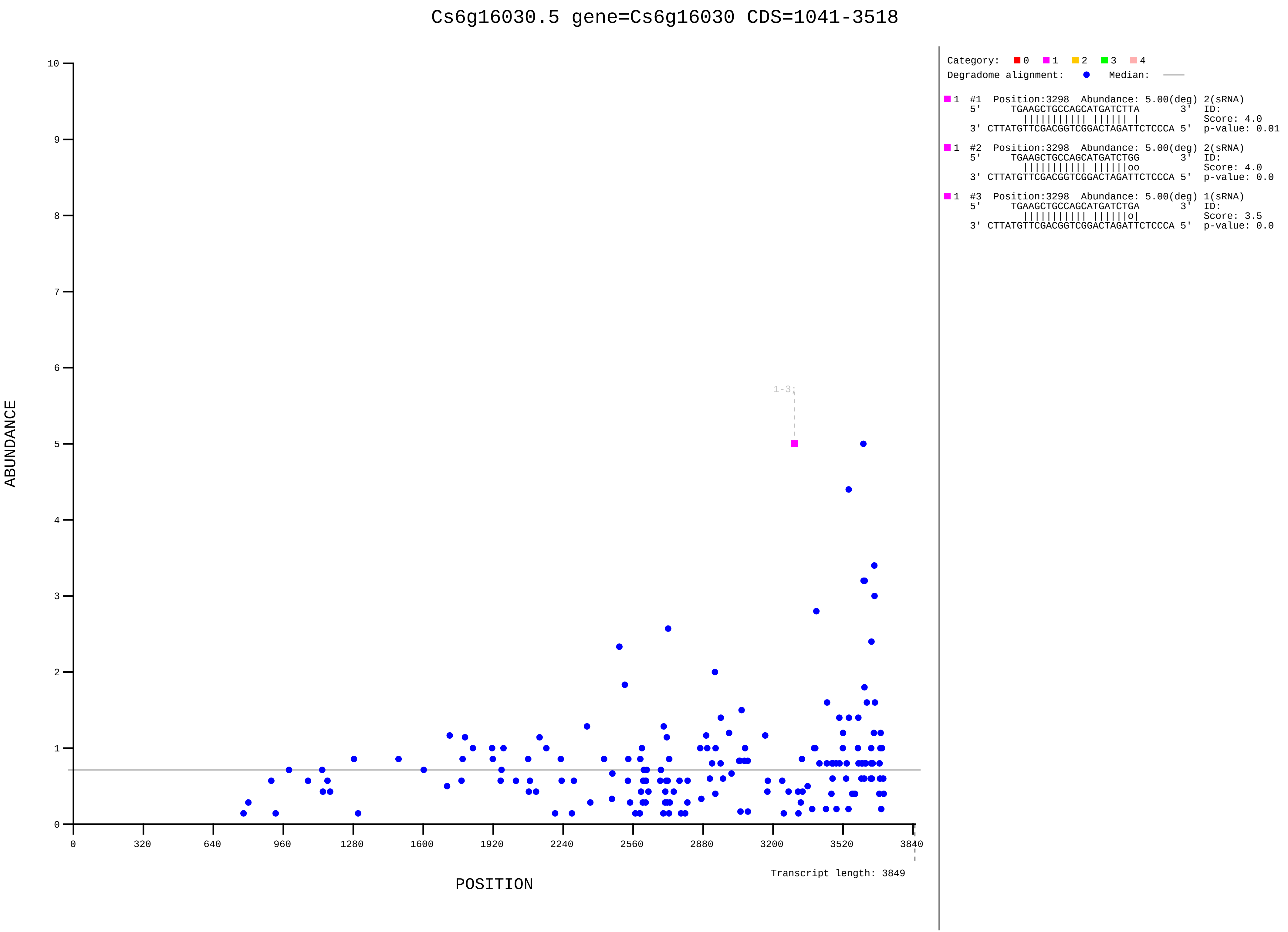

Cs6g16030.5 gene=Cs6g16030 CDS=1041-3518
ABUNDANCE
POSITION
Transcript length: 3849
1-3
0
320
640
960
1280
1600
1920
2240
2560
2880
3200
3520
3840
0
1
2
3
4
5
6
7
8
9
10
Category: 0 1 2 3 4
Degradome alignment:
Median:
1 #1 Position:3298 Abundance: 5.00(deg) 2(sRNA)
5' TGAAGCTGCCAGCATGATCTTA 3' ID:
||||||||||| |||||| | Score: 4.0
3' CTTATGTTCGACGGTCGGACTAGATTCTCCCA 5' p-value: 0.01
1 #2 Position:3298 Abundance: 5.00(deg) 2(sRNA)
5' TGAAGCTGCCAGCATGATCTGG 3' ID:
||||||||||| ||||||oo Score: 4.0
3' CTTATGTTCGACGGTCGGACTAGATTCTCCCA 5' p-value: 0.0
1 #3 Position:3298 Abundance: 5.00(deg) 1(sRNA)
5' TGAAGCTGCCAGCATGATCTGA 3' ID:
||||||||||| ||||||o| Score: 3.5
3' CTTATGTTCGACGGTCGGACTAGATTCTCCCA 5' p-value: 0.0

Cs6g16030.4 gene=Cs6g16030 CDS=495-2915

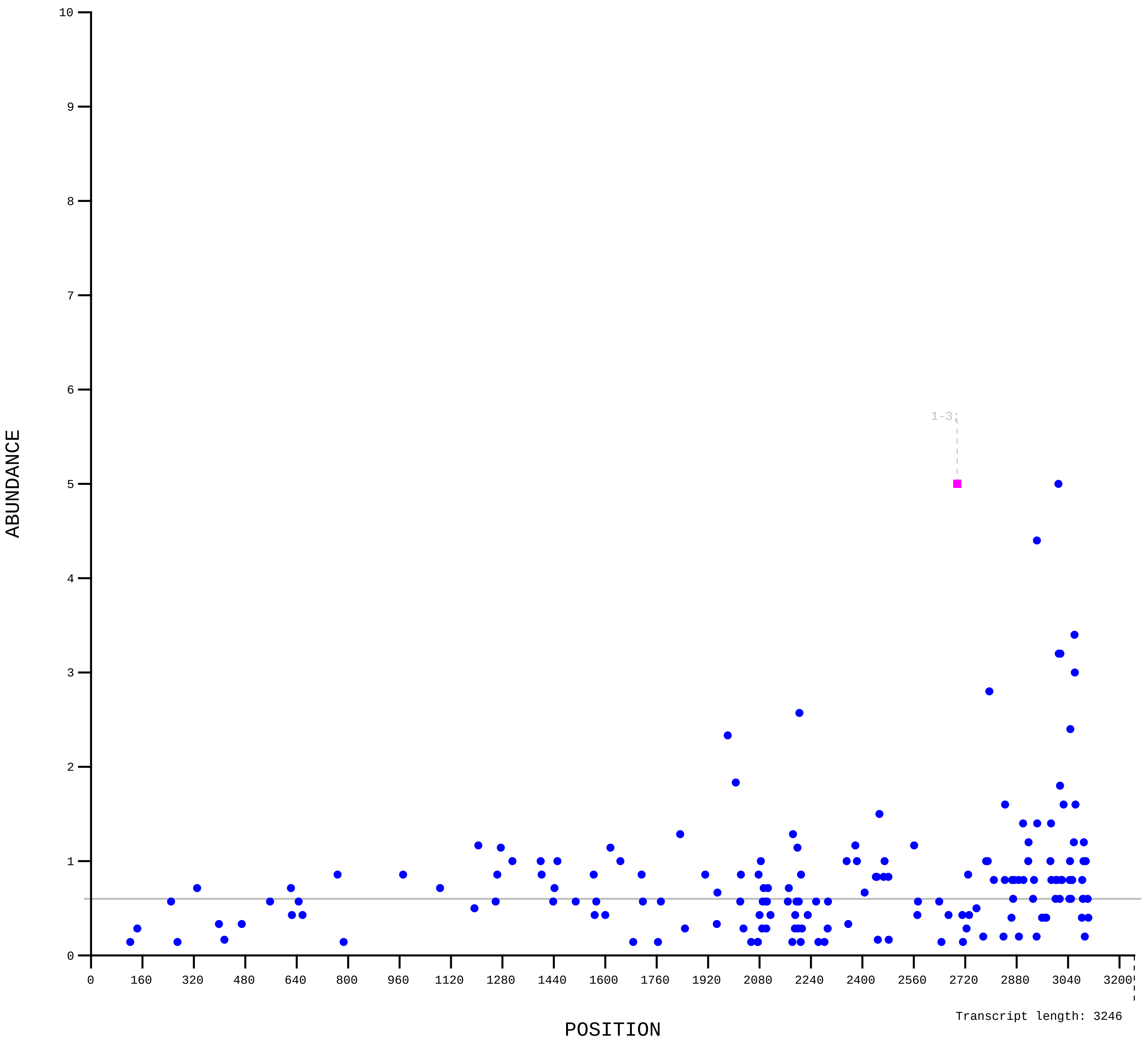

Category: 0 1 2 3 4
Degradome alignment: Median:

1 #1 Position:2695 Abundance: 5.00(deg) 2(sRNA)
```
5'     TGAAGCTGCCAGCATGATCTTA       3'  ID:
          ||||||||||| |||||| |              Score: 4.0
3' CTTATGTTCGACGGTCGGACTAGATTCTCCCA 5'  p-value: 0.0
```

1 #2 Position:2695 Abundance: 5.00(deg) 2(sRNA)
```
5'     TGAAGCTGCCAGCATGATCTGG       3'  ID:
          ||||||||||| ||||||oo              Score: 4.0
3' CTTATGTTCGACGGTCGGACTAGATTCTCCCA 5'  p-value: 0.01
```

1 #3 Position:2695 Abundance: 5.00(deg) 1(sRNA)
```
5'     TGAAGCTGCCAGCATGATCTGA       3'  ID:
          ||||||||||| ||||||o|              Score: 3.5
3' CTTATGTTCGACGGTCGGACTAGATTCTCCCA 5'  p-value: 0.0
```

Cs6g16030.1 gene=Cs6g16030 CDS=1151-3463

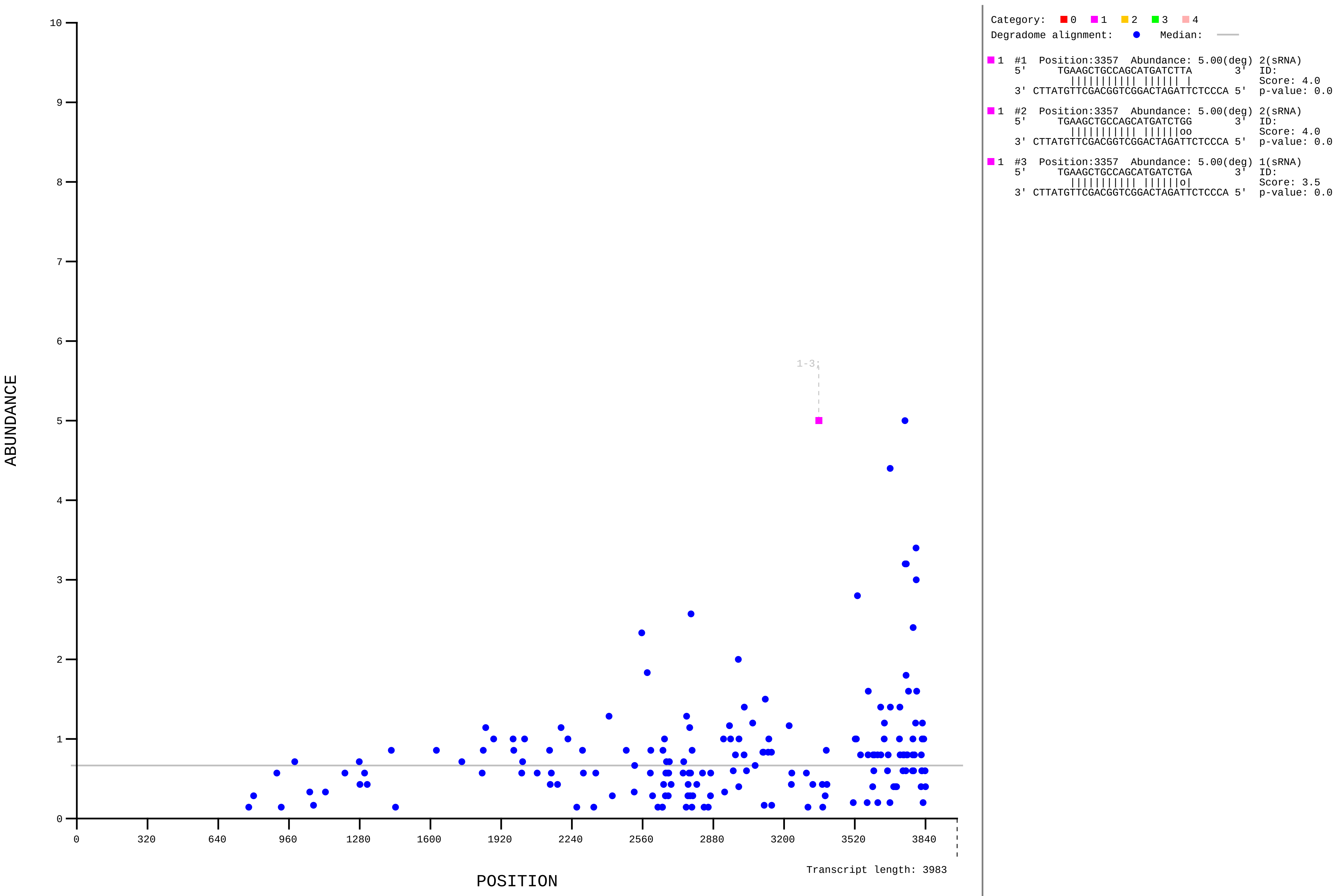

Cs6g16030.6 gene=Cs6g16030 CDS=495-3002

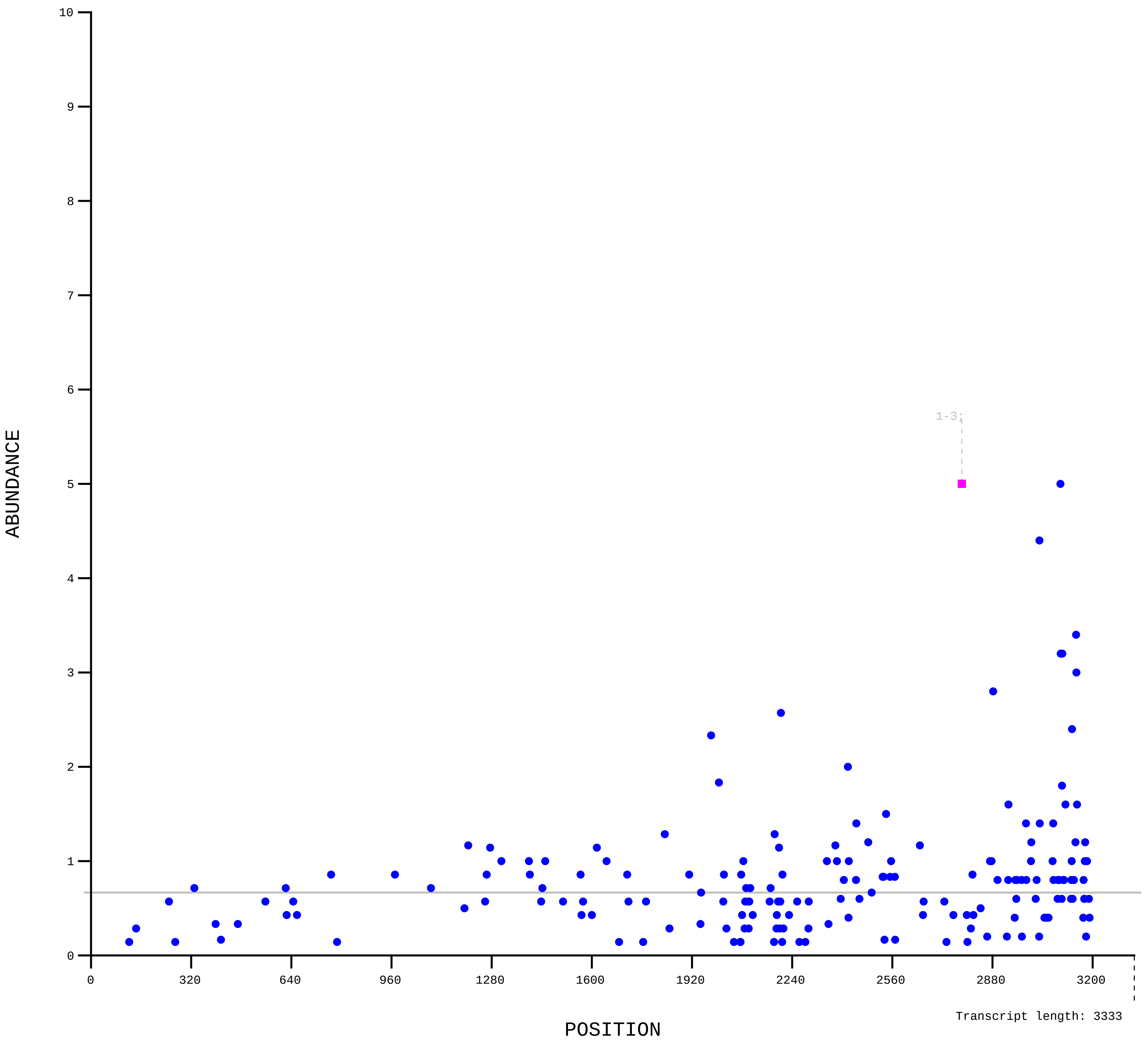

Category: 0 1 2 3 4
Degradome alignment: Median:

```
1  #1  Position:2782  Abundance: 5.00(deg) 2(sRNA)
   5'     TGAAGCTGCCAGCATGATCTTA       3'  ID:
            ||||||||||| |||||| |           Score: 4.0
   3' CTTATGTTCGACGGTCGGACTAGATTCTCCCA 5'  p-value: 0.01

1  #2  Position:2782  Abundance: 5.00(deg) 2(sRNA)
   5'     TGAAGCTGCCAGCATGATCTGG       3'  ID:
            ||||||||||| ||||||oo           Score: 4.0
   3' CTTATGTTCGACGGTCGGACTAGATTCTCCCA 5'  p-value: 0.0

1  #3  Position:2782  Abundance: 5.00(deg) 1(sRNA)
   5'     TGAAGCTGCCAGCATGATCTGA       3'  ID:
            ||||||||||| ||||||o|           Score: 3.5
   3' CTTATGTTCGACGGTCGGACTAGATTCTCCCA 5'  p-value: 0.0
```

Cs6g16030.3 gene=Cs6g16030 CDS=495-2930

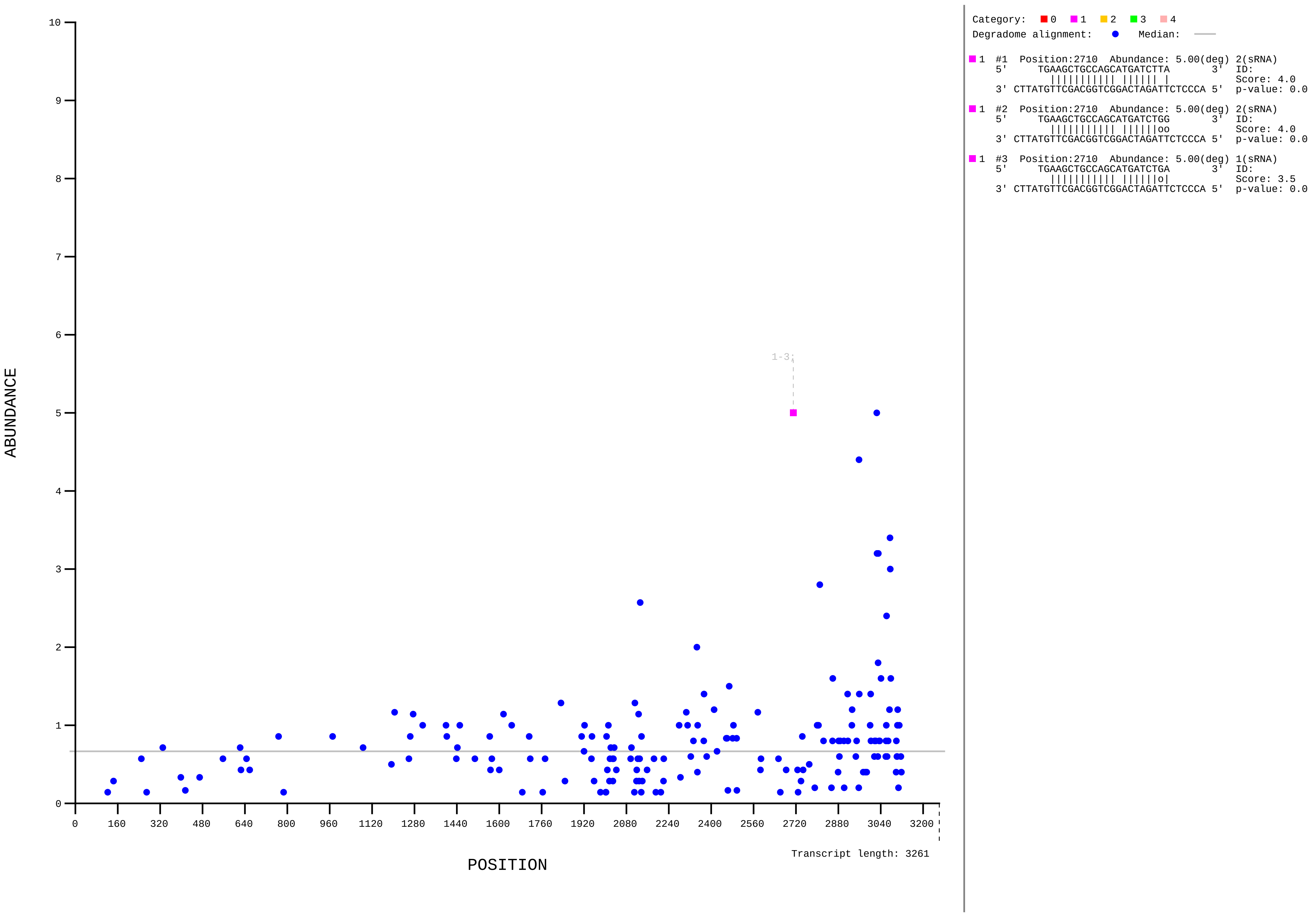

Cs9g08500.4 gene=Cs9g08500 CDS=1753-4947

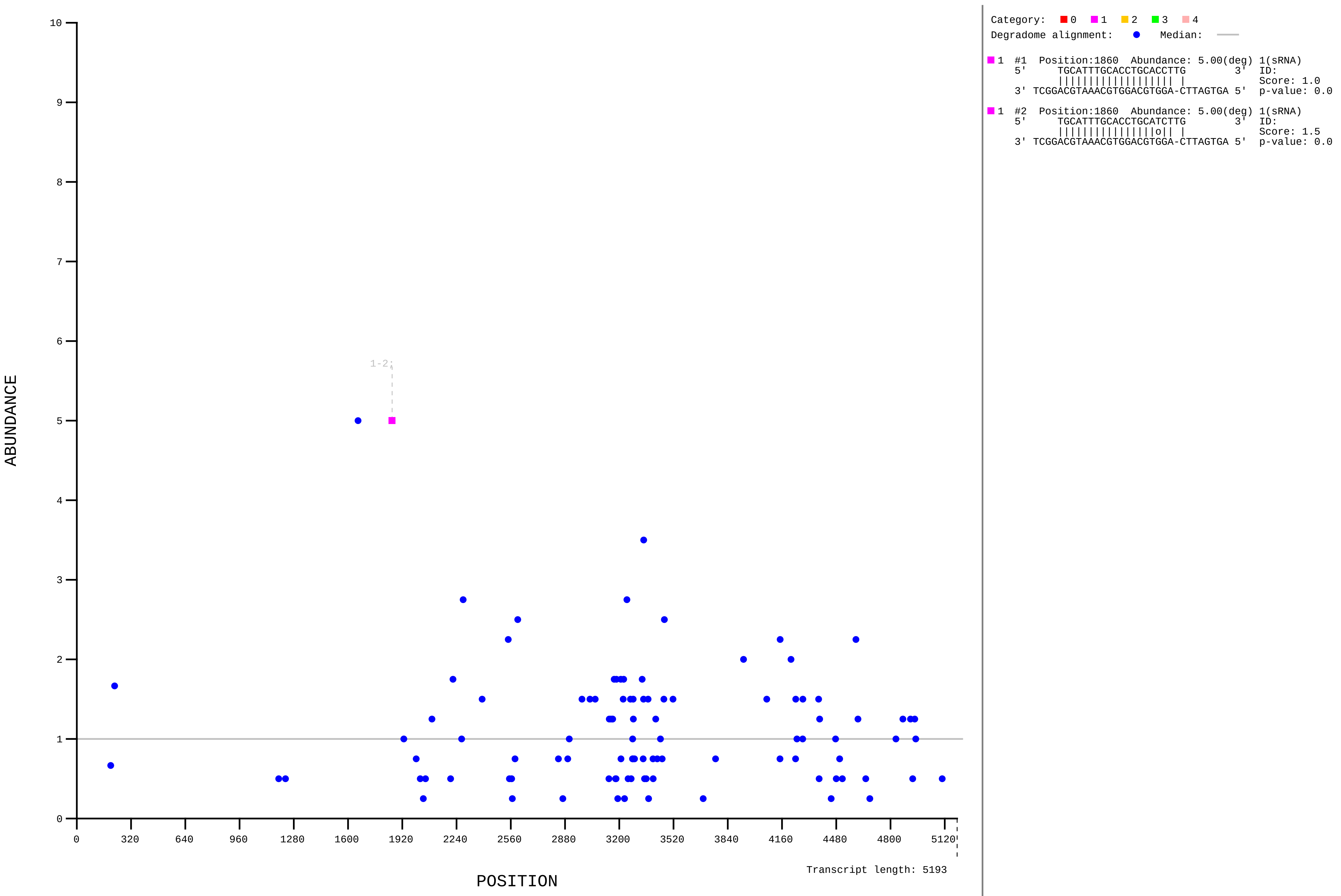

Cs9g08500.3 gene=Cs9g08500 CDS=365-3559

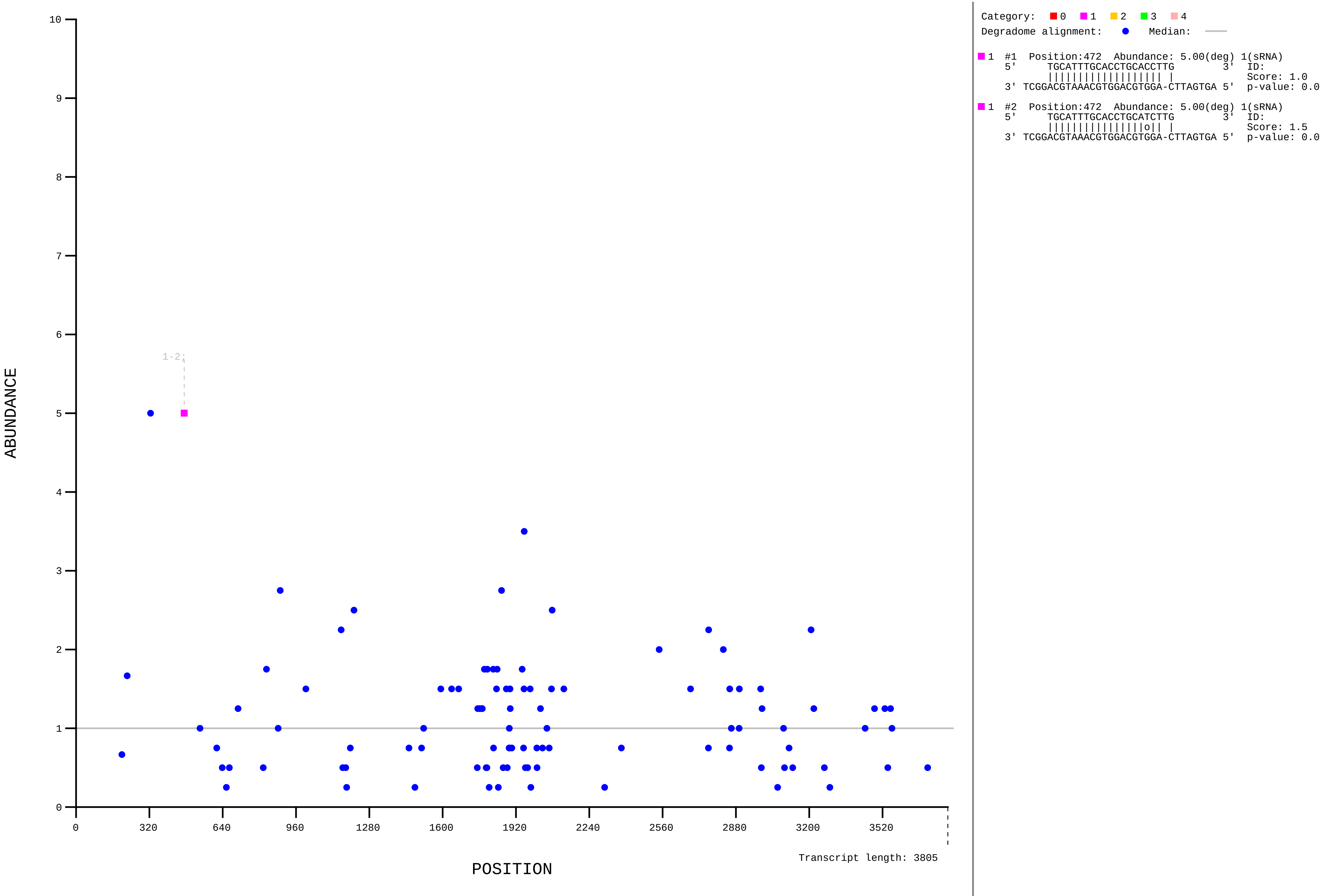

Cs7g11770.3 gene=Cs7g11770 CDS=381-1901

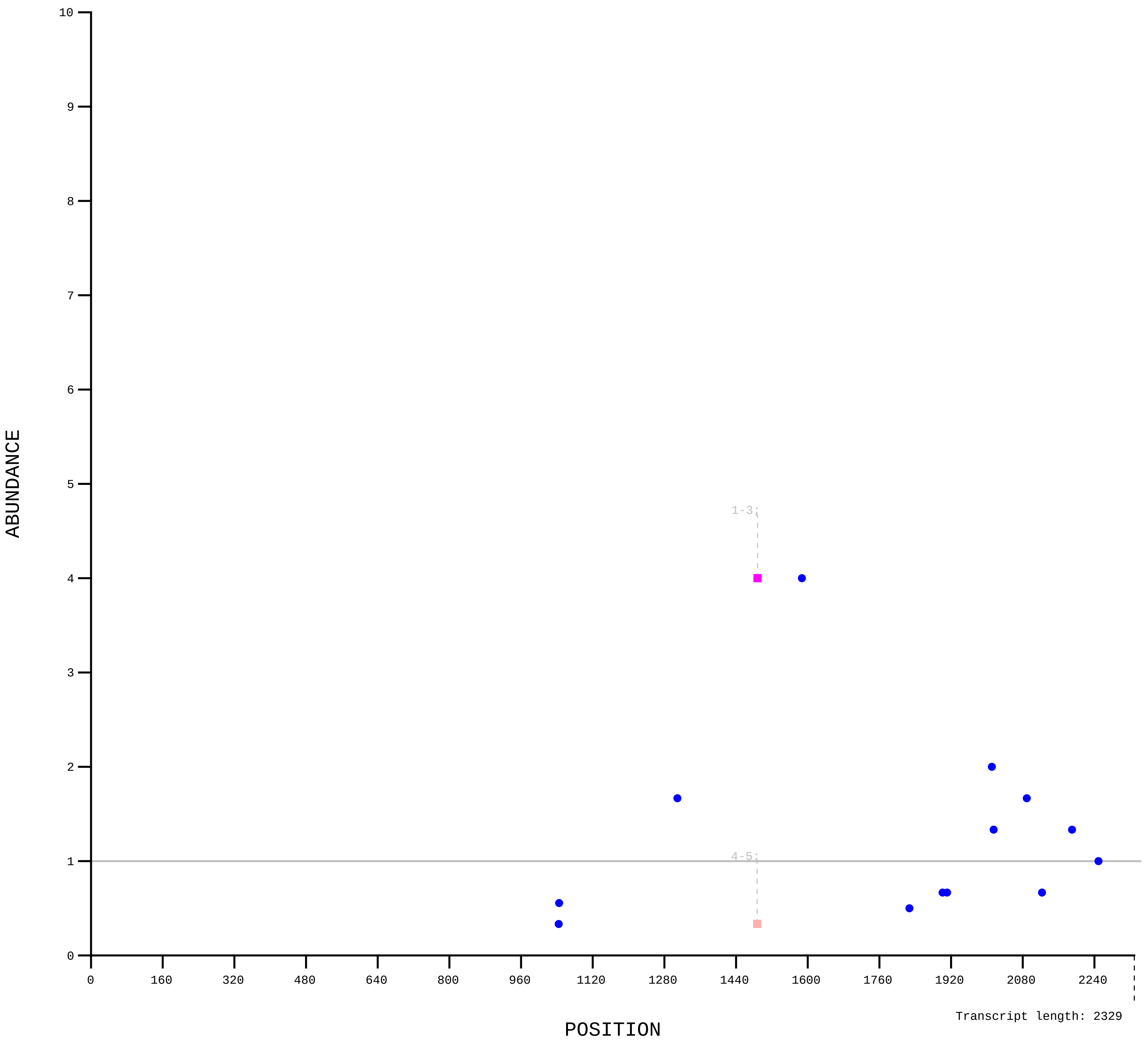

Category: 0 1 2 3 4
Degradome alignment: Median:

```
1  #1  Position:1488  Abundance: 4.00(deg) 1(sRNA)
   5'     CTGACAGAAGAGAGTGAGCAC        3'  ID:
          |||||||||||||| ||||||            Score: 1.0
   3' ACTCGACTGTCTTCTCTCTCTCGTGGTGACCT 5'  p-value: 0.0

1  #2  Position:1488  Abundance: 4.00(deg) 1(sRNA)
   5'     TTGACAGAAGAGAGTGAGCAC        3'  ID:
          o||||||||||||| ||||||            Score: 1.5
   3' ACTCGACTGTCTTCTCTCTCTCGTGGTGACCT 5'  p-value: 0.0

1  #3  Position:1488  Abundance: 4.00(deg) 1(sRNA)
   5'     GTGACAGAAGATAGAGAGCGC        3'  ID:
           |||||||||| |||||||o|            Score: 2.5
   3' ACTCGACTGTCTTCTCTCTCTCGTGGTGACCT 5'  p-value: 0.0

4  #4  Position:1487  Abundance: 0.33(deg) 1(sRNA)
   5'     TGACAGAAGAGAGTGAGCAC         3'  ID:
          ||||||||||||| ||||||             Score: 1.0
   3' CTCGACTGTCTTCTCTCTCTCGTGGTGACCTC 5'  p-value: 0.0

4  #5  Position:1487  Abundance: 0.33(deg) 1(sRNA)
   5'     TGACAGAAGATAGAGAGCGC         3'  ID:
          |||||||||| |||||||o|             Score: 1.5
   3' CTCGACTGTCTTCTCTCTCTCGTGGTGACCTC 5'  p-value: 0.0
```

Cs1g11750.2 gene=Cs1g11750 CDS=110-1318

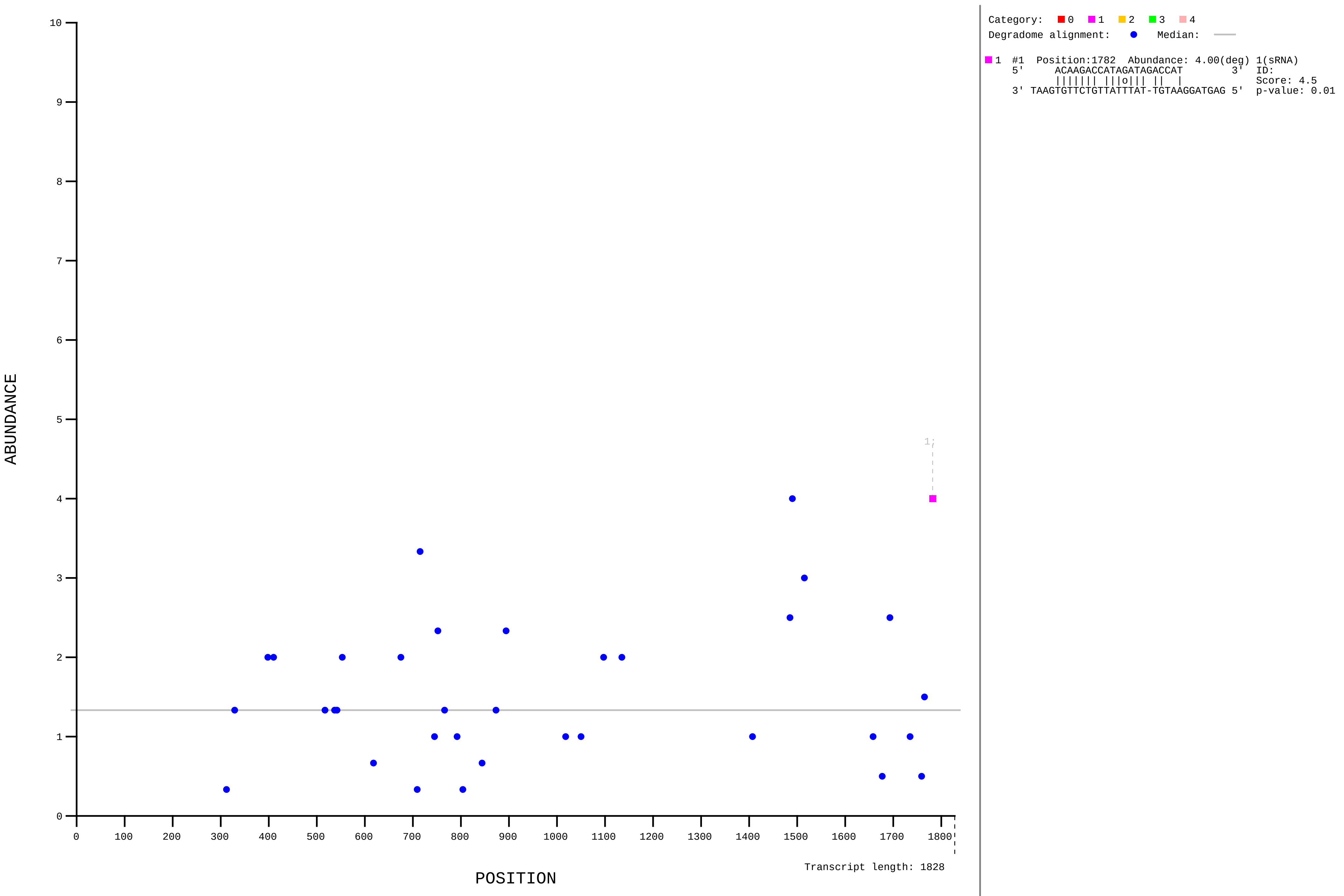

orange1.1t02555.1 gene=orange1.1t02555 CDS=289-1848

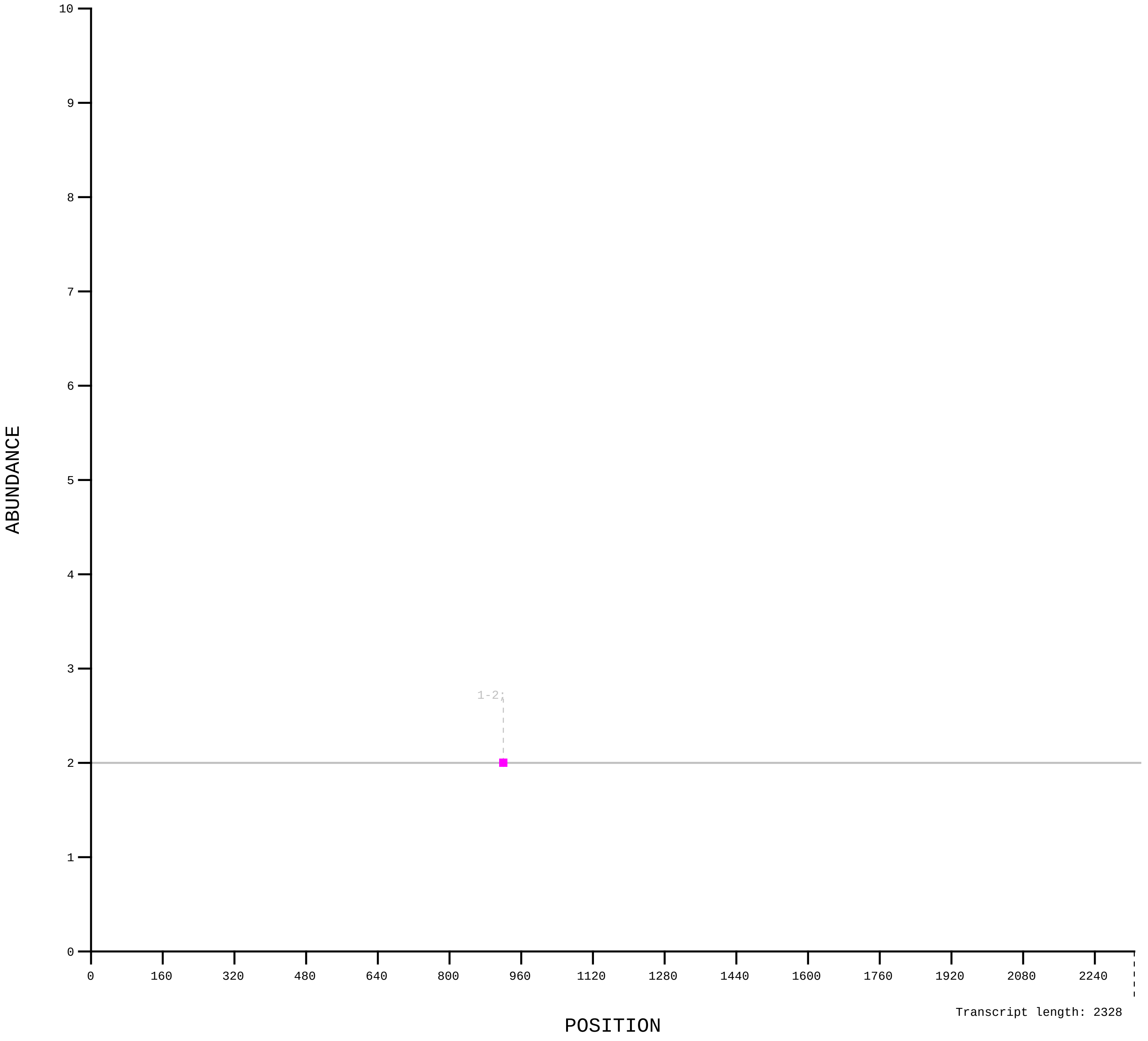

Category: 0 1 2 3 4
Degradome alignment: Median:

1 #1 Position:920 Abundance: 2.00(deg) 3(sRNA)

```
5'    TTCCACA-GCTTTCTTGAACTG        3'  ID:
      ||||||| ||||||||||||              Score: 3.0
3' TTCAAGGTGTCCGAAAGAACTTGCACCTGCCG 5'  p-value: 0.0
```

1 #2 Position:920 Abundance: 2.00(deg) 1(sRNA)

```
5'    TTCCAC-GGCTTTCTTGAACTT        3'  ID:
      |||||| ||||||||||||| |            Score: 2.0
3' TTCAAGGTGTCCGAAAGAACTTGCACCTGCCG 5'  p-value: 0.0
```

orange1.1t02555.2 gene=orange1.1t02555 CDS=289-1953

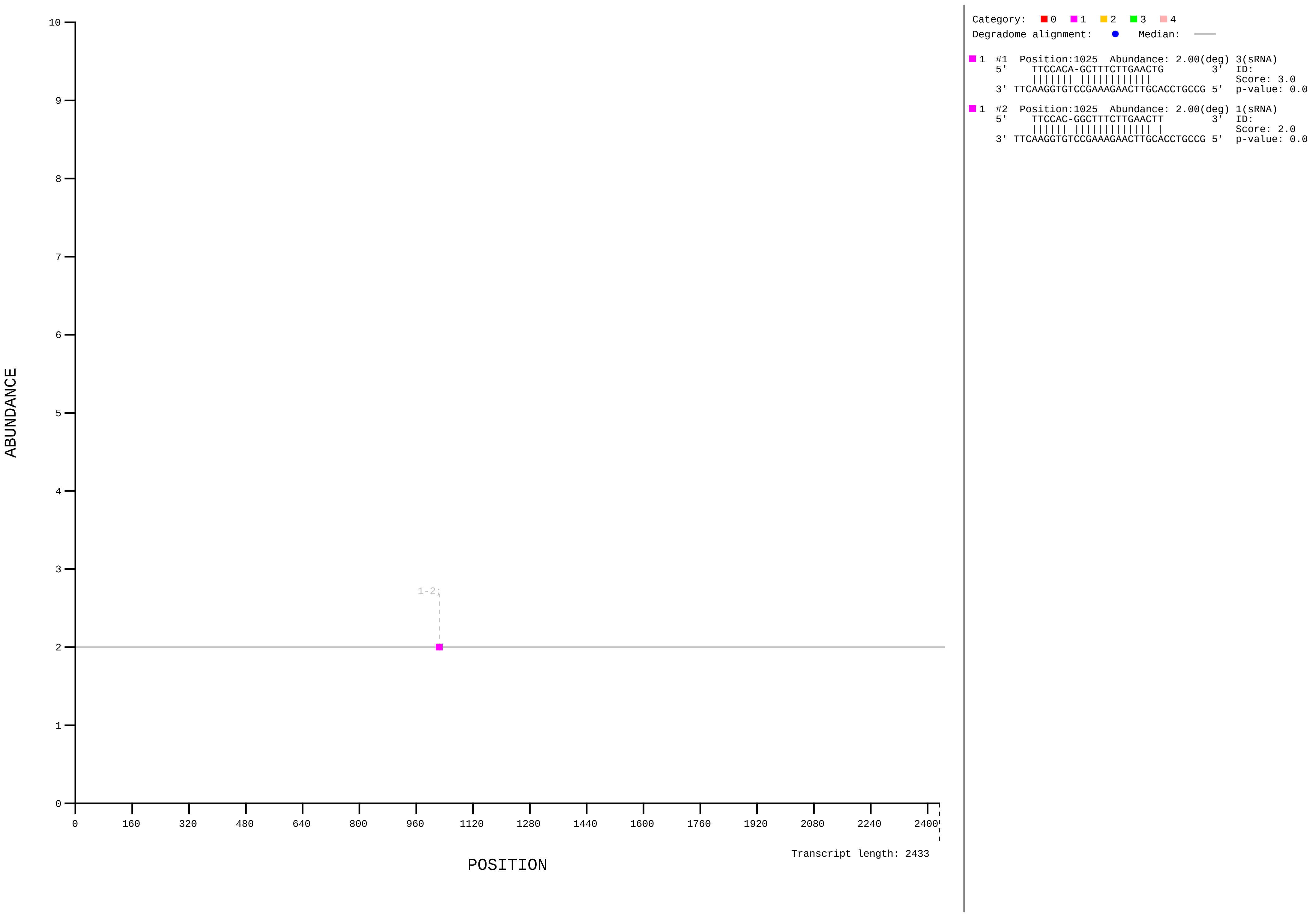

Cs2g09770.4 gene=Cs2g09770 CDS=484-2928

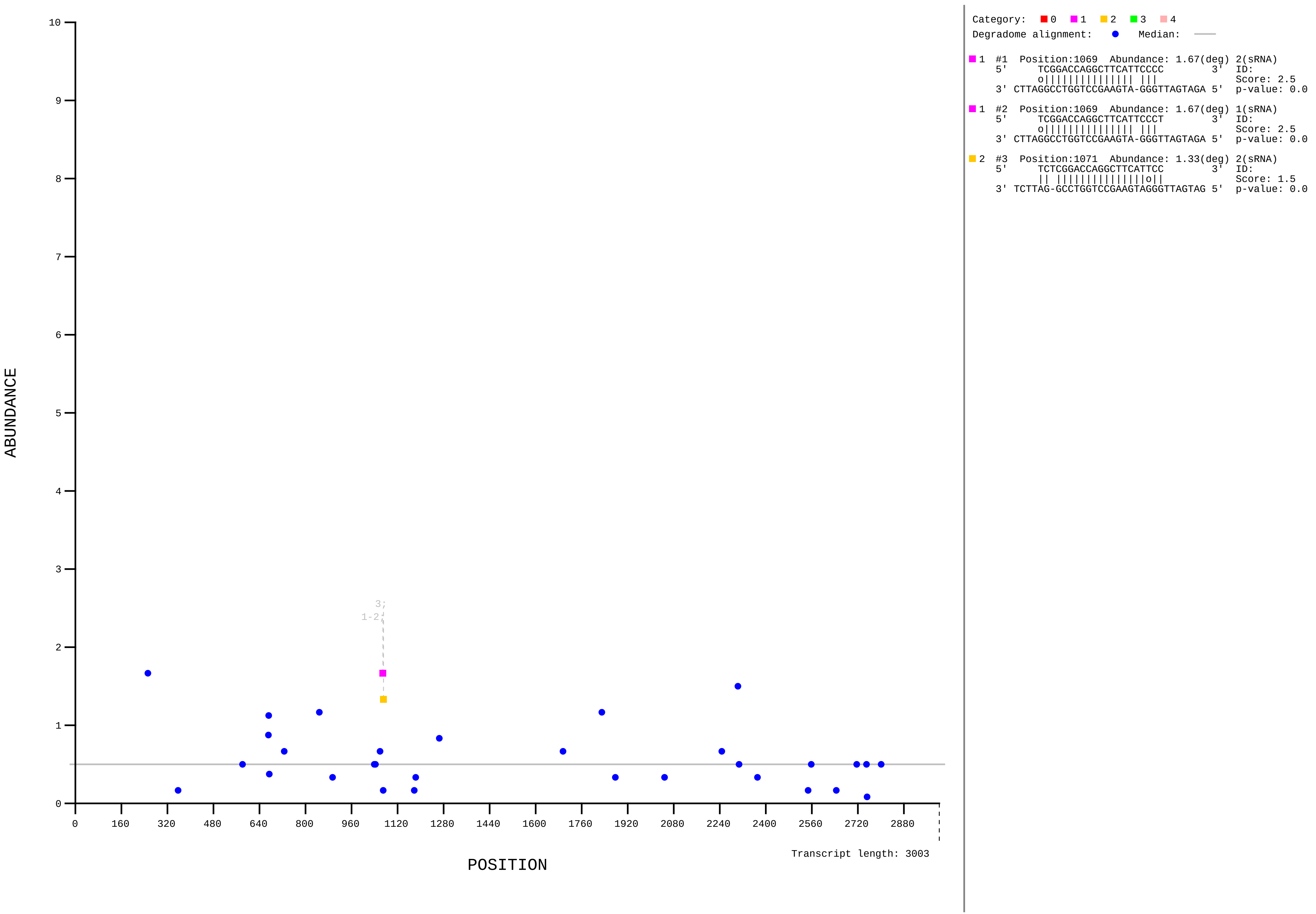

Cs7g22420.1 gene=Cs7g22420 CDS=222-1310

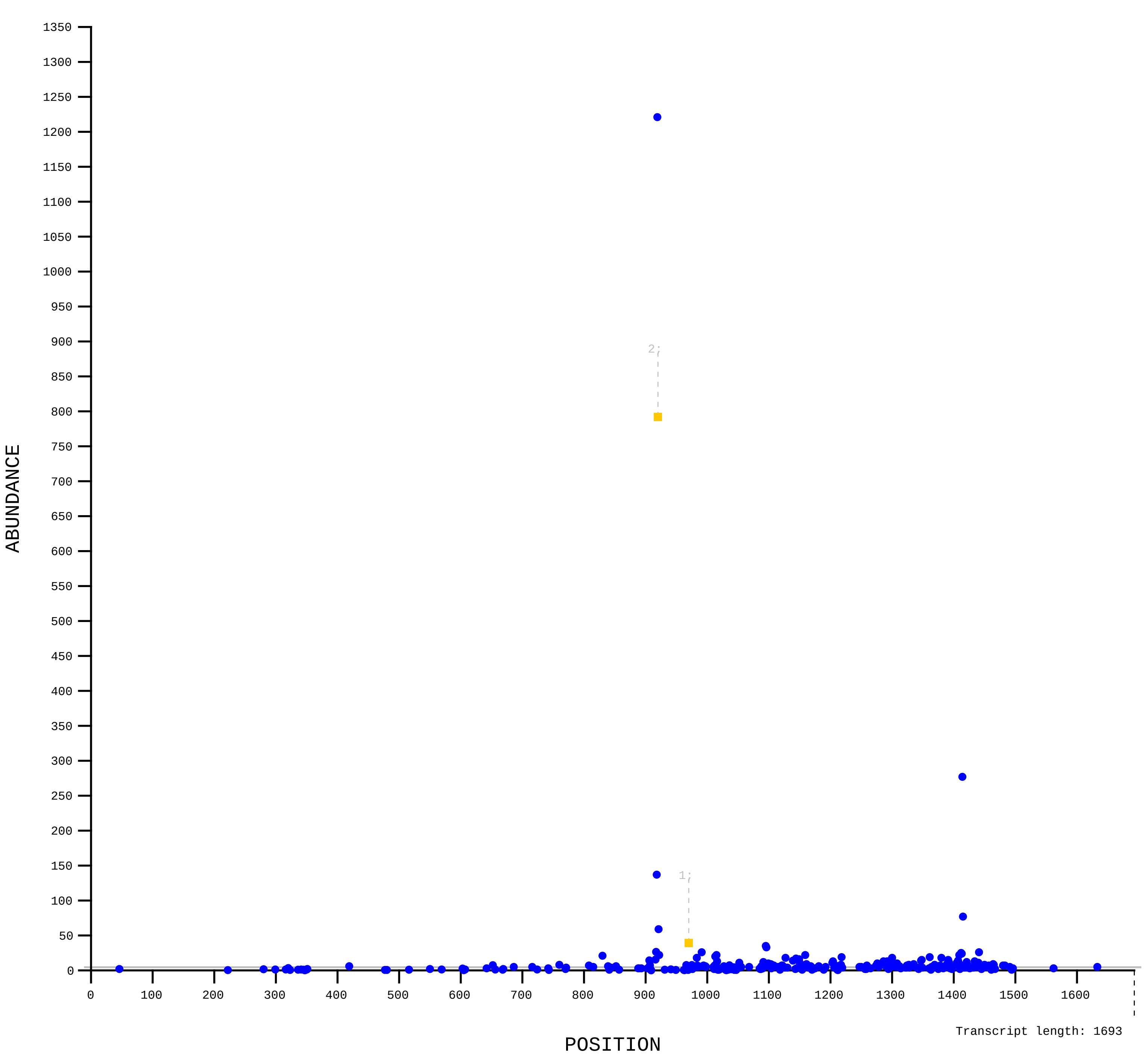

Category: 0 1 2 3 4
Degradome alignment: Median:

```
2  #1  Position:970  Abundance: 39.00(deg) 1(sRNA)
   5'     TTGAGTTCTGCAAGCCGTCGA        3'  ID:
          |||||||||||||||||||||            Score: 0.0
   3' AGCTAACTCAAGACGTTCGGCAGCTCAAAGAC 5'  p-value: 0.0

2  #2  Position:920  Abundance: 792.00(deg) 1(sRNA)
   5'     TTTTTCGGCAACATGATTTCT        3'  ID:
          |||||| ||||||||||||||            Score: 1.0
   3' GCTAAAAAAGACGTTGTACTAAAGACATTATT 5'  p-value: 0.0
```

Cs7g22430.1 gene=Cs7g22430 CDS=1-1167

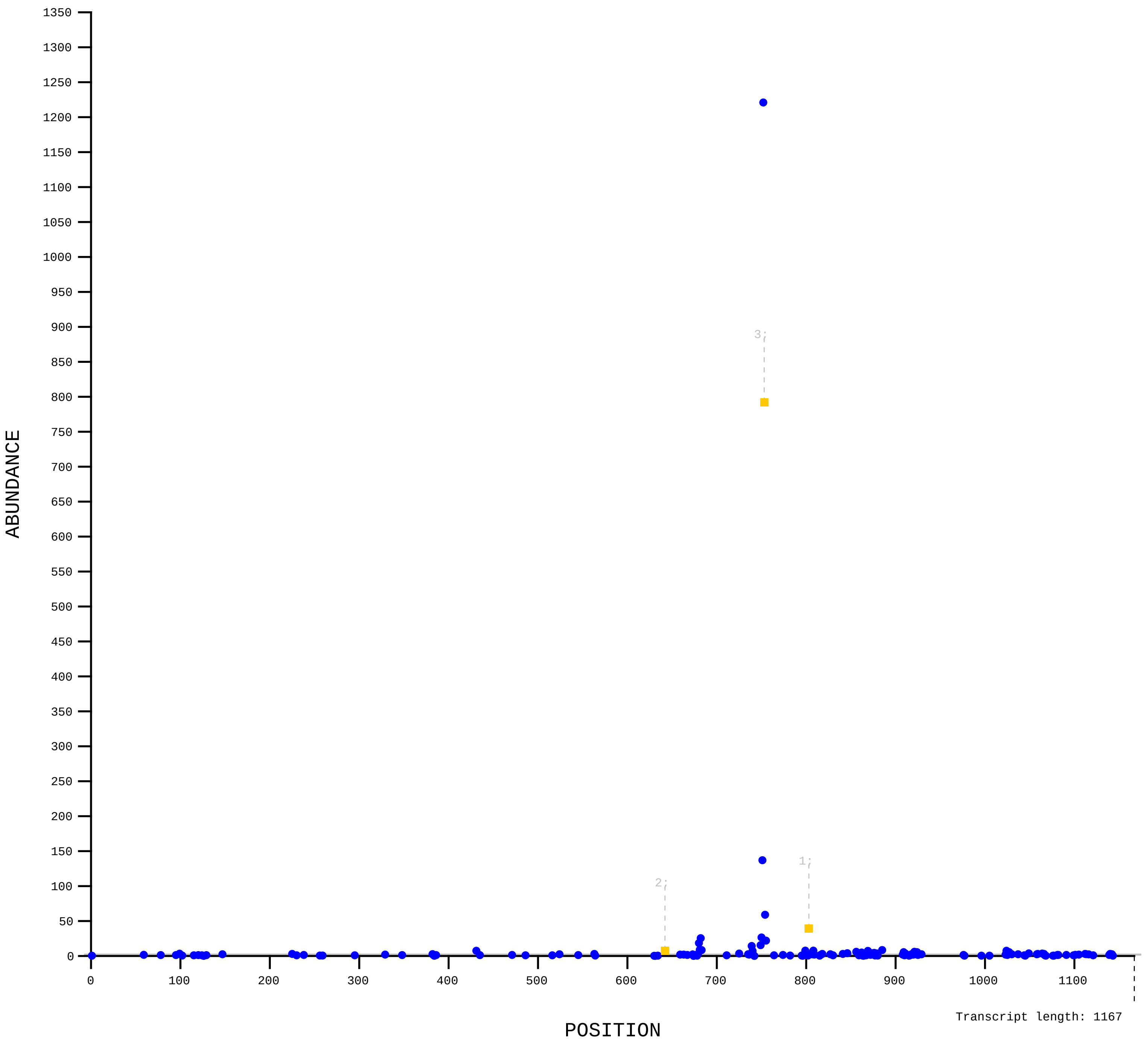

Category: 0 1 2 3 4
Degradome alignment: ● Median: —

```
2  #1  Position:803  Abundance: 39.00(deg) 1(sRNA)
   5'     TTGAGTTCTGCAAGCCGTCGA        3'  ID:
          |||||||||||||||||||||            Score: 0.0
   3' AGCTAACTCAAGACGTTCGGCAGCTCAAAGAC 5'  p-value: 0.0

2  #2  Position:642  Abundance: 7.50(deg) 1(sRNA)
   5'     TTTTTCGGCAACATGATTTCT        3'  ID:
          |||||| ||||||||||||||            Score: 1.0
   3' AATGAAAAAGACGTTGTACTAAAGATAATCAT 5'  p-value: 0.0

2  #3  Position:753  Abundance: 792.00(deg) 1(sRNA)
   5'     TTTTTCGGCAACATGATTTCT        3'  ID:
          |||||| ||||||||||||||            Score: 1.0
   3' GCTAAAAAAGACGTTGTACTAAAGACATTGTT 5'  p-value: 0.0
```

Cs7g22450.1 gene=Cs7g22450 CDS=72-1238

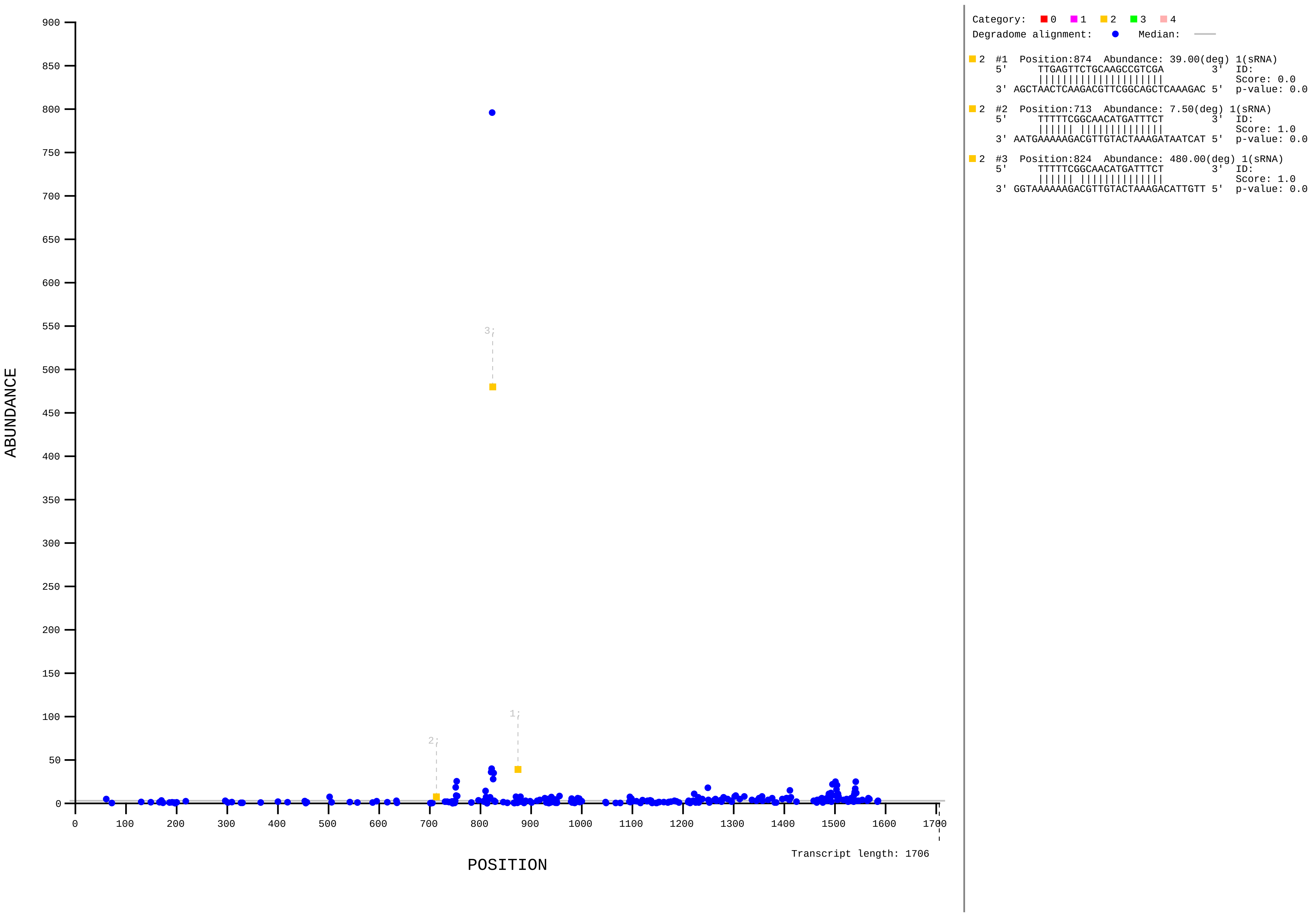

Cs1g09660.1 gene=Cs1g09660 CDS=74-1249

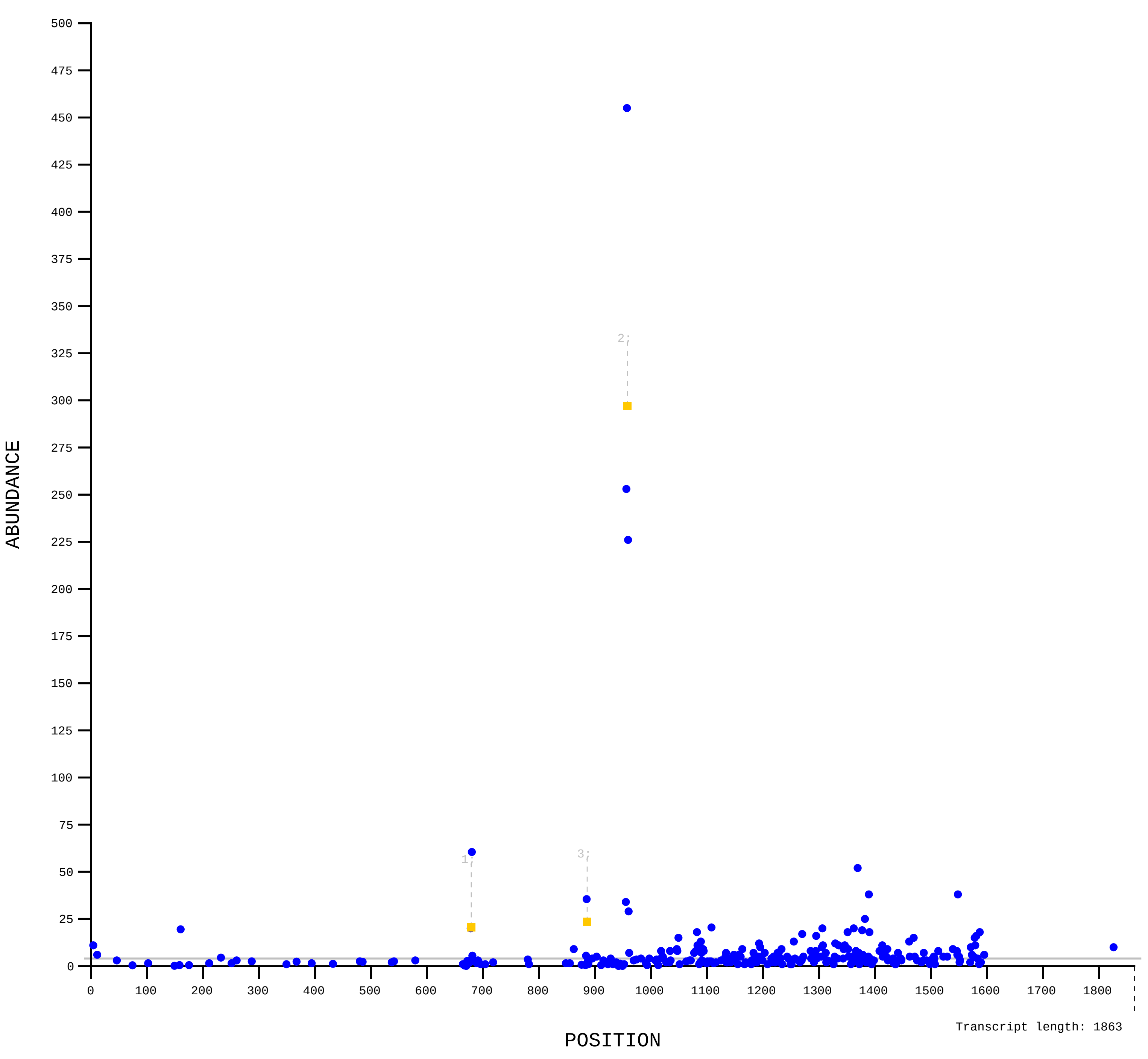


```
Category:  0  1  2  3  4
Degradome alignment:     Median:

2  #1  Position:679  Abundance: 20.50(deg) 1(sRNA)
   5'     TTTTTCGGCAACATGATTTCT         3'  ID:
           ||||| ||||||||||||||             Score: 2.0
   3' CTCATAAAAGACGTTGTACTAAAGATAGTCTT 5'  p-value: 0.02

2  #2  Position:958  Abundance: 297.00(deg) 1(sRNA)
   5'     TTTTTCGGCAACATGATTTCT         3'  ID:
           ||||| ||||||||||||||             Score: 2.0
   3' TTAATAAAAGACGTTGTACTAAAGATCTTGTT 5'  p-value: 0.01

2  #3  Position:886  Abundance: 23.50(deg) 1(sRNA)
   5'     TTTTTCGGCAACATGATTTCT         3'  ID:
           ||||| |||||||||||||o             Score: 2.5
   3' CTCGTAAAAGACGTTGTACTAAAGGTAATCTT 5'  p-value: 0.05
```

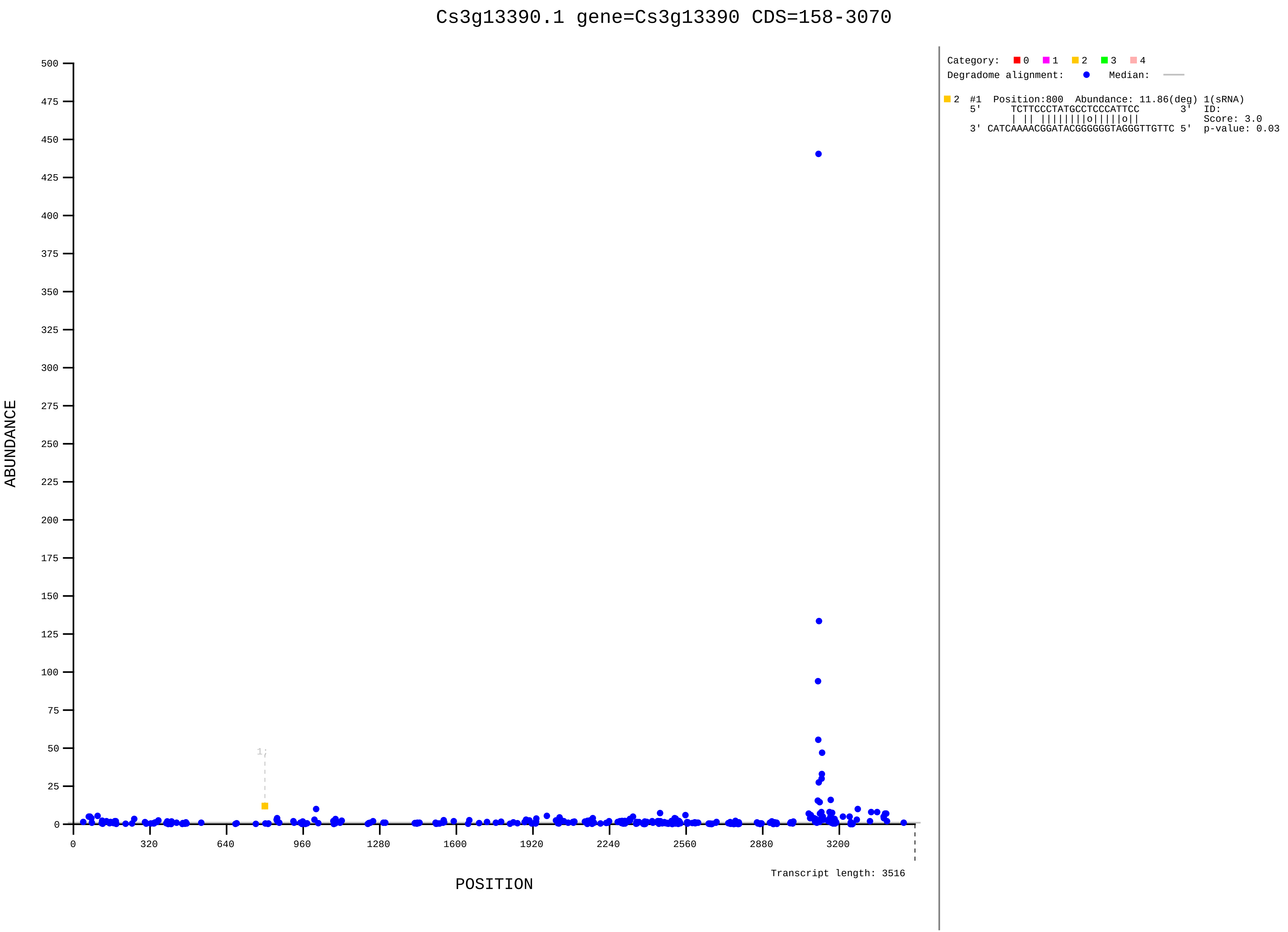
Cs3g13390.1 gene=Cs3g13390 CDS=158-3070
ABUNDANCE
POSITION
Transcript length: 3516
Category: 0 1 2 3 4
Degradome alignment:
Median:
2 #1 Position:800 Abundance: 11.86(deg) 1(sRNA)
5' TCTTCCCTATGCCTCCCATTCC 3' ID:
| || ||||||||o|||||o||
Score: 3.0
3' CATCAAAACGGATACGGGGGGTAGGGTTGTTC 5' p-value: 0.03

Cs8g16450.2 gene=Cs8g16450 CDS=828-3170

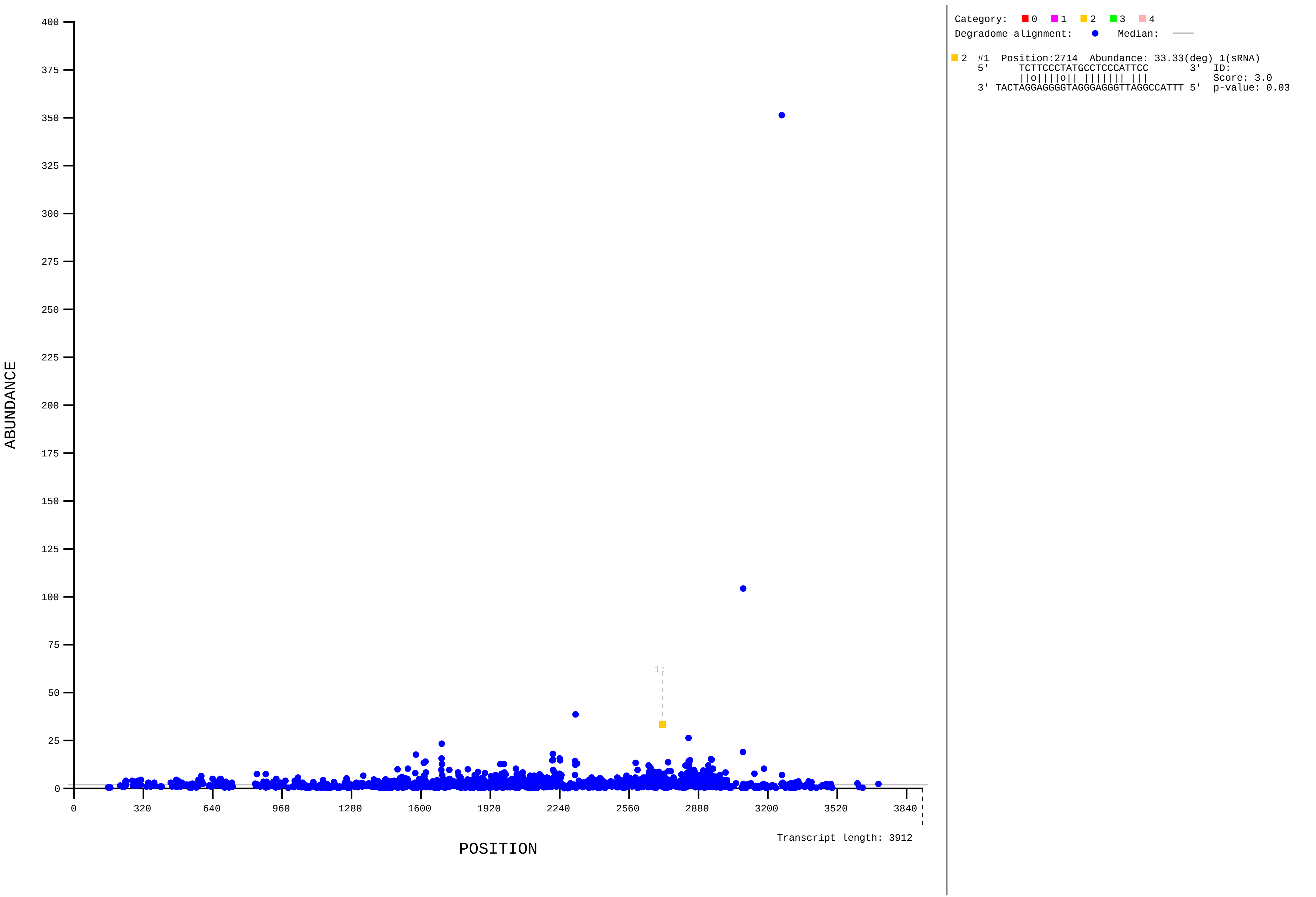

Cs8g16450.1 gene=Cs8g16450 CDS=828-3173

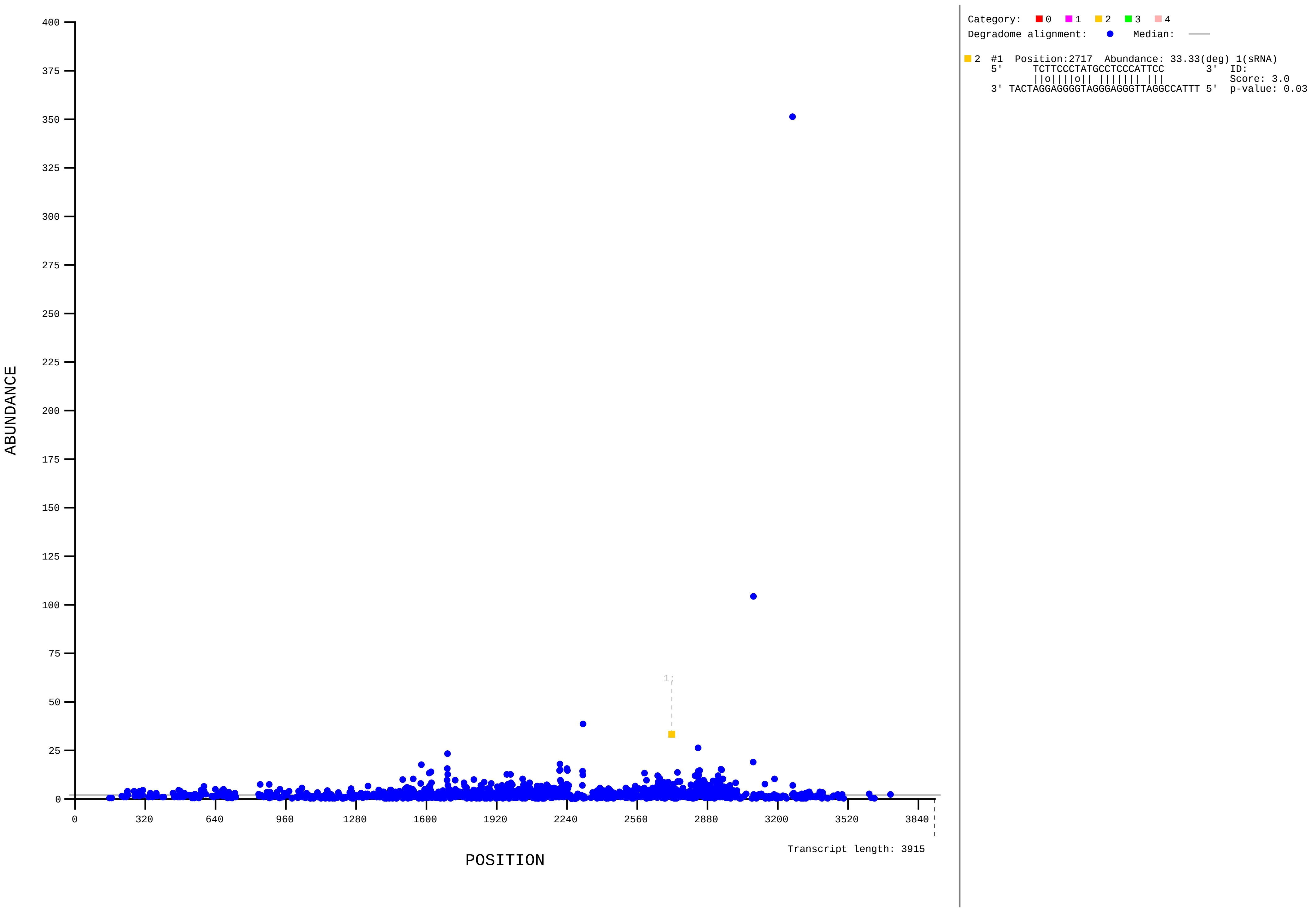

Cs8g16450.3 gene=Cs8g16450 CDS=93-2264

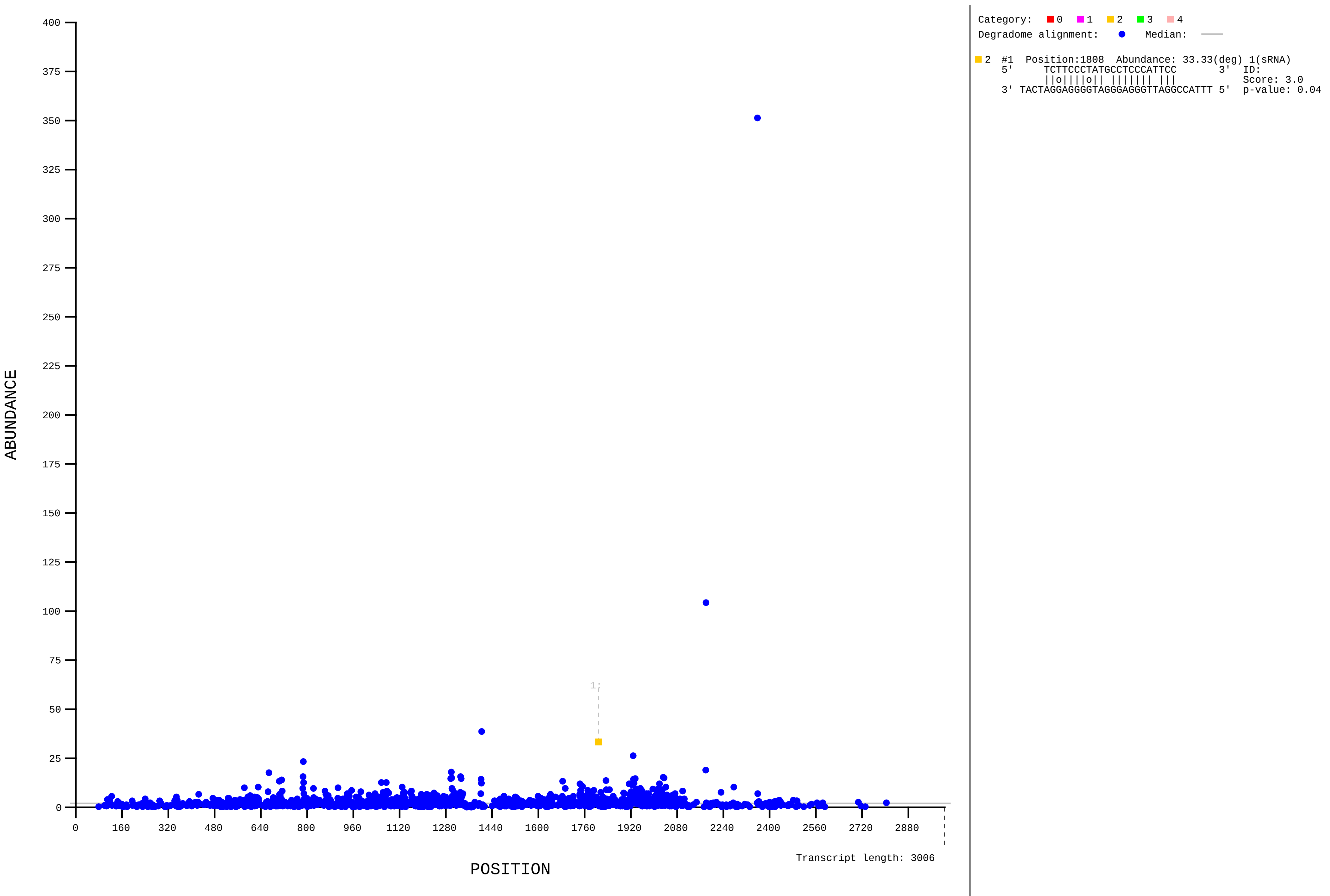

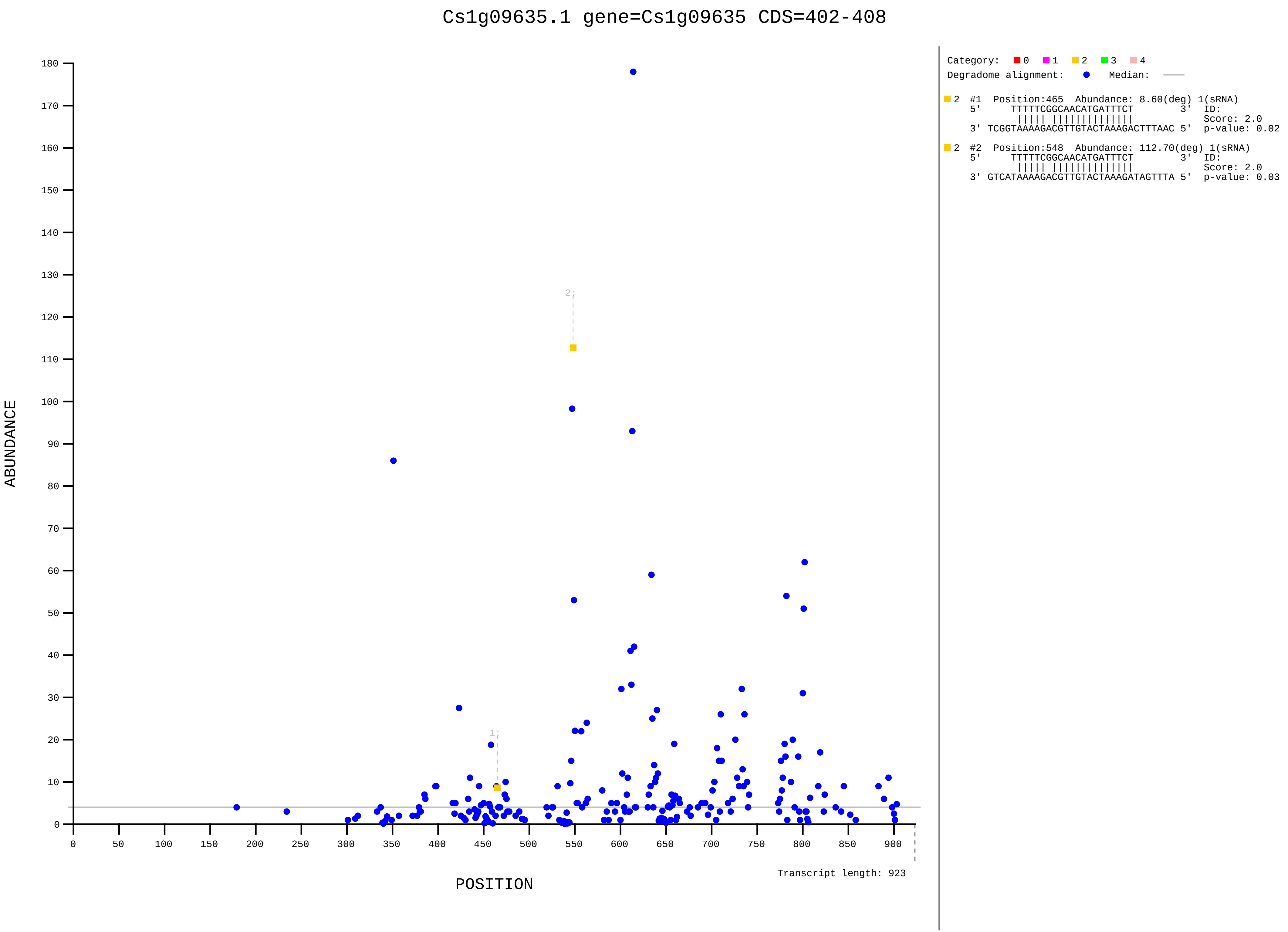
Cs1g09635.1 gene=Cs1g09635 CDS=402-408
ABUNDANCE
POSITION
0
10
20
30
40
50
60
70
80
90
100
110
120
130
140
150
160
170
180
0
50
100
150
200
250
300
350
400
450
500
550
600
650
700
750
800
850
900
1
2
Transcript length: 923
Category: 0 1 2 3 4
Degradome alignment:
Median:
2 #1 Position:465 Abundance: 8.60(deg) 1(sRNA)
5' TTTTTCGGCAACATGATTTCT 3' ID:
||||| ||||||||||||||| Score: 2.0
3' TCGGTAAAAGACGTTGTACTAAAGACTTTAAC 5' p-value: 0.02
2 #2 Position:548 Abundance: 112.70(deg) 1(sRNA)
5' TTTTTCGGCAACATGATTTCT 3' ID:
||||| ||||||||||||||| Score: 2.0
3' GTCATAAAAGACGTTGTACTAAAGATAGTTTA 5' p-value: 0.03

Cs7g12410.3 gene=Cs7g12410 CDS=1284-2378

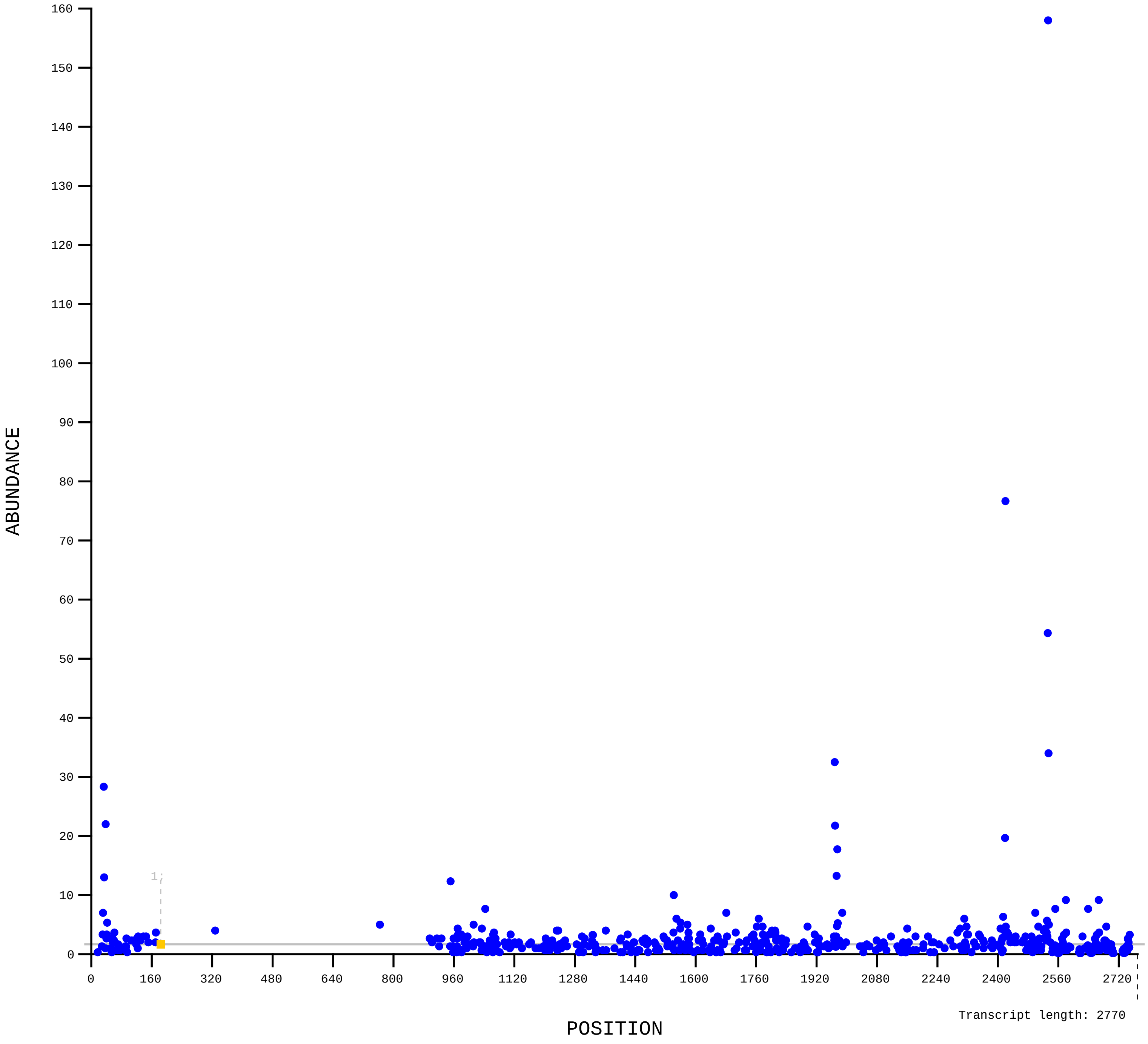


```
Category:  0  1  2  3  4
Degradome alignment:   Median:

2  #1  Position:184  Abundance: 1.67(deg) 1(sRNA)
   5'     TTGAGCCGCGCCAATATCACG        3'  ID:
          || ||||||| |||o| ||||            Score: 3.5
   3' ACATAA-TCGGCGCTGTTGTTGTGCAATAATT 5'  p-value: 0.05
```

Cs1g09600.2 gene=Cs1g09600 CDS=10-240

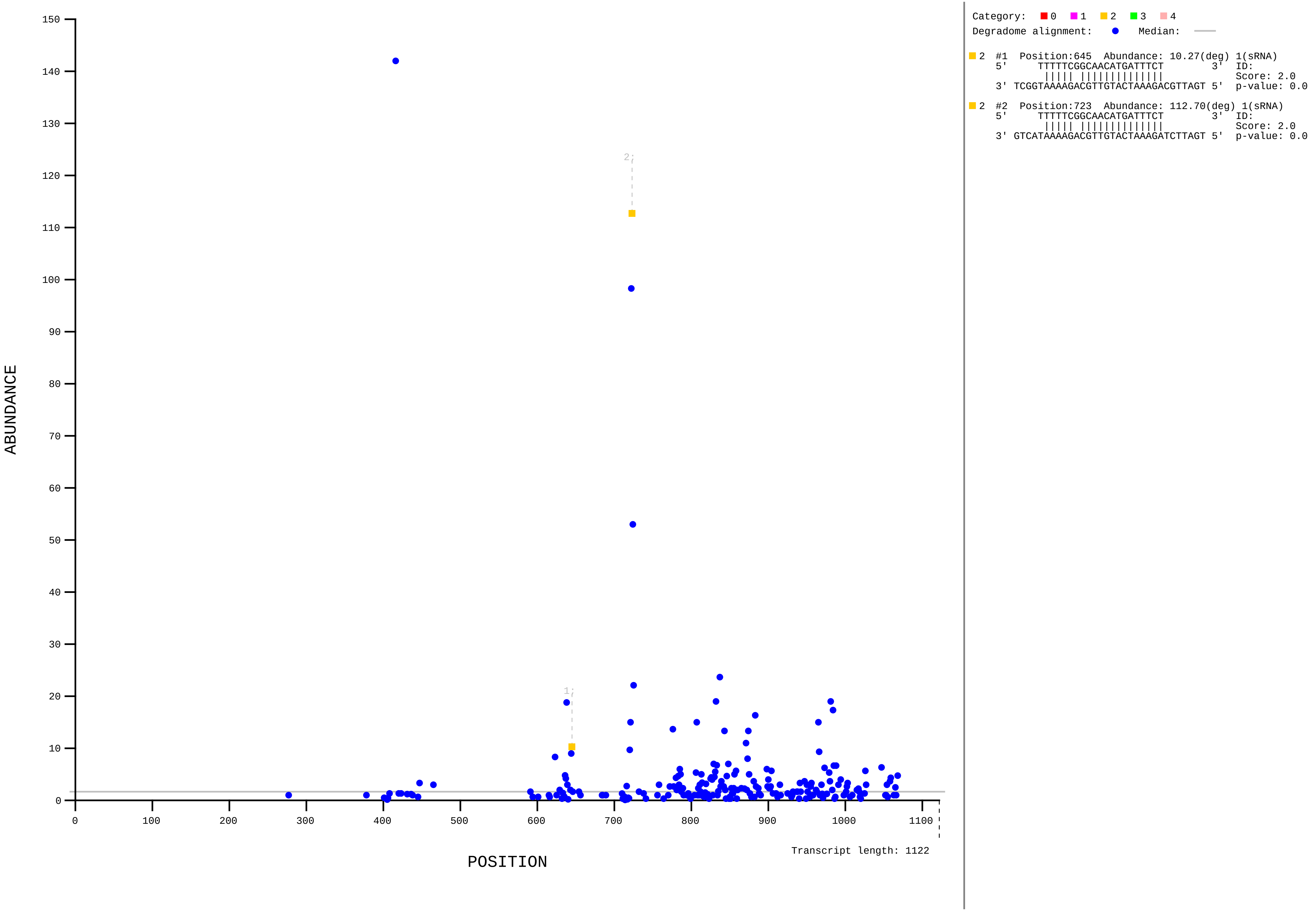

Cs1g09600.1 gene=Cs1g09600 CDS=10-240

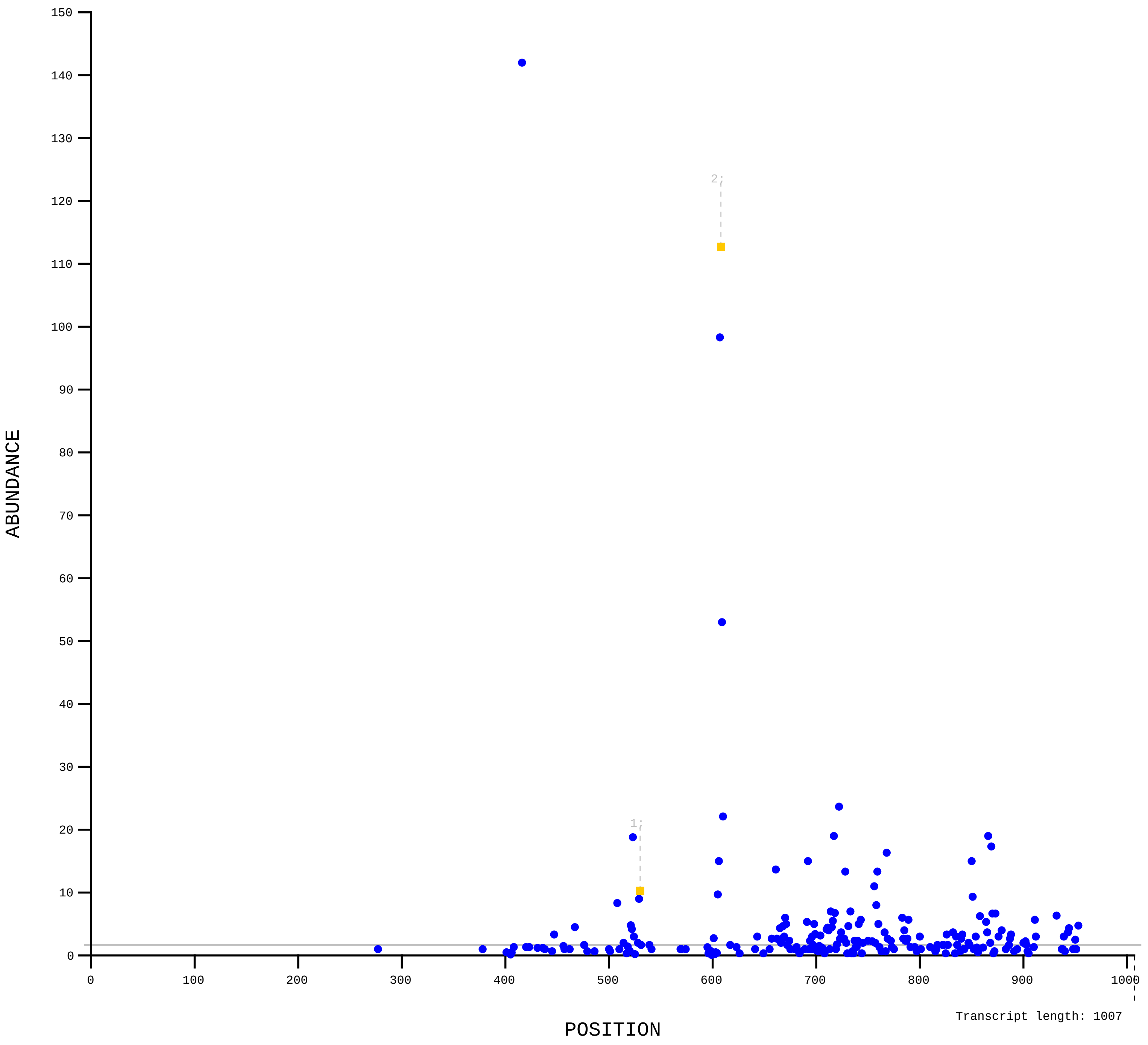


```
Category:  0  1  2  3  4
Degradome alignment:     Median:

2  #1  Position:530  Abundance: 10.27(deg) 1(sRNA)
   5'     TTTTTCGGCAACATGATTTCT        3'  ID:
           ||||| ||||||||||||||            Score: 2.0
   3' TCGGTAAAAGACGTTGTACTAAAGACGTTAGT 5'  p-value: 0.01

2  #2  Position:608  Abundance: 112.70(deg) 1(sRNA)
   5'     TTTTTCGGCAACATGATTTCT        3'  ID:
           ||||| ||||||||||||||            Score: 2.0
   3' GTCATAAAAGACGTTGTACTAAAGATCTTAGT 5'  p-value: 0.01
```

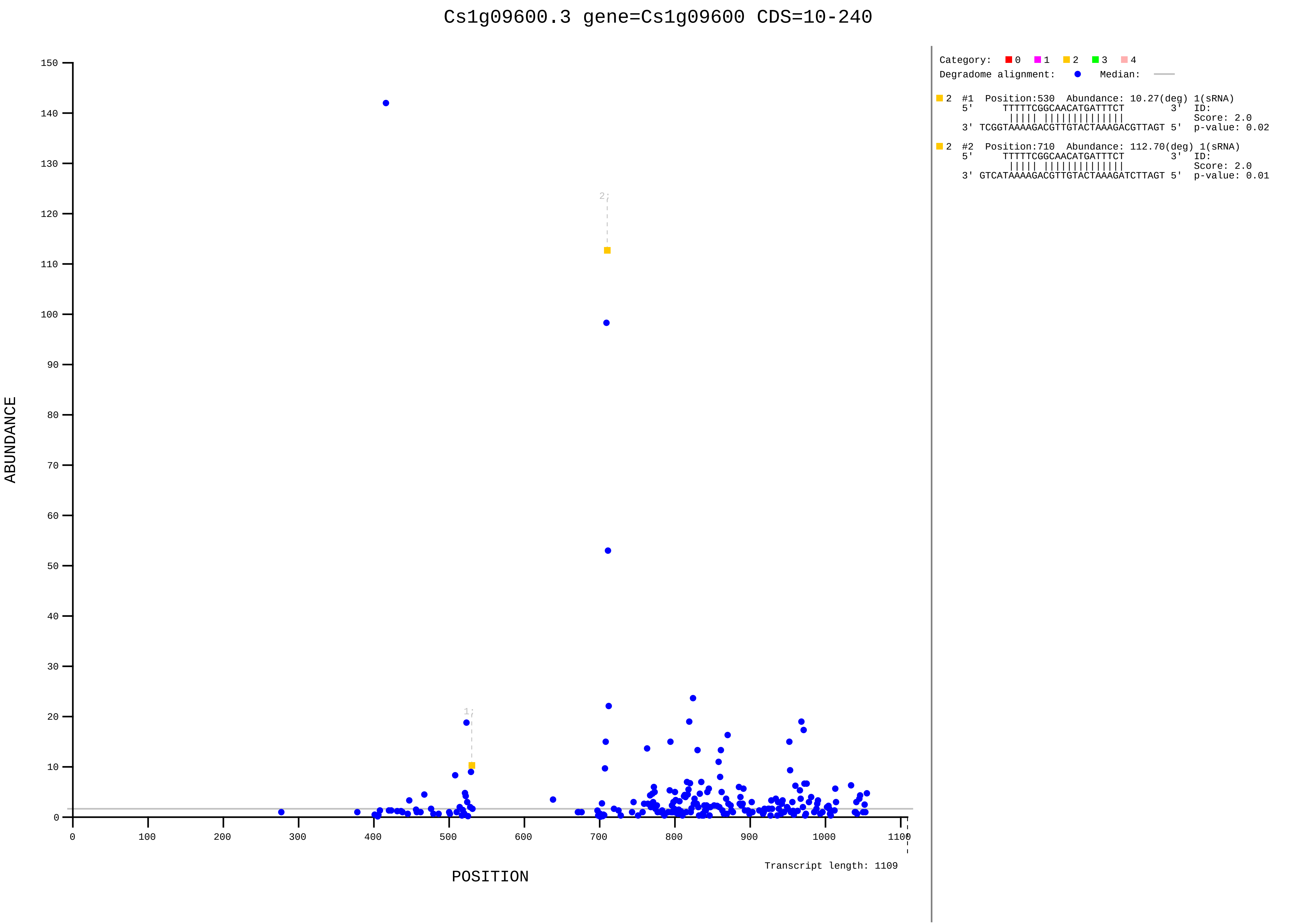

Cs1g09600.3 gene=Cs1g09600 CDS=10-240
ABUNDANCE
POSITION
Transcript length: 1109
Category: 0 1 2 3 4
Degradome alignment: Median:
2 #1 Position:530 Abundance: 10.27(deg) 1(sRNA)
5' TTTTTCGGCAACATGATTTCT 3' ID:
||||| ||||||||||||||| Score: 2.0
3' TCGGTAAAAGACGTTGTACTAAAGACGTTAGT 5' p-value: 0.02
2 #2 Position:710 Abundance: 112.70(deg) 1(sRNA)
5' TTTTTCGGCAACATGATTTCT 3' ID:
||||| ||||||||||||||| Score: 2.0
3' GTCATAAAAGACGTTGTACTAAAGATCTTAGT 5' p-value: 0.01

Cs5g32500.1 gene=Cs5g32500 CDS=368-2125

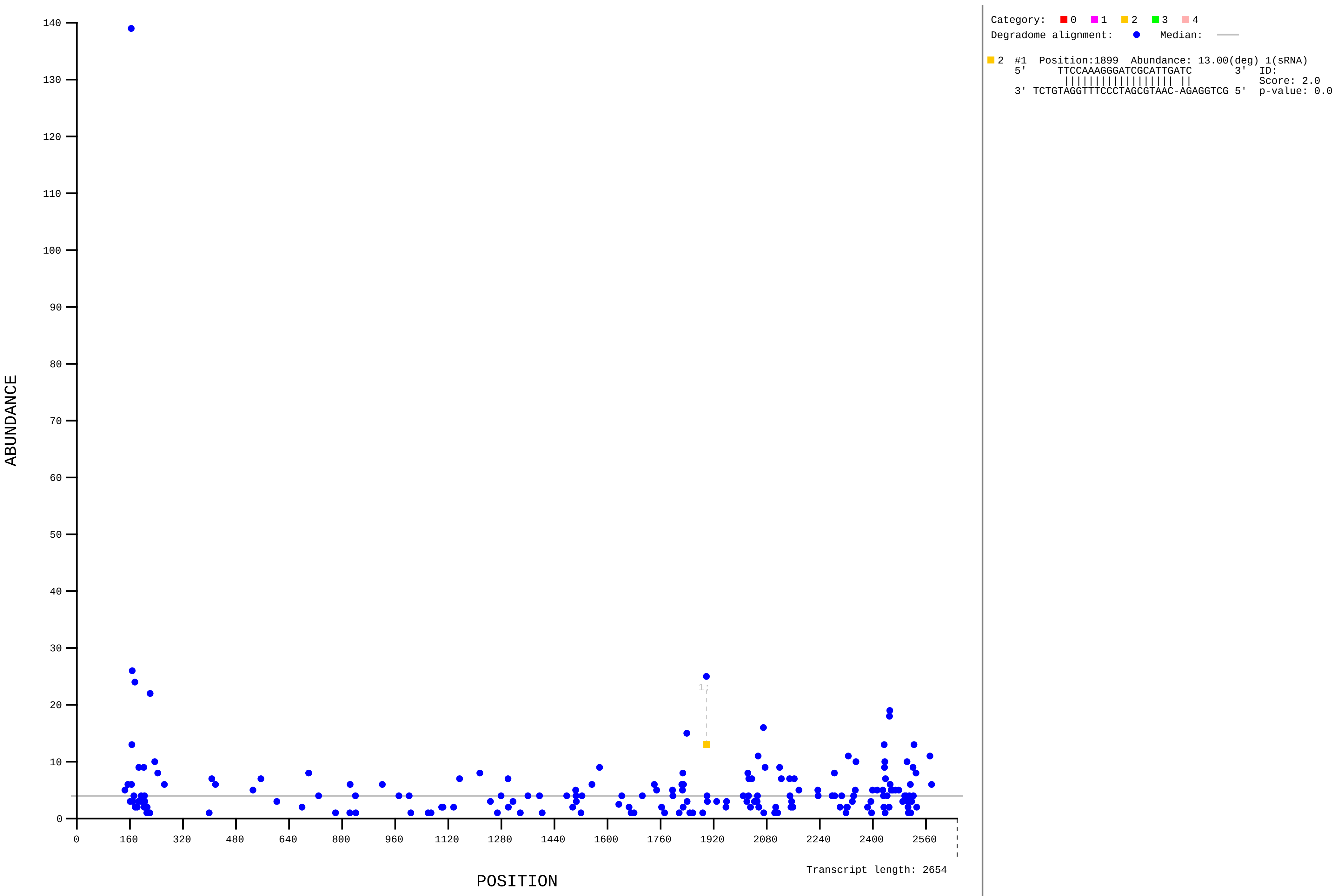

Cs2g14270.1 gene=Cs2g14270 CDS=985-2697

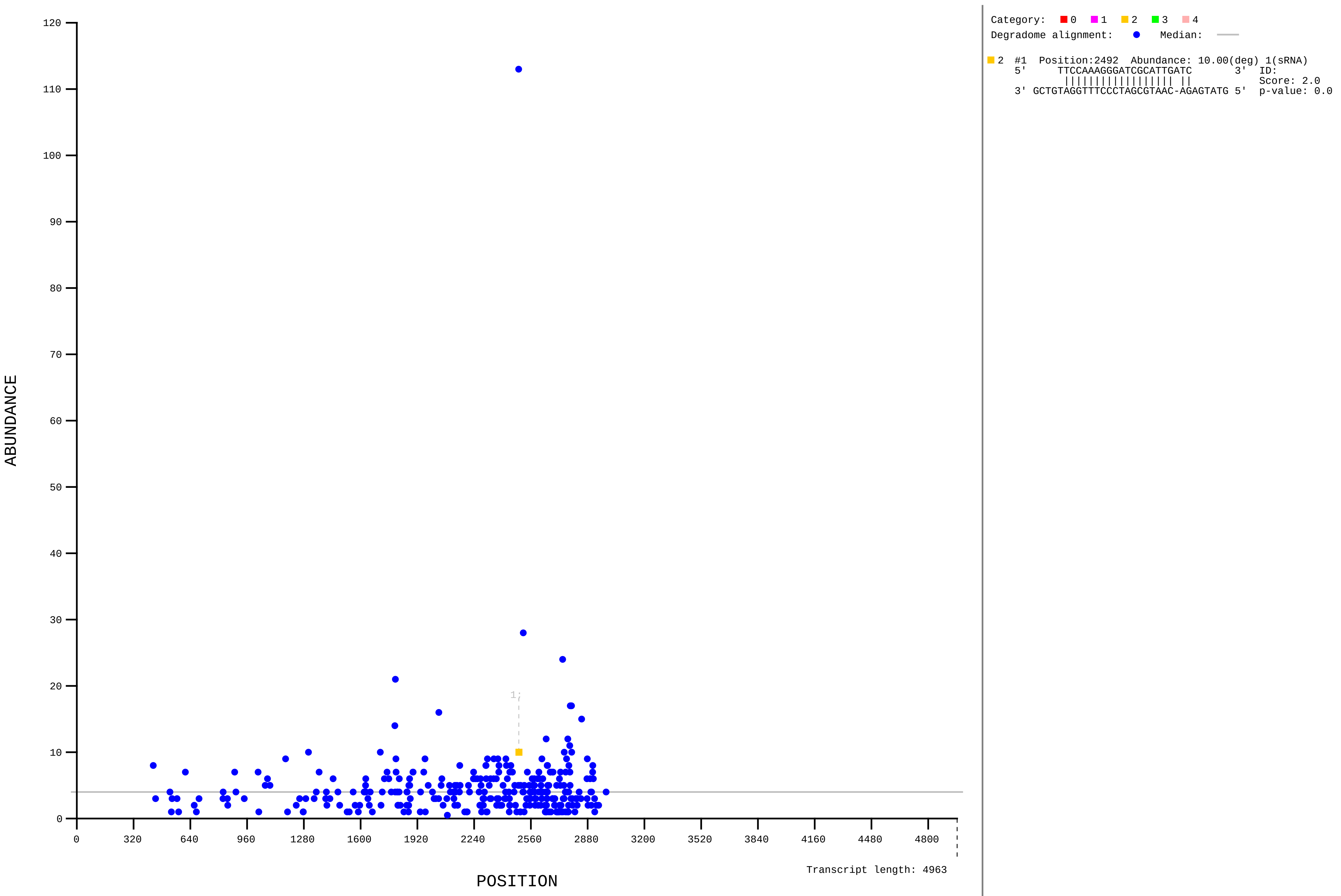

Cs8g13286.2 gene=Cs8g13286 CDS=183-310

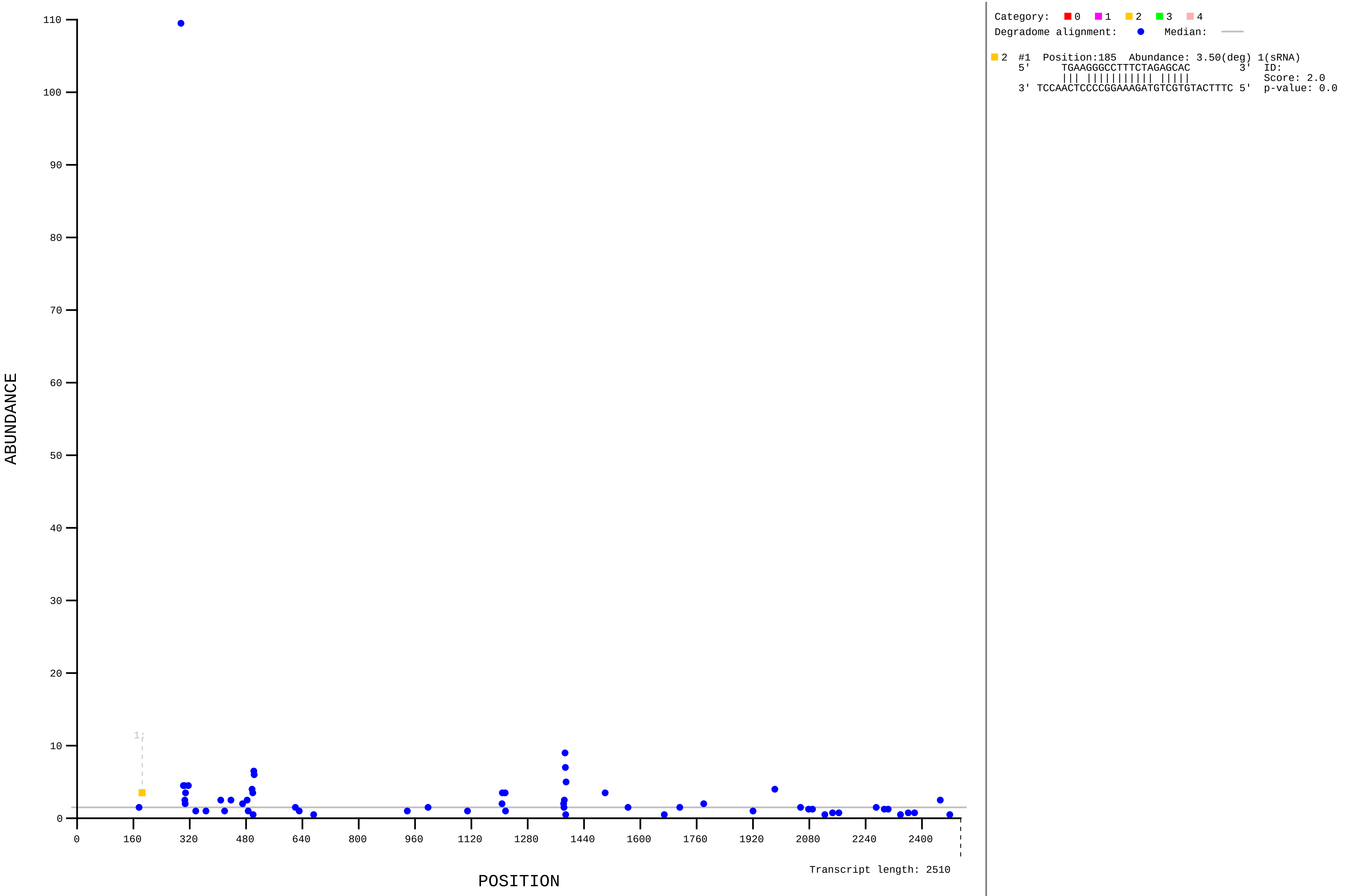

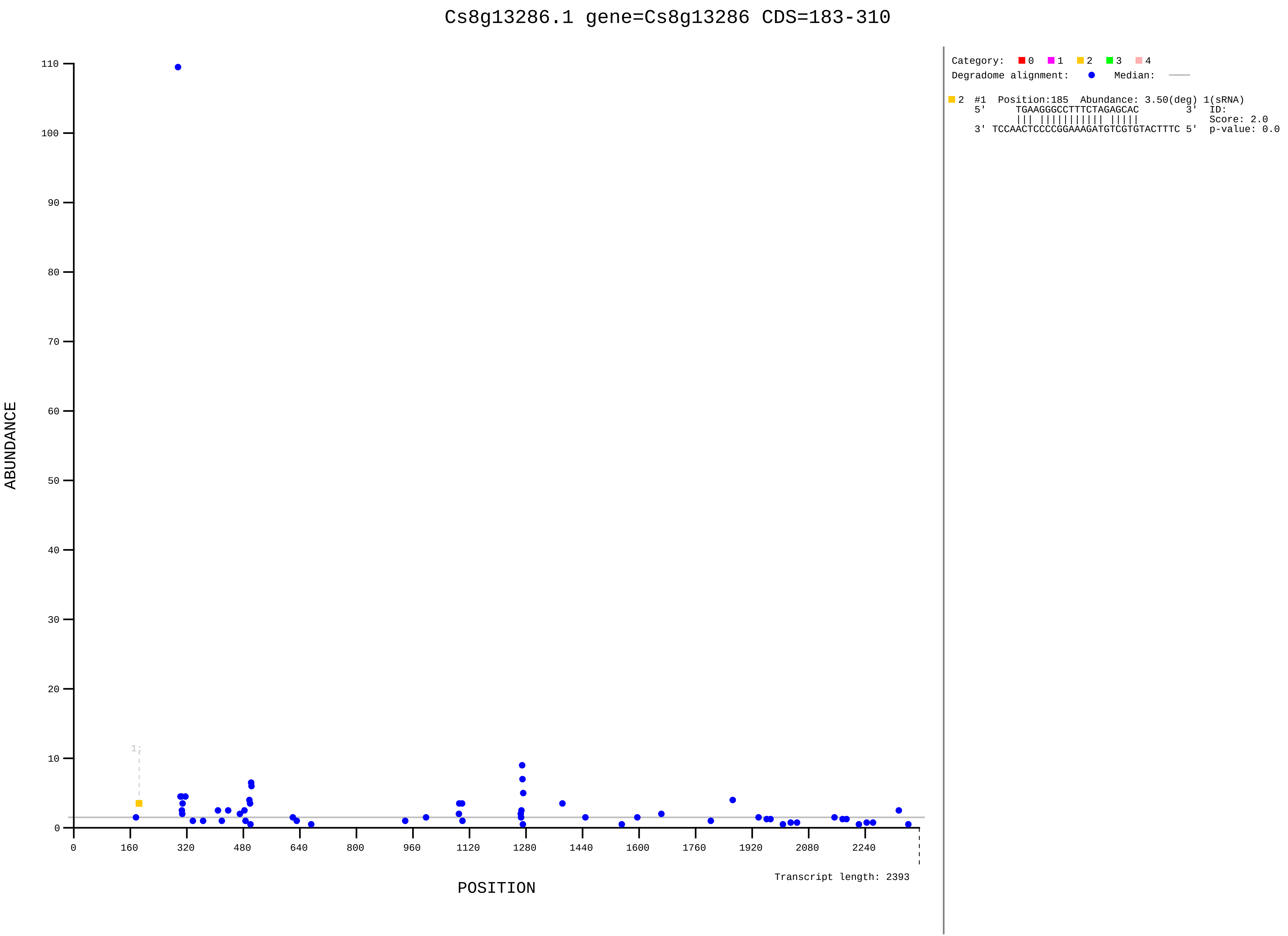

Cs8g13286.1 gene=Cs8g13286 CDS=183-310
ABUNDANCE
POSITION
Transcript length: 2393
0
10
20
30
40
50
60
70
80
90
100
110
0
160
320
480
640
800
960
1120
1280
1440
1600
1760
1920
2080
2240
1
Category: 0 1 2 3 4
Degradome alignment: Median:
2 #1 Position:185 Abundance: 3.50(deg) 1(sRNA)
5' TGAAGGGCCTTTCTAGAGCAC 3' ID:
||| ||||||||||| |||||
Score: 2.0
3' TCCAACTCCCCGGAAAGATGTCGTGTACTTTC 5' p-value: 0.0

Cs6g21580.1 gene=Cs6g21580 CDS=186-1454

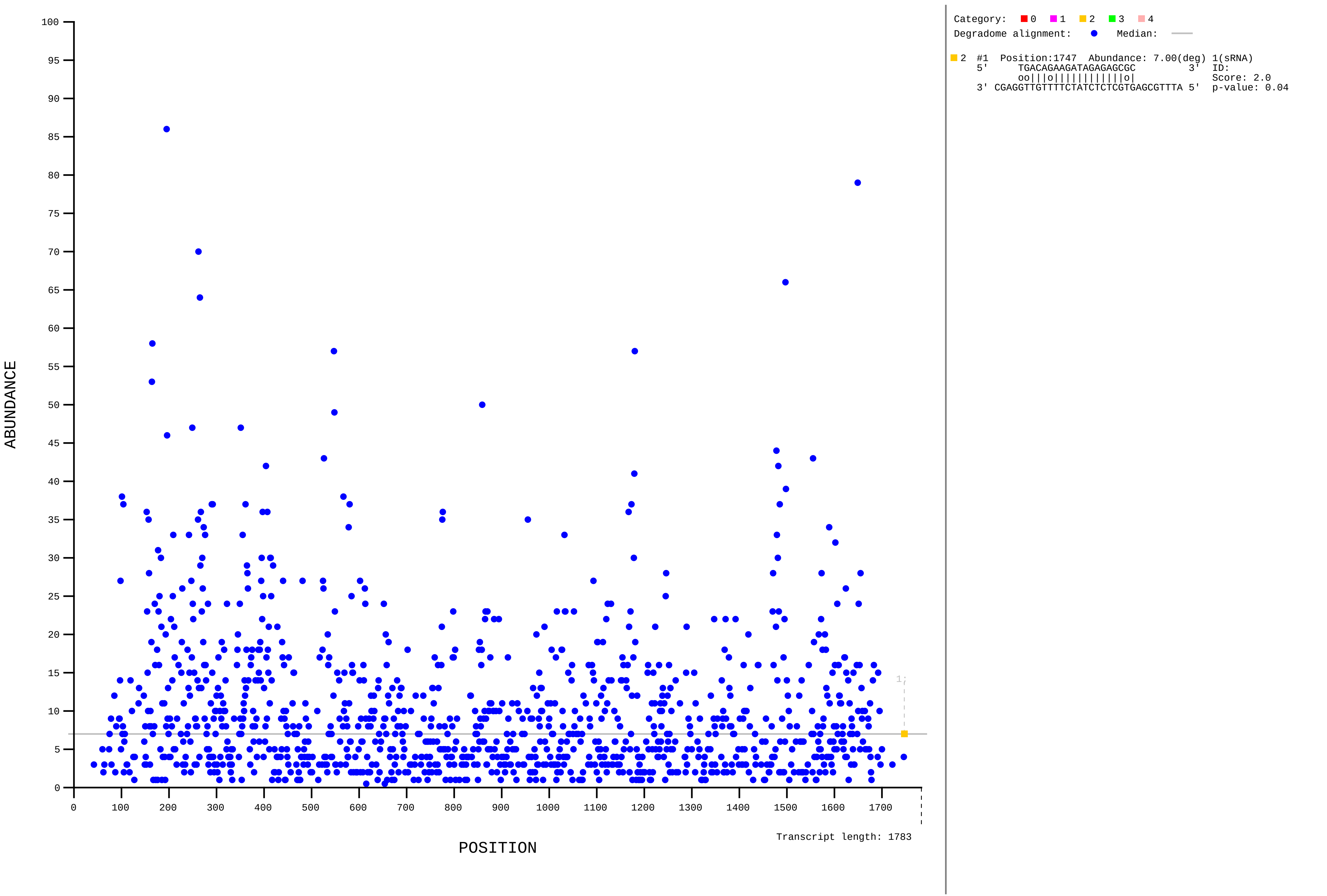

Cs3g18940.1 gene=Cs3g18940 CDS=177-1901

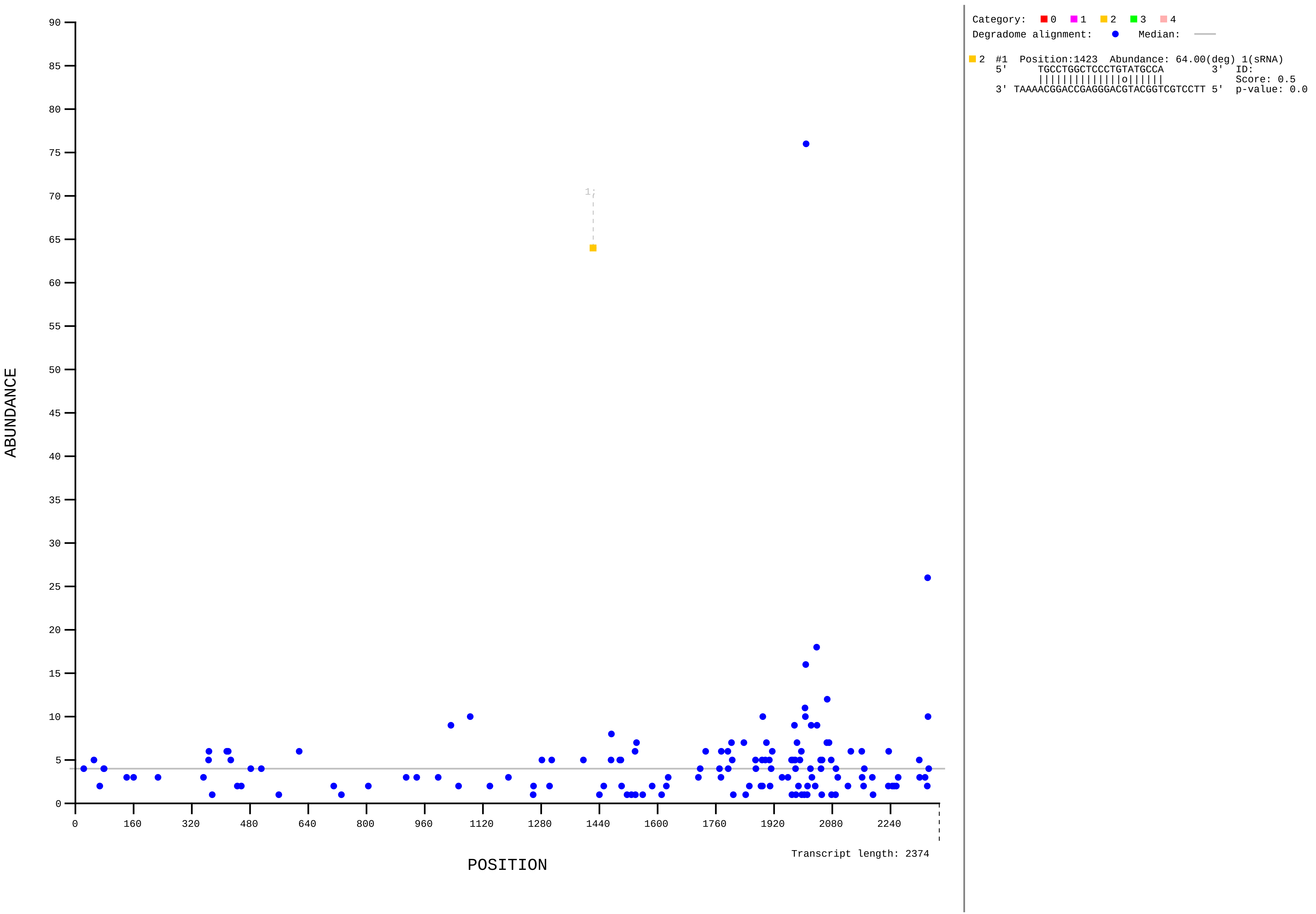

Cs2g14510.1 gene=Cs2g14510 CDS=164-2533

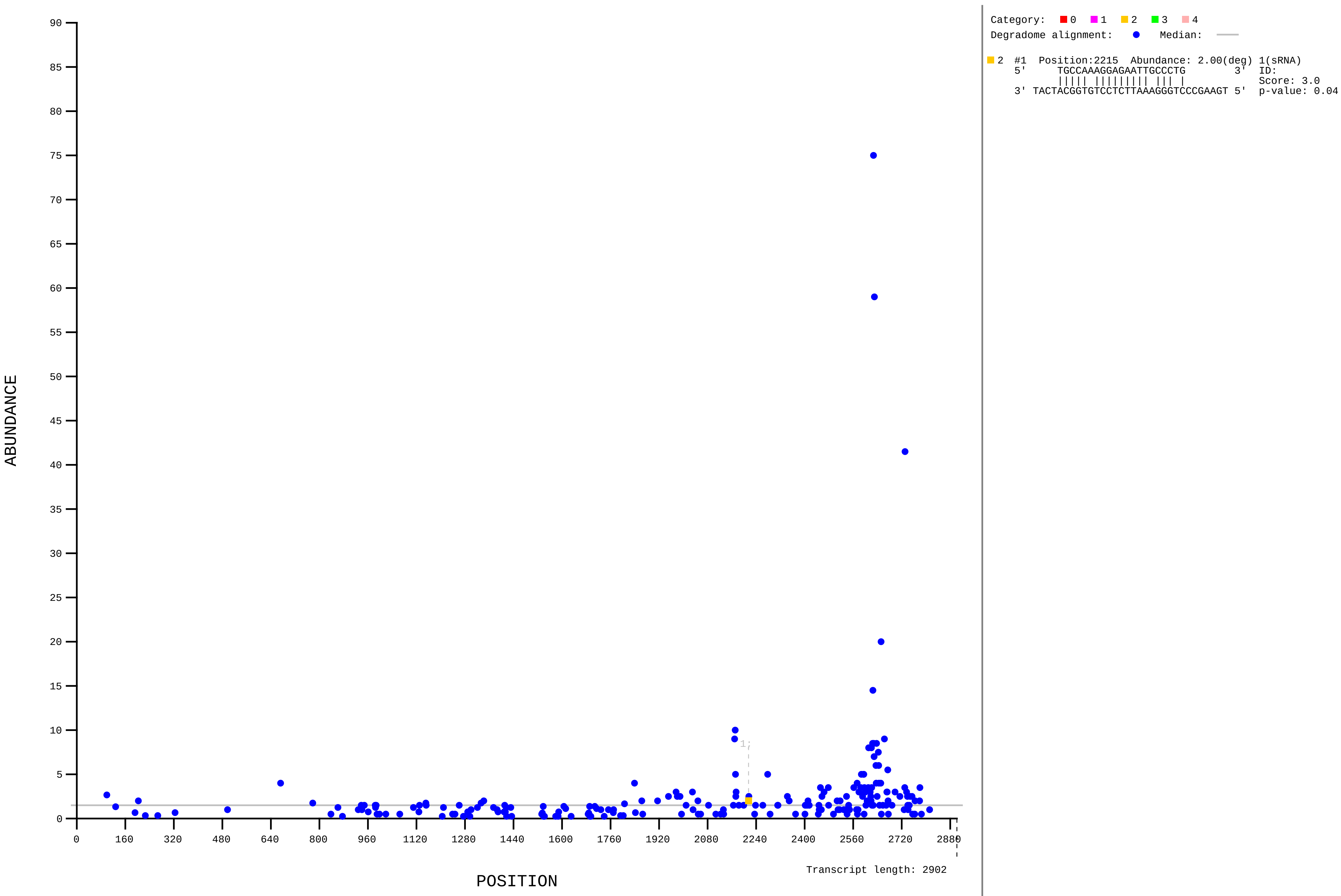

Cs2g20660.1 gene=Cs2g20660 CDS=96-2141

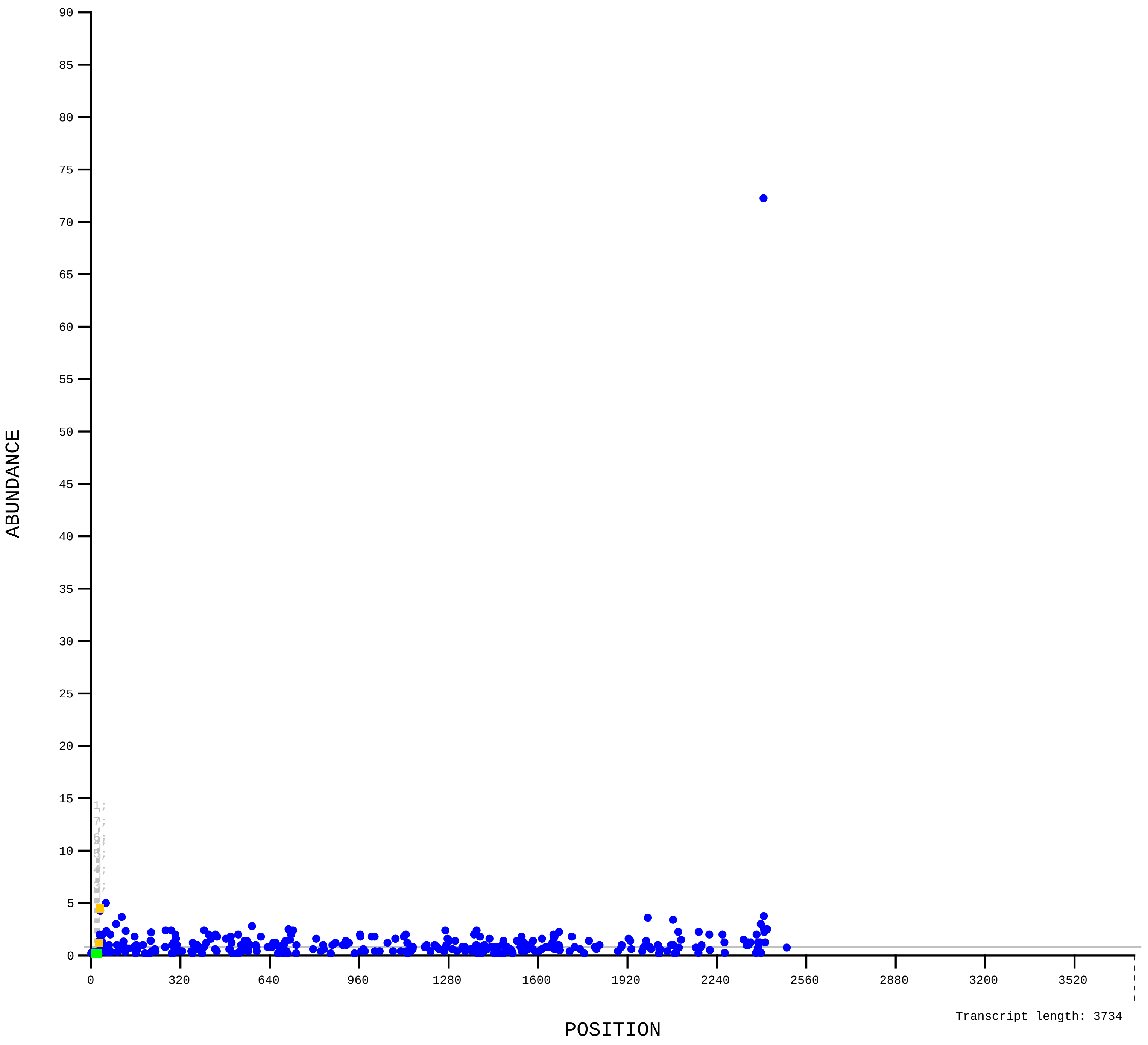


```
Category:  ■0  ■1  ■2  ■3  ■4
Degradome alignment:  ●    Median: ——

■ 2  #1  Position:29  Abundance: 1.22(deg) 1(sRNA)
    5'     AAGACGAAGAAGAAGAAGAAGAA      3'  ID:
           |||| ||||||||||||||||||          Score: 1.0
    3' CTTCTTCTTCTTCTTCTTCTTCTTCTTCTTCT 5'  p-value: 0.01

■ 2  #2  Position:32  Abundance: 4.50(deg) 1(sRNA)
    5'     AAGACGAAGAAGAAGAAGAAGAA      3'  ID:
           |||| ||||||||||||||||||          Score: 1.0
    3' CTTCTTCTTCTTCTTCTTCTTCTTCTTCTTCT 5'  p-value: 0.0

■ 3  #3  Position:14  Abundance: 0.19(deg) 1(sRNA)
    5'     AAGACGAAGAAGAAGAAGAAGAA      3'  ID:
           |||| ||||||||||||||||||          Score: 1.0
    3' CTTCTTCTTCTTCTTCTTCTTCTTCTT      5'  p-value: 0.02

■ 3  #4  Position:17  Abundance: 0.19(deg) 1(sRNA)
    5'     AAGACGAAGAAGAAGAAGAAGAA      3'  ID:
           |||| ||||||||||||||||||          Score: 1.0
    3' CTTCTTCTTCTTCTTCTTCTTCTTCTTCTT   5'  p-value: 0.0

■ 3  #5  Position:20  Abundance: 0.19(deg) 1(sRNA)
    5'     AAGACGAAGAAGAAGAAGAAGAA      3'  ID:
           |||| ||||||||||||||||||          Score: 1.0
    3' CTTCTTCTTCTTCTTCTTCTTCTTCTTCTTCT 5'  p-value: 0.0

■ 3  #6  Position:23  Abundance: 0.19(deg) 1(sRNA)
    5'     AAGACGAAGAAGAAGAAGAAGAA      3'  ID:
           |||| ||||||||||||||||||          Score: 1.0
    3' CTTCTTCTTCTTCTTCTTCTTCTTCTTCTTCT 5'  p-value: 0.0

■ 3  #7  Position:26  Abundance: 0.19(deg) 1(sRNA)
    5'     AAGACGAAGAAGAAGAAGAAGAA      3'  ID:
           |||| ||||||||||||||||||          Score: 1.0
    3' CTTCTTCTTCTTCTTCTTCTTCTTCTTCTTCT 5'  p-value: 0.0
```

Cs2g20660.4 gene=Cs2g20660 CDS=96-2132

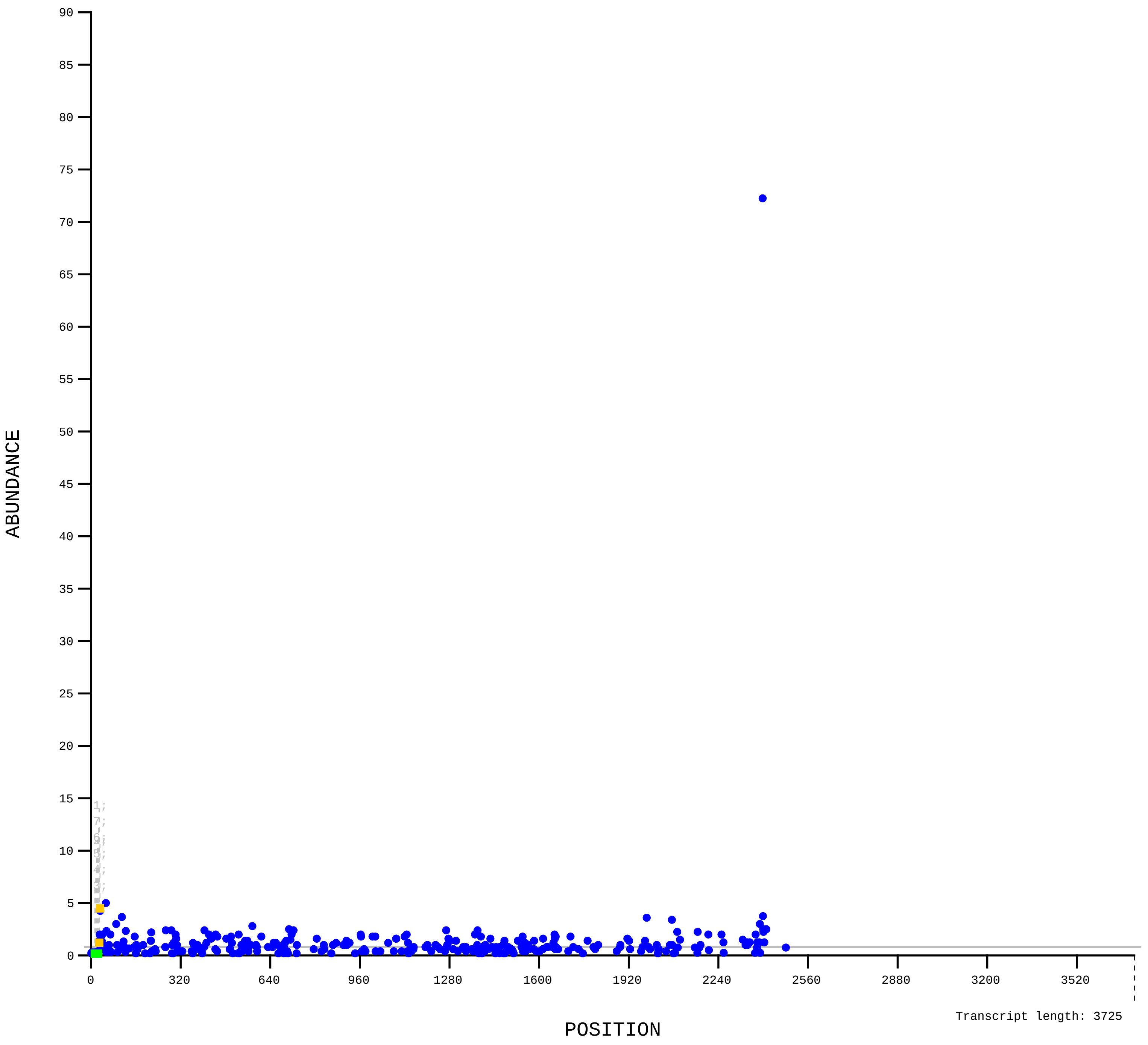


```
Category: 0 1 2 3 4
Degradome alignment:      Median:

2  #1  Position:29  Abundance: 1.22(deg) 1(sRNA)
   5'     AAGACGAAGAAGAAGAAGAAGAA      3'  ID:
          |||| ||||||||||||||||||          Score: 1.0
   3' CTTCTTCTTCTTCTTCTTCTTCTTCTTCTTCT 5'  p-value: 0.01

2  #2  Position:32  Abundance: 4.50(deg) 1(sRNA)
   5'     AAGACGAAGAAGAAGAAGAAGAA      3'  ID:
          |||| ||||||||||||||||||          Score: 1.0
   3' CTTCTTCTTCTTCTTCTTCTTCTTCTTCTTCT 5'  p-value: 0.02

3  #3  Position:14  Abundance: 0.19(deg) 1(sRNA)
   5'     AAGACGAAGAAGAAGAAGAAGAA      3'  ID:
          |||| ||||||||||||||||||          Score: 1.0
   3' CTTCTTCTTCTTCTTCTTCTTCTTCT      5'  p-value: 0.0

3  #4  Position:17  Abundance: 0.19(deg) 1(sRNA)
   5'     AAGACGAAGAAGAAGAAGAAGAA      3'  ID:
          |||| ||||||||||||||||||          Score: 1.0
   3' CTTCTTCTTCTTCTTCTTCTTCTTCTTCTT   5'  p-value: 0.0

3  #5  Position:20  Abundance: 0.19(deg) 1(sRNA)
   5'     AAGACGAAGAAGAAGAAGAAGAA      3'  ID:
          |||| ||||||||||||||||||          Score: 1.0
   3' CTTCTTCTTCTTCTTCTTCTTCTTCTTCTTCT 5'  p-value: 0.0

3  #6  Position:23  Abundance: 0.19(deg) 1(sRNA)
   5'     AAGACGAAGAAGAAGAAGAAGAA      3'  ID:
          |||| ||||||||||||||||||          Score: 1.0
   3' CTTCTTCTTCTTCTTCTTCTTCTTCTTCTTCT 5'  p-value: 0.01

3  #7  Position:26  Abundance: 0.19(deg) 1(sRNA)
   5'     AAGACGAAGAAGAAGAAGAAGAA      3'  ID:
          |||| ||||||||||||||||||          Score: 1.0
   3' CTTCTTCTTCTTCTTCTTCTTCTTCTTCTTCT 5'  p-value: 0.02
```

Cs3g13340.1 gene=Cs3g13340 CDS=60-2933

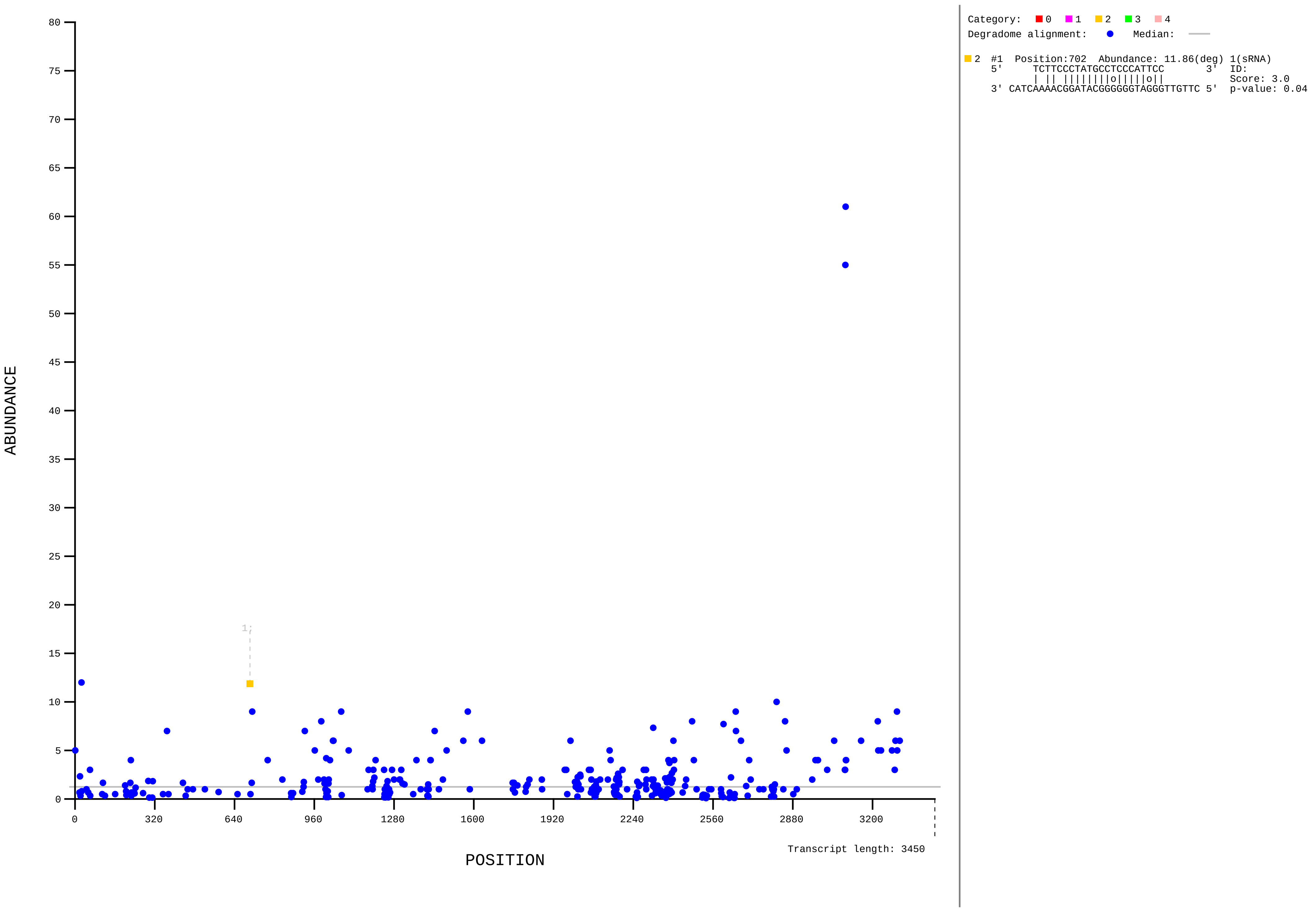

orange1.1t05093.1 gene=orange1.1t05093 CDS=1-699

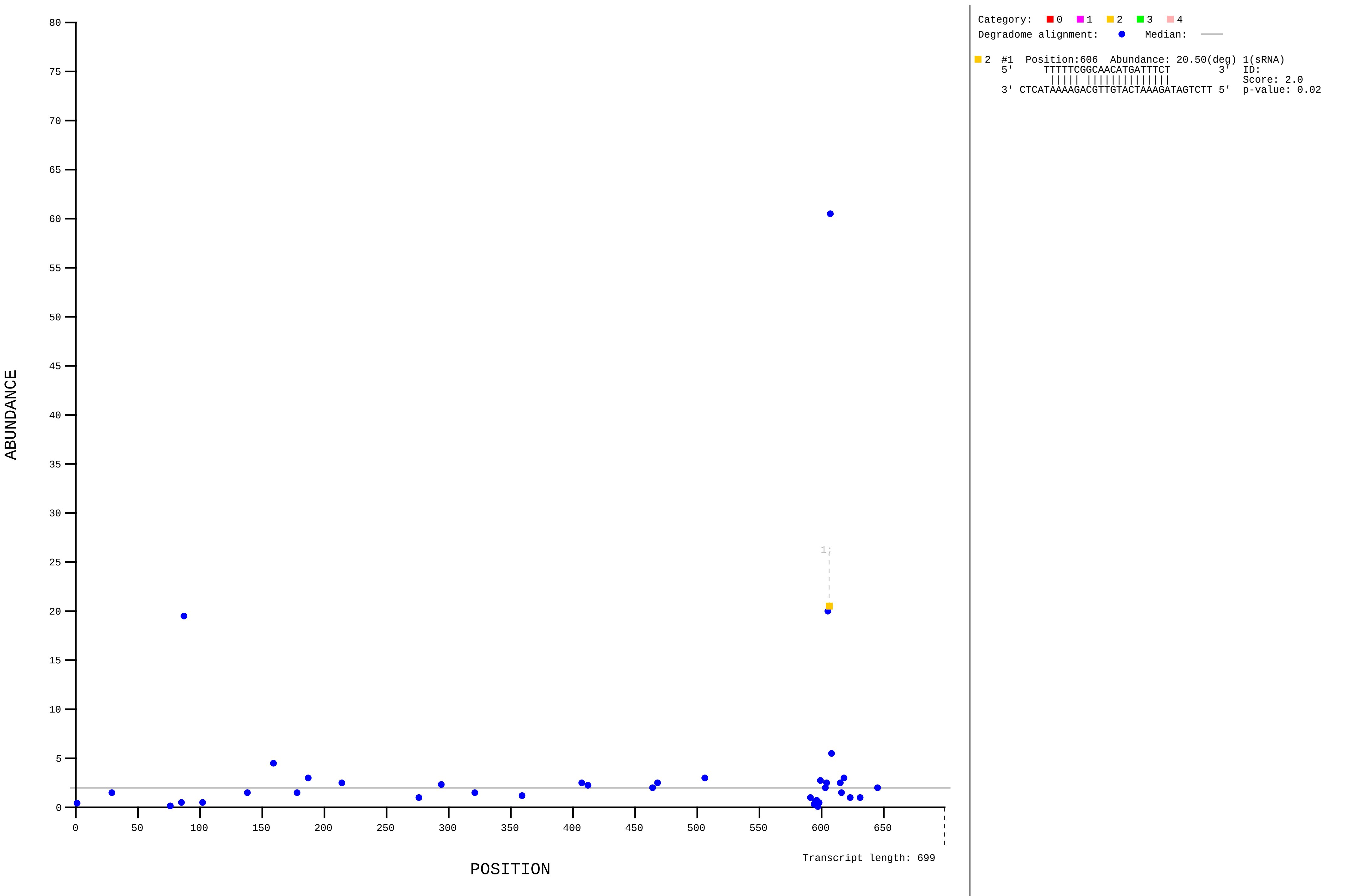

Cs6g21520.1 gene=Cs6g21520 CDS=283-1329

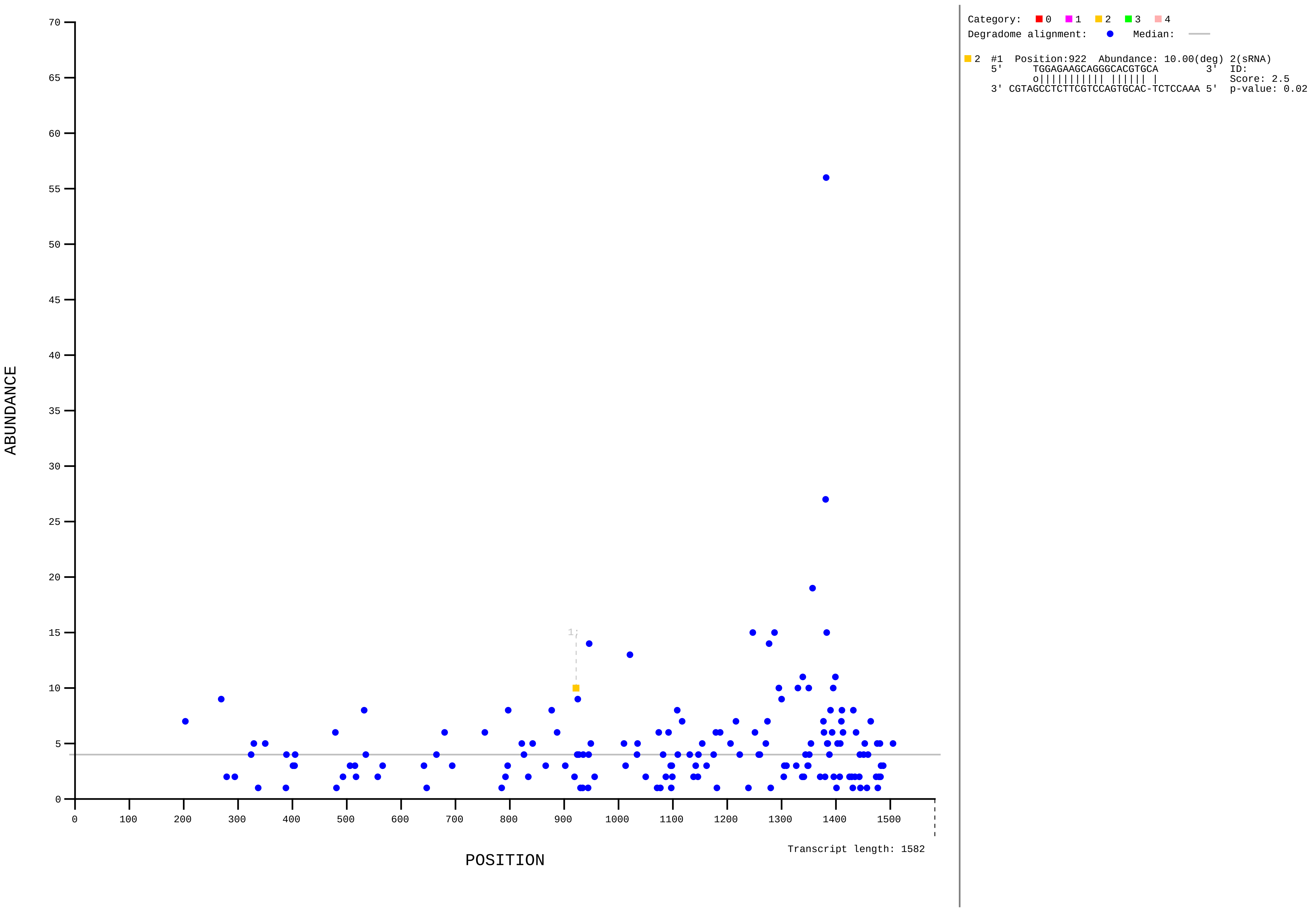

Cs3g08210.1 gene=Cs3g08210 CDS=1-4755

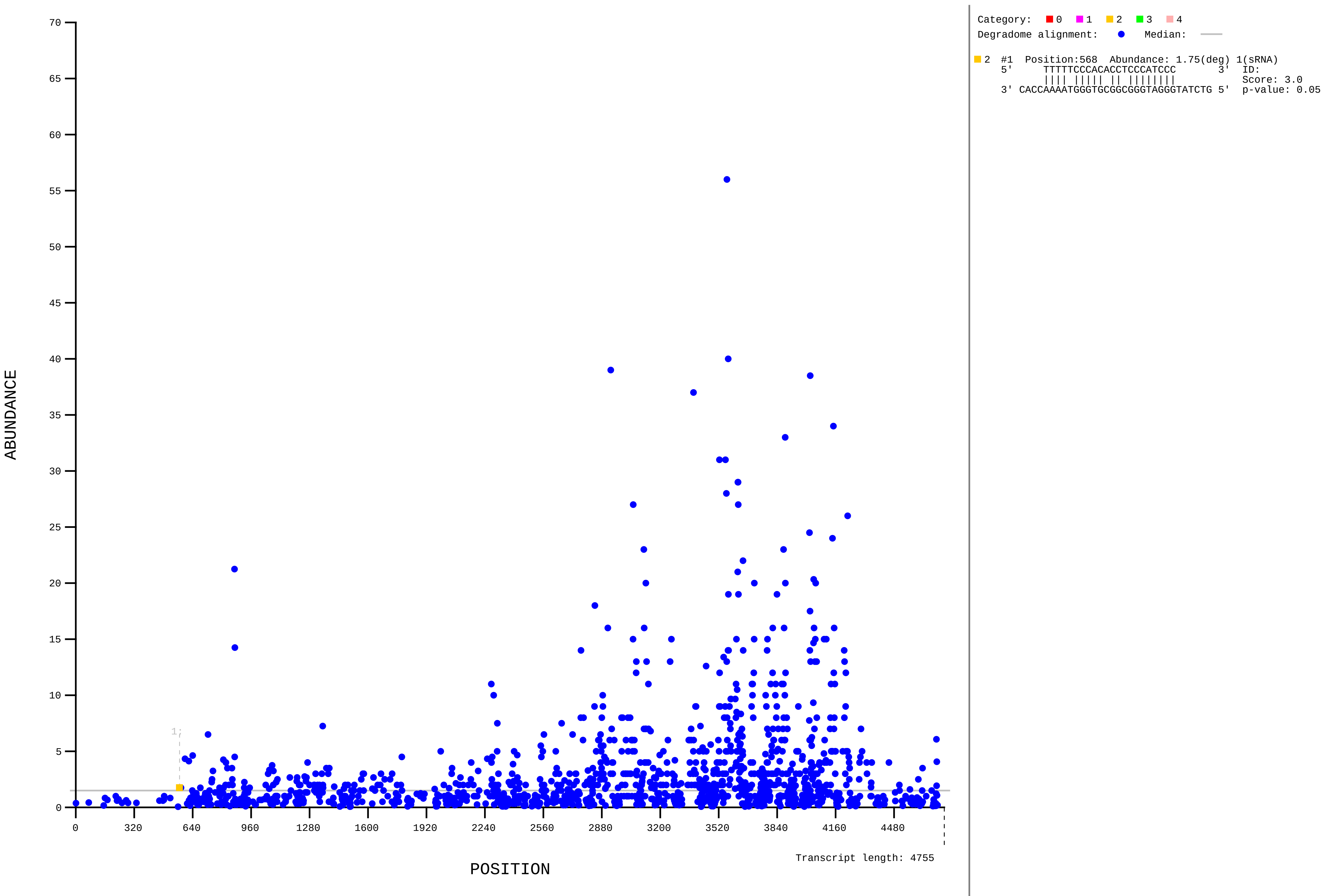

Cs9g07750.1 gene=Cs9g07750 CDS=208-1602

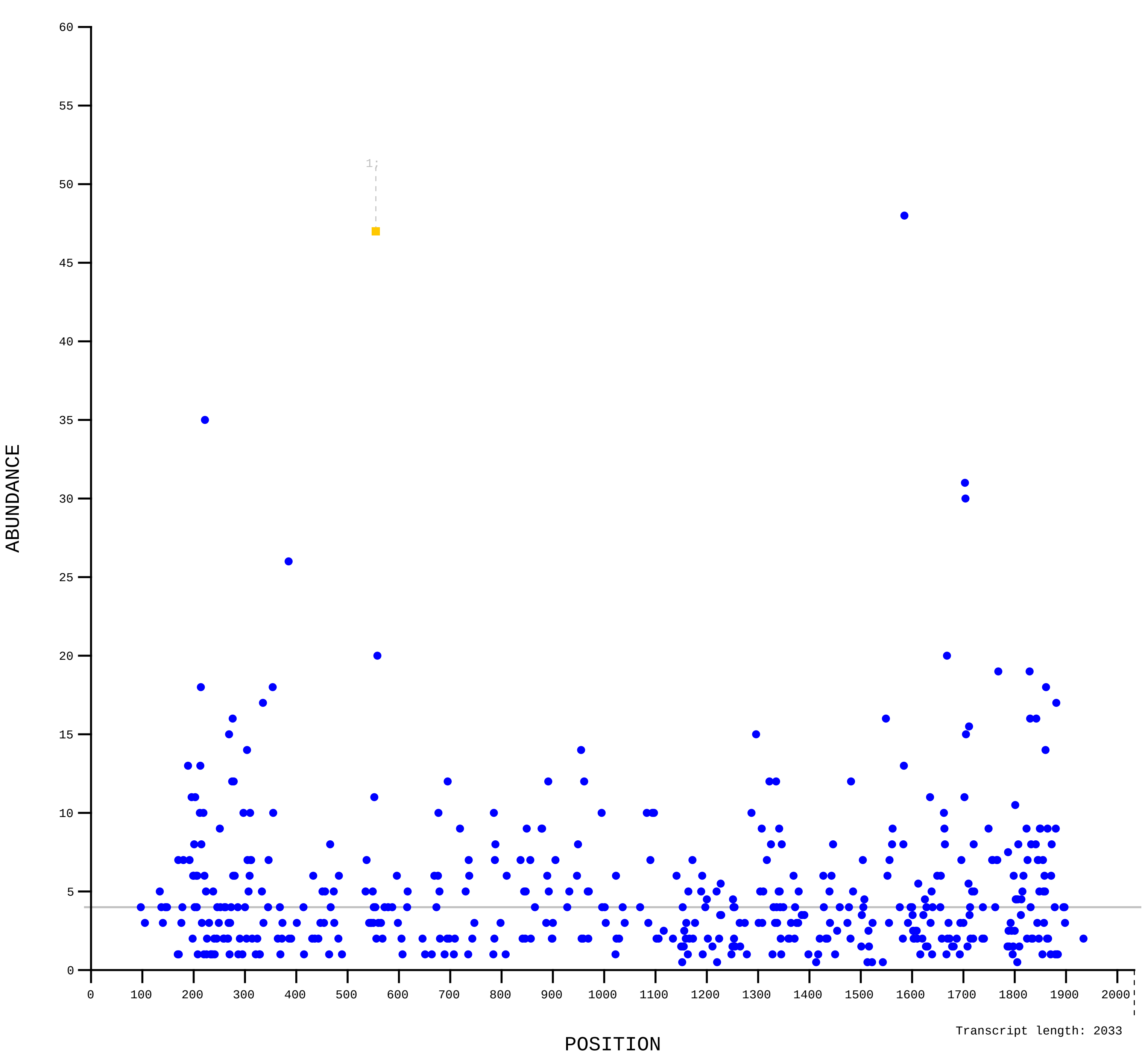


```
Category:  0  1  2  3  4
Degradome alignment:      Median:

2  #1  Position:555  Abundance: 47.00(deg) 1(sRNA)
   5'     CTGAAGTGTTTGGGGGAACTC        3'  ID:
            ||||| ||||||o|||||||           Score: 2.5
   3' ACTTTACTTCTCAAACCTCCTTGAGCCTAAGA 5'  p-value: 0.02
```

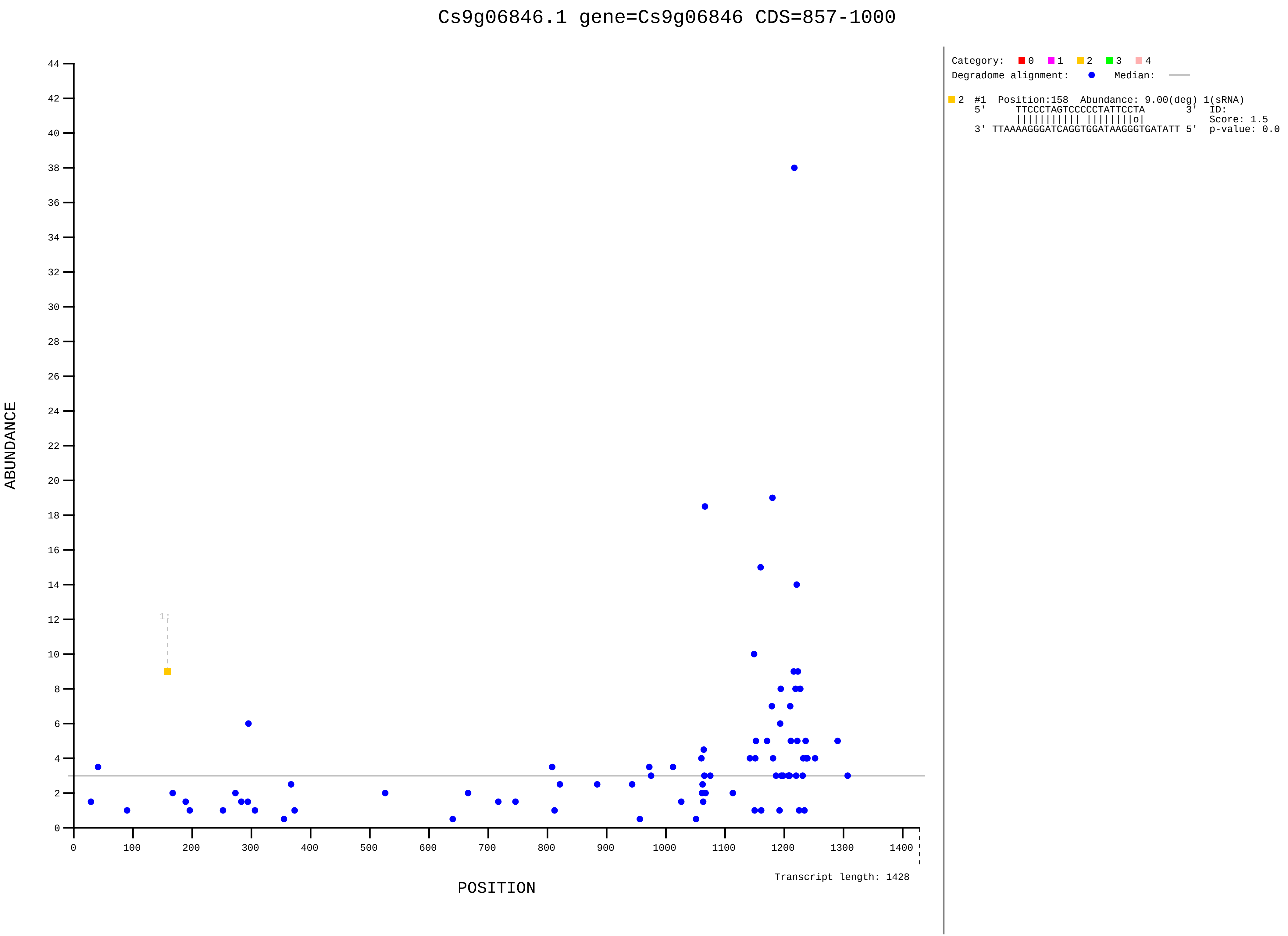
Cs9g06846.1 gene=Cs9g06846 CDS=857-1000
Category: 0 1 2 3 4
Degradome alignment:
Median:
2 #1 Position:158 Abundance: 9.00(deg) 1(sRNA)
5' TTCCCTAGTCCCCCTATTCCTA 3' ID:
||||||||||| ||||||||o|
Score: 1.5
3' TTAAAAGGGATCAGGTGGATAAGGGTGATATT 5' p-value: 0.0
ABUNDANCE
POSITION
Transcript length: 1428

Cs1g15640.1 gene=Cs1g15640 CDS=1220-3736

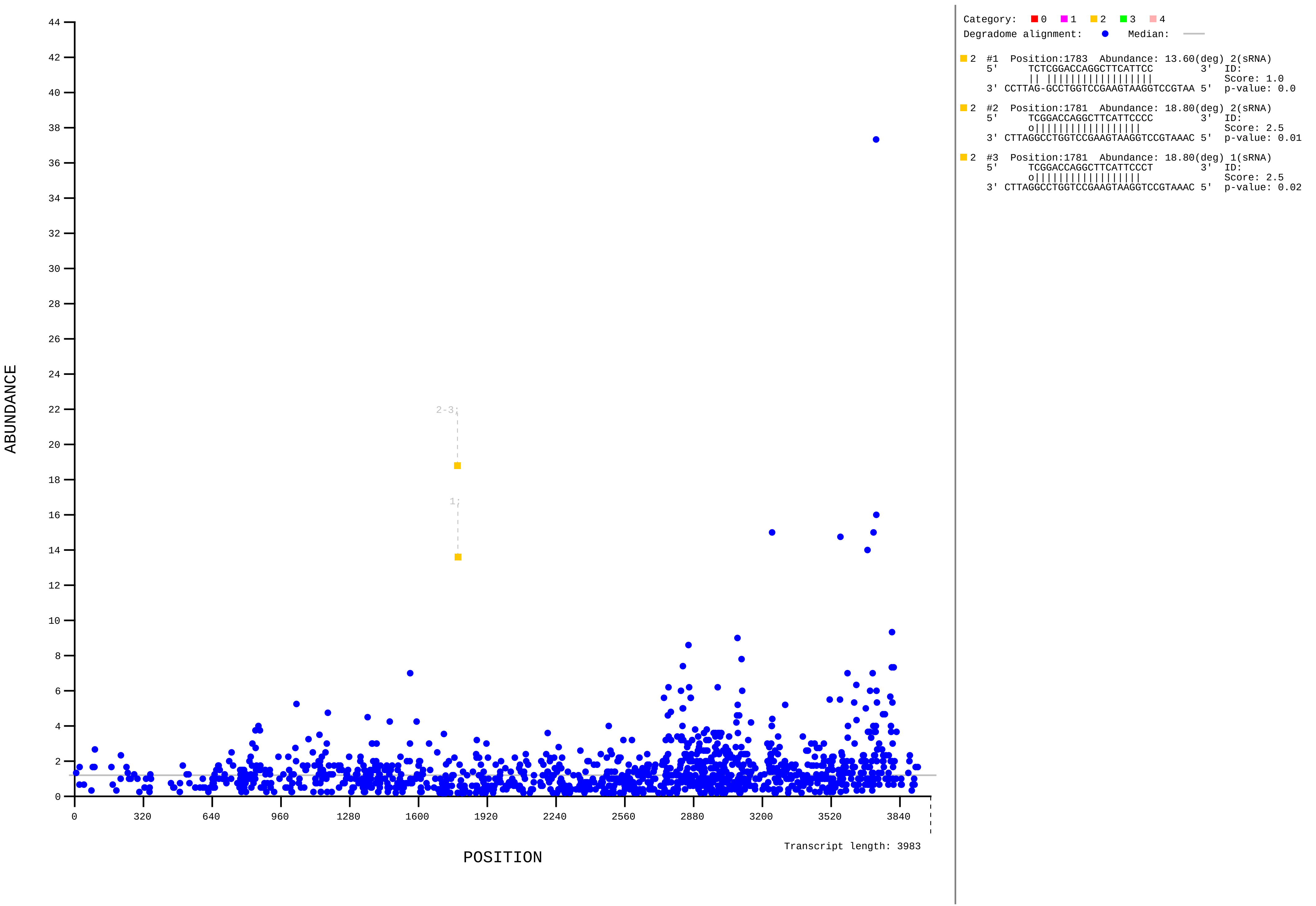

Cs1g15640.3 gene=Cs1g15640 CDS=1130-3646

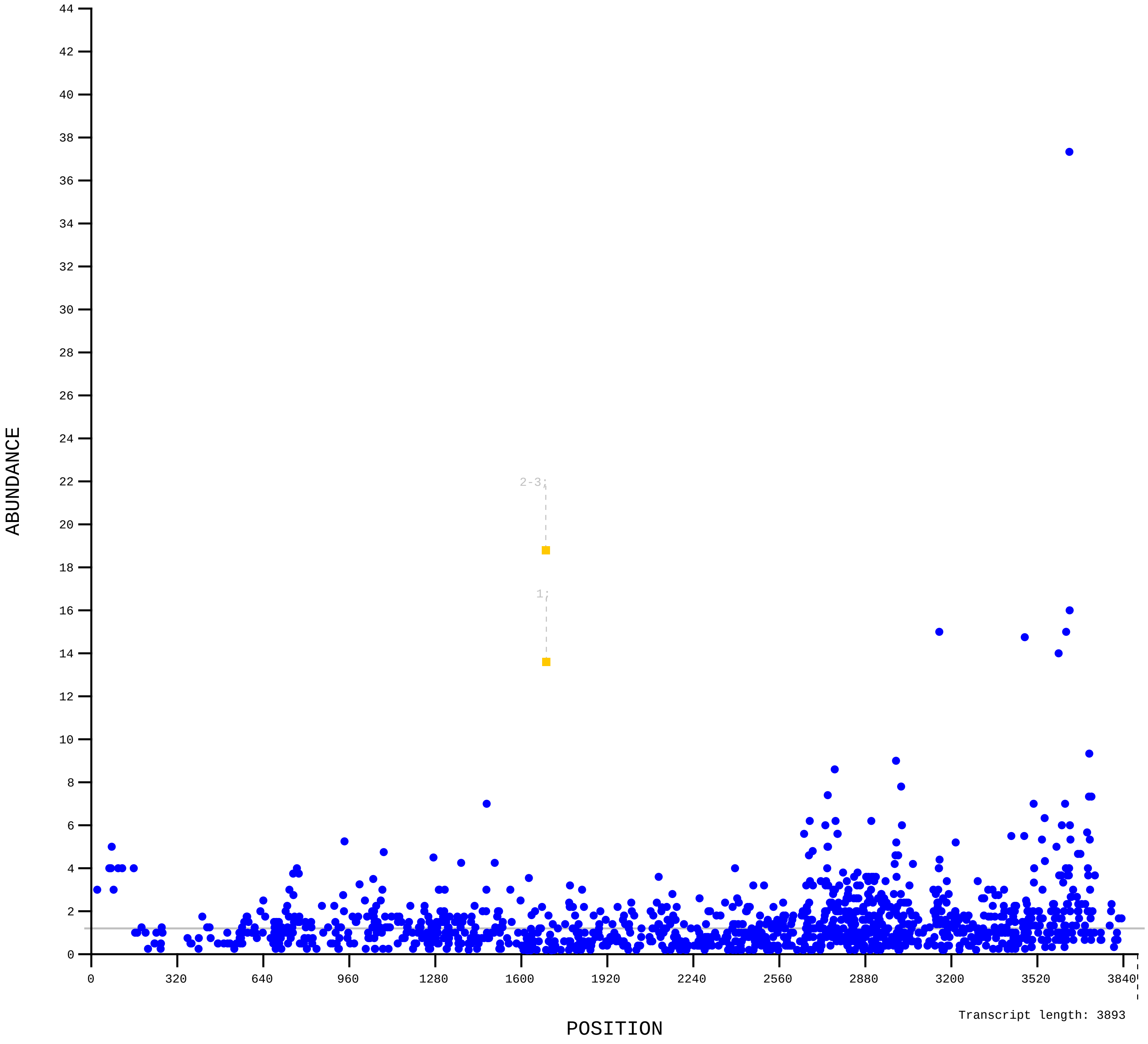

Category: 0 1 2 3 4
Degradome alignment: Median:

```
2  #1  Position:1693  Abundance: 13.60(deg) 2(sRNA)
   5'     TCTCGGACCAGGCTTCATTCC        3'  ID:
          || |||||||||||||||||             Score: 1.0
   3' CCTTAG-GCCTGGTCCGAAGTAAGGTCCGTAA 5'  p-value: 0.0

2  #2  Position:1691  Abundance: 18.80(deg) 2(sRNA)
   5'     TCGGACCAGGCTTCATTCCC        3'  ID:
          o||||||||||||||||||              Score: 2.5
   3' CTTAGGCCTGGTCCGAAGTAAGGTCCGTAAAC 5'  p-value: 0.01

2  #3  Position:1691  Abundance: 18.80(deg) 1(sRNA)
   5'     TCGGACCAGGCTTCATTCCCT        3'  ID:
          o||||||||||||||||||              Score: 2.5
   3' CTTAGGCCTGGTCCGAAGTAAGGTCCGTAAAC 5'  p-value: 0.02
```

Cs1g15640.5 gene=Cs1g15640 CDS=121-2091

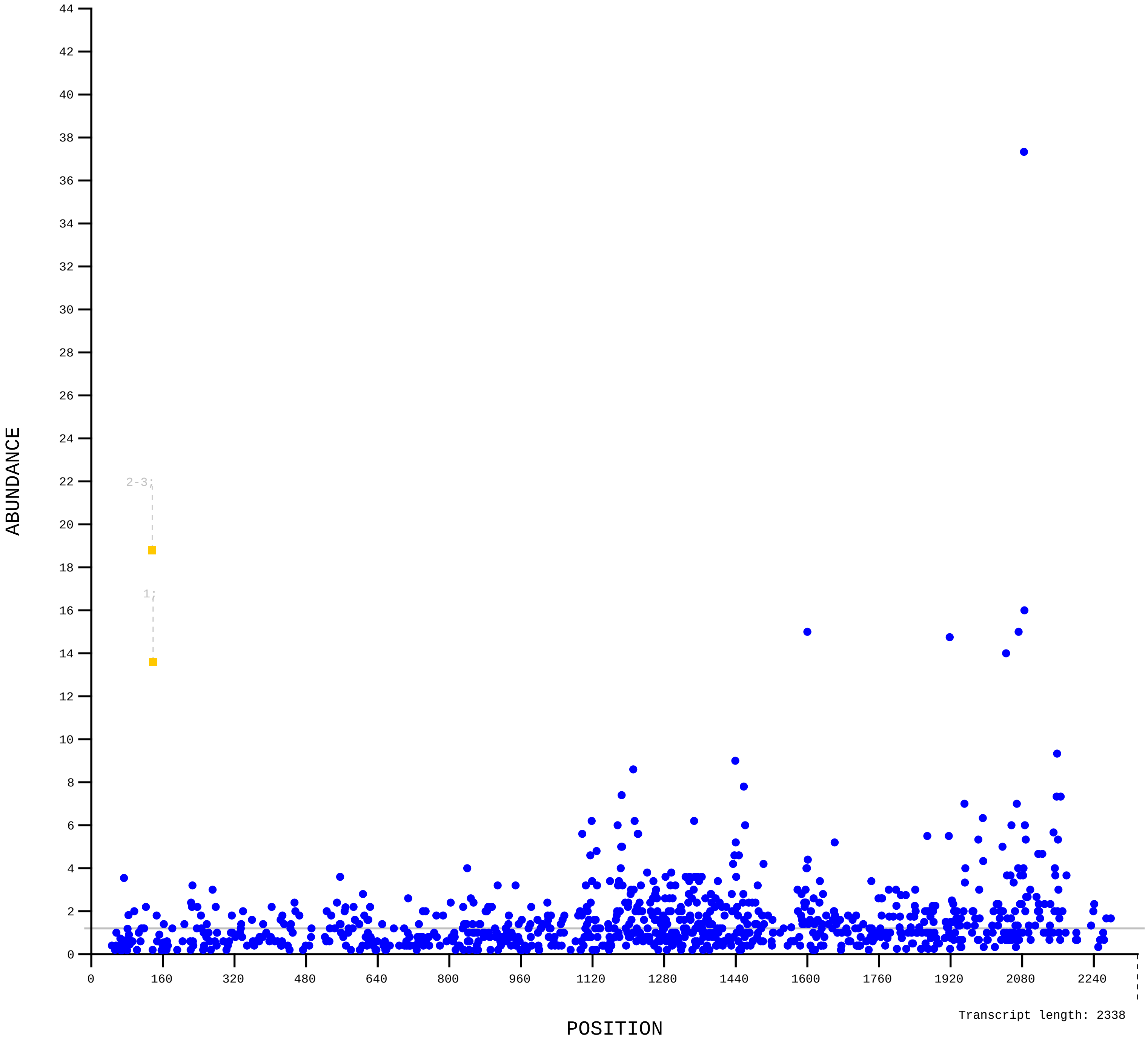

Category: 0 1 2 3 4
Degradome alignment: Median:

```
2  #1  Position:138  Abundance: 13.60(deg) 2(sRNA)
   5'     TCTCGGACCAGGCTTCATTCC        3'  ID:
          || ||||||||||||||||||            Score: 1.0
   3' CCTTAG-GCCTGGTCCGAAGTAAGGTCCGTAA 5'  p-value: 0.0

2  #2  Position:136  Abundance: 18.80(deg) 2(sRNA)
   5'     TCGGACCAGGCTTCATTCCCC        3'  ID:
          o||||||||||||||||||              Score: 2.5
   3' CTTAGGCCTGGTCCGAAGTAAGGTCCGTAAAC 5'  p-value: 0.01

2  #3  Position:136  Abundance: 18.80(deg) 1(sRNA)
   5'     TCGGACCAGGCTTCATTCCCT        3'  ID:
          o||||||||||||||||||              Score: 2.5
   3' CTTAGGCCTGGTCCGAAGTAAGGTCCGTAAAC 5'  p-value: 0.0
```

Cs1g09575.1 gene=Cs1g09575 CDS=160-273

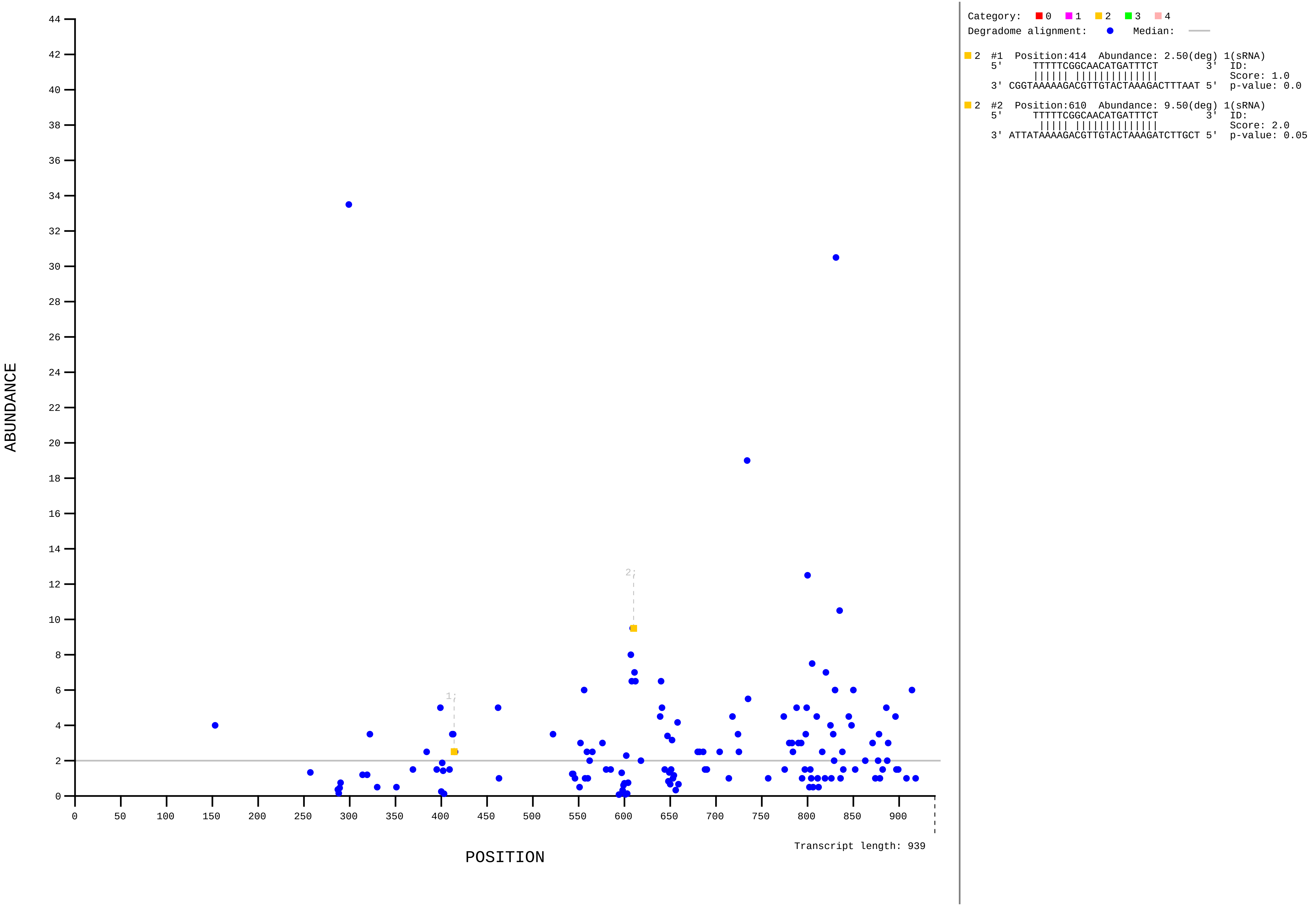

Cs1g09575.2 gene=Cs1g09575 CDS=160-273

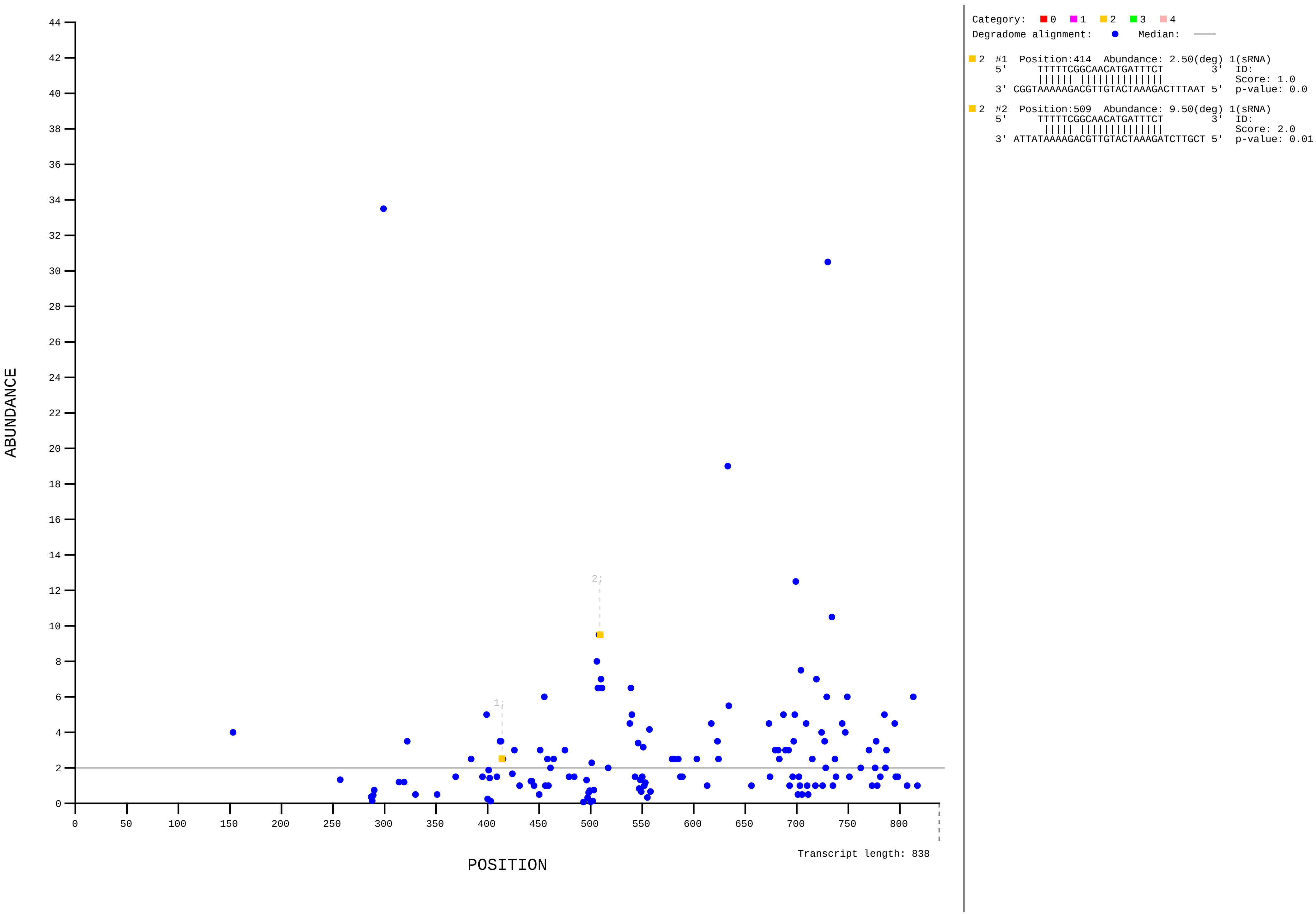

Cs5g05430.1 gene=Cs5g05430 CDS=437-2041

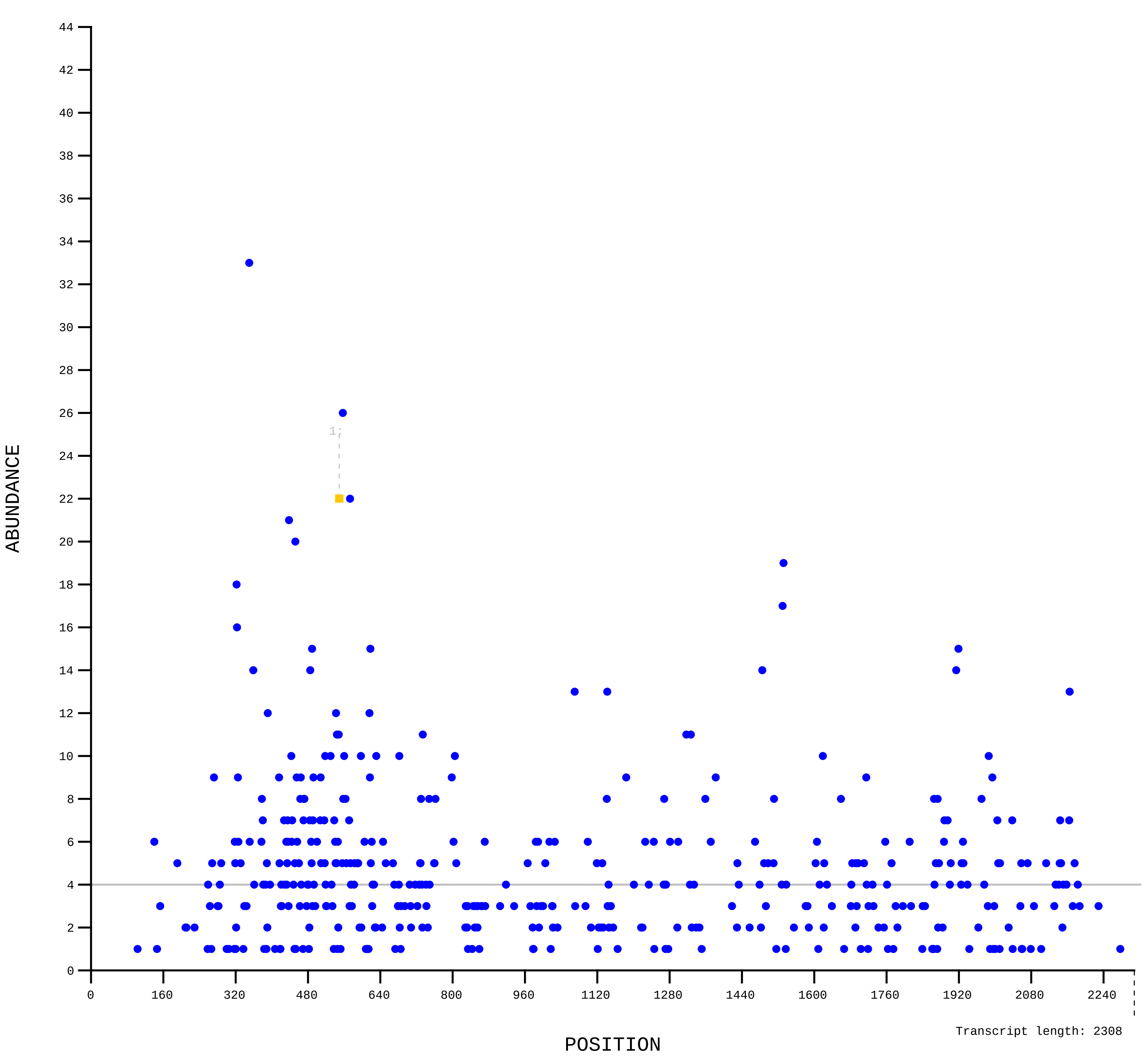

Category: 0 1 2 3 4

Degradome alignment: Median:

```
2  #1  Position:549  Abundance: 22.00(deg) 1(sRNA)
   5'    CT-TTCAGCAGCCTCCGGCGTC        3'  ID:
          || ||||||||||||||||||             Score: 3.0
   3' TCCGACAAGTCGTCGGAGGCCGCCCGGTAGAA 5'  p-value: 0.05
```

Cs7g03110.1 gene=Cs7g03110 CDS=185-2185

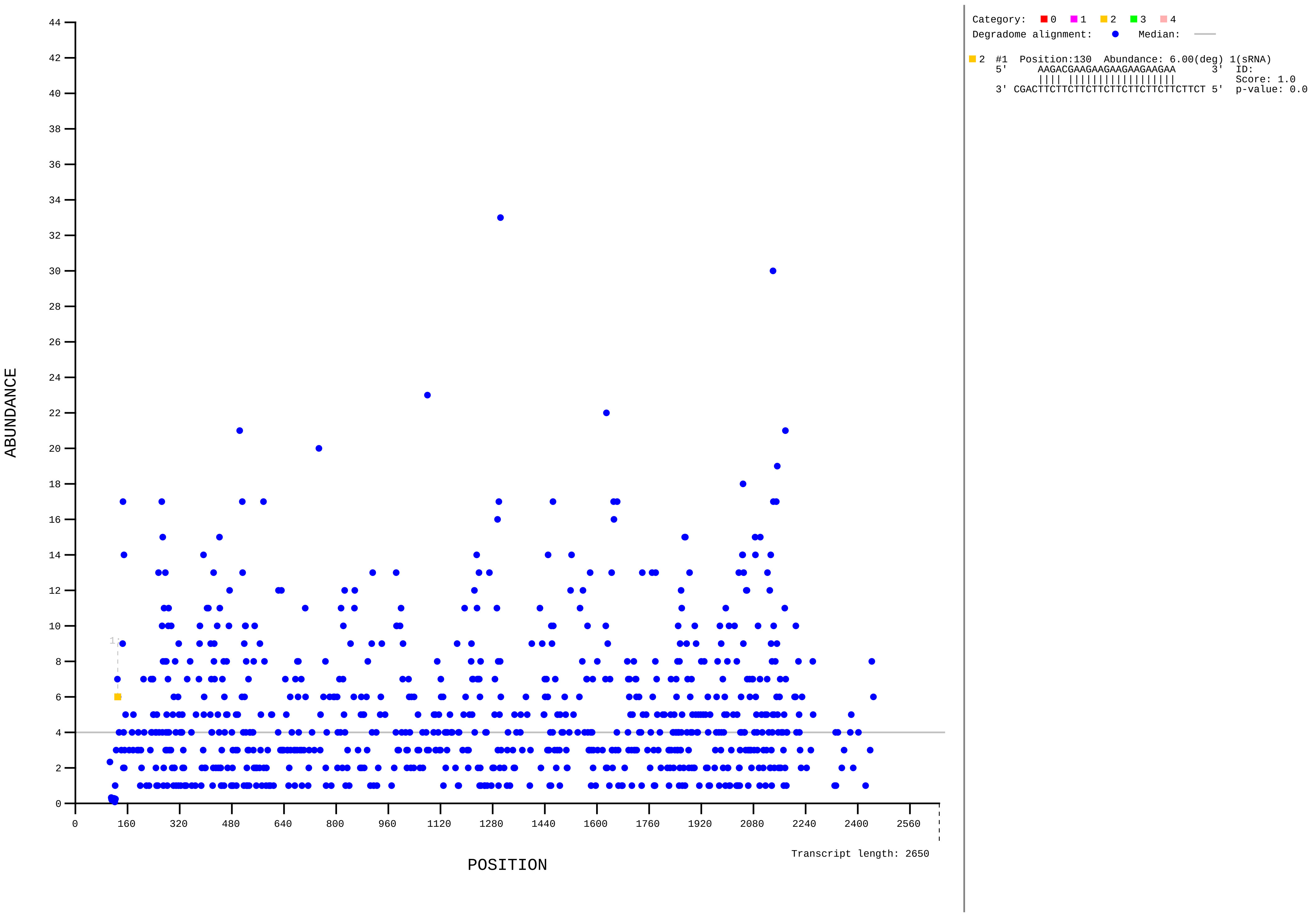

Cs4g03760.1 gene=Cs4g03760 CDS=503-3376

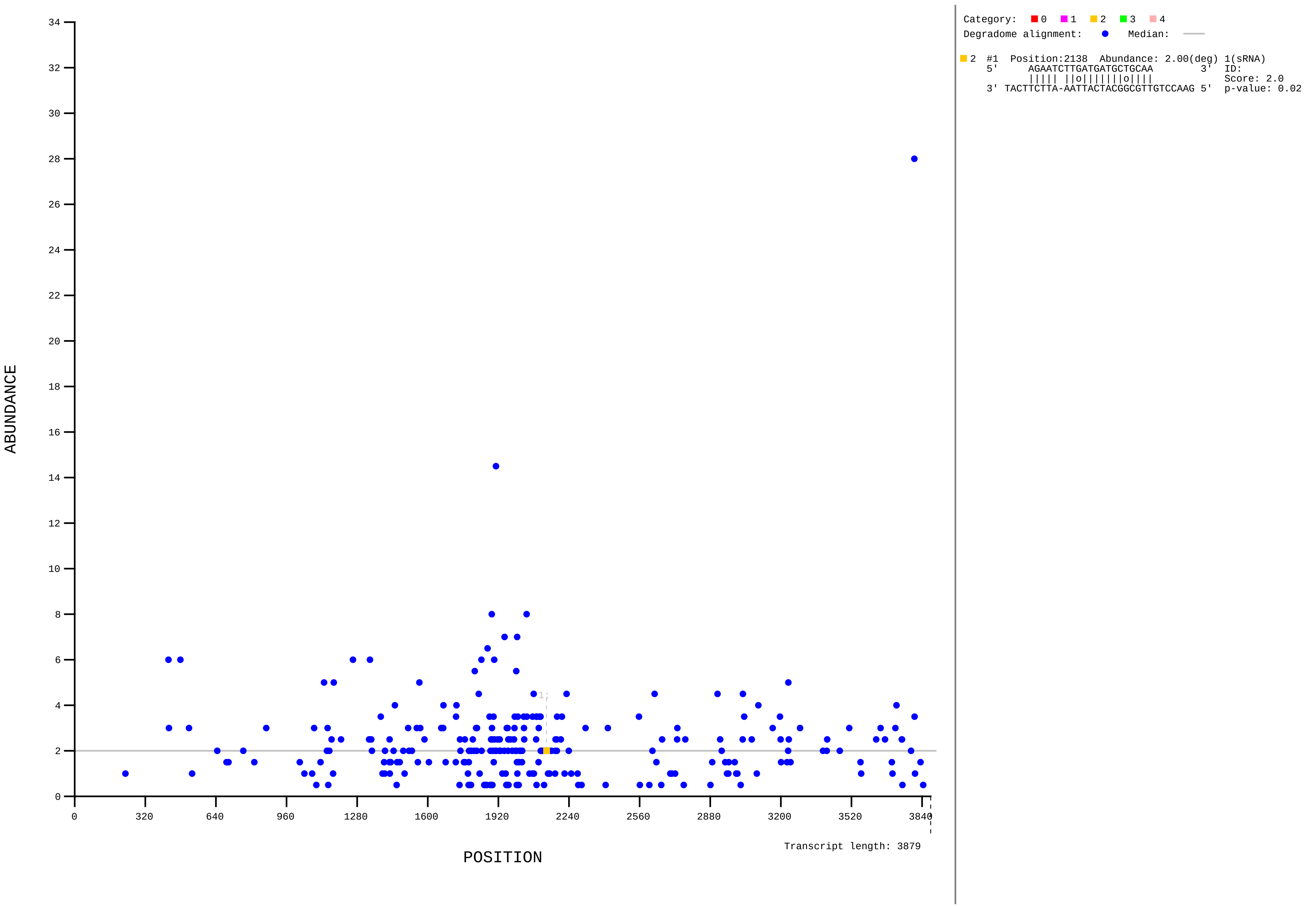

Cs4g03760.2 gene=Cs4g03760 CDS=1-2874

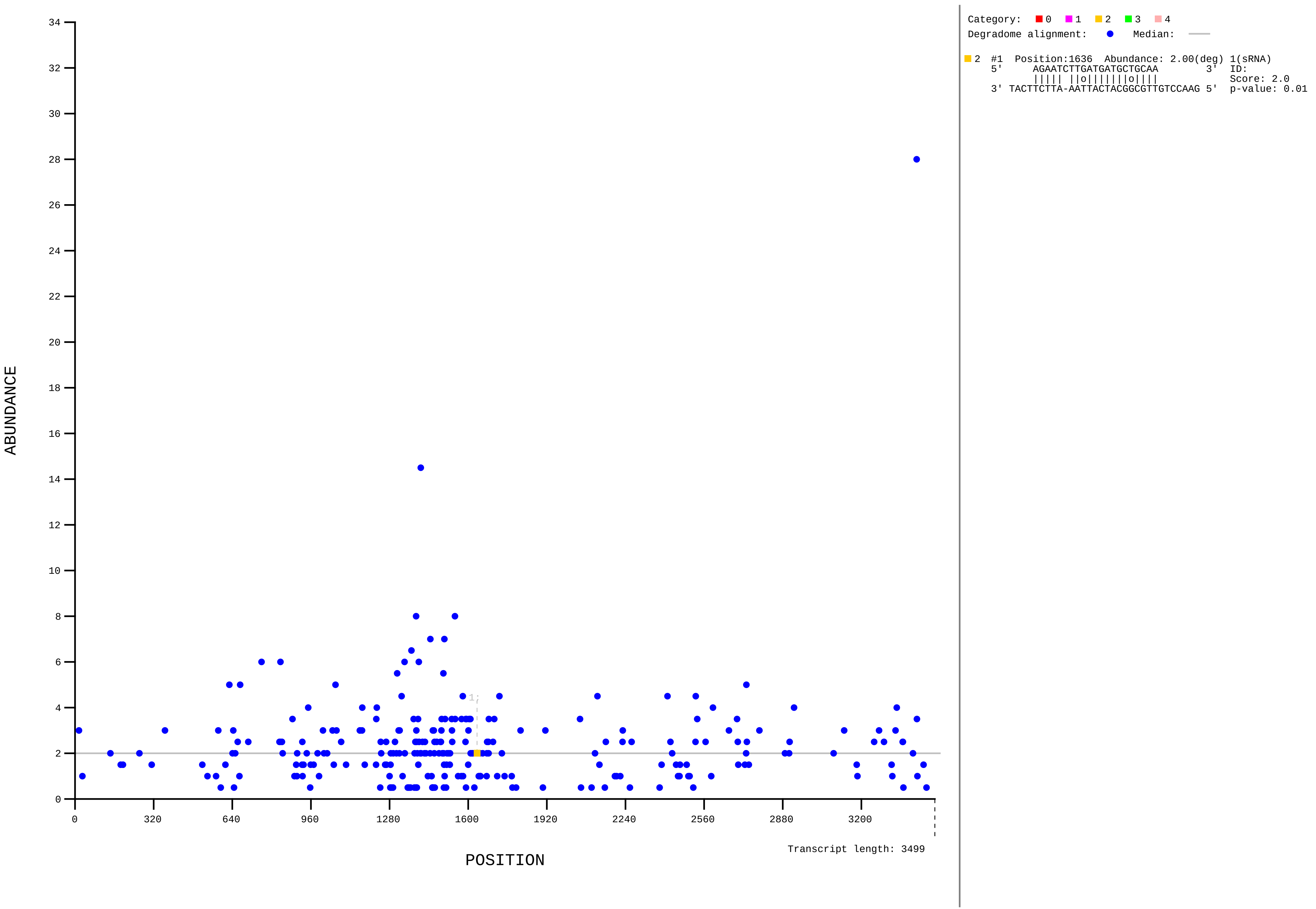

orange1.1t01926.1 gene=orange1.1t01926 CDS=90-2978

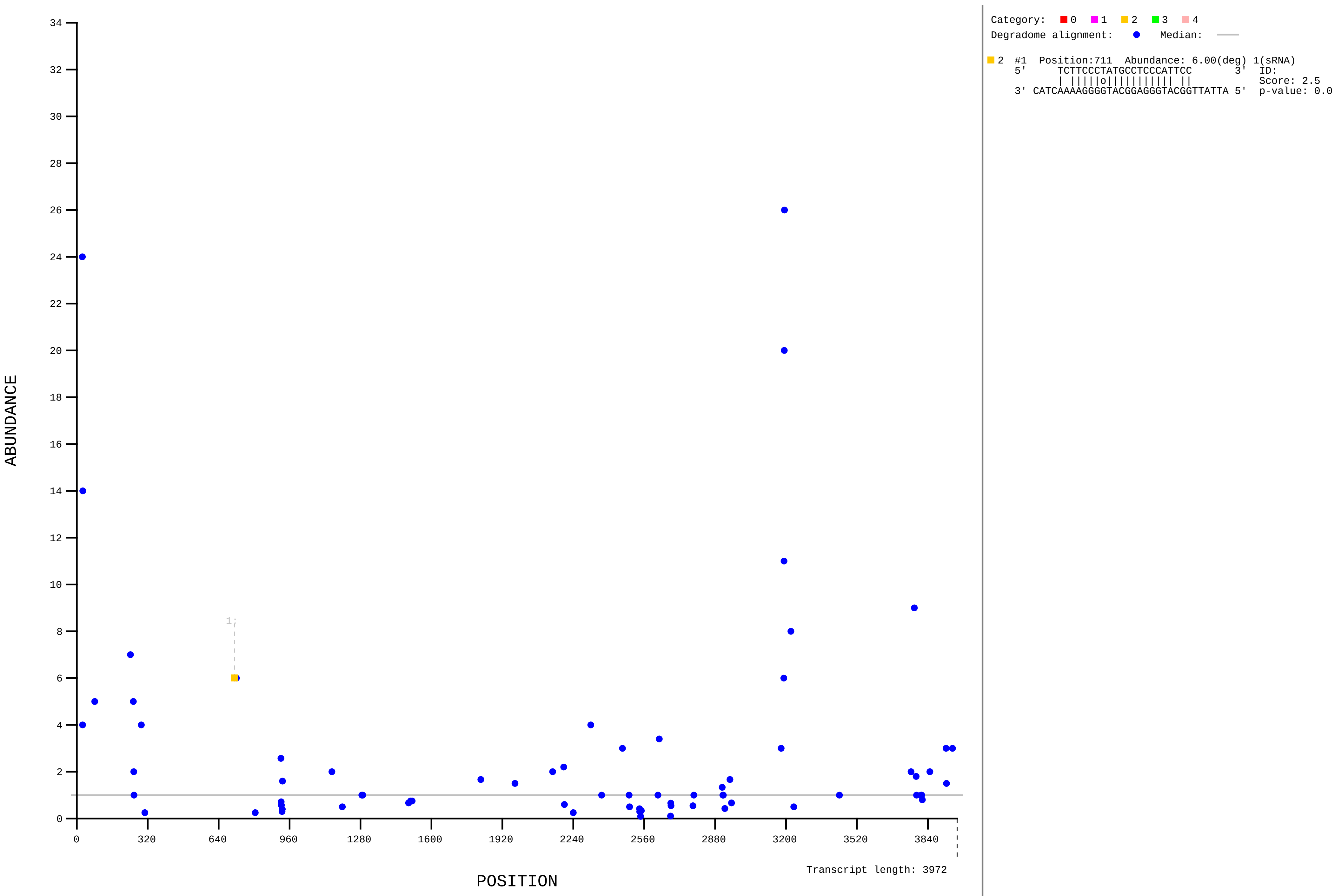

orange1.1t02948.1 gene=orange1.1t02948 CDS=177-3749

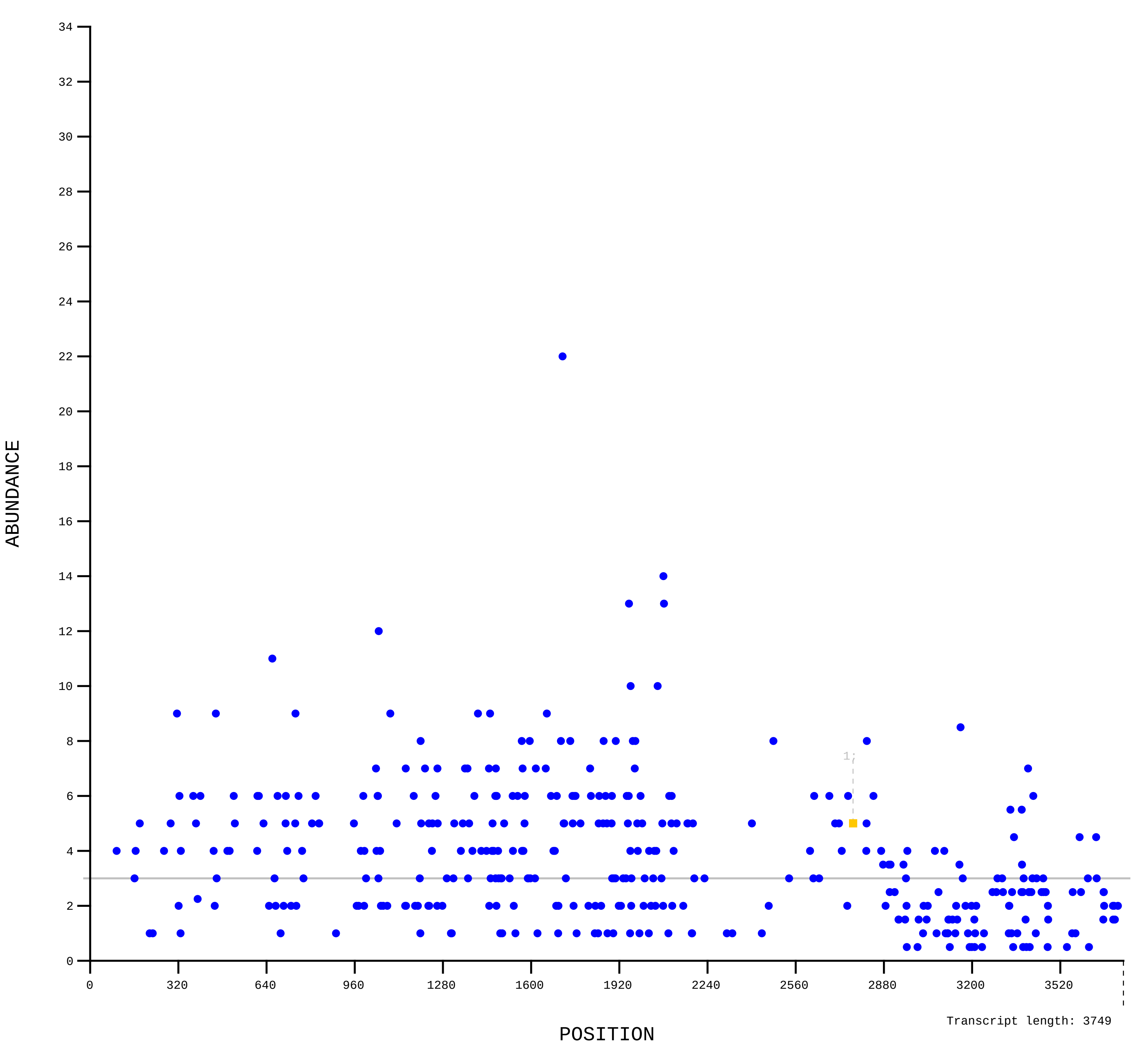

Category: 0 1 2 3 4
Degradome alignment: Median:

```
2  #1  Position:2768  Abundance: 5.00(deg) 1(sRNA)
   5'      TGCATTTGCACCTGCATCTTG        3'  ID:
           |||||o|||| |||||||| |            Score: 2.5
   3' AGTAACGTAGACGTAGACGTAGA-CGTTCGTC 5'  p-value: 0.04
```

orange1.1t03137.1 gene=orange1.1t03137 CDS=1-2916

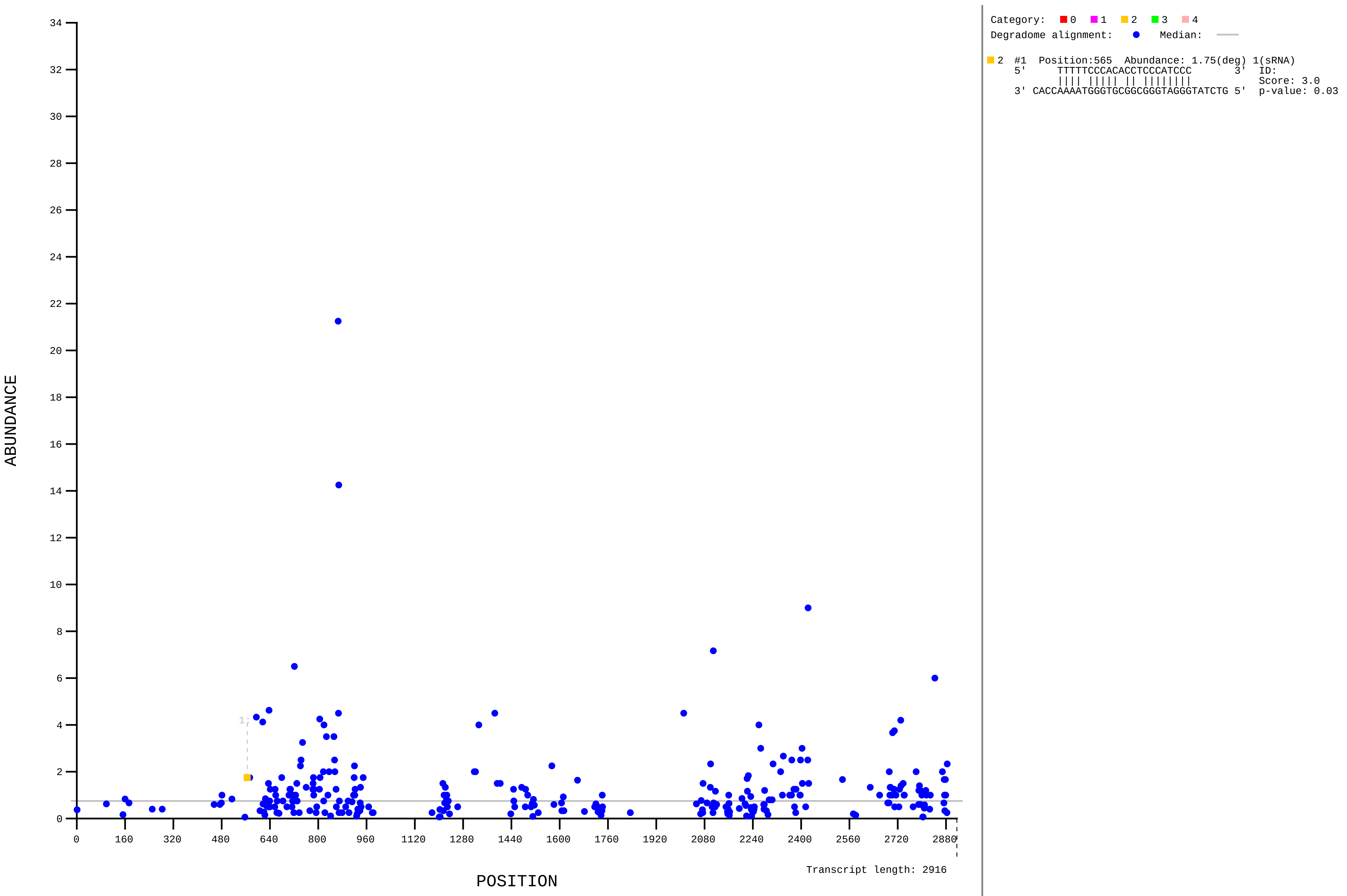

Cs3g13740.1 gene=Cs3g13740 CDS=44-2974

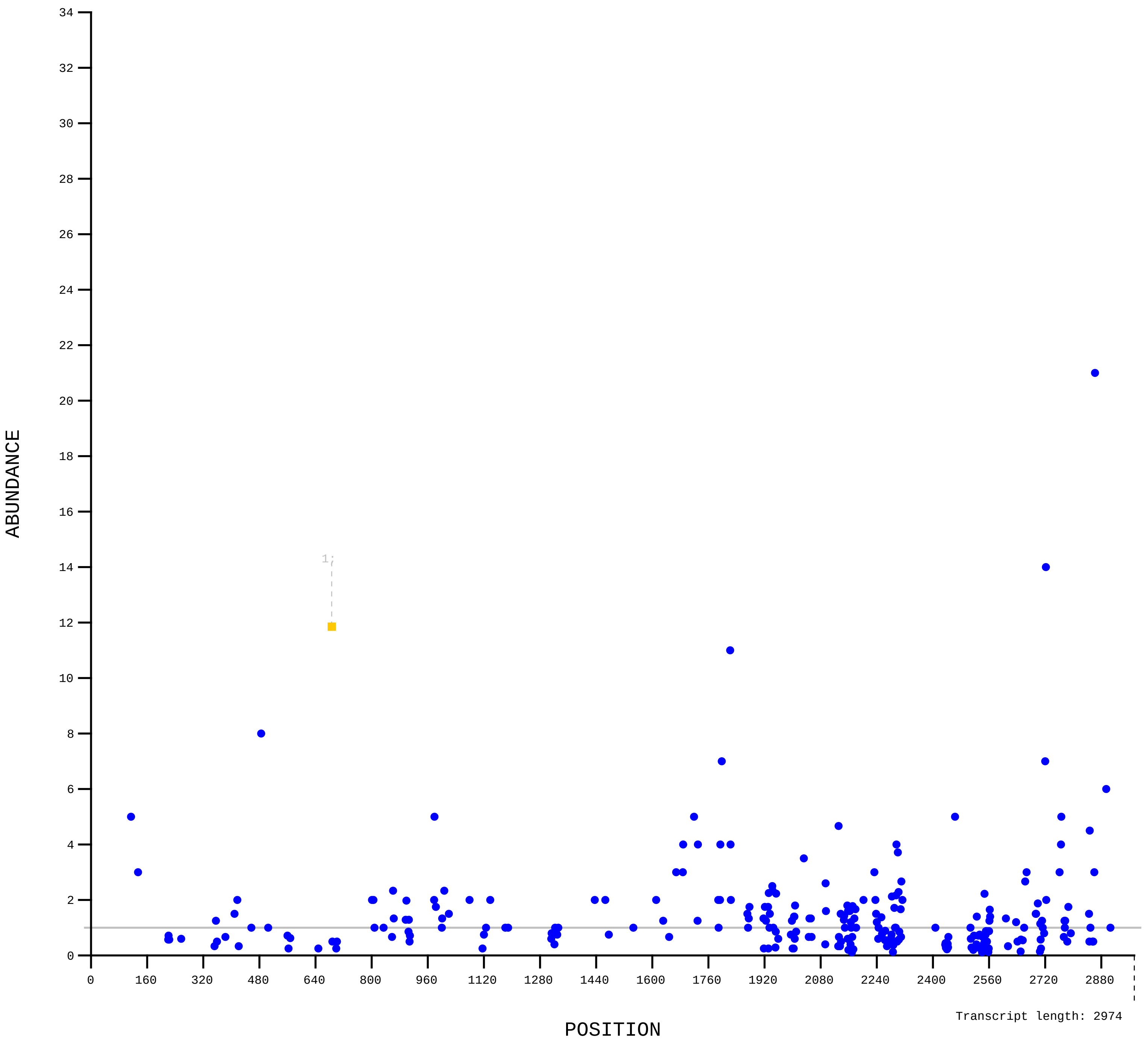

Category: 0 1 2 3 4
Degradome alignment: Median:

```
2  #1  Position:686  Abundance: 11.86(deg) 1(sRNA)
   5'     TCTTCCCTATGCCTCCCATTCC       3'  ID:
          | || ||||||||o|||||o||           Score: 3.0
   3' CATCAAAACGGATACGGGGGGTAGGGTTGTTC 5'  p-value: 0.02
```

Cs5g19200.2 gene=Cs5g19200 CDS=717-2126

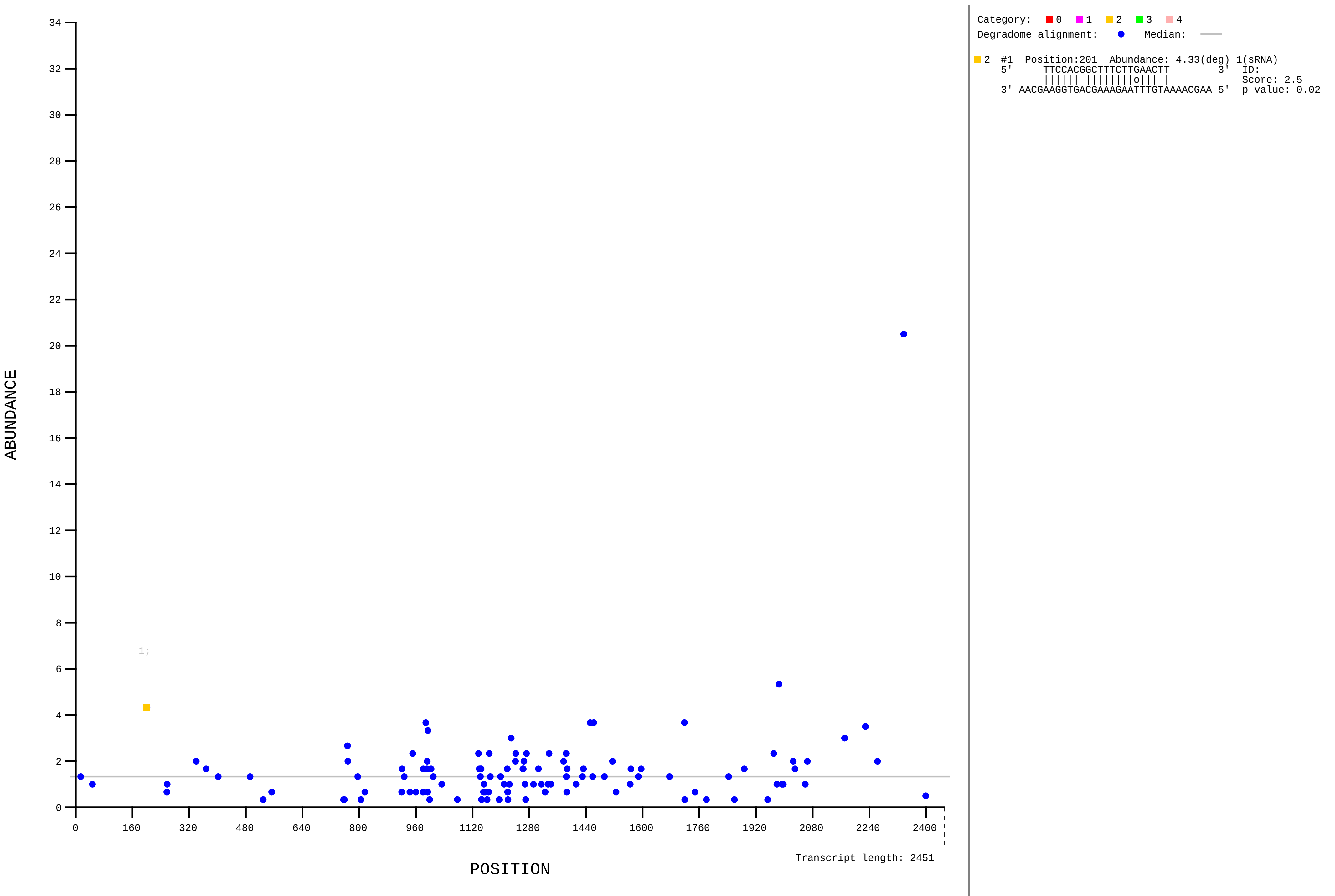

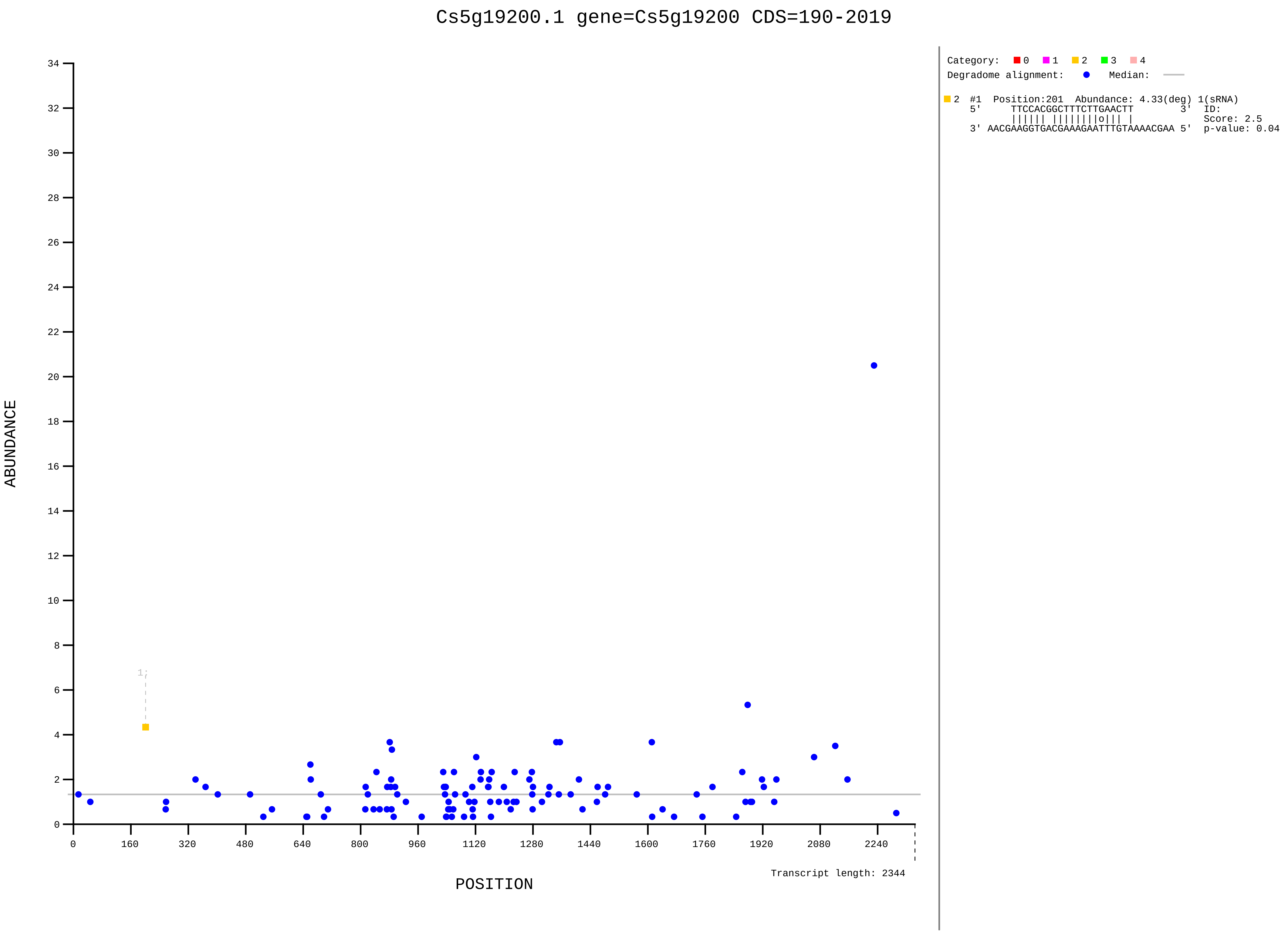

Cs5g19200.1 gene=Cs5g19200 CDS=190-2019
Category: 0 1 2 3 4
Degradome alignment: Median:
2 #1 Position:201 Abundance: 4.33(deg) 1(sRNA)
5' TTCCACGGCTTTCTTGAACTT 3' ID:
|||||| ||||||||o||| |
Score: 2.5
3' AACGAAGGTGACGAAAGAATTTGTAAAACGAA 5' p-value: 0.04
ABUNDANCE
0
2
4
6
8
10
12
14
16
18
20
22
24
26
28
30
32
34
POSITION
0
160
320
480
640
800
960
1120
1280
1440
1600
1760
1920
2080
2240
Transcript length: 2344
1

Cs9g01350.4 gene=Cs9g01350 CDS=451-1593

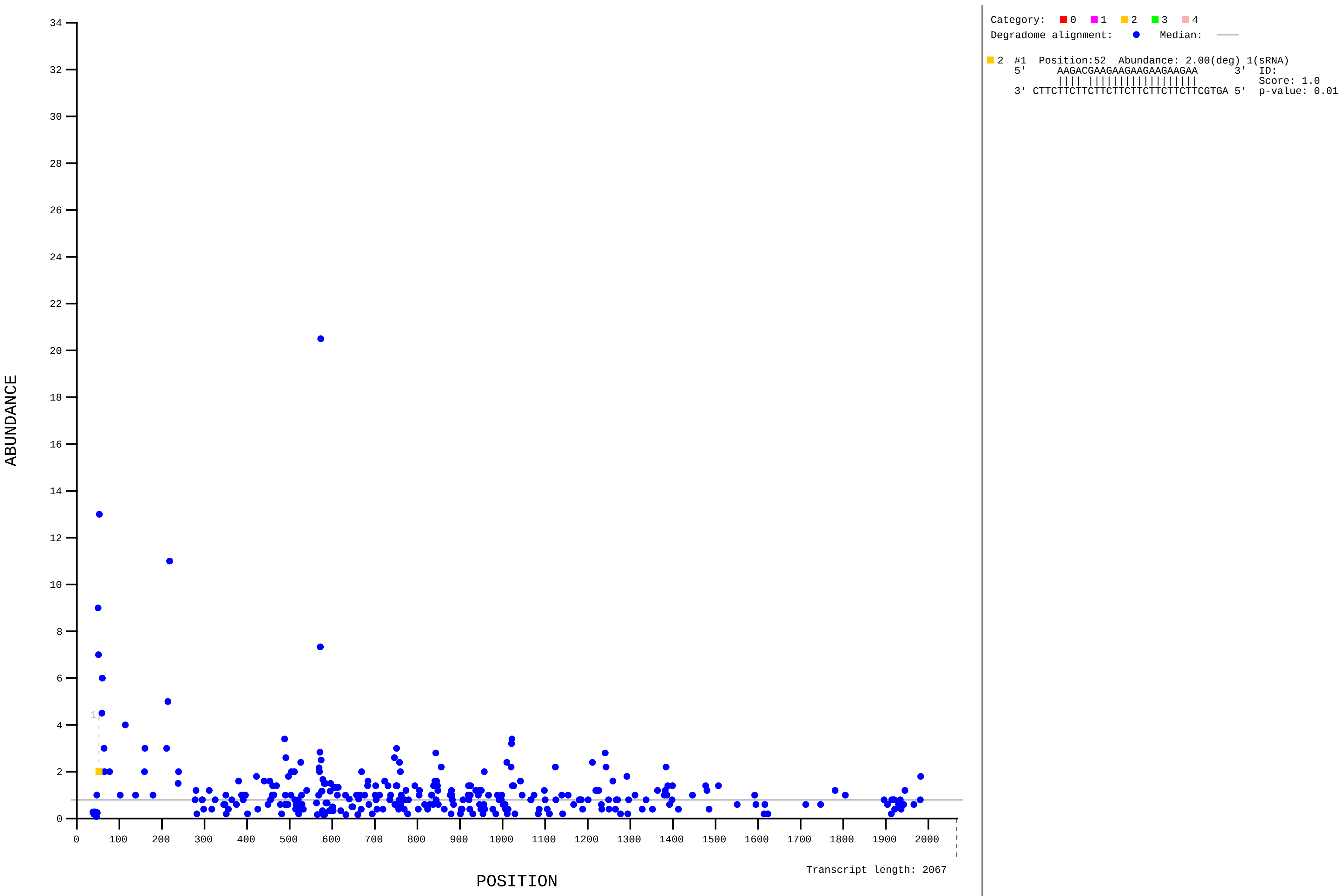

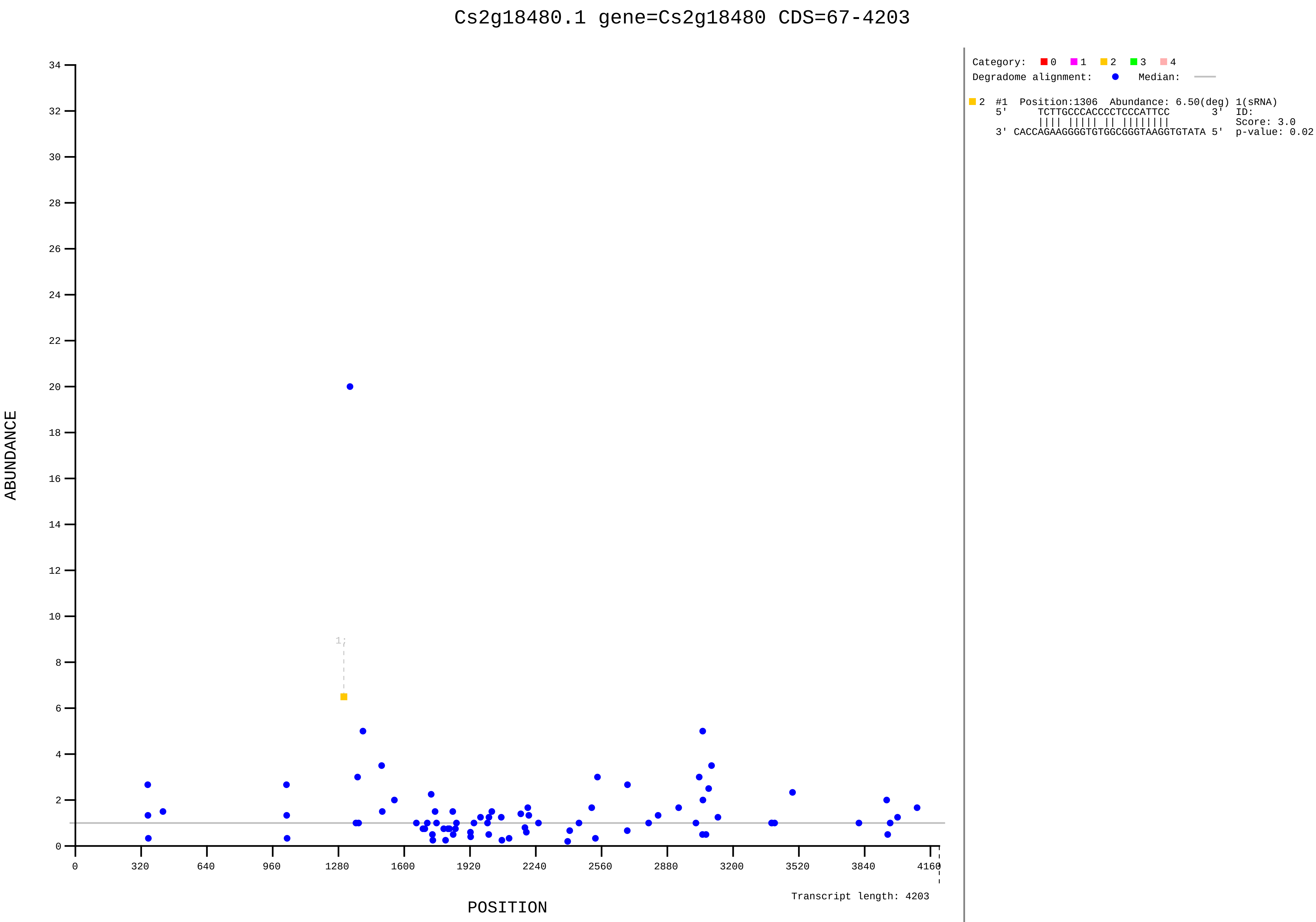
Cs2g18480.1 gene=Cs2g18480 CDS=67-4203
ABUNDANCE
POSITION
Transcript length: 4203
Category: 0 1 2 3 4
Degradome alignment:
Median:
2 #1 Position:1306 Abundance: 6.50(deg) 1(sRNA)
5' TCTTGCCCACCCCTCCCATTCC 3' ID:
Score: 3.0
3' CACCAGAAGGGGTGTGGCGGGTAAGGTGTATA 5' p-value: 0.02

Cs2g18470.1 gene=Cs2g18470 CDS=284-4603

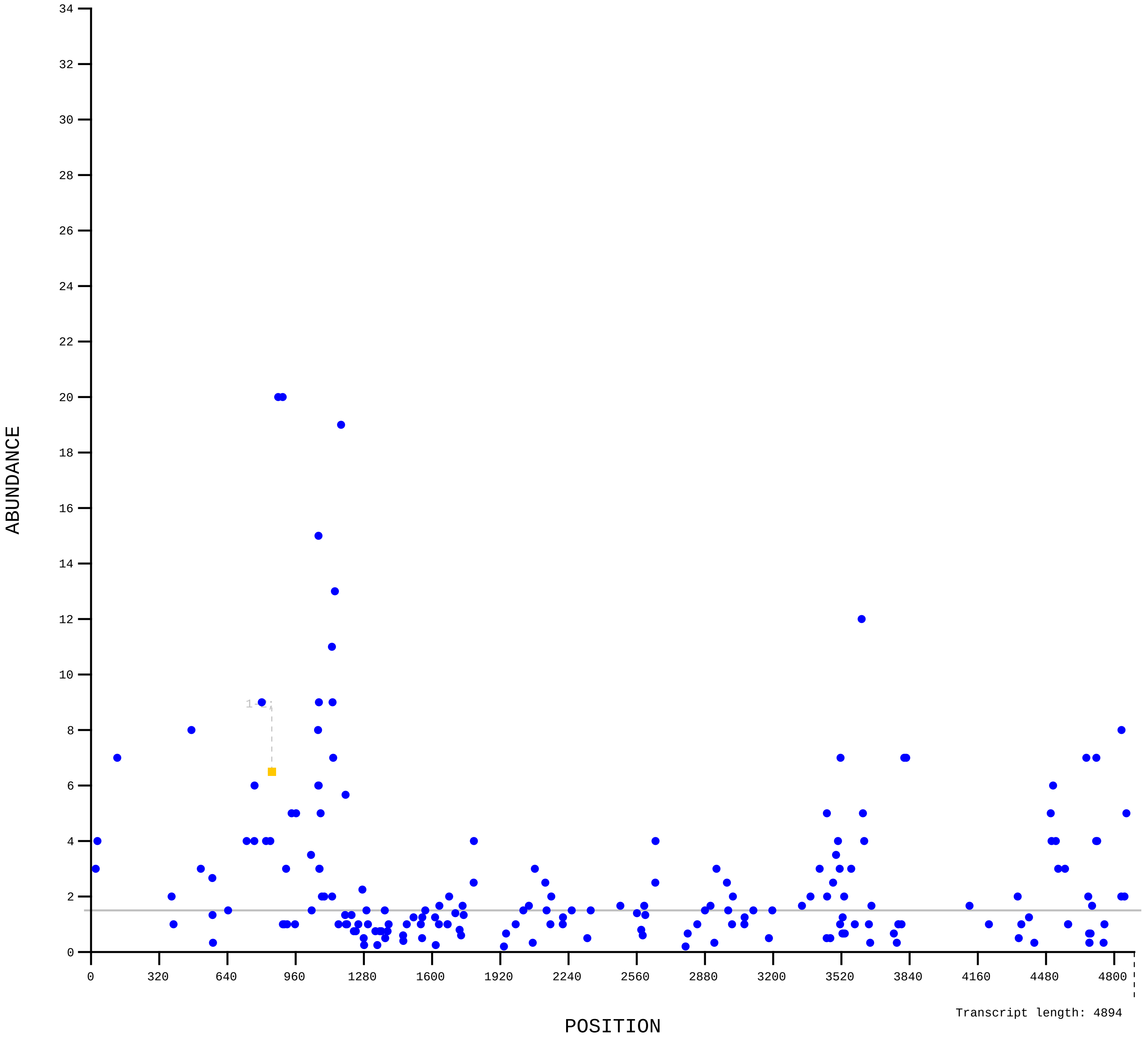


```
Category: 0 1 2 3 4
Degradome alignment:     Median:

2  #1  Position:848  Abundance: 6.50(deg) 1(sRNA)
   5'     TCTTGCCCACCCCTCCCATTCC       3'  ID:
          |||| ||||| ||o||||||||           Score: 2.5
   3' CACCAGAAGGGGTGTGGGGGGTAAGGCGTATA 5'  p-value: 0.01

2  #2  Position:848  Abundance: 6.50(deg) 1(sRNA)
   5'     TTTTTCCCACACCTCCCATCCC       3'  ID:
          |o||o||||||||o||||| ||           Score: 2.5
   3' CACCAGAAGGGGTGTGGGGGGTAAGGCGTATA 5'  p-value: 0.02
```

Cs9g06846.2 gene=Cs9g06846 CDS=857-1000

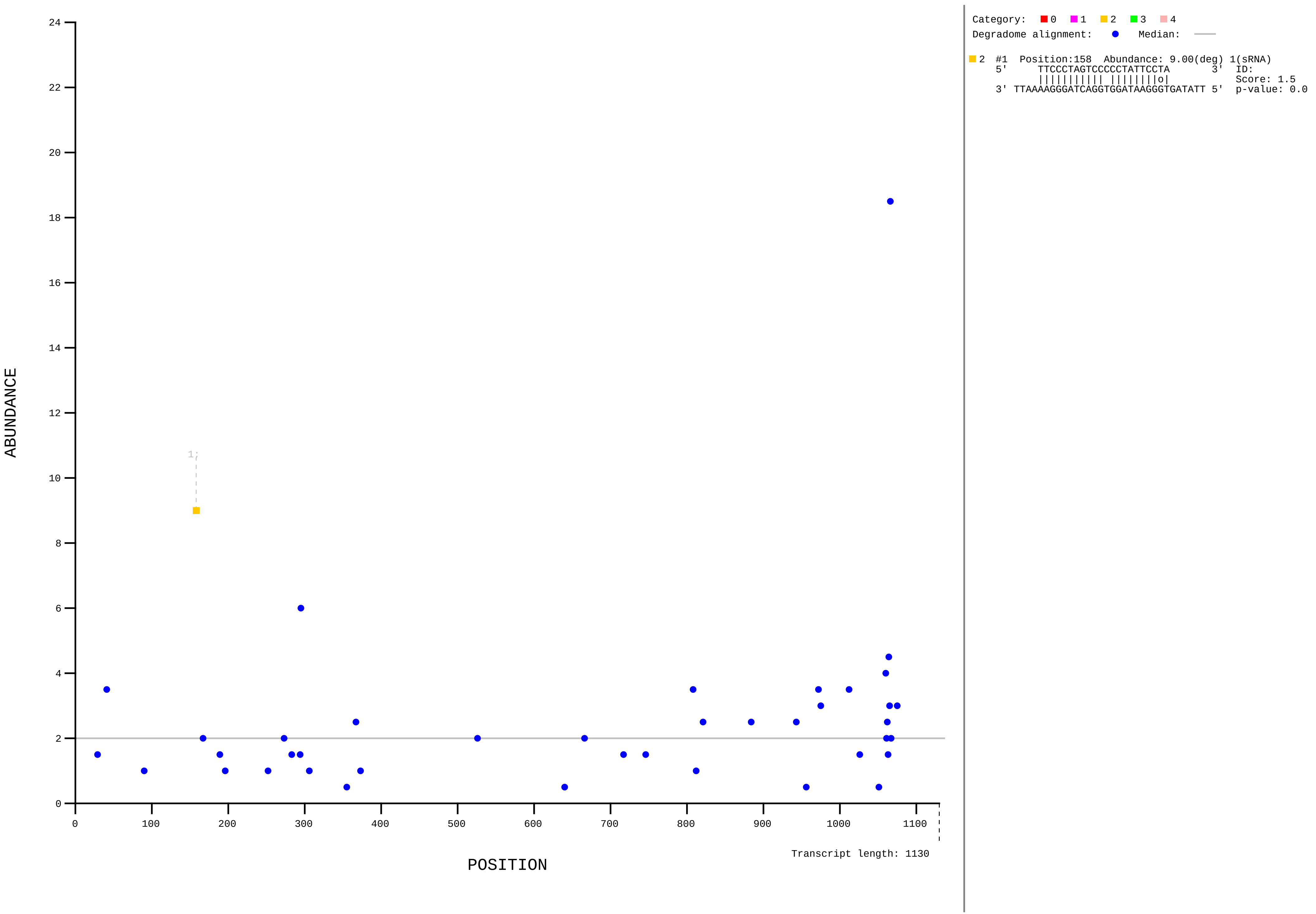

Cs3g07940.2 gene=Cs3g07940 CDS=1-4095

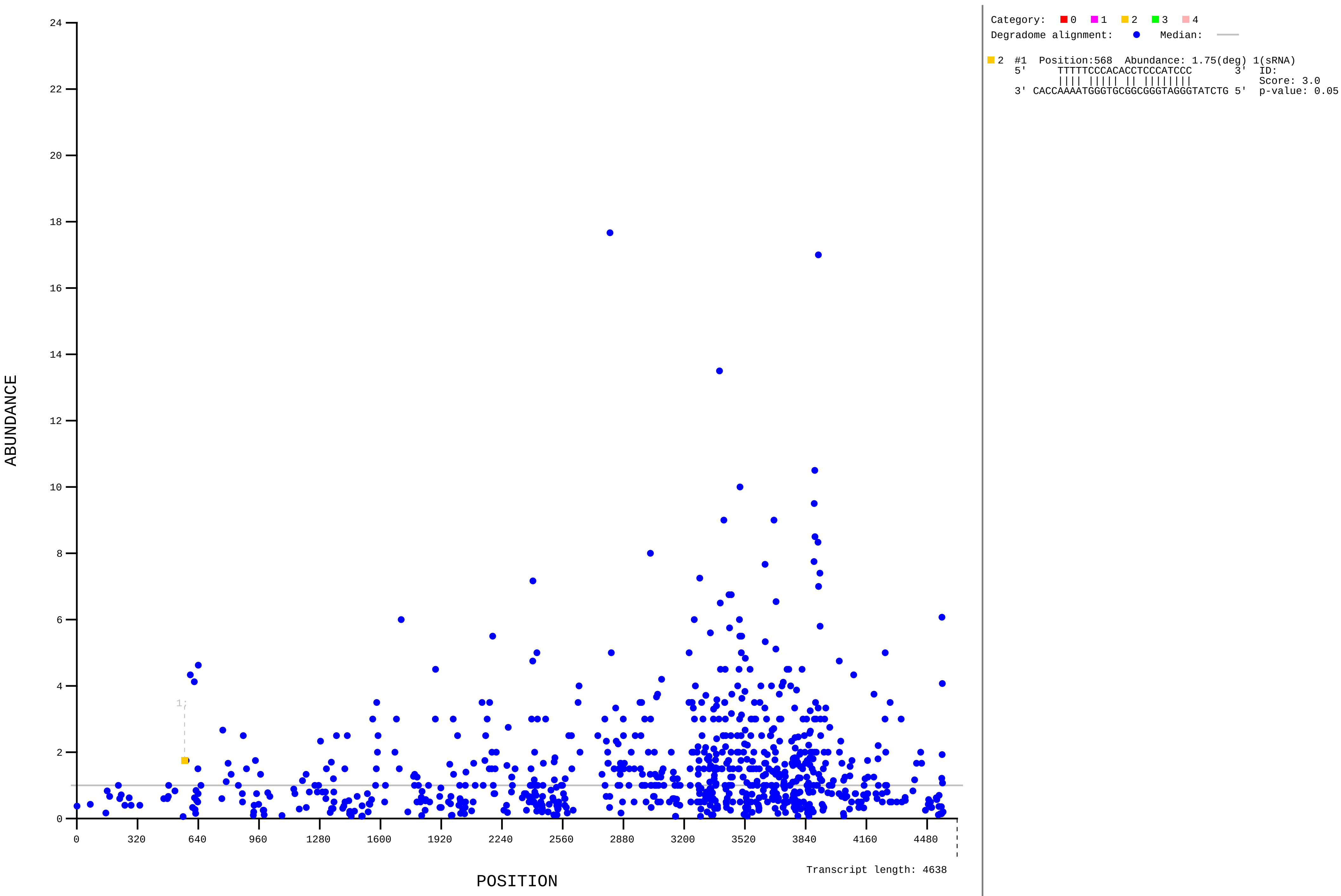

Cs3g07940.1 gene=Cs3g07940 CDS=1-4590

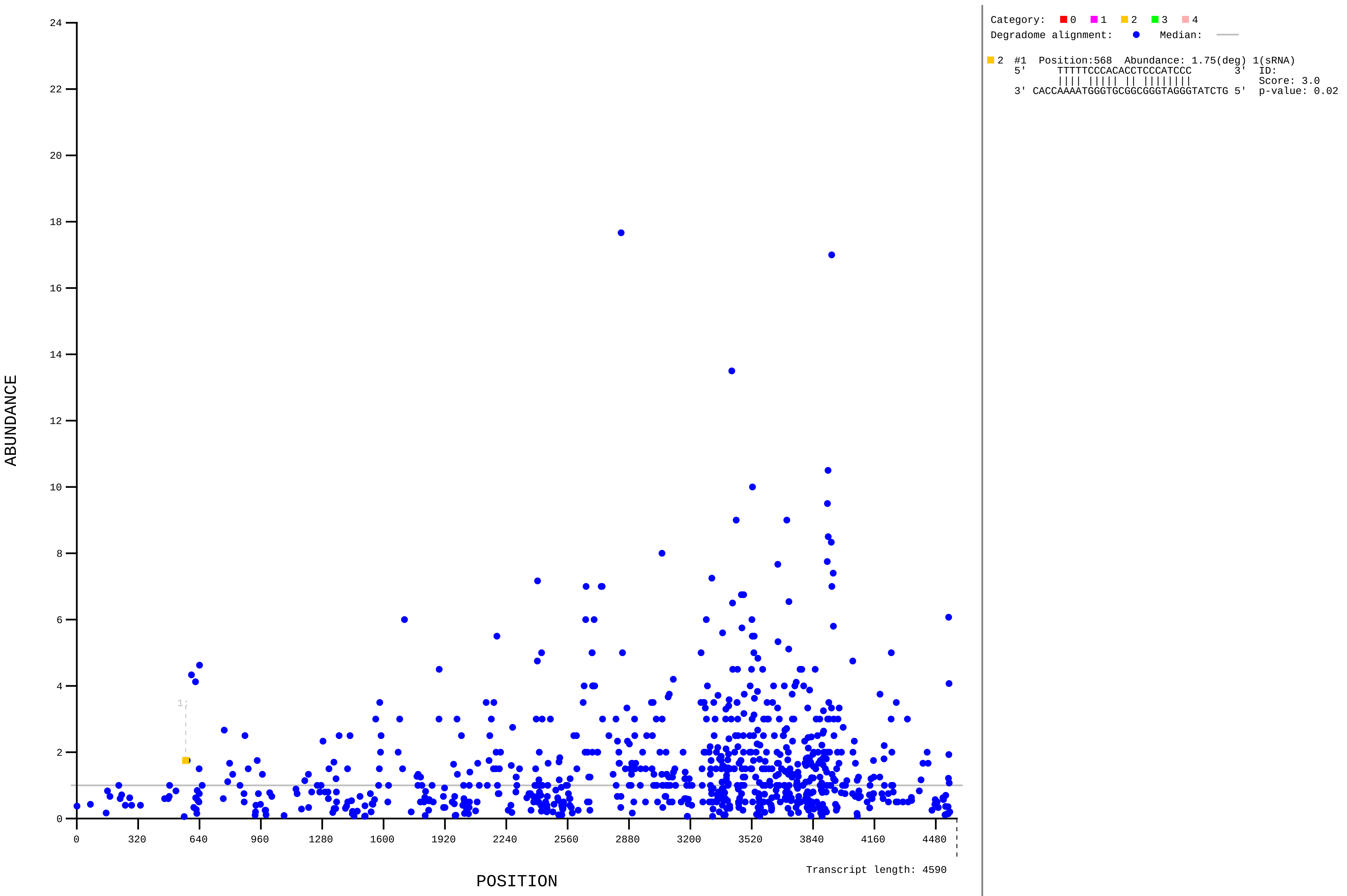

Cs6g14050.1 gene=Cs6g14050 CDS=123-1445

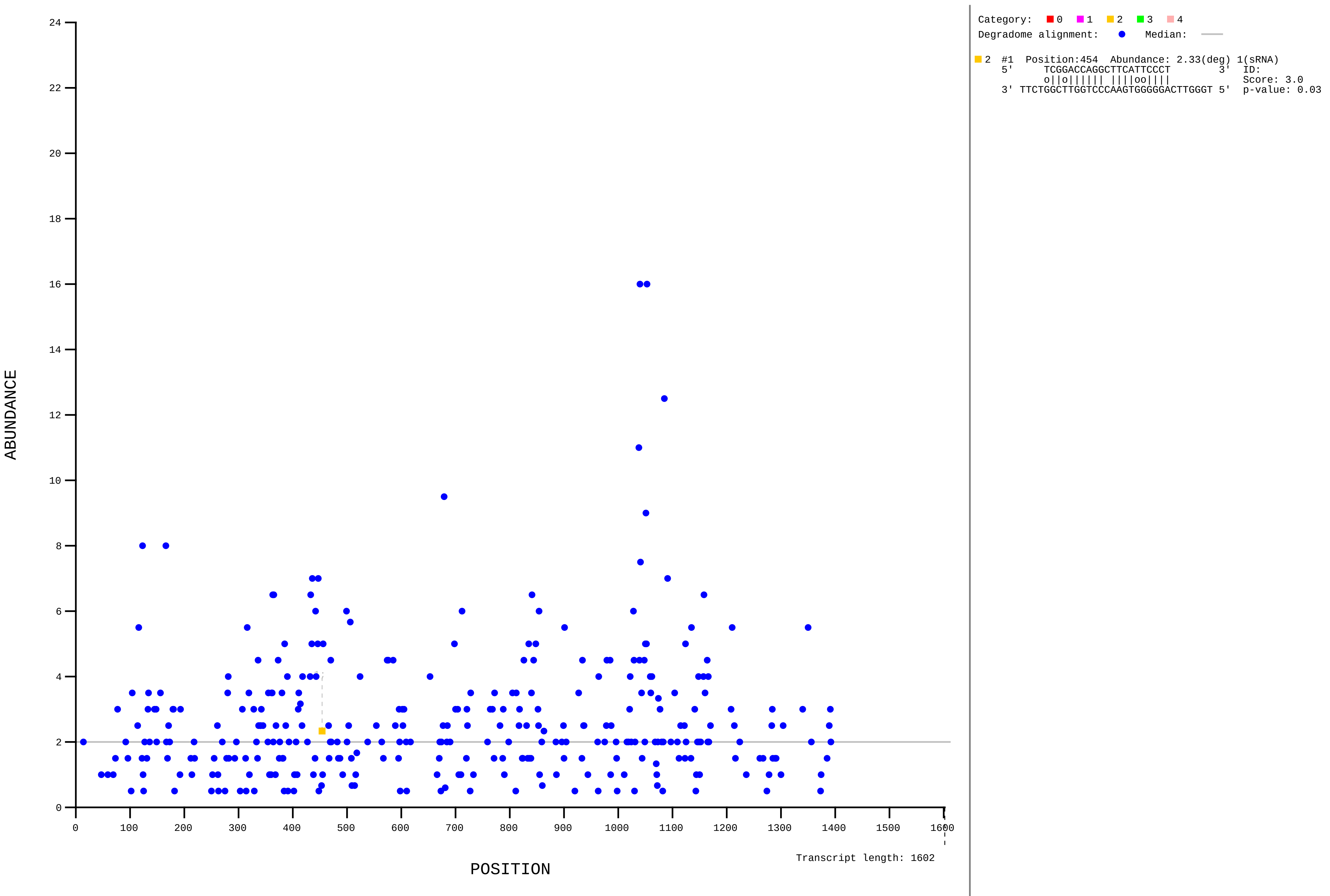

Cs3g13320.1 gene=Cs3g13320 CDS=1-2766

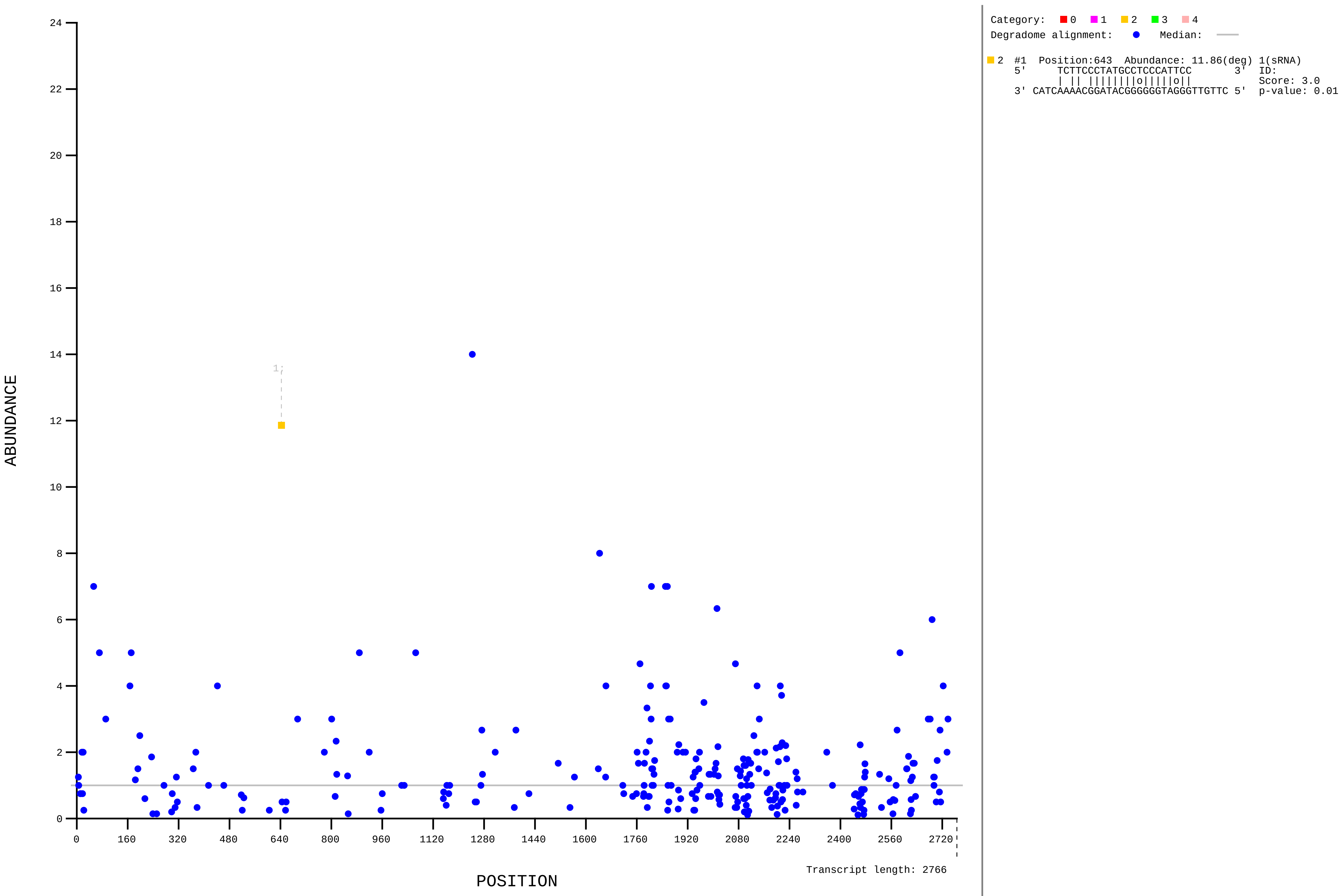

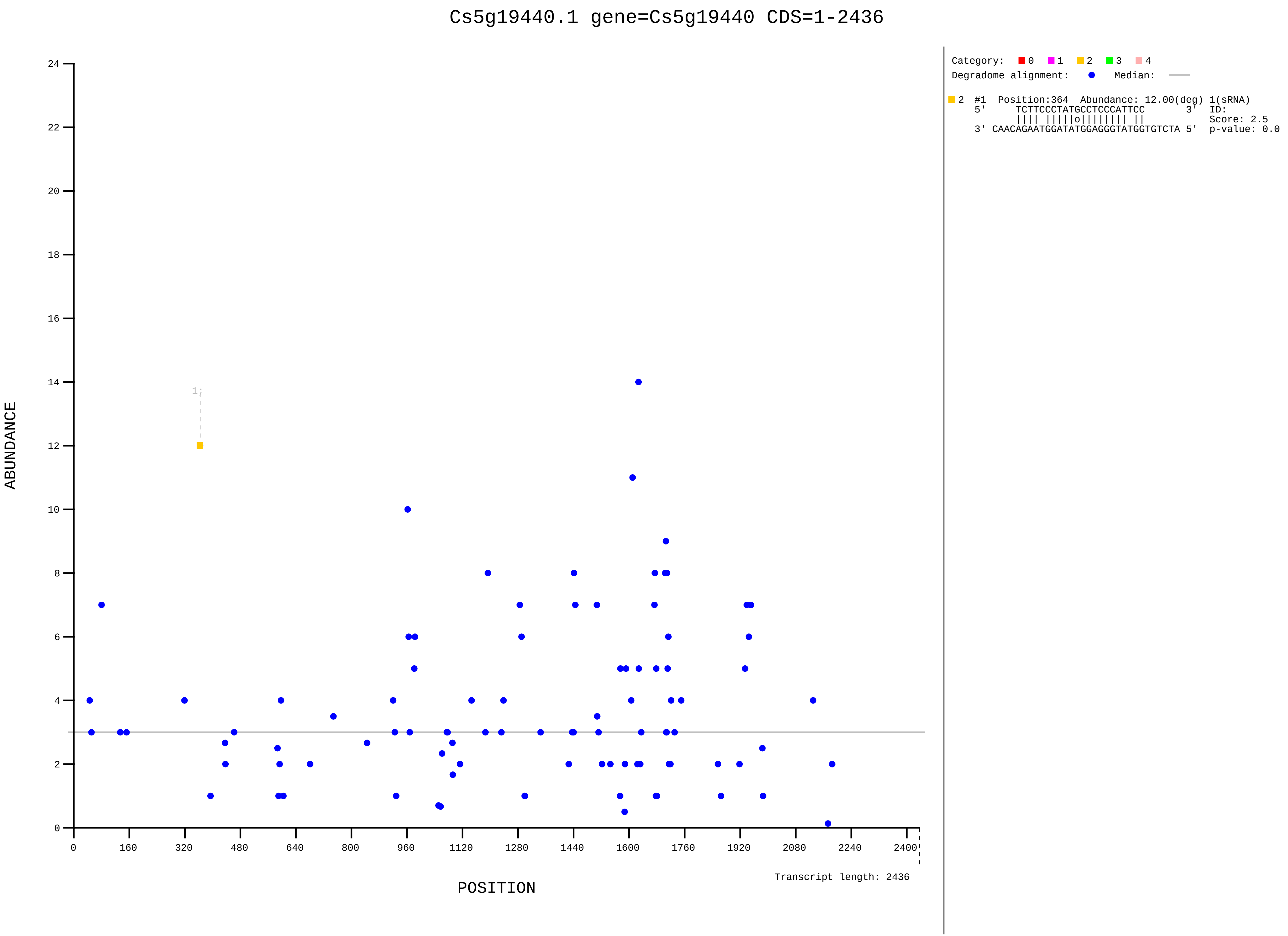

Cs5g19440.1 gene=Cs5g19440 CDS=1-2436
ABUNDANCE
POSITION
Transcript length: 2436
Category: 0 1 2 3 4
Degradome alignment:
Median:
2 #1 Position:364 Abundance: 12.00(deg) 1(sRNA)
5' TCTTCCCTATGCCTCCCATTCC 3' ID:
|||| |||||o|||||||| ||
Score: 2.5
3' CAACAGAATGGATATGGAGGGTATGGTGTCTA 5' p-value: 0.0

Cs6g11940.1 gene=Cs6g11940 CDS=361-2517

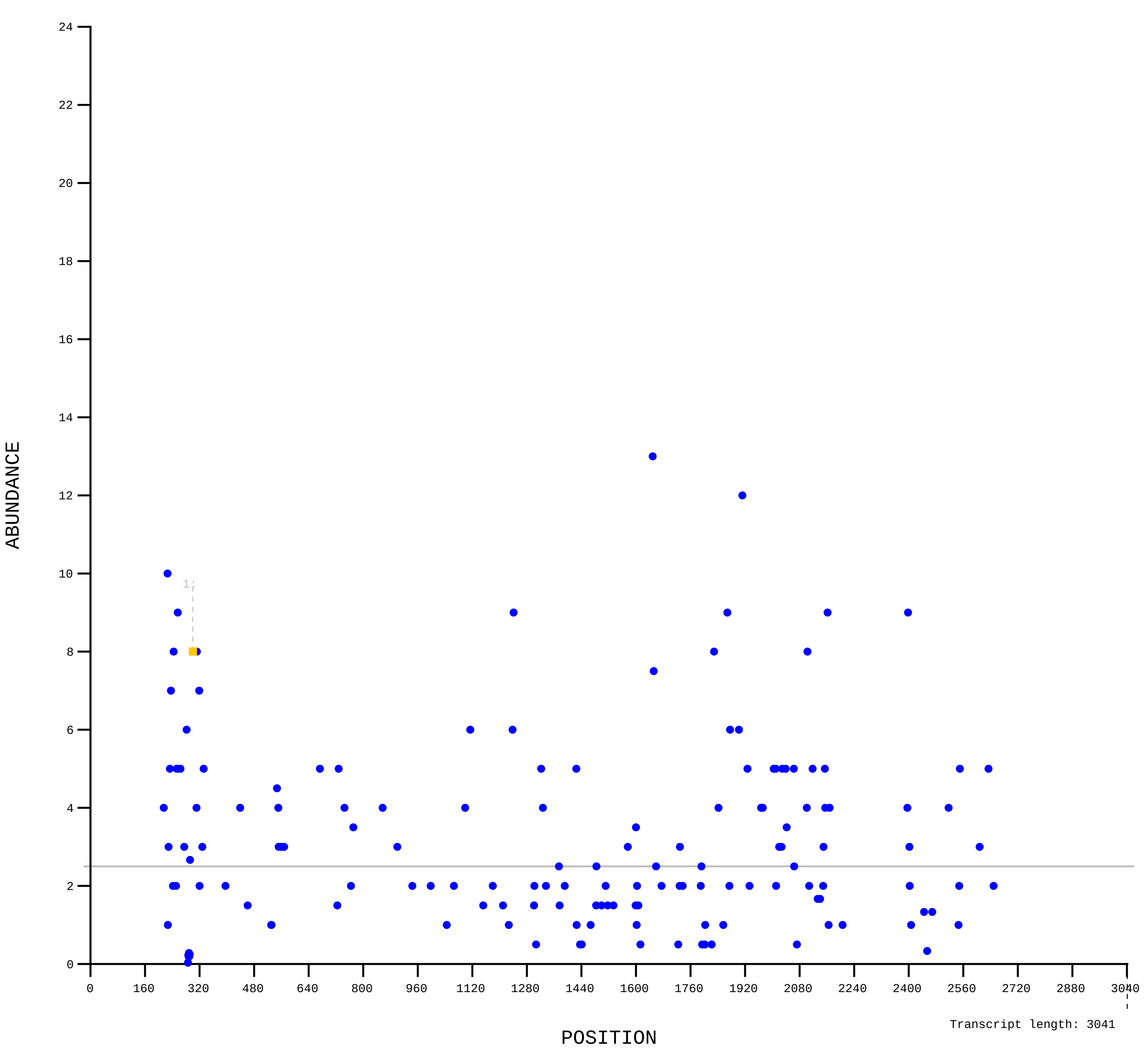

2 #1 Position:300 Abundance: 8.00(deg) 1(sRNA)

```
5'      AAGACGAAGAAGAAGAAGAAGAA      3'  ID:
        |||| |||||||||||||||||           Score: 1.0
3' ATCTTTCTTCTTCTTCTTCTTCTTACTTA 5'  p-value: 0.01
```

Cs3g11030.1 gene=Cs3g11030 CDS=1-6510

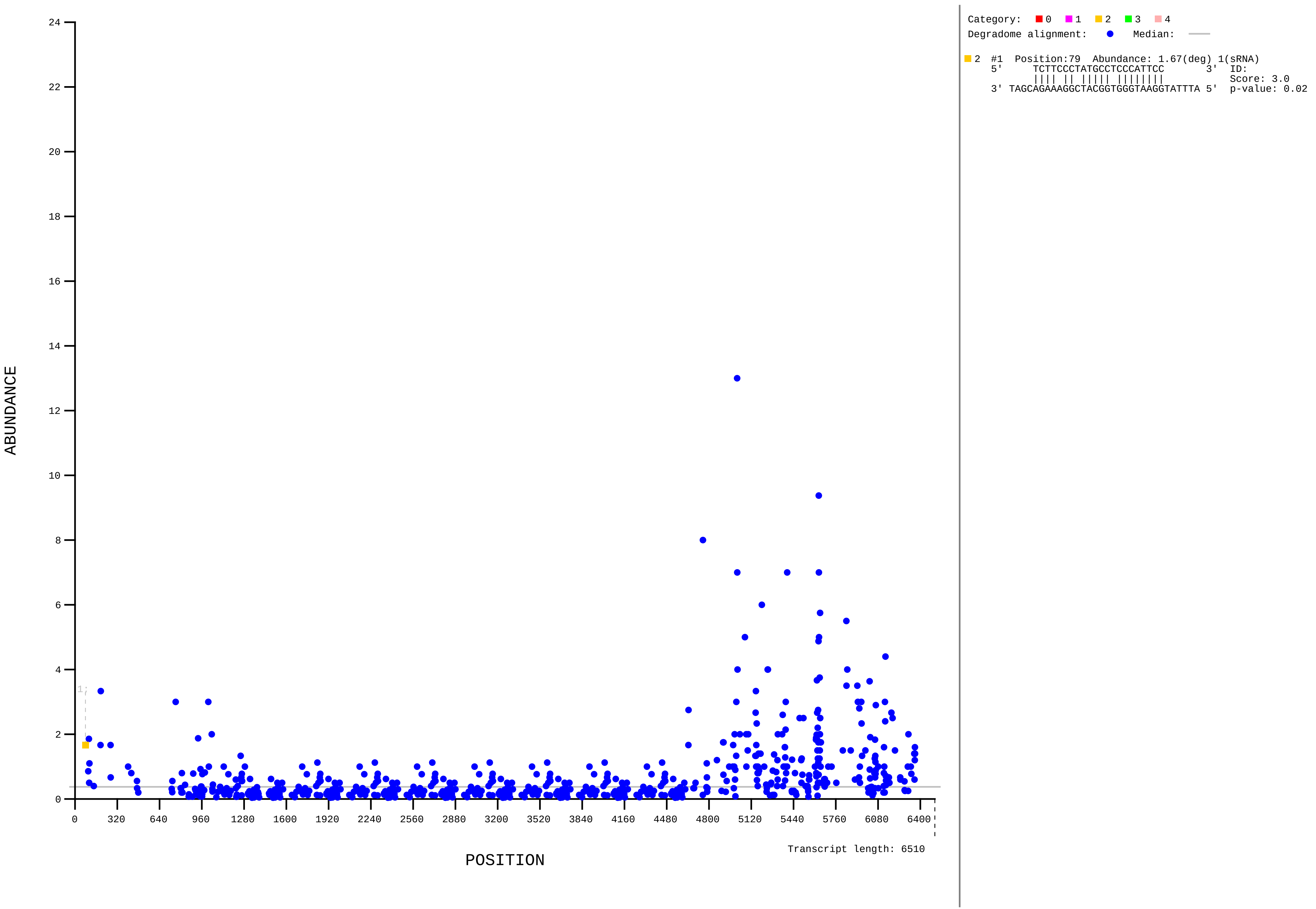

Cs5g26620.2 gene=Cs5g26620 CDS=125-2239

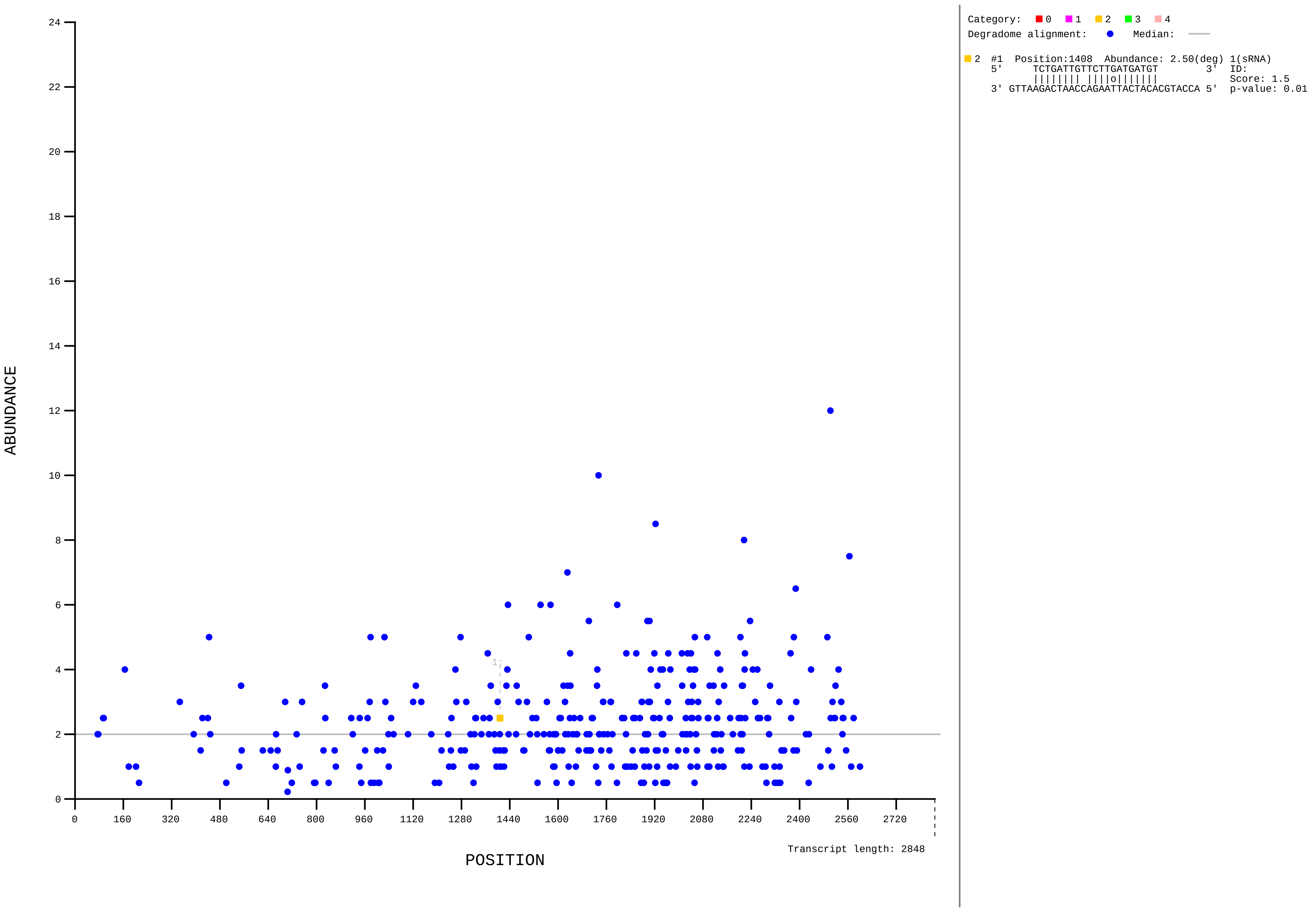

orange1.1t03059.3 gene=orange1.1t03059 CDS=574-2721

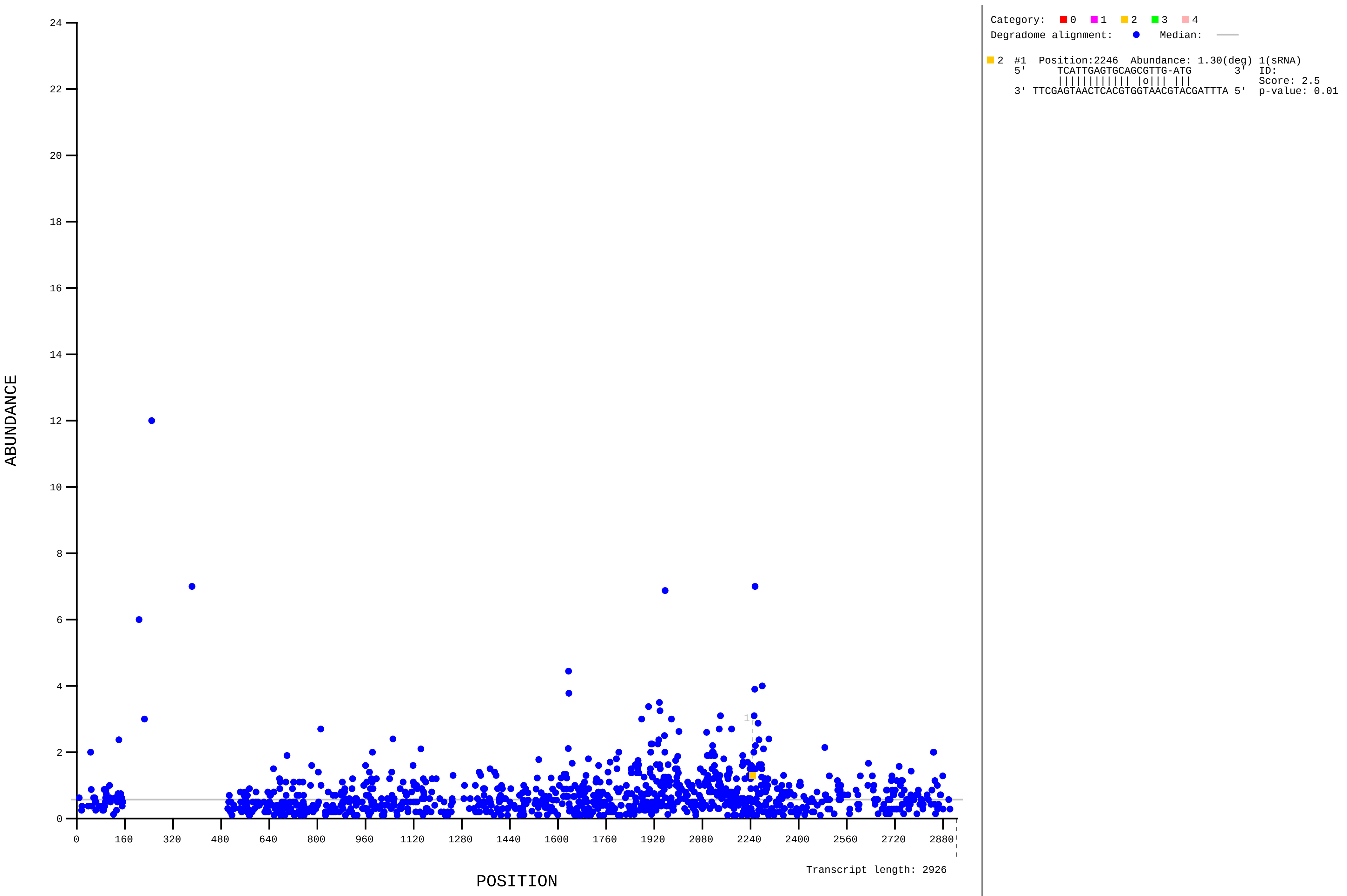

Cs6g02130.1 gene=Cs6g02130 CDS=1-4269

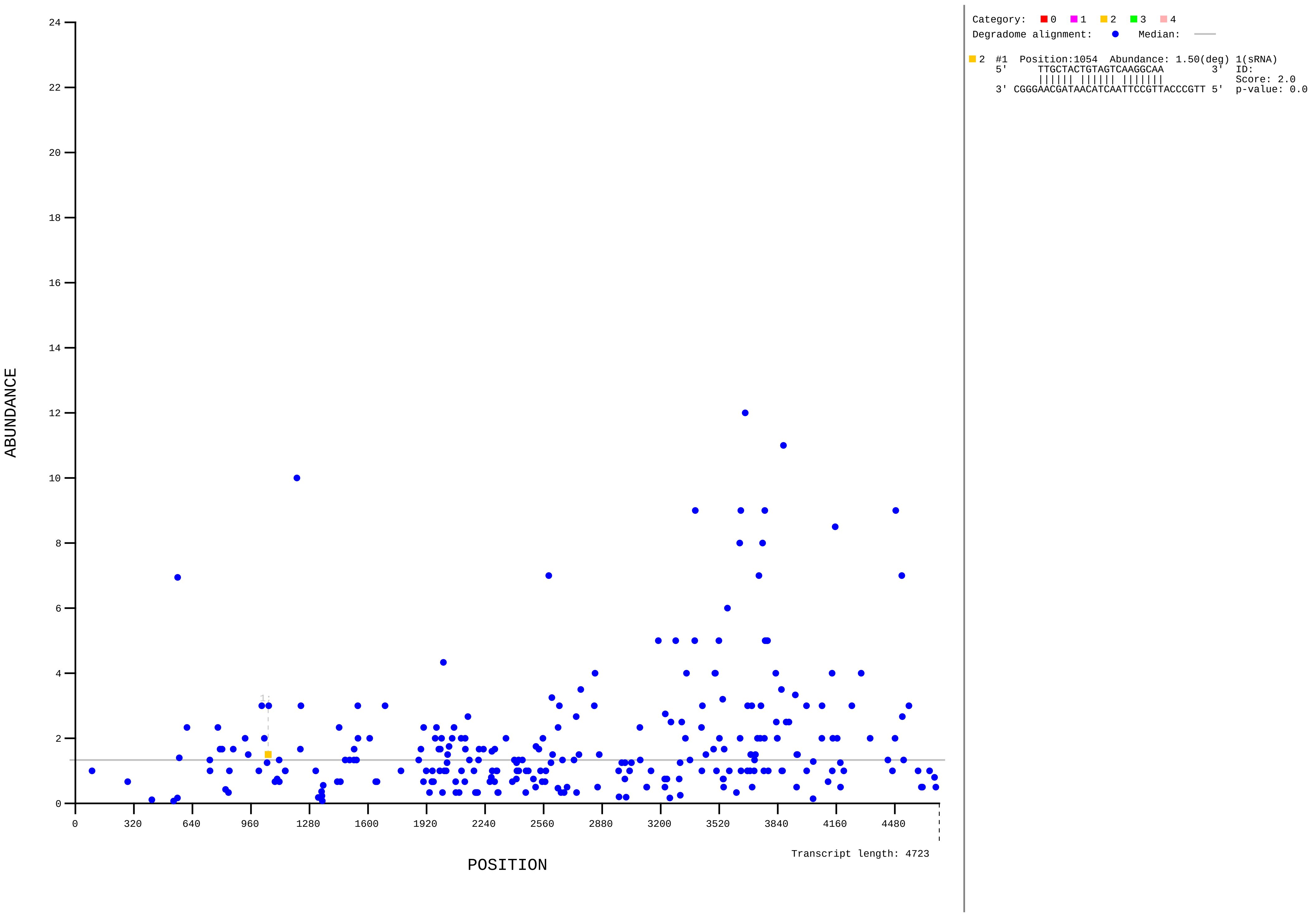

Cs5g26620.1 gene=Cs5g26620 CDS=399-2513

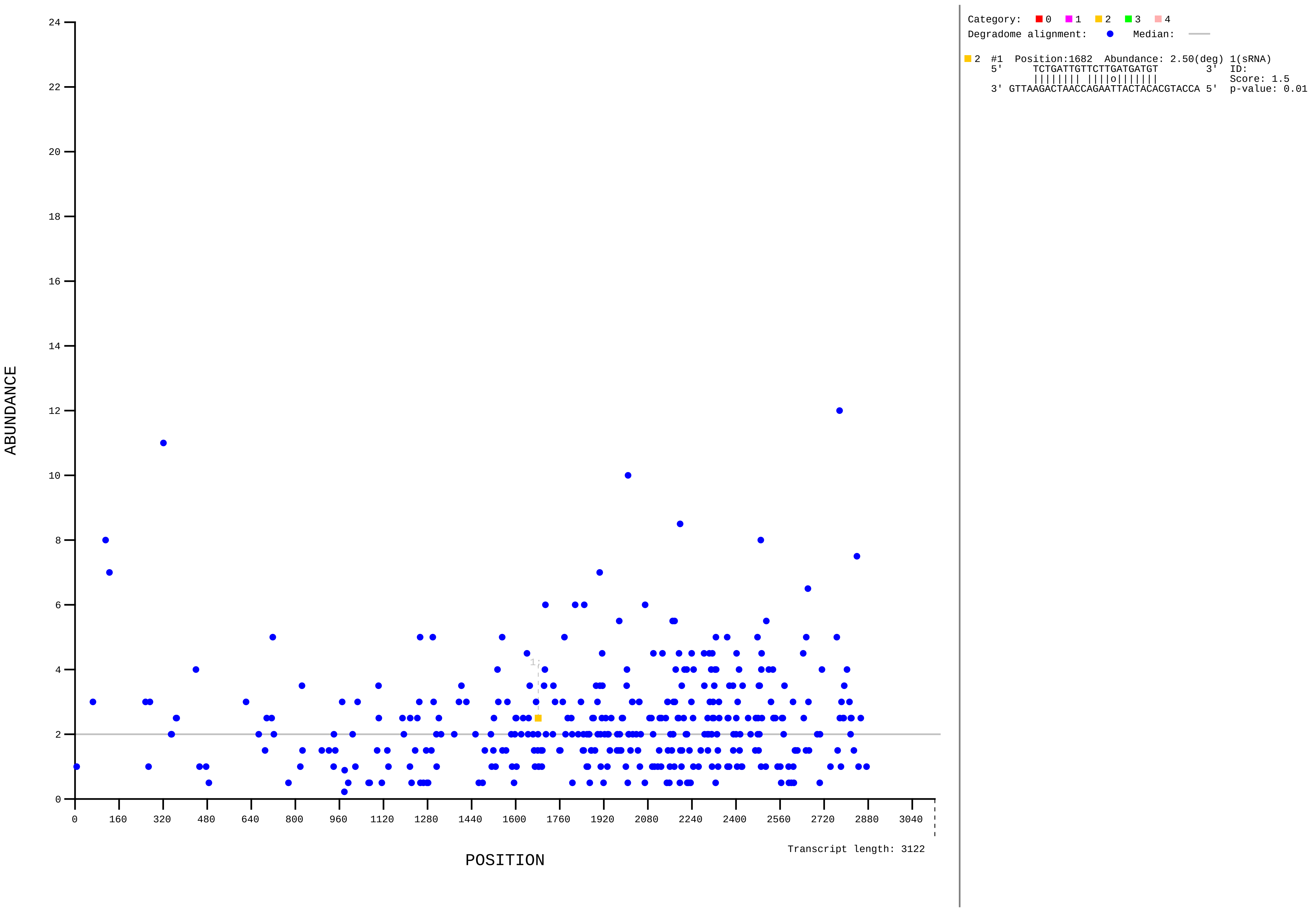

Cs1g11740.2 gene=Cs1g11740 CDS=151-741

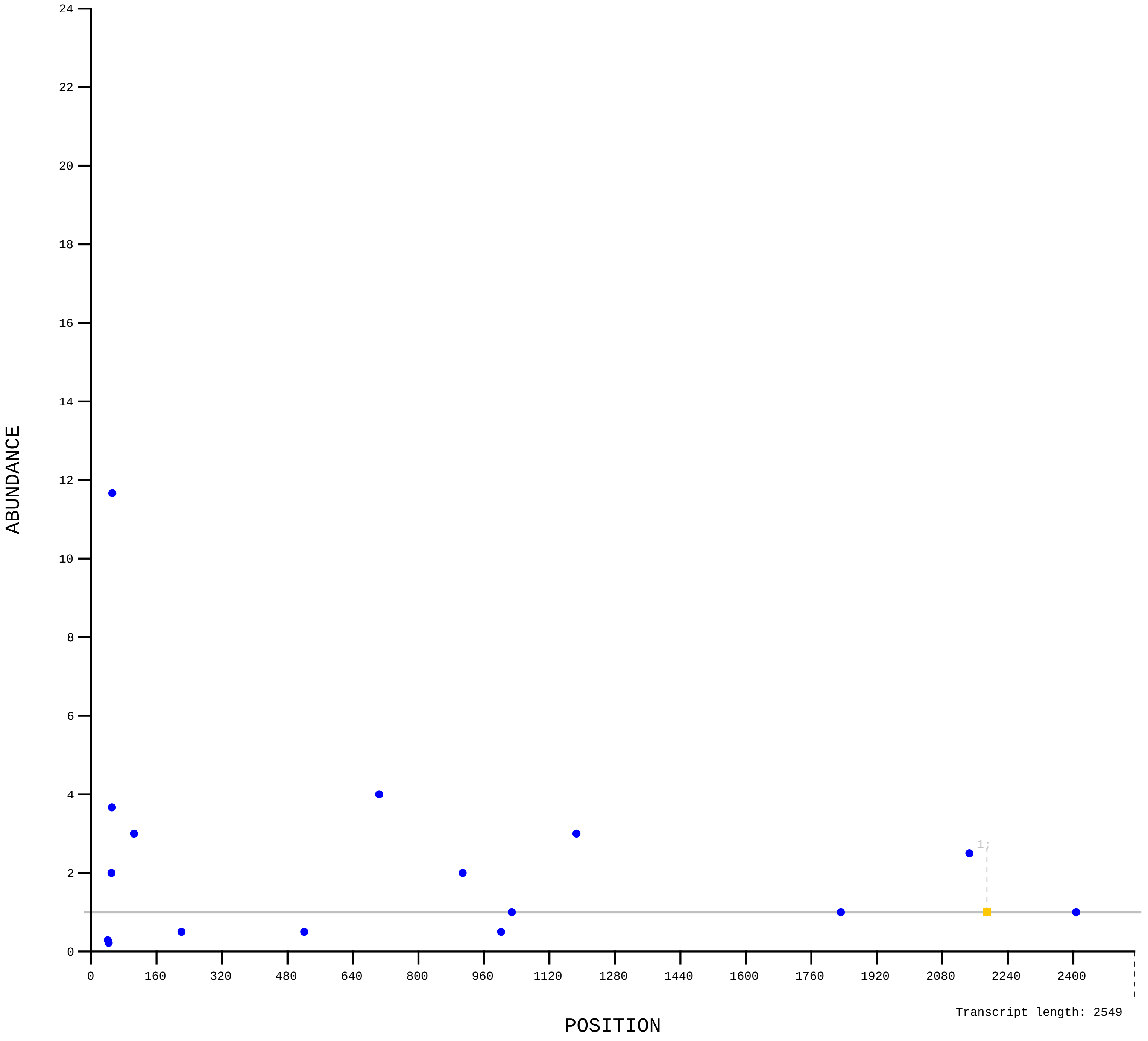

Category: 0 1 2 3 4
Degradome alignment: Median:

```
2  #1  Position:2189  Abundance: 1.00(deg) 1(sRNA)
   5'      TCTTGCCCACCCCTCCCATTCC        3'  ID:
           o|||||||||| ||o||||o ||            Score: 3.5
   3' TGTTGGAACGGGTGAGGGGGGTGCGGCGTCTC 5'  p-value: 0.04
```

Cs7g10830.6 gene=Cs7g10830 CDS=195-1637

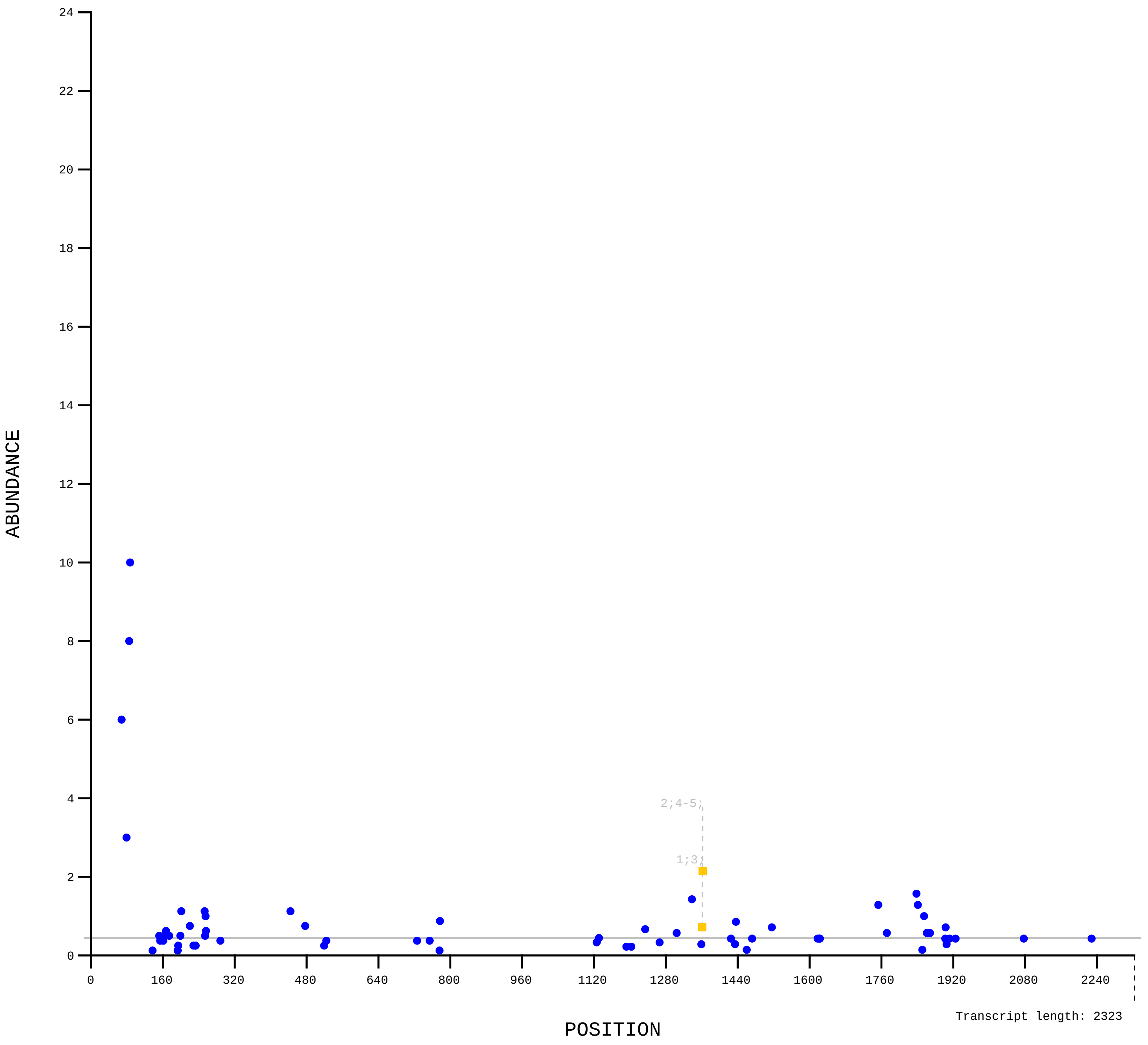

Category: 0 1 2 3 4
Degradome alignment: Median:

```
2  #1  Position:1361  Abundance: 0.71(deg) 1(sRNA)
   5'      TGACAGAAGAGAGTGAGCAC         3'  ID:
           ||||||||||||| ||||||             Score: 1.0
   3' AAAAACTGTCTTCTCTCTCTCGTGATACTGTT 5'  p-value: 0.0

2  #2  Position:1362  Abundance: 2.14(deg) 1(sRNA)
   5'      TTGACAGAAGAGAGTGAGCAC        3'  ID:
           |||||||||||||| ||||||            Score: 1.0
   3' CAAAAACTGTCTTCTCTCTCTCGTGATACTGT 5'  p-value: 0.0

2  #3  Position:1361  Abundance: 0.71(deg) 1(sRNA)
   5'      TGACAGAAGATAGAGAGCGC         3'  ID:
           |||||||||| |||||||o|             Score: 1.5
   3' AAAAACTGTCTTCTCTCTCTCGTGATACTGTT 5'  p-value: 0.0

2  #4  Position:1362  Abundance: 2.14(deg) 1(sRNA)
   5'      CTGACAGAAGAGAGTGAGCAC        3'  ID:
            ||||||||||||| ||||||            Score: 2.0
   3' CAAAAACTGTCTTCTCTCTCTCGTGATACTGT 5'  p-value: 0.0

2  #5  Position:1362  Abundance: 2.14(deg) 1(sRNA)
   5'      GTGACAGAAGATAGAGAGCGC        3'  ID:
            |||||||||| |||||||o|            Score: 2.5
   3' CAAAAACTGTCTTCTCTCTCTCGTGATACTGT 5'  p-value: 0.03
```

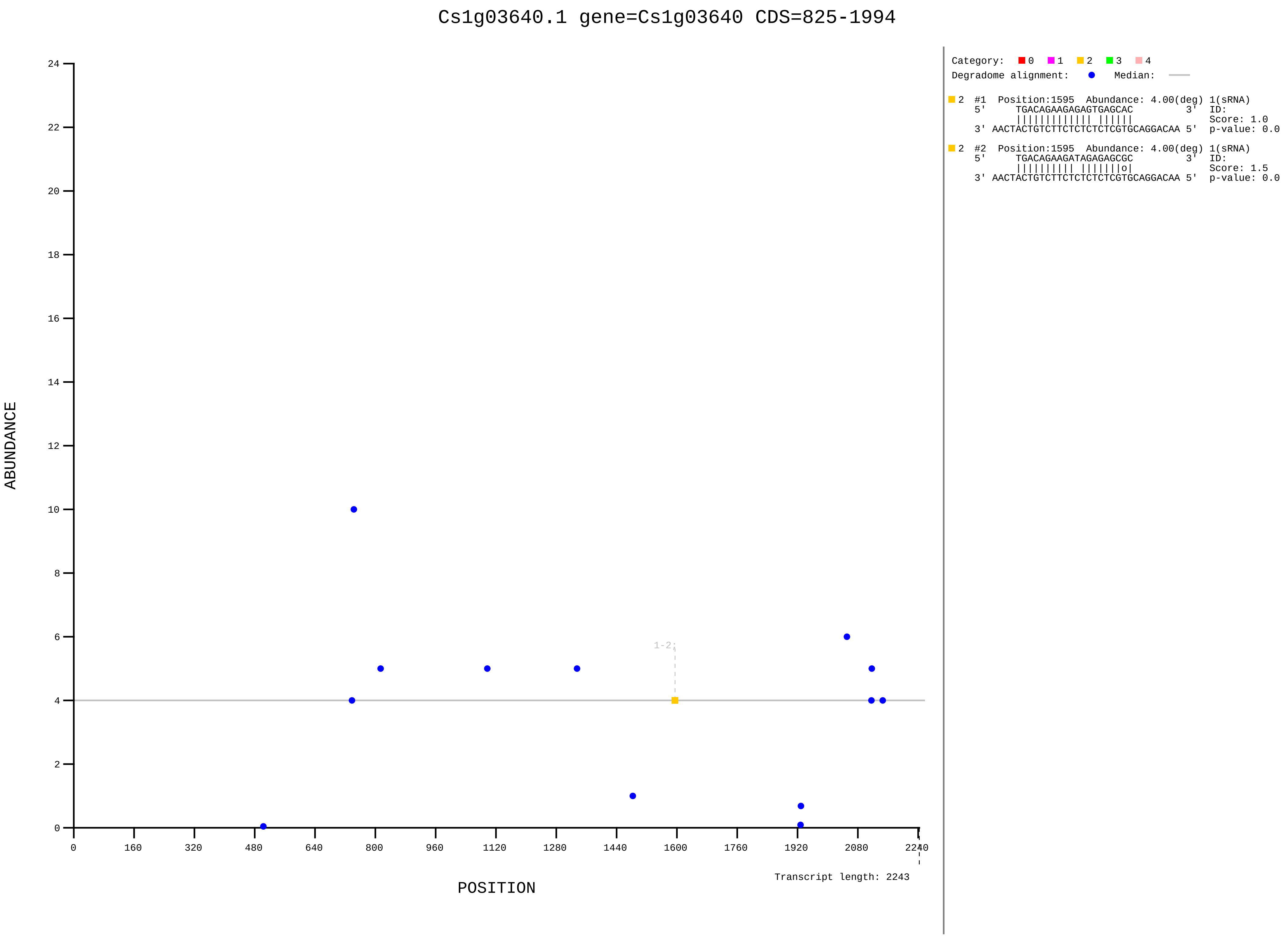
Cs1g03640.1 gene=Cs1g03640 CDS=825-1994
ABUNDANCE
POSITION
Transcript length: 2243
1-2
Category: 0 1 2 3 4
Degradome alignment: Median:
2 #1 Position:1595 Abundance: 4.00(deg) 1(sRNA)
5' TGACAGAAGAGAGTGAGCAC 3' ID:
||||||||||||| ||||||
Score: 1.0
3' AACTACTGTCTTCTCTCTCTCGTGCAGGACAA 5' p-value: 0.0
2 #2 Position:1595 Abundance: 4.00(deg) 1(sRNA)
5' TGACAGAAGATAGAGAGCGC 3' ID:
|||||||||| |||||||o|
Score: 1.5
3' AACTACTGTCTTCTCTCTCTCGTGCAGGACAA 5' p-value: 0.0

Cs7g22470.1 gene=Cs7g22470 CDS=104-2497

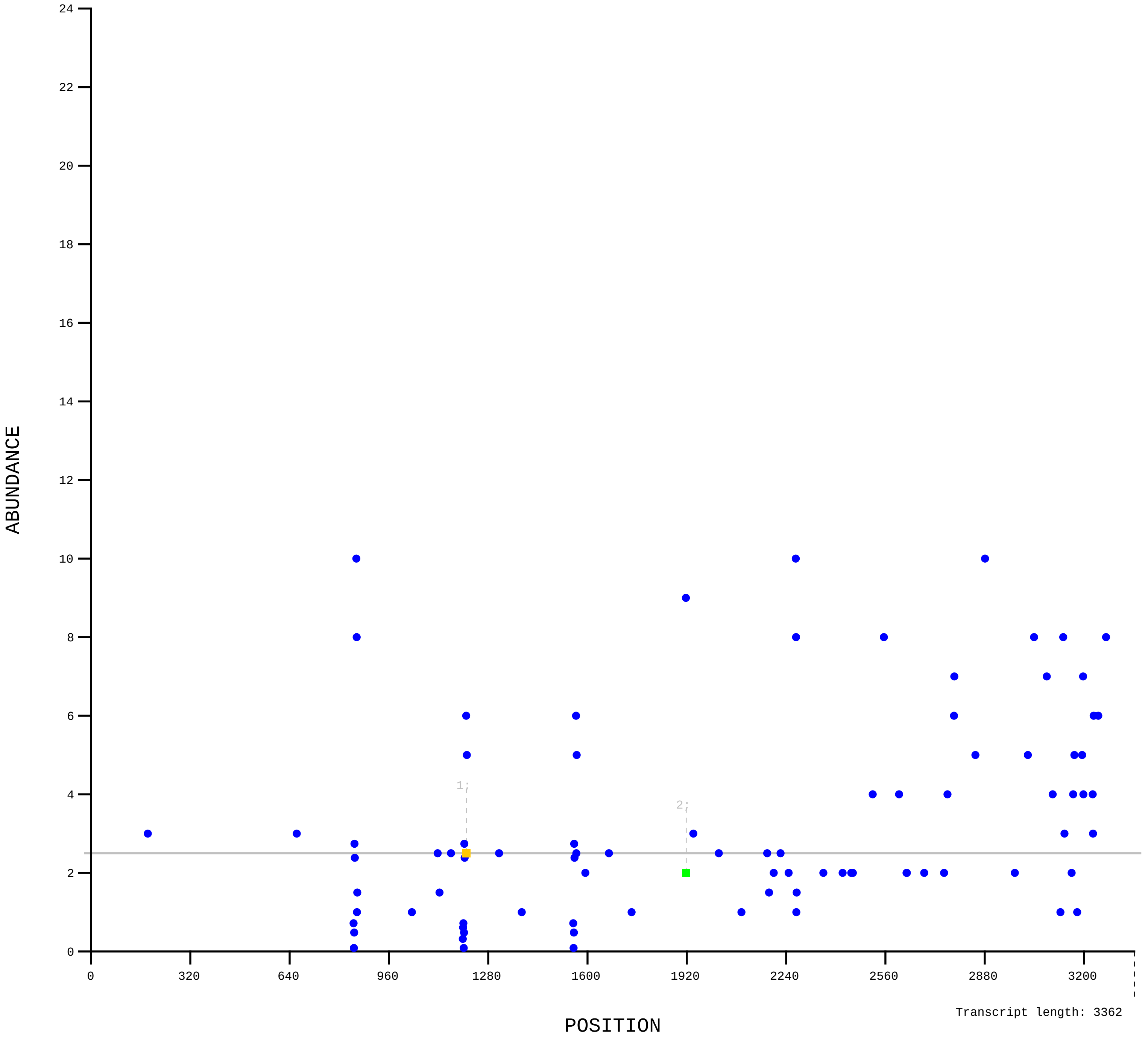

Category: 0 1 2 3 4
Degradome alignment: Median:

2 #1 Position:1210 Abundance: 2.50(deg) 1(sRNA)
```
5'     TTTTTCGGCAACATGATTTCT        3'  ID:
        ||||| |||||||||||||||           Score: 2.0
3' TTCATAAAAGACGTTGTACTAAAGATTTTGTT 5'  p-value: 0.01
```

3 #2 Position:1918 Abundance: 2.00(deg) 1(sRNA)
```
5'     TTTTTCGGCAACATGATTTCT        3'  ID:
        ||||| |||||||||||||o            Score: 2.5
3' TTCACAAAAGACGTTGTACTAAAGGTTTTGTT 5'  p-value: 0.03
```

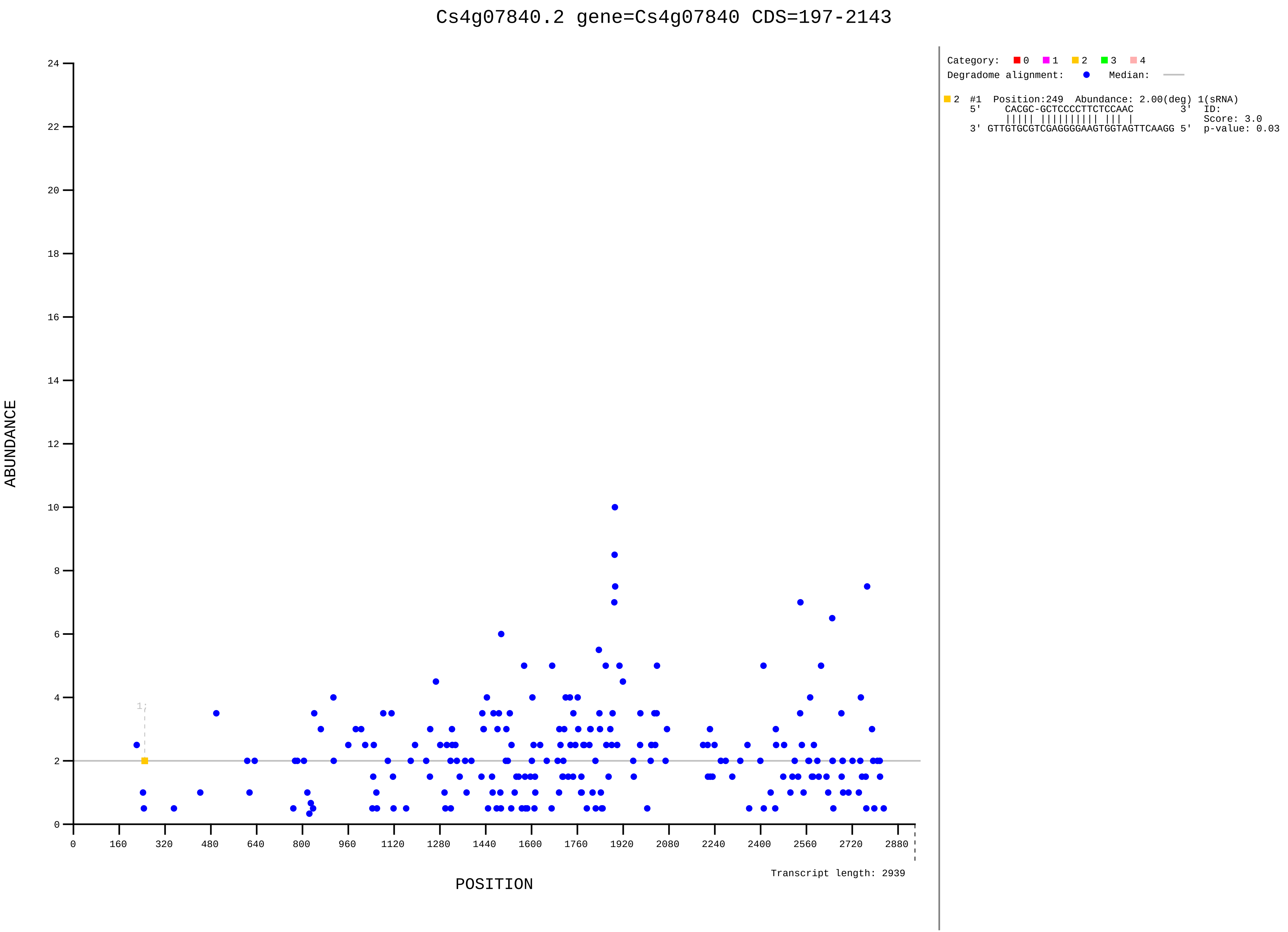
Cs4g07840.2 gene=Cs4g07840 CDS=197-2143
ABUNDANCE
POSITION
Transcript length: 2939
Category: 0 1 2 3 4
Degradome alignment: Median:
2 #1 Position:249 Abundance: 2.00(deg) 1(sRNA)
5' CACGC-GCTCCCCTTCTCCAAC 3' ID:
||||| |||||||||| ||| | Score: 3.0
3' GTTGTGCGTCGAGGGGAAGTGGTAGTTCAAGG 5' p-value: 0.03

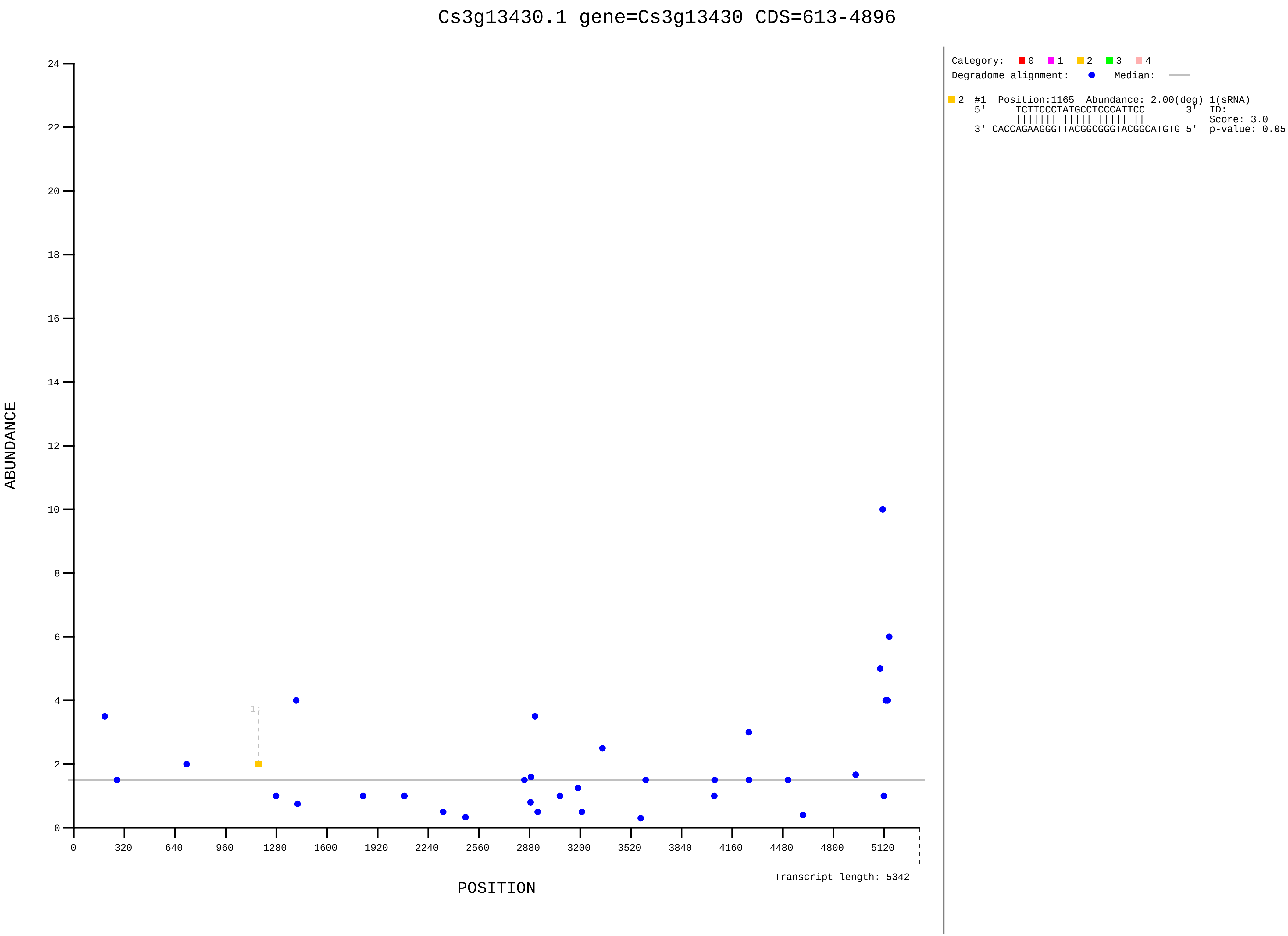
Cs3g13430.1 gene=Cs3g13430 CDS=613-4896
ABUNDANCE
POSITION
Transcript length: 5342
Category: 0 1 2 3 4
Degradome alignment:
Median:
2 #1 Position:1165 Abundance: 2.00(deg) 1(sRNA)
5' TCTTCCCTATGCCTCCCATTCC 3' ID:
||||||| ||||| ||||| ||
3' CACCAGAAGGGTTACGGCGGGTACGGCATGTG 5'
Score: 3.0
p-value: 0.05

orange1.1t03059.6 gene=orange1.1t03059 CDS=285-2258

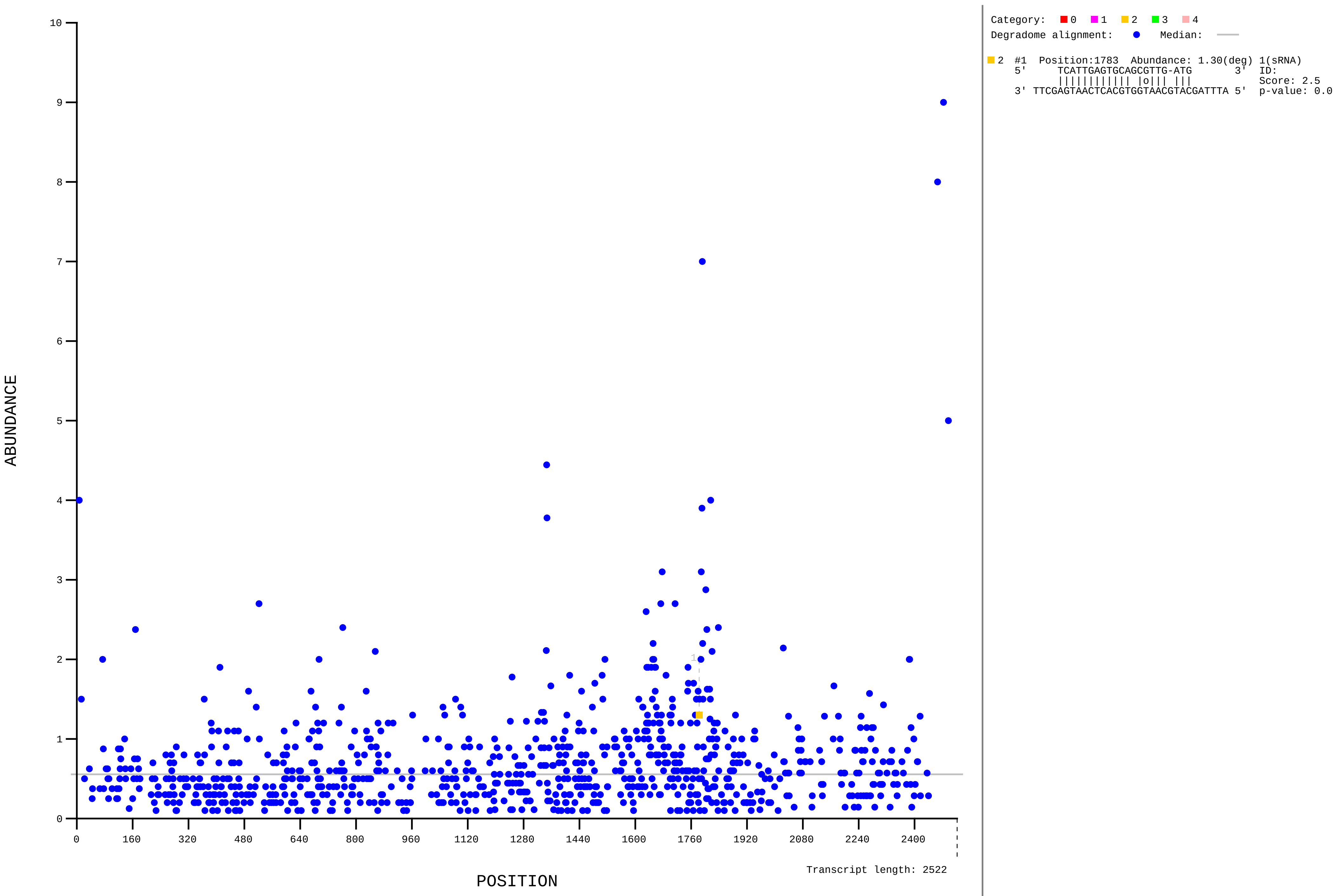

Cs6g02120.1 gene=Cs6g02120 CDS=1-4146

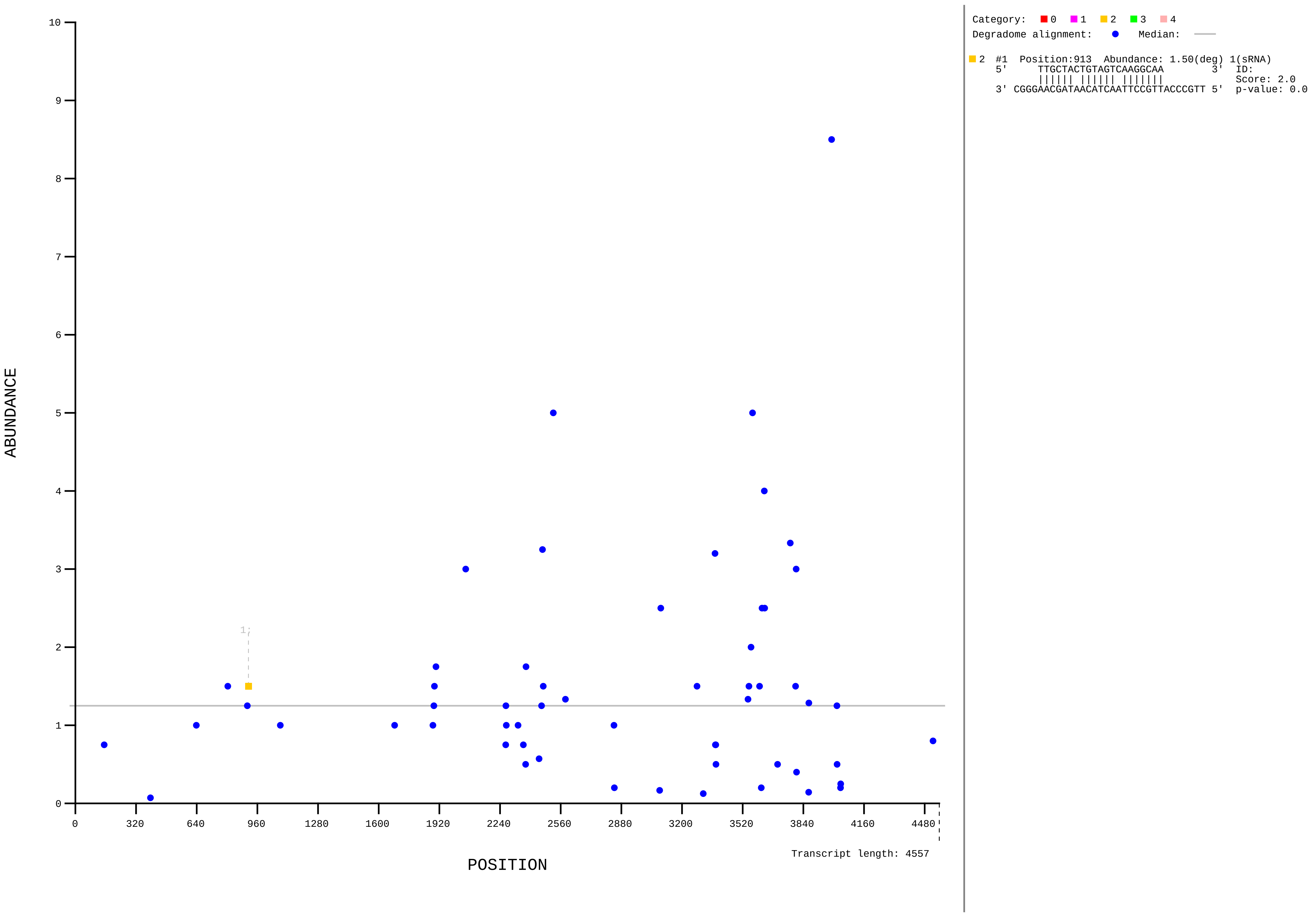

Cs6g02100.2 gene=Cs6g02100 CDS=1-3420

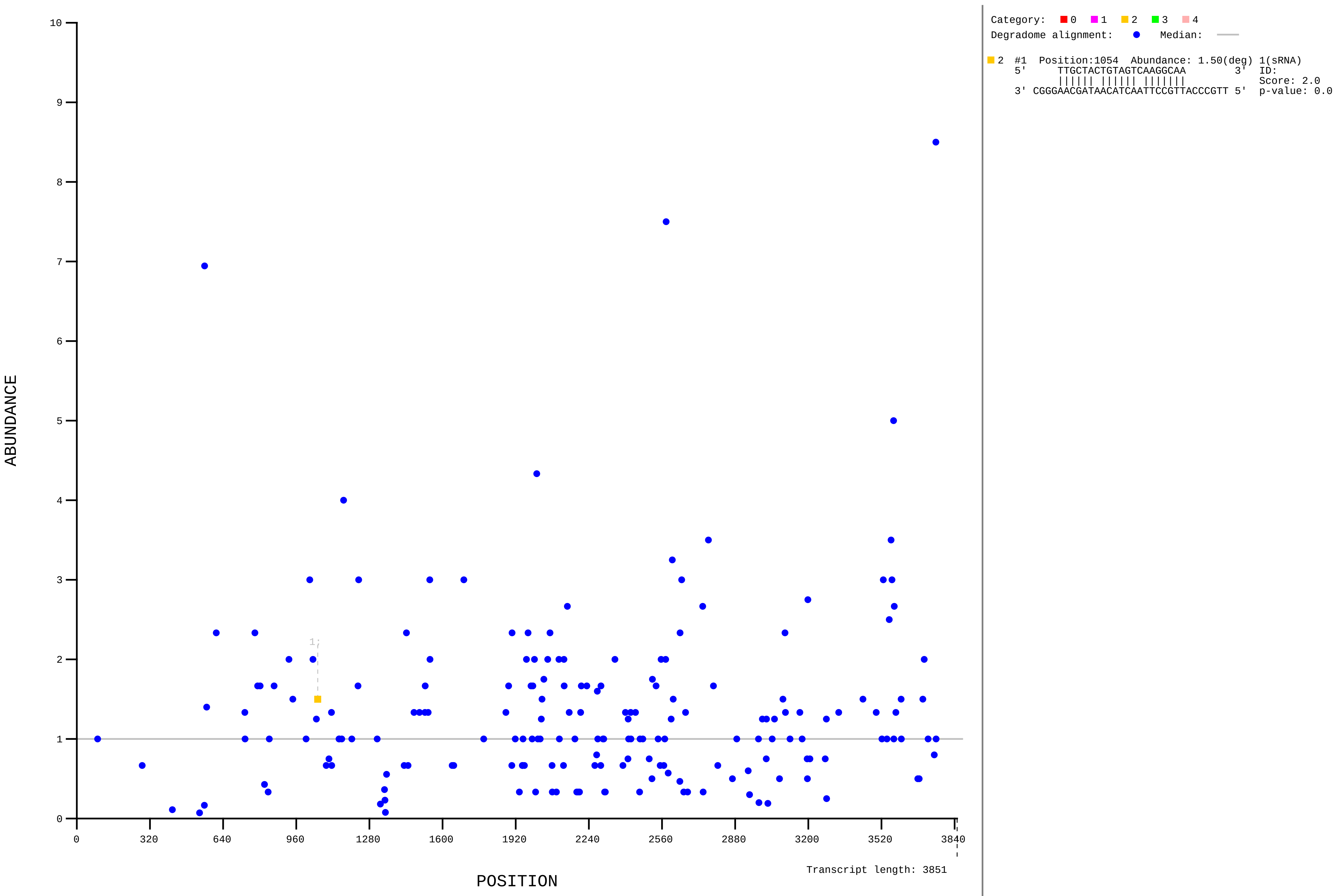

Cs6g02100.1 gene=Cs6g02100 CDS=1-4290

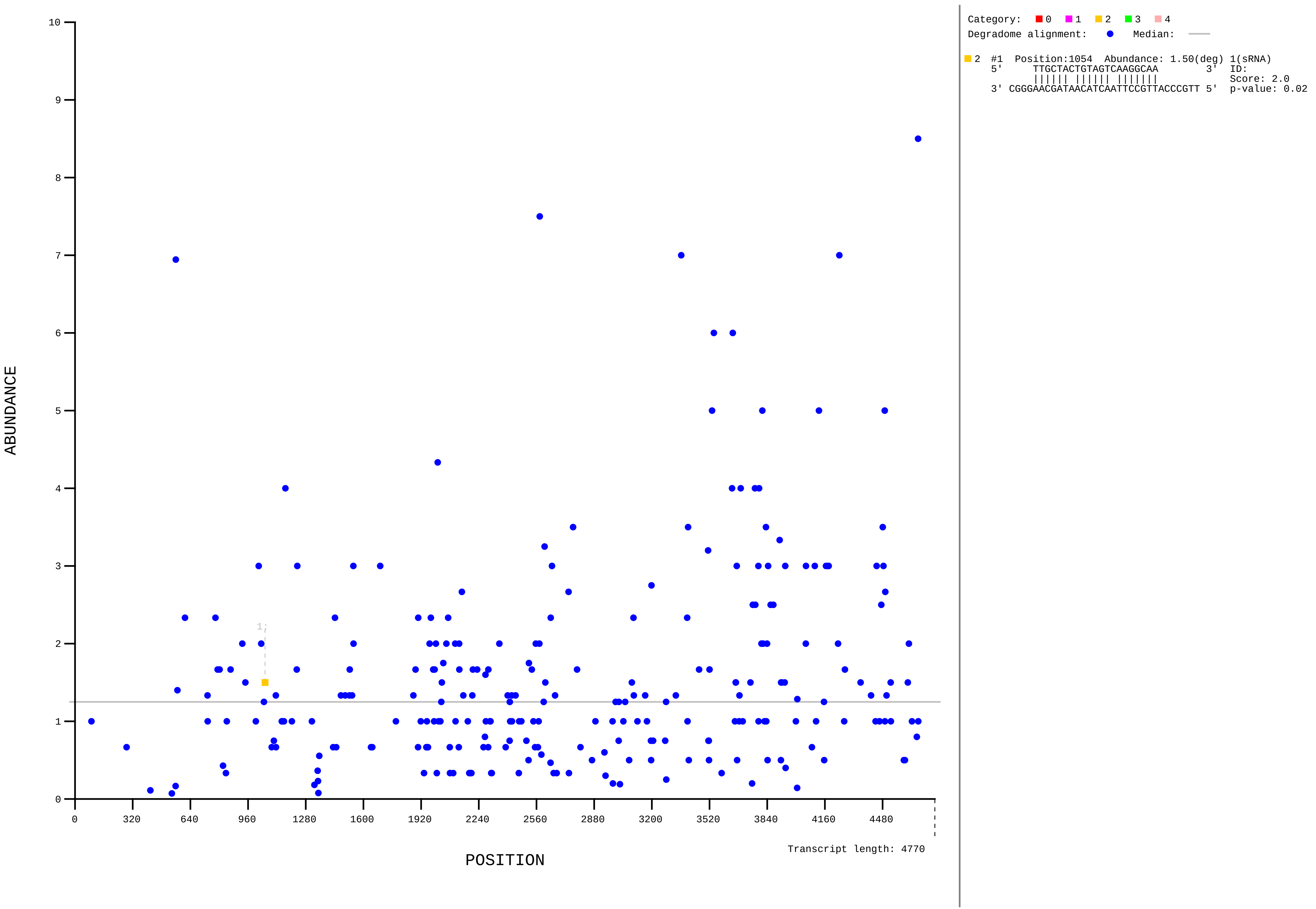

Cs2g06030.2 gene=Cs2g06030 CDS=1325-4183

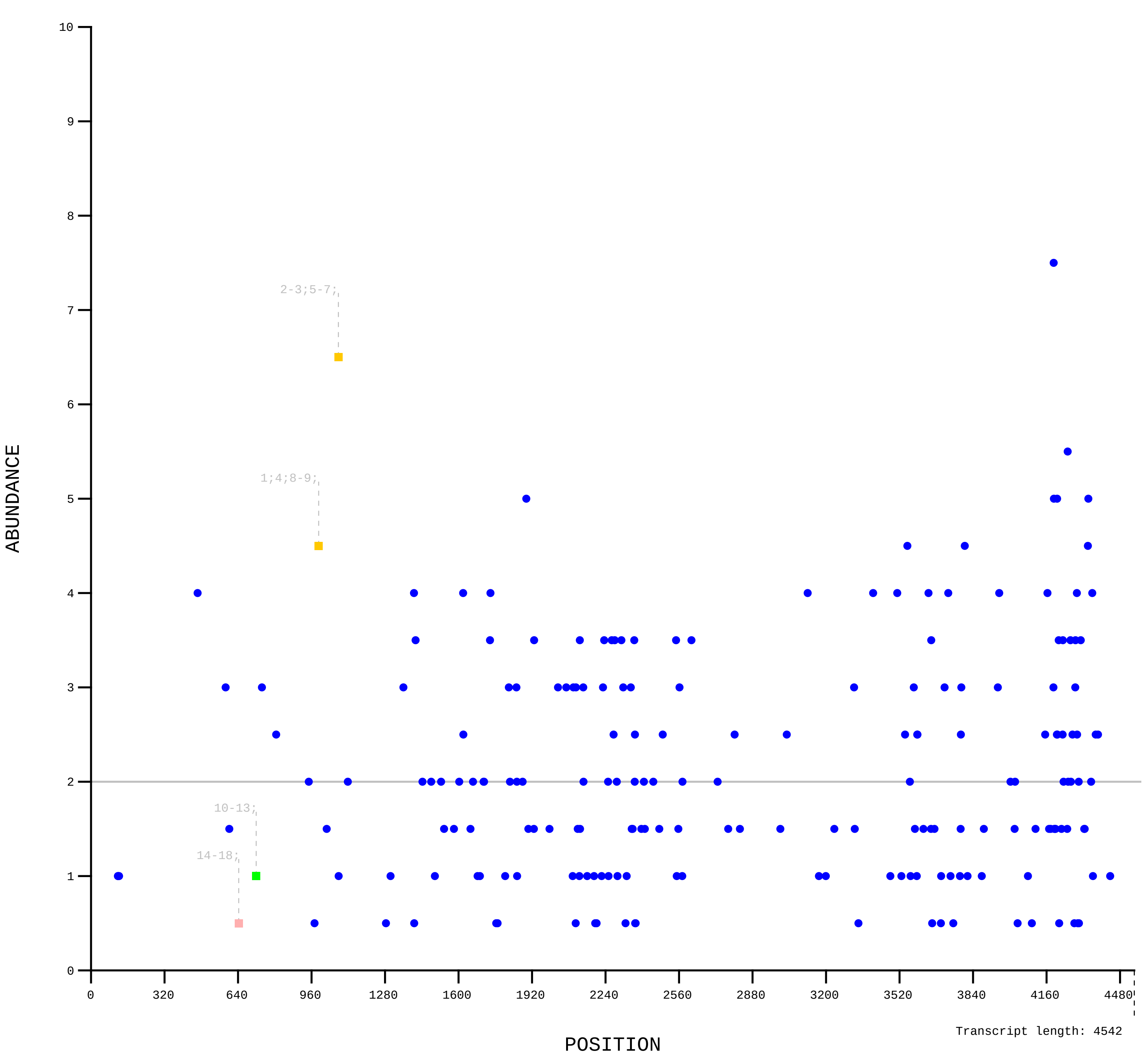


```
Category: ■0 ■1 ■2 ■3 ■4
Degradome alignment: ●   Median: ——

■2  #1  Position:991  Abundance: 4.50(deg) 3(sRNA)
    5'      TGCCAAAGGAGATTTGCCCGG        3'  ID:
            ||||||||||||||||||||oo            Score: 1.0
    3' GTTTACGGTTTCCTCTAAACGGGTTACTTGTC 5'  p-value: 0.0

■2  #2  Position:1077  Abundance: 6.50(deg) 3(sRNA)
    5'      TGCCAAAGGAGATTTGCCCGG        3'  ID:
            |||||||||||||||||||| o            Score: 1.5
    3' GATAACGGTTTCCTCTAAACGGGGTTGTCCCT 5'  p-value: 0.0

■2  #3  Position:1077  Abundance: 6.50(deg) 2(sRNA)
    5'      TGCCAAAGGAGAGTTGCCCTA        3'  ID:
            |||||||||||| ||||||o|            Score: 1.5
    3' GATAACGGTTTCCTCTAAACGGGGTTGTCCCT 5'  p-value: 0.0

■2  #4  Position:991  Abundance: 4.50(deg) 2(sRNA)
    5'      TGCCAAAGGAGAGTTGCCCTA        3'  ID:
            |||||||||||| |||||| |            Score: 2.0
    3' GTTTACGGTTTCCTCTAAACGGGTTACTTGTC 5'  p-value: 0.02

■2  #5  Position:1077  Abundance: 6.50(deg) 2(sRNA)
    5'      CGCCAAAGGAGAATTGCCCTG        3'  ID:
             ||||||||||| ||||||oo            Score: 3.0
    3' GATAACGGTTTCCTCTAAACGGGGTTGTCCCT 5'  p-value: 0.03

■2  #6  Position:1077  Abundance: 6.50(deg) 1(sRNA)
    5'      TGCCAAAGGAGAGTTGCCCTG        3'  ID:
            |||||||||||| ||||||oo            Score: 2.0
    3' GATAACGGTTTCCTCTAAACGGGGTTGTCCCT 5'  p-value: 0.0

■2  #7  Position:1077  Abundance: 6.50(deg) 1(sRNA)
    5'      TGCCAAAGGAGAATTGCCCTG        3'  ID:
            |||||||||||| ||||||oo            Score: 2.0
    3' GATAACGGTTTCCTCTAAACGGGGTTGTCCCT 5'  p-value: 0.02

■2  #8  Position:991  Abundance: 4.50(deg) 1(sRNA)
    5'      TGCCAAAGGAGAGTTGCCCTG        3'  ID:
            |||||||||||| |||||| o            Score: 2.5
    3' GTTTACGGTTTCCTCTAAACGGGTTACTTGTC 5'  p-value: 0.01

■2  #9  Position:991  Abundance: 4.50(deg) 1(sRNA)
    5'      TGCCAAAGGAGAATTGCCCTG        3'  ID:
            |||||||||||| |||||| o            Score: 2.5
    3' GTTTACGGTTTCCTCTAAACGGGTTACTTGTC 5'  p-value: 0.03

■3  #10  Position:719  Abundance: 1.00(deg) 3(sRNA)
    5'      TGCCAAAGGAGATTTGCCCGG        3'  ID:
            |||||||||||||||||||oo            Score: 1.0
    3' AAAAACGGTTTCCTCTAAACGGGTTGTCACCT 5'  p-value: 0.0

■3  #11  Position:719  Abundance: 1.00(deg) 2(sRNA)
    5'      TGCCAAAGGAGAGTTGCCCTA        3'  ID:
            |||||||||||| |||||| |            Score: 2.0
    3' AAAAACGGTTTCCTCTAAACGGGTTGTCACCT 5'  p-value: 0.0

■3  #12  Position:719  Abundance: 1.00(deg) 1(sRNA)
    5'      TGCCAAAGGAGAGTTGCCCTG        3'  ID:
            |||||||||||| |||||| o            Score: 2.5
    3' AAAAACGGTTTCCTCTAAACGGGTTGTCACCT 5'  p-value: 0.01

■3  #13  Position:719  Abundance: 1.00(deg) 1(sRNA)
    5'      TGCCAAAGGAGAATTGCCCTG        3'  ID:
            |||||||||||| |||||| o            Score: 2.5
    3' AAAAACGGTTTCCTCTAAACGGGTTGTCACCT 5'  p-value: 0.0

■4  #14  Position:643  Abundance: 0.50(deg) 3(sRNA)
    5'      TGCCAAAGGAGATTTGCCCGG        3'  ID:
            |||||||||||||||||||| o            Score: 1.5
    3' AATAACGGTTTCCTCTAAACGGGGTTTGACTT 5'  p-value: 0.0

■4  #15  Position:643  Abundance: 0.50(deg) 2(sRNA)
    5'      TGCCAAAGGAGAGTTGCCCTA        3'  ID:
            |||||||||||| ||||||o|            Score: 1.5
    3' AATAACGGTTTCCTCTAAACGGGGTTTGACTT 5'  p-value: 0.0

■4  #16  Position:643  Abundance: 0.50(deg) 2(sRNA)
    5'      CGCCAAAGGAGAATTGCCCTG        3'  ID:
             ||||||||||| ||||||oo            Score: 3.0
    3' AATAACGGTTTCCTCTAAACGGGGTTTGACTT 5'  p-value: 0.01

■4  #17  Position:643  Abundance: 0.50(deg) 1(sRNA)
    5'      TGCCAAAGGAGAGTTGCCCTG        3'  ID:
            |||||||||||| ||||||oo            Score: 2.0
    3' AATAACGGTTTCCTCTAAACGGGGTTTGACTT 5'  p-value: 0.0
```

List truncated - shown 17 of 18

Cs2g06030.1 gene=Cs2g06030 CDS=1325-4045

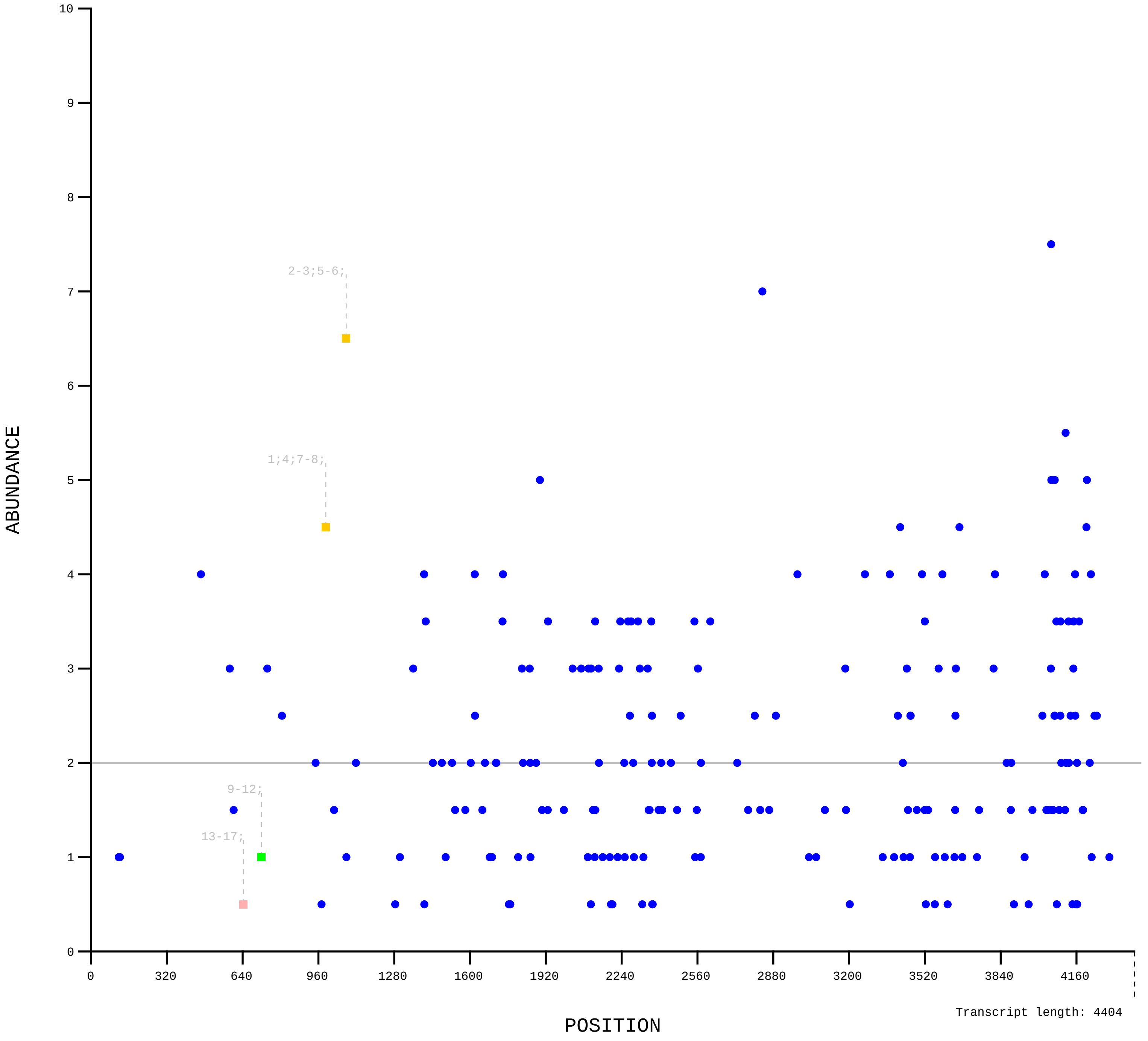

Category: 0 1 2 3 4
Degradome alignment: ● Median: —

```
2  #1  Position:991  Abundance: 4.50(deg) 3(sRNA)
   5'     TGCCAAAGGAGATTTGCCCGG        3'  ID:
          |||||||||||||||||||oo            Score: 1.0
   3' GTTTACGGTTTCCTCTAAACGGGTTACTTGTC 5'  p-value: 0.0

2  #2  Position:1077  Abundance: 6.50(deg) 3(sRNA)
   5'     TGCCAAAGGAGATTTGCCCGG        3'  ID:
          ||||||||||||||||||| o            Score: 1.5
   3' GATAACGGTTTCCTCTAAACGGGGTTGTCCCT 5'  p-value: 0.0

2  #3  Position:1077  Abundance: 6.50(deg) 2(sRNA)
   5'     TGCCAAAGGAGAGTTGCCCTA        3'  ID:
          |||||||||||| ||||||o|            Score: 1.5
   3' GATAACGGTTTCCTCTAAACGGGGTTGTCCCT 5'  p-value: 0.0

2  #4  Position:991  Abundance: 4.50(deg) 2(sRNA)
   5'     TGCCAAAGGAGAGTTGCCCTA        3'  ID:
          |||||||||||| |||||| |            Score: 2.0
   3' GTTTACGGTTTCCTCTAAACGGGTTACTTGTC 5'  p-value: 0.0

2  #5  Position:1077  Abundance: 6.50(deg) 1(sRNA)
   5'     TGCCAAAGGAGAGTTGCCCTG        3'  ID:
          |||||||||||| ||||||oo            Score: 2.0
   3' GATAACGGTTTCCTCTAAACGGGGTTGTCCCT 5'  p-value: 0.01

2  #6  Position:1077  Abundance: 6.50(deg) 1(sRNA)
   5'     TGCCAAAGGAGAATTGCCCTG        3'  ID:
          |||||||||||| ||||||oo            Score: 2.0
   3' GATAACGGTTTCCTCTAAACGGGGTTGTCCCT 5'  p-value: 0.01

2  #7  Position:991  Abundance: 4.50(deg) 1(sRNA)
   5'     TGCCAAAGGAGAGTTGCCCTG        3'  ID:
          |||||||||||| |||||| o            Score: 2.5
   3' GTTTACGGTTTCCTCTAAACGGGTTACTTGTC 5'  p-value: 0.03

2  #8  Position:991  Abundance: 4.50(deg) 1(sRNA)
   5'     TGCCAAAGGAGAATTGCCCTG        3'  ID:
          |||||||||||| |||||| o            Score: 2.5
   3' GTTTACGGTTTCCTCTAAACGGGTTACTTGTC 5'  p-value: 0.01

3  #9  Position:719  Abundance: 1.00(deg) 3(sRNA)
   5'     TGCCAAAGGAGATTTGCCCGG        3'  ID:
          |||||||||||||||||||oo            Score: 1.0
   3' AAAAACGGTTTCCTCTAAACGGGTTGTCACCT 5'  p-value: 0.0

3  #10  Position:719  Abundance: 1.00(deg) 2(sRNA)
   5'     TGCCAAAGGAGAGTTGCCCTA        3'  ID:
          |||||||||||| |||||| |            Score: 2.0
   3' AAAAACGGTTTCCTCTAAACGGGTTGTCACCT 5'  p-value: 0.0

3  #11  Position:719  Abundance: 1.00(deg) 1(sRNA)
   5'     TGCCAAAGGAGAGTTGCCCTG        3'  ID:
          |||||||||||| |||||| o            Score: 2.5
   3' AAAAACGGTTTCCTCTAAACGGGTTGTCACCT 5'  p-value: 0.01

3  #12  Position:719  Abundance: 1.00(deg) 1(sRNA)
   5'     TGCCAAAGGAGAATTGCCCTG        3'  ID:
          |||||||||||| |||||| o            Score: 2.5
   3' AAAAACGGTTTCCTCTAAACGGGTTGTCACCT 5'  p-value: 0.01

4  #13  Position:643  Abundance: 0.50(deg) 3(sRNA)
   5'     TGCCAAAGGAGATTTGCCCGG        3'  ID:
          ||||||||||||||||||| o            Score: 1.5
   3' AATAACGGTTTCCTCTAAACGGGGTTTGACTT 5'  p-value: 0.0

4  #14  Position:643  Abundance: 0.50(deg) 2(sRNA)
   5'     TGCCAAAGGAGAGTTGCCCTA        3'  ID:
          |||||||||||| ||||||o|            Score: 1.5
   3' AATAACGGTTTCCTCTAAACGGGGTTTGACTT 5'  p-value: 0.0

4  #15  Position:643  Abundance: 0.50(deg) 2(sRNA)
   5'     CGCCAAAGGAGAATTGCCCTG        3'  ID:
           ||||||||||| ||||||oo            Score: 3.0
   3' AATAACGGTTTCCTCTAAACGGGGTTTGACTT 5'  p-value: 0.0

4  #16  Position:643  Abundance: 0.50(deg) 1(sRNA)
   5'     TGCCAAAGGAGAGTTGCCCTG        3'  ID:
          |||||||||||| ||||||oo            Score: 2.0
   3' AATAACGGTTTCCTCTAAACGGGGTTTGACTT 5'  p-value: 0.0

4  #17  Position:643  Abundance: 0.50(deg) 1(sRNA)
   5'     TGCCAAAGGAGAATTGCCCTG        3'  ID:
          |||||||||||| ||||||oo            Score: 2.0
   3' AATAACGGTTTCCTCTAAACGGGGTTTGACTT 5'  p-value: 0.0
```

Cs1g06080.1 gene=Cs1g06080 CDS=133-1077

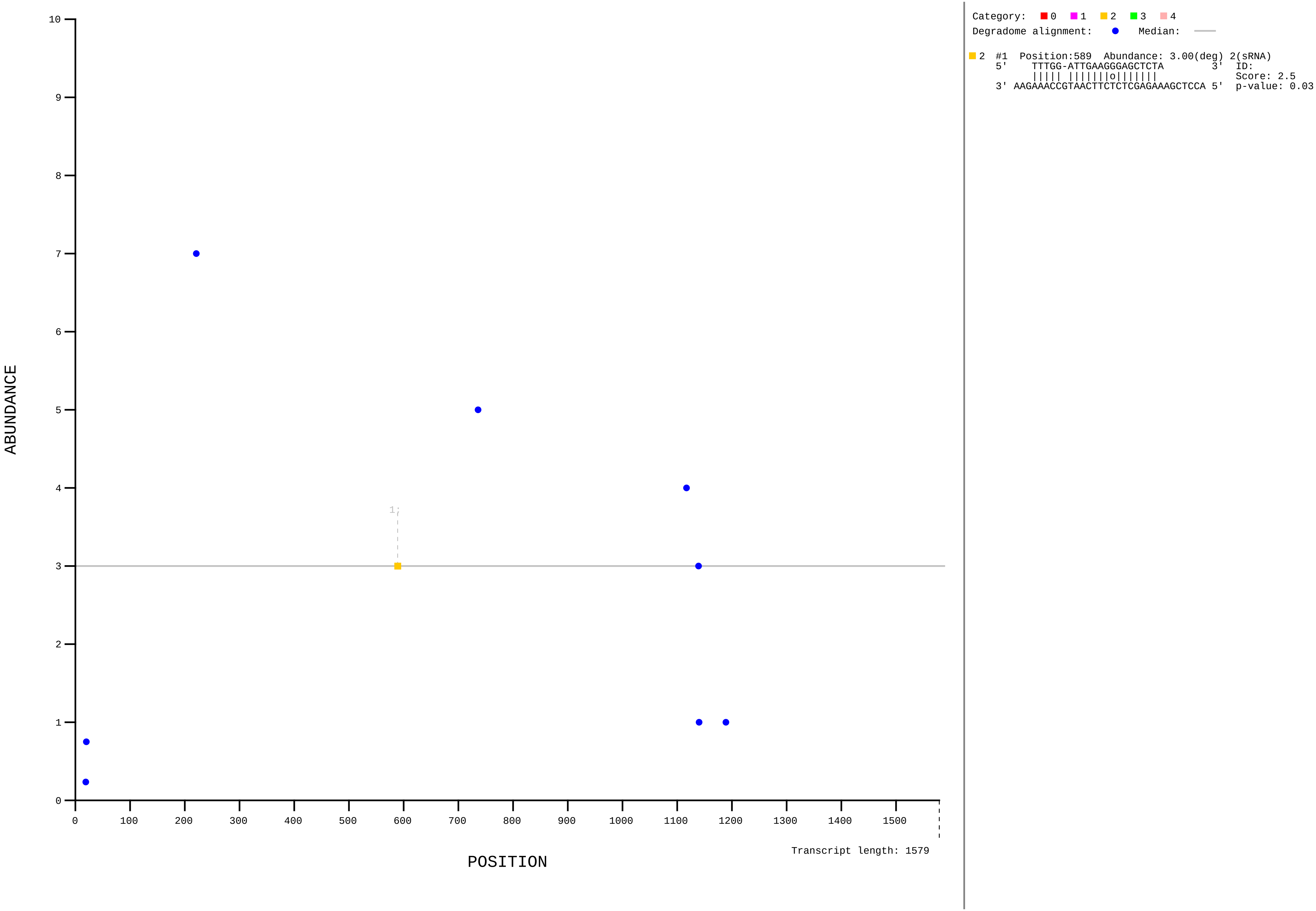

Cs1g19720.1 gene=Cs1g19720 CDS=236-1567

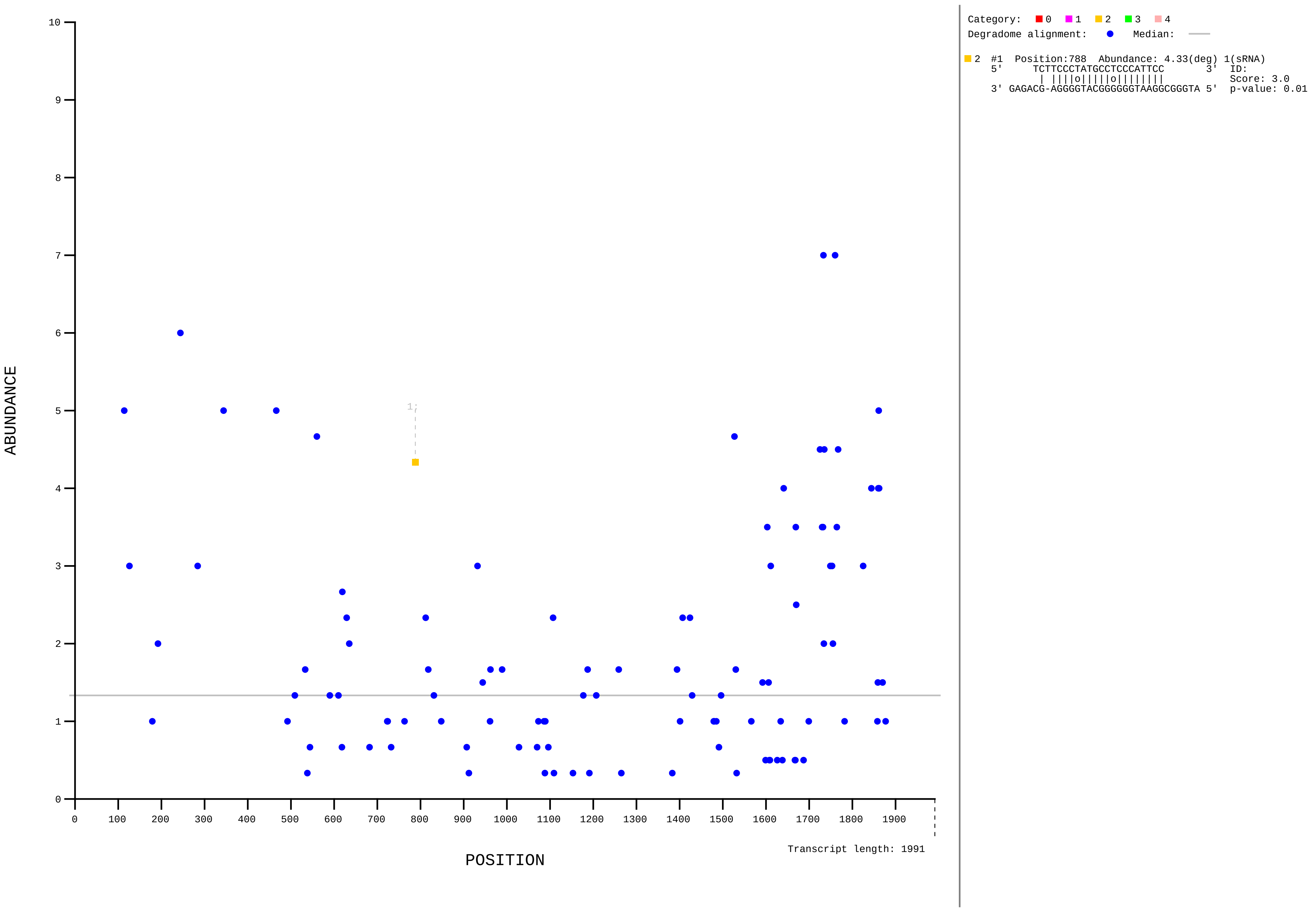

orange1.1t03059.10 gene=orange1.1t03059 CDS=257-2026

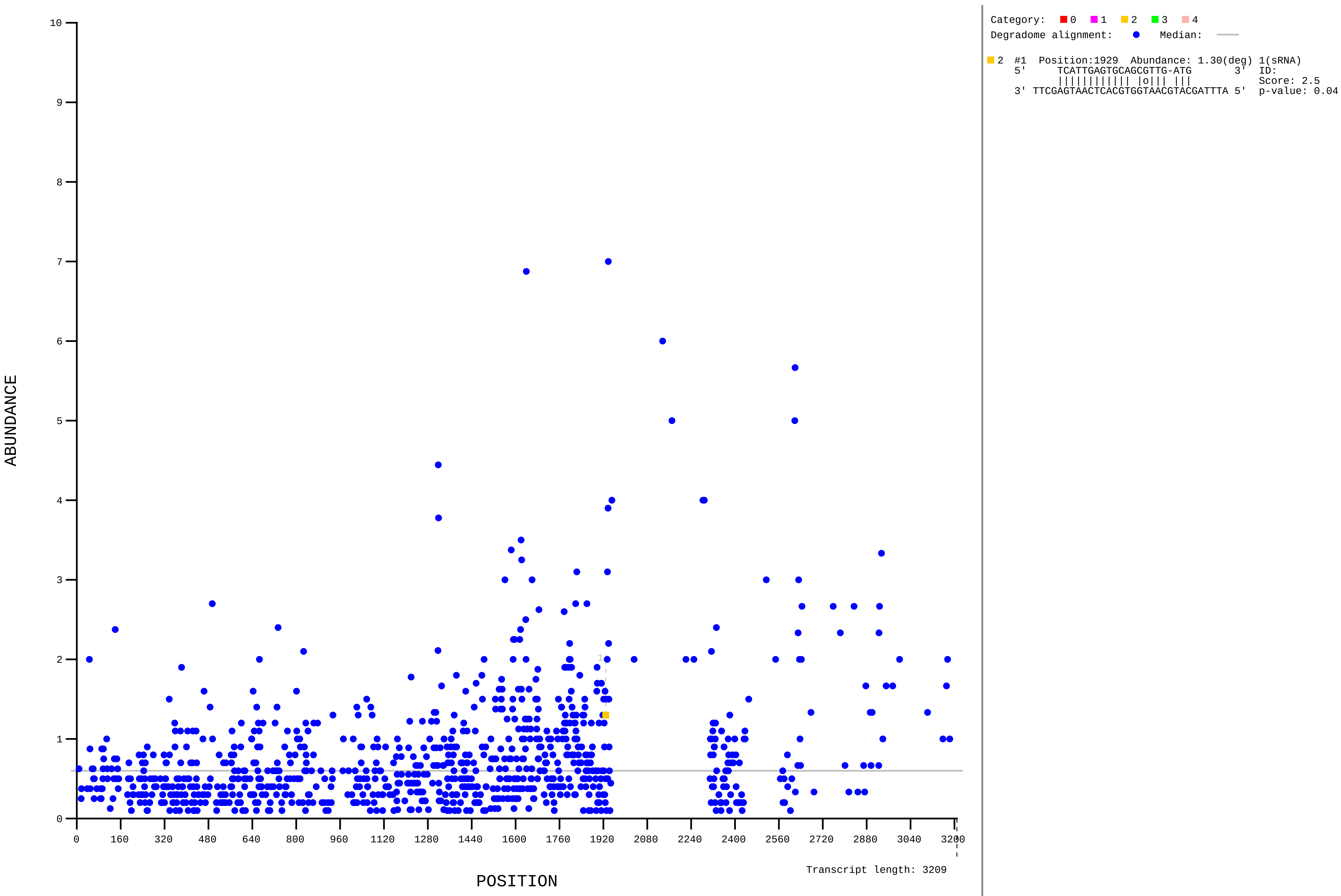

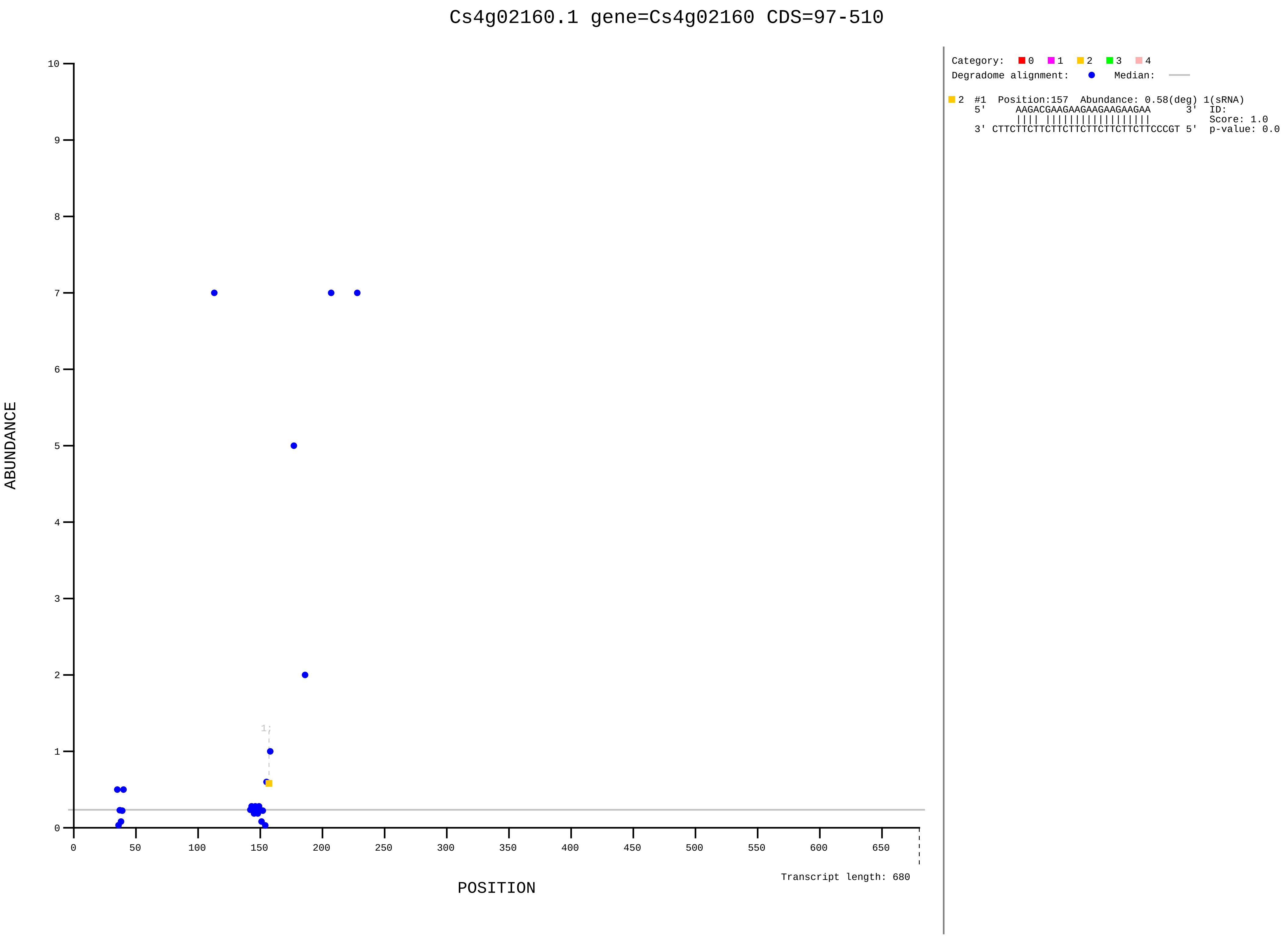

Cs4g02160.1 gene=Cs4g02160 CDS=97-510
ABUNDANCE
POSITION
Transcript length: 680
Category: 0 1 2 3 4
Degradome alignment:
Median:
2 #1 Position:157 Abundance: 0.58(deg) 1(sRNA)
5' AAGACGAAGAAGAAGAAGAAGAA 3' ID:
3' CTTCTTCTTCTTCTTCTTCTTCTTCTTCCCGT 5' p-value: 0.0
Score: 1.0

Cs1g19720.2 gene=Cs1g19720 CDS=12-1136

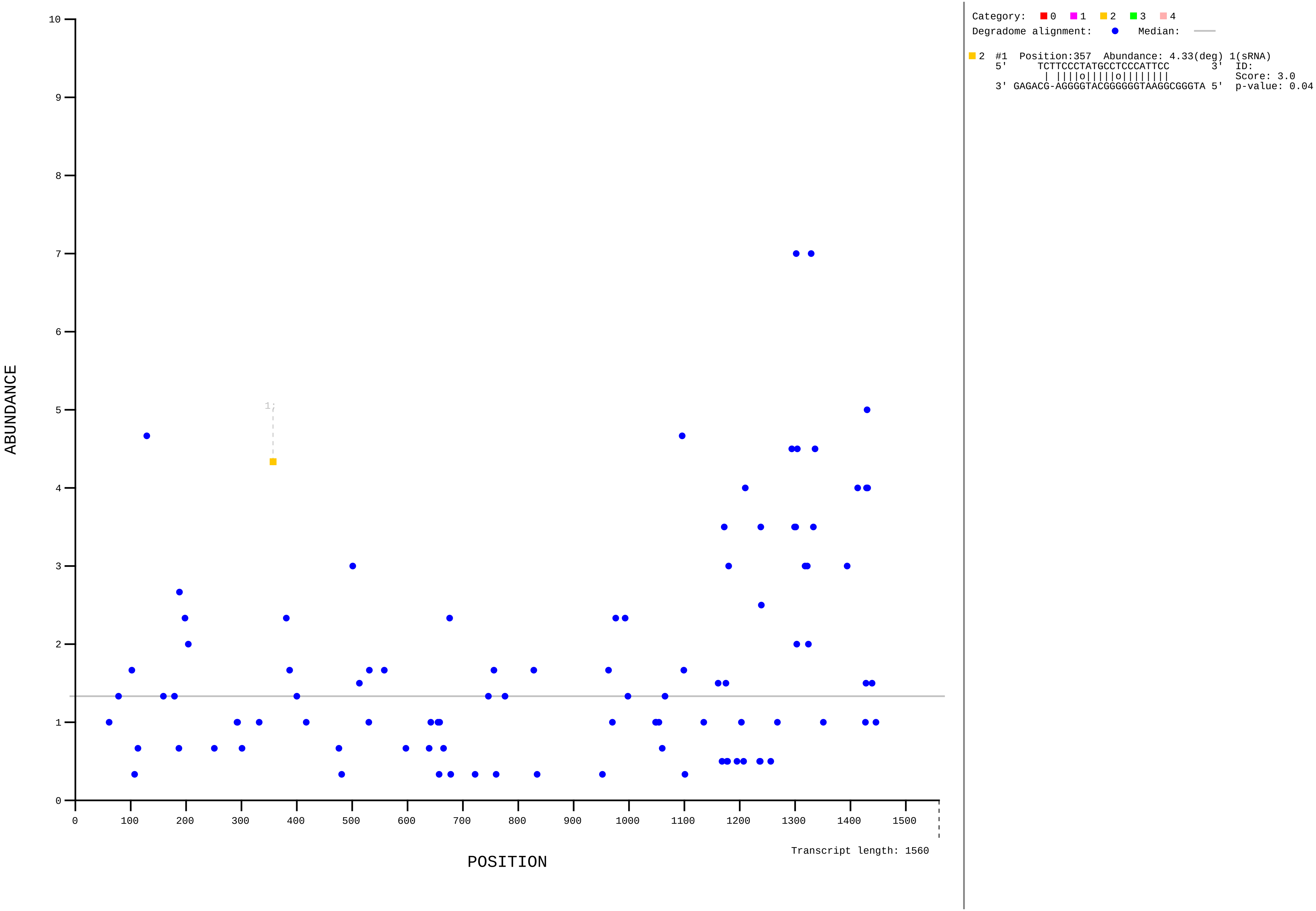

orange1.1t03059.5 gene=orange1.1t03059 CDS=515-2662

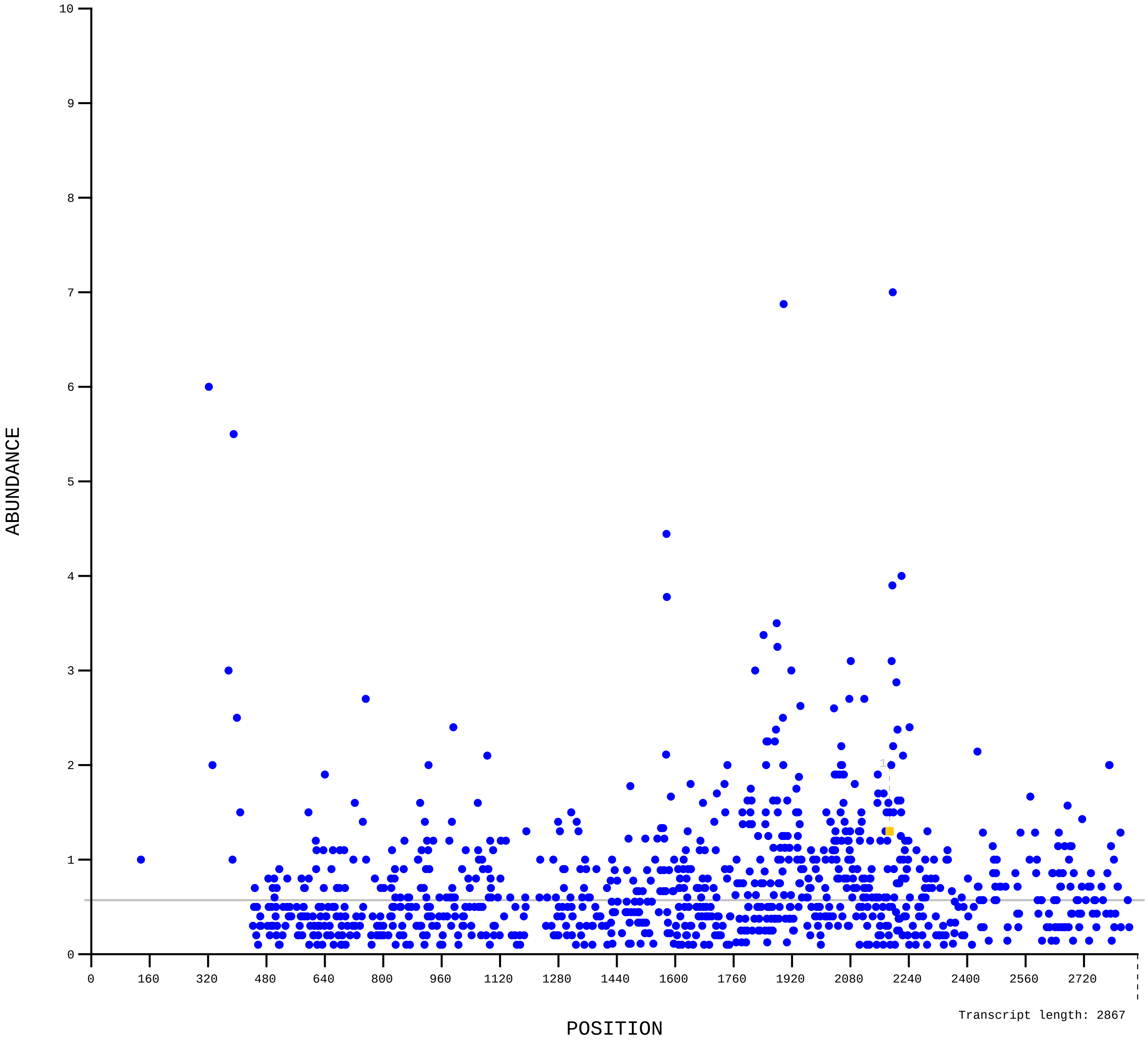

Category: 0 1 2 3 4
Degradome alignment: Median:

```
2  #1  Position:2187  Abundance: 1.30(deg) 1(sRNA)
   5'     TCATTGAGTGCAGCGTTG-ATG       3'  ID:
          |||||||||||| |o||| |||           Score: 2.5
   3' TTCGAGTAACTCACGTGGTAACGTACGATTTA 5'  p-value: 0.01
```

orange1.1t03059.9 gene=orange1.1t03059 CDS=257-2023

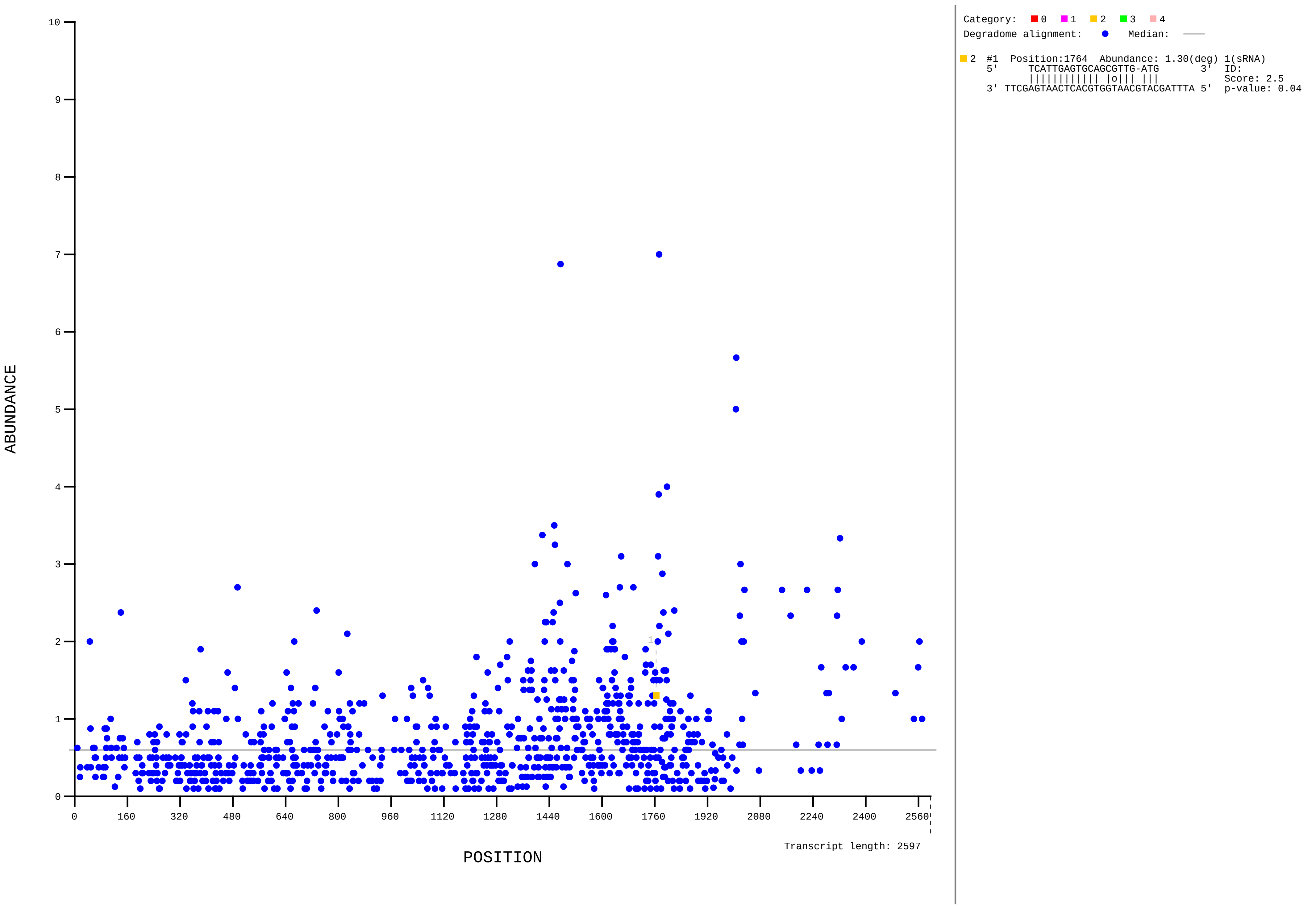

orange1.1t03059.7 gene=orange1.1t03059 CDS=257-2188

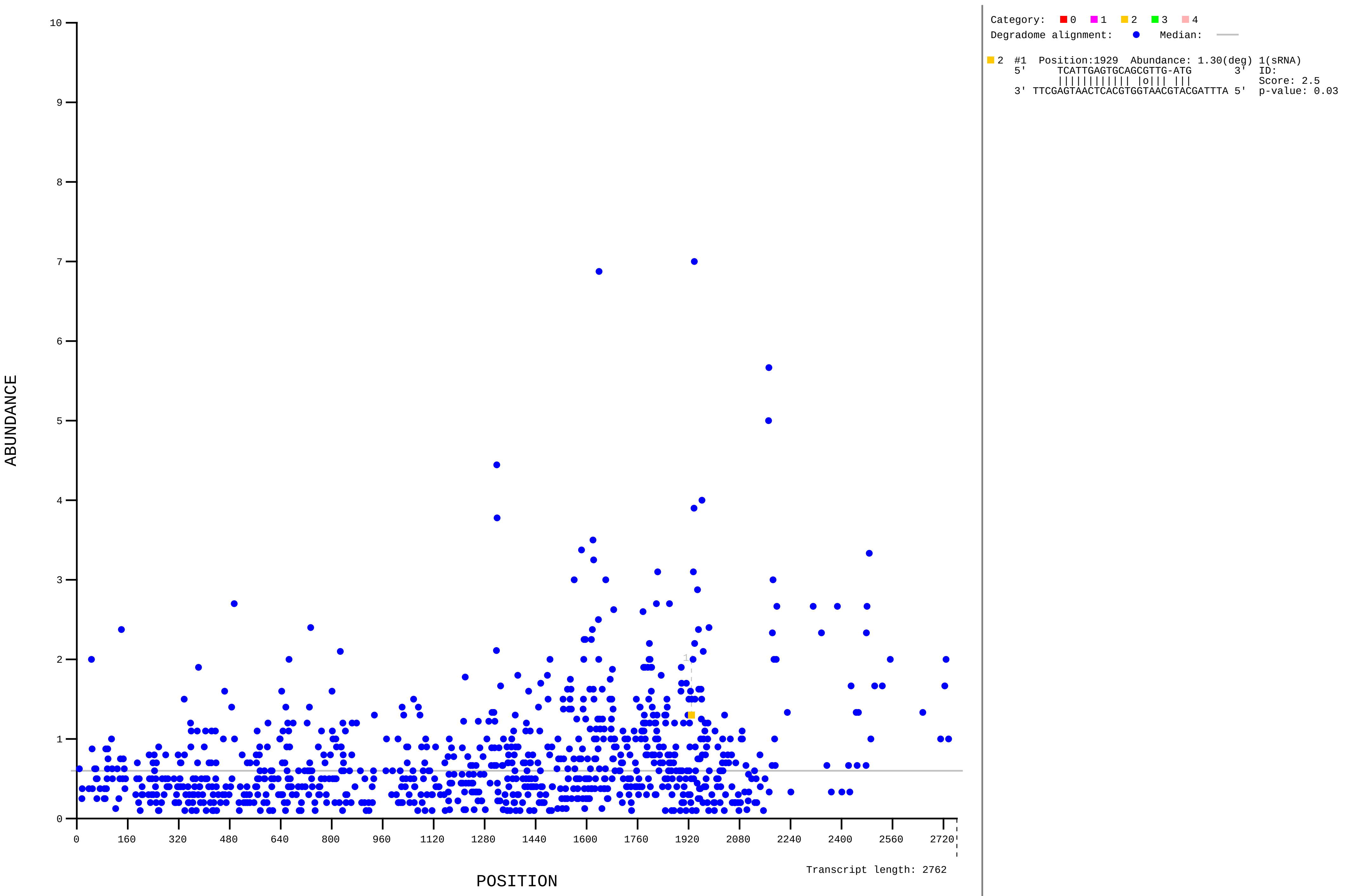

orange1.1t03059.4 gene=orange1.1t03059 CDS=276-2423

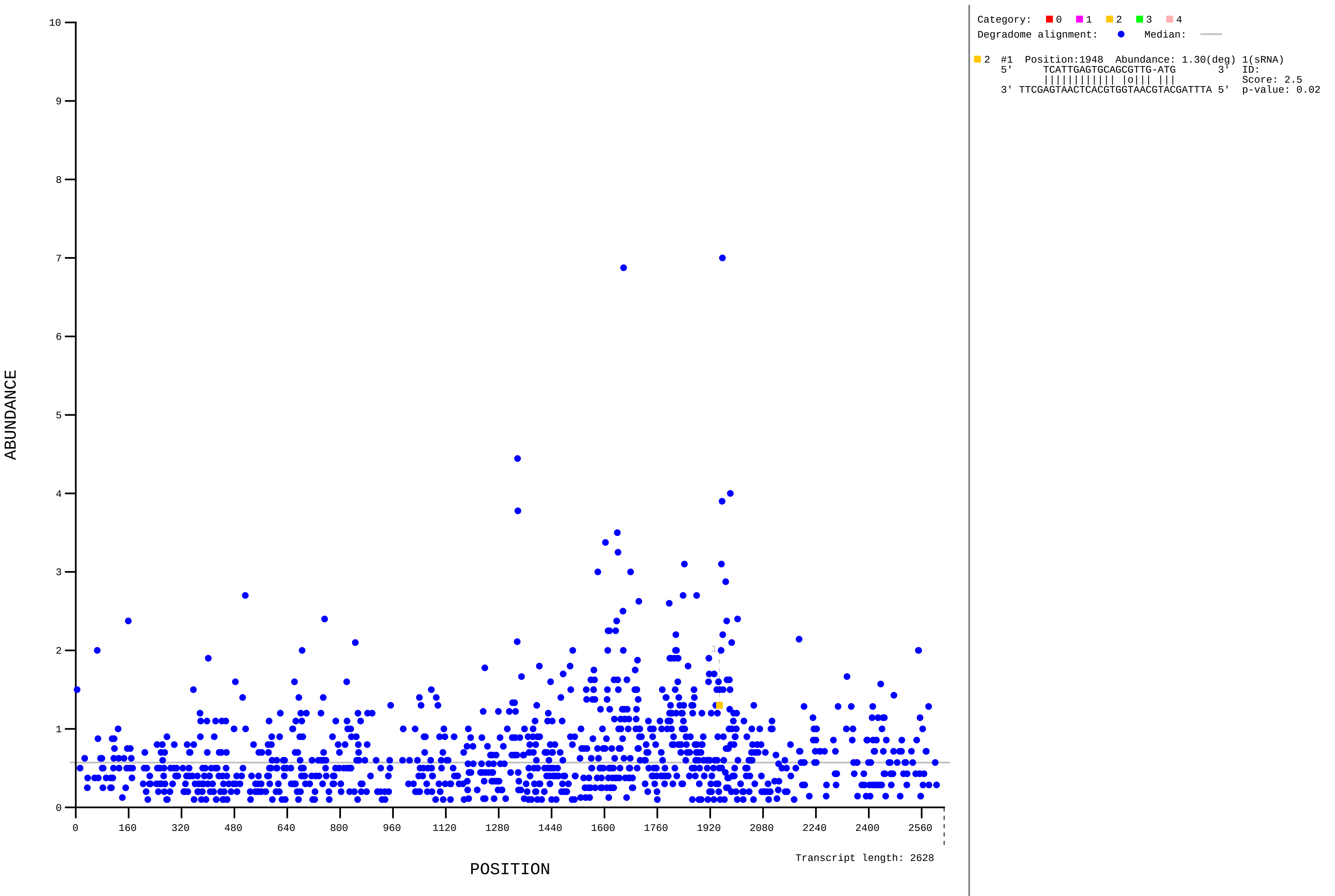

orange1.1t03059.2 gene=orange1.1t03059 CDS=257-2419

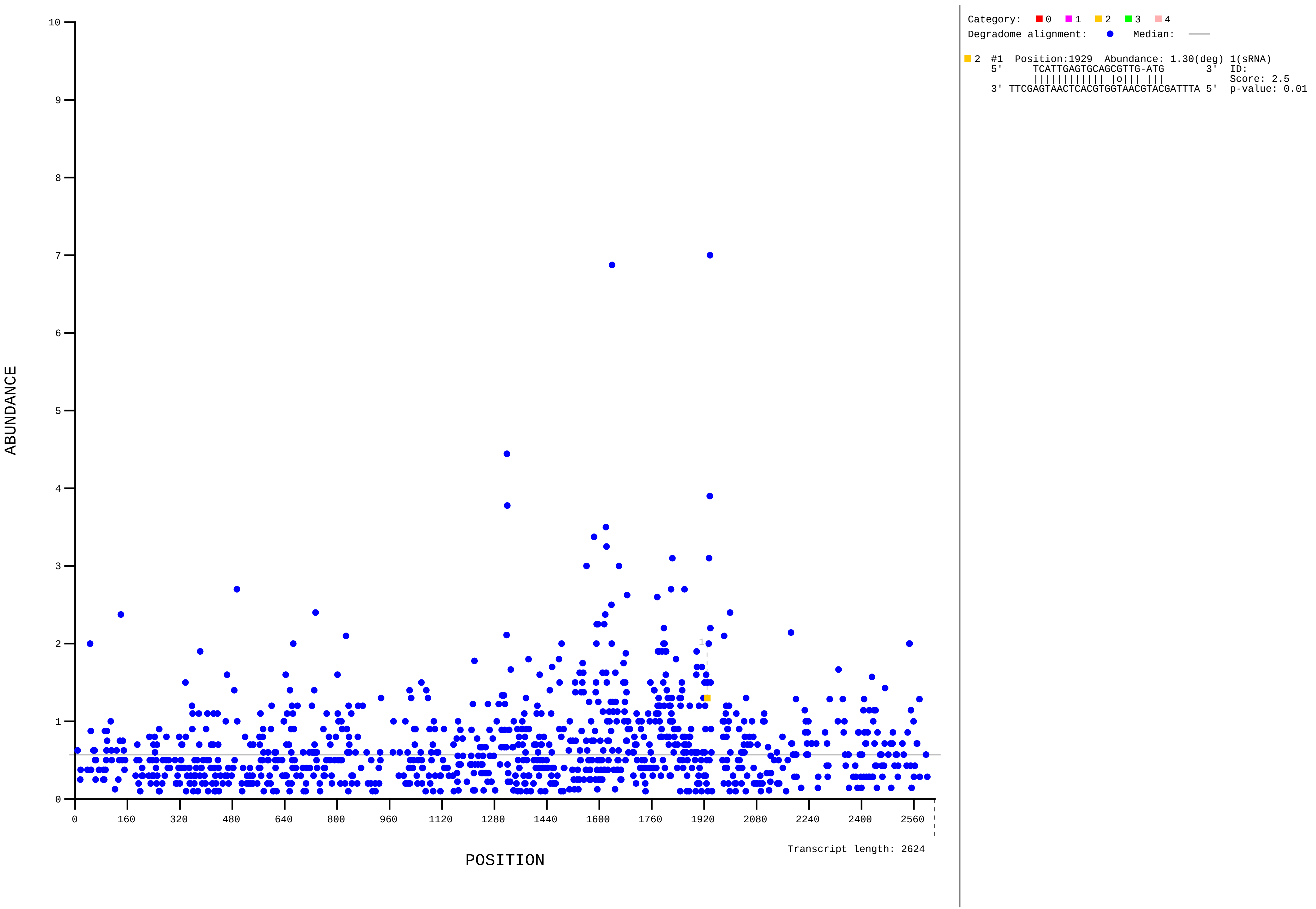

orange1.1t03059.1 gene=orange1.1t03059 CDS=257-2413

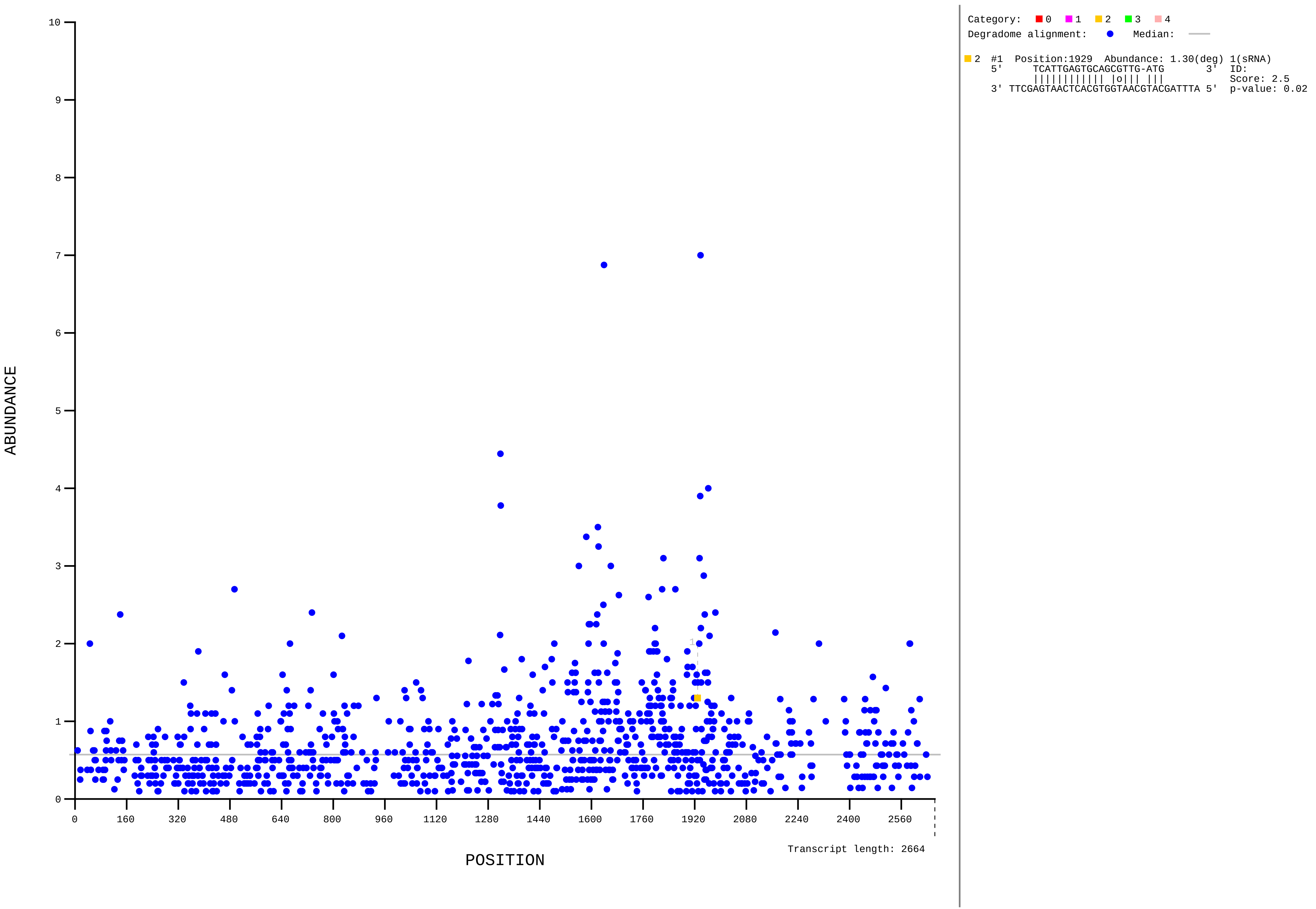

Cs3g11080.1 gene=Cs3g11080 CDS=1-3543

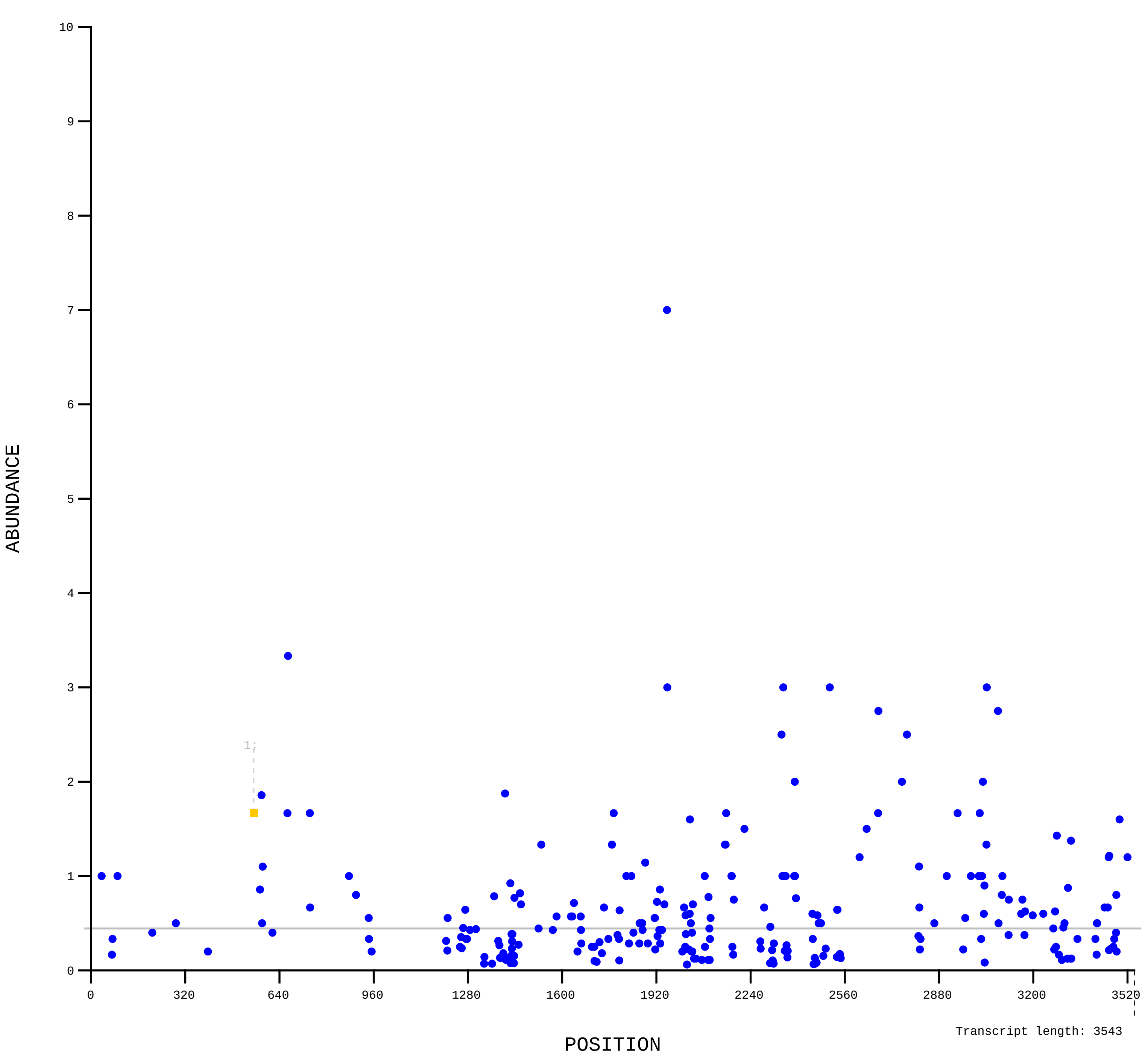

Category: 0 1 2 3 4
Degradome alignment: Median:

```
2  #1  Position:553  Abundance: 1.67(deg) 1(sRNA)
   5'      TCTTCCCTATGCCTCCCATTCC       3'  ID:
           |||| || ||||| ||||||||           Score: 3.0
   3' TAGCAGAAAGGCTACGGTGGGTAAGGTATTTA 5'  p-value: 0.04
```

Cs5g06850.1 gene=Cs5g06850 CDS=133-1677

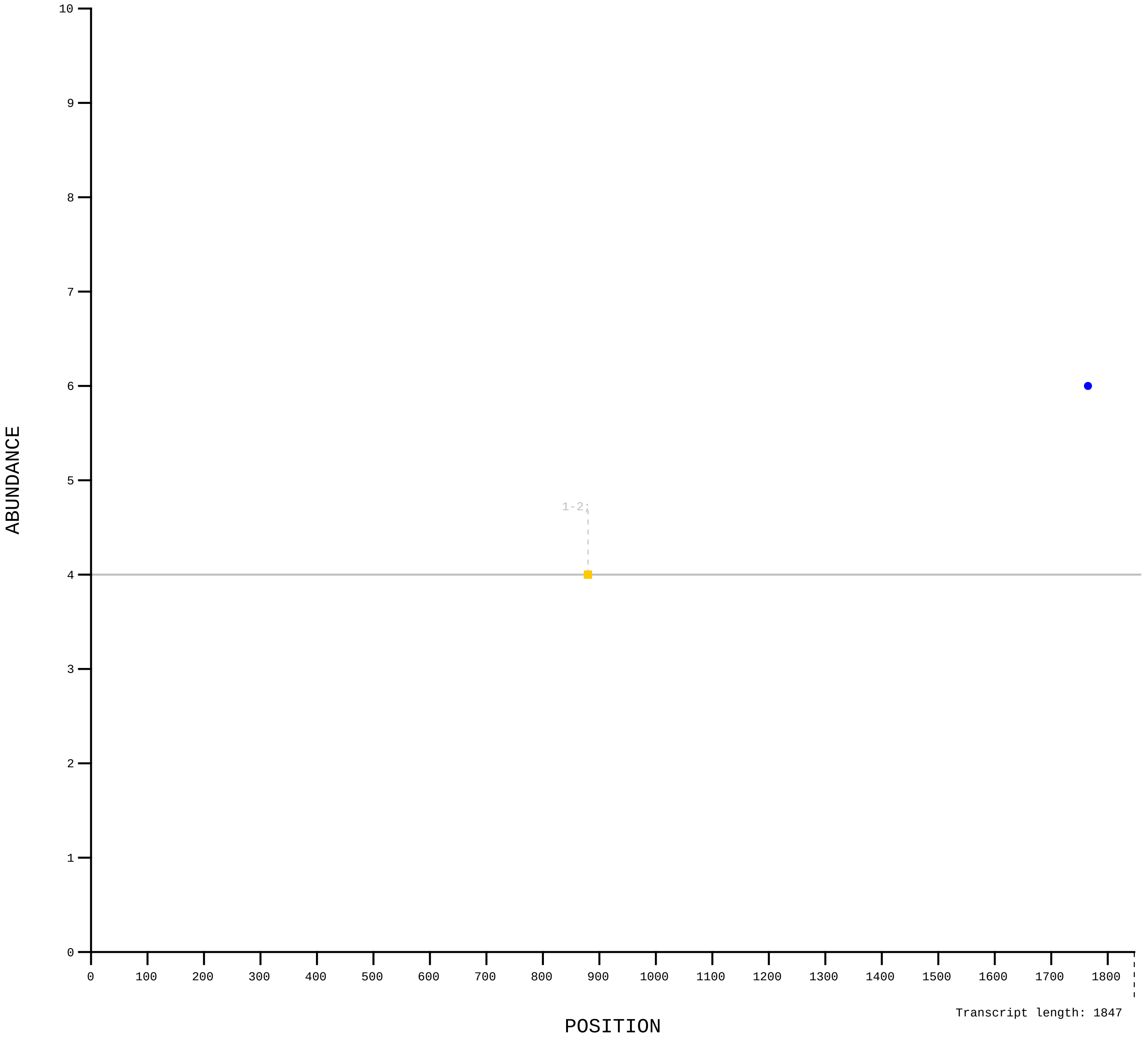


```
Category: 0 1 2 3 4
Degradome alignment:      Median:

2  #1  Position:880  Abundance: 4.00(deg) 1(sRNA)
   5'     ACTCTCCCTCAAGGGCTTCTC        3'  ID:
           |||||||||||o|||||| |            Score: 2.5
   3' TTTGAGAGAGGGAGTTTCCGAAG-GTTATACC 5'  p-value: 0.02

2  #2  Position:880  Abundance: 4.00(deg) 1(sRNA)
   5'     ACTCTCCCTCAAGGGCTTCGC        3'  ID:
           |||||||||||o|||||| |            Score: 2.5
   3' TTTGAGAGAGGGAGTTTCCGAAG-GTTATACC 5'  p-value: 0.0
```

Cs5g22030.3 gene=Cs5g22030 CDS=36-4382

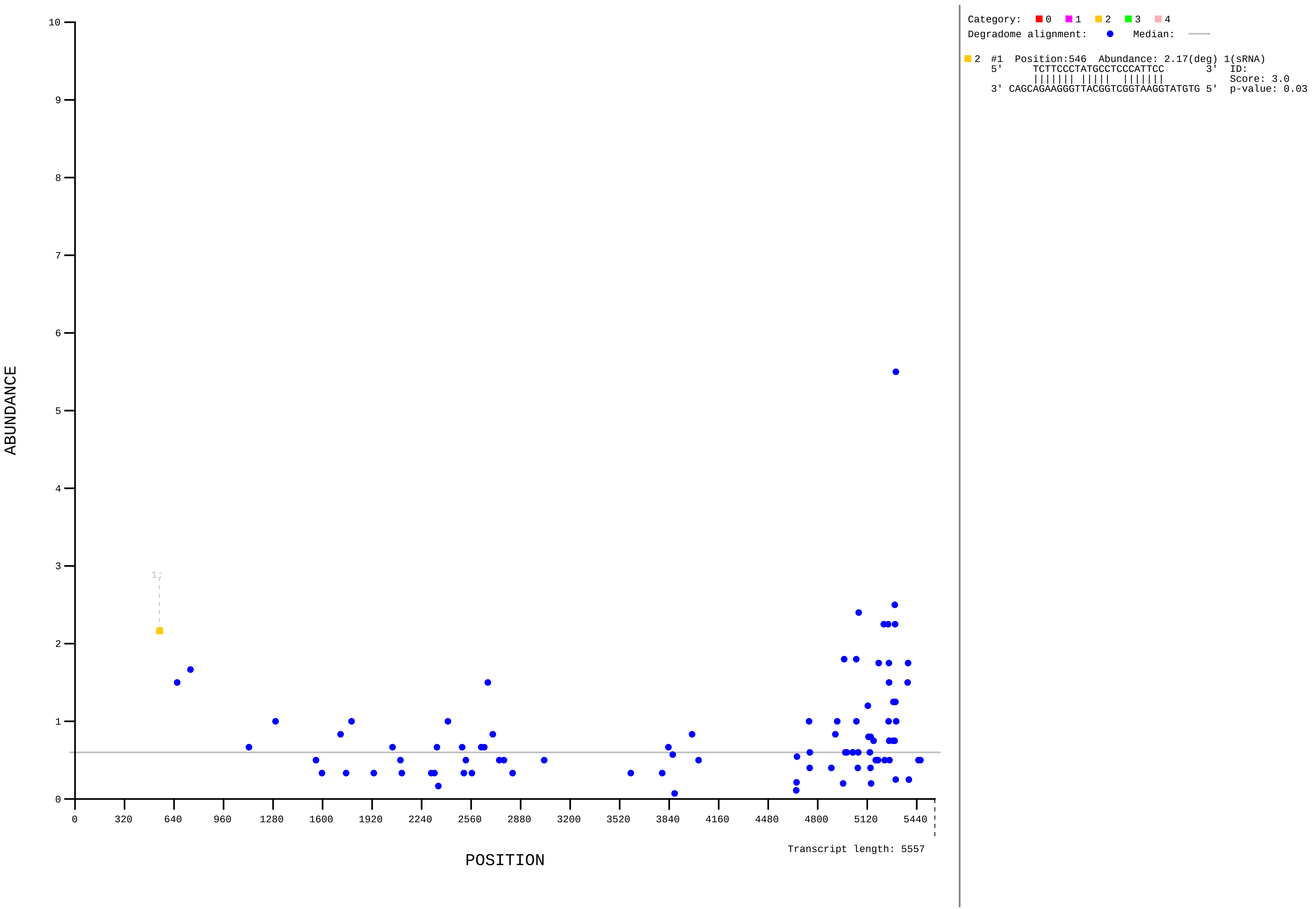

Cs5g22030.1 gene=Cs5g22030 CDS=36-4808

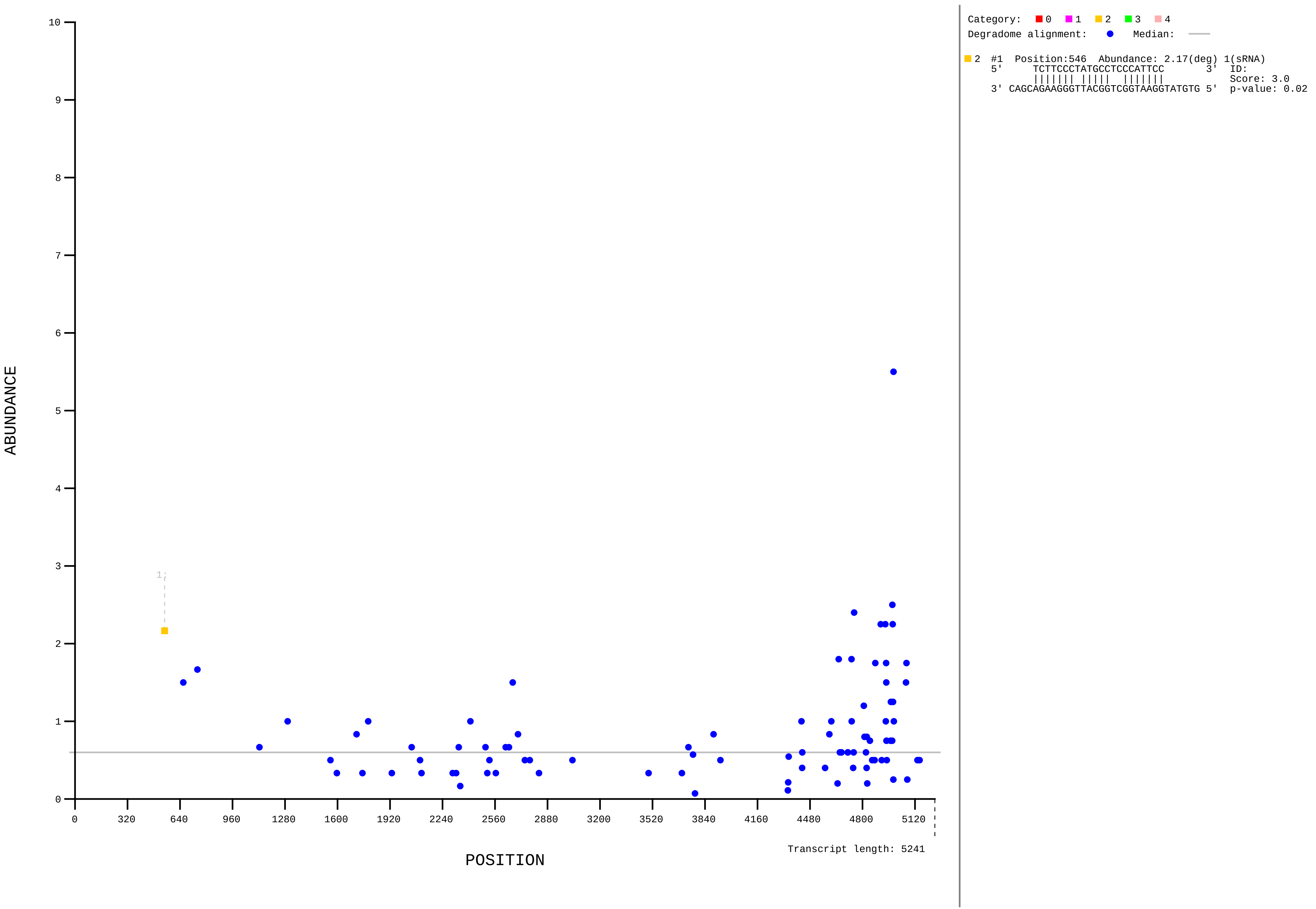

Cs5g22030.4 gene=Cs5g22030 CDS=36-4904

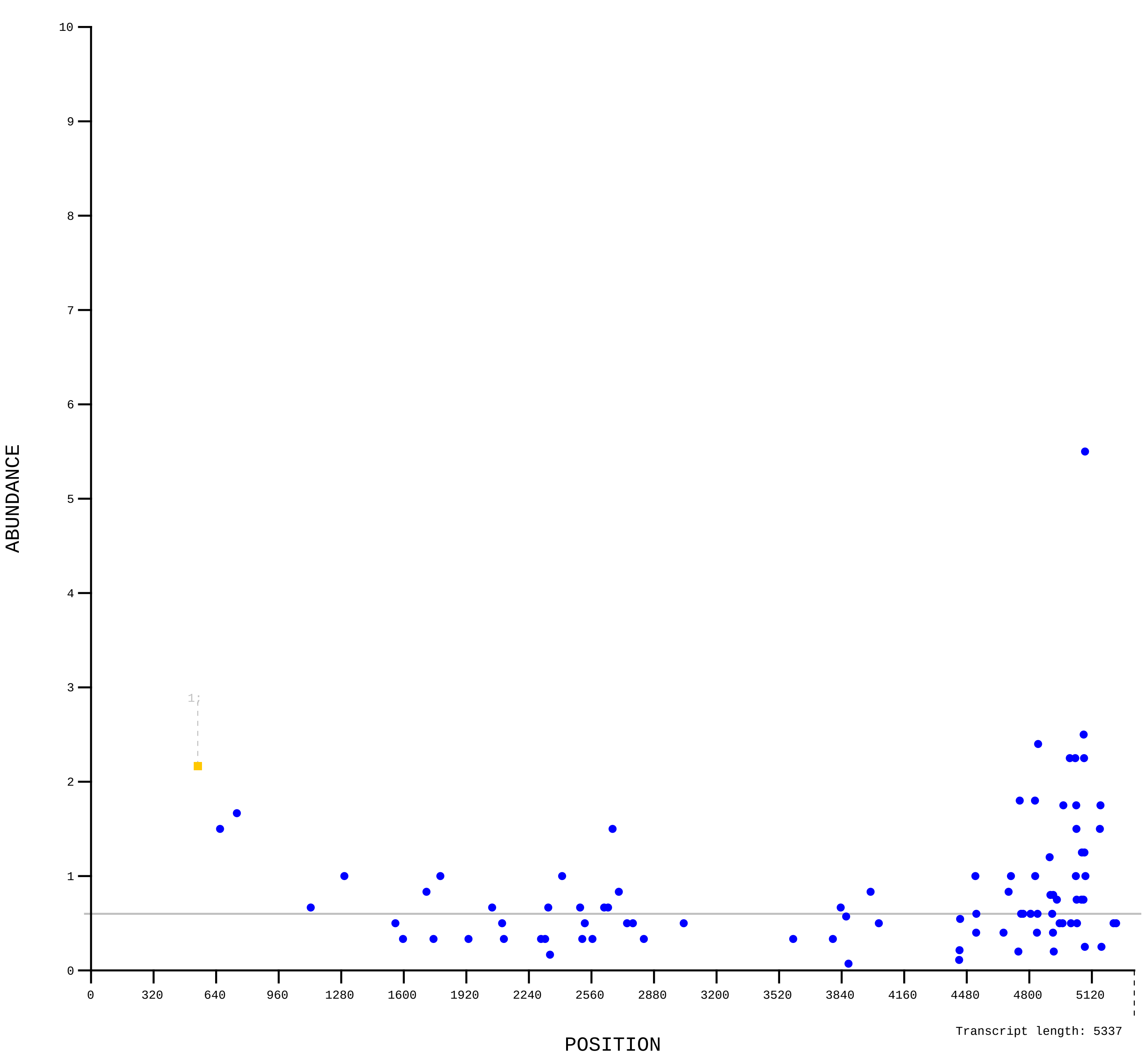

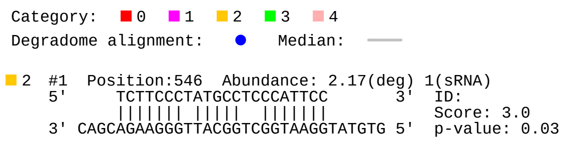

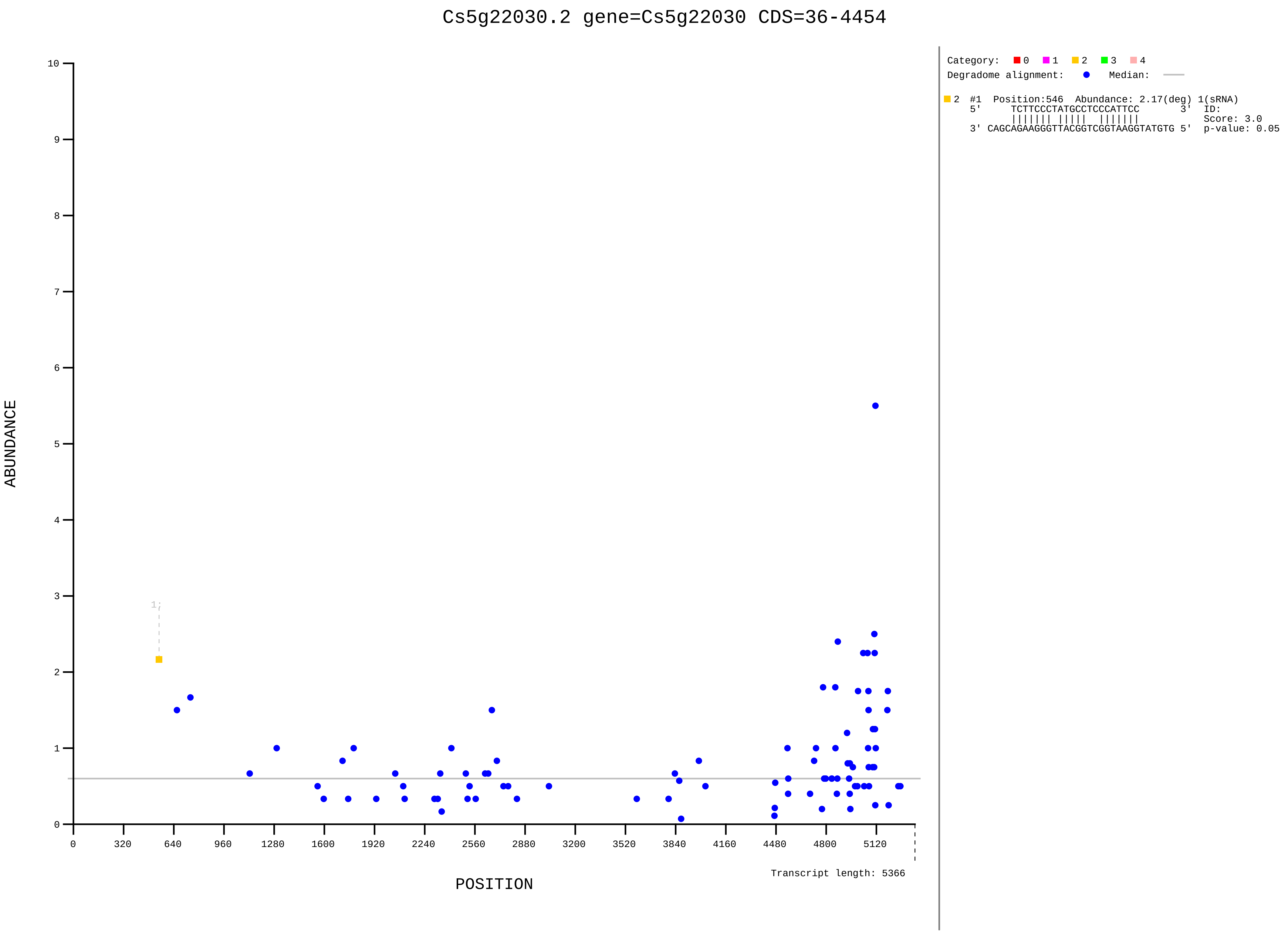

Cs5g22030.2 gene=Cs5g22030 CDS=36-4454
ABUNDANCE
POSITION
Transcript length: 5366
Category: 0 1 2 3 4
Degradome alignment: Median:
2 #1 Position:546 Abundance: 2.17(deg) 1(sRNA)
5' TCTTCCCTATGCCTCCCATTCC 3' ID:
||||||| ||||| |||||||
3' CAGCAGAAGGGTTACGGTCGGTAAGGTATGTG 5'
Score: 3.0
p-value: 0.05

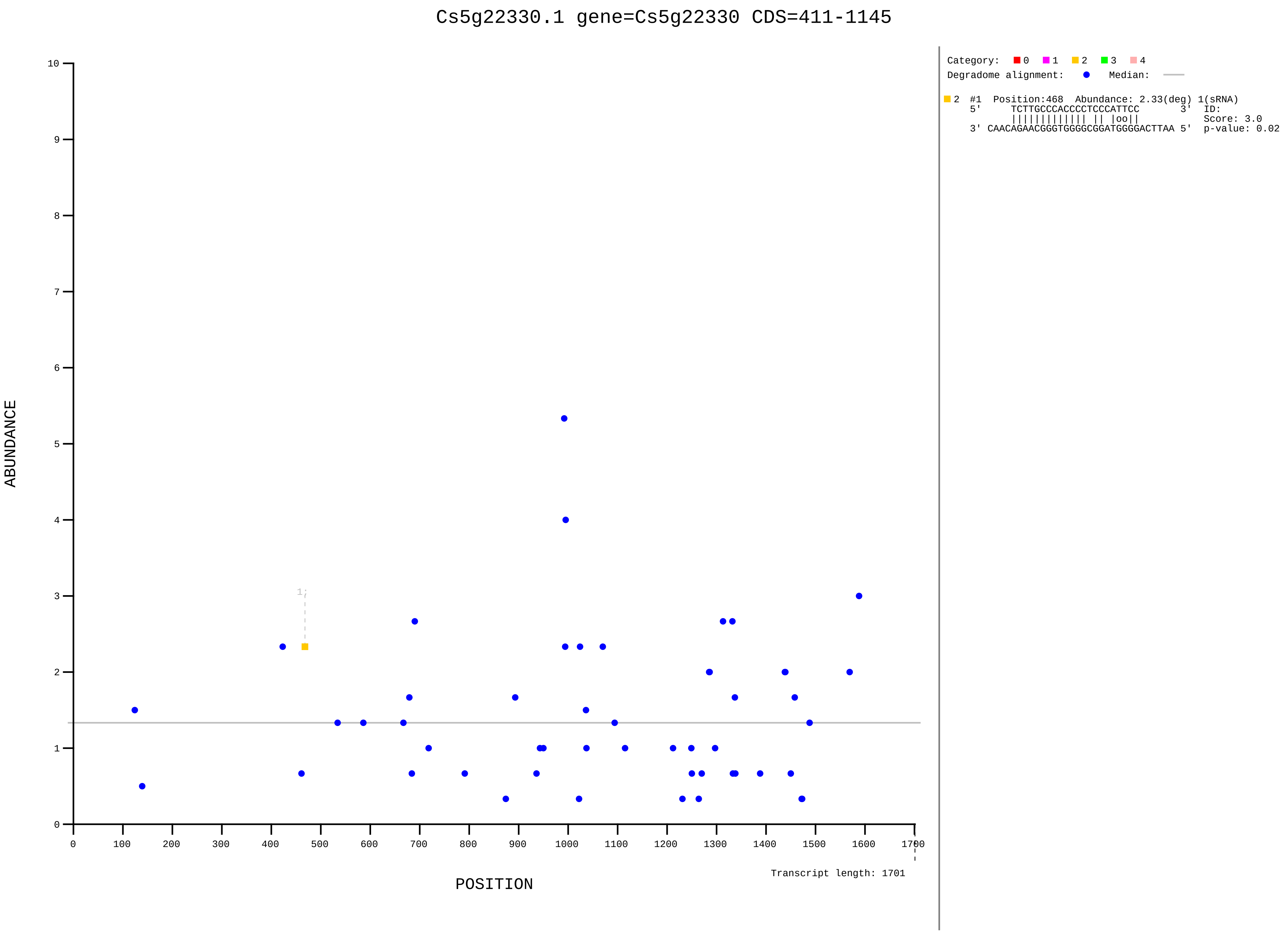
Cs5g22330.1 gene=Cs5g22330 CDS=411-1145
ABUNDANCE
POSITION
Transcript length: 1701
Category: 0 1 2 3 4
Degradome alignment:
Median:
2 #1 Position:468 Abundance: 2.33(deg) 1(sRNA)
5' TCTTGCCCACCCCTCCCATTCC 3' ID:
||||||||||||| || |oo||
Score: 3.0
3' CAACAGAACGGGTGGGGCGGATGGGGACTTAA 5' p-value: 0.02

Cs5g22330.3 gene=Cs5g22330 CDS=864-1496

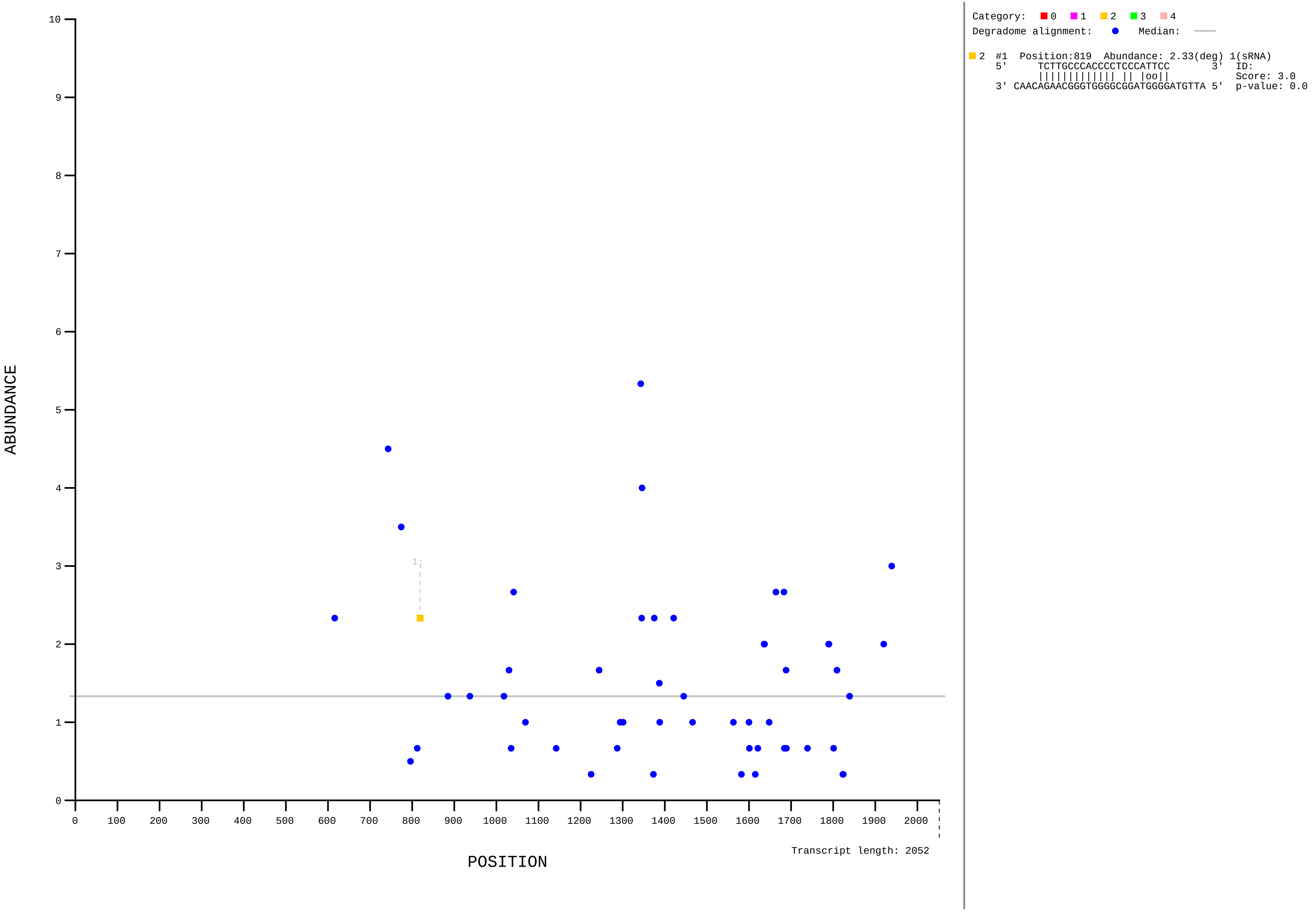

Cs5g22330.2 gene=Cs5g22330 CDS=682-1314

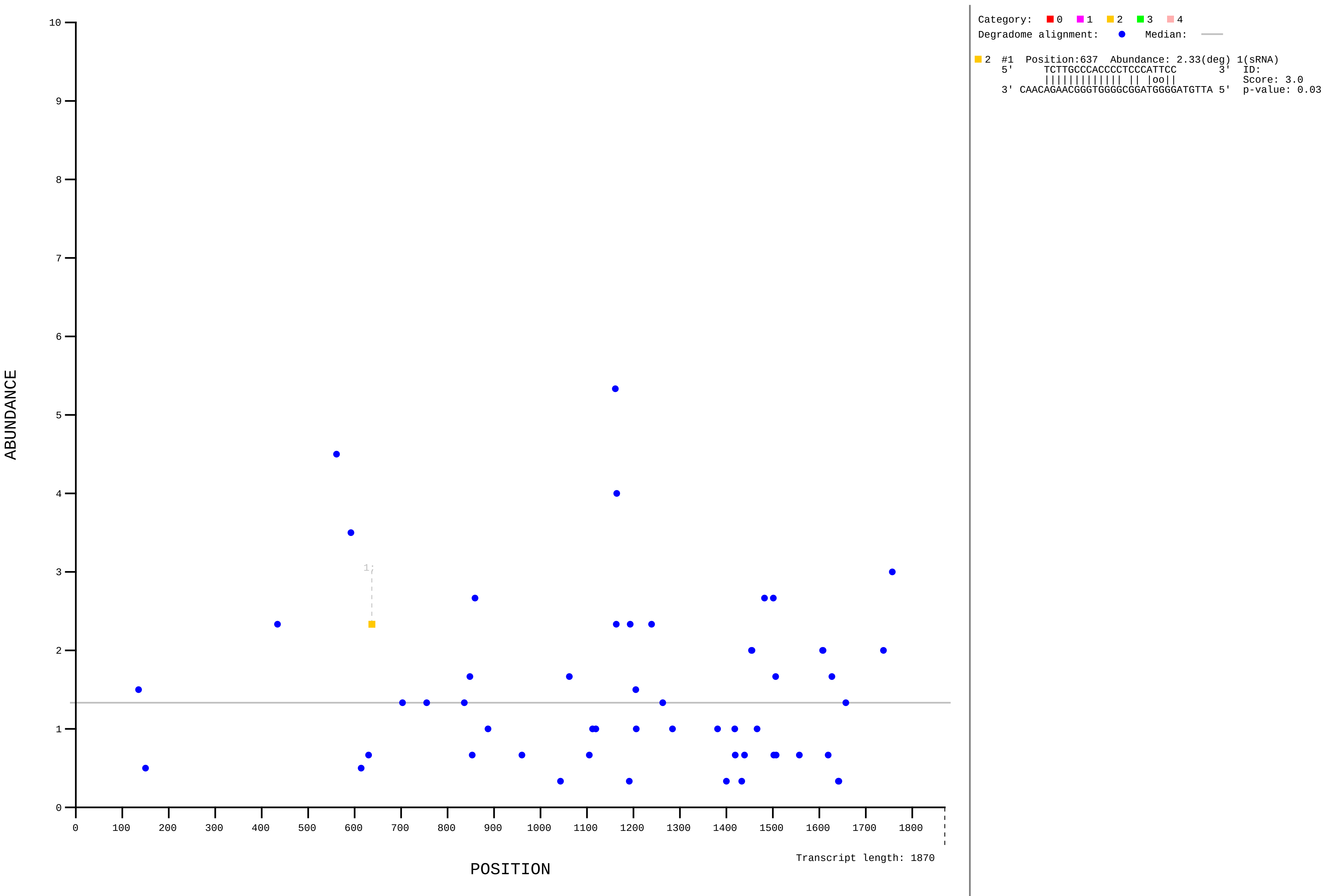

Cs2g20660.5 gene=Cs2g20660 CDS=96-2135

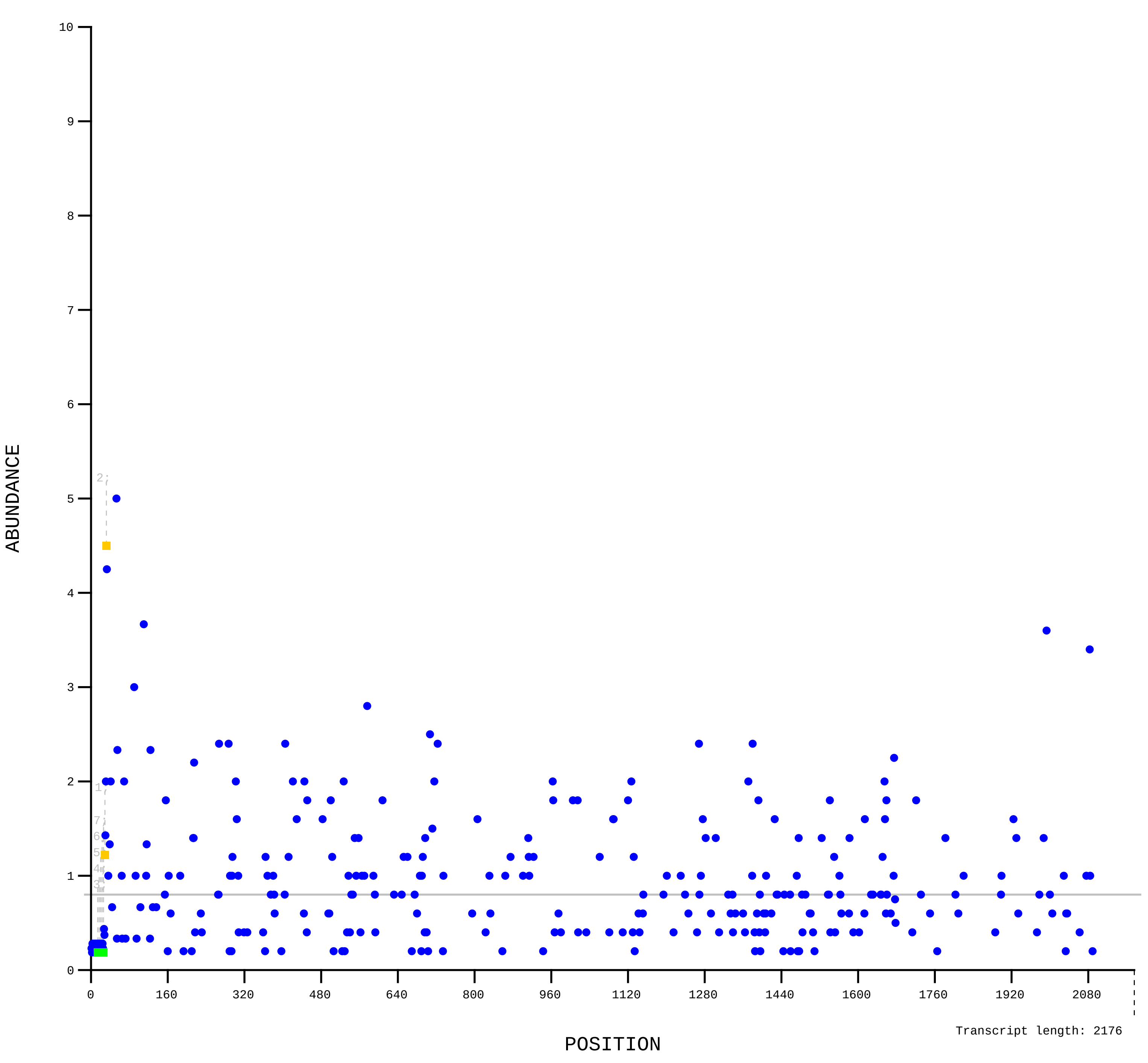

Category: 0 1 2 3 4
Degradome alignment: Median:

```
2  #1  Position:29  Abundance: 1.22(deg) 1(sRNA)
    5'     AAGACGAAGAAGAAGAAGAAGAA      3'  ID:
           |||| ||||||||||||||||||          Score: 1.0
    3' CTTCTTCTTCTTCTTCTTCTTCTTCTTCTTCT 5'  p-value: 0.0

2  #2  Position:32  Abundance: 4.50(deg) 1(sRNA)
    5'     AAGACGAAGAAGAAGAAGAAGAA      3'  ID:
           |||| ||||||||||||||||||          Score: 1.0
    3' CTTCTTCTTCTTCTTCTTCTTCTTCTTCTTCT 5'  p-value: 0.0

3  #3  Position:14  Abundance: 0.19(deg) 1(sRNA)
    5'     AAGACGAAGAAGAAGAAGAAGAA      3'  ID:
           |||| ||||||||||||||||||          Score: 1.0
    3' CTTCTTCTTCTTCTTCTTCTTCTTCTT      5'  p-value: 0.02

3  #4  Position:17  Abundance: 0.19(deg) 1(sRNA)
    5'     AAGACGAAGAAGAAGAAGAAGAA      3'  ID:
           |||| ||||||||||||||||||          Score: 1.0
    3' CTTCTTCTTCTTCTTCTTCTTCTTCTTCTT   5'  p-value: 0.0

3  #5  Position:20  Abundance: 0.19(deg) 1(sRNA)
    5'     AAGACGAAGAAGAAGAAGAAGAA      3'  ID:
           |||| ||||||||||||||||||          Score: 1.0
    3' CTTCTTCTTCTTCTTCTTCTTCTTCTTCTTCT 5'  p-value: 0.0

3  #6  Position:23  Abundance: 0.19(deg) 1(sRNA)
    5'     AAGACGAAGAAGAAGAAGAAGAA      3'  ID:
           |||| ||||||||||||||||||          Score: 1.0
    3' CTTCTTCTTCTTCTTCTTCTTCTTCTTCTTCT 5'  p-value: 0.0

3  #7  Position:26  Abundance: 0.19(deg) 1(sRNA)
    5'     AAGACGAAGAAGAAGAAGAAGAA      3'  ID:
           |||| ||||||||||||||||||          Score: 1.0
    3' CTTCTTCTTCTTCTTCTTCTTCTTCTTCTTCT 5'  p-value: 0.01
```

Cs9g06850.2 gene=Cs9g06850 CDS=291-1229

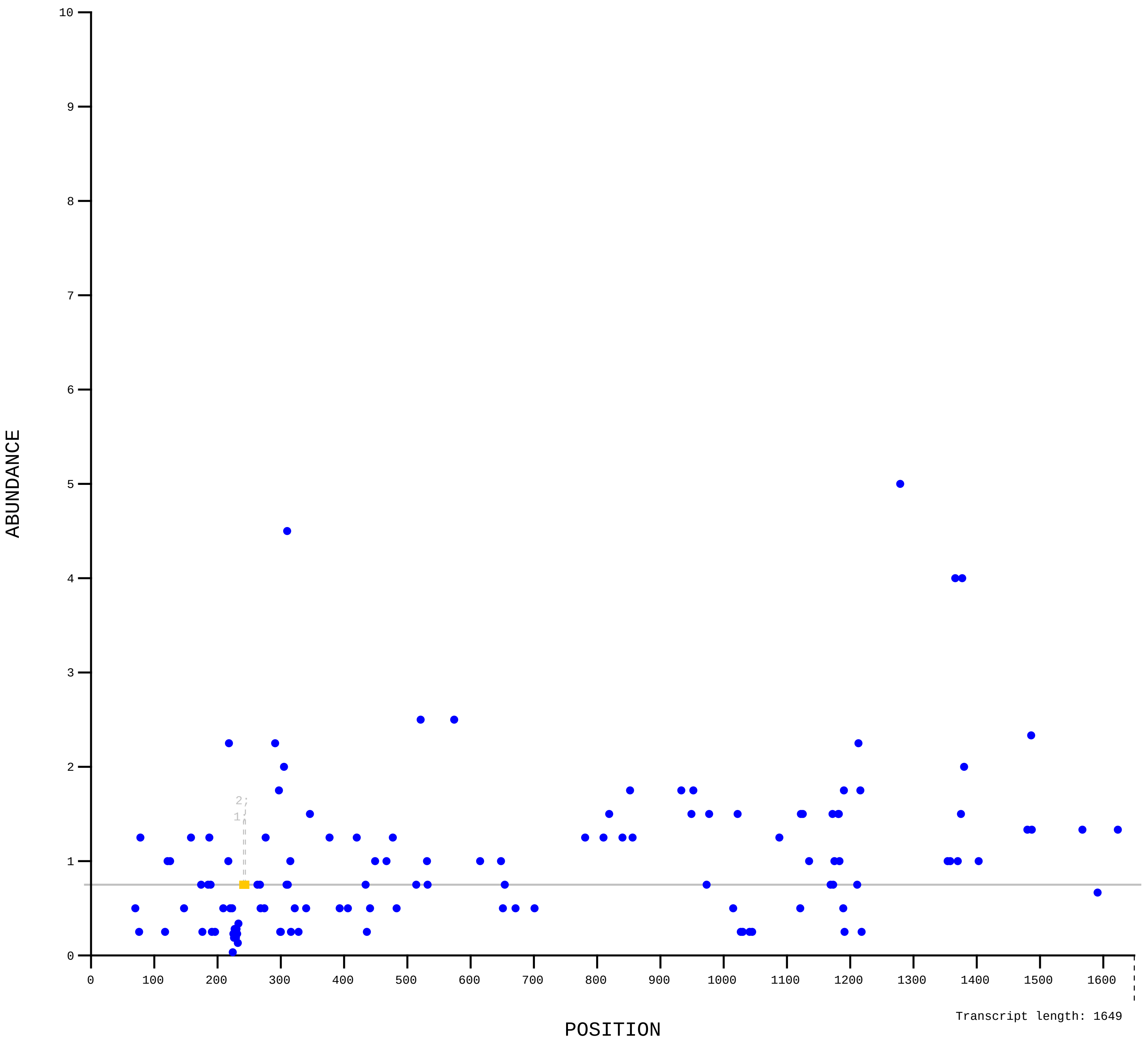

Category: 0 1 2 3 4
Degradome alignment: Median:

```
2  #1  Position:241  Abundance: 0.75(deg) 1(sRNA)
   5'     AAGACGAAGAAGAAGAAGAAGAA      3'  ID:
          |||| ||||||||||||||||||          Score: 1.0
   3' CTGCTTCTTCTTCTTCTTCTTCTTCTTAA 5'  p-value: 0.0

2  #2  Position:244  Abundance: 0.75(deg) 1(sRNA)
   5'     AAGACGAAGAAGAAGAAGAAGAA      3'  ID:
          | || ||||||||||||||||||          Score: 2.0
   3' CTGCTGCTTCTTCTTCTTCTTCTTCTTCT 5'  p-value: 0.05
```

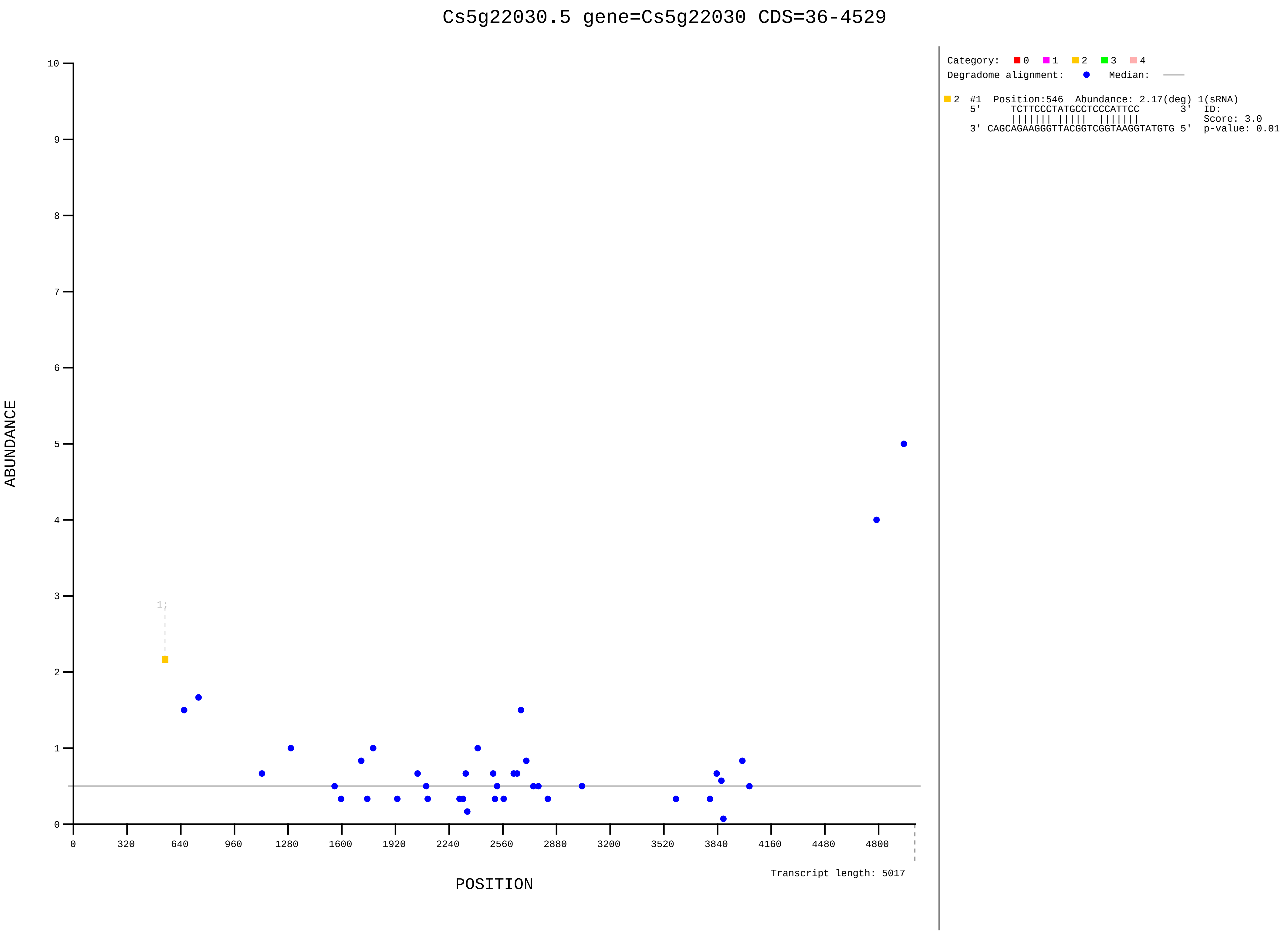

Cs5g22030.5 gene=Cs5g22030 CDS=36-4529
ABUNDANCE
POSITION
Transcript length: 5017
Category: 0 1 2 3 4
Degradome alignment:
Median:
2 #1 Position:546 Abundance: 2.17(deg) 1(sRNA)
5' TCTTCCCTATGCCTCCCATTCC 3' ID:
||||||| ||||| |||||||
Score: 3.0
3' CAGCAGAAGGGTTACGGTCGGTAAGGTATGTG 5' p-value: 0.01

Cs1g09350.2 gene=Cs1g09350 CDS=74-2956

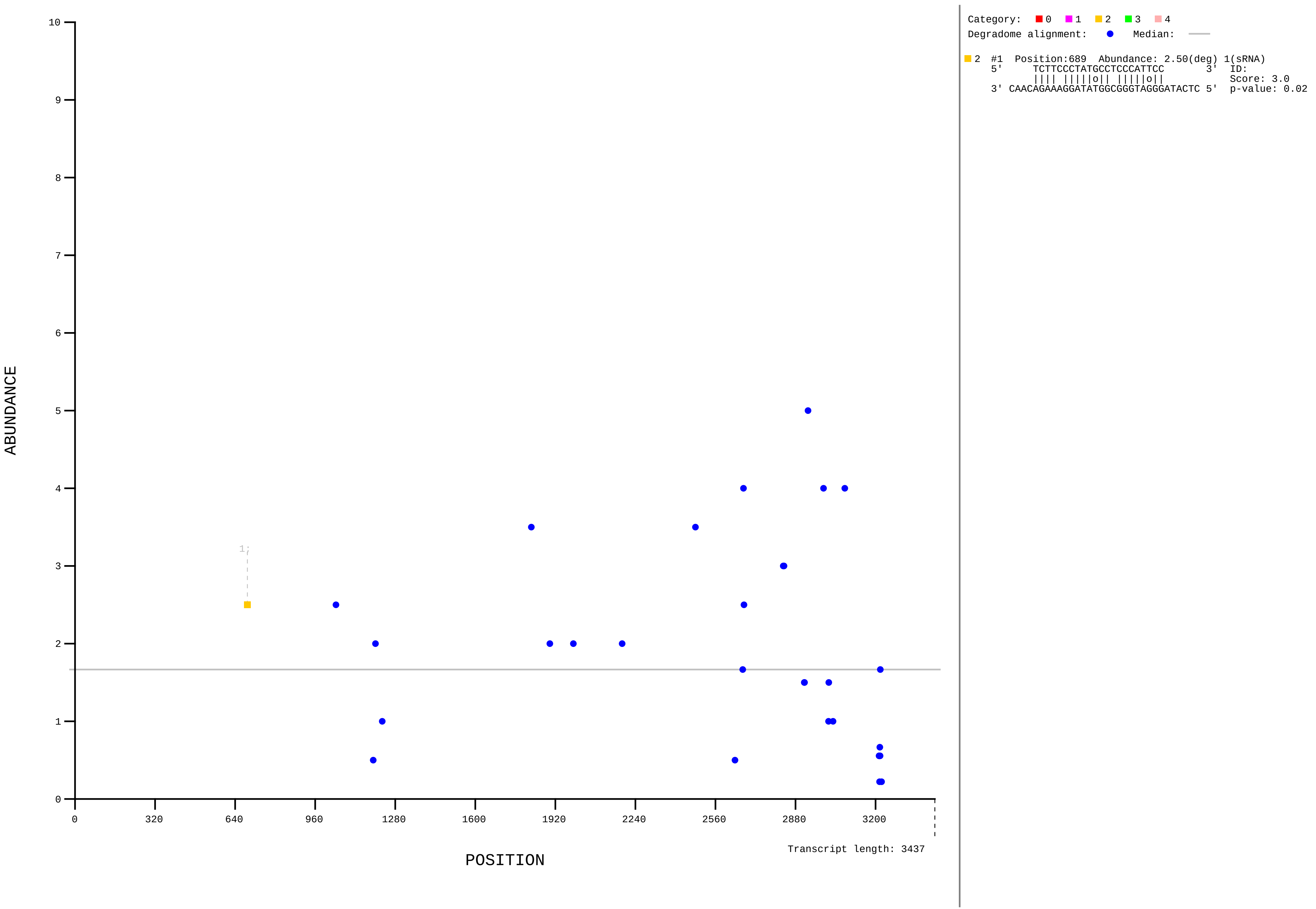

Cs3g18660.4 gene=Cs3g18660 CDS=76-2070

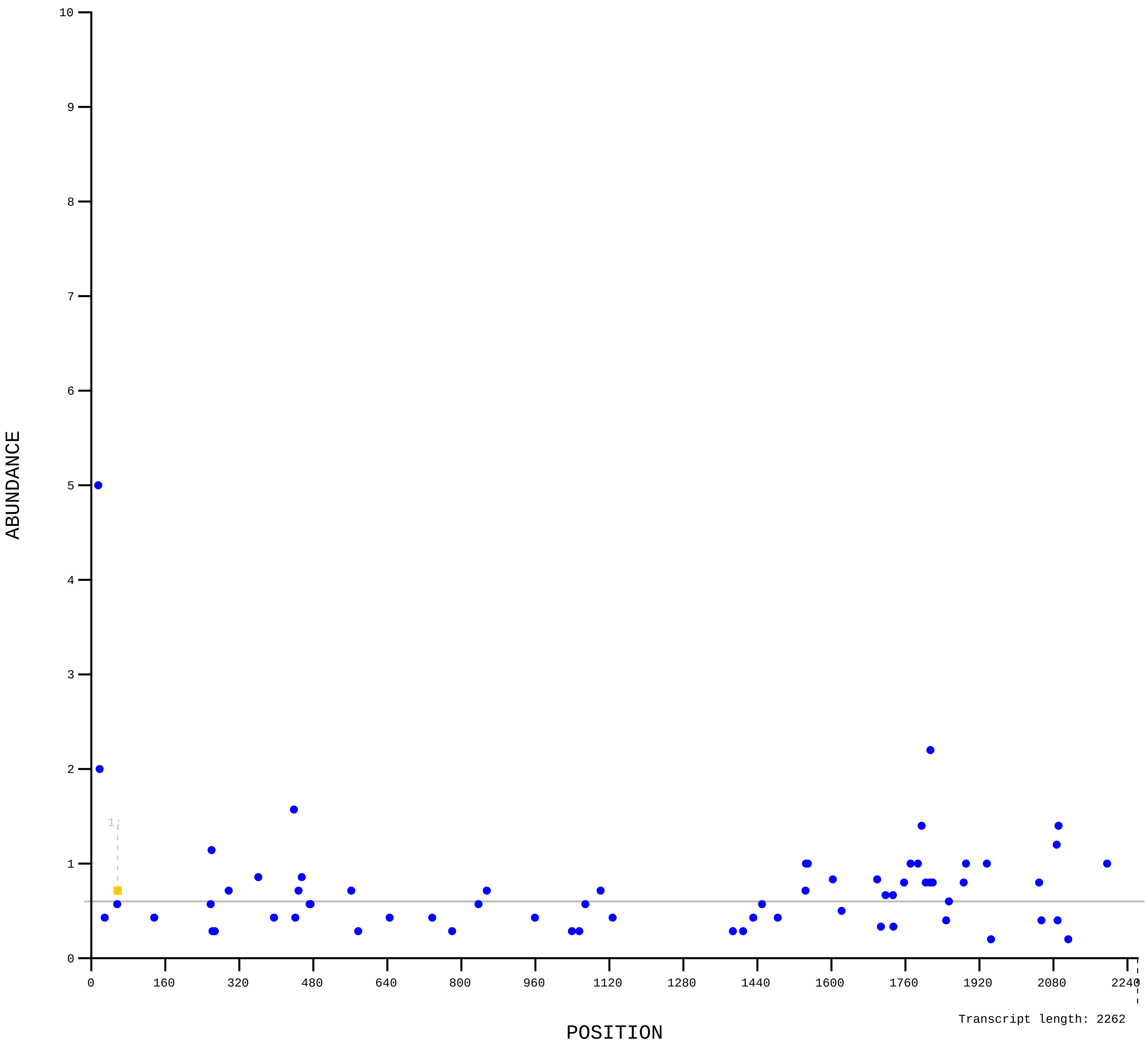

Category: 0 1 2 3 4
Degradome alignment: Median:

2 #1 Position:57 Abundance: 0.71(deg) 1(sRNA)

```
5'     CTGAAGTGTTTGGGGGAACTC        3'  ID:
         ||||||| |||||o||||||           Score: 2.5
3' AAACAACTTCACTAACCCTCTTGAGGGTGTCT 5'  p-value: 0.01
```

Cs1g08640.3 gene=Cs1g08640 CDS=276-1970

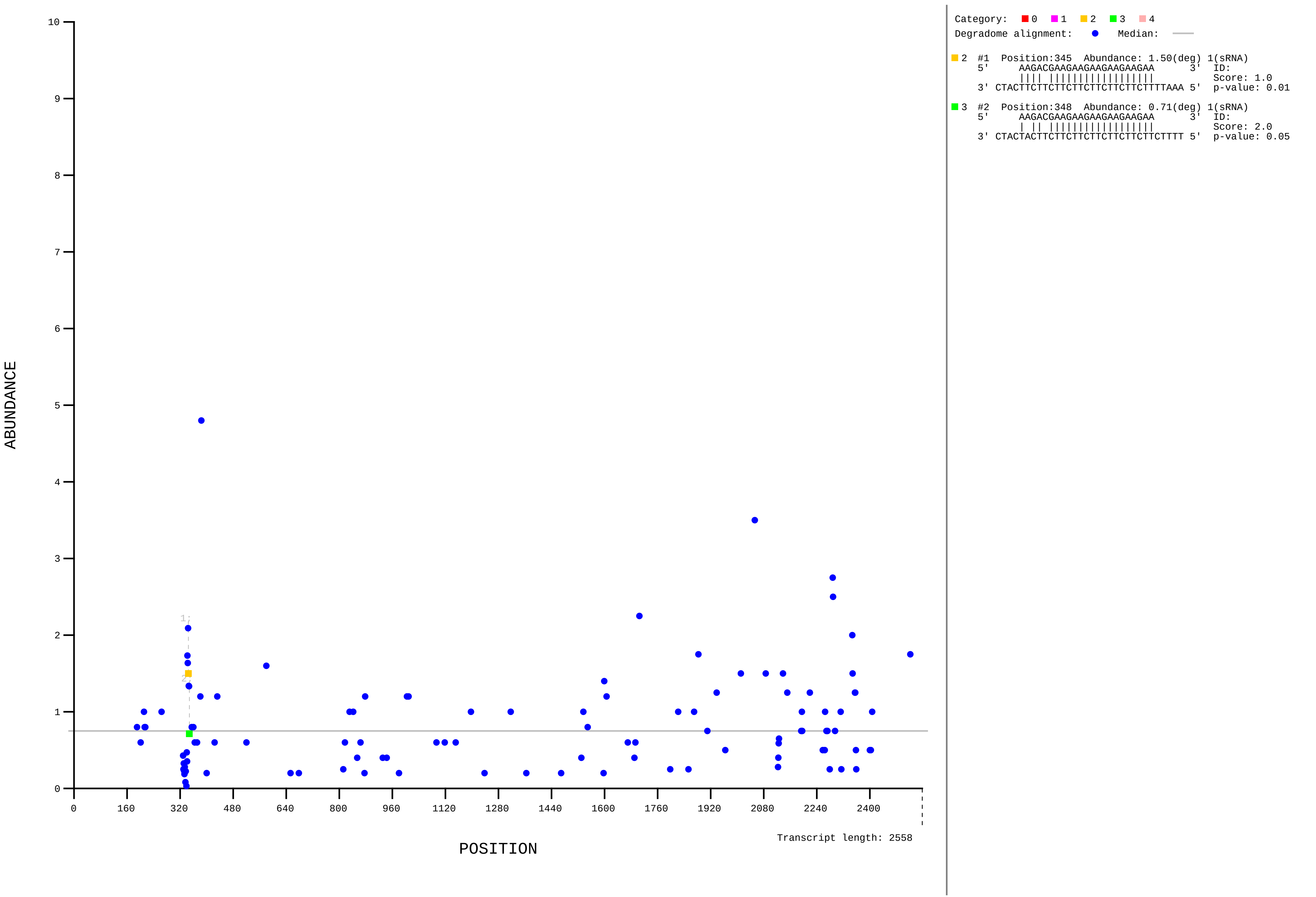

Cs1g08640.4 gene=Cs1g08640 CDS=115-1668

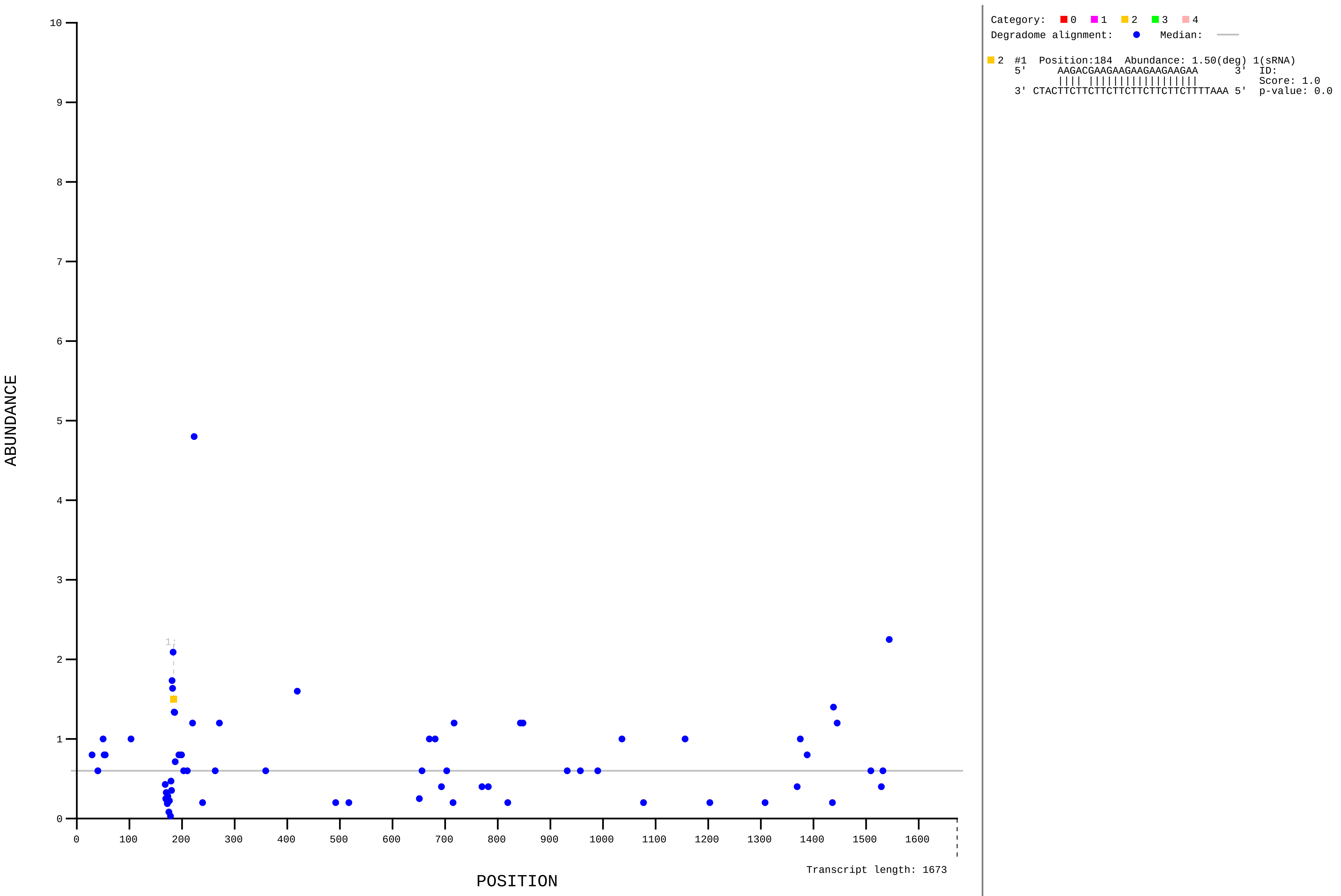

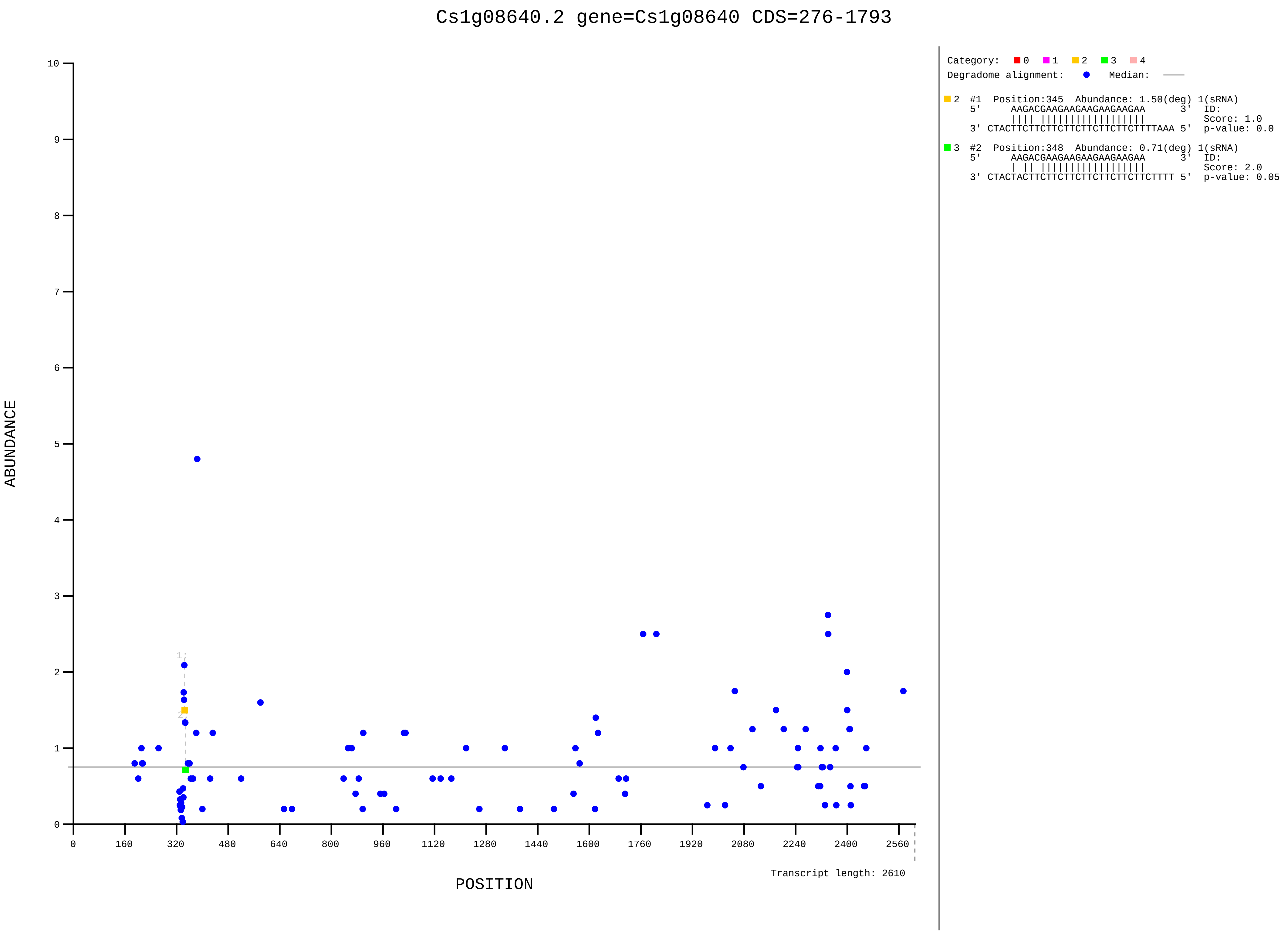

Cs1g08640.2 gene=Cs1g08640 CDS=276-1793
ABUNDANCE
POSITION
Transcript length: 2610
0 1 2 3 4 5 6 7 8 9 10
0 160 320 480 640 800 960 1120 1280 1440 1600 1760 1920 2080 2240 2400 2560
Category: 0 1 2 3 4
Degradome alignment: Median:
2 #1 Position:345 Abundance: 1.50(deg) 1(sRNA)
5' AAGACGAAGAAGAAGAAGAAGAA 3' ID:
|||| |||||||||||||||||| Score: 1.0
3' CTACTTCTTCTTCTTCTTCTTCTTCTTTTAAA 5' p-value: 0.0
3 #2 Position:348 Abundance: 0.71(deg) 1(sRNA)
5' AAGACGAAGAAGAAGAAGAAGAA 3' ID:
| || |||||||||||||||||| Score: 2.0
3' CTACTACTTCTTCTTCTTCTTCTTCTTTT 5' p-value: 0.05

Cs1g08640.1 gene=Cs1g08640 CDS=115-1809

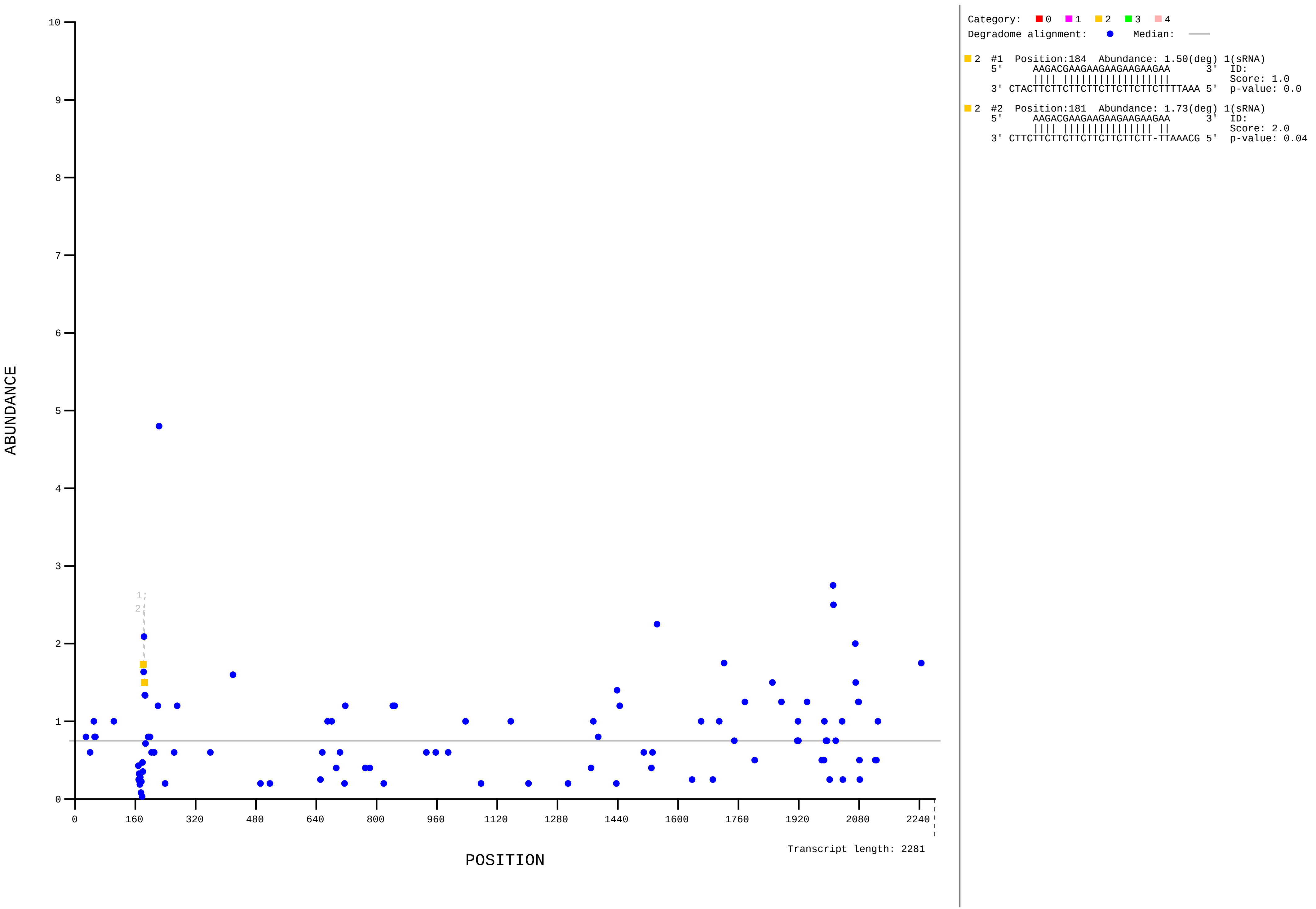

Cs1g08640.5 gene=Cs1g08640 CDS=618-1919

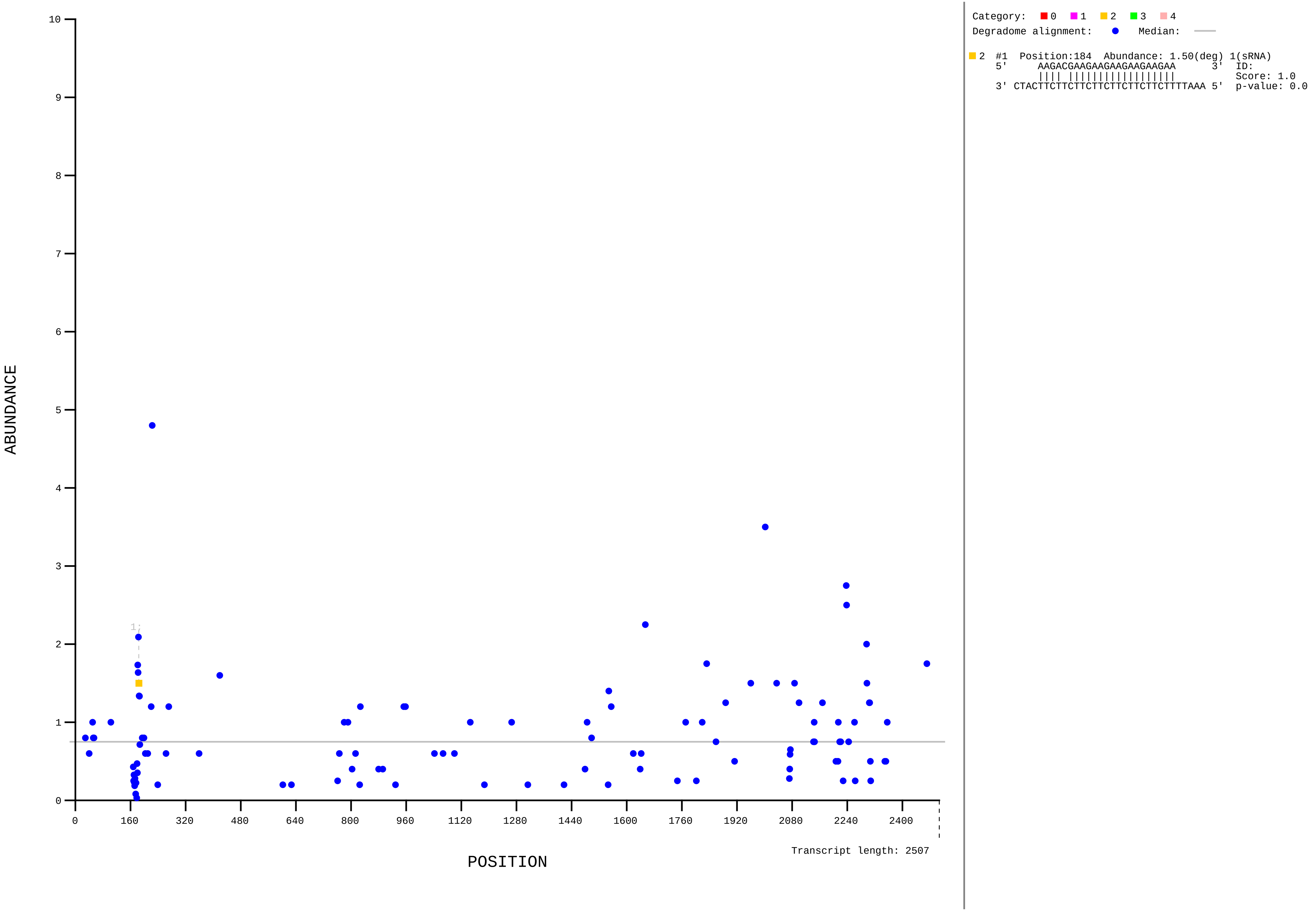

Cs9g06850.4 gene=Cs9g06850 CDS=291-1229

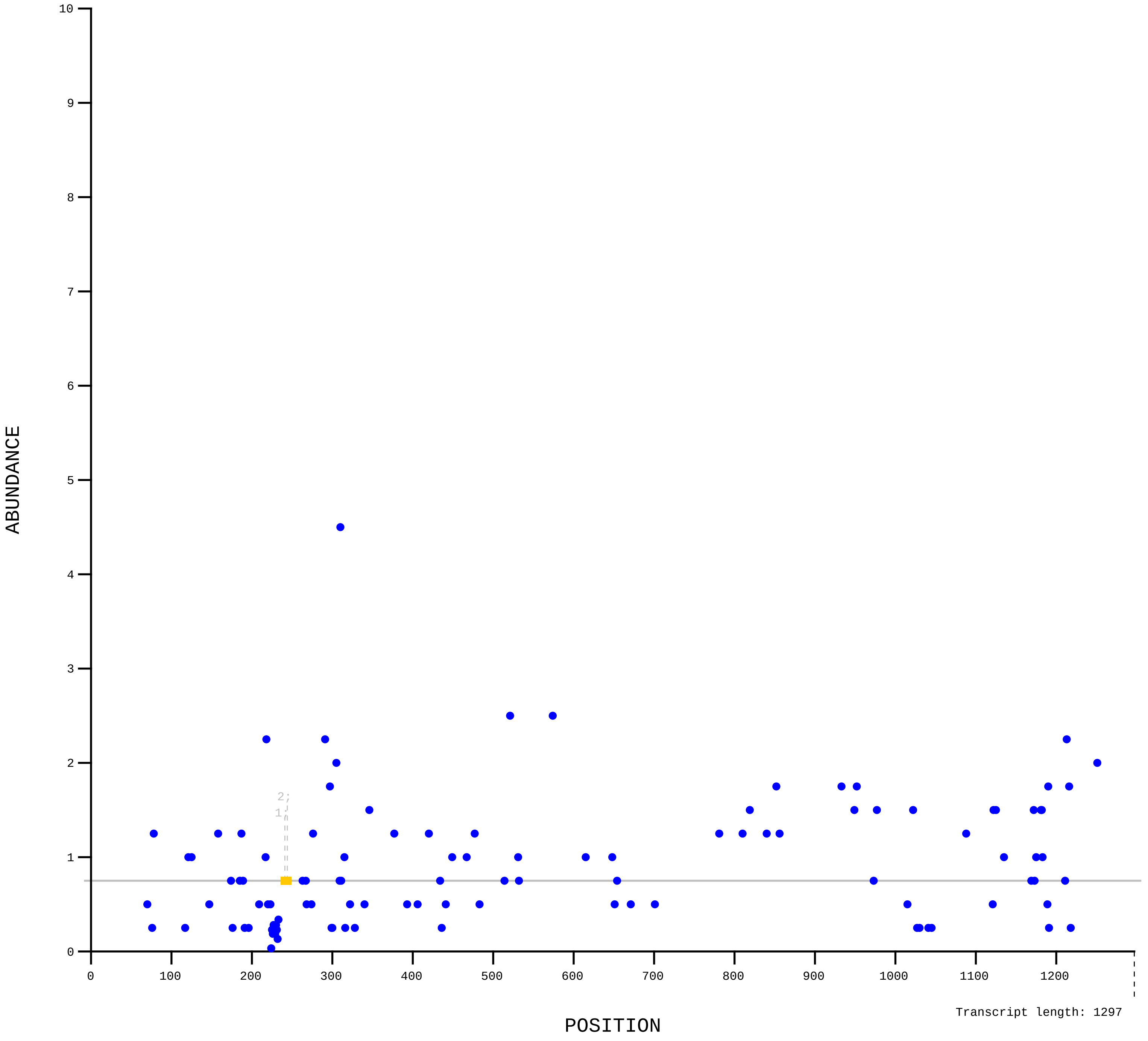


```
Category:  0  1  2  3  4
Degradome alignment:      Median:

2  #1  Position:241  Abundance: 0.75(deg) 1(sRNA)
   5'     AAGACGAAGAAGAAGAAGAAGAA      3'  ID:
          |||| ||||||||||||||||||          Score: 1.0
   3' CTGCTTCTTCTTCTTCTTCTTCTTCTTCTTAA 5'  p-value: 0.0

2  #2  Position:244  Abundance: 0.75(deg) 1(sRNA)
   5'     AAGACGAAGAAGAAGAAGAAGAA      3'  ID:
          | || ||||||||||||||||||          Score: 2.0
   3' CTGCTGCTTCTTCTTCTTCTTCTTCTTCTTCT 5'  p-value: 0.05
```

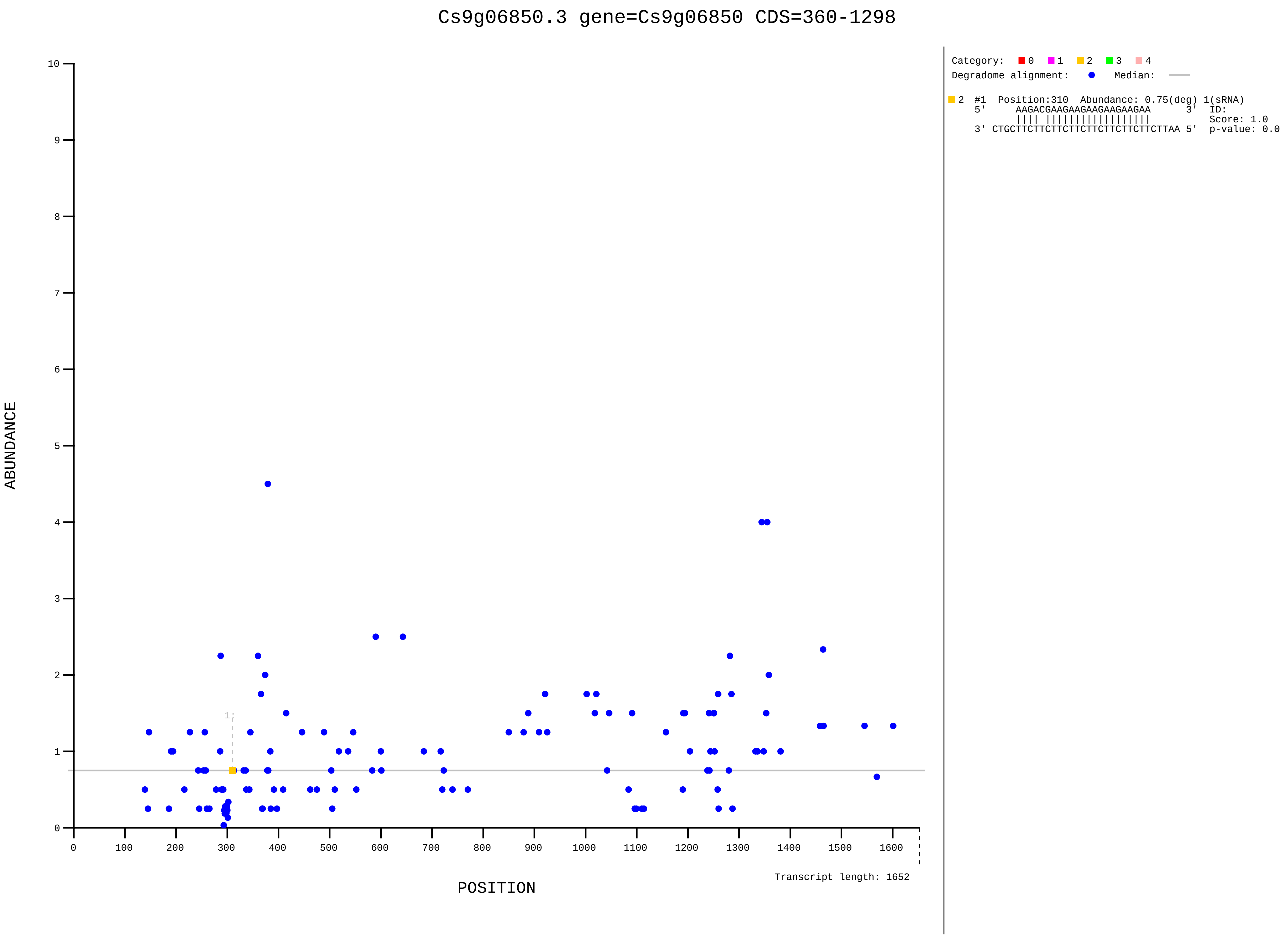

Cs9g06850.3 gene=Cs9g06850 CDS=360-1298
ABUNDANCE
0
1
2
3
4
5
6
7
8
9
10
POSITION
0
100
200
300
400
500
600
700
800
900
1000
1100
1200
1300
1400
1500
1600
Transcript length: 1652
Category: 0 1 2 3 4
Degradome alignment: Median:
2 #1 Position:310 Abundance: 0.75(deg) 1(sRNA)
5' AAGACGAAGAAGAAGAAGAA 3' ID:
Score: 1.0
3' CTGCTTCTTCTTCTTCTTCTTCTTCTTAA 5' p-value: 0.0

Cs9g06850.1 gene=Cs9g06850 CDS=291-1229

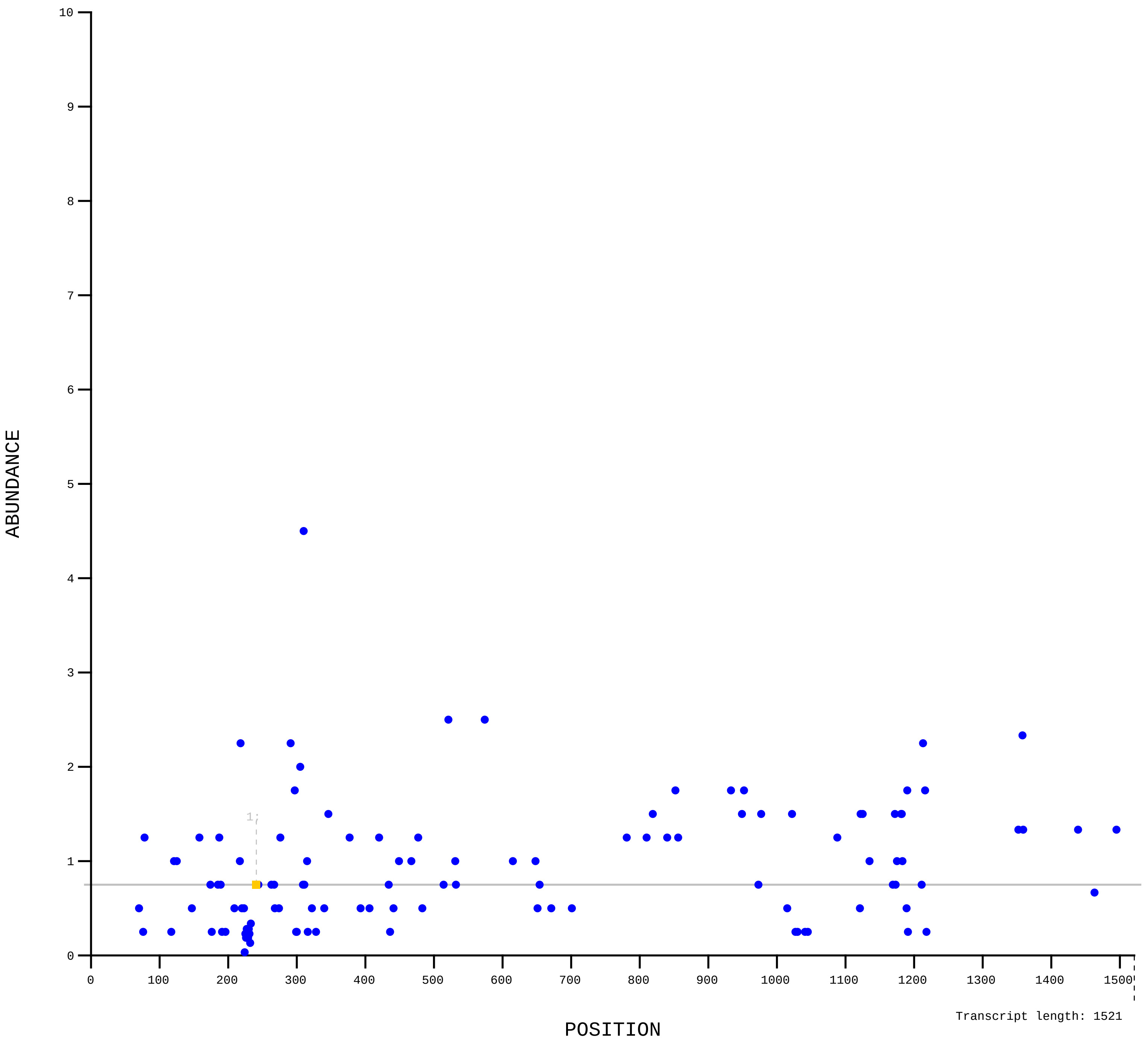

Category: 0 1 2 3 4
Degradome alignment: Median:

2 #1 Position:241 Abundance: 0.75(deg) 1(sRNA)

```
5'     AAGACGAAGAAGAAGAAGAAGAA      3'  ID:
       |||| |||||||||||||||||           Score: 1.0
3' CTGCTTCTTCTTCTTCTTCTTCTTCTTAA 5'  p-value: 0.0
```

Cs2g09770.5 gene=Cs2g09770 CDS=484-2712

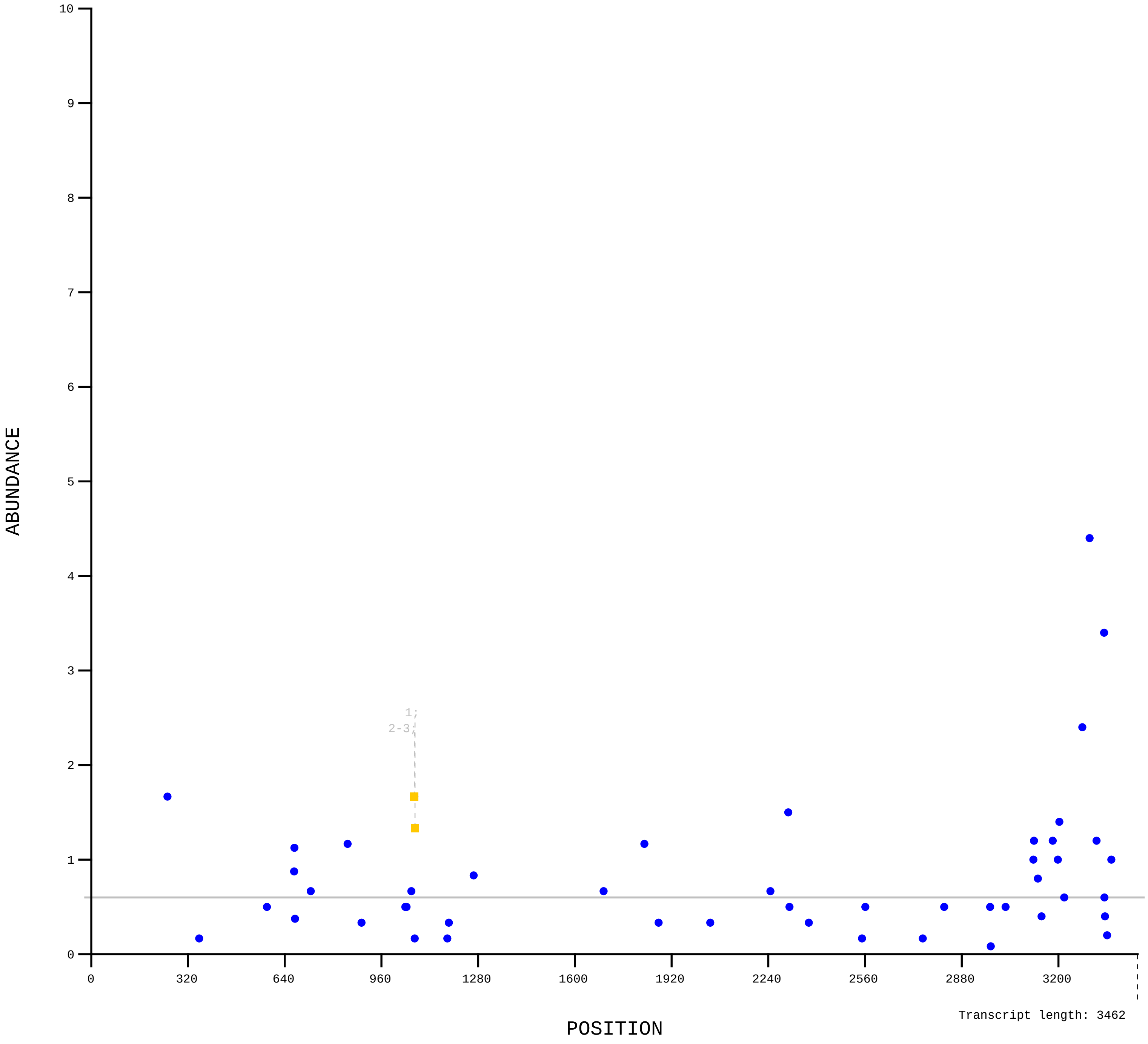

Category: 0 1 2 3 4
Degradome alignment: Median:

```
2  #1  Position:1071  Abundance: 1.33(deg) 2(sRNA)
   5'     TCTCGGACCAGGCTTCATTCC        3'  ID:
          || |||||||||||||||o||            Score: 1.5
   3' TCTTAG-GCCTGGTCCGAAGTAGGGTTAGTAG 5'  p-value: 0.01

2  #2  Position:1069  Abundance: 1.67(deg) 2(sRNA)
   5'     TCGGACCAGGCTTCATTCCCC        3'  ID:
          o||||||||||||||| |||             Score: 2.5
   3' CTTAGGCCTGGTCCGAAGTA-GGGTTAGTAGA 5'  p-value: 0.03

2  #3  Position:1069  Abundance: 1.67(deg) 1(sRNA)
   5'     TCGGACCAGGCTTCATTCCCT        3'  ID:
          o||||||||||||||| |||             Score: 2.5
   3' CTTAGGCCTGGTCCGAAGTA-GGGTTAGTAGA 5'  p-value: 0.01
```

Cs2g09770.3 gene=Cs2g09770 CDS=484-3039

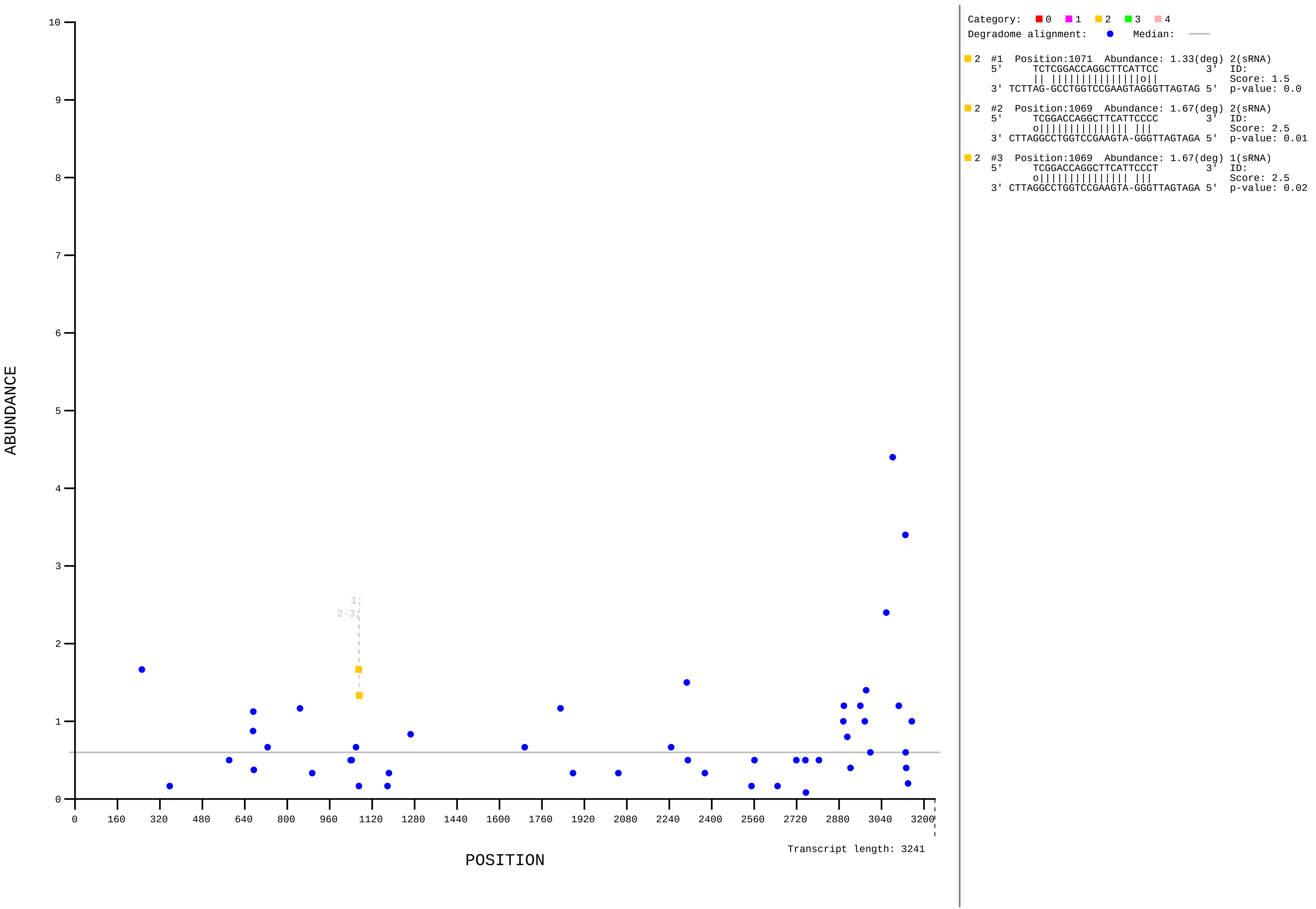

Cs2g09770.2 gene=Cs2g09770 CDS=484-3036

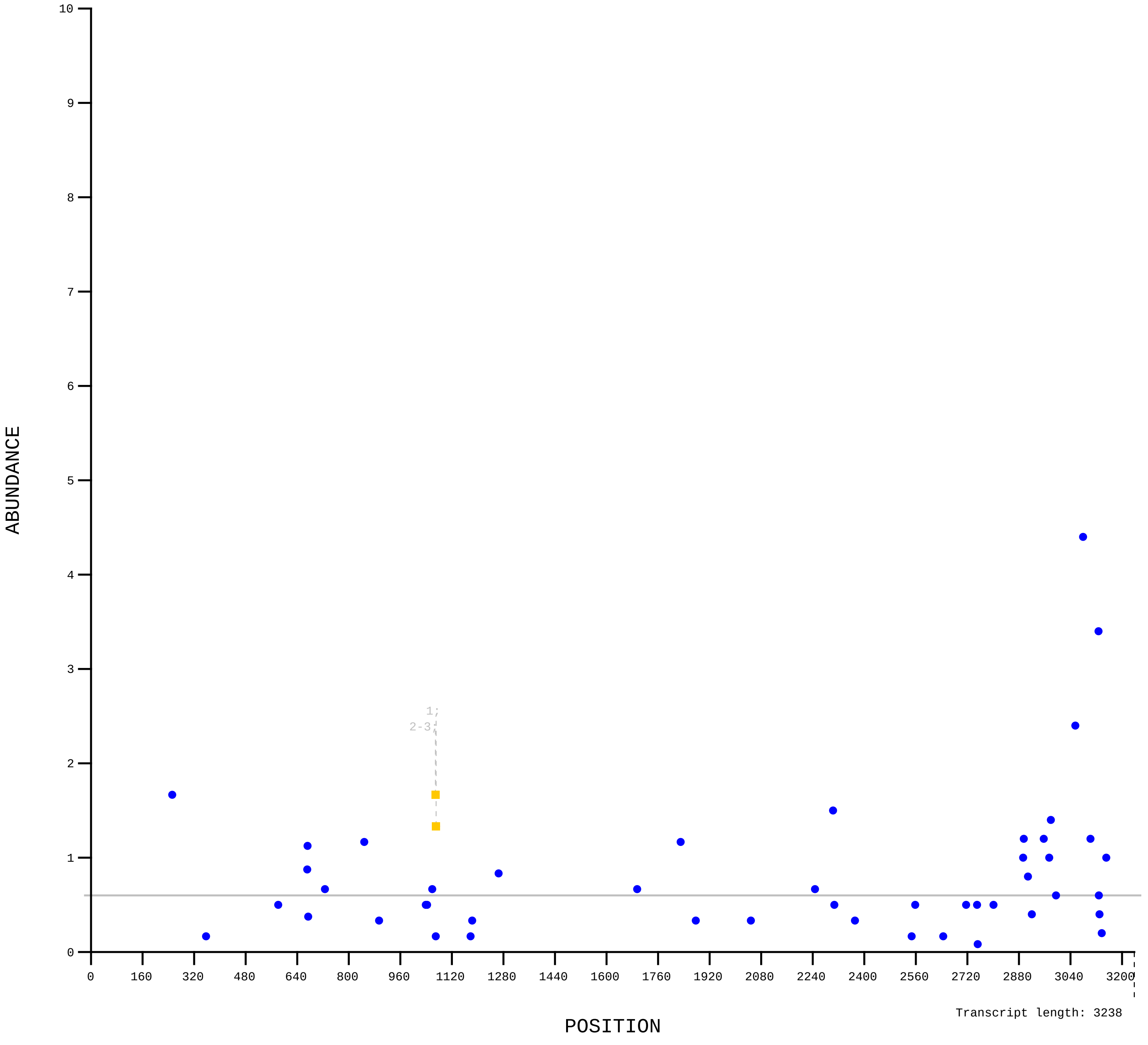


```
Category:  0  1  2  3  4
Degradome alignment:     Median:

2  #1  Position:1071  Abundance: 1.33(deg) 2(sRNA)
   5'     TCTCGGACCAGGCTTCATTCC        3'  ID:
          || |||||||||||||||o||            Score: 1.5
   3' TCTTAG-GCCTGGTCCGAAGTAGGGTTAGTAG 5'  p-value: 0.0

2  #2  Position:1069  Abundance: 1.67(deg) 2(sRNA)
   5'     TCGGACCAGGCTTCATTCCCC        3'  ID:
          o|||||||||||||||| |||            Score: 2.5
   3' CTTAGGCCTGGTCCGAAGTA-GGGTTAGTAGA 5'  p-value: 0.01

2  #3  Position:1069  Abundance: 1.67(deg) 1(sRNA)
   5'     TCGGACCAGGCTTCATTCCCT        3'  ID:
          o|||||||||||||||| |||            Score: 2.5
   3' CTTAGGCCTGGTCCGAAGTA-GGGTTAGTAGA 5'  p-value: 0.01
```

Cs2g09770.1 gene=Cs2g09770 CDS=959-3025

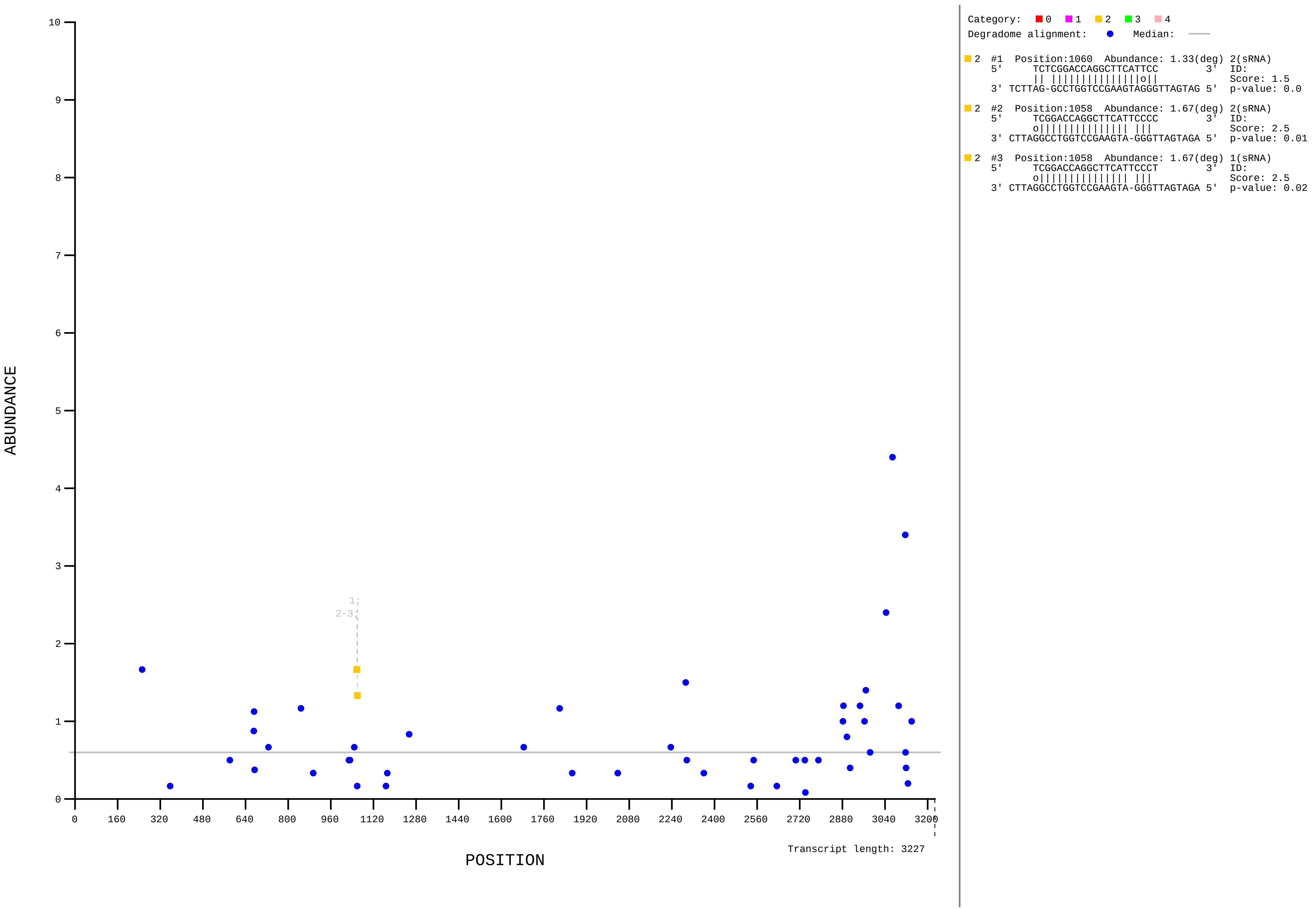

Cs2g09770.6 gene=Cs2g09770 CDS=1146-3131

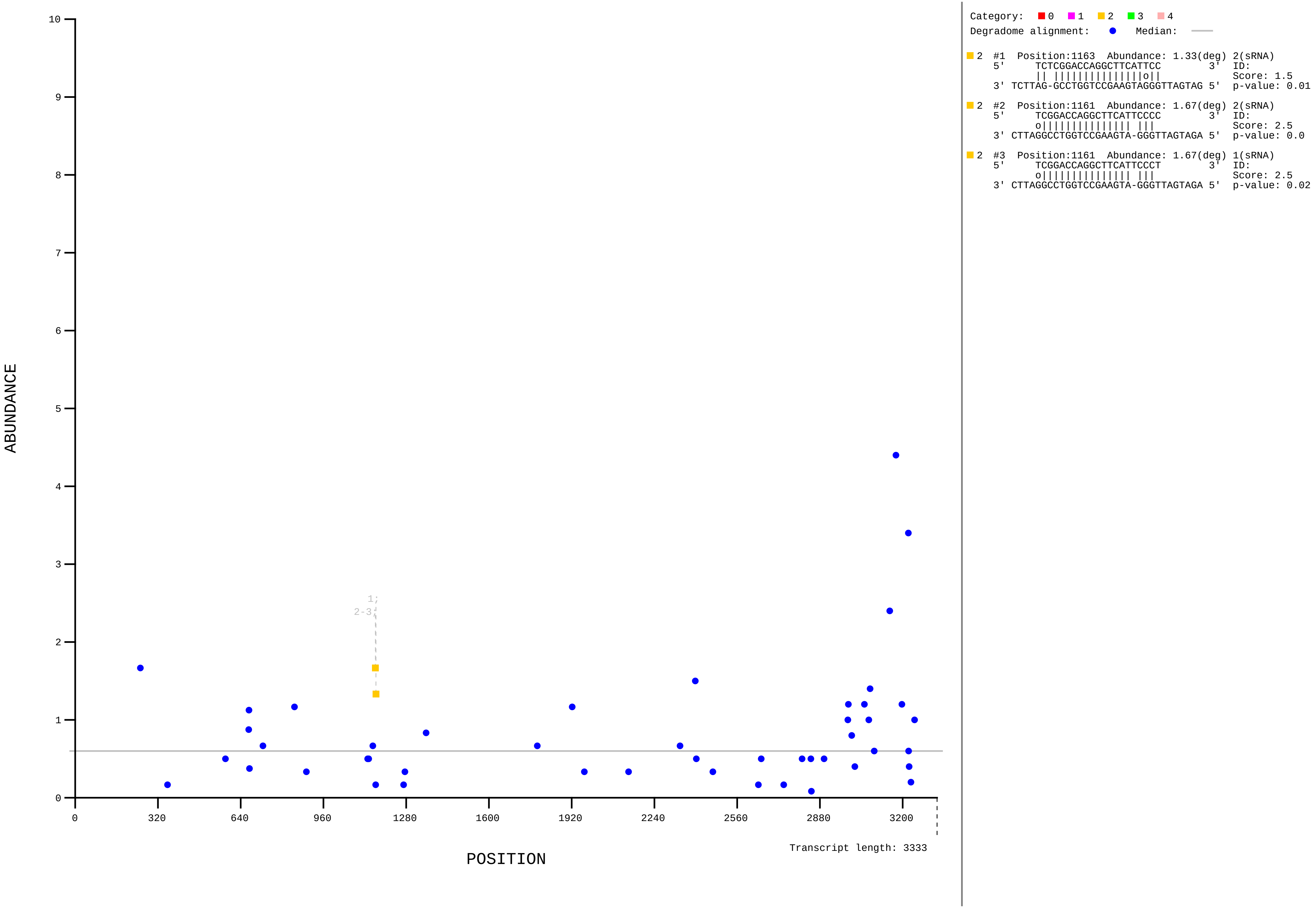

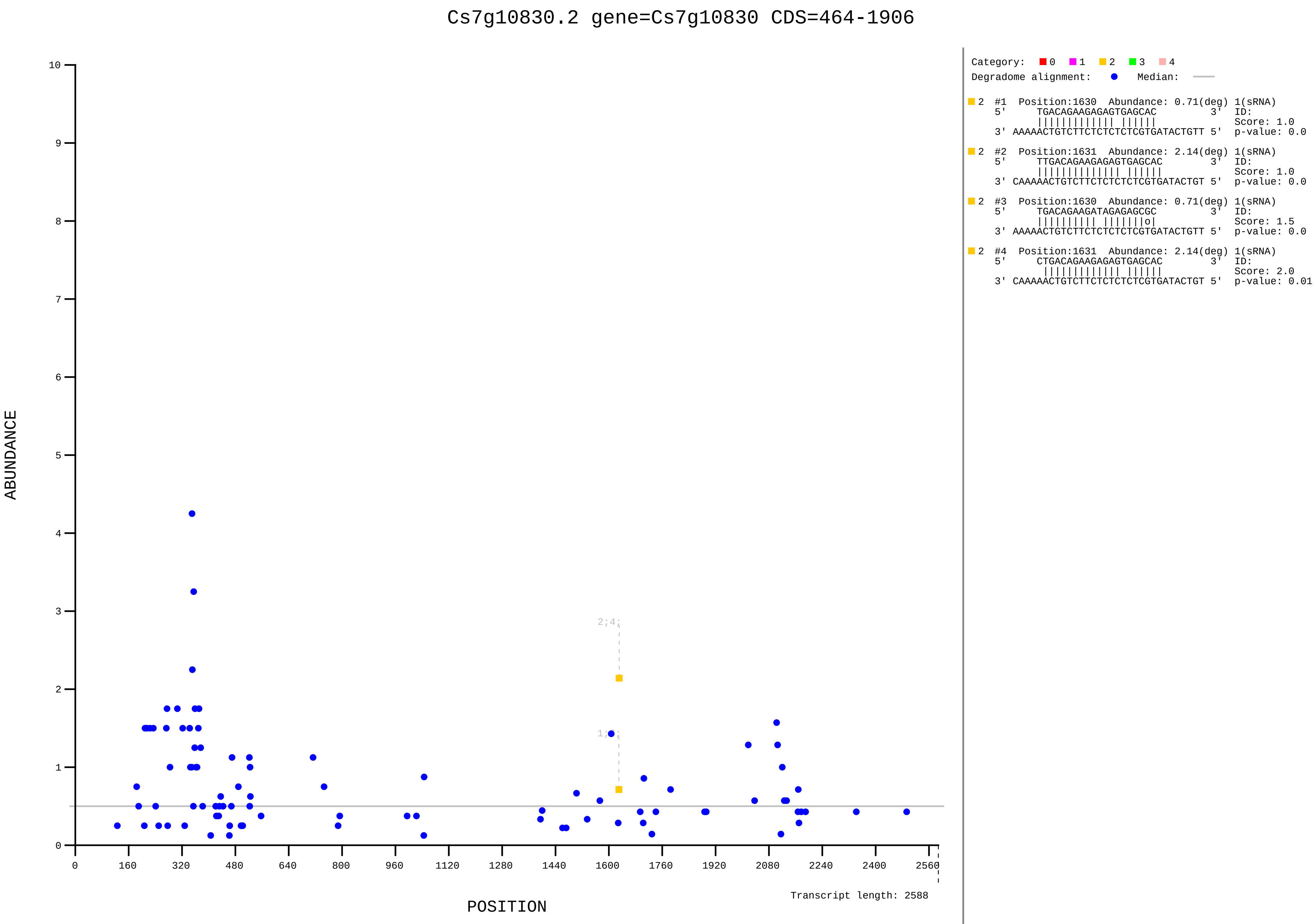
Cs7g10830.2 gene=Cs7g10830 CDS=464-1906
ABUNDANCE
POSITION
Transcript length: 2588
2;4;
1;3;
Category: 0 1 2 3 4
Degradome alignment:
Median:
2 #1 Position:1630 Abundance: 0.71(deg) 1(sRNA)
5' TGACAGAAGAGAGTGAGCAC 3' ID:
||||||||||||| ||||||
Score: 1.0
3' AAAAACTGTCTTCTCTCTCTCGTGATACTGTT 5' p-value: 0.0
2 #2 Position:1631 Abundance: 2.14(deg) 1(sRNA)
5' TTGACAGAAGAGAGTGAGCAC 3' ID:
|||||||||||||| ||||||
Score: 1.0
3' CAAAAACTGTCTTCTCTCTCTCGTGATACTGT 5' p-value: 0.0
2 #3 Position:1630 Abundance: 0.71(deg) 1(sRNA)
5' TGACAGAAGATAGAGAGCGC 3' ID:
|||||||||| |||||||o|
Score: 1.5
3' AAAAACTGTCTTCTCTCTCTCGTGATACTGTT 5' p-value: 0.0
2 #4 Position:1631 Abundance: 2.14(deg) 1(sRNA)
5' CTGACAGAAGAGAGTGAGCAC 3' ID:
||||||||||||| ||||||
Score: 2.0
3' CAAAAACTGTCTTCTCTCTCTCGTGATACTGT 5' p-value: 0.01

Cs7g10830.7 gene=Cs7g10830 CDS=464-1906

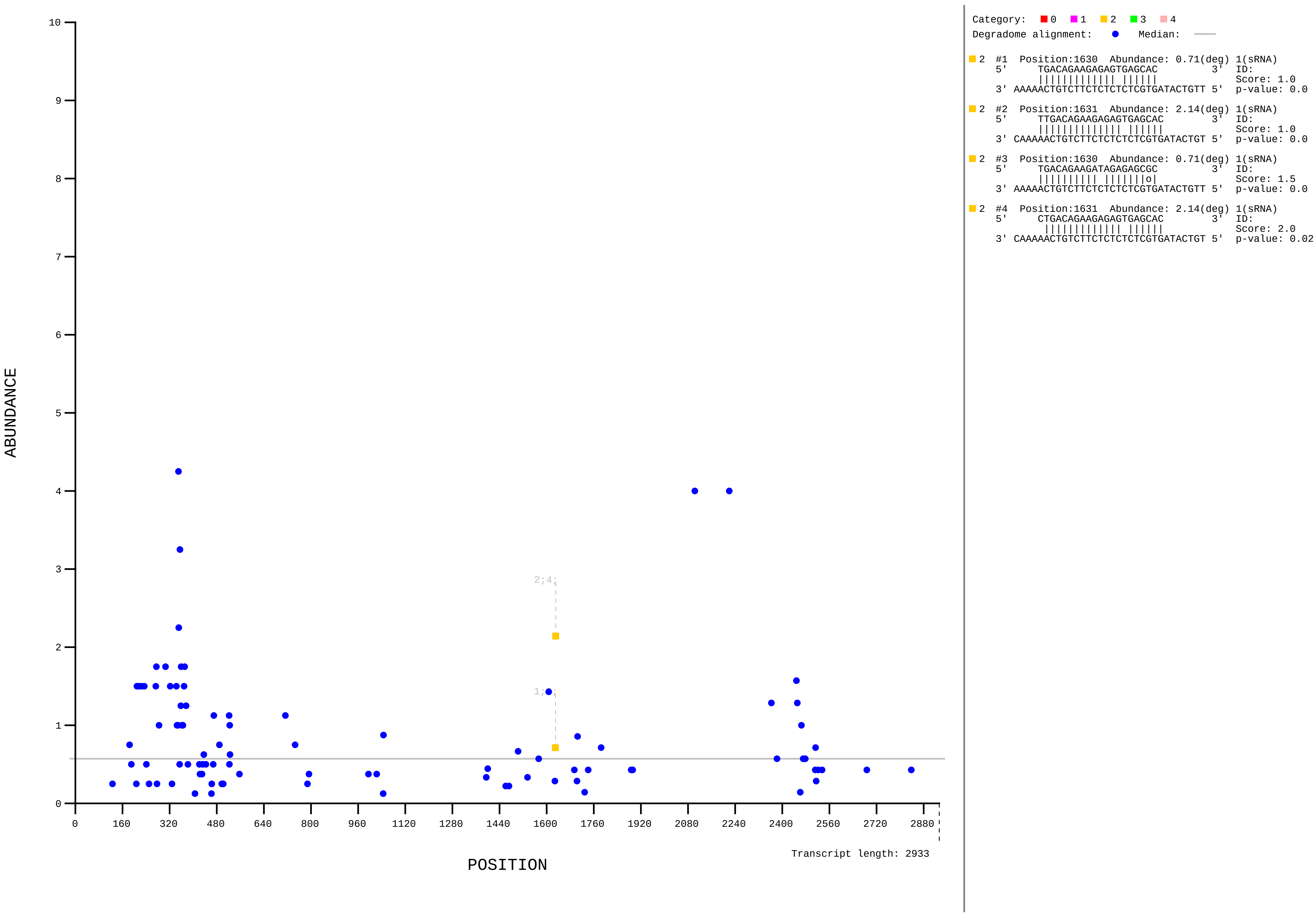

Cs7g10830.5 gene=Cs7g10830 CDS=464-1906

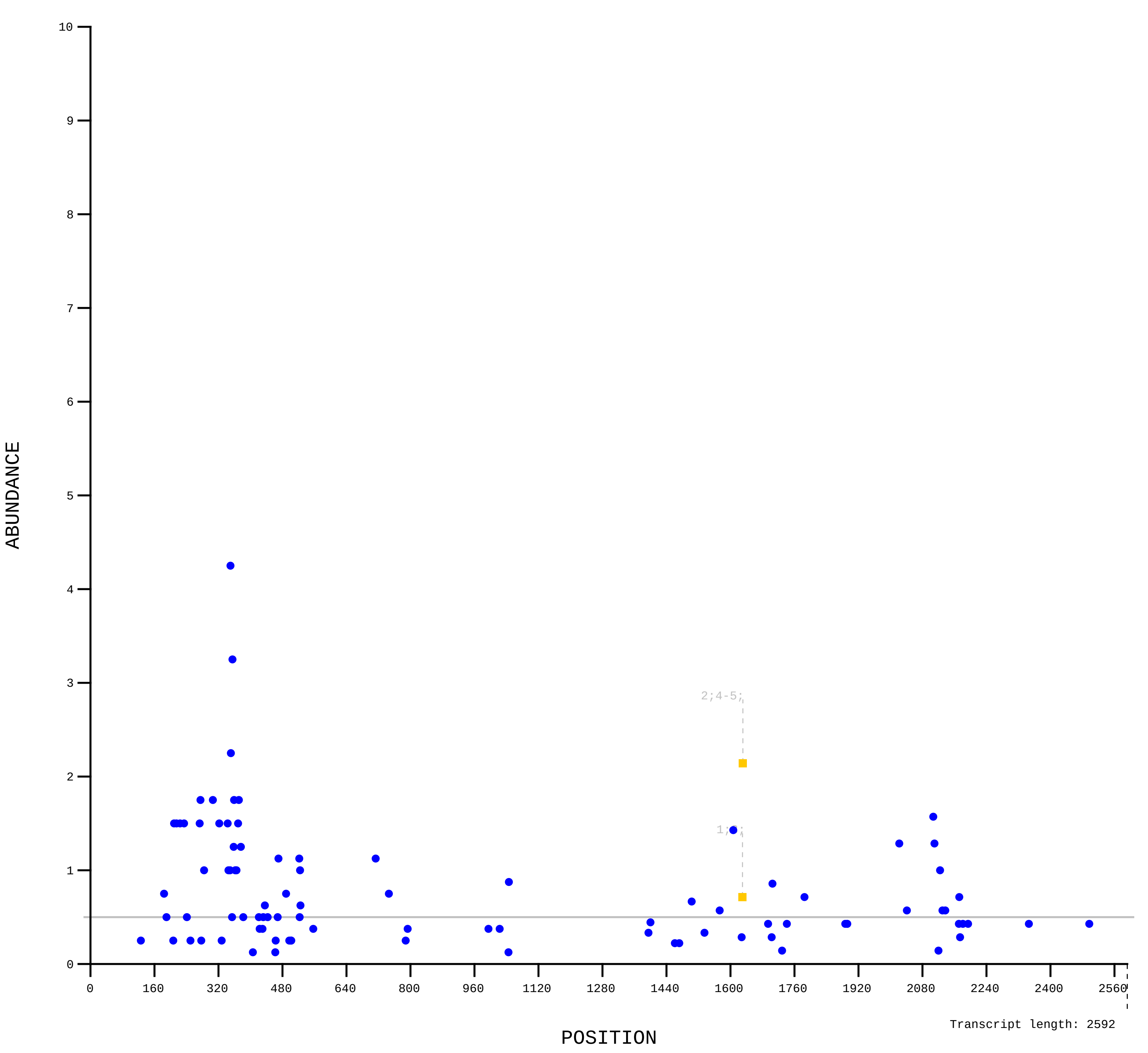


```
2  #1  Position:1630  Abundance: 0.71(deg) 1(sRNA)
   5'      TGACAGAAGAGAGTGAGCAC          3'  ID:
           ||||||||||||| ||||||              Score: 1.0
   3' AAAAACTGTCTTCTCTCTCTCGTGATACTGTT 5'  p-value: 0.0

2  #2  Position:1631  Abundance: 2.14(deg) 1(sRNA)
   5'      TTGACAGAAGAGAGTGAGCAC         3'  ID:
           |||||||||||||| ||||||             Score: 1.0
   3' CAAAAACTGTCTTCTCTCTCTCGTGATACTGT 5'  p-value: 0.0

2  #3  Position:1630  Abundance: 0.71(deg) 1(sRNA)
   5'      TGACAGAAGATAGAGAGCGC          3'  ID:
           |||||||||| |||||||o|              Score: 1.5
   3' AAAAACTGTCTTCTCTCTCTCGTGATACTGTT 5'  p-value: 0.01

2  #4  Position:1631  Abundance: 2.14(deg) 1(sRNA)
   5'      CTGACAGAAGAGAGTGAGCAC         3'  ID:
            ||||||||||||| ||||||             Score: 2.0
   3' CAAAAACTGTCTTCTCTCTCTCGTGATACTGT 5'  p-value: 0.01

2  #5  Position:1631  Abundance: 2.14(deg) 1(sRNA)
   5'      GTGACAGAAGATAGAGAGCGC         3'  ID:
            |||||||||| |||||||o|             Score: 2.5
   3' CAAAAACTGTCTTCTCTCTCTCGTGATACTGT 5'  p-value: 0.03
```

Cs5g05370.2 gene=Cs5g05370 CDS=332-2515

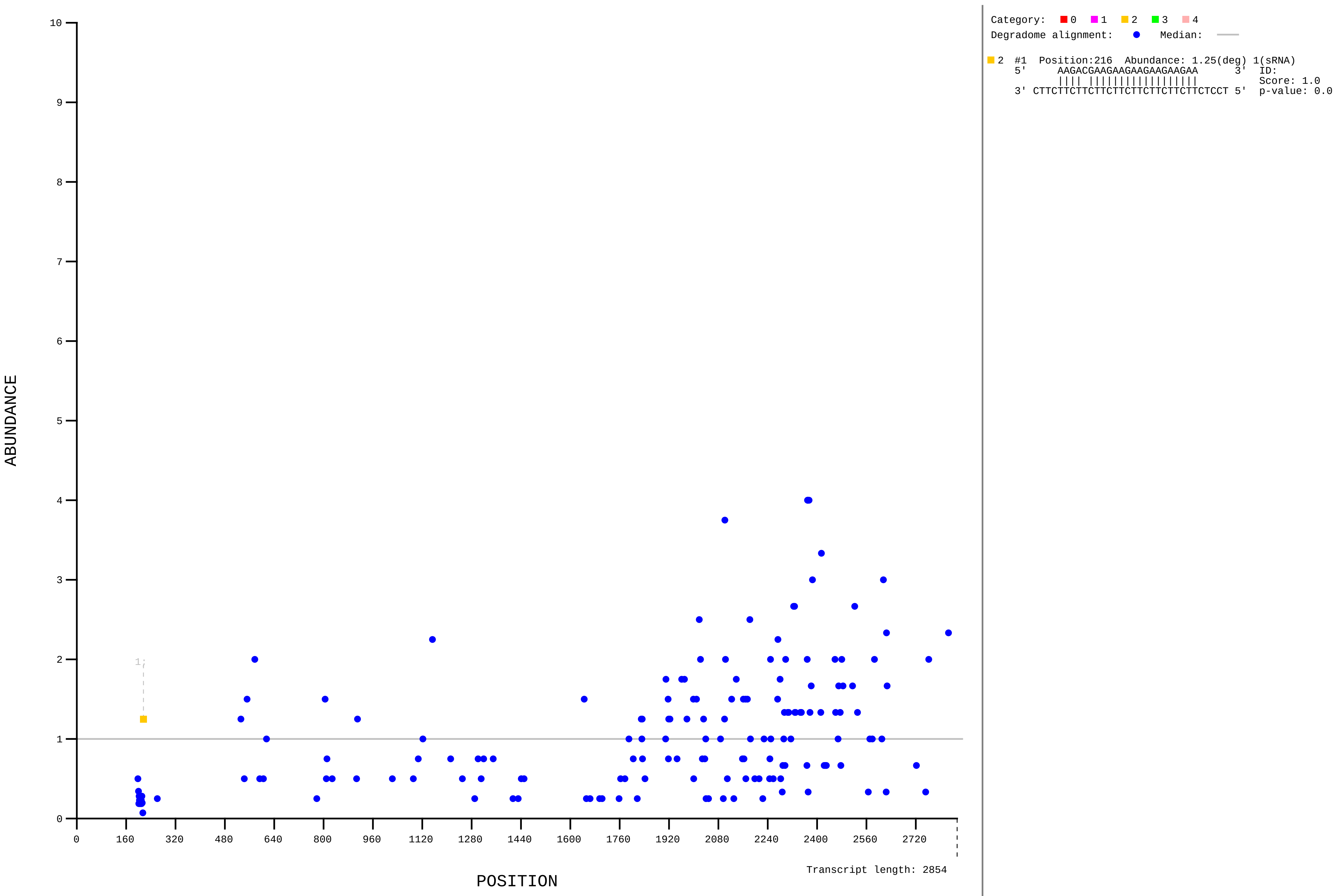

Cs3g13430.2 gene=Cs3g13430 CDS=613-4896

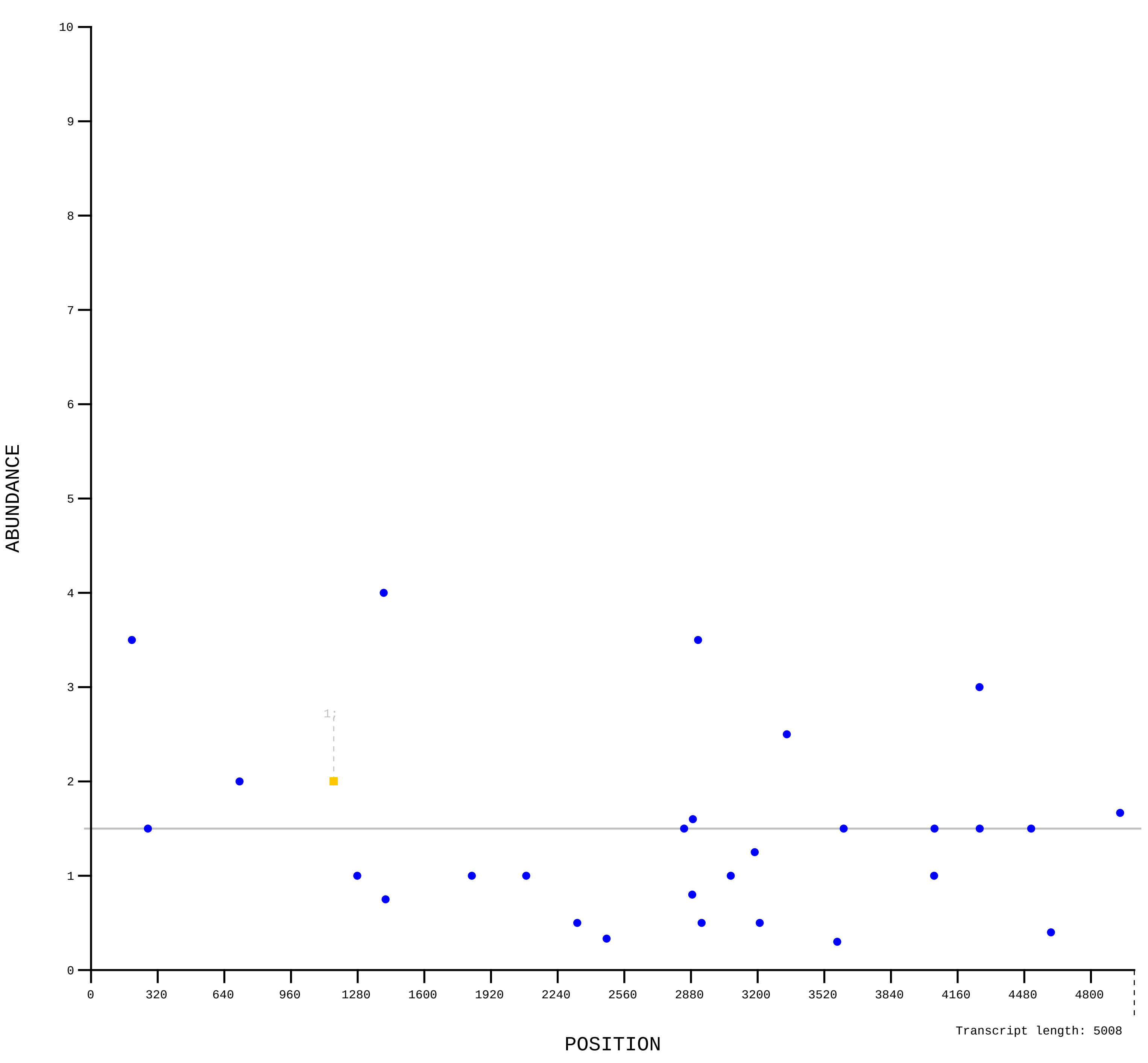

Category: 0 1 2 3 4
Degradome alignment: Median:

```
2  #1  Position:1165  Abundance: 2.00(deg) 1(sRNA)
   5'     TCTTCCCTATGCCTCCCATTCC       3'  ID:
          ||||||| ||||| ||||| ||           Score: 3.0
   3' CACCAGAAGGGTTACGGCGGGTACGGCATGTG 5'  p-value: 0.05
```

Cs5g05370.3 gene=Cs5g05370 CDS=471-2654

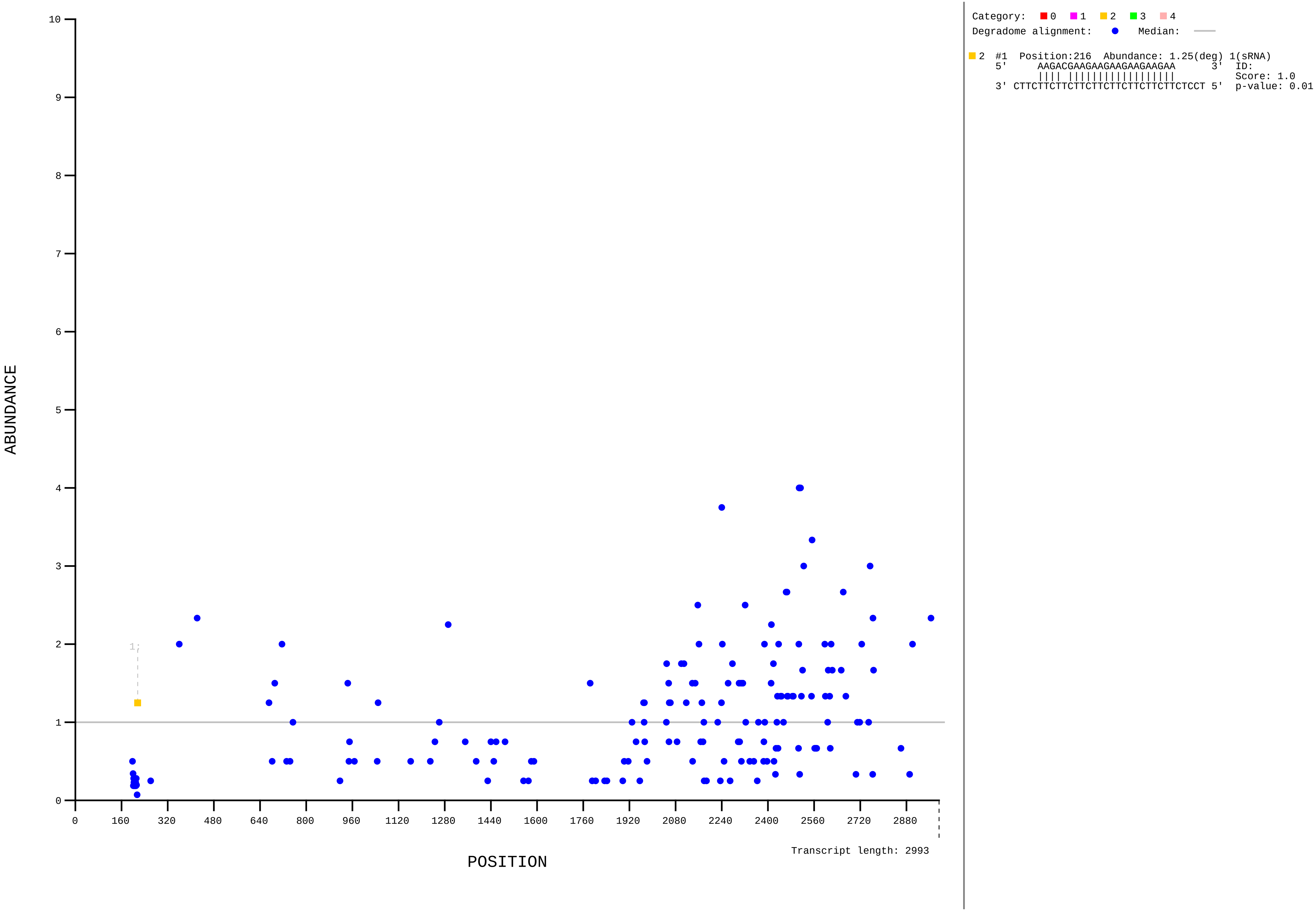

Cs1g14090.1 gene=Cs1g14090 CDS=1-2619

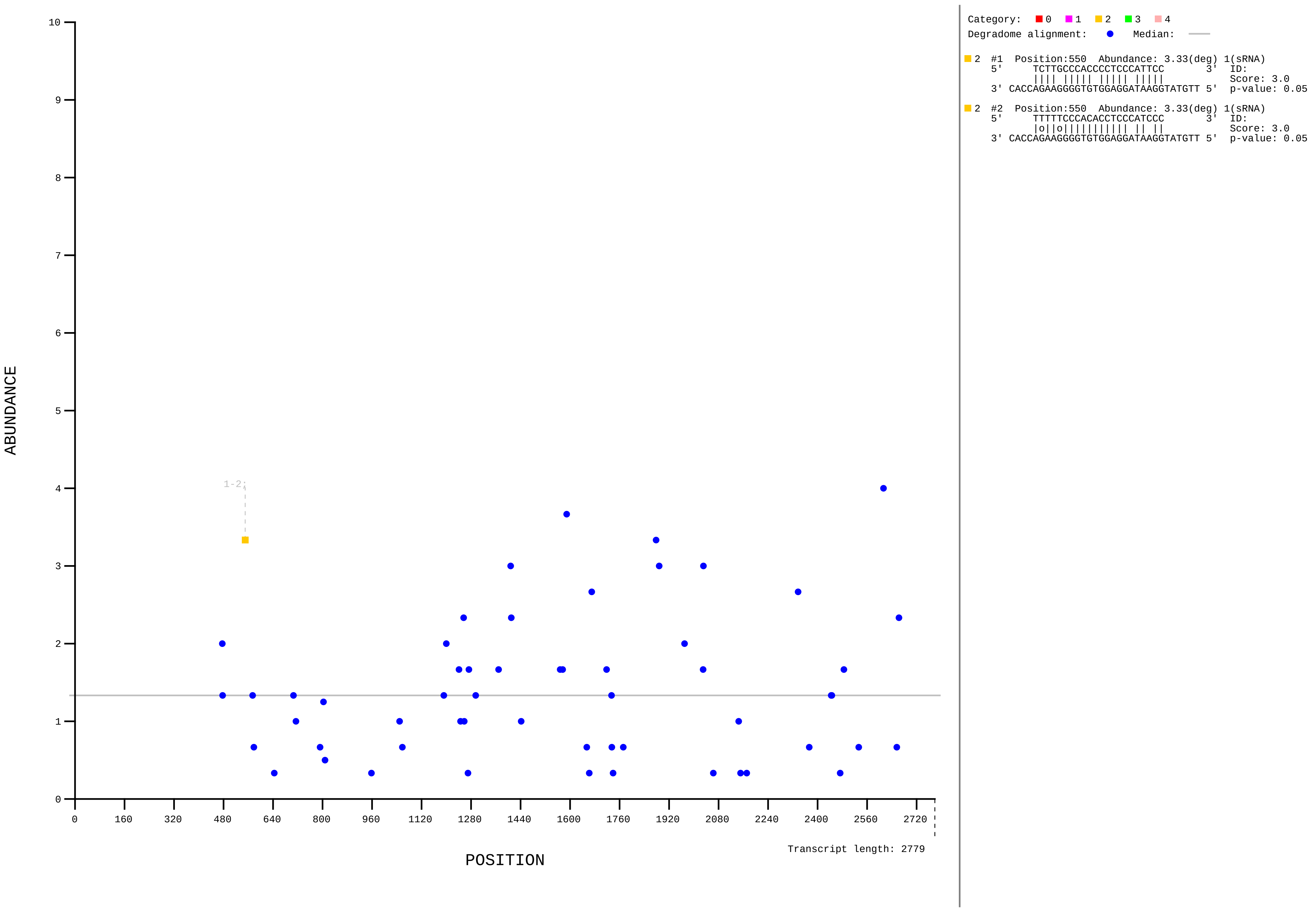

Cs5g05370.1 gene=Cs5g05370 CDS=387-2570

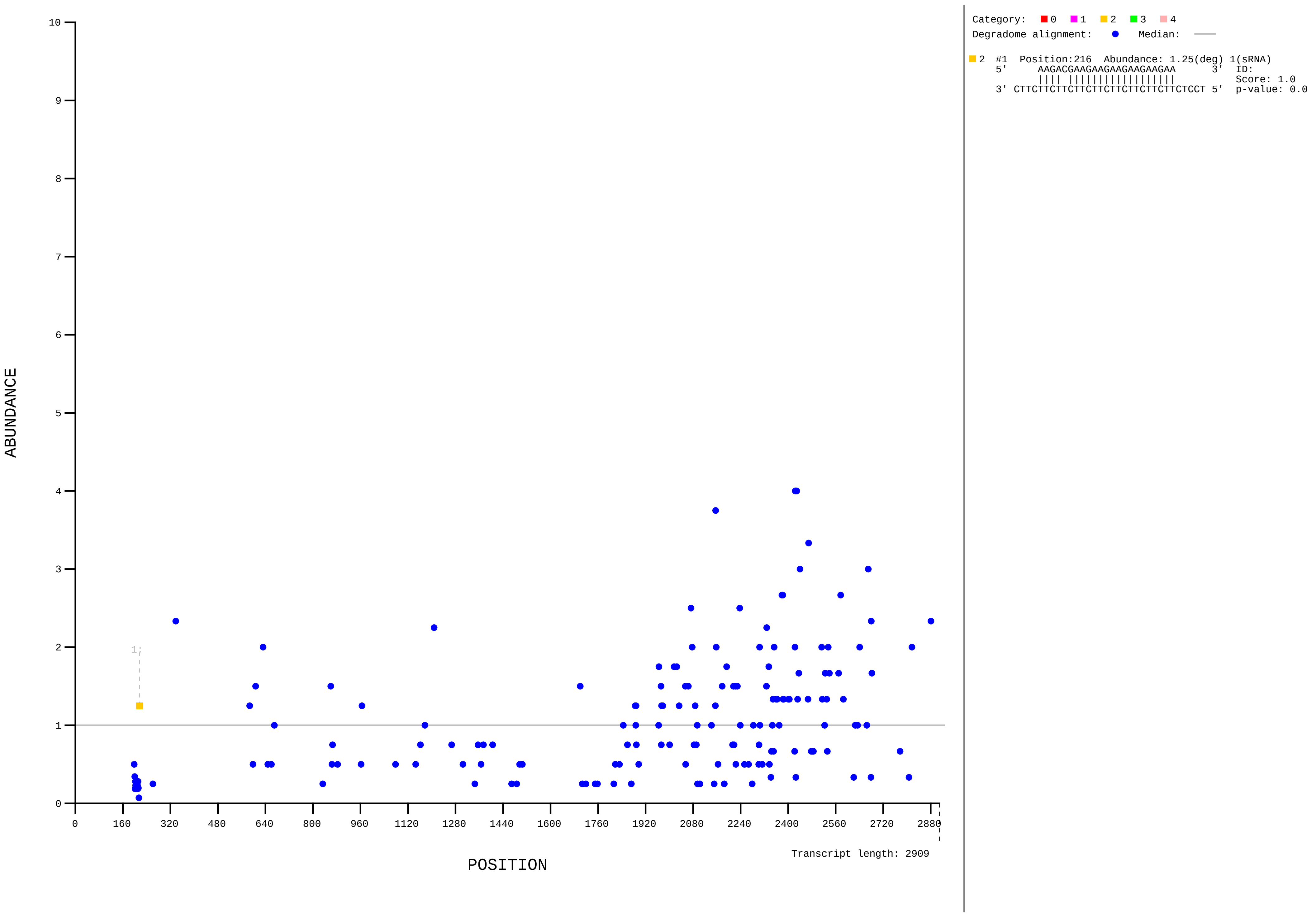

Cs5g05370.4 gene=Cs5g05370 CDS=471-2477

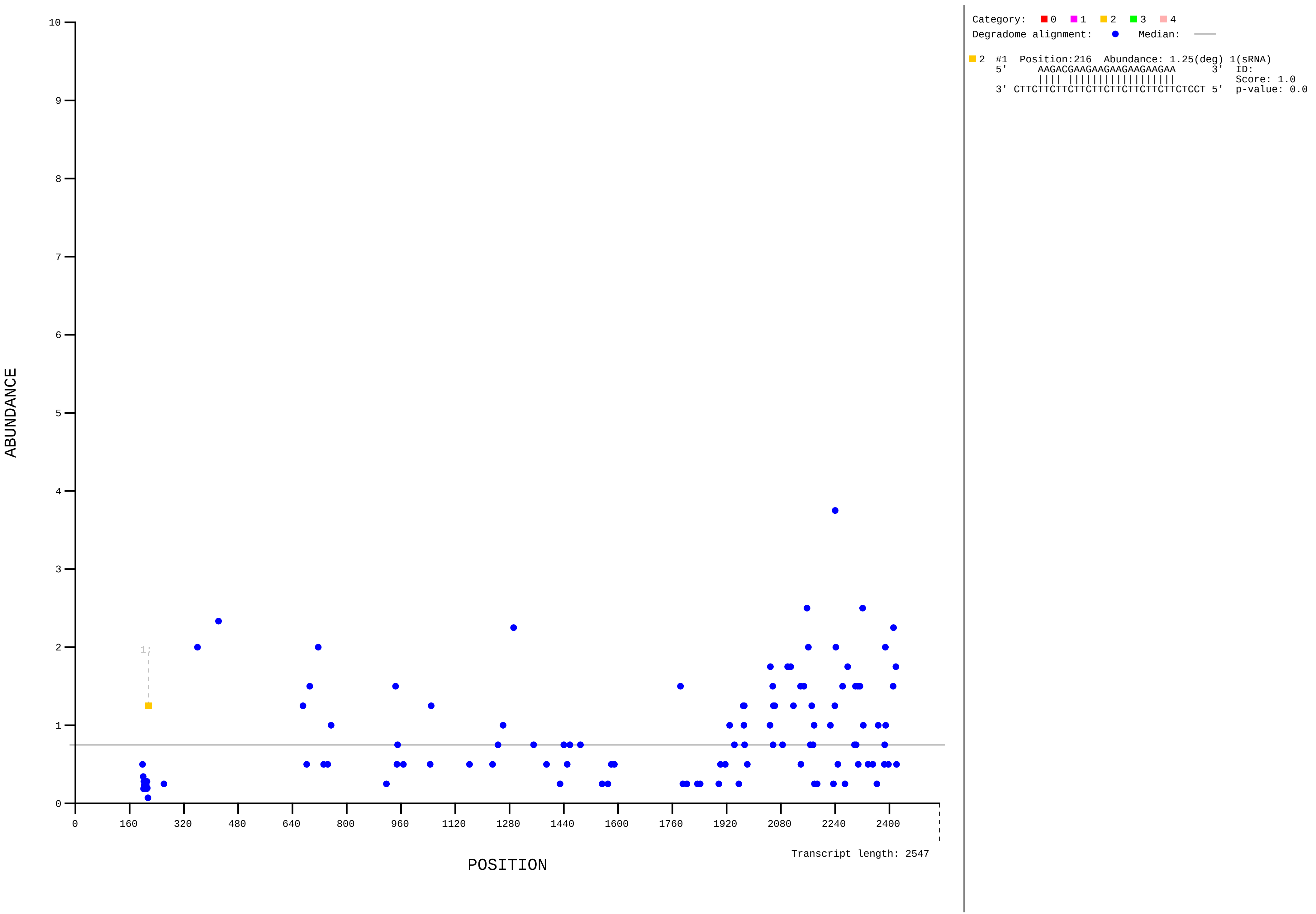

Cs1g14090.2 gene=Cs1g14090 CDS=467-3085

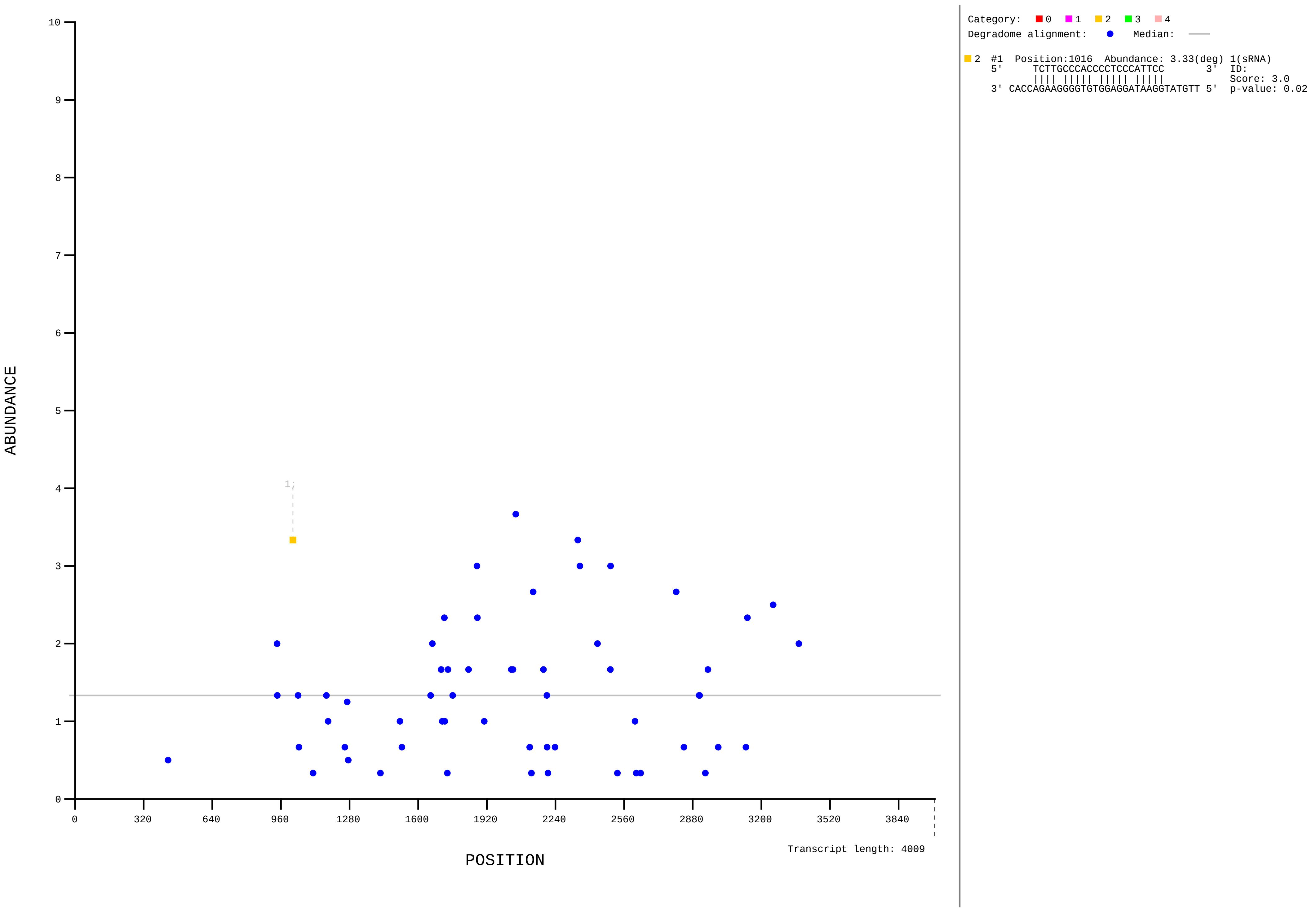

Cs1g14090.3 gene=Cs1g14090 CDS=467-3085

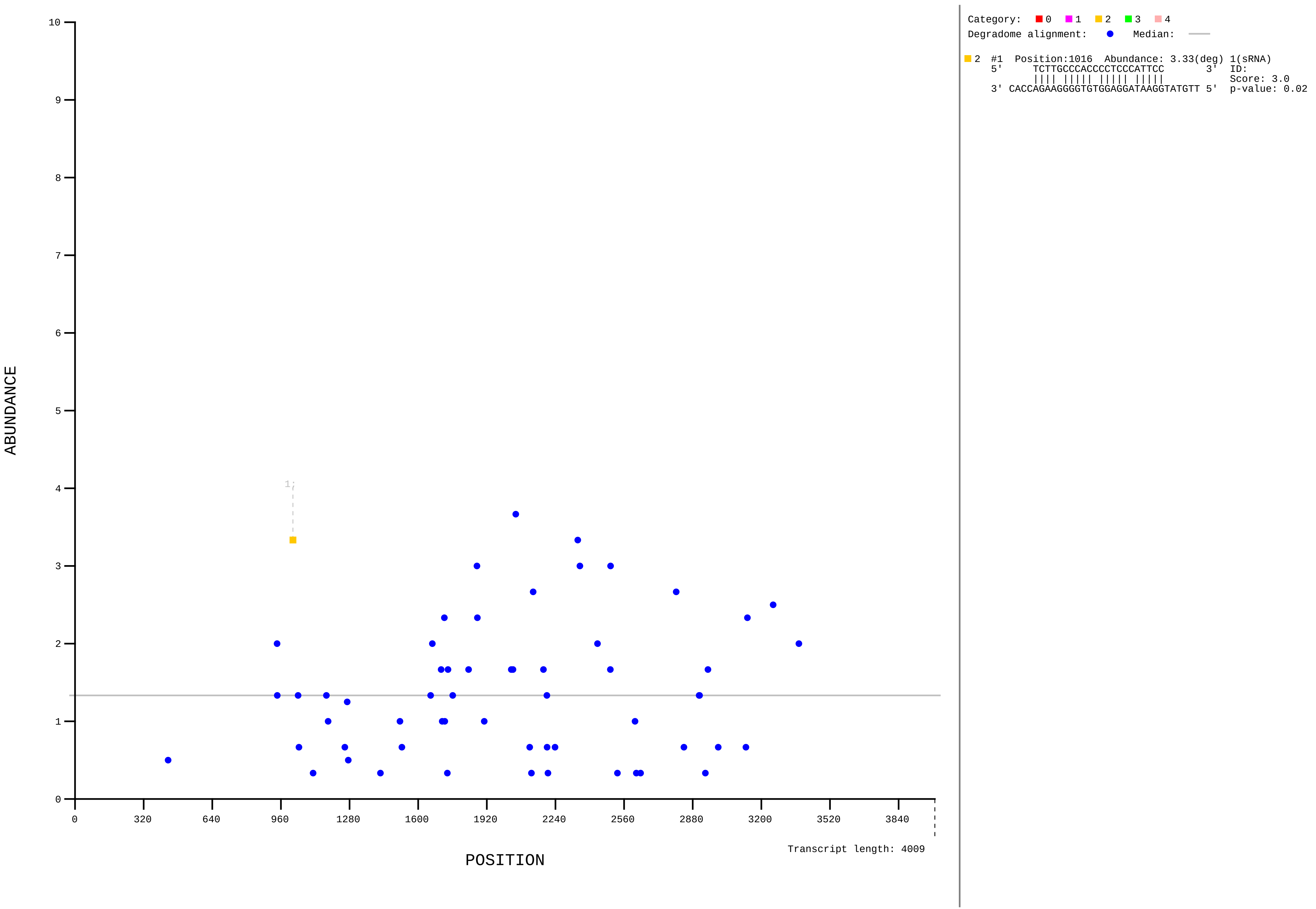

Cs8g11620.1 gene=Cs8g11620 CDS=1-1533

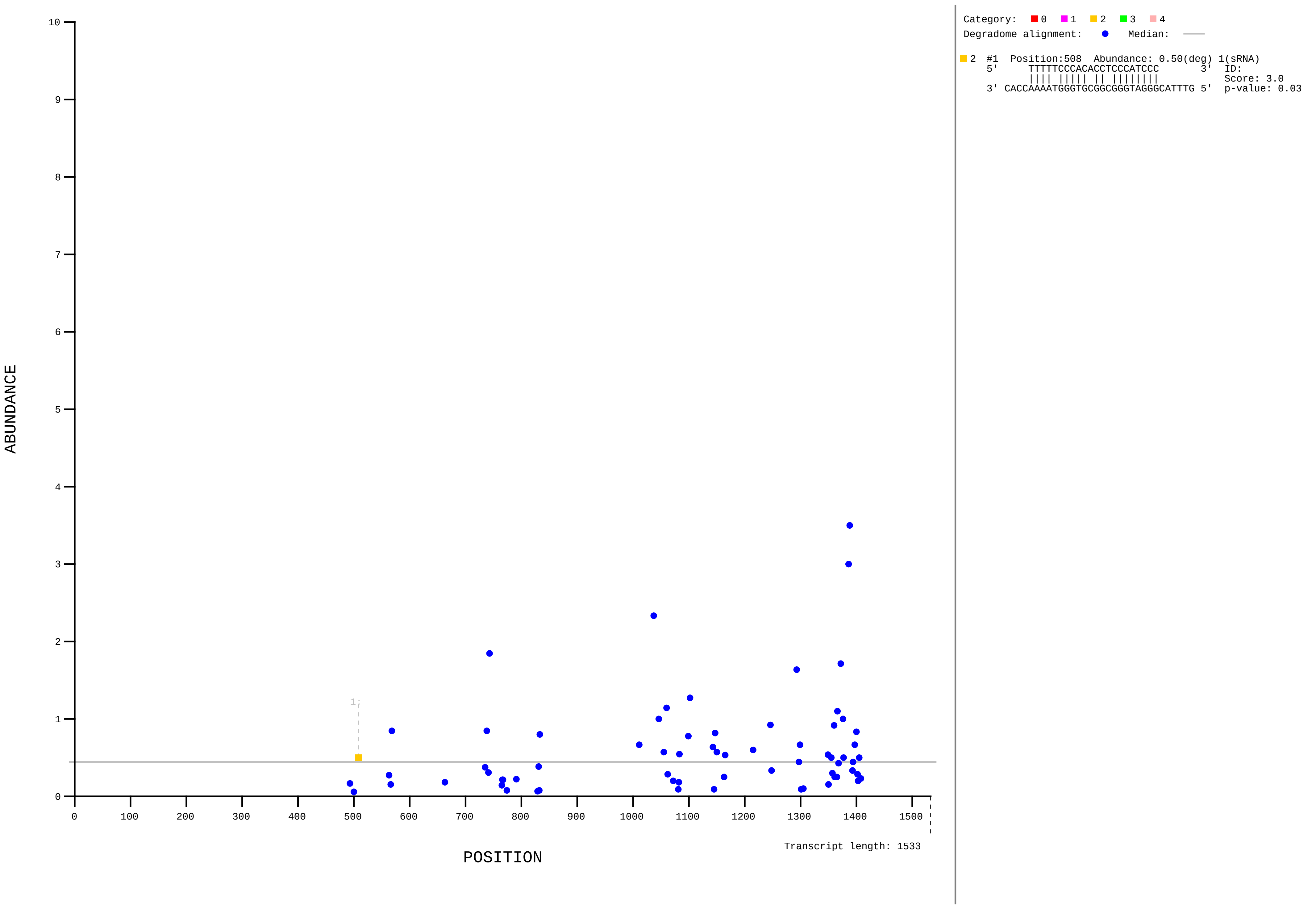

Cs3g11120.1 gene=Cs3g11120 CDS=1-1371

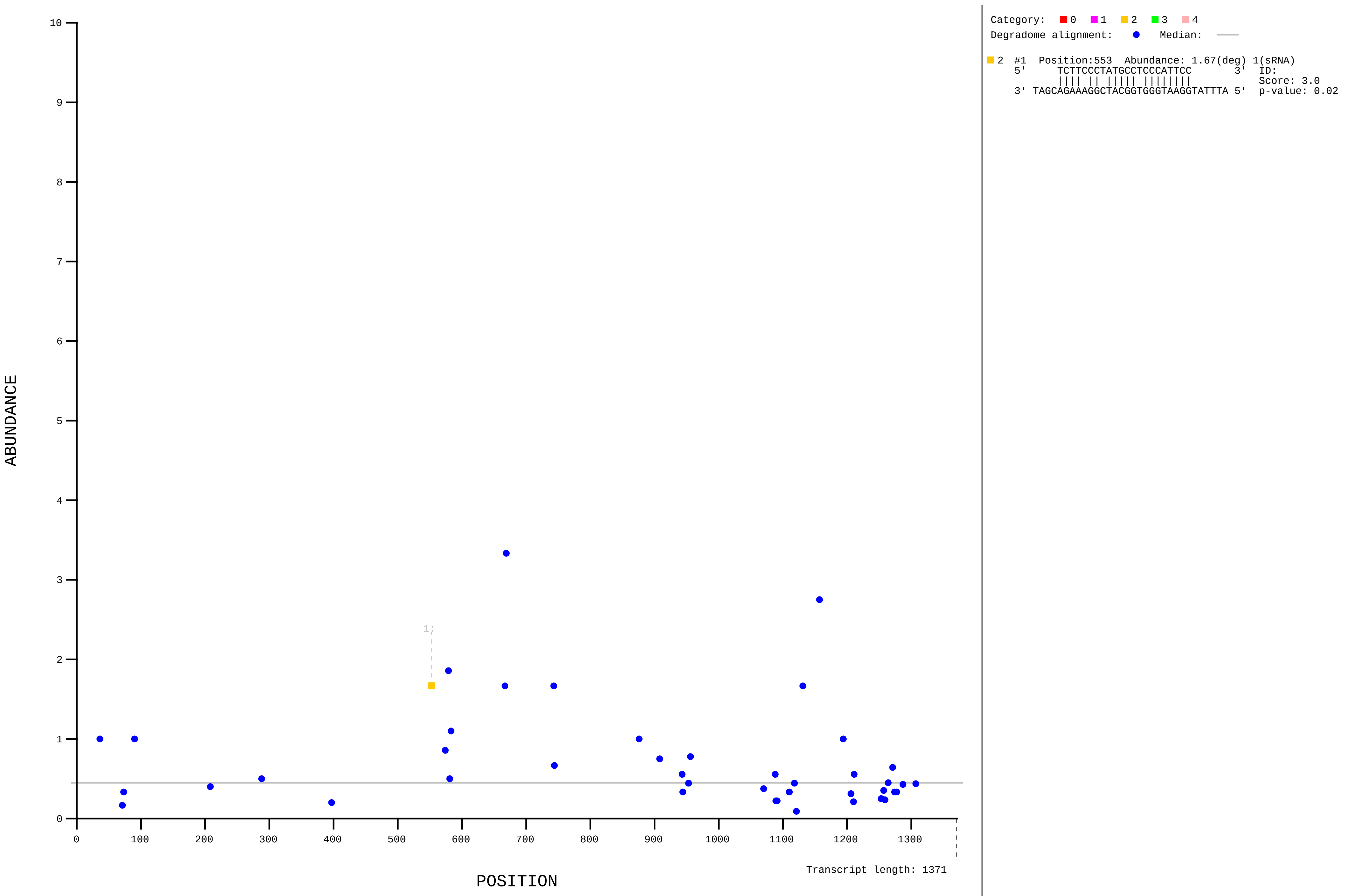

Cs6g03520.3 gene=Cs6g03520 CDS=243-3659

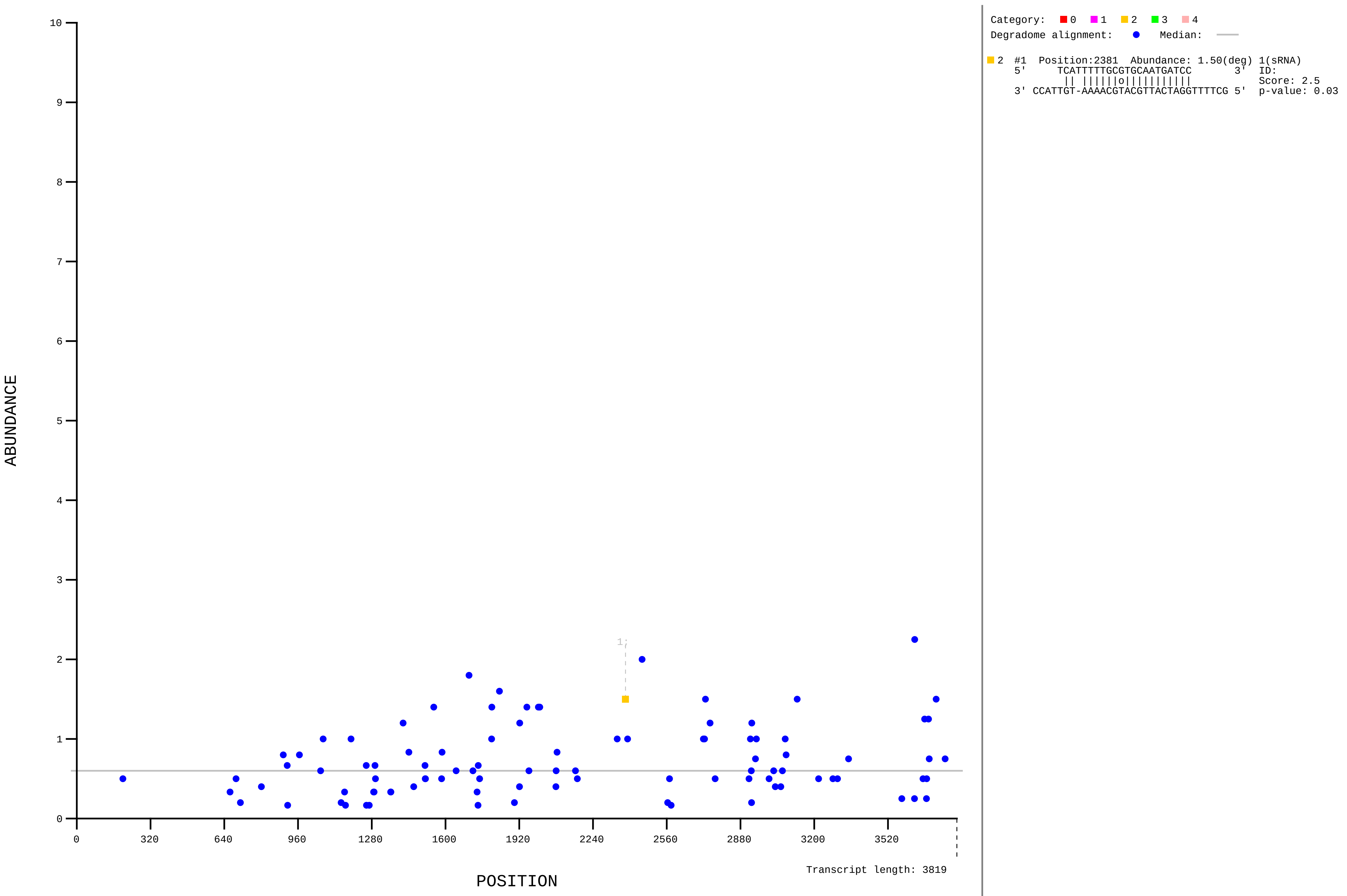

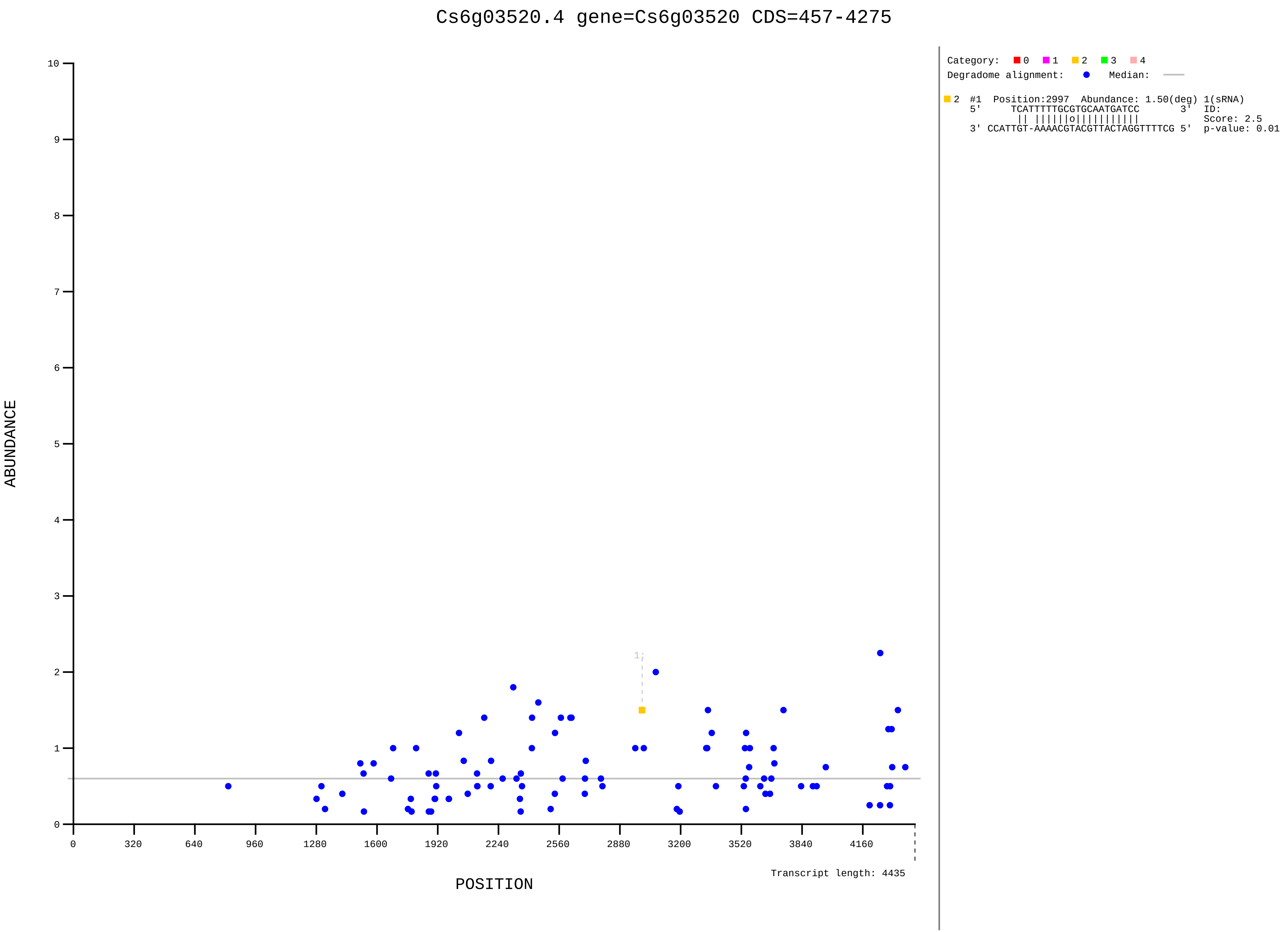

Cs6g03520.4 gene=Cs6g03520 CDS=457-4275
Category: 0 1 2 3 4
Degradome alignment:
Median:
2 #1 Position:2997 Abundance: 1.50(deg) 1(sRNA)
5' TCATTTTTGCGTGCAATGATCC 3' ID:
|| ||||||o|||||||||||
3' CCATTGT-AAAACGTACGTTACTAGGTTTTCG 5'
Score: 2.5
p-value: 0.01
ABUNDANCE
POSITION
Transcript length: 4435

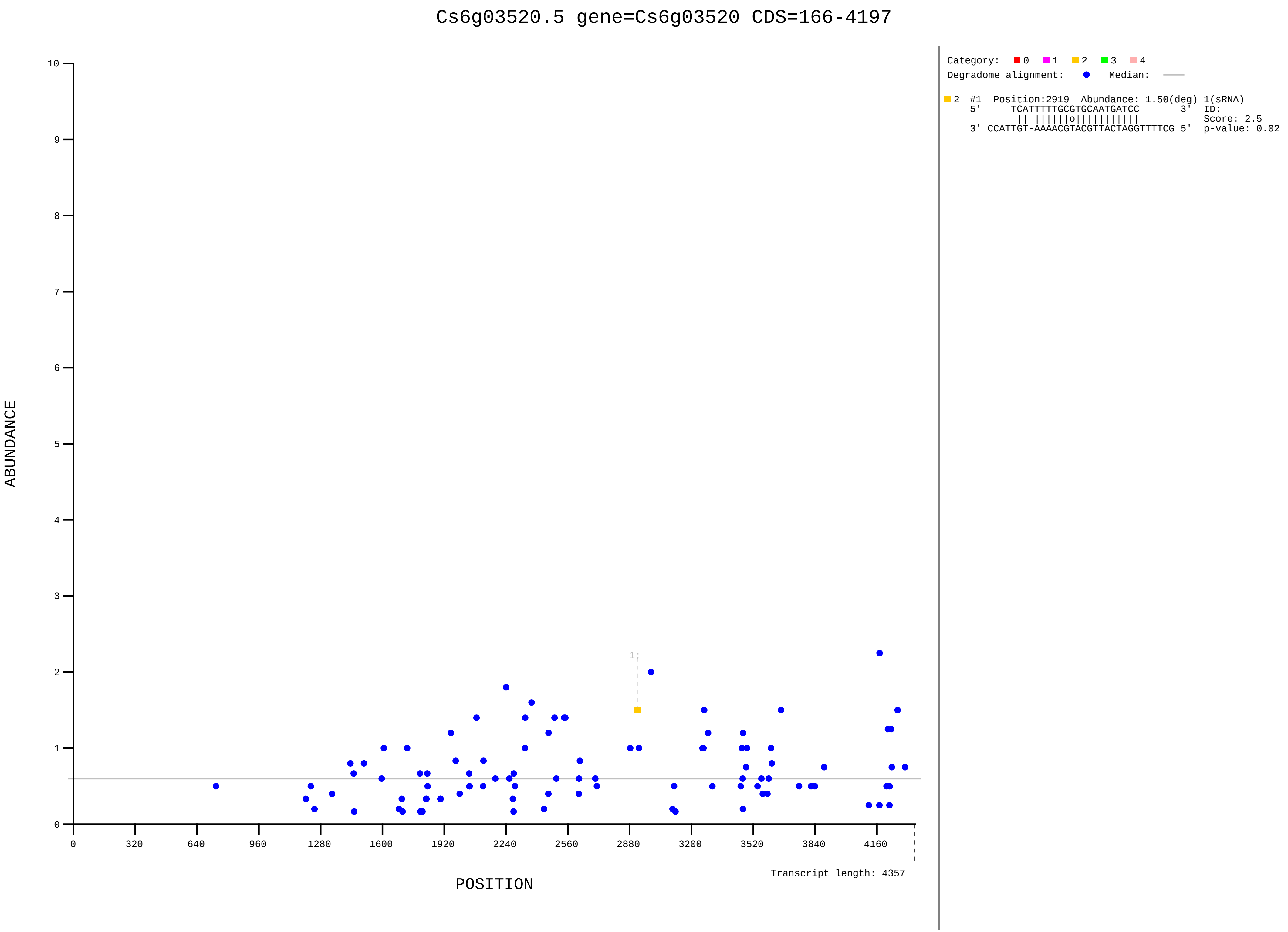

Cs6g03520.5 gene=Cs6g03520 CDS=166-4197
ABUNDANCE
0
1
2
3
4
5
6
7
8
9
10
POSITION
0
320
640
960
1280
1600
1920
2240
2560
2880
3200
3520
3840
4160
Transcript length: 4357
1
Category: 0 1 2 3 4
Degradome alignment:
Median:
2 #1 Position:2919 Abundance: 1.50(deg) 1(sRNA)
5' TCATTTTTGCGTGCAATGATCC 3'
|| ||||||o|||||||||||
3' CCATTGT-AAAACGTACGTTACTAGGTTTTCG 5'
ID:
Score: 2.5
p-value: 0.02

Cs6g03520.1 gene=Cs6g03520 CDS=223-4413

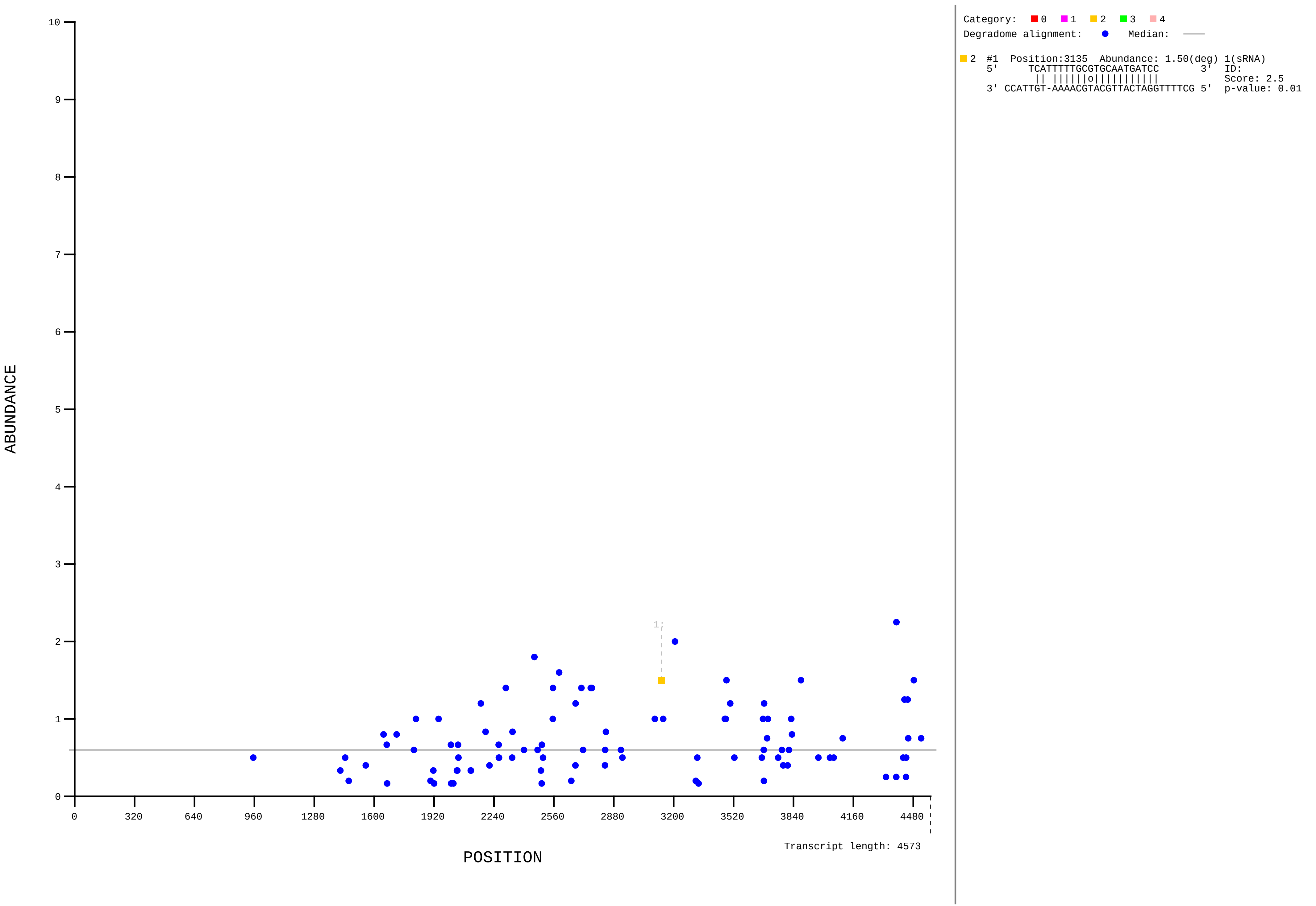

Cs3g18660.1 gene=Cs3g18660 CDS=275-2269

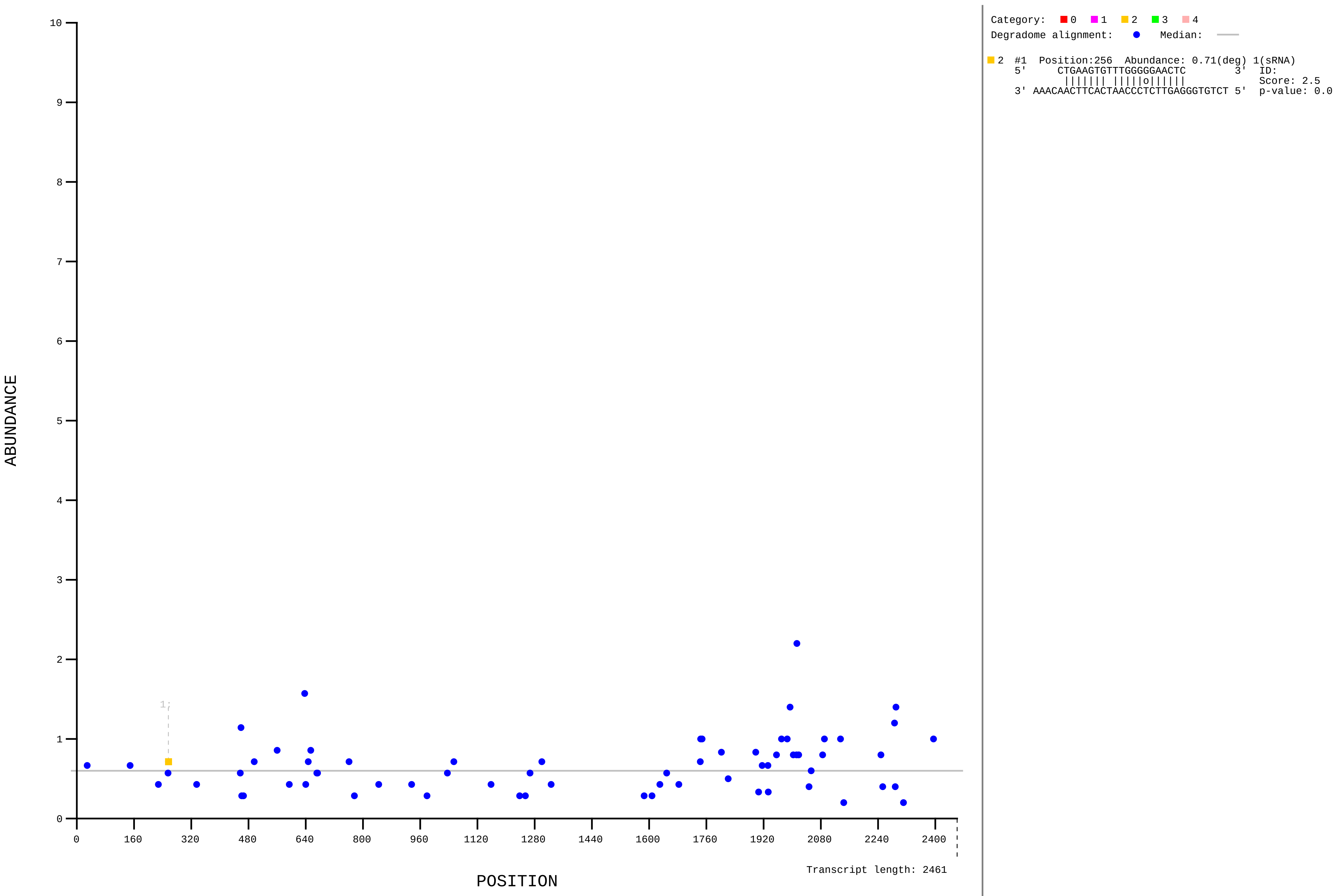

Cs3g18660.5 gene=Cs3g18660 CDS=286-2097

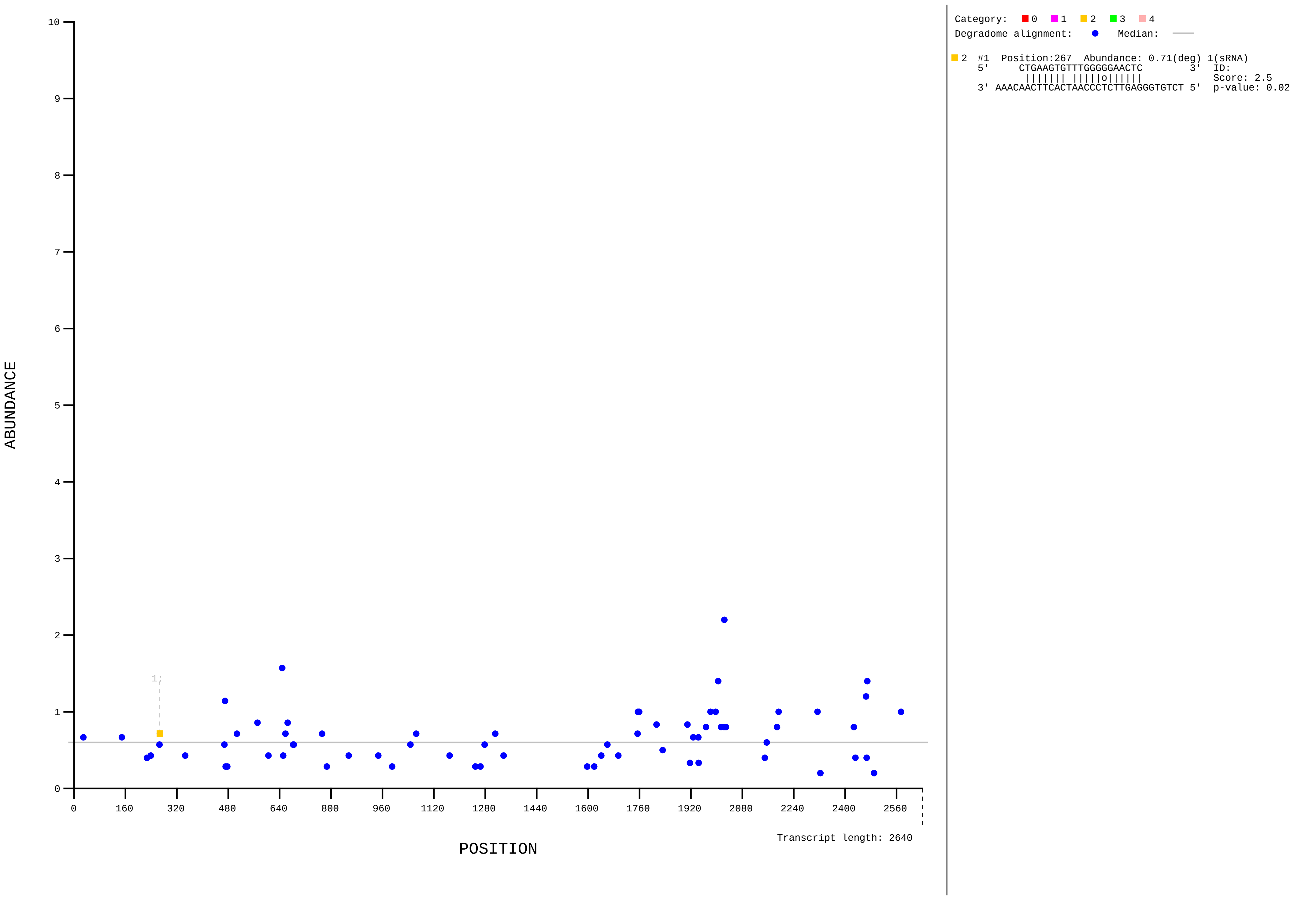

Cs3g18660.2 gene=Cs3g18660 CDS=286-2280

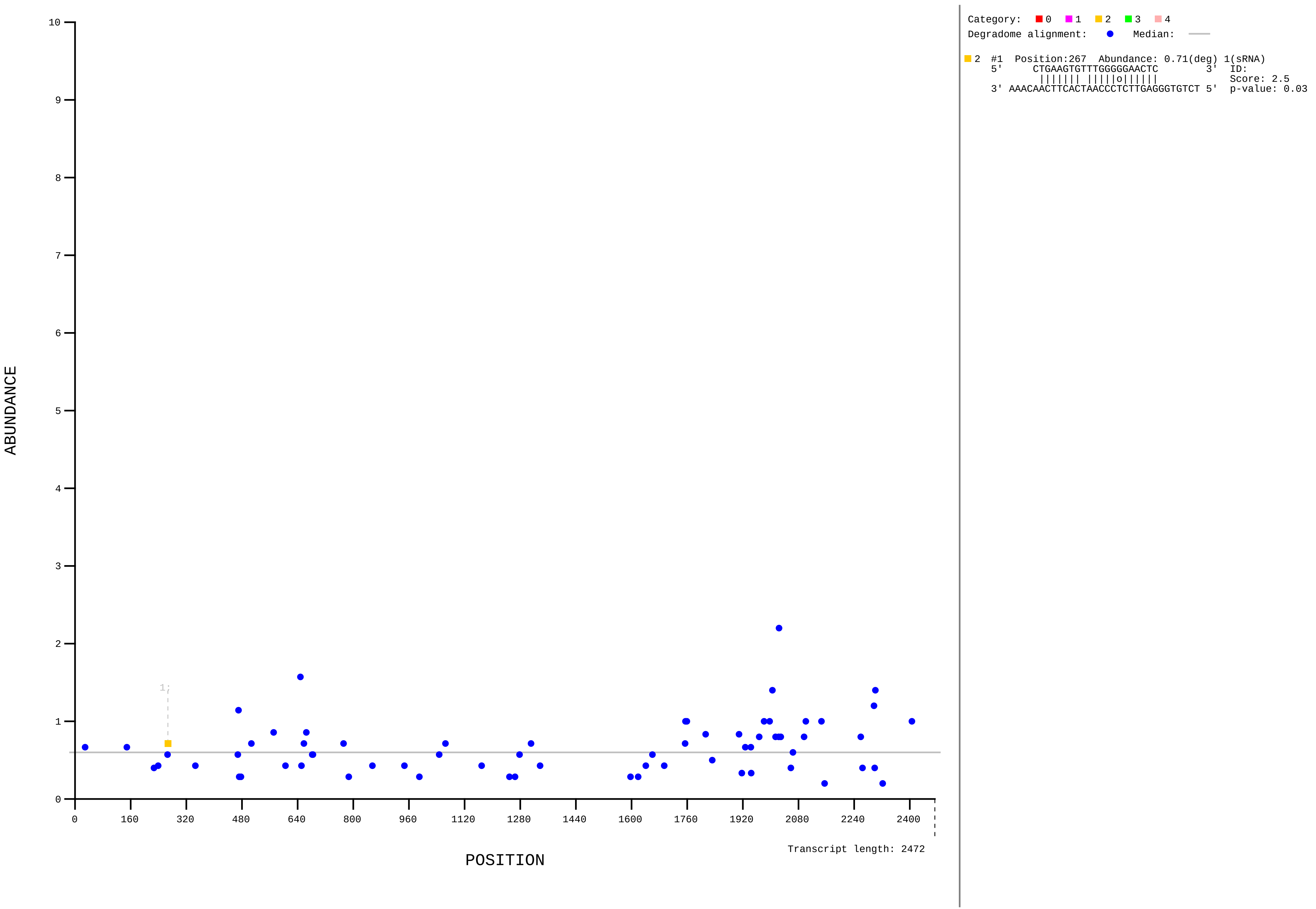

Cs3g18660.3 gene=Cs3g18660 CDS=286-2097

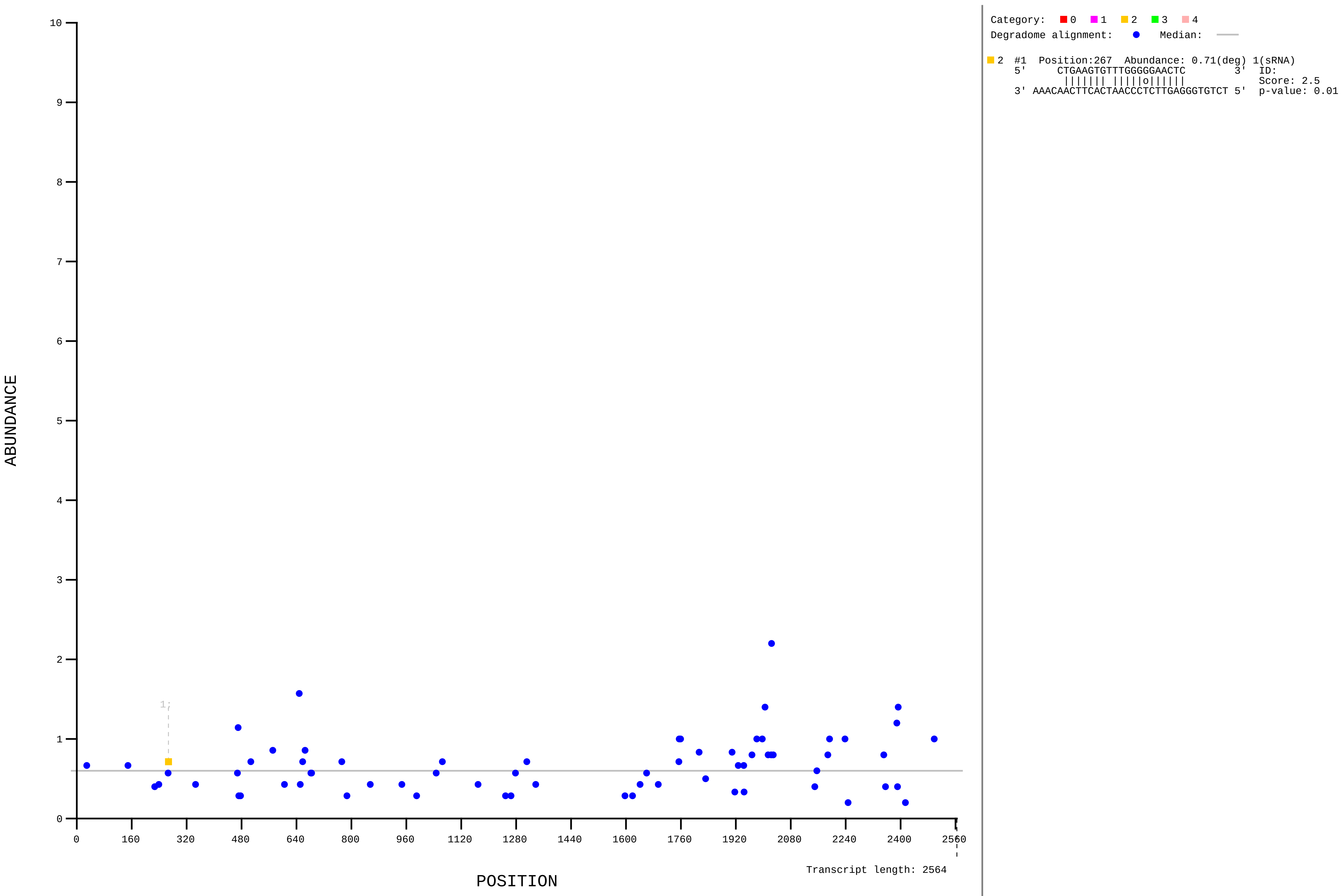

Cs6g03520.2 gene=Cs6g03520 CDS=223-3402

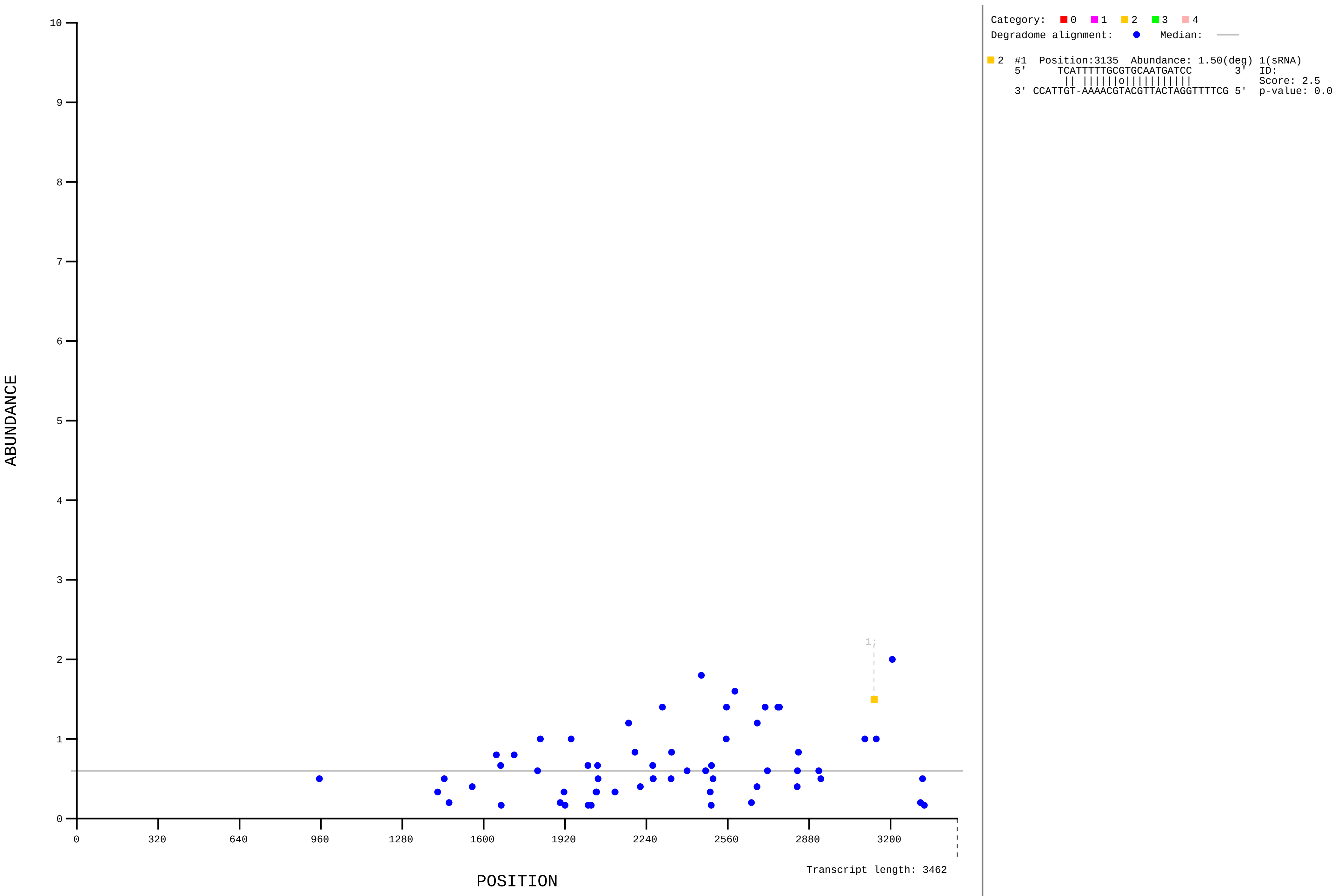

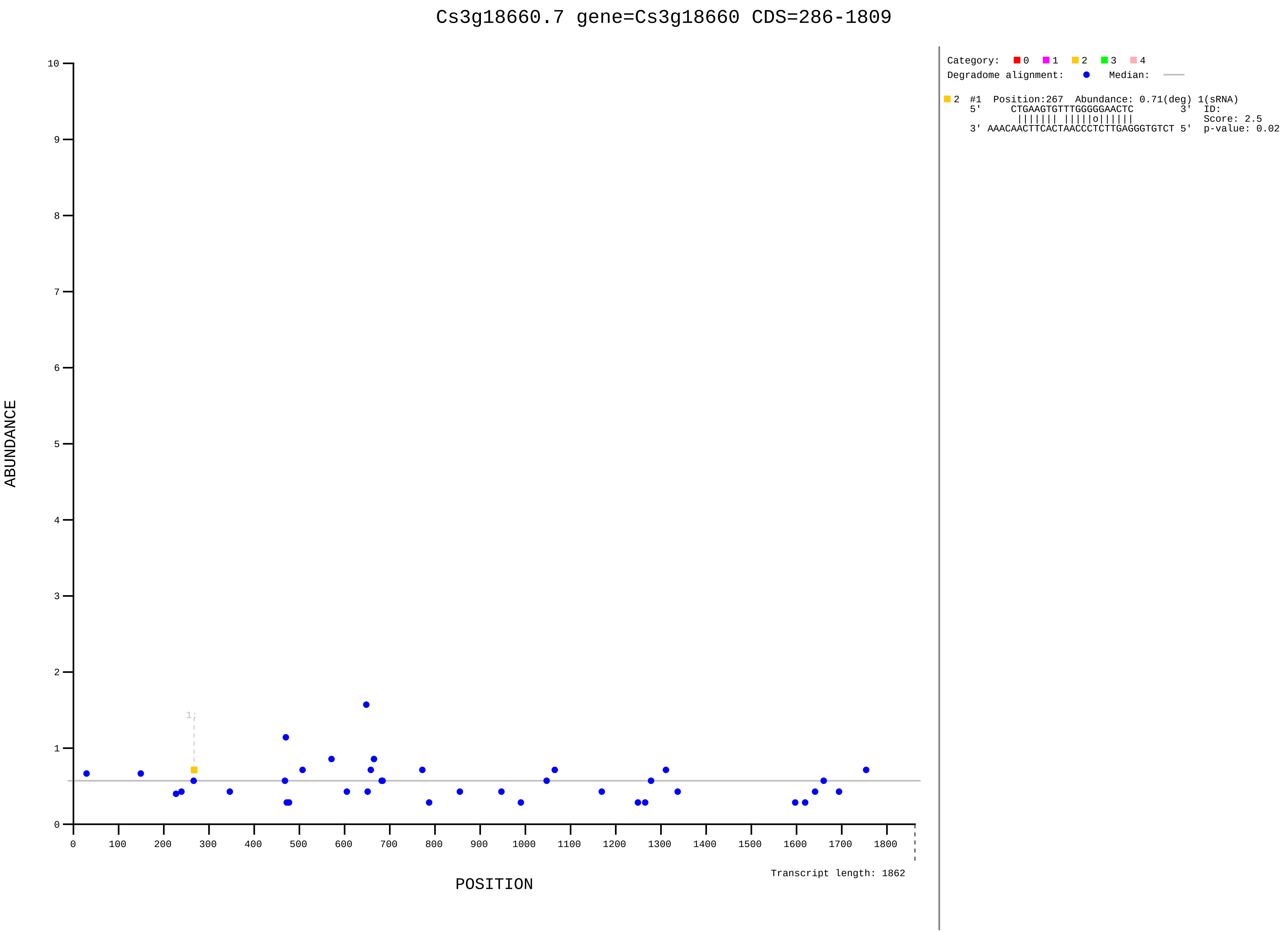

Cs3g18660.7 gene=Cs3g18660 CDS=286-1809
ABUNDANCE
POSITION
Transcript length: 1862
Category: 0 1 2 3 4
Degradome alignment:
Median:
2 #1 Position:267 Abundance: 0.71(deg) 1(sRNA)
5' CTGAAGTGTTTGGGGGAACTC 3' ID:
||||||| |||||o|||||| Score: 2.5
3' AAACAACTTCACTAACCCTCTTGAGGGTGTCT 5' p-value: 0.02

Cs3g18660.6 gene=Cs3g18660 CDS=286-1968

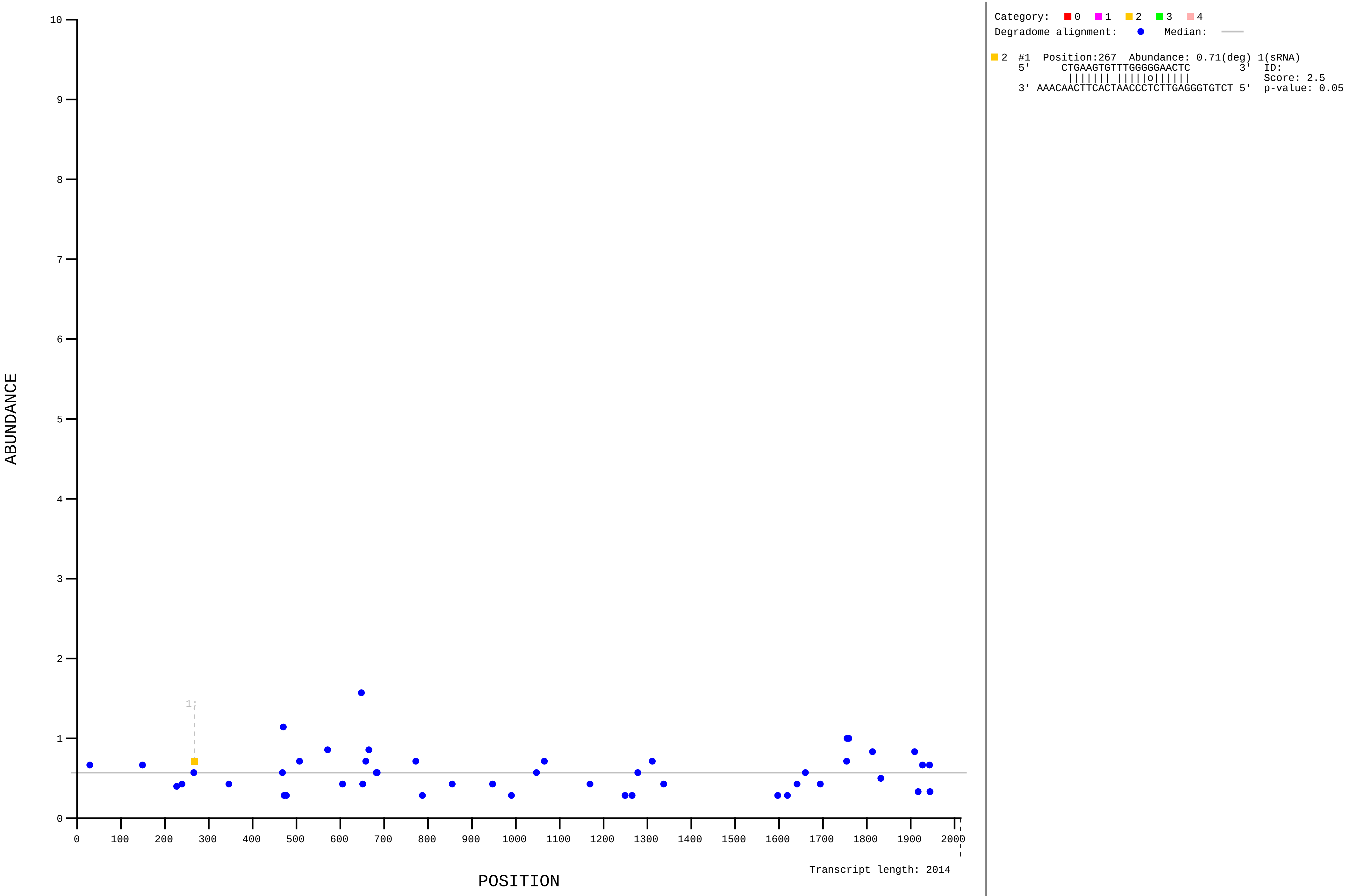

orange1.1t00796.1 gene=orange1.1t00796 CDS=1-648

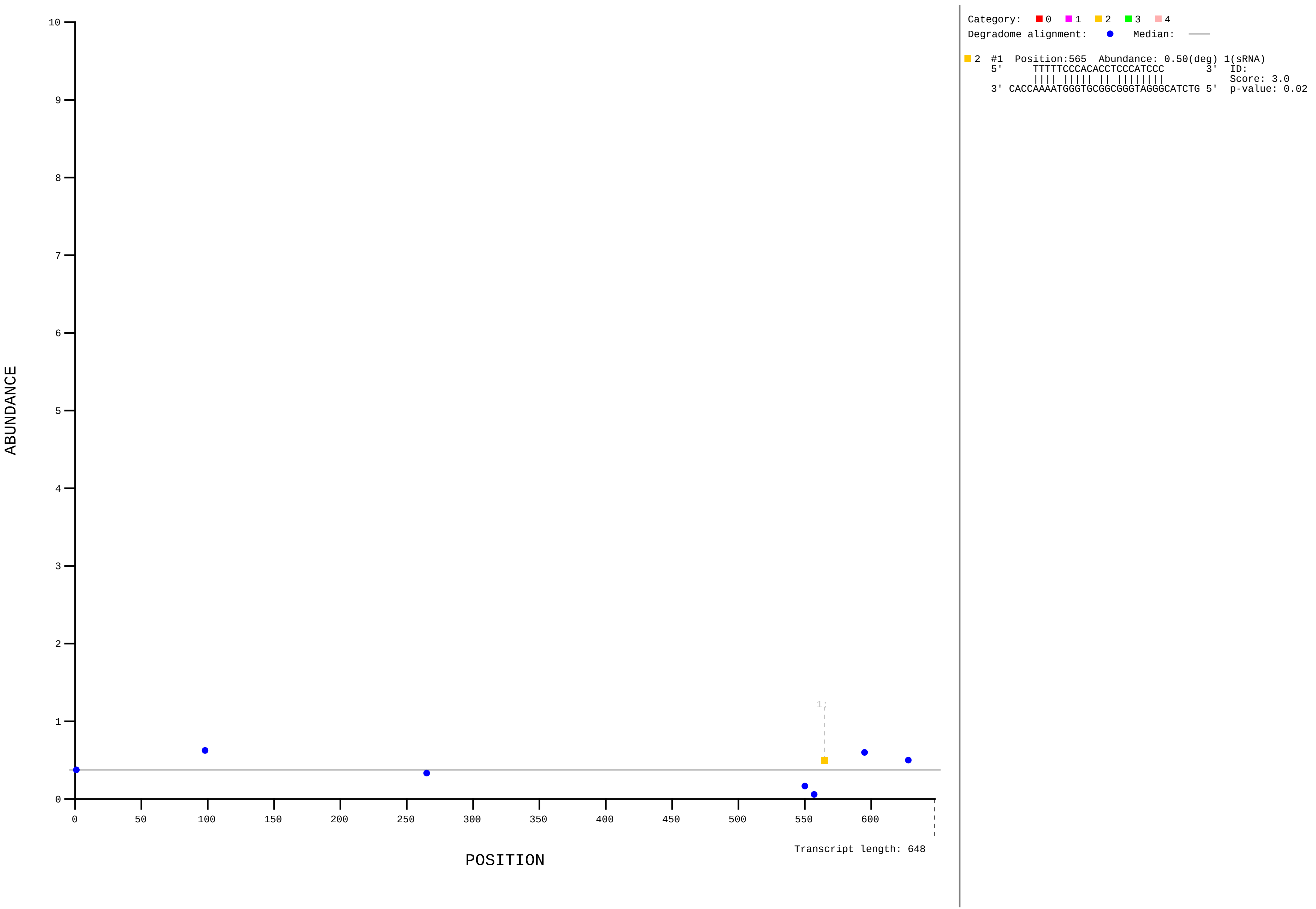

Cs9g17130.1 gene=Cs9g17130 CDS=187-906

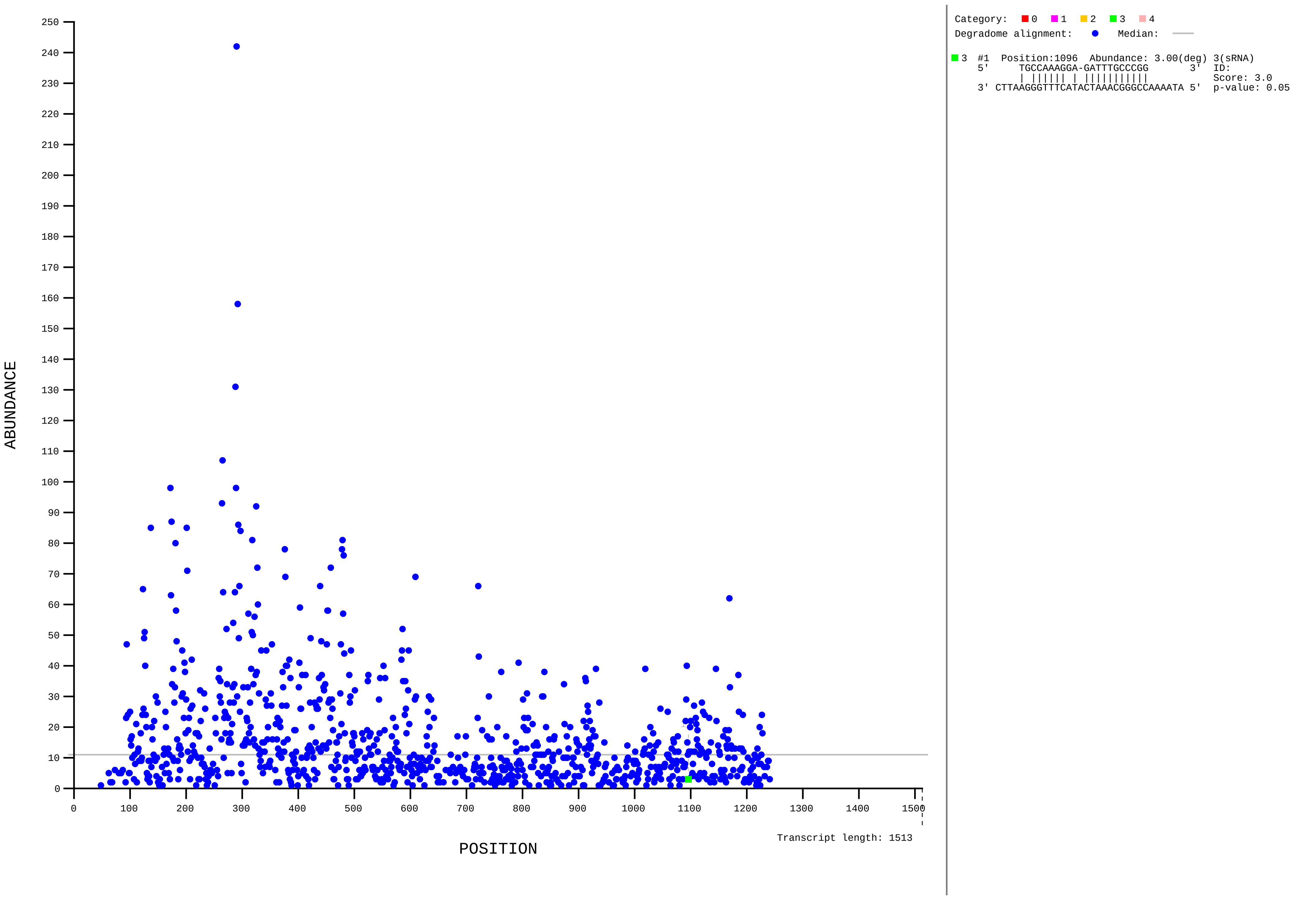

Cs4g12990.1 gene=Cs4g12990 CDS=811-2082

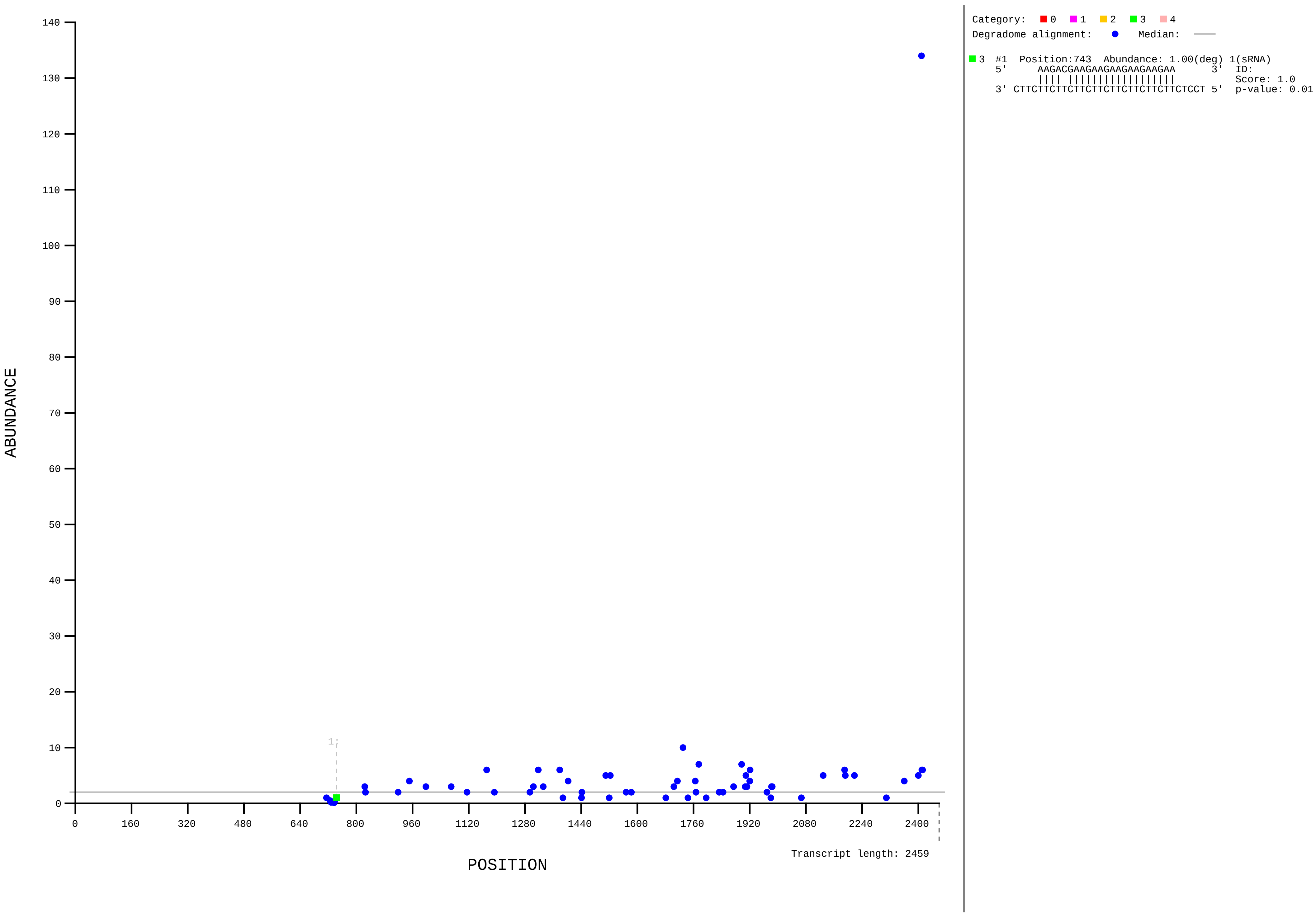

orange1.1t03712.1 gene=orange1.1t03712 CDS=5-4630

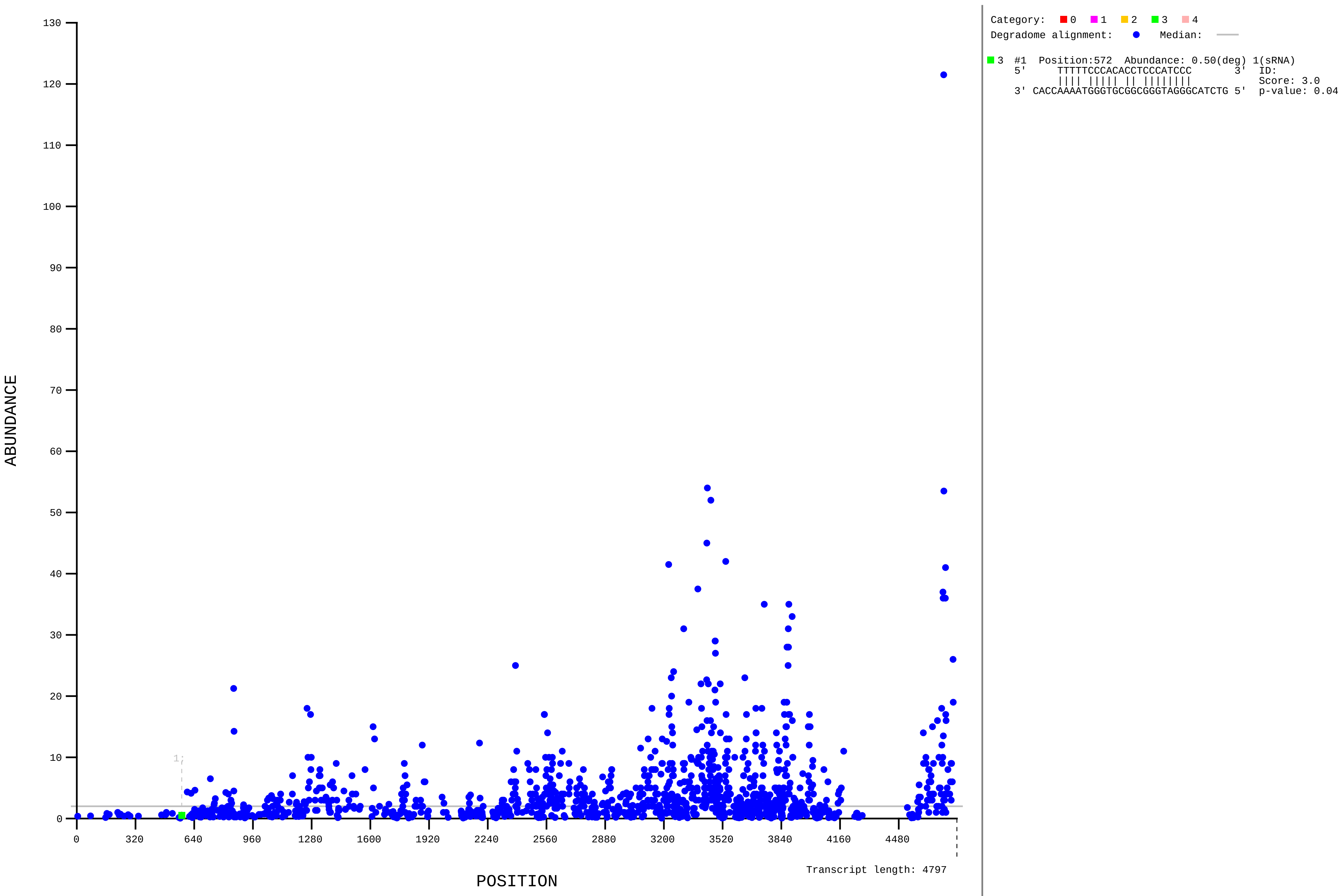

Cs4g08600.2 gene=Cs4g08600 CDS=356-11686

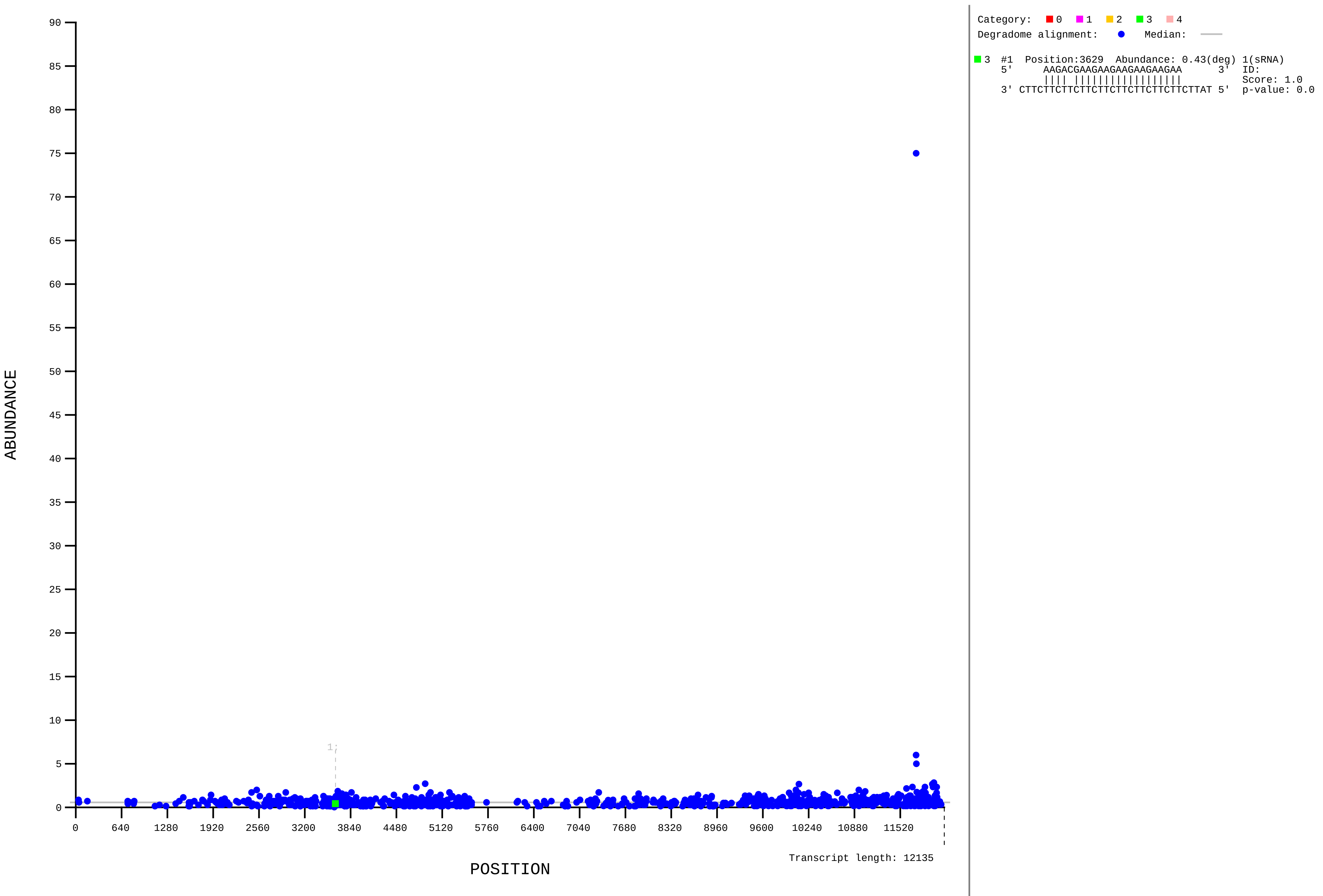

Cs2g10720.1 gene=Cs2g10720 CDS=1-1062

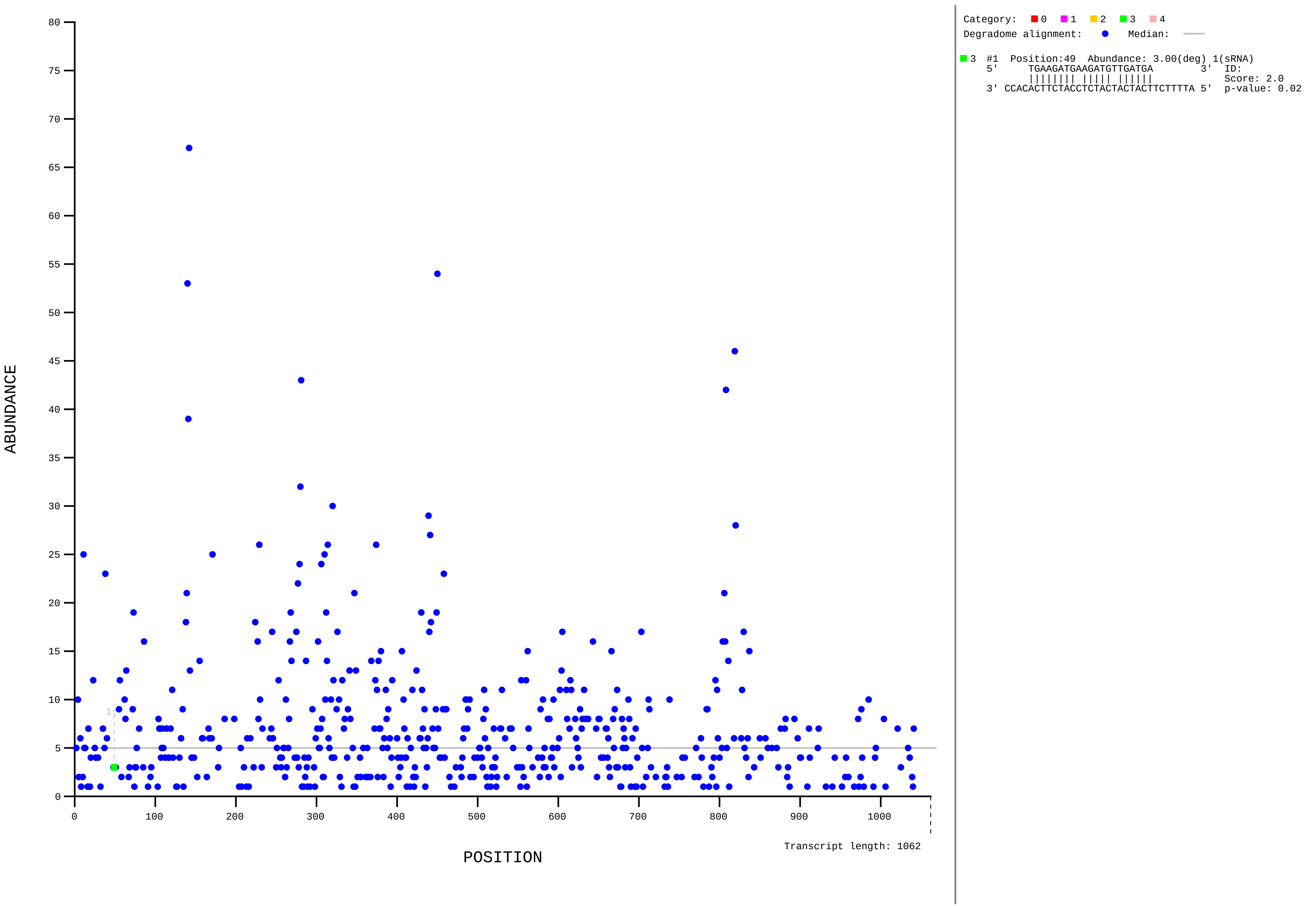

Cs8g17370.4 gene=Cs8g17370 CDS=423-1175

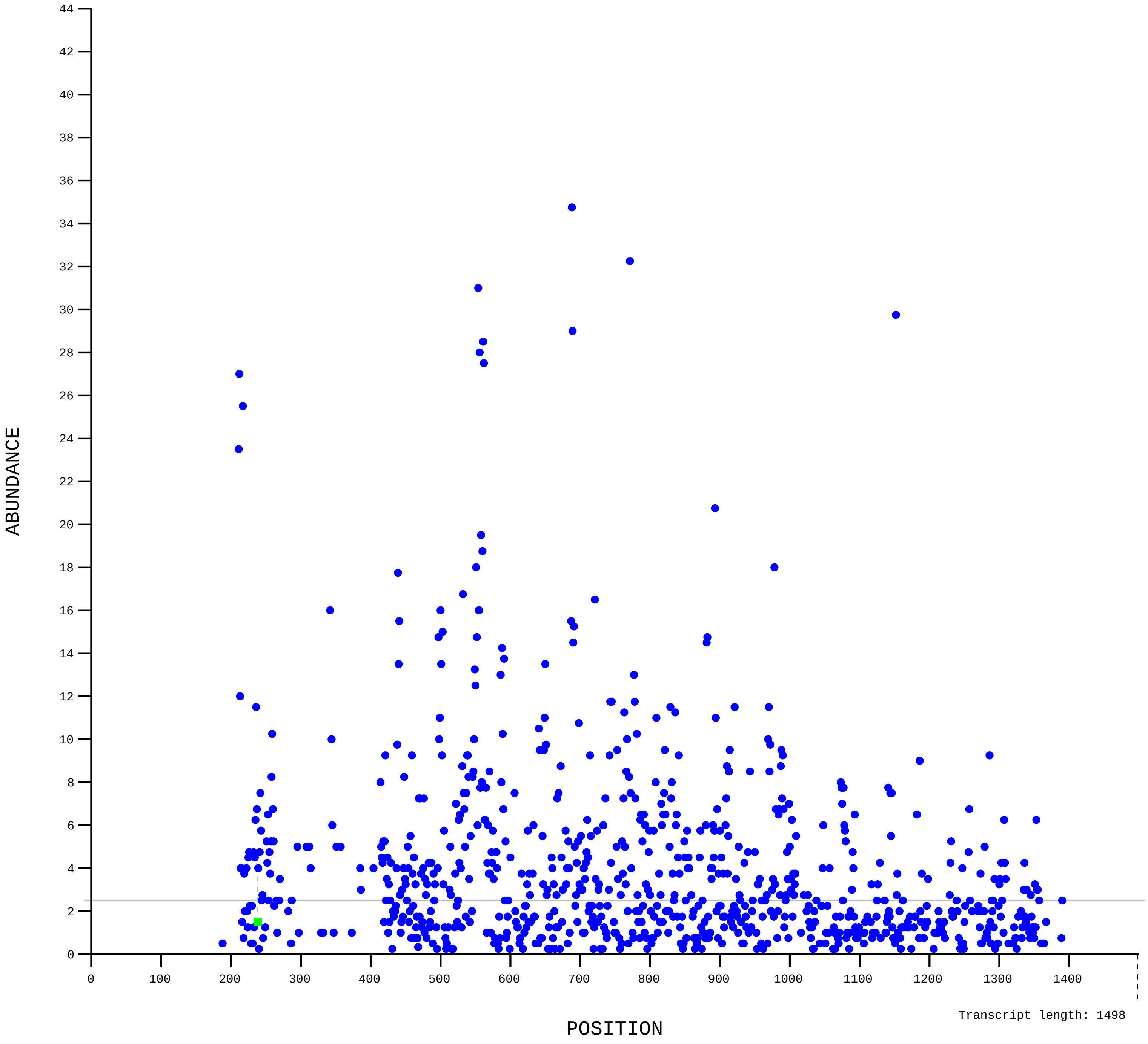


```
Category:  ■0  ■1  ■2  ■3  ■4
Degradome alignment: ●   Median: ——

■3  #1  Position:238  Abundance: 1.50(deg) 1(sRNA)
    5'     TTGACAGAAGAGAGTGAGCAC        3'  ID:
           ||o|||o| ||||||| ||||            Score: 3.0
    3' TAGGAATTGTTTGCTCTCAC-CGTGCTCGTCT 5'  p-value: 0.05
```

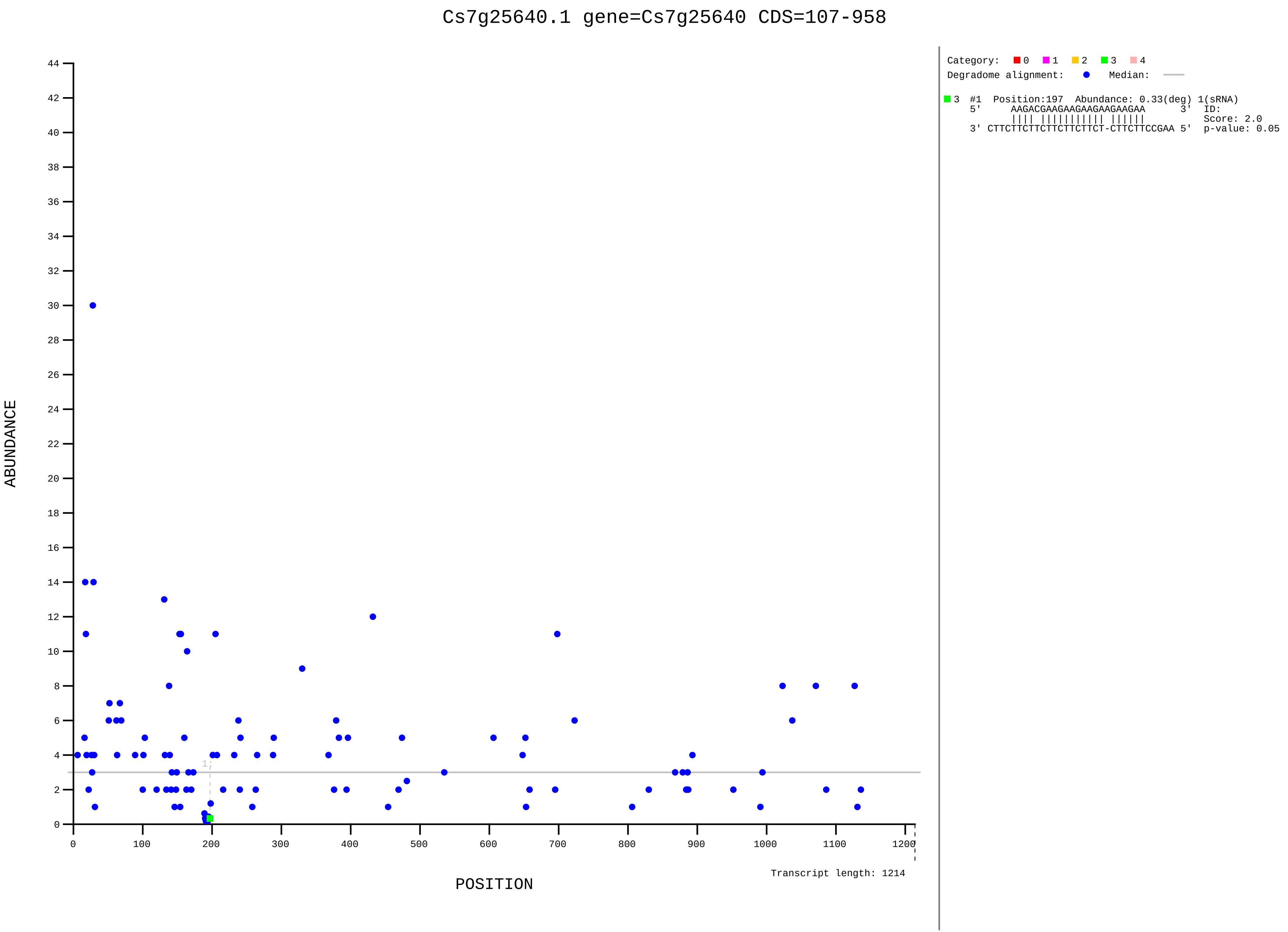

Cs7g25640.1 gene=Cs7g25640 CDS=107-958
Category: 0 1 2 3 4
Degradome alignment:
Median:
3 #1 Position:197 Abundance: 0.33(deg) 1(sRNA)
5' AAGACGAAGAAGAAGAAGAAGAA 3'
|||| ||||||||||| ||||||
3' CTTCTTCTTCTTCTTCTTCT-CTTCTTCCGAA 5'
ID:
Score: 2.0
p-value: 0.05
ABUNDANCE
POSITION
Transcript length: 1214

Cs2g15740.1 gene=Cs2g15740 CDS=102-1277

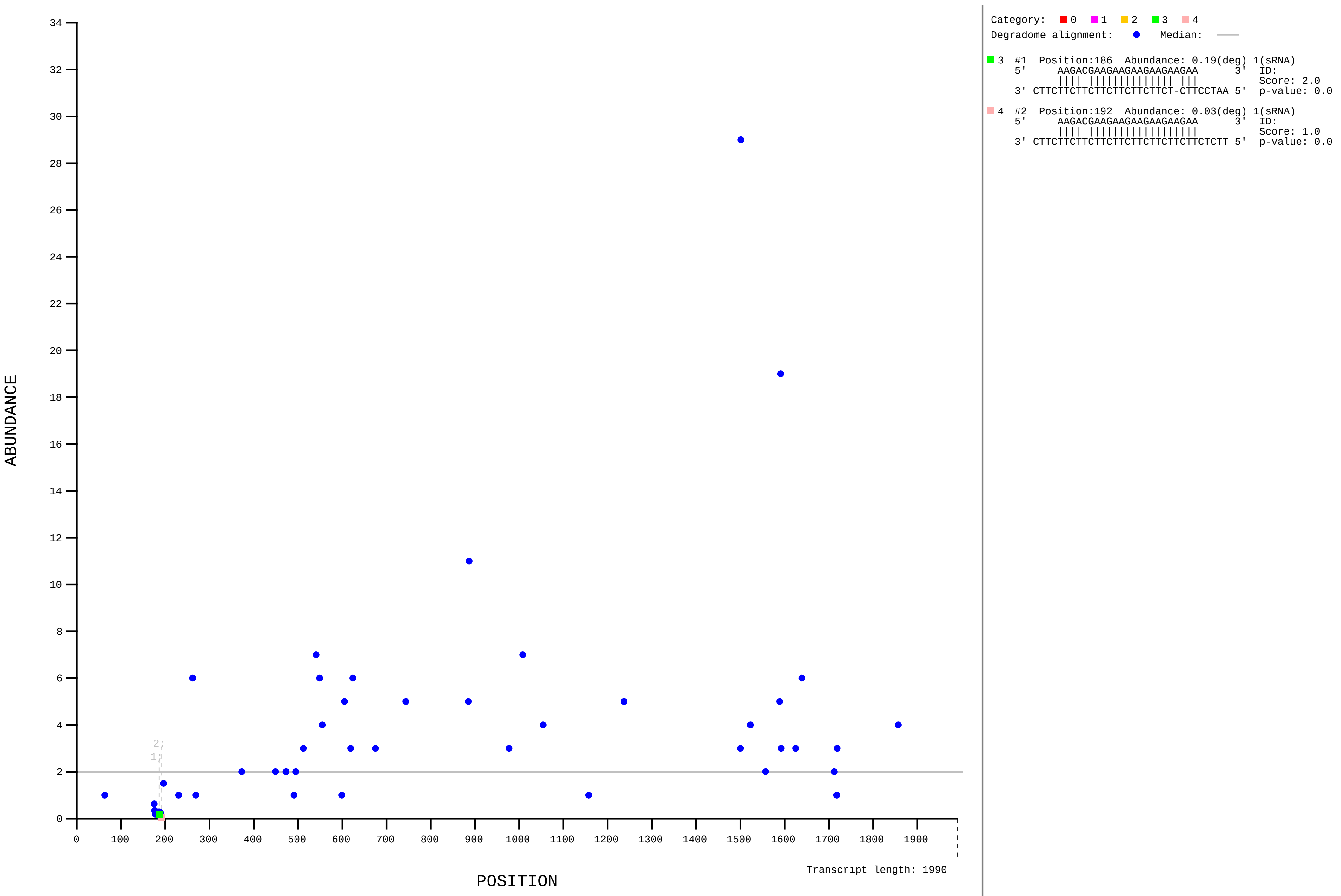

Cs8g20160.1 gene=Cs8g20160 CDS=185-661

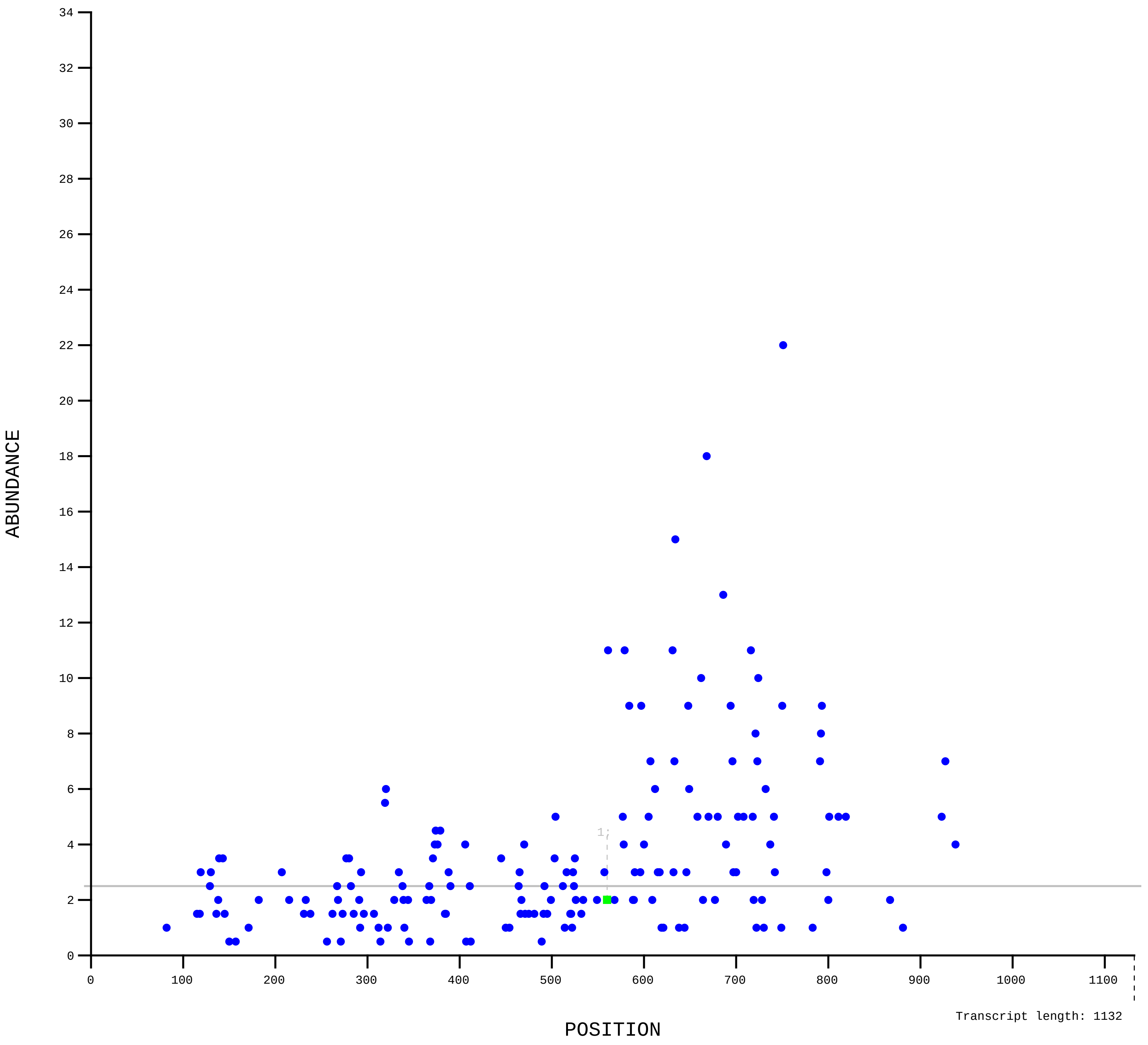

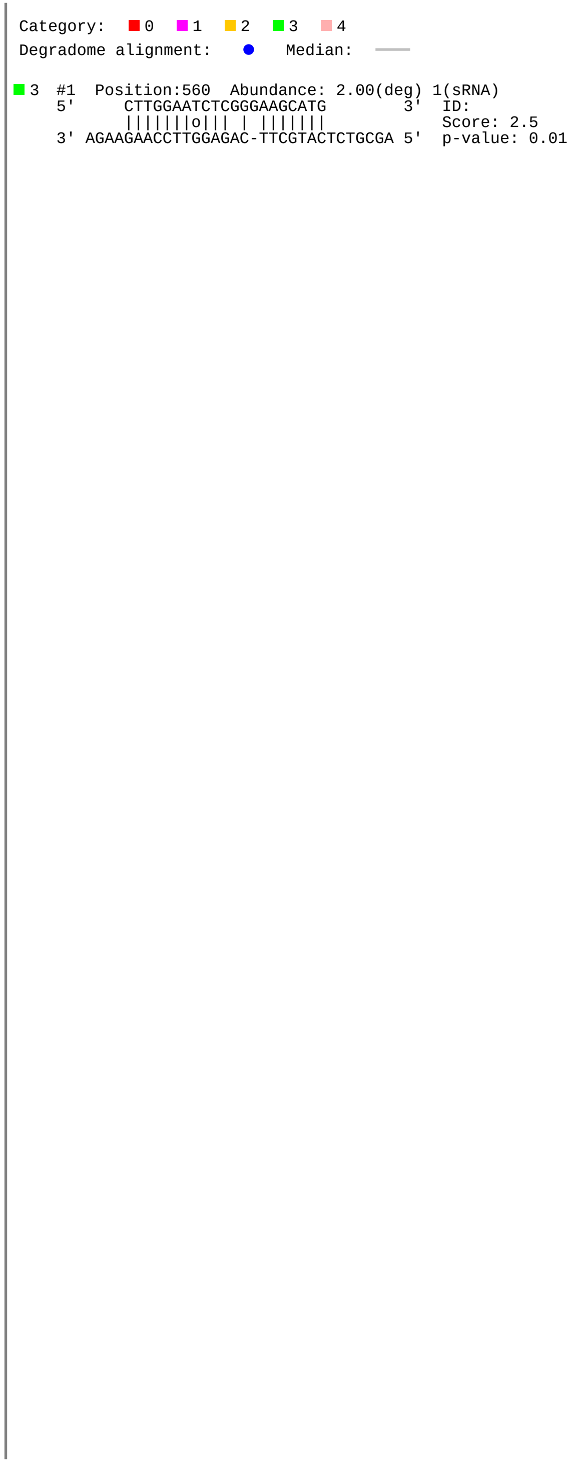

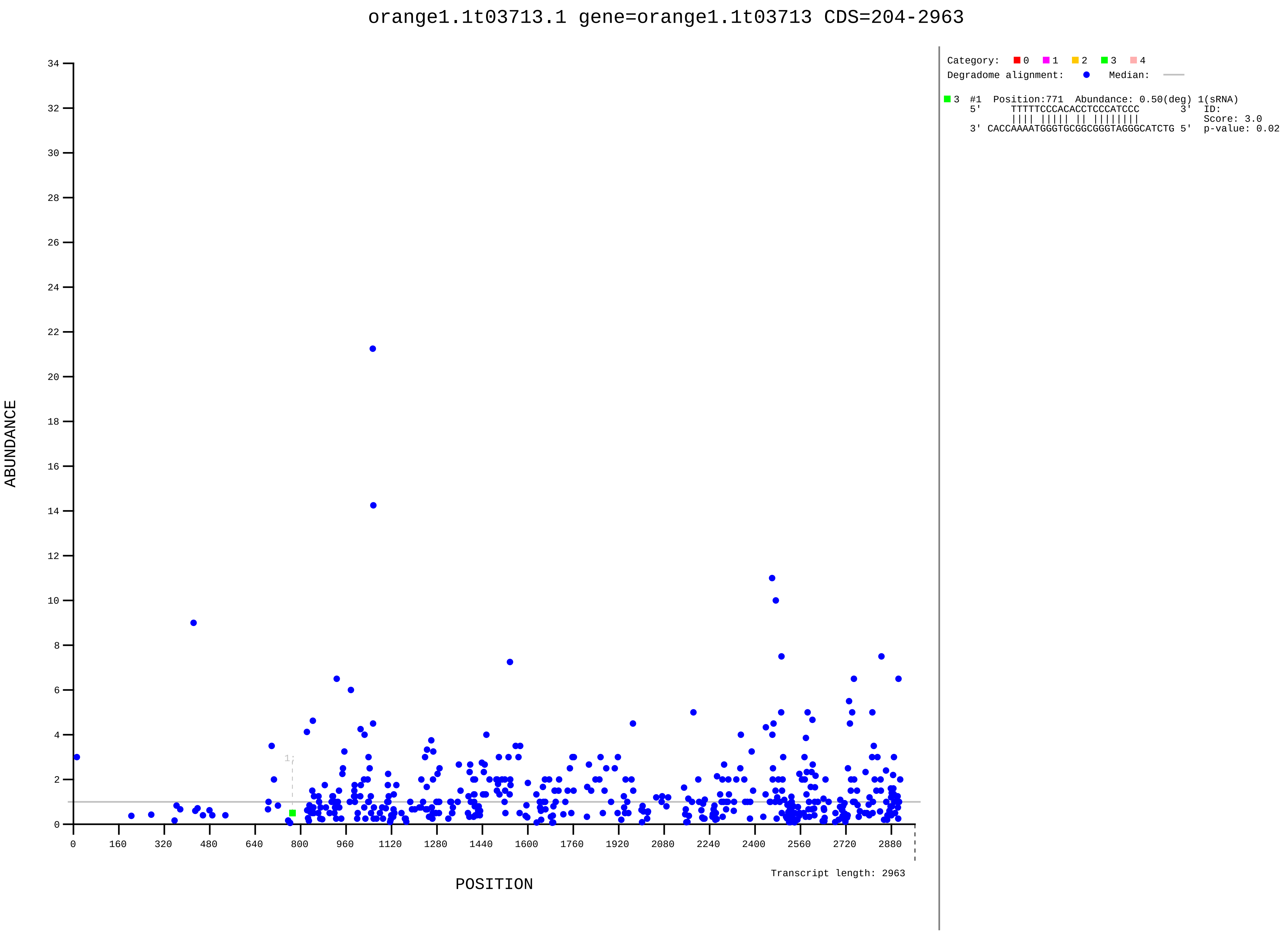
orange1.1t03713.1 gene=orange1.1t03713 CDS=204-2963
ABUNDANCE
POSITION
Transcript length: 2963
Category: 0 1 2 3 4
Degradome alignment:
Median:
3 #1 Position:771 Abundance: 0.50(deg) 1(sRNA)
5' TTTTTCCCACACCTCCCATCCC 3' ID:
|||| ||||| || ||||||||
3' CACCAAAATGGGTGCGGCGGGTAGGGCATCTG 5' p-value: 0.02
Score: 3.0

Cs7g22590.1 gene=Cs7g22590 CDS=30-1220

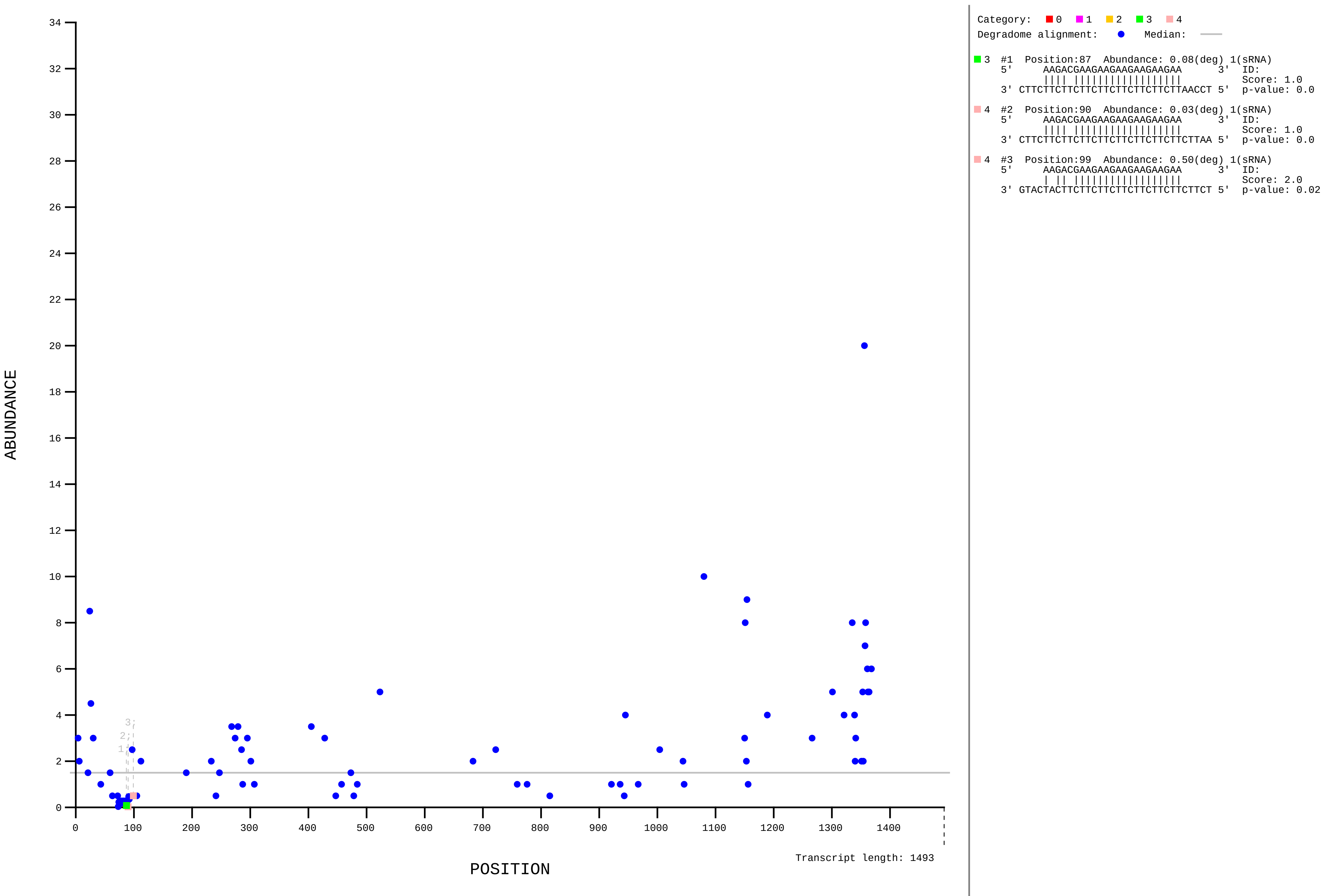

Cs2g04800.1 gene=Cs2g04800 CDS=157-684

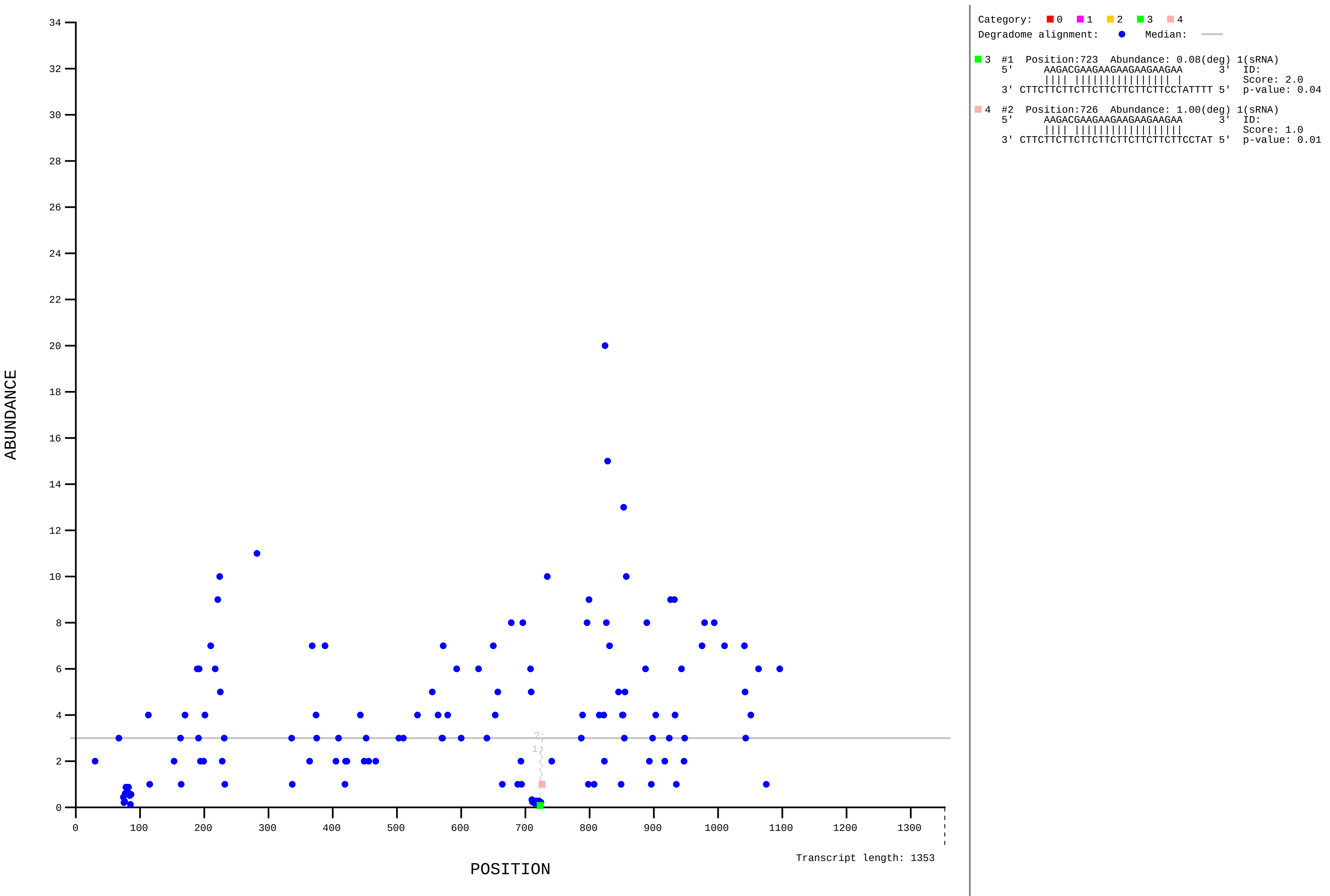

Cs8g14770.2 gene=Cs8g14770 CDS=814-2223

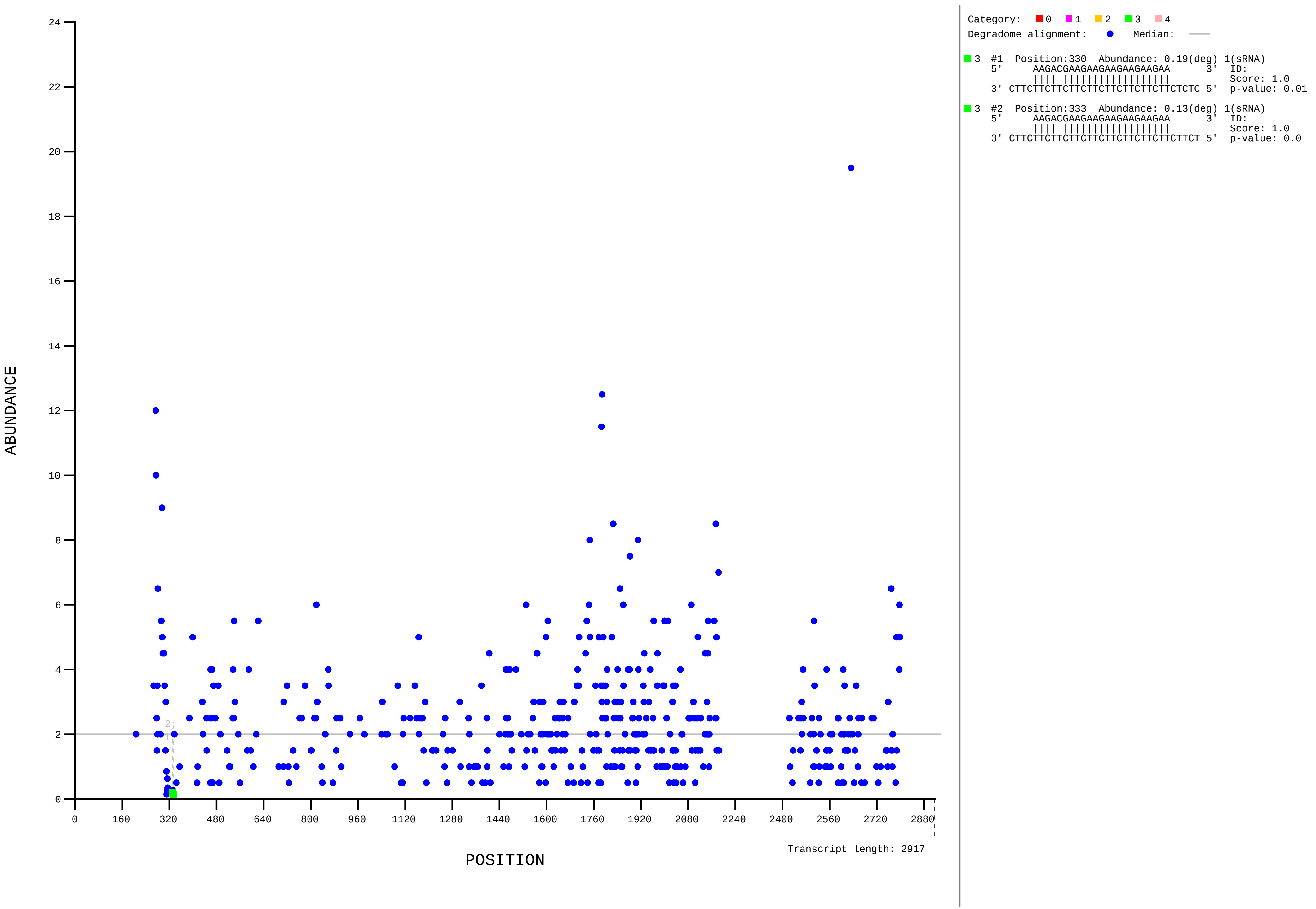

Cs8g14770.1 gene=Cs8g14770 CDS=814-2421

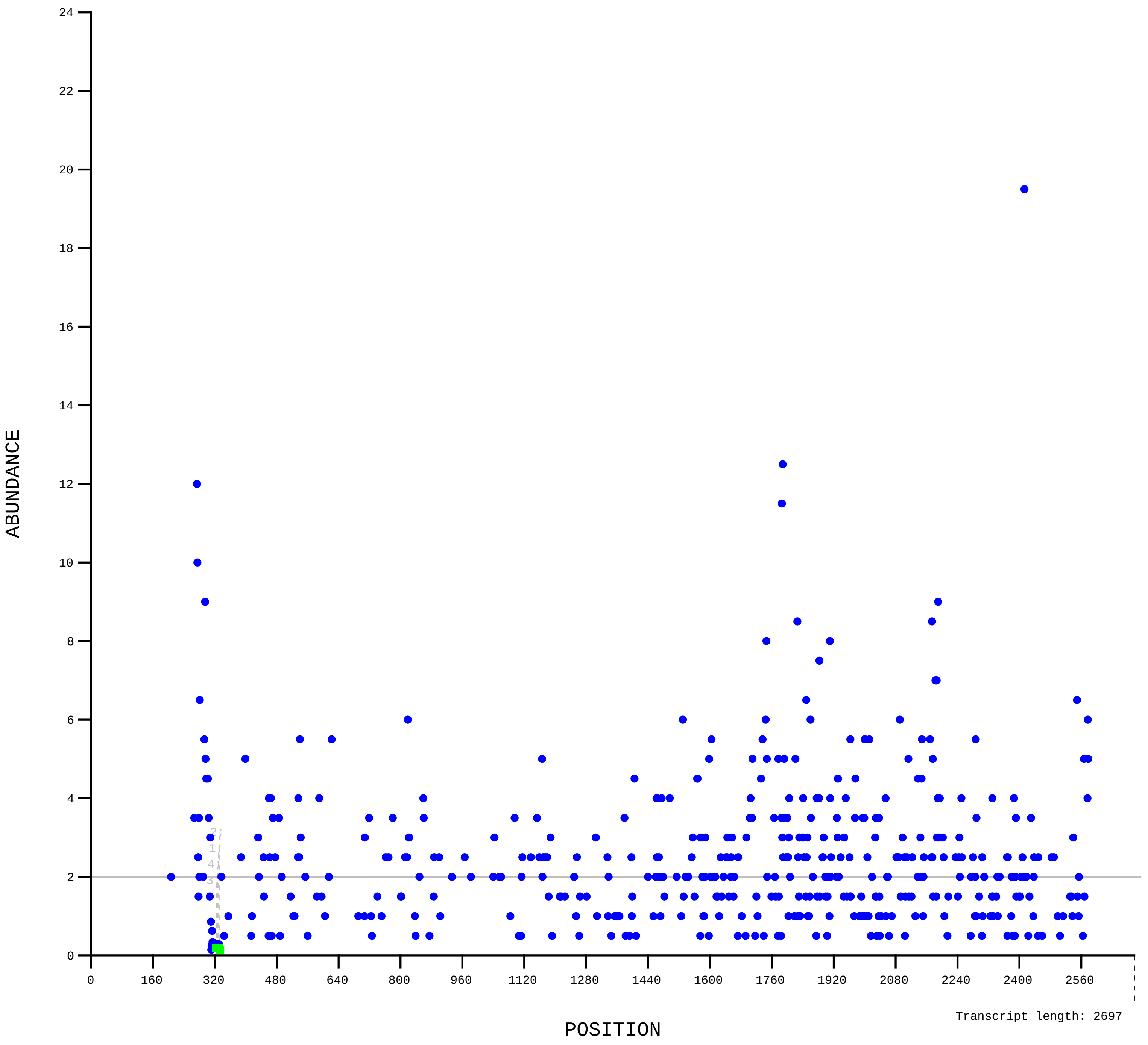

Category: 0 1 2 3 4
Degradome alignment: ● Median: —

3 #1 Position:330 Abundance: 0.19(deg) 1(sRNA)
```
5'     AAGACGAAGAAGAAGAAGAAGAA      3'  ID:
       |||| ||||||||||||||||||          Score: 1.0
3' CTTCTTCTTCTTCTTCTTCTTCTTCTTCTCTC 5'  p-value: 0.01
```

3 #2 Position:333 Abundance: 0.13(deg) 1(sRNA)
```
5'     AAGACGAAGAAGAAGAAGAAGAA      3'  ID:
       |||| ||||||||||||||||||          Score: 1.0
3' CTTCTTCTTCTTCTTCTTCTTCTTCTTCTTCT 5'  p-value: 0.0
```

3 #3 Position:324 Abundance: 0.19(deg) 1(sRNA)
```
5'     AAGACGAAGAAGAAGAAGA-AGAA     3'  ID:
       |||| |||||||||||||| ||||         Score: 2.0
3' CTTCTTCTTCTTCTTCTTCTTCTCTCTTCTAT 5'  p-value: 0.05
```

3 #4 Position:327 Abundance: 0.19(deg) 1(sRNA)
```
5'     AAGACGAAGAAGAAGAAGAAGAA      3'  ID:
       |||| |||||||||||||||||           Score: 2.0
3' CTTCTTCTTCTTCTTCTTCTTCTTCTCTCTTC 5'  p-value: 0.03
```

Cs5g26190.1 gene=Cs5g26190 CDS=1-4206

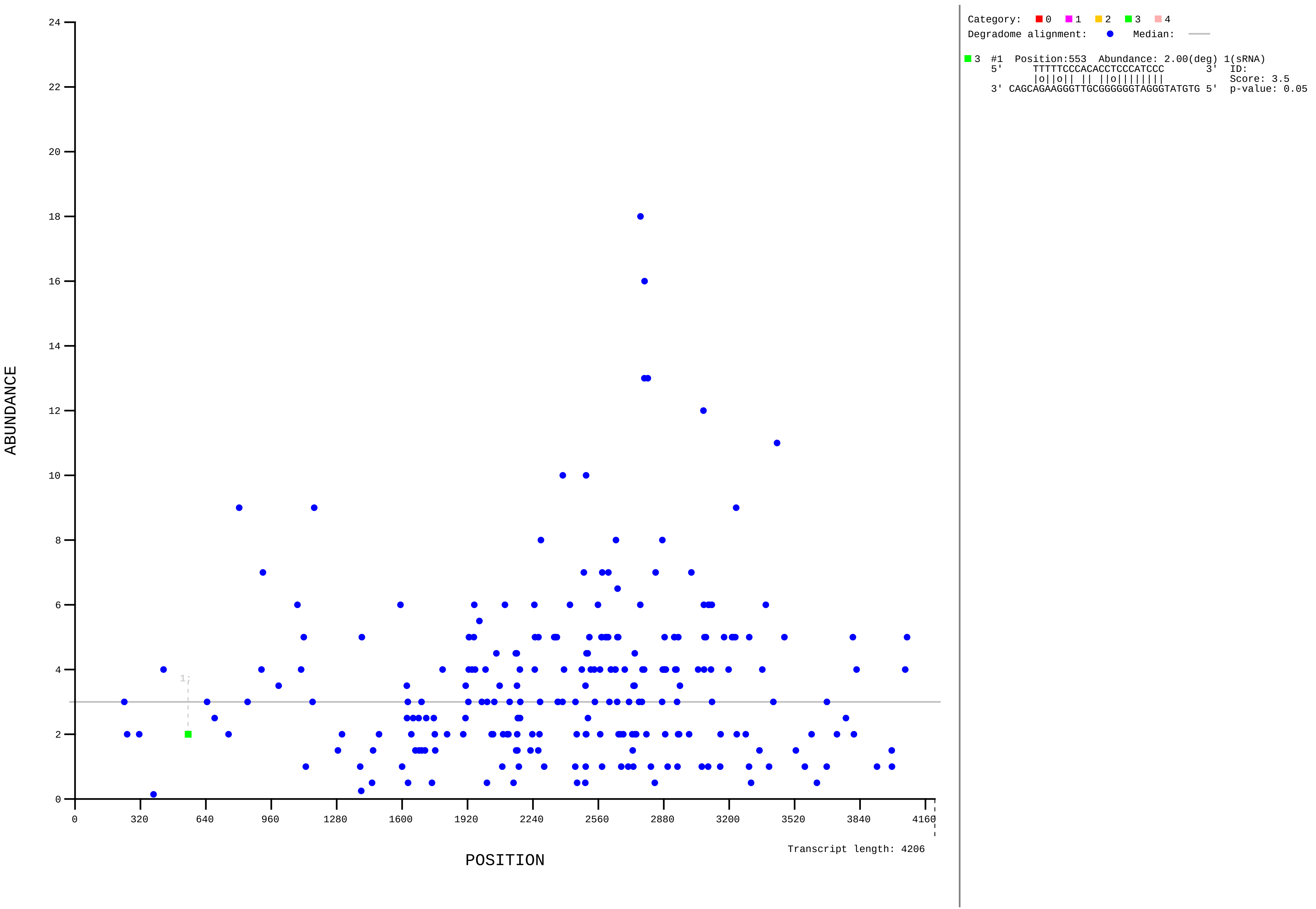

Cs4g10910.1 gene=Cs4g10910 CDS=57-7877

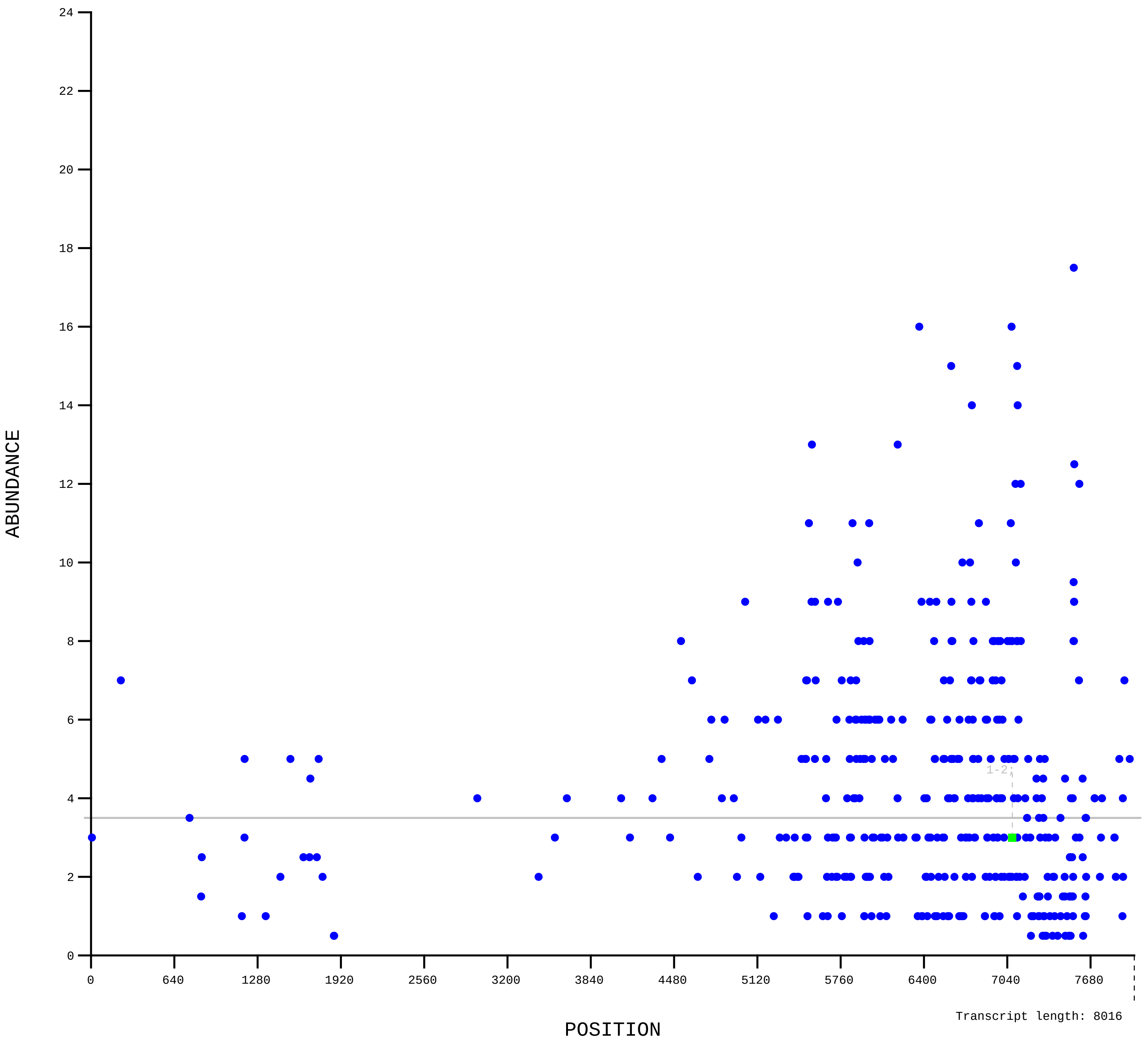

Category: 0 1 2 3 4
Degradome alignment: Median:

```
3  #1  Position:7079  Abundance: 3.00(deg) 1(sRNA)
   5'     TGCATTTGCACCTGCATCTTG        3'  ID:
          ||||||||||||| | ||||||           Score: 2.0
   3' CGAGACGTAAACGTGGTC-TAGAACGTCATAA 5'  p-value: 0.01

3  #2  Position:7079  Abundance: 3.00(deg) 1(sRNA)
   5'     TGCATTTGCACCTGCACCTTG        3'  ID:
          ||||||||||||| | | ||||           Score: 3.0
   3' CGAGACGTAAACGTGGTC-TAGAACGTCATAA 5'  p-value: 0.05
```

orange1.1t02876.1 gene=orange1.1t02876 CDS=1-918

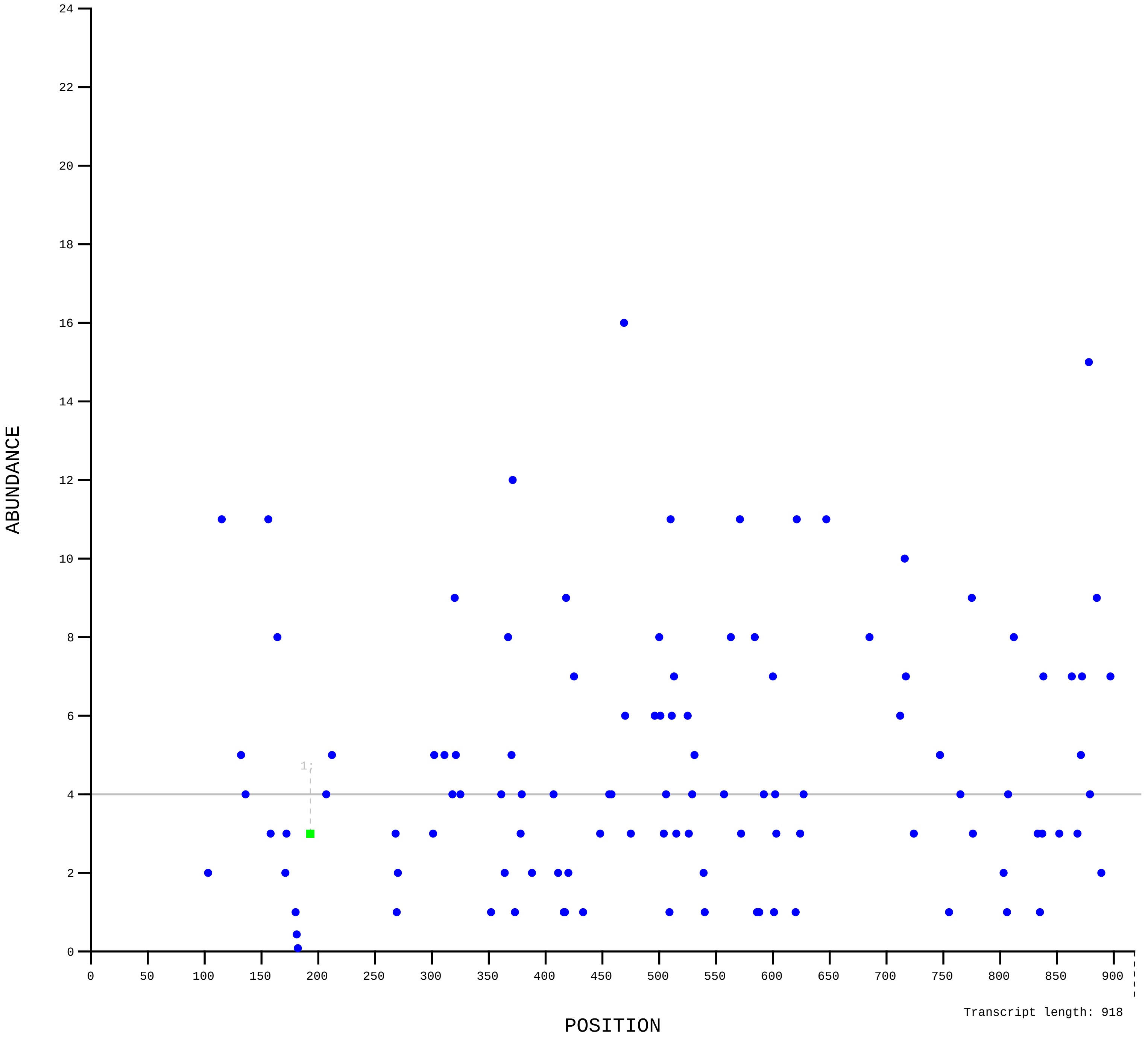

Category: 0 1 2 3 4
Degradome alignment: Median:

3 #1 Position:193 Abundance: 3.00(deg) 1(sRNA)
5' AAGACGAAGAAGAAGAAGAAGAA 3' ID:
|||| ||||||||||||||||| Score: 2.0
3' AAACTTCT-CTTCTTCTTCTTCTTCTATTTAG 5' p-value: 0.04

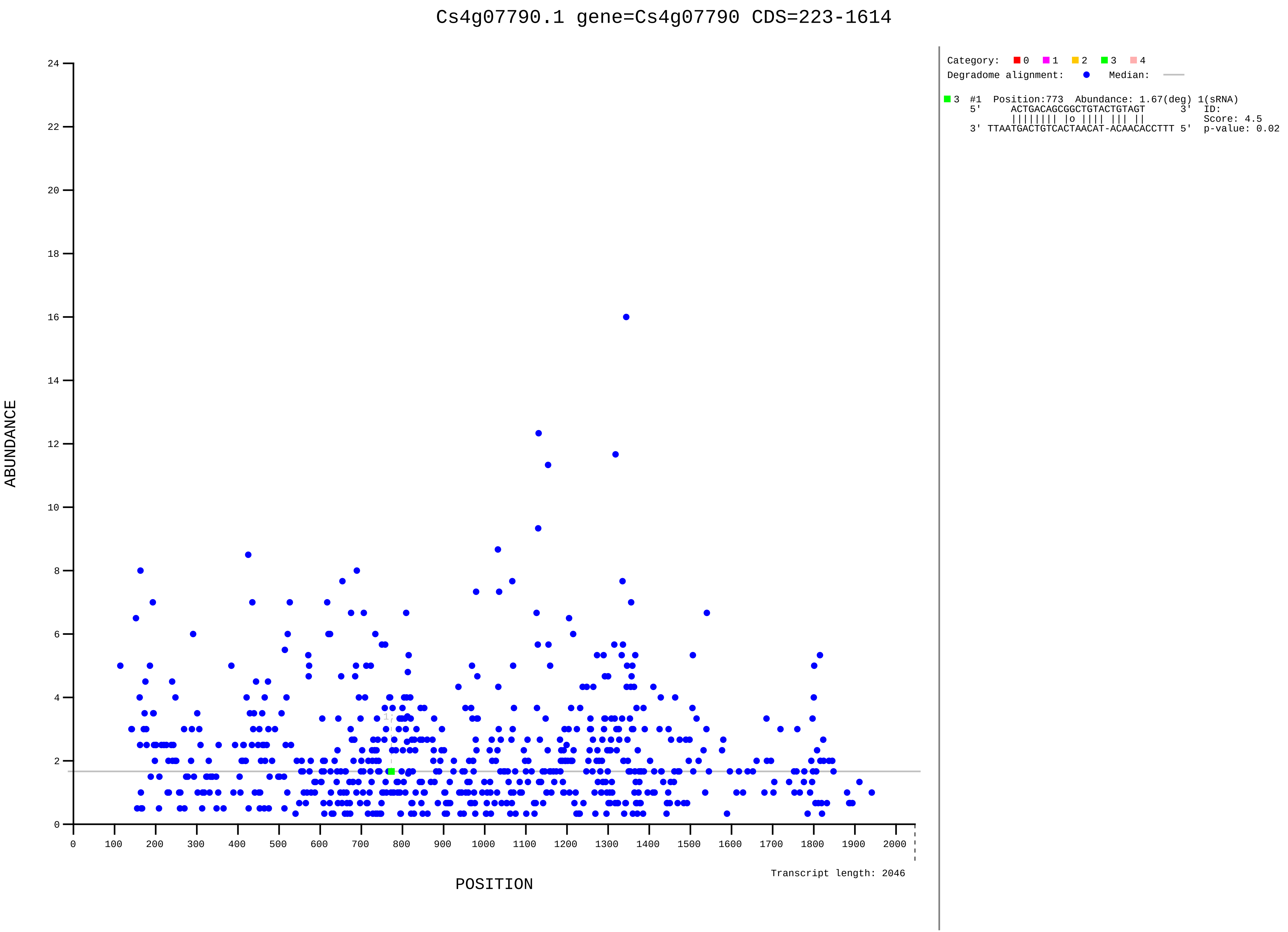

Cs4g07790.1 gene=Cs4g07790 CDS=223-1614
ABUNDANCE
POSITION
Transcript length: 2046
Category: 0 1 2 3 4
Degradome alignment:
Median:
3 #1 Position:773 Abundance: 1.67(deg) 1(sRNA)
5' ACTGACAGCGGCTGTACTGTAGT 3' ID:
||||||||| |o |||| ||| ||
3' TTAATGACTGTCACTAACAT-ACAACACCTTT 5'
Score: 4.5
p-value: 0.02

Cs6g03500.1 gene=Cs6g03500 CDS=289-4494

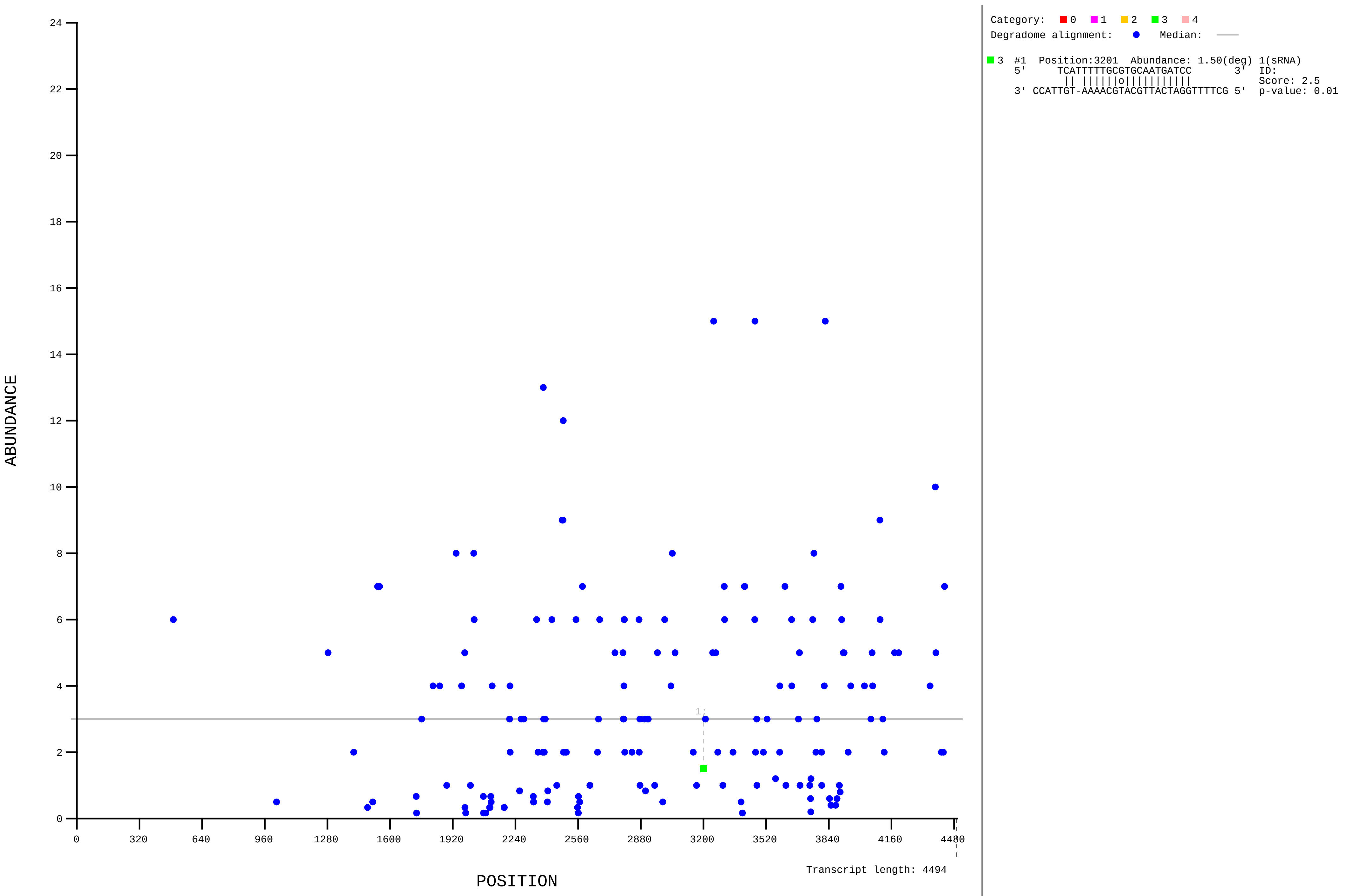

Cs1g19210.1 gene=Cs1g19210 CDS=506-2380

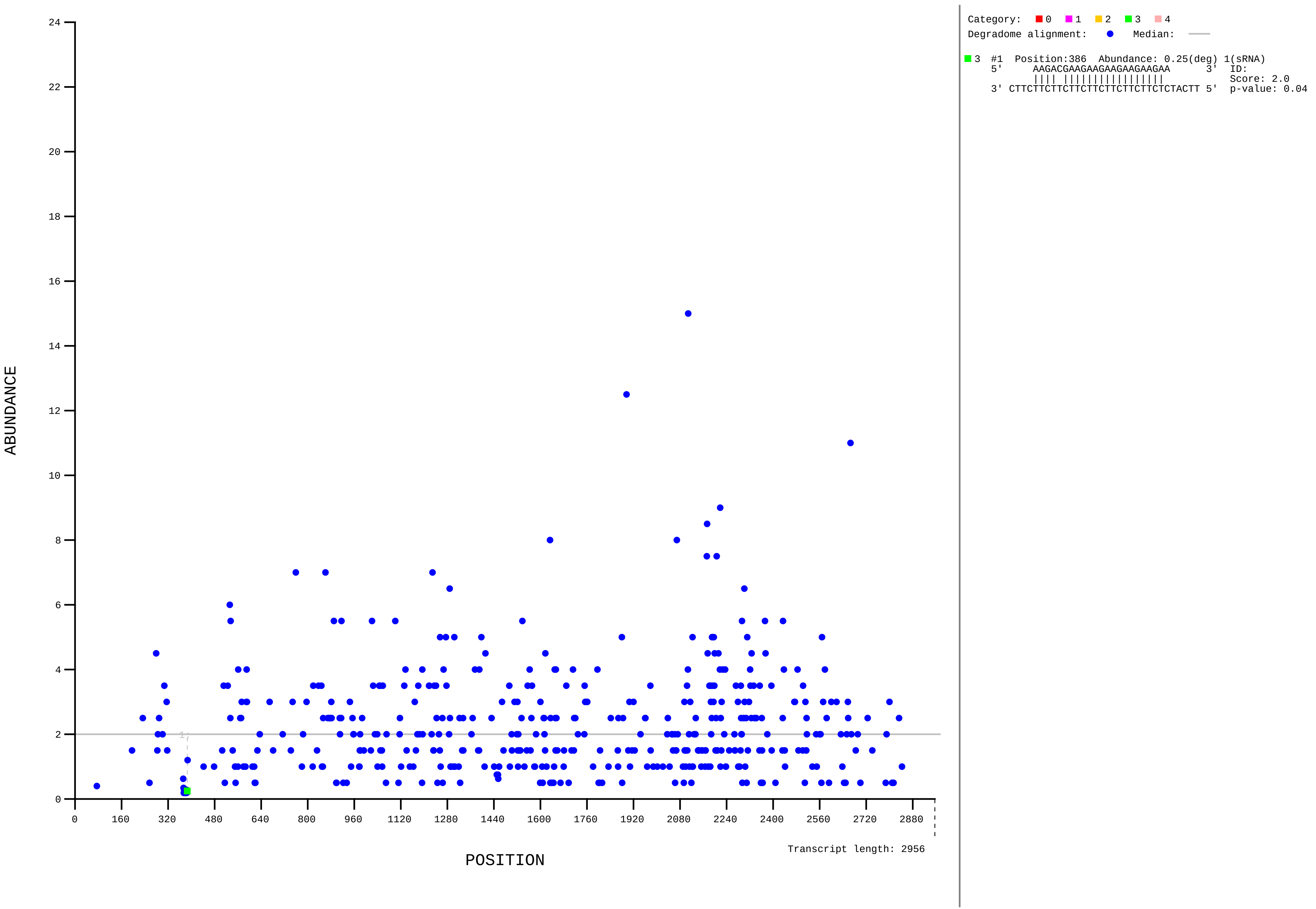

Cs1g19210.2 gene=Cs1g19210 CDS=1531-3063

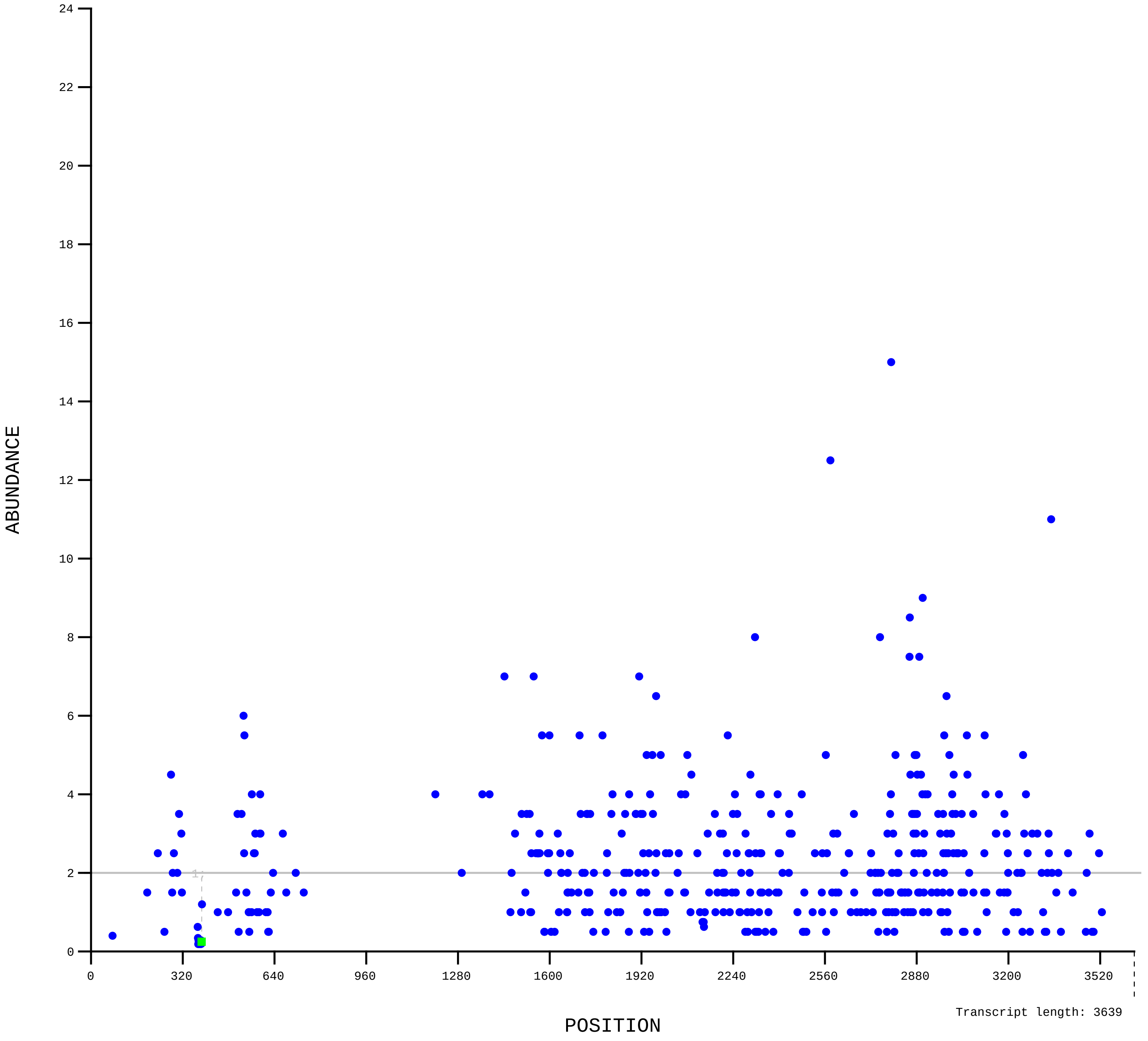

Category: 0 1 2 3 4
Degradome alignment: Median:

3 #1 Position:386 Abundance: 0.25(deg) 1(sRNA)

```
5'     AAGACGAAGAAGAAGAAGAAGAA      3'  ID:
         |||| ||||||||||||||||||          Score: 2.0
3' CTTCTTCTTCTTCTTCTTCTTCTTCTCTACTT 5'  p-value: 0.01
```

Cs6g15060.1 gene=Cs6g15060 CDS=429-932

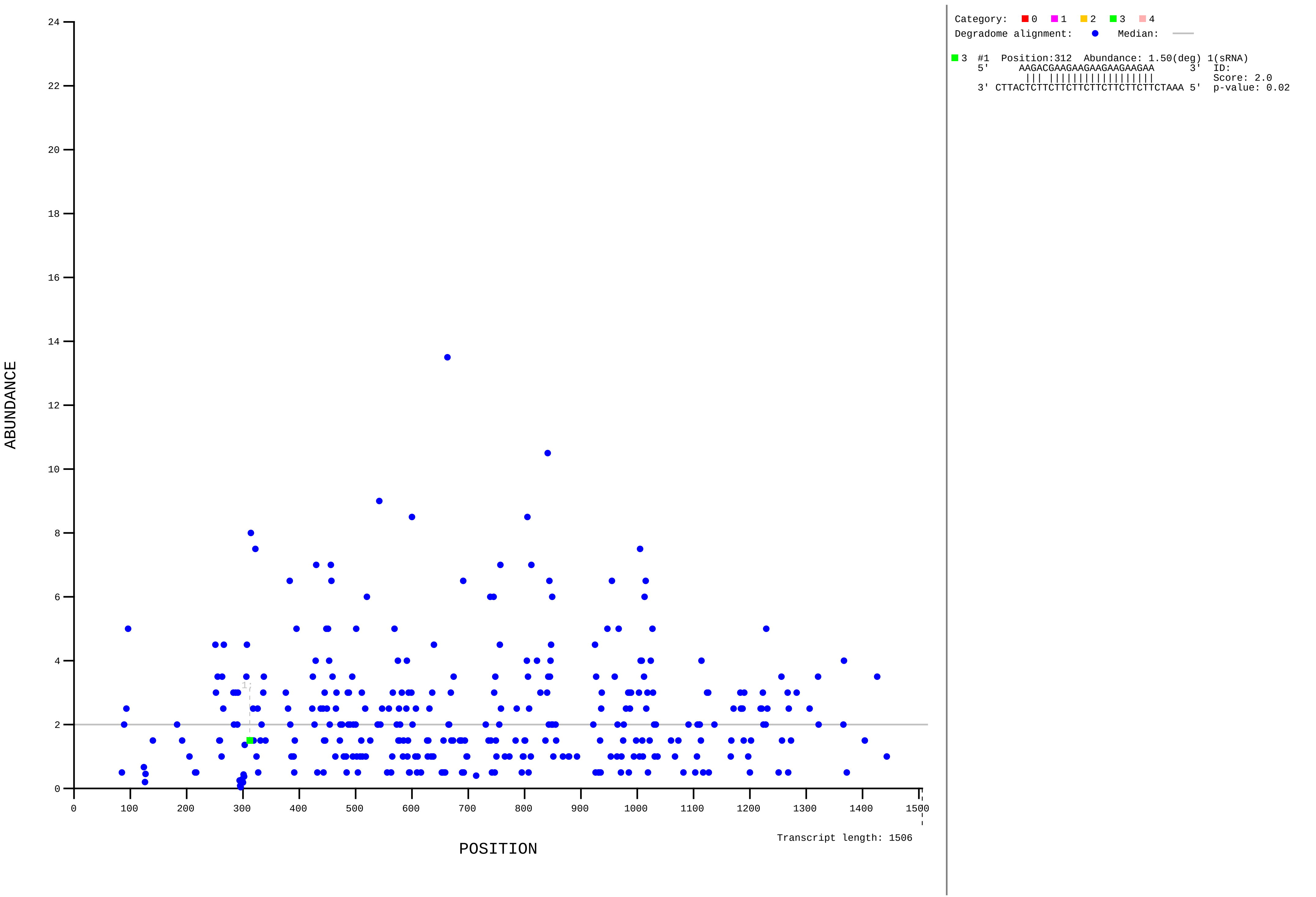

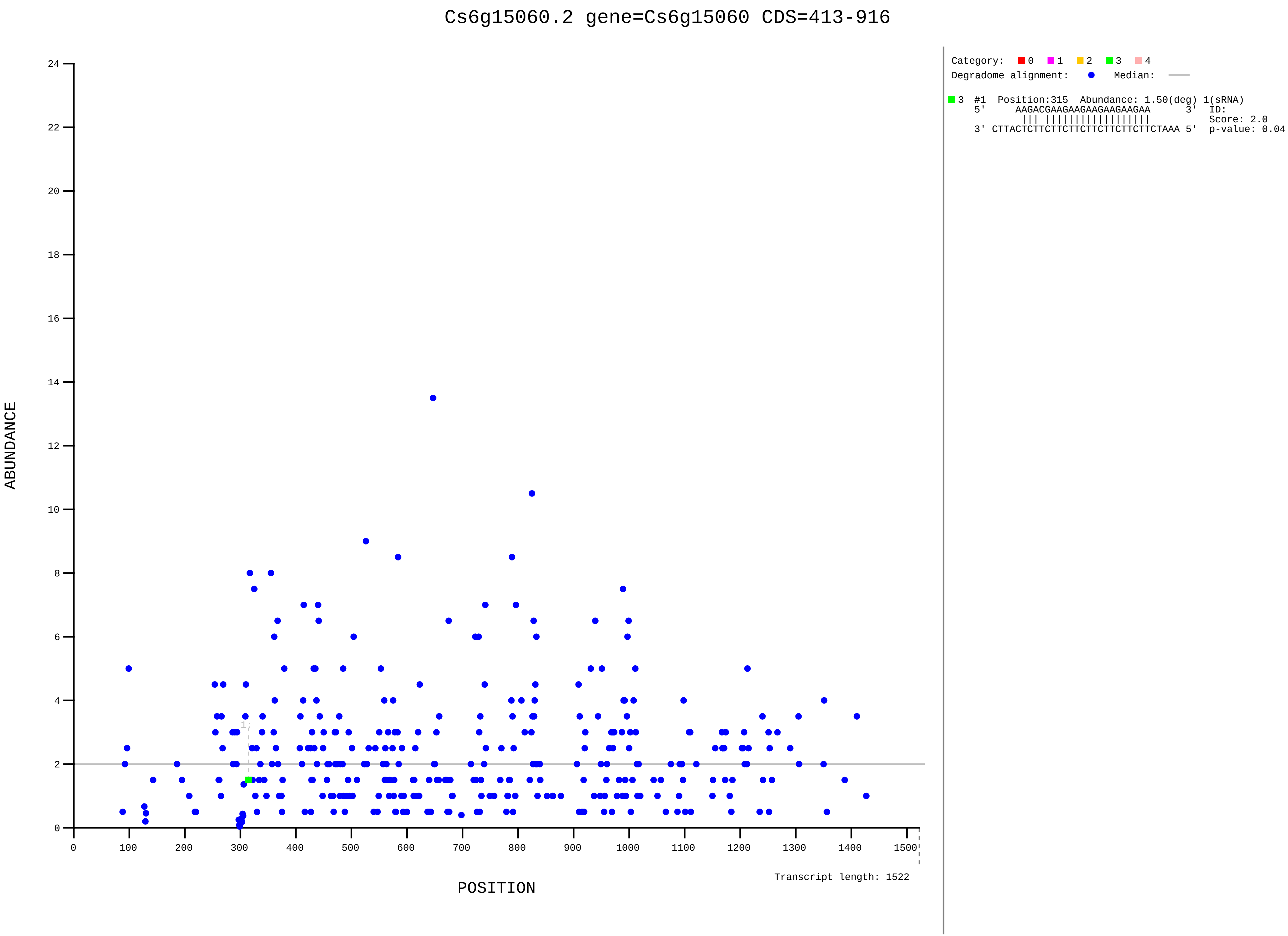

Cs6g15060.2 gene=Cs6g15060 CDS=413-916
ABUNDANCE
POSITION
Transcript length: 1522
Category: 0 1 2 3 4
Degradome alignment:
Median:
3 #1 Position:315 Abundance: 1.50(deg) 1(sRNA)
5' AAGACGAAGAAGAAGAAGAAGAA 3' ID:
||| |||||||||||||||||| Score: 2.0
3' CTTACTCTTCTTCTTCTTCTTCTTCTAAA 5' p-value: 0.04

orange1.1t03707.1 gene=orange1.1t03707 CDS=1-2796

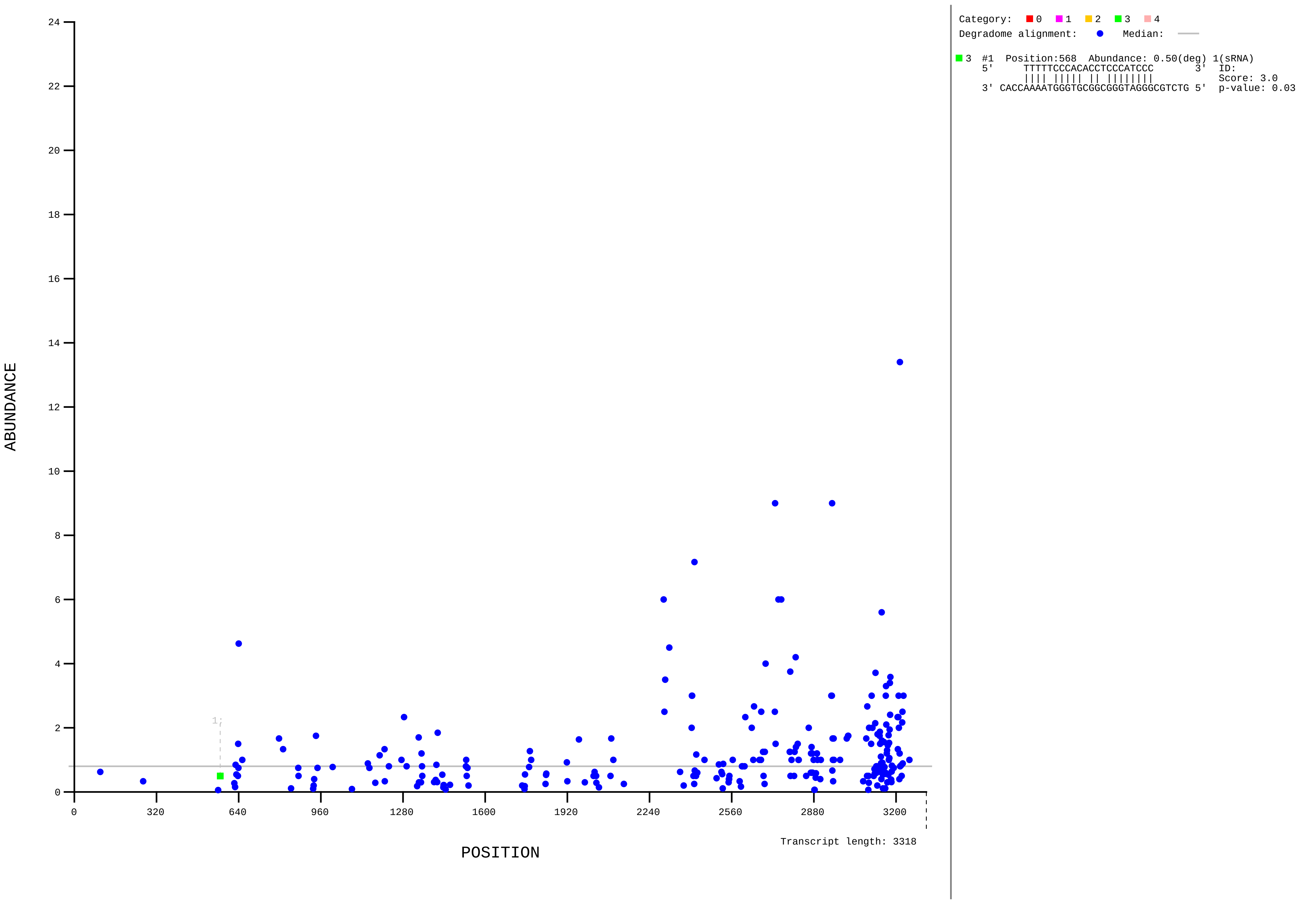

Cs1g19320.2 gene=Cs1g19320 CDS=158-1960

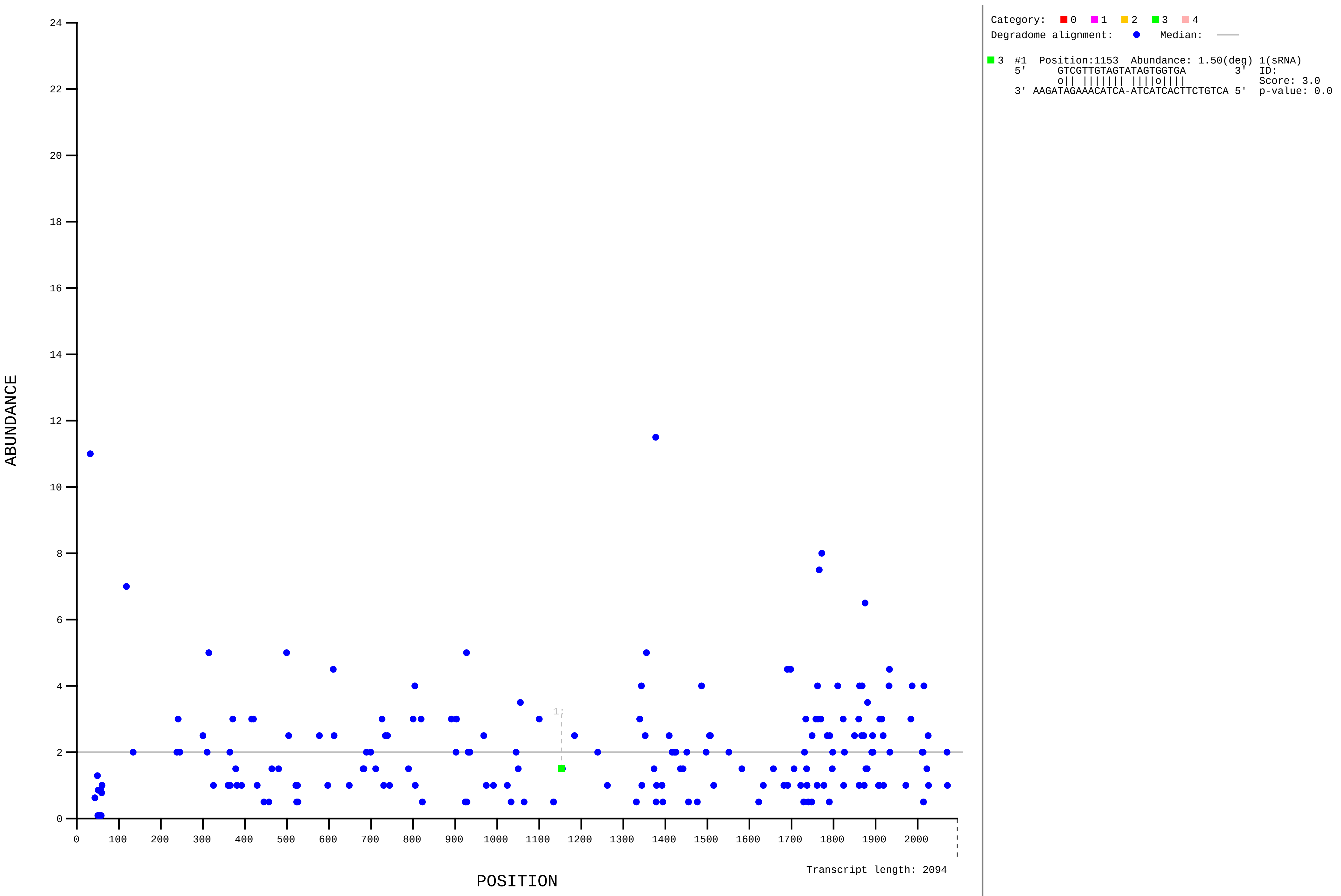

Cs1g19320.1 gene=Cs1g19320 CDS=66-1868

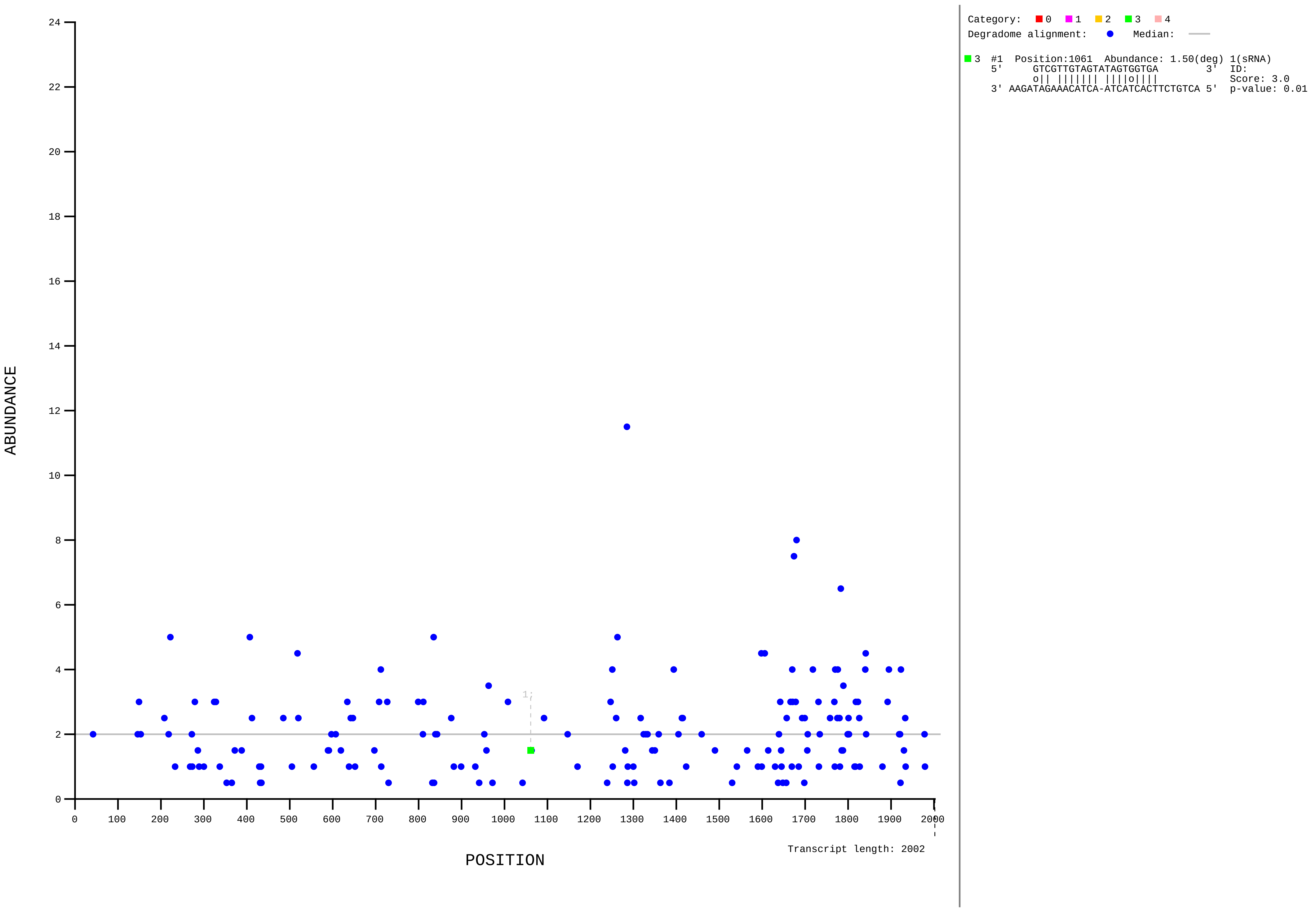

Cs1g08080.2 gene=Cs1g08080 CDS=116-3190

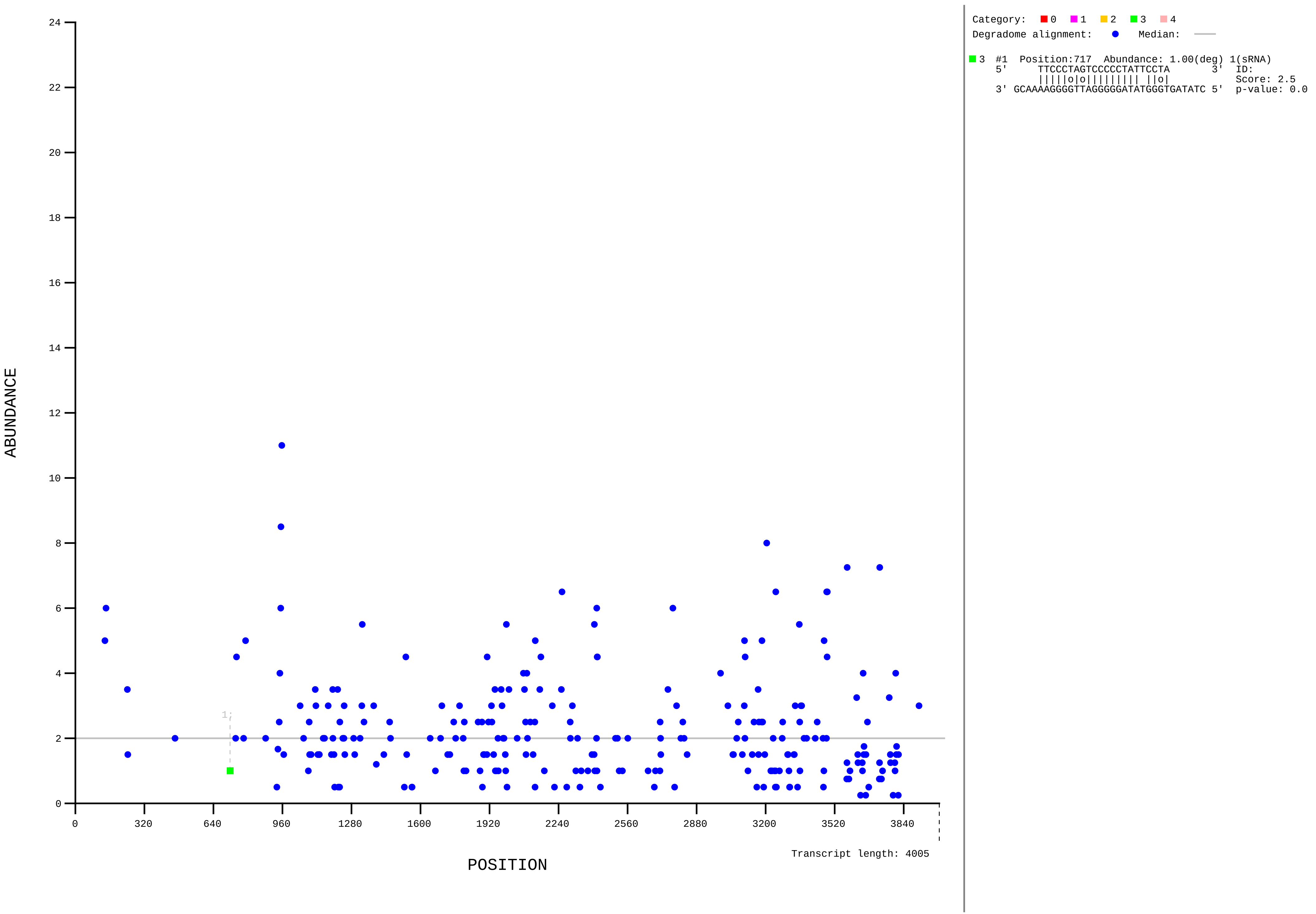

Cs1g08080.1 gene=Cs1g08080 CDS=116-3190

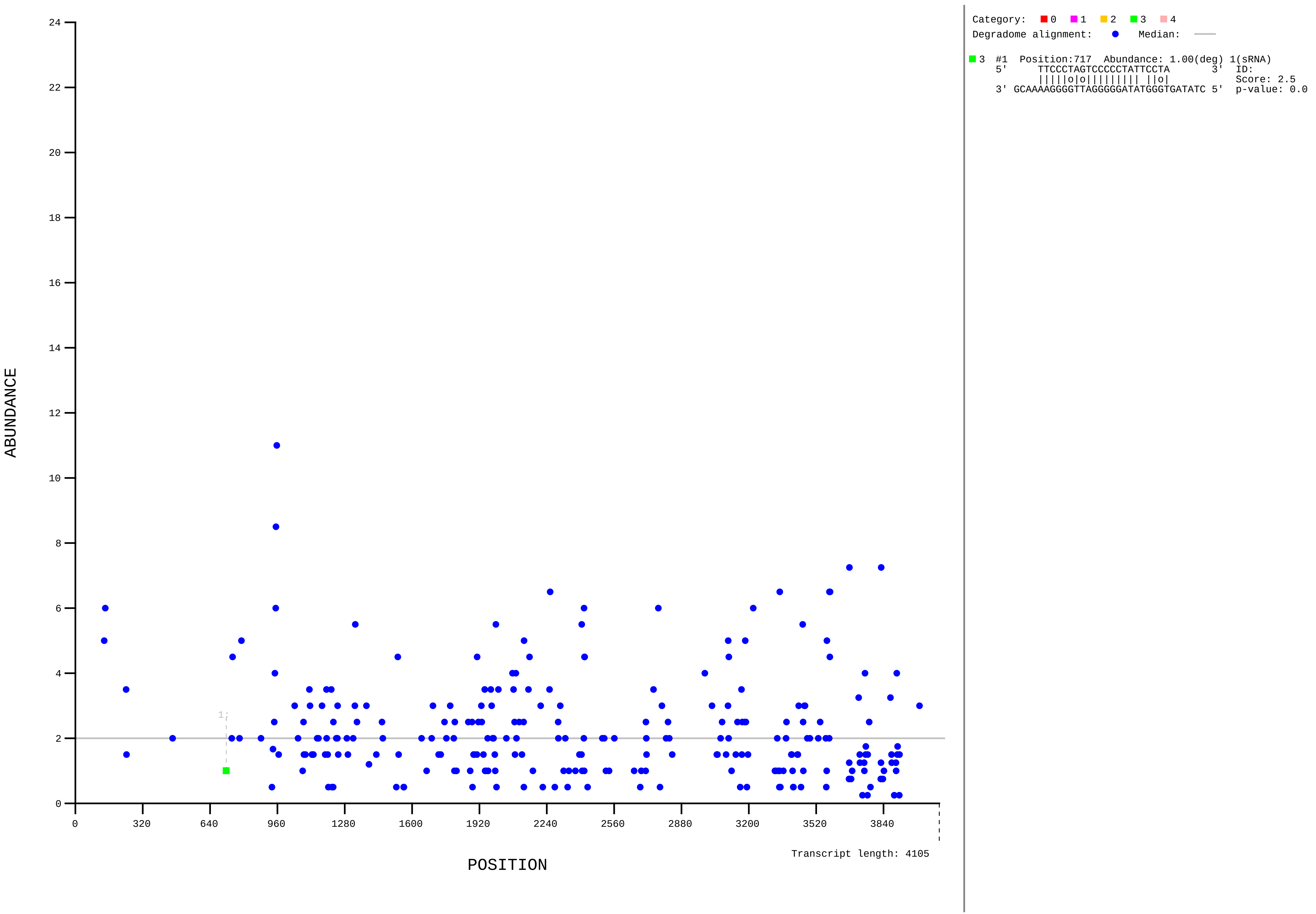

Cs5g03250.2 gene=Cs5g03250 CDS=312-1781

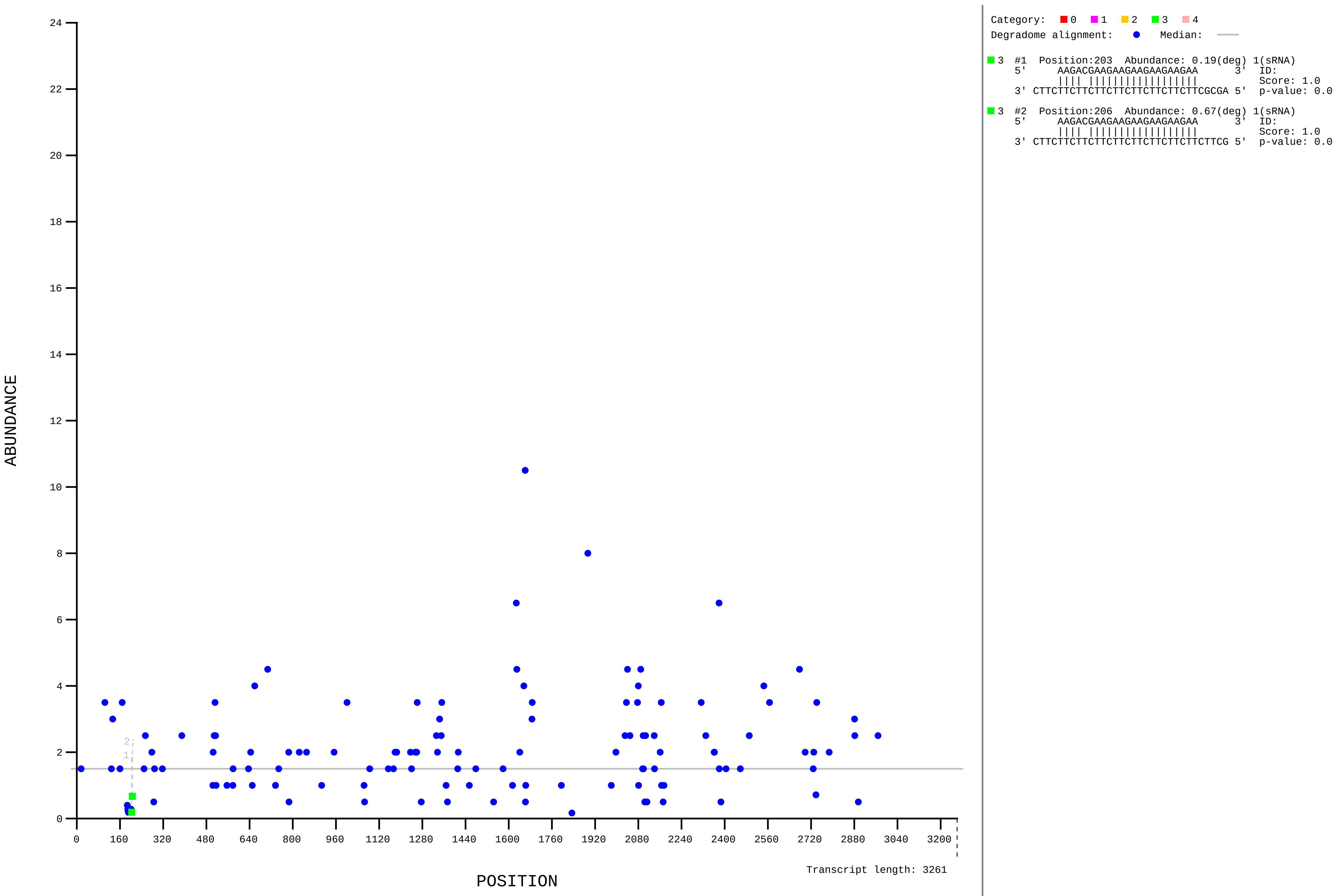

Cs5g03250.1 gene=Cs5g03250 CDS=312-2384

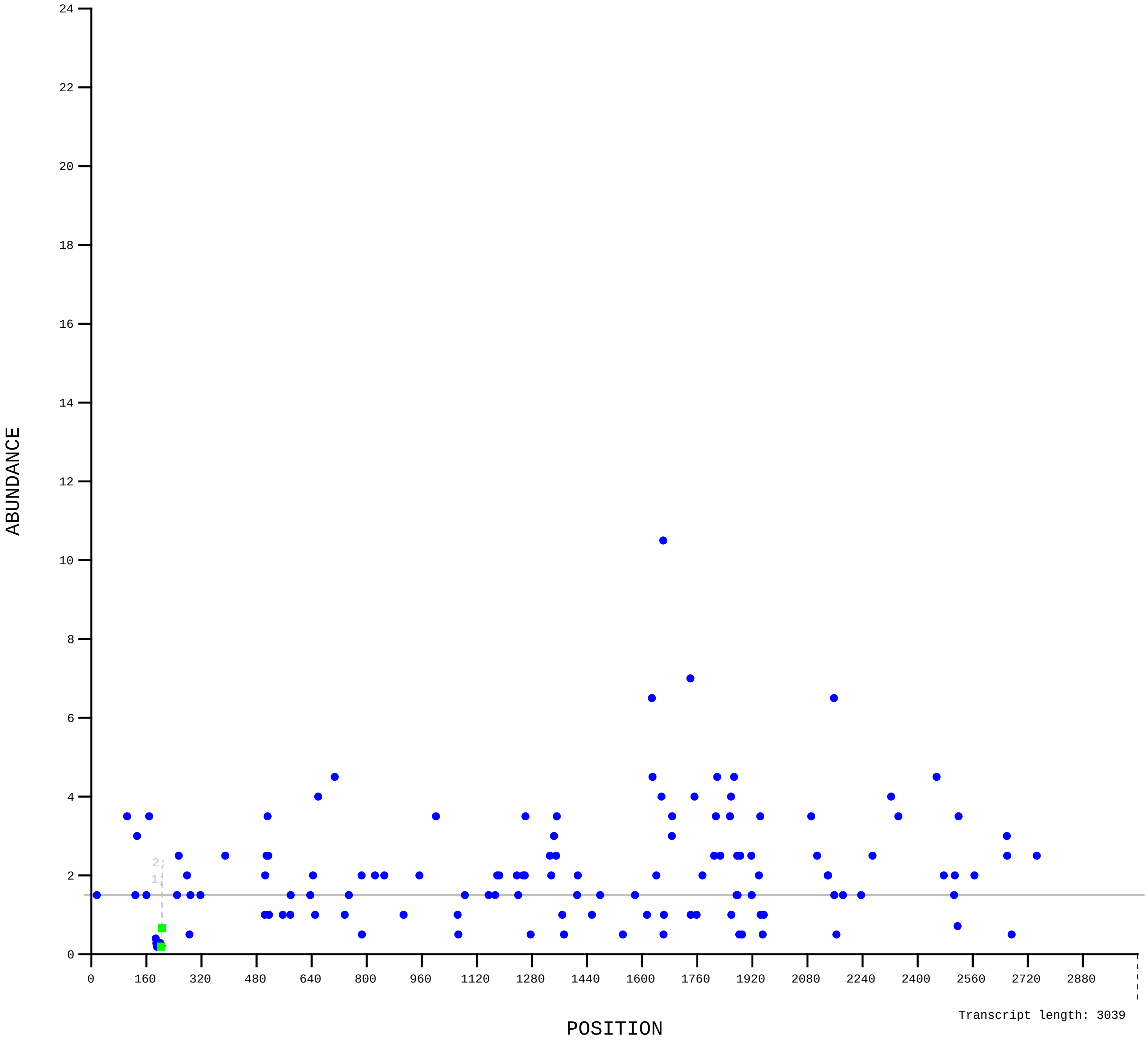

Category: 0 1 2 3 4
Degradome alignment: Median:

3 #1 Position:203 Abundance: 0.19(deg) 1(sRNA)
5' AAGACGAAGAAGAAGAAGAAGAA 3' ID:
|||| |||||||||||||||||| Score: 1.0
3' CTTCTTCTTCTTCTTCTTCTTCTTCTTCGCGA 5' p-value: 0.0

3 #2 Position:206 Abundance: 0.67(deg) 1(sRNA)
5' AAGACGAAGAAGAAGAAGAAGAA 3' ID:
|||| |||||||||||||||||| Score: 1.0
3' CTTCTTCTTCTTCTTCTTCTTCTTCTTCTTCG 5' p-value: 0.0

Cs7g22590.2 gene=Cs7g22590 CDS=30-1109

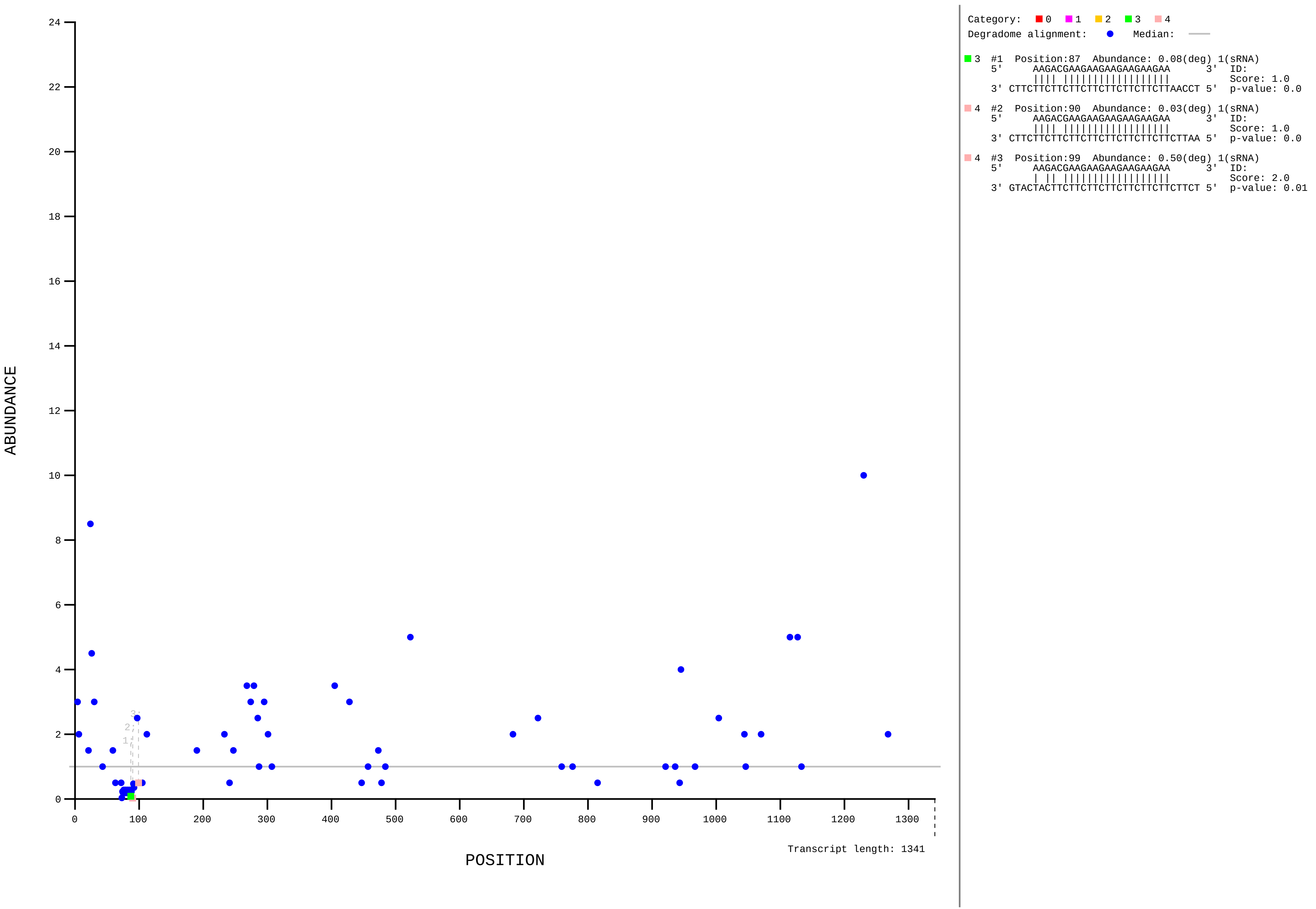

Cs7g04210.2 gene=Cs7g04210 CDS=387-2441

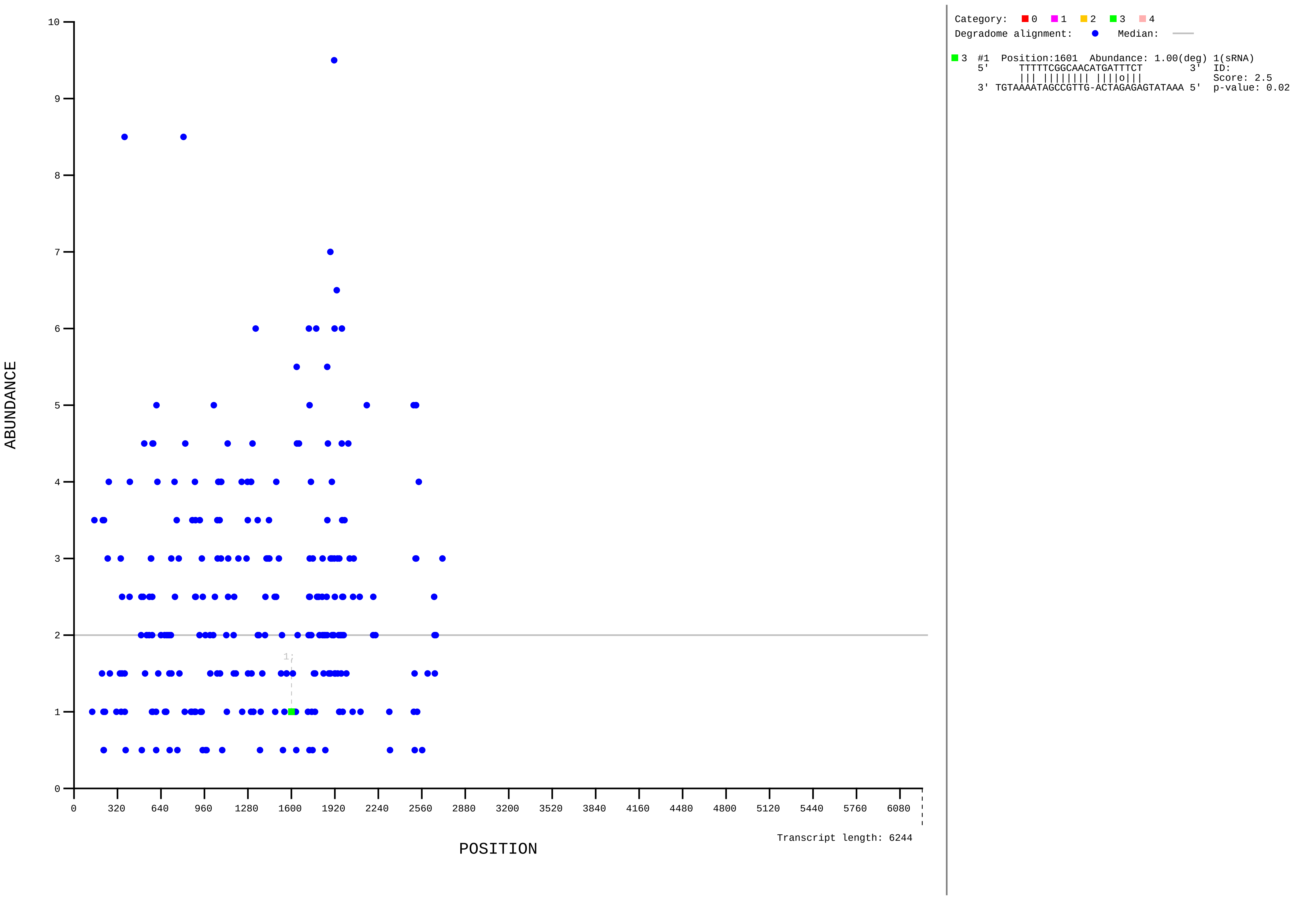

Cs7g04210.1 gene=Cs7g04210 CDS=387-2609

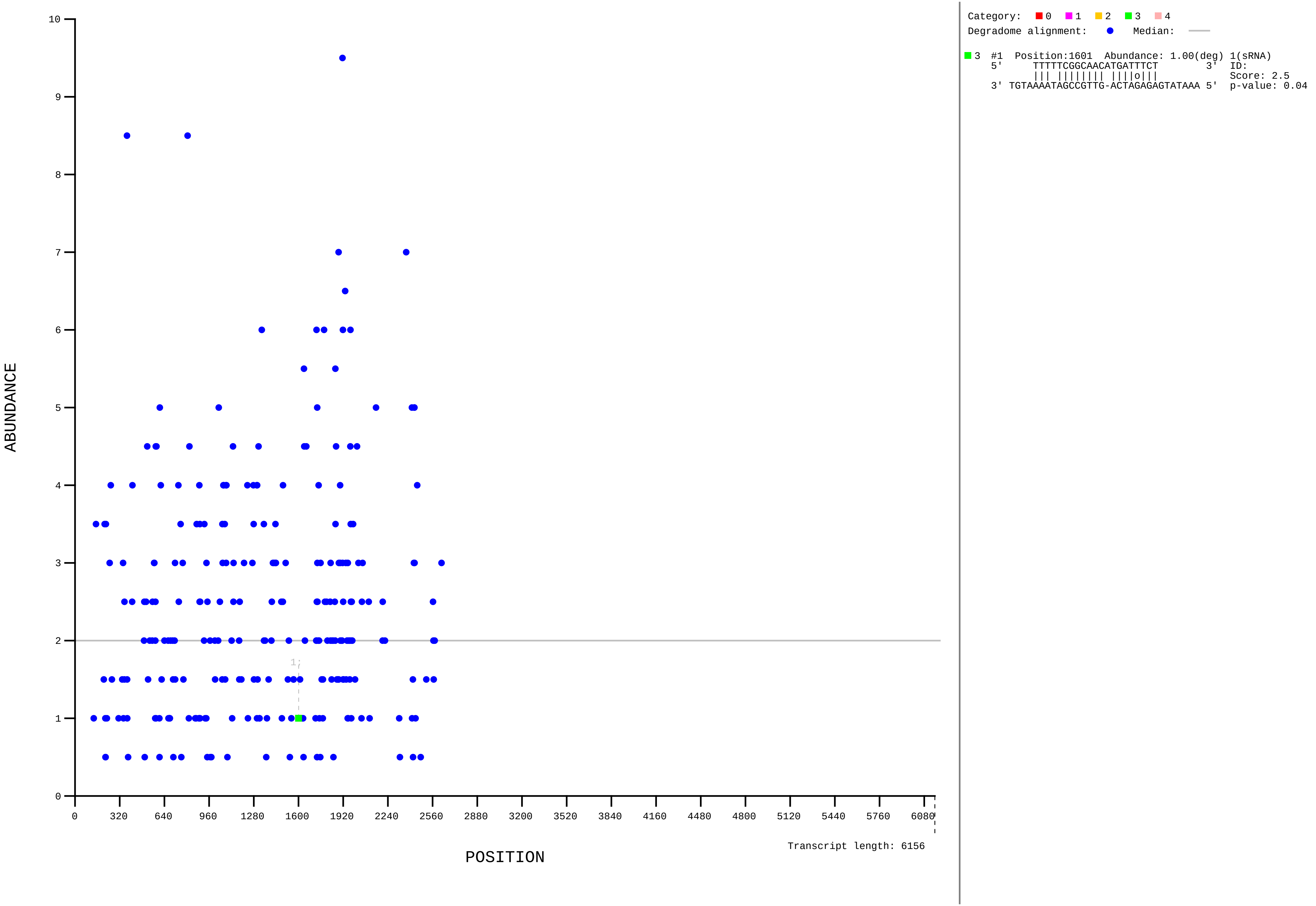

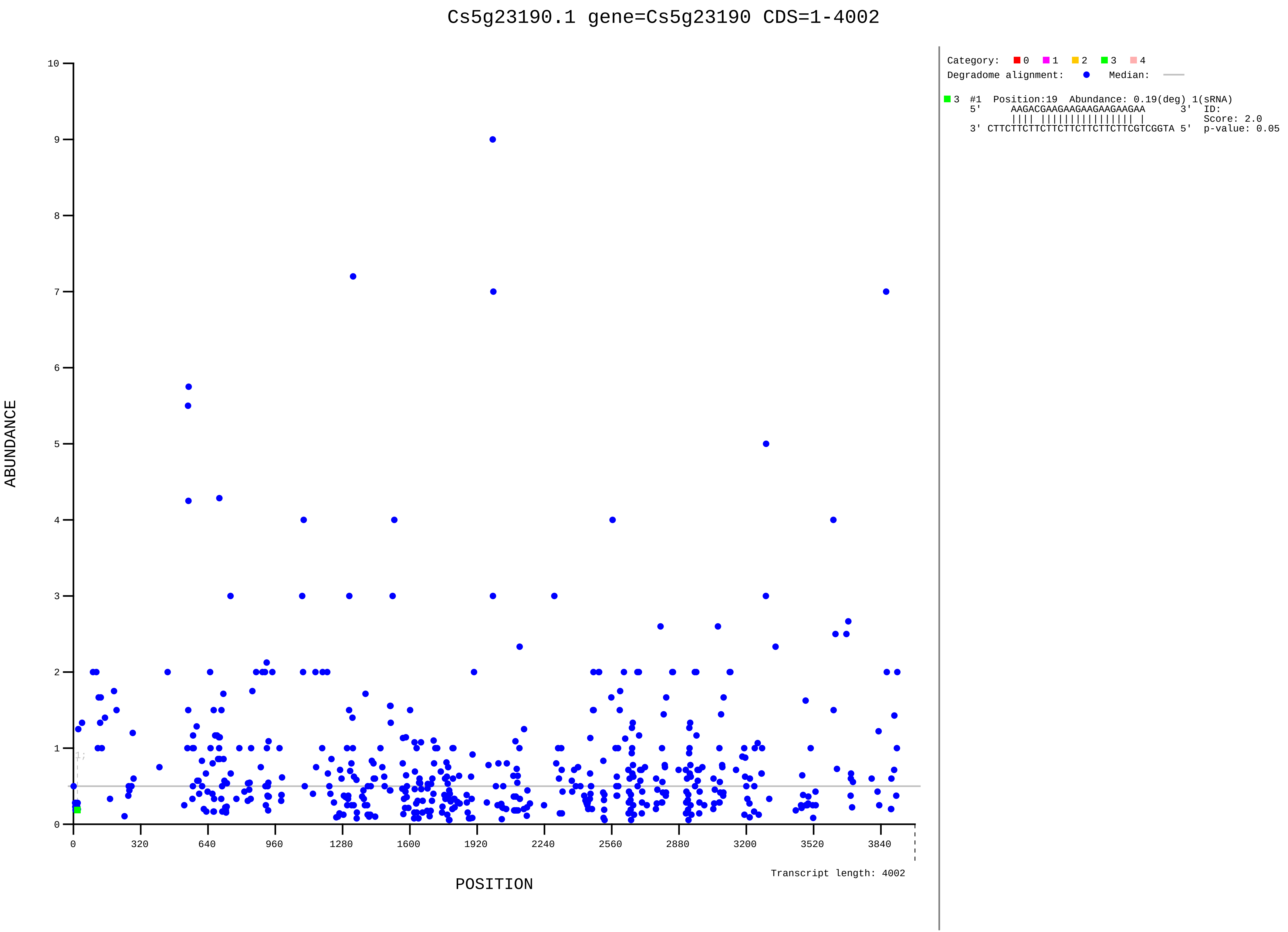
Cs5g23190.1 gene=Cs5g23190 CDS=1-4002
ABUNDANCE
POSITION
Transcript length: 4002
Category: 0 1 2 3 4
Degradome alignment:
Median:
3 #1 Position:19 Abundance: 0.19(deg) 1(sRNA)
5' AAGACGAAGAAGAAGAAGAAGAA 3' ID:
Score: 2.0
3' CTTCTTCTTCTTCTTCTTCTTCGTCGGTA 5' p-value: 0.05
0 1 2 3 4 5 6 7 8 9 10
0 320 640 960 1280 1600 1920 2240 2560 2880 3200 3520 3840

Cs9g09310.1 gene=Cs9g09310 CDS=251-1597

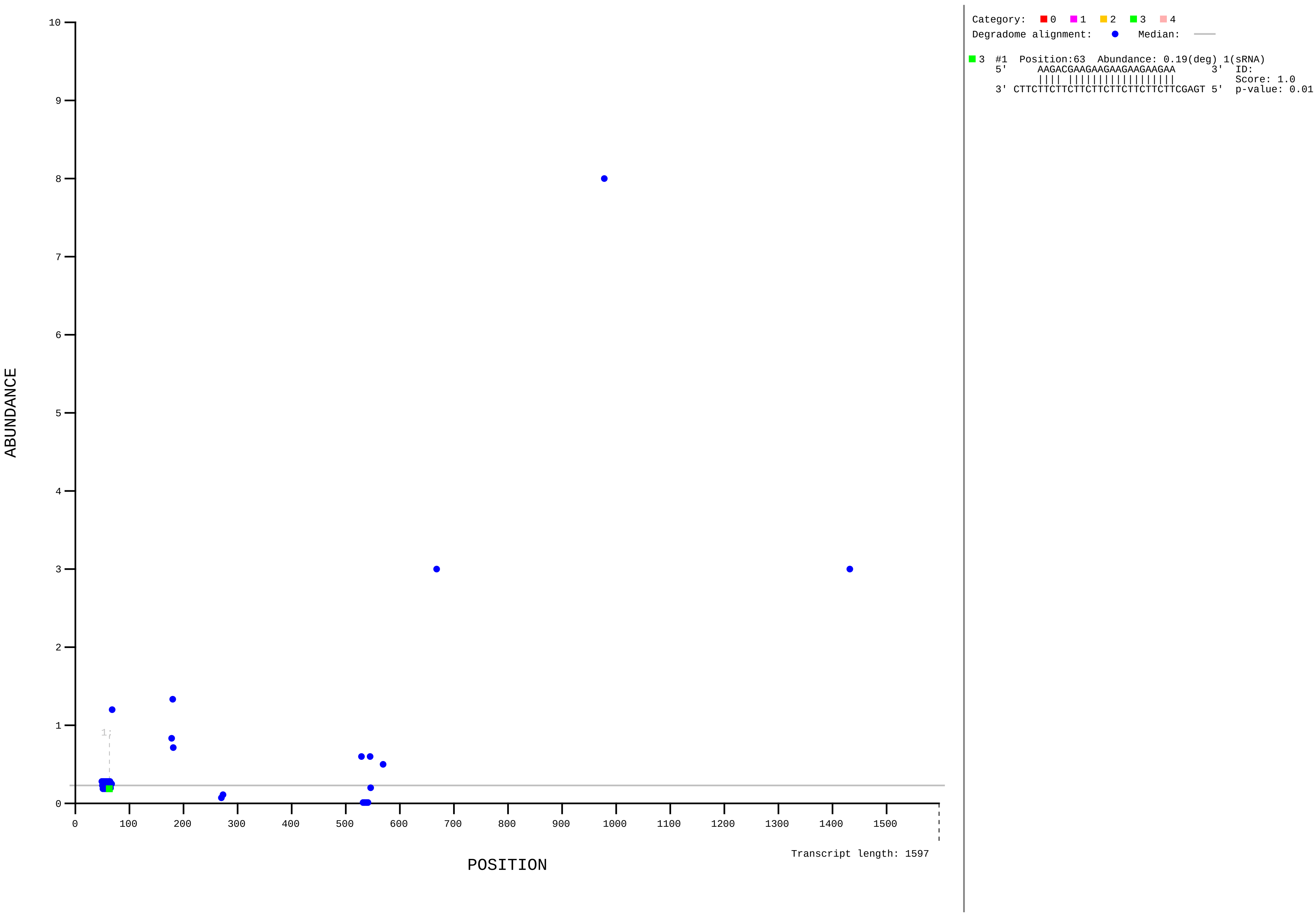

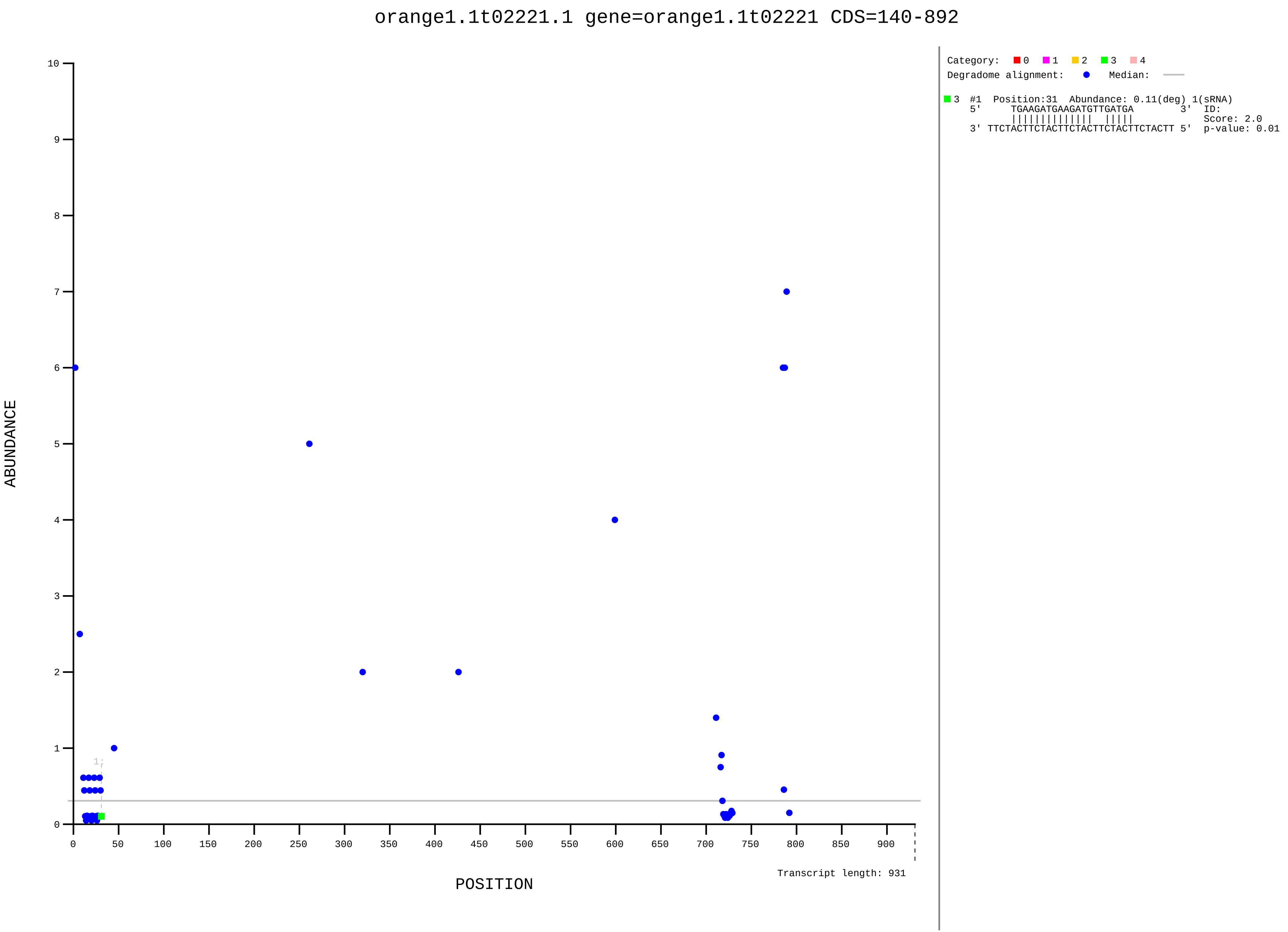
orange1.1t02221.1 gene=orange1.1t02221 CDS=140-892
Category: 0 1 2 3 4
Degradome alignment: Median:
3 #1 Position:31 Abundance: 0.11(deg) 1(sRNA)
5' TGAAGATGAAGATGTTGATGA 3' ID:
Score: 2.0
3' TTCTACTTCTACTTCTACTTCTACTTCTACTT 5' p-value: 0.01
ABUNDANCE
POSITION
Transcript length: 931

Cs9g06620.1 gene=Cs9g06620 CDS=195-1769

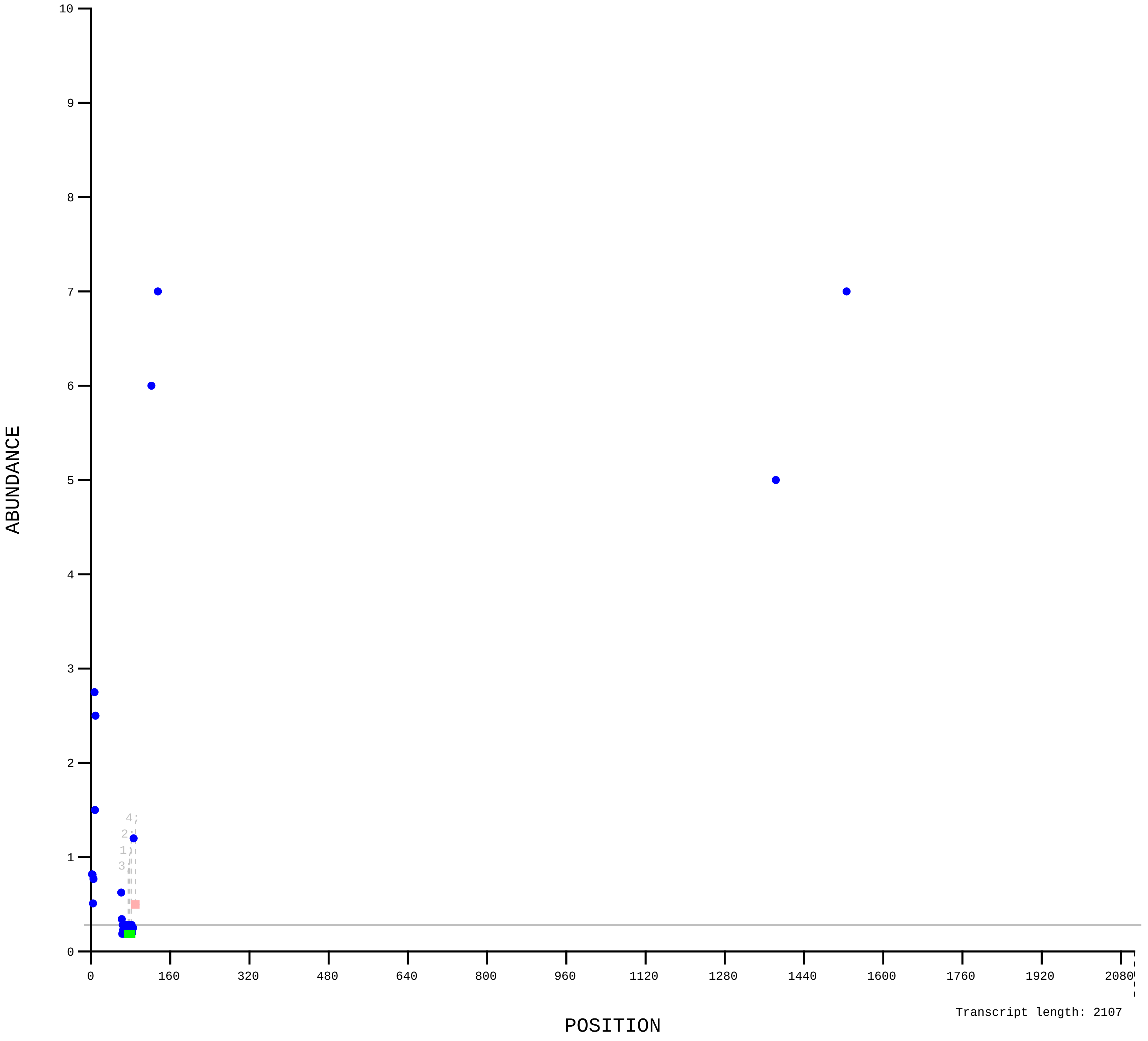


```
Category:  ■0  ■1  ■2  ■3  ■4
Degradome alignment: ●   Median: ——

■3  #1  Position:78  Abundance: 0.19(deg) 1(sRNA)
    5'      AAGACGAAGAAGAAGAAGAAGAA      3'  ID:
            |||| ||||||||||||||||||          Score: 1.0
    3' CTTCTTCTTCTTCTTCTTCTTCTTCTTCTCTA 5'  p-value: 0.0

■3  #2  Position:81  Abundance: 0.19(deg) 1(sRNA)
    5'      AAGACGAAGAAGAAGAAGAAGAA      3'  ID:
            |||| ||||||||||||||||||          Score: 1.0
    3' CTTCTTCTTCTTCTTCTTCTTCTTCTTCTTCT 5'  p-value: 0.0

■3  #3  Position:75  Abundance: 0.19(deg) 1(sRNA)
    5'      AAGACGAAGAAGAAGAAGAAGAA      3'  ID:
            |||| |||||||||||||||||           Score: 2.0
    3' CTTCTTCTTCTTCTTCTTCTTCTTCTCTAATA 5'  p-value: 0.05

■4  #4  Position:90  Abundance: 0.50(deg) 1(sRNA)
    5'      AAGACGAAGAAGAAGAAGAAGAA      3'  ID:
            |||| ||||||||||||||||||          Score: 1.0
    3' GTTCTTCTTCTTCTTCTTCTTCTTCTTCTTCT 5'  p-value: 0.0
```

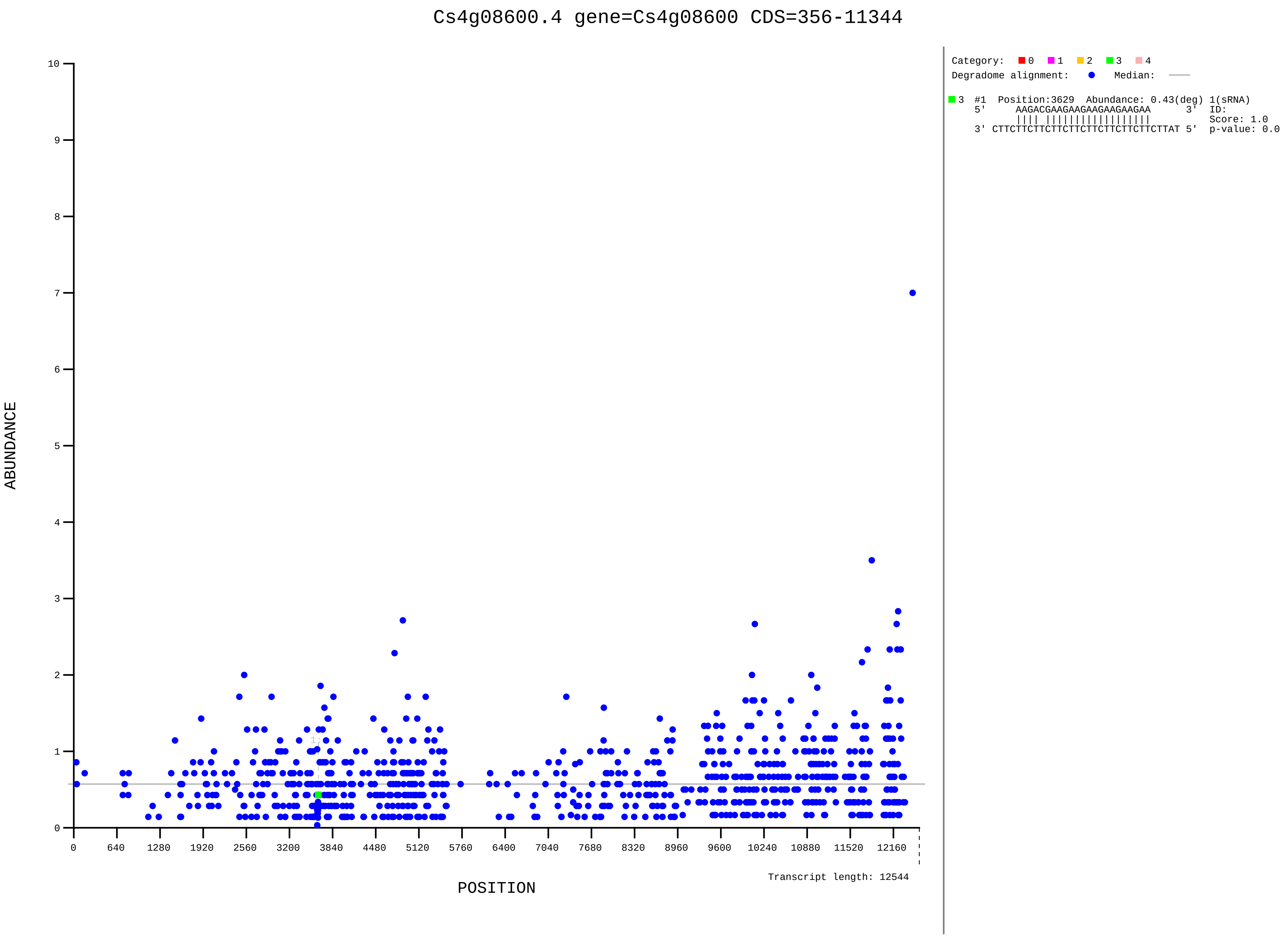

Cs4g08600.4 gene=Cs4g08600 CDS=356-11344
ABUNDANCE
POSITION
Transcript length: 12544
Category: 0 1 2 3 4
Degradome alignment: Median:
3 #1 Position:3629 Abundance: 0.43(deg) 1(sRNA)
5' AAGACGAAGAAGAAGAAGAAGAA 3' ID:
Score: 1.0
3' CTTCTTCTTCTTCTTCTTCTTCTTCTTCTTAT 5' p-value: 0.0

Cs6g05690.1 gene=Cs6g05690 CDS=509-2104

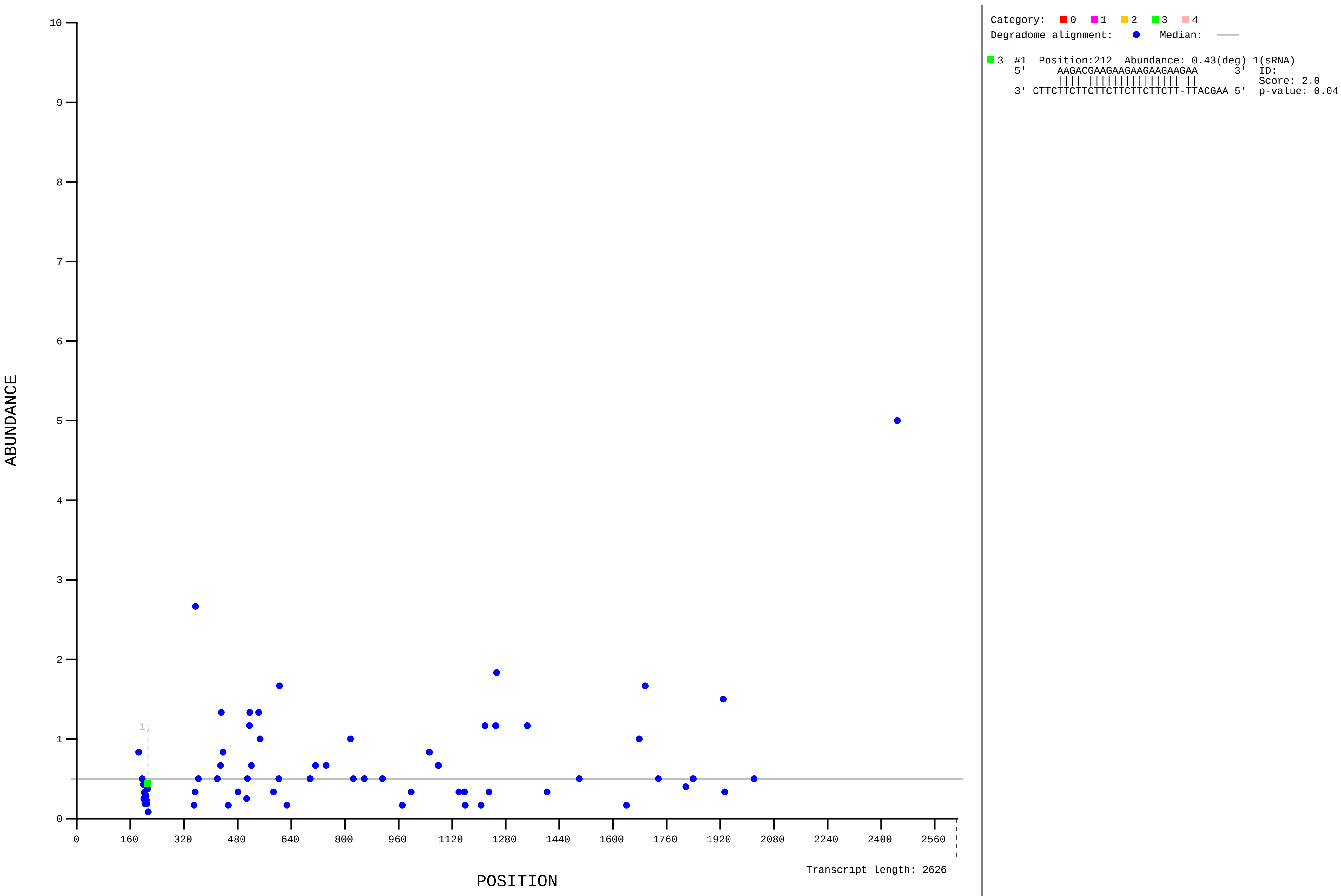

Cs1g02530.2 gene=Cs1g02530 CDS=37-1539

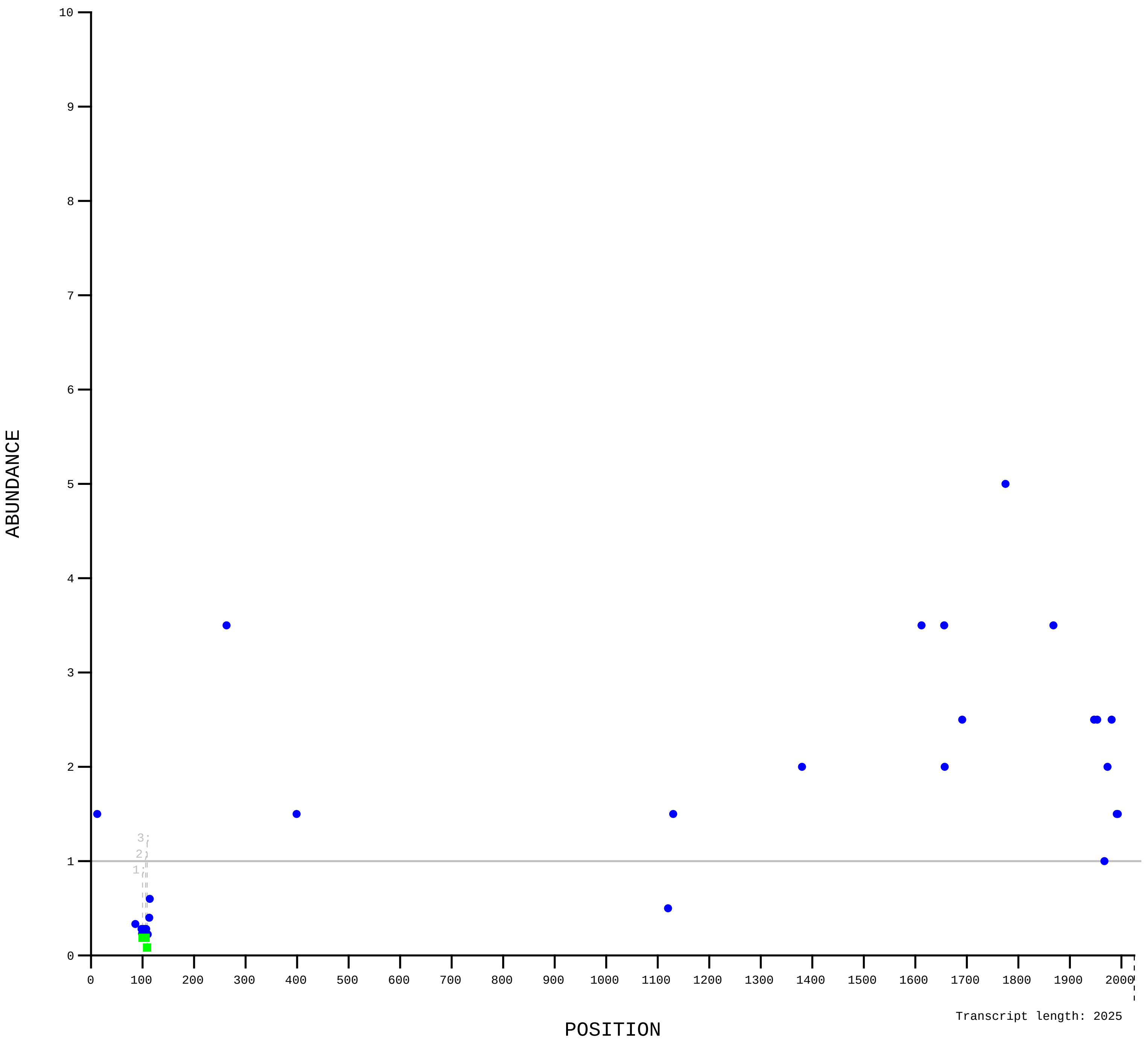

Category: 0 1 2 3 4
Degradome alignment: Median:

3 #1 Position:100 Abundance: 0.19(deg) 1(sRNA)

```
5'     AAGACGAAGAAGAAGAAGAAGAA      3'  ID:
       |||| ||||||| ||||||||||          Score: 2.0
3' CTTCTTCTTCTTCTTCGTCTTCTTCTTCTCTC 5'  p-value: 0.03
```

3 #2 Position:106 Abundance: 0.19(deg) 1(sRNA)

```
5'     AAGACGAAGAAGAAGAAGAAGAA      3'  ID:
       |||| ||||||||||||| ||||          Score: 2.0
3' CTTCTTCTTCTTCTTCTTCTTCGTCTTCTTCT 5'  p-value: 0.05
```

3 #3 Position:109 Abundance: 0.08(deg) 1(sRNA)

```
5'     AAGACGAAGAAGAAGAAGAAGAA      3'  ID:
       |||| |||||||||||||||| |          Score: 2.0
3' CTTCTTCTTCTTCTTCTTCTTCTTCGTCTTCT 5'  p-value: 0.04
```

Cs8g20960.1 gene=Cs8g20960 CDS=438-2624

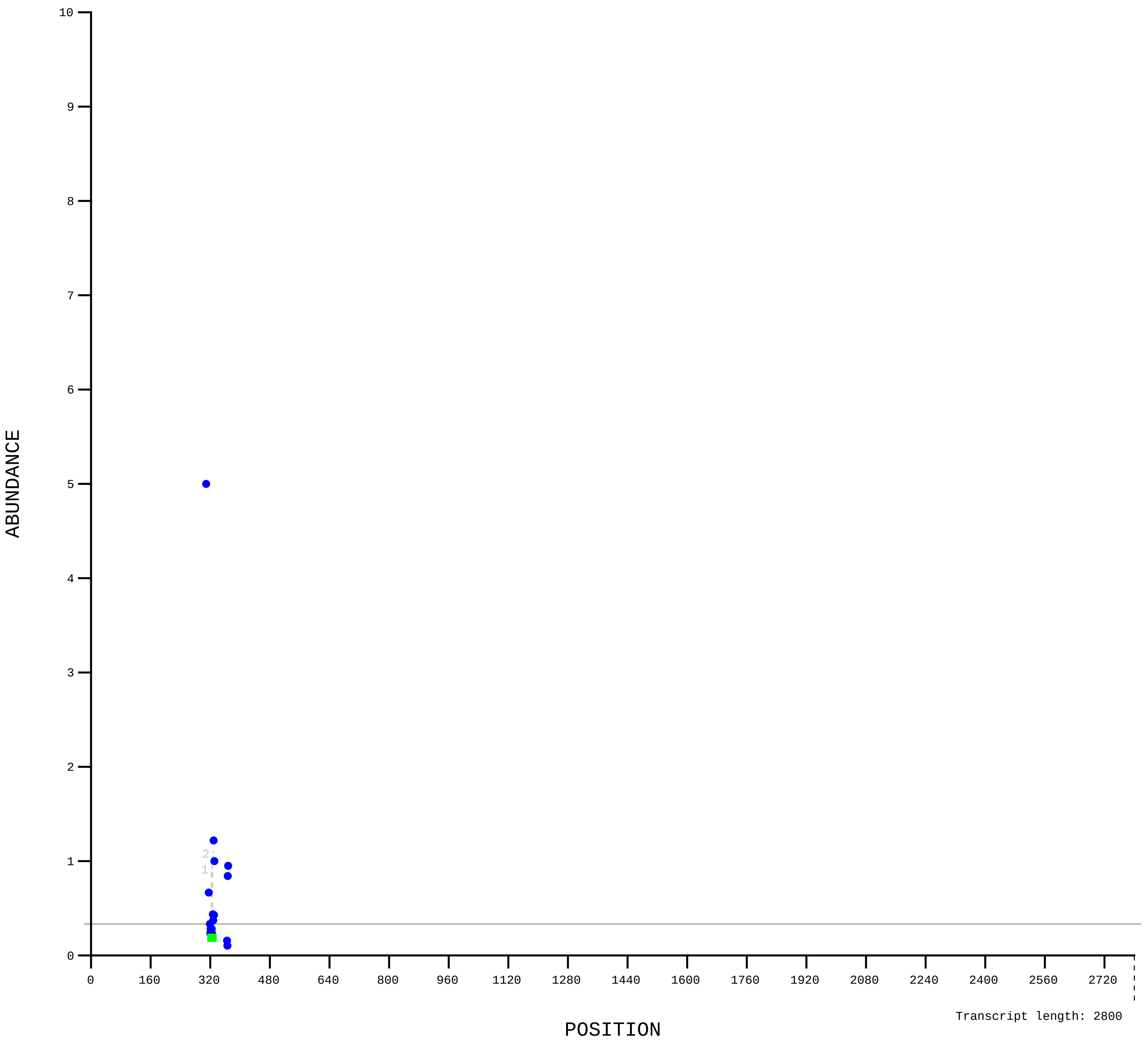

Category: 0 1 2 3 4
Degradome alignment: Median:

```
3  #1  Position:323  Abundance: 0.19(deg) 1(sRNA)
   5'      AAGACGAAGAAGAAGAAGAAGAA      3'  ID:
           |||| ||||||| ||||||||||          Score: 2.0
   3' CTTCTTCTTCTTCTTCCTCTTCTTCTTCTATT 5'  p-value: 0.04

3  #2  Position:326  Abundance: 0.19(deg) 1(sRNA)
   5'      AAGACGAAGAAGAAGAAGAAGAA      3'  ID:
           |||| |||||||||| |||||||          Score: 2.0
   3' CTTCTTCTTCTTCTTCTTCCTCTTCTTCTTCT 5'  p-value: 0.04
```

Cs1g02530.1 gene=Cs1g02530 CDS=431-1531

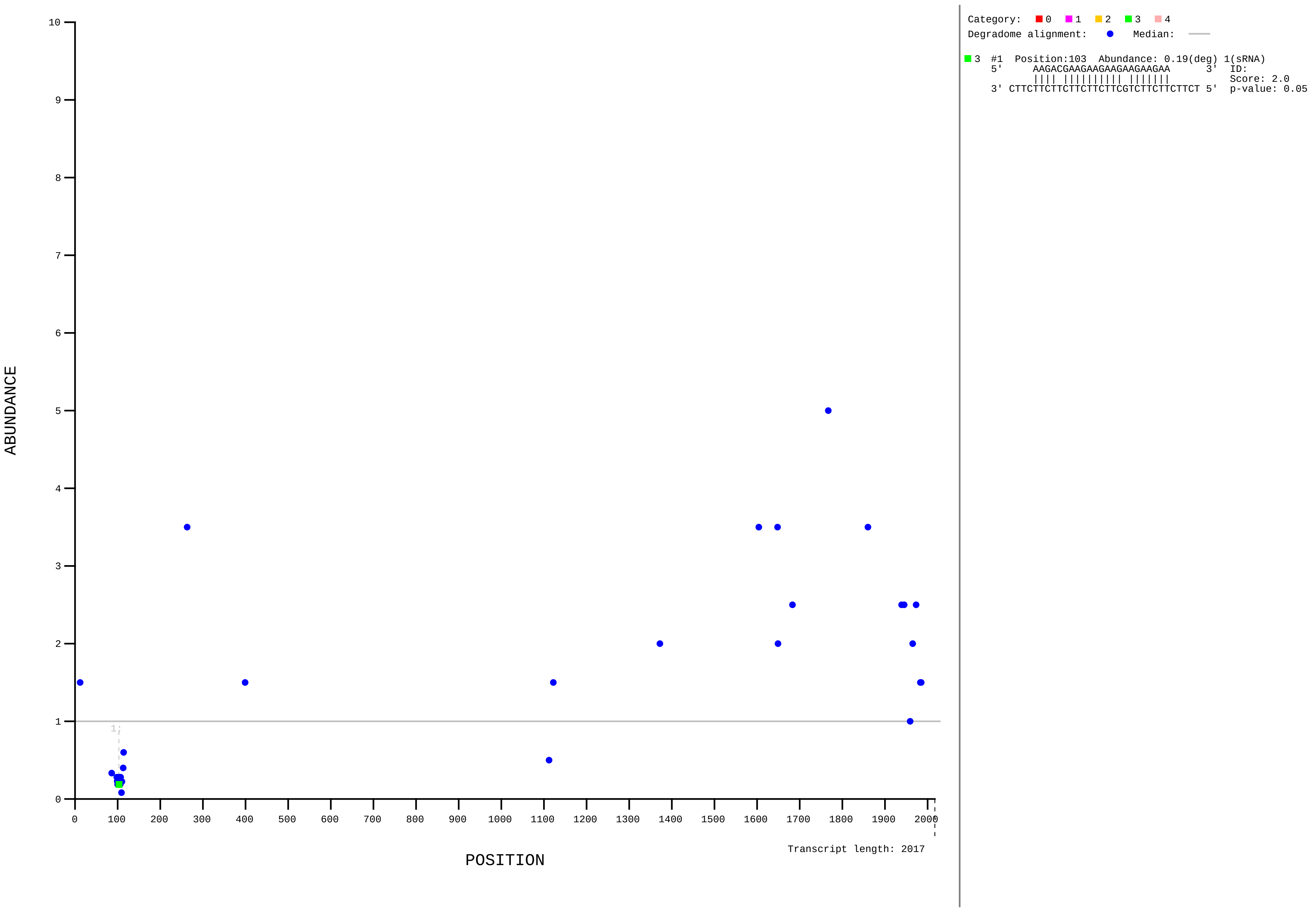

Cs4g06910.1 gene=Cs4g06910 CDS=556-1038

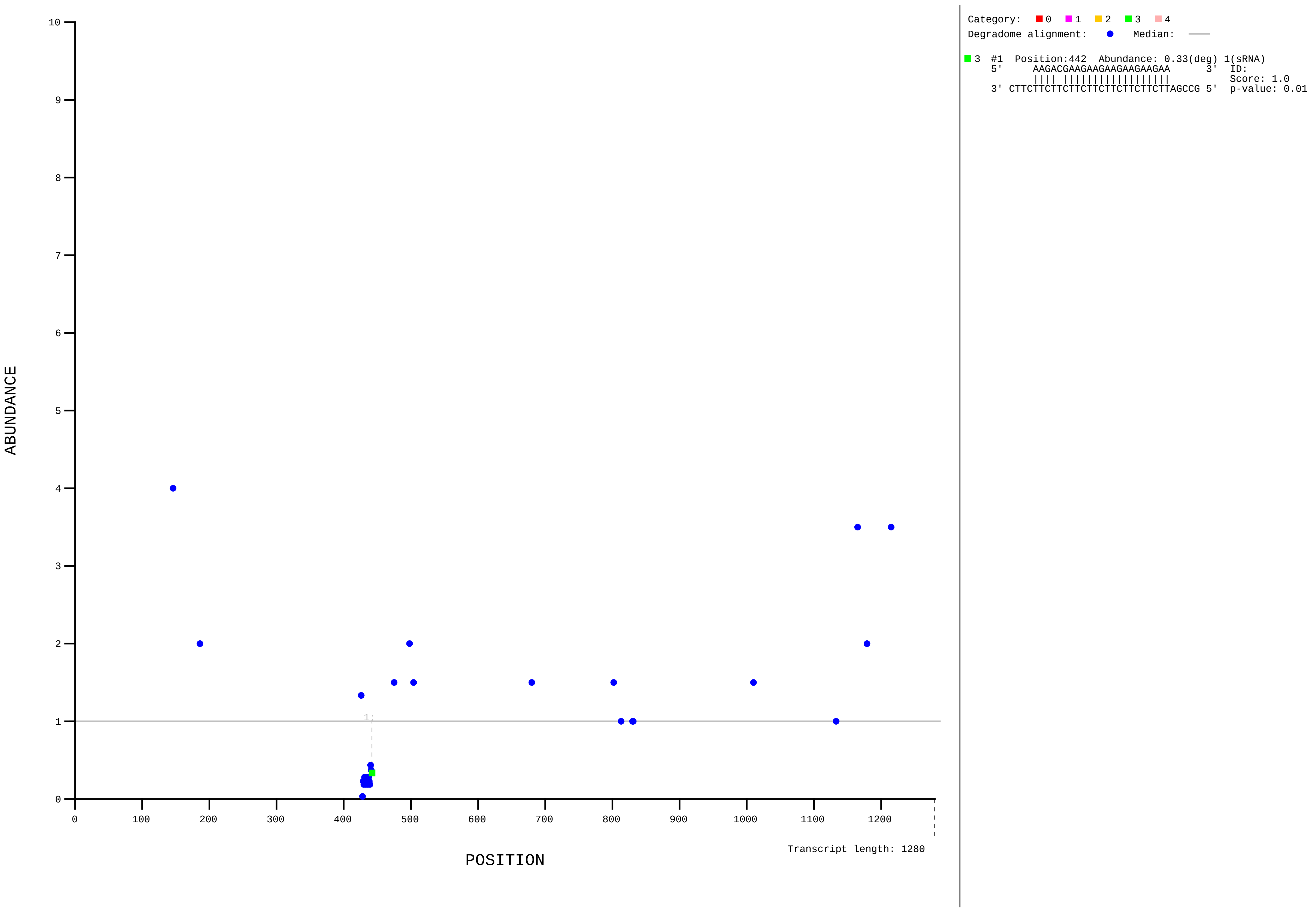

Cs4g06140.2 gene=Cs4g06140 CDS=240-875

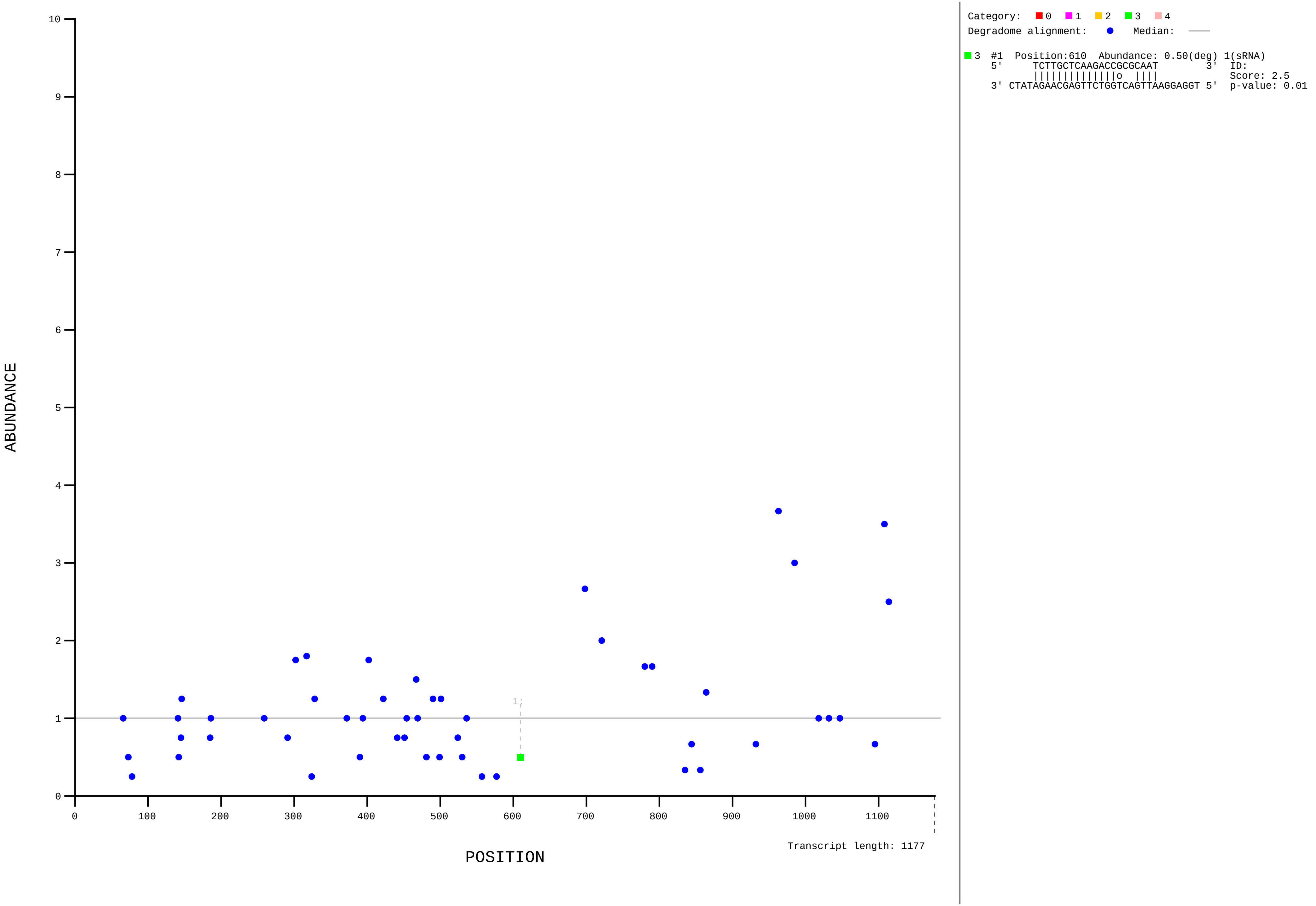

Cs4g06140.1 gene=Cs4g06140 CDS=240-875

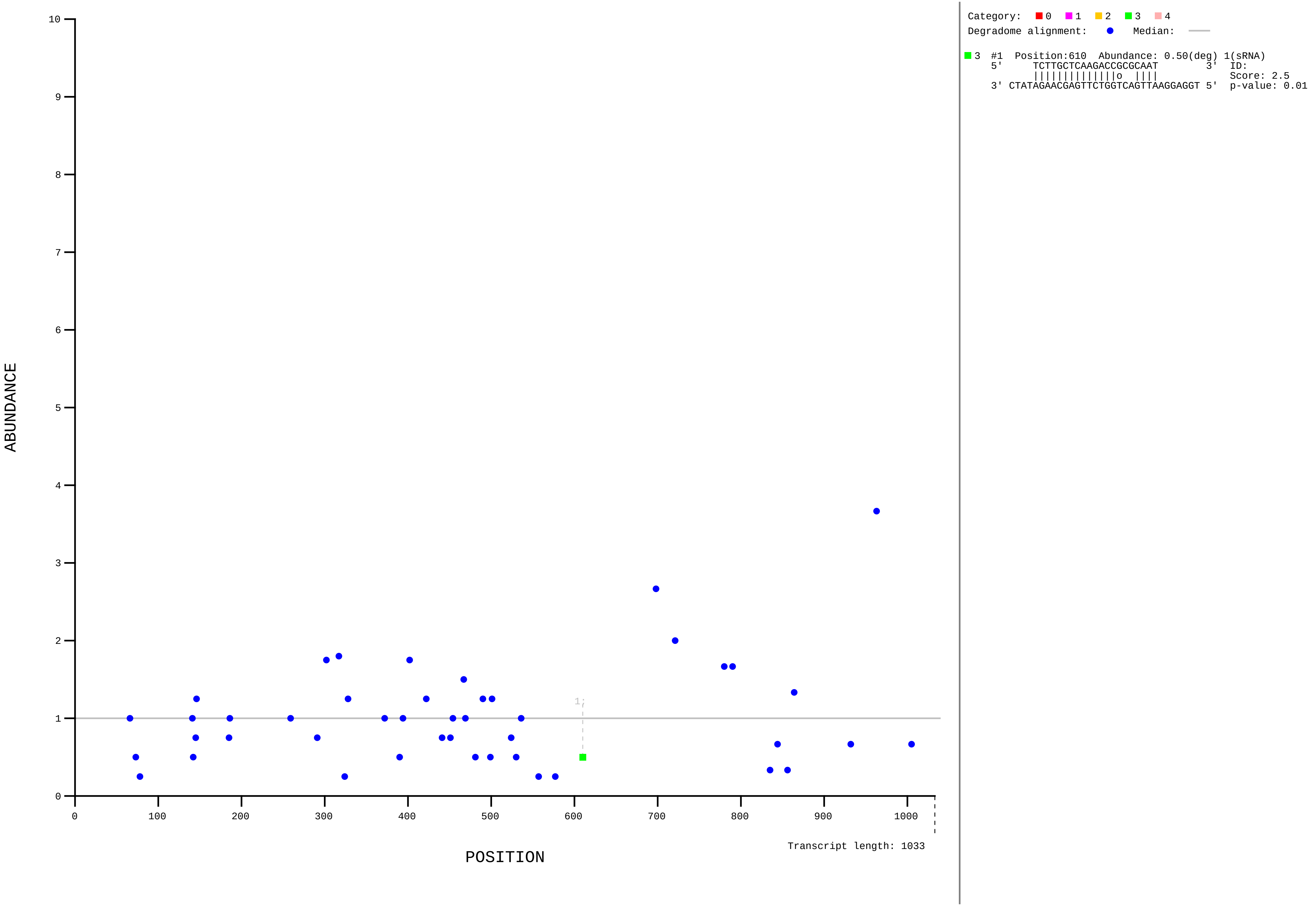

Cs4g06140.3 gene=Cs4g06140 CDS=240-956

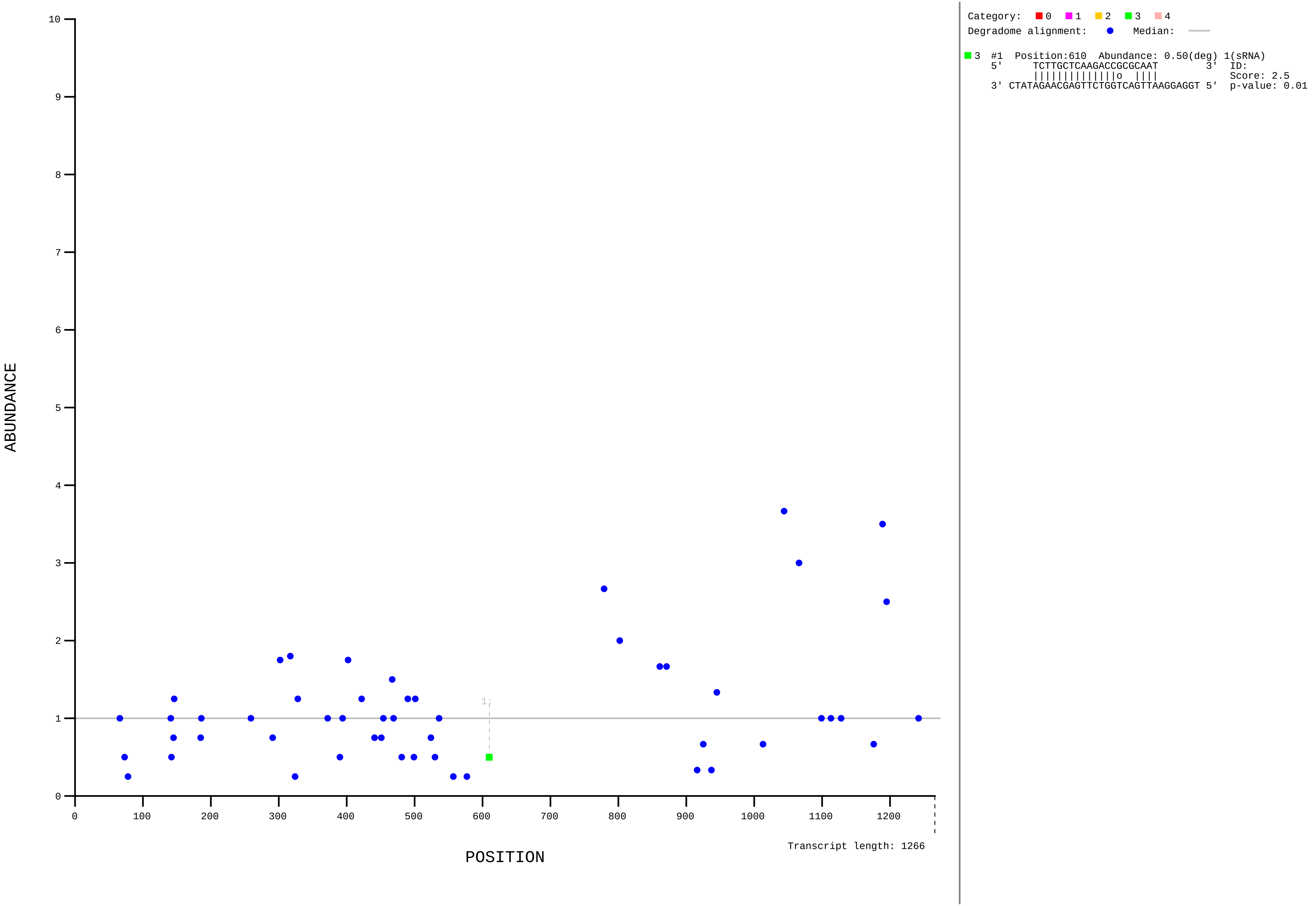

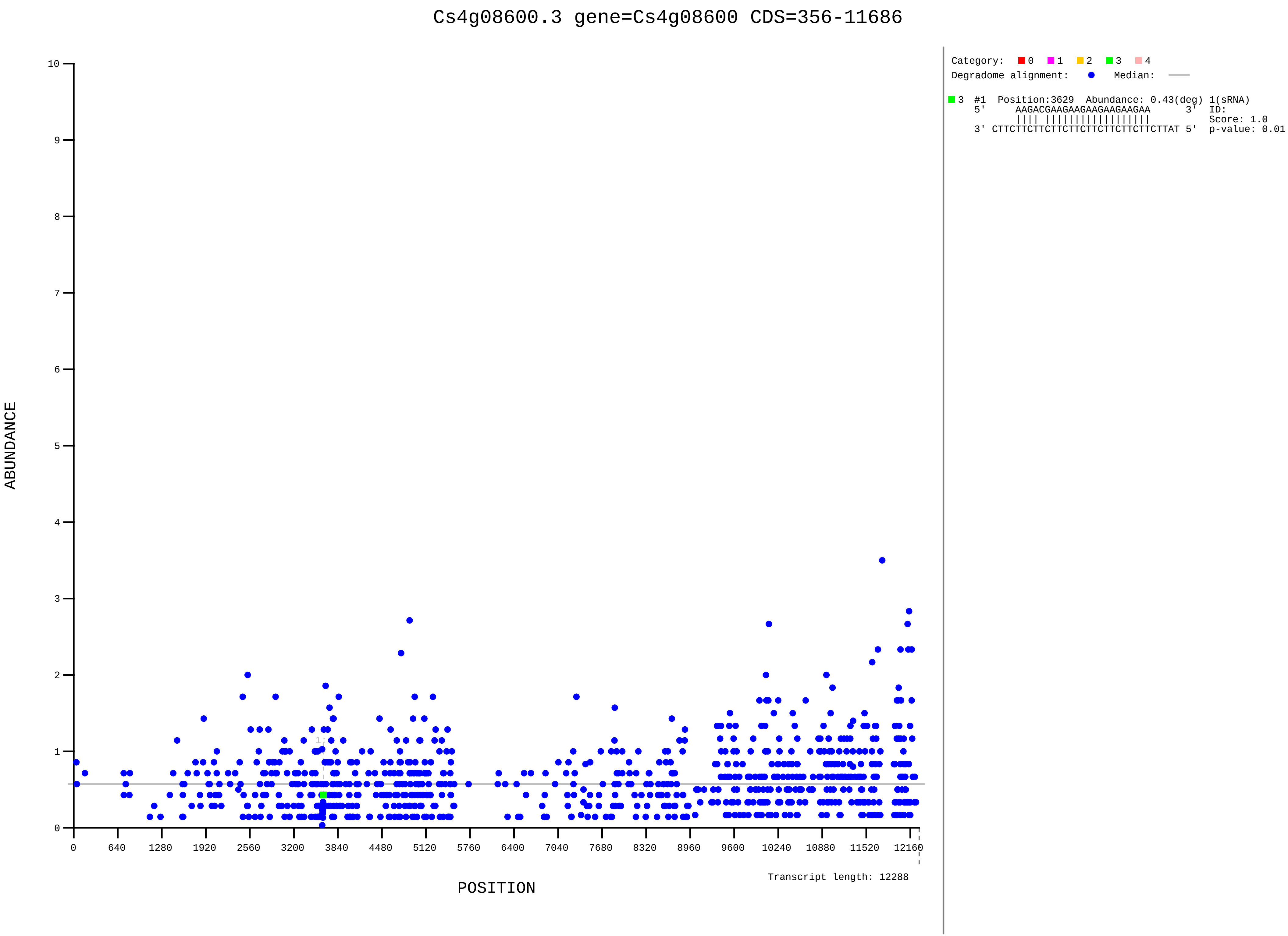

Cs4g08600.3 gene=Cs4g08600 CDS=356-11686
ABUNDANCE
POSITION
Transcript length: 12288
Category: 0 1 2 3 4
Degradome alignment: Median:
3 #1 Position:3629 Abundance: 0.43(deg) 1(sRNA)
5' AAGACGAAGAAGAAGAAGAAGAA 3' ID:
3' CTTCTTCTTCTTCTTCTTCTTCTTCTTAT 5'
Score: 1.0
p-value: 0.01

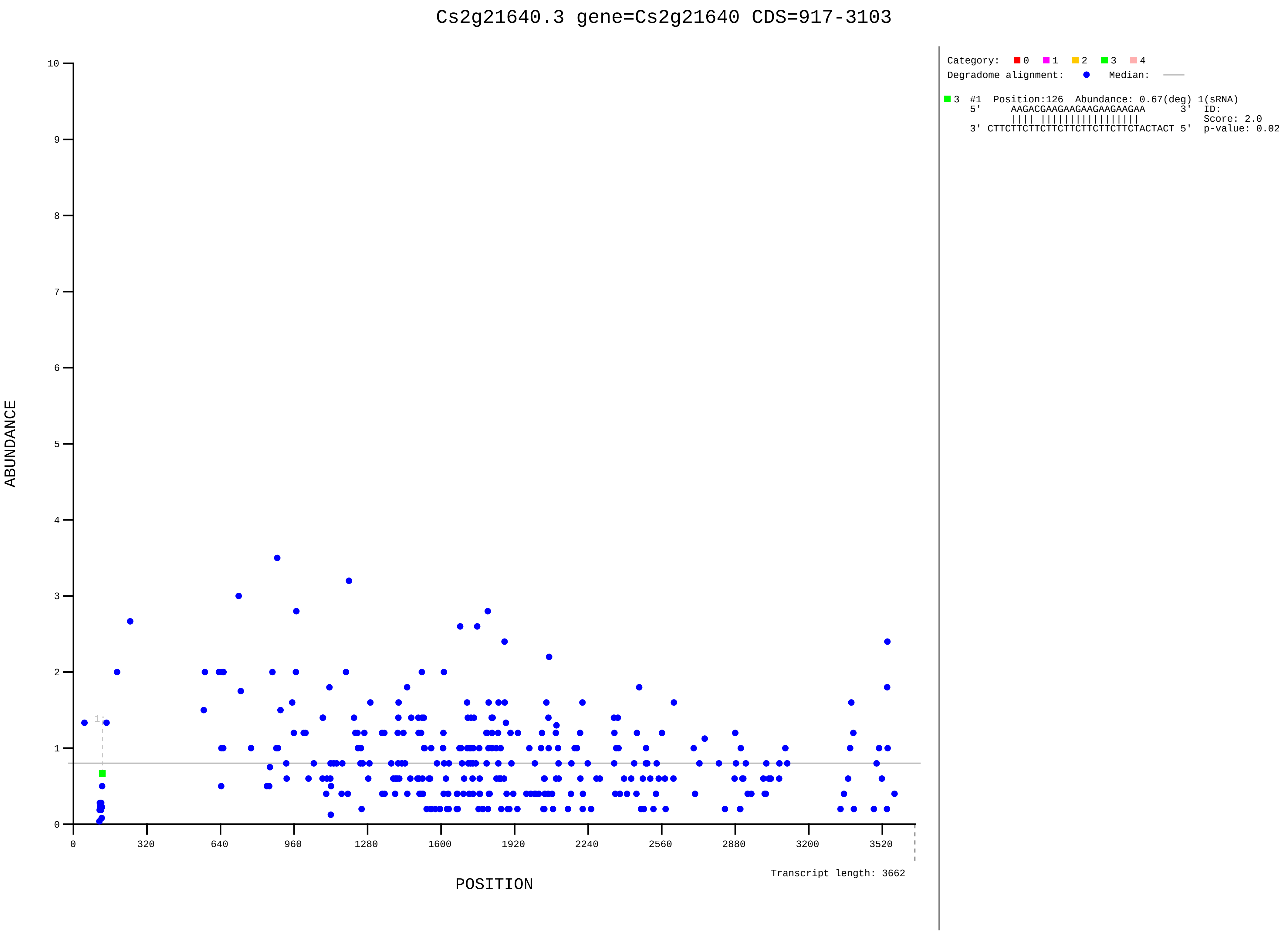

Cs2g21640.3 gene=Cs2g21640 CDS=917-3103
ABUNDANCE
POSITION
Transcript length: 3662
Category: 0 1 2 3 4
Degradome alignment:
Median:
3 #1 Position:126 Abundance: 0.67(deg) 1(sRNA)
5' AAGACGAAGAAGAAGAAGAAGAA 3' ID:
|||| ||||||||||||||||| Score: 2.0
3' CTTCTTCTTCTTCTTCTTCTTCTACTACT 5' p-value: 0.02

Cs2g21640.1 gene=Cs2g21640 CDS=457-2739

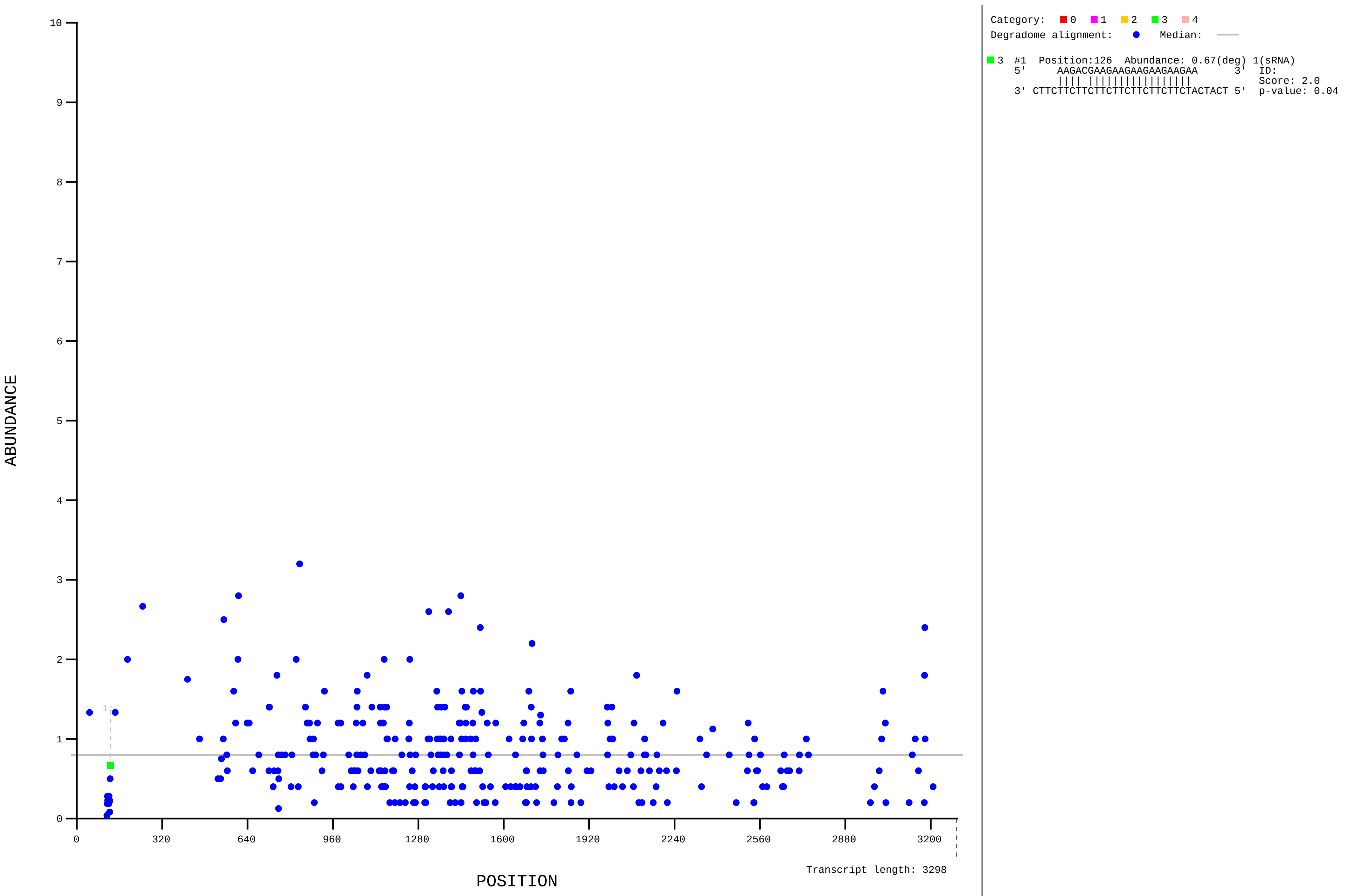

Cs3g19690.3 gene=Cs3g19690 CDS=142-1749

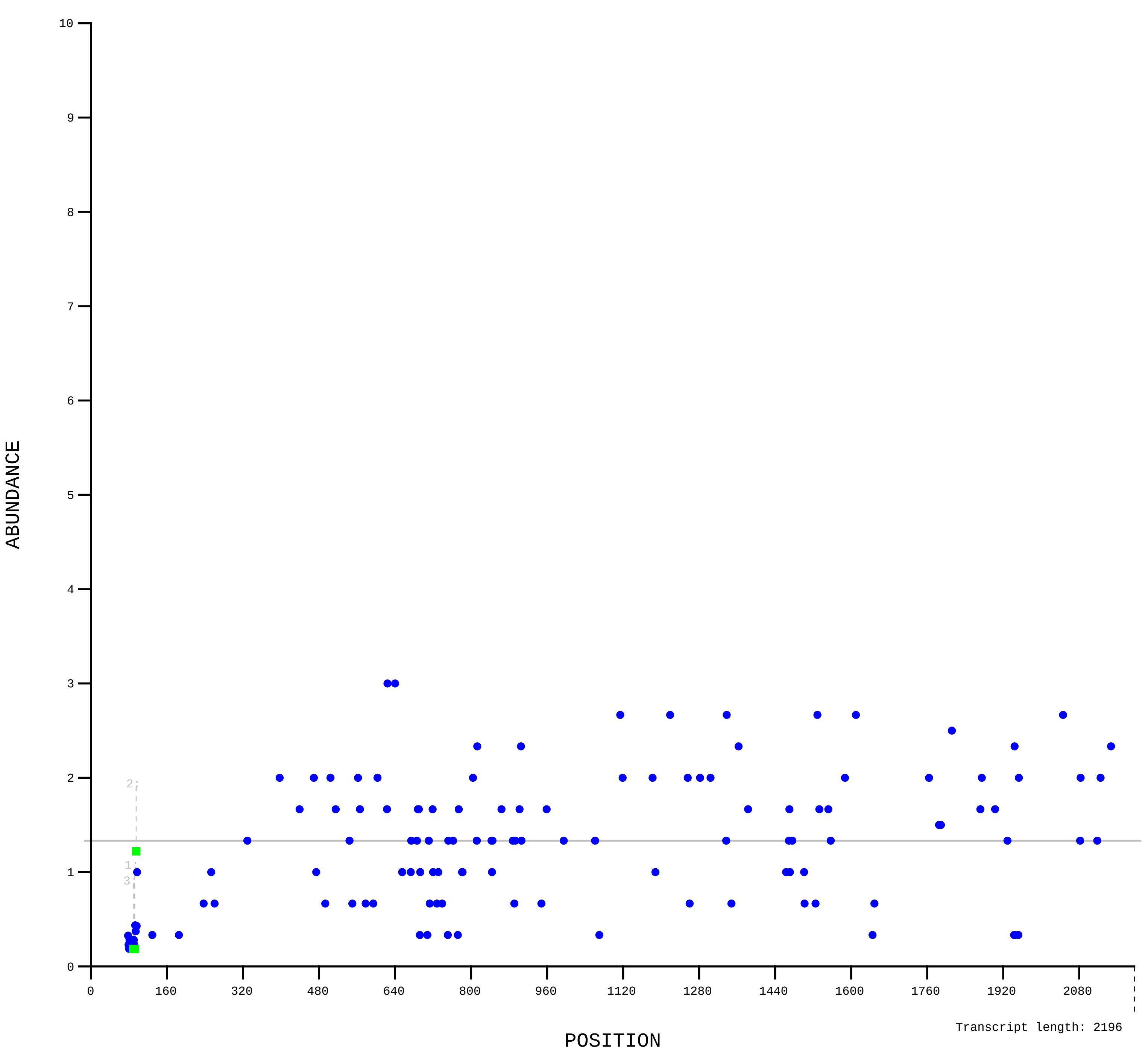


```
3  #1  Position:92  Abundance: 0.19(deg) 1(sRNA)
   5'      AAGACGAAGAAGAAGAAGAAGAA      3'  ID:
           |||| ||||||||||||||||||          Score: 1.0
   3' CTTCTTCTTCTTCTTCTTCTTCTTCTTTCTTT 5'  p-value: 0.0

3  #2  Position:95  Abundance: 1.22(deg) 1(sRNA)
   5'      AAGACGAAGAAGAAGAAGAAGAA      3'  ID:
           |||| ||||||||||||||||||          Score: 1.0
   3' CTTCTTCTTCTTCTTCTTCTTCTTCTTCTTTC 5'  p-value: 0.0

3  #3  Position:89  Abundance: 0.19(deg) 1(sRNA)
   5'      AAGACGAAGAAGAAGAAGAA-GAA     3'  ID:
           |||| ||||||||||||||| |||         Score: 2.0
   3' CTTCTTCTTCTTCTTCTTCTTCTTTCTTTCTT 5'  p-value: 0.05
```

Cs3g19690.1 gene=Cs3g19690 CDS=94-1713

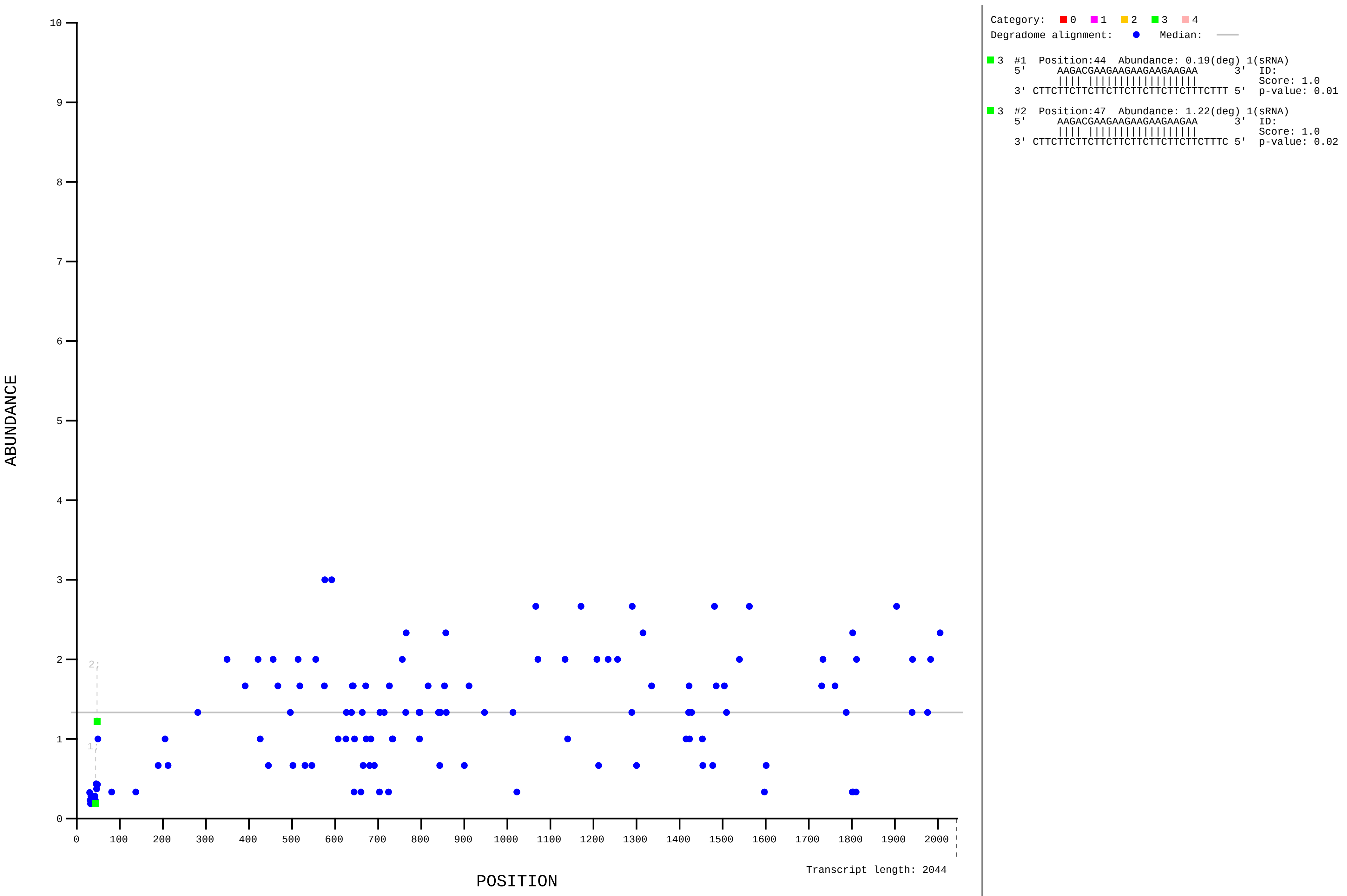

Cs3g19690.2 gene=Cs3g19690 CDS=94-1602

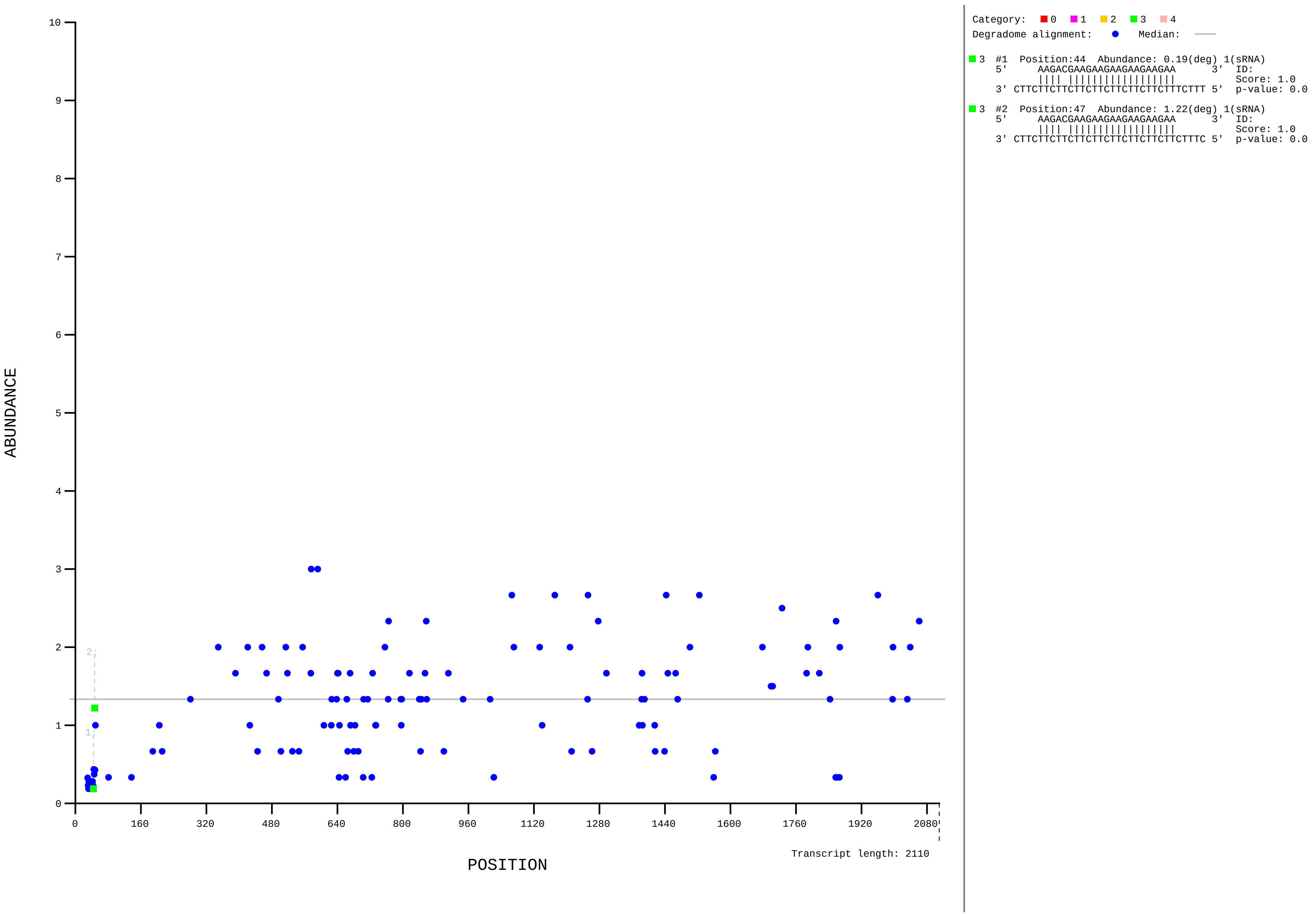

Cs4g08600.1 gene=Cs4g08600 CDS=356-11686

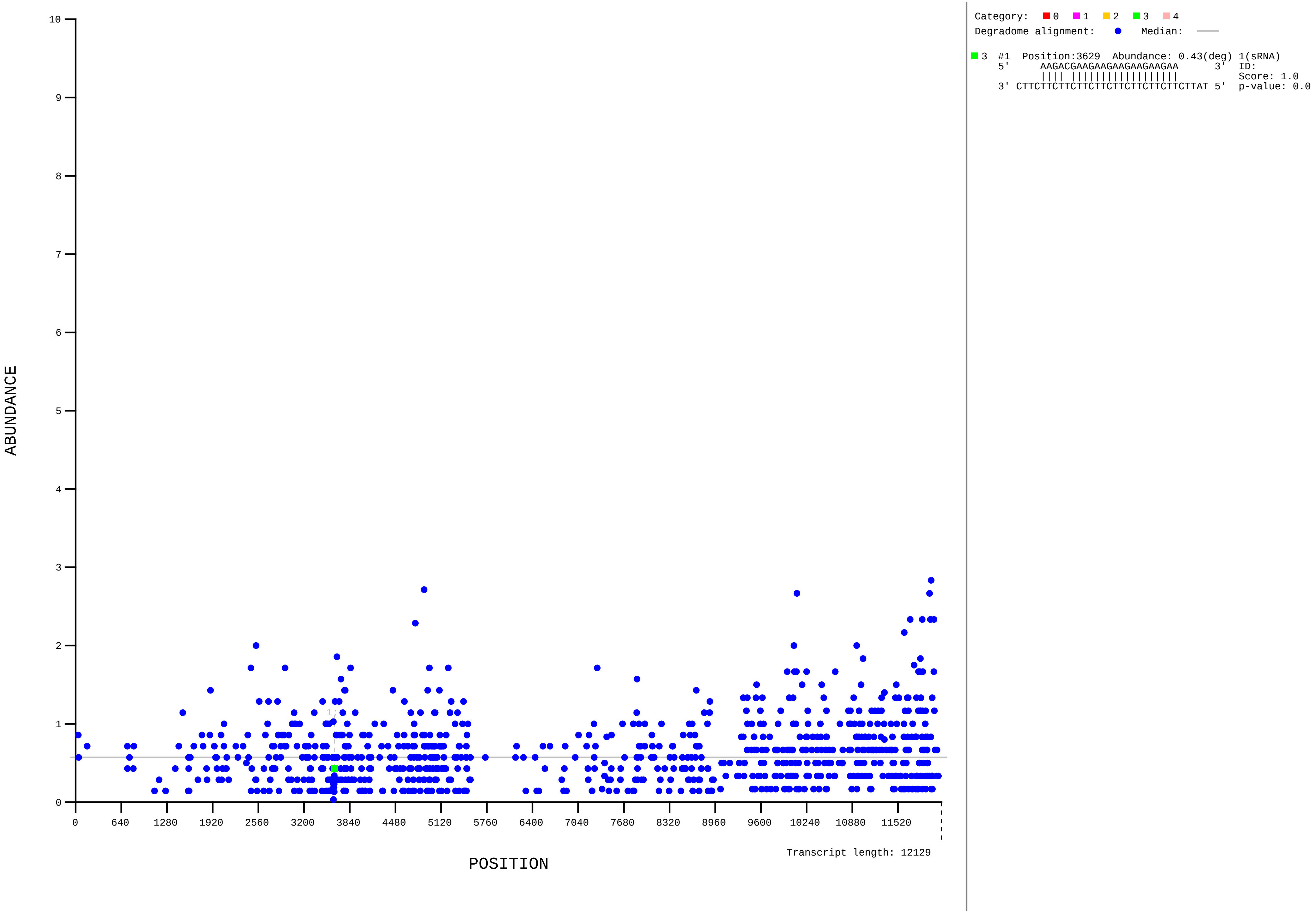

Cs4g08600.5 gene=Cs4g08600 CDS=356-11581

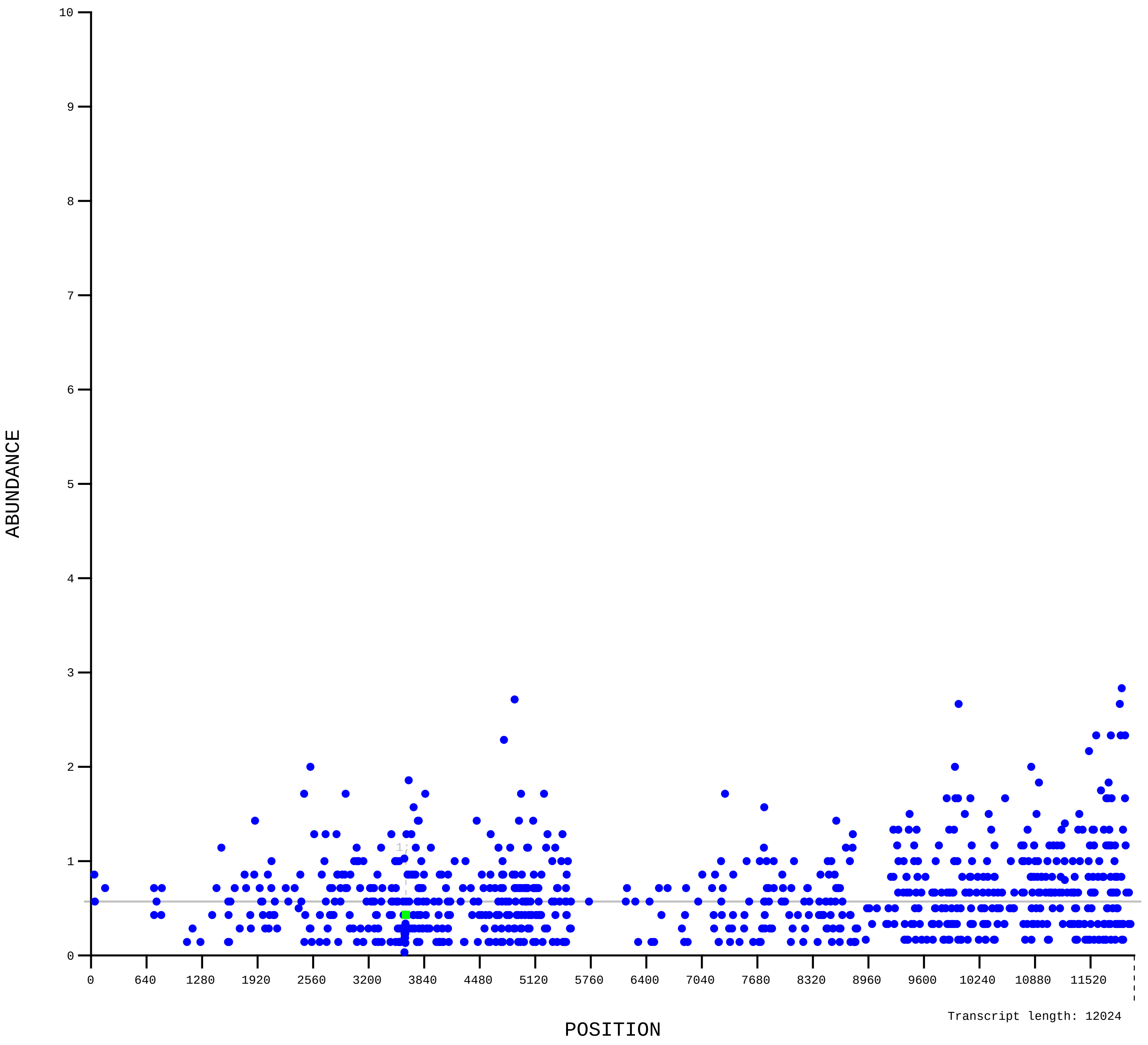

Category: 0 1 2 3 4
Degradome alignment: Median:

```
3  #1  Position:3629  Abundance: 0.43(deg) 1(sRNA)
   5'     AAGACGAAGAAGAAGAAGAA      3'  ID:
          |||| |||||||||||||||          Score: 1.0
   3' CTTCTTCTTCTTCTTCTTCTTCTTCTTAT 5'  p-value: 0.0
```

Cs4g08600.7 gene=Cs4g08600 CDS=561-11768

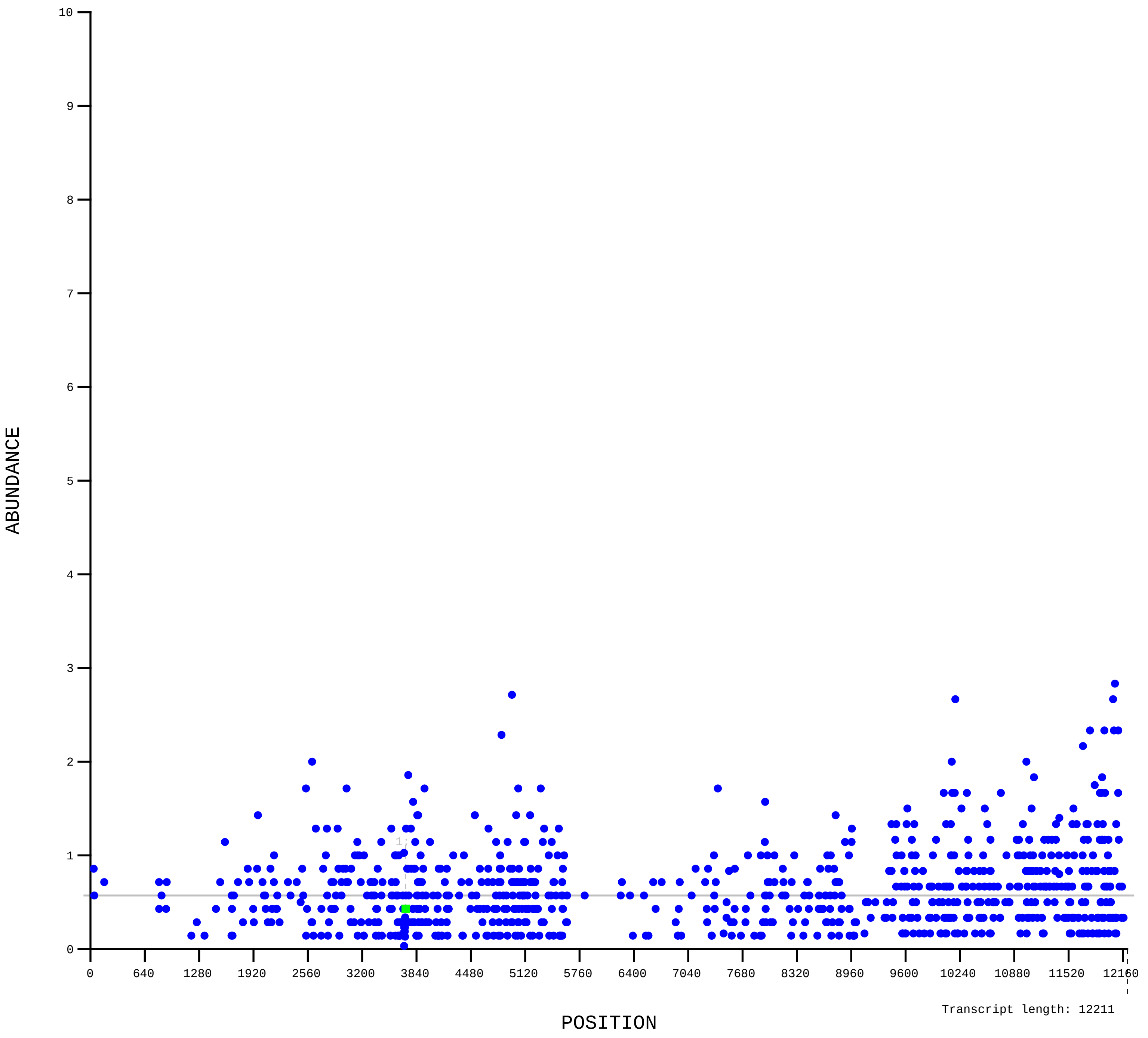

Category: 0 1 2 3 4
Degradome alignment: Median:

```
3  #1  Position:3711  Abundance: 0.43(deg) 1(sRNA)
   5'     AAGACGAAGAAGAAGAAGAAGAA      3'  ID:
          |||| ||||||||||||||||||          Score: 1.0
   3' CTTCTTCTTCTTCTTCTTCTTCTTCTTAT 5'  p-value: 0.0
```

Cs4g08600.6 gene=Cs4g08600 CDS=356-8983

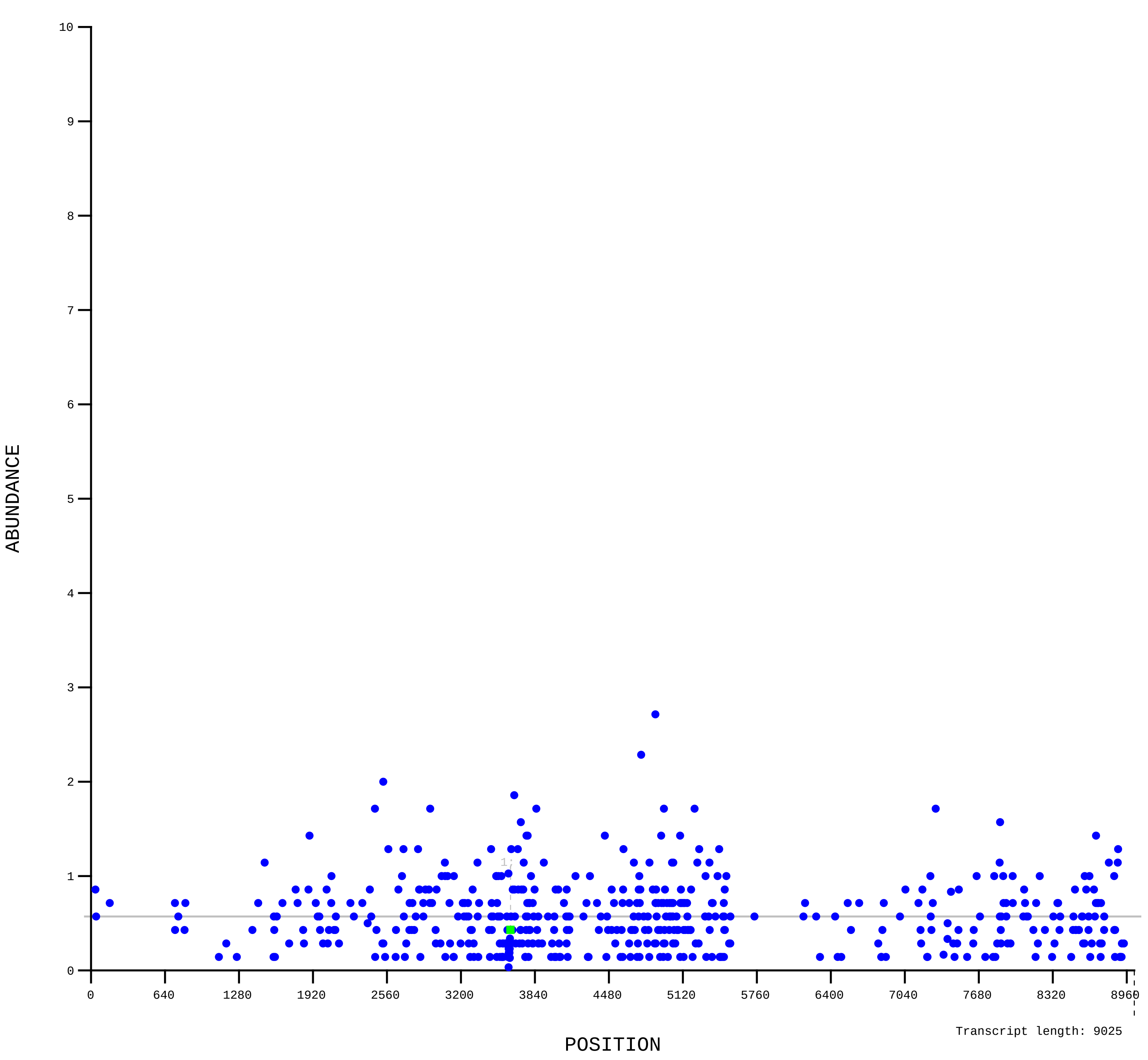

Category: 0 1 2 3 4
Degradome alignment: Median:

3 #1 Position:3629 Abundance: 0.43(deg) 1(sRNA)
5' AAGACGAAGAAGAAGAAGAAGAA 3' ID:
|||| |||||||||||||||||| Score: 1.0
3' CTTCTTCTTCTTCTTCTTCTTCTTCTTAT 5' p-value: 0.01

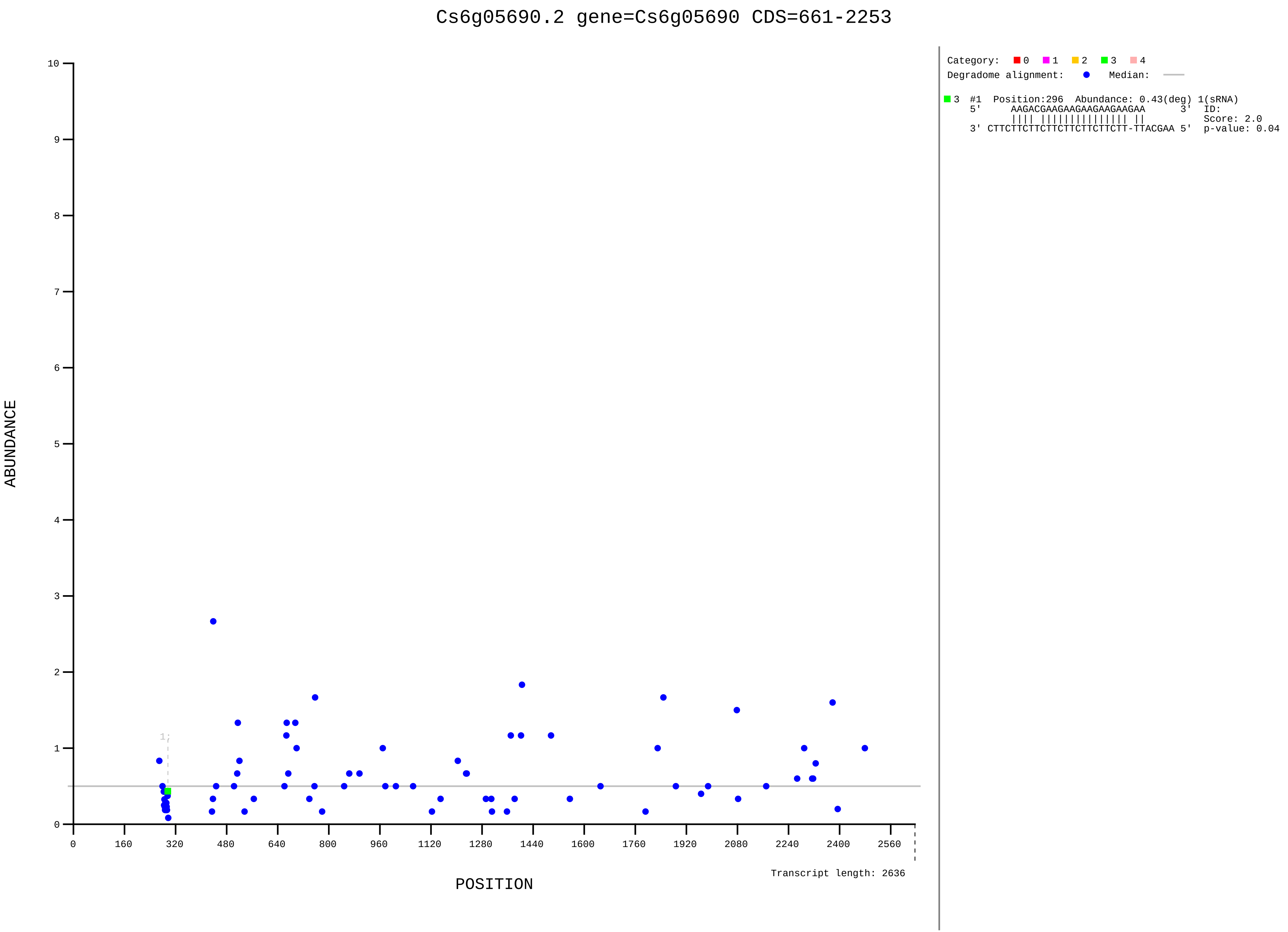
Cs6g05690.2 gene=Cs6g05690 CDS=661-2253
ABUNDANCE
POSITION
Transcript length: 2636
Category: 0 1 2 3 4
Degradome alignment:
Median:
3 #1 Position:296 Abundance: 0.43(deg) 1(sRNA)
5' AAGACGAAGAAGAAGAAGAAGAA 3' ID:
|||| ||||||||||||||| ||
Score: 2.0
3' CTTCTTCTTCTTCTTCTTCTTCTT-TTACGAA 5' p-value: 0.04

Cs6g05690.4 gene=Cs6g05690 CDS=509-2104

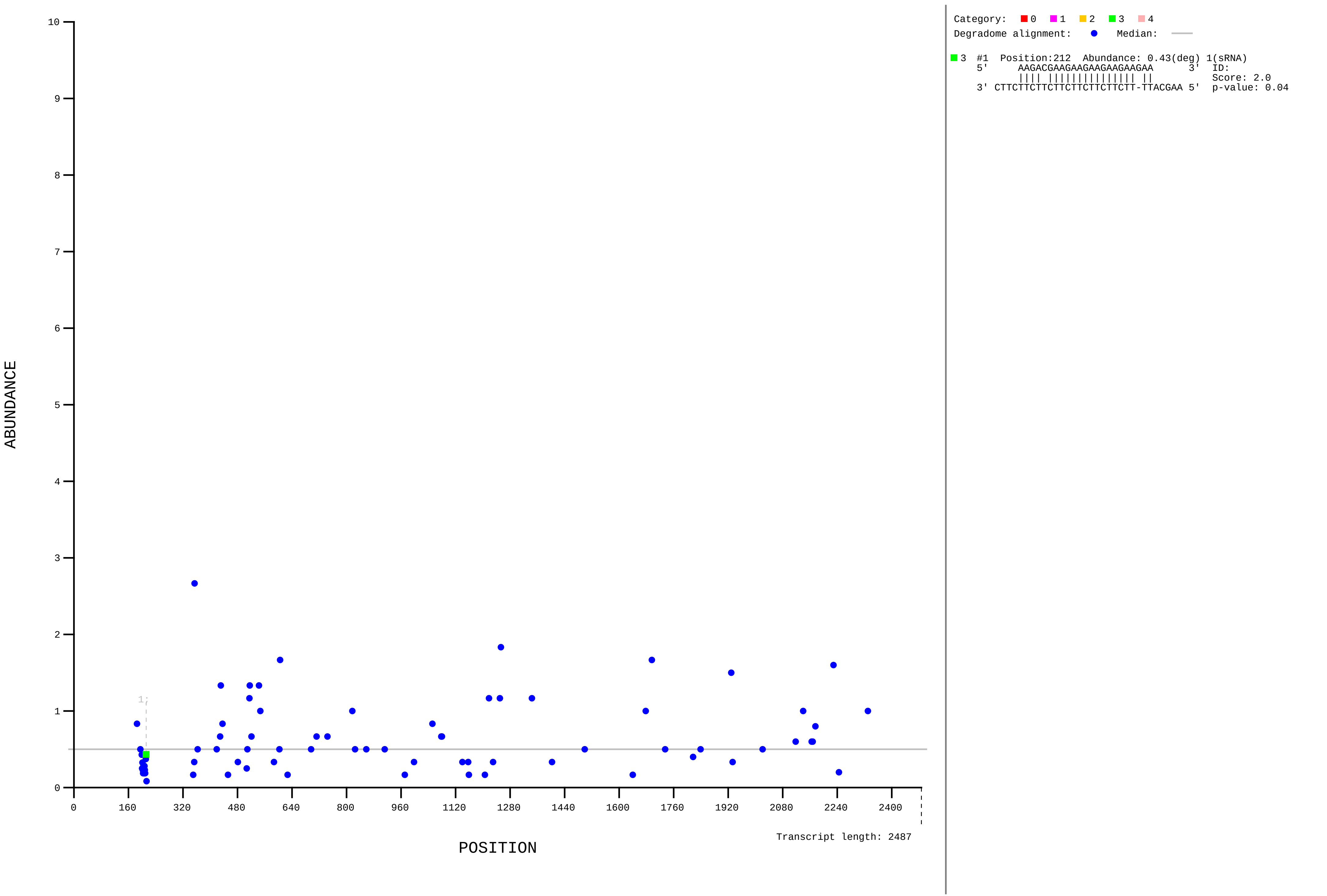

Cs4g06140.4 gene=Cs4g06140 CDS=240-728

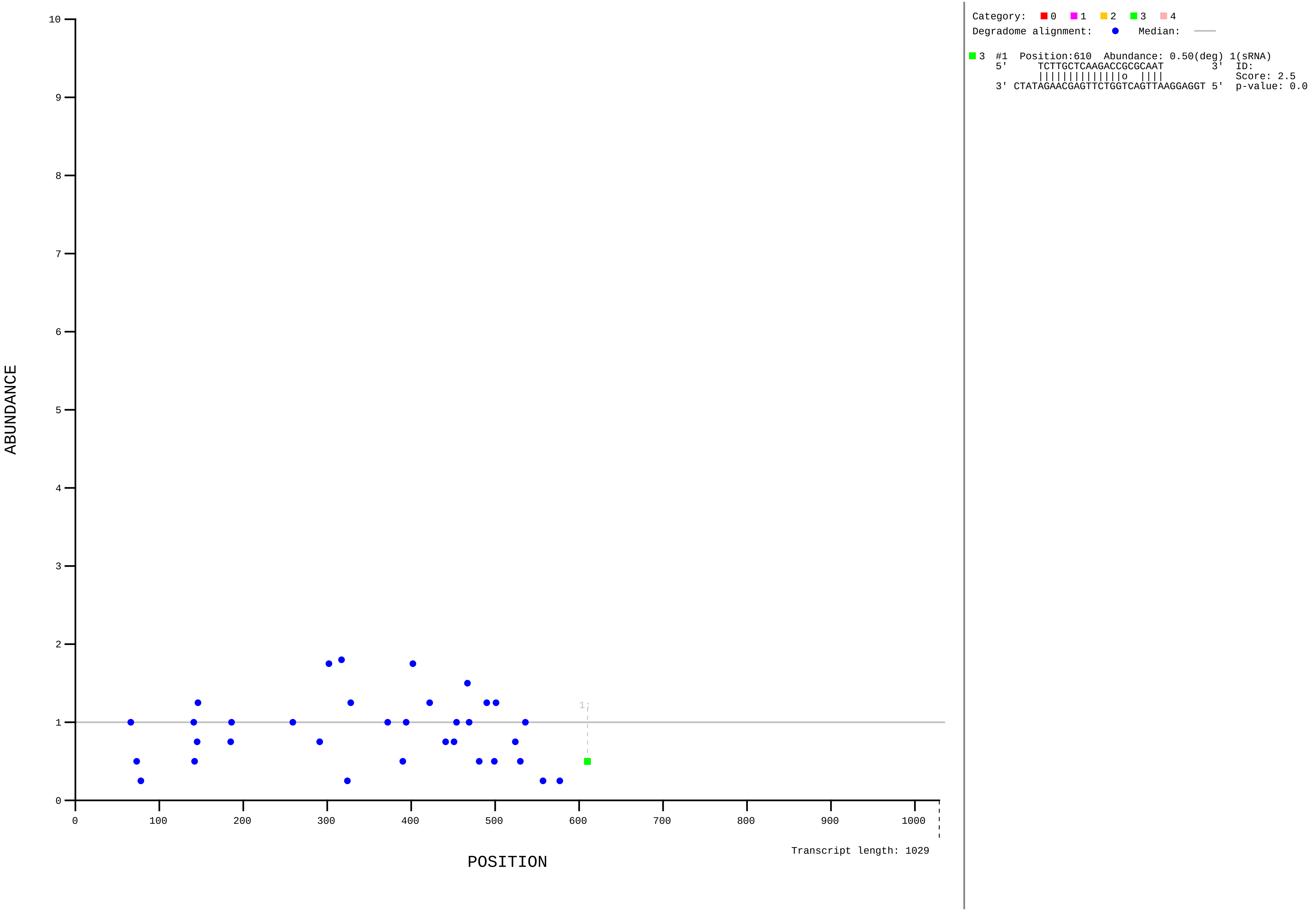

Cs3g15880.1 gene=Cs3g15880 CDS=599-2569

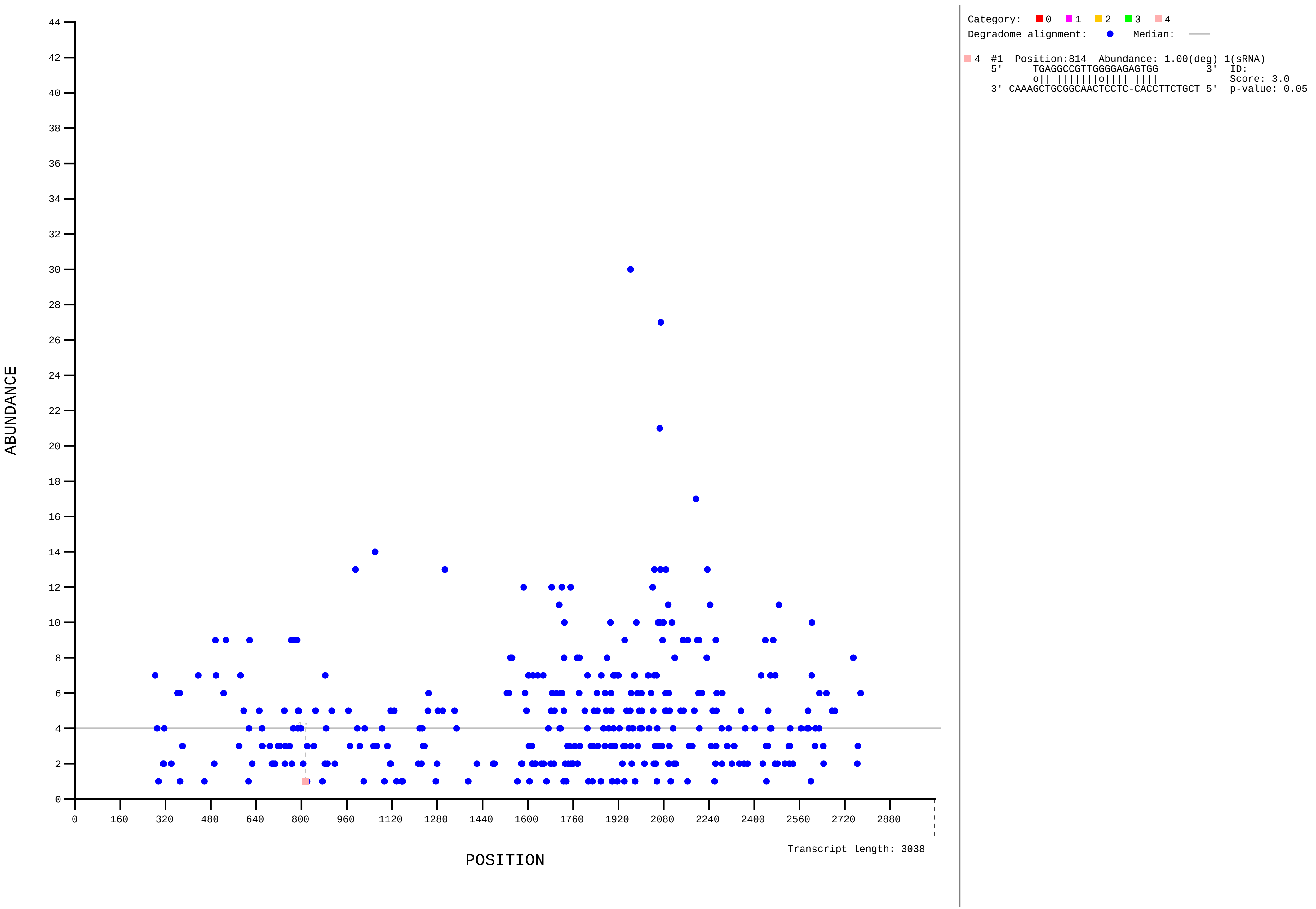

Cs5g09210.1 gene=Cs5g09210 CDS=58-1254

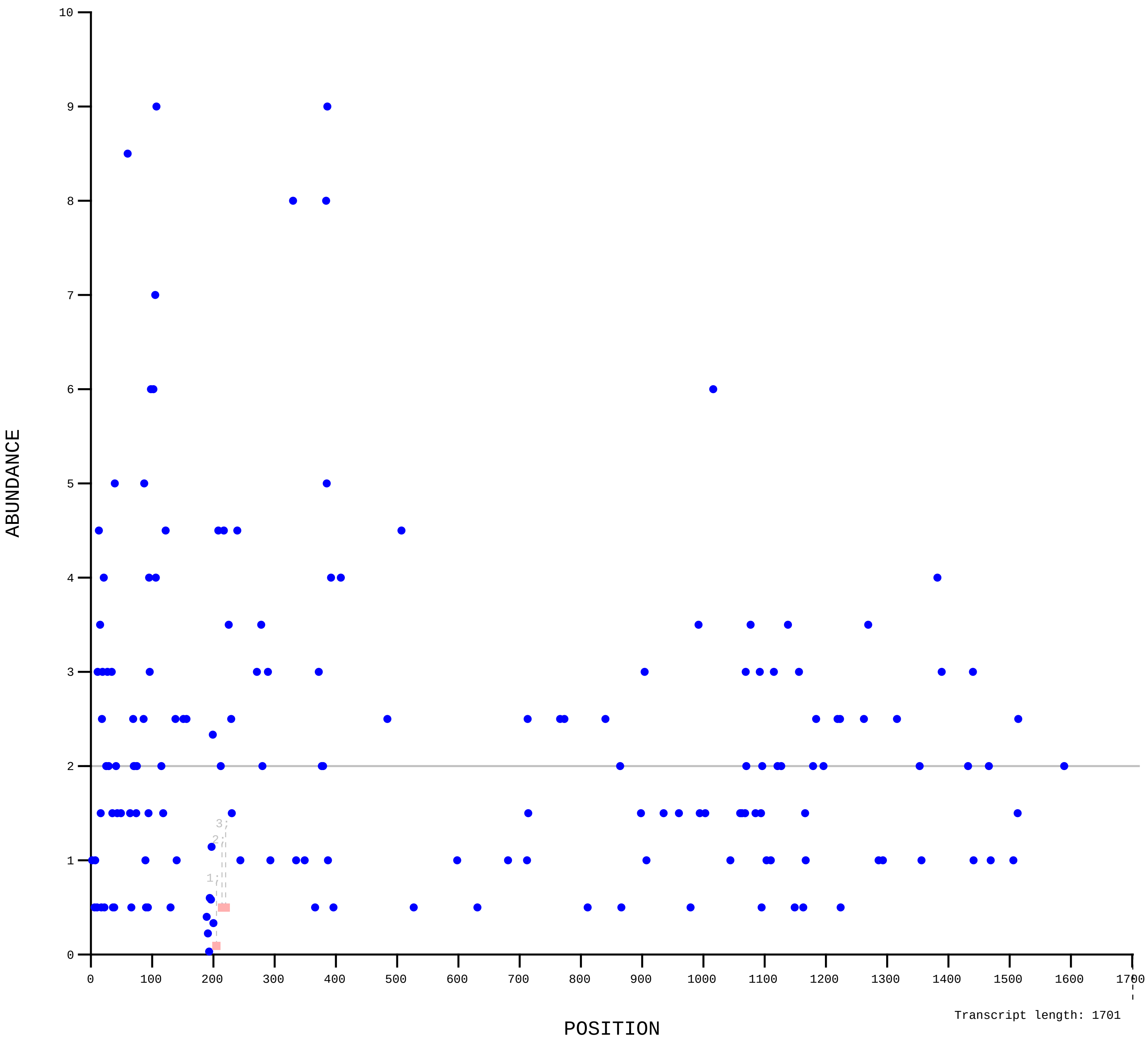

Category: 0 1 2 3 4
Degradome alignment: ● Median: —

```
4  #1  Position:205  Abundance: 0.09(deg) 1(sRNA)
   5'      AAGACGAAGAAGAAGAAGAAGAA      3'  ID:
           |||| |||||||||||||||||           Score: 1.0
   3' CTTCTTCTACTTCTTCTTCTTCTTCTTCGCGC 5'  p-value: 0.0

4  #2  Position:214  Abundance: 0.50(deg) 1(sRNA)
   5'      AAGACGAAGAAGAAGAAGAAGAA      3'  ID:
           |||| |||||||| |||||||||          Score: 2.0
   3' TCGCTTCTTCTTCTTCTACTTCTTCTTCTTCT 5'  p-value: 0.0

4  #3  Position:220  Abundance: 0.50(deg) 1(sRNA)
   5'      AAGACGAAGAAGAAGAAGAAGAA      3'  ID:
           ||| ||||||||||||||| |||          Score: 2.0
   3' GTAGTTC-GCTTCTTCTTCTTCTACTTCTTCT 5'  p-value: 0.01
```

Cs5g09210.2 gene=Cs5g09210 CDS=584-1435

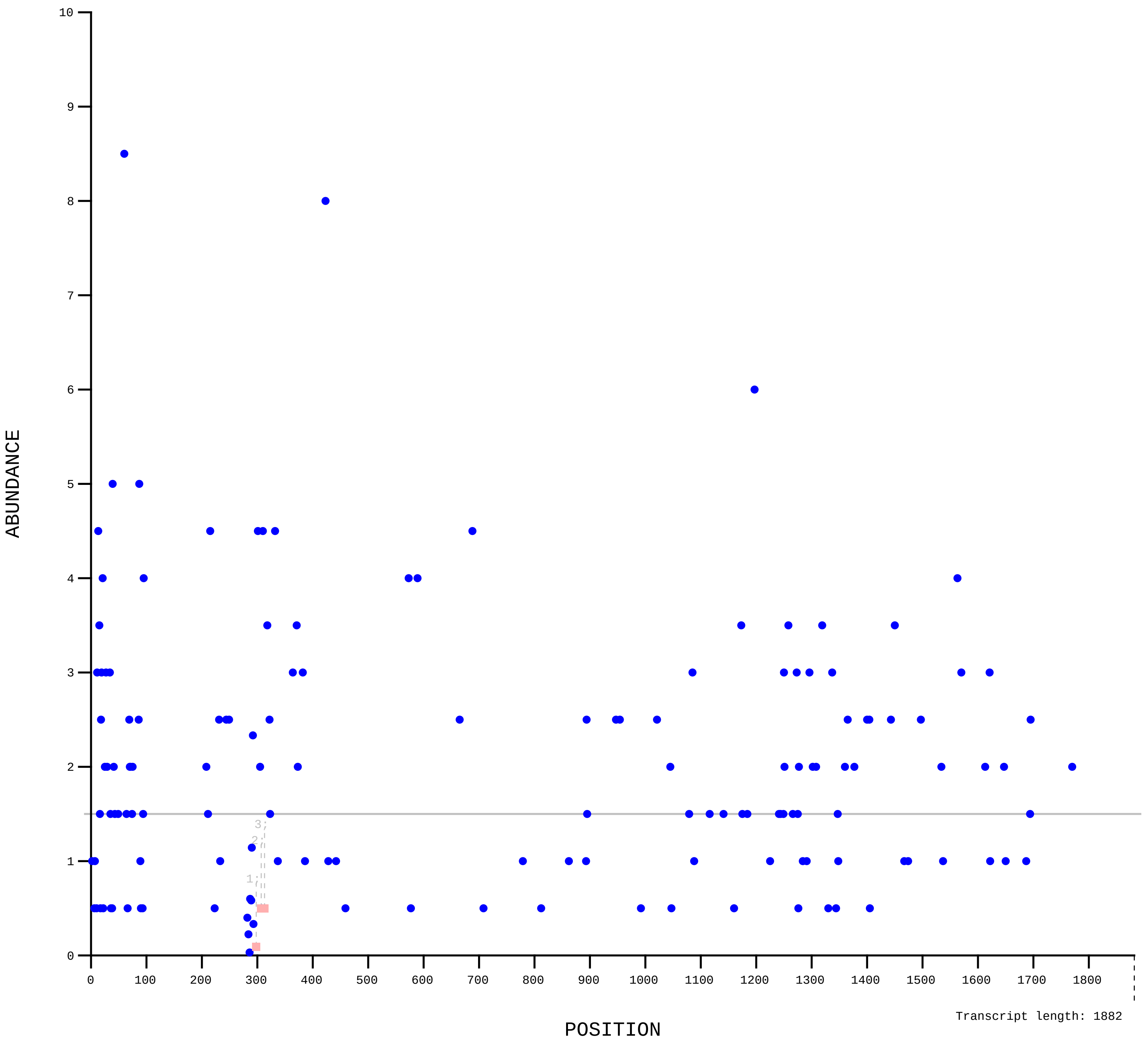


```
Category:  0  1  2  3  4
Degradome alignment:     Median:

4  #1  Position:298  Abundance: 0.09(deg) 1(sRNA)
   5'     AAGACGAAGAAGAAGAAGAAGAA      3'  ID:
          |||| ||||||||||||||||||          Score: 1.0
   3' CTTCTTCTACTTCTTCTTCTTCTTCTTCGCGC 5'  p-value: 0.0

4  #2  Position:307  Abundance: 0.50(deg) 1(sRNA)
   5'     AAGACGAAGAAGAAGAAGAAGAA      3'  ID:
          |||| |||||||| |||||||||          Score: 2.0
   3' TCGCTTCTTCTTCTTCTACTTCTTCTTCTTCT 5'  p-value: 0.0

4  #3  Position:313  Abundance: 0.50(deg) 1(sRNA)
   5'     AAGACGAAGAAGAAGAAGAAGAA      3'  ID:
          ||| ||||||||||||||| |||          Score: 2.0
   3' GTAGTTC-GCTTCTTCTTCTTCTACTTCTTCT 5'  p-value: 0.02
```

Cs9g03230.1 gene=Cs9g03230 CDS=18-2468

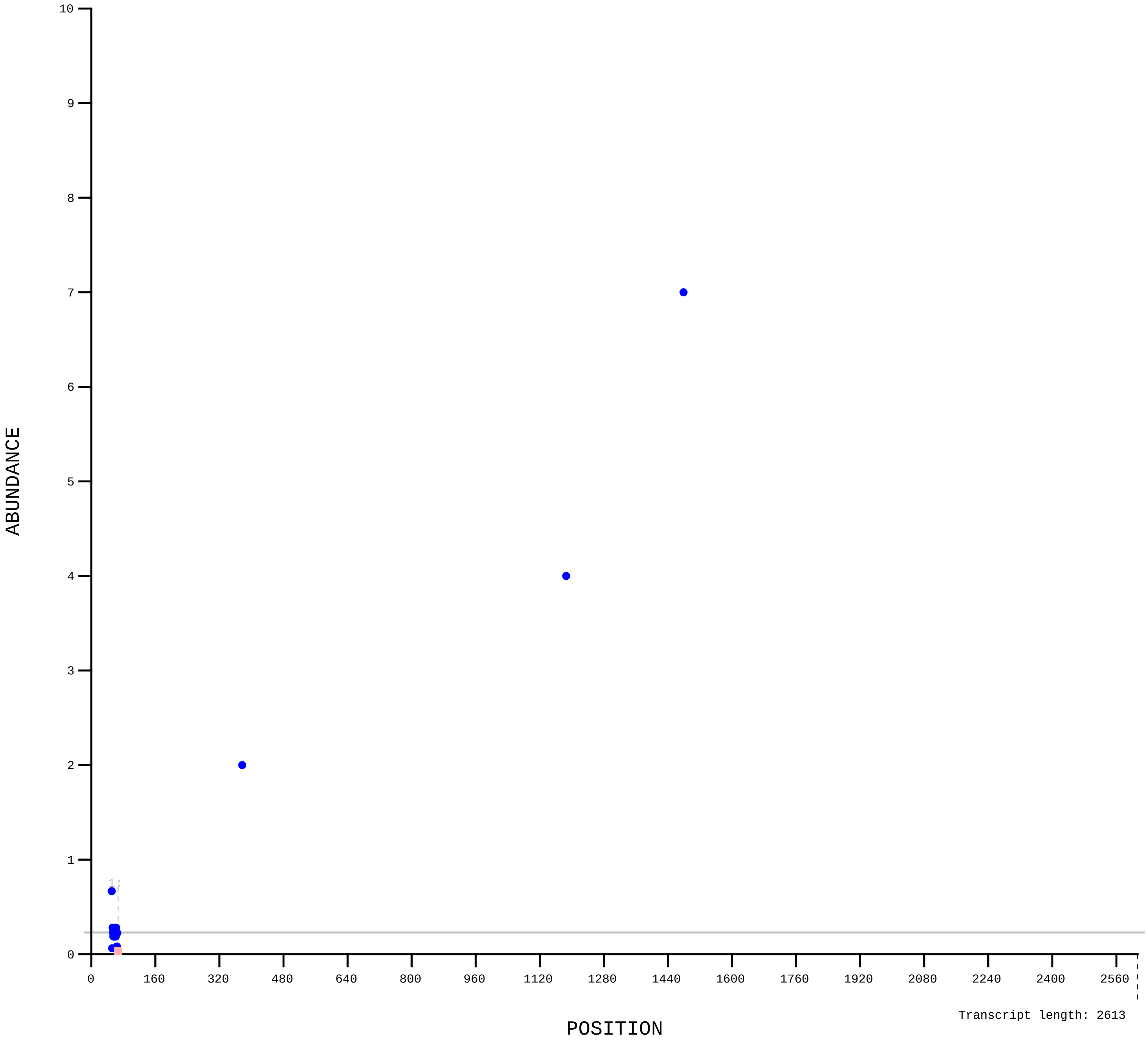

Category: 0 1 2 3 4
Degradome alignment: Median:

4 #1 Position:67 Abundance: 0.03(deg) 1(sRNA)

```
5'     AAGACGAAGAAGAAGAAGAAGAA      3'  ID:
       |||| ||||||||||||||||||          Score: 1.0
3' CTTCTTCTTCTTCTTCTTCTTCTTCTTCACCT 5'  p-value: 0.0
```

Cs3g12190.2 gene=Cs3g12190 CDS=22-1893

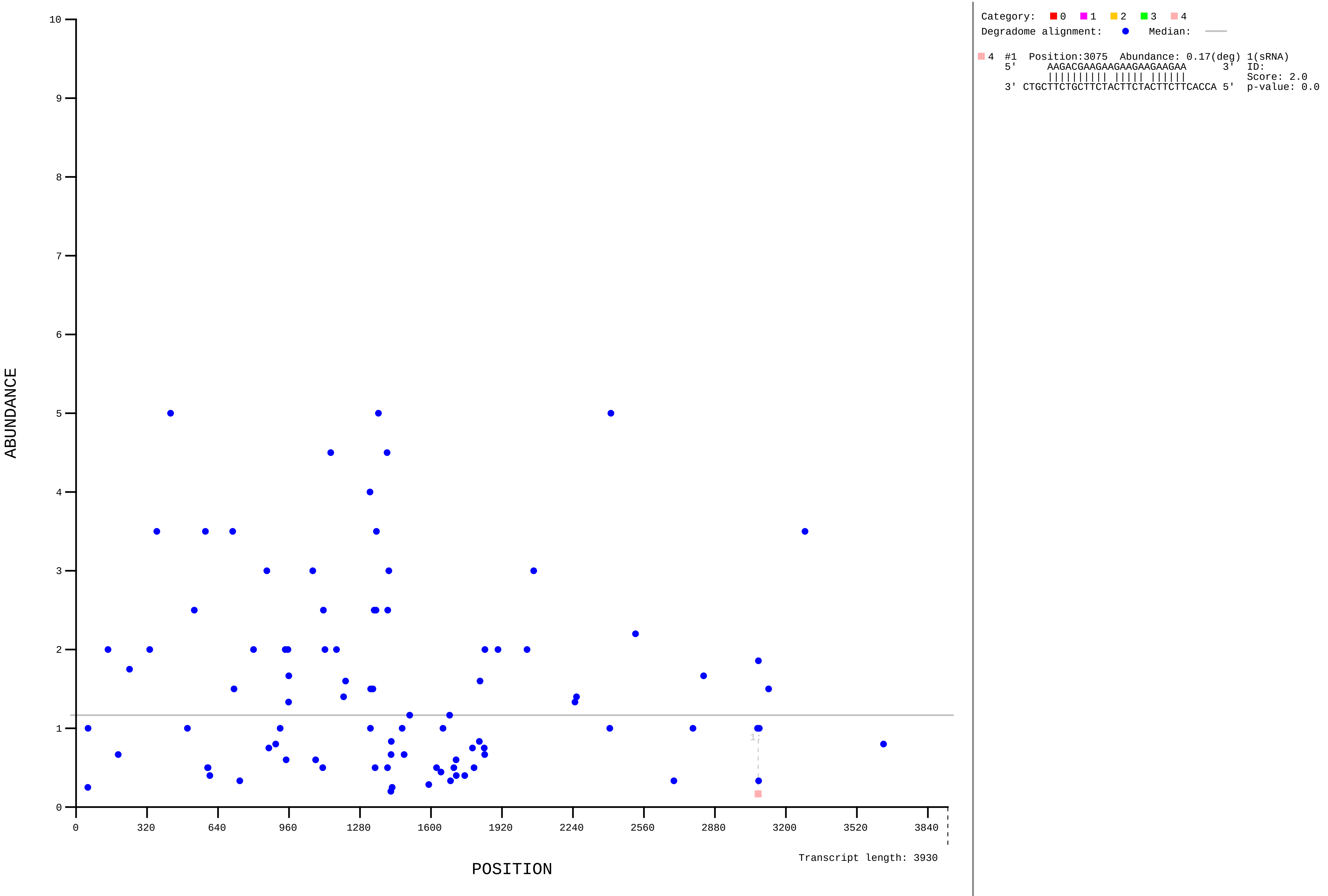

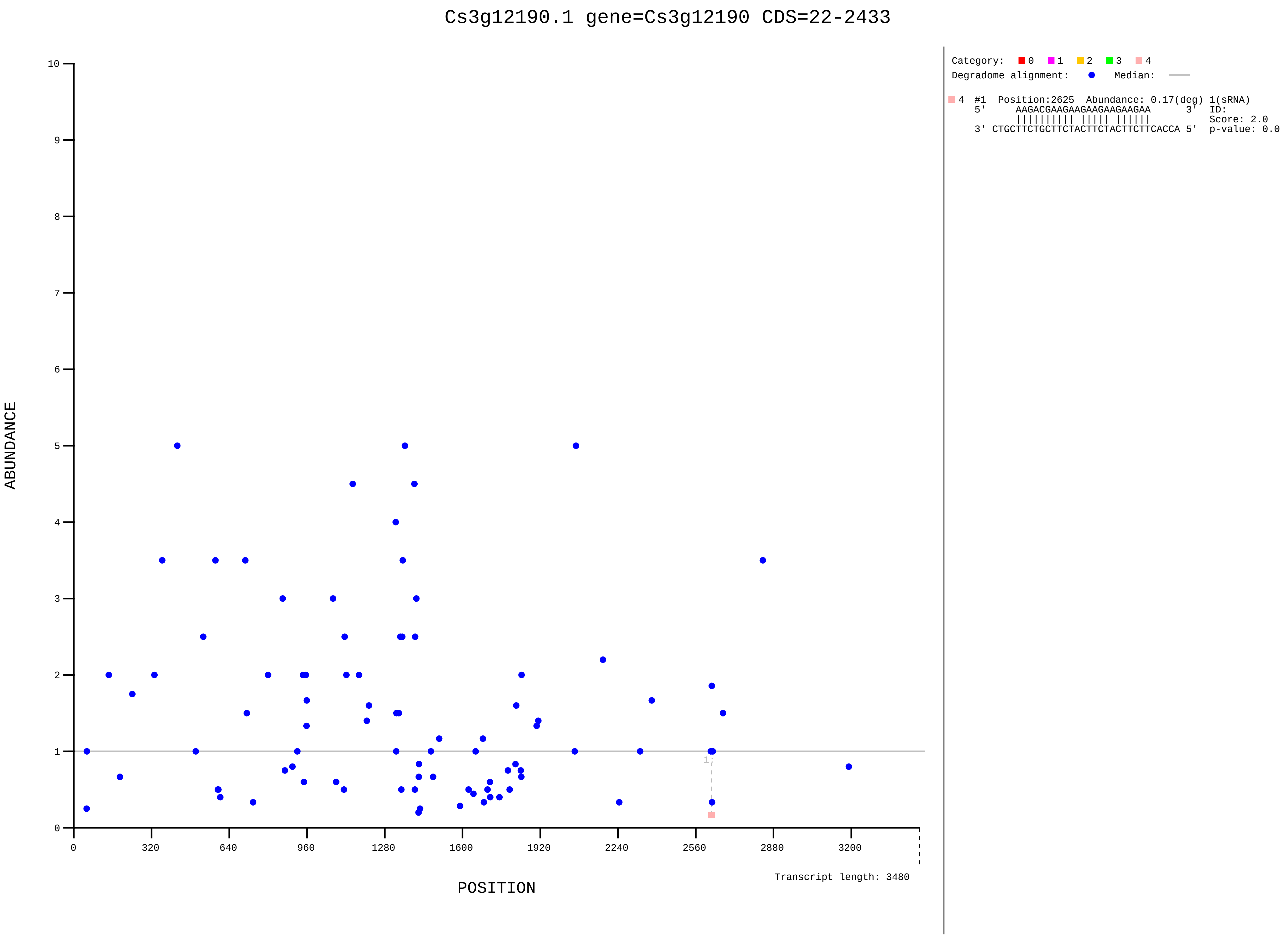

Cs3g12190.1 gene=Cs3g12190 CDS=22-2433
Category: 0 1 2 3 4
Degradome alignment:
Median:
4 #1 Position:2625 Abundance: 0.17(deg) 1(sRNA)
5' AAGACGAAGAAGAAGAAGAAGAA 3' ID:
|||||||||| ||||| ||||||
3' CTGCTTCTGCTTCTACTTCTACTTCTTCACCA 5'
Score: 2.0
p-value: 0.0
ABUNDANCE
POSITION
Transcript length: 3480
