# Supplementary material for: Comparative Analysis of miRNAs and Their Target Transcripts between a Spontaneous Late-Ripening Sweet Orange Mutant and Its Wild-Type Using Small RNA and Degradome Sequencing
Source: Front Plant Sci. 2016 Sep 21;7:1416. doi: 10.3389/fpls.2016.01416 (PMC5030777; doi:10.3389/fpls.2016.01416)
Supplement: Table S7 — Identified PHAS genes in citrus fruit. [file Table7.docx]

Table S7: Identified PHAS genes in citrus fruit.

| **miRNA** | ***PHAS* gene** | **Annotation** | **Average No. of phasiRNAs** | | **Source** |
| --- | --- | --- | --- | --- | --- |
|  |  |  | **MT** | **WT** |  |
| csi-miRN21 | Cs6g02100 | PREDICTED: disease resistance protein At4g27190-like | 11 | 10 | MT/WT |
| csi-miRN21 | Cs6g02130 | cc-nbs-lrr resistance protein | 10.5 | 8.5 | MT/WT |
| csi-miR1515 | Cs6g03500 | dicer-like protein | 9 | 7 | MT/WT |
| csi-miR1515 | Cs6g03520 | dicer-like protein | 11.5 | 12 | MT/WT |
| csi-miR167a | Cs6g16030 | ARF8 | 3.5 | 3 | MT/WT |
| csi-miR393h | Cs2g14270 | TIR1/AFB auxin receptor protein PintaAFB4A | 5.5 | 7.5 | MT/WT |
| csi-miR393h | Cs5g32500 | TIR1/AFB auxin receptor protein PintaAFB4A | 15.5 | 17.5 | MT/WT |
| csi-miR3950 | Cs1g09575 | unknown | 17 | 17 | MT/WT |
| csi-miR3950 | Cs1g09600 | unknown | 13.5 | 13.5 | MT/WT |
| csi-miR3950 | Cs1g09660 | NAC domain-containing protein 69-like | 10.5 | 6.5 | MT/WT |
| csi-miR3950 | Cs5g04670 | unknown | 9 | 7.5 | MT/WT |
| csi-miR3950 | Cs7g22470 | NAC domain-containing protein 4 | 17 | 16 | MT/WT |
| csi-miR3950 | orange1.1t05093 | NAC domain-containing protein 21/22 | 10.5 | 6.5 | MT/WT |
| csi-miR472 | Cs5g26190 | cc-nbs-lrr resistance protein | 12 | - | WT |
| csi-miR472 | orange1.1t03707 | Disease resistance protein RPS5 | 7 | - | WT |
| csi-miR472 | orange1.1t04592 | cc-nbs-lrr resistance protein | 6.5 | - | WT |
| csi-miR472 | Cs1g08330 | Disease resistance protein RPS5 | 10 | 10.5 | MT/WT |
| csi-miR472 | Cs1g13430 | NBS-LRR type disease resistance protein | 11.5 | 10 | MT/WT |
| csi-miR472 | Cs1g14090 | LRR and NB-ARC domain-containing disease resistance protein | 10.5 | 9 | MT/WT |
| csi-miR472 | Cs2g18470 | Disease resistance protein RFL1 | 14.5 | 12 | MT/WT |
| csi-miR472 | Cs3g04930 | cc-nbs-lrr resistance protein | 11 | 10.5 | MT/WT |
| csi-miR472 | Cs3g05280 | disease resistance protein At4g27190-like | 13 | 11 | MT/WT |
| csi-miR472 | Cs3g07940 | cc-nbs-lrr resistance protein | 9.5 | 8 | MT/WT |
| csi-miR472 | Cs3g08210 | cc-nbs-lrr resistance protein | 15 | 14.5 | MT/WT |
| csi-miR472 | Cs7g26730 | TMV resistance protein N | 10 | 11.5 | MT/WT |
| csi-miR472 | orange1.1t01362 | NBS-LRR type disease resistance protein | 13 | 12 | MT/WT |
| csi-miR472 | orange1.1t03137 | Disease resistance protein RPS5 | 15 | 14.5 | MT/WT |
| csi-miR472 | orange1.1t03712 | cc-nbs-lrr resistance protein | 15 | 15.5 | MT/WT |
| csi-miR472 | orange1.1t03713 | disease resistance protein At4g27190-like | 15 | 13 | MT/WT |
| csi-miR482a-3p | Cs3g06500 | cc-nbs-lrr resistance protein | 5 | - | WT |
| csi-miR482a-3p | Cs3g13430 | disease resistance protein At4g27190-like | 7 | - | WT |
| csi-miR482a-3p | Cs5g19850 | leucine-rich repeat-containing protein | 5 | - | WT |
| csi-miR482a-3p | orange1.1t01898 | cc-nbs-lrr resistance protein | 11.5 | - | WT |
| csi-miR482a-3p | orange1.1t03734 | PREDICTED: putative disease resistance protein RGA4-like | 14 | - | WT |
| csi-miR482a-3p | Cs1g09350 | cc-nbs-lrr resistance protein | 8.5 | 8 | MT/WT |
| csi-miR482a-3p | Cs1g15550 | cc-nbs-lrr resistance protein | 14 | 12.5 | MT/WT |
| csi-miR482a-3p | Cs1g19720 | chaperone protein DNAj, putative | 7.5 | 8 | MT/WT |
| csi-miR482a-3p | Cs3g06590 | Disease resistance protein RPS5, putative | 8 | 7 | MT/WT |
| csi-miR482a-3p | Cs3g06600 | cc-nbs-lrr resistance protein | 5.5 | 5.5 | MT/WT |
| csi-miR482a-3p | Cs3g12760 | cc-nbs-lrr resistance protein | 10.5 | 9 | MT/WT |
| csi-miR482a-3p | Cs3g12810 | Disease resistance protein RPS5, putative | 15 | 15.5 | MT/WT |
| csi-miR482a-3p | Cs3g12850 | cc-nbs-lrr resistance protein | 13.5 | 13 | MT/WT |
| csi-miR482a-3p | Cs3g13690 | putative disease resistance protein RGA4-like | 11 | 11.5 | MT/WT |
| csi-miR482a-3p | Cs5g19430 | PREDICTED: TMV resistance protein N-like | 10.5 | 10 | MT/WT |
| csi-miR482a-3p | Cs5g19440 | tir-nbs-lrr resistance protein | 13 | 10 | MT/WT |
| csi-miR482a-3p | Cs5g19570 | PREDICTED: TMV resistance protein N-like | 11 | 13 | MT/WT |
| csi-miR482a-3p | Cs5g19920 | putative resistance gene analogue protein | 13.5 | 13.5 | MT/WT |
| csi-miR482a-3p | Cs5g21975 | leucine-rich repeat containing protein, putative | 15.5 | 14 | MT/WT |
| csi-miR482a-3p | Cs5g22030 | cc-nbs-lrr resistance protein | 7 | 9 | MT/WT |
| csi-miR482a-3p | Cs8g10560 | Leucine-rich repeat containing protein, putative | 8.5 | 7.5 | MT/WT |
| csi-miR482a-3p | orange1.1t01815 | TMV resistance protein N, putative | 6.5 | 6.5 | MT/WT |
| csi-miR482a-3p | orange1.1t01918 | PREDICTED: putative disease resistance protein RGA4-like | 13.5 | 13.5 | MT/WT |
| csi-miR482a-3p | orange1.1t01926 | cc-nbs-lrr resistance protein | 7.5 | 7.5 | MT/WT |
| csi-miR482a-3p | orange1.1t04537 | cc-nbs-lrr resistance protein | 10.5 | 10.5 | MT/WT |
| csi-miR482a-3p | Cs3g06650 | cc-nbs-lrr resistance protein | - | 4.5 | MT |
| csi-miR482a-3p | Cs3g11030 | PREDICTED: importin subunit alpha-1 | - | 6.5 | MT |
| csi-miR482a-3p | Cs3g11080 | cc-nbs-lrr resistance protein | - | 7.5 | MT |
| csi-miR482a-3p | Cs3g13320 | cc-nbs-lrr resistance protein | - | 9.5 | MT |
| csi-miR482a-3p | Cs3g13340 | cc-nbs-lrr resistance protein | - | 7.5 | MT |
| csi-miR482a-3p | Cs3g13390 | cc-nbs-lrr resistance protein | - | 8.5 | MT |
| csi-miR482a-3p | orange1.1t00149 | Protein bem46, putative | - | 3 | MT |
| csi-miR482a-3p | orange1.1t01829 | TMV resistance protein N | - | 6 | MT |
| csi-miR482b | Cs5g22330 | resistance protein-like protein | 5.5 | - | WT |
| csi-miR482b | Cs2g18480 | PREDICTED: disease resistance protein At4g27190-like | 9 | 7 | MT/WT |
| csi-miR482c | Cs1g08080 | NBS-LRR resistance protein RGH1 | 14.5 | 13 | MT/WT |
| csi-miR482c | Cs9g06846 | unknown | 17.5 | 17 | MT/WT |
